# Design, Synthesis, and Biological Evaluation of Chemically and Biologically Diverse Pyrroquinoline Pseudo Natural Products

**DOI:** 10.1002/anie.202013731

**Published:** 2021-01-12

**Authors:** Jie Liu, Gregor S. Cremosnik, Felix Otte, Axel Pahl, Sonja Sievers, Carsten Strohmann, Herbert Waldmann

**Affiliations:** ^1^ Max Planck Institute of Molecular Physiology Department of Chemical Biology Otto-Hahn-Strasse 11 44227 Dortmund Germany; ^2^ Technical University Dortmund Faculty of Chemistry Chemical Biology Otto-Hahn-Strasse 6 44221 Dortmund Germany; ^3^ Technical University Dortmund Faculty of Chemistry Inorganic Chemistry Otto-Hahn-Strasse 6 44221 Dortmund Germany; ^4^ Compound Management and Screening Center Dortmund Germany

**Keywords:** cell painting, cheminformatics, cycloaddition, heterocycles, natural products

## Abstract

Natural product (NP) structures are a rich source of inspiration for the discovery of new biologically relevant chemical matter. In natural product inspired pseudo‐NPs, NP‐derived fragments are combined de novo in unprecedented arrangements. Described here is the design and synthesis of a 155‐member pyrroquinoline pseudo‐NP collection in which fragments characteristic of the tetrahydroquinoline and pyrrolidine NP classes are combined with eight different connectivities and regioisomeric arrangements. Cheminformatic analysis and biological evaluation of the compound collection by means of phenotyping in the morphological “cell painting” assay followed by principal component analysis revealed that the pseudo‐NP classes are chemically diverse and that bioactivity patterns differ markedly, and are dependent on connectivity and regioisomeric arrangement of the fragments.

## Graphical abstract

Natural product (NP) fragments are combined in unprecedented arrangements to deliver a 155‐member pseudo‐NP collection. Cheminformatic analysis and biological evaluation of the collection by means of phenotyping in the morphological cell painting assay and principal component analysis revealed that the pseudo‐NP classes are chemically diverse with markedly different bioactivity patterns, which are dependent on connectivity and regioisomeric arrangement of the fragments.

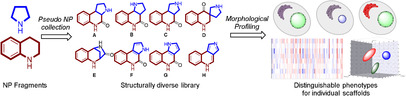

## Introduction

Biologically relevant natural products (NPs) generated through evolution have served as inspiration in the design of new bioactive small molecule classes,^[1]^ for example, in the establishment of Biology Oriented Synthesis (BIOS)^[2]^ and the complexity to diversity (CtD) approach.[^3^, ^4^] We have recently introduced the design and synthesis of pseudo natural products (pseudo‐NPs) as new strategy for the discovery of novel bioactive chemical matter.[^5^, ^6^]

Pseudo‐NPs are synthesized through de novo combinations of NP fragments^[7]^ in unprecedented arrangements. Thereby, they go beyond the chemical space explored in NP biosynthesis and may have new biological targets and modes of action. For instance, chromopynones^[8]^ and indotropane^[9]^ pseudo‐NPs are truly novel inhibitors of glucose uptake transporters GLUT1/3 and the pseudo‐NP myokinasib^[10]^ defines an unprecedented ATP competitive/mixed type kinase inhibitor chemotype.

**Figure 1:**
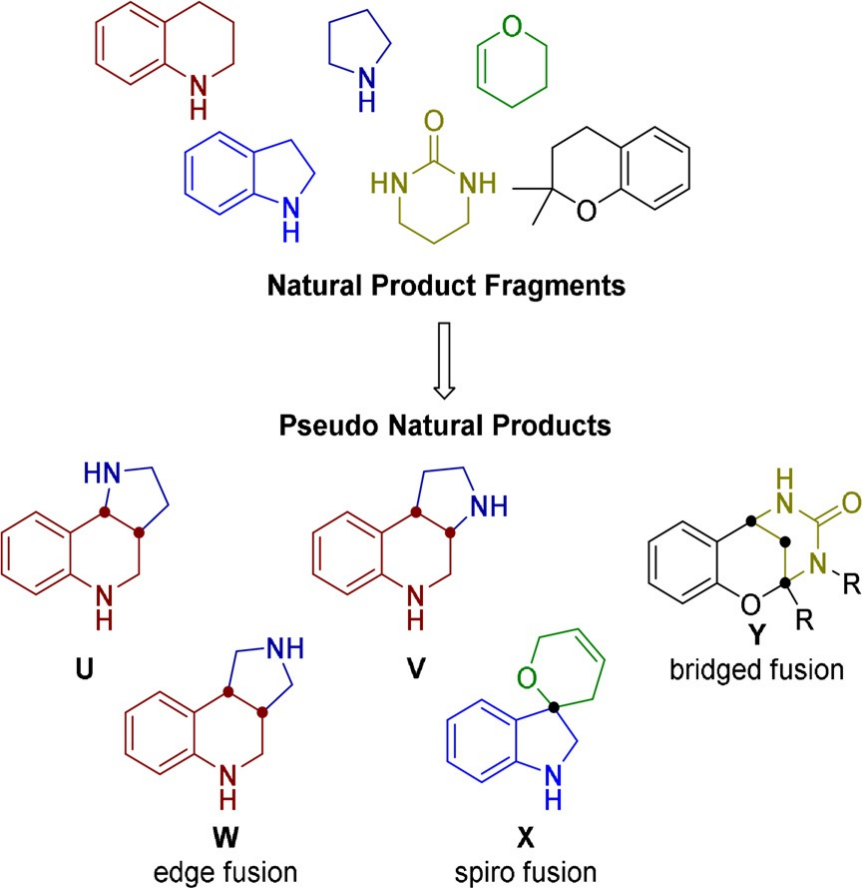
Concept and design of pseudo‐NPs. Biosynthetically unrelated NP fragments are recombined in diverse connectivities to yield a stereogenic compound collection.

In pseudo‐NP design, fragments characteristic for different NP classes are identified and combined in arrangements not accessible by biosynthesis pathways (Figure 1). These combinations may include different fragment connectivity patterns, for example, edge‐on, spiro or bridged bicyclic arrangements (Figure 1, see structures **W**, **X**, and **Y**), or the connectivity may be maintained but the fragments are combined at different regioisomeric connection points (Figure 1, see structures **U**, **V**, and **W**). This design principle will be particularly advantageous, if alternative connective or regioisomeric combinations of a given, common set of NP‐fragments will yield structurally related but still markedly different pseudo‐NP classes that will explore a wider fraction of chemical space and that may display different bioactivity patterns.

Here we provide proof‐of‐principle for this notion by means of design, synthesis, cheminformatic characterization and biological evaluation of a 155‐membered pyrroquinoline (PQ) pseudo‐NP collection which combines fragments characteristic for the biosynthetically unrelated tetrahydroquinoline‐ and pyrrolidine NP classes in eight different connectivities and regioisomeric arrangements. The library synthesis includes the discovery of a new methodology for oxidative cycloadditions to quinolinium salts. Cheminformatic analysis and biological evaluation of the compound collection by means of phenotyping in the morphological “cell painting” assay[^11^, ^12^, ^13^, ^14^, ^15^, ^16^, ^17^, ^18^, ^19^] revealed that the pseudo‐NP sub‐classes are chemically and biologically diverse. The NP‐likeness^[20]^ and bioactivity patterns differ markedly between the eight scaffolds, and depend on connectivity and regioisomeric arrangement of the fragments in the isomeric pseudo‐NP classes.

## Results and Discussion

### Design of Pyrroquinoline Pseudo Natural Products

**Figure 2:**
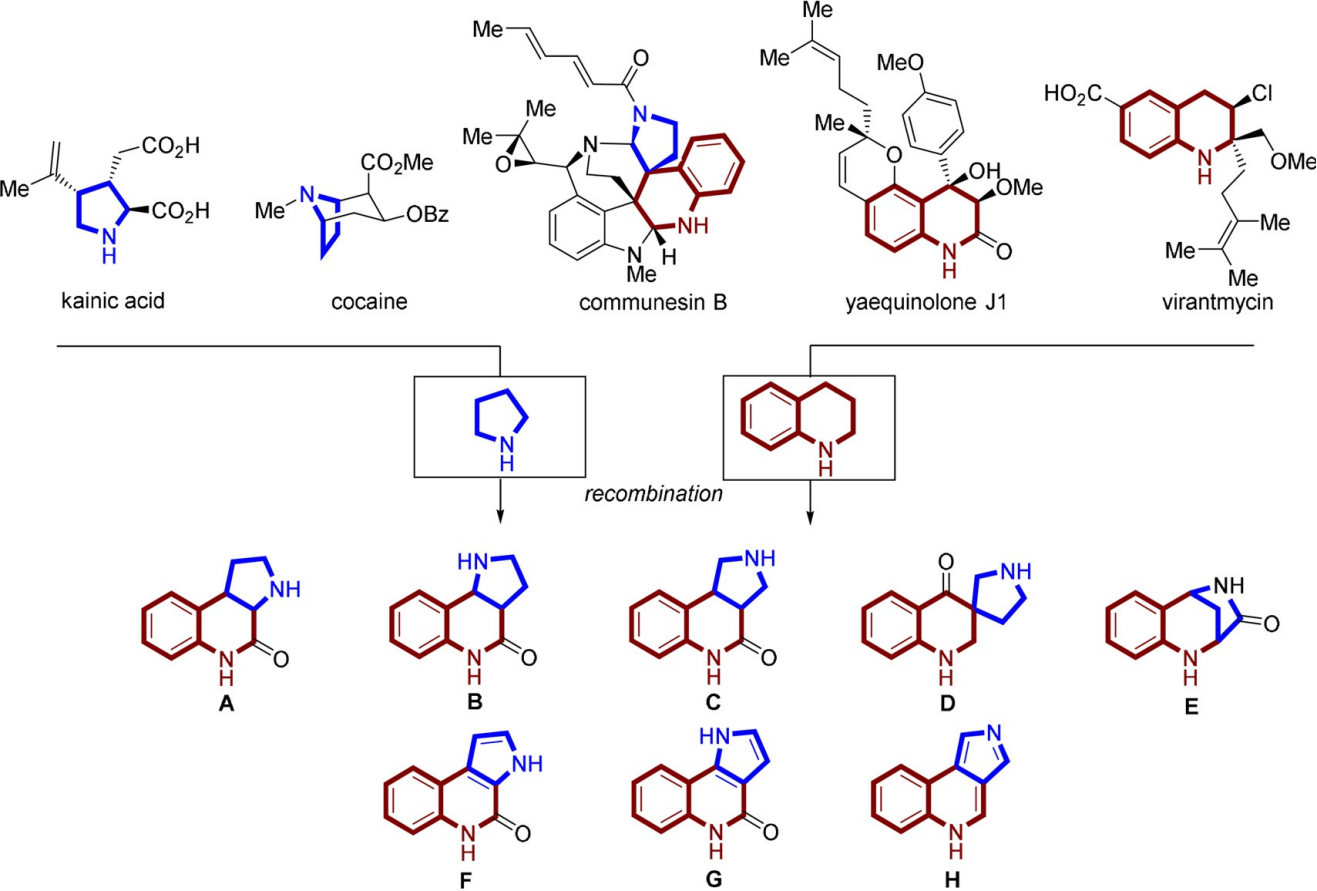
Design of pyrroquinoline (PQ) pseudo‐NPs. Diverse connectivities (edge, spiro and bridged fusion) and saturation states lead to eight pseudo‐NP classes **A**–**H**.

Pyrrolidine and tetrahydroquinoline are two fragments found in a variety of biologically active NPs (Figure 2). For instance, the pyrrolidine containing alkaloid kainic acid is a potent neuroexcitatory amino acid^[21]^ and cocaine is a triple reuptake inhibitor frequently used as a recreational drug.^[22]^ The tetrahydroquinoline containing NP yaequinolone J1 displayed insecticidal properties^[23]^ and virantmycin has been identified as a potent antiviral antibiotic.^[24]^ Pyrrolidine and tetrahydroquinoline fragments are rarely combined in natural products. A spiro fusion of these two fragments can be found for instance in communesin B, a cytotoxic marine fungal metabolite^[25]^ (for further examples see Figure S1 in the Supporting Information). Unprecedented recombinations of these two fragments may, therefore, result in the discovery of novel chemotypes with diverse biological activities.

We designed and synthesized eight different PQ scaffolds with the aim to cover variations in three‐dimensional arrangement and physiochemical properties. Pyrrolidine and tetrahydroquinoline were combined via edge fusion to the regioisomeric scaffolds **A**, **B** and **C** (Figure 2). Variation in the three‐dimensional arrangement of the fragments was obtained through a spiro fusion (scaffold **D**) and by forming the bridged bicyclic scaffold **E**.

Aromatization is a biomimetic transformation for natural products^[26]^ but only limited systematic studies on the differences in biological activity between three‐dimensional scaffolds and their two‐dimensional counterparts have been performed. Accordingly, the edge fused sp^3^‐rich PQs (scaffolds **A**, **B**, **C**) were also converted to their corresponding aromatic scaffold types **F**, **G** and **H**. A structurally diverse pyrroquinoline compound library was obtained containing members with different three‐dimensional and regioisomeric arrangements as well as saturation states.

### Synthesis of Pyrroquinolines

**Scheme 1:**
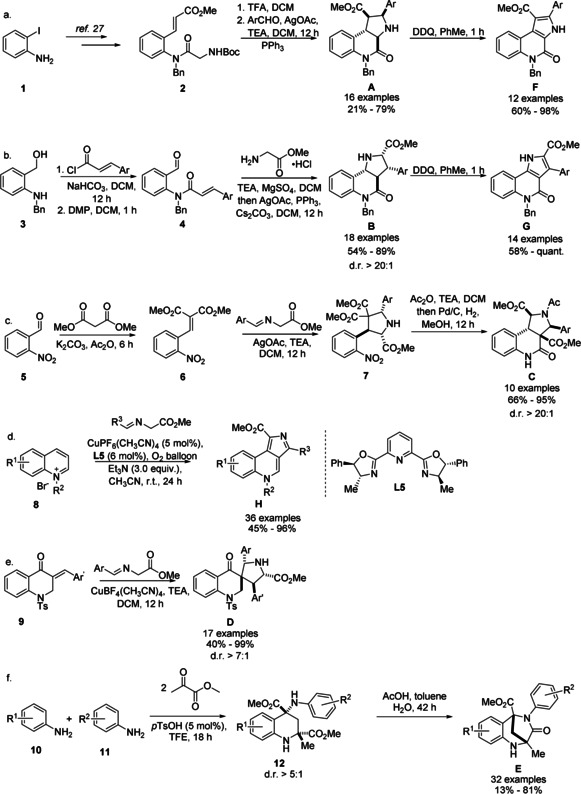
Synthesis of PQs. a,b) Scaffolds **A**, **B** were synthesized through intramolecular cycloadditions and were oxidized to their unsaturated structures **F**, **G** using DDQ. c, d) Scaffolds **C**, **D** were synthesized through intermolecular cycloaddition reactions. e) Scaffold **H** was synthesized via a Cu^I^‐catalyzed 1,3‐dipolar cycloaddition of quinolinium salts. f) A Mannich/Friedel–Crafts reaction cascade followed by intramolecular amidation afforded scaffold **D**.

1,3‐Dipolar cycloadditions were employed as powerful methods for the synthesis of several PQ scaffold libraries.^[27]^ Scaffold **A** was synthesized through an intramolecular cycloaddition of an in situ generated azomethine ylide according to a method previously published^[28]^ (Scheme 1 a). Subsequent DDQ oxidation of the obtained edge fused PQs **A** afforded the aromatized compounds **F**. A similar strategy was employed to access PQs with scaffold **B**. Amidation of aniline **3** with cinnamoyl chloride followed by alcohol oxidation afforded benzaldehydes **4** which served as precursors for an intramolecular cycloaddition (Scheme 1 b). Imine formation followed by a Ag^I^‐catalyzed cycloaddition afforded a collection of PQs **B** in good yields and with excellent diastereoselectivity. The reaction tolerated a variety of functional groups at the phenyl moiety. The unsaturated derivatives **G** were accessed through DDQ oxidation of the obtained PQs **B**.

A dual cyclisation strategy led to the synthesis of edge fused scaffold **C**. 1,3‐Dipolar cycloaddition of alkylidene malonate **6** with azomethine ylides afforded pyrrolidines **7** (Scheme 1 c). The cycloaddition tolerated electron‐donating as well as electron‐withdrawing functional groups on the arene moiety. In a one‐pot procedure, pyrrolidines **7** were first acetylated and then submitted to hydrogenation followed by an intramolecular amidation to afford PQs **C** in moderate yield as single diastereoisomers. Acetylation proved to be crucial to stabilize the cyclized products.

For the synthesis of PQs **H**, a dearomative 1,3‐dipolar cycloaddition of azomethine ylides with quinolinium salts was developed (Scheme 1 d; Table S1). Despite extensive investigations of 1,3‐dipolar cycloadditions, only a few reports using quinolinium salts as dipolarophiles are available.^[29]^ We discovered that under the catalysis of Cu(CH_3_CN)_4_PF_6_, the cycloaddition took place on the C3‐C4 edge of the quinolinium salt. As exemplified for compound **H1**, in situ oxidation using oxygen afforded the products in viable yields (Table S1, entry 5). Subsequent screening of common ligands employed in Cu‐O_2_ chemistry^[30]^ revealed that use of ligand **L5** increased the conversion considerably affording product **H1** in 89 % yield (Table S1, entry 10).

Exploration of the scope for azomethine ylides revealed that electron‐donating and electron‐withdrawing groups are tolerated on the arene moiety, including the sterically hindered *ortho*‐bromo‐substituted azomethine ylide leading to the formation of cycloadduct **H13** in 62 % yield (Figure S2), whose structure was unambiguously confirmed by X‐ray crystallographic analysis (Figure S8). However, only moderate yields were obtained with *ortho* ether‐containing substrates (**H15**, **H16**). A wide range of substituents on the benzyl group of the quinolinium salt could be included, thus affording the products (**H23**–**H27**) in good to excellent yields. The reaction further tolerated substitutions at the C6 position, but not on the C5 or C2 position of the quinolinium salts. In summary, a mild and concise dearomative 1,3‐dipolar cycloaddition for rapid generation of scaffold **H** was established.

Spirocycles **D** were synthesized through cycloadditions between (*E*)‐3‐benzylidene‐2,3‐dihydroquinolin‐4‐one **9** and azomethine ylides under Cu(CH_3_CN)_4_BF_4_ catalysis (Scheme 1 e) in good to excellent yields and diastereoselectivity. An alternative synthesis starting from the Boc‐protected dihydroquinolinone and subsequent removal of said protecting group allowed the separation of the diastereoisomers (see the Supporting Information). For biological investigations (see below) diastereomeric mixtures as well as the separated main diastereoisomers were used.

The scaffold of the bridged PQ **E** was formed through an intramolecular amidation reaction from tetrahydroquinolines (THQs) **12** (Scheme 1 f). Means to access these intermediates have previously been reported by Luo and Huang, but favor the *cis*‐diastereoisomer,^[31]^ which cannot undergo the desired amidation in the presence of electron‐neutral or ‐withdrawing substituents. We found that through the combination of acidic protic solvents, like trifluoroethanol (TFE), and catalytic amounts of *p*‐toluene sulfonic acid the desired THQs **12** could be obtained with good *trans*‐diastereoselectivity from anilines and methyl pyruvate (Table S2). An acid mediated intramolecular amidation reaction subsequently furnished the desired PQs **E** in good yields. Anilines with substituents in 3‐ and 4‐position were well tolerated by the reaction, while in the presence of substituents in the 2‐ and 5‐positions tetrahydroquinoline formation did not occur.

For each scaffold we prepared examples bearing different electron‐rich, electron‐poor, or electron‐neutral substituents in various positions. In total 155 compounds were synthesized for subsequent cheminformatic and biological analysis (Figure S3). For the analysis of the assembled collection, we wanted to answer (i) whether the combination of the tetrahydroquinoline and pyrrolidine fragments results in biologically active entities, (ii) if different connectivity patterns, regioisomers and saturation states induce morphological changes and (iii) if the observed phenotypes are distinguishable between the compound classes.

### Cheminformatic Analysis

**Figure 3:**
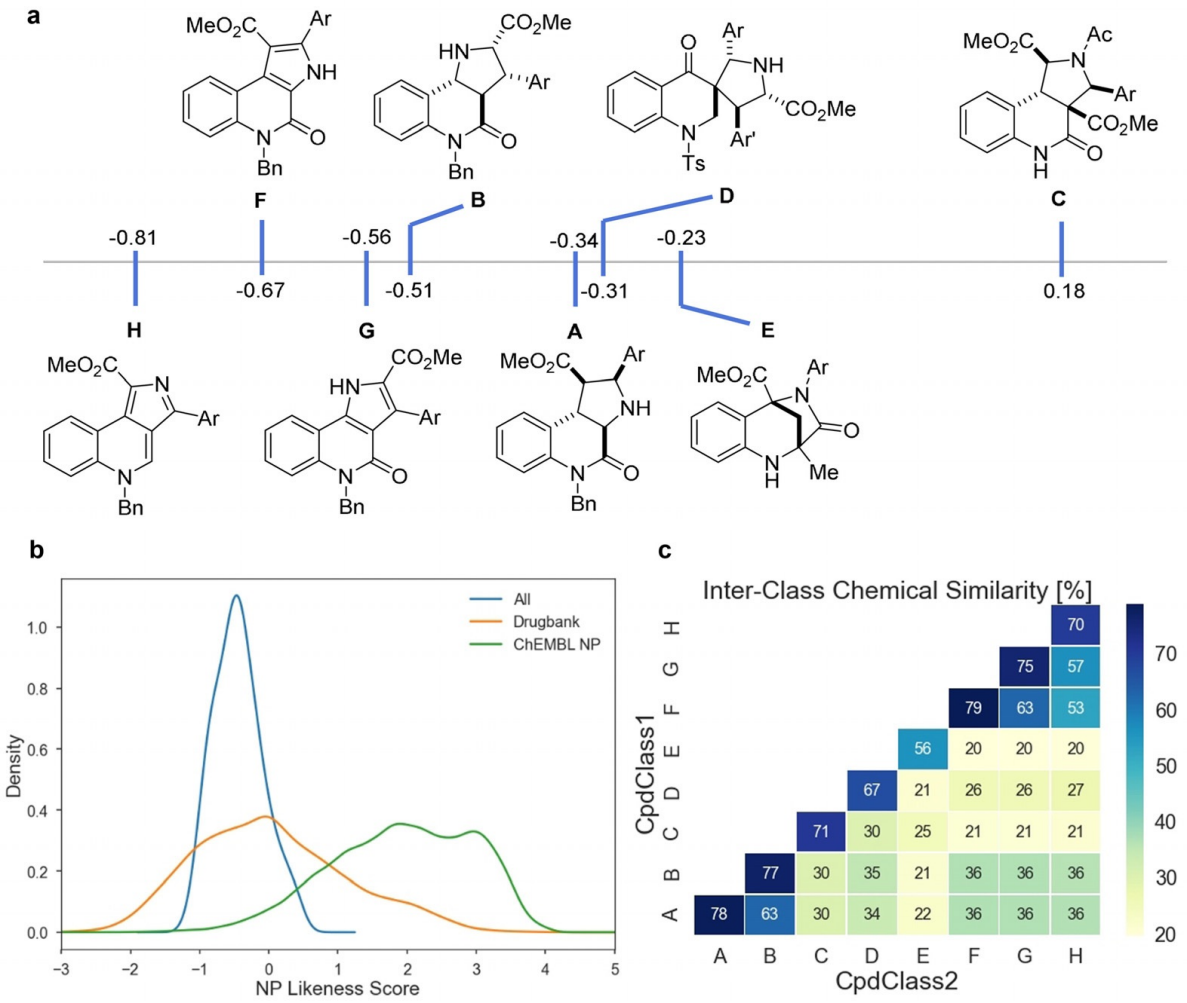
a) Average NP likeness scores for PQ scaffolds. b) NP likeness scores of PQs compared to compounds from Drugbank and the ChEMBL database. c) Chemical similarities of the different PQ scaffold libraries calculated from the Tanimoto index.

For characterization of the PQ scaffolds, their NP likeness score^[20]^ was computed using the open‐source software RDKit.^[32]^ The NP likeness score compares the frequency of a certain fragment found in natural products with the frequency of occurrence in commercial compounds. A score higher than 0 indicates that the fragments are more common in natural products. The highest average NP likeness score was calculated for scaffold **C** (Figure 3 a). The planar scaffolds **G**, **H** and **F** scored the lowest average values. Interestingly, scaffold **B** and its unsaturated analogue **G** obtained similar values and showed very similar distributions (Figure 3 a; Figure S4). For the other scaffolds, the saturated compounds (scaffolds **A** and **C**) scored higher than their unsaturated derivatives (scaffolds **F** and **H**). No dependency of the NP likeness score on the connectivity was observed with the highest and lowest scoring scaffolds (**C** and **H**) being analogues differing in saturation. The members of the spirocyclic collection with scaffold **D** and bridged PQs **E** covered a large area of NP likeness scores indicating a large dependency of the score on the varied aryl groups (Figure S4). We also compared the NP likeness scores of PQs to those of approved and experimental drugs obtained from Drugbank^[33]^ and NPs from the ChEMBL26 database.^[34]^ PQs have similar NP likeness scores as the compounds in Drugbank but only little overlap with NPs (Figure 3 b). This trend was previously observed with pyrano‐furo‐pyridone pseudo‐NPs^[16]^ and highlights the strength of the pseudo‐NP principle to yield compounds outside NP chemical space.^[5]^ Nevertheless, the combination of unrelated natural product fragments results in compounds which display a high similarity to therapeutically relevant molecules. We calculated the chemical similarity (Tanimoto similarity)^[35]^ between the members of the individual sub‐libraries based on their Morgan fingerprints (Figure 3 c). High intraclass similarities (mean >70 %) were observed for the edge fused scaffolds. The lower similarity (67 %) in scaffold **D** is likely due to the fact that both, tosylated and unprotected members were analyzed together. Also bridged scaffold **E** showed a low similarity within the library (56 %), which reflects the large variability in substituents and substitution patterns present. Comparison of all scaffolds showed that the chemical cross‐similarity in the scaffolds of the collection is low.

### Cell Painting Analysis

For characterization of bioactivity, the compound collection (155 compounds, 8 classes) was investigated in the cell painting assay^[14]^ at 10 μM compound concentration (Figure S3). This unbiased, morphological profiling method relies on the selective staining of cellular compartments through small molecule dyes. High‐content imaging and automated image analysis extract and quantify hundreds of cellular features. Comparison of the data obtained from compound‐treated cells with DMSO controls will highlight differences in cell‐morphologies. For the visual comparison of the data, fingerprints were assembled and the induction value (fraction of parameters that underwent significant changes compared to the DMSO control; see the SI for further information) was computed. Fingerprints have previously been used for the identification of possible mechanisms of action through comparison to compounds with annotated targets and activity.[^16^, ^17^, ^18^] We used these fingerprints and their underlying data as inputs for principal component analysis (PCA) to gauge the similarity of bioactivities within and between compound classes. This approach has previously been used by Schreiber et al. to demonstrate differences in cellular responses to stereoisomers^[36]^ and Aldrich et al. to illustrate the diverse performance of flavonoid‐inspired libraries.[^37^, ^38^]

In general, the different scaffolds induced pronounced morphological changes, which is expressed in the induction value. Most scaffolds displayed low induction values (<10 %, Table S3) at 10 μM compound concentration except for the unsaturated PQs **H**. Members of this compound class showed strong changes in cell morphology and induction values up to 62 %. Subsequently, compounds which led to small changes in morphology (0 %< induction <15 %) were investigated at two higher concentrations (30 μM and 50 μM), and increased induction values were observed for all scaffolds except for the saturated scaffold **C**. With two exceptions members of this scaffold class showed very low induction values (<2 %) at all concentrations investigated, as opposed to the findings for the analogous unsaturated scaffold **H** (see above).

**Figure 4:**
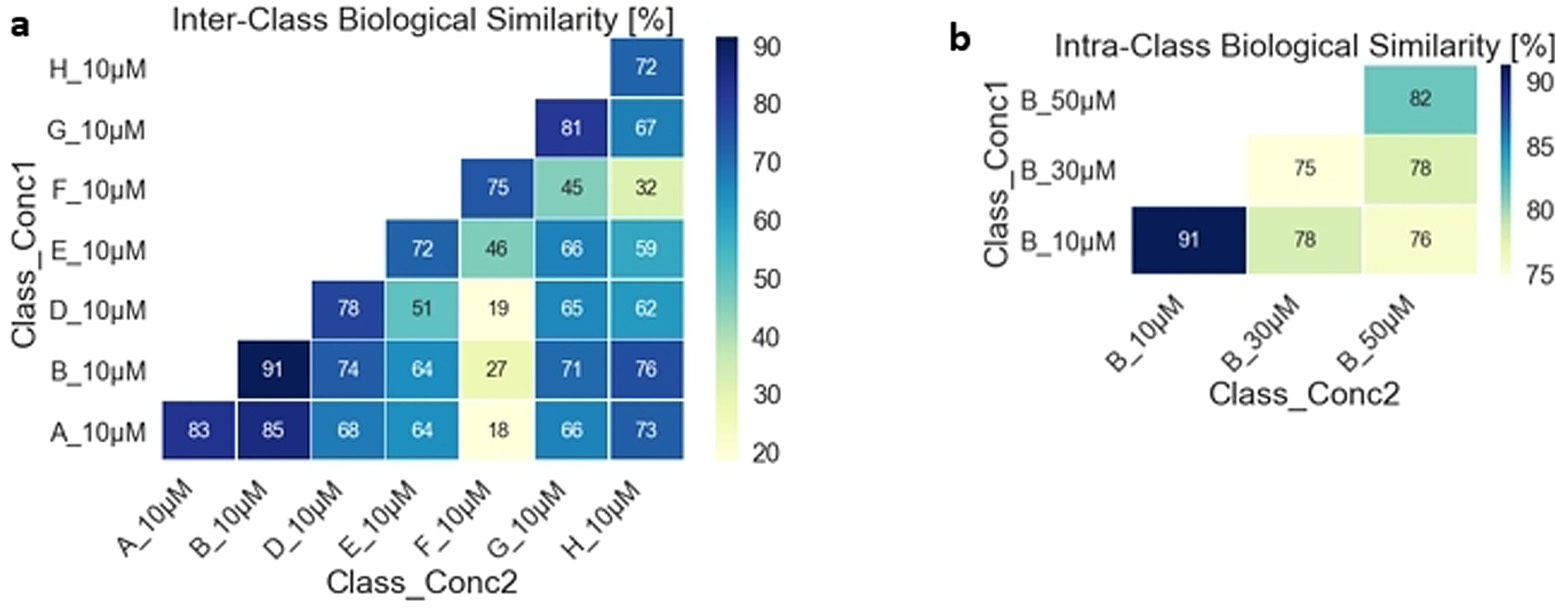
a) Biological similarity table for measurements at 10 μM calculated by Distance correlation. b) Biological similarities determined for measurements at different concentrations for scaffold **B** indicating a homogeneous cellular response at 10 μM but decreasingly so at higher concentrations.

For comparison of the obtained data, we calculated the cross‐similarities (distance correlation) between the fingerprints of individual compounds with an induction value >5 %. At 10 μM compound concentration, high similarities (>72 %) were observed within the sub‐libraries (Figure 4 a) and this trend was also apparent at higher concentrations for the edge fused scaffolds. The members of the spirocyclic scaffold **D** and bridged scaffold **E** on the other hand showed lower cross‐similarities at higher concentrations indicating a heterogeneous response of the cells to compound treatment (Figure S5a, b). Lower similarities were also found for the same scaffolds at different concentrations (Figure 4 b and Figure S5c)

For the comparison of the bioactivity profile displayed by different scaffolds we chose PCA to facilitate the characterization of the sub‐collections. In the initial attempt we observed a large dependency of the first two principal components on the induction value leading to compound clustering according to the fraction of significantly changed cellular parameters rather than the induced phenotypic changes (Figure S6). Reduction of the dataset to an induction window for better comparison revealed that within a range of 10 % (i.e. 5–15 % induction) the components of the PCA became independent of the induction value. Therefore, for further analysis measurements with an induction between 5 % and 15 % were chosen since this window represented the largest dataset accessible.

**Figure 5:**
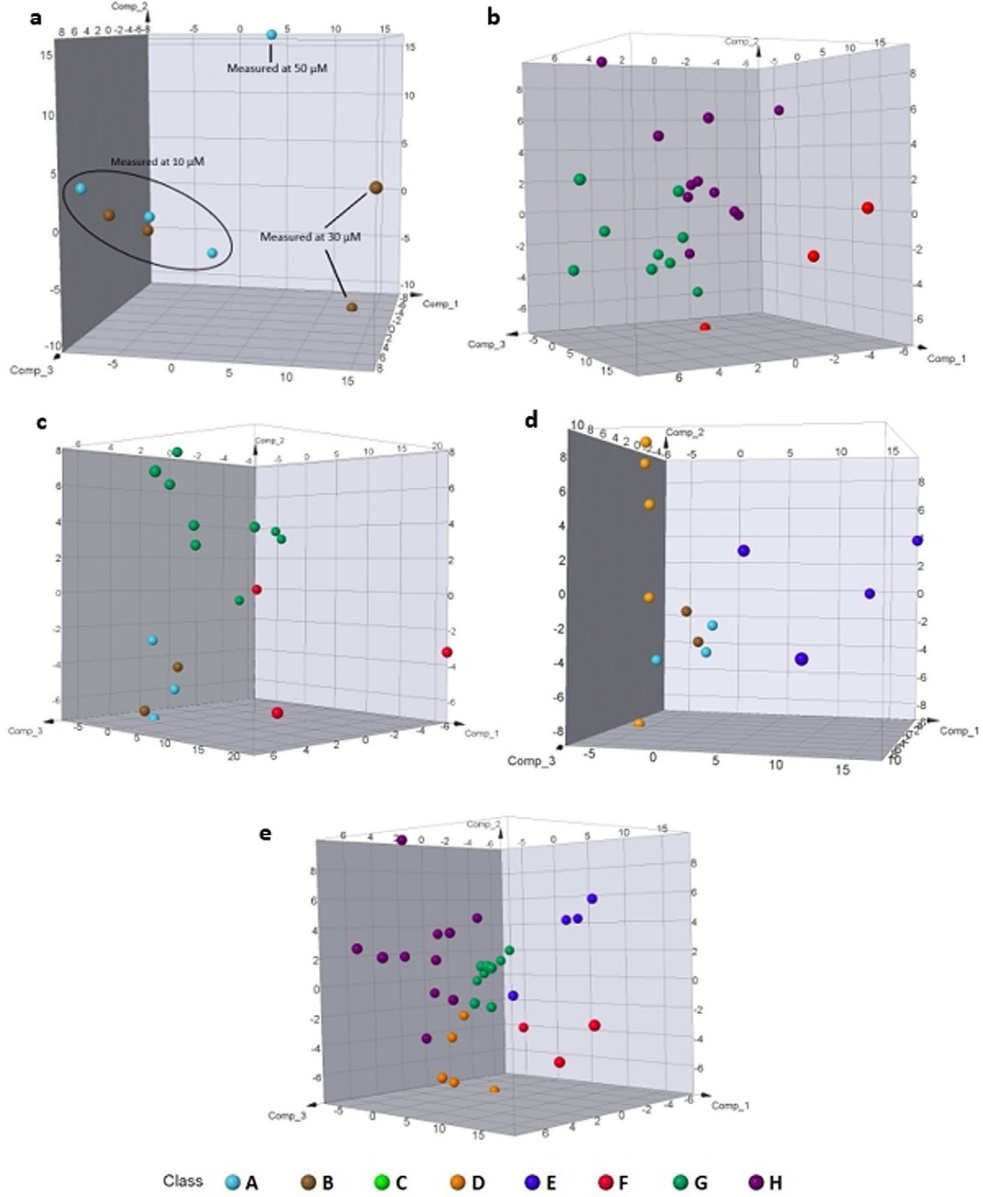
a) PCA of saturated PQ scaffolds **A** (marine) and **B** (brown) with induction 5*–*15 *%*. Explained variance: 76 *%*; b) PCA of unsaturated PQ scaffolds **F** (red), **G** (green), **H** (purple) with induction 5*–*15 *%*. Explained variance: 52 *%*; c) PCA of the saturated scaffolds **A** (marine) and **B** (brown) with their unsaturated derivatives **F** (red) and **G** (green). Explained variance: 64 *%*; d) PCA between three‐dimensional scaffolds with different connectivity. Explained variance: 51 *%*; e) PCA for the three‐dimensional scaffolds **D**, **E** (orange and blue) and the planar **F**, **G** and **H** (red, green and purple). Explained variance: 50 *%*.

Comparison of the unsaturated scaffolds **A** and **B** showed one cluster with outliers corresponding to measurements at higher concentrations than 10 μM (Figure 5 a). This observation is in line with the comparably low fingerprint similarities calculated for measurements at different concentrations (Figure 4 b; Figure S5c). To ensure homogeneity within a scaffold, only measurements at 10 μM were considered for all further PCA. Scaffold **C** was not included in this analysis since only one compound had an induction value >5 %, preventing conclusive judgements. Different from their saturated derivatives, the unsaturated scaffolds **F**, **G** and **H** were readily distinguished by the PCA and populated separate spaces (Figure 5 b). None of the scaffolds formed particularly tight clusters but covered wider areas. Thus, planar PQs induced a wider range of morphological changes compared to their sp^3^‐rich counterparts.

Comparison of saturated edge fused scaffolds **A** and **B** with their unsaturated derivatives **F** and **G** (Figure 5 c) revealed that the saturated scaffolds formed a common population (marine and brown dots), which was clearly separate from scaffolds **F** (red) and **G** (green) indicating distinguishable phenotypes.

In comparing the three‐dimensional scaffolds, we observed that the edge fused scaffolds **A** and **B** again formed one cluster, while spiro‐PQs **D** and bridged PQs **E** were distinctly different (Figure 5 d, compare marine and brown dots to orange and blue). Spiro‐PQs **D** (orange dots) did not cluster with each other but showed a large variance in the second component of the PCA, while the members of the bridged scaffold **E** (blue dots) exhibited their main variance in the third component creating two sub‐populations. A similar picture emerged when the three‐dimensional spiro and bridged scaffolds **D** and **E** were compared to the planar edge fused members of scaffolds **F**, **G** and **H** (Figure 5 e). The PCA showed distinguishable morphological changes between the five scaffolds. Spiro‐PQs **D** (orange) formed a tighter cluster than observed in the previous analysis (compare Figures 5 d and e). Three distinct populations for scaffolds **F**, **G** and **H** were observed (compare the red, green and purple points), indicating distinguishable phenotypes for different connections of the tetrahydroquinoline and pyrrolidine fragments. While the members of the saturated scaffolds **F** and **H** formed wide spread populations, we observed closer clustering of scaffold **G** (compare red, green and purple clusters in Figure 5 e to b). In order to determine whether inclusion of higher induction values in this analysis would lead to different conclusions, we repeated the above‐mentioned analyses including all available measurements with an induction >5 %. Gratifyingly, this analysis arrived at the same conclusions which shows that the effect of induction on the analyses mentioned above does not dominate the conclusions (Figure S7).

## Conclusion

We have combined pyrrolidine and tetrahydroquinoline NP fragments in biosynthetically unprecedented or rarely occurring manners affording eight pyrroquinoline scaffolds. The syntheses yielded collections differing in connectivity, regioisomeric arrangement and saturation state. Inter‐ and intramolecular 1,3‐dipolar cycloadditions and cascade reactions were strategically applied to synthesize these diverse compound classes. During the synthesis, we discovered an efficient dearomative 1,3‐dipolar cycloaddition to access pyrroquinoline scaffold **H** from quinolinium salts. The different scaffolds show NP likeness scores more similar to drugs than to NPs and are structurally diverse. This analysis indicates that the PQs synthesized according to the design principles proposed for pseudo natural products^[5]^ differ from NPs in terms of their structure but may retain their biological relevance.

Pyrroquinolines **A**–**H** were characterized for different bioactivity through the cell painting assay and subsequent statistical analysis by PCA. The analysis revealed that different arrangements of the tetrahydroquinoline and pyrrolidine fragments results in pseudo‐NP classes with distinct bioactivity profiles. Phenotypic variances were observed between different connectivity types, and regioisomers of the edge fused unsaturated PQs were clearly distinguished from each other. The saturated examples formed a common population. Our findings highlight that combination of a common set of NP‐fragments in different arrangements may yield chemically diverse compound collections based on natural product structure, retained biological relevance and with diversity in biological performance.

## Conflict of interest

The authors declare no conflict of interest.

## Supporting information

As a service to our authors and readers, this journal provides supporting information supplied by the authors. Such materials are peer reviewed and may be re‐organized for online delivery, but are not copy‐edited or typeset. Technical support issues arising from supporting information (other than missing files) should be addressed to the authors.

Supplementary
