# Supplementary material for: Design, Synthesis, and Biological Evaluation of Chemically and Biologically Diverse Pyrroquinoline Pseudo Natural Products
Source: Angew Chem Int Ed Engl. 2021 Jan 12;60(9):4648–56. doi: 10.1002/anie.202013731 (PMC7986669; doi:10.1002/anie.202013731)
Supplement: Supplementary file 1 — Supplementary [file ANIE-60-4648-s001.pdf]

Angewandte Chemie
Eine Zeitschrift der Gesellschaft Deutscher Chemiker

Supporting Information

Design, Synthesis, and Biological Evaluation of Chemically and Biologically Diverse Pyrroquinoline Pseudo Natural Products

*Jie Liu[+], Gregor S. Cremosnik[+], Felix Otte, Axel Pahl, Sonja Sievers, Carsten Strohmann, and Herbert Waldmann**

anie_202013731_sm_miscellaneous_information.pdf

Tables of Content

Supplementary Figures and Tables 2
Cell painting methodology 23
Synthesis of pyrroquinoline pseudo natural products 26
- General Information 26
- Synthesis of pyrroquinolines A 27
- Synthesis of pyrroquinolines B 36
- Synthesis of pyrroquinolines C 52
- Synthesis of pyrroquinolines D 61
- Synthesis of pyrroquinolines E 72
- Synthesis of pyrroquinolines F 87
- Synthesis of pyrroquinolines G 93
- Synthesis of pyrroquinolines H 99
- References 114
- NMR spectra of synthetic precursors 115
- NMR spectra for pyrroquinolines A-H 149

Full spectra of representative scaffolds 321

Supplementary Figures and Tables

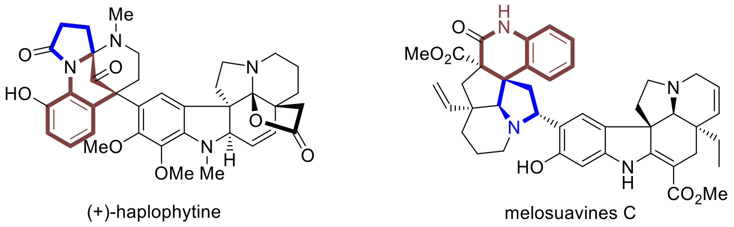

Figure S1. Representative natural products containing pyrrolidine and tetrahydroquinoline fragments.

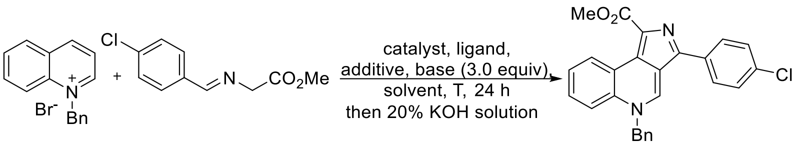

Entry	Catalyst	Ligand	Additive	T [°C]	Yield[c]
1[a]	$CuPF_6(CH_3CN)_4$	-	-	r.t.	49%
2[a]	$CuPF_6(CH_3CN)_4$	-	O_2	40 °C	73%
3[a]	$CuPF_6(CH_3CN)_4$	-	Pd/C[d]	40 °C	79%
4[a]	$CuPF_6(CH_3CN)_4$	-	DDQ[e]	40 °C	76%
5[b]	$CuPF_6(CH_3CN)_4$	-	O_2	r.t.	71%
6[b]	$CuPF_6(CH_3CN)_4$	L1	O_2	r.t.	77%
7[b]	$CuPF_6(CH_3CN)_4$	L2	O_2	r.t.	84%
8[b]	$CuPF_6(CH_3CN)_4$	L3	O_2	r.t.	81%
9[b]	$CuPF_6(CH_3CN)_4$	L4	O_2	r.t.	80%
10[b]	$CuPF_6(CH_3CN)_4$	L5	O_2	r.t.	89%
11[b]	-	-	O_2	r.t.	64%

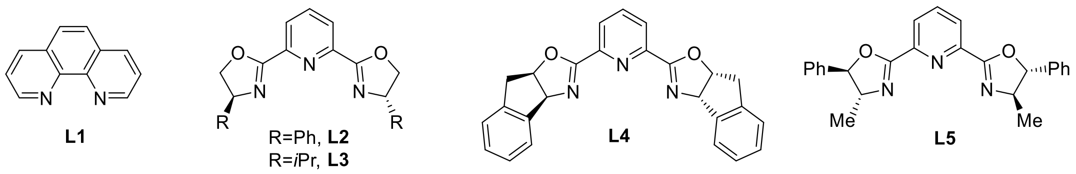

Table S1. Screening of conditions for dearomative 1,3-dipolar cycloaddition of quinolinium salts and tandem aerobic oxidation. [a] quinolinium salt (1.0 equiv), iminoester (1.5 equiv), catalyst (15 mol%), reaction scale 0.20 mmol, CH_3CN 2.0 mL. [b] quinolinium salt (1.0 equiv), iminoester (1.5 equiv), catalyst (5.0 mol%), ligand (6.0 mol%), reaction scale 0.10 mmol, CH_3CN 1.0 mL. [c] isolated yield. [d] 25 mol%. [e] 3.0 equiv.

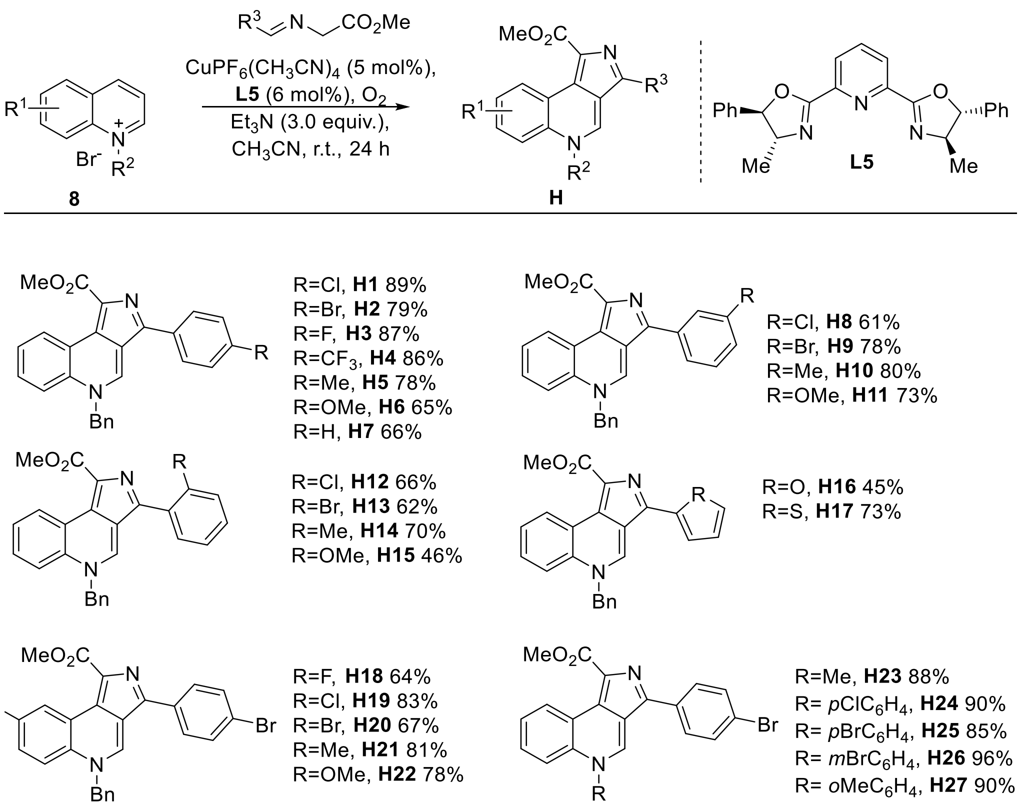

Figure S2. Substrate scope of the dearomative 1,3-dipolar cycloaddition of quinolinium salts.

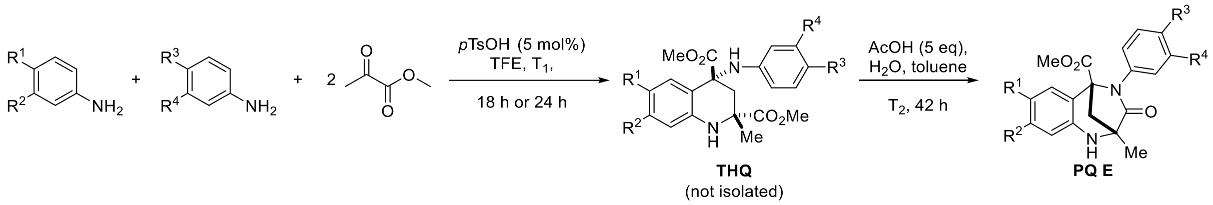

No.	R_1	R_2	R_3	R_4	R_5	T_1 [°C]	THQ d.r. (*trans*:*cis*)	T_2 [°C]	Yield
**E1**	Cl	H	Cl	H	H	r.t.	16:1	110	70%
**E2**	F	H	F	H	H	r.t.	20:1	110	61%
**E3**	Br	H	Br	H	H	r.t.	>90:1	110	71%
**E4**	I	H	I	H	H	r.t.	14:1	110	26%
**E5**	CF_3	H	CF_3	H	H	r.t.	8:1	110	50%
**E6**	H	H	H	H	H	5	7:1	50	43%
**E7**	Me	H	Me	H	H	5	11:1	50	81%
**E8**	Et	H	Et	H	H	5	11:1	50	55%
**E9**	*t*Bu	H	*t*Bu	H	H	5	10:1	50	63%
**E10**	*i*Pr	H	*i*Pr	H	H	5	11:1	50	48%
**E11**	OMe	H	OMe	H	H	0	5:1	50	53%
**E12**	O*i*Pr	H	O*i*Pr	H	H	5	6:1	50	55%
**E13**	OCF_3	H	OCF_3	H	H	5	9:1	50	31%
**E14**	H	F	H	F	H	5	11:1	50	47%
**E15**	H	Br	H	Br	H	5	12:1	90	13%
**E16**	H	CF_3	H	CF_3	H	r.t.	8:1	110	54%
**E17**	H	Et	H	Et	H	5	9:1	50	53%
**E18**	H	H	CF_3	H	H	5	14:1	90	37%
**E19**	Me	H	Br	H	H	5	18:1	90	53%

Table S2. Substrate scope of the concise synthesis of scaffold **E**. [a]: d.r. was calculated based on the crude ^{1}HNMR.

No.	R_1	R_2	R_3	R_4	R_5	T_1 [°C]	THQ d.r. (*trans*:*cis*)	T_2 [°C]	Yield
**E20**	*t*Bu	H	F	H	H	5	15:1	90	39%
**E21**	Et	H	Cl	H	H	5	13:1	90	41%
**E22**	H	Me	H	Br	H	5	n.d.	90	22%
**E23**	Me	H	Cl	CF_3	H	5	10:1	90	43%
**E24**	H	Br	Cl	CF_3	H	r.t.	15:1	90	60%
**E25**	Me	Br	Cl	H	H	r.t.	15:1	90	29%
**E25**	Me	Br	CF_3	H	H	5	12:1	90	31%
**E27**	Me	Br	H	CF_3	H	5	16:1	90	30%
**E28**	Me	Br	Me	Br	H	5	14:1	50	54%
**E29**	Cl	CF_3	Cl	CF_3	H	r.t.	5:1	110	24%
**E30**	Me	Br	Cl	CF_3	H	5	16:1	90	48%

Table S2 (continued). Substrate scope of the concise synthesis of scaffold **E**. [a]: d.r. was calculated based on the crude ^{1}HNMR.

Scaffold A

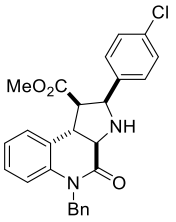

A1

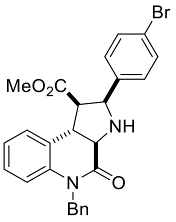

A2

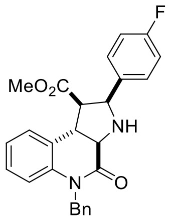

A3

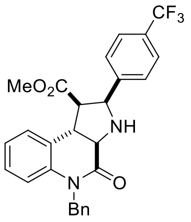

A4

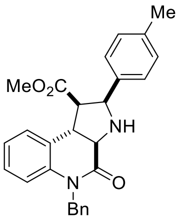

A5

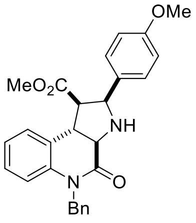

A6

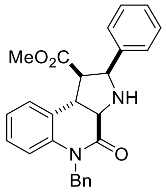

A7

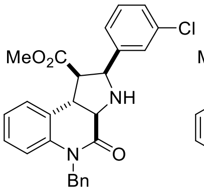

A8

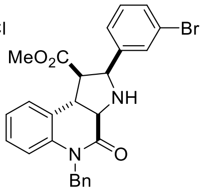

A9

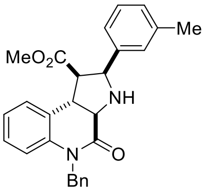

A10

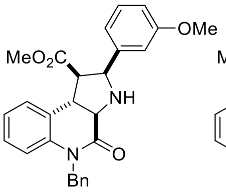

A11

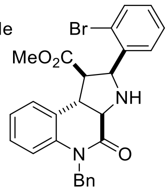

A12

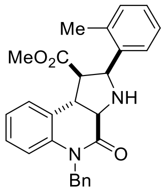

A13

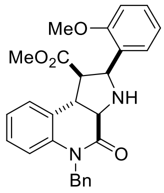

A14

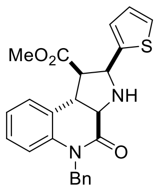

A15

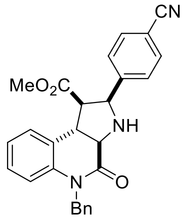

A16

Figure S3: Compounds included in the 155-membered pyrroquinoline pseudo natural product collection. Scaffold **A**: 16 members; Scaffold **B**: 18 members; Scaffold **C**: 10 members; Scaffold **D**: 17 members; Scaffold **E**: 32 members; Scaffold **F**: 12 members; Scaffold **G**: 14 members; Scaffold **H**: 36 members.

Scaffold B

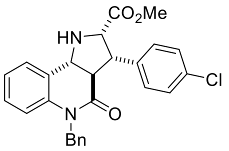
B1

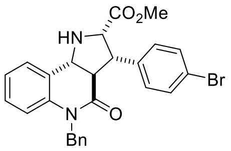
B2

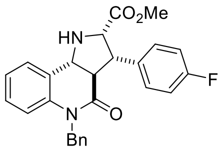
B3

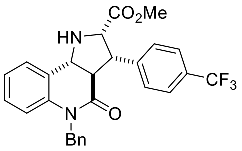
B4

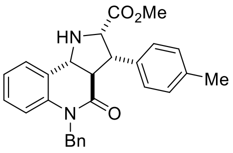
B5

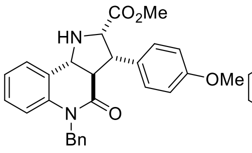
B6

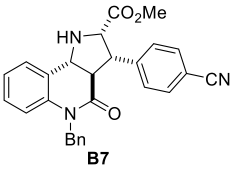
B7

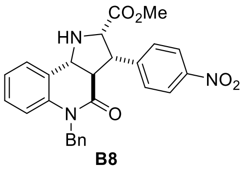
B8

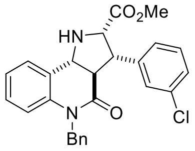
B9

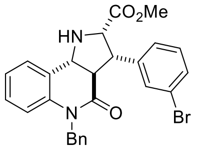
B10

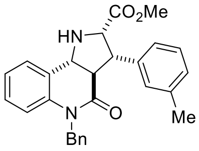
B11

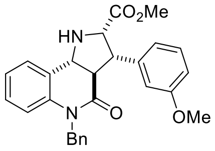
B12

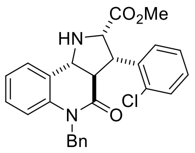
B13

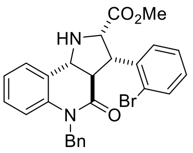
B14

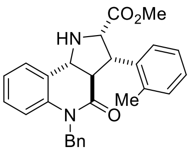
B15

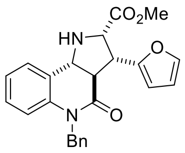
B16

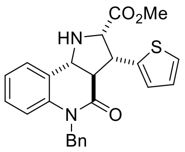
B17

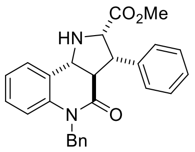
B18

Scaffold C

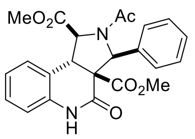
C1

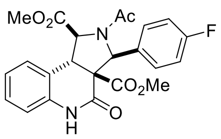
C2

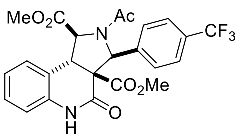
C3

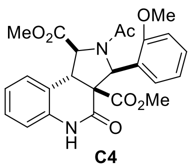
C4

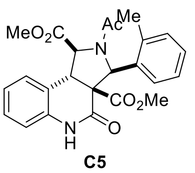
C5

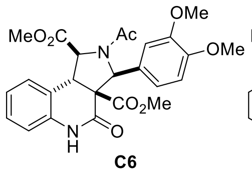
C6

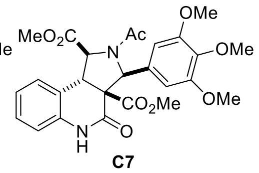
C7

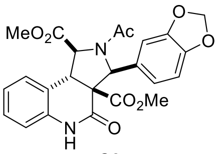
C8

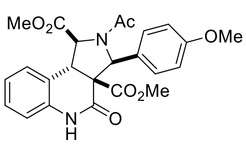
C9

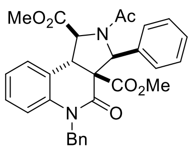
C10

Figure S3 (continued): Compounds included in the 155-membered pyrroquinoline pseudo natural product collection. Scaffold **A**: 16 members; Scaffold **B**: 18 members; Scaffold **C**: 10 members; Scaffold **D**: 17 members; Scaffold **E**: 32 members; Scaffold **F**: 12 members; Scaffold **G**: 14 members; Scaffold **H**: 36 members.

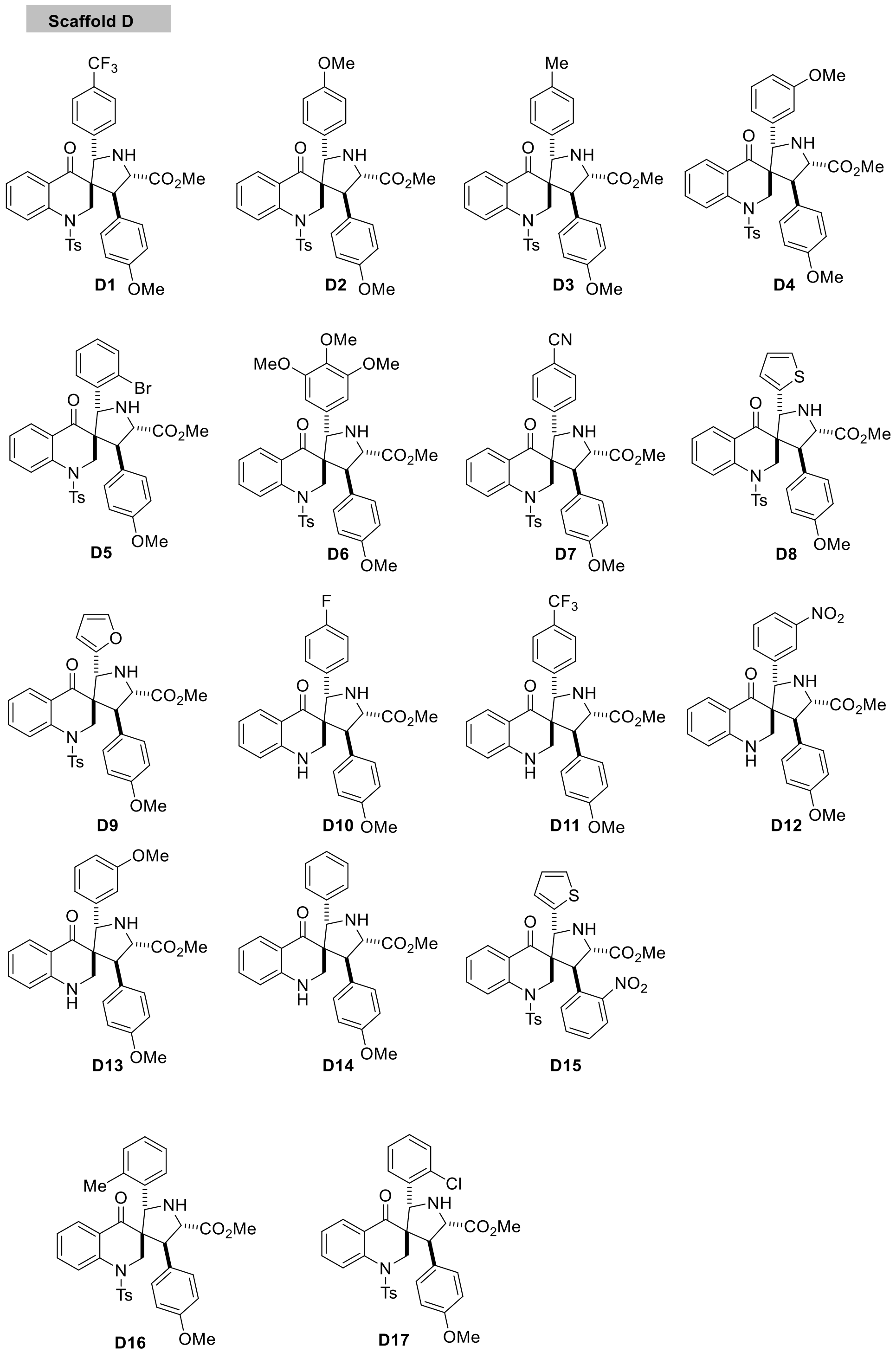

Figure S3 (continued): Compounds included in the 155-membered pyrroquinoline pseudo natural product collection. Scaffold **A**: 16 members; Scaffold **B**: 18 members; Scaffold **C**: 10 members; Scaffold **D**: 17 members; Scaffold **E**: 32 members; Scaffold **F**: 12 members; Scaffold **G**: 14 members; Scaffold **H**: 36 members.

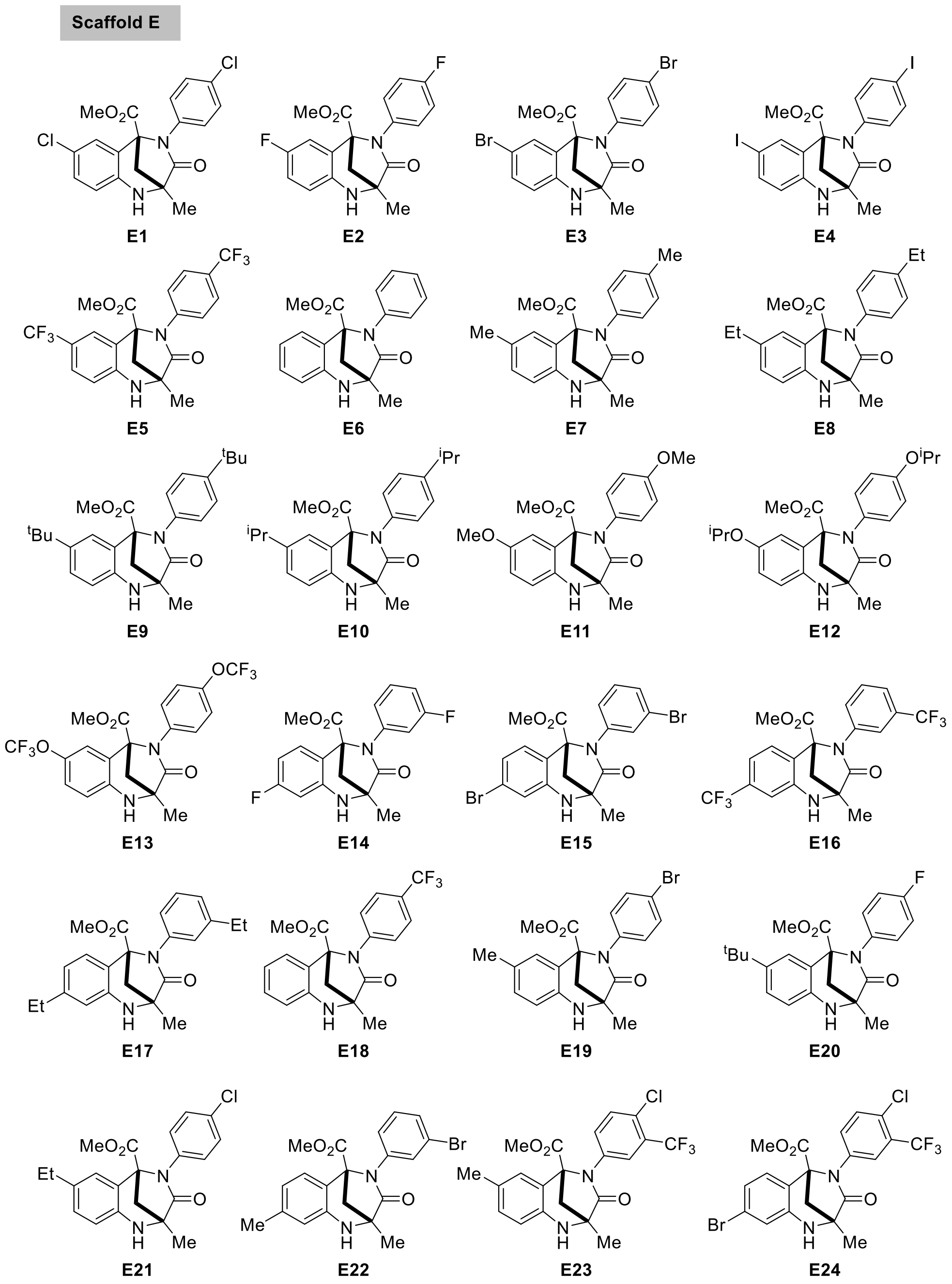

Figure S3 (continued): Compounds included in the 155-membered pyrroquinoline pseudo natural product collection. Scaffold **A**: 16 members; Scaffold **B**: 18 members; Scaffold **C**: 10 members; Scaffold **D**: 17 members; Scaffold **E**: 32 members; Scaffold **F**: 12 members; Scaffold **G**: 14 members; Scaffold **H**: 36 members.

Scaffold E (continued)

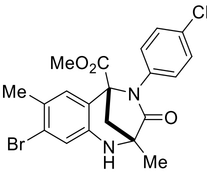

E25

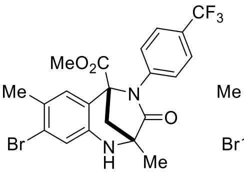

E26

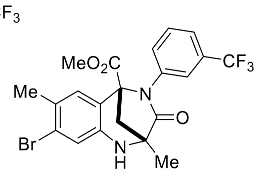

E27

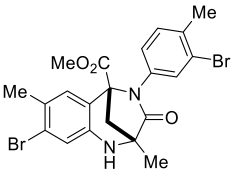

E28

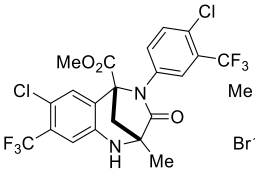

E29

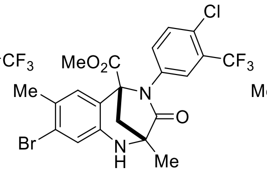

E30

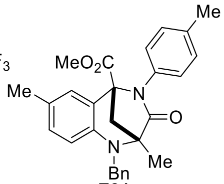

E31

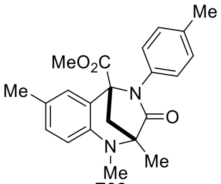

E32

Scaffold F

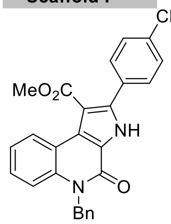

F1

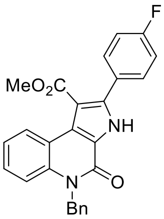

F2

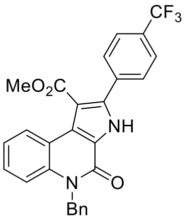

F3

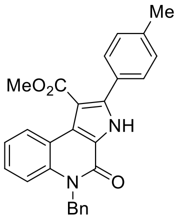

F4

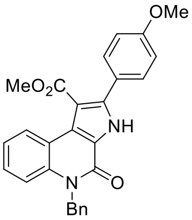

F5

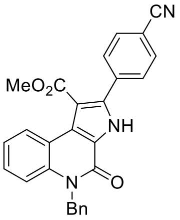

F6

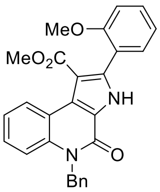

F7

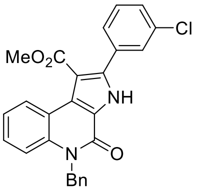

F8

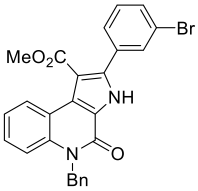

F9

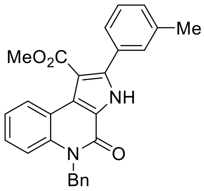

F10

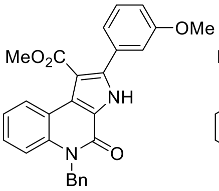

F11

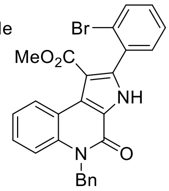

F12

Figure S3 (continued): Compounds included in the 155-membered pyrroquinoline pseudo natural product collection. Scaffold **A**: 16 members; Scaffold **B**: 18 members; Scaffold **C**: 10 members; Scaffold **D**: 17 members; Scaffold **E**: 32 members; Scaffold **F**: 12 members; Scaffold **G**: 14 members; Scaffold **H**: 36 members.

Scaffold G

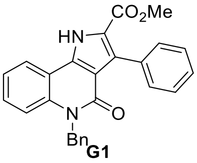
G1

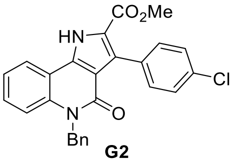
G2

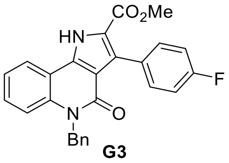
G3

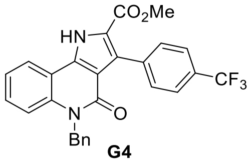
G4

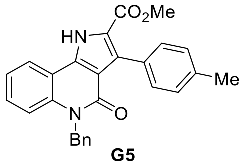
G5

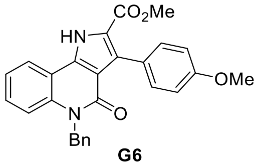
G6

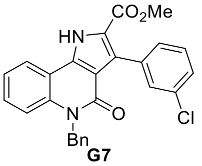
G7

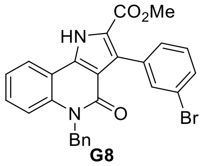
G8

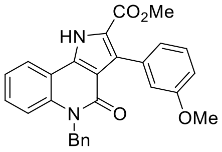
G9

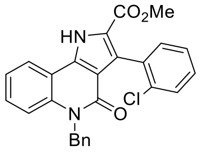
G10

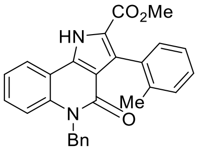
G11

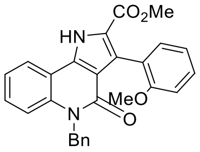
G12

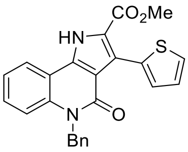
G13

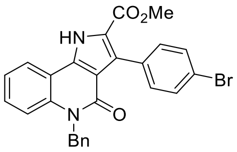
G14

Scaffold H

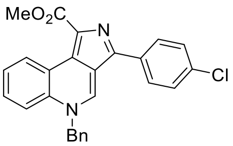
H1

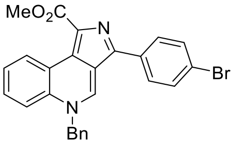
H2

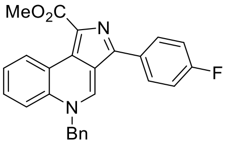
H3

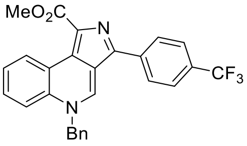
H4

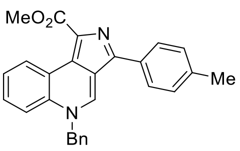
H5

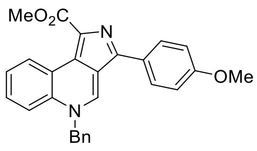
H6

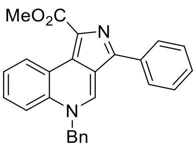
H7

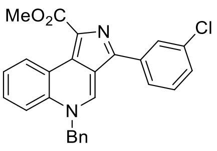
H8

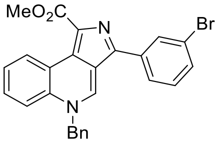
H9

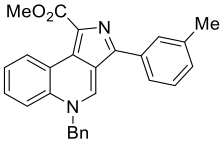
H10

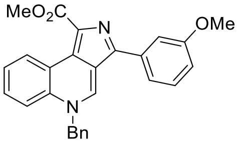
H11

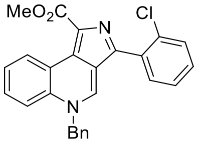
H12

Figure S3 (continued): Compounds included in the 155-membered pyrroquinoline pseudo natural product collection. Scaffold **A**: 16 members; Scaffold **B**: 18 members; Scaffold **C**: 10 members; Scaffold **D**: 17 members; Scaffold **E**: 32 members; Scaffold **F**: 12 members; Scaffold **G**: 14 members; Scaffold **H**: 36 members.

Scaffold H (continued)

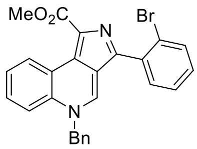
H13

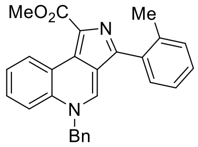
H14

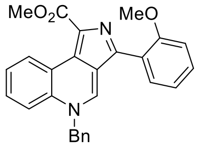
H15

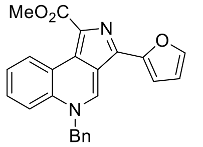
H16

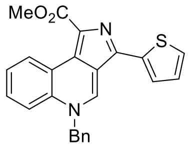
H17

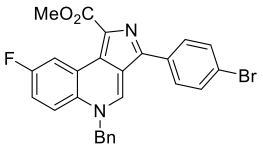
H18

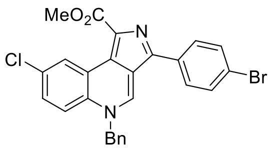
H19

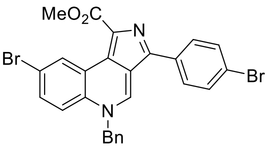
H20

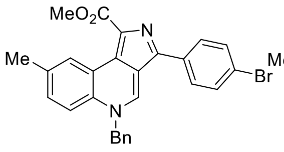
H21

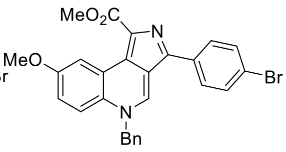
H22

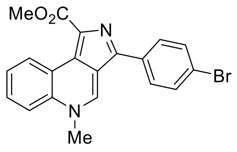
H23

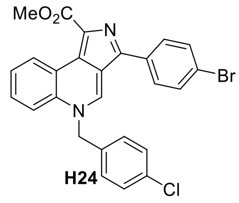
H24

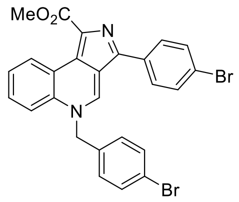
H25

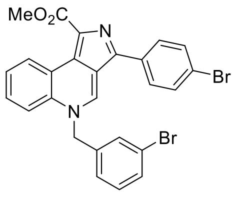
H26

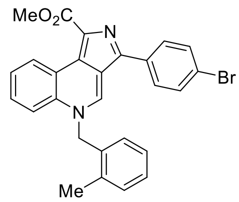
H27

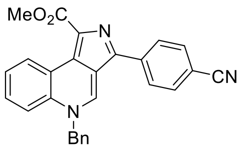
H28

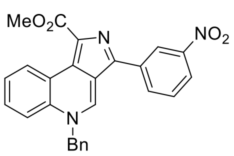
H29

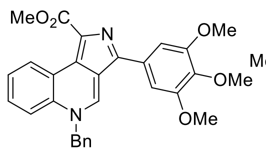
H30

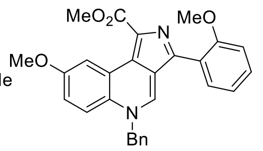
H31

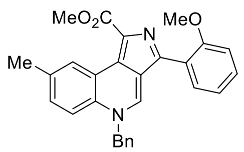
H32

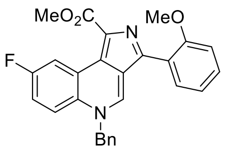
H33

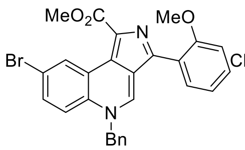
H34

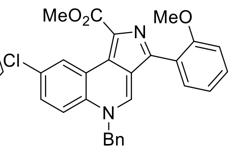
H35

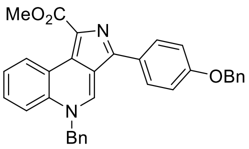
H36

Figure S3 (continued): Compounds included in the 155-membered pyrroquinoline pseudo natural product collection. Scaffold **A**: 16 members; Scaffold **B**: 18 members; Scaffold **C**: 10 members; Scaffold **D**: 17 members; Scaffold **E**: 32 members; Scaffold **F**: 12 members; Scaffold **G**: 14 members; Scaffold **H**: 36 members.

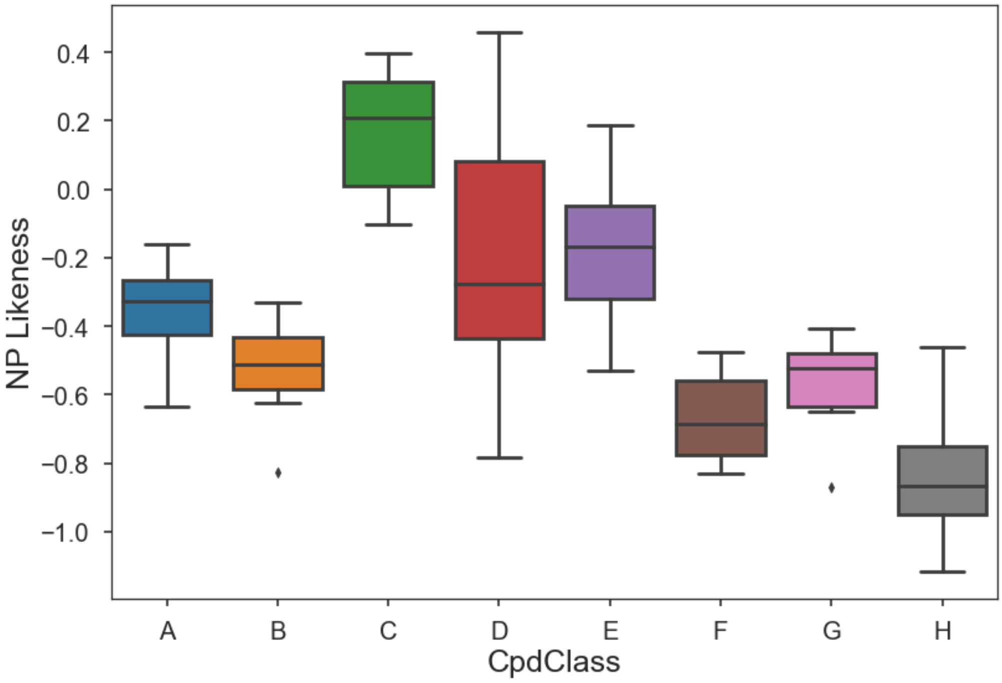

Figure S4. Individual distributions of the NP likeness scores for the eight PQ scaffolds. Spirocycle **D** and bridged bicycle **E** covered a large area of NP likeness scores. Sp^3-rich scaffolds **A-E** gave higher NP likeness scores than the planar structures **F**, **G** and **H**.

No.	Induction @ 10 µM	Induction @ 30 µM	Induction @ 50 µM	*No.*	Induction @ 10 µM	Induction @ 30 µM	Induction @ 50 µM
**A1**	1	28	54	**A9**	6	27	34
**A2**	6	37	64	**A10**	2	31	37
**A3**	0	22	34	**A11**	1	29	34
**A4**	0	69	87	**A12**	1	30	33
**A5**	1	31	40	**A13**	1	29	32
**A6**	1	36	48	**A14**	2	28	36
**A7**	1	18	20	**A15**	0	20	12
**A8**	7	28	16	**A16**	2	n.d.	n.d.
**B1**	2	29	31	**B10**	3	32	34
**B2**	0	27	31	**B11**	0	28	42
**B3**	0	19	24	**B12**	0	8	14
**B4**	7	32	66	**B13**	4	38	51
**B5**	1	37	30	**B14**	10	41	41
**B6**	0	23	25	**B15**	2	55	45
**B7**	2	16	21	**B16**	0	9	13
**B8**	4	52	29	**B17**	0	12	23
**B9**	4	25	47	**B18**	0	18	24
**C1**	0	0	0	**C6**	2	0	1
**C2**	0	0	1	**C7**	0	0	1
**C3**	0	0	1	**C8**	1	0	0
**C4**	0	n.d.	n.d.	**C9**	0	0	2
**C5**	0	1	0	**C10**	0	22	29
**D1**	5	5	2	**D10**	n.d.	17	23
**D2**	8	3	4	**D11**	0	24	12
**D3**	n.d.	4	6	**D12**	0	16	14
**D4**	4	14	3	**D13**	1	8	30
**D5**	n.d.	1	3	**D14**	1	13	30
**D6**	5	5	2	**D15**	8	n.d.	n.d.
**D7**	1	24	9	**D16**	3	n.d.	n.d.
**D8**	8	9	4	**D17**	3	2	2
**D9**	0	12	5				

Table S3. Induction values for PQs at different concentrations. Most scaffolds displayed low induction values (<10%) at 10 µM compound concentration with the exception of the unsaturated PQs **H**.

No.	Induction @ 10 µM	Induction @ 30 µM	Induction @ 50 µM	*No.*	Induction @ 10 µM	Induction @ 30 µM	Induction @ 50 µM
**E1**	0	1	1	**E17**	0	n.d.	n.d.
**E2**	0	n.d.	n.d.	**E18**	0	8	2
**E3**	0	n.d.	n.d.	**E19**	0	n.d.	n.d.
**E4**	0	n.d.	64	**E20**	0	n.d.	n.d.
**E5**	0	66	72	**E21**	0	n.d.	n.d.
**E6**	0	n.d.	n.d.	**E22**	1	16	11
**E7**	0	2	3	**E23**	1	n.d.	n.d.
**E8**	0	n.d.	n.d.	**E24**	9	n.d.	n.d.
**E9**	0	n.d.	n.d.	**E25**	3	n.d.	n.d.
**E10**	2	30	30	**E26**	3	n.d.	n.d.
**E11**	0	n.d.	n.d.	**E27**	9	n.d.	n.d.
**E12**	0	n.d.	n.d.	**E28**	4	61	71
**E13**	0	0	2	**E29**	7	91	92
**E14**	0	n.d.	n.d.	**E30**	0	2	3
**E15**	1	24	47	**E31**	2	26	25
**E16**	0	3	12	**E32**	1	3	6
**F1**	1	n.d.	n.d.	**F7**	10	61	66
**F2**	0	n.d.	n.d.	**F8**	0	26	25
**F3**	0	n.d.	2	**F9**	4	5	7
**F4**	0	50	64	**F10**	0	10	19
**F5**	1	n.d.	9	**F11**	10	59	66
**F6**	0	52	71	**F12**	2	25	39

Table S3 (continued). Induction values for PQs at different concentrations. Most scaffolds displayed low induction values (<10%) at 10 µM compound concentration with the exception of the unsaturated PQs **H**.

No.	**Induction @ 10 µM**	**Induction @ 30 µM**	**Induction @ 50 µM**	*No.*	**Induction @ 10 µM**	**Induction @ 30 µM**	**Induction @ 50 µM**
**G1**	0	23	27	**G8**	7	n.d.	n.d.
**G2**	10	33	23	**G9**	12	15	28
**G3**	25	64	49	**G10**	9	18	21
**G4**	11	20	26	**G11**	8	23	29
**G5**	8	14	12	**G12**	11	31	19
**G6**	15	21	12	**G13**	5	32	27
**G7**	5	9	10	**G14**	2	15	6
**H1**	4	n.d.	n.d.	**H19**	5	n.d.	n.d.
**H2**	16	n.d.	n.d.	**H20**	19	n.d.	n.d.
**H3**	8	n.d.	n.d.	**H21**	39	n.d.	n.d.
**H4**	24	n.d.	n.d.	**H22**	10	73	75
**H5**	36	n.d.	n.d.	**H23**	39	n.d.	n.d.
**H6**	26	n.d.	n.d.	**H24**	26	n.d.	n.d.
**H7**	6	n.d.	n.d.	**H25**	20	n.d.	n.d.
**H8**	5	58	56	**H26**	20	n.d.	n.d.
**H9**	23	n.d.	n.d.	**H27**	21	n.d.	n.d.
**H10**	18	n.d.	n.d.	**H28**	8	n.d.	n.d.
**H11**	19	n.d.	n.d.	**H29**	11	n.d.	n.d.
**H12**	3	n.d.	n.d.	**H30**	31	n.d.	n.d.
**H13**	9	n.d.	n.d.	**H31**	46	n.d.	n.d.
**H14**	5	70	88	**H32**	43	n.d.	n.d.
**H15**	25	n.d.	n.d.	**H33**	26	n.d.	n.d.
**H16**	2	n.d.	n.d.	**H34**	14	83	81
**H17**	11	n.d.	n.d.	**H35**	27	n.d.	n.d.
**H18**	13	n.d.	n.d.	**H36**	62	n.d.	n.d.

Table S3 (continued). Induction values for PQs at different concentrations. Most scaffolds displayed low induction values (<10%) at 10 µM compound concentration with the exception of the unsaturated PQs **H**.

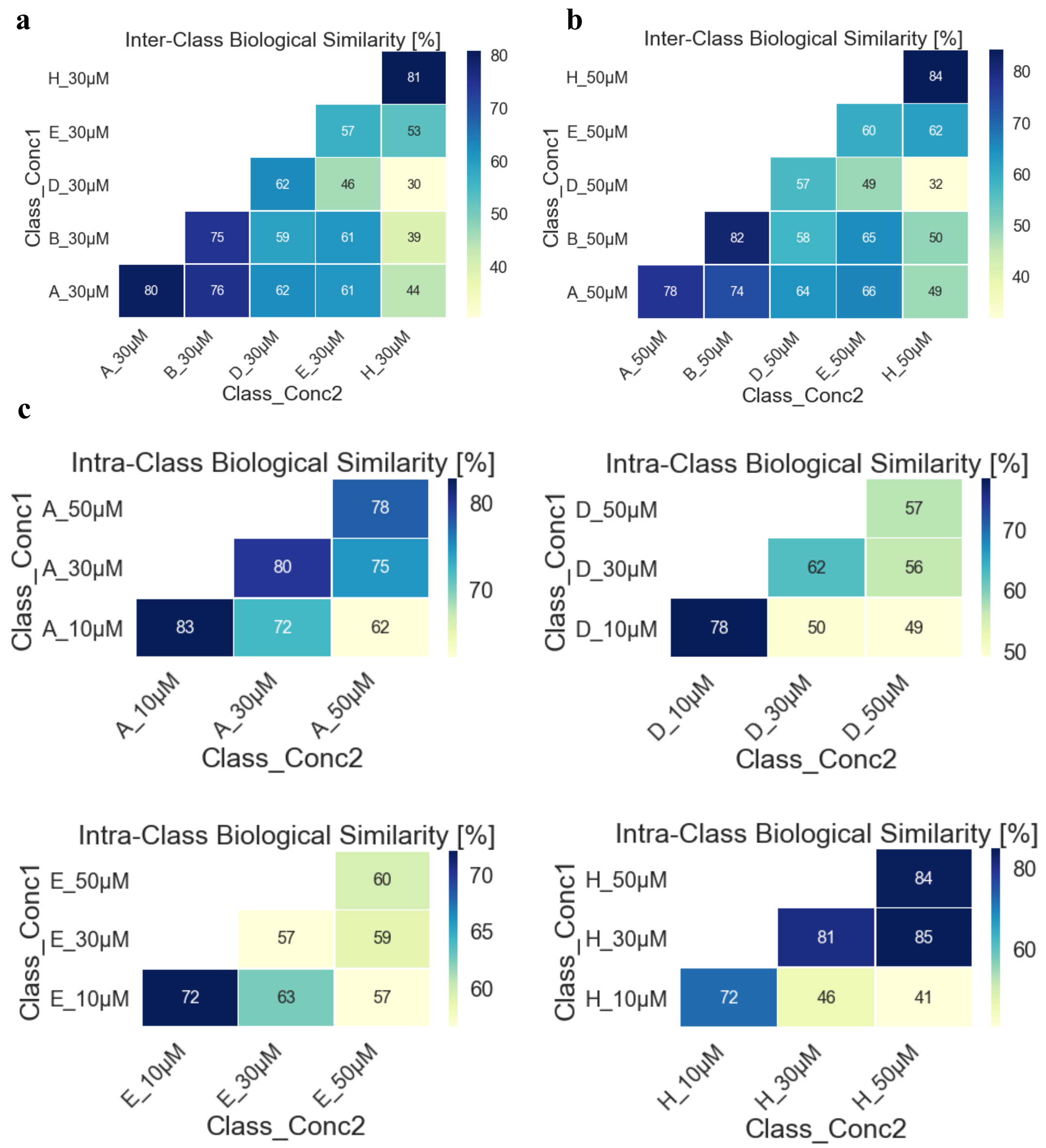

Figure S5. **a**, **b**: Biosimilarities between different scaffolds at high concentrations. **c**: Biosimilarities within one scaffold at different concentrations.

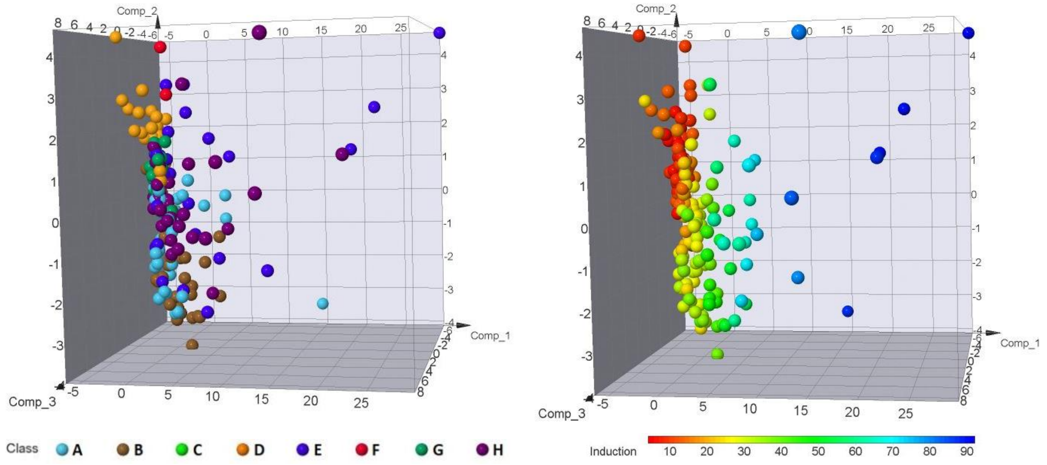

Figure S6. PCA including all available measurements. A clear clustering according to the induction parameter was observed.

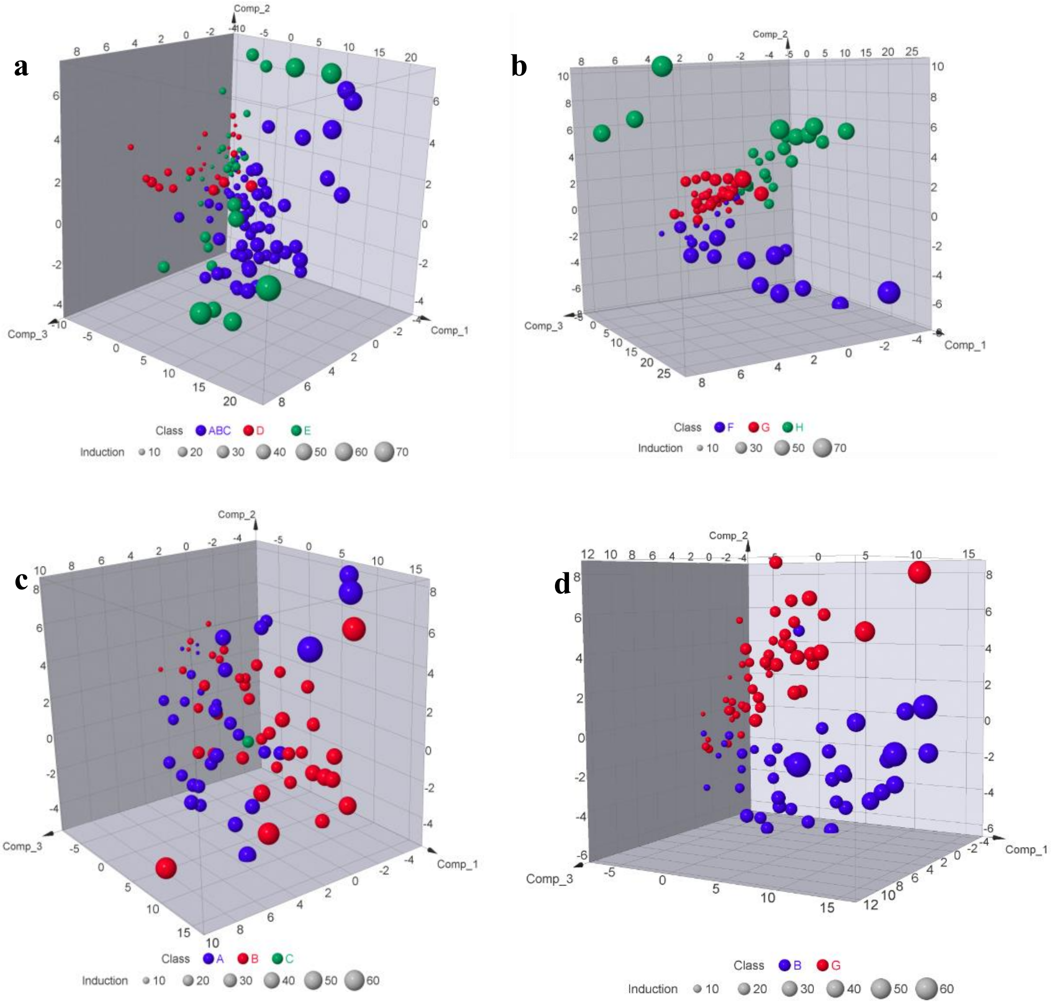

Figure S7. PCA using measurements with induction values > 5%. **a**: PCA of the three-dimensional scaffolds with different connectivities. Explained variance: 62%. **b**: PCA of the unsaturated regioisomeric structures **F**, **G** and **H**. Explained variance: 69%. **c**: PCA of the saturated regioisomeric structures **A**, **B** and **C**. Explained variance: 59%. **d**: PCA of the saturated structures **B** with their unsaturated derivatives **G**. Explained variance: 59%.

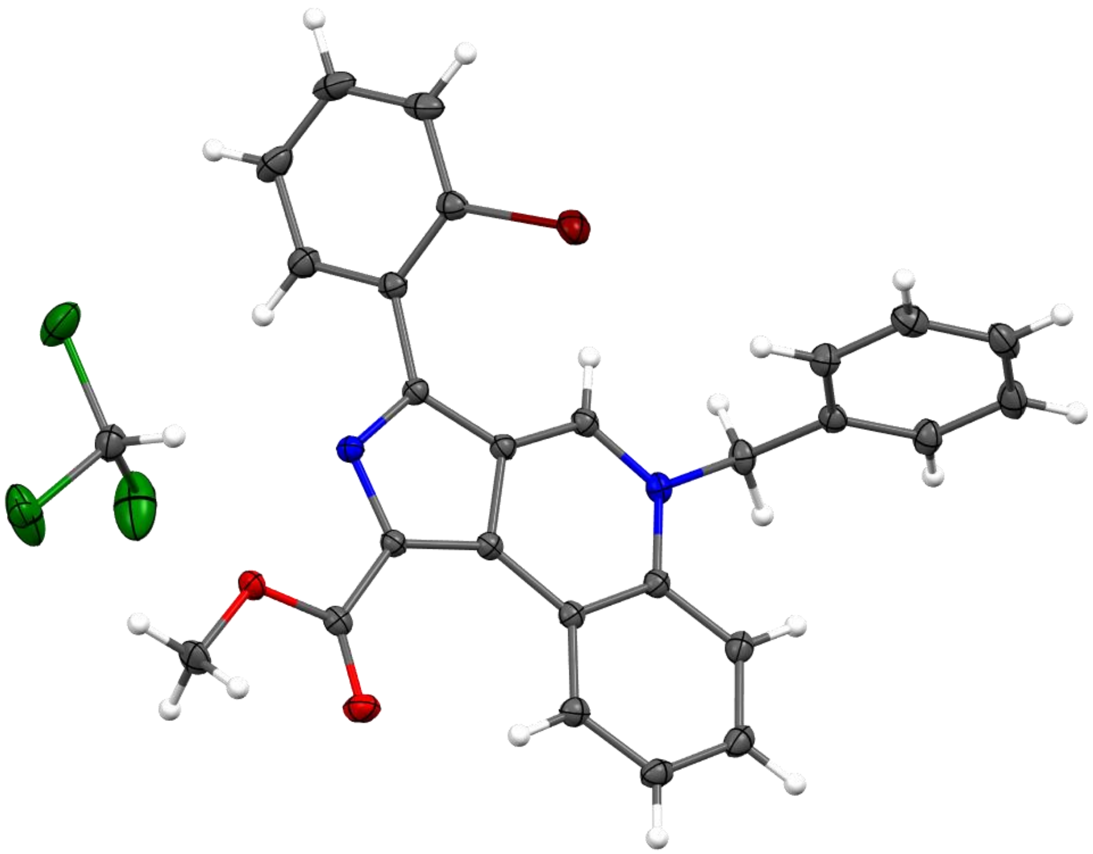

Figure S8. Crystal structure of the cycloadduct **H13**. ORTEP plot of $C_{27}H_{20}BrCl_3N_2O_2$ (M =590.71 g/mol) (**H13·CHCl$_3$**) at the 50% probability level. See Supplementary Table S4 for additional details. Crystallographic data have been deposited at the Cambridge Crystallographic Data Centre and copies can be obtained on request, free of charge, by quoting the publication citation and the deposition number CCDC 2017048.

Empirical formula	$C_{27}H_{20}BrCl_3N_2O_2$
Formula weight	590.71
Temperature/K	99.98
Crystal system	monoclinic
Space group	$P2_1/c$
a/Å	11.297(2)
b/Å	8.1074(10)
c/Å	26.975(5)
α/°	90
β/°	100.752(6)
γ/°	90
Volume/Å^3	2427.3(7)
Z	4
ρ_{calc}g/cm^3	1.616
μ/mm^{-1}	2.054
F(000)	1192
Crystal size/mm^3	0.195 × 0.139 × 0.055
Radiation	MoKα (λ = 0.71073)
2Θ range for data collection/°	5.208 to 61.996
Index ranges	$-16 \leq h \leq 16, -11 \leq k \leq 11, -39 \leq l \leq 39$
Reflections collected	132541
Independent reflections	7730 [R_{int} = 0.0407, R_{sigma} = 0.0152]
Data/restraints/parameters	7730/0/336
Goodness-of-fit on F^2	1.06
Final R indexes [I>=2σ (I)]	R_1 = 0.0310, wR_2 = 0.0791
Final R indexes [all data]	R_1 = 0.0380, wR_2 = 0.0838
Largest diff. peak/hole / e Å^{-3}	1.68/-0.80

Table S4. Crystal data and structure refinement for **H13·$CHCl_3$**.

Cell painting methodology

The described assay follows closely the method described by Bray et al[1].

Initially, 5 µl U2OS medium were added to each well of a 384-well plate (PerkinElmer CellCarrier-384 Ultra). Subsequently, U2OS cell were seeded with a density of 1600 cells per well in 20 µl medium. The plate was incubated for 10 min at the ambient temperature, followed by an additional 4 h incubation (37 °C, 5% CO_2). Compound treatment was performed with the Echo 520 acoustic dispenser (Labcyte) at final concentrations of 10 µM, 3 µM or 1 µM. Incubation with compound was performed for 20 h (37 °C, 5% CO2). Subsequently, mitochondria were stained with Mito Tracker Deep Red (Thermo Fisher Scientific, Cat. No. M22426). The Mito Tracker Deep Red stock solution (1 mM) was diluted to a final concentration of 100 nM in prewarmed medium. The medium was removed from the plate leaving 10 µl residual volume and 25 µl of the Mito Tracker solution were added to each well. The plate was incubated for 30 min in darkness (37 °C, 5% CO2). To fix the cells 7 µl of 18.5 % formaldehyde in PBS were added, resulting in a final formaldehyde concentration of 3.7 %. Subsequently, the plate was incubated for another 20 min in darkness (RT) and washed three times with 70 µl of PBS. (Biotek Washer Elx405). Cells were permeabilized by addition of 25 µl 0.1% Triton X-100 to each well, followed by 15 min incubation (RT) in darkness. The cells were washed three times with PBS leaving a final volume of 10 µl. To each well 25 µl of a staining solution were added, which contains 1% BSA, 50 µl Phalloidin (Alexa594 conjugate, Thermo Fisher Scientific, A12381), 25 µg/ml Concanavalin A (Alexa488 conjugate, Thermo Fisher Scientific, Cat. No. C11252), 50 µl/ml Hoechst 33342 (Sigma, Cat. No. B2261-25mg), 15 µl/ml WGA-Alexa594 conjugate (Thermo Fisher Scientific, Cat. No. W11262) and 0.3 µl/ml SYTO 14 solution (Thermo Fisher Scientific, Cat. No. S7576). The plate is incubated for 30 min (RT) in darkness and washed three times with 70 µl PBS. After the final washing step the PBS was not aspirated. The plates were sealed and centrifuged for 1 min at 500 rpm.

The plates were prepared in triplicates with shifted layouts to reduce plate effects and imaged using a Micro XL High-Content Screening System (Molecular Devices) in 5 channels (DAPI: Ex350-400/ Em410-480; FITC: Ex470-500/ Em510-540; Spectrum Gold: Ex520-545/ Em560-585; TxRed: Ex535-585/ Em600-650; Cy5: Ex605-650/ Em670-715) with 9 sites per well and 20x magnification (binning 2).

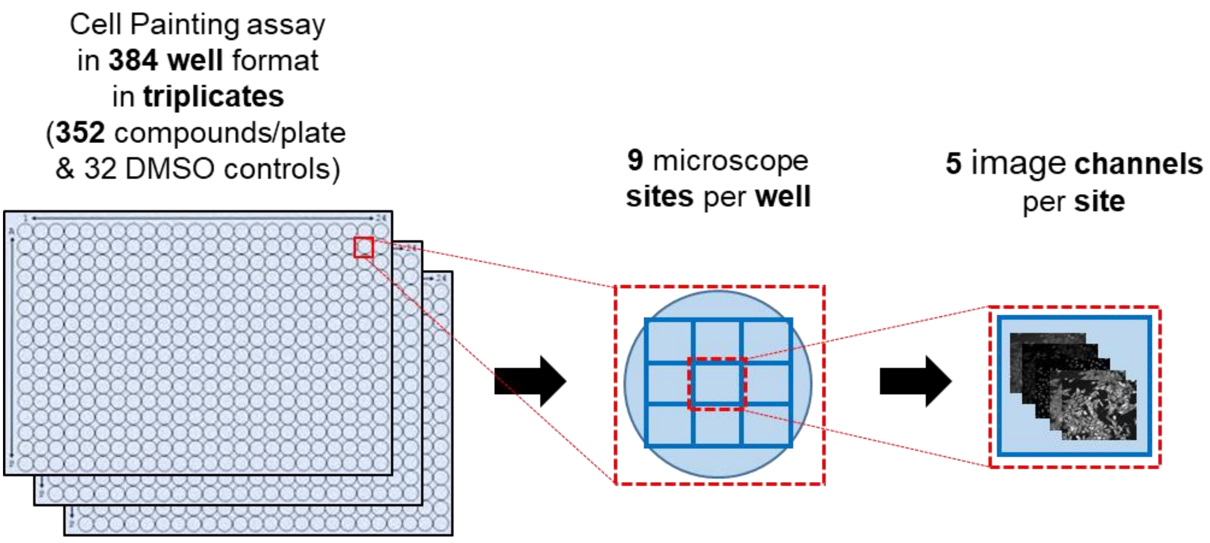

The generated images were processed with the *CellProfiler* package (https://cellprofiler.org/, version 3.0.0) on a computing cluster of the Max Planck Society to extract 1716 cell features (parameters) per

microscope site. The data was then further aggregated as medians per well (9 sites -> 1 well), then over the three replicates.

Further analysis was performed with custom *Python* (https://www.python.org/) scripts using the *Pandas* (https://pandas.pydata.org/) and *Dask* (https://dask.org/) data processing libraries as well as the *Scientific Python* (https://scipy.org/) package (separate publication to follow).

From the total set of 1716 parameters a subset of highly reproducible and robust parameters was determined using the procedure described by Woehrmann et al[2]. in the following way: Two biological repeats of one plate containing reference compounds were analysed. For every parameter, its full profile over each whole plate was calculated. If the profiles from the two repeats showed a similarity >= 0.8 (see below), the parameter was added to the set.

This procedure was only performed once and resulted in a set of 579 robust parameters out of the total of 1716 that was used for all further analyses.

Determination of reproducible Parameters

1716	*Determined by CellProfiler*
⬇	*Keep parameters that have a minimum correlation of 0.80 between repeats for all cpds.*
579	*Final set of relevant parameters. Used for all further analyses*

To determine the phenotypic profiles for each test compound Z-scores were then calculated for each parameter as how many times the Median Absolute Deviation (MAD) of the controls the measured parameter value of a test compound deviates from the Median of the controls:

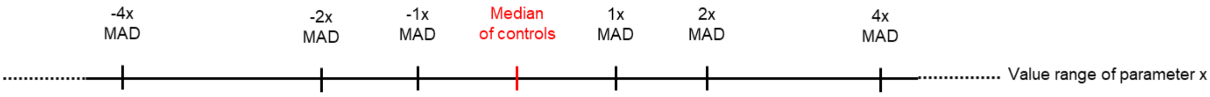

$$z\_score = \frac{value_{meas.} - Median_{Controls}}{MAD_{Controls}}$$

The phenotypic compound profile is then determined as the list of z-scores of all parameters for one compound.

In addition to the phenotypic profile, an induction value was determined for each compound as the fraction of significantly changed parameters, in percent:

$$Induction\ [\%] = \frac{number\ of\ parameters\ with\ abs.\ values > 3}{total\ number\ of\ parameters}$$

Similarities of phenotypic profiles were calculated from the correlation distances between two profiles (https://docs.scipy.org/doc/scipy/reference/generated/scipy.spatial.distance.correlation.html;

Similarity = 1 - Correlation Distance) and the compounds with the most similar profiles were determined from a set of 3000 reference compounds that was also measured in the assay.

An example for two compounds with highly similar profiles (96% similarity):

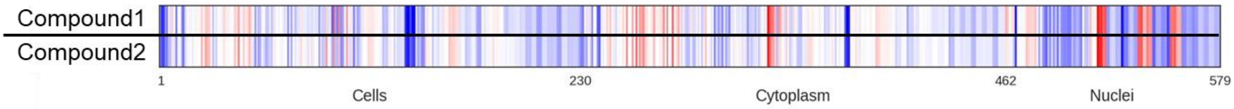

An example for two compounds with low similarity profiles (0% similarity):

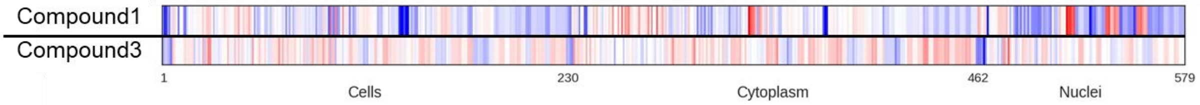

Each colored band represents one Z-score of a parameter.

Principal Component Analyses were performed with the full profiles using the PCA module from the *scikit-learn* package (https://scikit-learn.org/0.22/modules/generated/sklearn.decomposition.PCA.html; v0.22.1, last accessed 22-Apr-2020.)

Synthesis of pyrroquinoline pseudo natural products

General Information

Unless otherwise noted, all commercially available compounds were used as received without further purifications. Dry solvents were purchased from Acros or Sigma Aldrich and used without further treatment. Solvents for chromatography were technical grade. The iminoesters used for 1,3-dipolar cycloaddition were prepared according to the reference 9. Analytical thin-layer chromatography (TLC) was performed on Merck silica gel aluminum plates with F-254 indicator. Compounds were visualized by irradiation with UV light and stained with an appropriate staining reagent. Column chromatography was performed using silica gel Merck 60 (particle size 0.040-0.063 mm) or aluminum oxide (activated, neutral, Brockmann I, Sigma-Aldrich).

^{1}H-NMR and ^{13}C-NMR were recorded on a *Bruker DRX400* (400 MHz), *Bruker DRX500* (500 MHz), *INOVA500* (500 MHz) and *Bruker DRX700* using CD_2Cl_2, $CDCl_3$ or DMSO-*d6* as solvent. Data are reported in the following order: chemical shift (δ) values are reported in ppm with the solvent resonance as internal standard (CD_2Cl_2: δ = 5.32 ppm for ^{1}H, δ = 53.84 ppm for ^{13}C; $CDCl_3$: δ = 7.26 ppm for ^{1}H, δ = 77.16 ppm for ^{13}C; DMSO-*d6*: δ = 2.50 ppm for ^{1}H, δ = 39.52 ppm for ^{13}C); multiplicities are indicated br s (broadened singlet), s (singlet), d (doublet), t (triplet), q (quartet), m (multiplet); coupling constants (*J*) are given in Hertz (Hz).

High resolution mass spectra were recorded on a *LTQ Orbitrap* mass spectrometer coupled to an *Accela HPLC*-System (HPLC column: *Hypersyl GOLD*, 50 mm x 1 mm, particle size 1.9 µm, ionization method: electron spray ionization).

Data collection for single crystal X-ray structure analyses was conducted on a *Bruker D8 Venture* four-circle diffractometer by *Bruker AXS GmbH* using a *PHOTON II* CPAD detector by *Bruker AXS GmbH*. X-ray radiation was generated by microfocus sources *IµS 3.0* Mo by *Incoatec GmbH* with HELIOS mirror optics and a single-hole collimator by *Bruker AXS GmbH*.

For the data collection, the programs APEX 3 Suite (v.2018.7-2) with the integrated programs SAINT (integration) and SADABS (adsorption correction) by Bruker AXS GmbH were used. Using Olex2[3], the structures were solved with the ShelXT[4] structure solution program using Intrinsic Phasing and refined with the XL[5] refinement package using Least Squares minimization.

Chemical yields refer to isolated substances. Yields and enantiomeric excesses, diastereoselectivity and regioselectivity are given in the tables.

Synthesis of pyrroquinolines A

Precursor Synthesis:

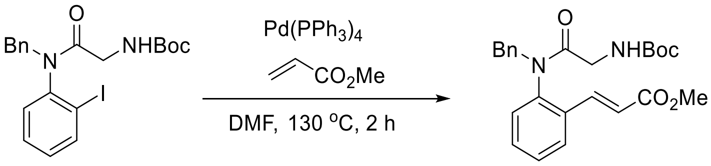

13 **2**

To a solution of iodo arene **13** (synthesized according to Ref [6])[6] (0.97 g, 2.1 mmol, 1.0 equiv.) in dry DMF (14 mL, 0.15 M), $Pd(PPh_3)_4$ (121 mg, 5 mol %), alkene (207 µL, 2.3 mmol, 1.1 equiv.) and triethylamine (590 µL, 4.2 mmol, 2.0 equiv.) were added under argon and the mixture was heated to 130 °C for 2 h. The reaction mixture was quenched with saturated ammonium chloride and extracted with ethyl acetate (3*50 mL). The combined organic layers were dried over $MgSO_4$, filtered and concentrated *in vacuo*. Purification by column chromatography using pentane/EA 10:1 to 4:1 gave the final substrates **2** (550 mg, 62% yield).

^{1}H NMR (700 MHz, $CDCl_3$) δ 7.62 (d, J = 7.9 Hz, 1H), 7.38 (d, J = 16.0 Hz, 1H), 7.35 (t, J = 7.8 Hz, 1H), 7.29 (t, J = 7.8 Hz, 1H), 7.23-7.17 (m, 3H), 7.14-7.09 (m, 2H), 6.85 (d, J = 7.8 Hz, 1H), 6.32 (dd, J = 16.0, 1.5 Hz, 1H), 5.45 (t, J = 4.6 Hz, 1H), 5.17 (dd, J = 14.1, 2.7 Hz, 1H), 4.46 (d, J = 14.1 Hz, 1H), 3.78 – 3.72 (m, 3H), 3.63 (dd, J = 18.0, 5.2 Hz, 1H), 3.30 (dd, J = 17.8, 4.0 Hz, 1H), 1.38 (s, 9H).

^{13}C NMR (176 MHz, $CDCl_3$) δ 168.6, 166.5, 155.6, 138.9, 138.2, 135.9, 132.9, 131.5, 130.2, 129.5 (2C), 128.5 (2C), 127.9, 127.9, 121.3, 79.6, 53.3, 51.8, 43.3, 28.3 (3C).

HRMS(ESI): $[M+H]^+$ calcd. $C_{24}H_{29}N_2O_5$ m/z 425.2071, found 425.2066.

General Procedure 1:

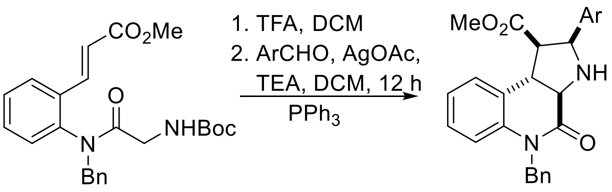

2 **A**

The synthesis was performed according to a literature procedure[6]: To a solution of *N*-Boc alkenyamine **2** (0.2 mmol, 1 equiv.) in DCM, TFA (2.0 mmol, 10 equiv.) was added at 0 °C. The mixture was stirred for 3 h at this temperature until TLC showed complete deprotection. The solvent was removed under reduced pressure. The remains were co-evaporated three times with DCM to remove excess TFA. Subsequently, dry DCM was added followed by TEA (0.4 mmol, 2.0 equiv.) and the mixture was stirred for 10 min. Aldehyde (0.24 mmol, 1.2 equiv.) and freshly activated 4 Å MS powder were added followed by AgOAc (10 mol%) and PPh_3 (10 mol%). Upon completion (typically 6-12 h), the reaction mixture was directly transferred onto a column and purified using silica gel chromatography to obtain the desired PQ **A** as a single diastereoisomer.

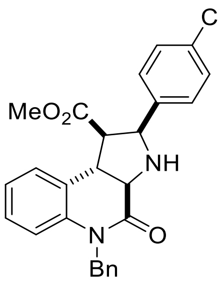

A1

(±)-Methyl (1*R*,2*S*,3a*R*,9b*S*)-5-benzyl-2-(4-chlorophenyl)-4-oxo-2,3,3a,4,5,9b-hexahydro-1*H*-pyrrolo[2,3-*c*]quinoline-1-carboxylate (A1)

PQ **A1** (60 mg, 134 µmol, 67% Yield) was synthesized according to the general procedure 1; column chromatography eluting with cyclohexane/EA 5:1 to 3:1.

^{1}H NMR (400 MHz, CDCl$_3$) δ 7.43 – 7.38 (m, 2H), 7.37 – 7.28 (m, 5H), 7.26 – 7.22 (m, 3H), 7.16 (dd, *J* = 8.2, 7.4, 1H), 7.05 – 6.91 (m, 3H), 5.50 (d, *J* = 16.2 Hz, 1H), 5.01 – 4.90 (m, 2H), 3.92 – 3.75 (m, 2H), 3.60 (t, *J* = 10.4 Hz, 1H), 3.29 (s, 3H).

^{13}C NMR (101 MHz, CDCl$_3$) δ 171.3, 170.3, 140.2, 139.0, 137.0, 133.8, 129.6 (2C), 129.0 (2C), 128.3 (2C), 128.1, 127.7, 127.4, 126.6 (2C), 124.6, 123.6, 116.4, 63.4, 61.9, 52.0, 51.9, 46.5, 43.1.

HRMS(ESI): $[M+H]^+$ calcd. $C_{26}H_{24}N_2O_3Cl$ m/z 447.1470, found 447.1468.

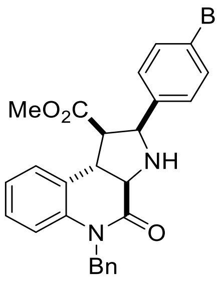

A2

(±)-Methyl (1*R*,2*S*,3a*R*,9b*S*)-5-benzyl-2-(4-bromophenyl)-4-oxo-2,3,3a,4,5,9b-hexahydro-*1H*-pyrrolo[2,3-*c*]quinoline-1-carboxylate (A2)

PQ **A2** (69 mg, 140 µmol, 70% Yield) was synthesized according to the general procedure 1; column chromatography eluting with cyclohexane/EA 5:1 to 3:1.

^{1}H NMR (400 MHz, CDCl$_3$) δ 7.49 – 7.42 (m, 2H), 7.37 – 7.29 (m, 4H), 7.27 – 7.22 (m, 3H), 7.15 (dd, *J* = 8.2, 1.6, 1H), 7.01 (td, *J* = 7.4, 1.1 Hz, 1H), 6.98-6.92 (m, 2H), 5.49 (d, *J* = 16.2 Hz, 1H), 5.03 – 4.88 (m, 2H), 3.93 – 3.72 (m, 2H), 3.65 – 3.55 (m, 1H), 3.30 (s, 3H).

^{13}C NMR (101 MHz, CDCl$_3$) δ 171.3, 170.3, 140.2, 139.5, 137.0, 131.2 (2C), 129.9 (2C), 129.0 (2C), 128.1, 127.7, 127.4, 126.6 (2C), 124.6, 123.6, 121.9, 116.4, 63.4, 61.9, 52.0, 51.9, 46.5, 43.1.

HRMS(ESI): $[M+H]^+$ calcd. $C_{26}H_{24}N_2O_3Br$ m/z 491.0965, found 491.0961.

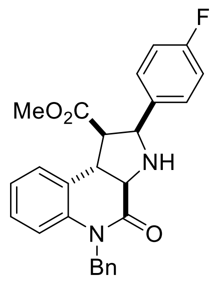

A3

(±)-Methyl (1*R*,2*S*,3a*R*,9b*S*)-5-benzyl-2-(4-fluorophenyl)-4-oxo-2,3,3a,4,5,9b-hexahydro-*1H*-pyrrolo[2,3-*c*]quinoline-1-carboxylate (A3)

PQ **A3** (58 mg, 134 µmol, 67% Yield) was synthesized according to the general procedure 1; column chromatography eluting with cyclohexane/EA 5:1 to 3:1.

^{1}H NMR (600 MHz, CDCl$_3$) δ 7.46 – 7.41 (m, 2H), 7.35-7.30 (m, 2H), 7.25 (m, 3H), 7. 16 (t, *J* =7.8 Hz, 1H), 7.04 – 6.98 (m, 3H), 6.97-6.94 (m, 2H), 5.50 (d, *J* = 16.2 Hz, 1H), 5.05 – 4.91 (m, 2H), 3.87 (dd, *J* = 14.0, 10.7 Hz, 1H), 3.80 (d, *J* = 14.0 Hz, 1H), 3.59 (t, *J* = 10.6 Hz, 1H), 3.28 (s, 3H).

^{13}C NMR (151 MHz, CDCl$_3$) δ 171.4, 170.3, 162.5 (d, *J*CF = 246.1 Hz), 140.2, 137.0, 136.1 (d, *J*CF = 3.0 Hz), 129.8 (d, *J*CF = 9.1 Hz, 2C), 128.9 (2C), 128.1, 127.7, 127.4, 126.6 (2C), 124.7, 123.6, 116.3, 114.9 (d, *J*CF = 21.1 Hz, 2C), 63.4, 61.9, 52.0, 51.9, 46.5, 43.1.

^{19}F NMR (470 MHz, CDCl$_3$) δ -114.4 (m, 1F).

HRMS(ESI): $[M+H]^+$ calcd. $C_{26}H_{24}N_2O_3F$ m/z 431.1766, found 431.1764.

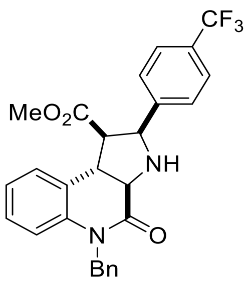

A4

(±)-Methyl (1*R*,2*S*,3a*R*,9b*S*)-5-benzyl-4-oxo-2-(4-(trifluoromethyl)phenyl)-2,3,3a,4,5,9b-hexahydro-*1H*-pyrrolo[2,3-*c*]quinoline-1-carboxylate (A4)

PQ **A4** (62 mg, 129 µmol, 65% Yield) was synthesized according to the general procedure 1; column chromatography eluting with cyclohexane/EA 5:1 to 3:1.

^{1}H NMR (600 MHz, CDCl$_3$) δ 7.62-7.58 (m, 4H), 7.36-7.30 (m, 2H), 7.26-7.24 (m, 3H), 7.17 (t, *J* = 7.8 Hz, 1H), 7.02 (td, *J* = 7.5, 1.1 Hz, 1H), 6.98 – 6.93 (m, 2H), 5.50 (d, *J* = 16.2 Hz, 1H), 5.02 (d, *J* = 10.6 Hz, 1H), 4.98 (d, *J* = 16.2 Hz, 1H), 3.94 – 3.75 (m, 2H), 3.65 (t, *J* = 10.6 Hz, 1H), 3.24 (s, 3H).

^{13}C NMR (151 MHz, CDCl$_3$) δ 171.2, 170.2, 144.6, 140.2, 136.9, 130.2 (q, *J*CF = 33.0 Hz), 129.0 (2C), 128.6 (2C), 128.1, 127.5, 127.4, 126.6 (2C), 125.0 (q, *J*CF = 3.0 Hz, 2C), 124.6, 124.3 (q, *J*CF = 271.8 Hz), 123.7, 116.4, 63.4, 61.9, 52.1, 51.9, 46.5, 43.0.

^{19}F NMR (470 MHz, CDCl$_3$) δ -62.4 (s, 3F).

HRMS(ESI): $[M+H]^+$ calcd. $C_{27}H_{24}N_2O_3F_3$ m/z 481.1733, found 481.1728.

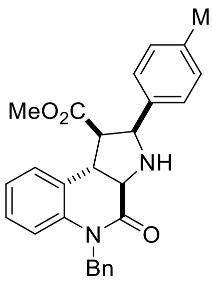

A5

(±)-Methyl (1*R*,2*S*,3a*R*,9b*S*)-5-benzyl-4-oxo-2-(p-tolyl)-2,3,3a,4,5,9b-hexahydro-*1H*-pyrrolo[2,3-*c*]quinoline-1-carboxylate (A5)

PQ **A5** (48 mg, 112 µmol, 56% Yield) was synthesized according to the general procedure 1; column chromatography eluting with cyclohexane/EA 5:1 to 3:1.

^{1}H NMR (600 MHz, $CDCl_3$) δ 7.38 – 7.33 (m, 3H), 7.32 – 7.24 (m, 5H), 7.19 – 7.11 (m, 3H), 6.99 (t, *J* = 7.5 Hz, 1H), 6.92 (d, *J* = 8.3 Hz, 1H), 5.48 (d, *J* = 16.1 Hz, 1H), 5.04 (d, *J* = 16.1 Hz, 1H), 4.78 (d, *J* = 10.6 Hz, 1H), 4.30 – 4.18 (m, 2H), 3.23 (t, *J* = 10.4 Hz, 1H), 3.20 (s, 3H), 2.34 (s, 3H).

^{13}C NMR (151 MHz, $CDCl_3$) δ 171.4, 171.2, 138.4, 138.2, 137.2, 136.6, 130.1, 129.0 (2C), 128.7 (2C), 128.5, 127.8 (2C), 127.5, 126.5 (2C), 124.2, 123.6, 115.9, 61.4, 59.2, 54.8, 51.5, 46.7, 41.5, 21.3.

HRMS(ESI): $[M+H]^+$ calcd. $C_{27}H_{27}N_2O_3$ m/z 427.2016, found 427.2015.

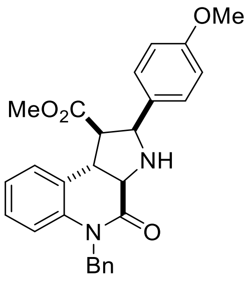

A6

(±)-Methyl (1*R*,2*S*,3a*R*,9b*S*)-5-benzyl-2-(4-methoxyphenyl)-4-oxo-2,3,3a,4,5,9b-hexahydro-*1H*-pyrrolo[2,3-*c*]quinoline-1-carboxylate (A6)

PQ **A6** (22 mg, 50 µmol, 25% Yield) was synthesized according to the general procedure 1; column chromatography eluting with cyclohexane/EA 5:1 to 2:1.

^{1}H NMR (500 MHz, $CDCl_3$) δ 7.37 – 7.31 (m, 5H), 7.29 – 7.21 (m, 3H), 7.15 (dd, *J* = 8.2, 7.5 Hz, 1H), 6.98 (td, *J* = 7.5, 1.1 Hz, 1H), 6.91 (d, *J* = 8.3 Hz, 1H), 6.88 – 6.83 (m, 2H), 5.47 (d, *J* = 16.1 Hz, 1H), 5.01 (d, *J* = 16.1 Hz, 1H), 4.76 (d, *J* = 10.7 Hz, 1H), 4.26-4.18 (m, 2H), 3.80 (s, 3H), 3.24-3.17 (m, 4H).

^{13}C NMR (126 MHz, $CDCl_3$) δ 171.4, 171.3, 159.1, 138.2, 136.6, 133.5, 130.1, 129.0 (2C), 129.0 (2C), 128.5, 127.5, 126.5 (2C), 124.2, 123.6, 115.9, 113.4 (2C), 61.1, 59.2, 55.4, 54.7, 51.6, 46.7, 41.5.

HRMS(ESI): $[M+H]^+$ calcd. $C_{27}H_{27}N_2O_4$ m/z 443.1965, found 443.1963.

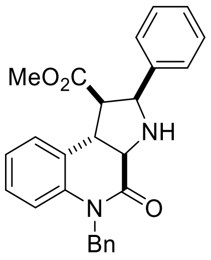

A7

(±)-Methyl (1*R*,2*S*,3a*R*,9b*S*)-5-benzyl-4-oxo-2-phenyl-2,3,3a,4,5,9b-hexahydro-*1H*-pyrrolo[2,3-*c*]quinoline-1-carboxylate (A7)

PQ **A7** (34 mg, 81 µmol, 41% Yield) was synthesized according to the general procedure 1; column chromatography eluting with cyclohexane/EA 5:1 to 3:1.

^{1}H NMR (500 MHz, $CDCl_3$) δ 7.44 – 7.39 (m, 2H), 7.38-7.30 (m, 5H), 7.29-7.24 (m, 4H), 7.16 (ddd, *J* = 8.7, 7.5, 1.7 Hz, 1H), 6.99 (td, *J* = 7.5, 1.1 Hz, 1H), 6.92 (dd, *J* = 8.3, 1.1 Hz, 1H), 5.49 (d, *J* = 16.1 Hz, 1H), 5.02 (d, *J* = 16.1 Hz, 1H), 4.80 (d, *J* = 10.7 Hz, 1H), 4.32 – 4.19 (m, 2H), 3.25 (t, *J* = 10.6 Hz, 1H), 3.16 (s, 3H).

^{13}C NMR (126 MHz, $CDCl_3$) δ 171.3, 171.1, 141.5, 138.2, 136.6, 130.1, 129.0 (2C), 128.5, 128.0 (2C), 128.0 (2C), 127.7, 127.5, 126.5 (2C), 124.1, 123.6, 115.9, 61.6, 59.2, 54.8, 51.5, 46.7, 41.5.

HRMS(ESI): $[M+H]^+$ calcd. $C_{26}H_{25}N_2O_3$ m/z 413.1860, found 413.1858.

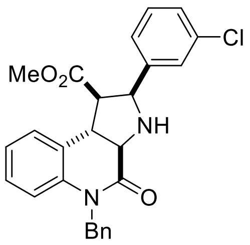

A8

(±)-Methyl (1*R*,2*S*,3a*R*,9b*S*)-5-benzyl-2-(3-chlorophenyl)-4-oxo-2,3,3a,4,5,9b-hexahydro-*1H*-pyrrolo[2,3-*c*]quinoline-1-carboxylate (A8)

PQ **A8** (60 mg, 135 µmol, 67% Yield) was synthesized according to the general procedure 1; column chromatography eluting with cyclohexane/EA 5:1 to 3:1.

^{1}H NMR (500 MHz, $CDCl_3$) δ 7.44 (d, *J* = 2.0 Hz, 1H), 7.37 – 7.31 (m, 3H), 7.31 – 7.23 (m, 6H), 7.16 (ddd, *J* = 8.2, 7.5, 1.7 Hz, 1H), 6.99 (td, *J* = 7.5, 1.1 Hz, 1H), 6.92 (d, *J* = 8.4 Hz, 1H), 5.48 (d, *J* = 16.1 Hz, 1H), 5.01 (d, *J* = 16.1 Hz, 1H), 4.77 (d, *J* = 10.7 Hz, 1H), 4.25 (d, *J* = 7.6 Hz, 1H), 4.18 (dd, *J* = 11.1, 7.6 Hz, 1H), 3.64 (s, 1H), 3.26-3.22 (m, 4H).

^{13}C NMR (126 MHz, $CDCl_3$) δ 171.2, 170.8, 143.8, 138.2, 136.5, 134.0, 130.2, 129.3, 129.1 (2C), 128.6, 128.2, 127.8, 127.5, 126.5 (2C), 126.1, 123.7, 116.0, 60.9, 59.2, 54.6, 51.7, 46.8, 41.4.

HRMS(ESI): $[M+H]^+$ calcd. $C_{26}H_{24}N_2O_3Cl$ m/z 447.1470, found 447.1467.

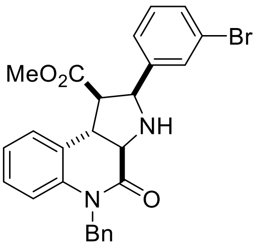

A9

(±)-Methyl (1*R*,2*S*,3a*R*,9b*S*)-5-benzyl-2-(3-bromophenyl)-4-oxo-2,3,3a,4,5,9b-hexahydro-*1H*-pyrrolo[2,3-*c*]quinoline-1-carboxylate (A9)

PQ **A9** (77 mg, 157 µmol, 79% Yield) was synthesized according to the general procedure 1; column chromatography eluting with cyclohexane/EA 5:1 to 3:1.

^{1}H NMR (400 MHz, CDCl$_3$) δ 7.59 (t, *J* = 1.9 Hz, 1H), 7.42 – 7.31 (m, 5H), 7.29 – 7.23 (m, 3H), 7.22 – 7.13 (m, 2H), 6.99 (td, *J* = 7.5, 1.1 Hz, 1H), 6.92 (dd, *J* = 8.3, 1.1 Hz, 1H), 5.48 (d, *J* = 16.1 Hz, 1H), 5.02 (d, *J* = 16.1 Hz, 1H), 4.76 (d, *J* = 10.6 Hz, 1H), 4.25 (d, *J* = 7.7 Hz, 1H), 4.18 (dd, *J* = 11.0, 7.6 Hz, 1H), 3.26-3.21 (m, 4H).

^{13}C NMR (101 MHz, CDCl$_3$) δ 171.2, 170.7, 144.1, 138.2, 136.6, 131.1, 130.8, 130.2, 129.6, 129.1 (2C), 128.6, 127.5, 126.6, 126.5 (2C), 123.7, 123.7, 122.2, 116.0, 60.9, 59.2, 54.7, 51.7, 46.8, 41.4.

HRMS(ESI): $[M+H]^+$ calcd. $C_{26}H_{24}N_2O_3Br$ m/z 491.0965, found 491.0960.

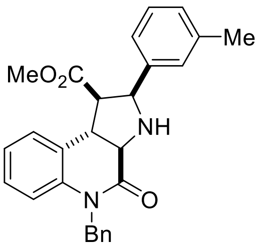

A10

(±)-Methyl (1*R*,2*S*,3a*R*,9b*S*)-5-benzyl-4-oxo-2-(*m*-tolyl)-2,3,3a,4,5,9b-hexahydro-*1H*-pyrrolo[2,3-*c*]quinoline-1-carboxylate (A10)

PQ **A10** (49 mg, 116 µmol, 58% Yield) was synthesized according to the general procedure 1; column chromatography eluting with cyclohexane/EA 5:1 to 3:1.

^{1}H NMR (500 MHz, CDCl$_3$) δ 7.39 – 7.33 (m, 3H), 7.30 – 7.19 (m, 6H), 7.16 (ddd, *J* = 8.8, 7.6, 1.6 Hz, 1H), 7.10 – 7.05 (m, 1H), 6.99 (t, *J* = 7.5 Hz, 1H), 6.92 (d, *J* = 8.3 Hz, 1H), 5.48 (d, *J* = 16.1 Hz, 1H), 5.03 (d, *J* = 16.1 Hz, 1H), 4.77 (d, *J* = 10.6 Hz, 1H), 4.37 – 4.16 (m, 2H), 3.64 (s, 1H), 3.25 (d, *J* = 10.3 Hz, 1H), 3.19 (s, 3H), 2.37 (s, 3H).

^{13}C NMR (126 MHz, CDCl$_3$) δ 171.3, 171.1, 141.4, 138.2, 137.6, 136.6, 130.1, 129.0 (2C), 128.5, 128.5, 128.4, 127.9, 127.5, 126.5 (2C), 125.0, 124.1, 123.6, 115.9, 61.6, 59.2, 54.8, 51.5, 46.7, 41.5, 21.6.

HRMS(ESI): $[M+H]^+$ calcd. $C_{27}H_{27}N_2O_3$ m/z 427.2016, found 427.2015.

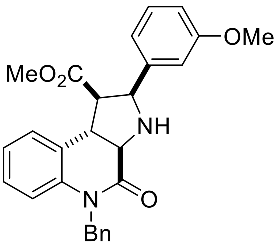

A11

(±)-Methyl (1*R*,2*S*,3a*R*,9b*S*)-5-benzyl-2-(3-methoxyphenyl)-4-oxo-2,3,3a,4,5,9b-hexahydro-*1H*-pyrrolo[2,3-*c*]quinoline-1-carboxylate (A11)

PQ **A11** (34 mg, 76 µmol, 67% Yield) was synthesized according to the general procedure 1; column chromatography eluting with cyclohexane/EA 5:1 to 2:1.

^{1}H NMR (400 MHz, $CDCl_3$) δ 7.39 – 7.31 (m, 3H), 7.30 – 7.21 (m, 4H), 7.19 – 7.12 (m, 1H), 7.04 – 6.95 (m, 3H), 6.92 (d, *J* = 8.2 Hz, 1H), 6.81 (dd, *J* = 8.2, 2.7 Hz, 1H), 5.48 (d, *J* = 16.1 Hz, 1H), 5.02 (d, *J* = 16.1 Hz, 1H), 4.78 (d, *J* = 10.6 Hz, 1H), 4.32 – 4.16 (m, 2H), 3.83 (s, 3H), 3.26-3.21 (m, 4H).

^{13}C NMR (101 MHz, $CDCl_3$) δ 171.3, 171.1, 159.5, 143.2, 138.2, 136.6, 130.1, 129.0 (2C), 129.0, 128.5, 127.5, 126.5 (2C), 124.1, 123.6, 120.3, 115.9, 113.4, 113.3, 61.5, 59.2, 55.4, 54.8, 51.6, 46.7, 41.5.

HRMS(ESI): $[M+H]^+$ calcd. $C_{27}H_{27}N_2O_4$ m/z 443.1965, found 443.1963.

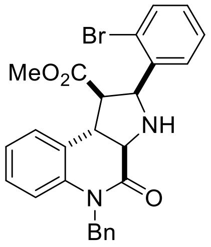

A12

(±)-Methyl (1*R*,2*S*,3a*R*,9b*S*)-5-benzyl-2-(2-bromophenyl)-4-oxo-2,3,3a,4,5,9b-hexahydro-*1H*-pyrrolo[2,3-*c*]quinoline-1-carboxylate (A12)

PQ **A12** (68 mg, 139 µmol, 70% Yield) was synthesized according to the general procedure 1; column chromatography eluting with cyclohexane/EA 5:1 to 3:1.

^{1}H NMR (400 MHz, $CDCl_3$) δ 7.88 (dd, *J* = 7.8, 1.8 Hz, 1H), 7.52 (dd, *J* = 8.0, 1.3 Hz, 1H), 7.37-7.29 (m, 3H), 7.27 – 7.26 (m, 2H), 7.25 (q, *J* = 1.0 Hz, 1H), 7.19 – 7.11 (m, 2H), 7.06 – 6.99 (m, 2H), 6.99 – 6.94 (m, 1H), 5.47 (d, *J* = 16.2 Hz, 1H), 5.32 (d, *J* = 10.4 Hz, 1H), 5.01 (d, *J* = 16.2 Hz, 1H), 4.00 – 3.88 (m, 1H), 3.84 – 3.72 (m, 2H), 3.25 (s, 3H).

^{13}C NMR (101 MHz, $CDCl_3$) δ 171.7, 170.2, 140.1, 139.2, 137.0, 132.2, 130.9, 129.3, 129.0 (2C), 128.0, 127.9, 127.5, 127.4, 126.6 (2C), 124.6, 123.8, 123.6, 116.3, 62.9, 61.7, 51.8, 50.1, 46.4, 43.9.

HRMS(ESI): $[M+H]^+$ calcd. $C_{26}H_{24}N_2O_3Br$ m/z 491.0965, found 491.0962.

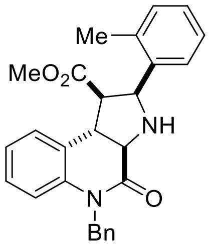

A13

(±)-Methyl (1*R*,2*S*,3a*R*,9b*S*)-5-benzyl-4-oxo-2-(*o*-tolyl)-2,3,3a,4,5,9b-hexahydro-*1H*-pyrrolo[2,3-*c*]quinoline-1-carboxylate (A13)

PQ **A13** (19 mg, 43 μmol, 22% Yield) was synthesized according to the general procedure 1; column chromatography eluting with cyclohexane/EA 5:1 to 3:1.

^{1}H NMR (500 MHz, $CDCl_3$) δ 7.75 (d, *J* = 7.8 Hz, 1H), 7.38 – 7.32 (m, 2H), 7.29 – 7.21 (m, 5H), 7.19 – 7.11 (m, 2H), 7.10 – 7.07 (m, 1H), 6.99 (td, *J* = 7.5, 1.1 Hz, 1H), 6.94 (dd, *J* = 8.3, 1.1 Hz, 1H), 5.47 (d, *J* = 16.1 Hz, 1H), 5.09 (d, *J* = 16.1 Hz, 1H), 4.95 (d, *J* = 10.7 Hz, 1H), 4.38 – 4.24 (m, 2H), 3.23 (dd, *J* = 10.7, 9.3 Hz, 1H), 3.09 (s, 3H), 2.32 (s, 3H).

^{13}C NMR (101 MHz, $CDCl_3$) δ 171.6, 171.4, 138.9, 138.1, 136.6, 135.8, 129.8, 129.8, 129.0 (2C), 128.5, 127.7, 127.5, 127.3, 126.6 (2C), 125.9, 124.7, 123.7, 116.0, 59.1, 57.8, 54.1, 51.5, 46.6, 42.2, 19.5.

HRMS(ESI): $[M+H]^+$ calcd. $C_{27}H_{27}N_2O_3$ m/z 427.2016, found 427.2015.

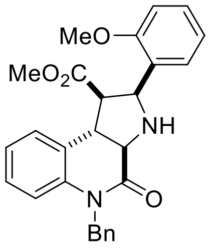

A14

(±)-Methyl (1*R*,2*S*,3a*R*,9b*S*)-5-benzyl-2-(2-methoxyphenyl)-4-oxo-2,3,3a,4,5,9b-hexahydro-*1H*-pyrrolo[2,3-*c*]quinoline-1-carboxylate (A14)

PQ **A14** (58 mg, 130 μmol, 65% Yield) was synthesized according to the general procedure 1; column chromatography eluting with cyclohexane/EA 5:1 to 2:1.

^{1}H NMR (400 MHz, $CDCl_3$) δ 7.70 (dd, *J* = 7.6, 1.8 Hz, 1H), 7.40 – 7.32 (m, 2H), 7.30 – 7.22 (m, 5H), 7.19 – 7.12 (m, 1H), 7.04-6.95 (m, 2H), 6.93 (d, *J* = 8.2 Hz, 1H), 6.82 (d, *J* = 8.2 Hz, 1H), 5.49 (d, *J* = 16.2 Hz, 1H), 5.17 – 5.04 (m, 2H), 4.33 – 4.20 (m, 2H), 3.80 (s, 3H), 3.27 (dd, *J* = 10.1, 9.5 Hz, 1H), 3.20 (s, 3H).

^{13}C NMR (101 MHz, $CDCl_3$) δ 172.0, 171.4, 156.8, 138.1, 136.6, 129.7, 129.3 (2C), 129.0, 128.4 (2C), 128.3, 127.4, 126.5 (2C), 124.6, 123.6, 120.5, 115.9, 109.7, 59.0, 55.7, 55.4, 54.3, 51.5, 46.6, 42.6.

HRMS(ESI): $[M+H]^+$ calcd. $C_{27}H_{27}N_2O_4$ m/z 443.1965, found 443.1963.

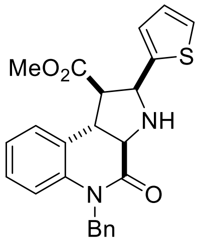

A15

(±)-Methyl (1*R*,2*S*,3a*R*,9b*S*)-5-benzyl-4-oxo-2-(thiophen-2-yl)-2,3,3a,4,5,9b-hexahydro-*1H*-pyrrolo[2,3-*c*]quinoline-1-carboxylate (A15)

PQ **A15** (55 mg, 131 μmol, 65% Yield) was synthesized according to the general procedure 1; column chromatography eluting with cyclohexane/EA 5:1 to 3:1.

^{1}H NMR (500 MHz, $CDCl_3$) δ 7.37 – 7.31 (m, 3H), 7.29 – 7.26 (m, 1H), 7.26-7.22 (m, 3H), 7.15 (ddd, *J* = 8.1, 7.4, 1.6 Hz, 1H), 7.01 – 6.95 (m, 2H), 6.90 (d, *J* = 8.3 Hz, 1H), 6.86 (d, *J* = 3.5 Hz, 1H), 5.45 (d, *J* = 16.0 Hz, 1H), 5.12 (d, *J* = 10.1 Hz, 1H), 4.99 (d, *J* = 16.1 Hz, 1H), 4.30 – 4.14 (m, 2H), 3.39 (s, 3H), 3.26 – 3.18 (m, 1H).

^{13}C NMR (126 MHz, $CDCl_3$) δ 171.1, 170.5, 147.5, 138.2, 136.5, 130.3, 129.1 (2C), 128.6, 127.5, 126.9, 126.5 (2C), 125.1, 124.0, 123.7, 123.5, 115.9, 58.8, 57.1, 54.7, 51.8, 46.8, 40.9.

HRMS(ESI): $[M+H]^+$ calcd. $C_{24}H_{23}N_2O_3S$ m/z 419.1424, found 419.1423.

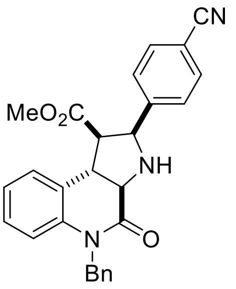

A16

(±)-Methyl (1*R*,2*S*,3a*R*,9b*S*)-5-benzyl-2-(4-cyanophenyl)-4-oxo-2,3,3a,4,5,9b-hexahydro-*1H*-pyrrolo[2,3-*c*]quinoline-1-carboxylate (A16)

PQ **A1** (60 mg, 137 µmol, 69% Yield) was synthesized according to the general procedure 1; column chromatography eluting with cyclohexane/EA 5:1 to 3:1.

^{1}H NMR (500 MHz, $CDCl_3$) δ 7.61 (s, 4H), 7.35 – 7.29 (m, 2H), 7.27 – 7.23 (m, 3H), 7.17 (d, J = 1.5 Hz, 1H), 7.01 (td, J = 7.4, 1.0 Hz, 1H), 6.98 – 6.91 (m, 2H), 5.49 (d, J = 16.2 Hz, 1H), 5.08 – 4.92 (m, 2H), 3.87-3.80 (m, 2H), 3.65 (ddd, J = 10.7, 8.2, 2.6 Hz, 1H), 3.27 (s, 3H).

^{13}C NMR (126 MHz, $CDCl_3$) δ 170.9, 170.1, 146.1, 140.1, 136.8, 131.9 (2C), 129.0 (4C), 128.2, 127.4, 127.2, 126.6 (2C), 124.5, 123.7, 118.9, 116.4, 111.7, 63.1, 61.8, 52.0, 52.0, 46.5, 42.8.

HRMS(ESI): $[M+H]^+$ calcd. $C_{27}H_{24}N_3O_3$ m/z 438.1812, found 438.1803.

Synthesis of pyrroquinolines B

General Procedure 2

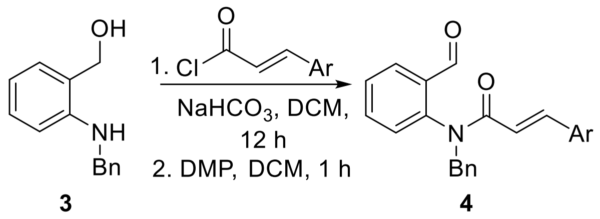

To a solution of **3**[7] (1.0 equiv.) in dry DCM (20 mL), $NaHCO_3$ (5.0 equiv.) was added followed by the desired acid chloride (1.1 equiv.). The reaction was stirred overnight at room temperature and quenched with saturated $NaHCO_3$ solution (20 mL) and then extracted with DCM (3*20 mL). The combined organic phases were dried over Na_2SO_4, filtered and concentrated under reduced pressure. The reaction residue was purified with flash column using pentane/EA.

The intermediate alcohol was dissolved in dry DCM (10 mL), and DMP (1.1 equiv.) was added in small portions at room temperature. The reaction was stirred for 1 h until full conversion of the starting material was observed. The reaction mixture was quenched with saturated $Na_2S_2O_3$ (10 mL) and extracted with DCM (3*20 mL). The combined organic phases were dried over Na_2SO_4, filtered and concentrated under reduced pressure. The reaction residue was purified by column chromatography using pentane/EA to give the desired product **4**.

General Procedure 3

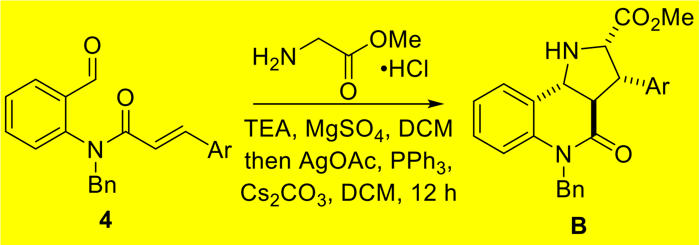

Glycine methyl ester salt (0.3 mmol, 1.3 equiv.) was suspended in dry DCM (1 mL) and $MgSO_4$ (0.3 mmol, 1.3 equiv.) and TEA (0.44 mmol, 2.0 equiv.) were added. The mixture was stirred for 30 mins followed by the addition of aldehyde **4** (0.22 mmol, 1.0 equiv.). The reaction was stirred for 12 h then filtered. The solution was diluted with EA (20 mL) and washed with $NaHCO_3$ solution (20 mL) and brine (20 mL) sequentially. The organic phase was dried over Na_2SO_4 and filtered. The solvent was removed under reduced pressure. Then the residue was dissolved in the dry DCM (1.0 mL) followed by the addition of a solution of AgOAc (0.02 mmol, 0.1 equiv.) and PPh_3 (0.03 mmol, 0.12 equiv.) in DCM (0.5 mL). Cs_2CO_3 (0.04 mmol, 0.2 equiv.) was added to the reaction and stirred overnight. The solvent was removed under reduced pressure. PQs **B** were purified by silica gel chromatography using cyclohexane/EA.

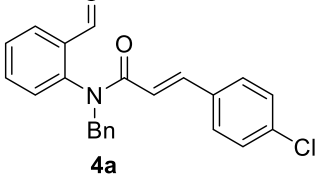

4a

(*E*)-*N*-benzyl-3-(4-chlorophenyl)-*N*-(2-formylphenyl)acrylamide (4a)

Acrylamide **4a** (352 mg, 0.94 mmol, 52% Yield) was synthesized according to the general procedure 2 from **3** in 1.8 mmol scale; column chromatography eluting with cyclohexane/EA 5:1 to 3:1.

^{1}H NMR (700 MHz, $CDCl_3$) δ 9.73 (s, 1H), 8.01 (dd, *J* = 7.8, 1.7 Hz, 1H), 7.79 (d, *J* = 15.4 Hz, 1H), 7.72 (td, *J* = 7.6, 1.7 Hz, 1H), 7.60 (t, *J* = 7.6 Hz, 1H), 7.34 – 7.30 (m, 3H), 7.28-7.25 (m, 4H), 7.24-7.20 (m, 3H), 6.12 (d, *J* = 15.4 Hz, 1H), 5.38 – 4.97 (m, 2H).

^{13}C NMR (176 MHz, $CDCl_3$) δ 188.9, 165.6, 143.5, 142.6, 135.9, 135.8, 135.4, 133.6, 133.2, 130.2, 129.5 (2C), 129.4, 129.1, 129.1 (2C), 129.0 (2C), 128.7 (2C), 128.1, 118.1, 54.1.

HRMS(ESI): $[M+H]^+$ calcd. $C_{23}H_{19}NO_2Cl$ m/z 376.1099, found 376.1096.

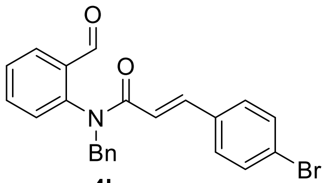

4b

(*E*)-*N*-benzyl-3-(4-bromophenyl)-*N*-(2-formylphenyl)acrylamide (4b)

Acrylamide **4b** (343 mg, 0.82 mmol, 46% Yield) was synthesized according to the general procedure 2 from **3** in 1.8 mmol scale; column chromatography eluting with cyclohexane/EA 5:1 to 3:1.

^{1}H NMR (500 MHz, $CDCl_3$) δ 9.65 (s, 1H), 7.95 (dd, *J* = 7.8, 1.7 Hz, 1H), 7.73 – 7.63 (m, 2H), 7.55 (t, *J* = 7.6 Hz, 1H), 7.40 – 7.35 (m, 2H), 7.29-7.22 (m, 3H), 7.22-7.18 (m, 2H), 7.16 (d, *J* = 7.9 Hz, 1H), 7.12 – 7.05 (m, 2H), 6.06 (d, *J* = 15.4 Hz, 1H), 5.13-5.00 (m, 2H).

^{13}C NMR (126 MHz, $CDCl_3$) δ 189.0, 165.7, 143.5, 142.8, 135.9, 135.5, 133.7, 133.6, 132.0 (2C), 130.2, 129.6 (2C), 129.5, 129.4 (2C), 129.2, 128.8 (2C), 128.2, 124.3, 118.2, 54.2.

HRMS(ESI): $[M+H]^+$ calcd. $C_{23}H_{19}NO_2Br$ m/z 420.0594, found 420.0598.

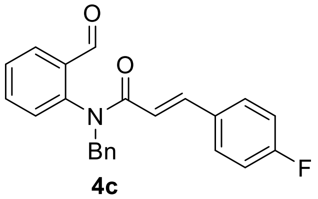

4c

(*E*)-*N*-benzyl-3-(4-fluorophenyl)-*N*-(2-formylphenyl)acrylamide (4c)

Acrylamide **4c** (316 mg, 0.88 mmol, 49% Yield) was synthesized according to the general procedure 2 from **3** in 1.8 mmol scale; column chromatography eluting with cyclohexane/EA 5:1 to 3:1.

^{1}H NMR (400 MHz, $CDCl_3$) δ 9.68 (s, 1H), 7.96 (dd, *J* = 7.8, 1.7 Hz, 1H), 7.75 (d, *J* = 15.4 Hz, 1H), 7.67 (td, *J* = 7.6, 1.7 Hz, 1H), 7.55 (t, *J* = 7.6 Hz, 1H), 7.34 – 7.16 (m, 8H), 6.97-6.90 (m, 2H), 6.02 (d, *J* = 15.4 Hz, 1H), 5.16 – 4.88 (m, 2H).

^{13}C NMR (101 MHz, $CDCl_3$) δ 189.0, 165.8, 163.7 (d, *J*CF = 250.9 Hz), 143.7, 142.8, 136.0, 135.4, 133.7, 131.0 (d, *J* = 3.3 Hz), 130.2, 129.9 (d, *J*CF = 8.5 Hz, 2C), 129.6 (2C), 129.4, 129.1, 128.7 (2C), 128.2, 117.4, 115.9 (d, *J*CF = 22.0 Hz, 2C), 54.1.

^{19}F NMR (470 MHz, $CDCl_3$) δ -109.9 (ddd, *J* = 13.8, 8.5, 5.4 Hz, 1F).

HRMS(ESI): $[M+H]^+$ calcd. $C_{23}H_{19}NO_2F$ m/z 360.1394, found 360.1391.

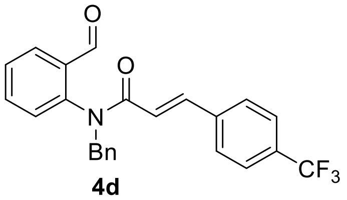

4d

(*E*)-*N*-benzyl-*N*-(2-formylphenyl)-3-(4-(trifluoromethyl)phenyl)acrylamide (4d)

Acrylamide **4d** (206 mg, 0.5 mmol, 28% Yield) was synthesized according to the general procedure 2 from **3** in 1.8 mmol scale; column chromatography eluting with cyclohexane/EA 5:1 to 3:1.

^{1}H NMR (700 MHz, CDCl$_3$) δ 9.66 (s, 1H), 7.95 (dd, *J* = 7.8, 1.7 Hz, 1H), 7.77 (d, *J* = 15.4 Hz, 1H), 7.66 (td, *J* = 7.6, 1.7 Hz, 1H), 7.55 (t, *J* = 7.6 Hz, 1H), 7.50 (d, *J* = 8.3 Hz, 2H), 7.32 (d, *J* = 8.2 Hz, 2H), 7.28 – 7.22 (m, 3H), 7.21 – 7.18 (m, 2H), 7.17 (dd, *J* = 7.9, 1.1 Hz, 1H), 6.14 (d, *J* = 15.4 Hz, 1H), 5.10 – 5.05 (m, 2H).

^{13}C NMR (176 MHz, CDCl$_3$) δ 189.0, 165.4, 143.4, 142.3, 138.2, 135.8, 135.5, 133.7, 131.5 (q, *J* = 32.6 Hz), 130.3, 129.7 (3C), 129.3, 128.8 (2C), 128.3, 128.2 (2C), 125.80 (q, *J* = 3.8 Hz, 2C), 123.89 (q, *J* = 272.3 Hz), 120.2, 54.3.

^{19}F NMR (470 MHz, CDCl$_3$) δ -62.9 (s, 3F).

HRMS(ESI): $[M+H]^+$ calcd. $C_{24}H_{19}NO_2F_3$ m/z 410.1362, found 410.1358.

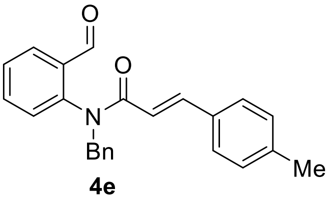

4e

(*E*)-*N*-benzyl-*N*-(2-formylphenyl)-3-(*p*-tolyl)acrylamide (4e)

Acrylamide **4e** (141 mg, 0.4 mmol, 40% Yield) was synthesized according to the general procedure 2 from **3** in 1.0 mmol scale; column chromatography eluting with cyclohexane/EA 5:1 to 3:1.

^{1}H NMR (700 MHz, CDCl$_3$) δ 9.67 (s, 1H), 7.94 (dd, *J* = 7.8, 1.7 Hz, 1H), 7.75 (d, *J* = 15.4 Hz, 1H), 7.65 (td, *J* = 7.6, 1.7 Hz, 1H), 7.53 (t, *J* = 7.6 Hz, 1H), 7.27-7.24 (m, 3H), 7.22-7.19 (m, 2H), 7.16 (dd, *J* = 7.9, 1.1 Hz, 1H), 7.13 (d, *J* = 8.1 Hz, 2H), 7.06 (d, *J* = 7.9 Hz, 2H), 6.02 (d, *J* = 15.4 Hz, 1H), 5.15 – 4.99 (m, 2H), 2.30 (s, 3H).

^{13}C NMR (176 MHz, CDCl$_3$) δ 189.1, 166.3, 144.2, 144.0, 140.5, 136.1, 135.4, 133.8, 132.1, 130.3, 129.7 (2C), 129.6 (2C), 129.3, 129.1, 128.8 (2C), 128.2, 128.1 (2C), 116.6, 54.2, 21.5.

HRMS(ESI): $[M+H]^+$ calcd. $C_{24}H_{22}NO_2$ m/z 356.1645, found 356.1646.

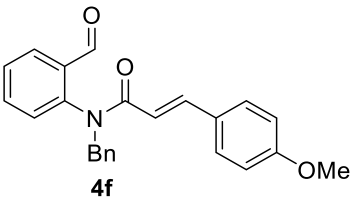

4f

(*E*)-*N*-benzyl-*N*-(2-formylphenyl)-3-(4-methoxyphenyl)acrylamide (4f)

Acrylamide **4f** (90 mg, 0.24 mmol, 24% Yield) was synthesized according to the general procedure 2 from **3** in 1.0 mmol scale; column chromatography eluting with cyclohexane/EA 5:1 to 2:1.

^{1}H NMR (500 MHz, CDCl$_3$) δ 9.65 (s, 1H), 7.94 (dd, *J* = 7.8, 1.7 Hz, 1H), 7.73 (d, *J* = 15.4 Hz, 1H), 7.65 (td, *J* = 7.7, 1.7 Hz, 1H), 7.53 (t, *J* = 7.6 Hz, 1H), 7.26 – 7.23 (m, 3H), 7.22 – 7.15 (m, 5H), 6.85 – 6.72 (m, 2H), 5.93 (d, *J* = 15.3 Hz, 1H), 5.15 – 4.97 (m, 2H), 3.77 (s, 3H).

^{13}C NMR (126 MHz, CDCl$_3$) δ 189.2, 166.4, 161.2, 144.1, 143.9, 136.1, 135.5, 133.8, 130.3, 129.8 (2C), 129.6 (2C), 129.2, 129.0, 128.8 (2C), 128.2, 127.5, 115.1, 114.3 (2C), 55.5, 54.2.

HRMS(ESI): $[M+H]^+$ calcd. $C_{24}H_{22}NO_3$ m/z 372.2594, found 372.1596.

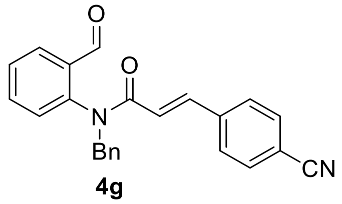

4g

(*E*)-*N*-benzyl-3-(4-cyanophenyl)-*N*-(2-formylphenyl)acrylamide (4g)

Acrylamide **4g** (130 mg, 0.36 mmol, 36% Yield) was synthesized according to the general procedure 2 from **3** in 1.0 mmol scale; column chromatography eluting with cyclohexane/EA 5:1 to 3:1.

^{1}H NMR (700 MHz, CDCl$_3$) δ 9.65 (s, 1H), 7.95 (dd, *J* = 7.7, 1.7 Hz, 1H), 7.74 (d, *J* = 15.5 Hz, 1H), 7.67 (td, *J* = 7.6, 1.7 Hz, 1H), 7.56 (t, *J* = 7.6 Hz, 1H), 7.54 – 7.52 (m, 2H), 7.32 – 7.29 (m, 2H), 7.27-7.23 (m, 3H), 7.20-7.17 (m, 2H), 7.16 (d, *J* = 7.9 Hz, 1H), 6.14 (d, *J* = 15.4 Hz, 1H), 5.06 (s, 2H).

^{13}C NMR (176 MHz, CDCl$_3$) δ 188.9, 165.1, 143.2, 141.7, 139.1, 135.7, 135.6, 133.7, 132.6 (2C), 130.2, 129.9, 129.7 (2C), 129.4, 128.8 (2C), 128.4 (2C), 128.3, 121.1, 118.5, 113.1, 54.3.

HRMS(ESI): $[M+H]^+$ calcd. $C_{24}H_{19}N_2O_2$ m/z 367.1441, found 367.1443.

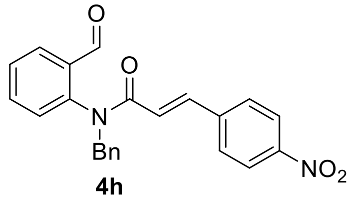

4h

(*E*)-*N*-benzyl-*N*-(2-formylphenyl)-3-(4-nitrophenyl)acrylamide (4h)

Acrylamide **4h** (201 mg, 0.52 mmol, 52% Yield) was synthesized according to the general procedure 2 from **3** in 1.0 mmol scale; column chromatography eluting with cyclohexane/EA 5:1 to 3:1.

^{1}H NMR (700 MHz, CDCl$_3$) δ 9.66 (s, 1H), 8.09 (d, *J* = 8.7 Hz, 2H), 7.95 (dd, *J* = 7.8, 1.7 Hz, 1H), 7.79 (d, *J* = 15.4 Hz, 1H), 7.68 (td, *J* = 7.6, 1.7 Hz, 1H), 7.57 (t, *J* = 7.6 Hz, 1H), 7.36 (d, *J* = 8.9 Hz, 1H), 7.28 – 7.23 (m, 3H), 7.21-7.16 (m, 3H), 6.20 (d, *J* = 15.4 Hz, 1H), 5.10-5.04 (m, 2H).

^{13}C NMR (176 MHz, CDCl$_3$) δ 188.9, 165.0, 148.3, 143.0, 141.1, 140.9, 135.7, 135.5, 133.6, 130.2, 129.9, 129.6 (2C), 129.4, 128.8 (2C), 128.6 (2C), 128.3, 124.1 (2C), 121.9, 54.3.

HRMS(ESI): $[M+H]^+$ calcd. $C_{23}H_{19}NO_2Cl$ m/z 387.1339, found 387.1344.

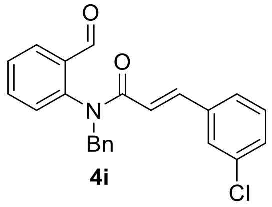

4i

(*E*)-*N*-benzyl-3-(3-chlorophenyl)-*N*-(2-formylphenyl)acrylamide (4i)

Acrylamide **4i** (292 mg, 0.78 mmol, 65% Yield) was synthesized according to the general procedure 2 from **3** in 1.2 mmol scale; column chromatography eluting with cyclohexane/EA 5:1 to 3:1.

^{1}H NMR (500 MHz, CDCl$_3$) δ 9.65 (s, 1H), 7.96 (dd, *J* = 7.8, 1.7 Hz, 1H), 7.74 – 7.62 (m, 2H), 7.56 (t, *J* = 7.6 Hz, 1H), 7.29-7.24 (m, 4H), 7.22 – 7.15 (m, 5H), 7.11 (dt, *J* = 7.7, 1.5 Hz, 1H), 6.06 (d, *J* = 15.4 Hz, 1H), 5.15 – 4.93 (m, 2H).

^{13}C NMR (126 MHz, CDCl$_3$) δ 189.0, 165.6, 143.5, 142.7, 136.6, 135.9, 135.6, 134.8, 133.7, 130.3, 130.1, 130.0, 129.7 (2C), 129.7, 129.3, 128.8 (2C), 128.3, 127.7, 126.5, 119.0, 54.3.

HRMS(ESI): $[M+H]^+$ calcd. $C_{23}H_{19}NO_2Cl$ m/z 376.1099, found 376.1099.

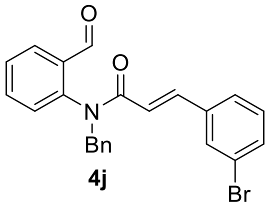

4j

(*E*)-*N*-benzyl-3-(3-bromophenyl)-*N*-(2-formylphenyl)acrylamide (4j)

Acrylamide **4j** (338 mg, 0.8 mmol, 67% Yield) was synthesized according to the general procedure 2 from **3** in 1.2 mmol scale; column chromatography eluting with cyclohexane/EA 5:1 to 3:1.

^{1}H NMR (700 MHz, CDCl$_3$) δ 9.67 (s, 1H), 7.95 (dd, *J* = 7.8, 1.7 Hz, 1H), 7.73 – 7.64 (m, 2H), 7.55 (t, *J* = 7.6 Hz, 1H), 7.39 (dt, *J* = 7.8, 1.6 Hz, 1H), 7.35 (t, *J* = 1.9 Hz, 1H), 7.28-7.24 (m, 3H), 7.21 – 7.18 (m, 2H), 7.17 – 7.15 (m, 2H), 7.12 (t, *J* = 7.8 Hz, 1H), 6.07 (d, *J* = 15.4 Hz, 1H), 5.09-5.03 (m, 2H).

^{13}C NMR (176 MHz, CDCl$_3$) δ 188.9, 165.5, 143.4, 142.4, 136.9, 135.8, 135.5, 133.6, 132.8, 130.6, 130.3, 130.2, 129.7, 129.6 (2C), 129.3, 128.8 (2C), 128.2, 126.7, 122.9, 119.0, 54.2.

HRMS(ESI): $[M+H]^+$ calcd. $C_{23}H_{19}NO_2Br$ m/z 420.0594, found 420.0594.

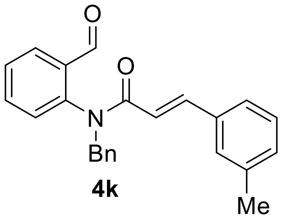

4k

(*E*)-*N*-benzyl-*N*-(2-formylphenyl)-3-(*m*-tolyl)acrylamide (4k)

Acrylamide **4k** (288 mg, 0.81 mmol, 68% Yield) was synthesized according to the general procedure 2 from **3** in 1.2 mmol scale; column chromatography eluting with cyclohexane/EA 5:1 to 3:1.

^{1}H NMR (500 MHz, CDCl$_3$) δ 9.66 (s, 1H), 7.95 (dd, *J* = 7.7, 1.7 Hz, 1H), 7.75 (d, *J* = 15.4 Hz, 1H), 7.66 (td, *J* = 7.7, 1.7 Hz, 1H), 7.54 (t, *J* = 7.6 Hz, 1H), 7.28-7.23 (m, 3H), 7.22 – 7.12 (m, 4H), 7.09 (d, *J* = 7.4 Hz, 1H), 7.05 – 7.01 (m, 2H), 6.05 (d, *J* = 15.4 Hz, 1H), 5.15 – 4.97 (m, 2H), 2.27 (s, 3H).

^{13}C NMR (126 MHz, CDCl$_3$) δ 189.1, 166.1, 144.5, 143.9, 138.5, 136.0, 135.5, 134.7, 133.7, 131.0, 130.3, 129.7 (2C), 129.3, 129.1, 128.9, 128.8 (2C), 128.7, 128.2, 125.2, 117.4, 54.2, 21.4.

HRMS(ESI): $[M+H]^+$ calcd. $C_{24}H_{22}NO_2$ m/z 356.1645, found 356.1645.

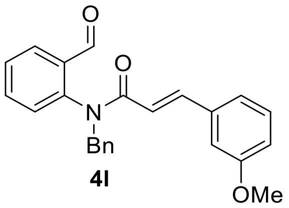

4l

(*E*)-*N*-benzyl-*N*-(2-formylphenyl)-3-(3-methoxyphenyl)acrylamide (4l)

Acrylamide **4g** (252 mg, 0.68 mmol, 57% Yield) was synthesized according to the general procedure 2 from **3** in 1.2 mmol scale; column chromatography eluting with cyclohexane/EA 5:1 to 2:1.

^{1}H NMR (500 MHz, CDCl$_3$) δ 9.66 (s, 1H), 7.94 (dd, *J* = 7.8, 1.7 Hz, 1H), 7.73 (d, *J* = 15.4 Hz, 1H), 7.65 (td, *J* = 7.7, 1.7 Hz, 1H), 7.53 (t, *J* = 7.6 Hz, 1H), 7.28-7.24 (m, 3H), 7.22 – 7.15 (m, 4H), 6.89 – 6.80 (m, 2H), 6.76 (t, *J* = 2.1 Hz, 1H), 6.05 (d, *J* = 15.4 Hz, 1H), 5.14 – 4.99 (m, 2H), 3.74 (s, 3H).

^{13}C NMR (126 MHz, CDCl$_3$) δ 189.1, 166.0, 159.8, 144.1, 143.8, 136.2, 136.0, 135.5, 133.7, 130.3, 129.9, 129.7 (2C), 129.4, 129.2, 128.8 (2C), 128.2, 120.6, 118.0, 115.3, 113.8, 55.4, 54.2.

HRMS(ESI): $[M+H]^+$ calcd. $C_{24}H_{22}NO_3$ m/z 372.1594, found 372.1592.

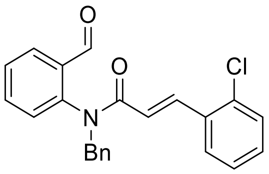

4m

(*E*)-*N*-benzyl-3-(2-chlorophenyl)-*N*-(2-formylphenyl)acrylamide (4m)

Acrylamide **4m** (247 mg, 0.66 mmol, 55% Yield) was synthesized according to the general procedure 2 from **3** in 1.2 mmol scale; column chromatography eluting with cyclohexane/EA 5:1 to 3:1.

^{1}H NMR (700 MHz, CDCl$_3$) δ 9.70 (s, 1H), 8.16 (d, *J* = 15.4 Hz, 1H), 7.95 (dd, *J* = 7.8, 1.7 Hz, 1H), 7.67 (td, *J* = 7.6, 1.7 Hz, 1H), 7.54 (t, *J* = 7.6 Hz, 1H), 7.33 (d, *J* = 8.1 Hz, 1H), 7.30 – 7.25 (m, 3H), 7.24-7.22 (m, 2H), 7.21-7.18 (m, 2H), 7.13 – 7.06 (m, 2H), 6.13 (d, *J* = 15.4 Hz, 1H), 5.19 – 4.97 (m, 2H).

^{13}C NMR (176 MHz, CDCl$_3$) δ 188.9, 165.4, 143.5, 139.9, 135.8, 135.4, 134.8, 133.6, 133.0, 130.7, 130.2, 130.1, 129.6 (2C), 129.4, 129.1, 128.7 (2C), 128.1, 127.7, 126.9, 120.5, 54.1.

HRMS(ESI): $[M+H]^+$ calcd. $C_{23}H_{19}NO_2Cl$ m/z 376.1099, found 376.1099.

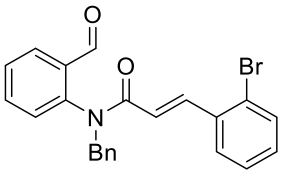

4n

(*E*)-*N*-benzyl-3-(2-bromophenyl)-*N*-(2-formylphenyl)acrylamide (4n)

Acrylamide **4n** (232 mg, 0.55 mmol, 46% Yield) was synthesized according to the general procedure 2 from **3** in 1.2 mmol scale; column chromatography eluting with cyclohexane/EA 5:1 to 3:1.

^{1}H NMR (700 MHz, CDCl$_3$) δ 9.67 (s, 1H), 8.09 (d, *J* = 15.4 Hz, 1H), 7.94 (dd, *J* = 7.7, 1.7 Hz, 1H), 7.65 (td, *J* = 7.6, 1.7 Hz, 1H), 7.57 – 7.49 (m, 2H), 7.29 – 7.24 (m, 3H), 7.23 – 7.19 (m, 2H), 7.17 (dd, *J* = 7.9, 1.1 Hz, 1H), 7.15-7.10 (m, 2H), 7.07 (dd, *J* = 7.3, 2.3 Hz, 1H), 6.04 (d, *J* = 15.4 Hz, 1H), 5.26 – 4.88 (m, 2H).

^{13}C NMR (176 MHz, CDCl$_3$) δ 189.1, 165.5, 143.7, 142.6, 135.9, 135.5, 135.0, 133.8, 133.5, 130.9, 130.3, 129.7 (2C), 129.5, 129.2, 128.8 (2C), 128.3, 128.0, 127.6, 125.3, 120.7, 54.3.

HRMS(ESI): $[M+H]^+$ calcd. $C_{23}H_{19}NO_2Br$ m/z 420.0594, found 420.0593.

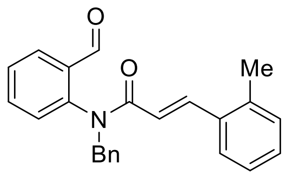

4o

(*E*)-*N*-benzyl-*N*-(2-formylphenyl)-3-(*o*-tolyl)acrylamide (4o)

Acrylamide **4o** (224 mg, 0.63 mmol, 53% Yield) was synthesized according to the general procedure 2 from **3** in 1.2 mmol scale; column chromatography eluting with cyclohexane/EA 5:1 to 3:1.

^{1}H NMR (700 MHz, $CDCl_3$) δ 9.68 (s, 1H), 8.06 (d, *J* = 15.3 Hz, 1H), 7.94 (dd, *J* = 7.8, 1.7 Hz, 1H), 7.65 (td, *J* = 7.6, 1.7 Hz, 1H), 7.52 (t, *J* = 7.6 Hz, 1H), 7.29 – 7.24 (m, 3H), 7.23-7.21 (m, 2H), 7.20-7.15 (m, 2H), 7.12 (d, *J* = 7.5 Hz, 1H), 7.06 – 7.01 (m, 2H), 6.00 (d, *J* = 15.3 Hz, 1H), 5.21 – 4.98 (m, 2H), 2.37 (s, 3H).

^{13}C NMR (176 MHz, $CDCl_3$) δ 189.0, 166.1, 143.8, 141.9, 137.7, 136.0, 135.4, 133.9, 133.7, 130.8, 130.1, 129.7, 129.6 (2C), 129.3, 129.0, 128.7 (2C), 128.1, 126.4, 126.1, 119.0, 54.2, 19.9.

HRMS(ESI): $[M+H]^+$ calcd. $C_{24}H_{22}NO_2$ m/z 356.1645, found 356.1644.

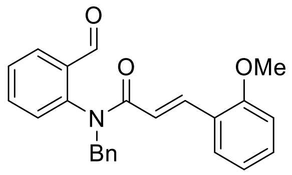

4p

(*E*)-*N*-benzyl-*N*-(2-formylphenyl)-3-(2-methoxyphenyl)acrylamide (4p)

Acrylamide **4p** (126 mg, 0.34 mmol, 28% Yield) was synthesized according to the general procedure 2 from **3** in 1.2 mmol scale; column chromatography eluting with cyclohexane/EA 5:1 to 2:1.

^{1}H NMR (700 MHz, $CDCl_3$) δ 9.67 (s, 1H), 7.98 (d, *J* = 15.5 Hz, 1H), 7.94 (dd, *J* = 7.7, 1.7 Hz, 1H), 7.64 (td, *J* = 7.6, 1.7 Hz, 1H), 7.51 (t, *J* = 7.6 Hz, 1H), 7.27 – 7.19 (m, 6H), 7.17 (dd, *J* = 7.9, 1.1 Hz, 1H), 7.14 (dd, *J* = 7.7, 1.7 Hz, 1H), 6.86 – 6.75 (m, 2H), 6.26 (d, *J* = 15.5 Hz, 1H), 5.14 (d, *J* = 14.1 Hz, 1H), 5.01 (d, *J* = 14.0 Hz, 1H), 3.67 (s, 3H).

^{13}C NMR (176 MHz, $CDCl_3$) δ 189.2, 166.7, 158.5, 144.3, 139.9, 136.2, 135.4, 133.9, 131.2, 130.3, 129.8, 129.7 (2C), 129.0, 128.8, 128.8 (2C), 128.2, 123.9, 120.6, 118.9, 111.2, 55.3, 54.1.

HRMS(ESI): $[M+H]^+$ calcd. $C_{24}H_{22}NO_3$ m/z 372.1594, found 372.1594.

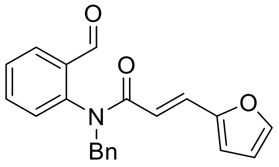

4q

(*E*)-*N*-benzyl-*N*-(2-formylphenyl)-3-(furan-2-yl)acrylamide (4q)

Acrylamide **4q** (77 mg, 0.23 mmol, 19% Yield) was synthesized according to the general procedure 2 from **3** in 1.2 mmol scale; column chromatography eluting with cyclohexane/EA 5:1 to 3:1.

^{1}H NMR (700 MHz, CDCl$_3$) δ 9.65 (s, 1H), 7.94 (dd, *J* = 7.8, 1.7 Hz, 1H), 7.64 (td, *J* = 7.6, 1.7 Hz, 1H), 7.55 – 7.49 (m, 2H), 7.28 (d, *J* = 1.8 Hz, 1H), 7.26-7.23 (m, 3H), 7.20 – 7.16 (m, 2H), 7.14 (dd, *J* = 7.9, 1.2 Hz, 1H), 6.50 (d, *J* = 3.4 Hz, 1H), 6.37 (dd, *J* = 3.4, 1.8 Hz, 1H), 5.95 (d, *J* = 15.1 Hz, 1H), 5.13 – 4.97 (m, 2H).

^{13}C NMR (176 MHz, CDCl$_3$) δ 189.2, 166.1, 151.3, 144.5, 143.8, 136.1, 135.5, 133.8, 130.6, 130.3, 129.7 (2C), 129.4, 129.1, 128.8 (2C), 128.2, 115.2, 115.0, 112.3, 54.2.

HRMS(ESI): $[M+H]^+$ calcd. $C_{21}H_{18}NO_3$ m/z 332.1281, found 332.1282.

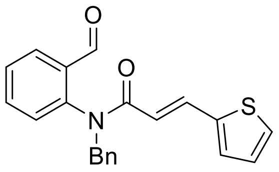

4r

(*E*)-*N*-benzyl-*N*-(2-formylphenyl)-3-(thiophen-2-yl)acrylamide (4r)

Acrylamide **4r** (143 mg, 0.41 mmol, 34% Yield) was synthesized according to the general procedure 2 from **3** in 1.2 mmol scale; column chromatography eluting with cyclohexane/EA 5:1 to 3:1.

^{1}H NMR (700 MHz, CDCl$_3$) δ 9.67 (s, 1H), 7.95 (dd, *J* = 7.8, 1.7 Hz, 1H), 7.87 (d, *J* = 15.1 Hz, 1H), 7.65 (td, *J* = 7.6, 1.7 Hz, 1H), 7.53 (t, *J* = 7.6 Hz, 1H), 7.27-7.24 (m, 3H), 7.23 (d, *J* = 5.0 Hz, 1H), 7.21-7.18 (m, 2H), 7.15 (dd, *J* = 7.9, 1.2 Hz, 1H), 7.12 (d, *J* = 3.7 Hz, 1H), 6.96 (dd, *J* = 5.1, 3.6 Hz, 1H), 5.86 (d, *J* = 15.1 Hz, 1H), 5.09-5.00 (m, 2H).

^{13}C NMR (176 MHz, CDCl$_3$) δ 189.1, 165.9, 143.7, 140.0, 136.6, 136.0, 135.4, 133.7, 130.8, 130.3, 129.6 (2C), 129.4, 129.1, 128.8 (2C), 128.2, 128.1, 128.1, 116.5, 54.2.

HRMS(ESI): $[M+H]^+$ calcd. $C_{21}H_{18}NO_2S$ m/z 348.1053, found 348.1053.

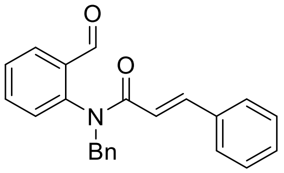

4s

***N*-benzyl-*N*-(2-formylphenyl)cinnamamide (4s)**

Acrylamide **4s** (559 mg, 1.63 mmol, 55% Yield) was synthesized according to the general procedure 2 from **3** in 3.0 mmol scale; column chromatography eluting with cyclohexane/EA 5:1 to 3:1.

^{1}H NMR (500 MHz, CDCl$_3$) δ 9.66 (s, 1H), 7.95 (dd, *J* = 7.8, 1.7 Hz, 1H), 7.78 (d, *J* = 15.4 Hz, 1H), 7.66 (td, *J* = 7.6, 1.7 Hz, 1H), 7.54 (t, *J* = 7.6 Hz, 1H), 7.29 – 7.22 (m, 8H), 7.22-7.19 (m, 2H), 7.16 (dd, *J* = 7.9, 1.1 Hz, 1H), 6.07 (d, *J* = 15.4 Hz, 1H), 5.16 – 4.97 (m, 2H).

^{13}C NMR (126 MHz, CDCl$_3$) δ 189.1, 166.1, 144.2, 143.8, 136.0, 135.5, 134.8, 133.8, 130.3, 130.1, 129.7 (2C), 129.4, 129.1, 128.9 (2C), 128.8 (2C), 128.2, 128.1 (2C), 117.7, 54.2.

HRMS(ESI): $[M+H]^+$ calcd. $C_{23}H_{20}NO_2$ m/z 342.1489, found 342.1486.

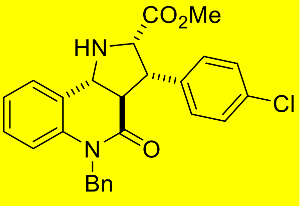

B1

(±)-Methyl (2*S*,3*S*,3a*R*,9b*R*)-5-benzyl-3-(4-chlorophenyl)-4-oxo-2,3,3a,4,5,9b-hexahydro-*1H*-pyrrolo[3,2-*c*]quinoline-2-carboxylate (B1)

PQ **B1** (87 mg, 0.2 mmol, 89% Yield, >20:1 d.r.) was synthesized according to the general procedure 3; column chromatography eluting with cyclohexane/EA 5:1 to 2:1.

^{1}H NMR (500 MHz, CDCl$_3$) δ 7.43 (dt, *J* = 7.4, 1.4 Hz, 1H), 7.29 – 7.26 (m, 2H), 7.25 – 7.19 (m, 4H), 7.18 – 7.10 (m, 5H), 7.00 (d, *J* = 8.1 Hz, 1H), 5.25 (d, *J* = 16.2 Hz, 1H), 5.02 (s, 1H), 4.48 (d, *J* = 10.4 Hz, 1H), 4.22 (d, *J* = 13.4 Hz, 1H), 4.04 (t, *J* = 10.7 Hz, 1H), 3.21 (s, 3H), 3.01 (dd, *J* = 13.5, 11.1 Hz, 1H).

^{13}C NMR (126 MHz, CDCl$_3$) δ 172.4, 169.5, 139.5, 137.3, 137.0, 133.1, 129.5 (2C), 128.9 (2C), 128.7 (2C), 128.4, 127.4, 126.7 (2C), 123.8, 123.3, 116.5, 66.7, 60.2, 54.5, 52.0, 49.1, 46.3.

HRMS(ESI): $[M+H]^+$ calcd. $C_{26}H_{24}N_2O_3Cl$ m/z 447.1470, found 447.1468.

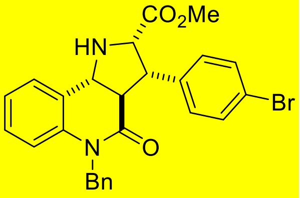

B2

(±)-Methyl (2*S*,3*S*,3a*R*,9b*R*)-5-benzyl-3-(4-bromophenyl)-4-oxo-2,3,3a,4,5,9b-hexahydro-*1H*-pyrrolo[3,2-*c*]quinoline-2-carboxylate (B2)

PQ **B2** (81 mg, 0.17 mmol, 75% Yield, >20:1 d.r.) was synthesized according to the general procedure 3; column chromatography eluting with cyclohexane/EA 5:1 to 2:1.

^{1}H NMR (500 MHz, CDCl$_3$) δ 7.45 – 7.39 (m, 3H), 7.30 – 7.25 (m, 2H), 7.24 – 7.19 (m, 2H), 7.18 – 7.15 (m, 2H), 7.15 – 7.08 (m, 3H), 7.00 (d, *J* = 8.1 Hz, 1H), 5.26 (d, *J* = 16.2 Hz, 1H), 5.01 (d, *J* = 16.2 Hz, 1H), 4.48 (d, *J* = 10.4 Hz, 1H), 4.22 (d, *J* = 13.4 Hz, 1H), 4.03 (t, *J* = 10.7 Hz, 1H), 3.21 (s, 3H), 3.01 (dd, *J* = 13.5, 11.0 Hz, 1H).

^{13}C NMR (126 MHz, CDCl$_3$) δ 172.4, 169.5, 139.5, 137.9, 136.9, 131.6 (2C), 129.9 (2C), 129.5, 128.9 (2C), 128.4, 127.4, 126.7 (2C), 123.8, 123.3, 121.2, 116.4, 66.6, 60.2, 54.5, 52.0, 49.1, 46.3.

HRMS(ESI): $[M+H]^+$ calcd. $C_{26}H_{24}N_2O_3Br$ m/z 491.0965, found 491.0961.

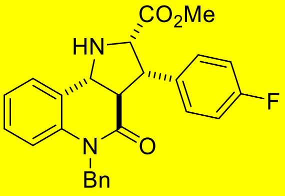

B3

(±)-Methyl (2*S*,3*S*,3a*R*,9b*R*)-5-benzyl-3-(4-fluorophenyl)-4-oxo-2,3,3a,4,5,9b-hexahydro-*1H*-pyrrolo[3,2-*c*]quinoline-2-carboxylate (B3)

PQ **B3** (63 mg, 0.15 mmol, 67% Yield, >20:1 d.r.) was synthesized according to the general procedure 3; column chromatography eluting with cyclohexane/EA 5:1 to 2:1.

^{1}H NMR (500 MHz, CDCl$_3$) δ 7.44 (d, *J* = 7.5 Hz, 1H), 7.32 – 7.11 (m, 9H), 7.03-6.94 (m, 3H), 5.25 (d, *J* = 16.0 Hz, 1H), 5.02 (d, *J* = 16.2 Hz, 1H), 4.48 (d, *J* = 10.4 Hz, 1H), 4.22 (d, *J* = 13.4 Hz, 1H), 4.10 – 3.99 (m, 1H), 3.20 (s, 3H), 3.02 (dd, *J* = 13.3, 11.0 Hz, 1H).

^{13}C NMR (126MHz, CDCl$_3$) δ 172.5, 169.6, 162.1 (d, *J*CF = 245.7 Hz), 139.5, 137.0, 134.5 (d, *J*CF = 2.5 Hz), 129.7 (d, *J*CF = 7.6 Hz, 2C), 129.6, 128.9 (2C), 128.4, 127.4, 126.7 (2C), 123.8, 123.3, 116.4, 115.4 (d, *J*CF = 21.4 Hz, 2C), 66.7, 60.2, 54.5, 52.0, 49.0, 46.3.

^{19}F NMR (470 MHz, CDCl$_3$) δ -115.6 (ddd, *J* = 13.8, 8.8, 5.3 Hz, 1F).

HRMS(ESI): $[M+H]^+$ calcd. $C_{26}H_{24}N_2O_3F$ m/z 431.1766, found 431.1764.

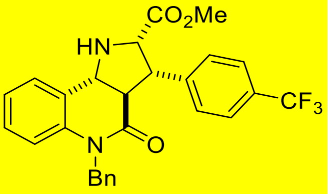

B4

(±)-Methyl (2*S*,3*S*,3a*R*,9b*R*)-5-benzyl-4-oxo-3-(4-(trifluoromethyl)phenyl)-2,3,3a,4,5,9b-hexahydro-*1H*-pyrrolo[3,2-*c*]quinoline-2-carboxylate (B4)

PQ **B4** (84 mg, 0.18 mmol, 80% Yield, >20:1 d.r.) was synthesized according to the general procedure 3; column chromatography eluting with cyclohexane/EA 5:1 to 2:1.

^{1}H NMR (500 MHz, CDCl$_3$) δ 7.55 (d, *J* = 8.1 Hz, 2H), 7.44 (d, *J* = 7.4 Hz, 1H), 7.35 (d, *J* = 8.1 Hz, 2H), 7.30 – 7.26 (m, 2H), 7.24 – 7.12 (m, 5H), 7.01 (d, *J* = 8.1 Hz, 1H), 5.26 (d, *J* = 16.2 Hz, 1H), 5.02 (d, *J* = 16.2 Hz, 1H), 4.53 (d, *J* = 10.4 Hz, 1H), 4.26 (d, *J* = 13.4 Hz, 1H), 4.13 (t, *J* = 10.7 Hz, 1H), 3.15 (s, 3H), 3.08 (dd, *J* = 13.4, 11.1 Hz, 1H).

^{13}C NMR (126 MHz, CDCl$_3$) δ 172.2, 169.3, 143.0, 139.4, 136.9, 129.6 (q, *J*CF = 32.8 Hz), 129.3, 128.9 (2C), 128.6 (2C), 128.5, 127.4, 126.7 (2C), 125.4 (q, *J*CF = 5.0 Hz, 2C), 124.2 (d, *J*CF = 272.2 Hz), 123.9, 123.4, 116.5, 66.7, 60.2, 54.4, 51.9, 49.4, 46.4.

^{19}F NMR (470 MHz, CDCl$_3$) δ -62.5 (s, 3F).

HRMS(ESI): $[M+H]^+$ calcd. $C_{27}H_{24}N_2O_3F_3$ m/z 481.1734, found 481.1727.

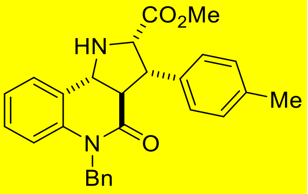

B5

(±)-Methyl (2*S*,3*S*,3a*R*,9b*R*)-5-benzyl-4-oxo-3-(*p*-tolyl)-2,3,3a,4,5,9b-hexahydro-*1H*-pyrrolo[3,2-*c*]quinoline-2-carboxylate (B5)

PQ **B5** (71 mg, 0.17 mmol, 75% Yield, >20:1 d.r.) was synthesized according to the general procedure 3; column chromatography eluting with cyclohexane/EA 5:1 to 2:1.

^{1}H NMR (500 MHz, CDCl$_3$) δ 7.44 (d, *J* = 7.4 Hz, 1H), 7.29 – 7.24 (m, 2H), 7.23 – 7.16 (m, 4H), 7.15 – 7.06 (m, 5H), 6.99 (d, *J* = 8.1 Hz, 1H), 5.25 (d, *J* = 16.2 Hz, 1H), 5.01 (d, *J* = 16.2 Hz, 1H), 4.47 (d, *J* = 10.4 Hz, 1H), 4.21 (d, *J* = 13.5 Hz, 1H), 4.04 (t, *J* = 10.7 Hz, 1H), 3.17 (s, 3H), 3.05 (dd, *J* = 13.5, 11.1 Hz, 1H), 2.29 (s, 3H).

^{13}C NMR (126MHz, CDCl$_3$) δ 172.6, 169.7, 139.6, 137.1, 136.8, 135.6, 129.7, 129.2 (2C), 128.8 (2C), 128.3, 127.9 (2C), 127.3, 126.7 (2C), 123.7, 123.3, 116.3, 66.9, 60.2, 54.4, 51.9, 49.4, 46.2, 21.2.

HRMS(ESI): $[M+H]^+$ calcd. $C_{27}H_{27}N_2O_3$ m/z 427.2016, found 427.2014.

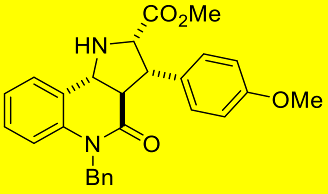

B6

(±)-Methyl (2*S*,3*S*,3a*R*,9b*R*)-5-benzyl-3-(4-methoxyphenyl)-4-oxo-2,3,3a,4,5,9b-hexahydro-*1H*-pyrrolo[3,2-*c*]quinoline-2-carboxylate (B6)

PQ **B6** (53 mg, 0.12 mmol, 55% Yield, >20:1 d.r.) was synthesized according to the general procedure 3; column chromatography eluting with cyclohexane/EA 5:1 to 1:1.

^{1}H NMR (400 MHz, CDCl$_3$) δ 7.44 (dt, *J* = 7.4, 1.5 Hz, 1H), 7.29 – 7.25 (m, 2H), 7.24 – 7.10 (m, 7H), 6.99 (dd, *J* = 8.1, 1.1 Hz, 1H), 6.85 – 6.77 (m, 2H), 5.25 (d, *J* = 16.2 Hz, 1H), 5.02 (d, *J* = 16.2 Hz, 1H), 4.46 (d, *J* = 10.4 Hz, 1H), 4.21 (d, *J* = 13.5 Hz, 1H), 4.03 (t, *J* = 10.7 Hz, 1H), 3.76 (s, 3H), 3.20 (s, 3H), 3.02 (dd, *J* = 13.5, 11.1 Hz, 1H).

^{13}C NMR (101 MHz, CDCl$_3$) δ 172.7, 169.7, 158.8, 139.6, 137.1, 130.7, 129.7, 129.1 (2C), 128.9 (2C), 128.3, 127.3, 126.7 (2C), 123.7, 123.3, 116.4, 114.0 (2C), 66.8, 60.2, 55.4, 54.3, 52.0, 49.0, 46.3.

HRMS(ESI): $[M+H]^+$ calcd. $C_{27}H_{27}N_2O_4$ m/z 443.1965, found 443.1963.

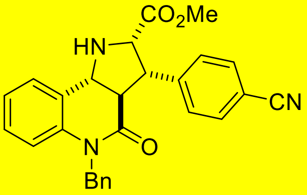

B7

(±)-Methyl (2*S*,3*S*,3a*R*,9b*R*)-5-benzyl-3-(4-cyanophenyl)-4-oxo-2,3,3a,4,5,9b-hexahydro-*1H*-pyrrolo[3,2-*c*]quinoline-2-carboxylate (B7)

PQ **B7** (60 mg, 0.14 mmol, 62% Yield, >20:1 d.r.) was synthesized according to the general procedure 3; column chromatography eluting with cyclohexane/EA 5:1 to 2:1.

^{1}H NMR (400 MHz, CDCl$_3$) δ 7.76 – 7.55 (m, 2H), 7.43 (dt, *J* = 7.4, 1.4 Hz, 1H), 7.37 – 7.32 (m, 2H), 7.30 – 7.25 (m, 2H), 7.24 – 7.19 (m, 2H), 7.18 – 7.12 (m, 3H), 7.02 (dd, *J* = 8.2, 1.1 Hz, 1H), 5.25 (d, *J* = 16.2 Hz, 1H), 5.01 (d, *J* = 16.2 Hz, 1H), 4.54 (d, *J* = 10.4 Hz, 1H), 4.26 (d, *J* = 13.4 Hz, 1H), 4.18 – 4.04 (m, 1H), 3.19 (s, 3H), 3.05 (dd, *J* = 13.4, 11.1 Hz, 1H).

^{13}C NMR (101 MHz, CDCl$_3$) δ 172.0, 169.1, 144.6, 139.4, 136.8, 132.3 (2C), 129.2, 129.1 (2C), 128.9 (2C), 128.6, 127.5, 126.7 (2C), 124.0, 123.4, 118.8, 116.6, 111.3, 66.6, 60.3, 54.5, 52.0, 49.6, 46.4.

HRMS(ESI): $[M+H]^+$ calcd. $C_{27}H_{24}N_3O_3$ m/z 438.1812, found 438.1810.

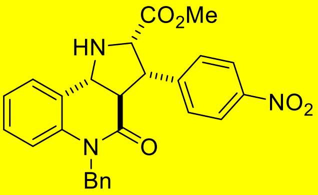

B8

(±)-Methyl (2*S*,3*S*,3a*R*,9b*R*)-5-benzyl-3-(4-nitrophenyl)-4-oxo-2,3,3a,4,5,9b-hexahydro-*1H*-pyrrolo[3,2-*c*]quinoline-2-carboxylate (B8)

PQ **B8** (73 mg, 0.16 mmol, 73% Yield, >20:1 d.r.) was synthesized according to the general procedure 3; column chromatography eluting with cyclohexane/EA 5:1 to 2:1.

^{1}H NMR (400 MHz, $CDCl_3$) δ 8.17 (d, *J* = 8.7 Hz, 2H), 7.50 – 7.38 (m, 3H), 7.33 – 7.11 (m, 7H), 7.03 (d, *J* = 8.2 Hz, 1H), 5.27 (d, *J* = 16.2 Hz, 1H), 5.01 (d, *J* = 16.2 Hz, 1H), 4.57 (d, *J* = 10.4 Hz, 1H), 4.29 (d, *J* = 13.4 Hz, 1H), 4.17 (t, *J* = 10.7 Hz, 1H), 3.21 (s, 3H), 3.10 (dd, *J* = 13.4, 11.1 Hz, 1H).

^{13}C NMR (101 MHz, $CDCl_3$) δ 172.0, 169.1, 147.2, 146.7, 139.4, 136.8, 129.2, 129.1 (2C), 128.9 (2C), 128.5, 127.4, 126.6 (2C), 124.0, 123.7 (2C), 123.4, 116.5, 66.6, 60.2, 54.7, 52.1, 49.3, 46.4.

HRMS(ESI): $[M+H]^+$ calcd. $C_{26}H_{24}N_3O_5$ m/z 458.1711, found 458.1701.

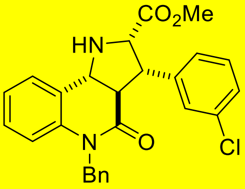

B9

(±)-Methyl (2*S*,3*S*,3a*R*,9b*R*)-5-benzyl-3-(3-chlorophenyl)-4-oxo-2,3,3a,4,5,9b-hexahydro-*1H*-pyrrolo[3,2-*c*]quinoline-2-carboxylate (B9)

PQ **B9** (77 mg, 0.17 mmol, 78% Yield, >20:1 d.r.) was synthesized according to the general procedure 3; column chromatography eluting with cyclohexane/EA 5:1 to 2:1.

^{1}H NMR (500 MHz, $CDCl_3$) δ 7.44 (d, *J* = 7.4 Hz, 1H), 7.30 – 7.26 (m, 2H), 7.25 – 7.16 (m, 7H), 7.15-7.09 (m, 2H), 7.01 (d, *J* = 8.1 Hz, 1H), 5.25 (d, *J* = 16.2 Hz, 1H), 5.03 (d, *J* = 16.2 Hz, 1H), 4.49 (d, *J* = 10.4 Hz, 1H), 4.22 (d, *J* = 13.4 Hz, 1H), 4.03 (t, *J* = 10.7 Hz, 1H), 3.22 (s, 3H), 3.02 (dd, *J* = 13.5, 11.1 Hz, 1H).

^{13}C NMR (126 MHz, $CDCl_3$) δ 172.3, 169.4, 140.9, 139.5, 136.9, 134.3, 129.7, 129.5, 128.9 (2C), 128.4, 128.4, 127.5, 127.4, 126.7 (2C), 126.3, 123.8, 123.3, 116.5, 66.8, 60.2, 54.4, 52.0, 49.3, 46.3.

HRMS(ESI): $[M+H]^+$ calcd. $C_{26}H_{24}N_2O_3Cl$ m/z 447.1470, found 447.1468.

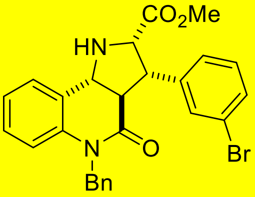

B10

(±)-Methyl (2*S*,3*S*,3a*R*,9b*R*)-5-benzyl-3-(3-bromophenyl)-4-oxo-2,3,3a,4,5,9b-hexahydro-*1H*-pyrrolo[3,2-*c*]quinoline-2-carboxylate (B10)

PQ **B10** (59 mg, 0.12 mmol, 59% Yield, >20:1 d.r.) was synthesized according to the general procedure 3; column chromatography eluting with cyclohexane/EA 5:1 to 2:1.

^{1}H NMR (500 MHz, $CDCl_3$) δ 7.43 (d, *J* = 7.4 Hz, 1H), 7.39 – 7.34 (m, 2H), 7.30-7.26 (m, 2H), 7.25 – 7.11 (m, 7H), 7.01 (d, *J* = 8.2 Hz, 1H), 5.25 (d, *J* = 16.2 Hz, 1H), 5.03 (d, *J* = 16.2 Hz, 1H), 4.49 (d, *J* = 10.4 Hz, 1H), 4.22 (d, *J* = 13.4 Hz, 1H), 4.02 (t, *J* = 10.8 Hz, 1H), 3.22 (s, 3H), 3.01 (dd, *J* = 13.5, 11.1 Hz, 1H).

^{13}C NMR (126 MHz, $CDCl_3$) δ 172.2, 169.4, 141.2, 139.5, 136.9, 131.3, 130.4, 130.1, 129.5, 128.9 (2C), 128.4, 127.4, 126.8, 126.7 (2C), 123.8, 123.3, 122.5, 116.5, 66.8, 60.2, 54.4, 52.0, 49.3, 46.3.

HRMS(ESI): $[M+H]^+$ calcd. $C_{26}H_{24}N_2O_3Br$ m/z 491.0965, found 491.0962.

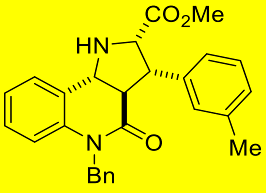

B11

(±)-Methyl (2*S*,3*S*,3a*R*,9b*R*)-5-benzyl-4-oxo-3-(*m*-tolyl)-2,3,3a,4,5,9b-hexahydro-*1H*-pyrrolo[3,2-*c*]quinoline-2-carboxylate (B11)

PQ **B11** (54 mg, 0.13 mmol, 57% Yield, >20:1 d.r.) was synthesized according to the general procedure 3; column chromatography eluting with cyclohexane/EA 5:1 to 2:1.

^{1}H NMR (500 MHz, CDCl$_3$) δ 7.45 (d, *J* = 7.4 Hz, 1H), 7.29 – 7.25 (m, 2H), 7.24 – 7.10 (m, 6H), 7.04 – 6.97 (m, 4H), 5.24 (d, *J* = 16.2 Hz, 1H), 5.03 (d, *J* = 16.2 Hz, 1H), 4.48 (d, *J* = 10.4 Hz, 1H), 4.21 (d, *J* = 13.5 Hz, 1H), 4.03 (t, *J* = 10.7 Hz, 1H), 3.15 (s, 3H), 3.05 (dd, *J* = 13.6, 11.0 Hz, 1H), 2.30 (s, 3H).

^{13}C NMR (126 MHz, CDCl$_3$) δ 172.5, 169.8, 139.6, 138.6, 138.0, 137.1, 129.7, 129.0, 128.8 (2C), 128.4, 128.3, 128.1, 127.3, 126.7 (2C), 125.0, 123.7, 123.3, 116.4, 67.0, 60.3, 54.5, 51.8, 49.7, 46.3, 21.5.

HRMS(ESI): $[M+H]^+$ calcd. $C_{27}H_{27}N_2O_3$ m/z 427.2016 found 427.2014.

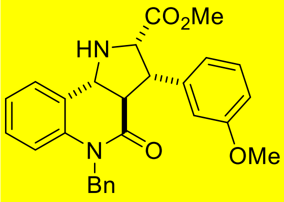

B12

(±)-Methyl (2*S*,3*S*,3a*R*,9b*R*)-5-benzyl-3-(3-methoxyphenyl)-4-oxo-2,3,3a,4,5,9b-hexahydro-*1H*-pyrrolo[3,2-*c*]quinoline-2-carboxylate (B12)

PQ **B12** (66 mg, 0.15 mmol, 68% Yield, >20:1 d.r.) was synthesized according to the general procedure 3; column chromatography eluting with cyclohexane/EA 5:1 to 1:1.

^{1}H NMR (400 MHz, CDCl$_3$) δ 7.45 (d, *J* = 7.4 Hz, 1H), 7.29 – 7.27 (m, 1H), 7.25 – 7.24 (m, 1H), 7.23 – 7.16 (m, 5H), 7.15 – 7.11 (m, 1H), 7.00 (d, *J* = 8.2 Hz, 1H), 6.82 – 6.72 (m, 3H), 5.25 (d, *J* = 16.2 Hz, 1H), 5.03 (d, *J* = 16.2 Hz, 1H), 4.50 (d, *J* = 10.4 Hz, 1H), 4.23 (d, *J* = 13.6 Hz, 1H), 4.04 (t, *J* = 10.7 Hz, 1H), 3.77 (s, 3H), 3.19 (s, 3H), 3.06 (dd, *J* = 13.6, 11.0 Hz, 1H).

^{13}C NMR (101 MHz, CDCl$_3$) δ 172.4, 169.6, 159.7, 140.2, 139.6, 137.1, 129.5, 129.5, 128.9 (2C), 128.4, 127.3, 126.7 (2C), 123.8, 123.4, 120.4, 116.4, 113.8, 113.1, 66.9, 60.3, 55.4, 54.4, 52.0, 49.7, 46.3.

HRMS(ESI): $[M+H]^+$ calcd. $C_{27}H_{27}N_2O_4$ m/z 443.1965, found 443.1955.

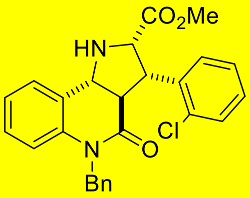

B13

(±)-Methyl (2*S*,3*S*,3a*R*,9b*R*)-5-benzyl-3-(2-chlorophenyl)-4-oxo-2,3,3a,4,5,9b-hexahydro-*1H*-pyrrolo[3,2-*c*]quinoline-2-carboxylate (B13)

PQ **B13** (68 mg, 0.15 mmol, 69% Yield, >20:1 d.r.) was synthesized according to the general procedure 3; column chromatography eluting with cyclohexane/EA 5:1 to 2:1.

^{1}H NMR (400 MHz, CDCl$_3$) δ 7.46 – 7.39 (m, 2H), 7.30-7.27 (m, 2H), 7.25 – 7.06 (m, 8H), 7.01 (d, *J* = 8.2 Hz, 1H), 5.27 (d, *J* = 16.2 Hz, 1H), 5.04 (d, *J* = 16.2 Hz, 1H), 4.67 (d, *J* = 10.1 Hz, 1H), 4.57 (dd, *J* = 11.5, 10.0 Hz, 1H), 4.33 (d, *J* = 13.3 Hz, 1H), 3.22 (dd, *J* = 13.4, 11.4 Hz, 1H), 3.11 (s, 3H).

^{13}C NMR (101 MHz, CDCl$_3$) δ 173.0, 169.2, 139.5, 137.0, 135.8, 135.5, 129.8, 129.6, 128.9 (2C), 128.4, 128.3, 127.4, 126.8, 126.7 (2C), 123.8, 123.4, 116.4, 64.6, 60.0, 51.8, 51.5, 46.3, 46.1.

HRMS(ESI): $[M+H]^+$ calcd. $C_{26}H_{24}N_2O_3Cl$ m/z 447.1470, found 447.1468.

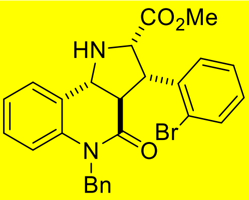

B14

(±)-Methyl (2*S*,3*S*,3a*R*,9b*R*)-5-benzyl-3-(2-bromophenyl)-4-oxo-2,3,3a,4,5,9b-hexahydro-*1H*-pyrrolo[3,2-*c*]quinoline-2-carboxylate (B14)

PQ **B14** (76 mg, 0.15 mmol, 70% Yield, >20:1 d.r.) was synthesized according to the general procedure 3; column chromatography eluting with cyclohexane/EA 5:1 to 2:1.

^{1}H NMR (400 MHz, CDCl$_3$) δ 7.60 (dd, *J* = 7.9, 1.3 Hz, 1H), 7.44 (dt, *J* = 7.4, 1.5 Hz, 1H), 7.31-7.26 (m, 2H), 7.25 – 7.17 (m, 5H), 7.16 – 7.04 (m, 3H), 7.01 (dd, *J* = 8.2, 1.1 Hz, 1H), 5.27 (d, *J* = 16.2 Hz, 1H), 5.03 (d, *J* = 16.2 Hz, 1H), 4.70 (d, *J* = 10.1 Hz, 1H), 4.56 (dd, *J* = 11.6, 10.1 Hz, 1H), 4.34 (d, *J* = 13.3, 1H), 3.22 (dd, *J* = 13.3, 11.5 Hz, 1H), 3.11 (s, 3H).

^{13}C NMR (101 MHz, CDCl$_3$) δ 172.8, 169.1, 139.5, 137.5, 137.0, 132.9, 129.7, 128.9 (2C), 128.6, 128.3, 127.5, 127.4, 127.3, 126.7 (2C), 126.5, 123.8, 123.4, 116.4, 64.5, 59.9, 51.8, 48.7, 46.3.

HRMS(ESI): $[M+H]^+$ calcd. $C_{26}H_{24}N_2O_3Br$ m/z 491.0965, found 491.0960.

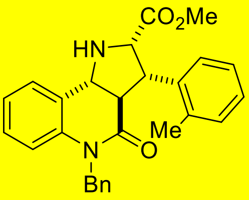

B15

(±)-Methyl (2*S*,3*S*,3a*R*,9b*R*)-5-benzyl-4-oxo-3-(*o*-tolyl)-2,3,3a,4,5,9b-hexahydro-*1H*-pyrrolo[3,2-*c*]quinoline-2-carboxylate (B15)

PQ **B15** (59 mg, 0.14 mmol, 63% Yield, >20:1 d.r.) was synthesized according to the general procedure 3; column chromatography eluting with cyclohexane/EA 5:1 to 2:1.

^{1}H NMR (400 MHz, CDCl$_3$) δ 7.45 (d, *J* = 7.2 Hz, 1H), 7.31 – 7.26 (m, 2H), 7.25 – 7.04 (m, 8H), 7.01-6.96 (m, 2H), 5.28 (d, *J* = 16.1 Hz, 1H), 5.01 (d, *J* = 16.2 Hz, 1H), 4.54 (d, *J* = 10.3 Hz, 1H), 4.33 (t, *J* = 10.7 Hz, 1H), 4.25 (d, *J* = 13.4 Hz, 1H), 3.17 (dd, *J* = 13.4, 10.9 Hz, 1H), 3.07 (s, 3H), 2.53 (s, 3H).

^{13}C NMR (101 MHz, CDCl$_3$) δ 172.6, 169.8, 139.7, 137.4, 137.1, 136.9, 130.3, 129.8, 128.9 (2C), 128.3, 127.3, 126.9, 126.7 (2C), 126.0, 125.6, 123.7, 123.3, 116.4, 65.7, 60.3, 53.9, 51.7, 46.3, 45.1, 20.3.

HRMS(ESI): $[M+H]^+$ calcd. $C_{27}H_{27}N_2O_3$ m/z 427.2016, found 427.2014.

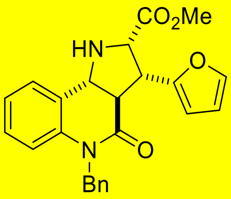

B16

(±)-Methyl (2*S*,3*R*,3a*R*,9b*R*)-5-benzyl-3-(furan-2-yl)-4-oxo-2,3,3a,4,5,9b-hexahydro-*1H*-pyrrolo[3,2-*c*]quinoline-2-carboxylate

PQ **B16** (55 mg, 0.14 mmol, 63% Yield, >20:1 d.r.) was synthesized according to the general procedure 3; column chromatography eluting with cyclohexane/EA 5:1 to 2:1.

^{1}H NMR (500 MHz, CDCl$_3$) δ 7.43 (d, *J* = 7.4 Hz, 1H), 7.31 – 7.26 (m, 3H), 7.23-7.18 (m, 4H), 7.11 (t, *J* = 7.5 Hz, 1H), 6.99 (d, *J* = 8.2 Hz, 1H), 6.35 – 6.27 (m, 1H), 6.20 (d, *J* = 3.3 Hz, 1H), 5.28 (d, *J* = 16.2 Hz, 1H), 5.02 (d, *J* = 16.3 Hz, 1H), 4.41 (d, *J* = 10.1 Hz, 1H), 4.29 – 4.11 (m, 2H), 3.43 (s, 3H), 3.06 (dd, *J* = 13.6, 11.0 Hz, 1H).

^{13}C NMR (126 MHz, CDCl$_3$) δ 172.2, 169.4, 152.0, 141.9, 139.5, 137.0, 129.4, 128.9 (2C), 128.4, 127.4, 126.7 (2C), 123.8, 123.3, 116.4, 110.8, 107.4, 65.2, 60.2, 52.8, 52.6, 46.3, 43.2.

HRMS(ESI): $[M+H]^+$ calcd. $C_{24}H_{23}N_2O_4$ m/z 403.1652, found 403.1651.

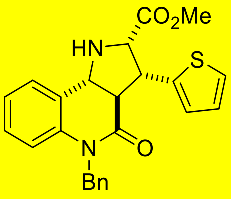

B17

(±)-Methyl (2*S*,3*R*,3a*R*,9b*R*)-5-benzyl-4-oxo-3-(thiophen-2-yl)-2,3,3a,4,5,9b-hexahydro-*1H*-pyrrolo[3,2-*c*]quinoline-2-carboxylate (B17)

PQ **B17** (68 mg, 0.16 mmol, 74% Yield, >20:1 d.r.) was synthesized according to the general procedure 3; column chromatography eluting with cyclohexane/EA 5:1 to 2:1.

^{1}H NMR (400 MHz, CDCl$_3$) δ 7.44 (d, *J* = 7.4 Hz, 1H), 7.31 – 7.26 (m, 2H), 7.25 – 7.09 (m, 6H), 7.01 (d, *J* = 8.2 Hz, 1H), 6.93 (dd, *J* = 5.1, 3.5 Hz, 1H), 6.89 – 6.85 (m, 1H), 5.28 (d, *J* = 16.1 Hz, 1H), 5.02 (d, *J* = 16.2 Hz, 1H), 4.47 (d, *J* = 10.2 Hz, 1H), 4.35 (t, *J* = 10.5 Hz, 1H), 4.19 (dt, *J* = 13.6, 1.1 Hz, 1H), 3.32 (s, 3H), 3.01 (dd, *J* = 13.5, 10.9 Hz, 1H).

^{13}C NMR (101 MHz, CDCl$_3$) δ 172.2, 169.2, 141.4, 139.5, 137.0, 129.4, 128.9 (2C), 128.4, 127.4, 127.1, 126.7 (2C), 125.3, 124.1, 123.8, 123.3, 116.4, 67.0, 60.2, 56.0, 52.2, 46.3, 44.7.

HRMS(ESI): $[M+H]^+$ calcd. $C_{24}H_{23}N_2O_3S$ m/z 419.1424, found 419.1423.

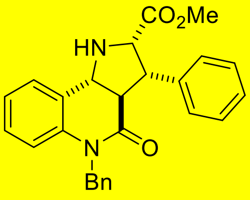

B18

(±)-Methyl (2*S*,3*S*,3a*R*,9b*R*)-5-benzyl-4-oxo-3-phenyl-2,3,3a,4,5,9b-hexahydro-*1H*-pyrrolo[3,2-*c*]quinoline-2-carboxylate (B18)

PQ **B18** (71 mg, 0.18 mmol, 78% Yield, >20:1 d.r.) was synthesized according to the general procedure 3; column chromatography eluting with cyclohexane/EA 5:1 to 2:1.

^{1}H NMR (400 MHz, $CDCl_3$) δ 7.45 (dt, *J* = 7.4, 1.3 Hz, 1H), 7.30 – 7.25 (m, 4H), 7.24 – 7.16 (m, 7H), 7.12 (td, *J* = 7.4, 1.1 Hz, 1H), 7.00 (dd, *J* = 8.2, 1.1 Hz, 1H), 5.25 (d, *J* = 16.2 Hz, 1H), 5.03 (d, *J* = 16.1 Hz, 1H), 4.50 (d, *J* = 10.4 Hz, 1H), 4.23 (d, *J* = 13.5 Hz, 1H), 4.08 (t, *J* = 10.7 Hz, 1H), 3.13 (s, 3H), 3.07 (dd, *J* = 13.5, 11.1 Hz, 1H).

^{13}C NMR (101 MHz, $CDCl_3$) δ 172.5, 169.7, 139.6, 138.7, 137.1, 129.7, 128.9 (2C), 128.5 (2C), 128.3, 128.1 (2C), 127.3, 127.3, 126.7 (2C), 123.7, 123.3, 116.4, 67.0, 60.3, 54.4, 51.8, 49.8, 46.3.

HRMS(ESI): $[M+H]^+$ calcd. $C_{26}H_{25}N_2O_3$ m/z 413.1860, found 413.1854.

Synthesis of pyrroquinolines C

General Procedure 4

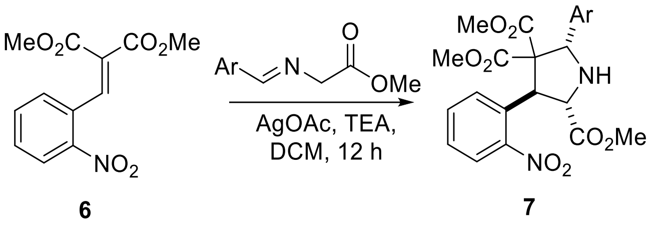

Ester **6**[8] (0.15 mmol, 1.0 equiv.) and the desired iminoester[9] (0.23 mmol, 1.5 equiv.) were dissolved in dry DCM (1.0 mL). Then silver acetate (2.5 mg, 0.015 mmol, 0.1 equiv.) was added to the mixture followed by TEA (21 µL, 0.15 mmol, 1.0 equiv.). The reaction was stirred until full conversion of the starting material was observed by TLC. The solvent was removed under reduced pressure and the product was purified by column chromatography using cyclohexane/EA mixtures as a single diastereoisomer.

General Procedure 5

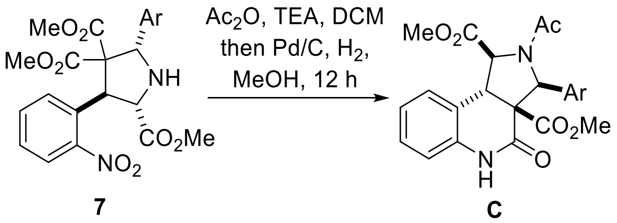

Pyrrolidine **7** (30 mg, 1.0 equiv.) was dissolved in DCM (1.0 mL). Then the acetic anhydride (3.0 equiv.) was added followed by TEA (4.0 equiv.) and the mixture was stirred overnight. Then the reaction was diluted with EA (20 mL) and washed with sat. aq. $NaHCO_3$ solution (10 mL) and brine (10 mL) sequentially. The organic phase was dried over Na_2SO_4 and filtered. The solvent was removed under reduced pressure. The residue was dissolved in MeOH (2.0 mL) and 10% Pd/C (30 mg) were added. The reaction vessel was flushed three times with H_2. The reaction was stirred under a H_2 atmosphere until full conversion of the starting material was observed. The reaction was filtered through a short pad of Celite and the solvent was then removed under reduced pressure. PQs C were purified by column chromatography using cyclohexane/acetone mixtures as a single diastereoisomer.

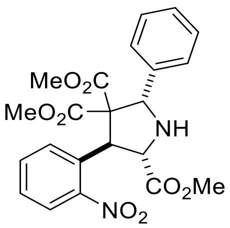

7a

(±)-Trimethyl (2*S*,3*S*,5*S*-3-(2-nitrophenyl)-5-phenylpyrrolidine-2,4,4-tricarboxylate (7a)

Pyrrolidine **7a** (68 mg, 0.1 mmol, quant. Yield) was synthesized according to the general procedure 4; column chromatography eluting with cyclohexane/EA 5:1 to 3:1.

^{1}H NMR (500 MHz, CDCl$_3$) δ 7.78 (dd, *J* = 8.0, 1.1 Hz, 1H), 7.66 – 7.56 (m, 2H), 7.49 – 7.40 (m, 3H), 7.37 – 7.26 (m, 3H), 5.33 (s, 1H), 5.20 (d, *J* = 6.1 Hz, 1H), 4.20 (d, *J* = 6.3 Hz, 1H), 3.82 (s, 3H), 3.20 (s, 3H), 3.13 (s, 3H).

^{13}C NMR (126 MHz, $CDCl_3$) δ 172.4, 169.2, 168.9, 151.0, 137.0, 133.4, 132.7, 129.5, 128.6, 128.5, 128.5 (2C), 127.4 (2C), 124.5, 71.2, 68.8, 67.4, 52.9, 52.6, 52.3, 49.3.

HRMS(ESI): $[M+H]^+$ calcd. $C_{22}H_{23}N_2O_8$ m/z 443.1449, found 443.1440.

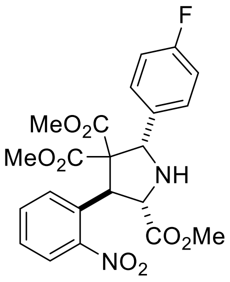

7b

(±)-Trimethyl (*2S,3S,5S*)-5-(4-fluorophenyl)-3-(2-nitrophenyl)pyrrolidine-2,4,4-tricarboxylate (7b)

Pyrrolidine **7b** (68 mg, 0.15 mmol, Yield 98%) was synthesized according to the general procedure 4; column chromatography eluting with cyclohexane/EA 5:1 to 3:1.

^1H NMR (400 MHz, $CDCl_3$) δ 7.78 (dd, J = 8.1, 1.3 Hz, 1H), 7.65 – 7.55 (m, 2H), 7.52 – 7.37 (m, 3H), 7.08 – 6.96 (m, 2H), 5.30 (s, 1H), 5.18 (d, J = 6.4 Hz, 1H), 4.16 (d, J = 6.4 Hz, 1H), 3.80 (s, 3H), 3.19 (s, 3H+3H).

^{13}C NMR (101 MHz, $CDCl_3$) δ 172.4, 169.1, 169.0, 162.8 (d, JCF = 247.1 Hz), 151.0, 133.4, 133.2 (d, JCF = 3.2 Hz), 132.7, 129.4, 129.2 (d, JCF = 8.1 Hz, 2C), 128.6, 124.5, 115.3 (d, JCF = 21.5 Hz, 2C), 71.0, 68.1, 67.3, 52.9, 52.6, 52.3, 49.2.

^{19}F NMR (470 MHz, $CDCl_3$) δ -113.6 (ddd, J = 13.8, 8.8, 5.3 Hz, 1F).

HRMS(ESI): $[M+H]^+$ calcd. $C_{22}H_{22}N_2O_8F$ m/z 461.1355, found 461.1345.

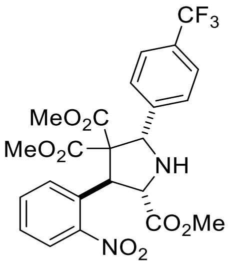

7c

(±)-Trimethyl (*2S,3S,5S*)-3-(2-nitrophenyl)-5-(4-(trifluoromethyl)phenyl)pyrrolidine-2,4,4-tricarboxylate (7c)

Pyrrolidine **7c** (75 mg, 0.15 mmol, Yield 98%) was synthesized according to the general procedure 4; column chromatography eluting with cyclohexane/EA 5:1 to 3:1.

^1H NMR (400 MHz, $CDCl_3$) δ 7.79 (dd, J = 8.2, 1.4 Hz, 1H), 7.68 – 7.52 (m, 6H), 7.45 (ddd, J = 8.5, 7.2, 1.6 Hz, 1H), 5.38 (s, 1H), 5.22 (d, J = 6.3 Hz, 1H), 4.19 (d, J = 6.3 Hz, 1H), 3.82 (s, 3H), 3.20 (s, 3H), 3.14 (s, 3H).

^{13}C NMR (101 MHz, $CDCl_3$) δ 172.4, 168.9, 168.8, 151.0, 141.6, 133.3, 132.8, 130.7 (q, JCF = 32.4 Hz), 129.4, 128.7, 128.0 (2C), 125.3 (q, JCF = 3.7 Hz, 2C), 124.6, 124.1 (q, JCF = 272.7 Hz), 71.1, 68.1, 67.4, 52.9, 52.6, 52.4, 49.1.

^{19}F NMR (470 MHz, $CDCl_3$) δ -62.7 (s, 3F).

HRMS(ESI): $[M+H]^+$ calcd. $C_{23}H_{22}N_2O_8F_3$ m/z 511.1323, found 511.1299.

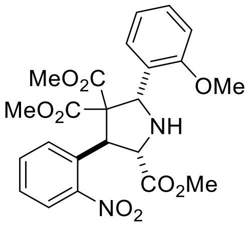

7d

(±)-Trimethyl (*2S,3S,5S*)-5-(2-methoxyphenyl)-3-(2-nitrophenyl)pyrrolidine-2,4,4-tricarboxylate (7d)

Pyrrolidine **7d** (48 mg, 0.10 mmol, Yield 68%) was synthesized according to the general procedure 4; column chromatography eluting with cyclohexane/EA 5:1 to 2:1.

^{1}H NMR (400 MHz, CDCl$_3$) δ 7.76 (dd, *J* = 8.1, 1.3 Hz, 1H), 7.66 – 7.60 (m, 2H), 7.48 – 7.40 (m, 2H), 7.30-7.26 (m, 1H), 6.94 (td, *J* = 7.5, 1.1 Hz, 1H), 6.85 (dd, *J* = 8.3, 1.1 Hz, 1H), 5.41 (s, 1H), 5.31 (d, *J* = 9.2 Hz, 1H), 4.28 (d, *J* = 9.1 Hz, 1H), 3.83 (s, 3H), 3.73 (s, 3H), 3.24 (s, 3H), 3.22 (s, 3H).

^{13}C NMR (101 MHz, CDCl$_3$) δ 171.5, 169.0, 157.8, 151.8, 131.9, 131.7, 131.4, 129.8, 129.2, 128.4, 124.7, 120.9, 110.5, 70.9, 68.5, 66.1, 55.1, 52.7, 52.5, 52.3, 49.5.

MS(ESI): $[M+H]^+$ calcd. $C_{23}H_{25}N_2O_9$ m/z 473.2, found 473.2.

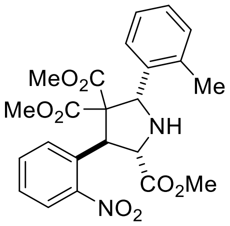

7e

(±)-Trimethyl (*2S,3S,5S*)-3-(2-nitrophenyl)-5-(*o*-tolyl)pyrrolidine-2,4,4-tricarboxylate (7e)

Pyrrolidine **7e** (60 mg, 0.13 mmol, Yield 88%) was synthesized according to the general procedure 4; column chromatography eluting with cyclohexane/EA 5:1 to 3:1.

^{1}H NMR (400 MHz, CDCl$_3$) δ 7.74 (dd, *J* = 8.1, 1.4 Hz, 1H), 7.57 (dd, *J* = 7.6, 1.4 Hz, 1H), 7.49 (dd, *J* = 8.0, 1.4 Hz, 1H), 7.45 – 7.36 (m, 2H), 7.21 (td, *J* = 7.3, 2.3 Hz, 1H), 7.18 – 7.11 (m, 2H), 5.61 (s, 1H), 5.20 (d, *J* = 9.0 Hz, 1H), 4.26 (d, *J* = 9.0 Hz, 1H), 3.74 (s, 3H), 3.27 (s, 3H), 3.09 (s, 3H), 2.45 (s, 3H).

^{13}C NMR (101 MHz, CDCl$_3$) δ 172.0, 169.9, 169.1, 151.7, 137.5, 137.3, 131.9, 131.3, 130.7, 129.2, 128.5, 128.2, 126.5, 126.2, 124.7, 71.9, 65.7, 64.1, 52.8, 52.5, 52.4, 49.3, 20.2.

HRMS(ESI): $[M+H]^+$ calcd. $C_{23}H_{25}N_2O_8$ m/z 457.1605, found 457.1596.

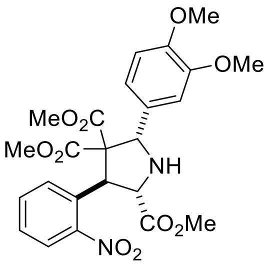

7f

(±)-Trimethyl (*2S,3S,5S*)-5-(3,4-dimethoxyphenyl)-3-(2-nitrophenyl)pyrrolidine-2,4,4-tricarboxylate (7f)

Pyrrolidine **7f** (61 mg, 0.12 mmol, Yield 81%) was synthesized according to the general procedure 4; column chromatography eluting with cyclohexane/EA 5:1 to 1:1.

^{1}H NMR (500 MHz, CDCl$_3$) δ 7.78 (d, *J* = 8.1 Hz, 1H), 7.67-7.54 (m, 2H), 7.43 (t, *J* = 7.3 Hz, 1H), 7.08 (t, *J* = 1.5 Hz, 1H), 6.98 (dt, *J* = 8.3, 1.6 Hz, 1H), 6.87 – 6.78 (m, 1H), 5.26 (s, 1H), 5.23 – 5.17 (m, 1H), 4.20 (d, *J* = 6.4 Hz, 1H), 3.89 (s, 3H), 3.85 (s, 3H), 3.80 (s, 3H), 3.22 (s, 3H), 3.20 (s, 3H).

^{13}C NMR (126 MHz, CDCl$_3$) δ 172.5, 169.2, 169.0, 151.0, 149.0, 148.8, 133.1, 132.7, 129.6, 129.4, 128.6, 124.5, 119.5, 110.8, 110.8, 70.8, 68.4, 66.9, 56.0, 56.0, 53.0, 52.8, 52.3, 48.7.

HRMS(ESI): [M+H]$^+$ calcd. $C_{24}H_{27}N_2O_{10}$ m/z 503.1660, found 503.1651.

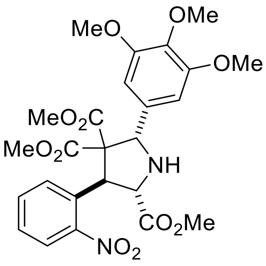

7g

(±)-Trimethyl (*2S,3S,5S*)-3-(2-nitrophenyl)-5-(3,4,5-trimethoxyphenyl)pyrrolidine-2,4,4-tricarboxylate (7g)

Pyrrolidine **7g** (68 mg, 0.13 mmol, Yield 85%) was synthesized according to the general procedure 4; column chromatography eluting with cyclohexane/EA 5:1 to 1:1.

^{1}H NMR (700 MHz, CDCl$_3$) δ 7.78 (d, *J* = 7.9 Hz, 1H), 7.64 – 7.60 (m, 2H), 7.43 (dt, *J* = 8.4, 4.3 Hz, 1H), 6.75 (s, 2H), 5.27 (d, *J* = 6.4 Hz, 1H), 5.25 (s, 1H), 4.23 (d, *J* = 6.4 Hz, 1H), 3.87 (s, 6H), 3.82 (s, 3H), 3.81 (s, 3H), 3.25 (s, 3H), 3.22 (s, 3H).

^{13}C NMR (176 MHz, CDCl$_3$) δ 172.5, 169.1, 168.9, 153.2 (2C), 151.1, 138.0, 133.1, 132.8, 132.6, 129.5, 128.7, 124.6, 104.7 (2C), 70.8, 68.7, 66.8, 61.0, 56.3 (2C), 53.0, 52.8, 52.4, 48.4.

HRMS(ESI): [M+H]$^+$ calcd. $C_{25}H_{29}N_2O_{11}$ m/z 533.1766, found 533.1755.

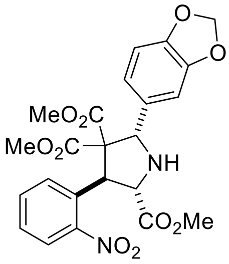

7h

(±)-Trimethyl (*2S,3S,5S*)-5-(benzo[*d*][1,3]dioxol-5-yl)-3-(2-nitrophenyl)pyrrolidine-2,4,4-tricarboxylate (7h)

Pyrrolidine **7h** (70 mg, 0.14 mmol, Yield 96%) was synthesized according to the general procedure 4; column chromatography eluting with cyclohexane/EA 5:1 to 1:1.

^{1}H NMR (500 MHz, CDCl$_3$) δ 7.78 (dd, *J* = 8.1, 1.3 Hz, 1H), 7.64 – 7.55 (m, 2H), 7.43 (ddd, *J* = 8.4, 7.1, 1.6 Hz, 1H), 6.99 (d, *J* = 1.8 Hz, 1H), 6.94 (dd, *J* = 8.1, 1.9 Hz, 1H), 6.77 (d, *J* = 8.1 Hz, 1H), 6.00 – 5.90 (m, 2H), 5.23 (s, 1H), 5.18 (d, *J* = 6.2 Hz, 1H), 4.16 (d, *J* = 6.1 Hz, 1H), 3.81 (s, 3H), 3.27 (s, 3H), 3.19 (s, 3H).

^{13}C NMR (126 MHz, CDCl$_3$) δ 172.4, 169.2, 168.9, 150.9, 147.7, 147.7, 133.4, 132.7, 130.8, 129.5, 128.6, 124.5, 120.5, 108.4, 108.2, 101.3, 70.8, 68.5, 67.1, 52.9, 52.8, 52.3, 49.1.

HRMS(ESI): $[M+H]^+$ calcd. $C_{23}H_{23}N_2O_{10}$ m/z 487.1347, found 487.1340.

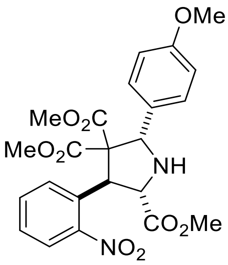

7i

(±)-Trimethyl (*2S,3S,5S*)-5-(4-methoxyphenyl)-3-(2-nitrophenyl)pyrrolidine-2,4,4-tricarboxylate (7i)

Pyrrolidine **7i** (43 mg, 0.09 mmol, Yield 60%) was synthesized according to the general procedure 4; column chromatography eluting with cyclohexane/EA 5:1 to 1:1.

^{1}H NMR (600 MHz, CDCl$_3$) δ 7.78 (dd, *J* = 8.1, 1.4 Hz, 1H), 7.64 – 7.56 (m, 2H), 7.43 (ddd, *J* = 8.4, 7.2, 1.5 Hz, 1H), 7.41 – 7.37 (m, 2H), 6.90 – 6.83 (m, 2H), 5.27 (s, 1H), 5.17 (d, *J* = 6.3 Hz, 1H), 4.16 (d, *J* = 6.3 Hz, 1H), 3.81 (s, 3H), 3.79 (s, 3H), 3.20 (s, 3H+3H).

^{13}C NMR (151 MHz, CDCl$_3$) δ 172.5, 169.4, 169.1, 159.7, 151.0, 133.6, 132.7, 129.5, 129.2, 128.6 (2C), 128.5, 124.5, 113.8 (2C), 71.1, 68.5, 67.4, 55.4, 52.9, 52.7, 52.2, 49.3.

HRMS(ESI): $[M+H]^+$ calcd. $C_{23}H_{25}N_2O_9$ m/z 473.1555, found 473.1549.

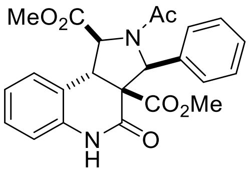

C1

(±)-Dimethyl (1*S*,3*S*,3a*S*,9b*S*)-2-acetyl-4-oxo-3-phenyl-1,2,3,4,5,9b-hexahydro-3a*H*-pyrrolo[3,4-*c*]quinoline-1,3a-dicarboxylate (C1)

PQ **C1** (60 mg, 0.14 mmol, 68% Yield) was synthesized according to the general procedure 5; column chromatography eluting with cyclohexane/EA 5:1 to 1:1 then cyclohexane/acetone 1:1.

^{1}H NMR (500 MHz, CDCl$_3$) δ 8.65 (s, 1H), 7.85 – 7.77 (m, 2H), 7.46 – 7.39 (m, 2H), 7.37 – 7.33 (m, 1H), 7.30 – 7.25 (m, 1H), 7.12 (d, *J* = 7.6 Hz, 1H), 7.05 (td, *J* = 7.5, 1.1 Hz, 1H), 6.88 (d, *J* = 7.9 Hz, 1H), 6.09 (s, 1H), 4.32 (d, *J* = 11.3 Hz, 1H), 4.18 (d, *J* = 11.4 Hz, 1H), 3.82 (s, 3H), 3.30 (s, 3H), 1.90 (s, 3H).

^{13}C NMR (126 MHz, CDCl$_3$) δ 171.6, 170.7, 165.0, 164.6, 137.2, 135.0, 129.6, 129.4, 129.0 (2C), 128.9, 127.7 (2C), 124.3, 119.2, 116.3, 66.3, 64.1, 63.2, 53.2, 52.7, 44.9, 22.1.

HRMS(ESI): $[M+H]^+$ calcd. $C_{23}H_{23}N_2O_6$ m/z 423.1551, found 423.1550.

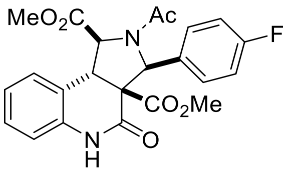

C2

(±)-Dimethyl (1*S*,3*S*,3a*S*,9b*S*)-2-acetyl-3-(4-fluorophenyl)-4-oxo-1,2,3,4,5,9b-hexahydro-3a*H*-pyrrolo[3,4-*c*]quinoline-1,3a-dicarboxylate (C2)

PQ **C2** (15 mg, 0.03 mmol, 53% Yield) was synthesized according to the general procedure 5; column chromatography eluting with cyclohexane/EA 5:1 to 1:1 then cyclohexane/acetone 1:1.

^{1}H NMR (500 MHz, CDCl$_3$) δ 8.60 (s, 1H), 7.92 – 7.76 (m, 2H), 7.30 – 7.26 (m, 1H), 7.14-7.09 (m, 3H), 7.06 (td, *J* = 7.5, 1.1 Hz, 1H), 6.87 (d, *J* = 8.0 Hz, 1H), 6.07 (s, 1H), 4.27 (d, *J* = 11.3 Hz, 1H), 4.15 (d, *J* = 11.3 Hz, 1H), 3.81 (s, 3H), 3.33 (s, 3H), 1.89 (s, 3H).

^{13}C NMR (126 MHz, CDCl$_3$) δ 171.7, 170.6, 164.9, 164.3, 163.0 (d, *J*CF = 248.2 Hz), 134.9, 133.1 (d, *J*CF = 3.8 Hz), 129.6, 129.6 (d, *J*CF = 7.6 Hz, 2C), 129.4, 124.3, 119.0, 116.3, 116.0 (d, *J*CF = 22.7 Hz, 2C), 65.6, 64.1, 63.2, 53.3, 52.7, 44.8, 22.1.

^{19}F NMR (470 MHz, CDCl$_3$) δ -112.7 (ddd, *J* = 13.6, 8.5, 5.2 Hz, 1F)

HRMS(ESI): $[M+H]^+$ calcd. $C_{23}H_{22}N_2O_6F$ m/z 441.1456, found 441.1454.

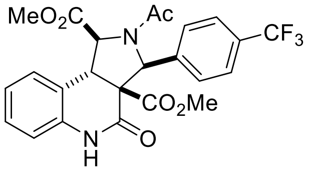

C3

(±)-Dimethyl (1*S*,3*S*,3a*S*,9b*S*)-2-acetyl-4-oxo-3-(4-(trifluoromethyl)phenyl)-1,2,3,4,5,9b-hexahydro-3a*H*-pyrrolo[3,4-*c*]quinoline-1,3a-dicarboxylate (C3)

PQ **C3** (20 mg, 0.04 mmol, 70% Yield) was synthesized according to the general procedure 5; column chromatography eluting with cyclohexane/EA 5:1 to 1:1 then cyclohexane/acetone 1:1.

^{1}H NMR (500 MHz, CDCl$_3$) δ 8.61 (s, 1H), 7.99 (d, *J* = 8.1 Hz, 2H), 7.70 (d, *J* = 8.2 Hz, 2H), 7.29 (td, *J* = 7.6, 1.6 Hz, 1H), 7.13-7.04 (m, 2H), 6.88 (d, *J* = 7.9 Hz, 1H), 6.15 (s, 1H), 4.26 (d, *J* = 11.4 Hz, 1H), 4.18 (d, *J* = 11.3 Hz, 1H), 3.82 (s, 3H), 3.32 (s, 3H), 1.88 (s, 3H).

^{13}C NMR (126 MHz, CDCl$_3$) δ 171.6, 170.5, 164.8, 164.1, 141.4, 134.8, 131.2 (q, *J*CF = 32.8 Hz), 129.7, 129.4, 128.2 (2C), 126.0 (q, *J*CF = 3.8 Hz, 2C), 124.4, 124.0 (q, *J*CF = 272.2 Hz), 118.8, 116.3, 65.7, 64.2, 63.2, 53.3, 52.8, 45.0, 22.1.

^{19}F NMR (470 MHz, CDCl$_3$) δ -62.7 (s, 3F).

HRMS(ESI): $[M+H]^+$ calcd. $C_{24}H_{22}N_2O_6F_3$ m/z 491.1425, found 491.1420.

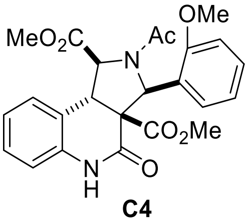

C4

(±)-Dimethyl (1*S*,3*S*,3a*S*,9b*S*)-2-acetyl-3-(2-methoxyphenyl)-4-oxo-1,2,3,4,5,9b-hexahydro-3a*H*-pyrrolo[3,4-*c*]quinoline-1,3a-dicarboxylate (C4)

PQ **C4** (14 mg, 0.03 mmol, 45% Yield) was synthesized according to the general procedure 5; column chromatography eluting with cyclohexane/EA 5:1 to 1:1 then cyclohexane/acetone 1:1.

^{1}H NMR (700 MHz, CDCl$_3$) δ 8.34 (dd, *J* = 7.7, 1.6 Hz, 1H), 8.25 (s, 1H), 7.33 (td, *J* = 7.8, 1.7 Hz, 1H), 7.27 (dd, *J* = 7.6, 1.6 Hz, 1H), 7.11 (t, *J* = 7.6 Hz, 1H), 7.06 (d, *J* = 7.5 Hz, 1H), 7.02 (t, *J* = 7.5 Hz, 1H), 6.87-6.84 (m, 2H), 6.53 (s, 1H), 4.19 – 4.11 (m, 2H), 3.83 (s, 3H), 3.79 (s, 3H), 3.31 (s, 3H), 1.88 (s, 3H).

^{13}C NMR (176 MHz, CDCl$_3$) δ 171.5, 170.7, 166.0, 165.4, 156.4, 135.4, 129.8, 129.6, 129.4, 129.2, 125.7, 124.0, 121.4, 119.0, 116.0, 109.6, 62.9, 62.8, 61.0, 55.4, 52.9, 52.6, 46.7, 21.7.

HRMS(ESI): $[M+H]^+$ calcd. $C_{24}H_{25}N_2O_7$ m/z 453.1656, found 453.1653.

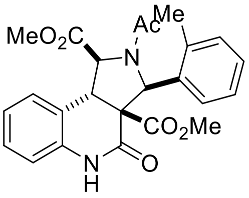

C5

(±)-Dimethyl (1*S*,3*S*,3a*S*,9b*S*)-2-acetyl-4-oxo-3-(*o*-tolyl)-1,2,3,4,5,9b-hexahydro-3a*H*-pyrrolo[3,4-*c*]quinoline-1,3a-dicarboxylate (C5)

PQ **C5** (20 mg, 0.05 mmol, 69% Yield) was synthesized according to the general procedure 5; column chromatography eluting with cyclohexane/EA 5:1 to 1:1 then cyclohexane/acetone 1:1.

^{1}H NMR (500 MHz, CDCl$_3$) δ 8.27 (d, *J* = 7.9 Hz, 1H), 8.13 (s, 1H), 7.39 – 7.33 (m, 1H), 7.29 (td, *J* = 7.7, 1.5 Hz, 1H), 7.24 (td, *J* = 7.4, 1.3 Hz, 1H), 7.19 – 7.11 (m, 2H), 7.06 (td, *J* = 7.5, 1.1 Hz, 1H), 6.84 (d, *J* = 8.0 Hz, 1H), 6.39 (s, 1H), 4.46 (d, *J* = 11.4 Hz, 1H), 4.14 (d, *J* = 11.4 Hz, 1H), 3.82 (s, 3H), 3.20 (s, 3H), 2.50 (s, 3H), 1.82 (s, 3H).

^{13}C NMR (126 MHz, CDCl$_3$) δ 171.6, 170.5, 165.4, 164.8, 135.6, 135.0, 130.7, 129.7, 129.5, 128.8, 128.4, 127.3, 124.3, 118.8, 116.1, 63.9, 63.3, 62.7, 53.0, 52.7, 45.7, 21.8, 19.4.

HRMS(ESI): $[M+H]^+$ calcd. $C_{24}H_{25}N_2O_6$ m/z 437.1707, found 437.1705.

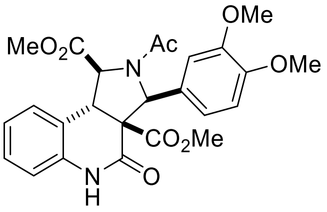

C6

(±)-Dimethyl (1*S*,3*S*,3a*S*,9b*S*)-2-acetyl-3-(3,4-dimethoxyphenyl)-4-oxo-1,2,3,4,5,9b-hexahydro-3a*H*-pyrrolo[3,4-*c*]quinoline-1,3a-dicarboxylate (C6)

PQ **C6** (25 mg, 0.05 mmol, 86% Yield) was synthesized according to the general procedure 5; column chromatography eluting with cyclohexane/EA 5:1 to 1:1 then cyclohexane/acetone 1:1.

^{1}H NMR (500 MHz, CDCl$_3$) δ 8.14 (s, 1H), 7.54 (s, 1H), 7.31 – 7.24 (m, 2H), 7.13 (d, *J* = 7.7 Hz, 1H), 7.06 (t, *J* = 7.5 Hz, 1H), 6.88 (d, *J* = 8.3 Hz, 1H), 6.84 (d, *J* = 8.0 Hz, 1H), 6.02 (s, 1H), 4.29 (d, *J* = 11.3 Hz, 1H), 4.14 (d, *J* = 11.3 Hz, 1H), 3.97 (s, 3H), 3.89 (s, 3H), 3.80 (s, 3H), 3.37 (s, 3H), 1.90 (s, 3H).

^{13}C NMR (126 MHz, CDCl$_3$) δ 171.7, 170.6, 164.9, 164.5, 149.5, 149.2, 134.8, 129.6, 129.6, 129.5, 124.3, 119.9, 119.3, 116.1, 111.0, 110.6, 66.0, 64.1, 63.2, 56.4, 56.0, 53.3, 52.7, 45.0, 22.0.

HRMS(ESI): $[M+H]^+$ calcd. $C_{25}H_{27}N_2O_8$ m/z 483.1762, found 483.1757.

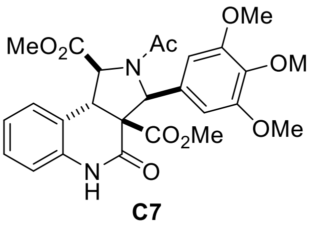

C7

(±)-Dimethyl (1*S*,3*S*,3a*S*,9b*S*)-2-acetyl-4-oxo-3-(3,4,5-trimethoxyphenyl)-1,2,3,4,5,9b-hexahydro-3a*H*-pyrrolo[3,4-*c*]quinoline-1,3a-dicarboxylate (C7)

PQ **C7** (27 mg, 0.05 mmol, 95% Yield) was synthesized according to the general procedure 5; column chromatography eluting with cyclohexane/EA 5:1 to 1:1 then cyclohexane/acetone 1:1.

^{1}H NMR (700 MHz, $CDCl_3$) δ 7.77 (s, 1H), 7.29 (td, *J* = 7.7, 1.5 Hz, 1H), 7.15 – 7.10 (m, 3H), 7.07 (td, *J* = 7.5, 1.1 Hz, 1H), 6.81 (d, *J* = 7.9 Hz, 1H), 6.00 (s, 1H), 4.26 (d, *J* = 11.3 Hz, 1H), 4.13 (d, *J* = 11.3 Hz, 1H), 3.93 (s, 6H), 3.86 (s, 3H), 3.79 (s, 3H), 3.38 (s, 3H), 1.92 (s, 3H).

^{13}C NMR (176 MHz, $CDCl_3$) δ 171.7, 170.6, 164.9, 164.3, 153.7, 138.0, 134.8, 132.7, 129.7, 129.5, 124.4, 119.3, 116.0, 104.6, 66.3, 64.3, 63.2, 61.0, 56.6 (2C), 53.3, 52.7, 45.1, 22.0.

HRMS(ESI): $[M+H]^+$ calcd. $C_{26}H_{29}N_2O_9$ m/z 513.1868, found 513.1863.

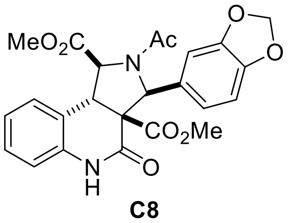

C8

(±)-Dimethyl (1*S*,3*S*,3a*S*,9b*S*)-2-acetyl-3-(benzo[*d*][1,3]dioxol-5-yl)-4-oxo-1,2,3,4,5,9b-hexahydro-3a*H*-pyrrolo[3,4-*c*]quinoline-1,3a-dicarboxylate (C8)

PQ **C8** (20 mg, 0.04 mmol, 71% Yield) was synthesized according to the general procedure 5; column chromatography eluting with cyclohexane/EA 5:1 to 1:1 then cyclohexane/acetone 1:1.

^{1}H NMR (700 MHz, $CDCl_3$) δ 8.14 (s, 1H), 7.47 (d, *J* = 1.8 Hz, 1H), 7.31 – 7.27 (m, 1H), 7.20 (dd, *J* = 8.1, 1.8 Hz, 1H), 7.12 (dd, *J* = 7.7, 1.4 Hz, 1H), 7.06 (td, *J* = 7.5, 1.1 Hz, 1H), 6.89 – 6.81 (m, 2H), 6.05 – 5.95 (m, 3H), 4.28 (d, *J* = 11.3 Hz, 1H), 4.13 (d, J = 11.3 Hz, 1H), 3.81 (s, 3H), 3.39 (s, 3H), 1.91 (s, 3H).

^{13}C NMR (176 MHz, $CDCl_3$) δ 171.5, 170.6, 164.9, 164.4, 148.5, 148.1, 134.9, 131.0, 129.6, 129.5, 124.4, 121.3, 119.3, 116.1, 108.5, 108.2, 101.5, 66.1, 64.1, 63.2, 53.3, 52.7, 44.9, 22.0.

HRMS(ESI): $[M+H]^+$ calcd. $C_{24}H_{23}N_2O_8$ m/z 467.1449, found 467.1445.

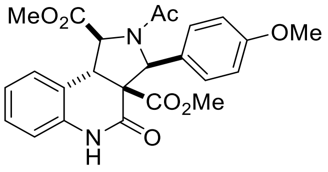

C9

(±)-Dimethyl (1*S*,3*S*,3a*S*,9b*S*)-2-acetyl-3-(4-methoxyphenyl)-4-oxo-1,2,3,4,5,9b-hexahydro-3a*H*-pyrrolo[3,4-*c*]quinoline-1,3a-dicarboxylate (C9)

PQ **C9** (9 mg, 20 μmol, 31% Yield) was synthesized according to the general procedure 5; column chromatography eluting with cyclohexane/EA 5:1 to 1:1 then cyclohexane/acetone 1:1.

^{1}H NMR (400 MHz, $CDCl_3$) δ 8.36 (s, 1H), 7.81 – 7.66 (m, 2H), 7.30 – 7.25 (m, 1H), 7.12 (dd, *J* = 7.6, 1.5 Hz, 1H), 7.05 (td, *J* = 7.5, 1.1 Hz, 1H), 6.96 – 6.91 (m, 2H), 6.85 (d, *J* = 8.0 Hz, 1H), 6.03 (s, 1H), 4.30 (d, *J* = 11.3 Hz, 1H), 4.14 (d, *J* = 11.3 Hz, 1H), 3.81 (s, 6H), 3.33 (s, 3H), 1.89 (s, 3H).

^{13}C NMR (101 MHz, $CDCl_3$) δ 171.7, 170.7, 165.0, 164.5, 160.0, 135.0, 129.5, 129.4, 129.1, 129.0 (2C), 124.3, 119.3, 116.2, 114.3 (2C), 65.9, 64.1, 63.2, 55.4, 53.2, 52.6, 44.8, 22.1.

HRMS(ESI): $[M+H]^+$ calcd. $C_{24}H_{25}N_2O_7$ m/z 453.1656, found 453.1654.

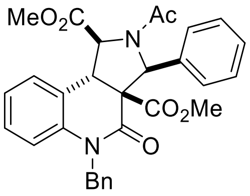

C10

(±)-Dimethyl (1*S*,3*S*,3a*S*,9b*S*)-2-acetyl-5-benzyl-4-oxo-3-phenyl-1,2,3,4,5,9b-hexahydro-3a*H*-pyrrolo[3,4-*c*]quinoline-1,3a-dicarboxylate (C10)

PQ **C1** (11 mg, 25 µmol, 1.0 equiv.) was dissolved in dry DMF (0.25 ml) and cooled in an ice bath. NaH (60% in mineral oil, 1.1 mg, 28 µmol, 1.1 equiv.) was added and the reaction was stirred for 30 minutes. Benzyl bromide (13 mg, 75 µmol, 3.0 equiv.) was added and the reaction was stirred at room temperature overnight. The reaction was quenched with sat. aq. NH_4Cl solution (20 mL) and extracted with DCM (3*20 mL). The combined organic phases were washed four times with water and dried over Na_2SO_4. The solvent was removed under reduced pressure. The product was purified by column chromatography using cyclohexane/acetone 4:1 to 2:1 affording PQ **C10** (9.6 mg, 19 µmol, 75% Yield).

^{1}H NMR (700 MHz, $CDCl_3$) δ 7.82 (d, *J* = 7.2 Hz, 2H), 7.43 (t, *J* = 7.6 Hz, 2H), 7.38 – 7.31 (m, 3H), 7.28 – 7.26 (m, 1H), 7.24-7.19 (m, 3H), 7.12 (dd, *J* = 7.5, 1.6 Hz, 1H), 7.04 (td, *J* = 7.5, 1.0 Hz, 1H), 6.94 (d, *J* = 8.3 Hz, 1H), 6.22 (s, 1H), 5.76 (d, *J* = 16.1 Hz, 1H), 4.75 (d, *J* = 16.1 Hz, 1H), 4.30 (d, *J* = 11.4 Hz, 1H), 4.19 (d, *J* = 11.3 Hz, 1H), 3.83 (s, 3H), 3.33 (s, 3H), 1.90 (s, 3H).

^{13}C NMR (176 MHz, $CDCl_3$) δ 171.6, 170.7, 165.2, 164.3, 137.9, 137.4, 136.5, 129.7, 129.6, 129.1 (2C), 129.1 (2C), 128.9, 127.6, 127.6 (2C), 126.4 (2C), 124.2, 120.4, 116.4, 66.9, 64.4, 63.0, 53.0, 52.7, 48.5, 44.6, 22.1.

HRMS(ESI): $[M+H]^+$ calcd. $C_{30}H_{29}N_2O_6$ m/z 513.2020, found 513.2015.

Synthesis of pyrroquinolines D

General Procedure 6

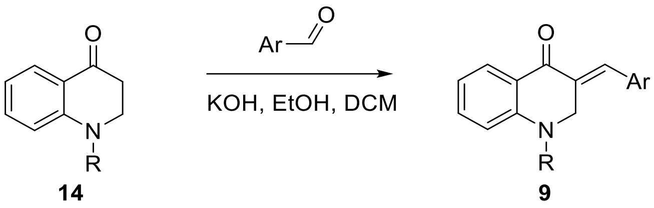

Ketone **14** (synthesized according to Ref 10)[10] (1.0 equiv.) was dissolved in a EtOH/DCM (1:2, 0.1 M) mixture followed by the addition of aldehyde (1.0 equiv.) and KOH (2.0 equiv.). The reaction was stirred until the full conversion of the starting material was observed by TLC (less than 10 min). The reaction was quenched with sat. NH_4Cl solution (30 mL) and extracted with DCM (3*50 mL). The combined organic phases were then washed with brine, dried over Na_2SO_4 and filtered. The solvent was removed under reduced pressure. The products 9 were purified by column chromatography using cyclohexane/EA.

General Procedure 7

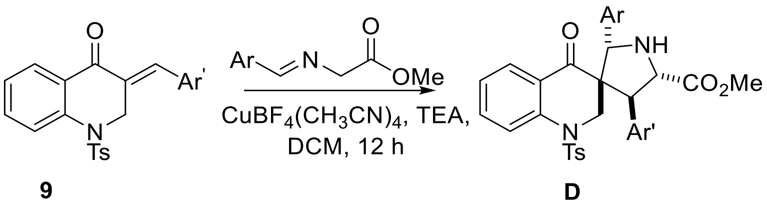

Ketone **9** (0.1 mmol, 1.0 equiv.) and the desired iminoester (0.15 mmol, 1.5 equiv.) were dissolved in dry DCM (1.0 mL). Then $Cu(CH_3CN)_4BF_4$ (3.1 mg, 0.01 mmol, 0.1 equiv.) was added to the mixture followed by TEA (14 µL, 0.1 mmol, 1.0 equiv.). The reaction was stirred until full conversion of the starting material was observed. The solvent was removed under reduced pressure and the residue used to determine the diastereomeric ratio of the products. PQs **D** were purified by column chromatography using cyclohexane/EA.

General Procedure 8

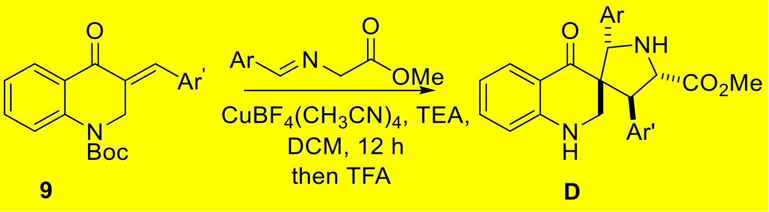

Ketone **9** (0.1 mmol, 1.0 equiv.) and the desired iminoester (0.15 mmol, 1.5 equiv.) were dissolved in dry DCM (1.0 mL). Then $Cu(CH_3CN)_4BF_4$ (3.1 mg, 0.01 mmol, 0.1 equiv.) was added to the mixture followed by TEA (14 µL, 0.1 mmol, 1.0 equiv.). After full conversion was observed, TFA (10 equiv.) was added to the reaction mixture and stirred for another 1 h. The solvent was removed under reduced pressured. The remains were three times dissolved in DCM and the solvent evaporated to remove excess TFA. Then the residue was dissolved in EA (20 mL) and washed with $NaHCO_3$ solution (10 mL) and brine (10 mL) sequentially. The organic phase was dried over Na_2SO_4 and filtered. The solvent was removed under reduced pressure and deprotected PQs **D** were purified by column chromatography using cyclohexane/EA as a single diastereoisomer.

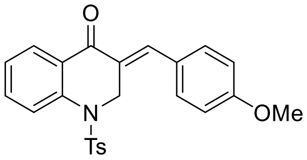

9a

(*E*)-3-(4-Methoxybenzylidene)-1-tosyl-2,3-dihydroquinolin-4(*1H*)-one (9a)

Ketone **9a** (1.4 g, 3.34 mmol, 50% Yield) was synthesized according to the general procedure 6 from **14** (2g, 6.64 mmol); column chromatography eluting with cyclohexane/EA 10:1 to 3:1.

^{1}H NMR (700 MHz, CDCl$_3$) δ 7.94 (dd, *J* = 7.8, 1.6 Hz, 1H), 7.82 (dd, *J* = 8.1, 1.1 Hz, 1H), 7.63 (ddd, *J* = 8.1, 7.3, 1.7 Hz, 1H), 7.41 – 7.35 (m, 2H), 7.34 – 7.28 (m, 2H), 7.05 – 6.99 (m, 4H), 6.99 – 6.92 (m, 2H), 5.07 (d, *J* = 1.8 Hz, 2H), 3.91 (s, 3H), 2.33 (s, 3H).

^{13}C NMR (151 MHz, CDCl$_3$) δ 182.8, 161.1, 144.3, 141.3, 138.5, 134.6, 134.1, 132.2 (2C), 129.6 (2C), 129.2, 128.3, 127.7, 127.5, 127.5 (2C), 127.2, 127.0, 114.7 (2C), 55.6, 48.2, 21.7.

HRMS(ESI): $[M+H]^+$ calcd. $C_{24}H_{22}NO_4S$ m/z 420.1264, found 420.1263.

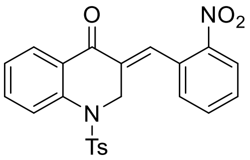

9b

(*E*)-3-(2-Nitrobenzylidene)-1-tosyl-2,3-dihydroquinolin-4(*1H*)-one (9b)

Ketone **9b** (0.63 g, 1.45 mmol, 44% Yield) was synthesized according to the general procedure 6 from **14** (1g, 3.32 mmol); column chromatography eluting with cyclohexane/EA 10:1 to 3:1.

^{1}H NMR (700 MHz, CDCl$_3$) δ 8.28 (dd, *J* = 8.3, 1.3 Hz, 1H), 7.95 (dd, *J* = 7.8, 1.7 Hz, 1H), 7.92 (s, 1H), 7.83 (dd, *J* = 8.2, 1.1 Hz, 1H), 7.75 (td, *J* = 7.5, 1.3 Hz, 1H), 7.67 – 7.62 (m, 2H), 7.38 (td, *J* = 7.6, 1.1 Hz, 1H), 7.29 – 7.27 (m, 1H), 7.15 – 7.12 (m, 2H), 7.11 – 7.08 (m, 2H), 4.90 (d, *J* = 2.0 Hz, 2H), 2.36 (s, 3H).

^{13}C NMR (176 MHz, CDCl$_3$) δ 181.8, 148.2, 145.0, 141.7, 134.7, 134.7, 134.2, 133.9, 131.6, 130.9, 130.5, 130.1, 129.9 (2C), 128.8, 128.7, 127.4 (2C), 127.3, 127.0, 125.9, 48.3, 21.8.

HRMS(ESI): $[M+H]^+$ calcd. $C_{23}H_{19}N_2O_5S$ m/z 435.1009, found 435.1008.

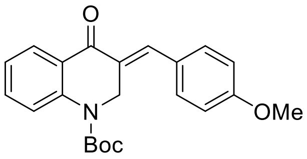

9c

***Tert*-butyl (*E*)-3-(4-methoxybenzylidene)-4-oxo-3,4-dihydroquinoline-1(*2H*)-carboxylate (9c)**

Ketone **9c** (0.42 g, 1.15 mmol, 28% Yield) was synthesized according to the general procedure 6 from **14** (1g, 4.04 mmol); column chromatography eluting with cyclohexane/EA 10:1 to 4:1.

^{1}H NMR (500 MHz, $CDCl_3$) δ 8.04 (dd, *J* = 7.8, 1.7 Hz, 1H), 7.80 (d, *J* = 1.8 Hz, 1H), 7.64 (d, *J* = 8.3 Hz, 1H), 7.50 (ddd, *J* = 8.5, 7.2, 1.7 Hz, 1H), 7.48 – 7.45 (m, 2H), 7.21 (ddd, *J* = 8.0, 7.4, 1.1 Hz, 1H), 6.99 – 6.95 (m, 2H), 5.06 (d, *J* = 1.7 Hz, 2H), 3.86 (s, 3H), 1.44 (s, 9H).

^{13}C NMR (126 MHz, $CDCl_3$) δ 184.7, 160.8, 152.8, 143.2, 137.1, 133.4, 132.1 (2C), 130.2, 128.2, 127.4, 127.2, 124.6, 124.2, 114.4 (2C), 82.1, 55.5, 45.9, 28.3 (3C).

HRMS(ESI): $[M+H]^+$ calcd. $C_{22}H_{24}NO_4$ m/z 366.1700, found 366.1700.

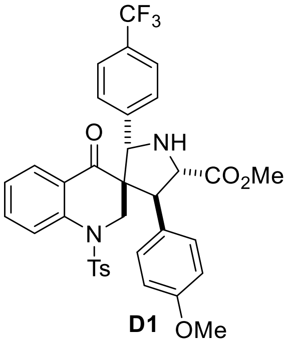

D1

(±)-Methyl (2*S*,3*R*,4*S*,5*S*)-4-(4-methoxyphenyl)-4'-oxo-1'-tosyl-2-(4-(trifluoromethyl)phenyl)-1',4'-dihydro-2'*H*-spiro[pyrrolidine-3,3'-quinoline]-5-carboxylate (D1)

PQ **D1** (65 mg, 0.1 mmol, 98% Yield, d.r. 12:1) was synthesized according to the general procedure 7; column chromatography eluting with cyclohexane/EA 5:1 to 2:1.

^{1}H NMR (500 MHz, $CDCl_3$) δ 7.64 – 7.58 (m, 2H), 7.52 (dd, *J* = 7.9, 1.7 Hz, 1H), 7.39 (m, 3H), 7.29 (m, 6H), 7.23 (ddd, *J* = 8.8, 7.2, 1.8 Hz, 1H), 6.90 – 6.86 (m, 2H), 6.83 (dd, *J* = 8.0, 7.1 Hz, 1H), 5.05 (s, 1H), 4.66 (d, *J* = 10.0 Hz, 1H), 4.53 – 4.47 (m, 2H), 3.80 (s, 3H), 3.76 (s, 3H), 3.05 (d, *J* = 12.8 Hz, 1H), 2.38 (s, 3H).

^{13}C NMR (126 MHz, $CDCl_3$) δ 192.6, 172.9, 159.2, 144.9, 142.7, 141.5, 136.0, 134.7, 130.3 (2C), 130.0 (q, *J*CF = 32.8 Hz), 130.0 (2C), 128.9, 128.7 (2C), 127.2, 126.9 (2C), 124.0 (q, *J*CF = 272.2 Hz), 124.8 (q, *J*CF = 3.8 Hz, 2C), 123.1, 122.7, 117.5, 114.3 (2C), 68.0, 63.1, 60.3, 55.4, 53.4, 52.7, 51.2, 21.7.

^{19}F NMR (470 MHz, $CDCl_3$) δ -62.7 (s, 3F).

HRMS(ESI): $[M+H]^+$ calcd. $C_{35}H_{32}N_2O_6F_3S$ m/z 665.1928, found 665.1928.

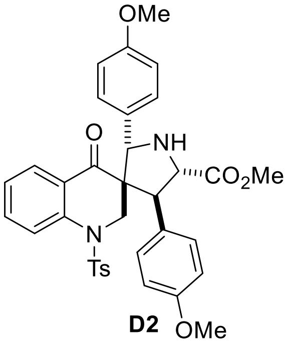

D2

(±)-Methyl (2*S*,3*R*,4*S*,5*S*)-2,4-bis(4-methoxyphenyl)-4'-oxo-1'-tosyl-1',4'-dihydro-2'*H*-spiro[pyrrolidine-3,3'-quinoline]-5-carboxylate (D2)

PQ **D2** (31 mg, 0.05 mmol, 50% Yield, d.r. >20:1) was synthesized according to the general procedure 7; column chromatography eluting with cyclohexane/EA 5:1 to 1:1.

^{1}H NMR (500 MHz, $CDCl_3$) δ 7.62 – 7.58 (m, 2H), 7.56 (dd, *J* = 7.9, 1.7 Hz, 1H), 7.42 (d, *J* = 8.7 Hz, 1H), 7.30 – 7.24 (m, 4H), 7.20 (ddd, *J* = 8.8, 7.2, 1.8 Hz, 1H), 7.17 – 7.14 (m, 2H), 6.89 – 6.84 (m, 2H), 6.82 (dd, *J* =

8.0, 7.2 Hz, 1H), 6.59 – 6.55 (m, 2H), 4.95 (s, 1H), 4.61 (d, *J* = 9.9 Hz, 1H), 4.50 – 4.41 (m, 2H), 3.80 (s, 3H), 3.74 (s, 3H), 3.65 (s, 3H), 3.01 (d, *J* = 12.7 Hz, 1H), 2.37 (s, 3H).

^{13}C NMR (126 MHz, CDCl$_3$) δ 193.3, 172.9, 159.3, 159.1, 144.7, 141.6, 136.2, 134.3, 130.2 (2C), 130.0 (2C), 129.4 (2C), 128.8, 127.7, 126.9 (2C), 122.9, 122.9, 117.5, 114.2 (2C), 113.4 (2C), 68.7, 63.1, 60.0, 55.4, 55.3, 53.7, 52.6, 52.0, 21.7.

HRMS(ESI): $[M+H]^+$ calcd. $C_{35}H_{35}N_2O_7S$ m/z 627.2160, found 627.2158.

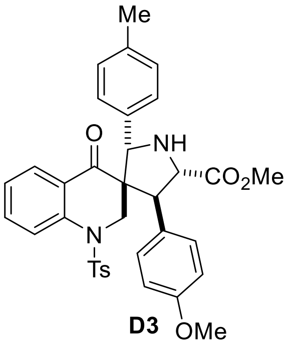

D3

(±)-Methyl (2*S*,3*R*,4*S*,5*S*)-4-(4-methoxyphenyl)-4'-oxo-2-(*p*-tolyl)-1'-tosyl-1',4'-dihydro-2'*H*-spiro[pyrrolidine-3,3'-quinoline]-5-carboxylate (D3)

PQ **D3** (52 mg, 0.09 mmol, 85% Yield, d.r. >20:1) was synthesized according to the general procedure 7; column chromatography eluting with cyclohexane/EA 5:1 to 2:1.

^{1}H NMR (700 MHz, CDCl$_3$) δ 7.62 – 7.60 (m, 2H), 7.54 (dd, *J* = 7.9, 1.7 Hz, 1H), 7.43 (d, *J* = 8.7 Hz, 1H), 7.30 – 7.27 (m, 2H), 7.26 – 7.24 (m, 2H), 7.21 (ddd, *J* = 8.7, 7.2, 1.8 Hz, 1H), 7.11 – 7.08 (m, 2H), 6.89 – 6.86 (m, 2H), 6.84 (d, *J* = 8.0 Hz, 2H), 6.81 (dd, *J* = 8.0, 7.2 Hz, 1H), 4.95 (s, 1H), 4.61 (d, *J* = 10.0 Hz, 1H), 4.50 – 4.43 (m, 2H), 3.80 (s, 3H), 3.75 (s, 3H), 3.03 (d, *J* = 12.7 Hz, 1H), 2.37 (s, 3H), 2.14 (s, 3H).

^{13}C NMR (176 MHz, CDCl$_3$) δ 193.2, 173.0, 159.2, 144.7, 141.6, 137.7, 136.2, 134.3, 130.2 (2C), 130.0 (2C), 128.9, 128.7 (2C), 128.1 (2C), 127.7, 126.9 (2C), 123.0, 122.8, 117.5, 114.2 (2C), 68.9, 63.2, 60.3, 55.4, 53.6, 52.6, 52.1, 21.7, 21.1.

HRMS(ESI): $[M+H]^+$ calcd. $C_{35}H_{35}N_2O_6S$ m/z 611.2210, found 611.2208.

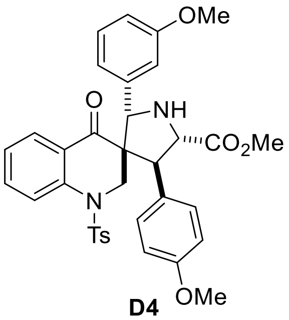

D4

(±)-Methyl (2*S*,3*R*,4*S*,5*S*)-2-(3-methoxyphenyl)-4-(4-methoxyphenyl)-4'-oxo-1'-tosyl-1',4'-dihydro-2'*H*-spiro[pyrrolidine-3,3'-quinoline]-5-carboxylate (D4)

PQ **D4** (47 mg, 0.07 mmol, 74% Yield, d.r. >20:1) was synthesized according to the general procedure 7; column chromatography eluting with cyclohexane/EA 5:1 to 1:1.

^{1}H NMR (700 MHz, CDCl$_3$) δ 7.62 (d, *J* = 8.5 Hz, 2H), 7.56 (dd, *J* = 7.9, 1.8 Hz, 1H), 7.42 (d, *J* = 8.6 Hz, 1H), 7.30 – 7.25 (m, 4H), 7.21 (ddd, *J* = 8.8, 7.2, 1.8 Hz, 1H), 6.96 (t, *J* = 8.1 Hz, 1H), 6.86 (d, *J* = 8.8 Hz, 2H), 6.84 – 6.77 (m, 3H), 6.59 – 6.53 (m, 1H), 4.96 (s, 1H), 4.64 (d, *J* = 10.2 Hz, 1H), 4.50 (d, *J* = 12.7 Hz, 1H), 4.45 (d, *J* = 10.2 Hz, 1H), 3.79 (s, 3H), 3.74 (s, 3H), 3.62 (s, 3H), 3.04 (d, *J* = 12.7 Hz, 1H), 2.37 (s, 3H).

^{13}C NMR (176 MHz, CDCl$_3$) δ 192.8, 173.1, 159.1, 159.1, 144.7, 141.6, 140.2, 136.3, 134.2, 130.2 (2C), 130.0 (2C), 129.0, 128.9, 127.6, 126.9 (2C), 123.0, 122.9, 120.2, 117.4, 115.0, 114.2 (2C), 112.8, 69.1, 63.1, 60.5, 55.4, 55.2, 53.6, 52.6, 51.8, 21.7.

HRMS(ESI): $[M+H]^+$ calcd. $C_{35}H_{35}N_2O_7S$ m/z 627.2160, found 627.2151.

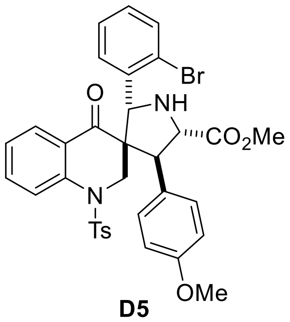

D5

(±)-Methyl (2*R*,3*R*,4*S*,5*S*)-2-(2-bromophenyl)-4-(4-methoxyphenyl)-4'-oxo-1'-tosyl-1',4'-dihydro-2'*H*-spiro[pyrrolidine-3,3'-quinoline]-5-carboxylate (D5)

PQ **D5** (67 mg, 0.1 mmol, 99% Yield, d.r. 14:1) was synthesized according to the general procedure 7; column chromatography eluting with cyclohexane/EA 5:1 to 2:1.

^{1}H NMR (500 MHz, CDCl$_3$) δ 7.82 (dd, *J* = 7.9, 1.7 Hz, 1H), 7.70 (d, *J* = 8.4 Hz, 2H), 7.64 (d, *J* = 8.5 Hz, 1H), 7.57 (dd, *J* = 7.9, 1.8 Hz, 1H), 7.34 – 7.21 (m, 7H), 6.98 (td, *J* = 7.6, 1.7 Hz, 1H), 6.91 – 6.86 (m, 1H), 6.82 (d, *J* = 8.7 Hz, 2H), 5.65 (s, 1H), 4.76 (d, *J* = 11.3 Hz, 1H), 4.60 (dd, *J* = 12.0, 6.2 Hz, 2H), 3.76 (s, 3H), 3.72 (s, 3H), 3.18 (d, *J* = 12.7 Hz, 1H), 2.38 (s, 3H).

^{13}C NMR (126 MHz, CDCl$_3$) δ 191.5, 173.6, 159.1, 144.7, 142.5, 139.8, 136.0, 134.5, 132.5, 130.9, 130.2 (2C), 130.2 (2C), 129.1, 128.6, 127.0 (2C), 126.3, 123.8, 123.7, 123.0, 118.0, 114.0 (2C), 64.8, 61.9, 55.3, 52.6, 52.1, 50.7, 21.7.

HRMS(ESI): $[M+H]^+$ calcd. $C_{34}H_{32}N_2O_6BrS$ m/z 675.1160, found 675.1161.

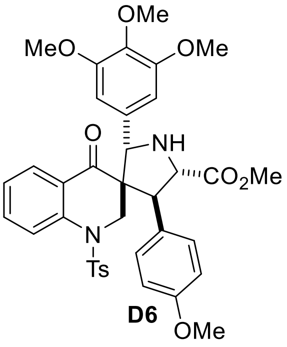

D6

(±)-Methyl (2*S*,3*R*,4*S*,5*S*)-4-(4-methoxyphenyl)-4'-oxo-1'-tosyl-2-(3,4,5-trimethoxyphenyl)-1',4'-dihydro-2'*H*-spiro[pyrrolidine-3,3'-quinoline]-5-carboxylate (D6)

PQ **D6** (42 mg, 0.06 mmol, 62% Yield, d.r. >20:1) was synthesized according to the general procedure 7; column chromatography eluting with cyclohexane/EA 5:1 to 1:1.

^{1}H NMR (700 MHz, CDCl$_3$) δ 7.63 – 7.56 (m, 3H), 7.42 (dd, *J* = 8.6, 0.9 Hz, 1H), 7.30 – 7.24 (m, 4H), 7.21 (ddd, *J* = 8.8, 7.2, 1.8 Hz, 1H), 6.88 (d, *J* = 8.8 Hz, 2H), 6.84 (ddd, *J* = 8.0, 7.2, 1.0 Hz, 1H), 6.53 (s, 2H), 4.91 (s, 1H), 4.67 (d, *J* = 10.1 Hz, 1H), 4.48 (d, *J* = 12.7 Hz, 1H), 4.43 (d, *J* = 10.1 Hz, 1H), 3.81 (s, 3H), 3.74 (s, 3H), 3.71 (s, 6H), 3.66 (s, 3H), 2.98 (d, *J* = 12.7 Hz, 1H), 2.38 (s, 3H).

^{13}C NMR (176 MHz, CDCl$_3$) δ 193.0, 173.1, 159.1, 152.6 (2C), 144.8, 141.6, 137.3, 136.1, 134.1, 130.2 (2C), 129.9 (2C), 128.8, 127.6, 126.8 (2C), 123.0, 122.9, 117.3, 114.2 (2C), 105.4 (2C), 69.5, 63.0, 60.8, 59.9, 56.0 (2C), 55.4, 53.8, 52.5, 51.4, 21.7.

HRMS(ESI): $[M+H]^+$ calcd. $C_{37}H_{39}N_2O_9S$ m/z 687.2371, found 687.2374.

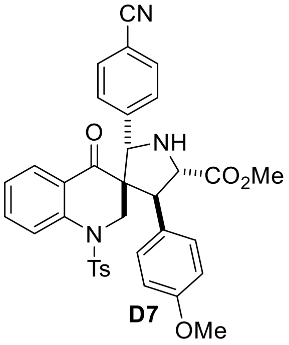

D7

(±)-Methyl (2*S*,3*R*,4*S*,5*S*)-2-(4-cyanophenyl)-4-(4-methoxyphenyl)-4'-oxo-1'-tosyl-1',4'-dihydro-2'*H*-spiro[pyrrolidine-3,3'-quinoline]-5-carboxylate (D7)

PQ **D7** (54 mg, 0.09 mmol, 86% Yield, d.r. 13:1) was synthesized according to the general procedure 7; column chromatography eluting with cyclohexane/EA 5:1 to 2:1.

^{1}H NMR (700 MHz, $CDCl_3$) δ 7.65 (d, *J* = 8.4 Hz, 2H), 7.55 (dd, *J* = 7.9, 1.8 Hz, 1H), 7.42 (d, *J* = 8.3 Hz, 2H), 7.40 (d, *J* = 8.6 Hz, 1H), 7.34 (d, *J* = 8.4 Hz, 2H), 7.31 – 7.23 (m, 5H), 6.90 – 6.84 (m, 3H), 5.06 (s, 1H), 4.67 (d, *J* = 10.3 Hz, 1H), 4.57 – 4.46 (m, 2H), 3.79 (s, 3H), 3.75 (s, 3H), 3.10 (d, *J* = 12.8 Hz, 1H), 2.40 (s, 3H).

^{13}C NMR (176 MHz, $CDCl_3$) δ 192.2, 173.0, 159.2, 145.0, 145.0, 141.5, 136.2, 134.7, 131.6 (2C), 130.3 (2C), 130.0 (2C), 129.1 (2C), 128.9, 126.9, 126.8 (2C), 123.3, 122.8, 118.9, 117.7, 114.2 (2C), 111.5, 67.6, 62.9, 60.7, 55.4, 53.3, 52.6, 50.8, 21.7.

HRMS(ESI): $[M+H]^+$ calcd. $C_{35}H_{32}N_3O_6S$ m/z 622.2006, found 622.1992.

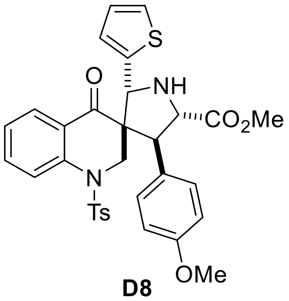

D8

(±)-Methyl (2*R*,3*R*,4*S*,5*S*)-4-(4-methoxyphenyl)-4'-oxo-2-(thiophen-2-yl)-1'-tosyl-1',4'-dihydro-2'*H*-spiro[pyrrolidine-3,3'-quinoline]-5-carboxylate (D8)

PQ **D8** (49 mg, 0.08 mmol, 82% Yield, d.r. >20:1) was synthesized according to the general procedure 7; column chromatography eluting with cyclohexane/EA 5:1 to 2:1.

^{1}H NMR (500 MHz, $CDCl_3$) δ 7.70 (dd, *J* = 8.0, 1.7 Hz, 1H), 7.66 (d, *J* = 8.4 Hz, 2H), 7.49 (d, *J* = 8.6 Hz, 1H), 7.33 – 7.22 (m, 5H), 7.03 (dd, *J* = 5.0, 1.2 Hz, 1H), 6.91 (ddd, *J* = 8.1, 7.2, 1.0 Hz, 1H), 6.86 (d, *J* = 8.8 Hz, 2H), 6.78 (dd, *J* = 3.5, 1.2 Hz, 1H), 6.66 (dd, *J* = 5.1, 3.5 Hz, 1H), 5.29 (s, 1H), 4.70 (d, *J* = 10.4 Hz, 1H), 4.52 (d, *J* = 12.8 Hz, 1H), 4.44 (d, *J* = 10.4 Hz, 1H), 3.79 (s, 3H), 3.73 (s, 3H), 3.10 (d, *J* = 12.8 Hz, 1H), 2.39 (s, 3H).

^{13}C NMR (126 MHz, $CDCl_3$) δ 192.4, 172.6, 159.1, 144.8, 142.8, 141.7, 136.2, 134.4, 130.2 (2C), 130.0 (2C), 129.0, 127.3, 126.9 (2C), 126.4, 126.4, 125.3, 123.1, 123.0, 117.7, 114.1 (2C), 63.8, 62.6, 60.1, 55.4, 53.2, 52.6, 50.9, 21.7.

HRMS(ESI): $[M+H]^+$ calcd. $C_{32}H_{31}N_2O_6S_2$ m/z 603.1618, found 603.1602.

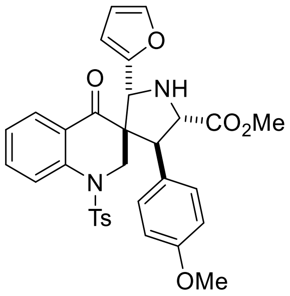

D9

(±)-Methyl (2*R*,3*R*,4*S*,5*S*)-2-(furan-2-yl)-4-(4-methoxyphenyl)-4'-oxo-1'-tosyl-1',4'-dihydro-2'*H*-spiro[pyrrolidine-3,3'-quinoline]-5-carboxylate (D9)

PQ **D9** (41 mg, 0.07 mmol, 70% Yield, d.r. >20:1) was synthesized according to the general procedure 7; column chromatography eluting with cyclohexane/EA 5:1 to 2:1.

^{1}H NMR (500 MHz, CDCl$_3$) δ 7.80 (dd, *J* = 7.9, 1.8 Hz, 1H), 7.63 (d, *J* = 8.5 Hz, 2H), 7.44 (d, *J* = 8.6 Hz, 1H), 7.32 – 7.23 (m, 5H), 7.08 (dd, *J* = 1.9, 0.8 Hz, 1H), 6.94 (ddd, *J* = 8.1, 7.2, 1.0 Hz, 1H), 6.88 (d, *J* = 8.7 Hz, 2H), 6.25 (d, *J* = 3.3 Hz, 1H), 6.03 (dd, *J* = 3.3, 1.8 Hz, 1H), 5.00 (s, 1H), 4.58 (d, *J* = 9.5 Hz, 1H), 4.43 (d, *J* = 12.9 Hz, 1H), 4.34 (d, *J* = 9.4 Hz, 1H), 3.81 (s, 3H), 3.75 (s, 3H), 3.08 (d, *J* = 12.9 Hz, 1H), 2.39 (s, 3H).

^{13}C NMR (126 MHz, CDCl$_3$) δ 192.7, 172.9, 159.1, 150.4, 144.7, 142.2, 141.8, 136.5, 134.4, 130.2 (2C), 129.9 (2C), 128.8, 127.9, 126.8 (2C), 123.0, 122.6, 117.7, 114.2 (2C), 110.2, 109.5, 63.8, 63.2, 59.4, 55.4, 53.1, 52.6, 52.2, 21.7.

HRMS(ESI): $[M+H]^+$ calcd. $C_{32}H_{31}N_2O_7S$ m/z 587.1847, found 587.1828.

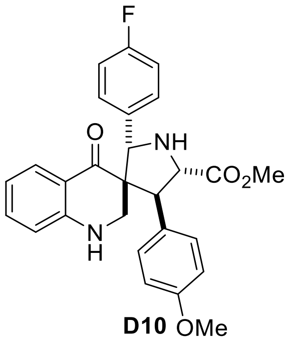

D10

(±)-Methyl (2*S*,3*R*,4*S*,5*S*)-2-(4-fluorophenyl)-4-(4-methoxyphenyl)-4'-oxo-1',4'-dihydro-2'*H*-spiro[pyrrolidine-3,3'-quinoline]-5-carboxylate (D10)

PQ **D10** (28 mg, 0.06 mmol, 60% Yield) was synthesized according to the general procedure 8; column chromatography eluting with cyclohexane/acetone 5:1 to 1:1.

^{1}H NMR (700 MHz,CD$_2$Cl$_2$) δ 7.32 (dd, *J* = 8.1, 1.6 Hz, 1H), 7.23 (d, *J* = 8.7 Hz, 2H), 7.17-7.14 (m, 2H), 7.11 (ddd, *J* = 8.3, 7.1, 1.6 Hz, 1H), 6.84 (d, *J* = 8.7 Hz, 2H), 6.72-6.68 (m, 2H), 6.49 (t, *J* = 6.9 Hz, 1H), 6.44 (dd, *J* = 8.3, 1.2 Hz, 1H), 4.76 (s, 1H), 4.50 (d, *J* = 9.5 Hz, 1H), 4.27 (d, *J* = 9.5 Hz, 1H), 3.76 (s, 3H), 3.72 (s, 3H), 3.24 – 3.15 (m, 1H), 3.01 (dd, *J* = 12.4, 6.7 Hz, 1H).

^{13}C NMR (176 MHz, CD$_2$Cl$_2$) δ 193.4, 174.0, 162.4 (d, *J*CF = 244.6 Hz), 159.3, 150.7, 136.1 (d, *J*CF = 1.8 Hz), 135.0, 130.3 (2C), 130.1 (d, *J*CF = 8.8 Hz, 2C), 129.2, 127.9, 119.9, 117.9, 115.4, 114.3 (d, *J*CF = 21.1 Hz, 2C), 114.1 (2C), 68.8, 63.9, 60.2, 55.6, 52.6, 51.5, 49.8.

^{19}F NMR (470 MHz, CDCl$_3$) δ -115.0 (ddd, *J* = 14.2, 8.9, 5.4 Hz, 1F).

HRMS(ESI): $[M+H]^+$ calcd. $C_{27}H_{26}N_2O_4F$ m/z 461.1871, found 461.1859.

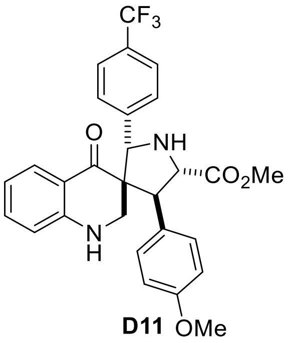

D11

(±)-Methyl (2*S*,3*R*,4*S*,5*S*)-4-(4-methoxyphenyl)-4'-oxo-2-(4-(trifluoromethyl)phenyl)-1',4'-dihydro-2'*H*-spiro[pyrrolidine-3,3'-quinoline]-5-carboxylate (D11)

PQ **D11** (25 mg, 0.05 mmol, 48% Yield) was synthesized according to the general procedure 8; column chromatography eluting with cyclohexane/acetone 5:1 to 1:1.

^{1}H NMR (700 MHz, CD_2Cl_2) δ 7.31 (d, *J* = 8.1 Hz, 2H), 7.30 – 7.25 (m, 3H), 7.23 (d, *J* = 8.7 Hz, 2H), 7.11 (ddd, *J* = 8.4, 7.1, 1.6 Hz, 1H), 6.84 (d, *J* = 8.7 Hz, 2H), 6.48 (t, *J* = 7.5 Hz, 1H), 6.45 (d, *J* = 8.2 Hz, 1H), 4.85 (s, 1H), 4.53 (d, *J* = 9.5 Hz, 1H), 4.32 (d, *J* = 9.6 Hz, 1H), 3.77 (s, 3H), 3.73 (s, 3H), 3.23 (dd, *J* = 12.4, 3.9 Hz, 1H), 3.04 (d, *J* = 12.4 Hz, 1H).

^{13}C NMR (176 MHz, CD_2Cl_2) δ 193.0, 173.9, 159.4, 150.6, 144.8, 135.1, 130.3 (2C), 129.5 (q, *J*CF = 31.7 Hz), 129.0, 128.9 (2C), 128.0, 124.6 (q, *J*CF = 271.0 Hz), 124.5 (q, *J*CF = 3.5 Hz, 2C), 119.9, 118.1, 115.4, 114.1 (2C), 68.7, 63.9, 60.6, 55.6, 52.6, 51.1, 49.6.

^{19}F NMR (470 MHz, $CDCl_3$) δ -62.7 (s, 3F).

HRMS(ESI): $[M+H]^+$ calcd. $C_{28}H_{26}N_2O_4F_3$ m/z 511.1839, found 511.1824.

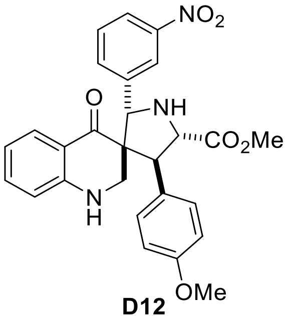

D12

(±)-Methyl (2*S*,3*R*,4*S*,5*S*)-4-(4-methoxyphenyl)-2-(3-nitrophenyl)-4'-oxo-1',4'-dihydro-2'*H*-spiro[pyrrolidine-3,3'-quinoline]-5-carboxylate (D12)

PQ **D12** (21 mg, 0.04 mmol, 44% Yield) was synthesized according to the general procedure 8; column chromatography eluting with cyclohexane/acetone 5:1 to 1:1.

^{1}H NMR (700 MHz, CD_2Cl_2) δ 8.09 (s, 1H), 7.84 (dd, *J* = 8.2, 2.3 Hz, 1H), 7.54 (d, *J* = 7.7 Hz, 1H), 7.27 (d, *J* = 8.0 Hz, 1H), 7.26 – 7.18 (m, 3H), 7.12 – 7.08 (m, 1H), 6.84 (d, *J* = 8.7 Hz, 2H), 6.49 – 6.44 (m, 2H), 4.91 (s, 1H), 4.56 (d, *J* = 9.5 Hz, 1H), 4.36 (d, *J* = 9.6 Hz, 1H), 3.77 (s, 3H), 3.75 (s, 3H), 3.24 (d, *J* = 12.5 Hz, 1H), 3.04 (d, *J* = 12.4 Hz, 1H).

^{13}C NMR (176 MHz, CD_2Cl_2) δ 192.8, 173.8, 159.4, 150.6, 147.7, 143.0, 135.3, 134.4, 130.3 (2C), 128.7, 128.6, 127.9, 123.8, 122.6, 119.9, 118.3, 115.5, 114.2 (2C), 68.1, 63.8, 60.4, 52.6, 50.9, 50.6, 49.6.

HRMS(ESI): $[M+H]^+$ calcd. $C_{27}H_{26}N_3O_6$ m/z 488.1816, found 488.1807.

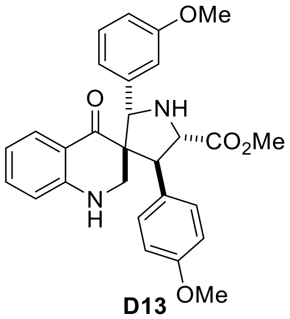

D13

(±)-Methyl (2*S*,3*R*,4*S*,5*S*)-2-(3-methoxyphenyl)-4-(4-methoxyphenyl)-4'-oxo-1',4'-dihydro-2'*H*-spiro[pyrrolidine-3,3'-quinoline]-5-carboxylate (D13)

PQ **D13** (19 mg, 0.04 mmol, 40% Yield) was synthesized according to the general procedure 8; column chromatography eluting with cyclohexane/acetone 5:1 to 1:1.

^{1}H NMR (700 MHz, CD_2Cl_2) δ 7.33 (dd, *J* = 8.4, 1.7 Hz, 1H), 7.24 (d, *J* = 8.8 Hz, 2H), 7.12 (ddd, *J* = 8.5, 7.1, 1.6 Hz, 1H), 6.95 (t, *J* = 7.9 Hz, 1H), 6.83 (d, *J* = 8.8 Hz, 2H), 6.75 (d, *J* = 7.7 Hz, 1H), 6.71 – 6.68 (m, 1H), 6.57 (dd, *J* = 8.2, 2.6 Hz, 1H), 6.53 – 6.49 (m, 2H), 4.79 (s, 1H), 4.50 (d, *J* = 9.9 Hz, 1H), 4.30 (d, *J* = 10.0 Hz, 1H), 3.76 (s, 3H), 3.71 (s, 3H), 3.58 (s, 3H), 3.26 (dd, *J* = 12.3, 3.9 Hz, 1H), 3.05 (d, *J* = 12.3 Hz, 1H).

^{13}C NMR (176 MHz, CD_2Cl_2) δ 193.1, 174.1, 159.3, 159.3, 150.8, 142.3, 134.8, 130.4 (2C), 129.0, 128.7, 128.0, 120.6, 120.2, 117.8, 115.4, 114.0 (2C), 113.8, 113.7, 68.8, 63.7, 60.8, 55.6, 55.4, 52.5, 51.4, 49.5.

HRMS(ESI): $[M+H]^+$ calcd. $C_{28}H_{29}N_2O_5$ m/z 473.2071, found 473.2061.

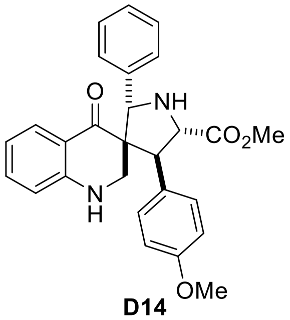

D14

(±)-Methyl (2*S*,3*R*,4*S*,5*S*)-4-(4-methoxyphenyl)-4'-oxo-2-phenyl-1',4'-dihydro-2'*H*-spiro[pyrrolidine-3,3'-quinoline]-5-carboxylate (D14)

PQ **D14** (24 mg, 0.05 mmol, 54% Yield) was synthesized according to the general procedure 8; column chromatography eluting with cyclohexane/acetone 5:1 to 1:1.

^{1}H NMR (700 MHz, CD_2Cl_2) δ 7.29 (d, *J* = 8.0 Hz, 1H), 7.24 (d, *J* = 8.7 Hz, 2H), 7.18 – 7.13 (m, 2H), 7.08 (ddd, *J* = 8.4, 7.0, 1.6 Hz, 1H), 7.03-7.00 (m, 3H), 6.84 (d, *J* = 8.7 Hz, 2H),, 6.51 – 6.39 (m, 2H), 4.77 (s, 1H), 4.50 (d, *J* = 9.6 Hz, 1H), 4.45 (d, *J* = 3.8 Hz, 1H), 4.28 (d, *J* = 9.6 Hz, 1H), 3.76 (s, 3H), 3.72 (s, 3H), 3.27 – 3.19 (m, 1H), 3.03 (d, *J* = 12.4 Hz, 1H).

^{13}C NMR (176 MHz, CD_2Cl_2) δ 193.4, 174.0, 159.3, 150.8, 140.2, 134.8, 130.3 (2C), 129.3, 128.4 (2C), 127.9, 127.7 (2C), 120.0, 117.7, 115.3, 114.0 (2C), 69.6, 64.0, 60.6, 55.6, 52.6, 51.8, 49.8.

HRMS(ESI): $[M+H]^+$ calcd. $C_{27}H_{27}N_2O_4$ m/z 443.1965, found 443.1950.

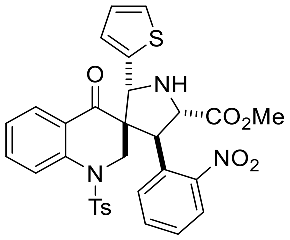

D15

(±)-Methyl (2*R*,3*R*,4*S*,5*S*)-4-(2-nitrophenyl)-4'-oxo-2-(thiophen-2-yl)-1'-tosyl-1',4'-dihydro-2'*H*-spiro[pyrrolidine-3,3'-quinoline]-5-carboxylate (D15)

PQ **D15** (16 mg, 0.03 mmol, 51% Yield) was synthesized as a single diastereoisomer according to the general procedure 7 from **9b** (22 mg, 0.05 mmol); column chromatography eluting with cyclohexane/EA 5:1 to 1:1.

^{1}H NMR (500 MHz, CDCl$_3$) δ 7.93 (d, *J* = 8.0 Hz, 1H), 7.87 – 7.79 (m, 3H), 7.56 – 7.52 (m, 1H), 7.48 (d, *J* = 8.4 Hz, 2H),, 7.38 (d, *J* = 8.6 Hz, 1H), 7.25-7.20 (m, 3H), 7.12 (dd, *J* = 3.6, 1.2 Hz, 1H), 7.01 (dd, *J* = 5.1, 1.2 Hz, 1H), 6.94 (ddd, *J* = 8.0, 7.2, 1.0 Hz, 1H), 6.71 (dd, *J* = 5.1, 3.6 Hz, 1H), 5.30 (s, 1H), 4.88 (d, *J* = 6.1 Hz, 1H), 4.32 (d, *J* = 6.1 Hz, 1H), 4.26 (d, *J* = 12.5 Hz, 1H), 3.83 (s, 3H), 3.46 (d, *J* = 12.5 Hz, 1H), 2.36 (s, 3H).

^{13}C NMR (126 MHz, CDCl$_3$) δ 193.9, 172.1, 151.0, 144.7, 141.9, 136.0, 134.7, 133.5, 132.7, 130.3, 130.2 (2C), 129.2, 129.0, 128.4, 127.0, 126.7 (2C), 126.5, 125.8, 124.8, 123.2, 122.7, 117.5, 67.5, 66.4, 59.3, 53.1, 52.8, 48.6, 21.7.

HRMS(ESI): $[M+H]^+$ calcd. $C_{31}H_{28}N_3O_7S_2$ m/z 618.1363, found 618.1361.

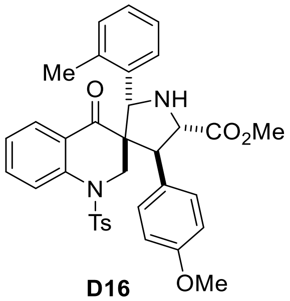

D16

(±)-Methyl (2*S*,3*R*,4*S*,5*S*)-4-(4-methoxyphenyl)-4'-oxo-2-(*o*-tolyl)-1'-tosyl-1',4'-dihydro-2'*H*-spiro[pyrrolidine-3,3'-quinoline]-5-carboxylate (D16)

PQ **D16** (55 mg, 0.09 mmol, 91% Yield, d.r. 7:1) was synthesized according to the general procedure 7; column chromatography eluting with cyclohexane/EA 5:1 to 2:1.

^{1}H NMR (600 MHz, CDCl$_3$) δ 7.67 (d, *J* = 8.7 Hz, 1H), 7.56 (d, *J* = 8.4 Hz, 2H), 7.47 (dd, *J* = 7.9, 1.8 Hz, 1H), 7.35 (d, *J* = 7.9 Hz, 1H), 7.31 (d, *J* = 8.8 Hz, 2H), 7.25 – 7.18 (m, 3H), 7.07 – 7.03 (m, 1H), 6.93 (td, *J* = 7.5, 1.4 Hz, 1H), 6.86 (d, *J* = 8.9 Hz, 2H), 6.82 – 6.78 (m, 2H), 5.28 (s, 1H), 4.71 (d, *J* = 11.0 Hz, 1H), 4.54 – 4.45 (m, 2H), 3.79 (s, 3H), 3.71 (s, 3H), 2.92 (d, *J* = 12.5 Hz, 1H), 2.34 (d, *J* = 2.7 Hz, 3H), 2.20 (s, 3H).

^{13}C NMR (151 MHz, CDCl$_3$) δ 192.7, 173.1, 159.1, 144.9, 141.3, 137.4, 136.5, 134.9, 134.3, 130.3, 130.1 (2C), 130.0 (2C), 128.8, 128.2, 127.5, 127.1 (2C), 126.0, 123.2, 123.0, 117.4, 114.1 (2C), 63.4, 62.7, 61.3, 55.4, 53.3, 52.6, 52.5, 21.6, 19.5.

HRMS(ESI): $[M+H]^+$ calcd. $C_{35}H_{35}N_2O_6S$ m/z 611.2210, found 611.2196.

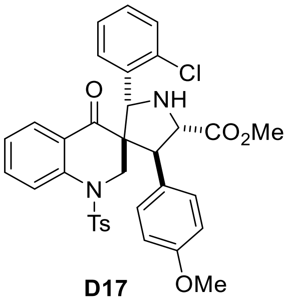

D17

(±)-Methyl (2*R*,3*R*,4*S*,5*S*)-2-(2-chlorophenyl)-4-(4-methoxyphenyl)-4'-oxo-1'-tosyl-1',4'-dihydro-2'*H*-spiro[pyrrolidine-3,3'-quinoline]-5-carboxylate (D17)

PQ **D17** (52 mg, 0.08 mmol, 82% Yield, d.r. >20:1) was synthesized according to the general procedure 7; column chromatography eluting with cyclohexane/EA 5:1 to 2:1.

^{1}H NMR (500 MHz, $CDCl_3$) δ 7.83 (d, *J* = 7.9 Hz, 1H), 7.71 (d, *J* = 8.4 Hz, 2H), 7.61-7.56 (m, 2H), 7.32 – 7.22 (m, 6H), 7.08 – 7.01 (m, 2H), 6.91 – 6.86 (m, 1H), 6.82 (d, *J* = 8.8 Hz, 2H), 5.68 (s, 1H), 4.74 (d, J = 11.3 Hz, 1H), 4.61 (dd, J = 12.0, 10.2 Hz, 2H), 3.77 (s, 3H), 3.73 (s, 3H), 3.22 (d, J = 12.7 Hz, 1H), 2.39 (s, 3H).

^{13}C NMR (126 MHz, $CDCl_3$) δ 191.6, 173.6, 159.1, 144.7, 142.4, 136.2, 134.5, 133.3, 130.6, 130.2 (2C), 130.2 (2C), 129.1, 128.8, 128.6, 127.0 (2C), 126.9, 126.3, 123.6, 123.0, 117.9, 114.0 (2C), 62.2, 62.0, 61.9, 55.3, 52.6, 52.1, 50.7, 21.7.

HRMS(ESI): $[M+H]^+$ calcd. $C_{34}H_{32}N_2O_6ClS$ m/z 631.1664, found 631.1662.

Synthesis of pyrroquinolines E

General Procedure 9

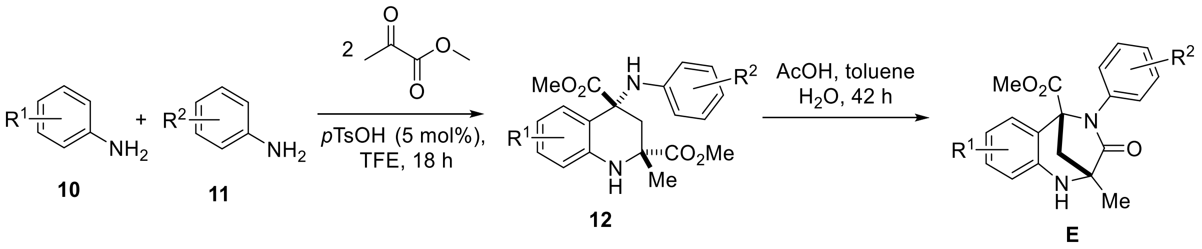

Aniline **10** (200 µmol, 1 equiv.), aniline **11** (200 µmol, 1 equiv.) and methyl pyruvate (400 µM, 2 equiv.) were dissolved in TFE (2 mL) and stirred for 5 minutes at the indicated temperature (T_1 in the table below). Subsequently, *p*-toluene sulfonic acid monohydrate (20 µmol) was added. The reaction mixture was stirred until full conversion was observed (18 h at r.t. or 24 h at lower temperatures). Frequently a white precipitate was observed during the reaction. The suspension was diluted with DCM (usually 2-4 mL) until the precipitate dissolved. The mixture was washed with water (2 mL) and subsequently dried over Na_2SO_4. The solvent was removed under reduced pressure. The obtained crude solid was used to determine the diastereomeric ratio of the THQ intermediate **12** by ^{1}H-NMR in DCM-*d2* (see Tab. **S2**).

The solid was then dissolved in toluene (2 mL) and two drops of water were added. After the addition of glacial acetic acid (1 mmol, 5 equiv.) the reaction mixture was heated to the indicated temperature (T_2 in the table below) for 42 hours. The reaction was cooled to room temperature and diluted with DCM (4 mL). The organic phase was washed with water (2 mL) and subsequently dried over Na_2SO_4. The product was purified by column chromatography using the indicated solvent mixtures.

Procedure	T_1 [°C]	T_2 [°C]
a	r.t.	110
b	r.t.	90
c	5	50
d	5	90
e	0	50

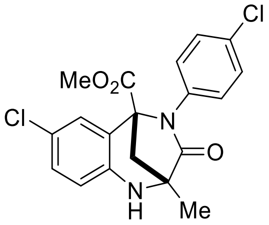

E1

(±)-Methyl 7-chloro-4-(4-chlorophenyl)-2-methyl-3-oxo-1,2,3,4-tetrahydro-5*H*-2,5-methanobenzo[*e*][1,4]diazepine-5-carboxylate (E1)

PQ **E1** (55 mg, 140 µmol, 70%) was synthesised according to the general procedure 9a; column chromatography eluting with DCM/EA 98:2.

^{1}H NMR (500 MHz, $CDCl_3$) δ = 7.26 - 7.23 (m, 2H), 7.14 (dd, *J* = 8.6, 2.4 Hz, 1H), 7.11 (d, *J* = 2.3 Hz, 1H), 6.88 - 6.84 (m, 2H), 6.57 (d, *J* = 8.5 Hz, 1H), 4.48 - 4.45 (m, 1H), 3.79 (s, 3H), 2.49 (d, *J* = 11.1 Hz, 1H), 2.44 (dd, *J* = 11.1, 1.6 Hz, 1H), 1.59 (s, 3H).

^{13}C NMR (126 MHz, $CDCl_3$) δ = 172.4, 169.3, 140.5, 135.8, 133.3, 130.0, 129.1 (2C), 128.4 (2C), 126.4, 122.7, 122.2, 117.0, 67.6, 57.2, 53.0, 41.1, 19.0.

HRMS(ESI): $[M+H]^+$ calcd. $C_{19}H_{17}N_2O_3Cl_2$ m/z 390.0538, found 390.0536.

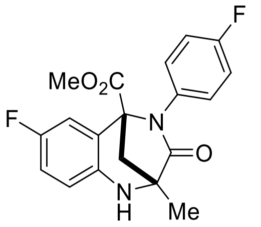

E2

(±)-Methyl 7-fluoro-4-(4-fluorophenyl)-2-methyl-3-oxo-1,2,3,4-tetrahydro-5*H*-2,5-methanobenzo[*e*][1,4]diazepine-5-carboxylate (E2)

PQ **E2** (44 mg, 120 µmol, 61%) was synthesised according to the general procedure 9a; column chromatography eluting with DCM/EA 98:2.

^{1}H NMR (500 MHz, CDCl$_3$) δ 6.98 - 6.91 (m, 3H), 6.88 - 6.82 (m, 3H), 6.60 (dd, *J* = 8.8, 4.7 Hz, 1H), 4.34 (s, 1H), 3.79 (s, 3H), 2.51 (d, *J* = 11.1 Hz, 1H), 2.46 (d, *J* = 11.1 Hz, 1H), 1.59 (s, 3H).

^{13}C NMR (126 MHz, CDCl$_3$) δ = 173.0, 169.4, 161.8 (d, *J*CF = 247.3 Hz),155.3 (d, *J*CF = 237.6 Hz), 138.2 (d, *J*CF = 2.2 Hz), 133.1 (d, *J*CF = 3.2 Hz), 129.4 (d, *J*CF = 8.6 Hz, 2C), 122.0 (d, *J*CF = 6.7 Hz), 116.9 (d, *J*CF = 1.8 Hz), 116.8 (d, *J*CF = 13.2 Hz), 115.8 (d, *J*CF = 22.4 Hz, 2C), 113.6 (d, *J*CF = 24.1 Hz), 67.6, 57.2, 52.8, 41.3, 19.2.

^{19}F NMR (470 MHz, CDCl$_3$) δ -113.75 - -113.84 (m), -125.28 (ddd, J = 8.6, 8,6, 4.6 Hz)

HRMS(ESI): $[M+H]^+$ calcd. $C_{19}H_{17}N_2O_3F_2$ m/z 359.1201, found 359.1214.

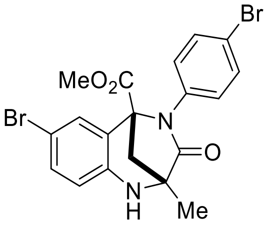

E3

(±)-Methyl 7-bromo-4-(4-bromophenyl)-2-methyl-3-oxo-1,2,3,4-tetrahydro-5*H*-2,5-methanobenzo[*e*][1,4]diazepine-5-carboxylate (E3)

PQ **E3** (69 mg, 143 µmol, 71%) was synthesised according to the general procedure 9a; column chromatography eluting with DCM/EA 98:2.

^{1}H NMR (500 MHz, CDCl$_3$) δ 7.43 - 7.38 (m, 2H), 7.26 (td, *J* = 9.0, 2.3 Hz, 2H), 6.83 - 6.78 (m, 2H), 6.51 (d, *J* = 8.5 Hz, 1H), 4.52 (d, *J* = 1.7 Hz, 1H), 3.79 (s, 3H), 2.48 (d, *J* = 11.2 Hz, 1H), 2.43 (dd, *J* = 11.1, 1.7 Hz, 1H), 1.58 (s, 4H).

^{13}C NMR (126 MHz, CDCl$_3$) δ = 172.3, 169.2, 140.9, 136.4, 132.8 (2C), 132.1, 129.2, 128.7 (2C), 122.6, 121.4, 117.4, 109.6, 67.5, 57.2, 53.0, 41.1, 19.0.

HRMS(ESI): $[M+H]^+$ calcd. $C_{19}H_{17}N_2O_3Br_2$ m/z 478.9600, found 478.9611.

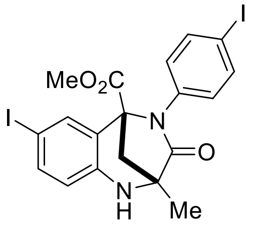

E4

(±)-Methyl 7-iodo-4-(4-iodophenyl)-2-methyl-3-oxo-1,2,3,4-tetrahydro-5*H*-2,5-methanobenzo[*e*][1,4]diazepine-5-carboxylate (E4)

PQ **E4** (30 mg, 52.2 µmol, 26%) was synthesised according to the general procedure 9a; column chromatography eluting with DCM/EA 98:2.

^{1}H NMR (600 MHz, CDCl$_3$) δ 7.62 - 7.58 (m, 2H), 7.45 (dd, J = 8.4, 2.0 Hz, 1H), 7.41 (d, J = 1.9 Hz, 1H), 6.71 - 6.68 (m, 2H), 6.42 (d, J = 8.5 Hz, 1H), 4.42 (d, J = 1.8 Hz, 1H), 3.79 (s, 3H), 2.48 (d, J = 11.2 Hz, 1H), 2.42 (dd, J = 11.2, 1.8 Hz, 1H), 1.58 (s, 3H).

^{13}C NMR (151 MHz, CDCl$_3$) δ 172.1, 169.3, 141.6, 138.6, 138.0 (2C), 137.2, 134.9, 128.7 (2C), 123.3, 117.9, 92.8, 78.7, 67.3, 57.2, 53.0, 41.1, 19.0.

HRMS(ESI): $[M+H]^+$ calcd. $C_{19}H_{17}N_2O_3I_2$ m/z 574.9323, found 574.9346.

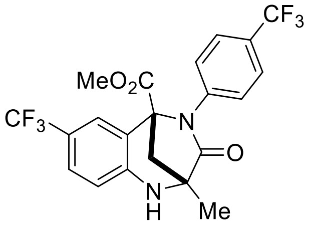

E5

(±)-Methyl 2-methyl-3-oxo-7-(trifluoromethyl)-4-(4-(trifluoromethyl)phenyl)-1,2,3,4-tetrahydro-5*H*-2,5-methanobenzo[*e*][1,4]diazepine-5-carboxylate (E5)

PQ **E5** (46 mg, 100 µmol, 50%) was synthesised according to the general procedure 9a; column chromatography eluting with DCM/EA 97:3.

^{1}H NMR (700 MHz, CDCl$_3$) δ 7.54 (d, *J* = 8.4 Hz, 2H), 7.51 (d, *J* = 2.0 Hz, 1H), 7.42 (dd, *J* = 8.6, 2.1 Hz, 1H), 7.13 (d, *J* = 8.4 Hz, 2H), 6.67 (d, *J* = 8.4 Hz, 1H), 4.81 (s, 1H), 3.79 (s, 3H), 2.52 (d, *J* = 11.2 Hz, 1H), 2.50 (dd, *J* = 11.3, 1.5 Hz, 1H), 1.64 (s, 3H).

^{13}C NMR (176 MHz, CDCl$_3$) δ 171.7, 169.1, 144.7, 140.7, 129.0 (q, *J*CF=32.8), 127.2 (q, *J*CF=3.7, 2C), 125.9 (2C), 124.5 (q, *J*CF=270.9 Hz), 124.0 (q, *J*CF=3.9 Hz), 123.9 (q, *J*CF=272.4 Hz), 120.4, 119.8 (q, *J*CF=33.1 Hz), 115.5, 67.4, 57.3, 53.1, 41.0, 18.8.

^{19}F NMR (565 MHz, CDCl$_3$) δ -61.2 (s, 3F), -62.6 (s, 3F).

HRMS(ESI): $[M+H]^+$ calcd. $C_{21}H_{17}N_2O_3F_6$ m/z 459.1137, found 459.1157.

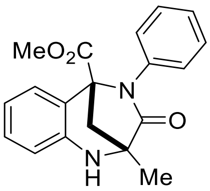

E6

(±)-Methyl 2-methyl-3-oxo-4-phenyl-1,2,3,4-tetrahydro-5*H*-2,5-methanobenzo[*e*][1,4]diazepine-5-carboxylate (E6)

PQ **E6** (28 mg, 86.3 µmol, 43%) was synthesised according to the general procedure 9c; column chromatography eluting with DCM/EA 97:3.

^{1}H NMR (600 MHz, CDCl$_3$) δ 7.28 - 7.22 (m, 2H), 7.20 (td, J = 7.7, 1.4 Hz, 1H), 7.14 (dd, J = 7.7, 1.5 Hz, 1H), 6.97 - 6.94 (m, 2H), 6.76 (td, J = 7.5, 1.1 Hz, 1H), 6.66 (dd, J = 8.0, 1.1 Hz, 1H), 4.46 (s, 1H), 3.78 (s, 3H), 2.57 (d, J = 11.0 Hz, 1H), 2.48 (d, J = 11.0 Hz, 1H), 1.62 (s, 3H).

^{13}C NMR (151 MHz, $CDCl_3$) δ 172.7, 170.0, 142.1, 137.7, 129.9, 128.6 (2C), 127.3, 127.1 (2C), 126.6, 121.5, 117.8, 115.9, 68.0, 57.3, 52.6, 41.6, 19.3.

HRMS(ESI): $[M+H]^+$ calcd. $C_{19}H_{19}N_2O_3$ m/z 323.1390, found 323.1411.

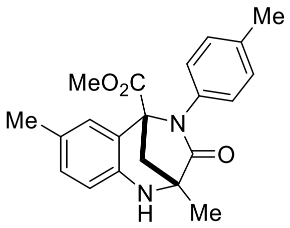

E7

(±)-Methyl 2,7-dimethyl-3-oxo-4-(*p*-tolyl)-1,2,3,4-tetrahydro-5*H*-2,5-methanobenzo[*e*][1,4]diazepine-5-carboxylate (E7)

PQ **E7** (57 mg, 162 µmol, 81%) was synthesised according to the general procedure 9c; column chromatography eluting with DCM/EA 97:3.

^{1}H NMR (700 MHz, $CDCl_3$) δ 7.04 (d, *J* = 8.2 Hz, 2H), 7.00 (dd, *J* = 8.1, 2.0 Hz, 1H), 6.86 - 6.84 (m, 1H), 6.77 - 6.74 (m, 2H), 6.57 (d, *J* = 8.1 Hz, 1H), 4.24 - 4.20 (m, 1H), 3.77 (s, 3H), 2.53 (d, *J* = 11.0 Hz, 1H), 2.44 (dd, *J* = 11.0, 1.8 Hz, 1H), 2.29 (s, 3H), 2.23 (s, 3H), 1.58 (s, 3H)

^{13}C NMR (176 MHz, $CDCl_3$) δ 173.1, 170.1, 139.7, 137.2, 135.0, 130.4, 129.3 (2C), 127.5 (2C), 127.2, 126.9, 121.7, 116.0, 68.1, 57.3, 52.5, 41.9, 21.2, 20.8, 19.4.

HRMS(ESI): $[M+H]^+$ calcd. $C_{21}H_{23}N_2O_3$ m/z 351.1703, found 351.1713.

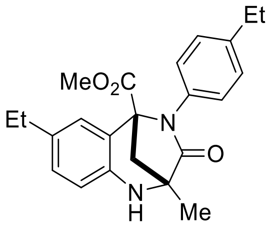

E8

(±)-Methyl 7-ethyl-4-(4-ethylphenyl)-2-methyl-3-oxo-1,2,3,4-tetrahydro-5*H*-2,5-methanobenzo[*e*][1,4]diazepine-5-carboxylate (E8)

PQ **E8** (42 mg, 111 µmol, 55%) was synthesised according to the general procedure 9c; column chromatography eluting with DCM/EA 98:2.

^{1}H NMR (700 MHz, $CDCl_3$) δ 6.99 - 6.97 (m, 2H), 6.95 (dd, *J* = 8.2, 2.0 Hz, 1H), 6.81 (d, *J* = 1.9 Hz, 1H), 6.72 - 6.68 (m, 2H), 6.52 (d, *J* = 8.2 Hz, 1H), 4.14 (s, 1H), 3.70 (s, 3H), 2.52 (q, *J* = 7.6 Hz, 2H), 2.49 - 2.43 (m, 3H), 2.37 (dd, *J* = 11.0, 1.8 Hz, 1H), 1.51 (s, 3H), 1.11 (t, *J* = 7.6 Hz, 3H), 1.08 (t, *J* = 7.6 Hz, 3H).

^{13}C NMR (176 MHz, $CDCl_3$) δ 173.0, 170.1, 143.2, 139.8, 135.1, 133.9, 129.1, 127.9 (2C), 127.3 (2C), 126.0, 121.6, 115.9, 68.0, 57.3, 52.4, 41.8, 28.4, 28.2, 19.4, 16.2, 15.2.

HRMS(ESI): $[M+H]^+$ calcd. $C_{23}H_{27}N_2O_3$ m/z 379.2016, found 379.2034.

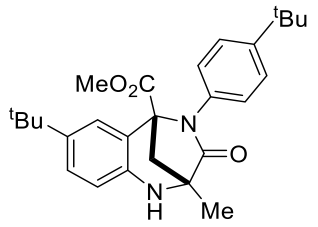

E9

(±)-Methyl 7-(*tert*-butyl)-4-(4-(*tert*-butyl)phenyl)-2-methyl-3-oxo-1,2,3,4-tetrahydro-5*H*-2,5-methanobenzo[*e*][1,4]diazepine-5-carboxylate (E9)

PQ **E9** (55 mg, 126 µmol, 63%) was synthesised according to the general procedure 9c; column chromatography eluting with DCM/EA 97:3.

^{1}H NMR (700 MHz, CDCl$_3$) δ 7.22 - 7.18 (m, 3H), 7.07 (d, *J* = 2.2 Hz, 1H), 6.80 - 6.77 (m, 2H), 6.59 (d, *J* = 8.4 Hz, 1H), 4.21 (s, 1H), 3.79 (s, 3H), 2.56 (d, *J* = 10.9 Hz, 1H), 2.44 (dd, *J* = 11.0, 1.6 Hz, 1H), 1.59 (s, 3H), 1.25 (s, 9H), 1.24 (s, 9H).

^{13}C NMR (176 MHz, CDCl$_3$) δ 173.1, 170.3, 149.9, 140.8, 139.7, 134.9, 126.7 (2C), 126.4, 125.5 (2C), 124.1, 121.4, 115.5, 68.3, 57.5, 52.5, 41.9, 34.6, 34.2, 31.6 (3C), 31.4 (3C), 19.5.

HRMS(ESI): $[M+H]^+$ calcd. $C_{27}H_{35}N_2O_3$ m/z 435.2642, found 435.2659.

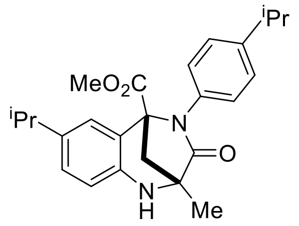

E10

(±)-Methyl 7-isopropyl-4-(4-isopropylphenyl)-2-methyl-3-oxo-1,2,3,4-tetrahydro-5*H*-2,5-methanobenzo[*e*][1,4]diazepine-5-carboxylate (E10)

PQ **E10** (39 mg, 95.9 µmol, 48%) was synthesised according to the general procedure 9c; column chromatography eluting with DCM/EA 97:3.

^{1}H NMR (700 MHz, CDCl$_3$) δ 7.07 - 7.03 (m, 3H), 6.91 (d, *J* = 2.0 Hz, 1H), 6.78 - 6.75 (m, 2H), 6.59 (d, *J* = 8.2 Hz, 1H), 4.20 (d, *J* = 1.8 Hz, 1H), 3.78 (s, 3H), 2.87 - 2.75 (m, 2H), 2.56 (d, *J* = 11.0 Hz, 1H), 2.44 (dd, *J* = 11.0, 1.8 Hz, 1H), 1.58 (s, 3H), 1.21 - 1.15 (m, 12H).

^{13}C NMR (176 MHz, CDCl$_3$) δ 173.1, 170.2, 147.8, 140.0, 138.6, 135.2, 127.5, 127.2 (2C), 126.6 (2C), 125.0, 121.7, 115.9, 68.2, 57.5, 52.5, 41.9, 33.8, 33.5, 24.6, 24.0, 24.0, 23.9, 19.5.

HRMS(ESI): $[M+H]^+$ calcd. $C_{25}H_{31}N_2O_3$ m/z 407.2329, found 407.2345.

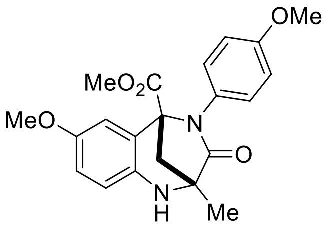

E11

(±)-Methyl 7-methoxy-4-(4-methoxyphenyl)-2-methyl-3-oxo-1,2,3,4-tetrahydro-5*H*-2,5-methanobenzo[*e*][1,4]diazepine-5-carboxylate (E11)

PQ **E11** (41 mg, 106 μmol, 53%) was synthesised according to the general procedure 9e; column chromatography eluting with DCM/EA 98:2 to 9:1.

^{1}H NMR (700 MHz, CDCl$_3$) δ 6.80 (dd, *J* = 8.7, 2.8 Hz, 1H), 6.76 - 6.76 (m, 4H), 6.65 (d, *J* = 2.9 Hz, 1H), 6.62 (d, *J* = 8.7 Hz, 1H), 5.30 (s, 1H), 4.12 (s, 1H), 3.78 (s, 3H), 3.75 (s, 3H), 3.71 (s, 3H), 2.52 (d, *J* = 11.0 Hz, 1H), 2.45 (d, *J* = 11.1 Hz, 1H), 1.57 (s, 3H).

^{13}C NMR (176 MHz, CDCl$_3$) δ 173.6, 170.0, 158.9, 152.0, 136.0, 130.1, 129.3 (2C), 122.7, 117.0, 115.6, 114.0 (2C), 113.0, 68.0, 57.3, 56.1, 55.4, 52.6, 41.9, 19.5.

HRMS(ESI): $[M+H]^+$ calcd. $C_{21}H_{23}N_2O_5$ m/z 383.1601, found 383.1615.

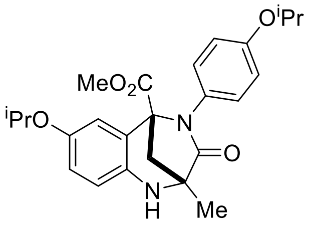

E12

(±)-Methyl 7-isopropoxy-4-(4-isopropoxyphenyl)-2-methyl-3-oxo-1,2,3,4-tetrahydro-5*H*-2,5-methanobenzo[*e*][1,4]diazepine-5-carboxylate (E12)

PQ **E12** (48 mg, 109 μmol, 55%) was synthesised according to the general procedure 9c; column chromatography eluting with DCM/EA 98:2 to 9:1.

^{1}H NMR (600 MHz, CDCl$_3$) δ 6.80 (dd, *J* = 8.7, 2.7 Hz, 1H), 6.75 - 6.69 (m, 4H), 6.65 (d, *J* = 2.7 Hz, 1H), 6.59 (d, *J* = 8.7 Hz, 1H), 4.45 (sept., *J* = 6.1 Hz, 1H), 4.31 (sept., *J* = 6.1 Hz, 1H), 3.77 (s, 3H), 2.53 (d, *J* = 11.0 Hz, 1H), 2.43 (d, J = 11.0 Hz, 1H), 1.57 (s, 3H), 1.29 (dd, J = 6.1, 1.9 Hz, 6H), 1.25 (d, J = 6.1 Hz, 3H), 1.23 (d, J = 6.0 Hz, 3H).

^{13}C NMR (151 MHz, CDCl$_3$) δ 173.5, 169.9, 157.2, 149.7, 136.1, 129.6, 129.3 (2C), 122.5, 119.0, 116.9, 116.0, 115.5 (2C), 71.5, 69.9, 68.0, 57.2, 52.5, 41.7, 22.1, 22.1, 22.0, 22.0, 19.4.

HRMS(ESI): $[M+H]^+$ calcd. $C_{25}H_{31}N_2O_5$ m/z 439.2227, found 439.2245.

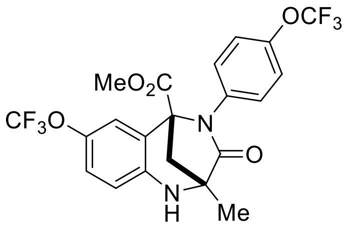

E13

(±)-Methyl 2-methyl-3-oxo-7-(trifluoromethoxy)-4-(4-(trifluoromethoxy)phenyl)-1,2,3,4-tetrahydro-5*H*-2,5-methanobenzo[*e*][1,4]diazepine-5-carboxylate (E13)

PQ **E13** (30 mg, 61.2 μmol, 31%) was synthesised according to the general procedure 9c; column chromatography eluting with DCM/EA 97:3.

^{1}H NMR (600 MHz, CDCl$_3$) δ 7.12 - 7.05 (m, 5H), 6.97 - 6.92 (m, 2H), 6.63 (dd, *J* = 8.6, 2.7 Hz, 1H), 4.48 (s, 1H), 3.79 (s, 3H), 2.52 (dd, *J* = 11.2, 1.2 Hz, 1H), 2.47 (d, *J* = 11.2 Hz, 1H), 1.61 (s, 3H).

^{13}C NMR (151 MHz, CDCl$_3$) δ 172.3, 169.2, 148.1, 140.8, 140.3, 140.3, 135.7, 128.4 (2C), 123.4, 121.5, 121.2, 120.8 (d, *J*CF=256.7 Hz) 120.6, 120.5 (d, *J*CF=257.6 Hz), 116.4 (2C), 67.5, 57.3, 53.0, 41.0, 19.1.

^{19}F NMR (565 MHz, CDCl$_3$) δ -57.9 (s, 3F), -58.7 (s, 3F).

HRMS(ESI): $[M+H]^+$ calcd. $C_{21}H_{17}N_2O_5F_6$ m/z 491.1042, found 123.0680.

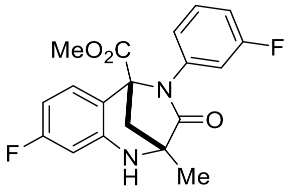

E14

(±)-Methyl 8-fluoro-4-(3-fluorophenyl)-2-methyl-3-oxo-1,2,3,4-tetrahydro-5*H*-2,5-methanobenzo[*e*][1,4]diazepine-5-carboxylate (E14)

PQ **E14** (34 mg, 94.3 µmol, 47%) was synthesised according to the general procedure 9c; column chromatography eluting with DCM/EA 98:2.

^{1}H NMR (700 MHz, CDCl$_3$) δ 7.21 (td, *J* = 8.2, 6.4 Hz, 1H), 7.15 (dd, *J* = 8.6, 5.9 Hz, 1H), 6.93 (tdd, *J* = 8.4, 2.6, 0.9 Hz, 1H), 6.80 (dt, *J* = 10.2, 2.3 Hz, 1H), 6.74 (ddd, *J* = 8.1, 2.0, 0.9 Hz, 1H), 6.44 (td, *J* = 8.5, 2.6 Hz, 1H), 6.32 (dd, *J* = 10.1, 2.5 Hz, 1H), 4.65 – 4.59 (m, 1H), 3.77 (d, *J* = 1.8 Hz, 3H), 2.49 (d, *J* = 11.1 Hz, 1H), 2.44 (dd, *J* = 11.1, 1.7 Hz, 1H), 1.59 (s, 3H).

^{13}C NMR (176 MHz, CDCl$_3$) δ 172.1, 169.5, 163.8 (d, *JCF* = 242.1 Hz), 162.4 (d, *JCF* = 242.3 Hz), 143.5 (d, *JCF* = 11.4 Hz), 138.9 (d, *JCF* = 10.3 Hz), 129.6 (d, *JCF* =9.1), 128.1 (d, *JCF* =10.2), 121.9 (d, *JCF* =3.0), 116.8 (d, *JCF* =2.7), 114.1 (d, *JCF* =15.9), 114.0 (d, *JCF* =18.8), 104.7 (d, *JCF* =22.1), 102.4 (d, *JCF* =25.4), 67.4, 57.2, 52.7, 41.6, 18.9.

^{19}F NMR (377 MHz, CDCl$_3$) δ -111.35 (ddd, *J* = 10.1, 8.3, 5.8 Hz, 1F), -111.83 (ddd, *J* = 10.0, 8.2, 6.6 Hz, 1F)**.**

HRMS(ESI): $[M+H]^+$ calcd. $C_{19}H_{17}N_2O_3F_2$ m/z 359.1201, found 359.1224.

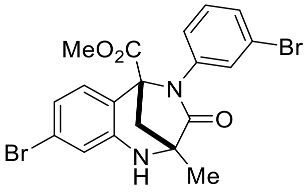

E15

(±)-Methyl 8-bromo-4-(3-bromophenyl)-2-methyl-3-oxo-1,2,3,4-tetrahydro-5*H*-2,5-methanobenzo[*e*][1,4]diazepine-5-carboxylate (E15)

PQ **E15** (12 mg, 25.6 µmol, 13%) was synthesised according to the general procedure 9d; column chromatography eluting with DCM to DCM/EA 98:2.

^{1}H NMR (700 MHz, CDCl$_3$) δ 7.36 (ddd, *J* = 8.0, 1.9, 1.0 Hz, 1H), 7.24 (t, *J* = 2.0 Hz, 1H), 7.12 (t, *J* = 8.1 Hz, 1H), 7.04 (d, *J* = 8.2 Hz, 1H), 6.88 (dd, *J* = 8.3, 1.9 Hz, 1H), 6.84 (ddd, *J* = 8.0, 2.0, 0.9 Hz, 1H), 6.81 (d, *J* = 1.9 Hz, 1H), 4.46 (s, 1H), 3.77 (s, 3H), 2.48 (d, *J* = 11.1 Hz, 1H), 2.43 (d, *J* = 11.1 Hz, 1H), 1.59 (s, 3H)**.**

^{13}C NMR (176 MHz, CDCl$_3$) δ 172.0, 169.3, 143.2, 138.8, 130.4, 130.1, 129.9, 128.0, 125.0, 123.7, 122.1, 120.8, 119.9, 118.5, 67.5, 57.2, 52.9, 41.3, 19.0.

HRMS(ESI): $[M+H]^+$ calcd. $C_{19}H_{17}N_2O_3Br_2$ m/z 478.9600, found 478.9598.

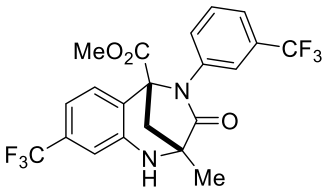

E16

(±)-Methyl 2-methyl-3-oxo-8-(trifluoromethyl)-4-(3-(trifluoromethyl)phenyl)-1,2,3,4-tetrahydro-5*H*-2,5-methanobenzo[*e*][1,4]diazepine-5-carboxylate (E16)

PQ **E16** (50 mg, 110 µmol, 54%) was synthesised according to the general procedure 9a; column chromatography eluting with DCM/EA 98:2.

^{1}H NMR (600 MHz, CDCl$_3$) δ 7.51 - 7.47 (m, 1H), 7.38 (t, *J* = 8.0 Hz, 1H), 7.27 (d, *J* = 6.9 Hz, 2H), 7.12 (dt, *J* = 8.1, 1.6 Hz, 1H), 6.99 (dd, *J* = 8.1, 1.8 Hz, 1H), 6.91 (d, *J* = 1.8 Hz, 1H), 4.61 (s, 1H), 3.79 (s, 3H), 2.54 (d, *J* = 11.3 Hz, 1H), 2.50 (dd, *J* = 11.2, 1.5 Hz, 1H), 1.64 (s, 3H).

^{13}C NMR (151 MHz, CDCl$_3$) δ 172.0, 169.1, 142.1, 137.8, 132.3 (q, *J*CF=32.5), 131.2 (q, *J*CF=32.8), 124.0 (q, *J*CF=3.8), 123.8 (dt, *J*CF=7.7, 3.2), 123.7 (q, *J*CF=272.5), 123.6 (q, *J*CF=272.4), 114.3 (q, *J*CF=4.1), 112.5 (q, *J*CF=3.8)., 67.4, 57.2, 52.9, 40.7, 18.8.

^{19}F NMR (565 MHz, CDCl$_3$) δ -62.9 (s, 3F), -63.2 (s, 3F).

HRMS(ESI): $[M+H]^+$ calcd. $C_{21}H_{17}N_2O_3F_6$ m/z 459.1137, found 459.1156.

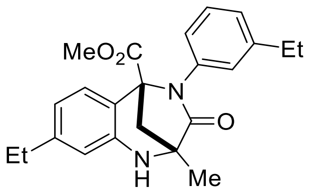

E17

(±)-Methyl 7-ethyl-4-(4-ethylphenyl)-2-methyl-3-oxo-1,2,3,4-tetrahydro-5*H*-2,5-methanobenzo[*e*][1,4]diazepine-5-carboxylate (E17)

PQ **E17** (40 mg, 106 µmol, 53%) was synthesised according to the general procedure 9c; column chromatography eluting with DCM/EA 97:3.

^{1}H NMR (700 MHz, CDCl$_3$) δ 7.15 (t, *J* = 7.8 Hz, 1H), 7.03 (d, *J* = 7.9 Hz, 2H), 6.86 - 6.78 (m, 1H), 6.69 (t, *J* = 1.9 Hz, 1H), 6.57 (dd, *J* = 7.9, 1.7 Hz, 1H), 6.50 (d, *J* = 1.7 Hz, 1H), 4.40 - 4.14 (m, 1H), 3.75 (s, 3H), 2.59 - 2.51 (m, 6H), 2.45 (dd, *J* = 10.9, 1.8 Hz, 1H), 1.55 (s, 3H), 1.20 (t, *J* = 7.6 Hz, 3H), 1.11 (t, *J* = 7.6 Hz, 3H).

^{13}C NMR (176 MHz, CDCl$_3$) δ 172.8, 170.0, 146.2, 144.4, 142.0, 137.6, 128.3, 126.8, 126.7, 126.4, 124.3, 119.1, 117.6, 115.2, 67.8, 57.3, 52.4, 41.9, 28.7, 28.5, 19.3, 15.5, 15.0.

HRMS(ESI): $[M+H]^+$ calcd. $C_{23}H_{27}N_2O_3$ m/z 379.2016, found 123.379.2031.

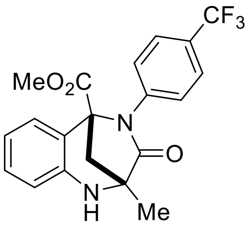

E18

(±)-Methyl 2-methyl-3-oxo-4-(4-(trifluoromethyl)phenyl)-1,2,3,4-tetrahydro-5*H*-2,5-methanobenzo[*e*][1,4]diazepine-5-carboxylate (E18)

PQ **E18** (29 mg, 74.3 µmol, 37%) was synthesised according to the general procedure 9d; column chromatography eluting with DCM/EA 98:2.

^{1}H NMR (700 MHz, $CDCl_3$) δ 7.51 (d, *J* = 8.3 Hz, 2H), 7.19 (td, *J* = 7.7, 1.5 Hz, 1H), 7.17 - 7.15 (m, 3H), 6.77 (td, *J* = 7.5, 1.2 Hz, 1H), 6.64 (dd, *J* = 8.1, 1.2 Hz, 1H), 4.40 (d, *J* = 1.9 Hz, 1H), 3.78 (s, 3H), 2.57 (d, *J* = 11.1 Hz, 1H), 2.46 (dd, *J* = 11.1, 1.8 Hz, 1H), 1.62 (s, 3H).

^{13}C NMR (176 MHz, $CDCl_3$) δ 172.4, 169.8, 141.9, 141.2 (q, *J*CF = 1.1 Hz), 130.1, 128.6 (q, *J*CF = 32.7 Hz), 126.5, 126.3, 125.8 (q, *J*CF = 3.7 Hz), 124.1 (q, *J*CF = 272.0 Hz), 121.0, 118.1, 116.1, 68.0, 57.4, 52.8, 41.6, 19.2.

^{19}F NMR (470 MHz, $CDCl_3$) δ -62.5 8 (s, 3F).

HRMS(ESI): $[M+H]^+$ calcd. $C_{20}H_{18}N_2O_3F_3$ m/z 413.1083, found 413.1099.

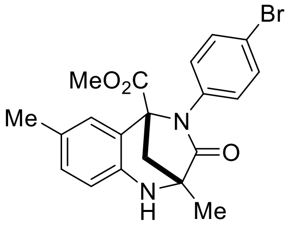

E19

(±)-Methyl 4-(4-bromophenyl)-2,7-dimethyl-3-oxo-1,2,3,4-tetrahydro-5*H*-2,5-methanobenzo[*e*][1,4]diazepine-5-carboxylate (E19)

PQ **E19** (44 mg, 106 µmol, 53%) was synthesised according to the general procedure 9d; column chromatography eluting with DCM/EA 97:3.

^{1}H NMR (700 MHz, $CDCl_3$) δ 7.38 - 7.35 (m, 2H), 7.00 (dd, *J* = 8.2, 2.0 Hz, 1H), 6.84 (d, *J* = 1.9 Hz, 1H), 6.81 - 6.78 (m, 2H), 6.56 (d, *J* = 8.2 Hz, 1H), 4.26 (s, 1H), 3.78 (s, 3H), 2.53 (d, *J* = 11.1 Hz, 1H), 2.42 (dd, *J* = 11.0, 1.3 Hz, 1H), 2.24 (s, 3H), 1.58 (s, 3H).

^{13}C NMR (176 MHz, $CDCl_3$) δ 173.0, 170.0, 139.5, 136.9, 131.8 (2C), 130.7, 129.0 (2C), 127.4, 126.7, 121.2, 121.1, 116.1, 68.1, 57.3, 52.7, 41.7, 20.8, 19.3.

HRMS(ESI): $[M+H]^+$ calcd. $C_{20}H_{20}N_2O_3Br$ m/z 415.0651, found 415.0671.

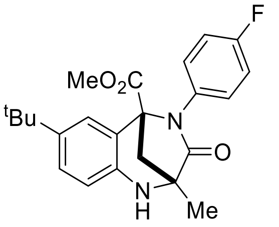

E20

(±)-Methyl 7-(*tert*-butyl)-4-(4-fluorophenyl)-2-methyl-3-oxo-1,2,3,4-tetrahydro-5*H*-2,5-methanobenzo[*e*][1,4]diazepine-5-carboxylate (E20)

PQ **E20** (31 mg, 78.2 µmol, 33%) was synthesised according to the general procedure 9d; column chromatography eluting with DCM/EA 97:3.

^{1}H NMR (700 MHz, $CDCl_3$) δ 7.22 (dd, *J* = 8.4, 2.3 Hz, 1H), 6.95 (d, *J* = 2.4 Hz, 1H), 6.91 - 6.87 (m, 2H), 6.78 - 6.75 (m, 2H), 6.61 (d, *J* = 8.4 Hz, 1H), 4.25 (d, *J* = 1.8 Hz, 1H), 3.79 (s, 3H), 2.58 (d, *J* = 11.0 Hz, 1H), 2.44 (dd, *J* = 11.0, 1.9 Hz, 1H), 1.58 (s, 3H), 1.23 (s, 9H).

^{13}C NMR (176 MHz, CDCl$_3$) δ 173.4, 170.1, 161.8 (d, *J*CF = 246.9 Hz), 141.0, 139.5,133.5 (d, *J*CF = 3.3 Hz),129.9 (d, *J*CF = 8.4, 2C), 126.7, 123.8, 121.0, 115.6, 115.4 (d, *J*CF = 22.5 Hz, 2C), 68.5, 57.4, 52.5, 41.6, 34.1, 31.5 (3C), 19.3.

^{19}F NMR (470 MHz, CDCl$_3$) δ -114.2 (ddd, *J* = 13.3, 8.7, 5.0, 1F).

HRMS(ESI): $[M+H]^+$ calcd. $C_{23}H_{26}N_2O_3F$ m/z 397.1927, found 123.0680.

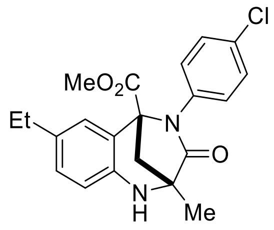

E21

(±)-Methyl 4-(4-chlorophenyl)-7-ethyl-2-methyl-3-oxo-1,2,3,4-tetrahydro-5*H*-2,5-methanobenzo[*e*][1,4]diazepine-5-carboxylate (E21)

PQ **E21** (15 mg, 40.7 µmol, 41%) was synthesised according to the general procedure 9d; column chromatography eluting with DCM/EA 98:2.

^{1}H NMR (700 MHz, CDCl$_3$) δ 7.22 - 7.18 (m, 2H), 7.03 (dd, *J* = 8.2, 2.0 Hz, 1H), 6.85 - 6.81 (m, 3H), 6.59 (d, *J* = 8.2 Hz, 1H), 4.26 (s, 1H), 3.78 (s, 3H), 2.57 - 2.50 (m, 3H), 2.43 (dd, *J* = 11.0, 1.7 Hz, 1H), 1.58 (s, 3H), 1.15 (t, *J* = 7.6 Hz, 3H).

^{13}C NMR (176 MHz, CDCl$_3$) δ 173.0, 170.0, 139.8, 136.4, 134.1, 133.1, 129.5, 128.8 (2C), 128.8 (2C), 125.8, 121.2, 116.1, 68.2, 57.4, 52.6, 41.7, 28.2, 19.3, 16.3.

HRMS(ESI): $[M+H]^+$ calcd. $C_{21}H_{22}N_2O_3Cl$ m/z 385.1313, found 385.1331.

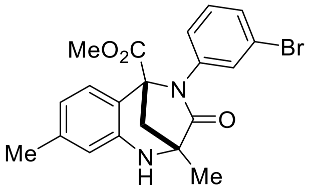

E22

(±)-Methyl 4-(3-bromophenyl)-2,8-dimethyl-3-oxo-1,2,3,4-tetrahydro-5*H*-2,5-methanobenzo[*e*][1,4]diazepine-5-carboxylate (E22)

PQ **E22** (16 mg, 43.8 µmol, 22%) was synthesised according to the general procedure 9d; column chromatography eluting with DCM/EA 98:2.

^{1}H NMR (700 MHz, CDCl$_3$) δ 7.34 (ddd, *J* = 8.0, 1.9, 1.0 Hz, 1H), 7.14 – 7.10 (m, 2H), 7.02 – 7.00 (m, 1H), 6.90 – 6.86 (m, 2H), 6.57 (d, *J* = 8.1 Hz, 1H), 3.79 (s, 3H), 2.54 (d, *J* = 11.0 Hz, 1H), 2.42 (d, *J* = 11.1 Hz, 1H), 2.26 (s, 3H), 1.58 (s, 3H).

^{13}C NMR (176 MHz, CDCl$_3$) δ 172.8, 169.9, 139.5, 139.2, 130.7, 130.3, 130.2, 129.8, 127.5, 126.8, 125.6, 121.9, 121.2, 116.2, 68.1, 57.4, 52.7, 41.8, 20.7, 19.3.

HRMS(ESI): $[M+H]^+$ calcd. $C_{20}H_{20}N_2O_3Br$ m/z 415.0651, found 415.0650.

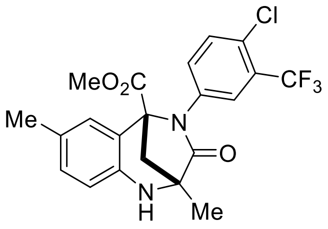

E23

(±)-Methyl 4-(4-chloro-3-(trifluoromethyl)phenyl)-2,7-dimethyl-3-oxo-1,2,3,4-tetrahydro-5*H*-2,5-methanobenzo[*e*][1,4]diazepine-5-carboxylate (E23)

PQ **E23** (37 mg, 84.3 µmol, 42%) was synthesised according to the general procedure 9d; column chromatography eluting with DCM.

^{1}H NMR (600 MHz, $CDCl_3$) δ 7.38 (d, *J*=8.6 Hz, 1H), 7.22 (d, *J*=2.5 Hz, 1H), 7.13 (dd, *J*=8.6, 2.5 Hz, 1H), 7.02 (dd, *J*=8.2, 1.2 Hz, 1H), 6.81 (dd, *J*=1.9, 0.9 Hz, 1H), 6.58 (d, *J*=8.2 Hz, 1H), 3.80 (s, 3H), 2.58 (d, *J*=11.1 Hz, 1H), 2.43 (d, *J*=11.1 Hz, 1H), 2.24 (s, 3H), 1.59 (s, 3H).

^{13}C NMR (151 MHz, $CDCl_3$) δ 173.0, 169.8, 139.4, 136.8, 131.7, 131.7, 131.0, 130.6 (q, *J*CF=2.1 Hz), 128.5 (q, *J*CF=31.8 Hz), 127.9, 126.7, 126.0 (q, *J*CF=5.5 Hz), 122.5 (q, *J*CF=273.4 Hz), 120.9, 116.3, 68.3, 57.4, 52.9, 41.5, 20.6, 19.3.

^{19}F NMR (565 MHz, $CDCl_3$) δ -62.98 (s, 3F).

HRMS(ESI): $[M+H]^+$ calcd. $C_{21}H_{19}N_2O_3ClF_3$ m/z 439.1036, found 439.1040.

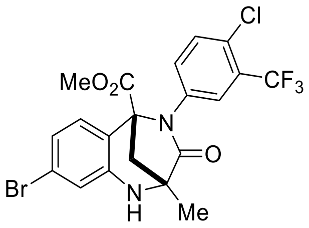

E24

(±)-Methyl 8-bromo-4-(4-chloro-3-(trifluoromethyl)phenyl)-2-methyl-3-oxo-1,2,3,4-tetrahydro-5*H*-2,5-methanobenzo[*e*][1,4]diazepine-5-carboxylate (E24)

PQ **E24** (61 mg, 121 µmol, 60%) was synthesised according to the general procedure 9b; column chromatography eluting with DCM.

^{1}H NMR (600 MHz, $CDCl_3$) δ 7.41 – 7.37 (m, 2H), 7.05 (dd, *J*=8.6, 2.5 Hz, 1H), 6.95 (d, *J*=8.2 Hz, 1H), 6.88 (dd, *J*=8.2, 1.9 Hz, 1H), 6.83 (d, *J*=1.9 Hz, 1H), 4.49 (s, 1H), 3.79 (s, 3H), 2.51 (d, *J*=11.2 Hz, 1H), 2.44 (d, *J*=11.2 Hz, 1H), 1.60 (s, 3H).

^{13}C NMR (151 MHz, $CDCl_3$) δ 172.2, 169.1, 142.9, 136.2, 131.7, 130.7, 128.6 (q, *J*CF=31.8 Hz), 127.6,126.0 (q, *J*CF=5.4 Hz), 123.8, 122.4 (q, *J*CF=273.5 Hz), 120.9, 119.4, 118.5, 67.5, 57.2, 52.9, 40.9, 18.8.

^{19}F NMR (565 MHz, $CDCl_3$) δ -62.93 (s, 3F).

HRMS(ESI): $[M+H]^+$ calcd. $C_{20}H_{16}N_2O_3F_3ClBr$ m/z 502.9979, found 502.9980.

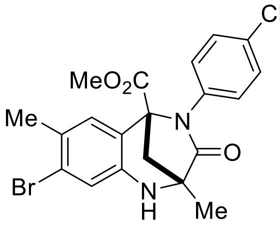

E25

(±)-Methyl 8-bromo-4-(4-chlorophenyl)-2,7-dimethyl-3-oxo-1,2,3,4-tetrahydro-5*H*-2,5-methanobenzo[*e*][1,4]diazepine-5-carboxylate (E25)

PQ **E25** (26 mg, 57.8 µmol, 29%) was synthesised according to the general procedure 9b; column chromatography eluting with DCM/EA 99:1.

^{1}H NMR (600 MHz, CDCl$_3$) δ 7.24 (d, *J* = 8.8 Hz, 2H), 6.93 (s, 1H), 6.89 - 6.85 (m, 4H), 3.77 (s, 3H), 2.47 (d, *J* = 11.1 Hz, 1H), 2.42 (d, *J* = 11.2 Hz, 1H), 2.28 (d, *J* = 0.6 Hz, 3H), 1.56 (s, 3H).

^{13}C NMR (151 MHz, CDCl$_3$) δ 172.7, 169.6, 140.9, 136.1, 133.2, 129.0 (2C), 128.5 (2C), 128.1, 126.8, 125.9, 120.4, 119.3, 67.6, 57.2, 52.8, 41.5, 22.2, 19.1.

HRMS(ESI): $[M+H]^+$ calcd. $C_{20}H_{19}N_2O_3ClBr$ m/z 449.0262, found 449.0262.

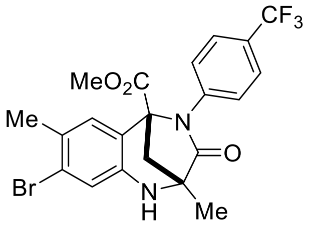

E26

(±)-Methyl 8-bromo-2,7-dimethyl-3-oxo-4-(4-(trifluoromethyl)phenyl)-1,2,3,4-tetrahydro-5*H*-2,5-methanobenzo[*e*][1,4]diazepine-5-carboxylate (E26)

PQ **E26** (30 mg, 62.0 µmol, 31%) was synthesised according to the general procedure 9d; column chromatography eluting with DCM.

^{1}H NMR (600 MHz, CDCl$_3$) δ 7.56 - 7.52 (m, 2H), 7.19 - 7.16 (m, 2H), 7.05 (d, *J* = 0.8 Hz, 1H), 6.87 (s, 1H), 3.77 (s, 3H), 2.50 (d, *J* = 11.2 Hz, 1H), 2.44 (d, *J* = 11.2 Hz, 1H), 2.31 (d, *J* = 0.6 Hz, 3H), 1.59 (s, 3H).

^{13}C NMR (151 MHz, CDCl$_3$) δ 172.3, 169.5, 141.1, 140.9, 128.8 (q, *J*CF=32.7 Hz), 128.1, 127.1, 126.1, 126.0, 126.0 - 125.8 (m, 4C), 124.04 (q, *J*CF= 272.1 Hz), 120.3, 119.5, 67.5, 57.3, 53.0, 41.7, 22.3, 19.2**.**

^{19}F NMR (565 MHz, CDCl$_3$) δ -62.51 (3F).

HRMS(ESI): $[M+H]^+$ calcd. $C_{21}H_{19}N_2O_3F_3Br$ m/z 483.0525, found 483.0501.

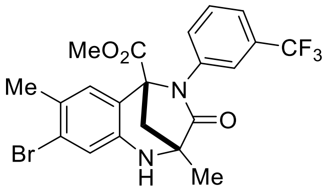

E27

(±)-Methyl 8-bromo-2,7-dimethyl-3-oxo-4-(3-(trifluoromethyl)phenyl)-1,2,3,4-tetrahydro-5*H*-2,5-methanobenzo[*e*][1,4]diazepine-5-carboxylate (E27)

PQ **E27** (29 mg, 60.0 µmol, 30%) was synthesised according to the general procedure 9d; column chromatography eluting with DCM/EA 99:1.

^{1}H NMR (600 MHz, CDCl$_3$) δ 7.48 (d, *J*=7.8 Hz, 1H), 7.39 (t, *J*=7.9 Hz, 1H), 7.26 – 7.25 (m, 1H), 7.16 (d, *J*=8.0 Hz, 1H), 6.97 (s, 1H), 6.89 (s, 1H), 3.77 (s, 3H), 2.52 (d, *J*=11.1 Hz, 1H), 2.45 (d, *J*=11.2 Hz, 1H), 2.28 (s, 3H), 1.59 (s, 3H).

^{13}C NMR (151 MHz, CDCl$_3$) δ 172.5, 169.4, 140.8, 138.2, 131.1 (q, *J*CF = 32.6 Hz), 130.1, 129.3, 128.2, 127.2, 125.9, 124.0, 123.9 (q, *J*CF = 3.6 Hz), 123.8 (q, *J*CF = 3.9 Hz), 123.7 (q, *J*CF = 272.5 Hz), 120.4, 119.4, 67.7, 57.3, 52.9, 41.5, 22.0, 19.1.

^{19}F NMR (565 MHz, CDCl$_3$) δ -62.8 (s, 3F).

HRMS(ESI): $[M+H]^+$ calcd. $C_{21}H_{19}N_2O_3BrF_3$ m/z 483.0525, found 483.0504.

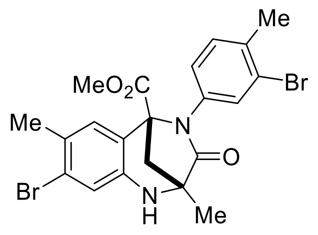

E28

(±)-Methyl 8-bromo-4-(3-bromo-4-methylphenyl)-2,7-dimethyl-3-oxo-1,2,3,4-tetrahydro-5*H*-2,5-methanobenzo[*e*][1,4]diazepine-5-carboxylate (E28)

PQ **E28** (55 mg, 105 µmol, 54%) was synthesised according to the general procedure 9c; column chromatography eluting with DCM/EA 97:3.

^{1}H NMR (700 MHz, CDCl$_3$) δ 7.17 (d, *J* = 2.2 Hz, 1H), 7.11 (d, *J* = 8.2 Hz, 1H), 6.94 (s, 1H), 6.87 (s, 1H), 6.72 (dd, *J* = 8.1, 2.2 Hz, 1H), 4.44 – 4.39 (m, 1H), 3.79 (s, 3H), 2.47 (d, *J* = 11.1 Hz, 1H), 2.42 (dd, *J* = 11.1, 1.8 Hz, 1H), 2.34 (s, 3H), 2.28 (s, 3H), 1.56 (s, 3H).

^{13}C NMR (176 MHz, CDCl$_3$) δ 172.6, 169.5, 140.9, 137.2, 136.1, 131.2, 130.6, 128.2, 126.9, 125.9, 125.8, 124.4, 120.5, 119.3, 67.6, 57.2, 52.8, 41.5, 22.6, 22.1, 19.1.

HRMS(ESI): $[M+H]^+$ calcd. $C_{21}H_{21}N_2O_3Br_2$ m/z 506.9913, found 506.9937.

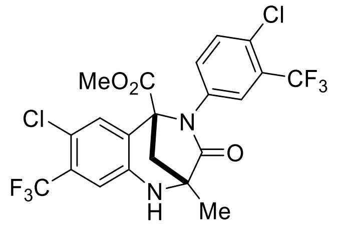

E29

(±)-Methyl 7-chloro-4-(4-chloro-3-(trifluoromethyl)phenyl)-2-methyl-3-oxo-8-(trifluoromethyl)-1,2,3,4-tetrahydro-5*H*-2,5-methanobenzo[*e*][1,4]diazepine-5-carboxylate (E29)

PQ **E29** (25 mg, 47.4 µmol, 24%) was synthesised according to the general procedure 9a; column chromatography eluting with DCM/EA 99:1.

^{1}H NMR (600 MHz, CDCl$_3$) δ 7.43 (d, *J* = 8.6 Hz, 1H), 7.35 (d, *J* = 2.6 Hz, 1H), 7.32 (s, 1H), 7.07 (dd, *J* = 8.6, 2.6 Hz, 1H), 7.00 (s, 1H), 4.64 (s, 1H), 3.82 (s, 3H), 2.50 (s, 2H), 1.63 (s, 3H).

^{13}C NMR (151 MHz, CDCl$_3$) δ 171.6, 168.4, 140.3, 135.7, 132.0, 131.0, 130.4, 129.7 (q, *J*CF = 31.8), 129.2, 128.8 (d, *J*CF = 32.1), 125.7 (q, *J*CF = 5.5), 124.4, 122.3 (q, *J*CF = 273.20), 122.2 (q, *J*CF = 273.6), 120.1, 114.8 (q, *J*CF = 5.5), 66.8, 57.2, 53.2, 40.3, 18.6.

^{19}**F NMR** (565 MHz, $CDCl_3$) δ -63.0 (s, 3F), -63.1 (s, 3F).

HRMS(ESI): $[M+H]^+$ calcd. $C_{21}H_{15}N_2O_3Cl_2F_6$ m/z 527.0358, found 527.0383.

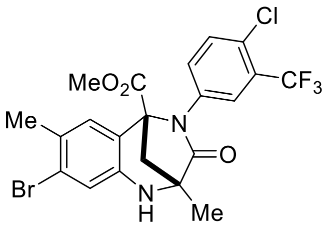

E30

(±)-Methyl 8-bromo-4-(4-chloro-3-(trifluoromethyl)phenyl)-2,7-dimethyl-3-oxo-1,2,3,4-tetrahydro-5*H*-2,5-methanobenzo[*e*][1,4]diazepine-5-carboxylate (E30)

PQ **E30** (50 mg, 96.6 µmol, 48%) was synthesised according to the general procedure 9d; column chromatography eluting with DCM.

^{1}**H NMR (700 MHz, $CDCl_3$)** δ 7.40 (d, *J* = 8.6 Hz, 1H), 7.32 (d, *J* = 2.5 Hz, 1H), 7.12 (dd, *J* = 8.6, 2.5 Hz, 1H), 6.92 (s, 1H), 6.88 (s, 1H), 4.47 (d, *J* = 1.9 Hz, 1H), 3.79 (s, 3H), 2.51 (d, *J* = 11.2 Hz, 1H), 2.43 (dd, *J* = 11.3, 1.6 Hz, 1H), 2.28 (s, 3H), 1.57 (s, 3H).

^{13}**C NMR (176 MHz, $CDCl_3$)** δ 172.6, 169.3, 140.8, 136.5, 131.8, 131.1, 130.7 (q, *J*CF = 1.9), 128.6 (q, *J*CF = 31.7 Hz), 128.0, 127.2, 126.1, 125.8 (q, *J*CF = 5.4), 122.5 (q, *J*CF = 273.5 Hz), 120.0, 119.4, 67.7, 57.3, 53.0, 41.3, 22.0, 19.0.

HRMS(ESI): $[M+H]^+$ calcd. $C_{21}H_{18}BrClF_3N_2O_3$ m/z 517.0135, found 517.0134.

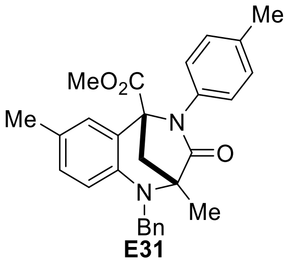

E31

(±)-Methyl 1-benzyl-2,7-dimethyl-3-oxo-4-(*p*-tolyl)-1,2,3,4-tetrahydro-5*H*-2,5-methanobenzo[*e*][1,4]diazepine-5-carboxylate (E31)

PQ **E7** (17.5 mg, 50.0 µmol) was dissolved in dry DMF (1 ml) and cooled in a n ice bath. NaH (60% in mineral oil, 2.4 mg, 60.0 µmol) was added and the reaction was stirred for 30 minutes. Benzyl bromide (17.8 µl, 150 µmol) was added and the reaction was heated to 60 °C for 18 hours. The reaction was quenched with sat. aq. NH_4Cl solution (3 ml) and extracted three times with DCM (3 ml). The combined organic phases were washed four times with water (5 ml) and dried over Na_2SO_4. The solvent was removed under reduced pressure. The product was purified by column chromatography (Pent:EtOAc 96:4). This procedure afforded pure PQ **E31** (8 mg, 18.1 µmol, 36%)

^{1}**H NMR (500 MHz, $CDCl_3$)** δ 7.40 – 7.33 (m, 5H), 7.08 – 7.05 (m, 2H), 7.00 – 6.95 (m, 1H), 6.88 (d, *J* = 2.0 Hz, 1H), 6.85 (d, *J* = 8.4 Hz, 2H), 6.55 (d, *J* = 8.4 Hz, 1H), 4.91 (d, *J* = 18.5 Hz, 1H), 4.25 (d, *J* = 18.5 Hz, 1H), 3.79 (s, 3H), 2.76 (d, *J* = 11.3 Hz, 1H), 2.48 (d, *J* = 11.3 Hz, 1H), 2.30 (s, 3H), 2.24 (s, 3H), 1.54 (s, 3H)..

^{13}**C NMR (126 MHz, $CDCl_3$)** δ 170.7, 170.2, 143.2, 140.9, 137.0, 135.2, 130.6, 129.3 (2C), 128.8 (2C), 128.7, 127.8, 127.2 (2C), 127.1, 126.8, 126.8, 126.2, 125.8 (2C), 122.4, 115.0, 67.7, 65.5, 60.6, 53.4, 52.6, 44.0, 21.2, 20.6, 18.5.

HRMS(ESI): $[M+H]^+$ calcd. $C_{28}H_{29}N_2O_3$ m/z 441.2178, found 441.2181.

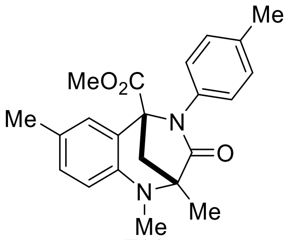

E32

(±)-Methyl 1,2,7-trimethyl-3-oxo-4-(*p*-tolyl)-1,2,3,4-tetrahydro-5*H*-2,5-methanobenzo[*e*][1,4]diazepine-5-carboxylate (E32)

PQ **E7** (17.5 mg, 50.0 µmol) was dissolved in dry DMF (1 ml) and cooled in a n ice bath. NaH (60% in mineral oil, 2.4 mg, 60.0 µmol) was added and the reaction was stirred for 30 minutes. Methyl iodide (9.4 ul, 150 µmol) was added and the reaction was heated to 60 °C for 18 hours. The reaction was quenched with sat. aq. NH_4Cl solution and extracted three times with DCM (3 mL). The combined organic phases were washed four times with water (5 mL) and dried over Na_2SO_4. The solvent was removed under reduced pressure. The product was purified by column chromatography (Pent/EtOAc 96:4, R_f 0.63). This procedure afforded pure PQ **E32** (10 mg, 27.4 µmol, 55%).

^{1}H NMR (500 MHz, $CDCl_3$) δ 7.08 (dd, *J* = 8.5, 2.1 Hz, 1H), 7.05 – 7.01 (m, 2H), 6.83 (d, *J* = 2.1 Hz, 1H), 6.81 (d, *J* = 8.5 Hz, 1H), 6.79 (d, *J* = 8.3 Hz, 2H), 3.76 (s, 3H), 2.97 (s, 3H), 2.58 (d, *J* = 11.2 Hz, 1H), 2.40 (d, *J* = 11.2 Hz, 1H), 2.28 (s, 3H), 2.24 (s, 3H), 1.65 (s, 3H).

^{13}C NMR (126 MHz, $CDCl_3$) δ 170.2, 169.9, 143.0, 136.9, 135.2, 130.3, 129.3 (2C), 127.2 (2C), 126.4, 126.3, 122.9, 115.3, 67.7, 60.4, 52.5, 43.7, 34.6, 21.2, 20.6, 18.7.

HRMS(ESI): $[M+H]^+$ calcd. $C_{22}H_{25}N_2O_3$m/z 365.1865, found 365.1875.

Synthesis of pyrroquinolines F

General Procedure 10

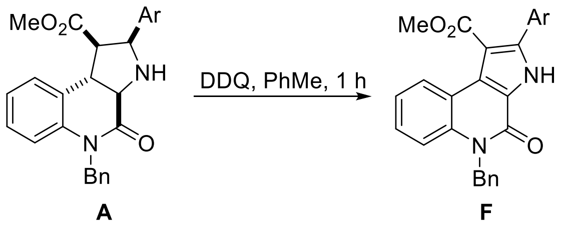

PQ **A** (0.05 mmol, 1.0 equiv.) was dissolved in toluene (0.5 mL) and DDQ (0.2 mmol, 4.0 equiv.) was added. The reaction was stirred for 1 h. Sat. $NaHCO_3$ solution (10 mL) was then added to quench the reaction and the mixture was extracted with DCM (3*10 mL). The combined organic phases were dried over Na_2SO_4 and filtered. The solvent was removed under reduced pressure and the PQs **F** were purified by column chromatography using DCM/MeOH mixtures.

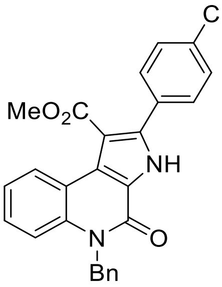

F1

Methyl 5-benzyl-2-(4-chlorophenyl)-4-oxo-4,5-dihydro-3*H*-pyrrolo[2,3-*c*]quinoline-1-carboxylate (F1)

PQ **F1** (21 mg, 48 µmol, 96% Yield) was synthesized according to the general procedure 10; column chromatography eluting with cyclohexane/EA 1:1 then DCM/MeOH 20:1.

^{1}H NMR (500 MHz, DMSO-*d6*) δ 13.30 (s, 1H), 8.52 (dd, J = 8.1, 1.5 Hz, 1H), 7.62 – 7.54 (m, 4H), 7.45 – 7.35 (m, 2H), 7.31 (dd, J = 8.3, 6.6 Hz, 2H), 7.27 – 7.18 (m, 4H), 5.68 (s, 2H), 3.72 (s, 3H).

^{13}C NMR (126 MHz, DMSO-*d6*) δ 166.4, 154.6, 141.5, 137.1, 136.0, 133.7, 130.9 (2C), 129.8, 128.7 (2C), 128.3 (2C), 127.7, 127.1, 126.4 (2C), 125.1, 124.6, 123.0, 122.2, 117.4, 116.3, 108.7, 51.9, 44.5.

HRMS(ESI): $[M+H]^+$ calcd. $C_{26}H_{20}N_2O_3Cl$ m/z 443.1157, found 443.1171.

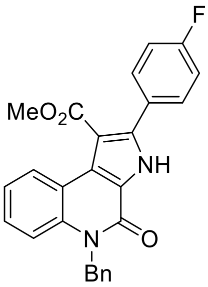

F2

Methyl 5-benzyl-2-(4-fluorophenyl)-4-oxo-4,5-dihydro-3*H*-pyrrolo[2,3-*c*]quinoline-1-carboxylate (F2)

PQ **F2** (18 mg, 41 µmol, 83% Yield) was synthesized according to the general procedure 10; column chromatography eluting with cyclohexane/EA 1:1 then DCM/MeOH 20:1.

^{1}H NMR (500 MHz, DMSO-*d6*) δ 13.24 (s, 1H), 8.53 (dd, J = 8.1, 1.5 Hz, 1H), 7.66 – 7.60 (m, 2H), 7.43 (dd, J = 8.7, 1.3 Hz, 1H), 7.41 – 7.28 (m, 5H), 7.28 – 7.19 (m, 4H), 5.68 (s, 2H), 3.71 (s, 3H).

^{13}C NMR (126 MHz, DMSO-*d6*) δ 166.4, 162.4 (d, *J*CF = 247.0 Hz), 154.6, 141.8, 137.2, 136.0, 131.4 (d, *J*CF = 8.8 Hz, 2C), 128.7 (2C), 127.6, 127.5, 127.4, 127.0, 126.4 (2C), 125.1, 124.6, 122.8, 122.1, 117.5, 116.3, 115.2 (d, *J*CF = 21.4 Hz, 2C), 108.5, 51.8, 44.5.

^{19}F NMR (470 MHz, DMSO-*d6*) δ -112.6 (ddd, *J* = 14.3, 8.9, 5.3 Hz, 1F).

HRMS(ESI): $[M+H]^+$ calcd. $C_{26}H_{20}N_2O_3F$ m/z 427.1453, found 427.1452.

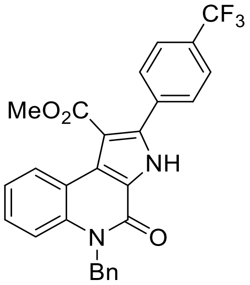

F3

Methyl 5-benzyl-4-oxo-2-(4-(trifluoromethyl)phenyl)-4,5-dihydro-3*H*-pyrrolo[2,3-*c*]quinoline-1-carboxylate (F3)

PQ **F3** (18 mg, 37 µmol, 74% Yield) was synthesized according to the general procedure 10; column chromatography eluting with cyclohexane/EA 1:1 then DCM/MeOH 20:1.

^{1}H NMR (500 MHz, DMSO-*d6*) δ 13.44 (s, 1H), 8.54 (dd, *J* = 8.0, 1.4 Hz, 1H), 7.92 – 7.73 (m, 4H), 7.49 – 7.36 (m, 2H), 7.34 – 7.19 (m, 6H), 5.69 (s, 2H), 3.72 (s, 3H).

^{13}C NMR (126 MHz, DMSO-*d6*) δ 166.2, 154.6, 140.9, 137.1, 136.0, 135.0, 130.0 (2C), 128.9 (q, *J*CF = 31.5 Hz), 128.7 (2C), 127.8, 127.1, 126.4 (2C), 125.1 (2C), 124.6, 124.2 (q, *J*CF = 272.2 Hz), 123.4, 122.2, 117.4, 116.4, 109.3, 51.9, 44.5.

^{19}F NMR (470 MHz, $CDCl_3$) δ -62.3 (s, 3F).

HRMS(ESI): $[M+H]^+$ calcd. $C_{27}H_{20}F_3N_2O_3$ m/z 477.1421, found 477.1417.

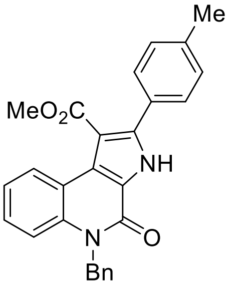

F4

Methyl 5-benzyl-4-oxo-2-(*p*-tolyl)-4,5-dihydro-3*H*-pyrrolo[2,3-*c*]quinoline-1-carboxylate (F4)

PQ **F4** (10 mg, 24 µmol, 56% Yield) was synthesized according to the general procedure 10 from **A5** (18 mg, 42 µmol); column chromatography eluting with cyclohexane/EA 1:1 then DCM/MeOH 20:1.

^{1}H NMR (500 MHz, DMSO-*d6*) δ 13.13 (s, 1H), 8.43 (dd, *J* = 8.1, 1.5 Hz, 1H), 7.49 – 7.45 (m, 2H), 7.42 (dd, *J* = 8.7, 1.3 Hz, 1H), 7.37 (ddd, *J* = 8.6, 7.0, 1.5 Hz, 1H), 7.33 – 7.28 (m, 4H), 7.27 – 7.19 (m, 4H), 5.68 (s, 2H), 3.72 (s, 3H), 2.38 (s, 3H).

^{13}C NMR (126 MHz, DMSO-*d6*) δ 166.9, 154.6, 142.5, 138.4, 137.2, 135.9, 128.9 (2C), 128.8 (2C), 128.7 (2C), 128.0, 127.6, 127.0, 126.4 (2C), 124.8, 124.6, 122.6, 122.1, 117.5, 116.3, 108.2, 51.8, 44.4, 21.0.

HRMS(ESI): $[M+H]^+$ calcd. $C_{27}H_{23}N_2O_3$ m/z 423.1703, found 423.1703.

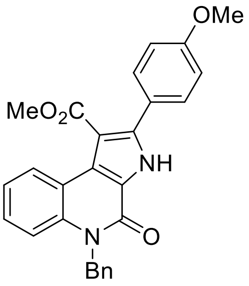

F5

Methyl 5-benzyl-2-(4-methoxyphenyl)-4-oxo-4,5-dihydro-3*H*-pyrrolo[2,3-*c*]quinoline-1-carboxylate (F5)

PQ **F5** (13 mg, 30 µmol, 60% Yield) was synthesized according to the general procedure 10; column chromatography eluting with cyclohexane/EA 1:1 then DCM/MeOH 20:1.

^{1}H NMR (500 MHz, DMSO-*d6*) δ 13.07 (s, 1H), 8.44 (dd, *J* = 8.1, 1.5 Hz, 1H), 7.56 – 7.50 (m, 2H), 7.42 (dd, *J* = 8.7, 1.3 Hz, 1H), 7.37 (ddd, *J* = 8.5, 6.9, 1.6 Hz, 1H), 7.33-7.28 (m, 2H), 7.27 – 7.19 (m, 4H), 7.08 – 7.03 (m, 2H), 5.68 (s, 2H), 3.83 (s, 3H), 3.73 (s, 3H).

^{13}C NMR (126 MHz, DMSO-*d6*) δ 166.9, 159.8, 154.5, 142.5, 137.2, 135.9, 130.3 (2C), 128.7 (2C), 127.5, 127.0, 126.4 (2C), 124.8, 124.6, 123.1, 122.4, 122.1, 117.5, 116.3, 113.7 (2C), 107.9, 55.3, 51.8, 44.4.

HRMS(ESI): $[M+H]^+$ calcd. $C_{27}H_{23}N_2O_4$ m/z 439.1652, found 439.1651.

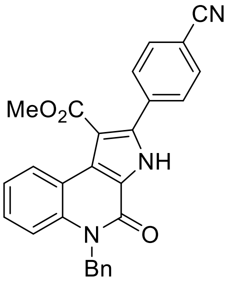

F6

Methyl 5-benzyl-2-(4-cyanophenyl)-4-oxo-4,5-dihydro-3*H*-pyrrolo[2,3-*c*]quinoline-1-carboxylate (F6)

PQ **F6** (16 mg, 38 µmol, 75% Yield) was synthesized according to the general procedure 10; column chromatography eluting with cyclohexane/EA 1:1 then DCM/MeOH 20:1.

^{1}H NMR (600 MHz, DMSO-*d6*) δ 13.45 (s, 1H), 8.55 (dd, J = 8.0, 1.5 Hz, 1H), 8.12 – 7.89 (m, 2H), 7.89 – 7.68 (m, 2H), 7.47 – 7.37 (m, 2H), 7.34 – 7.19 (m, 6H), 5.69 (s, 2H), 3.72 (s, 3H).

^{13}C NMR (151 MHz, DMSO-*d6*) δ 166.0, 154.6, 140.7, 137.0, 136.0, 135.5, 132.0 (2C), 130.0 (2C), 128.7 (2C), 127.8, 127.0, 126.4 (2C), 125.1, 124.6, 123.5, 122.2, 118.6, 117.3, 116.3, 111.2, 109.4, 51.9, 44.5.

HRMS(ESI): $[M+H]^+$ calcd. $C_{27}H_{20}N_3O_3$ m/z 434.1499, found 434.1497.

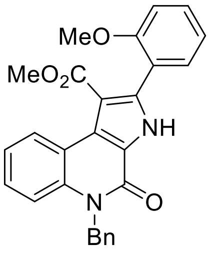

F7

Methyl 5-benzyl-2-(2-methoxyphenyl)-4-oxo-4,5-dihydro-3*H*-pyrrolo[2,3-*c*]quinoline-1-carboxylate (F7)

PQ **F7** (22 mg, 49 µmol, 98% Yield) was synthesized according to the general procedure 10; column chromatography eluting with cyclohexane/EA 1:1 then DCM/MeOH 20:1.

^{1}H NMR (500 MHz, DMSO-*d6*) δ 13.03 (s, 1H), 8.70 (dd, *J* = 8.1, 1.5 Hz, 1H), 7.47 – 7.40 (m, 3H), 7.37 (ddd, *J* = 8.6, 7.0, 1.6 Hz, 1H), 7.33 – 7.28 (m, 2H), 7.26 – 7.18 (m, 4H), 7.13 – 7.09 (m, 1H), 7.06 (td, *J* = 7.5, 1.0 Hz, 1H), 5.68 (s, 2H), 3.75 (s, 3H), 3.59 (s, 3H).

^{13}C NMR (126 MHz, DMSO-*d6*) δ 166.2, 156.5, 154.5, 139.9, 137.2, 135.9, 130.9, 130.4, 128.7 (2C), 127.4, 127.0, 126.4 (2C), 125.4, 124.4, 122.5, 121.9, 120.5, 120.1, 117.7, 116.2, 110.9, 110.1, 55.4, 51.3, 44.4.

HRMS(ESI): $[M+H]^+$ calcd. $C_{27}H_{23}N_2O_4$ m/z 439.1652, found 439.1651.

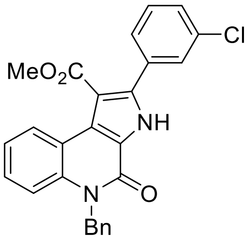

F8

Methyl 5-benzyl-2-(3-chlorophenyl)-4-oxo-4,5-dihydro-3*H*-pyrrolo[2,3-*c*]quinoline-1-carboxylate (F8)

PQ **F8** (20 mg, 45 µmol, 90% Yield) was synthesized according to the general procedure 10; column chromatography eluting with cyclohexane/EA 1:1 then DCM/MeOH 20:1.

^{1}H NMR (500 MHz, DMSO-*d6*) δ 13.34 (s, 1H), 8.52 (dd, *J* = 8.1, 1.5 Hz, 1H), 7.69-7.68 (m, 1H), 7.55-7.51 (m, 3H), 7.45 – 7.36 (m, 2H), 7.33 – 7.28 (m, 2H), 7.28 – 7.19 (m, 4H), 5.68 (s, 2H), 3.72 (s, 3H).

^{13}C NMR (126 MHz, DMSO-*d6*) δ 166.2, 154.6, 141.0, 137.1, 136.0, 132.9, 132.8, 130.0, 128.8, 128.7 (2C), 127.9, 127.7, 127.0, 126.4 (2C), 125.1, 124.6, 123.1, 122.2, 117.4, 116.3, 108.9, 51.8, 44.5.

HRMS(ESI): $[M+H]^+$ calcd. $C_{26}H_{20}N_2O_3Cl$ m/z 443.1157, found 443.1155.

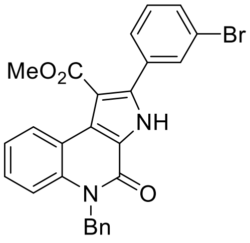

F9

Methyl 5-benzyl-2-(3-bromophenyl)-4-oxo-4,5-dihydro-3*H*-pyrrolo[2,3-*c*]quinoline-1-carboxylate (F9)

PQ **F9** (20 mg, 42 µmol, 83% Yield) was synthesized according to the general procedure 10; column chromatography eluting with cyclohexane/EA 1:1 then DCM/MeOH 20:1.

^{1}H NMR (500 MHz, DMSO-*d6*) δ 13.32 (s, 1H), 8.52 (dd, *J* = 8.0, 1.5 Hz, 1H), 7.82 (t, *J* = 1.9 Hz, 1H), 7.67 (ddd, *J* = 8.0, 2.1, 1.0 Hz, 1H), 7.56 (ddd, *J* = 7.7, 1.7, 1.0 Hz, 1H), 7.49 – 7.42 (m, 2H), 7.39 (ddd, *J* = 8.5, 7.0, 1.5 Hz, 1H), 7.31-7.29 (m, 2H), 7.28 – 7.18 (m, 4H), 5.68 (s, 2H), 3.73 (s, 3H).

^{13}C NMR (126 MHz, DMSO-*d6*) δ 166.2, 154.5, 140.8, 137.1, 136.0, 133.1, 131.6, 131.6, 130.2, 128.7 (2C), 128.2, 127.7, 127.0, 126.4 (2C), 125.0, 124.5, 123.1, 122.2, 121.3, 117.4, 116.3, 108.9, 51.8, 44.5.

HRMS(ESI): $[M+H]^+$ calcd. $C_{26}H_{20}N_2O_3Br$ m/z 487.0652, found 487.0648.

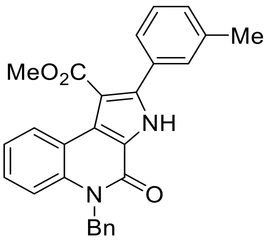

F10

Methyl 5-benzyl-4-oxo-2-(*m*-tolyl)-4,5-dihydro-3*H*-pyrrolo[2,3-*c*]quinoline-1-carboxylate (F10)

PQ **F10** (16 mg, 38 µmol, 76% Yield) was synthesized according to the general procedure 10; column chromatography eluting with cyclohexane/EA 1:1 then DCM/MeOH 20:1.

^{1}H NMR (400 MHz, DMSO-*d6*) δ 13.14 (s, 1H), 8.44 (dd, *J* = 8.1, 1.5 Hz, 1H), 7.46 – 7.11 (m, 12H), 5.68 (s, 2H), 3.72 (s, 3H), 2.39 (s, 3H).

^{13}C NMR (101 MHz, DMSO-*d6*) δ 166.8, 154.5, 142.5, 137.4, 137.2, 135.9, 130.7, 129.5, 128.7 (2C), 128.1, 127.5, 127.0, 126.4 (2C), 126.0, 124.8, 124.5, 122.6, 122.1, 117.4, 116.3, 108.4, 51.8, 44.4, 21.0.

HRMS(ESI): $[M+H]^+$ calcd. $C_{27}H_{23}N_2O_3$ m/z 423.1703, found 423.1702.

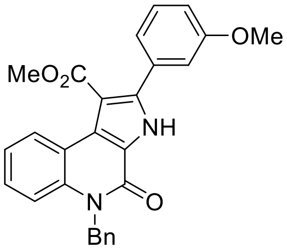

F11

Methyl 5-benzyl-2-(3-methoxyphenyl)-4-oxo-4,5-dihydro-3*H*-pyrrolo[2,3-*c*]quinoline-1-carboxylate (F11)

PQ **F11** (20 mg, 45 µmol, 89% Yield) was synthesized according to the general procedure 10; column chromatography eluting with cyclohexane/EA 1:1 then DCM/MeOH 20:1.

^{1}H NMR (400 MHz, DMSO-*d6*) δ 13.18 (s, 1H), 8.39 (dd, *J* = 8.1, 1.5 Hz, 1H), 7.46 – 7.35 (m, 3H), 7.34 – 7.19 (m, 7H), 7.14 – 7.09 (m, 1H), 7.02 (ddd, *J* = 8.3, 2.6, 0.9 Hz, 1H), 5.69 (s, 2H), 3.85 (s, 3H), 3.75 (s, 3H).

^{13}C NMR (101 MHz, DMSO-*d6*) δ 166.8, 159.0, 154.5, 141.8, 137.1, 135.9, 132.0, 129.4, 128.7 (2C), 127.5, 127.0, 126.4 (2C), 124.6, 124.4, 122.6, 122.1, 121.1, 117.4, 116.3, 115.0, 113.7, 108.6, 55.2, 51.9, 44.4.

MS(ESI): $[M+H]^+$ calcd. $C_{27}H_{23}N_2O_4$ m/z 439.2, found 439.1.

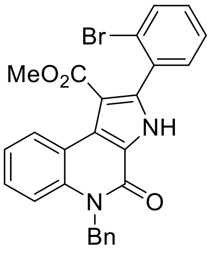

F12

Methyl 5-benzyl-2-(2-bromophenyl)-4-oxo-4,5-dihydro-3*H*-pyrrolo[2,3-*c*]quinoline-1-carboxylate (F12)

PQ **F12** (11 mg, 22 µmol, 90% Yield) was synthesized according to the general procedure 10 from **A12** (12 mg, 24 µmol); column chromatography eluting with cyclohexane/EA 1:1 then DCM/MeOH 20:1.

^{1}H NMR (500 MHz, DMSO-*d6*) δ 13.29 (s, 1H), 9.00 (dd, *J* = 8.1, 1.5 Hz, 1H), 7.76 (dd, *J* = 8.0, 1.0 Hz, 1H), 7.53-7.49 (m, 2H), 7.46 – 7.38 (m, 3H), 7.32 (dd, *J* = 8.7, 6.8 Hz, 2H), 7.29 – 7.21 (m, 4H), 5.68 (s, 2H), 3.53 (s, 3H).

^{13}C NMR (126 MHz, DMSO-*d6*) δ 165.0, 154.5, 143.1, 137.1, 136.1, 133.7, 132.0, 132.0, 130.7, 128.7 (2C), 127.7, 127.2, 127.0, 126.4 (2C), 126.2, 124.6, 123.3, 122.9, 122.0, 117.7, 116.1, 109.8, 51.3, 44.5.

HRMS(ESI): $[M+H]^+$ calcd. $C_{26}H_{20}N_2O_3Br$ m/z 487.0652, found 487.0647.

Synthesis of pyrroquinolines G

General Procedure 11

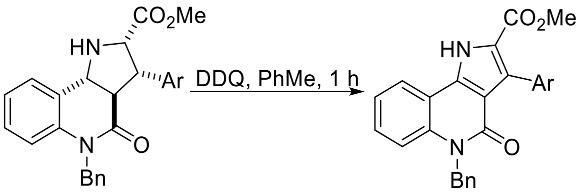

B **G**

PQ **B** (0.05 mmol, 1.0 equiv.) was dissolved in toluene (0.5 mL) and DDQ (0.2 mmol, 4.0 equiv.) was added. The reaction was stirred for 1 h. Sat. $NaHCO_3$ solution (10 mL) was added to quench the reaction and the mixture was extracted with DCM (3*10 mL). The combined organic phases were dried over Na_2SO_4 and filtered. The solvent was removed under reduced pressure and PQs **G** were purified by column chromatography using cyclohexane/EA mixtures.

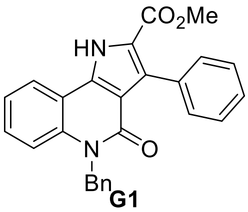

G1

Methyl 5-benzyl-4-oxo-3-phenyl-4,5-dihydro-1*H*-pyrrolo[3,2-*c*]quinoline-2-carboxylate (G1)

PQ **G1** (17 mg, 41 µmol, 82%) was synthesized using the general procedure 11; column chromatography eluting with cyclohexane/EA 10:1 to 2:1.

^{1}H NMR (700 MHz, $CDCl_3$) δ 9.95 (s, 1H), 7.92 (d, J = 7.8 Hz, 1H), 7.54 (d, J = 7.5 Hz, 2H), 7.44 – 7.37 (m, 3H), 7.35 (t, J = 7.4 Hz, 1H), 7.29 (d, J = 8.5 Hz, 1H), 7.28 – 7.22 (m, 4H), 7.22-7.16 (m, 3H), 3.77 (s, 3H).

^{13}C NMR (176 MHz, $CDCl_3$) δ 162.0, 159.6, 138.5, 137.1, 135.7, 132.6, 131.4, 130.8 (2C), 129.8, 128.8 (2C), 127.7, 127.3 (2C), 127.1, 126.6 (2C), 122.1, 121.9, 121.5, 116.6, 113.7, 113.1, 52.1, 45.6.

HRMS(ESI): $[M+H]^+$ calcd. $C_{26}H_{21}N_2O_3$ m/z 409.1547, found 409.1546.

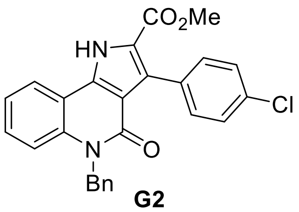

G2

Methyl 5-benzyl-3-(4-chlorophenyl)-4-oxo-4,5-dihydro-1*H*-pyrrolo[3,2-*c*]quinoline-2-carboxylate (G2)

PQ **G2** (20 mg, 50 µmol, quantitative yield) was synthesized using the general procedure 11; column chromatography eluting with cyclohexane/EA 10:1 to 2:1.

^{1}H NMR (400 MHz, $CDCl_3$) δ 10.49 (s, 1H), 8.02 (dd, J = 7.9, 1.5 Hz, 1H), 7.47 – 7.42 (m, 2H), 7.38 (ddd, J = 8.6, 7.1, 1.5 Hz, 1H), 7.29 (dd, J = 8.7, 1.0 Hz, 1H), 7.27 – 7.22 (m, 4H), 7.22 – 7.15 (m, 4H), 5.56 (s, 2H), 3.69 (s, 3H).

^{13}C NMR (101 MHz, $CDCl_3$) δ 161.9, 159.8, 138.4, 137.0, 136.1, 133.6, 132.2 (2C), 131.2, 130.0, 129.8, 128.8 (2C), 127.4 (2C), 127.2, 126.6 (2C), 122.2, 122.1, 122.1, 116.5, 113.4, 113.2, 52.1, 45.7.

HRMS(ESI): $[M+H]^+$ calcd. $C_{26}H_{20}N_2O_3Cl$ m/z 443.1157, found 443.1155.

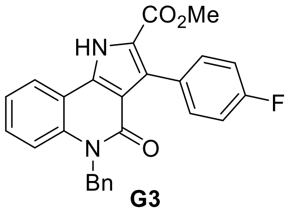

G3

Methyl 5-benzyl-3-(4-fluorophenyl)-4-oxo-4,5-dihydro-1*H*-pyrrolo[3,2-*c*]quinoline-2-carboxylate (G3)

PQ **G3** (13 mg, 31 µmol, 63% Yield) was synthesized using the general procedure 11; column chromatography eluting with cyclohexane/EA 10:1 to 2:1.

^{1}H NMR (500 MHz, CDCl$_3$) δ 10.36 (s, 1H), 8.01 (dd, *J* = 7.8, 1.5 Hz, 1H), 7.57 – 7.44 (m, 2H), 7.39 (ddd, *J* = 8.6, 7.2, 1.5 Hz, 1H), 7.32 – 7.17 (m, 7H), 7.08 – 6.97 (m, 2H), 5.57 (s, 2H), 3.74 (s, 3H).

^{13}C NMR (126 MHz, CDCl$_3$) δ 162.5 (d, *J*CF = 247.0 Hz), 162.0, 159.7, 138.4, 137.0, 136.0, 132.5 (d, *J*CF = 7.6 Hz, 2C), 130.3, 129.8, 128.8 (2C), 128.5 (d, *J*CF = 2.5 Hz), 127.2, 126.5 (2C), 122.2, 122.0, 121.9, 116.5, 114.2 (d, *J*CF = 21.4 Hz, 2C), 113.5, 113.2, 52.1, 45.6.

^{19}F NMR (470 MHz, CDCl$_3$) δ -115.0 (ddd, *J* = 14.4, 8.8, 5.5 Hz, 1F).

HRMS(ESI): $[M+H]^+$ calcd. $C_{26}H_{20}N_2O_3F$ m/z 427.1453, found 427.1451.

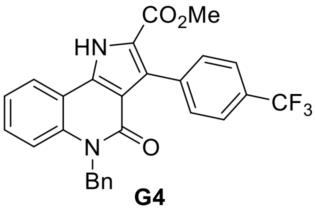

G4

Methyl 5-benzyl-4-oxo-3-(4-(trifluoromethyl)phenyl)-4,5-dihydro-1*H*-pyrrolo[3,2-*c*]quinoline-2-carboxylate (G4)

PQ **G4** (17 mg, 35 µmol, 71% Yield) was synthesized using the general procedure 11; column chromatography eluting with cyclohexane/EA 10:1 to 2:1.

^{1}H NMR (500 MHz, CDCl$_3$) δ 10.51 (s, 1H), 8.04 (dd, *J* = 7.9, 1.5 Hz, 1H), 7.70 – 7.62 (m, 2H), 7.57 (d, *J* = 8.2 Hz, 2H), 7.41 (ddd, *J* = 8.6, 7.2, 1.5 Hz, 1H), 7.31 (dd, *J* = 8.7, 1.0 Hz, 1H), 7.28 – 7.15 (m, 6H), 5.57 (s, 2H), 3.72 (s, 3H).

^{13}C NMR (126 MHz, CDCl$_3$) δ 161.8, 159.7, 138.4, 136.9, 136.6, 136.2, 131.2 (2C), 129.9, 129.6, 129.4 (q, *J*CF = 31.5 Hz), 128.8 (2C), 127.2, 126.5 (2C), 124.5 (q, *J*CF = 272.2 Hz), 124.1 (q, *J*CF = 3.8 Hz, 2C), 122.3, 122.2, 122.1, 116.6, 113.4, 113.1, 52.2, 45.7.

^{19}F NMR (470 MHz, CDCl$_3$) δ -62.4 (s, 3F).

HRMS(ESI): $[M+H]^+$ calcd. $C_{27}H_{20}N_2O_3F_3$ m/z 477.1421, found 477.1416.

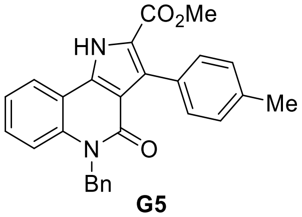

G5

Methyl 5-benzyl-4-oxo-3-(*p*-tolyl)-4,5-dihydro-1*H*-pyrrolo[3,2-*c*]quinoline-2-carboxylate (G5)

PQ **G5** (18 mg, 43 µmol, 87% Yield) was synthesized using the general procedure 11; column chromatography eluting with cyclohexane/EA 10:1 to 2:1.

^{1}H NMR (500 MHz, $CDCl_3$) δ 10.34 (s, 1H), 8.01 (dd, *J* = 7.8, 1.5 Hz, 1H), 7.43 (d, *J* = 8.1 Hz, 2H),, 7.37 (ddd, *J* = 8.6, 7.1, 1.5 Hz, 1H), 7.29 (dd, *J* = 8.7, 1.0 Hz, 1H), 7.27 – 7.22 (m, 2H), 7.21 – 7.16 (m, 4H), 7.15 (d, *J* = 7.8 Hz, 2H), 5.57 (s, 2H), 3.73 (s, 3H), 2.30 (s, 3H).

^{13}C NMR (126 MHz, $CDCl_3$) δ 162.1, 159.7, 138.4, 137.2, 137.2, 135.9, 131.6, 130.6 (2C), 129.6, 129.5, 128.7 (2C), 128.0 (2C), 127.1, 126.6 (2C), 122.1, 122.0, 121.8, 116.4, 113.5, 113.3, 52.0, 45.6, 21.5.

HRMS(ESI): $[M+H]^+$ calcd. $C_{27}H_{23}N_2O_3$ m/z 423.1703, found 423.1702.

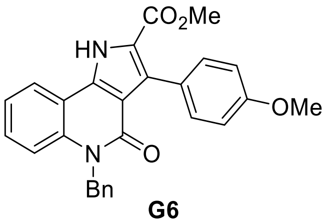

G6

Methyl 5-benzyl-3-(4-methoxyphenyl)-4-oxo-4,5-dihydro-1*H*-pyrrolo[3,2-*c*]quinoline-2-carboxylate (G6)

PQ **G6** (18 mg, 41 µmol, 81% Yield) was synthesized using the general procedure 11; column chromatography eluting with cyclohexane/EA 10:1 to 2:1.

^{1}H NMR (500 MHz, $CDCl_3$) δ 10.38 (s, 1H), 8.02 (dd, *J* = 7.9, 1.5 Hz, 1H), 7.63 – 7.44 (m, 2H), 7.37 (ddd, *J* = 8.6, 7.2, 1.5 Hz, 1H), 7.29 (dd, *J* = 8.6, 1.0 Hz, 1H), 7.28 – 7.23 (m, 2H), 7.22 – 7.16 (m, 4H), 6.99 – 6.71 (m, 2H), 5.58 (s, 2H), 3.75 (s, 3H), 3.73 (s, 3H).

^{13}C NMR (126 MHz, $CDCl_3$) δ 162.1, 159.8, 159.1, 138.3, 137.1, 136.0, 132.1 (2C), 131.3, 129.6, 128.8 (2C), 127.1, 126.6 (2C), 124.7, 122.1, 122.0, 121.8, 116.4, 113.4, 113.3, 112.7 (2C), 55.2, 52.0, 45.6.

HRMS(ESI): $[M+H]^+$ calcd. $C_{27}H_{23}N_2O_4$ m/z 439.1652, found 439.1650.

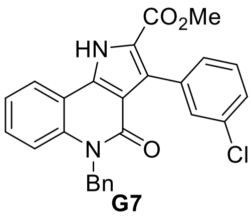

G7

Methyl 5-benzyl-3-(3-chlorophenyl)-4-oxo-4,5-dihydro-1*H*-pyrrolo[3,2-*c*]quinoline-2-carboxylate (G7)

PQ **G7** (16 mg, 36 µmol, 71% Yield) was synthesized using the general procedure 11; column chromatography eluting with cyclohexane/EA 10:1 to 2:1.

^{1}H NMR (400 MHz, $CDCl_3$) δ 10.36 (s, 1H), 7.99 (dd, *J* = 7.9, 1.5 Hz, 1H), 7.51-7.50 (m, 1H), 7.44 – 7.40 (m, 1H), 7.40 – 7.36 (m, 1H), 7.32 – 7.16 (m, 9H), 5.57 (s, 2H), 3.75 (s, 3H).

^{13}C NMR (101 MHz, $CDCl_3$) δ 161.9, 159.6, 138.4, 137.0, 136.0, 134.6, 132.9, 130.9, 129.8, 129.6, 129.1, 128.8 (2C), 128.4, 127.7, 127.2, 126.6 (2C), 122.3, 121.9, 116.6, 113.5, 113.1, 52.2, 45.6.

HRMS(ESI): $[M+H]^+$ calcd. $C_{26}H_{20}N_2O_3Cl$ m/z 443.1157, found 443.1156.

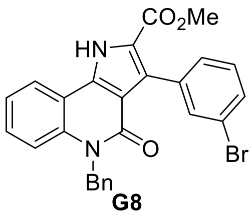

G8

Methyl 5-benzyl-3-(3-bromophenyl)-4-oxo-4,5-dihydro-1*H*-pyrrolo[3,2-*c*]quinoline-2-carboxylate (G8)

PQ **G8** (24 mg, 49 µmol, 97% Yield) was synthesized using the general procedure 11; column chromatography eluting with cyclohexane/EA 10:1 to 2:1.

^{1}H NMR (500 MHz, CDCl$_3$) δ 10.44 (s, 1H), 8.01 (dd, *J* = 8.0, 1.5 Hz, 1H), 7.67 (t, *J* = 1.8 Hz, 1H), 7.48 (dt, *J* = 7.7, 1.3 Hz, 1H), 7.42 – 7.36 (m, 2H), 7.31 – 7.16 (m, 8H), 5.57 (s, 2H), 3.74 (s, 3H).

^{13}C NMR (126 MHz, CDCl$_3$) δ 161.9, 159.6, 138.4, 137.0, 136.0, 134.9, 133.7, 130.5, 129.8, 129.6, 129.4, 128.8 (2C), 128.6, 127.2, 126.6 (2C), 122.3, 122.2, 122.0, 121.1, 116.5, 113.4, 113.2, 52.2, 45.6.

HRMS(ESI): $[M+H]^+$ calcd. $C_{26}H_{20}N_2O_3Br$ m/z 487.0652, found 487.0648.

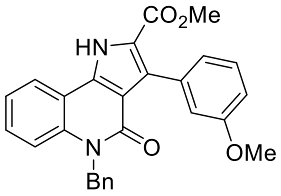

G9

Methyl 5-benzyl-3-(3-methoxyphenyl)-4-oxo-4,5-dihydro-1*H*-pyrrolo[3,2-*c*]quinoline-2-carboxylate (G9)

PQ **G9** (36 mg, 37 µmol, 74% Yield) was synthesized using the general procedure 11; column chromatography eluting with cyclohexane/EA 10:1 to 2:1.

^{1}H NMR (500 MHz, CDCl$_3$) δ 10.29 (s, 1H), 7.99 (dd, *J* = 7.9, 1.4 Hz, 1H), 7.38 (ddd, *J* = 8.6, 7.2, 1.5 Hz, 1H), 7.30 – 7.23 (m, 4H), 7.22 – 7.16 (m, 4H), 7.12 (dt, *J* = 7.5, 1.2 Hz, 1H), 7.07 (dd, *J* = 2.6, 1.5 Hz, 1H), 6.84 (dd, *J* = 8.3, 2.7 Hz, 1H), 5.57 (s, 2H), 3.76 (s, 3H), 3.74 (s, 3H).

^{13}C NMR (126 MHz, CDCl$_3$) δ 162.1, 159.6, 158.5, 138.4, 137.1, 135.9, 134.0, 131.0, 129.6, 128.8 (2C), 128.1, 127.1, 126.6 (2C), 123.3, 122.1, 122.1, 121.9, 116.7, 116.5, 113.6, 113.2, 112.9, 55.2, 52.1, 45.6.

HRMS(ESI): $[M+H]^+$ calcd. $C_{27}H_{23}N_2O_4$ m/z 439.1652, found 439.1651.

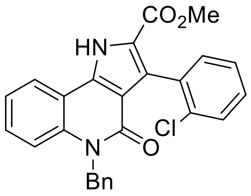

G10

Methyl 5-benzyl-3-(2-chlorophenyl)-4-oxo-4,5-dihydro-1*H*-pyrrolo[3,2-*c*]quinoline-2-carboxylate (G10)

PQ **G10** (18 mg, 41 µmol, 82% Yield) was synthesized using the general procedure 11; column chromatography eluting with cyclohexane/EA 10:1 to 2:1.

^{1}H NMR (400 MHz, CDCl$_3$) δ 10.68 (s, 1H), 8.05 (d, *J* = 7.8 Hz, 1H), 7.43 (dd, *J* = 7.4, 1.9 Hz, 1H), 7.40-7.34 (m, 2H), 7.31 – 7.16 (m, 9H), 5.57 (s, 2H), 3.70 (s, 3H).

^{13}C NMR (101 MHz, CDCl$_3$) δ 162.0, 159.6, 138.3, 137.1, 136.1, 134.1, 132.9, 131.9, 129.6, 128.9, 128.8, 128.8 (2C), 127.4, 127.2, 126.6 (2C), 125.9, 122.8, 122.3, 122.1, 116.4, 114.1, 113.5, 52.2, 45.6.

HRMS(ESI): $[M+H]^+$ calcd. $C_{26}H_{20}N_2O_3Cl$ m/z 443.1157, found 443.1156.

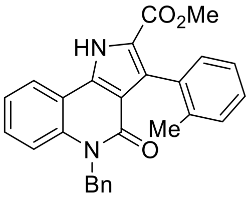

G11

Methyl 5-benzyl-4-oxo-3-(*o*-tolyl)-4,5-dihydro-1*H*-pyrrolo[3,2-*c*]quinoline-2-carboxylate (G11)

PQ **G11** (16 mg, 39 µmol, 77% Yield) was synthesized using the general procedure 11; column chromatography eluting with cyclohexane/EA 10:1 to 2:1.

^{1}H NMR (400 MHz, CDCl$_3$) δ 10.52 (s, 1H), 8.04 (dd, *J* = 7.9, 1.5 Hz, 1H), 7.38 (ddd, *J* = 8.6, 7.1, 1.5 Hz, 1H), 7.32 – 7.23 (m, 4H), 7.21 – 7.12 (m, 7H), 5.57 (s, 2H), 3.69 (s, 3H), 2.20 (s, 3H).

^{13}C NMR (101 MHz, CDCl$_3$) δ 162.3, 159.6, 138.4, 137.2, 136.7, 136.1, 133.3, 130.5, 130.0, 129.5, 129.4, 128.8 (2C), 127.6, 127.1, 126.6 (2C), 124.9, 122.2, 122.1, 122.0, 116.4, 114.2, 113.5, 52.1, 45.5, 20.5.

HRMS(ESI): $[M+H]^+$ calcd. $C_{27}H_{23}N_2O_3$ m/z 423.1703, found 423.1702.

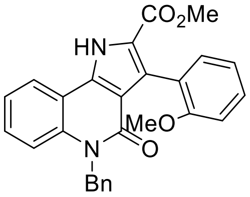

G12

Methyl 5-benzyl-3-(2-methoxyphenyl)-4-oxo-4,5-dihydro-1*H*-pyrrolo[3,2-*c*]quinoline-2-carboxylate (G12)

PQ **G12** (14 mg, 32 µmol, 65% Yield) was synthesized using the general procedure 11; column chromatography eluting with cyclohexane/EA 10:1 to 1:1.

^{1}H NMR (400 MHz, CDCl$_3$) δ 10.36 (s, 1H), 7.97 (dd, *J* = 7.9, 1.5 Hz, 1H), 7.39 (dd, *J* = 7.5, 1.8 Hz, 1H), 7.36 – 7.32 (m, 1H), 7.31 – 7.22 (m, 4H), 7.21 – 7.13 (m, 4H), 6.98 (td, *J* = 7.5, 1.1 Hz, 1H), 6.93 (d, *J* = 8.3 Hz, 1H), 5.53 (s, 2H), 3.76 (s, 3H), 3.71 (s, 3H).

^{13}C NMR (101 MHz, CDCl$_3$) δ 162.3, 159.5, 157.5, 138.4, 137.3, 136.0, 131.9, 129.3, 129.1, 128.7 (2C), 127.1, 126.9, 126.6 (2C), 122.6, 122.4, 121.9, 121.9, 119.9, 116.3, 114.3, 113.5, 110.6, 55.7, 52.0, 45.4.

HRMS(ESI): $[M+H]^+$ calcd. $C_{27}H_{23}N_2O_4$ m/z 439.1652, found 439.1651.

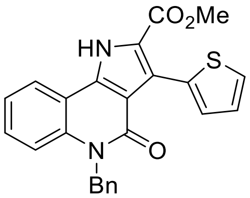

G13

Methyl 5-benzyl-4-oxo-3-(thiophen-2-yl)-4,5-dihydro-1*H*-pyrrolo[3,2-*c*]quinoline-2-carboxylate (G13)

PQ **G13** (12 mg, 29 µmol, 58% Yield) was synthesized using the general procedure 11; column chromatography eluting with cyclohexane/EA 10:1 to 2:1.

^{1}H NMR (400 MHz, CDCl$_3$) δ 10.39 (s, 1H), 7.99 (dd, *J* = 7.9, 1.5 Hz, 1H), 7.42 – 7.35 (m, 2H), 7.29 (dd, *J* = 5.1, 3.8 Hz, 2H), 7.25 – 7.16 (m, 6H), 7.05 (dd, *J* = 5.1, 3.5 Hz, 1H), 5.57 (s, 2H), 3.80 (s, 3H).

^{13}C NMR (101 MHz, CDCl$_3$) δ 161.8, 159.4, 138.4, 137.0, 136.0, 132.4, 129.8, 129.6, 128.8 (2C), 127.2, 126.6 (2C), 126.4, 126.3, 123.2, 123.1, 122.2, 121.9, 116.5, 114.1, 113.0, 52.2, 45.6.

HRMS(ESI): $[M+H]^+$ calcd. $C_{24}H_{19}N_2O_3S$ m/z 415.1111, found 415.1110.

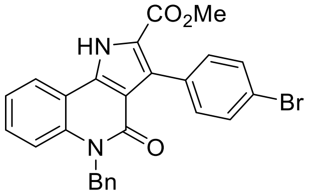

G14

Methyl 5-benzyl-3-(4-bromophenyl)-4-oxo-4,5-dihydro-1*H*-pyrrolo[3,2-*c*]quinoline-2-carboxylate (G14)

PQ **G14** (18 mg, 37 µmol, 75% Yield) was synthesized using the general procedure 11; column chromatography eluting with cyclohexane/EA 10:1 to 2:1.

^{1}H NMR (500 MHz, CDCl$_3$) δ 10.52 (s, 1H), 8.04 (dd, *J* = 7.9, 1.5 Hz, 1H), 7.43 – 7.37 (m, 5H), 7.31 (d, *J* = 8.7 Hz, 1H), 7.28 – 7.24 (m, 2H), 7.23 – 7.17 (m, 4H), 5.57 (s, 2H), 3.70 (s, 3H).

^{13}C NMR (126 MHz, CDCl$_3$) δ 161.9, 159.8, 138.4, 137.0, 136.2, 132.5 (2C), 131.7, 130.3 (2C), 129.9, 129.8, 128.8 (2C), 127.2, 126.6 (2C), 122.2, 122.2, 122.0, 121.9, 116.5, 113.4, 113.2, 52.1, 45.7.

HRMS(ESI): $[M+H]^+$ calcd. $C_{26}H_{20}N_2O_3Br$ m/z 487.0652, found 487.0648.

Synthesis of pyrroquinolines H

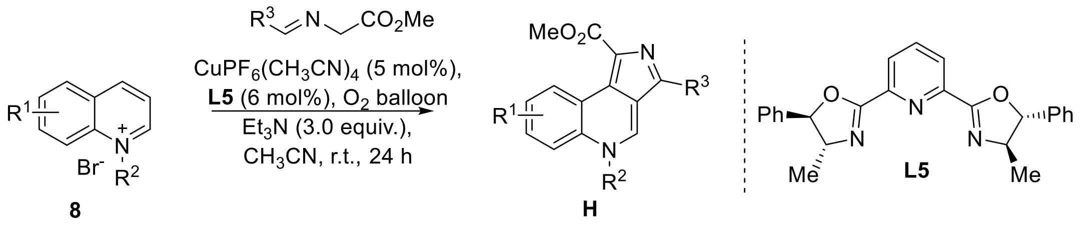

General Procedure 12

To a solution of quinolinium salt **8**[11] (0.1 mmol, 1.0 equiv.) and the iminoester[9] (0.15 mmol, 1.5 equiv.) in CH_3CN (1.0 mL) was added $Cu(CH_3CN)_4PF_6$ (0.005 mmol, 0.05 equiv.), TEA (0.3 mmol, 3.0 equiv.) and ligand **L5** (0.005 mmol, 0.05 equiv.) in one portion. The reaction vessel was three times evacuated and refilled with O_2. The reaction was stirred for 24 h under an O_2 atmosphere. Then 20% KOH solution (20 mL) was added to quench the reaction and the mixture was extracted with EA (3*50 mL). The combined organic phases were dried over Na_2SO_4 and filtered. The solvent was removed under reduced pressure and PQs **H** were purified by column chromatography using cyclohexane/acetone.

General Procedure 13

To a solution of quinolinium salt **8** (0.1 mmol, 1.0 equiv.) and iminoester (0.15 mmol, 1.5 equiv.) in CH_3CN (1.0 mL) was added $Cu(CH_3CN)_4PF_6$ (0.015 mmol, 0.15 equiv.), TEA (0.3 mmol, 3.0 equiv.) and 10% Pd/C (30 mg) in one portion. The reaction was stirred for 24 h and then filtered through a short pad of Celite. Then 20% KOH solution (20 mL) was added to the filtrate and the mixture was extracted with EA (3*50 mL). The combined organic phases were dried over Na_2SO_4 and filtered. The solvent was removed under reduced pressure and PQs **H** were purified by column chromatography using cyclohexane/acetone.

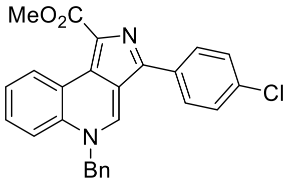

H1

Methyl 5-benzyl-3-(4-chlorophenyl)-5*H*-pyrrolo[3,4-*c*]quinoline-1-carboxylate (H1)

PQ **H1** (38 mg, 89 µmol, 89%) was synthesized according to the general procedure 12; column chromatography eluting with cyclohexane/acetone 2:1 to 1:2.

^{1}H NMR (500 MHz, $CDCl_3$) δ 10.08 (dd, *J* = 8.4, 1.5 Hz, 1H), 8.58 (s, 1H), 7.81 – 7.67 (m, 2H), 7.58 (ddd, *J* = 8.3, 6.9, 1.2 Hz, 1H), 7.54 (d, *J* = 8.8 Hz, 1H), 7.44 (ddd, *J* = 8.6, 7.0, 1.6 Hz, 1H), 7.35 (m, 5H), 7.15 – 7.02 (m, 2H), 5.68 (s, 2H), 4.06 (s, 3H).

^{13}C NMR (126 MHz, $CDCl_3$) δ 166.2, 146.5, 141.3, 134.1, 133.8 (2C), 132.5, 129.9, 129.6 (2C), 129.4 (2C), 129.0 (2C), 129.0, 128.3, 128.1, 127.4, 127.1, 126.3 (2C), 123.5, 119.3, 116.9, 59.5, 52.0.

HRMS(ESI): $[M+H]^+$ calcd. $C_{26}H_{20}N_2O_2Cl$ m/z 427.1208, found 427.1204.

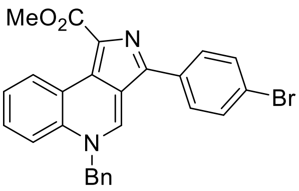

H2

Methyl 5-benzyl-3-(4-bromophenyl)-5*H*-pyrrolo[3,4-*c*]quinoline-1-carboxylate (H2)

PQ **H2** (37 mg, 79 µmol, 79% Yield) was synthesized using the general procedure 12; column chromatography eluting with cyclohexane/acetone 2:1 to 1:2.

^{1}H NMR (500 MHz, CDCl$_3$) δ 10.07 (dd, *J* = 8.4, 1.5 Hz, 1H), 8.57 (s, 1H), 7.66 – 7.61 (m, 2H), 7.57 (ddd, *J* = 8.3, 6.9, 1.2 Hz, 1H), 7.55 – 7.47 (m, 3H), 7.43 (ddd, *J* = 8.6, 6.9, 1.5 Hz, 1H), 7.38 – 7.32 (m, 3H), 7.15 – 7.03 (m, 2H), 5.67 (s, 2H), 4.05 (s, 3H).

^{13}C NMR (126 MHz, CDCl$_3$) δ 166.2, 146.5, 141.3, 134.5, 133.8, 132.5, 132.0 (2C), 129.8, 129.6 (2C), 129.6 (2C), 129.0, 128.3, 128.1, 127.4, 127.1, 126.3 (2C), 123.5, 122.1, 119.2, 116.9, 59.5, 52.0.

HRMS(ESI): $[M+H]^+$ calcd. $C_{26}H_{20}N_2O_2Br$ m/z 471.0703, found 471.0693.

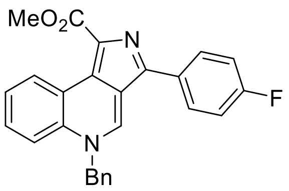

H3

Methyl 5-benzyl-3-(4-fluorophenyl)-5*H*-pyrrolo[3,4-*c*]quinoline-1-carboxylate (H3)

PQ **H3** (36 mg, 87 µmol, 87% Yield) was synthesized using the general procedure 12; column chromatography eluting with cyclohexane/acetone 2:1 to 1:2.

^{1}H NMR (500 MHz, CDCl$_3$) δ 10.08 (dd, *J* = 8.4, 1.6 Hz, 1H), 8.55 (s, 1H), 7.80 – 7.69 (m, 2H), 7.57 (ddd, *J* = 8.2, 7.0, 1.2 Hz, 1H), 7.51 (d, *J* = 8.6 Hz, 1H), 7.46 – 7.39 (m, 1H), 7.38 – 7.29 (m, 3H), 7.20 – 7.01 (m, 4H), 5.66 (s, 2H), 4.06 (s, 3H).

^{13}C NMR (126 MHz, CDCl$_3$) δ 166.2, 162.8 (d, *J*CF = 248.2 Hz), 147.0, 141.4, 133.9, 132.5, 131.8 (d, *J*CF = 3.8 Hz), 129.9 (d, *J*CF = 7.6 Hz, 2C), 129.9, 129.6 (2C), 128.9, 128.2, 127.8, 127.2, 127.0, 126.3 (2C), 123.5, 119.3, 116.9, 115.8 (d, *J*CF = 22.7 Hz, 2C), 59.5, 52.0.

^{19}F NMR (470 MHz, CDCl$_3$) δ -114.0 (m, 1F).

HRMS(ESI): $[M+H]^+$ calcd. $C_{26}H_{20}N_2O_2F$ m/z 411.1503, found 411.1493.

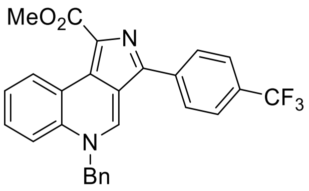

H4

Methyl 5-benzyl-3-(4-(trifluoromethyl)phenyl)-5*H*-pyrrolo[3,4-*c*]quinoline-1-carboxylate (H4)

PQ **H4** (39 mg, 86 µmol, 86% Yield) was synthesized using the general procedure 12; column chromatography eluting with cyclohexane/acetone 2:1 to 1:2.

^{1}H NMR (500 MHz, CDCl$_3$) δ 10.06 (dd, *J* = 8.4, 1.5 Hz, 1H), 8.63 (s, 1H), 7.99 – 7.81 (m, 2H), 7.65 – 7.53 (m, 4H), 7.51 – 7.40 (m, 1H), 7.37-7.32 (m, 3H), 7.15 – 7.04 (m, 2H), 5.70 (s, 2H), 4.06 (s, 3H).

^{13}C NMR (126 MHz, CDCl$_3$) δ 166.2, 145.8, 141.3, 139.0, 133.7, 132.5, 129.8, 129.6 (2C), 129.5 (q, *J*CF = 32.8 Hz), 129.1, 129.0, 128.4, 128.2 (2C), 127.6, 127.2, 126.3 (2C), 125.8 (q, *J*CF = 3.8 Hz, 2C), 124.3 (q, *J*CF = 272.2 Hz), 123.5, 119.3, 116.9, 59.6, 52.1.

^{19}F NMR (470 MHz, CDCl$_3$) δ -62.4 (s, 3F).

HRMS(ESI): $[M+H]^+$ calcd. $C_{27}H_{20}N_2O_2F_3$ m/z 461.1471, found 461.1458.

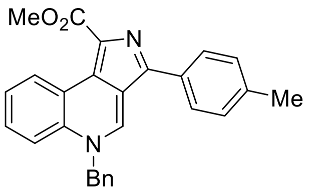

H5

Methyl 5-benzyl-3-(*p*-tolyl)-5*H*-pyrrolo[3,4-*c*]quinoline-1-carboxylate (H5)

PQ **H5** (32 mg, 78 µmol, 78% Yield) was synthesized using the general procedure 12; column chromatography eluting with cyclohexane/acetone 2:1 to 1:2.

^{1}H NMR (700 MHz, CDCl$_3$) δ 10.06 (dd, *J* = 8.3, 1.5 Hz, 1H), 8.57 (s, 1H), 7.68 (d, *J* = 7.9 Hz, 2H), 7.55 (ddd, *J* = 8.2, 6.9, 1.2 Hz, 1H), 7.49 (d, *J* = 8.6 Hz, 1H), 7.39 (ddd, *J* = 8.5, 7.0, 1.5 Hz, 1H), 7.34 – 7.29 (m, 3H), 7.19 (d, *J* = 7.8 Hz, 2H), 7.07 (d, *J* = 7.7 Hz, 2H), 5.63 (s, 2H), 4.06 (s, 3H), 2.36 (s, 3H).

^{13}C NMR (176 MHz, CDCl$_3$) δ 166.3, 148.2, 141.7, 137.8, 134.1, 132.7, 132.6, 129.8, 129.6 (2C), 129.5 (2C), 128.8, 128.2 (2C), 127.7, 127.1, 126.9, 126.2 (2C), 123.6, 119.5, 116.8, 59.4, 51.9, 21.4.

HRMS(ESI): $[M+H]^+$ calcd. $C_{27}H_{23}N_2O_2$ m/z 407.1754, found 407.1746.

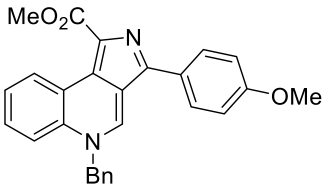

H6

Methyl 5-benzyl-3-(4-methoxyphenyl)-5*H*-pyrrolo[3,4-*c*]quinoline-1-carboxylate (H6)

PQ **H6** (28 mg, 65 µmol, 65% Yield) was synthesized using the general procedure 12; column chromatography eluting with cyclohexane/acetone 2:1 to 1:3.

^{1}H NMR (600 MHz, CDCl$_3$) δ 10.05 (dd, *J* = 8.4, 1.5 Hz, 1H), 8.61 (s, 1H), 7.75 – 7.65 (m, 2H), 7.62 – 7.51 (m, 2H), 7.44 (ddd, *J* = 8.6, 7.0, 1.5 Hz, 1H), 7.36 – 7.28 (m, 3H), 7.12 – 7.05 (m, 2H), 6.93 – 6.85 (m, 2H), 5.68 (s, 2H), 4.03 (s, 3H), 3.78 (s, 3H).

^{13}C NMR (151 MHz, CDCl$_3$) δ 165.8, 159.8, 147.6, 142.3, 134.1, 132.7, 129.9, 129.6 (2C), 129.5 (2C), 128.8, 128.3, 127.6, 127.0, 126.9, 126.8, 126.2 (2C), 123.6, 119.1, 117.0, 114.3 (2C), 59.5, 55.4, 51.9.

HRMS(ESI): $[M+H]^+$ calcd. $C_{27}H_{23}N_2O_3$ m/z 423.1703, found 423.1700.

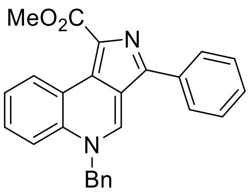

H7

Methyl 5-benzyl-3-phenyl-5*H*-pyrrolo[3,4-*c*]quinoline-1-carboxylate (H7)

PQ **H7** (26 mg, 66 μmol, 66% Yield) was synthesized using the general procedure 12; column chromatography eluting with cyclohexane/acetone 2:1 to 1:2.

^{1}H NMR (500 MHz, CDCl$_3$) δ 10.09 (dd, *J* = 8.4, 1.5 Hz, 1H), 8.64 (s, 1H), 7.85 – 7.74 (m, 2H), 7.58 (ddd, *J* = 8.2, 6.9, 1.2 Hz, 1H), 7.53 (dd, *J* = 8.6, 1.2 Hz, 1H), 7.46 – 7.38 (m, 3H), 7.37 – 7.30 (m, 4H), 7.09-7.07 (m, 2H), 5.67 (s, 2H), 4.06 (s, 3H).

^{13}C NMR (126 MHz, CDCl$_3$) δ 166.2, 148.0, 141.8, 135.4, 134.0, 132.6, 129.9, 129.6 (2C), 128.9 (2C), 128.9, 128.4 (2C), 128.2, 128.0, 127.8, 127.2, 127.0, 126.2 (2C), 123.6, 119.4, 116.9, 59.5, 52.0.

HRMS(ESI): $[M+H]^+$ calcd. $C_{26}H_{21}N_2O_2$ m/z 393.1598, found 393.1589.

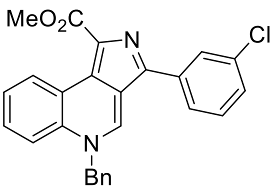

H8

Methyl 5-benzyl-3-(3-chlorophenyl)-5*H*-pyrrolo[3,4-*c*]quinoline-1-carboxylate (H8)

PQ **H8** (26 mg, 61 μmol, 61% Yield) was synthesized using the general procedure 12; column chromatography eluting with cyclohexane/acetone 2:1 to 1:2.

^{1}H NMR (500 MHz, CDCl$_3$) δ 10.10 (dd, *J* = 8.4, 1.5 Hz, 1H), 8.62 (s, 1H), 7.81 (t, *J* = 1.8 Hz, 1H), 7.65 – 7.55 (m, 3H), 7.46 (ddd, *J* = 8.6, 7.0, 1.5 Hz, 1H), 7.40 – 7.34 (m, 3H), 7.33 – 7.26 (m, 2H), 7.11 (dd, *J* = 7.6, 2.0 Hz, 2H), 5.71 (s, 2H), 4.07 (s, 3H).

^{13}C NMR (126 MHz, CDCl$_3$) δ 166.2, 146.1, 141.3, 137.3, 134.8, 133.7, 132.6, 130.1, 129.9, 129.7 (2C), 129.1, 128.4, 128.1, 128.1, 127.8, 127.4, 127.2, 126.5 (2C), 126.3, 123.6, 119.2, 116.9, 59.6, 52.1.

HRMS(ESI): $[M+H]^+$ calcd. $C_{26}H_{20}N_2O_2Cl$ m/z 427.1208, found 427.1203.

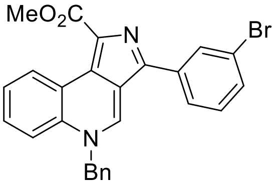

H9

Methyl 5-benzyl-3-(3-bromophenyl)-5*H*-pyrrolo[3,4-*c*]quinoline-1-carboxylate (H9)

PQ **H9** (37 mg, 78 μmol, 78% Yield) was synthesized using the general procedure 12; column chromatography eluting with cyclohexane/acetone 2:1 to 1:2.

^{1}H NMR (500 MHz, CDCl$_3$) δ 10.09 (dd, *J* = 8.4, 1.4 Hz, 1H), 8.59 (s, 1H), 7.98 (t, *J* = 1.8 Hz, 1H), 7.65 (dt, *J* = 7.7, 1.4 Hz, 1H), 7.62 – 7.54 (m, 2H), 7.45 (ddd, *J* = 8.6, 6.9, 1.5 Hz, 1H), 7.41 (d, *J* = 8.0 Hz, 1H), 7.38 – 7.32 (m, 3H), 7.23 (t, *J* = 7.8 Hz, 1H), 7.11 (dd, *J* = 7.4, 2.2 Hz, 2H), 5.70 (s, 2H), 4.06 (s, 3H).

^{13}C NMR (126 MHz, CDCl$_3$) δ 166.2, 145.9, 141.2, 137.6, 133.7, 132.6, 130.9, 130.7, 130.3, 129.9, 129.7 (2C), 129.1, 128.3, 128.1, 127.4, 127.2, 126.7, 126.5 (2C), 123.5, 123.1, 119.2, 116.9, 59.5, 52.1.

HRMS(ESI): [M+H]$^+$ calcd. $C_{26}H_{20}N_2O_2Br$ m/z 471.0703, found 471.0696.

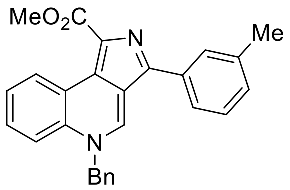

H10

Methyl 5-benzyl-3-(*m*-tolyl)-5*H*-pyrrolo[3,4-*c*]quinoline-1-carboxylate (H10)

PQ **H10** (33 mg, 80 µmol, 80% Yield) was synthesized using the general procedure 12; column chromatography eluting with cyclohexane/acetone 2:1 to 1:2.

^{1}H NMR (500 MHz, DMSO-*d6*) δ 9.97 (dd, *J* = 8.2, 1.6 Hz, 1H), 9.66 (s, 1H), 7.95 (d, *J* = 8.6 Hz, 1H), 7.86-7.80 (m, 2H), 7.67-7.58 (m, 2H), 7.39 (t, *J* = 7.5 Hz, 1H), 7.37 – 7.32 (m, 2H), 7.31 – 7.24 (m, 3H), 7.23 – 7.18 (m, 1H), 6.13 (s, 2H), 3.89 (s, 3H), 2.43 (s, 3H).

^{13}C NMR (126 MHz, DMSO-*d6*) δ 165.7, 146.1, 144.1, 137.9, 135.8, 135.7, 132.1, 129.0 (2C), 128.6, 128.4, 128.3, 128.2, 128.0, 127.9, 126.7, 126.5, 126.5 (2C), 126.5, 125.0, 122.9, 118.4, 118.3, 57.9, 51.0, 21.2.

HRMS(ESI): [M+H]$^+$ calcd. $C_{27}H_{23}N_2O_2$ m/z 407.1754, found 407.1750.

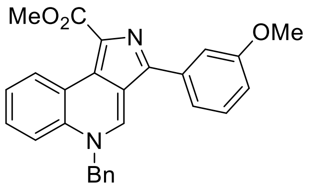

H11

Methyl 5-benzyl-3-(3-methoxyphenyl)-5*H*-pyrrolo[3,4-*c*]quinoline-1-carboxylate (H11)

PQ **H11** (31 mg, 73 µmol, 73% Yield) was synthesized using the general procedure 12; column chromatography eluting with cyclohexane/acetone 2:1 to 1:3.

^{1}H NMR (500 MHz, CDCl$_3$) δ 10.11 (dd, *J* = 8.2, 4.5 Hz, 1H), 8.81 – 8.59 (m, 1H), 7.63 – 7.52 (m, 2H), 7.47 – 7.41 (m, 2H), 7.37 – 7.27 (m, 5H), 7.11-7.06 (m, 2H), 6.90-6.87 (m, 1H), 5.68 (s, 2H), 4.06 (s, 3H), 3.86 (s, 3H).

^{13}C NMR (126 MHz, CDCl$_3$) δ 166.2, 160.1, 147.8, 141.9, 136.9, 133.9, 132.6, 129.9, 129.8, 129.6 (2C), 128.9, 128.2, 127.3, 127.0, 126.2 (2C), 123.6, 123.6, 120.8, 119.5, 116.9, 114.1, 113.4, 59.5, 55.6, 51.9.

HRMS(ESI): [M+H]$^+$ calcd. $C_{27}H_{23}N_2O_3$ m/z 423.1703, found 423.1695.

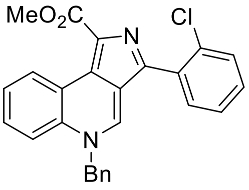

H12

Methyl 5-benzyl-3-(2-chlorophenyl)-5*H*-pyrrolo[3,4-*c*]quinoline-1-carboxylate (H12)

PQ **H12** (28 mg, 66 µmol, 66% Yield) was synthesized using the general procedure 12; column chromatography eluting with cyclohexane/acetone 2:1 to 1:2.

^{1}H NMR (500 MHz, $CDCl_3$) δ 10.21 (dd, *J* = 8.7, 1.5 Hz, 1H), 8.64 (s, 1H), 7.78 – 7.72 (m, 1H), 7.69 – 7.64 (m, 2H), 7.55 – 7.49 (m, 1H), 7.49 – 7.43 (m, 1H), 7.39 – 7.29 (m, 5H), 7.13 (dd, *J* = 7.7, 1.8 Hz, 2H), 5.72 (s, 2H), 4.05 (s, 3H).

^{13}C NMR (126 MHz, $CDCl_3$) δ 166.1, 145.4, 142.8, 134.7, 133.9, 133.3, 132.9, 132.5, 130.2, 129.9, 129.6 (2C), 129.6, 129.0, 128.3, 128.1, 127.3, 127.3, 127.2, 126.2 (2C), 124.0, 119.8, 117.1, 59.8, 52.0.

HRMS(ESI): $[M+H]^+$ calcd. $C_{26}H_{20}N_2O_2Cl$ m/z 427.1208, found 427.1198.

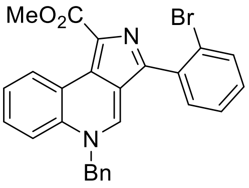

H13

Methyl 5-benzyl-3-(2-bromophenyl)-5*H*-pyrrolo[3,4-*c*]quinoline-1-carboxylate (H13)

PQ **H13** (29 mg, 62 µmol, 62% Yield) was synthesized using the general procedure 12; column chromatography eluting with cyclohexane/acetone 2:1 to 1:2.

^{1}H NMR (500 MHz, DMSO-*d6*) δ 10.00 (dd, *J* = 8.2, 1.6 Hz, 1H), 9.39 (s, 1H), 7.99 (dd, *J* = 8.7, 1.3 Hz, 1H), 7.81 (d, *J* = 8.1 Hz, 1H), 7.72 – 7.60 (m, 3H), 7.54 (t, *J* = 7.5 Hz, 1H), 7.44 – 7.39 (m, 1H), 7.36 – 7.31 (m, 2H), 7.30 – 7.25 (m, 3H), 6.06 (s, 2H), 3.86 (s, 3H).

^{13}C NMR (126 MHz, DMSO-*d6*) δ 165.6, 145.8, 144.4, 136.8, 135.7, 133.0, 132.9, 132.0, 129.8, 129.0 (2C), 128.5, 128.0, 128.0, 127.7, 126.7, 126.5 (2C), 126.0, 123.0, 123.0, 118.9, 118.5, 57.7, 50.9.

HRMS(ESI): $[M+H]^+$ calcd. $C_{26}H_{20}N_2O_2Br$ m/z 471.0703, found 471.0694.

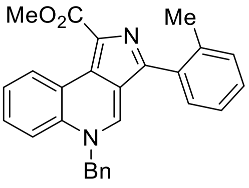

H14

Methyl 5-benzyl-3-(*o*-tolyl)-5*H*-pyrrolo[3,4-*c*]quinoline-1-carboxylate (H14)

PQ **H14** (29 mg, 70 µmol, 70% Yield) was synthesized using the general procedure 12; column chromatography eluting with cyclohexane/acetone 2:1 to 1:2.

^{1}H NMR (500 MHz, $CDCl_3$) δ 10.18 (dt, *J* = 8.4, 1.7 Hz, 1H), 8.42 (s, 1H), 7.67 – 7.58 (m, 2H), 7.49 (t, *J* = 8.2 Hz, 1H), 7.42 (d, *J* = 8.0 Hz, 1H), 7.36 – 7.27 (m, 5H), 7.25 – 7.21 (m, 1H), 7.08 (d, J = 7.3 Hz, 2H), 5.68 (s, 2H), 4.04 (s, 3H), 2.39 (s, 3H).

^{13}C NMR (126 MHz, CDCl$_3$) δ 166.3, 148.7, 141.8, 137.8, 134.7, 134.1, 132.7, 130.8, 130.8, 130.1, 129.6 (2C), 128.8, 128.2, 127.5, 127.1, 126.3, 126.0 (2C), 125.6, 123.8, 120.9, 117.0, 59.5, 51.9, 20.8.

HRMS(ESI): [M+H]$^+$ calcd. $C_{27}H_{23}N_2O_2$ m/z 407.1754, found 407.1742.

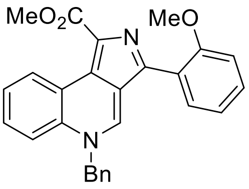

H15

Methyl 5-benzyl-3-(2-methoxyphenyl)-5*H*-pyrrolo[3,4-*c*]quinoline-1-carboxylate (H15)

PQ **H15** (20 mg, 46 µmol, 46% Yield) was synthesized using the general procedure 12; column chromatography eluting with cyclohexane/acetone 2:1 to 1:3.

^{1}H NMR (700 MHz, CDCl$_3$) δ 10.18 (dd, *J* = 8.3, 1.5 Hz, 1H), 8.87 (s, 1H), 7.92 (dd, *J* = 7.5, 1.8 Hz, 1H), 7.67-7.62 (m, 2H), 7.53 – 7.48 (m, 1H), 7.38 – 7.31 (m, 4H), 7.19 – 7.14 (m, 2H), 7.11 (t, *J* = 7.5 Hz, 1H), 6.99 (d, *J* = 8.3 Hz, 1H), 5.72 (s, 2H), 4.05 (s, 3H), 3.70 (s, 3H).

^{13}C NMR (176 MHz, CDCl$_3$) δ 166.1, 156.6, 145.0, 143.8, 134.3, 132.6, 132.5, 130.2, 129.6, 129.6 (2C), 128.9, 128.2, 127.7, 127.4, 127.0, 126.3 (2C), 125.0, 124.0, 121.8, 120.1, 116.9, 111.9, 59.5, 56.1, 51.9.

HRMS(ESI): [M+H]$^+$ calcd. $C_{27}H_{23}N_2O_3$ m/z 423.1703, found 423.1698.

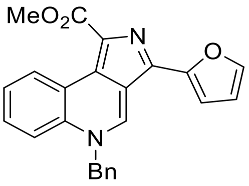

H16

Methyl 5-benzyl-3-(furan-2-yl)-5*H*-pyrrolo[3,4-*c*]quinoline-1-carboxylate (H16)

PQ **H16** (17 mg, 45 µmol, 45% Yield) was synthesized using the general procedure 12; column chromatography eluting with cyclohexane/acetone 2:1 to 1:2.

^{1}H NMR (600 MHz, CDCl$_3$) δ 10.09 (dd, *J* = 8.3, 1.5 Hz, 1H), 9.16 (s, 1H), 7.64 – 7.51 (m, 2H), 7.51 – 7.39 (m, 2H), 7.38 – 7.30 (m, 3H), 7.21 – 7.09 (m, 3H), 6.54 (dd, *J* = 3.4, 1.8 Hz, 1H), 5.73 (s, 2H), 4.06 (s, 3H).

^{13}C NMR (151 MHz, CDCl$_3$) δ 165.9, 152.2, 143.0, 141.9, 138.5, 134.0, 132.7, 130.0, 129.6 (2C), 128.9, 128.3, 128.0, 127.2, 126.4, 126.3 (2C), 123.8, 118.9, 117.1, 112.1, 107.5, 59.7, 52.1.

HRMS(ESI): [M+H]$^+$ calcd. $C_{24}H_{19}N_2O_3$ m/z 383.1390, found 383.1391.

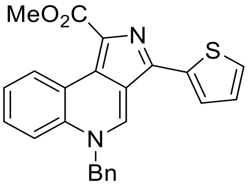

H17

Methyl 5-benzyl-3-(thiophen-2-yl)-5*H*-pyrrolo[3,4-*c*]quinoline-1-carboxylate (H17)

PQ **H17** (29 mg, 73 µmol, 73% Yield) was synthesized using the general procedure 12; column chromatography eluting with cyclohexane/acetone 2:1 to 1:2.

^{1}H NMR (500 MHz, CDCl$_3$) δ 10.14 (dd, *J* = 8.7, 1.5 Hz, 1H), 8.86 (s, 1H), 7.67 – 7.61 (m, 2H), 7.54 – 7.48 (m, 2H), 7.41 – 7.36 (m, 3H), 7.34 (dd, *J* = 5.1, 1.1 Hz, 1H), 7.19 – 7.15 (m, 2H), 7.12 (dd, *J* = 5.1, 3.6 Hz, 1H), 5.75 (s, 2H), 4.06 (s, 3H).

^{13}C NMR (126 MHz, CDCl$_3$) δ 166.0, 141.9, 141.3, 138.6, 133.8, 132.8, 130.2, 129.7 (2C), 129.1, 128.4, 128.0, 127.9, 127.3, 127.0, 126.5 (2C), 125.6, 124.8, 123.8, 119.4, 116.9, 59.7, 52.1.

HRMS(ESI): $[M+H]^+$ calcd. $C_{24}H_{19}N_2O_2S$ m/z 399.1162, found 399.1154.

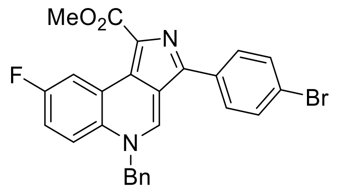

H18

Methyl 5-benzyl-3-(4-bromophenyl)-8-fluoro-5*H*-pyrrolo[3,4-*c*]quinoline-1-carboxylate (H18)

PQ **H18** (31 mg, 64 µmol, 64% Yield) was synthesized using the general procedure 12; column chromatography eluting with cyclohexane/acetone 2:1 to 1:2.

^{1}H NMR (500 MHz, CDCl$_3$) δ 9.82 (dd, *J* = 11.1, 2.9 Hz, 1H), 8.67 (s, 1H), 7.70 – 7.63 (m, 2H), 7.57 (dd, *J* = 9.4, 4.8 Hz, 1H), 7.51 – 7.47 (m, 2H), 7.39-7.33 (m, 3H), 7.17 (ddd, *J* = 9.8, 7.0, 3.0 Hz, 1H), 7.13 – 7.08 (m, 2H), 5.75 (s, 2H), 4.05 (s, 3H).

^{13}C NMR (126 MHz, CDCl$_3$) δ 165.6, 160.6 (d, *J*CF = 248.2 Hz), 146.3, 141.8, 133.7, 133.5, 132.0 (2C), 129.7 (4C), 129.2 (2C), 127.8, 126.7 (d, *J*CF = 3.8 Hz), 126.4 (2C), 125.4 (d, *J*CF = 11.3 Hz), 122.5, 119.1 (d, *J*CF = 10.1 Hz), 118.4, 116.8 (d, *J*CF = 25.2 Hz), 114.9 (d, *J*CF = 26.5 Hz), 60.0, 52.2.

^{19}F NMR (470 MHz, CDCl$_3$) δ -110.1 (m, 1F).

HRMS(ESI): $[M+H]^+$ calcd. $C_{26}H_{19}N_2O_2BrF$ m/z 489.0609, found 489.0597.

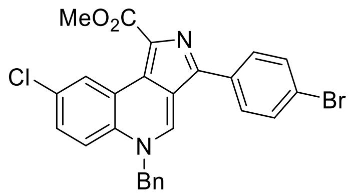

H19

Methyl 5-benzyl-3-(4-bromophenyl)-8-chloro-5*H*-pyrrolo[3,4-*c*]quinoline-1-carboxylate (H19)

PQ **H19** (42 mg, 83 µmol, 83% Yield) was synthesized using the general procedure 12; column chromatography eluting with cyclohexane/acetone 2:1 to 1:2.

^{1}H NMR (500 MHz, DMSO-*d6*) δ 10.13 (d, *J* = 2.5 Hz, 1H), 9.76 (s, 1H), 8.05 – 7.99 (m, 2H), 7.97 (d, *J* = 9.3 Hz, 1H), 7.72 – 7.64 (m, 3H), 7.37-7.31 (m, 2H), 7.31 – 7.23 (m, 3H), 6.11 (s, 2H), 3.91 (s, 3H).

^{13}C NMR (126 MHz, DMSO-*d6*) δ 165.7, 145.0, 144.7, 135.5, 134.7, 131.6 (2C), 131.2, 130.7, 129.7 (2C), 129.0 (2C), 128.1, 127.8, 127.4, 127.3, 126.4 (2C), 125.6, 124.2, 120.8, 120.6, 118.3, 58.3, 51.3.

HRMS(ESI): $[M+H]^+$ calcd. $C_{26}H_{19}N_2O_2ClBr$ m/z 505.0313, found 505.0305.

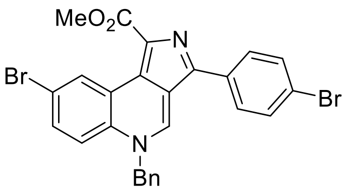

H20

Methyl 5-benzyl-8-bromo-3-(4-bromophenyl)-5*H*-pyrrolo[3,4-*c*]quinoline-1-carboxylate (H20)

PQ **H20** (37 mg, 67 µmol, 67% Yield) was synthesized using the general procedure 12; column chromatography eluting with cyclohexane/acetone 2:1 to 1:2.

^{1}H NMR (500 MHz, DMSO-*d6*) δ 10.28 (d, *J* = 2.4 Hz, 1H), 9.76 (s, 1H), 8.09 – 7.99 (m, 2H), 7.89 (d, *J* = 9.3 Hz, 1H), 7.78 (dd, *J* = 9.2, 2.4 Hz, 1H), 7.73 – 7.63 (m, 2H), 7.36 – 7.32 (m, 2H), 7.31 – 7.27 (m, 1H), 7.27 – 7.23 (m, 2H), 6.10 (s, 2H), 3.91 (s, 3H).

^{13}C NMR (126 MHz, DMSO-*d6*) δ 165.7, 145.0, 144.8, 135.5, 134.7, 131.6 (2C), 131.1, 130.6, 130.4, 129.7 (2C), 129.0 (2C), 128.0, 127.4, 126.4 (2C), 125.5, 124.5, 120.8, 120.7, 119.7, 118.4, 58.2, 51.3.

HRMS(ESI): $[M+H]^+$ calcd. $C_{26}H_{19}N_2O_2Br_2$ m/z 548.9808, found 548.9805.

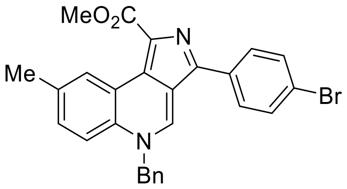

H21

Methyl 5-benzyl-3-(4-bromophenyl)-8-methyl-5*H*-pyrrolo[3,4-*c*]quinoline-1-carboxylate (H21)

PQ **H21** (39 mg, 81 µmol, 81% Yield) was synthesized using the general procedure 12; column chromatography eluting with cyclohexane/acetone 2:1 to 1:2.

^{1}H NMR (500 MHz, CDCl$_3$) δ 9.90 (d, *J* = 2.1 Hz, 1H), 8.73 (s, 1H), 7.75 – 7.68 (m, 2H), 7.56 – 7.48 (m, 3H), 7.37 – 7.32 (m, 4H), 7.13-7.09 (m, 2H), 5.77 (s, 2H), 4.06 (s, 3H), 2.57 (s, 3H).

^{13}C NMR (126 MHz, CDCl$_3$) δ 165.5, 145.6, 141.8, 137.8, 133.9, 132.0 (2C), 130.9, 130.1, 129.9 (2C), 129.6 (2C), 129.5, 129.0, 127.2, 126.3 (2C), 123.7, 122.4, 118.7, 116.9, 59.8, 52.1, 21.9.

HRMS(ESI): $[M+H]^+$ calcd. $C_{27}H_{22}N_2O_2Br$ m/z 485.0859, found 485.0853.

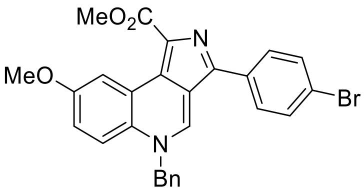

H22

Methyl 5-benzyl-3-(4-bromophenyl)-8-methoxy-5*H*-pyrrolo[3,4-*c*]quinoline-1-carboxylate (H22)

PQ **H22** (39 mg, 78 µmol, 78% Yield) was synthesized using the general procedure 12; column chromatography eluting with cyclohexane/acetone 2:1 to 1:3.

^{1}H NMR (500 MHz, DMSO-*d6*) δ 9.70 – 9.59 (m, 2H), 8.09 – 7.98 (m, 2H), 7.89 (d, *J* = 9.5 Hz, 1H), 7.71 – 7.64 (m, 2H), 7.38 – 7.21 (m, 6H), 6.09 (s, 2H), 3.93 (s, 3H), 3.89 (s, 3H).

^{13}C NMR (126 MHz, DMSO-*d6*) δ 165.8, 157.4, 143.8, 142.5, 135.8, 135.1, 131.5 (2C), 129.5 (2C), 129.0 (2C), 128.0, 127.1, 126.8, 126.6, 126.4 (2C), 124.7, 120.4, 120.0, 117.8, 117.3, 109.5, 58.2, 55.5, 51.1.

HRMS(ESI): $[M+H]^+$ calcd. $C_{27}H_{22}N_2O_3Br$ m/z 501.0808, found 501.0799.

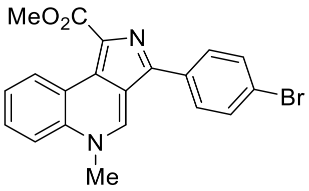

H23

Methyl 3-(4-bromophenyl)-5-methyl-5*H*-pyrrolo[3,4-*c*]quinoline-1-carboxylate (H23)

PQ **H23** (35mg, 88 µmol, 88% Yield) was synthesized using the general procedure 12; column chromatography eluting with cyclohexane/acetone 2:1 to 1:2.

^{1}H NMR (500 MHz, CDCl$_3$) δ 10.10 (d, *J* = 7.8 Hz, 1H), 8.56 (s, 1H), 7.73 – 7.61 (m, 5H), 7.59 – 7.55 (m, 2H), 4.20 (s, 3H), 4.05 (s, 3H).

^{13}C NMR (126 MHz, CDCl$_3$) δ 166.0, 146.0, 141.8, 134.4, 133.2, 132.0 (2C), 129.9, 129.8 (2C), 128.4, 127.7, 127.4, 127.3, 123.3, 122.2, 119.0, 116.0, 52.1, 44.1.

HRMS(ESI): $[M+H]^+$ calcd. $C_{20}H_{16}N_2O_2Br$ m/z 395.0390, found 395.0382.

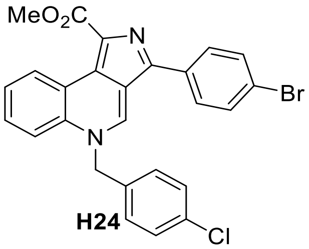

H24

Methyl 3-(4-bromophenyl)-5-(4-chlorobenzyl)-5*H*-pyrrolo[3,4-*c*]quinoline-1-carboxylate (H24)

PQ **H24** (46 mg, 79 µmol, 90% Yield) was synthesized using the general procedure 12; column chromatography eluting with cyclohexane/acetone 2:1 to 1:2.

^{1}H NMR (500 MHz, DMSO-*d6*) δ 9.96 (dd, *J* = 8.2, 1.7 Hz, 1H), 9.74 (s, 1H), 8.07 – 7.97 (m, 2H), 7.92 (dd, *J* = 8.7, 1.4 Hz, 1H), 7.71 – 7.58 (m, 4H), 7.43 – 7.37 (m, 2H), 7.34 – 7.27 (m, 2H), 6.11 (s, 2H), 3.89 (s, 3H).

^{13}C NMR (126 MHz, DMSO-*d6*) δ 165.7, 144.5, 144.4, 134.9, 134.9, 132.5, 131.9, 131.6 (2C), 129.7 (2C), 128.9 (2C), 128.5 (2C), 128.5, 128.1, 127.0, 126.7, 126.7, 122.9, 120.6, 118.3, 118.2, 57.4, 51.1.

HRMS(ESI): $[M+H]^+$ calcd. $C_{26}H_{19}N_2O_2ClBr$ m/z 505.0313, found 505.0307.

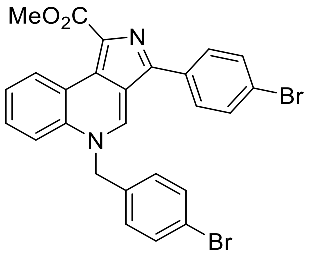

H25

Methyl 5-(4-bromobenzyl)-3-(4-bromophenyl)-5*H*-pyrrolo[3,4-*c*]quinoline-1-carboxylate (H25)

PQ **H25** (47 mg, 85 µmol, 85% Yield) was synthesized using the general procedure 12; column chromatography eluting with cyclohexane/acetone 2:1 to 1:2.

^{1}H NMR (500 MHz, DMSO-*d6*) δ 9.97 (dd, *J* = 8.2, 1.6 Hz, 1H), 9.73 (s, 1H), 8.07 – 7.98 (m, 2H), 7.91 (dd, *J* = 8.7, 1.4 Hz, 1H), 7.71 – 7.59 (m, 4H), 7.56 – 7.51 (m, 2H), 7.26 – 7.20 (m, 2H), 6.09 (s, 2H), 3.89 (s, 3H).

^{13}C NMR (126 MHz, DMSO-*d6*) δ 165.7, 144.5, 144.3, 135.3, 135.0, 131.9, 131.8 (2C), 131.6 (2C), 129.7 (2C), 128.8 (2C), 128.5, 128.1, 127.1, 126.7, 122.9, 121.1, 120.6, 118.3, 118.2, 57.5, 51.0.

HRMS(ESI): $[M+H]^+$ calcd. $C_{26}H_{19}N_2O_2Br_2$ m/z 548.9808, found 548.9798.

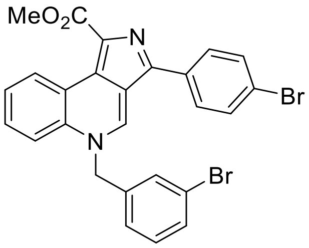

H26

Methyl 5-(3-bromobenzyl)-3-(4-bromophenyl)-5*H*-pyrrolo[3,4-*c*]quinoline-1-carboxylate (H26)

PQ **H26** (53 mg, 96 µmol, 96% Yield) was synthesized using the general procedure 12; column chromatography eluting with cyclohexane/acetone 2:1 to 1:2.

^{1}H NMR (500 MHz, DMSO-*d6*) δ 9.97 (dd, *J* = 8.1, 1.8 Hz, 1H), 9.73 (s, 1H), 8.08 – 7.98 (m, 2H), 7.95 – 7.87 (m, 1H), 7.71 – 7.61 (m, 4H), 7.60 (t, *J* = 1.9 Hz, 1H), 7.49 (ddd, *J* = 8.0, 2.1, 1.0 Hz, 1H), 7.27 (t, *J* = 7.9 Hz, 1H), 7.18 (dt, *J* = 8.1, 1.2 Hz, 1H), 6.11 (s, 2H), 3.89 (s, 3H).

^{13}C NMR (126 MHz, DMSO-*d6*) δ 165.7, 144.5, 138.6, 134.9, 131.9, 131.6 (2C), 131.1, 130.9, 129.7 (2C), 129.3, 128.5, 128.1, 127.0, 126.7, 126.7, 125.4, 122.9, 122.1, 120.6, 118.2, 118.2, 57.4, 51.1.

HRMS(ESI): $[M+H]^+$ calcd. $C_{26}H_{19}N_2O_2Br_2$ m/z 548.9808, found 548.9803.

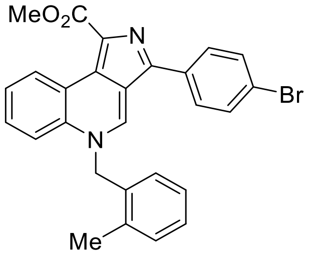

H27

Methyl 3-(4-bromophenyl)-5-(2-methylbenzyl)-5*H*-pyrrolo[3,4-*c*]quinoline-1-carboxylate (H27)

PQ **H27** (44 mg, 90 µmol, 90% Yield) was synthesized using the general procedure 12; column chromatography eluting with cyclohexane/acetone 2:1 to 1:2.

^{1}H NMR (500 MHz, DMSO-*d6*) δ 10.01 (dd, *J* = 8.4, 1.5 Hz, 1H), 9.62 (s, 1H), 8.03 – 7.92 (m, 2H), 7.76 (d, *J* = 8.7 Hz, 1H), 7.69 (dd, *J* = 8.4, 7.0 Hz, 1H), 7.67 – 7.59 (m, 3H), 7.38 – 7.27 (m, 1H), 7.18 (t, *J* = 7.5 Hz, 1H), 6.98 (t, *J* = 7.7 Hz, 1H), 6.36 – 6.23 (m, 1H), 6.07 (s, 2H), 3.90 (s, 3H), 2.49 (s, 3H).

^{13}C NMR (126 MHz, DMSO-*d6*) δ 165.7, 144.3, 144.2, 134.9, 134.9, 134.0, 132.3, 131.6 (2C), 130.4, 129.5 (2C), 128.4, 128.2, 127.5, 127.1, 126.8, 126.7, 126.4 (2C), 122.7, 120.5, 118.4, 118.3, 56.4, 51.1, 18.8.

HRMS(ESI): $[M+H]^+$ calcd. $C_{27}H_{22}N_2O_2Br$ m/z 485.0859, found 485.0850.

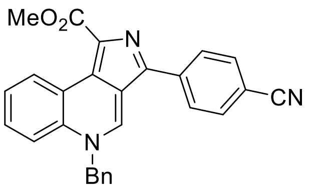

H28

Methyl 5-benzyl-3-(4-cyanophenyl)-5*H*-pyrrolo[3,4-*c*]quinoline-1-carboxylate (H28)

PQ **H28** (8 mg, 20 µmol, 20%) was synthesized according to the general procedure 13; column chromatography eluting with cyclohexane/acetone 2:1 to 1:2.

^{1}H NMR (700 MHz, DMSO-*d6*) δ 9.97 (dd, *J* = 8.3, 1.6 Hz, 1H), 9.87 (s, 1H), 8.36 – 8.24 (m, 2H), 7.99 (d, *J* = 8.7 Hz, 1H), 7.96 – 7.89 (m, 2H), 7.68 (dd, *J* = 8.2, 7.0 Hz, 1H), 7.64 (ddd, *J* = 8.6, 6.9, 1.6 Hz, 1H), 7.36-7.32 (m, 2H), 7.32 – 7.25 (m, 3H), 6.15 (s, 2H), 3.91 (s, 3H).

^{13}C NMR (176 MHz, DMSO-*d6*) δ 165.6, 144.4, 143.0, 140.1, 135.6, 132.6 (2C), 132.0, 128.9 (2C), 128.4, 128.2, 128.0, 128.0 (2C), 127.7, 127.2, 126.8, 126.4 (2C), 122.9, 119.2, 118.5, 118.4, 109.1, 58.3, 51.1.

HRMS(ESI): $[M+H]^+$ calcd. $C_{27}H_{20}N_3O_2$ m/z 418.1550, found 418.1548.

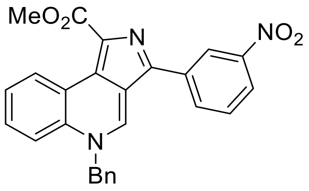

H29

Methyl 5-benzyl-3-(3-nitrophenyl)-5*H*-pyrrolo[3,4-*c*]quinoline-1-carboxylate (H29)

PQ **H29** (7 mg, 16 µmol, 16%) was synthesized according to the general procedure 13; column chromatography eluting with cyclohexane/acetone 2:1 to 1:2.

^{1}H NMR (600 MHz, DMSO-*d6*) δ 9.98 (dd, *J* = 8.3, 1.6 Hz, 1H), 9.88 (s, 1H), 8.82 (t, *J* = 2.0 Hz, 1H), 8.52 (d, *J* = 7.8 Hz, 1H), 8.20 (dd, *J* = 8.2, 2.4 Hz, 1H), 7.99 (d, *J* = 8.6 Hz, 1H), 7.79 (t, *J* = 8.0 Hz, 1H), 7.68 (ddd, *J* = 8.3, 7.0, 1.2 Hz, 1H), 7.64 (ddd, *J* = 8.6, 7.0, 1.7 Hz, 1H), 7.37 – 7.32 (m, 2H), 7.31 – 7.26 (m, 3H), 6.14 (s, 2H), 3.92 (s, 3H).

^{13}C NMR (151 MHz, DMSO-*d6*) δ 165.6, 148.4, 144.3, 142.7, 137.3, 135.6, 133.6, 132.0, 130.3, 128.9 (2C), 128.4, 128.1, 128.0, 127.3, 127.0, 126.8, 126.5 (2C), 122.9, 121.7, 121.5, 118.5, 118.1, 58.3, 51.1.

HRMS(ESI): $[M+H]^+$ calcd. $C_{26}H_{20}N_3O_4$ m/z 438.1448, found 438.1443.

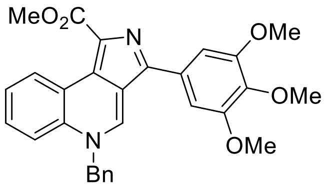

H30

Methyl 5-benzyl-3-(3,4,5-trimethoxyphenyl)-5*H*-pyrrolo[3,4-*c*]quinoline-1-carboxylate (H30)

PQ **H30**(11 mg, 23 µmol, 23%) was synthesized according to the general procedure 13; column chromatography eluting with cyclohexane/acetone 2:1 to 1:3.

^{1}H NMR (700 MHz, DMSO-*d6*) δ 9.95 (dd, *J* = 8.2, 1.7 Hz, 1H), 9.61 (s, 1H), 7.99 (dd, *J* = 8.6, 1.3 Hz, 1H), 7.66-7.60 (m, 2H), 7.37 – 7.32 (m, 2H), 7.30 – 7.27 (m, 3H), 7.17 (s, 2H), 6.10 (s, 2H), 3.91 (s, 6H), 3.89 (s, 3H), 3.73 (s, 3H).

^{13}C NMR (176 MHz, DMSO-*d6*) δ 165.7, 153.2 (2C), 146.4, 144.2, 137.3, 135.8, 132.2, 131.3, 128.9 (2C), 128.4, 128.0, 127.9, 126.7 (2C), 126.5, 126.3, 122.9, 118.3, 118.2, 105.3 (2C), 60.1, 57.8, 56.0 (2C), 51.0, 26.3.

HRMS(ESI): $[M+H]^+$ calcd. $C_{29}H_{27}N_2O_5$ m/z 483.1915, found 483.1907.

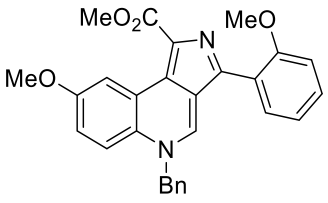

H31

Methyl 5-benzyl-8-methoxy-3-(2-methoxyphenyl)-5*H*-pyrrolo[3,4-*c*]quinoline-1-carboxylate (H31)

PQ **H31** (9 mg, 21 µmol, 21% Yield) was synthesized using the general procedure 12; column chromatography eluting with cyclohexane/acetone 2:1 to 1:3.

^{1}H NMR (500 MHz, CDCl$_3$) δ 9.81 (d, *J* = 2.9 Hz, 1H), 8.91 (s, 1H), 7.96 (dd, *J* = 7.5, 1.8 Hz, 1H), 7.59 (d, *J* = 9.4 Hz, 1H), 7.39 – 7.29 (m, 4H), 7.17 – 7.09 (m, 4H), 6.99 (d, *J* = 8.3 Hz, 1H), 5.77 (s, 2H), 4.06 (s, 3H), 4.04 (s, 3H), 3.73 (s, 3H).

^{13}C NMR (126 MHz, CDCl$_3$) δ 165.6, 158.3, 156.5, 143.7, 142.7, 134.3, 132.6, 129.7, 129.5 (2C), 128.9, 127.6, 127.0, 126.4, 126.3 (2C), 125.7, 124.3, 121.9, 119.3, 118.7, 118.5, 111.9, 110.3, 59.7, 56.1, 56.1, 52.0.

HRMS(ESI): $[M+H]^+$ calcd. $C_{28}H_{25}N_2O_4$ m/z 453.1809, found 453.1801.

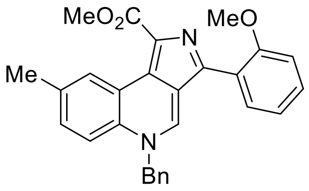

H32

Methyl 5-benzyl-3-(2-methoxyphenyl)-8-methyl-5*H*-pyrrolo[3,4-*c*]quinoline-1-carboxylate (H32)

PQ **H32** (18 mg, 40 µmol, 40% Yield) was synthesized using the general procedure 12; column chromatography eluting with cyclohexane/acetone 2:1 to 1:3.

^{1}H NMR (500 MHz, CDCl$_3$) δ 9.97 (d, *J* = 2.1 Hz, 1H), 8.83 (s, 1H), 7.91 (dd, *J* = 7.6, 1.8 Hz, 1H), 7.55 (d, *J* = 8.7 Hz, 1H), 7.38 – 7.31 (m, 5H), 7.16-7.12 (m, 2H), 7.10 (t, *J* = 7.5 Hz, 1H), 6.98 (d, *J* = 8.4 Hz, 1H), 5.70 (s, 2H), 4.04 (s, 3H), 3.70 (s, 3H), 2.58 (s, 3H).

^{13}C NMR (126 MHz, CDCl$_3$) δ 165.9, 156.5, 144.6, 143.4, 137.3, 134.3, 132.6, 130.6, 129.7, 129.6, 129.6, 129.5 (2C), 128.9, 127.4, 127.2, 126.3 (2C), 124.8, 124.0, 121.8, 119.9, 116.8, 111.8, 59.5, 56.1, 51.9, 21.9.

HRMS(ESI): $[M+H]^+$ calcd. $C_{28}H_{25}N_2O_3$ m/z 437.1860, found 437.1854.

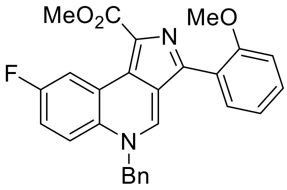

H33

Methyl 5-benzyl-8-fluoro-3-(2-methoxyphenyl)-5*H*-pyrrolo[3,4-*c*]quinoline-1-carboxylate (H33)

PQ **H33** (25 mg, 57 µmol, 57% Yield) was synthesized using the general procedure 12; column chromatography eluting with cyclohexane/acetone 2:1 to 1:3.

^{1}H NMR (700 MHz, $CDCl_3$) δ 10.00 (dd, *J* = 11.2, 3.0 Hz, 1H), 8.85 (s, 1H), 7.90 (dd, *J* = 7.5, 1.8 Hz, 1H), 7.63 (dd, *J* = 9.4, 4.8 Hz, 1H), 7.39-7.34 (m, 4H), 7.22 (ddd, *J* = 9.6, 7.0, 3.0 Hz, 1H), 7.18 – 7.14 (m, 2H), 7.12 (t, *J* = 7.4 Hz, 1H), 7.00 (d, *J* = 8.2 Hz, 1H), 5.71 (s, 2H), 4.06 (s, 3H), 3.71 (s, 3H).

^{13}C NMR (176 MHz, $CDCl_3$) δ 166.0, 160.5 (d, *J*CF = 248.2 Hz), 156.6, 145.3, 143.6, 134.0, 132.6, 129.7 (2C), 129.5, 129.1, 129.0, 128.4, 126.9 (d, *J*CF = 3.5 Hz), 126.3 (2C), 125.9 (d, *J*CF = 10.6 Hz), 124.9, 121.9, 119.7, 118.9 (d, *J*CF = 8.8 Hz), 116.5 (d, *J*CF = 24.6 Hz), 115.2 (d, *J*CF = 26.4 Hz), 111.9, 59.9, 56.1, 52.0.

^{19}F NMR (470 MHz, $CDCl_3$) δ -110.8 (m, 1F).

HRMS(ESI): $[M+H]^+$ calcd. $C_{27}H_{22}N_2O_3F$ m/z 441.1609, found 441.1604.

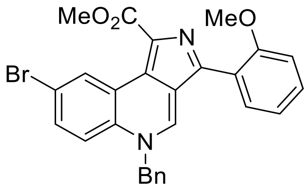

H34

Methyl 5-benzyl-8-bromo-3-(2-methoxyphenyl)-5*H*-pyrrolo[3,4-*c*]quinoline-1-carboxylate (H34)

PQ **H34** (9 mg, 19 µmol, 19% Yield) was synthesized using the general procedure 12; column chromatography eluting with cyclohexane/acetone 2:1 to 1:3.

^{1}H NMR (500 MHz, $CDCl_3$) δ 10.35 (d, *J* = 2.2 Hz, 1H), 8.85 (s, 1H), 7.83 (dd, *J* = 7.6, 1.8 Hz, 1H), 7.58 (dd, *J* = 9.1, 2.3 Hz, 1H), 7.52 (d, *J* = 9.1 Hz, 1H), 7.40 – 7.32 (m, 4H), 7.15-7.12 (m, 2H), 7.07 (t, *J* = 7.5 Hz, 1H), 6.97 (d, *J* = 8.4 Hz, 1H), 5.71 (s, 2H), 4.04 (s, 3H), 3.71 (s, 3H).

^{13}C NMR (126 MHz, $CDCl_3$) δ 165.3, 156.5, 144.8, 144.5, 133.7, 132.4, 132.3, 131.4, 131.3, 130.1, 129.7 (2C), 129.1, 127.1, 126.3 (2C), 125.7, 125.2, 123.6, 121.8, 121.2, 119.8, 118.7, 111.8, 59.8, 56.0, 52.2.

HRMS(ESI): $[M+H]^+$ calcd. $C_{27}H_{22}N_2O_3Br_2$ m/z 501.0808, found 501.0797.

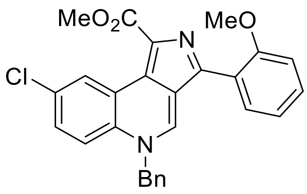

H35

Methyl 5-benzyl-8-chloro-3-(2-methoxyphenyl)-5*H*-pyrrolo[3,4-*c*]quinoline-1-carboxylate (H35)

PQ **H35** (13 mg, 28 µmol, 28% Yield) was synthesized using the general procedure 12; column chromatography eluting with cyclohexane/acetone 2:1 to 1:3.

^{1}H NMR (500 MHz, CDCl$_3$) δ 10.24 (d, *J* = 2.4 Hz, 1H), 8.78 (s, 1H), 7.87 (dd, *J* = 7.5, 1.8 Hz, 1H), 7.53 (d, *J* = 9.1 Hz, 1H), 7.41 – 7.33 (m, 5H), 7.15-7.12 (m, 2H), 7.10 (td, *J* = 7.5, 1.1 Hz, 1H), 6.98 (d, *J* = 8.4 Hz, 1H), 5.66 (s, 2H), 4.06 (s, 3H), 3.69 (s, 3H).

^{13}C NMR (126 MHz, CDCl$_3$) δ 166.1, 156.5, 145.8, 143.6, 133.9, 132.7, 132.5, 130.7, 129.7 (2C), 129.6, 129.2, 129.1, 128.7, 128.2, 126.3 (2C), 126.2, 125.0, 125.0, 121.7, 120.3, 118.2, 111.8, 59.6, 56.0, 52.0.

HRMS(ESI): $[M+H]^+$ calcd. $C_{27}H_{22}N_2O_3Cl$ m/z 457.1314, found 457.1303.

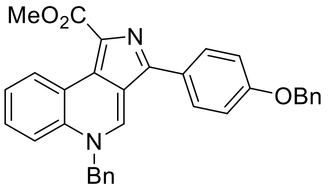

H36

Methyl 5-benzyl-3-(4-(benzyloxy)phenyl)-5*H*-pyrrolo[3,4-*c*]quinoline-1-carboxylate (H36)

PQ **H36** (64 mg, 128 µmol, 64% Yield) was synthesized using the general procedure 12 for 0.2 mmol scale; column chromatography eluting with cyclohexane/acetone 2:1 to 1:2.

^{1}H NMR (700 MHz, DMSO-*d6*) δ 9.96 (dd, *J* = 8.2, 1.6 Hz, 1H), 9.63 (s, 1H), 8.02 – 7.95 (m, 2H), 7.92 (d, *J* = 8.6 Hz, 1H), 7.64 – 7.60 (m, 1H), 7.60 – 7.55 (m, 1H), 7.51 – 7.48 (m, 2H), 7.43-7.39 (m, 2H), 7.36 – 7.31 (m, 3H), 7.29 – 7.23 (m, 3H), 7.18 – 7.11 (m, 2H), 6.09 (s, 2H), 5.19 (s, 2H), 3.88 (s, 3H).

^{13}C NMR (126 MHz, DMSO-*d6*) δ 165.8, 158.2, 146.1, 144.3, 137.2, 135.9, 132.2, 129.2 (2C), 129.1 (2C), 128.6 (3C), 128.5, 128.1, 128.0 (2C), 127.8 (3C), 126.6, 126.5 (2C), 126.3, 123.0, 118.4, 118.2, 115.2 (2C), 69.4, 58.0, 51.1.

HRMS(ESI): $[M+H]^+$ calcd. $C_{33}H_{27}N_2O_3$ m/z 499.2016, found 499.2004.

References

[1] M.-A. Bray, S. Singh, H. Han, C. T. Davis, B. Borgeson, C. Hartland, M. Kost-Alimova, S. M. Gustafsdottir, C. C. Gibson, A. E. Carpenter, *Nat. Protoc.* **2016**, *11*, 1757-1774.

[2] M. H. Woehrmann, W. M. Bray, J. K. Durbin, S. C. Nisam, A. K. Michael, E. Glassey, J. M. Stuart, R. S. Lokey, *Mol. Biosyst.* **2013**, *9*, 2604-2617.

[3] O. V. Dolomanov, L. J. Bourhis, R. J. Gildea, J. A. K. Howard, H. Puschmann, *J. Appl. Crystallogr.* **2009**, *42*, 339-341.

[4] G. Sheldrick, *Acta Crystallogr. Sect. A* **2015**, *71*, 3-8.

[5] G. Sheldrick, *Acta Crystallogr. Sect. A* **2008**, *64*, 112-122.

[6] S. R. Vidadala, C. Golz, C. Strohmann, C.-G. Daniliuc, H. Waldmann, *Angew. Chem. Int. Ed.* **2015**, *54*, 651-655.

[7] G. M. Coppola, *J. Heterocyclic Chem.* **1986**, *23*, 223-224.

[8] H. Cui, X. Peng, J. Liu, C. Ma, Y. Ji, W. Zhang, M. Geng, Y. Li, *Bioorg. Med. Chem. Lett.* **2016**, *26*, 4483-4486.

[9] A. P. Antonchick, C. Gerding-Reimers, M. Catarinella, M. Schürmann, H. Preut, S. Ziegler, D. Rauh, H. Waldmann, *Nat. Chem.* **2010**, *2*, 735-740.

[10] S. B. L. T. Silva, A.D.; De Carvalho, J.E.; Ruiz, A.L.T.G.; Silva, L.F., Jr, *Molecules* **2015**, *20*, 1475-1494.

[11] Z. Tan, C. Ci, J. Yang, Y. Wu, L. Cao, H. Jiang, M. Zhang, *ACS Catal.* **2020**, *10*, 5243-5249.

NMR spectra of synthetic precursors

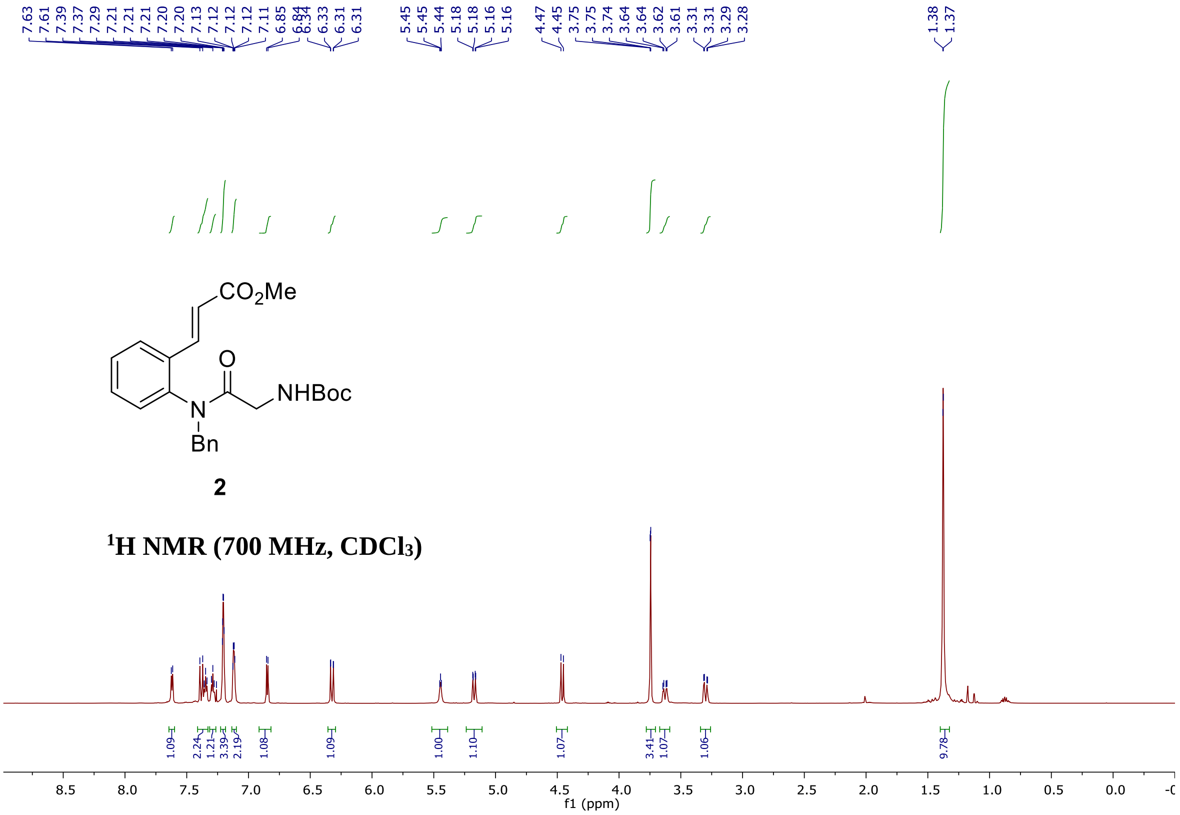

2

^{1}H NMR (700 MHz, CDCl3)

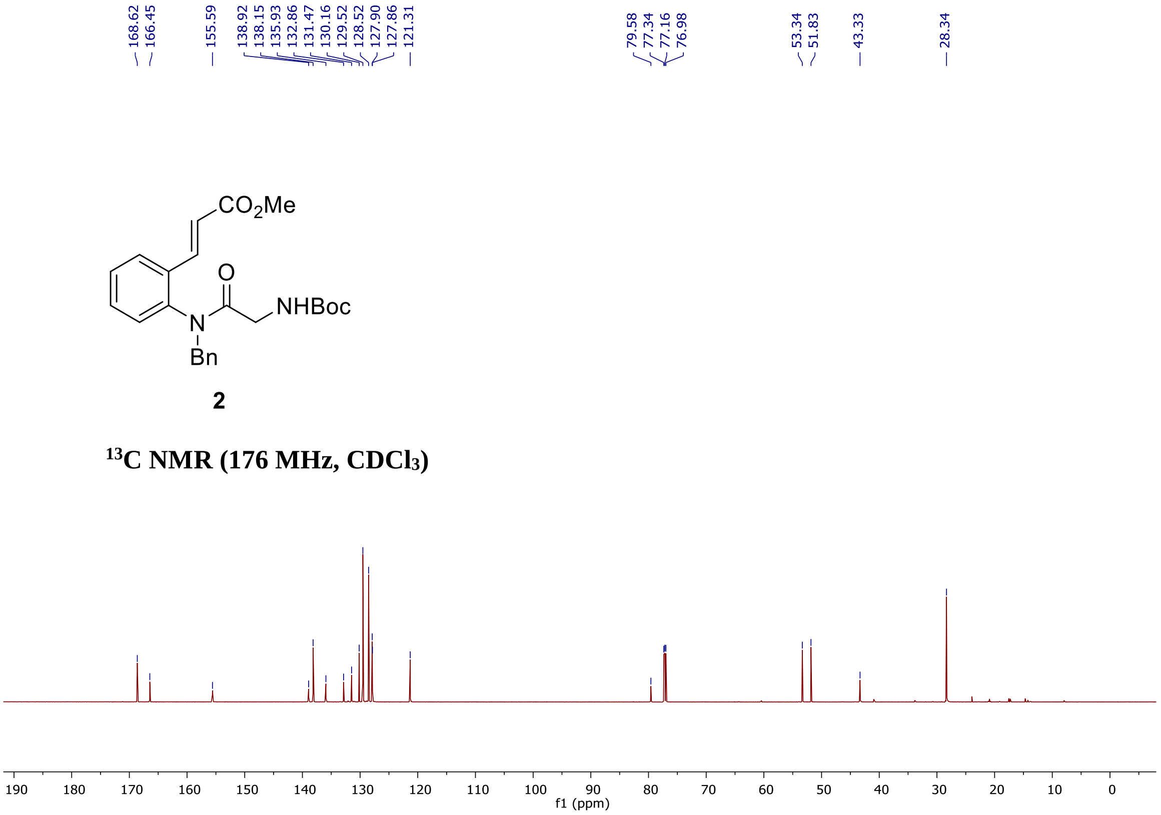

2

^{13}C NMR (176 MHz, CDCl3)

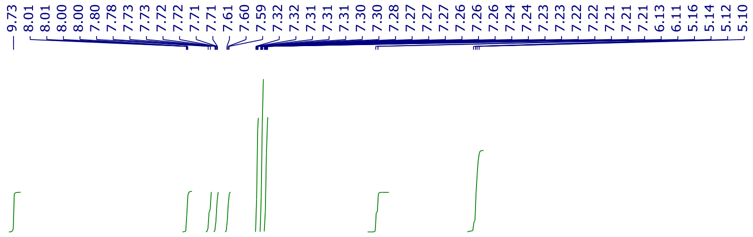

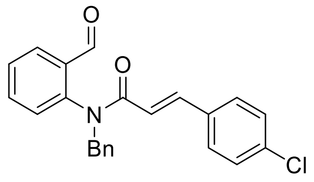

4a

^{1}H NMR (700 MHz, CDCl$_3$)

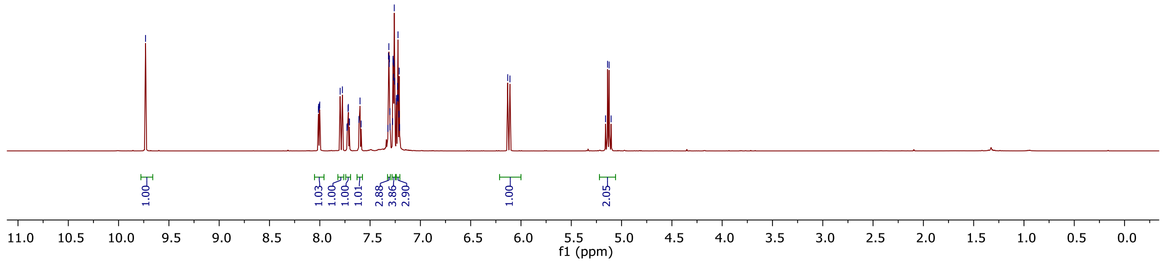

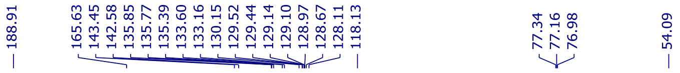

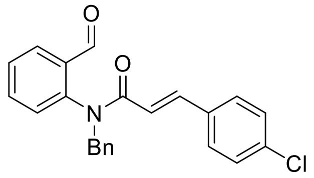

4a

^{13}C NMR (176 MHz, CDCl$_3$)

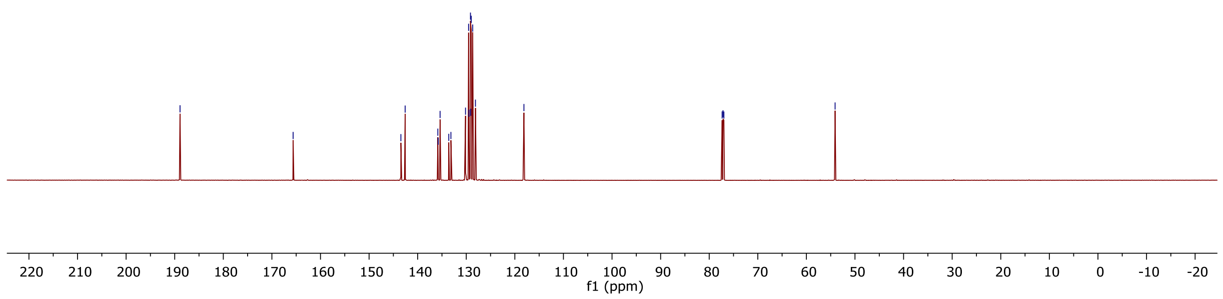

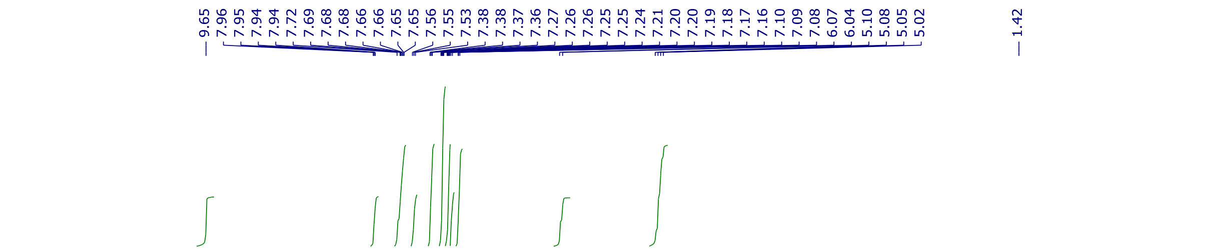

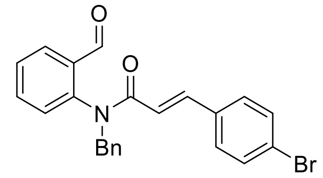

4b

^{1}H NMR (500 MHz, CDCl$_3$)

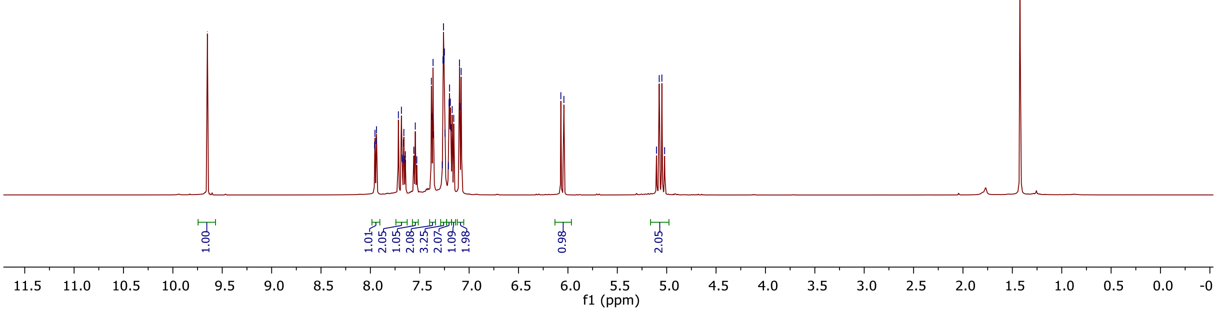

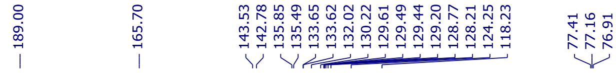

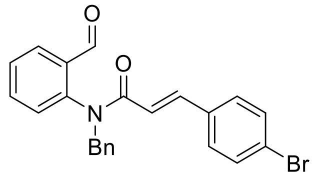

4b

^{13}C NMR (126 MHz, CDCl$_3$)

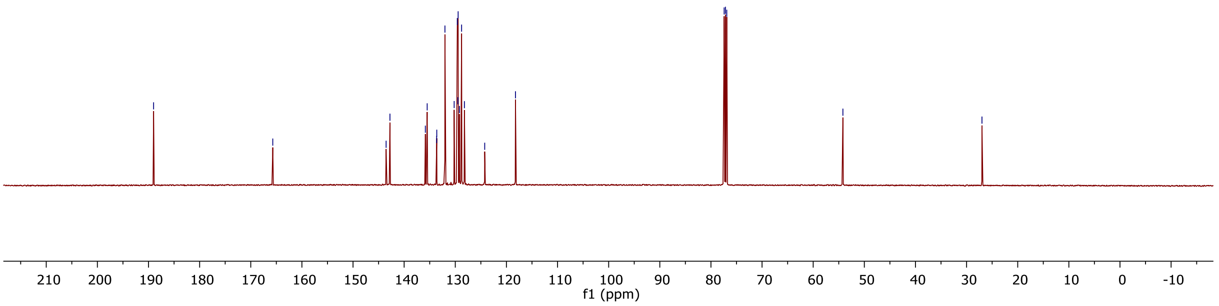

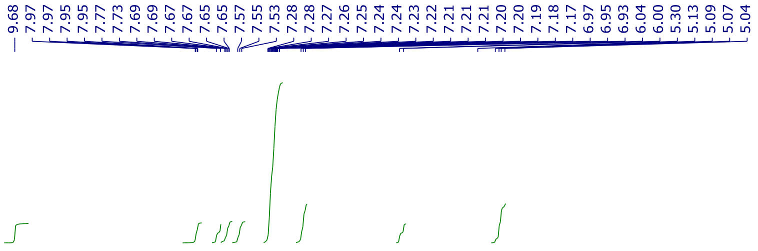

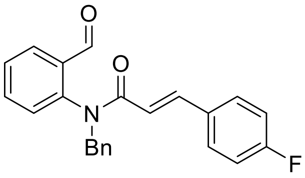

4c

^{1}H NMR (400 MHz, CDCl$_3$)

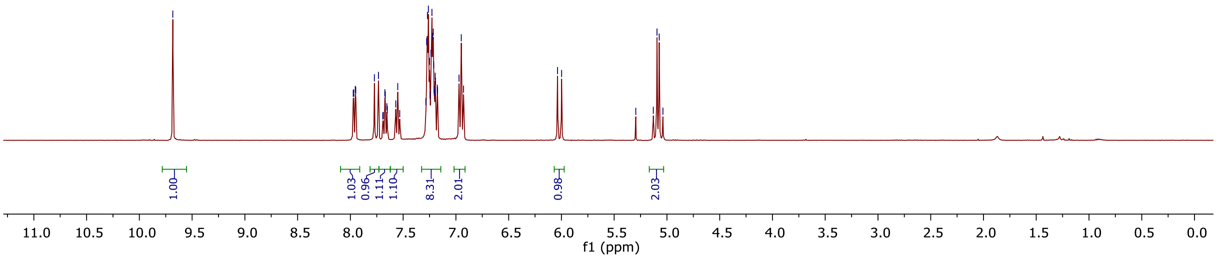

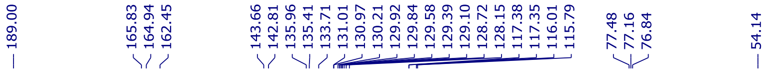

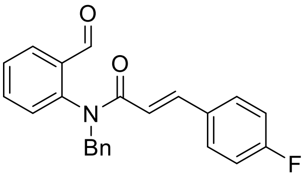

4c

^{13}C NMR (101 MHz, CDCl$_3$)

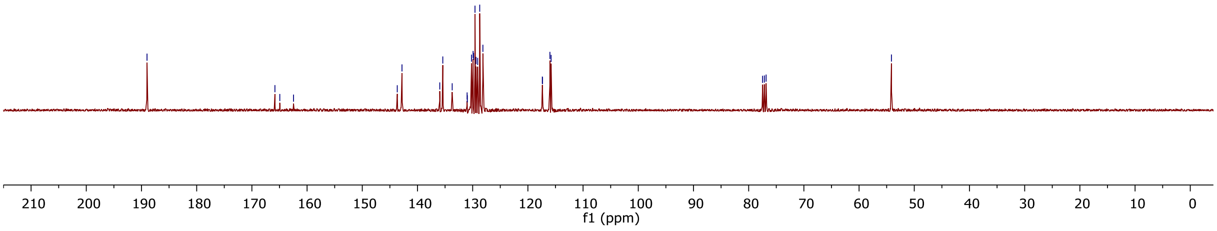

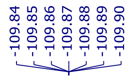

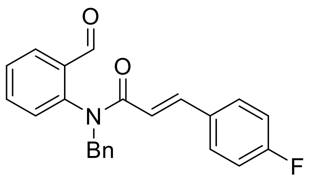

4c

^{19}F NMR (470 MHz, CDCl$_3$)

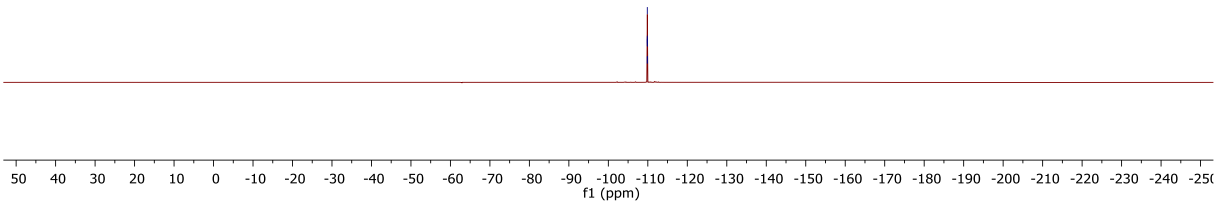

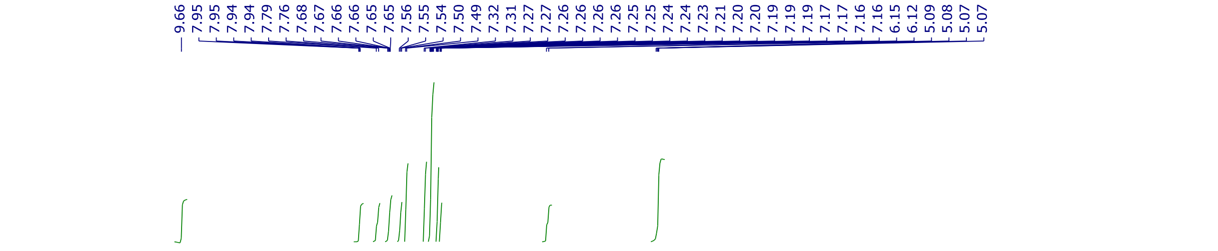

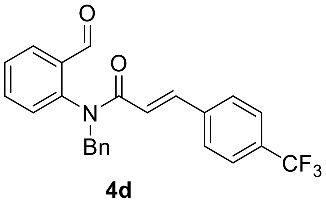

4d

^{1}H NMR (700 MHz, CDCl$_3$)

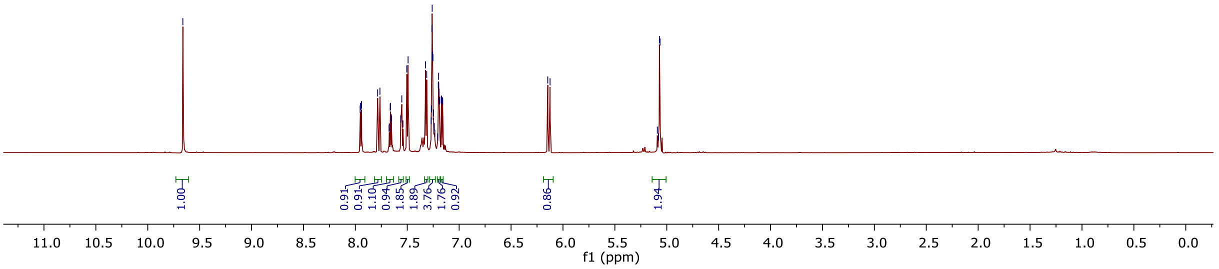

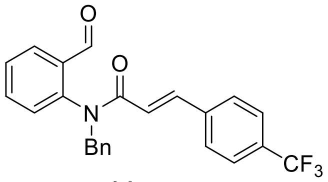

4d

^{13}C NMR (176 MHz, $CDCl_3$)

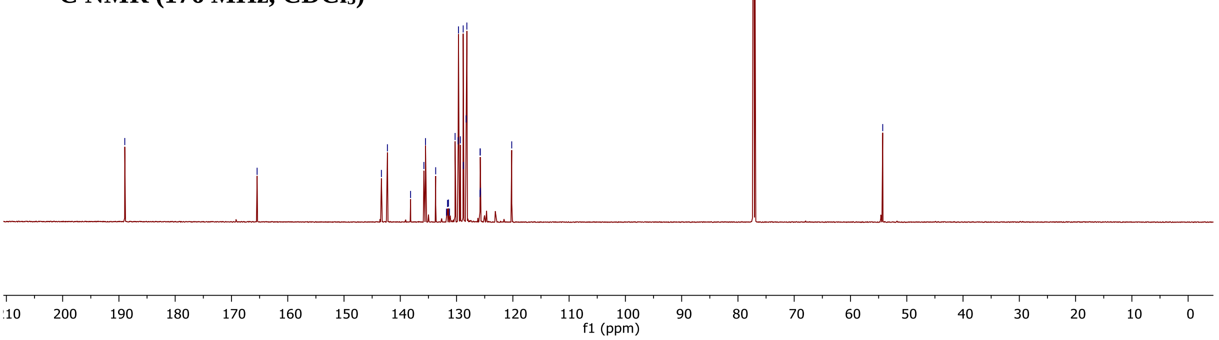

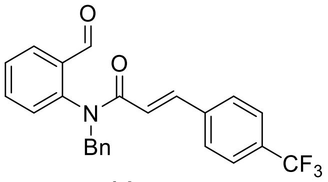

4d

^{19}F NMR (470 MHz, $CDCl_3$)

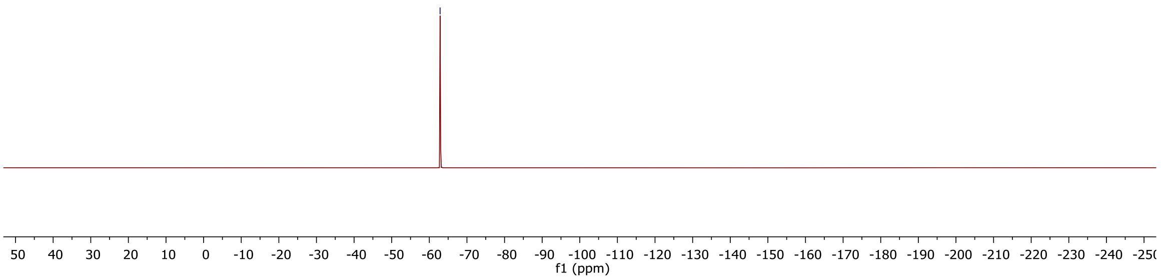

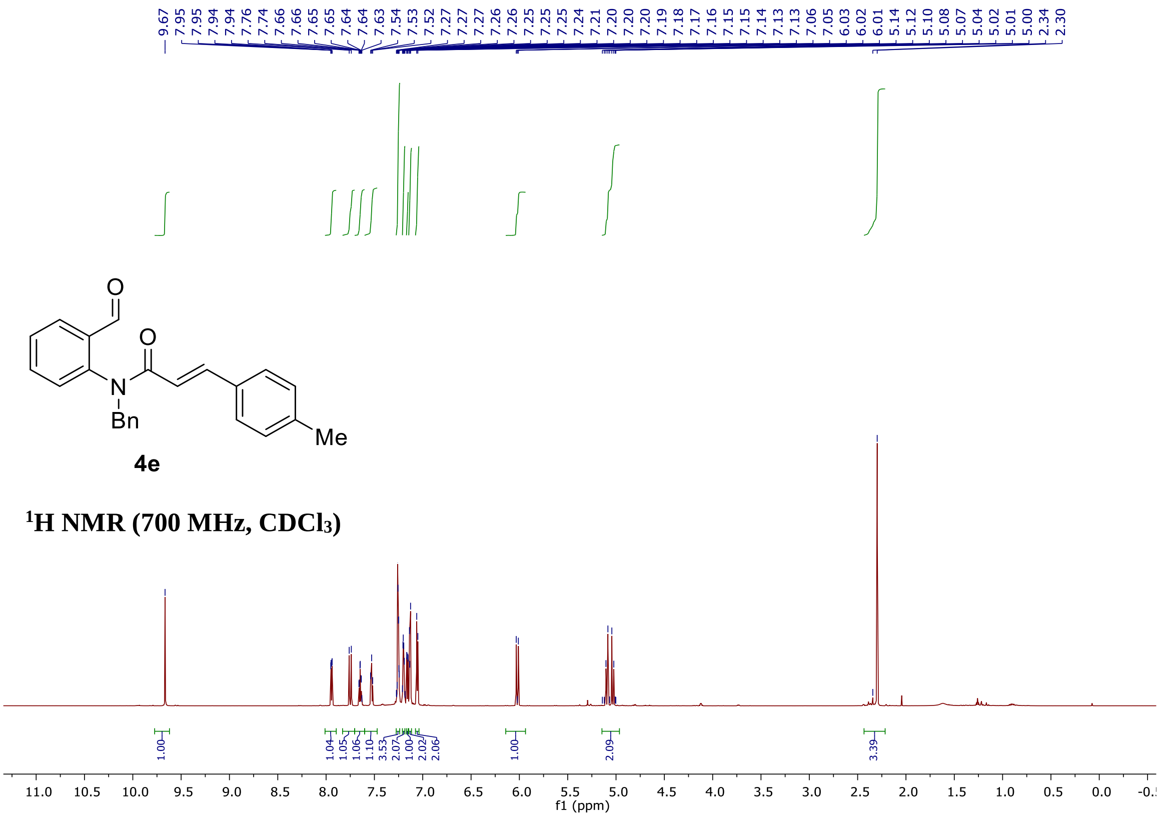

4e

^{1}H NMR (700 MHz, $CDCl_3$)

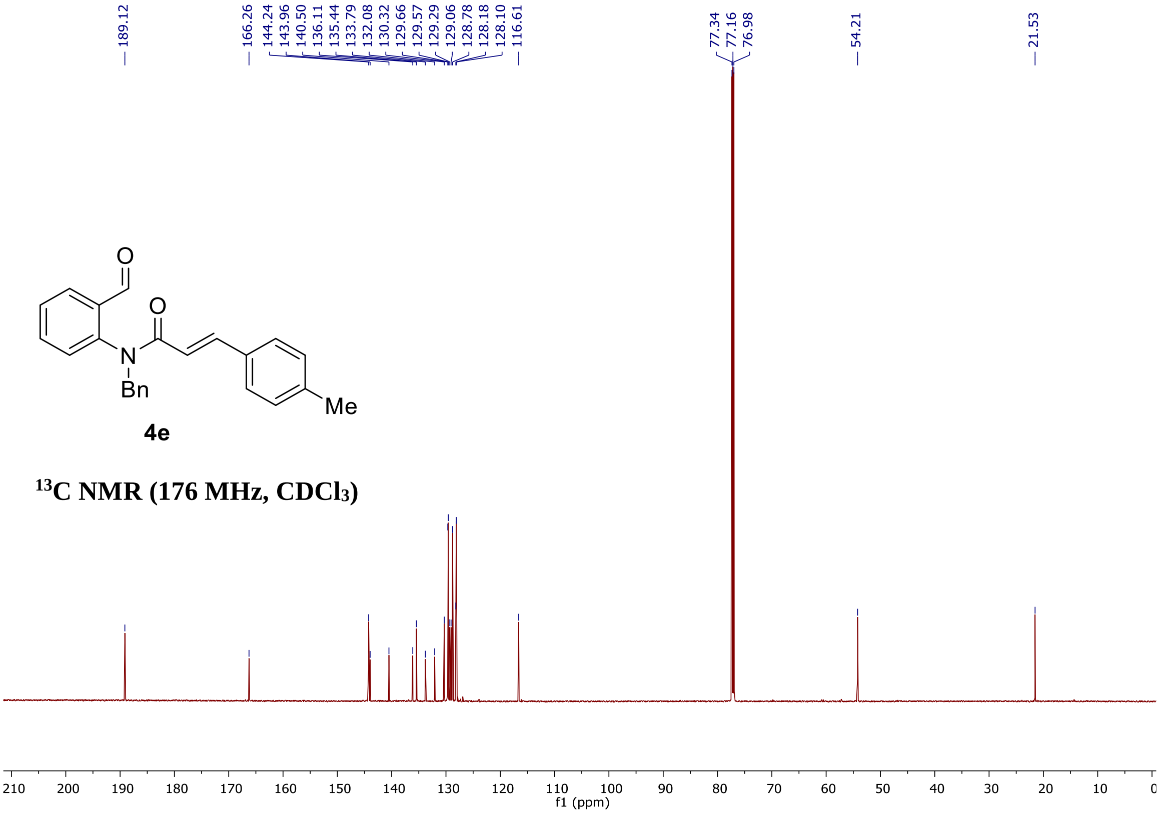

4e

^{13}C NMR (176 MHz, $CDCl_3$)

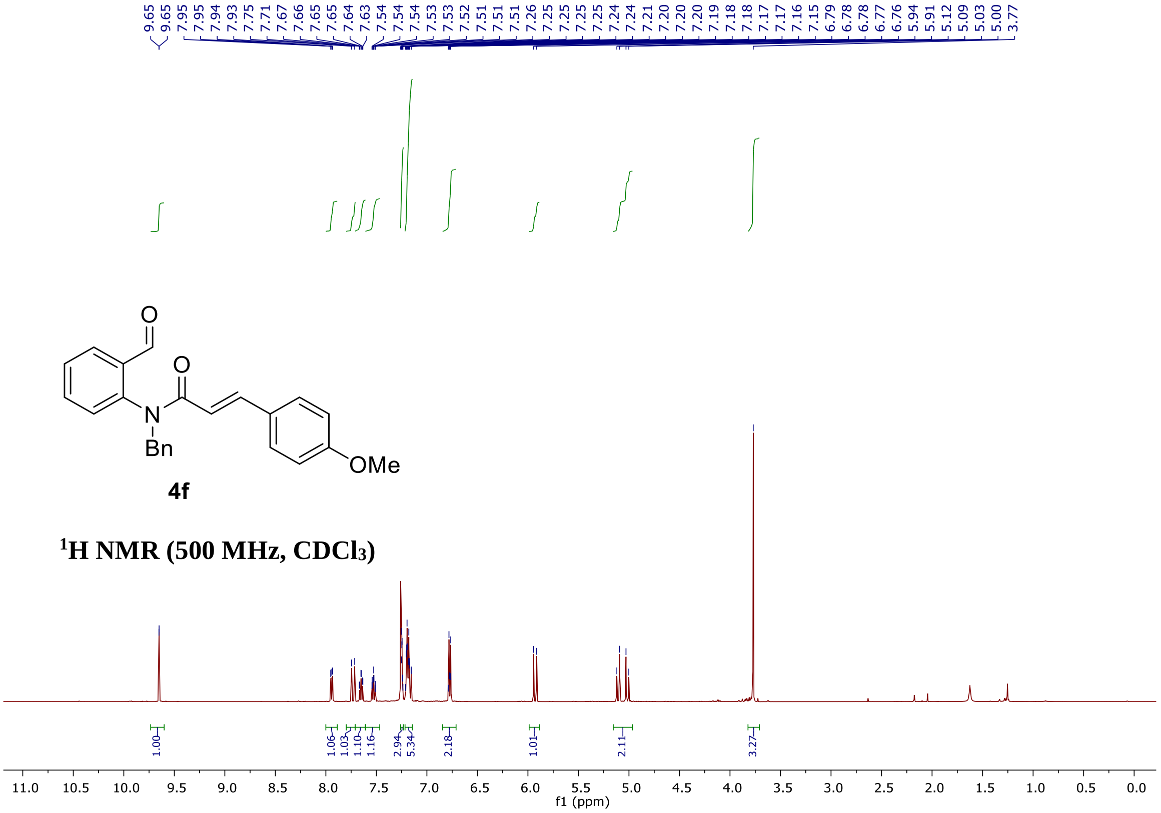
9.65 9.65 7.95 7.95 7.94 7.93 7.75 7.71 7.67 7.66 7.65 7.65 7.64 7.63 7.54 7.54 7.54 7.53 7.53 7.52 7.51 7.51 7.51 7.26 7.25 7.25 7.25 7.25 7.24 7.24 7.21 7.20 7.20 7.20 7.19 7.18 7.18 7.17 7.17 7.16 7.15 6.79 6.78 6.77 6.76 5.94 5.91 5.12 5.09 5.03 5.00 3.77
O
N
Bn
OMe
4f
1H NMR (500 MHz, CDCl3)
1.00
1.06 1.03 1.10 1.16
2.94 5.34
2.18
1.01
2.11
3.27
11.0 10.5 10.0 9.5 9.0 8.5 8.0 7.5 7.0 6.5 6.0 5.5 5.0 4.5 4.0 3.5 3.0 2.5 2.0 1.5 1.0 0.5 0.0
f1 (ppm)

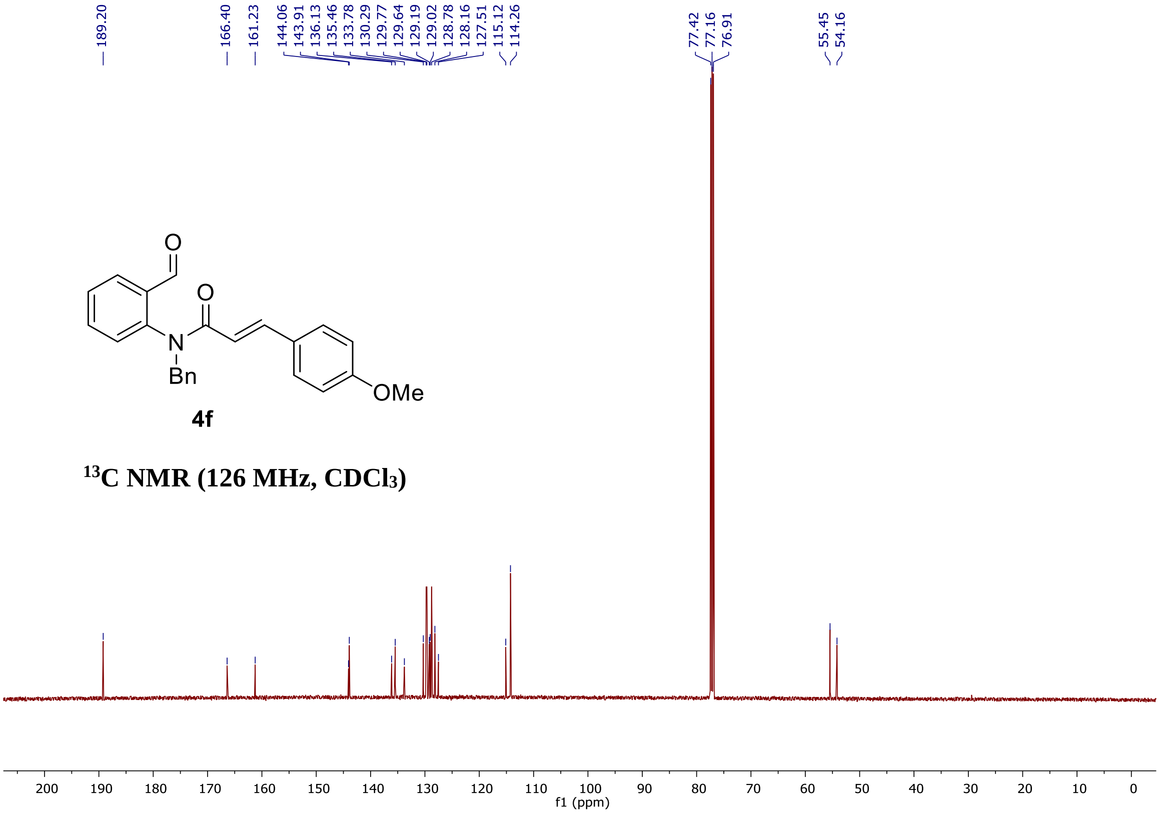
189.20
166.40
161.23
144.06 143.91 136.13 135.46 133.78 130.29 129.77 129.64 129.19 129.02 128.78 128.16 127.51 115.12 114.26
77.42 77.16 76.91
55.45 54.16
O
N
Bn
OMe
4f
13C NMR (126 MHz, CDCl3)
200 190 180 170 160 150 140 130 120 110 100 90 80 70 60 50 40 30 20 10 0
f1 (ppm)

4g

^{1}H NMR (700 MHz, $CDCl_3$)

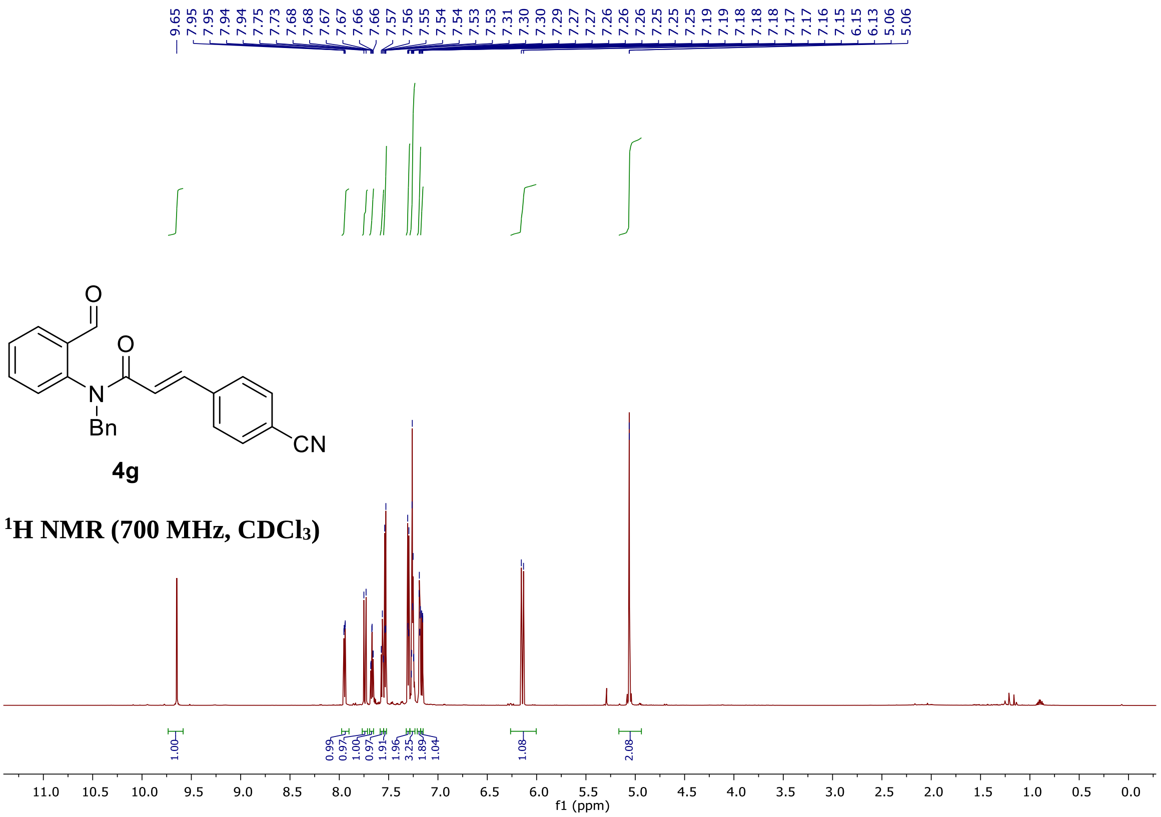

4g

^{13}C NMR (176 MHz, $CDCl_3$)

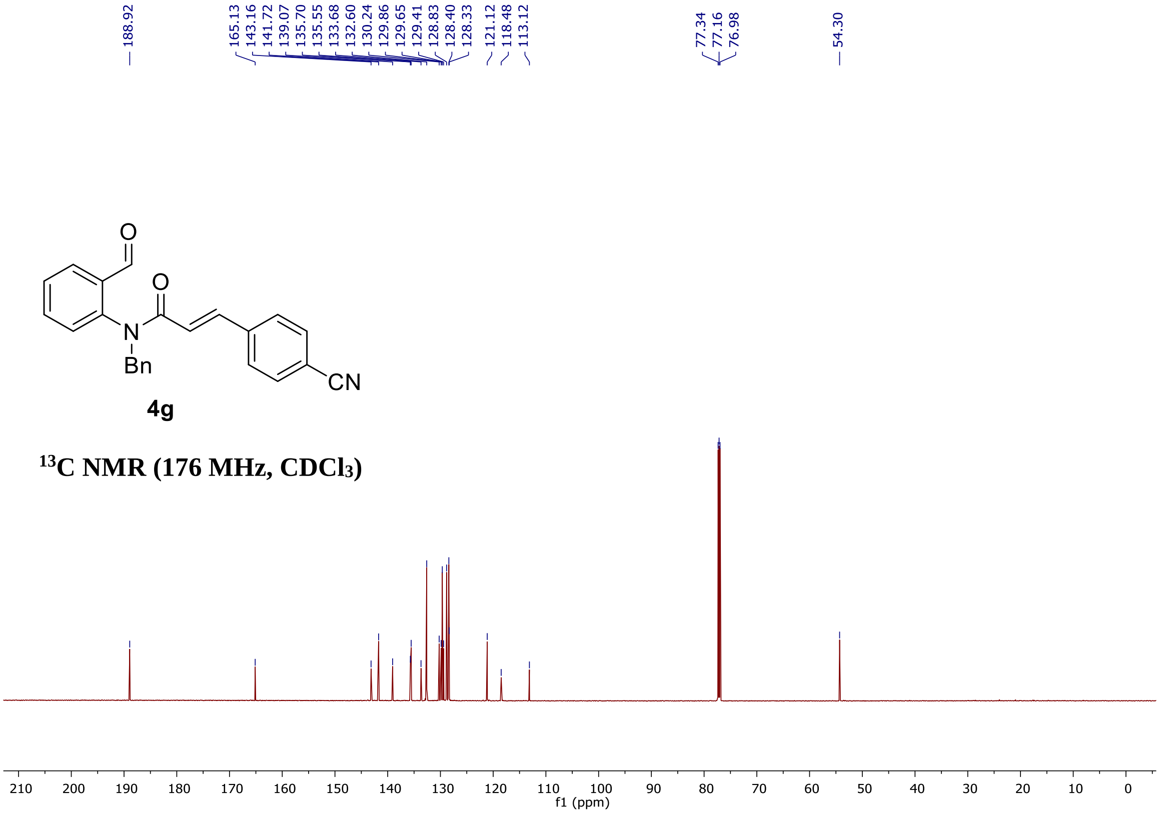

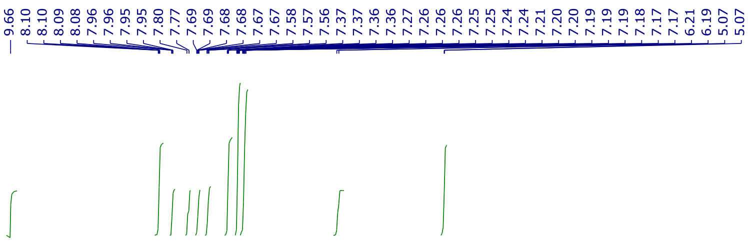

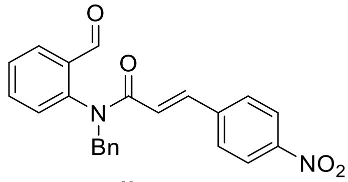

4h

^{1}H NMR (700 MHz, $CDCl_3$)

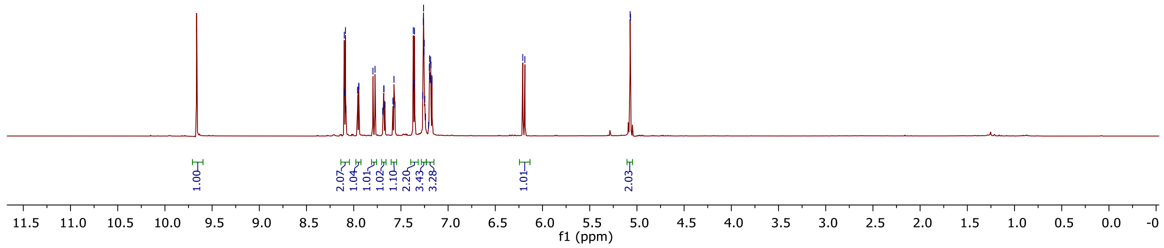

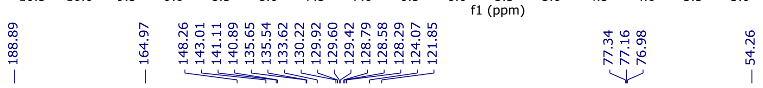

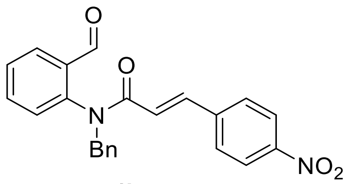

4h

^{13}C NMR (176 MHz, $CDCl_3$)

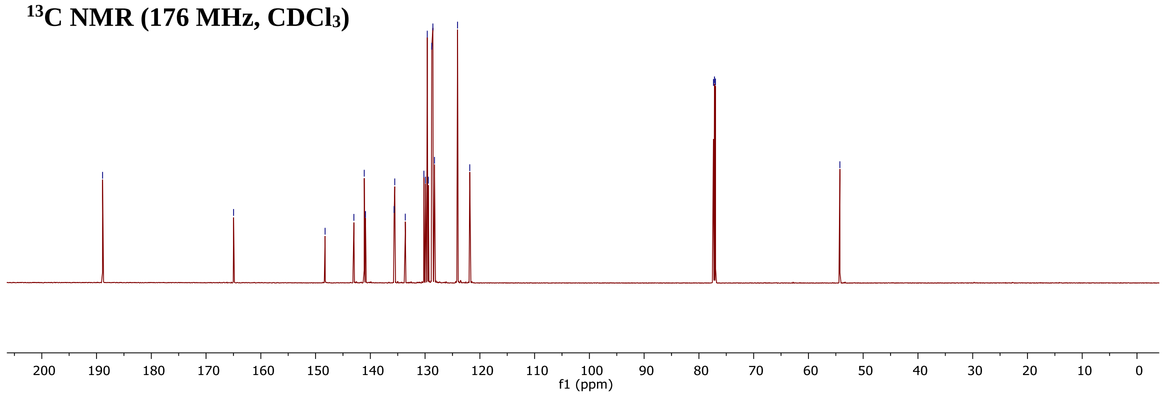

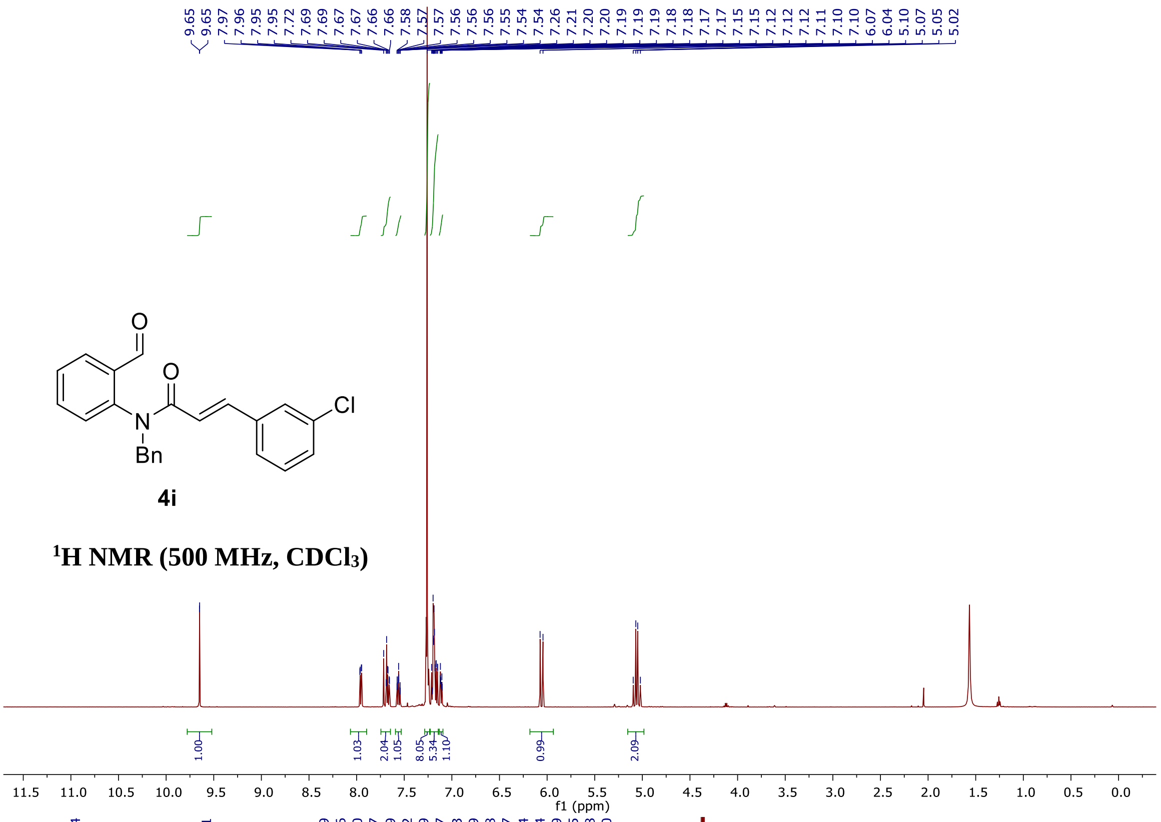
9.65 9.65 7.97 7.96 7.95 7.95 7.72 7.69 7.69 7.67 7.67 7.66 7.66 7.58 7.57 7.57 7.56 7.56 7.56 7.55 7.54 7.54 7.26 7.21 7.20 7.20 7.19 7.19 7.19 7.18 7.18 7.17 7.17 7.15 7.15 7.12 7.12 7.12 7.11 7.10 7.10 6.07 6.04 5.10 5.07 5.05 5.02
O
O
Cl
N
Bn
4i
¹H NMR (500 MHz, CDCl₃)
1.00
1.03
2.04
1.05
8.05
5.34
1.10
0.99
2.09
11.5 11.0 10.5 10.0 9.5 9.0 8.5 8.0 7.5 7.0 6.5 6.0 5.5 5.0 4.5 4.0 3.5 3.0 2.5 2.0 1.5 1.0 0.5 0.0
f1 (ppm)

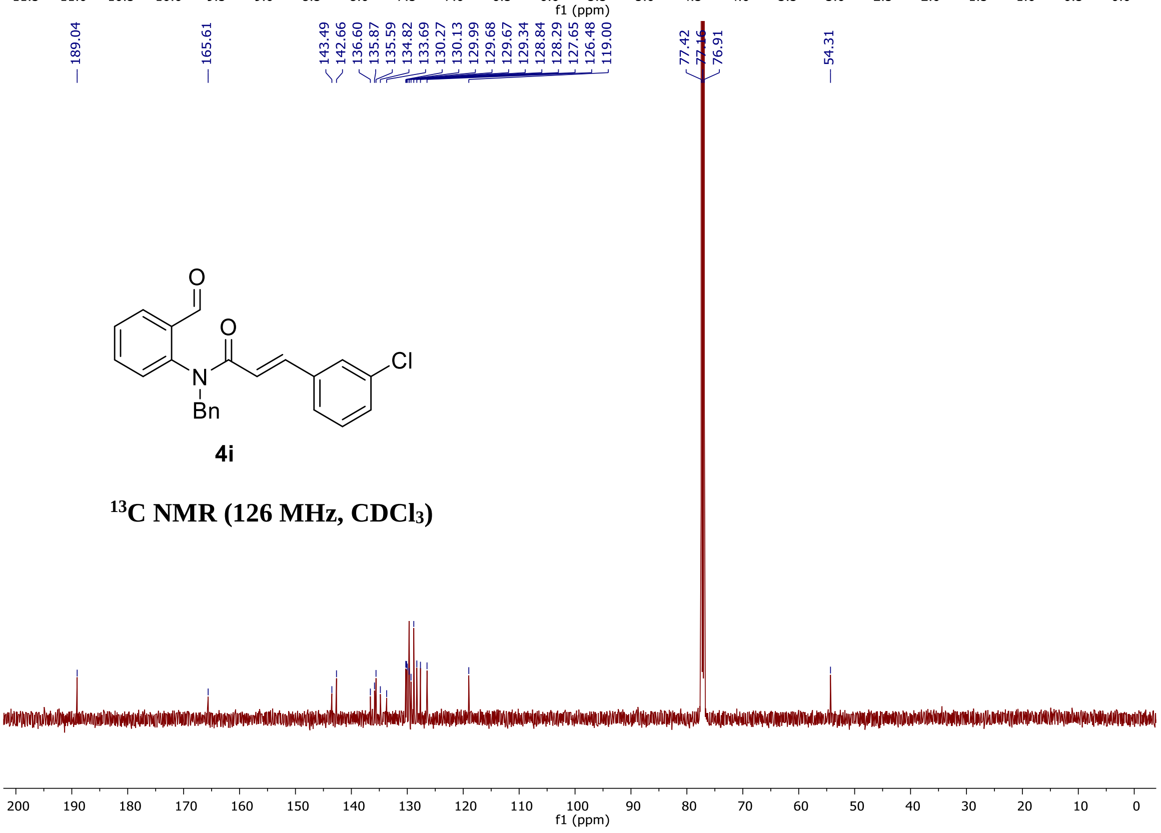
189.04
165.61
143.49 142.66 136.60 135.87 135.59 134.82 133.69 130.27 130.13 129.99 129.68 129.67 129.34 128.84 128.29 127.65 126.48 119.00
77.42 77.16 76.91
54.31
O
O
Cl
N
Bn
4i
¹³C NMR (126 MHz, CDCl₃)
200 190 180 170 160 150 140 130 120 110 100 90 80 70 60 50 40 30 20 10 0
f1 (ppm)

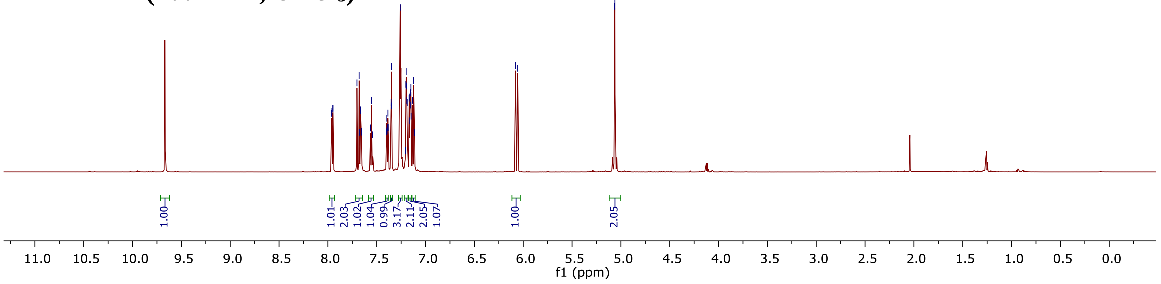

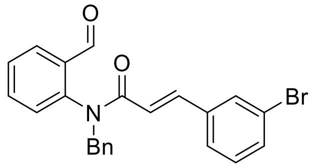

4j

^{1}H NMR (700 MHz, CDCl$_3$)

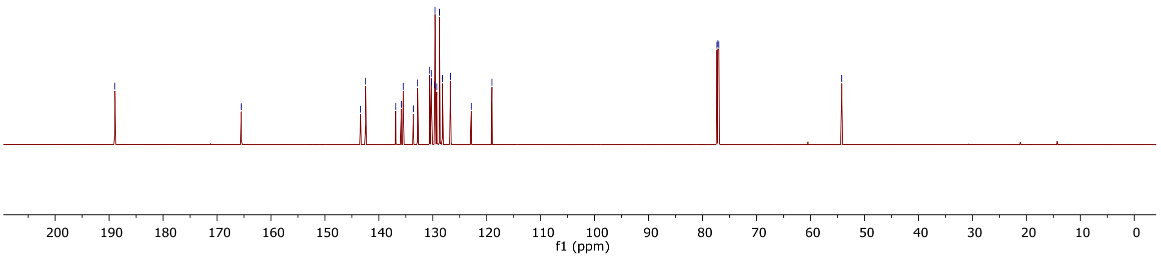

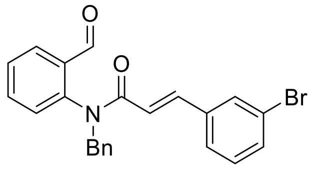

4j

^{13}C NMR (176 MHz, CDCl$_3$)

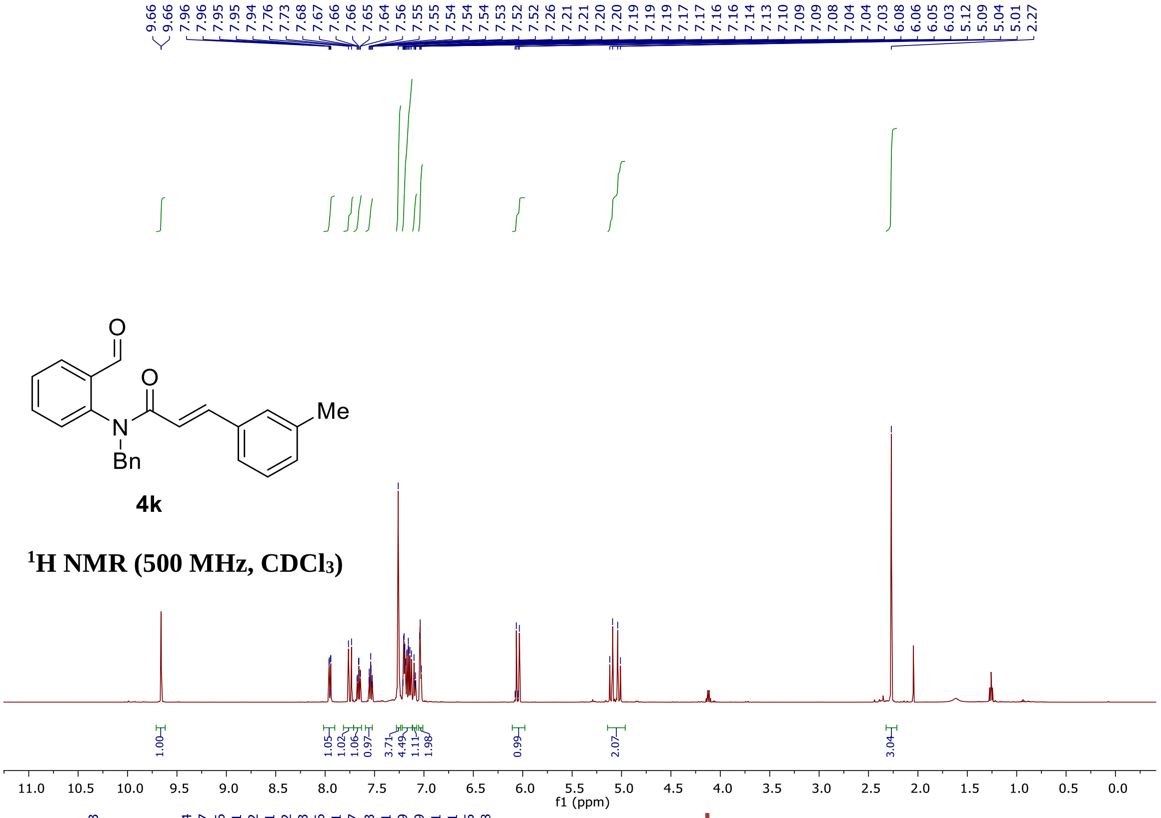
9.66 9.66 7.96 7.96 7.95 7.95 7.94 7.76 7.73 7.68 7.67 7.66 7.66 7.65 7.64 7.56 7.55 7.55 7.54 7.54 7.54 7.53 7.52 7.52 7.26 7.21 7.21 7.20 7.20 7.19 7.19 7.19 7.17 7.17 7.16 7.16 7.14 7.13 7.10 7.09 7.09 7.08 7.04 7.04 7.03 6.08 6.06 6.05 6.03 5.12 5.09 5.04 5.01 2.27
O
O
Me
N
Bn
4k
1H NMR (500 MHz, CDCl3)
1.00
1.05
1.02
1.06
0.97
3.71
4.49
1.11
1.98
0.99
2.07
3.04
f1 (ppm)
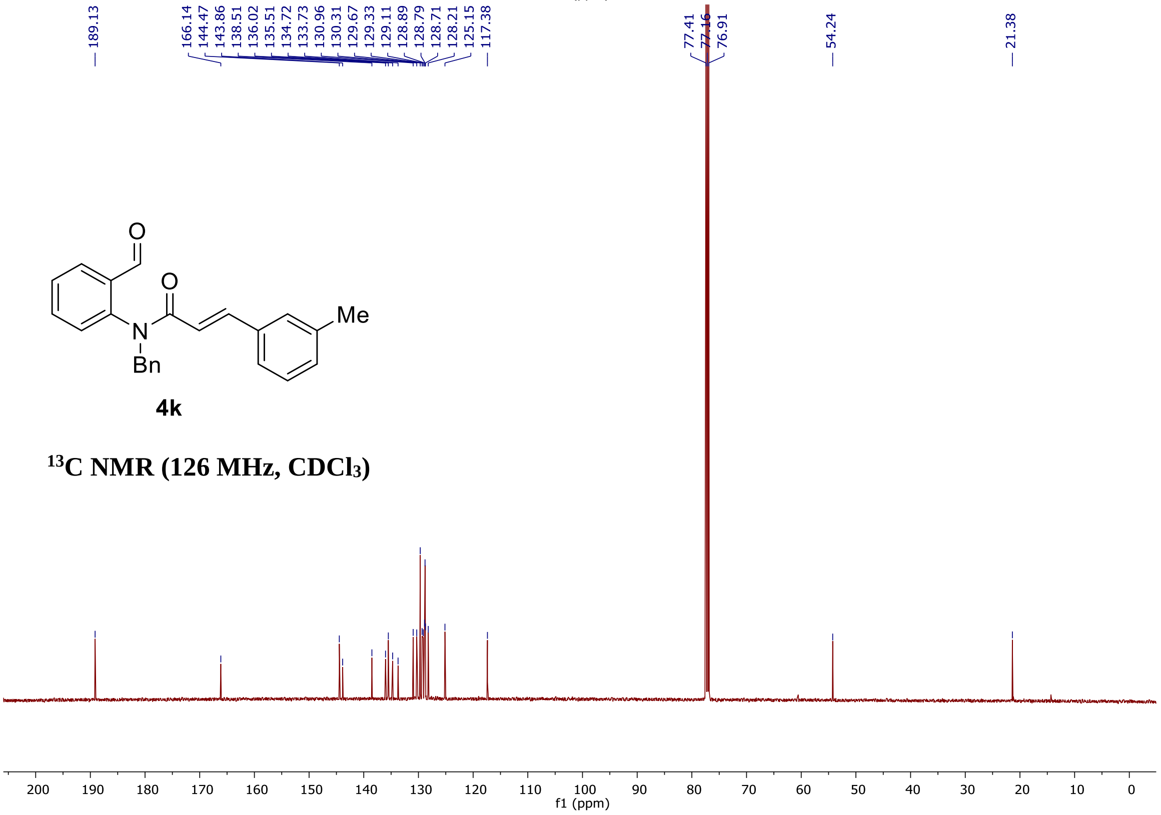
189.13
166.14 144.47 143.86 138.51 136.02 135.51 134.72 133.73 130.96 130.31 129.67 129.33 129.11 128.89 128.79 128.71 128.21 125.15 117.38
77.41 77.16 76.91
54.24
21.38
O
O
Me
N
Bn
4k
13C NMR (126 MHz, CDCl3)
f1 (ppm)

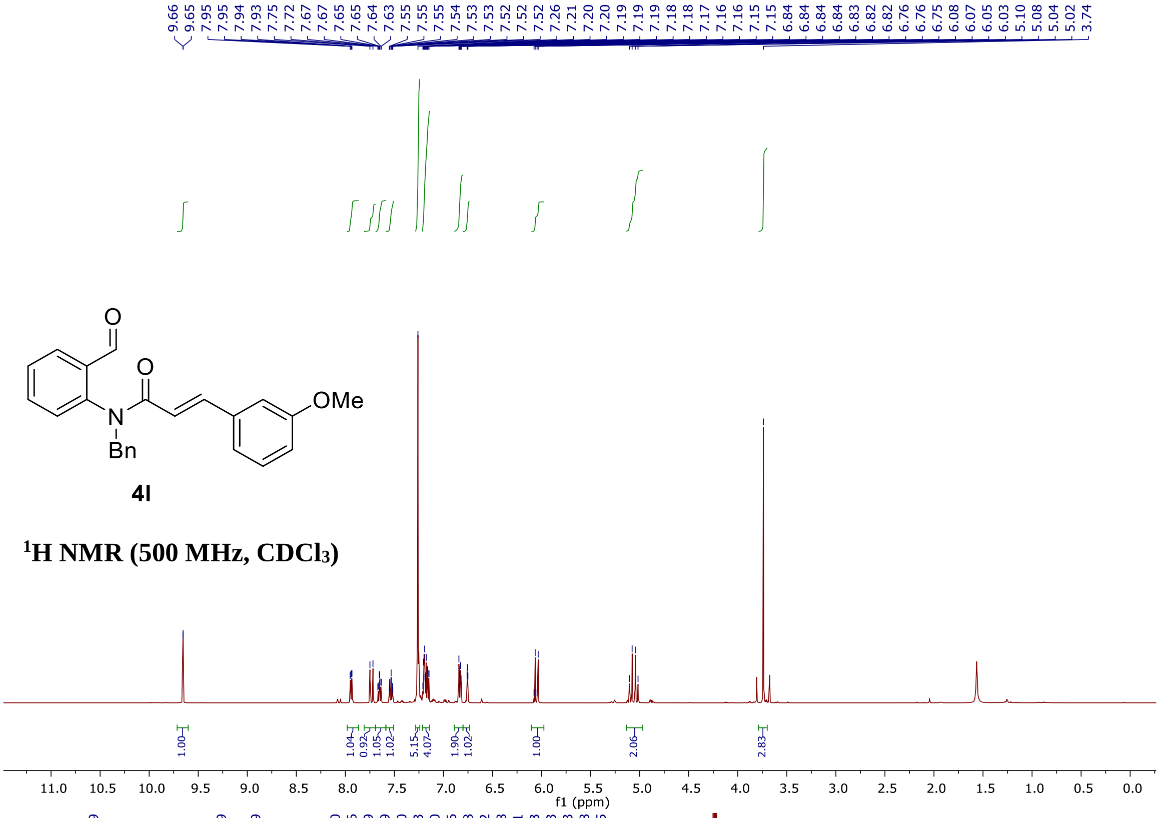

4l
^{1}H NMR (500 MHz, CDCl$_3$)

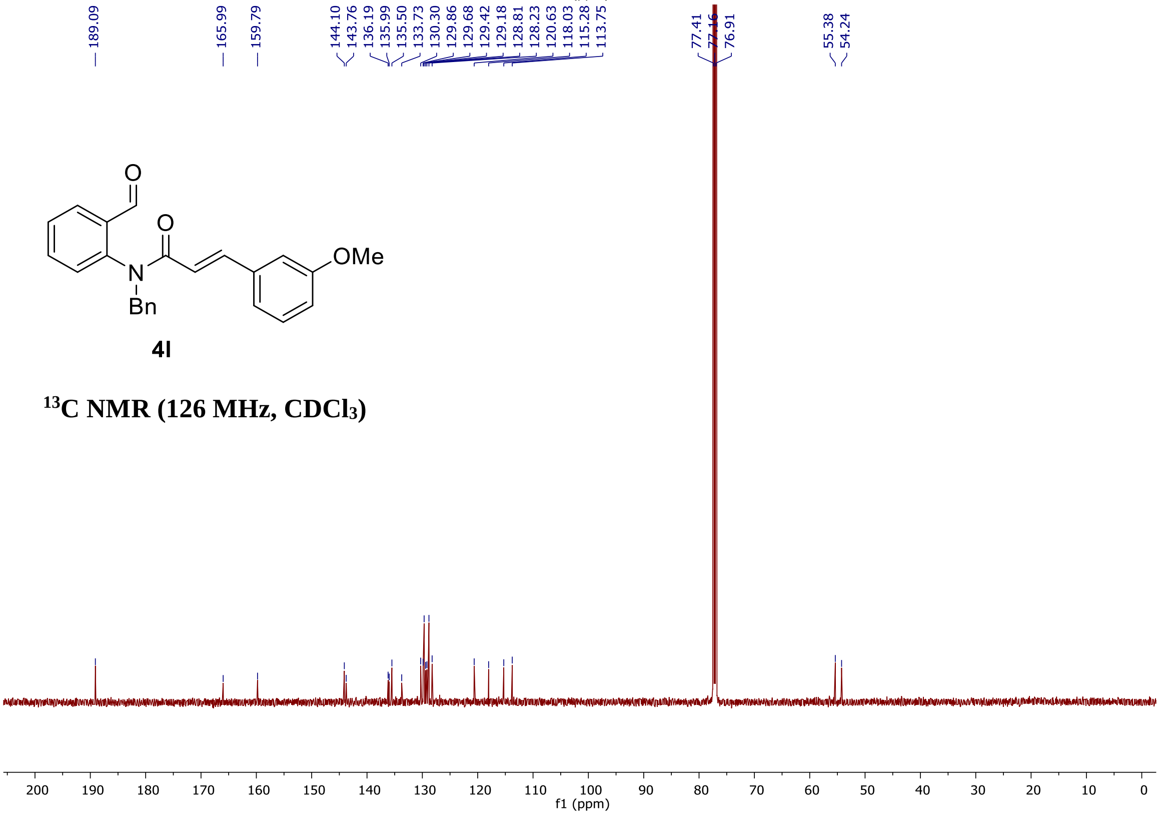

4l
^{13}C NMR (126 MHz, CDCl$_3$)

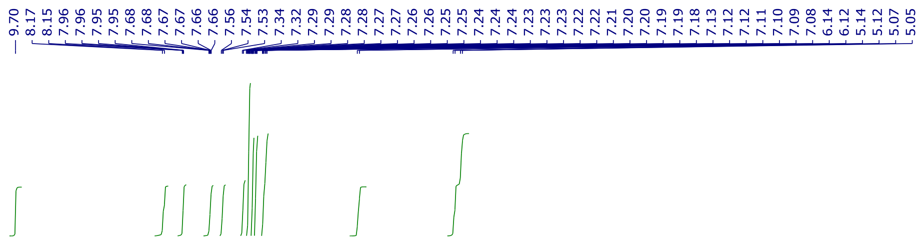

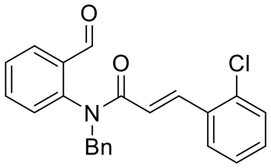

4m

^{1}H NMR (700 MHz, CDCl3)

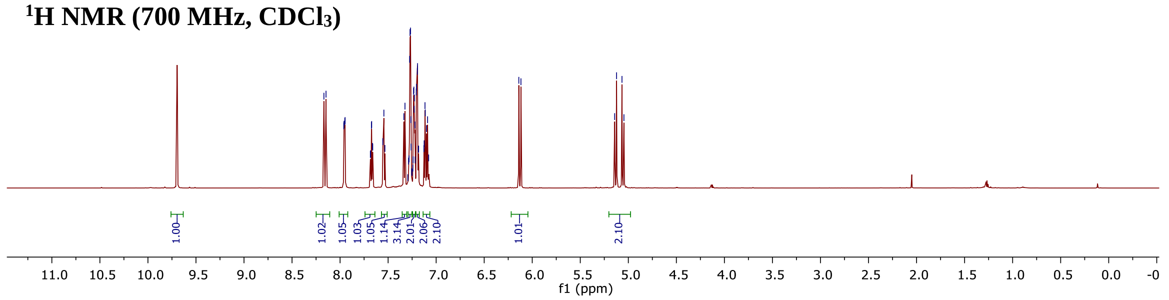

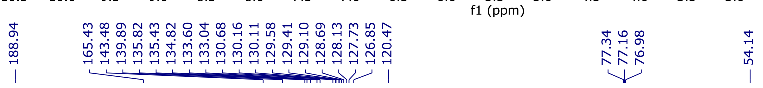

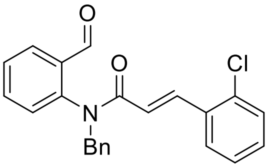

4m

^{13}C NMR (176 MHz, CDCl3)

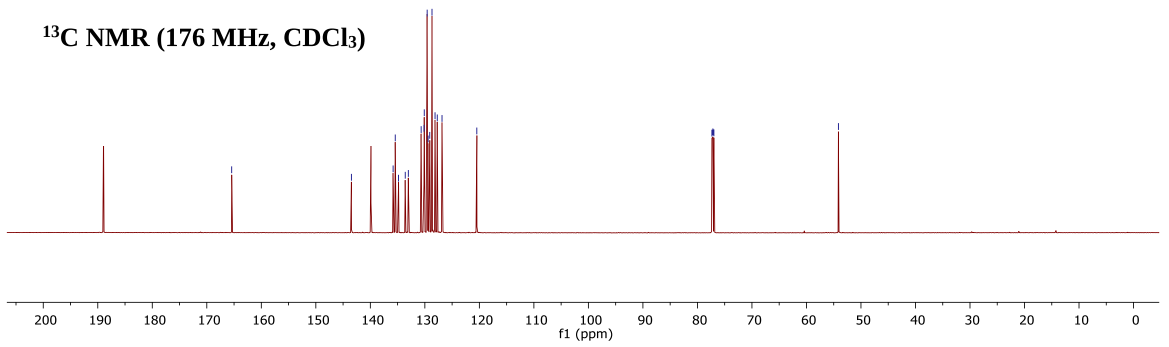

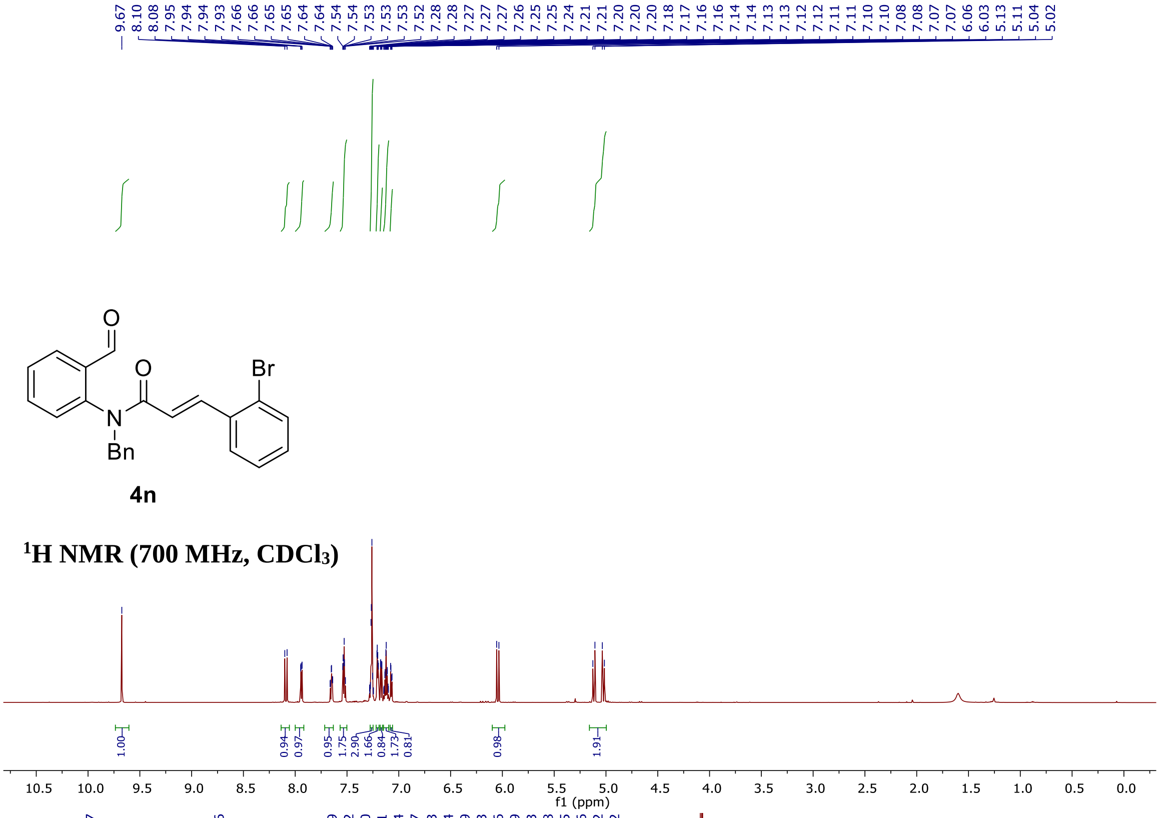

Br
O
O
N
Bn
4n
^{1}H NMR (700 MHz, CDCl$_3$)
f1 (ppm)

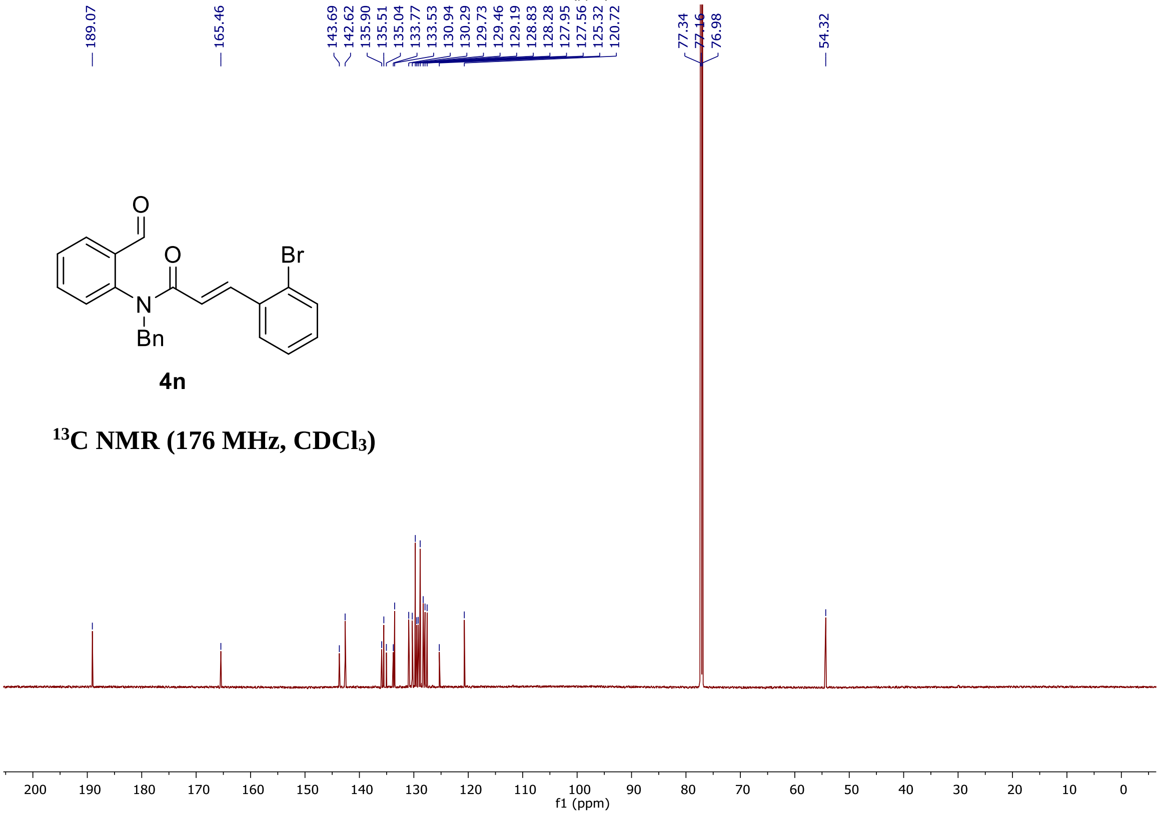

Br
O
O
N
Bn
4n
^{13}C NMR (176 MHz, CDCl$_3$)
f1 (ppm)

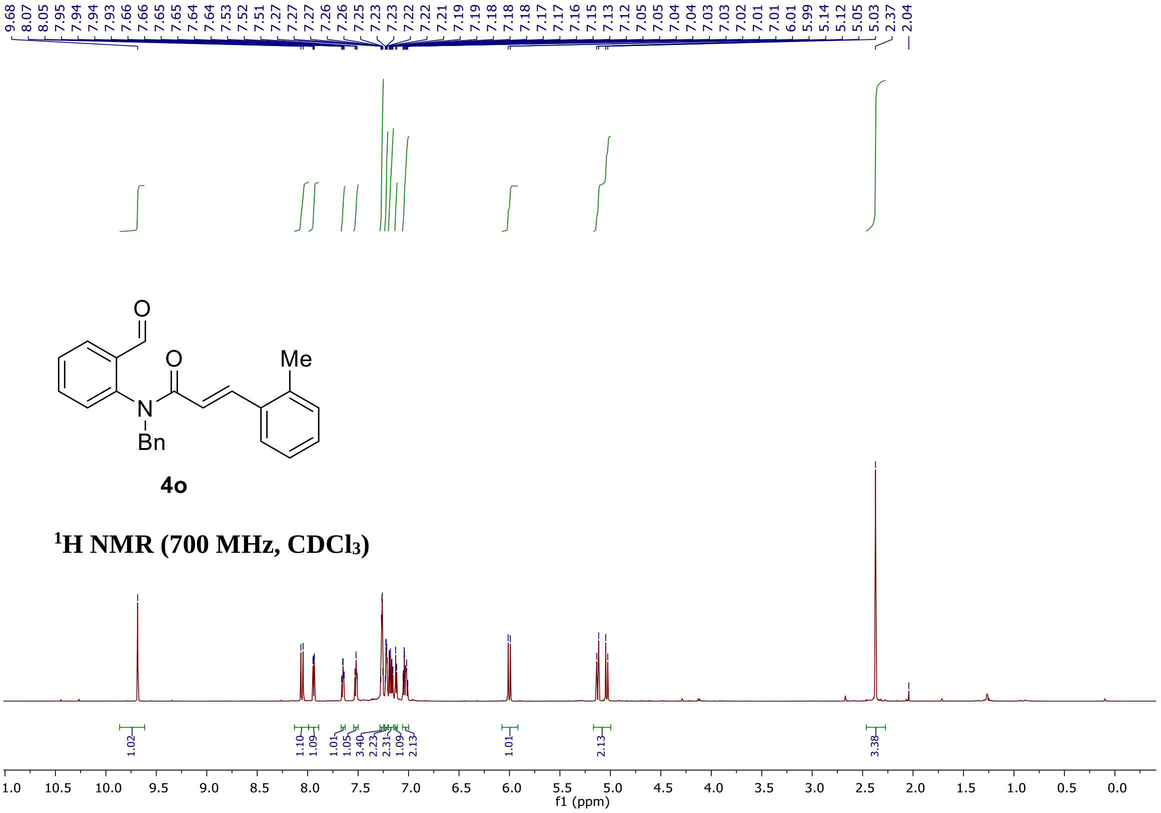

4o

^{1}H NMR (700 MHz, CDCl$_3$)

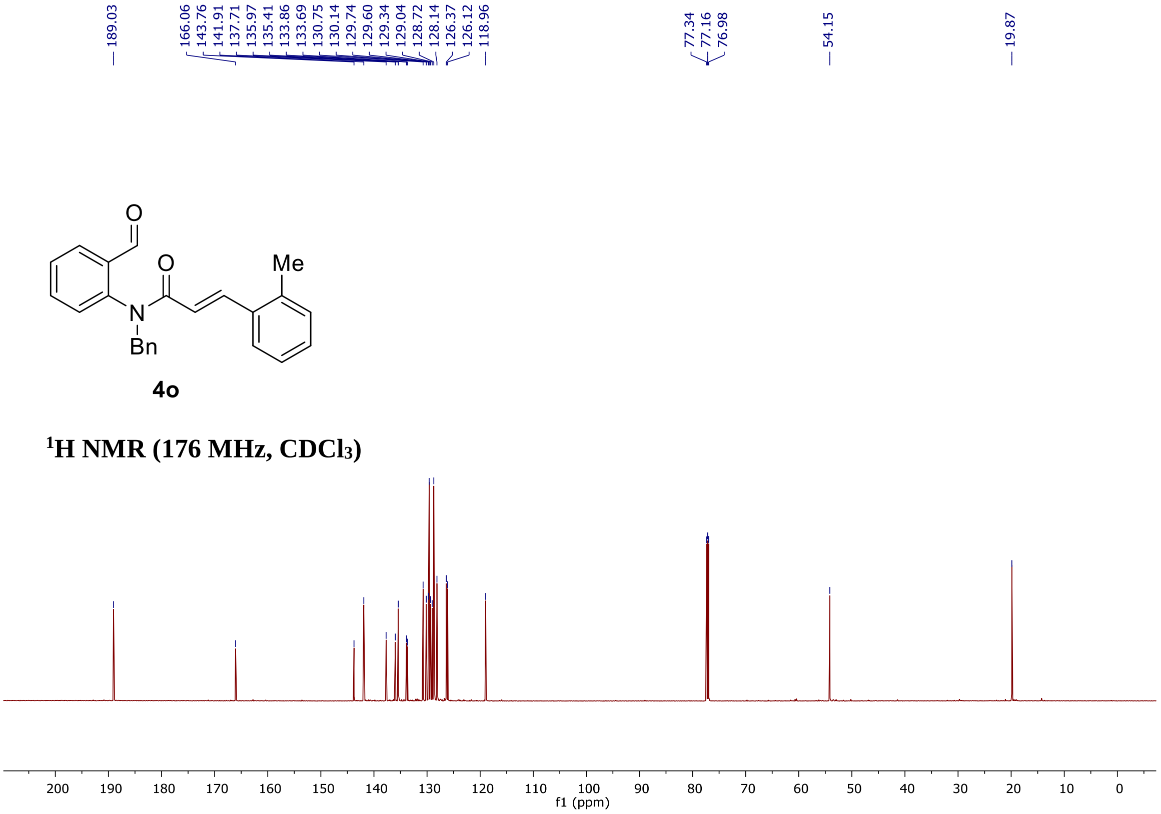

4o

^{1}H NMR (176 MHz, CDCl$_3$)

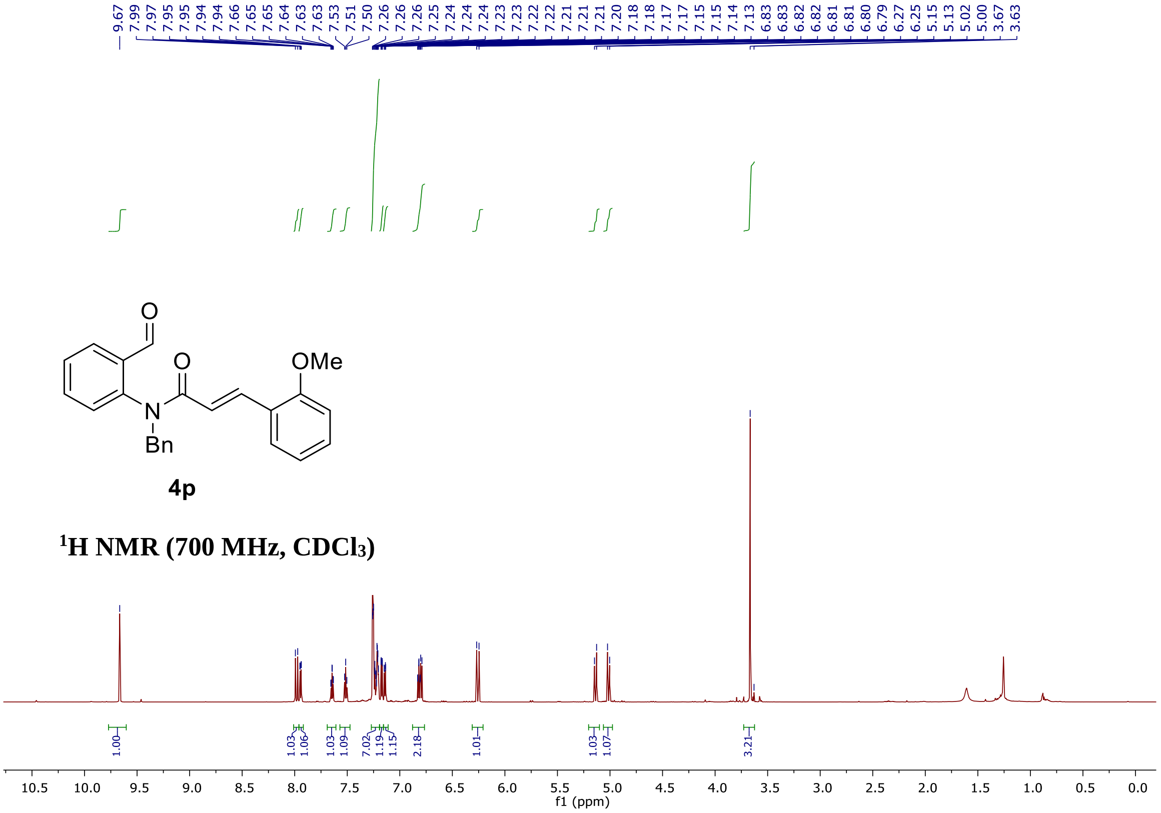

4p

^{1}H NMR (700 MHz, $CDCl_3$)

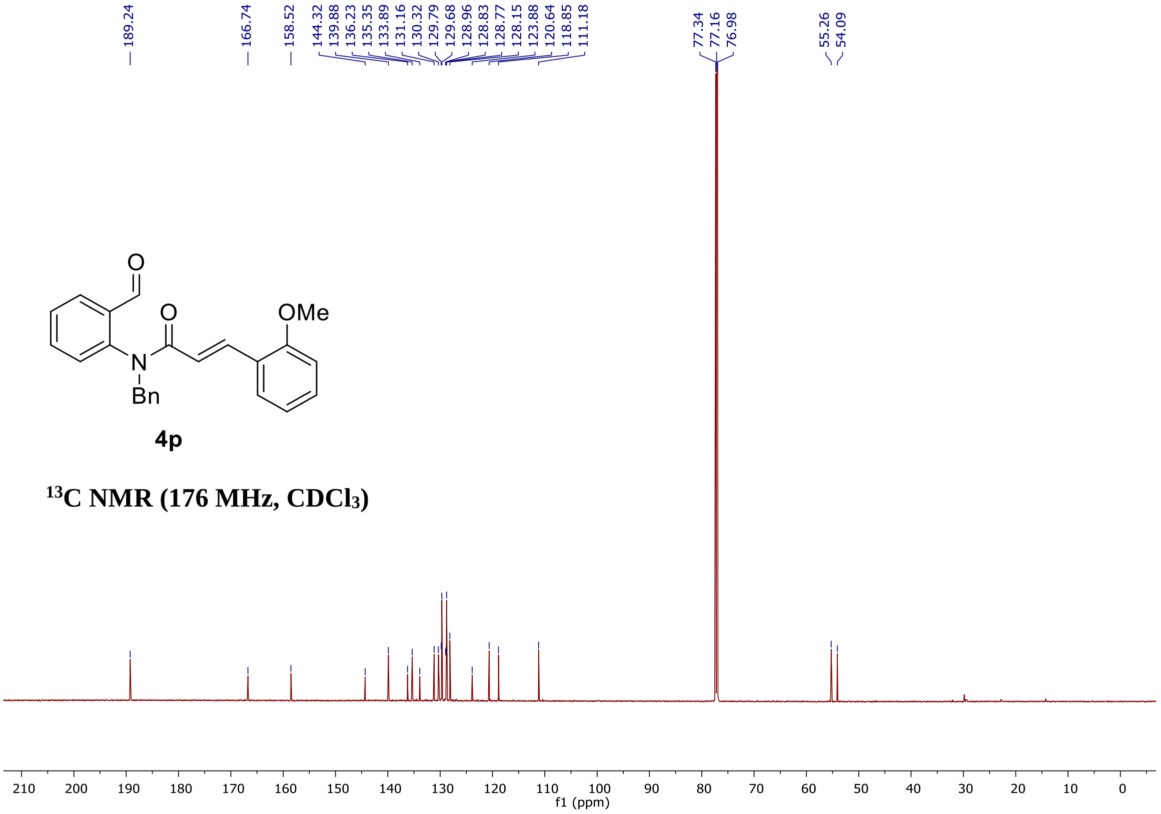

4p

^{13}C NMR (176 MHz, $CDCl_3$)

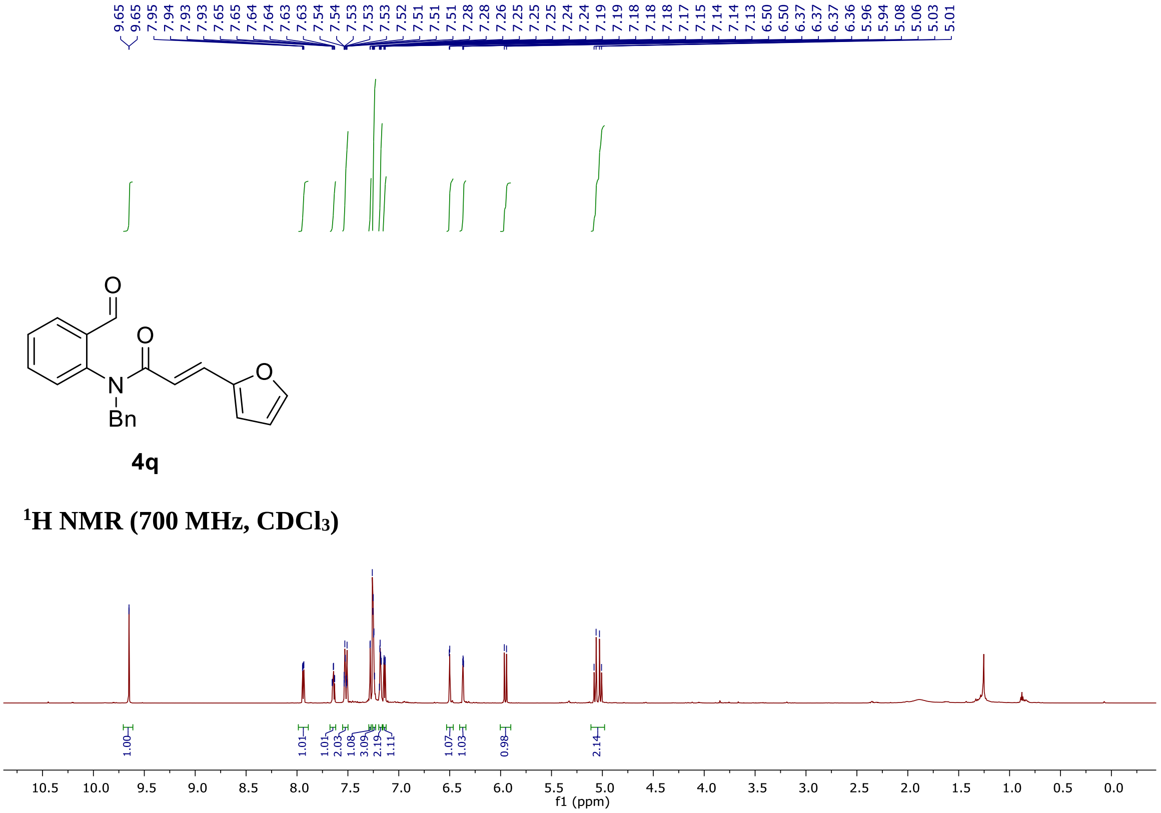

4q

^{1}H NMR (700 MHz, $CDCl_3$)

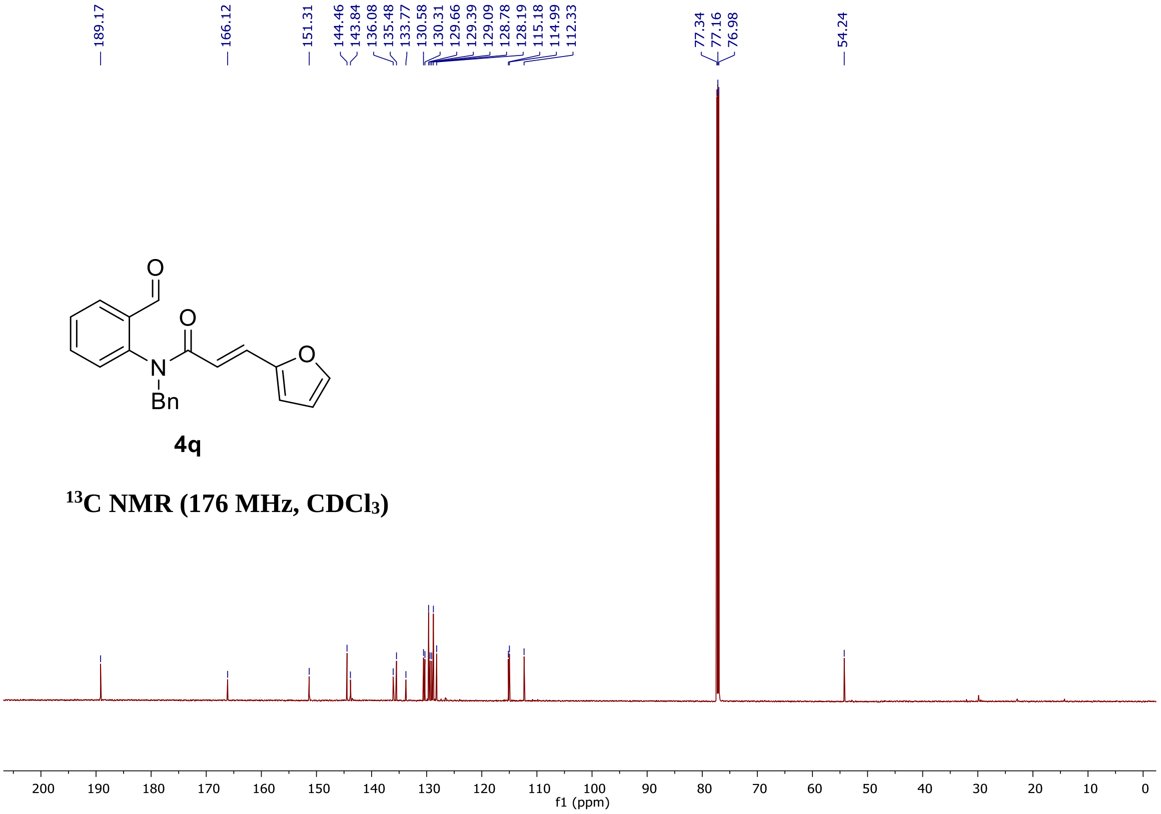

4q

^{13}C NMR (176 MHz, $CDCl_3$)

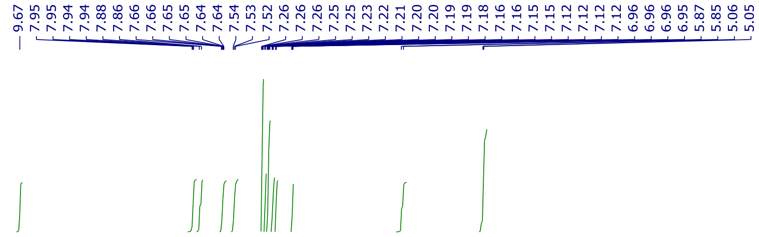

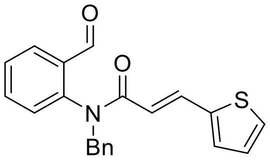

4r

^{1}H NMR (700 MHz, CDCl$_3$)

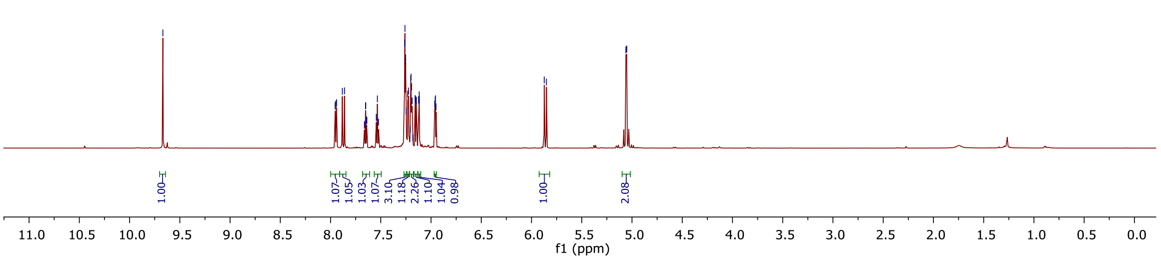

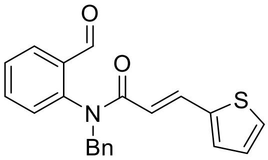

4r

^{13}C NMR (176 MHz, CDCl$_3$)

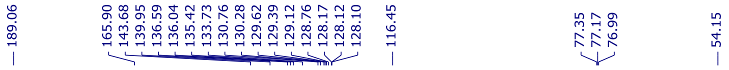
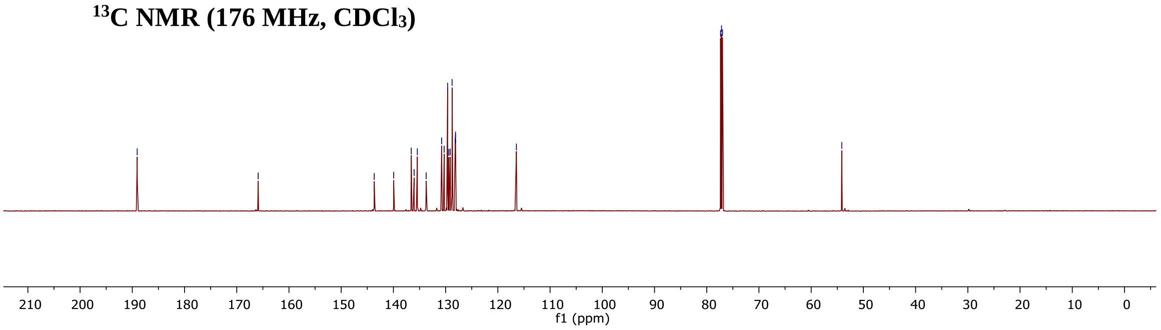

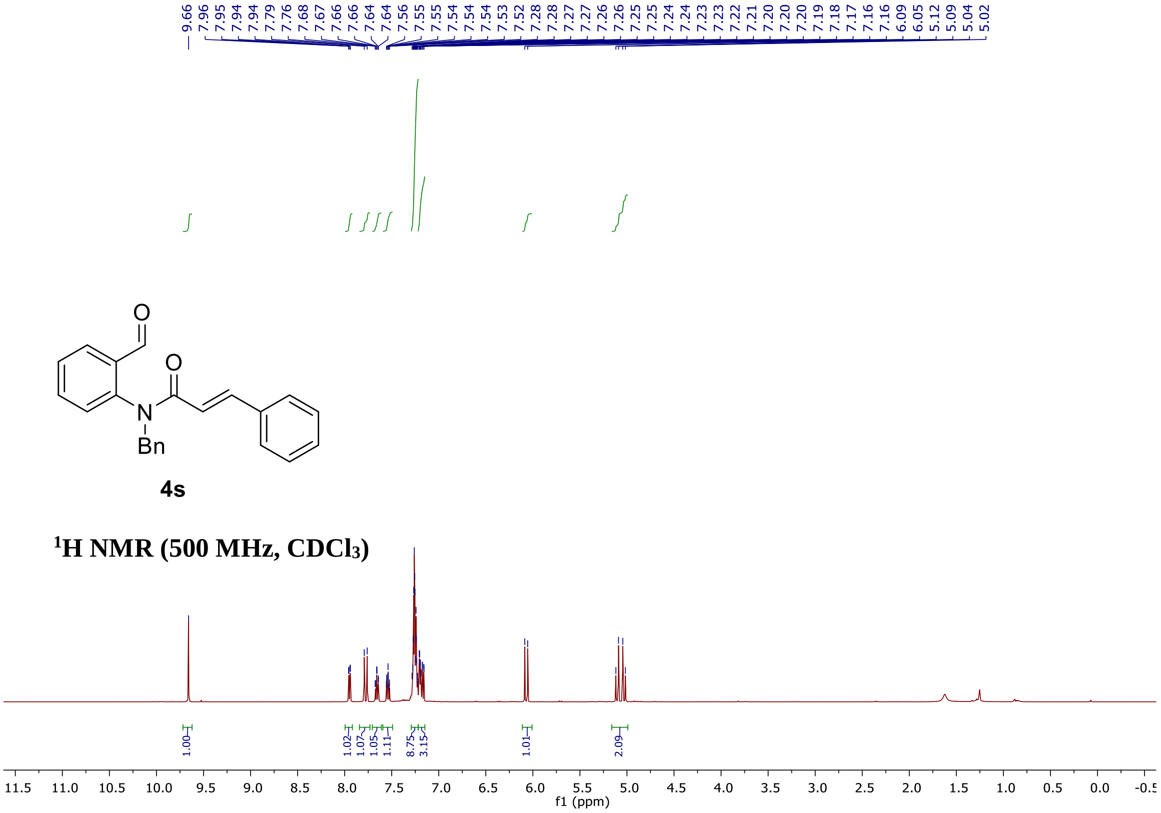

4s

^{1}H NMR (500 MHz, CDCl3)

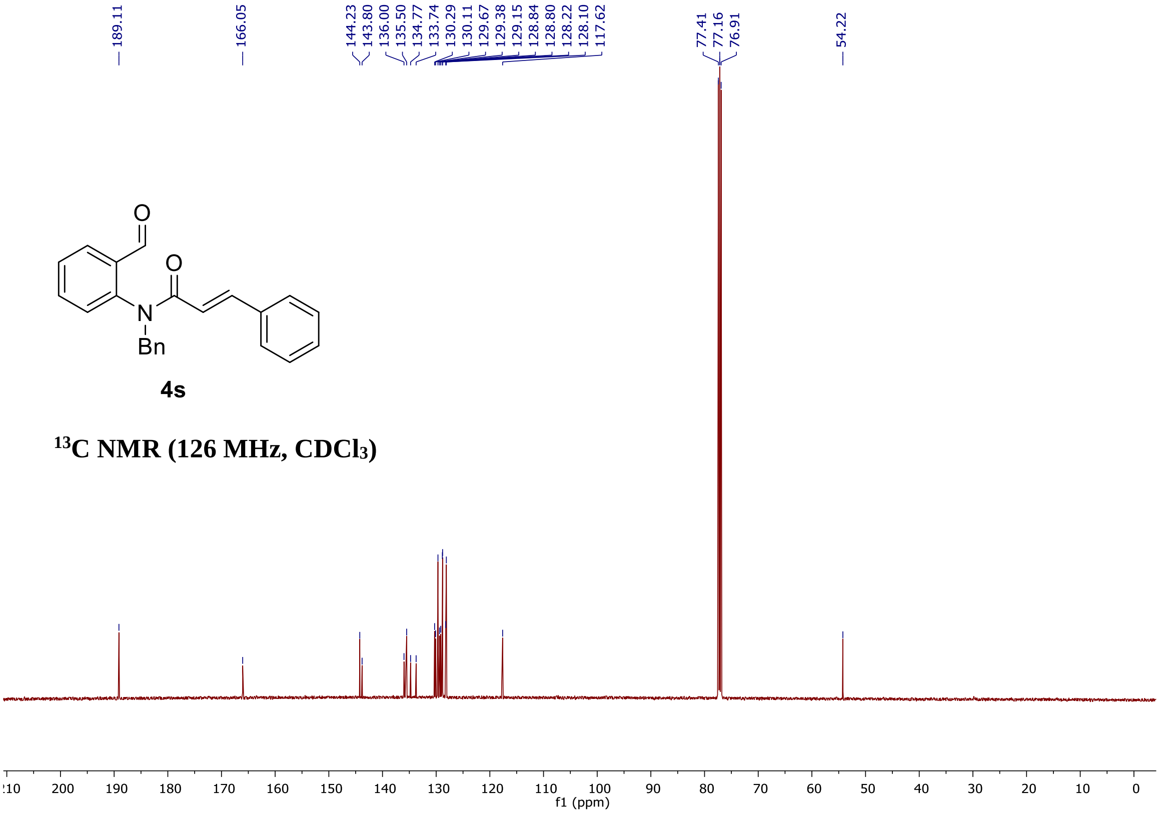

4s

^{13}C NMR (126 MHz, CDCl3)

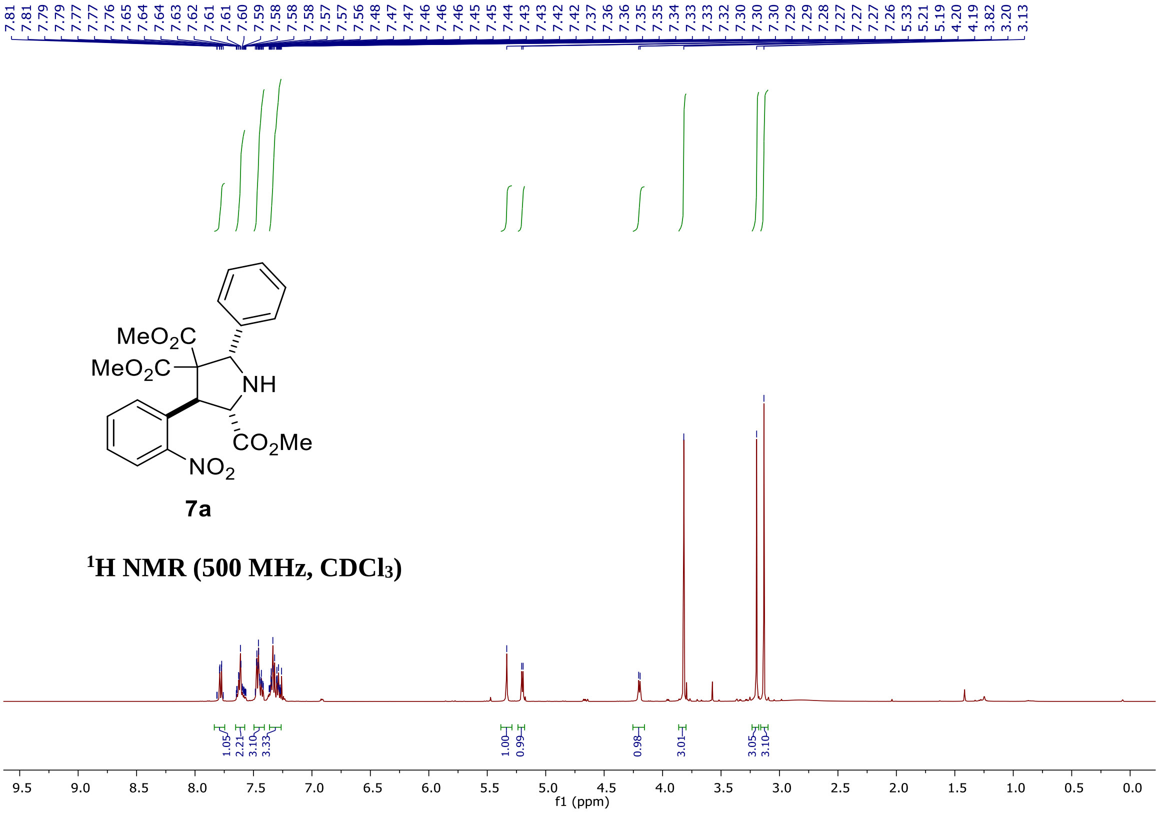

^{1}H NMR (500 MHz, CDCl3)

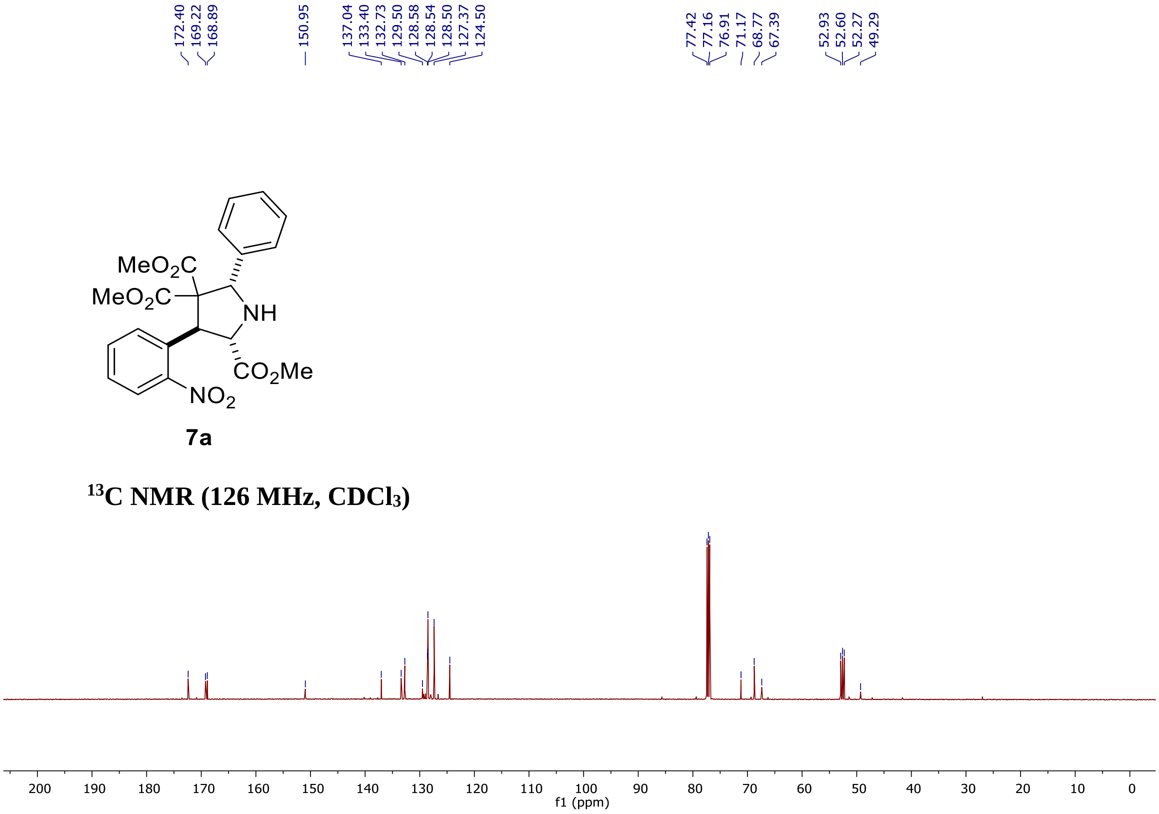

^{13}C NMR (126 MHz, CDCl3)

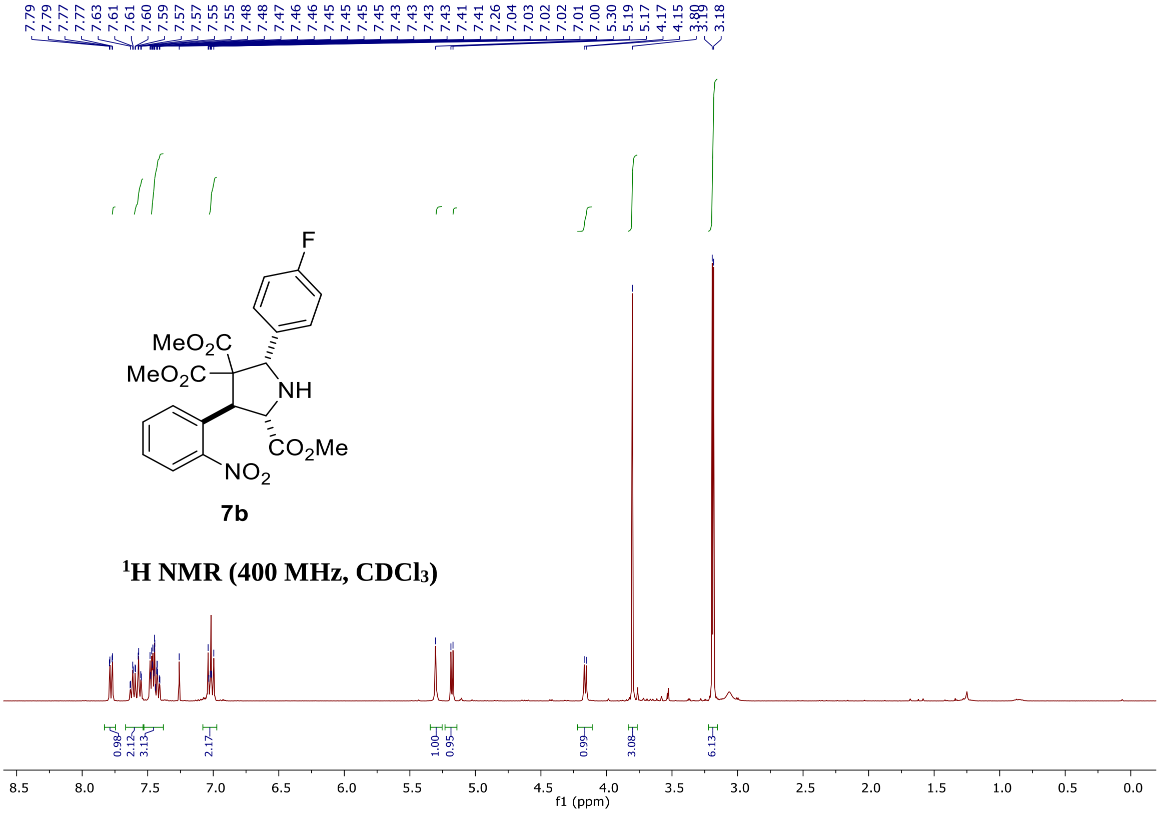

7b

^{1}H NMR (400 MHz, CDCl3)

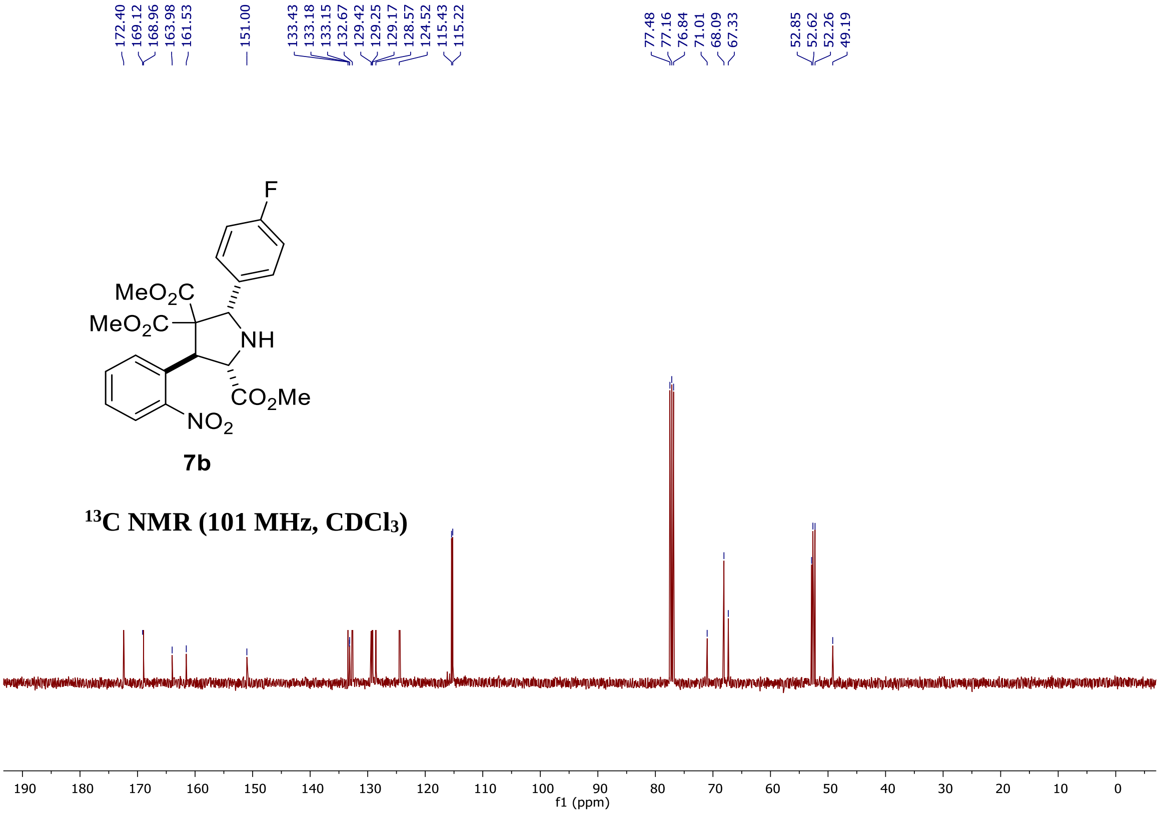

7b

^{13}C NMR (101 MHz, CDCl3)

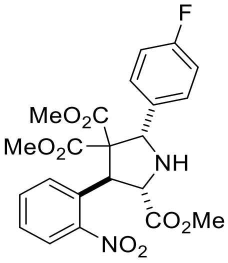

7b

^{19}F NMR (470 MHz, CDCl3)

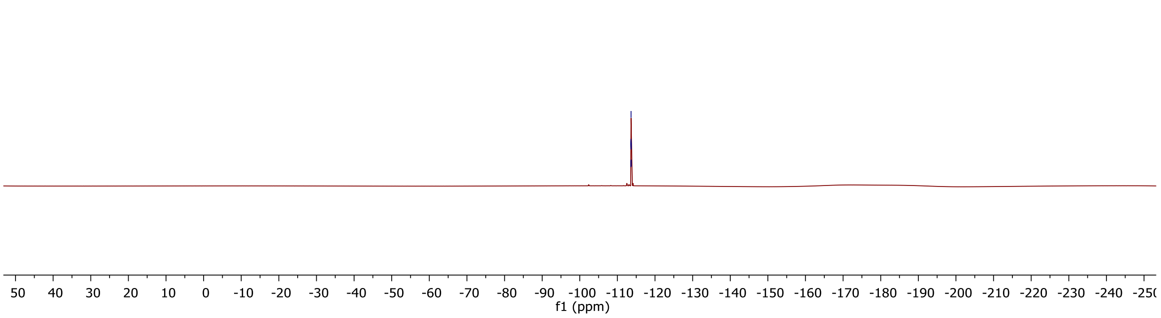

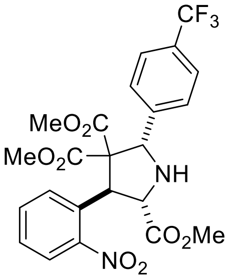

7c

^{1}H NMR (400 MHz, CDCl3)

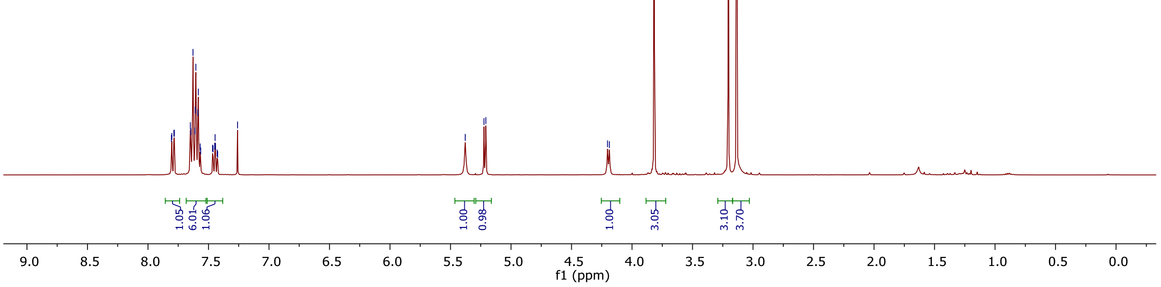

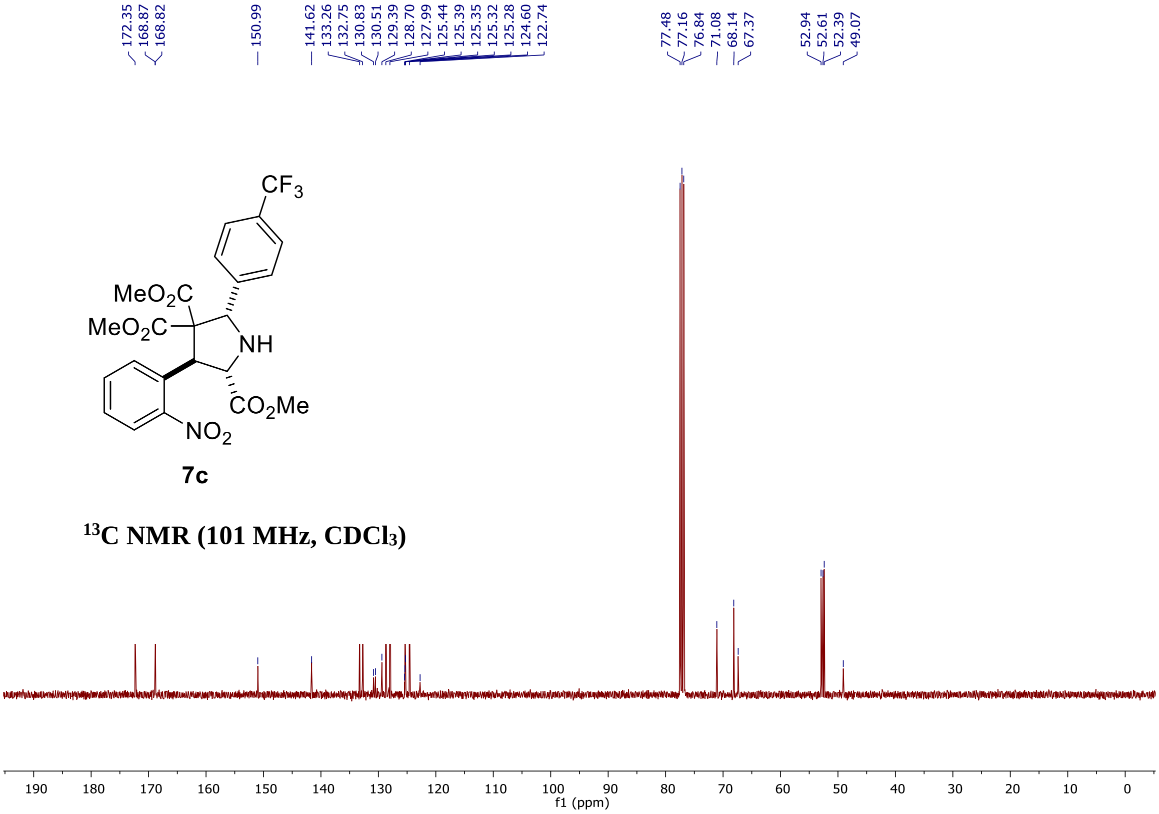

7c

^{13}C NMR (101 MHz, CDCl3)

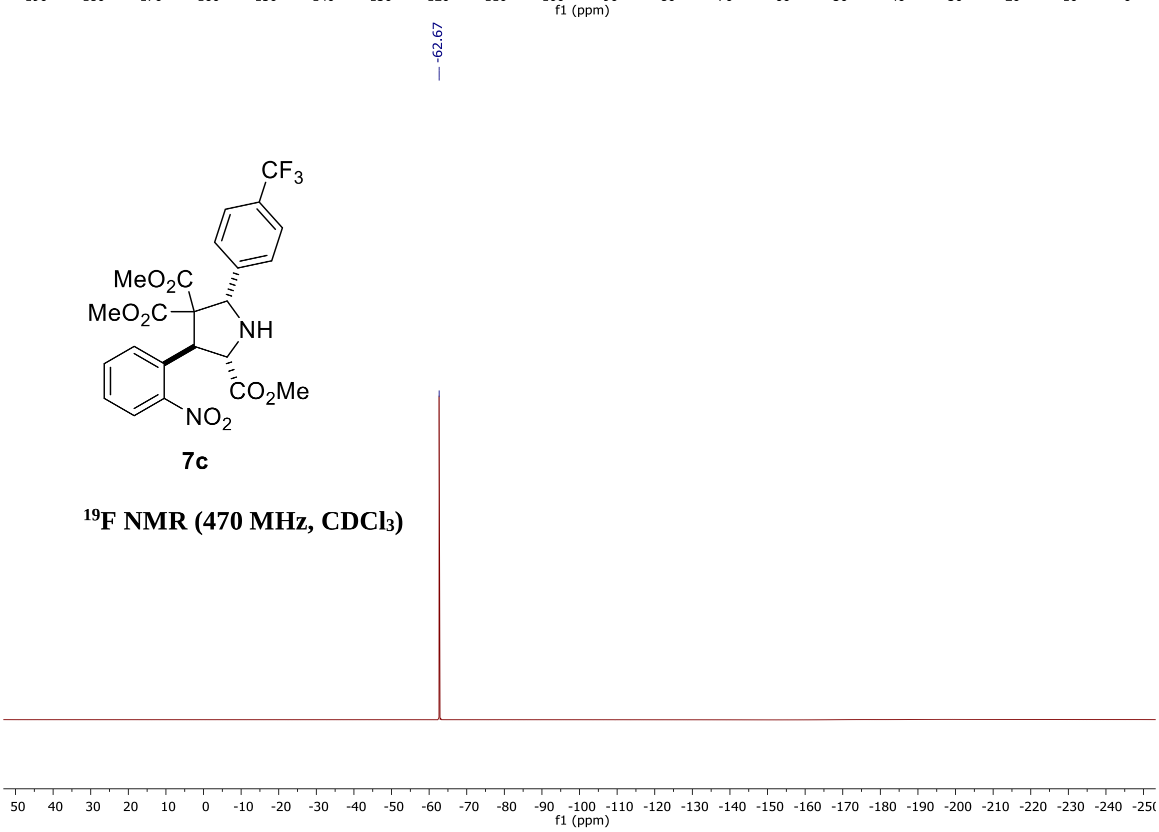

7c

^{19}F NMR (470 MHz, CDCl3)

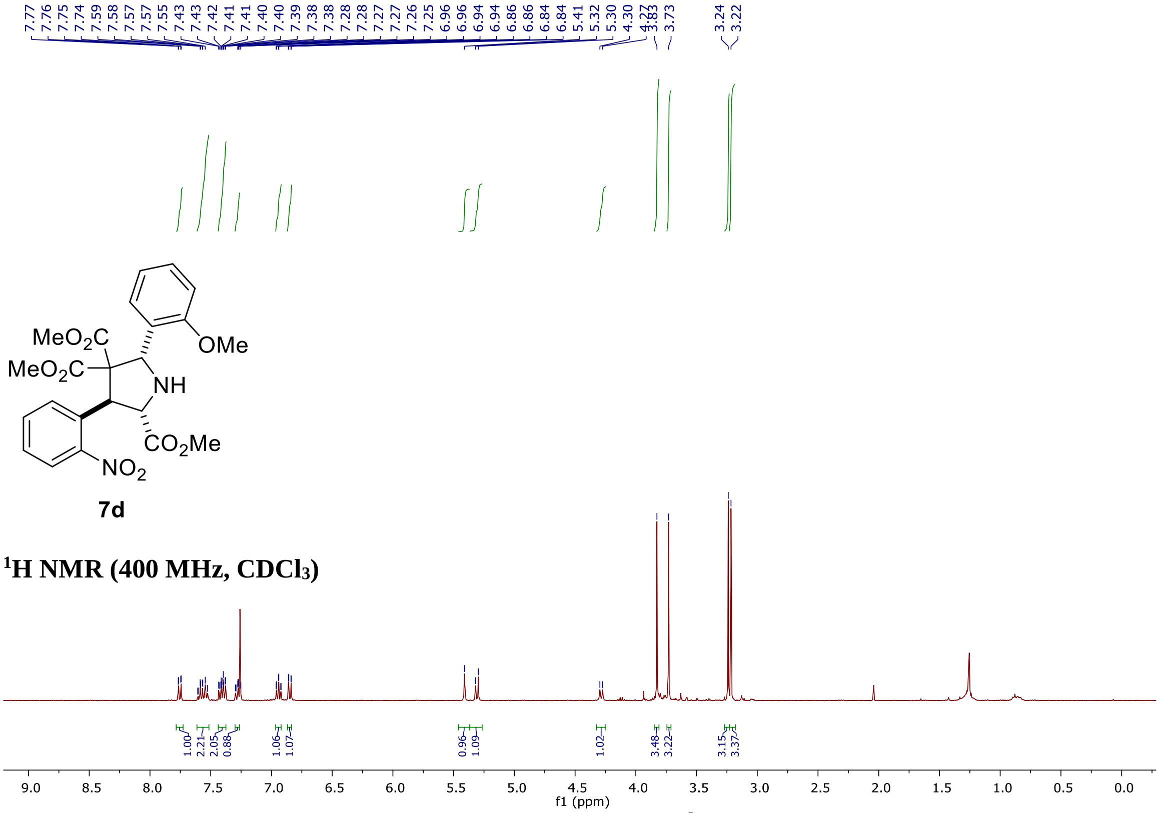
7.77
7.76
7.75
7.74
7.59
7.58
7.57
7.57
7.55
7.43
7.43
7.42
7.41
7.41
7.40
7.39
7.38
7.38
7.28
7.28
7.27
7.27
7.26
7.25
6.96
6.96
6.94
6.94
6.86
6.86
6.84
6.84
5.41
5.32
5.30
4.30
4.27
3.83
3.73
3.24
3.22
MeO2C
MeO2C
OMe
NH
CO2Me
NO2
7d
1H NMR (400 MHz, CDCl3)
1.00
2.21
2.05
0.88
1.06
1.07
0.96
1.09
1.02
3.48
3.22
3.15
3.37
9.0 8.5 8.0 7.5 7.0 6.5 6.0 5.5 5.0 4.5 4.0 3.5 3.0 2.5 2.0 1.5 1.0 0.5 0.0
f1 (ppm)

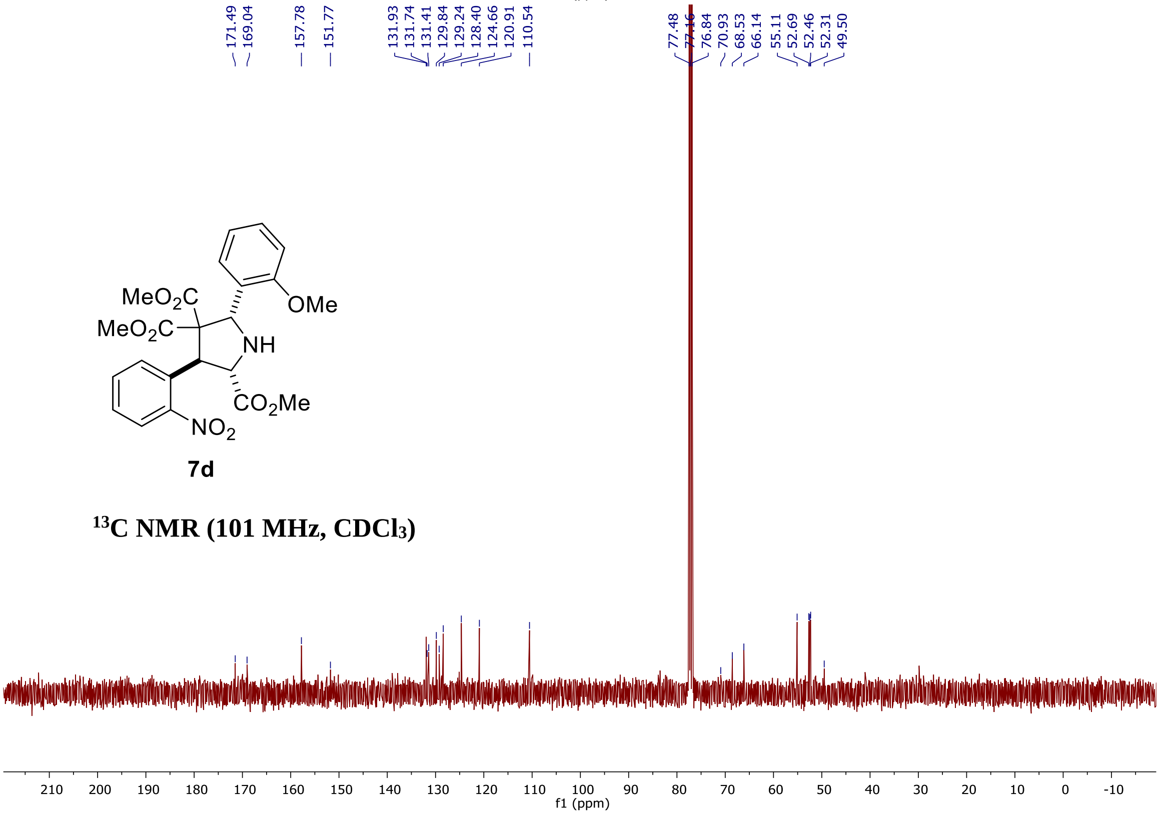
171.49
169.04
157.78
151.77
131.93
131.74
131.41
129.84
129.24
128.40
124.66
120.91
110.54
77.48
76.84
70.93
68.53
66.14
55.11
52.69
52.46
52.31
49.50
MeO2C
MeO2C
OMe
NH
CO2Me
NO2
7d
13C NMR (101 MHz, CDCl3)
210 200 190 180 170 160 150 140 130 120 110 100 90 80 70 60 50 40 30 20 10 0 -10
f1 (ppm)

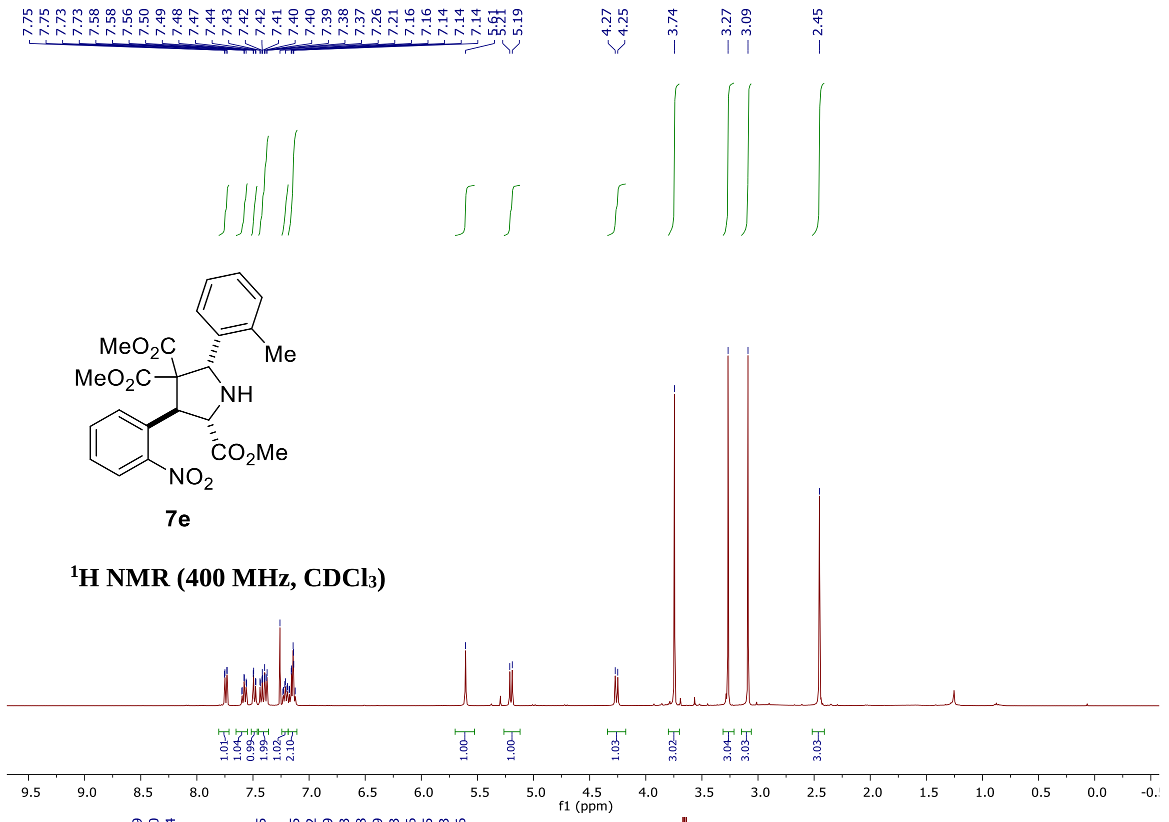
MeO_2C
MeO_2C
Me
NH
CO_2Me
NO_2
7e
^{1}H NMR (400 MHz, CDCl$_3$)
f1 (ppm)

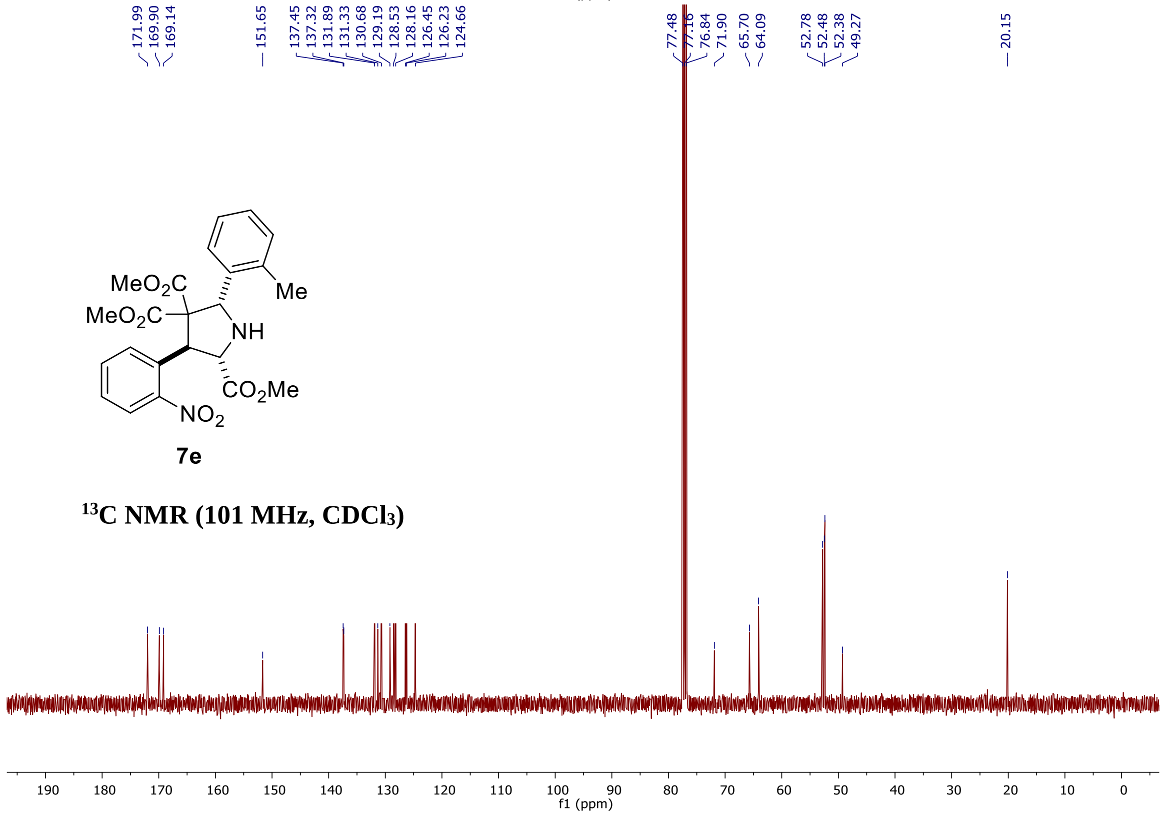
MeO_2C
MeO_2C
Me
NH
CO_2Me
NO_2
7e
^{13}C NMR (101 MHz, CDCl$_3$)
f1 (ppm)

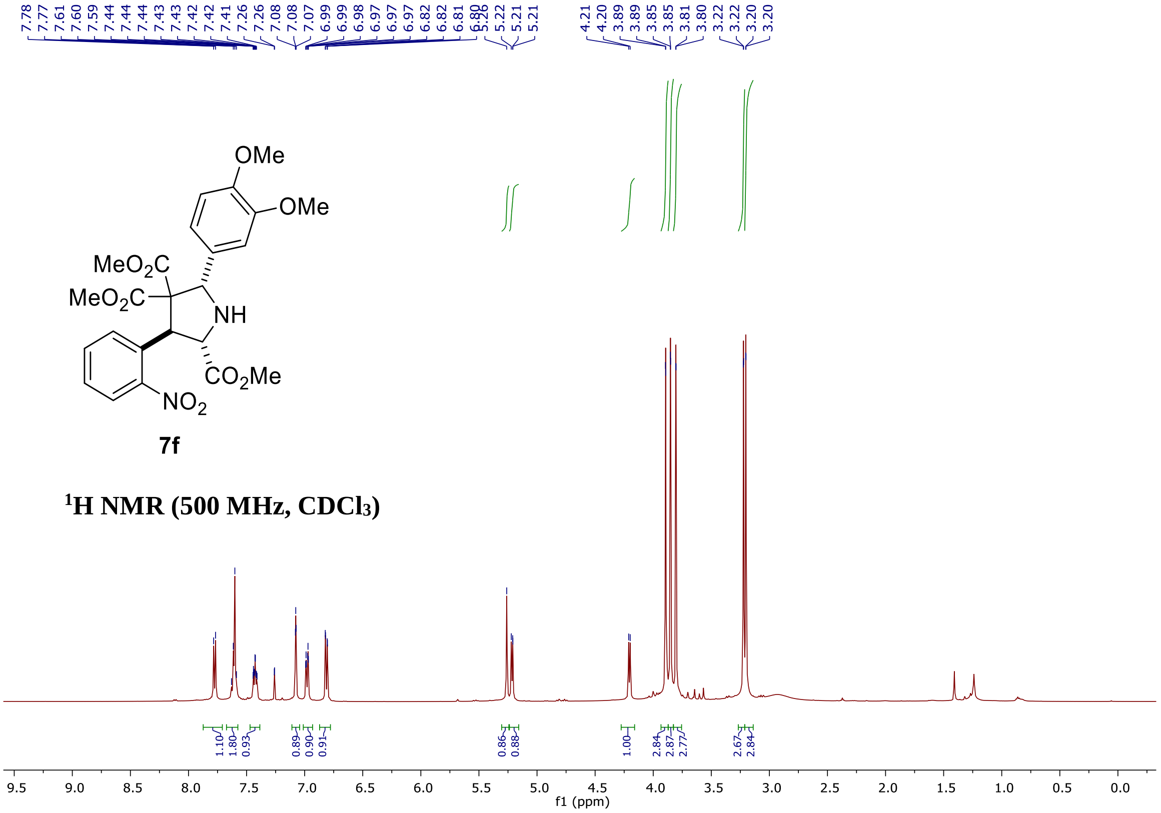

7.78
7.77
7.61
7.60
7.59
7.44
7.44
7.43
7.43
7.42
7.42
7.41
7.26
7.08
7.08
7.07
6.99
6.99
6.98
6.97
6.97
6.82
6.82
6.81
6.80
5.26
5.22
5.21
5.21
4.21
4.20
3.89
3.89
3.85
3.85
3.81
3.80
3.22
3.22
3.20
3.20
OMe
OMe
MeO2C
MeO2C
NH
CO2Me
NO2
7f
1H NMR (500 MHz, CDCl3)
1.10
1.80
0.93
0.89
0.90
0.91
0.86
0.88
1.00
2.84
2.87
2.77
2.67
2.84
f1 (ppm)

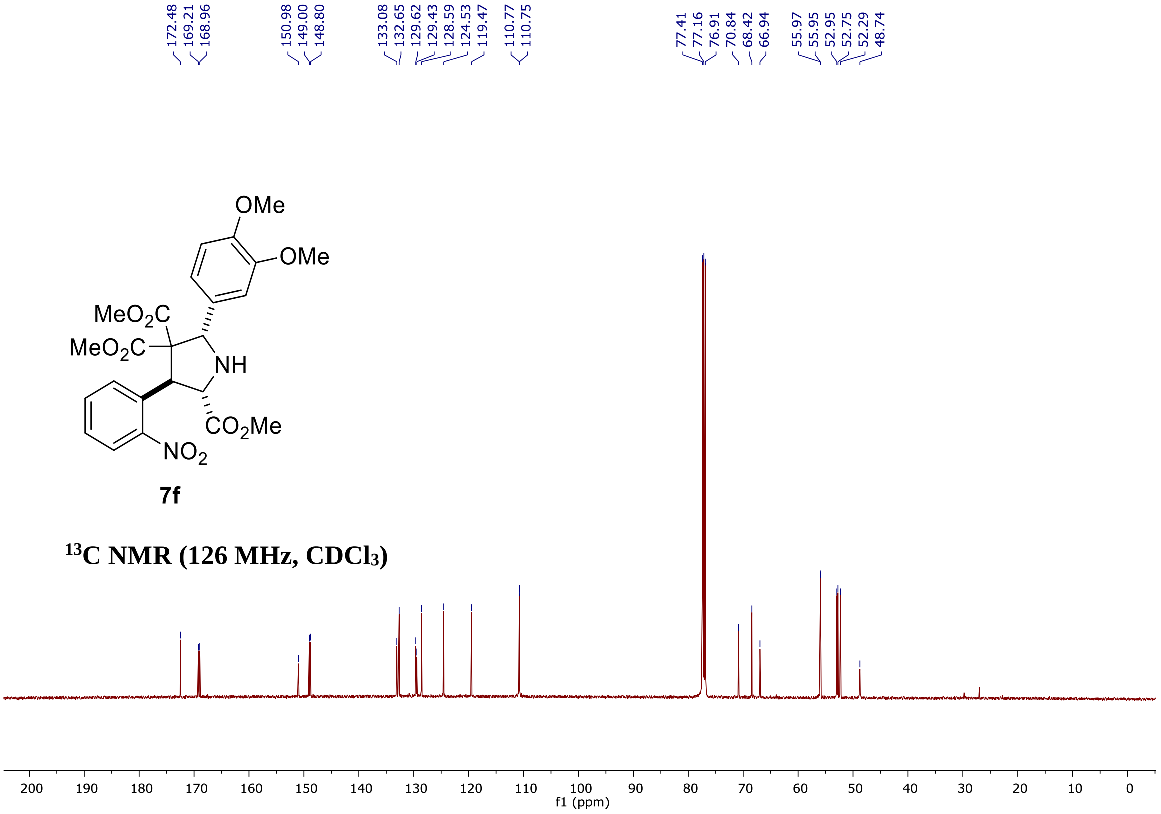

172.48
169.21
168.96
150.98
149.00
148.80
133.08
132.65
129.62
129.43
128.59
124.53
119.47
110.77
110.75
77.41
77.16
76.91
70.84
68.42
66.94
55.97
55.95
52.95
52.75
52.29
48.74
OMe
OMe
MeO2C
MeO2C
NH
CO2Me
NO2
7f
13C NMR (126 MHz, CDCl3)
f1 (ppm)

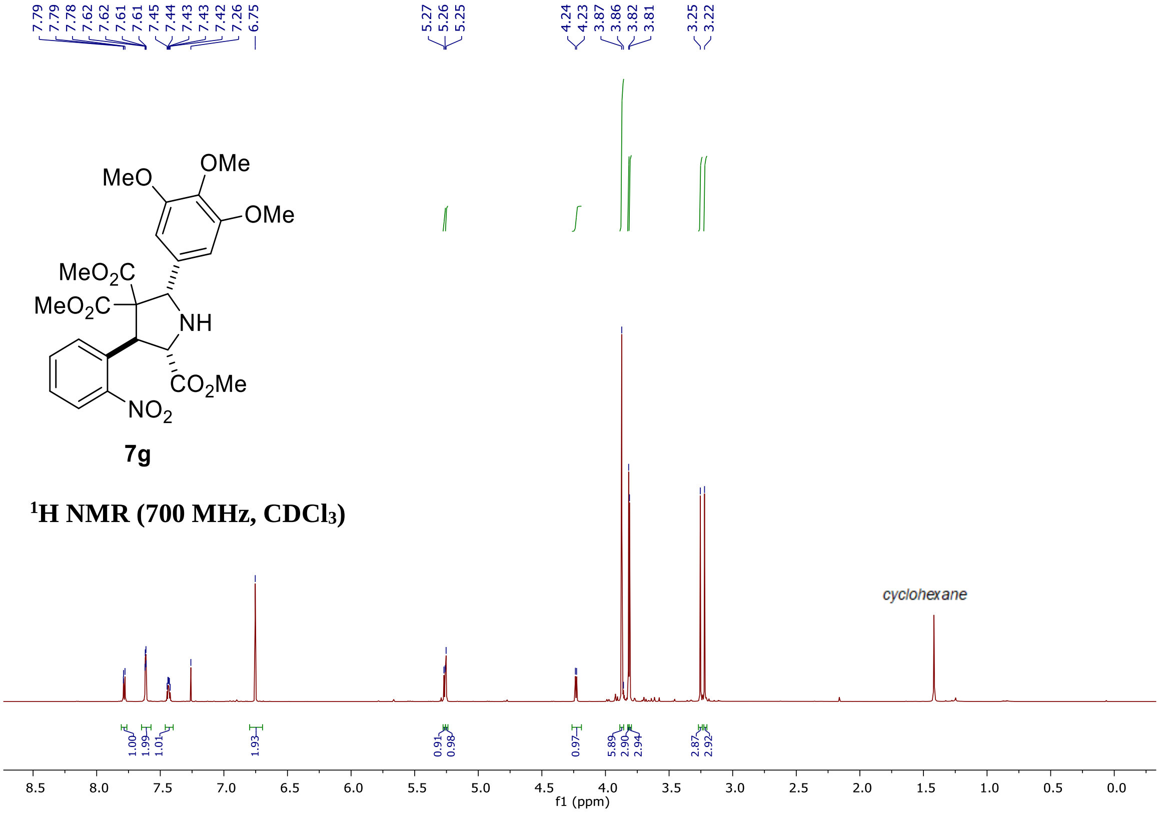
7.79 7.79 7.78 7.62 7.62 7.61 7.61 7.45 7.44 7.43 7.43 7.42 7.26 6.75
5.27 5.26 5.25
4.24 4.23 3.87 3.86 3.82 3.81
3.25 3.22
MeO
OMe
OMe
MeO2C
MeO2C
NH
CO2Me
NO2
7g
1H NMR (700 MHz, CDCl3)
cyclohexane
1.00 1.99 1.01 1.93 0.91 0.98 0.97 5.89 2.90 2.94 2.87 2.92
8.5 8.0 7.5 7.0 6.5 6.0 5.5 5.0 4.5 4.0 3.5 3.0 2.5 2.0 1.5 1.0 0.5 0.0
f1 (ppm)

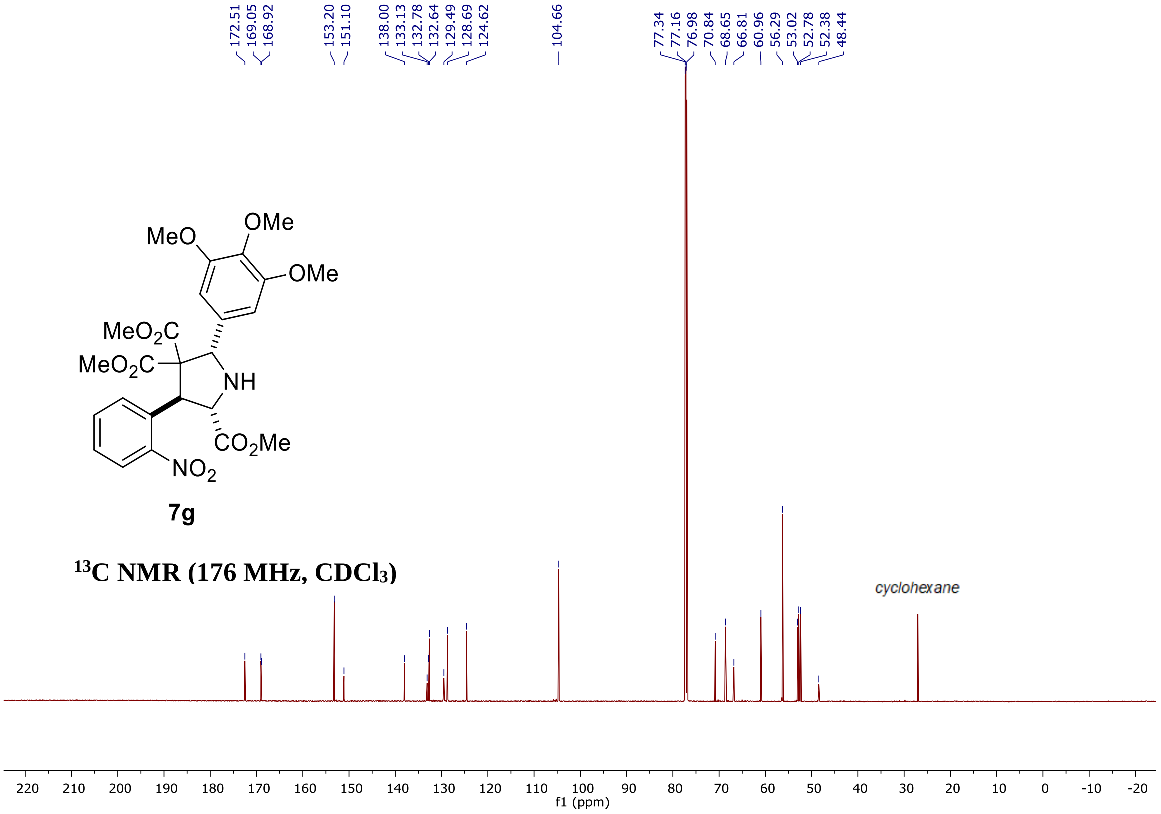
172.51 169.05 168.92
153.20 151.10
138.00 133.13 132.78 132.64 129.49 128.69 124.62
104.66
77.34 77.16 76.98 70.84 68.65 66.81 60.96 56.29 53.02 52.78 52.38 48.44
MeO
OMe
OMe
MeO2C
MeO2C
NH
CO2Me
NO2
7g
13C NMR (176 MHz, CDCl3)
cyclohexane
220 210 200 190 180 170 160 150 140 130 120 110 100 90 80 70 60 50 40 30 20 10 0 -10 -20
f1 (ppm)

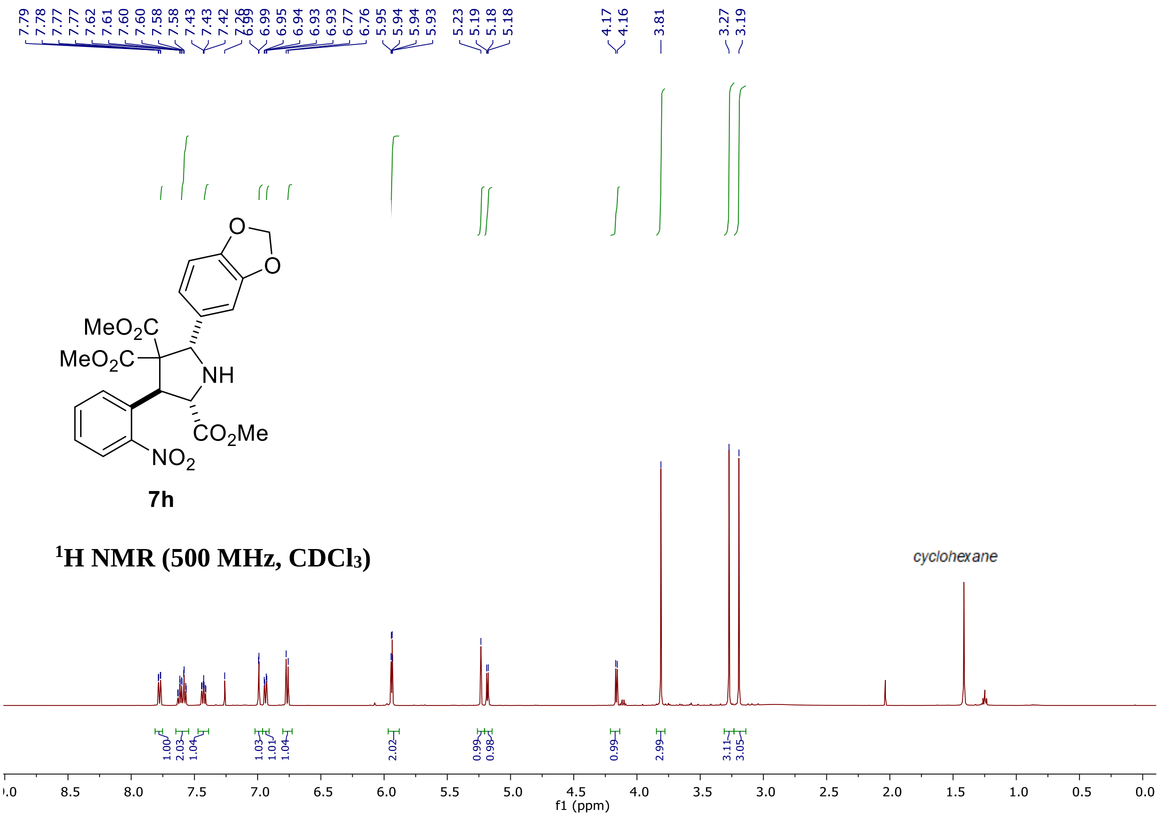
7.79
7.78
7.77
7.77
7.62
7.61
7.60
7.60
7.58
7.58
7.43
7.43
7.42
6.99
6.95
6.94
6.93
6.93
6.77
6.76
5.95
5.94
5.94
5.93
5.23
5.19
5.18
5.18
4.17
4.16
3.81
3.27
3.19
MeO2C
MeO2C
NH
CO2Me
NO2
O
O
7h
1H NMR (500 MHz, CDCl3)
cyclohexane
1.00
2.03
1.04
1.03
1.01
1.04
2.02
0.99
0.98
0.99
2.99
3.11
3.05
f1 (ppm)

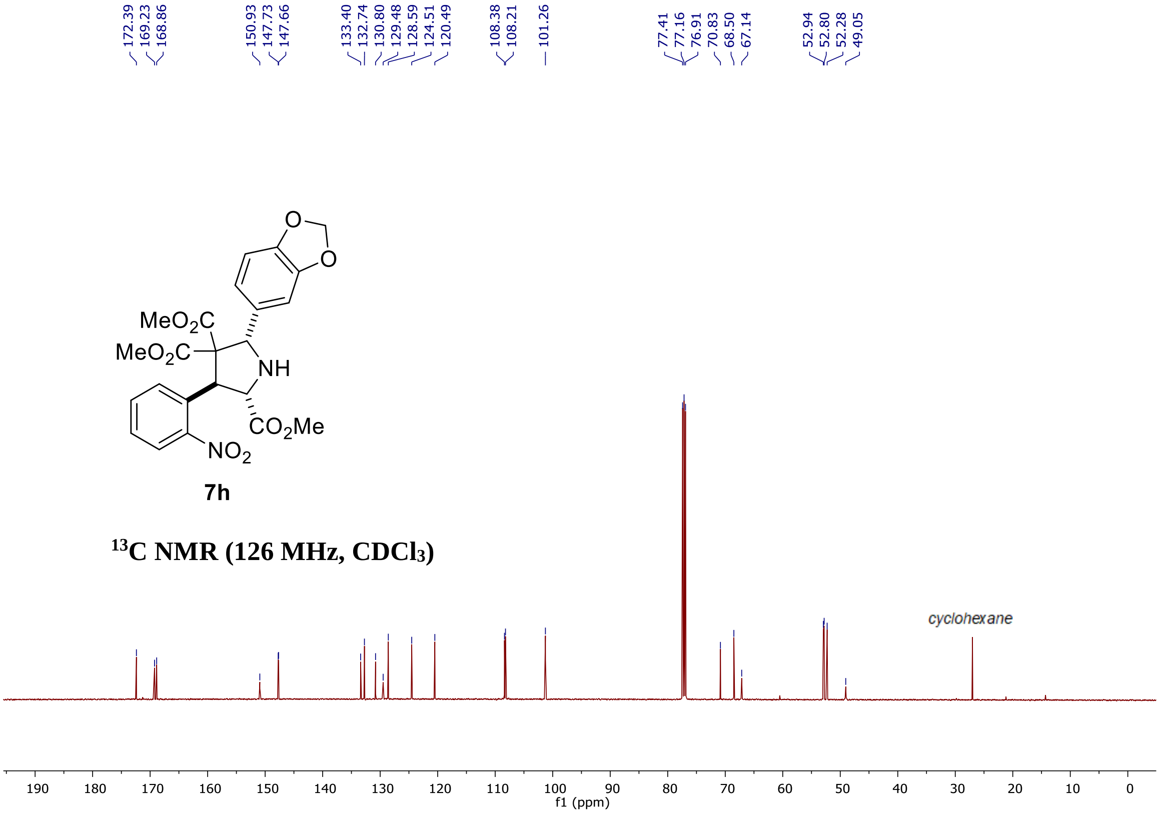
172.39
169.23
168.86
150.93
147.73
147.66
133.40
132.74
130.80
129.48
128.59
124.51
120.49
108.38
108.21
101.26
77.41
77.16
76.91
70.83
68.50
67.14
52.94
52.80
52.28
49.05
MeO2C
MeO2C
NH
CO2Me
NO2
O
O
7h
13C NMR (126 MHz, CDCl3)
cyclohexane
f1 (ppm)

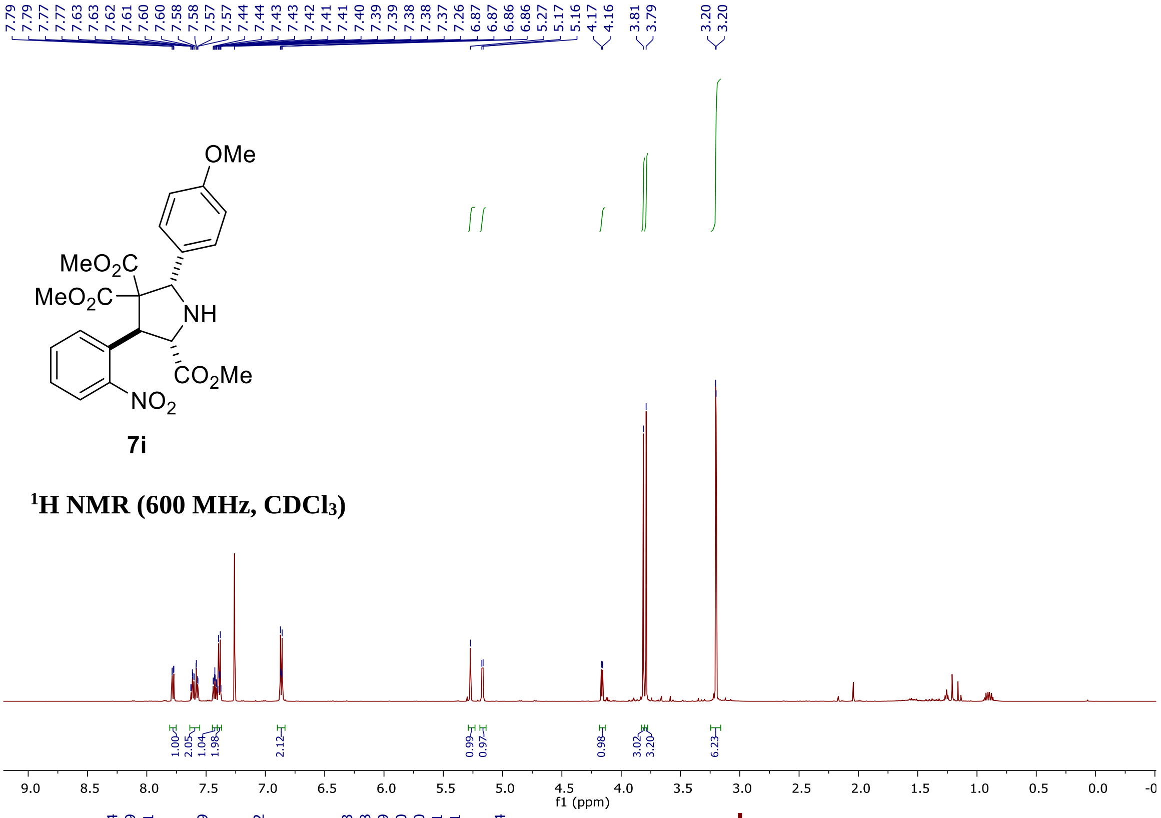
7.79 7.79 7.77 7.77 7.63 7.63 7.62 7.61 7.60 7.60 7.58 7.58 7.57 7.57 7.44 7.44 7.43 7.43 7.42 7.41 7.41 7.40 7.39 7.39 7.38 7.38 7.37 7.26 6.87 6.87 6.86 6.86 5.27 5.17 5.16 4.17 4.16 3.81 3.79 3.20 3.20
OMe
MeO_2C
MeO_2C
NH
CO_2Me
NO_2
7i
^1H NMR (600 MHz, $CDCl_3$)
1.00 2.05 1.04 1.98 2.12 0.99 0.97 0.98 3.02 3.20 6.23
9.0 8.5 8.0 7.5 7.0 6.5 6.0 5.5 5.0 4.5 4.0 3.5 3.0 2.5 2.0 1.5 1.0 0.5 0.0
f1 (ppm)

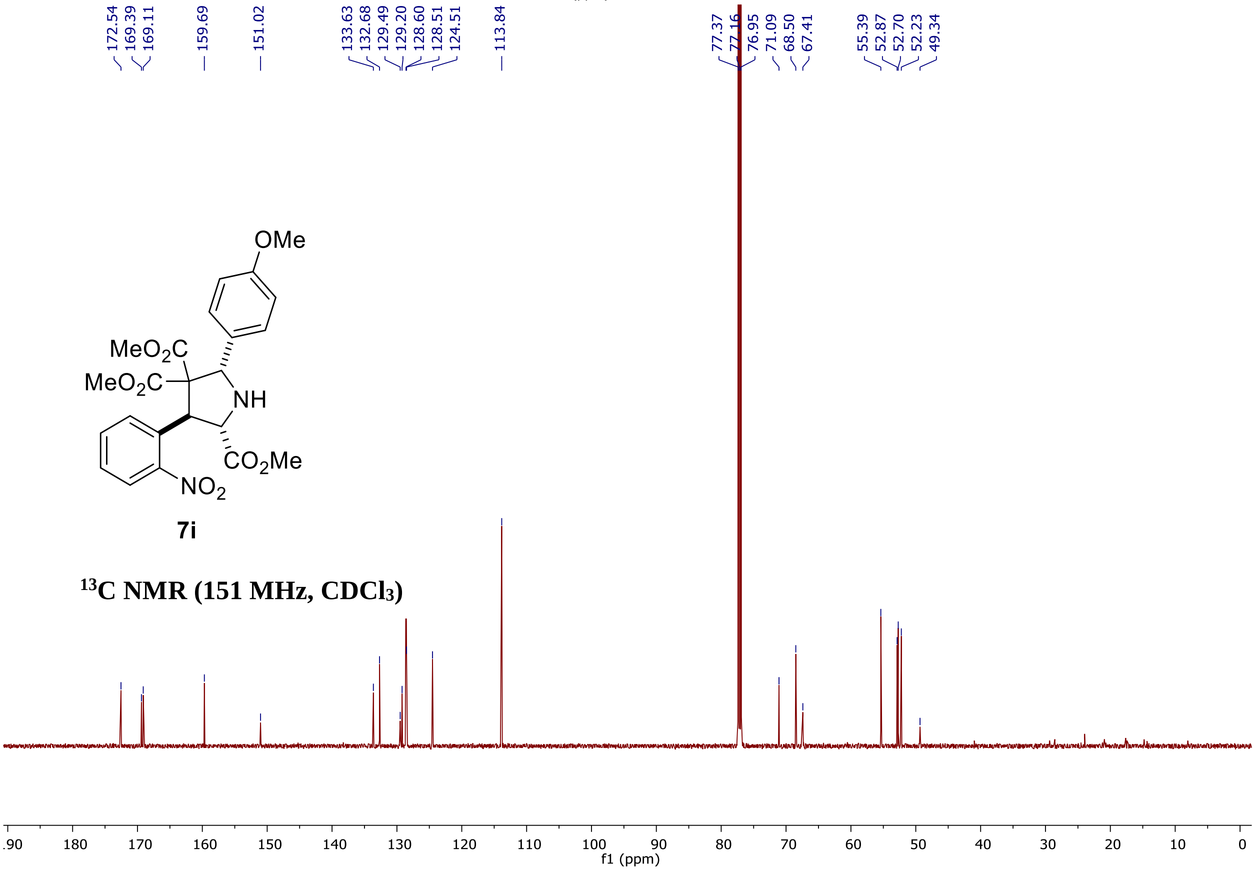
172.54 169.39 169.11 159.69 151.02 133.63 132.68 129.49 129.20 128.60 128.51 124.51 113.84 77.37 77.16 76.95 71.09 68.50 67.41 55.39 52.87 52.70 52.23 49.34
OMe
MeO_2C
MeO_2C
NH
CO_2Me
NO_2
7i
^{13}C NMR (151 MHz, $CDCl_3$)
180 170 160 150 140 130 120 110 100 90 80 70 60 50 40 30 20 10 0
f1 (ppm)

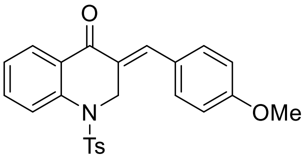

9a

^{1}H NMR (700 MHz, CDCl$_3$)

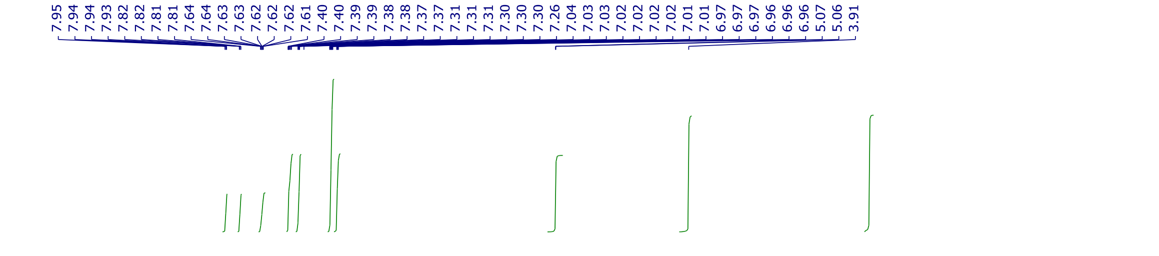
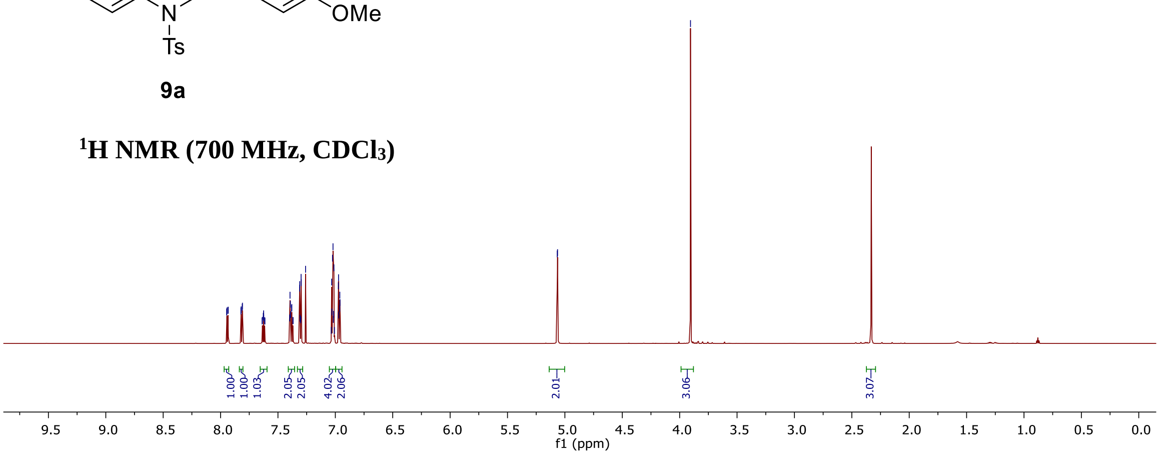

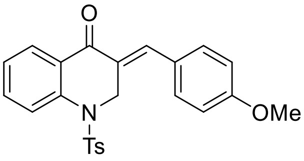

9a

^{13}C NMR (176 MHz, CDCl$_3$)

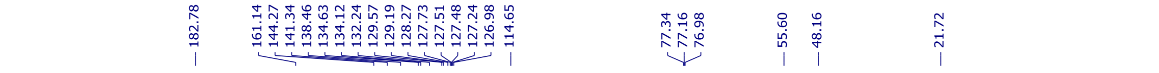
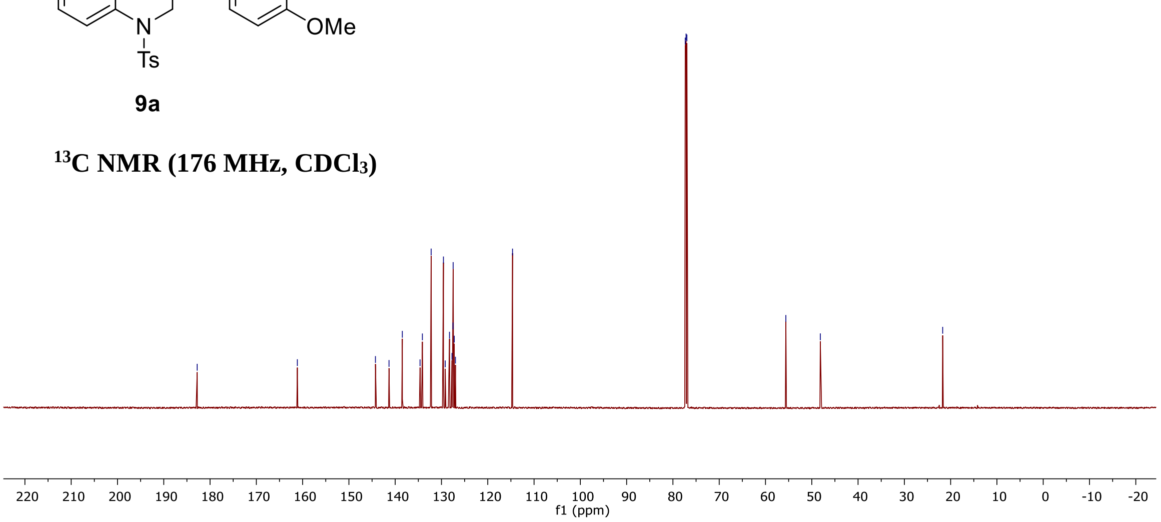

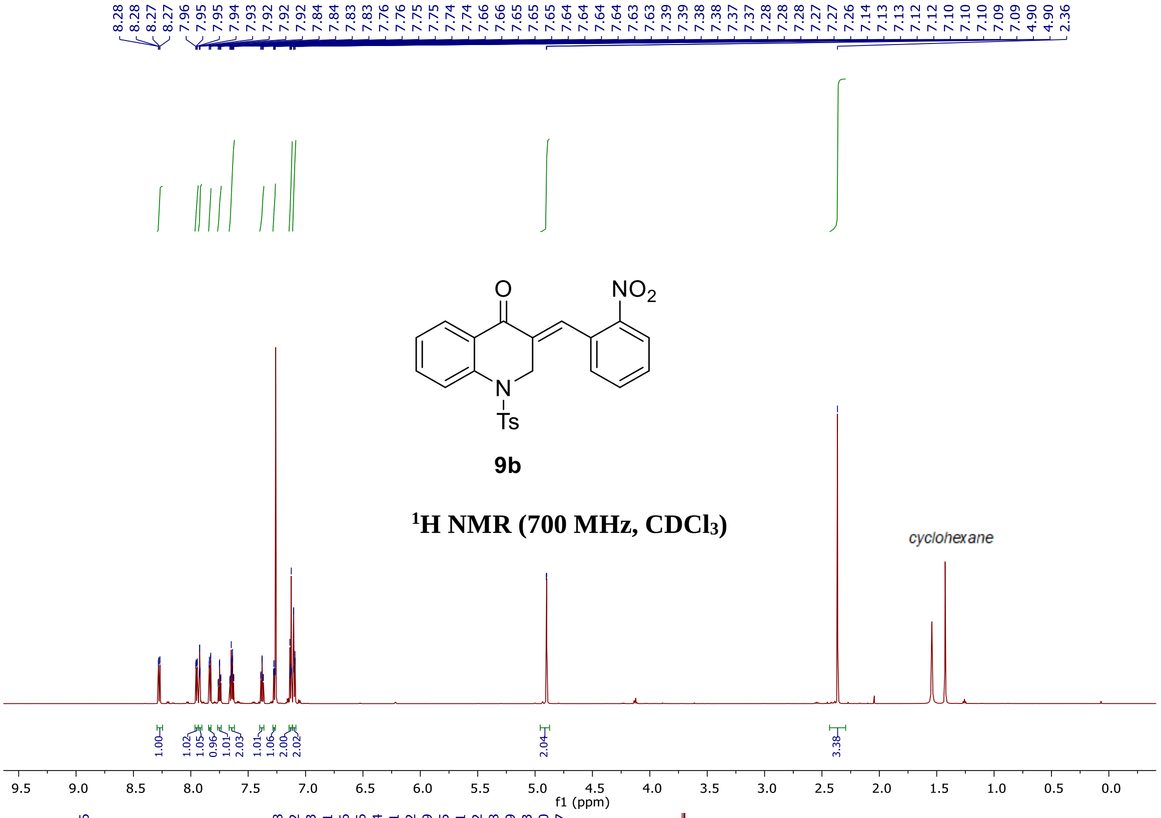
8.28 8.28 8.27 8.27 7.96 7.95 7.95 7.94 7.93 7.92 7.92 7.92 7.84 7.84 7.83 7.83 7.76 7.76 7.75 7.75 7.74 7.74 7.66 7.66 7.65 7.65 7.65 7.64 7.64 7.64 7.64 7.63 7.63 7.39 7.39 7.38 7.38 7.37 7.37 7.28 7.28 7.28 7.27 7.27 7.26 7.14 7.13 7.13 7.12 7.12 7.10 7.10 7.10 7.09 7.09 4.90 4.90 2.36
O
NO2
N
Ts
9b
1H NMR (700 MHz, CDCl3)
cyclohexane
1.00 1.02 1.05 0.96 1.01 2.03 1.01 1.06 2.00 2.02 2.04 3.38
9.5 9.0 8.5 8.0 7.5 7.0 6.5 6.0 5.5 5.0 4.5 4.0 3.5 3.0 2.5 2.0 1.5 1.0 0.5 0.0
f1 (ppm)

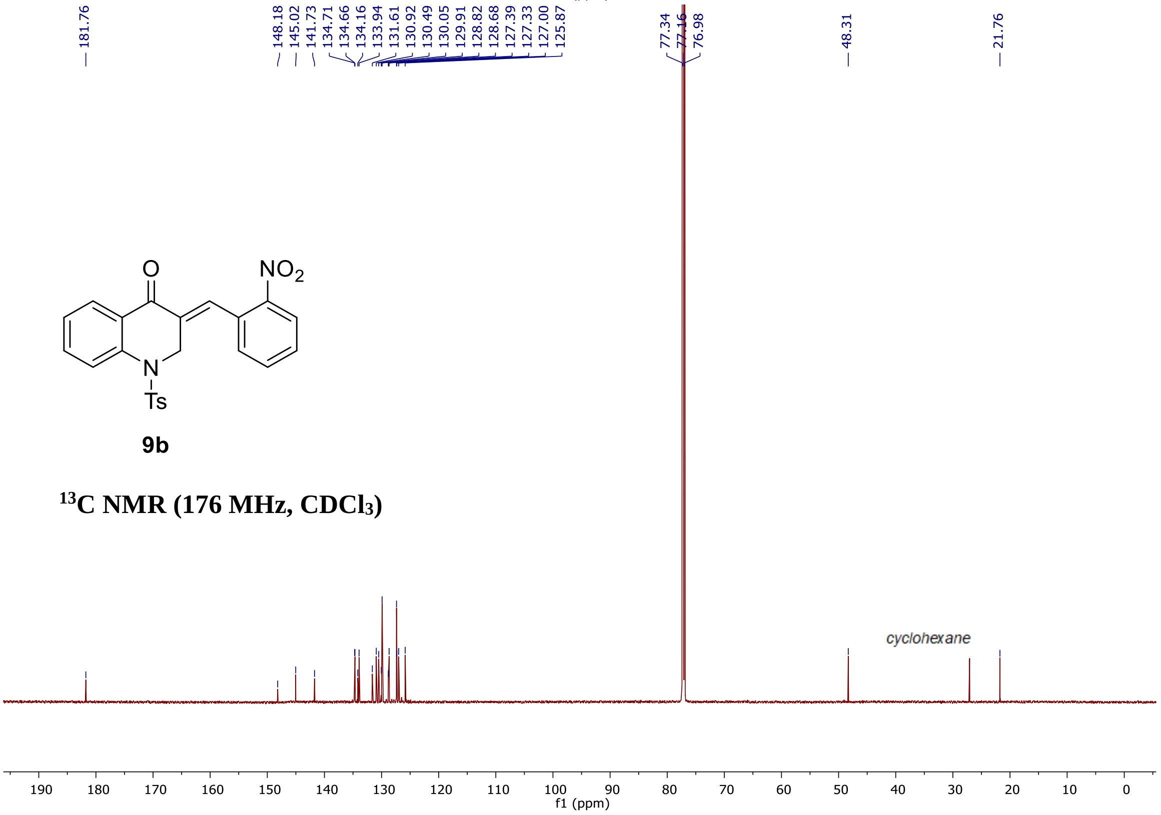
181.76 148.18 145.02 141.73 134.71 134.66 134.16 133.94 131.61 130.92 130.49 130.05 129.91 128.82 128.68 127.39 127.33 127.00 125.87 77.34 77.16 76.98 48.31 21.76
O
NO2
N
Ts
9b
13C NMR (176 MHz, CDCl3)
cyclohexane
190 180 170 160 150 140 130 120 110 100 90 80 70 60 50 40 30 20 10 0
f1 (ppm)

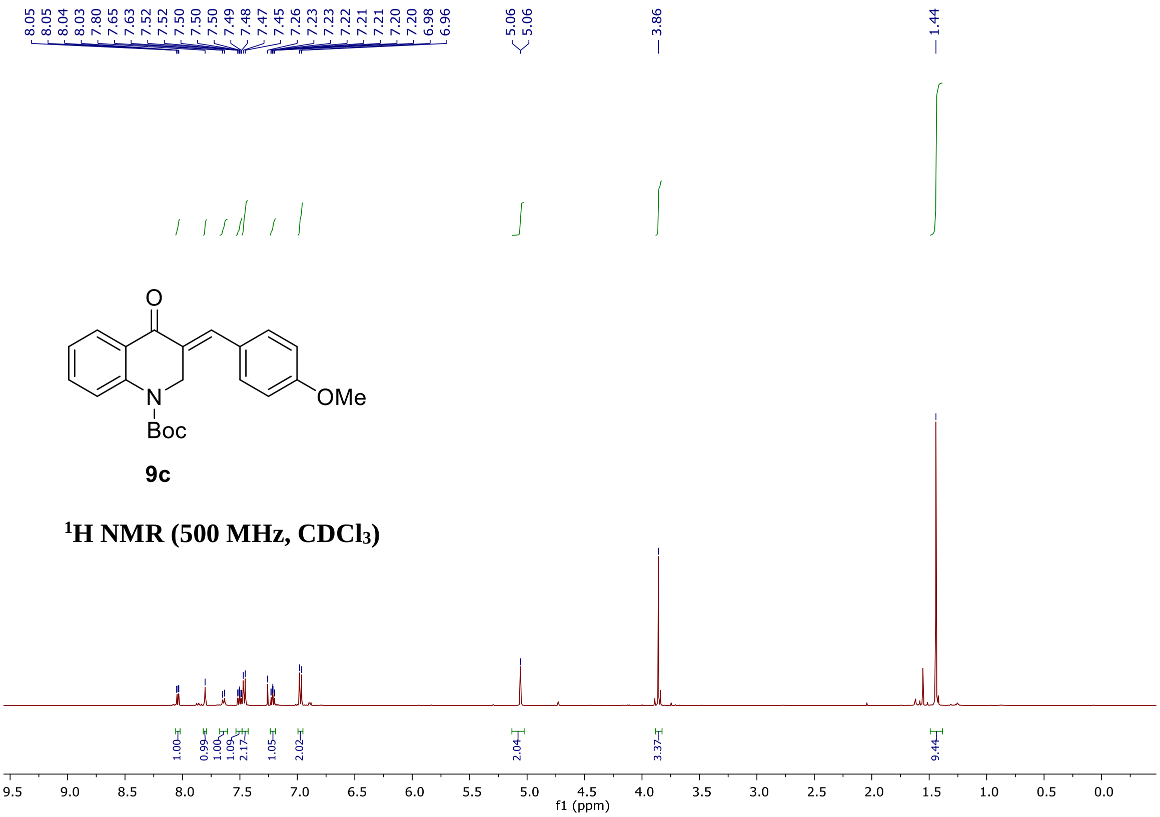

8.05
8.05
8.04
8.03
7.80
7.65
7.63
7.52
7.52
7.50
7.50
7.50
7.49
7.48
7.47
7.45
7.26
7.23
7.23
7.22
7.21
7.21
7.20
7.20
6.98
6.96
5.06
5.06
3.86
1.44
O
N
Boc
OMe
9c
¹H NMR (500 MHz, CDCl₃)
1.00
0.99
1.00
1.09
2.17
1.05
2.02
2.04
3.37
9.44
f1 (ppm)

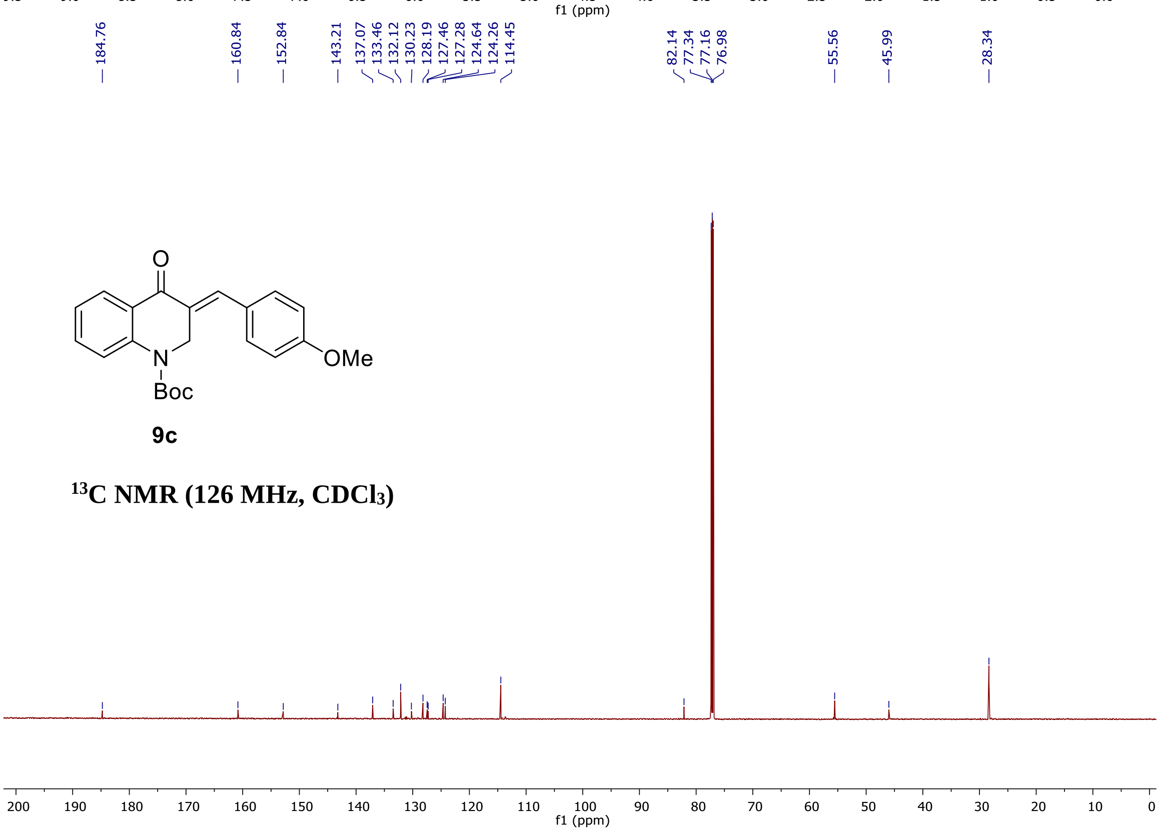

184.76
160.84
152.84
143.21
137.07
133.46
132.12
130.23
128.19
127.46
127.28
124.64
124.26
114.45
82.14
77.34
77.16
76.98
55.56
45.99
28.34
O
N
Boc
OMe
9c
¹³C NMR (126 MHz, CDCl₃)
f1 (ppm)

NMR spectra for pyrroquinolines A-H

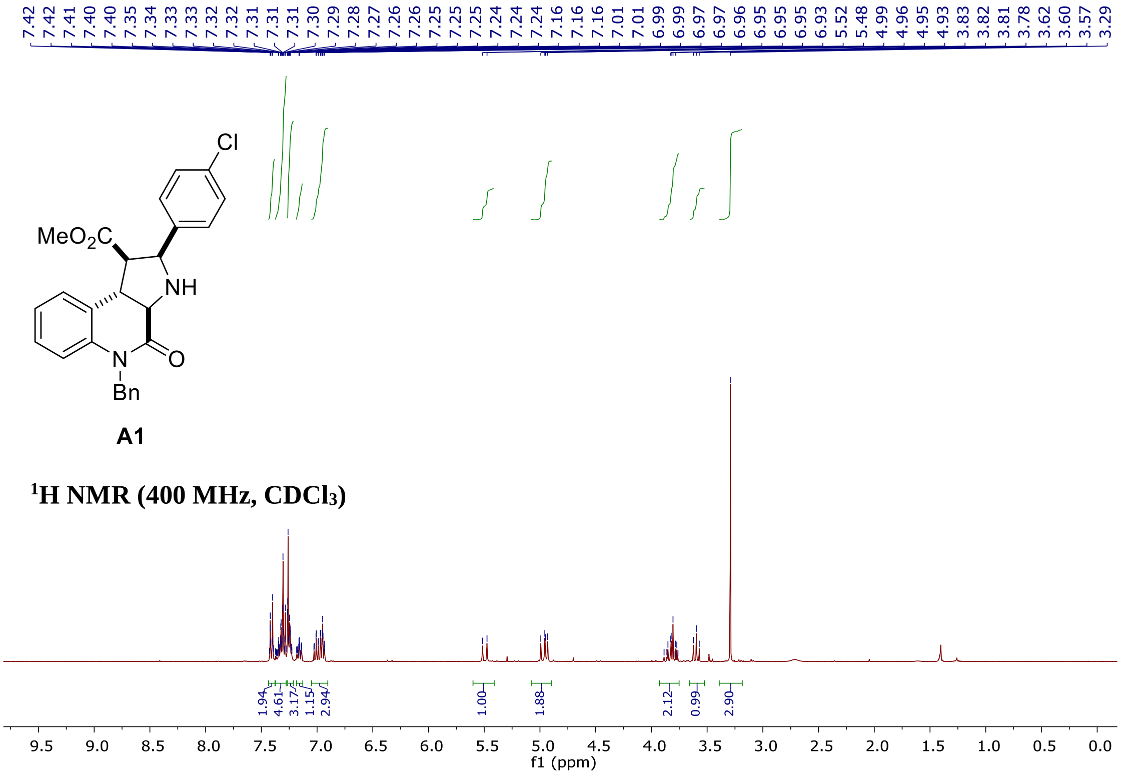

A1

^{1}H NMR (400 MHz, $CDCl_3$)

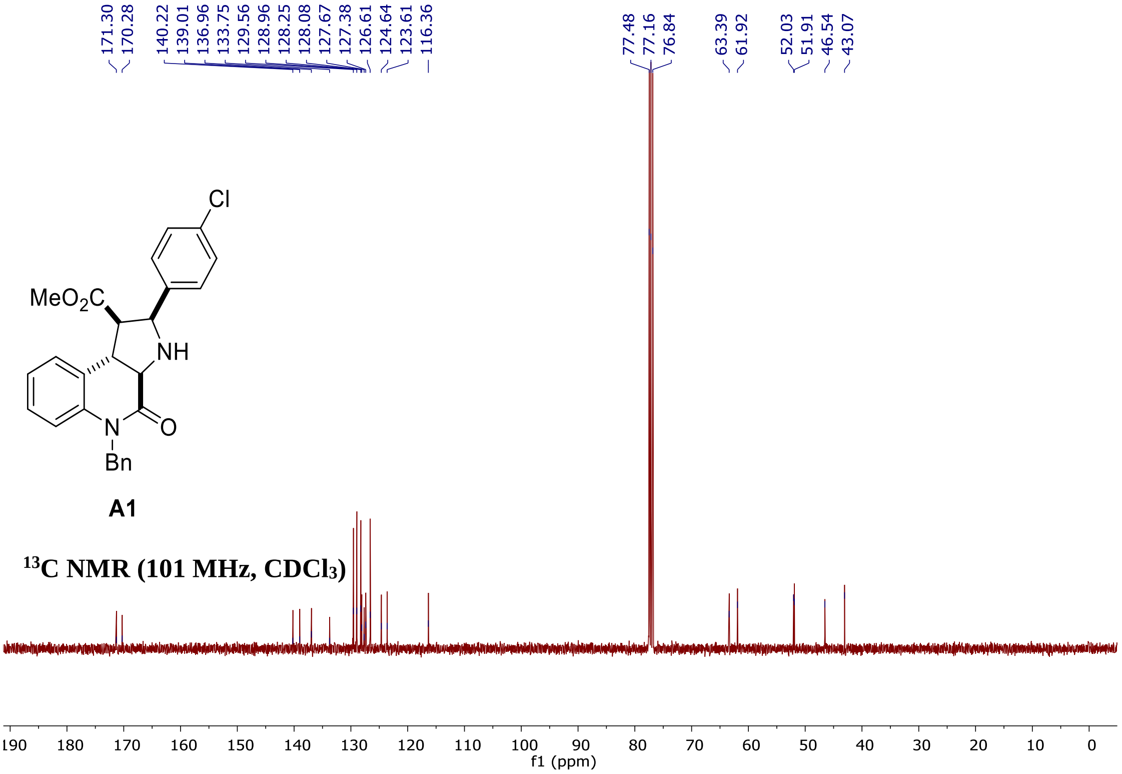

A1

^{13}C NMR (101 MHz, $CDCl_3$)

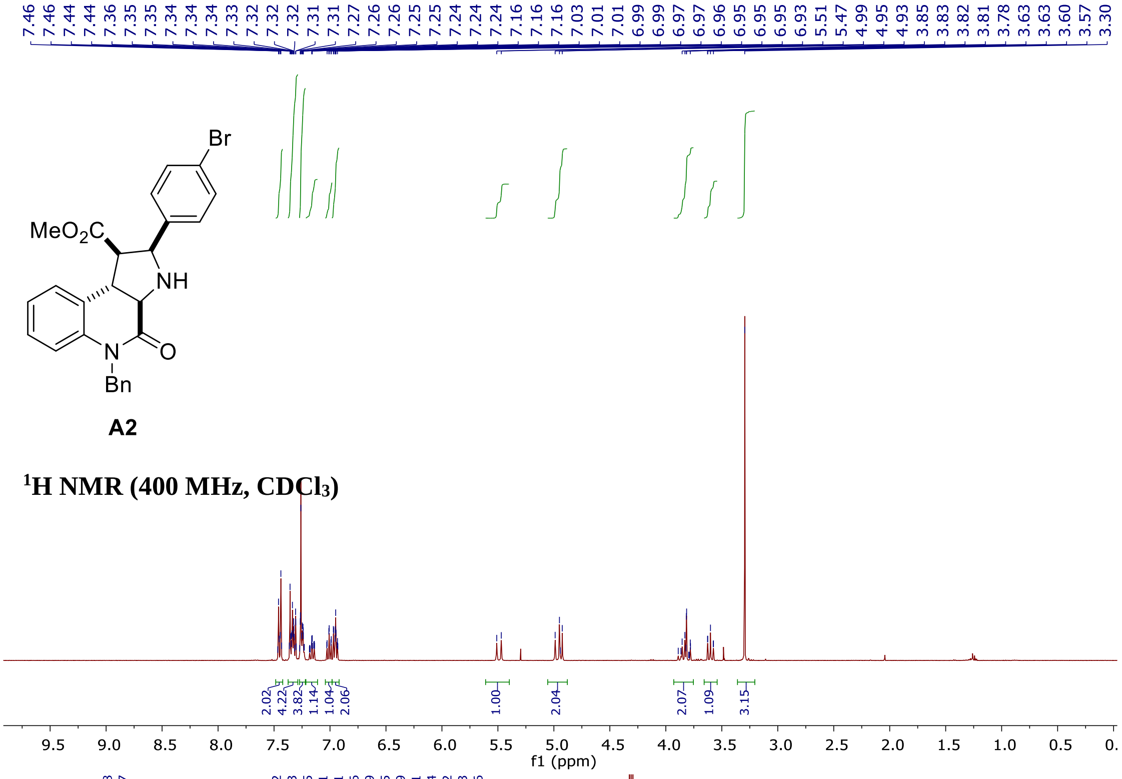
7.46 7.46 7.44 7.44 7.36 7.35 7.35 7.34 7.34 7.34 7.33 7.32 7.32 7.32 7.31 7.31 7.27 7.26 7.26 7.25 7.25 7.24 7.24 7.24 7.24 7.16 7.16 7.16 7.03 7.01 7.01 6.99 6.99 6.97 6.97 6.96 6.95 6.95 6.95 6.93 5.51 5.47 4.99 4.95 4.93 3.85 3.83 3.82 3.81 3.78 3.63 3.63 3.60 3.57 3.30
Br
MeO2C
NH
N
O
Bn
A2
1H NMR (400 MHz, CDCl3)
2.02 4.22 3.82 1.14 1.04 2.06 1.00 2.04 2.07 1.09 3.15
f1 (ppm)

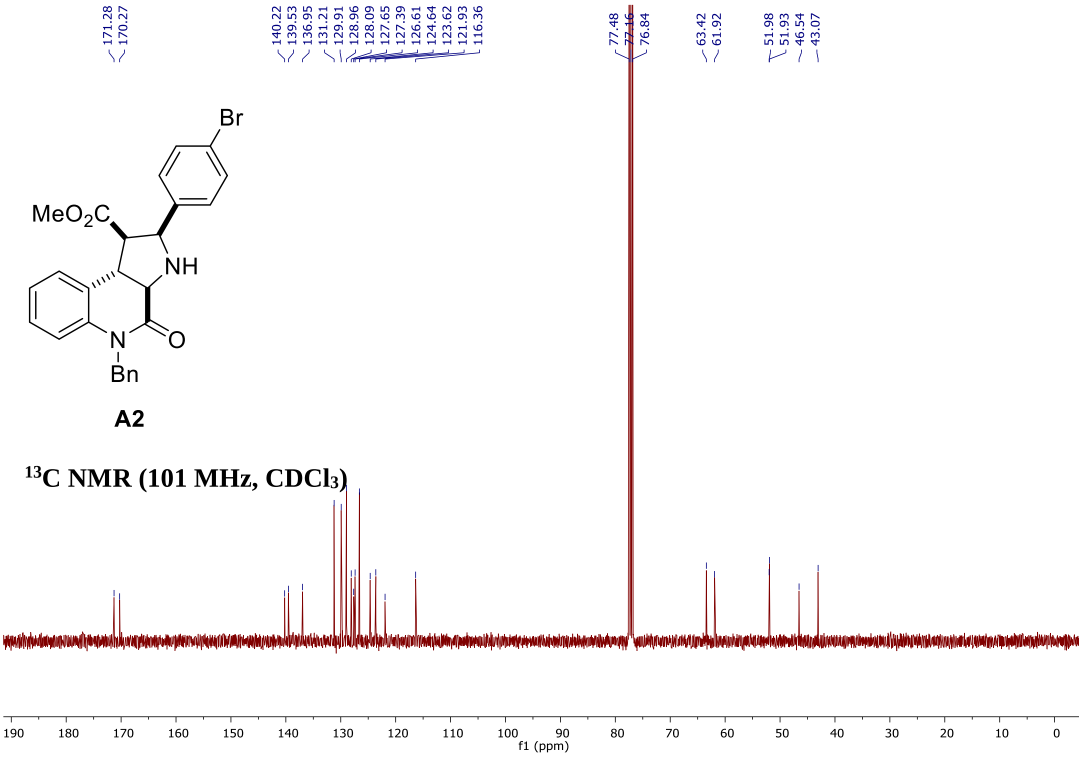
171.28 170.27 140.22 139.53 136.95 131.21 129.91 128.96 128.09 127.65 127.39 126.61 124.64 123.62 121.93 116.36 77.48 77.16 76.84 63.42 61.92 51.98 51.93 46.54 43.07
Br
MeO2C
NH
N
O
Bn
A2
13C NMR (101 MHz, CDCl3)
f1 (ppm)

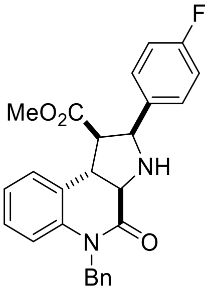

A3

^{1}H NMR (600 MHz, $CDCl_3$)

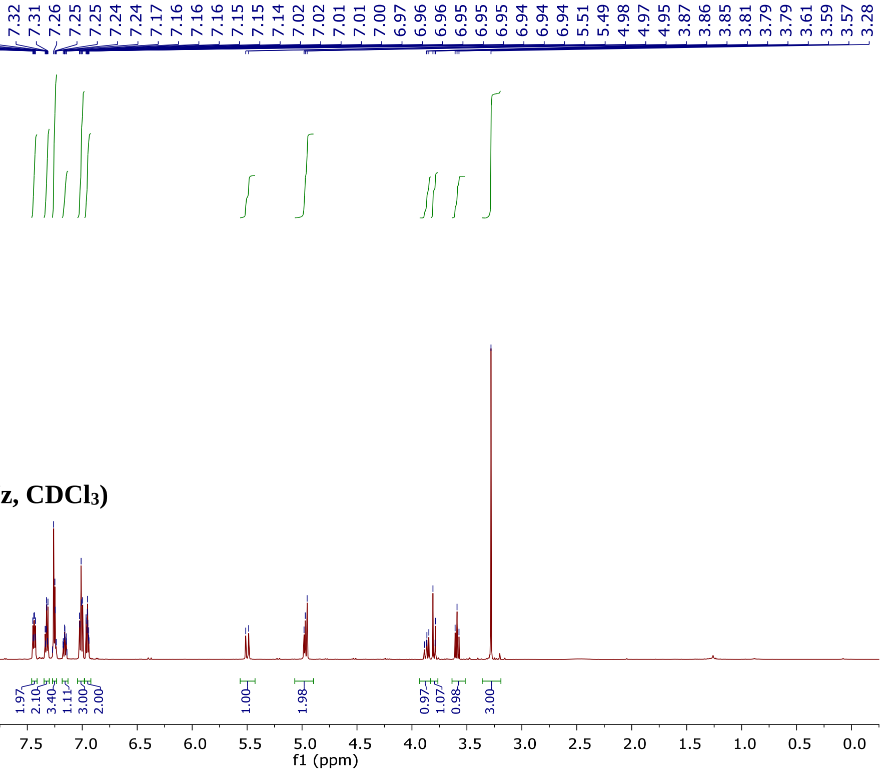

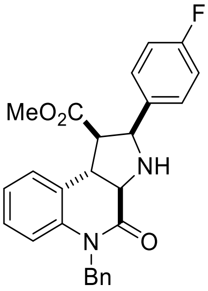

A3

^{13}C NMR (151 MHz, $CDCl_3$)

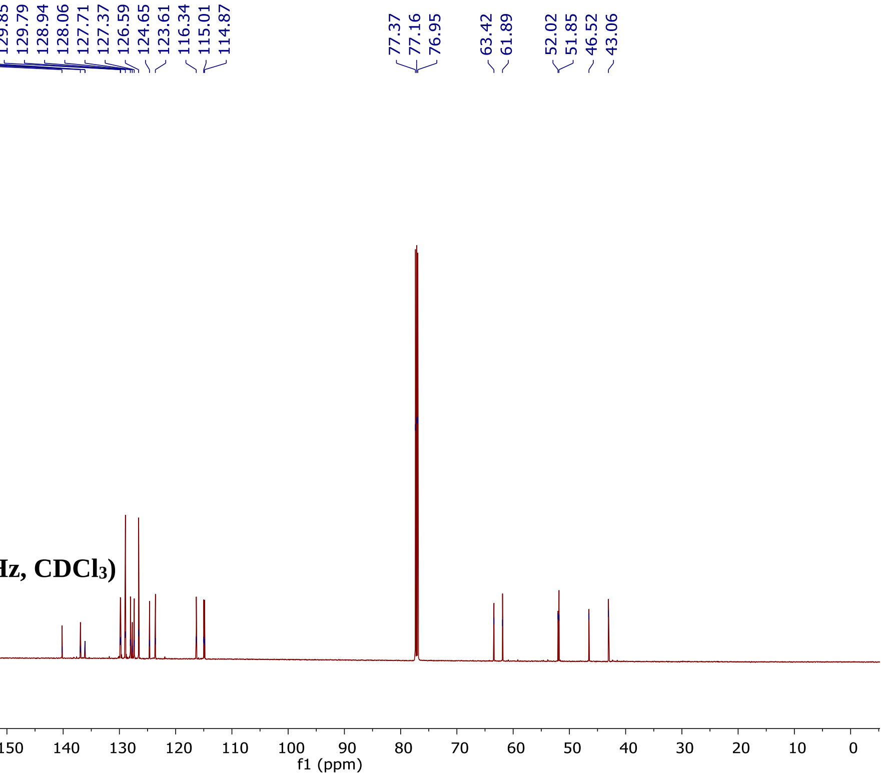

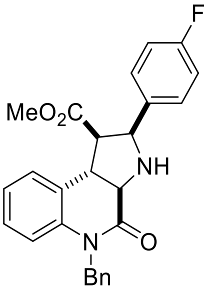

A3

^{19}F NMR (470 MHz, $CDCl_3$)

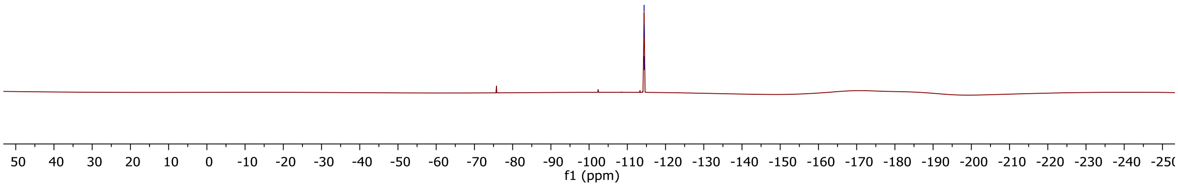

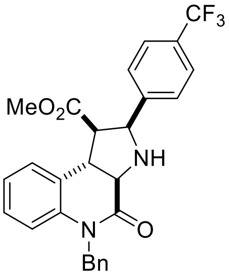

A4

^{19}F NMR (470 MHz, $CDCl_3$)

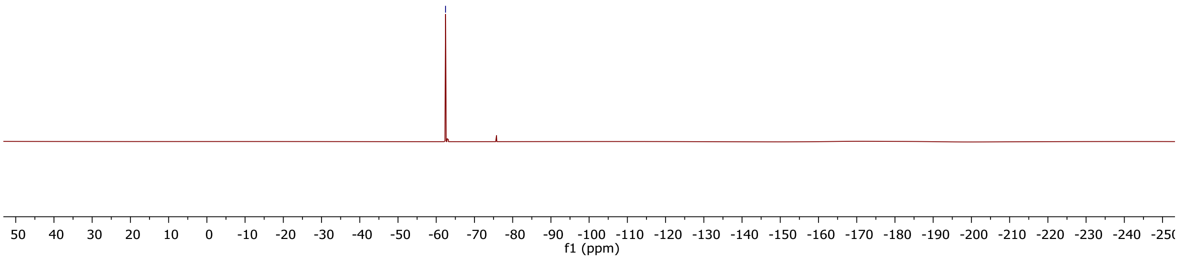

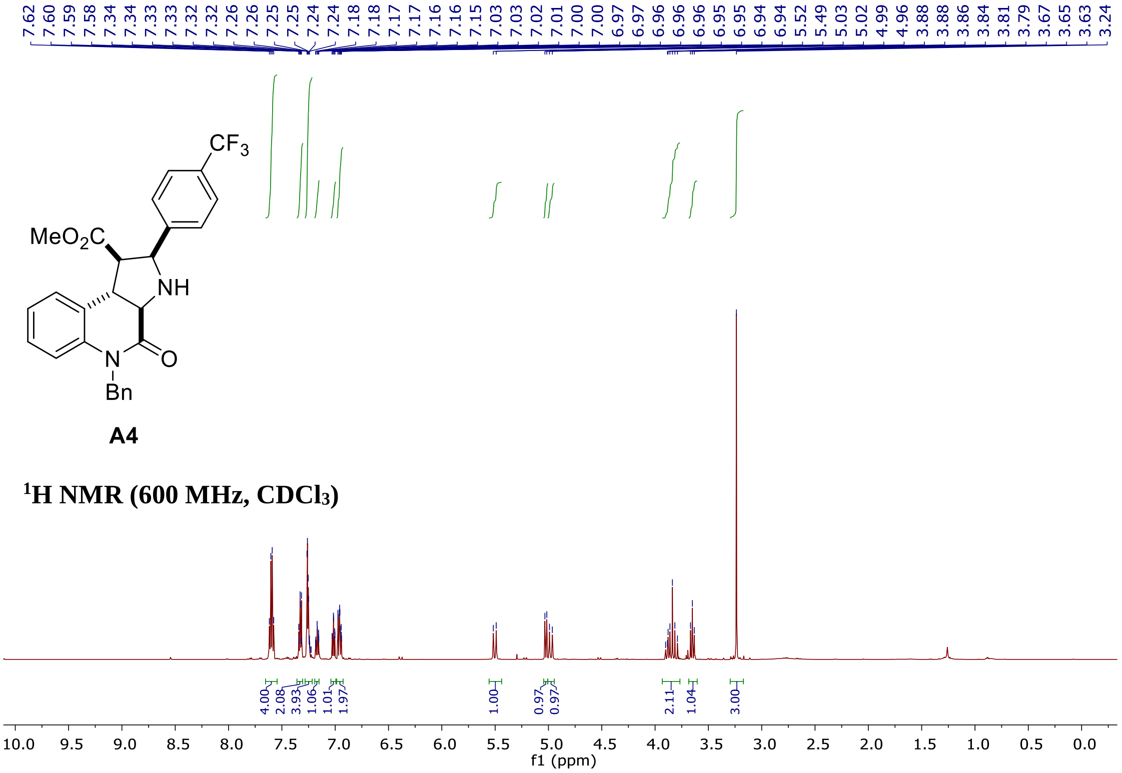

A4

^{1}H NMR (600 MHz, $CDCl_3$)

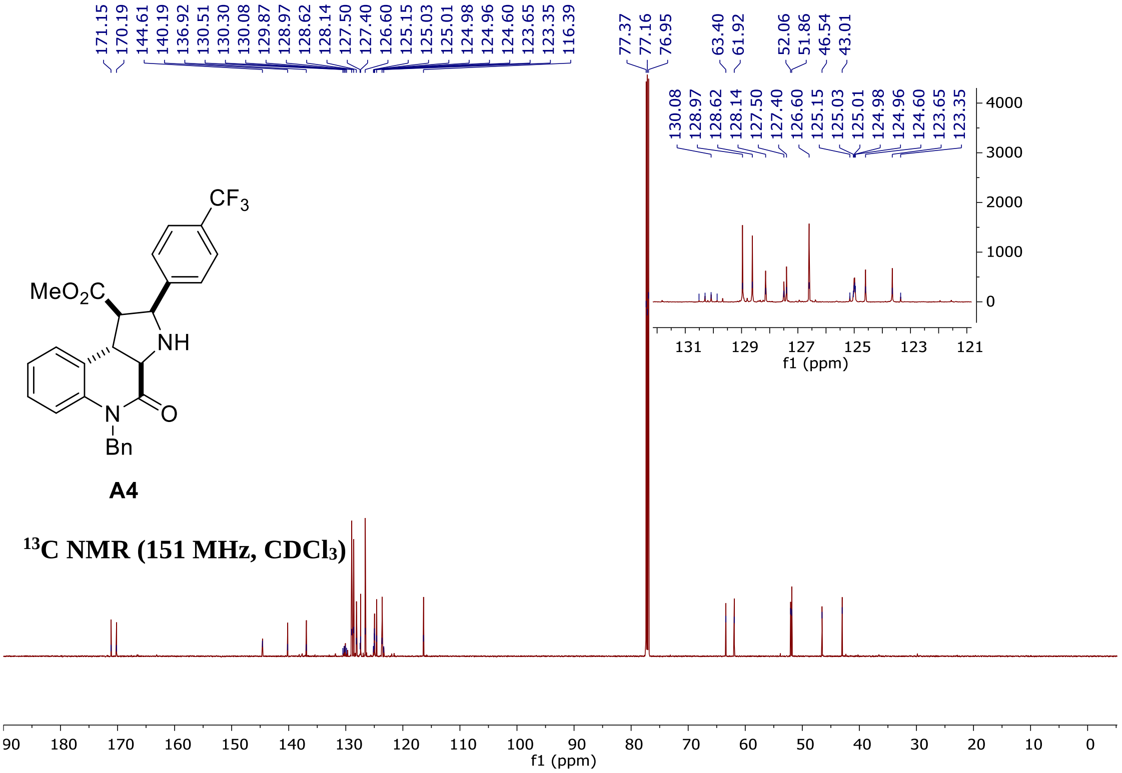

A4

^{13}C NMR (151 MHz, $CDCl_3$)

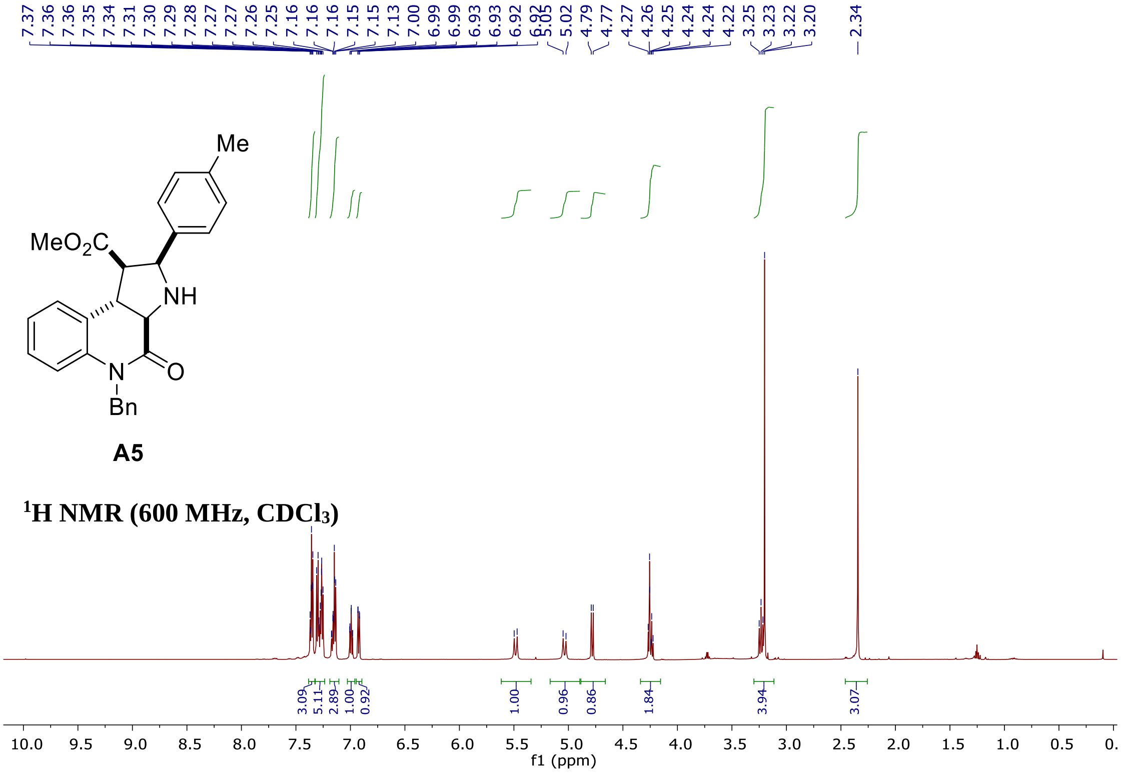

A5

^{1}H NMR (600 MHz, $CDCl_3$)

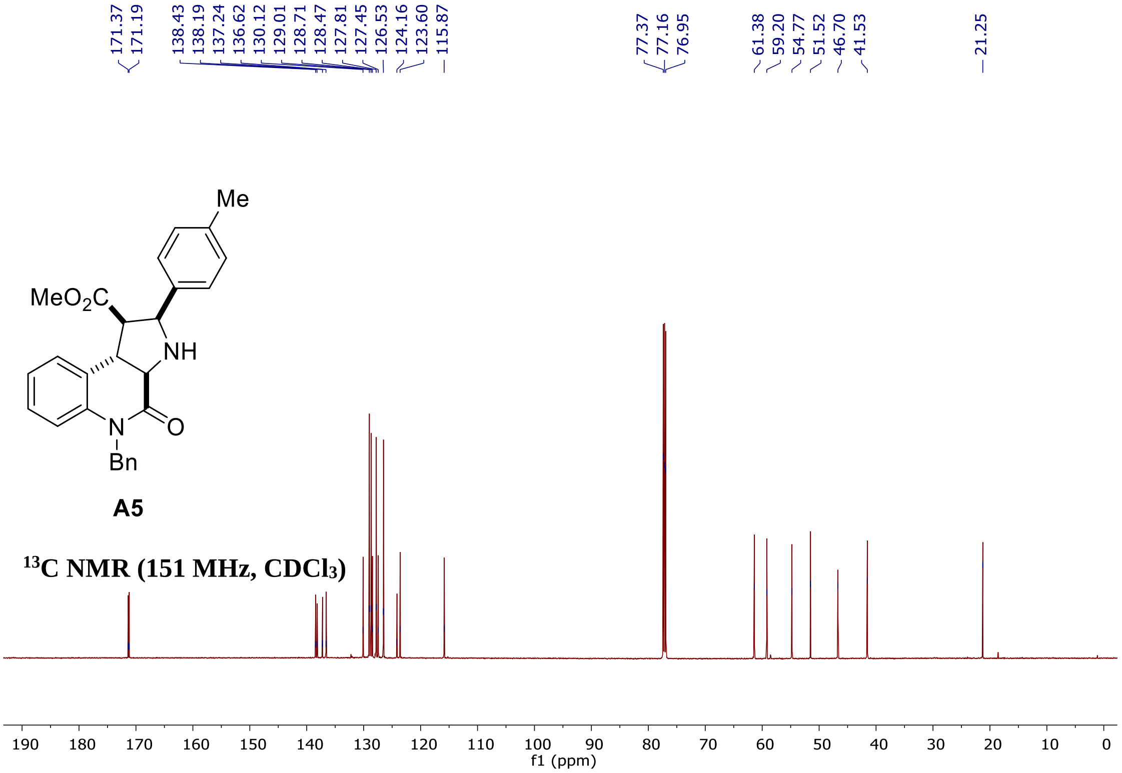

A5

^{13}C NMR (151 MHz, $CDCl_3$)

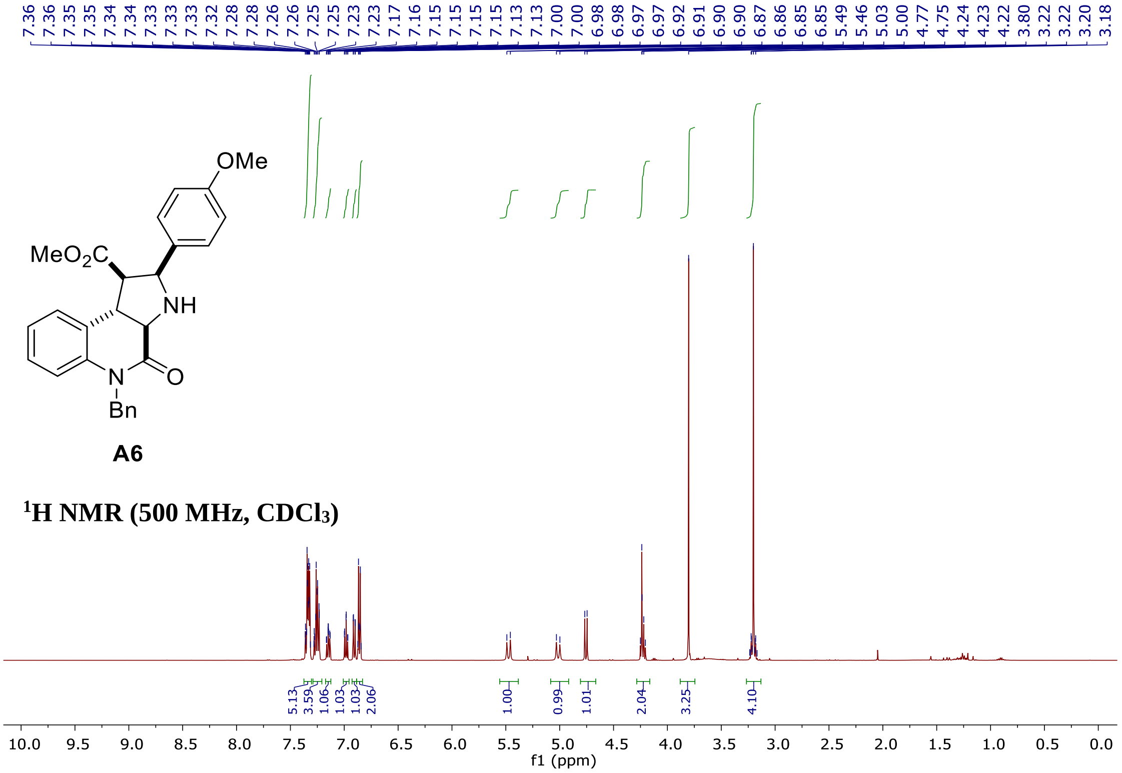

A6

^{1}H NMR (500 MHz, $CDCl_3$)

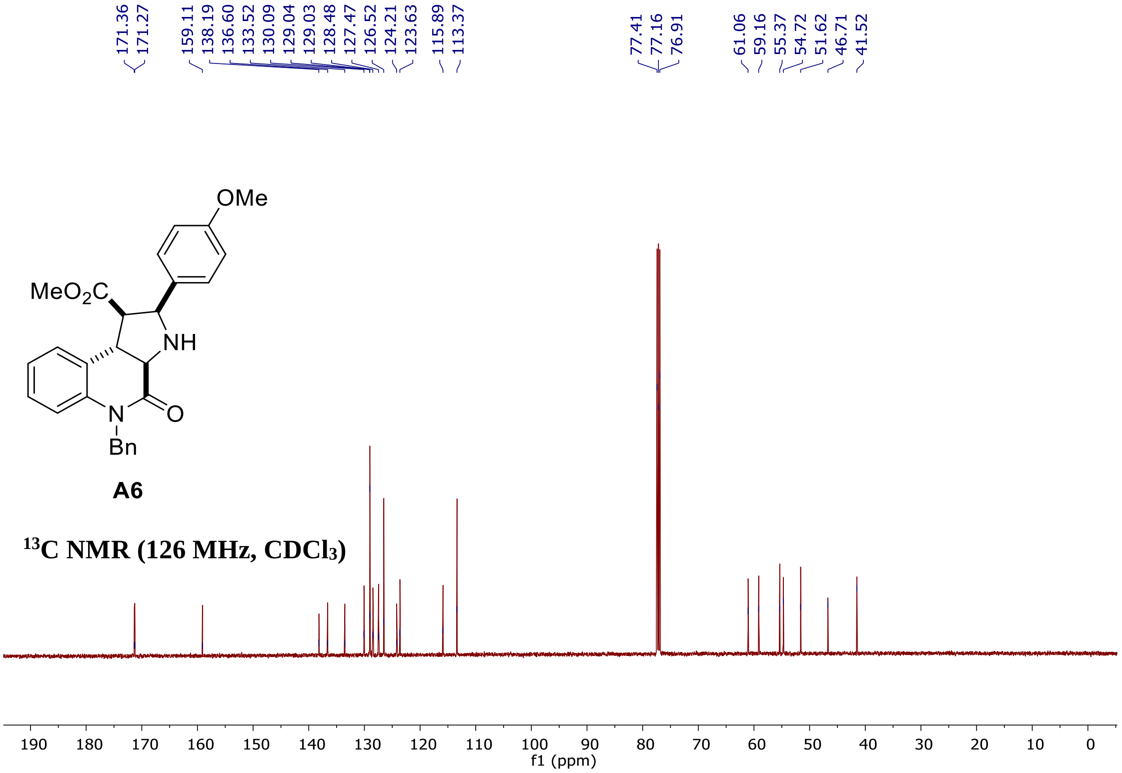

A6

^{13}C NMR (126 MHz, $CDCl_3$)

A7

^{1}H NMR (500 MHz, CDCl₃)

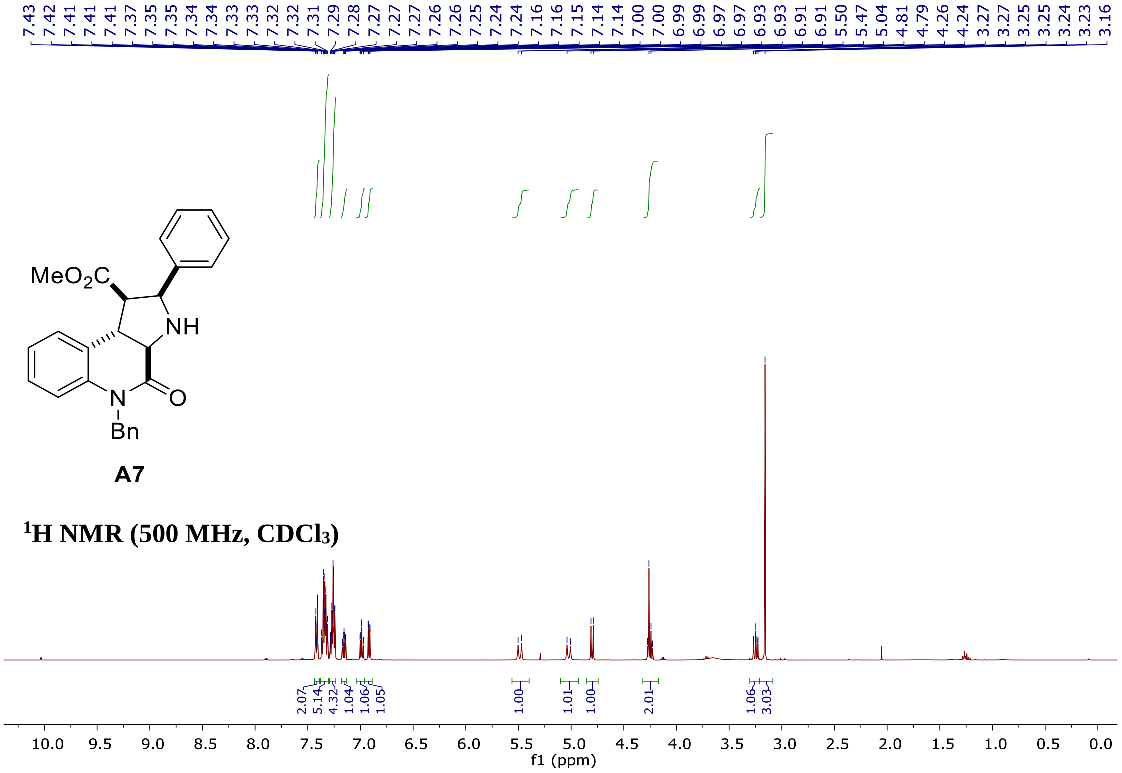

A7

^{13}C NMR (126 MHz, CDCl₃)

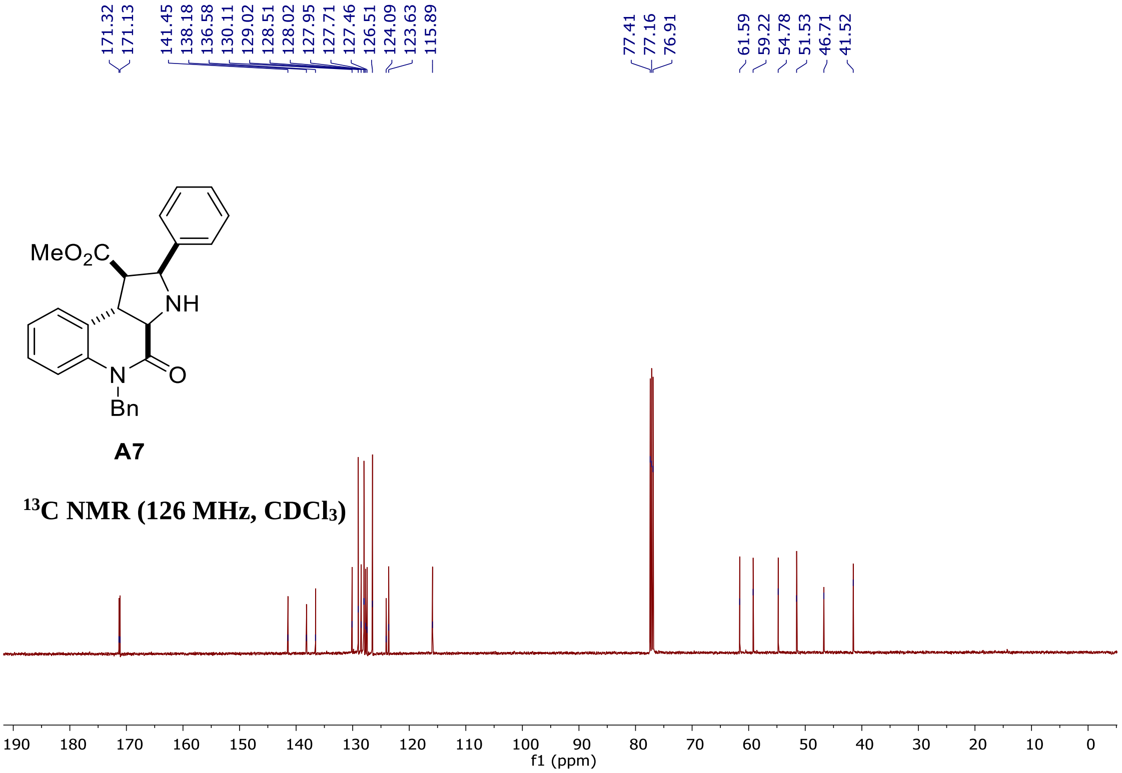

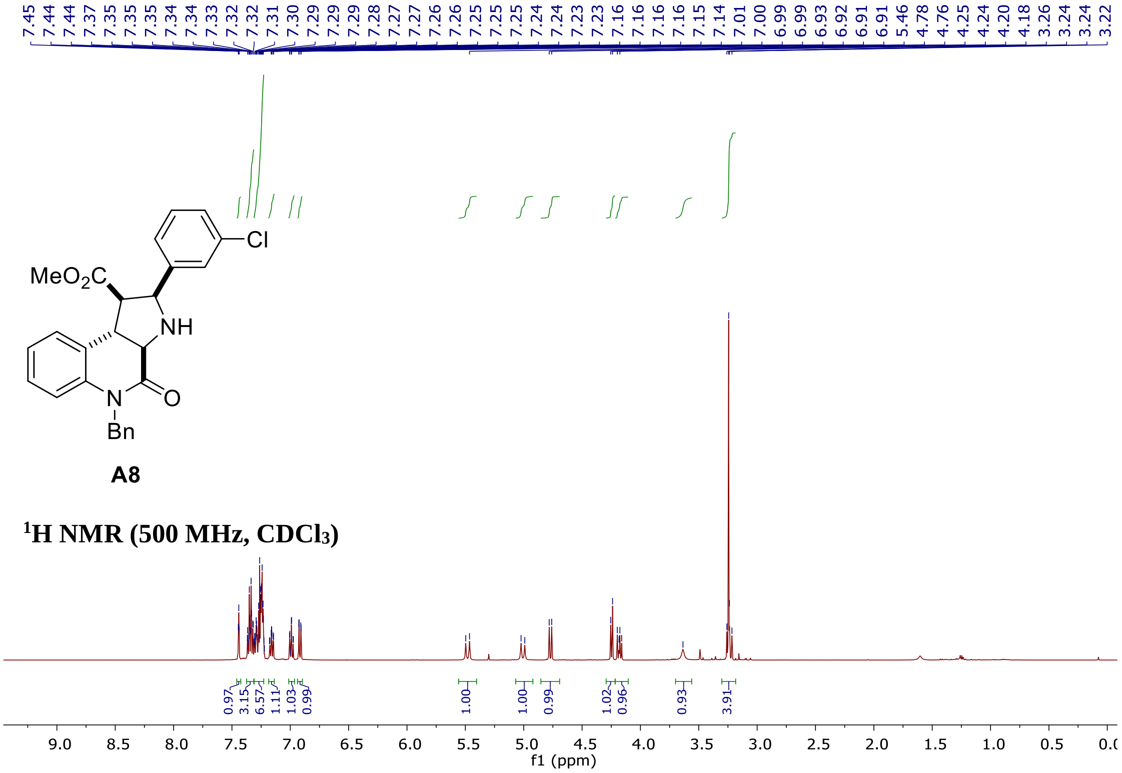

^{1}H NMR (500 MHz, CDCl$_3$)

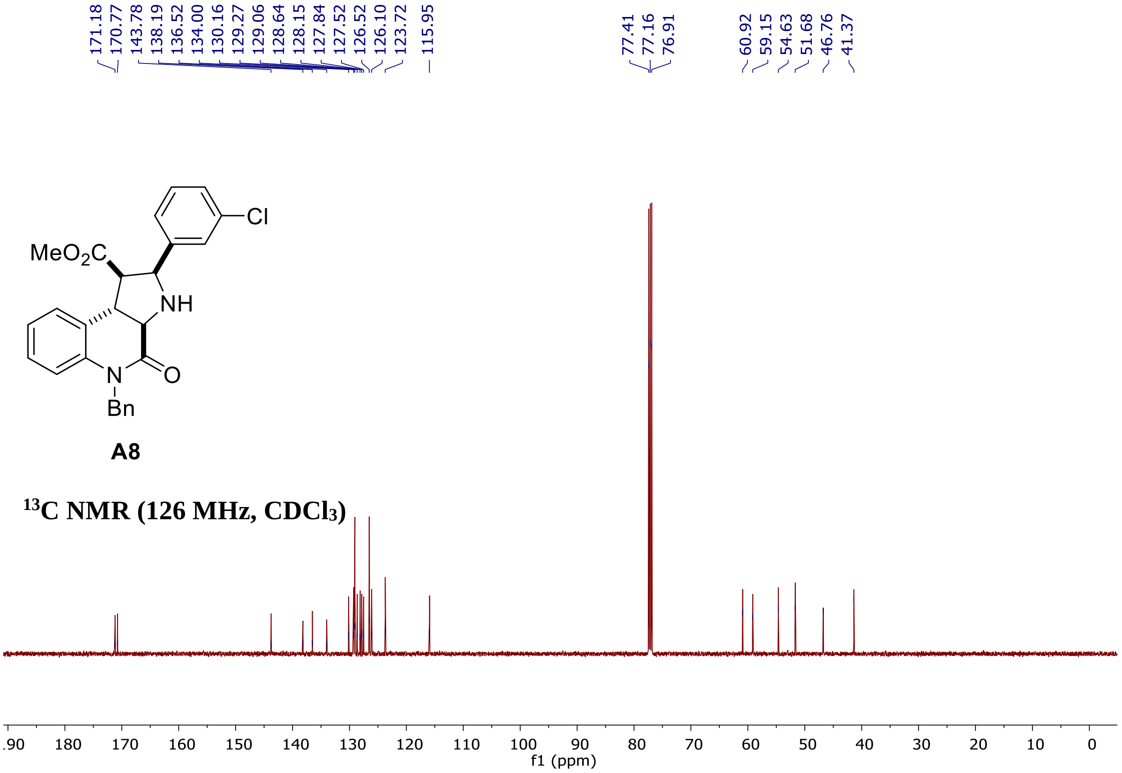

^{13}C NMR (126 MHz, CDCl$_3$)

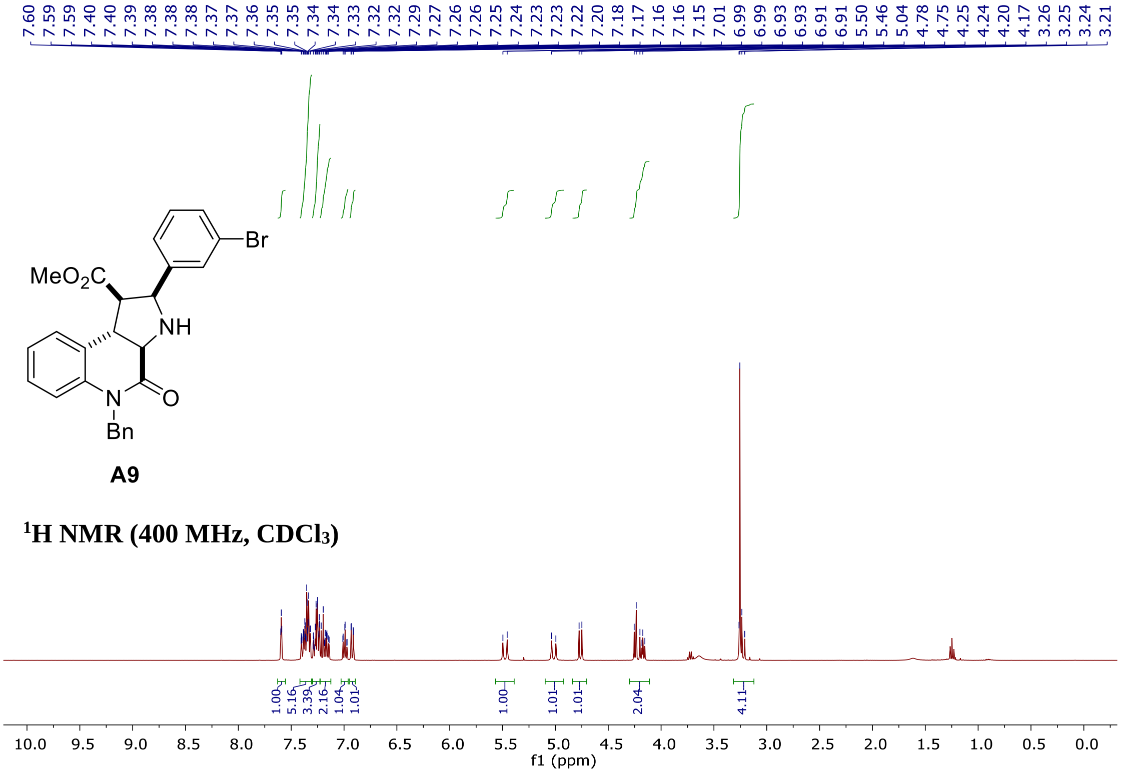

A9

^{1}H NMR (400 MHz, CDCl3)

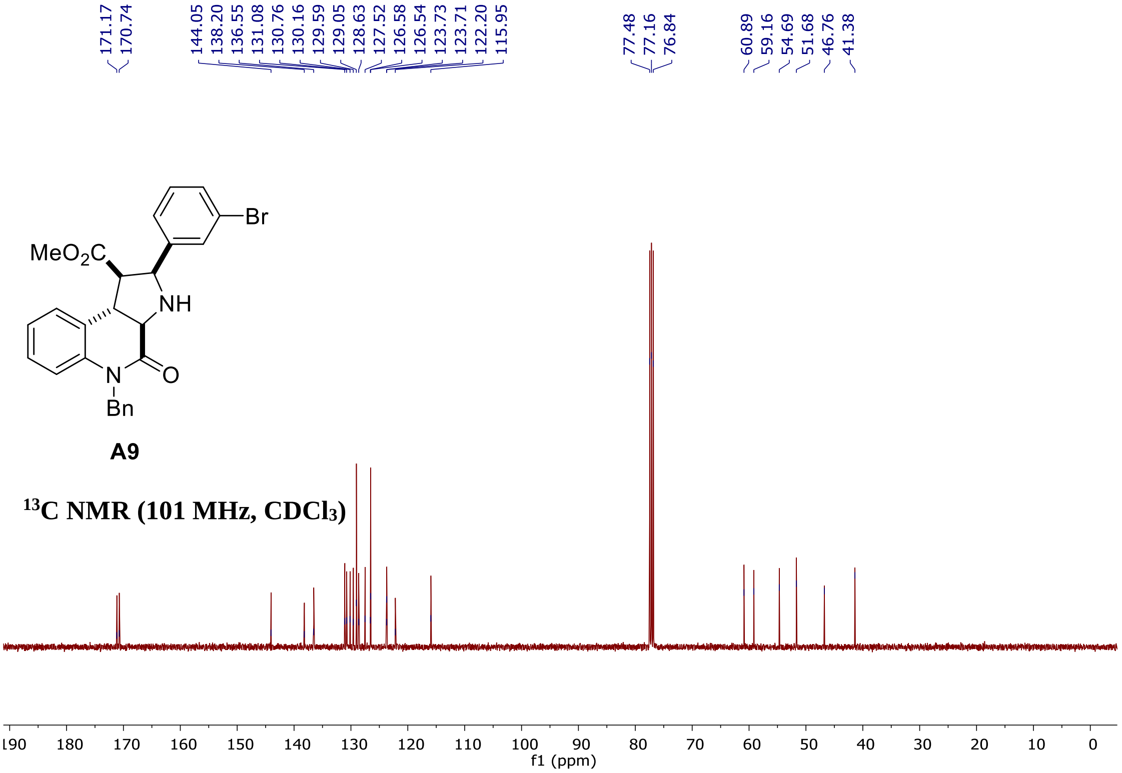

A9

^{13}C NMR (101 MHz, CDCl3)

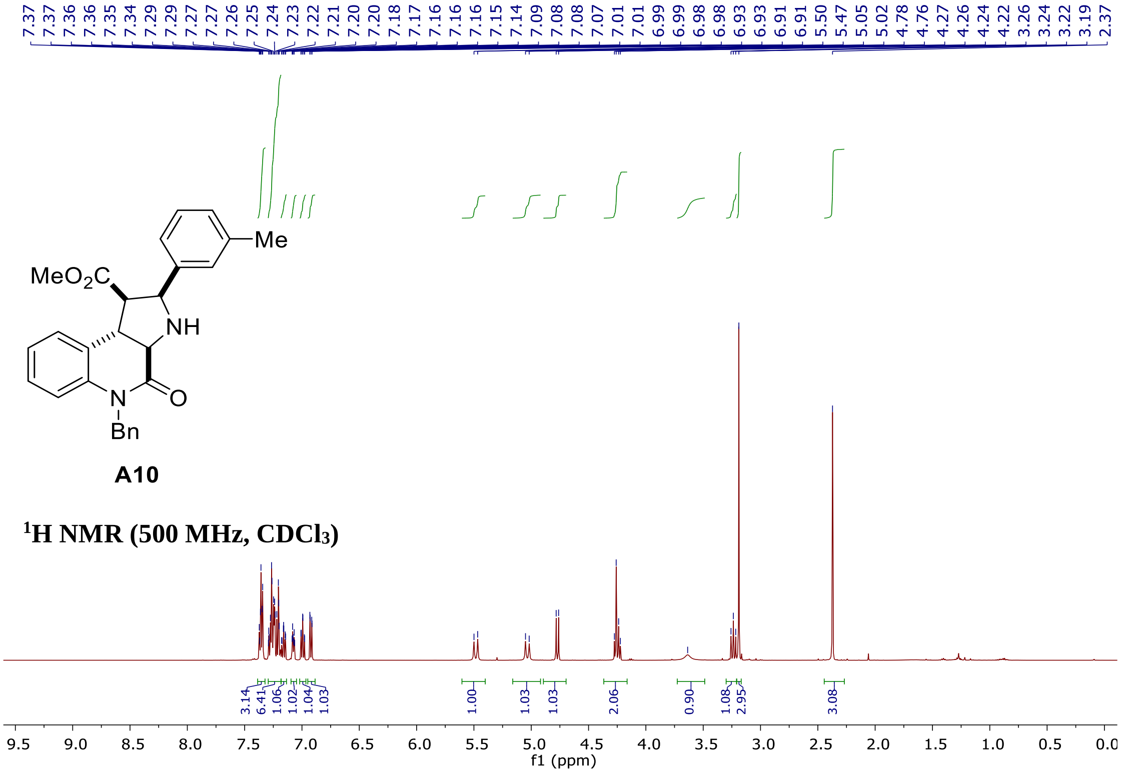
7.37 7.37 7.36 7.36 7.35 7.34 7.29 7.29 7.27 7.27 7.26 7.25 7.24 7.23 7.22 7.21 7.20 7.20 7.18 7.17 7.16 7.16 7.16 7.15 7.14 7.09 7.08 7.08 7.07 7.01 7.01 6.99 6.99 6.98 6.98 6.93 6.93 6.91 6.91 5.50 5.47 5.05 5.02 4.78 4.76 4.27 4.26 4.24 4.22 3.26 3.24 3.22 3.19 2.37
MeO2C
Me
NH
N
O
Bn
A10
1H NMR (500 MHz, CDCl3)
3.14 6.41 1.06 1.02 1.04 1.03 1.00 1.03 1.03 2.06 0.90 1.08 2.95 3.08
9.5 9.0 8.5 8.0 7.5 7.0 6.5 6.0 5.5 5.0 4.5 4.0 3.5 3.0 2.5 2.0 1.5 1.0 0.5 0.0
f1 (ppm)

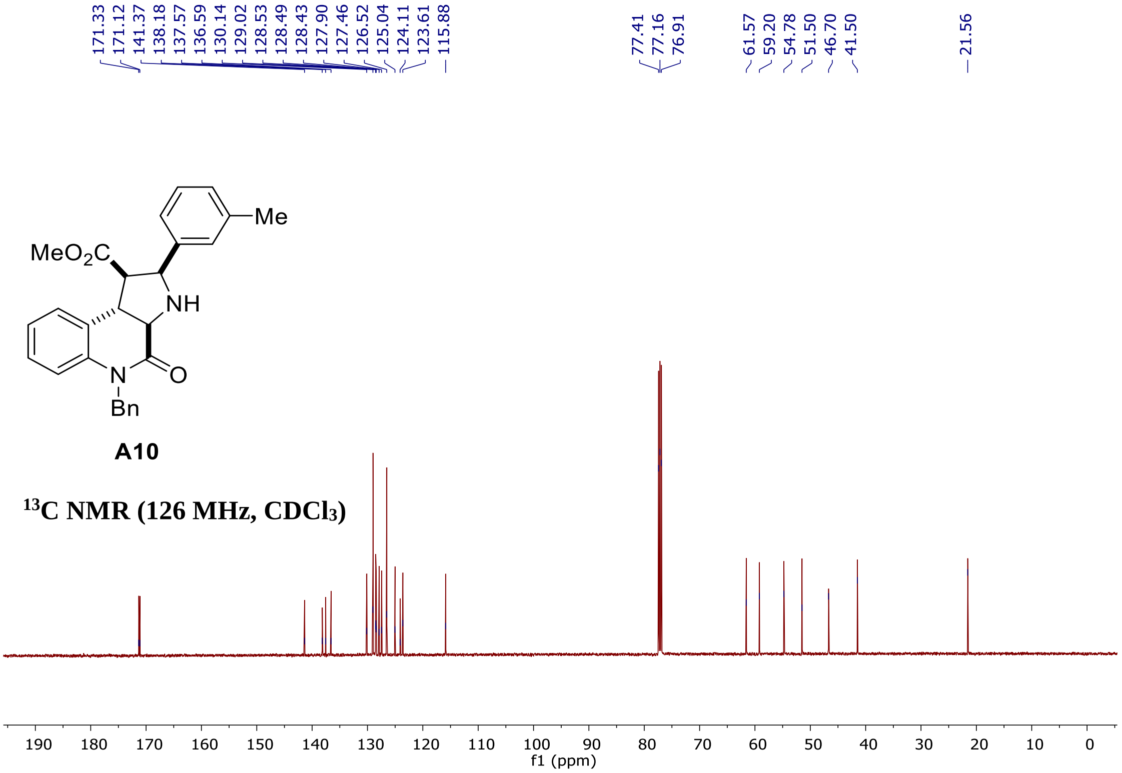
171.33 171.12 141.37 138.18 137.57 136.59 130.14 129.02 128.53 128.49 128.43 127.90 127.46 126.52 125.04 124.11 123.61 115.88 77.41 77.16 76.91 61.57 59.20 54.78 51.50 46.70 41.50 21.56
MeO2C
Me
NH
N
O
Bn
A10
13C NMR (126 MHz, CDCl3)
190 180 170 160 150 140 130 120 110 100 90 80 70 60 50 40 30 20 10 0
f1 (ppm)

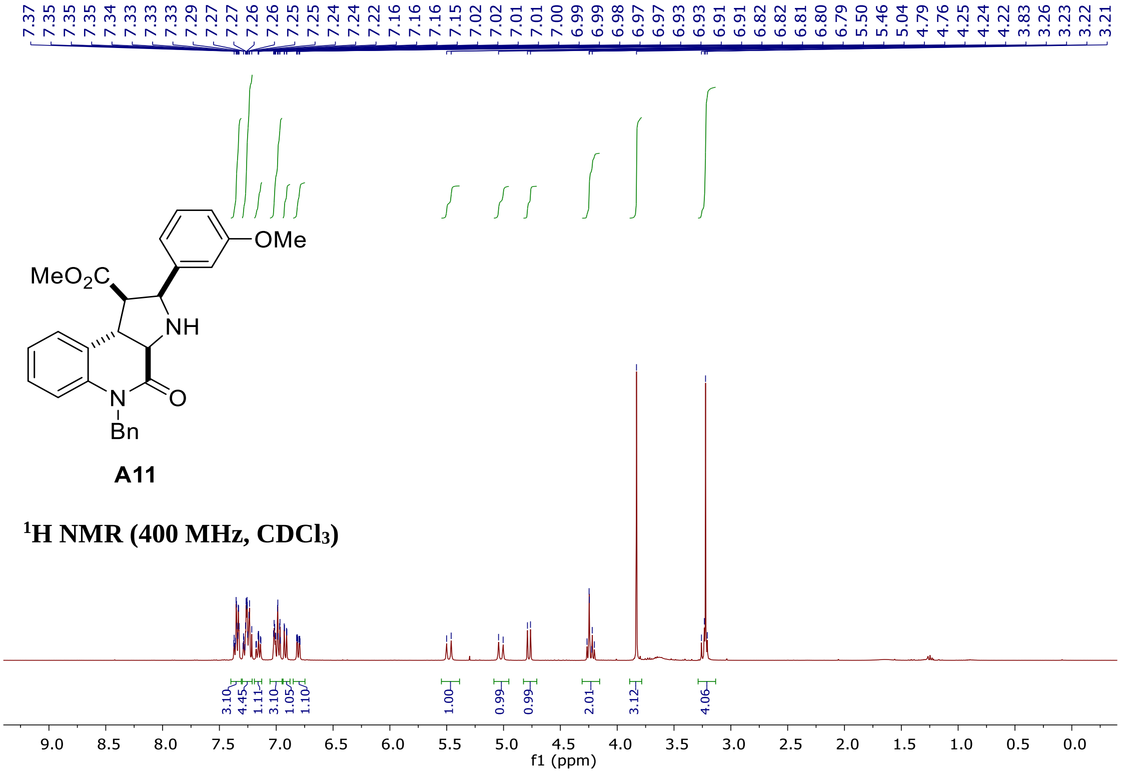
7.37 7.35 7.35 7.35 7.34 7.33 7.33 7.33 7.33 7.29 7.27 7.27 7.26 7.26 7.25 7.25 7.24 7.24 7.22 7.16 7.16 7.15 7.02 7.02 7.01 7.01 7.00 6.99 6.99 6.98 6.97 6.97 6.93 6.93 6.91 6.91 6.82 6.82 6.81 6.80 6.79 5.50 5.46 5.04 4.79 4.76 4.25 4.24 4.22 3.83 3.26 3.23 3.22 3.21
OMe
MeO2C
NH
N
O
Bn
A11
1H NMR (400 MHz, CDCl3)
3.10 4.45 1.11 3.10 1.05 1.10 1.00 0.99 0.99 2.01 3.12 4.06
9.0 8.5 8.0 7.5 7.0 6.5 6.0 5.5 5.0 4.5 4.0 3.5 3.0 2.5 2.0 1.5 1.0 0.5 0.0
f1 (ppm)

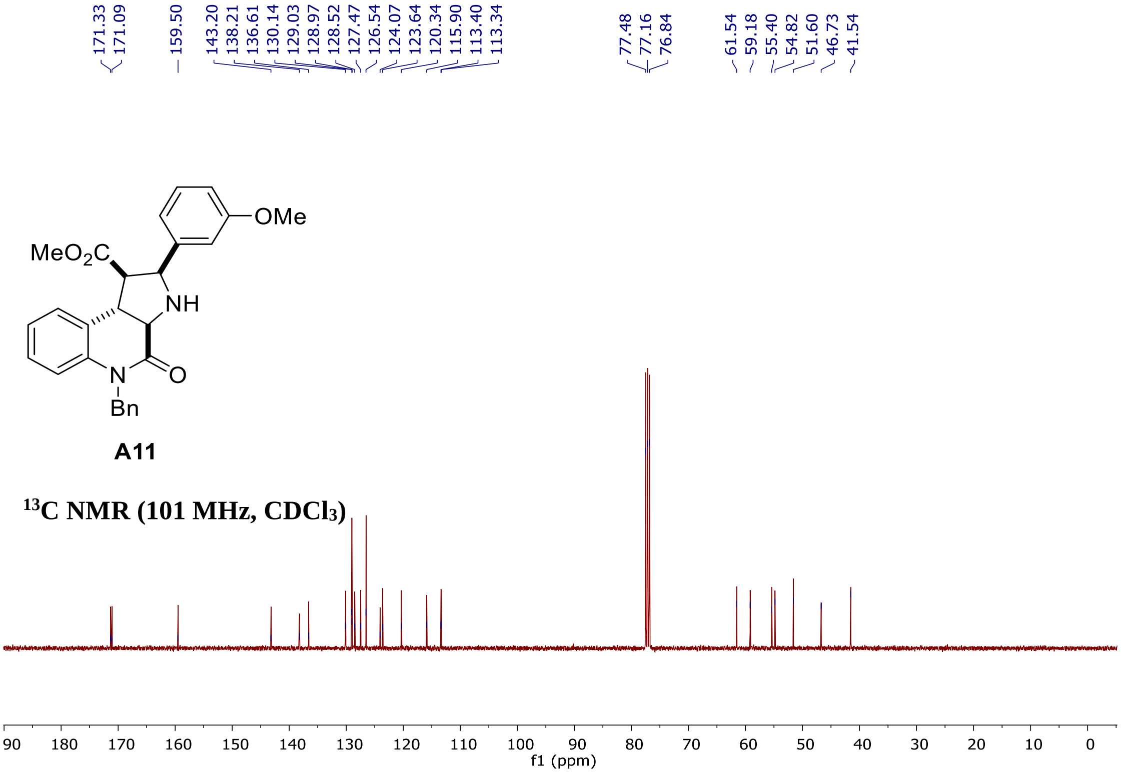
171.33 171.09 159.50 143.20 138.21 136.61 130.14 129.03 128.97 128.52 127.47 126.54 124.07 123.64 120.34 115.90 113.40 113.34 77.48 77.16 76.84 61.54 59.18 55.40 54.82 51.60 46.73 41.54
OMe
MeO2C
NH
N
O
Bn
A11
13C NMR (101 MHz, CDCl3)
90 180 170 160 150 140 130 120 110 100 90 80 70 60 50 40 30 20 10 0
f1 (ppm)

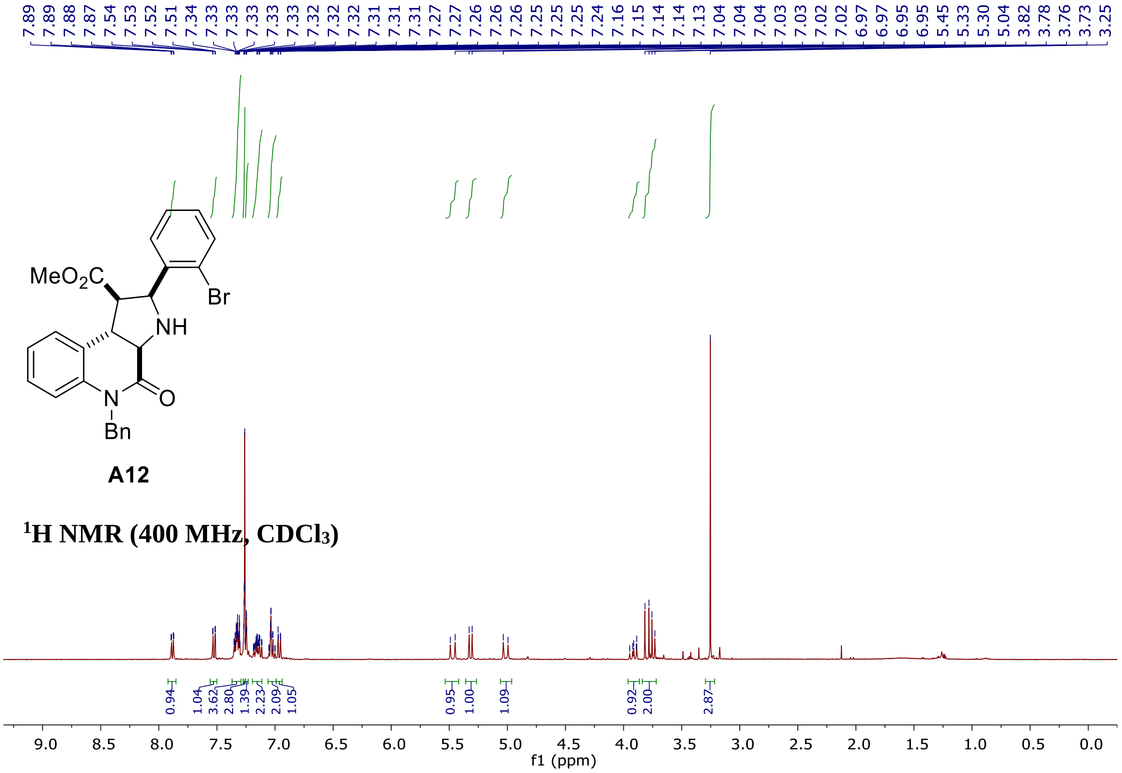
7.89 7.89 7.88 7.87 7.54 7.53 7.52 7.51 7.34 7.33 7.33 7.33 7.33 7.33 7.32 7.32 7.31 7.31 7.31 7.27 7.26 7.26 7.25 7.25 7.25 7.24 7.16 7.15 7.14 7.13 7.04 7.04 7.04 7.03 7.03 7.02 7.02 6.97 6.97 6.95 6.95 5.45 5.33 5.30 5.04 3.82 3.78 3.76 3.73 3.25
MeO2C
Br
NH
N
O
Bn
A12
1H NMR (400 MHz, CDCl3)
0.94 1.04 3.62 2.80 1.39 2.23 2.09 1.05 0.95 1.00 1.09 0.92 2.00 2.87
9.0 8.5 8.0 7.5 7.0 6.5 6.0 5.5 5.0 4.5 4.0 3.5 3.0 2.5 2.0 1.5 1.0 0.5 0.0
f1 (ppm)

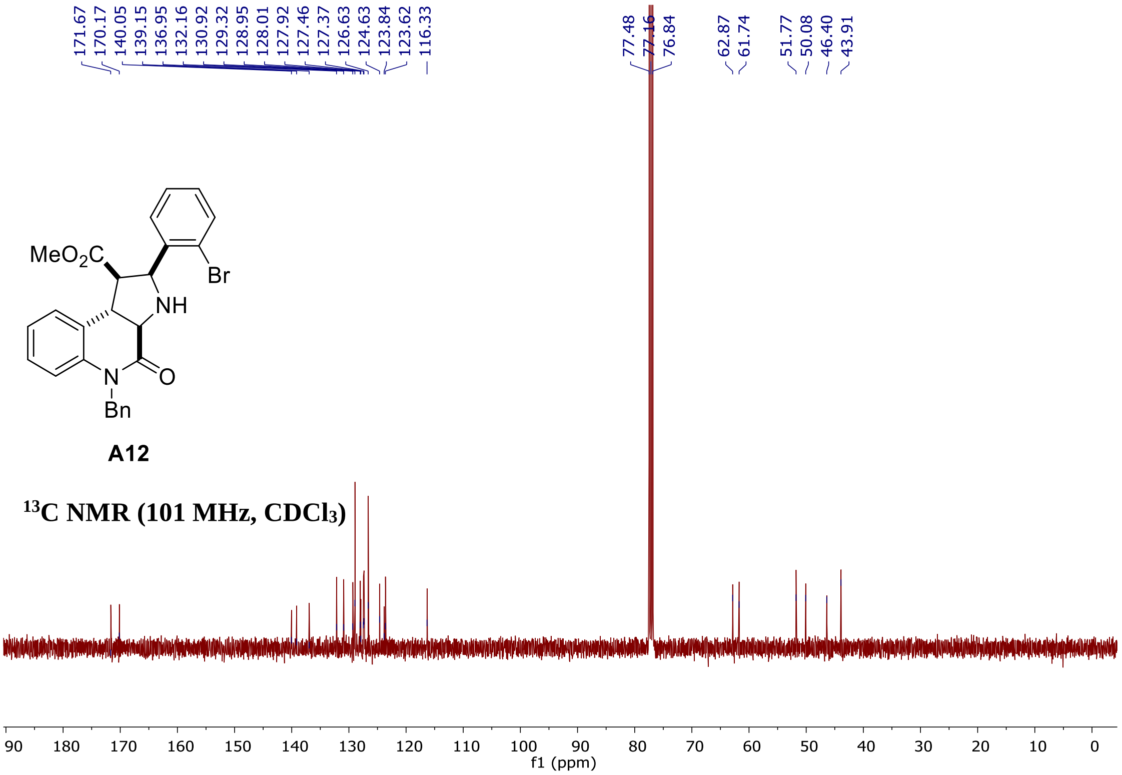
171.67 170.17 140.05 139.15 136.95 132.16 130.92 129.32 128.95 128.01 127.92 127.46 127.37 126.63 124.63 123.84 123.62 116.33
77.48 77.16 76.84
62.87 61.74
51.77 50.08 46.40 43.91
MeO2C
Br
NH
N
O
Bn
A12
13C NMR (101 MHz, CDCl3)
90 180 170 160 150 140 130 120 110 100 90 80 70 60 50 40 30 20 10 0
f1 (ppm)

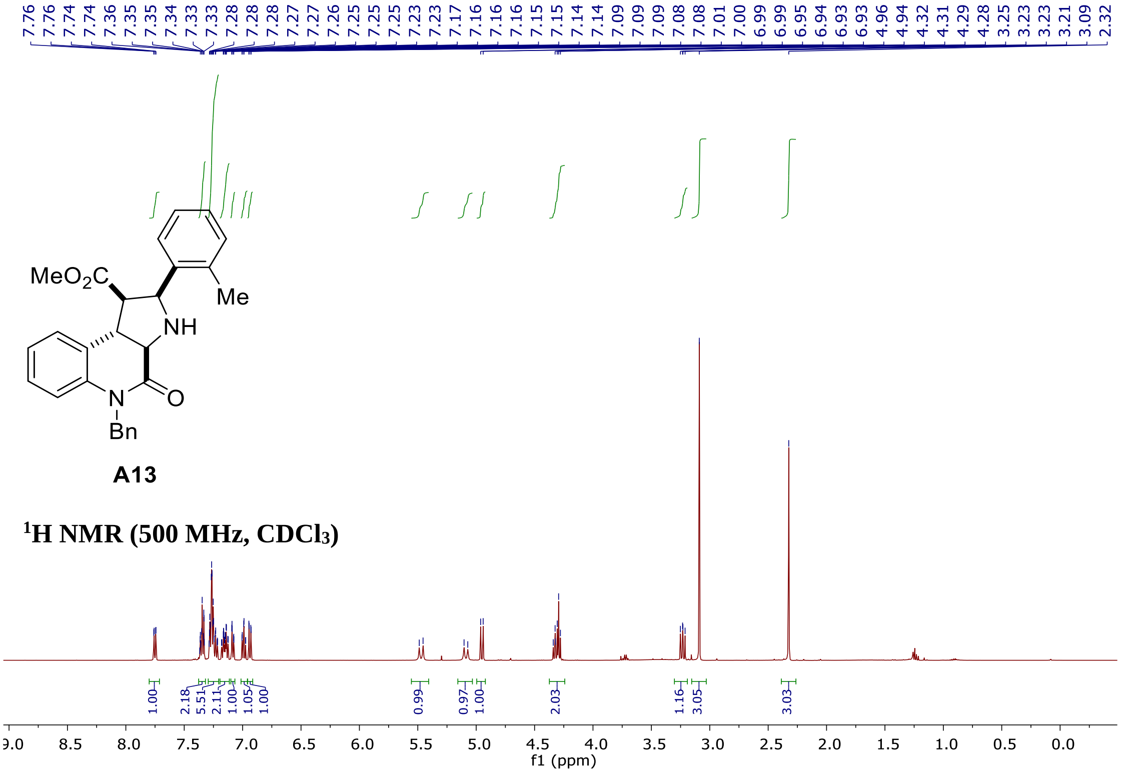
7.76
7.76
7.74
7.74
7.36
7.35
7.35
7.34
7.33
7.33
7.28
7.28
7.27
7.27
7.26
7.25
7.25
7.25
7.23
7.23
7.17
7.16
7.16
7.15
7.15
7.14
7.09
7.09
7.09
7.08
7.08
7.01
7.00
6.99
6.99
6.95
6.94
6.93
6.93
4.96
4.94
4.32
4.31
4.29
4.28
3.25
3.23
3.23
3.21
3.09
2.32
MeO2C
Me
NH
N
O
Bn
A13
1H NMR (500 MHz, CDCl3)
1.00
2.18
5.51
2.11
1.00
1.05
1.00
0.99
0.97
1.00
2.03
1.16
3.05
3.03
f1 (ppm)

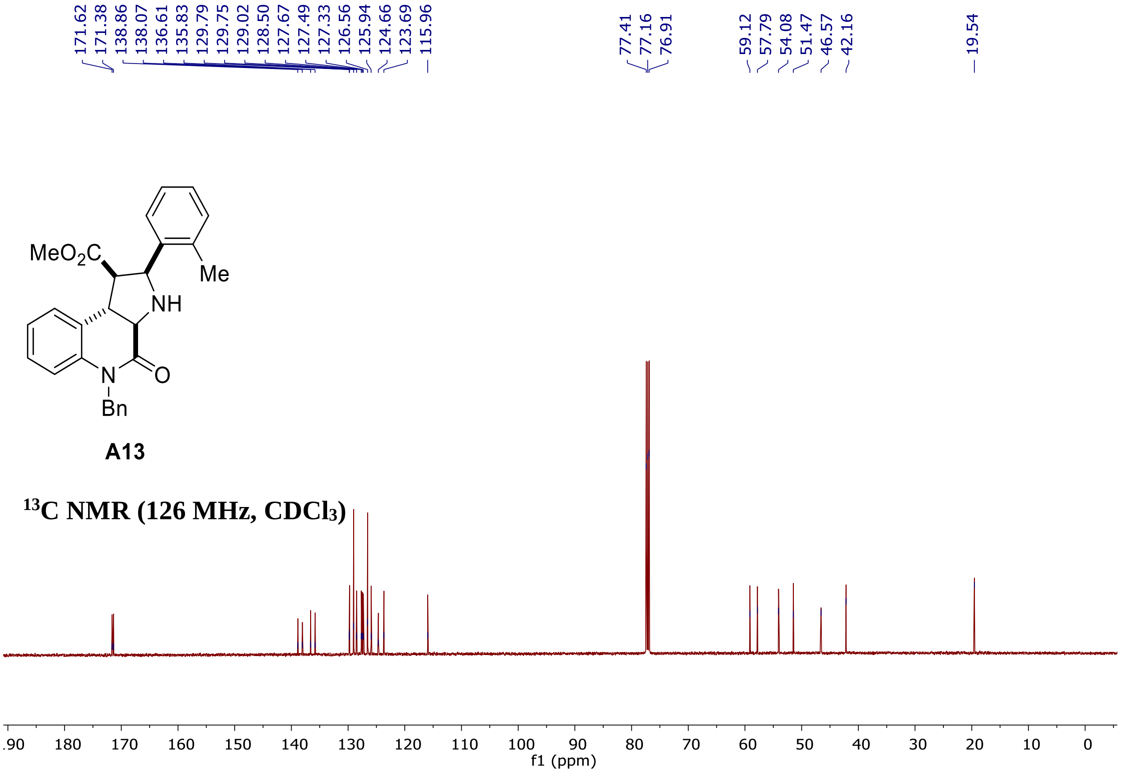
171.62
171.38
138.86
138.07
136.61
135.83
129.79
129.75
129.02
128.50
127.67
127.49
127.33
126.56
125.94
124.66
123.69
115.96
77.41
77.16
76.91
59.12
57.79
54.08
51.47
46.57
42.16
19.54
MeO2C
Me
NH
N
O
Bn
A13
13C NMR (126 MHz, CDCl3)
f1 (ppm)

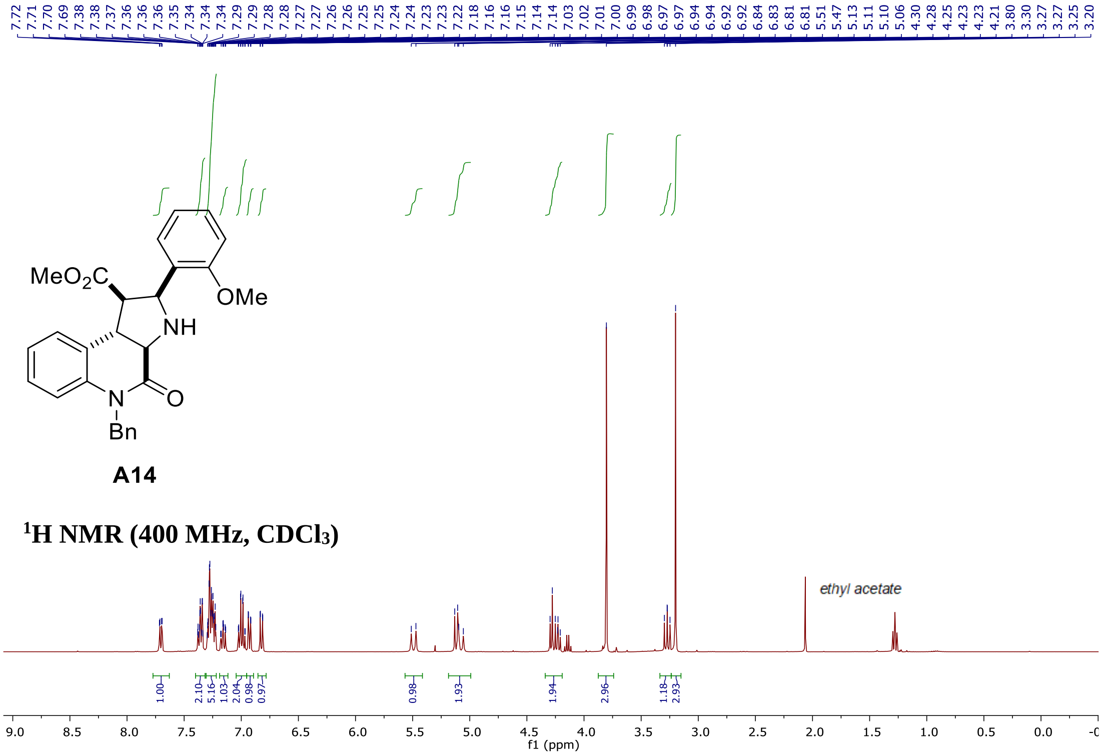

A14

^{1}H NMR (400 MHz, $CDCl_3$)

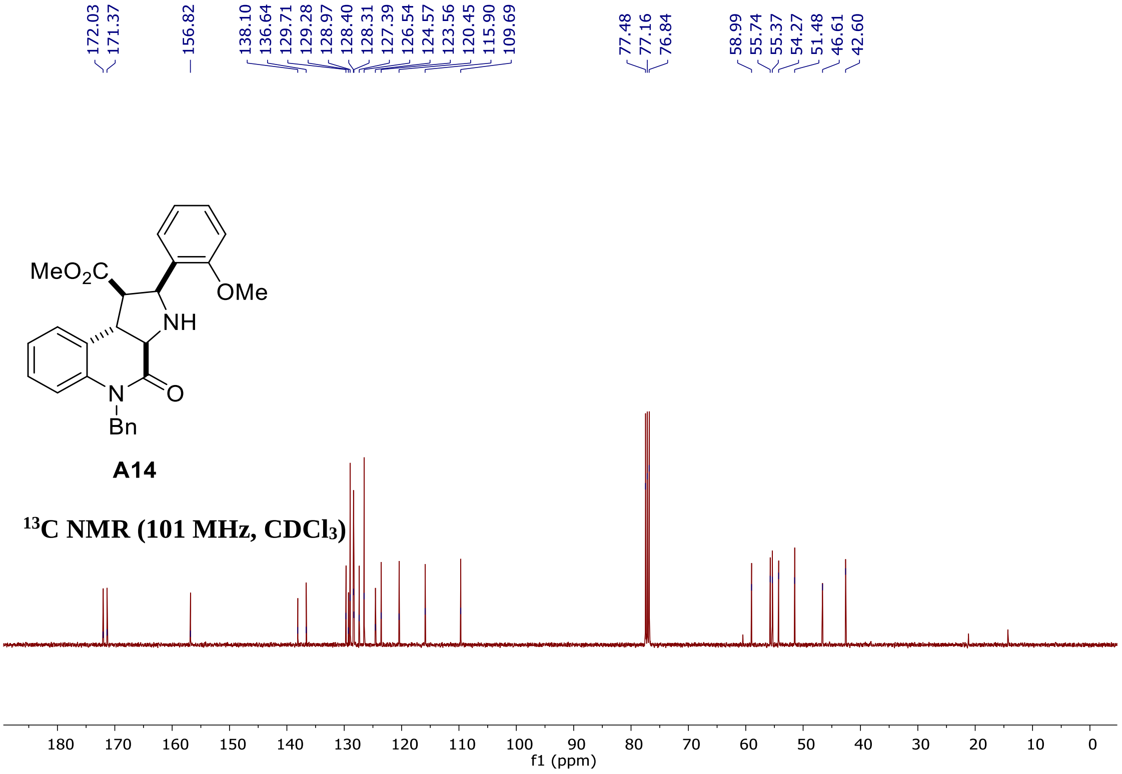

A14

^{13}C NMR (101 MHz, $CDCl_3$)

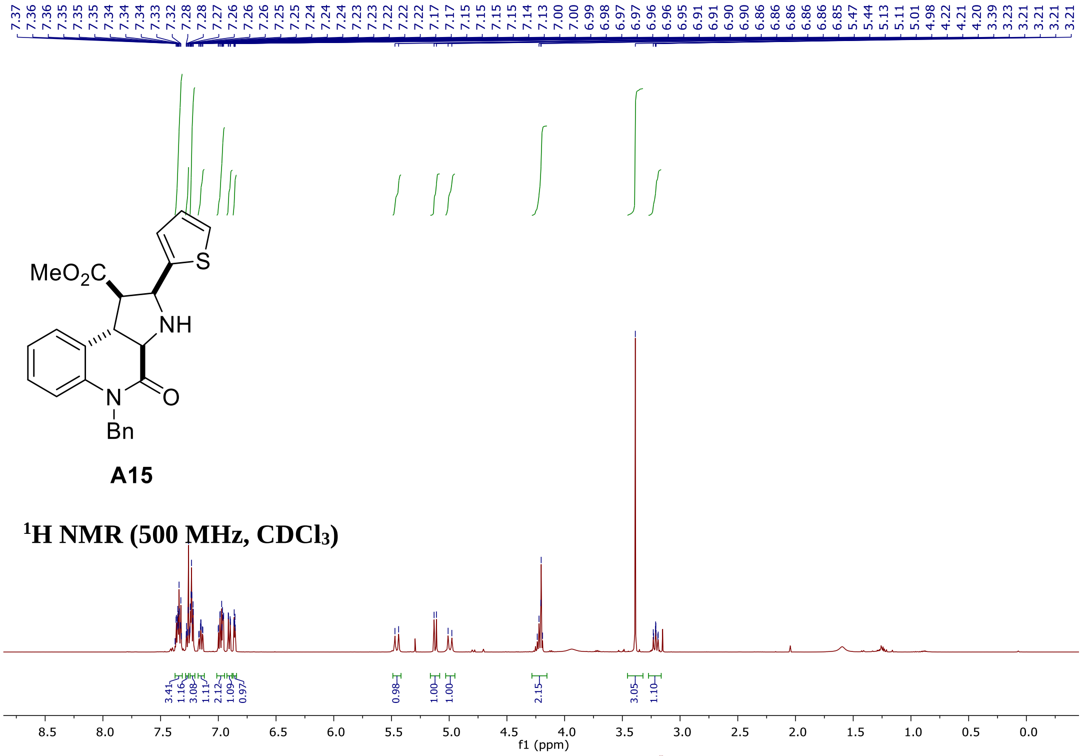
7.37 7.36 7.35 7.35 7.35 7.34 7.34 7.34 7.33 7.32 7.28 7.28 7.27 7.26 7.26 7.26 7.25 7.25 7.24 7.24 7.24 7.23 7.23 7.22 7.22 7.22 7.22 7.17 7.17 7.15 7.15 7.15 7.15 7.14 7.13 7.00 6.99 6.98 6.97 6.97 6.96 6.96 6.95 6.91 6.91 6.90 6.90 6.86 6.86 6.86 6.86 6.85 5.47 5.44 5.13 5.11 5.01 4.98 4.22 4.21 4.20 3.39 3.23 3.21 3.21 3.21
MeO2C
S
NH
N
O
Bn
A15
1H NMR (500 MHz, CDCl3)
3.41 1.16 3.08 1.11 2.12 1.09 0.97 0.98 1.00 1.00 2.15 3.05 1.10
8.5 8.0 7.5 7.0 6.5 6.0 5.5 5.0 4.5 4.0 3.5 3.0 2.5 2.0 1.5 1.0 0.5 0.0
f1 (ppm)

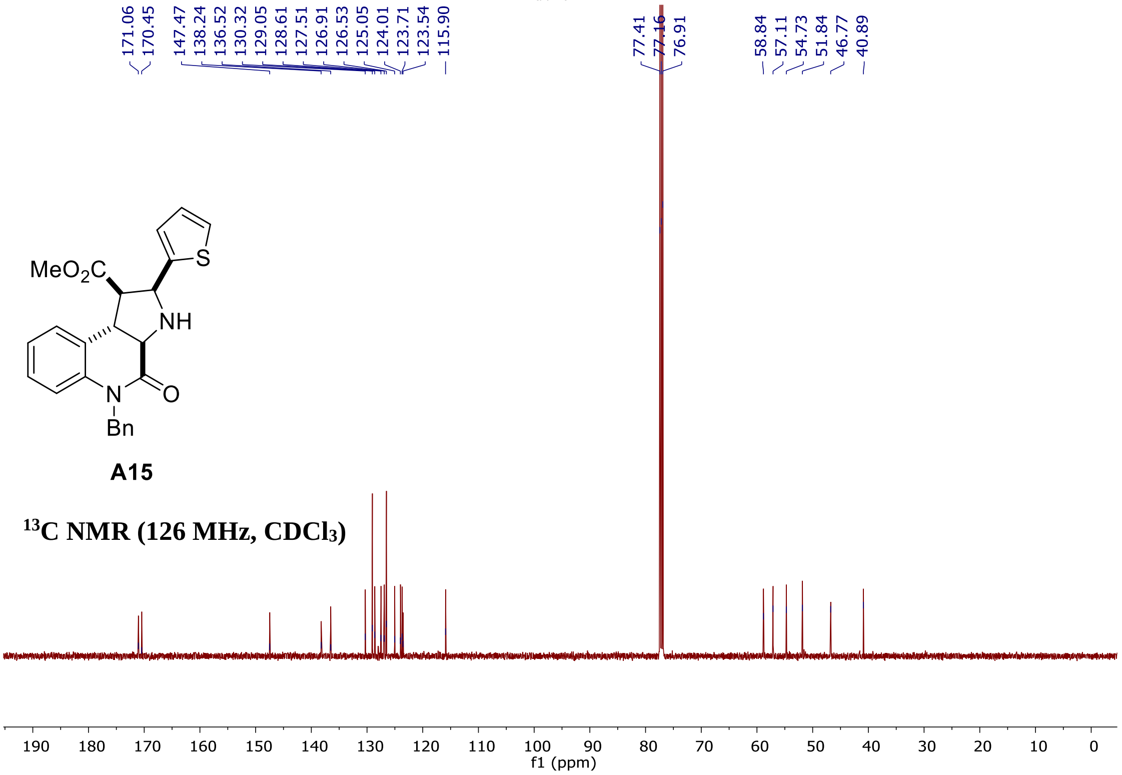
171.06 170.45 147.47 138.24 136.52 130.32 129.05 128.61 127.51 126.91 126.53 125.05 124.01 123.71 123.54 115.90 77.41 77.16 76.91 58.84 57.11 54.73 51.84 46.77 40.89
MeO2C
S
NH
N
O
Bn
A15
13C NMR (126 MHz, CDCl3)
190 180 170 160 150 140 130 120 110 100 90 80 70 60 50 40 30 20 10 0
f1 (ppm)

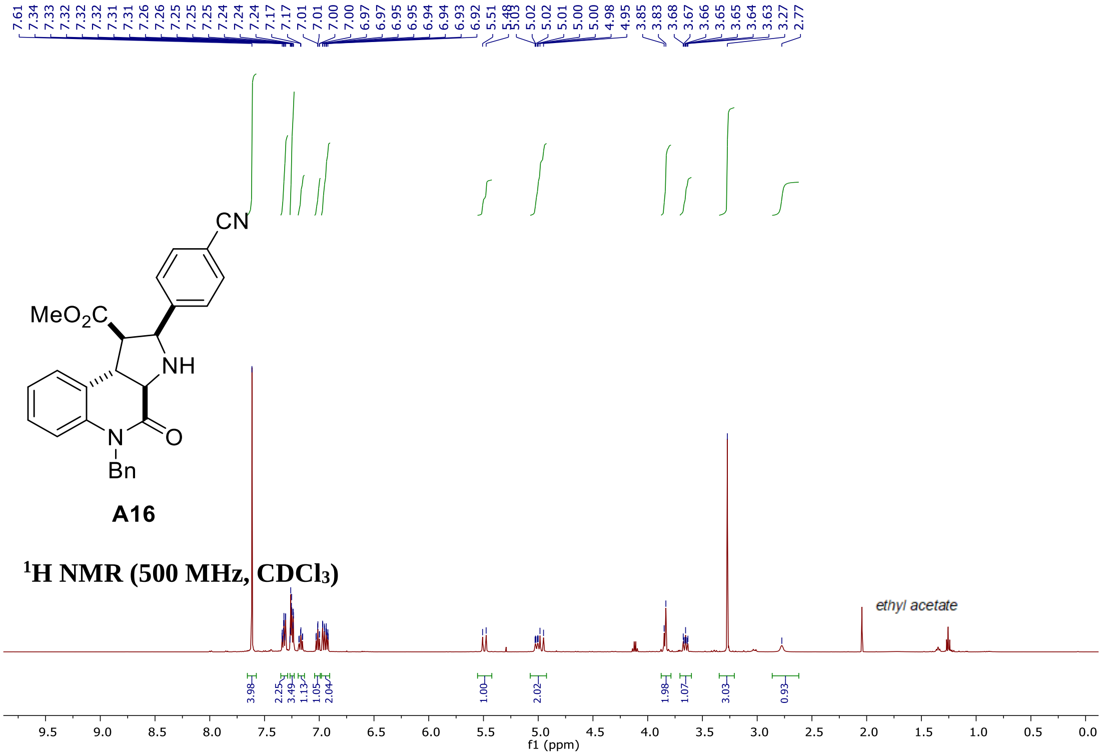
7.61
7.34
7.33
7.32
7.32
7.32
7.31
7.31
7.26
7.26
7.25
7.25
7.25
7.24
7.24
7.24
7.17
7.17
7.01
7.01
7.00
7.00
6.97
6.97
6.95
6.95
6.94
6.94
6.93
6.92
5.51
5.48
5.03
5.02
5.02
5.01
5.00
5.00
4.98
4.95
3.85
3.83
3.68
3.67
3.66
3.65
3.65
3.64
3.63
3.27
2.77
CN
MeO2C
NH
N
O
Bn
A16
1H NMR (500 MHz, CDCl3)
ethyl acetate
3.98
2.25
3.49
1.13
1.05
2.04
1.00
2.02
1.98
1.07
3.03
0.93
f1 (ppm)

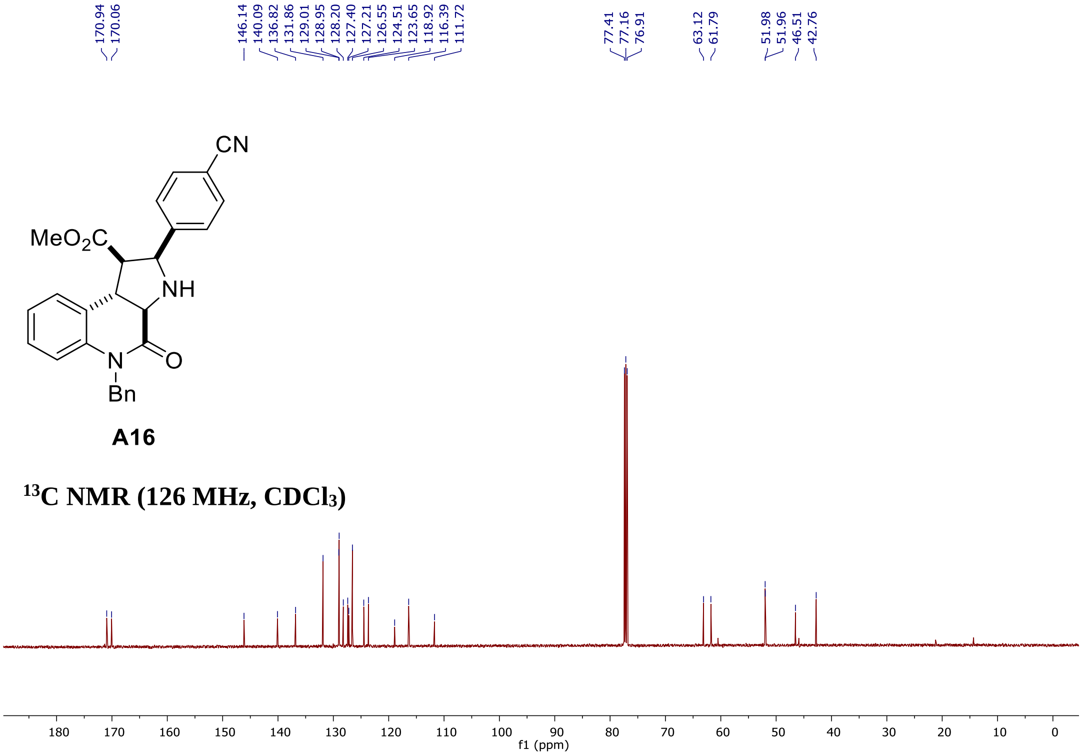
170.94
170.06
146.14
140.09
136.82
131.86
129.01
128.95
128.20
127.40
127.21
126.55
124.51
123.65
118.92
116.39
111.72
77.41
77.16
76.91
63.12
61.79
51.98
51.96
46.51
42.76
CN
MeO2C
NH
N
O
Bn
A16
13C NMR (126 MHz, CDCl3)
f1 (ppm)

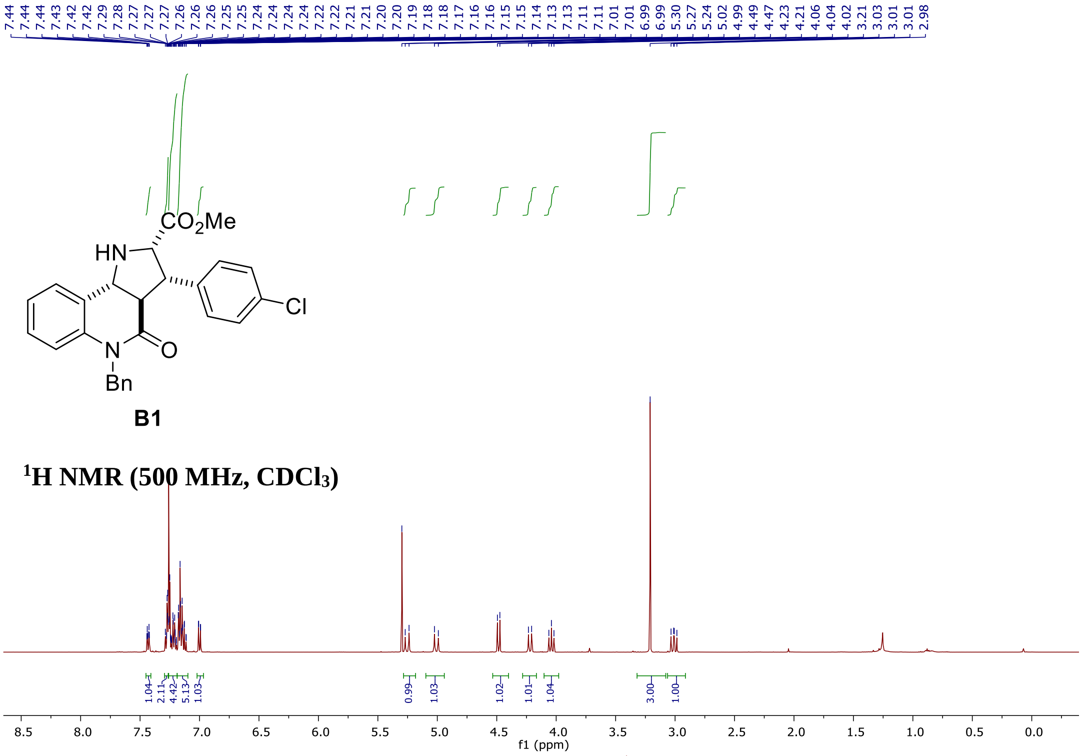

B1

^{1}H NMR (500 MHz, $CDCl_3$)

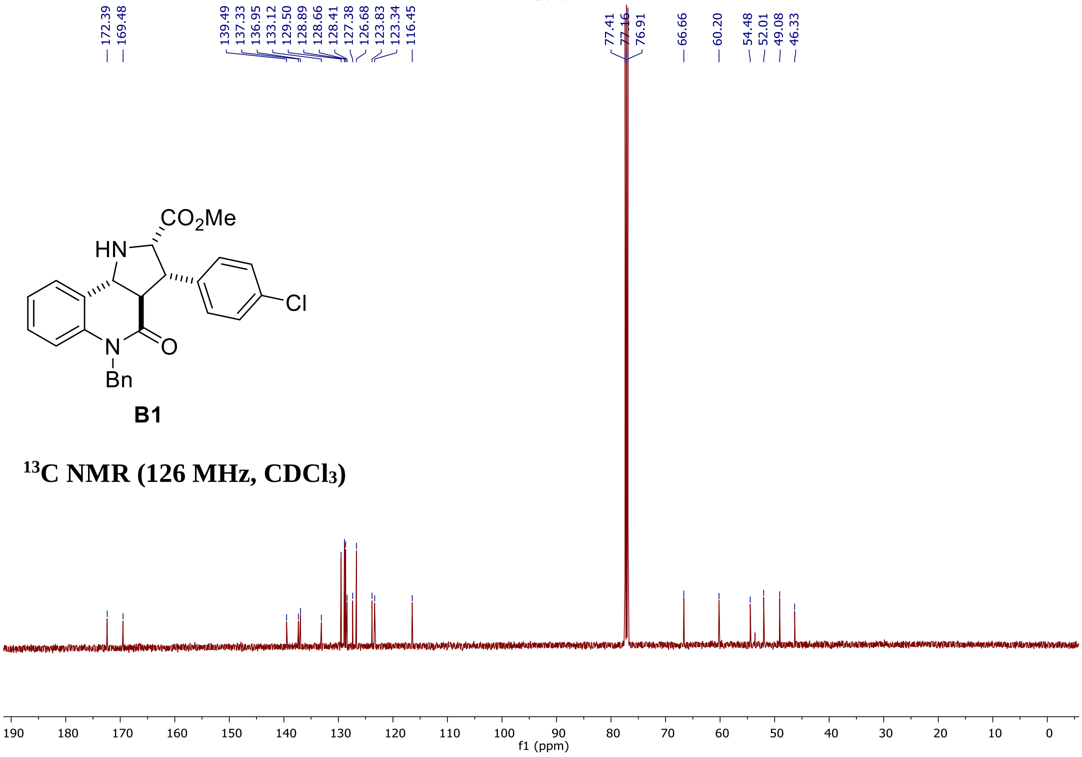

B1

^{13}C NMR (126 MHz, $CDCl_3$)

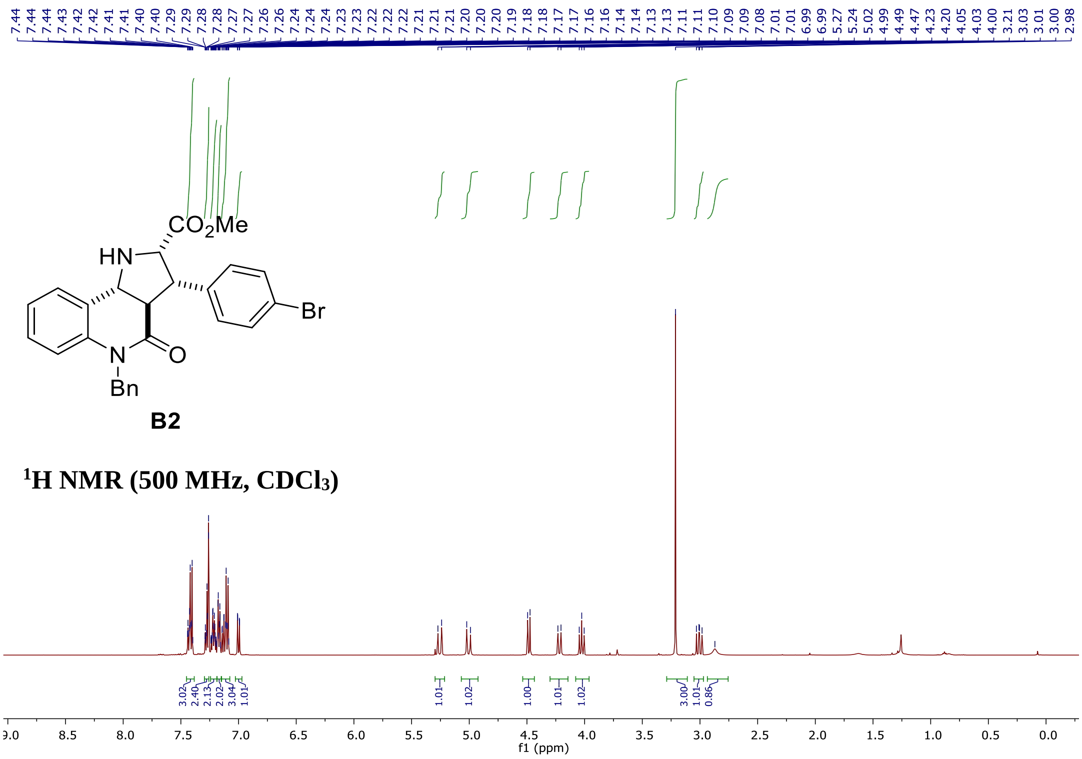
7.44 7.44 7.44 7.43 7.42 7.42 7.41 7.41 7.40 7.40 7.29 7.29 7.28 7.28 7.27 7.27 7.26 7.26 7.24 7.24 7.24 7.23 7.23 7.22 7.22 7.22 7.21 7.21 7.21 7.20 7.20 7.20 7.19 7.18 7.18 7.17 7.17 7.16 7.16 7.14 7.14 7.13 7.13 7.11 7.11 7.10 7.09 7.09 7.08 7.01 7.01 6.99 6.99 5.27 5.24 5.02 4.99 4.49 4.47 4.23 4.20 4.05 4.03 4.00 3.21 3.03 3.01 3.00 2.98
CO2Me
HN
Br
N
O
Bn
B2
1H NMR (500 MHz, CDCl3)
3.02 2.40 2.13 2.02 3.04 1.01 1.01 1.02 1.00 1.01 1.02 3.00 1.01 0.86
9.0 8.5 8.0 7.5 7.0 6.5 6.0 5.5 5.0 4.5 4.0 3.5 3.0 2.5 2.0 1.5 1.0 0.5 0.0
f1 (ppm)

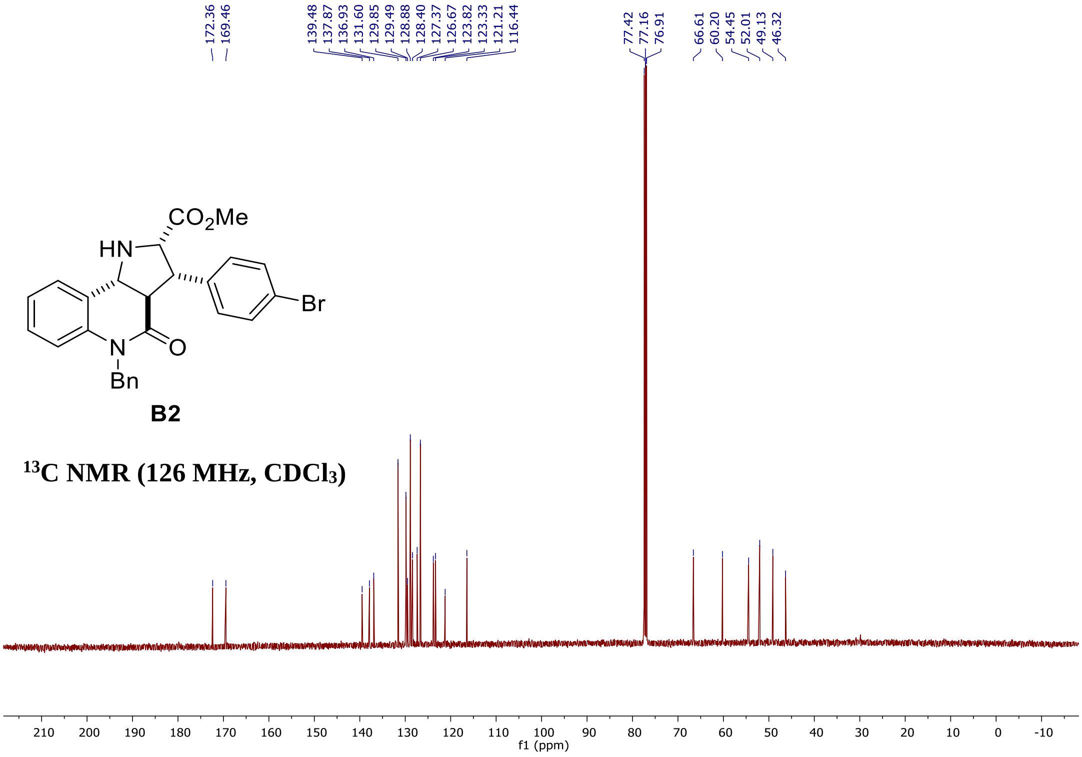
172.36 169.46 139.48 137.87 136.93 131.60 129.85 129.49 128.88 128.40 127.37 126.67 123.82 123.33 121.21 116.44 77.42 77.16 76.91 66.61 60.20 54.45 52.01 49.13 46.32
CO2Me
HN
Br
N
O
Bn
B2
13C NMR (126 MHz, CDCl3)
210 200 190 180 170 160 150 140 130 120 110 100 90 80 70 60 50 40 30 20 10 0 -10
f1 (ppm)

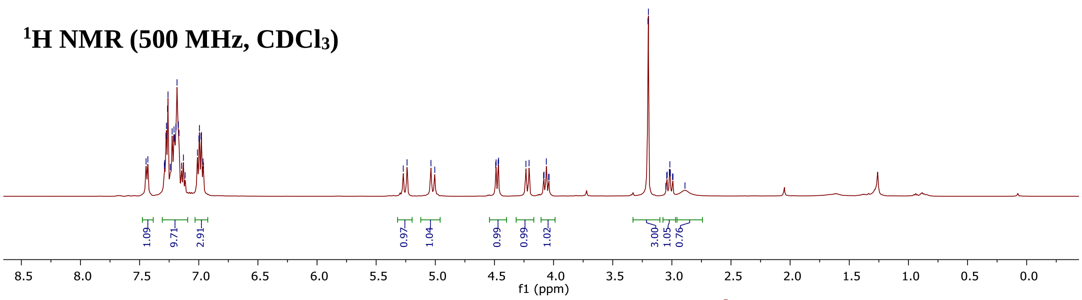

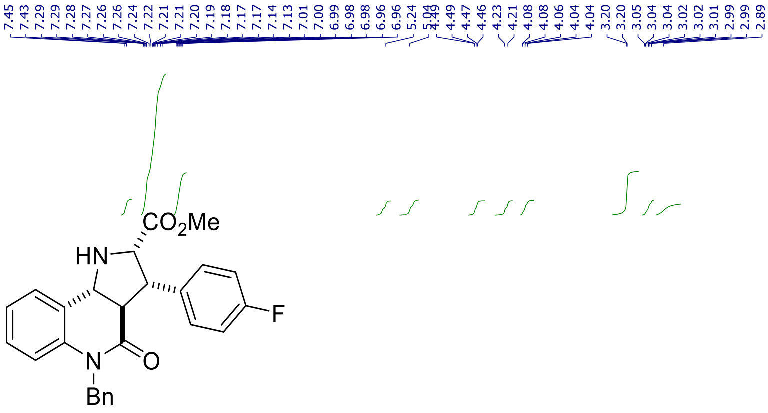

B3

^{1}H NMR (500 MHz, CDCl3)

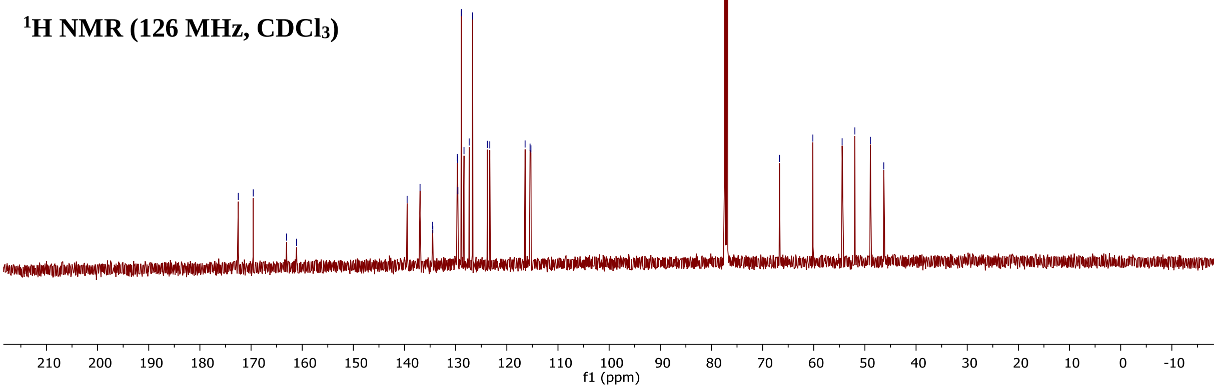

B3

^{1}H NMR (126 MHz, CDCl3)

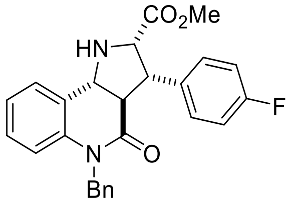

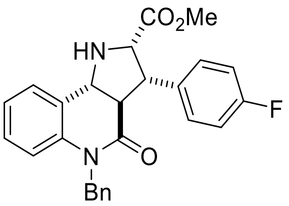

B3

^{19}F NMR (470 MHz, $CDCl_3$)

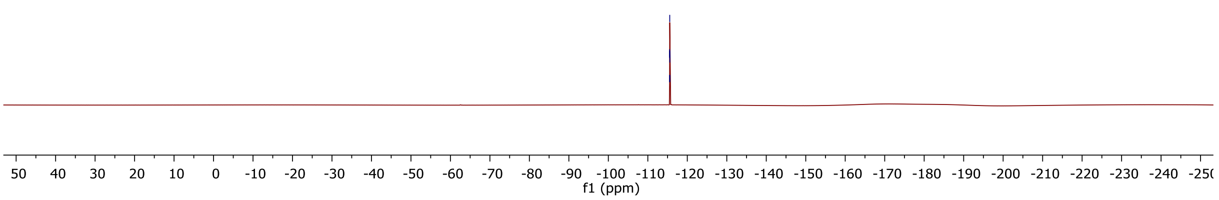

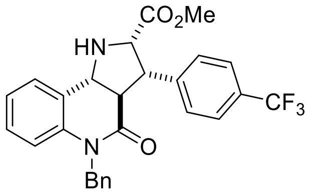

B4

^{19}F NMR (470 MHz, $CDCl_3$)

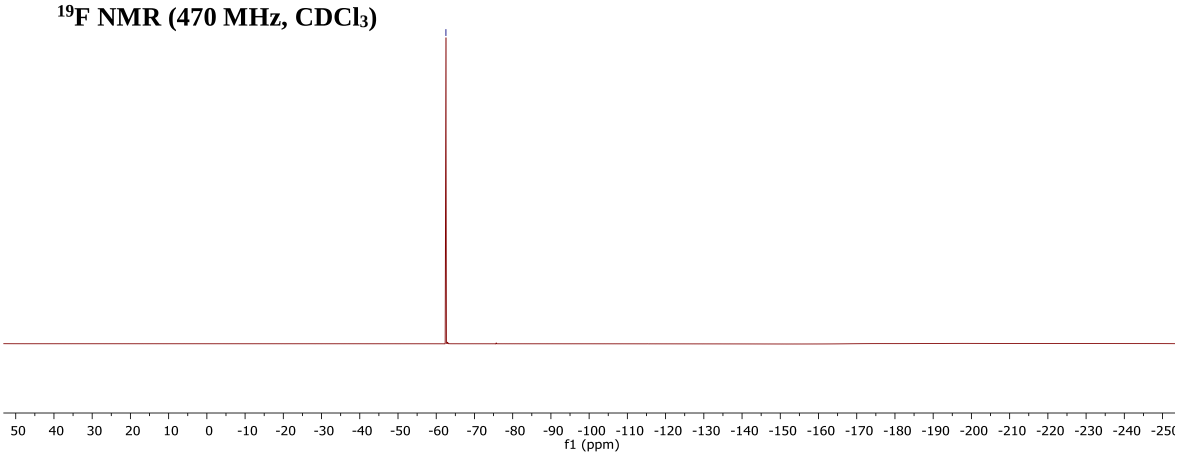

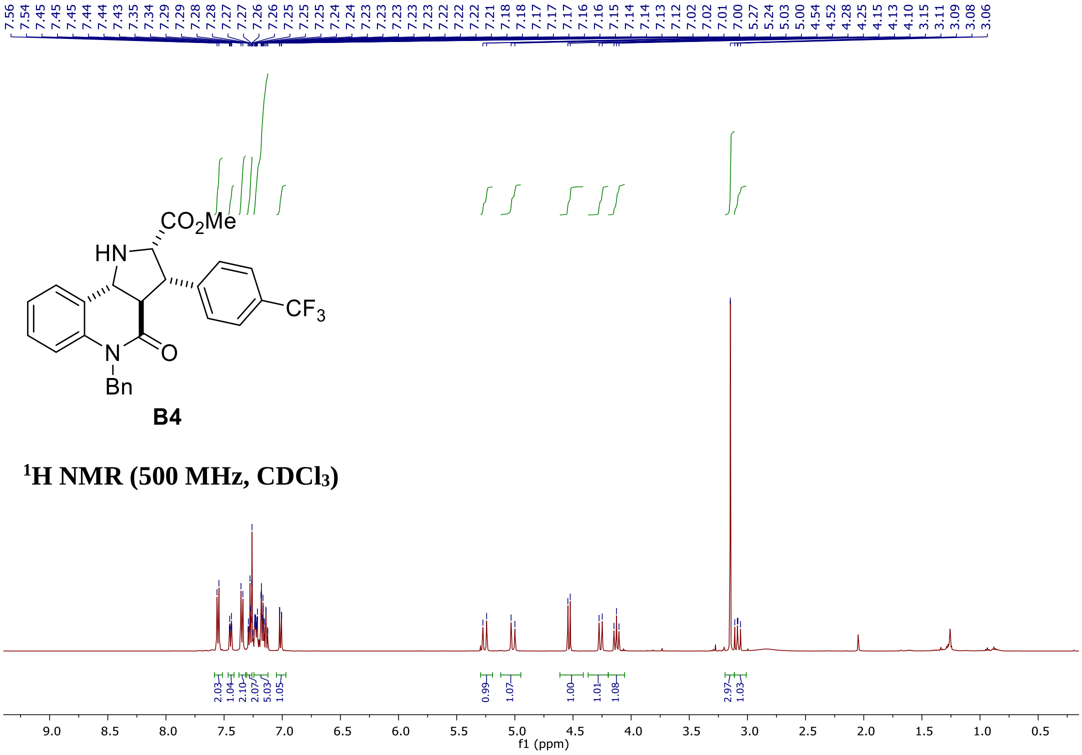

B4

^{1}H NMR (500 MHz, CDCl$_3$)

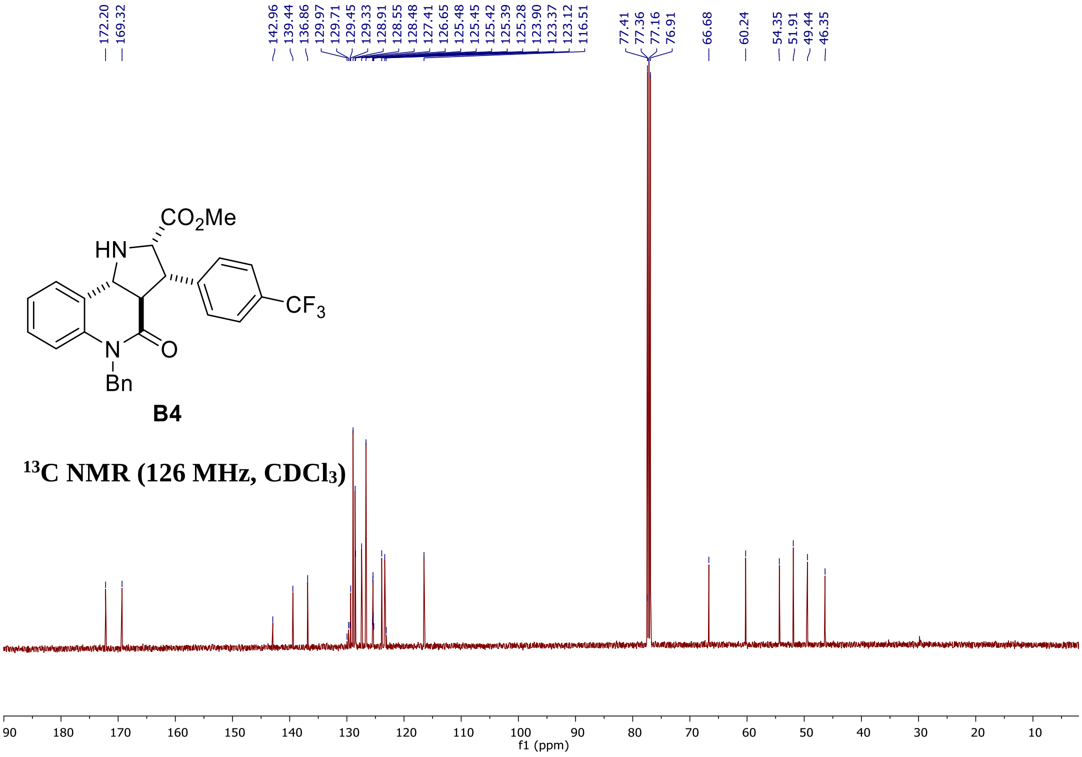

B4

^{13}C NMR (126 MHz, CDCl$_3$)

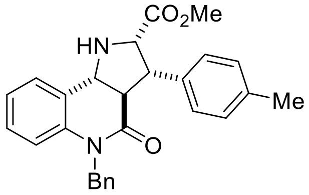

B5

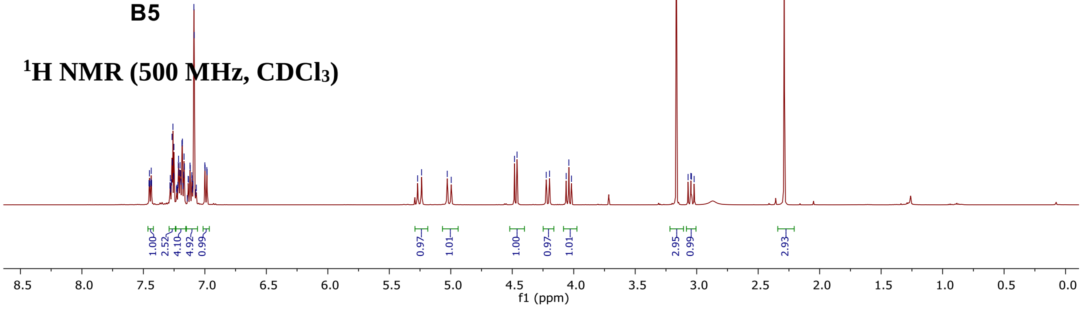

^{1}H NMR (500 MHz, $CDCl_3$)

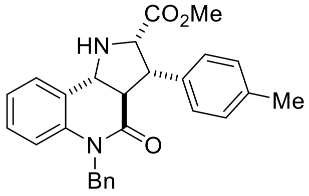

B5

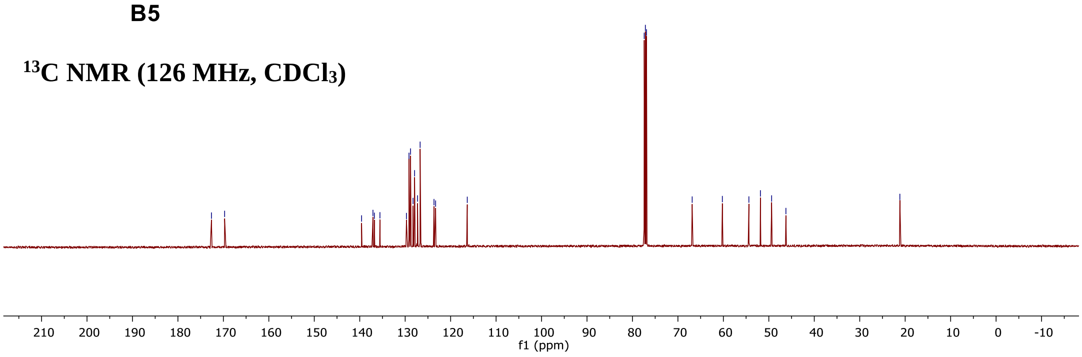

^{13}C NMR (126 MHz, $CDCl_3$)

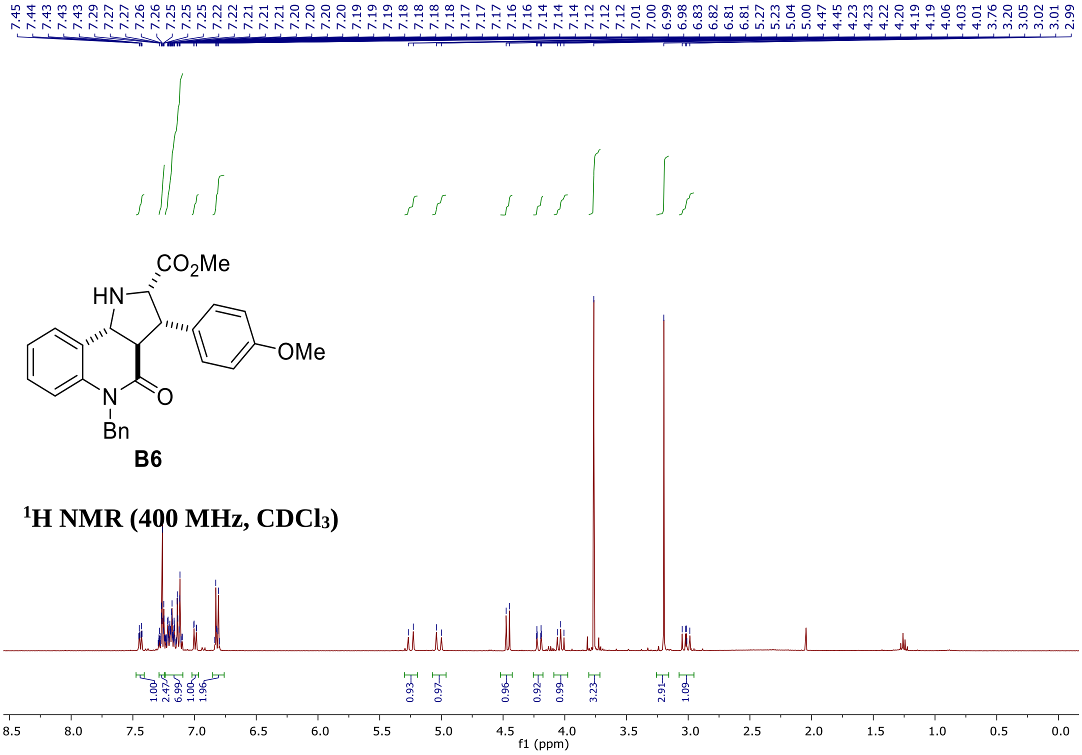

B6

^{1}H NMR (400 MHz, CDCl$_3$)

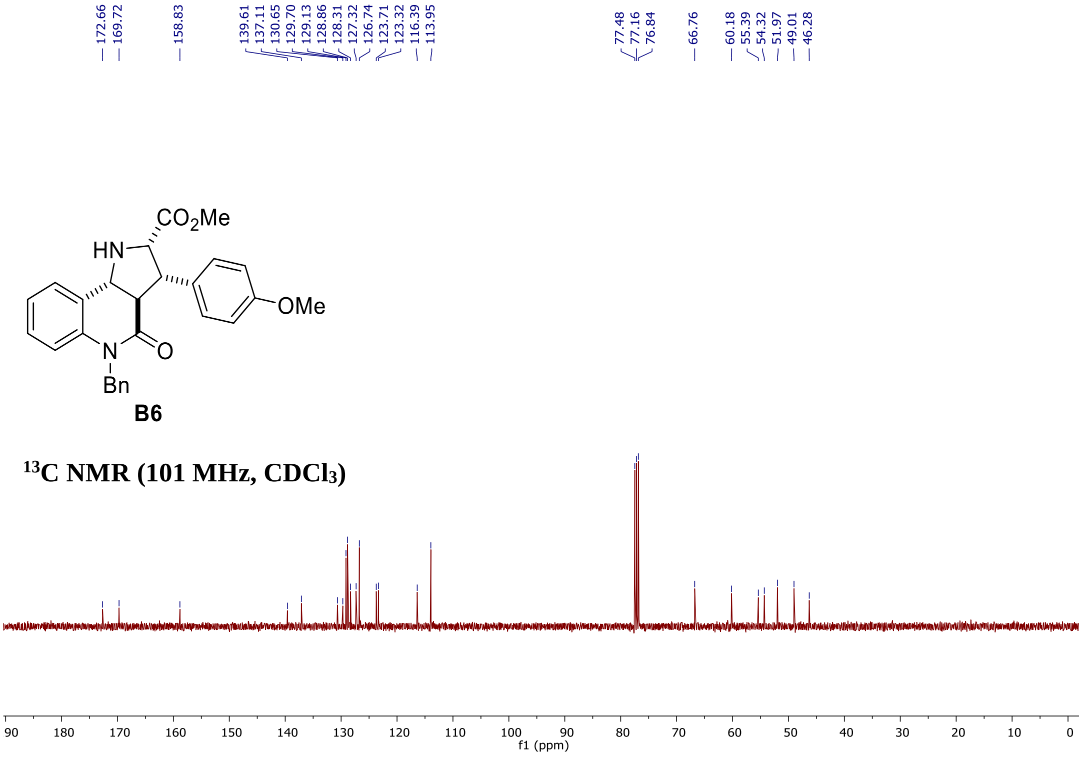

B6

^{13}C NMR (101 MHz, CDCl$_3$)

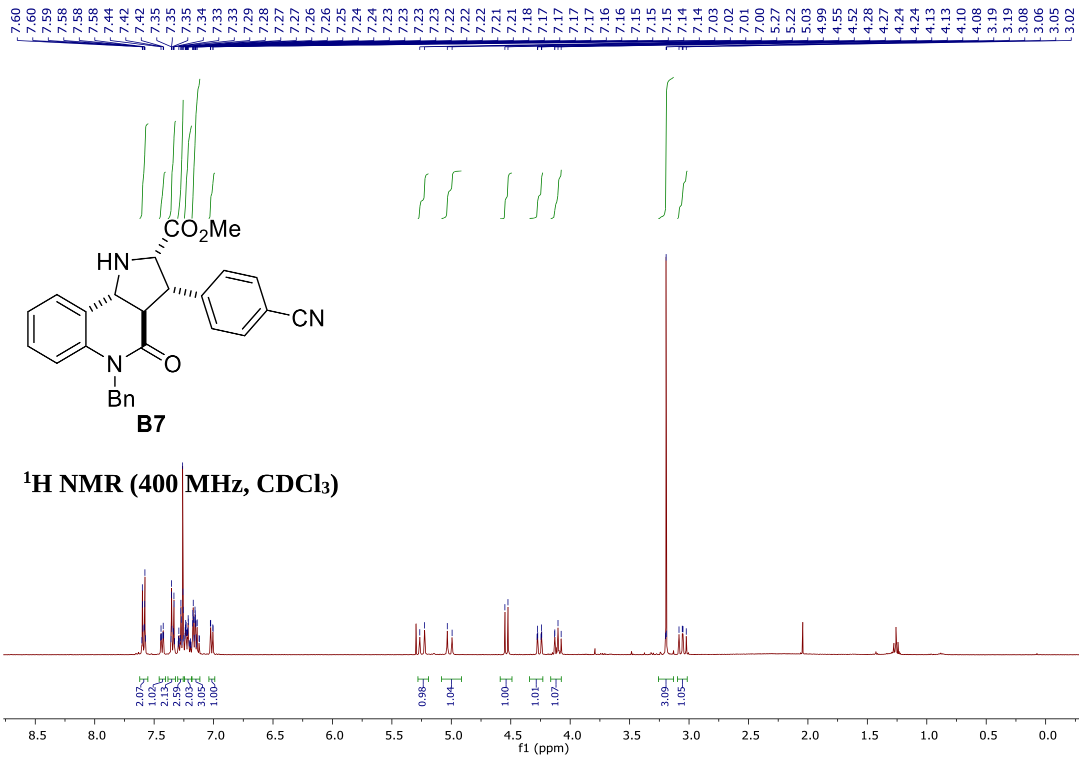

^{1}H NMR (400 MHz, $CDCl_3$)

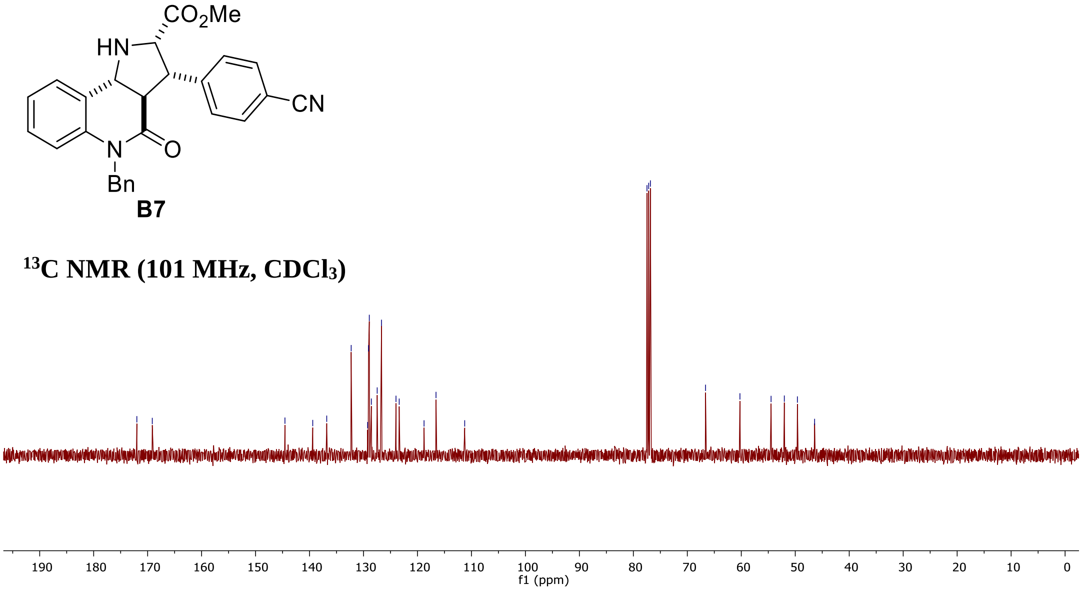

^{13}C NMR (101 MHz, $CDCl_3$)

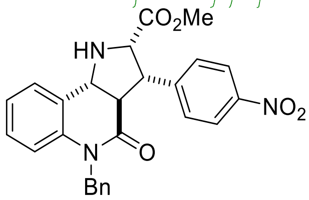

B8

^{1}H NMR (400 MHz, $CDCl_3$)

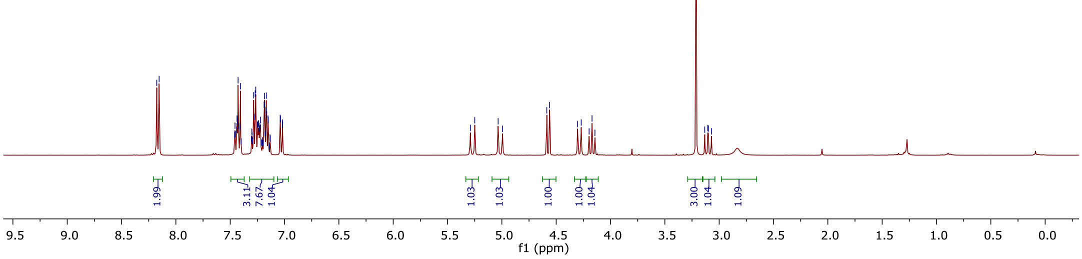

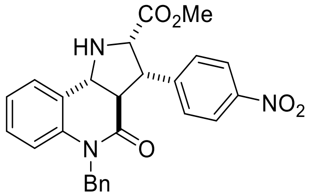

B8

^{13}C NMR (101 MHz, $CDCl_3$)

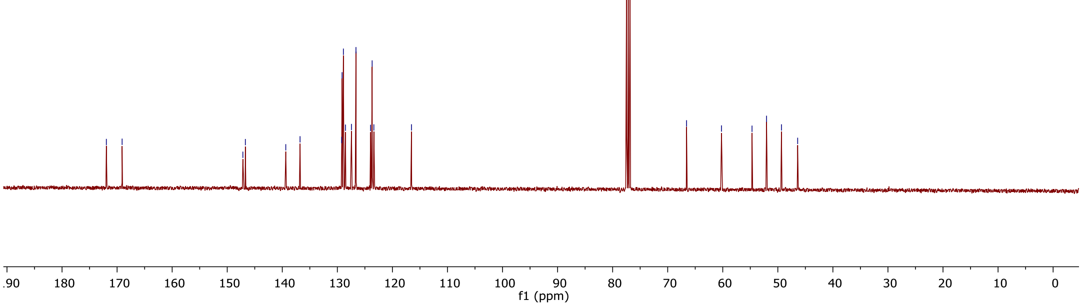

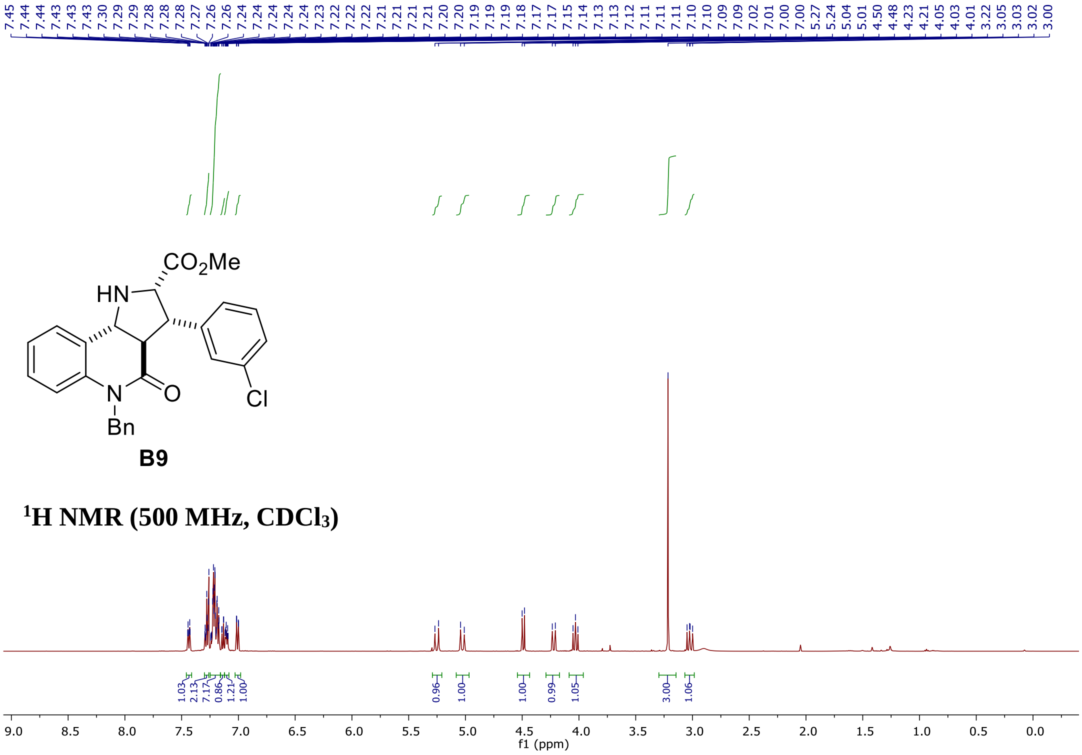
7.45 7.44 7.44 7.43 7.43 7.30 7.29 7.29 7.28 7.28 7.27 7.26 7.26 7.24 7.24 7.24 7.24 7.24 7.23 7.22 7.22 7.22 7.21 7.21 7.21 7.21 7.20 7.20 7.19 7.19 7.18 7.17 7.17 7.15 7.14 7.13 7.13 7.12 7.11 7.11 7.11 7.10 7.10 7.09 7.09 7.02 7.01 7.00 7.00 5.27 5.24 5.04 5.01 4.50 4.48 4.23 4.21 4.05 4.03 4.01 3.22 3.05 3.03 3.02 3.00
CO2Me
HN
N
Bn
O
Cl
B9
1H NMR (500 MHz, CDCl3)
1.03 2.13 7.17 0.86 1.21 1.00 0.96 1.00 1.00 0.99 1.05 3.00 1.06
f1 (ppm)

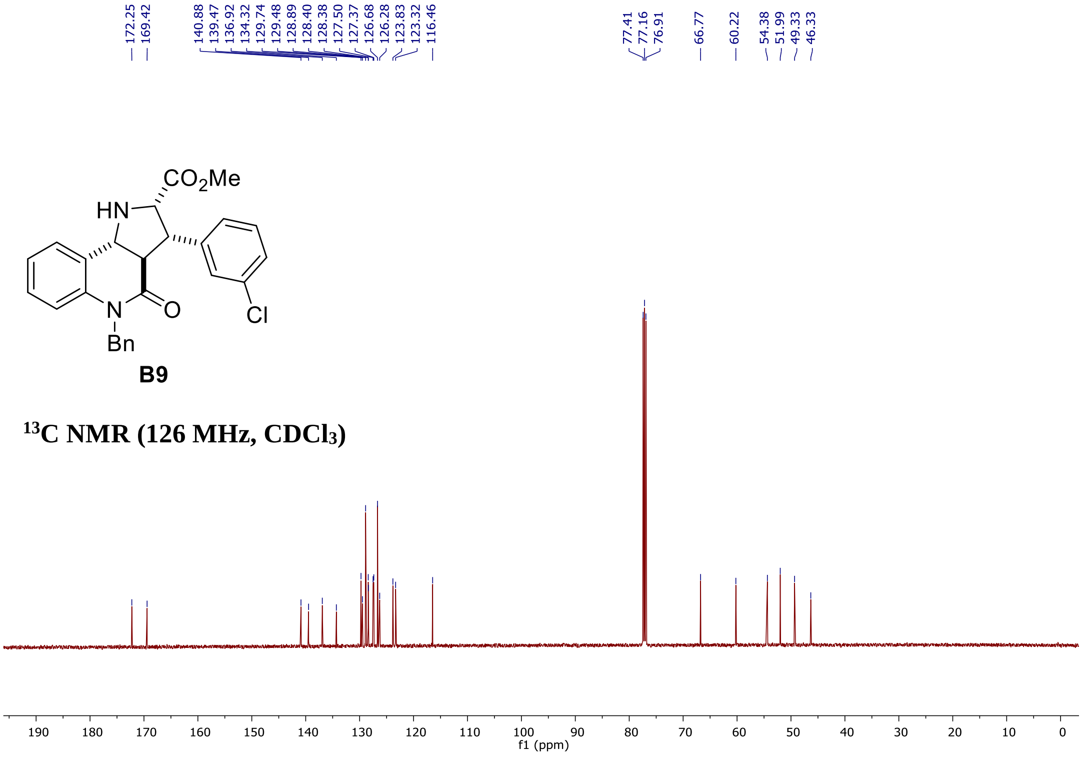
172.25 169.42 140.88 139.47 136.92 134.32 129.74 129.48 128.89 128.40 128.38 127.50 127.37 126.68 126.28 123.83 123.32 116.46 77.41 77.16 76.91 66.77 60.22 54.38 51.99 49.33 46.33
CO2Me
HN
N
Bn
O
Cl
B9
13C NMR (126 MHz, CDCl3)
f1 (ppm)

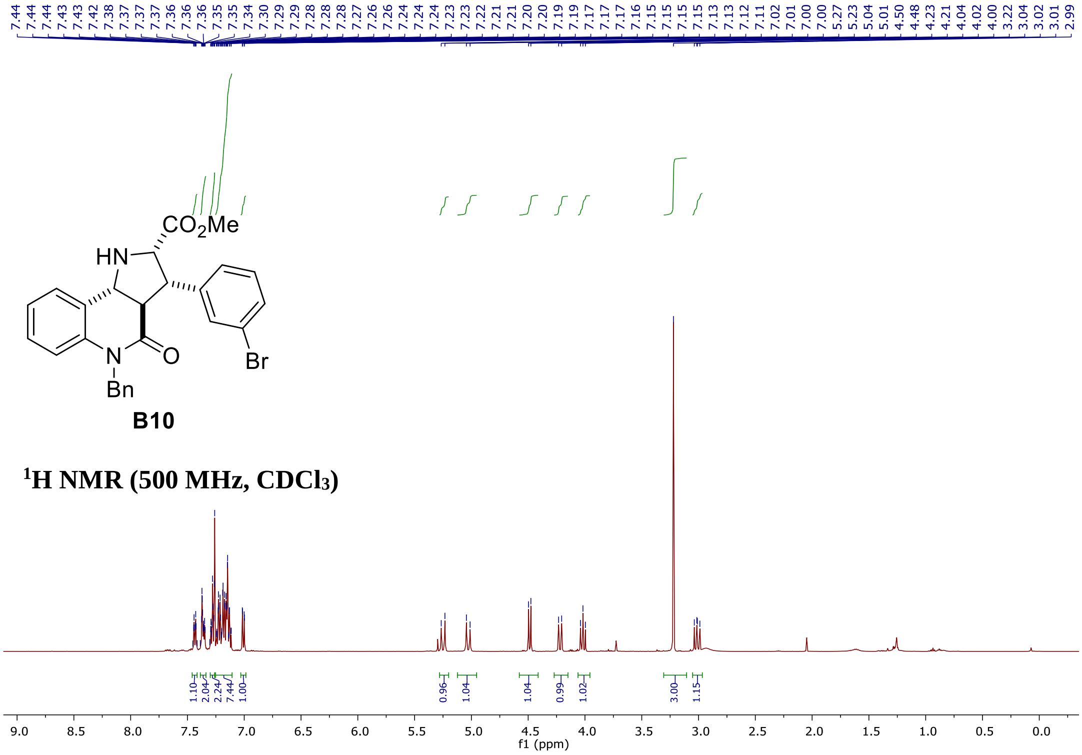

B10

^{1}H NMR (500 MHz, CDCl₃)

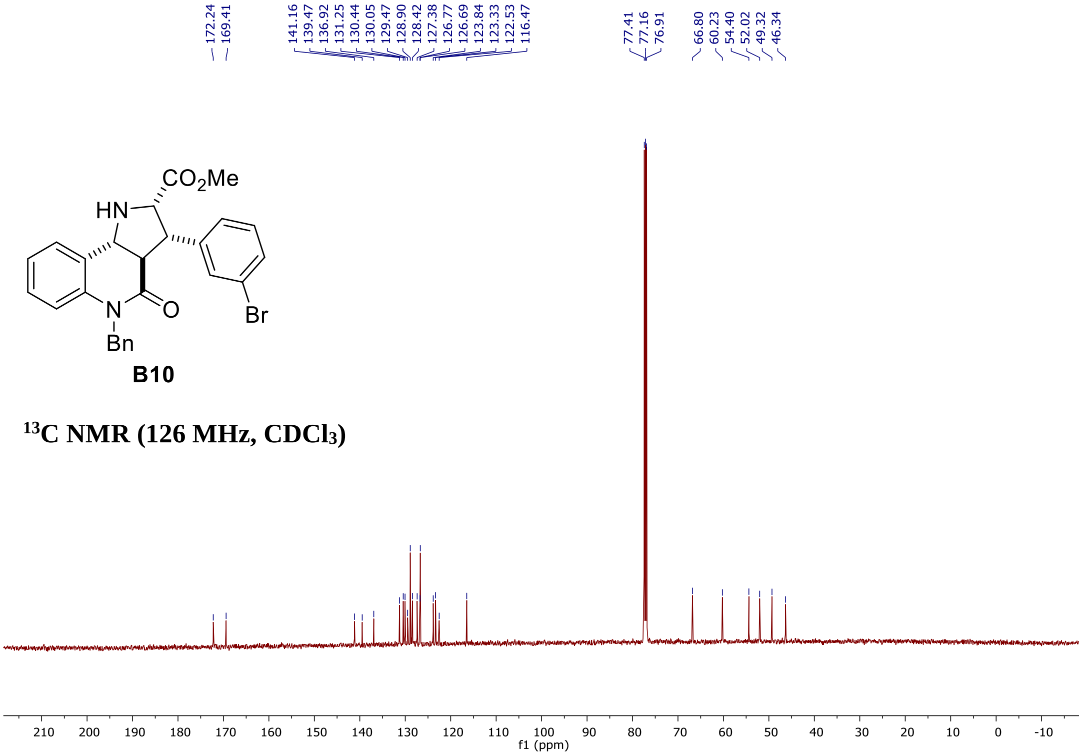

B10

^{13}C NMR (126 MHz, CDCl₃)

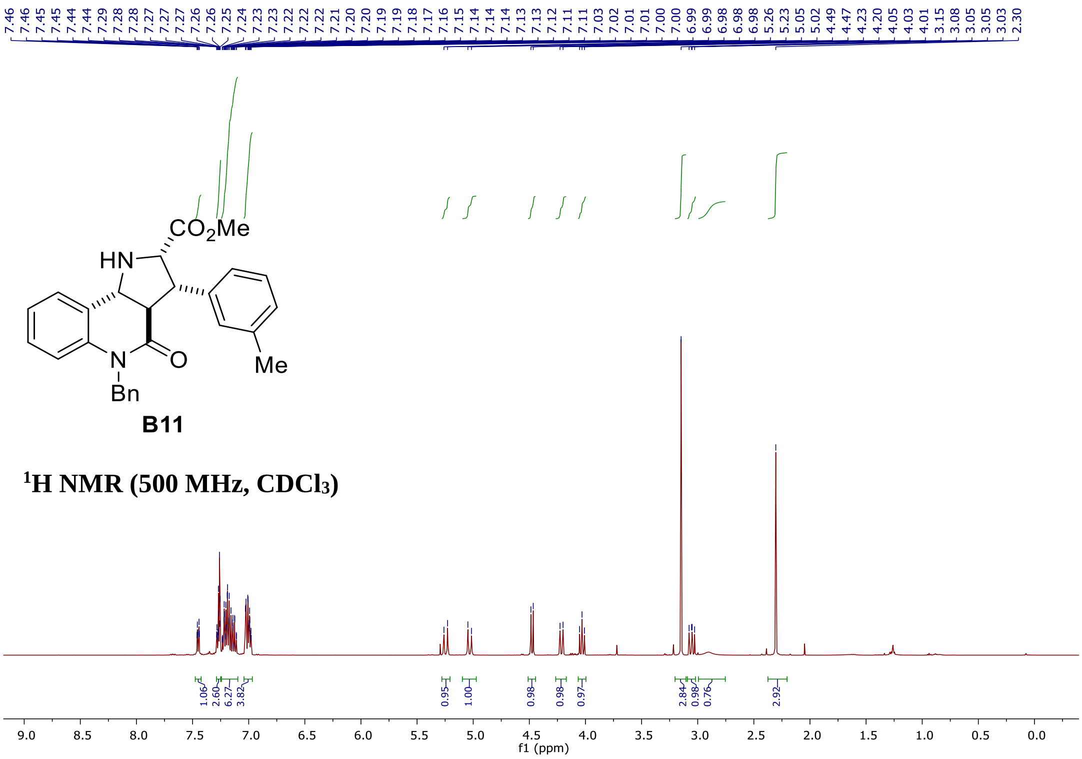

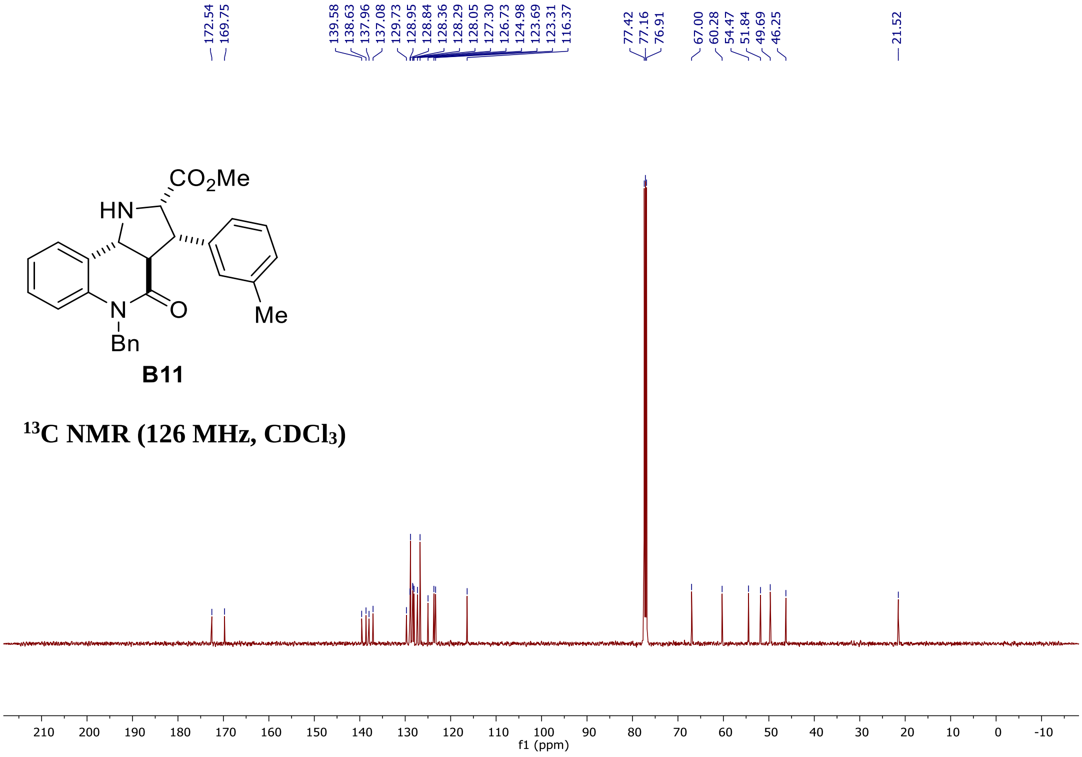

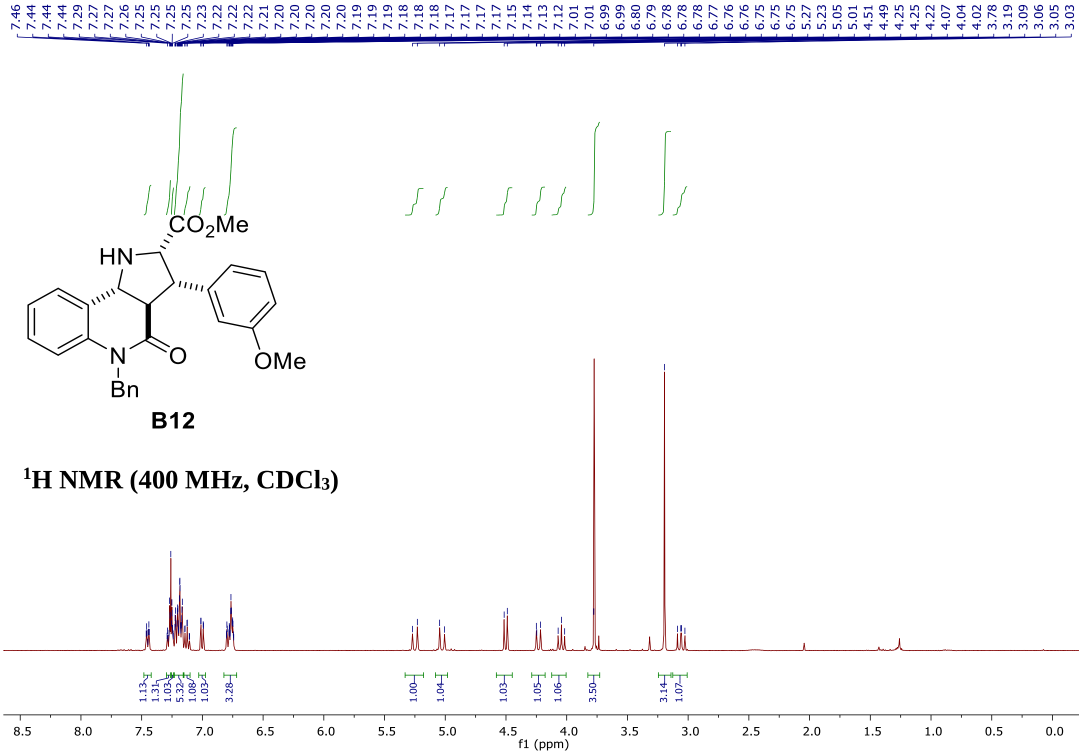

B12

^{1}H NMR (400 MHz, $CDCl_3$)

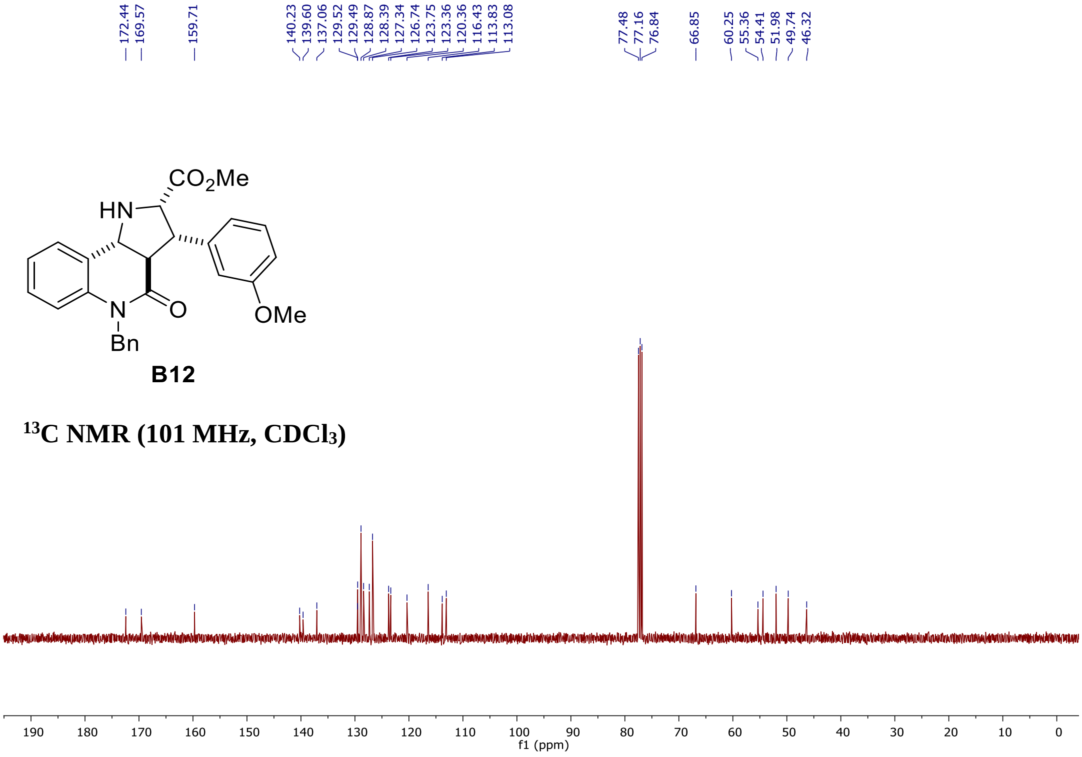

B12

^{13}C NMR (101 MHz, $CDCl_3$)

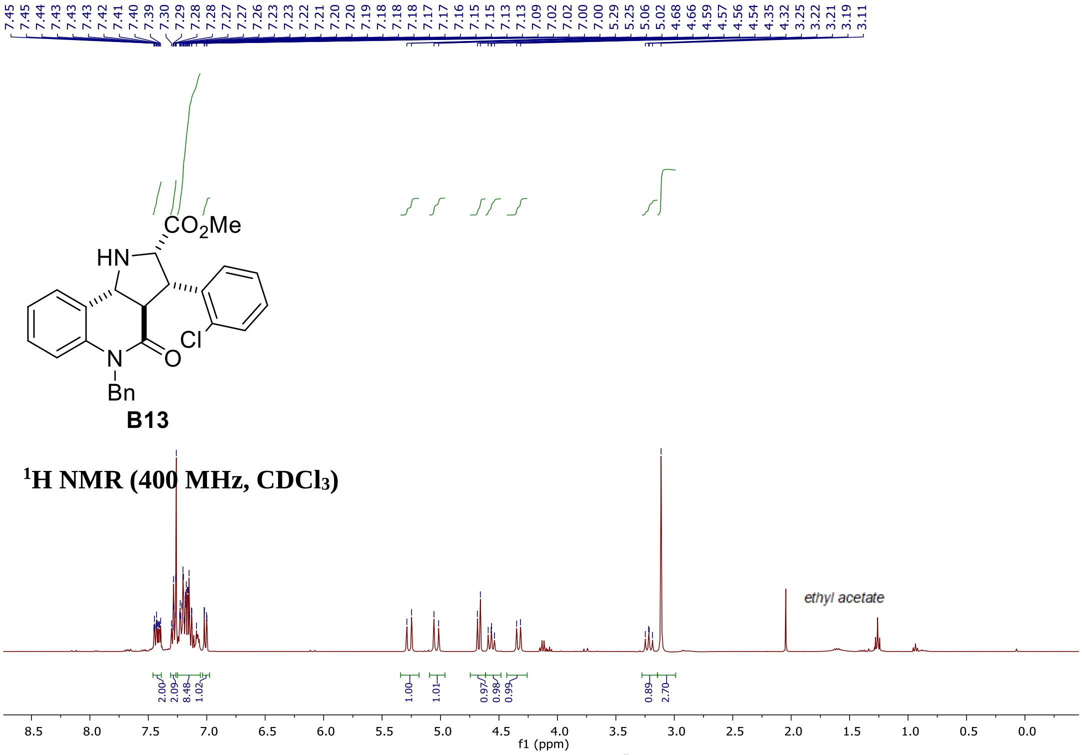

7.45 7.45 7.44 7.43 7.43 7.43 7.42 7.41 7.40 7.39 7.30 7.29 7.28 7.28 7.27 7.27 7.26 7.23 7.23 7.22 7.21 7.20 7.20 7.19 7.18 7.18 7.18 7.17 7.17 7.16 7.15 7.15 7.13 7.13 7.09 7.02 7.02 7.00 7.00 5.29 5.25 5.06 5.02 4.68 4.66 4.59 4.57 4.56 4.54 4.35 4.32 3.25 3.22 3.21 3.19 3.11
CO2Me
HN
Cl
N
O
Bn
B13
1H NMR (400 MHz, CDCl3)
ethyl acetate
2.00 2.09 8.48 1.02 1.00 1.01 0.97 0.98 0.99 0.89 2.70
f1 (ppm)

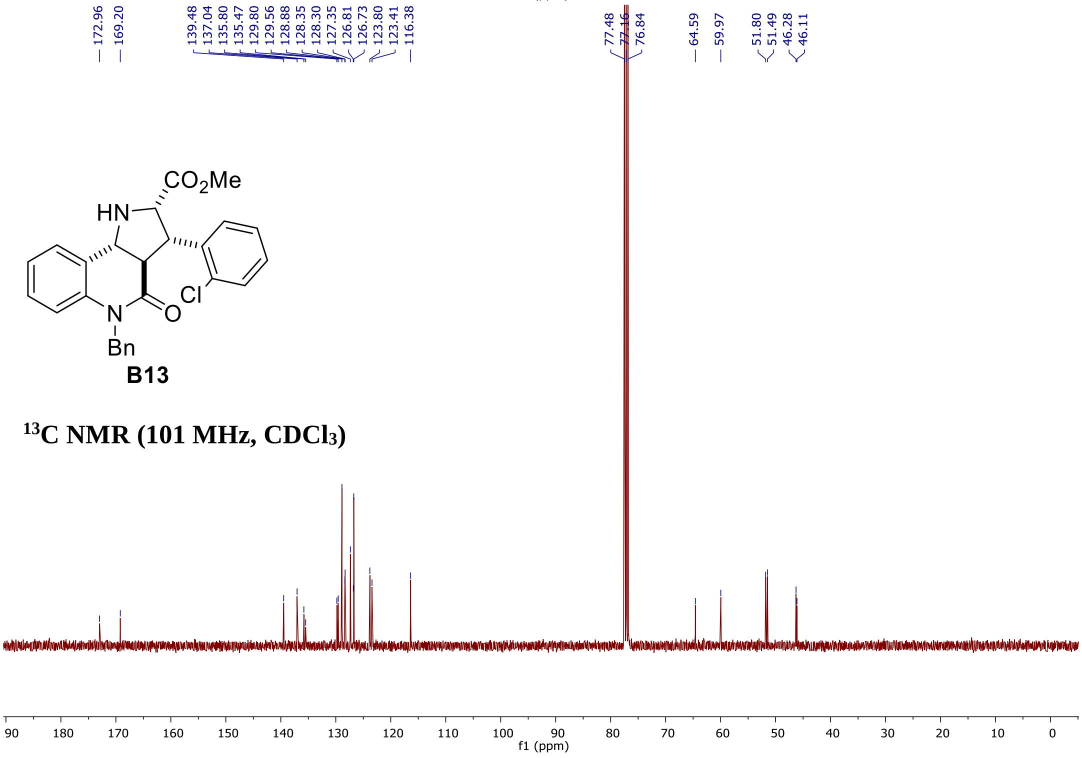

172.96 169.20 139.48 137.04 135.80 135.47 129.80 129.56 128.88 128.35 128.30 127.35 126.81 126.73 123.80 123.41 116.38 77.48 77.16 76.84 64.59 59.97 51.80 51.49 46.28 46.11
CO2Me
HN
Cl
N
O
Bn
B13
13C NMR (101 MHz, CDCl3)
f1 (ppm)

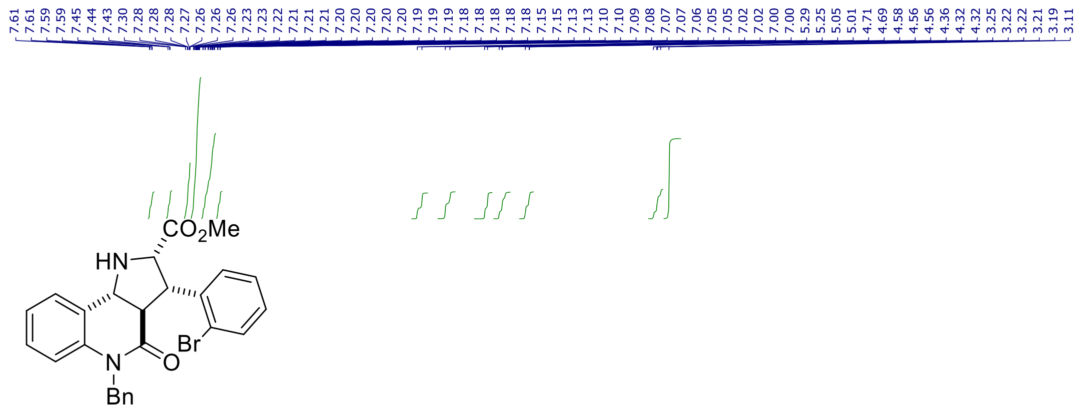

B14

^{1}H NMR (400 MHz, $CDCl_3$)

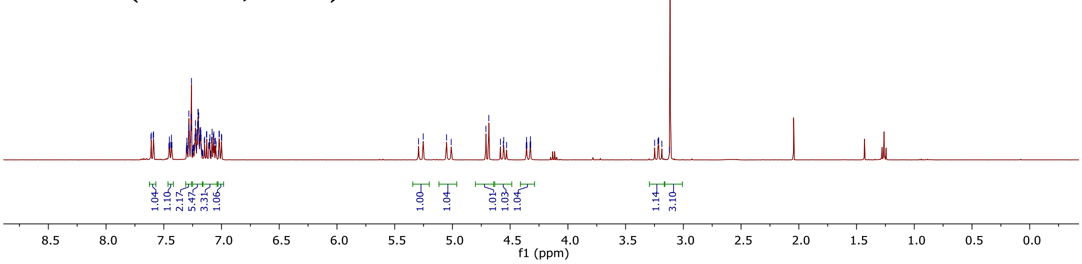

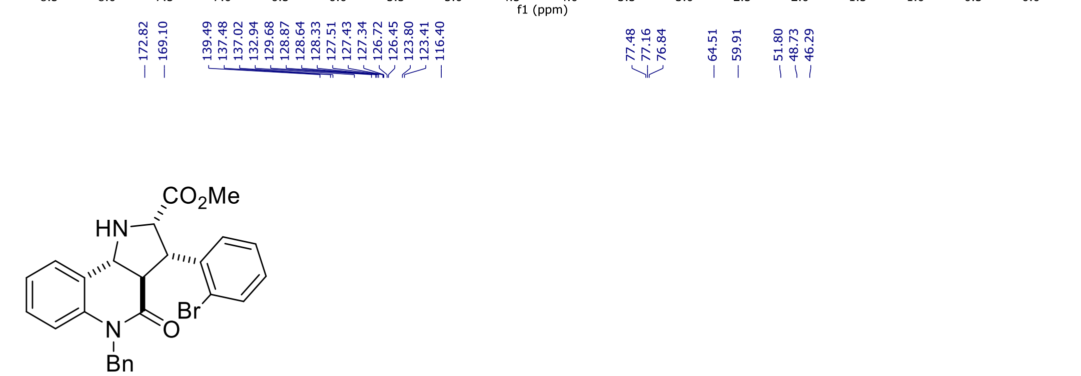

B14

^{13}C NMR (101 MHz, $CDCl_3$)

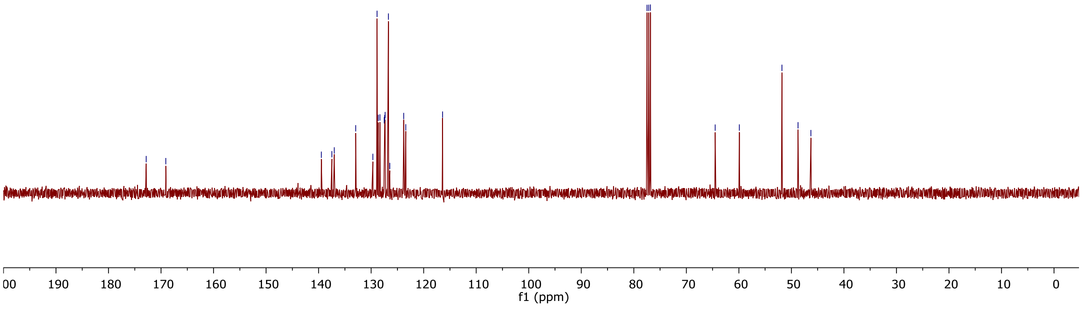

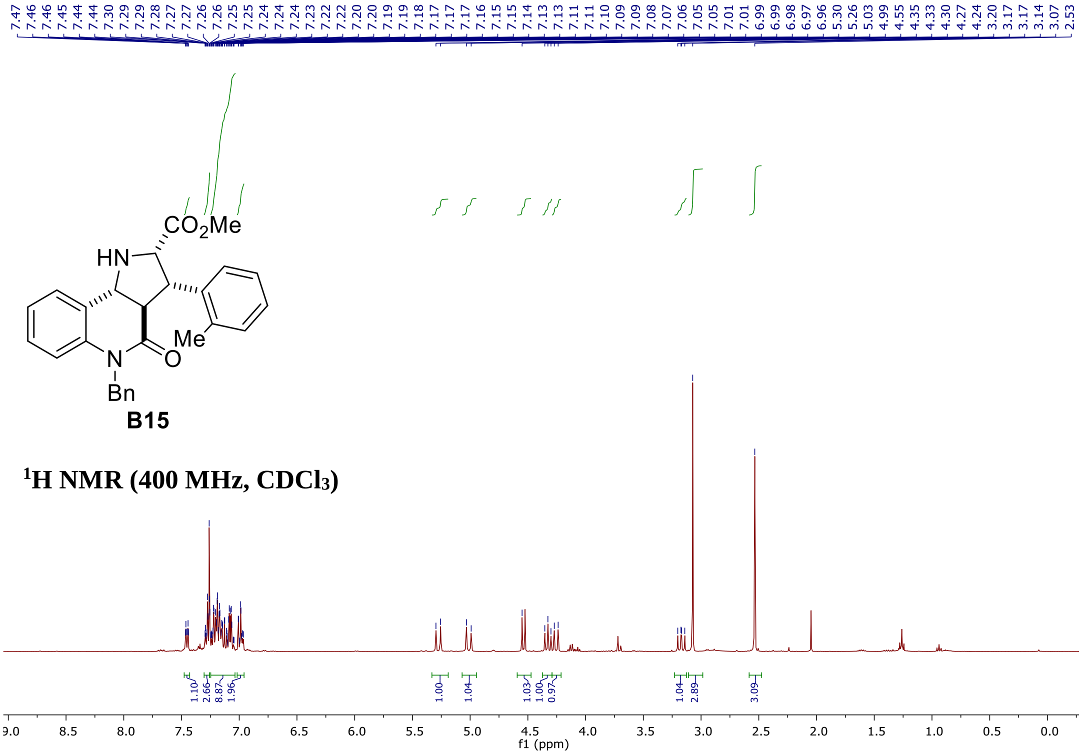
CO2Me
HN
Me
N
O
Bn
B15
1H NMR (400 MHz, CDCl3)
f1 (ppm)

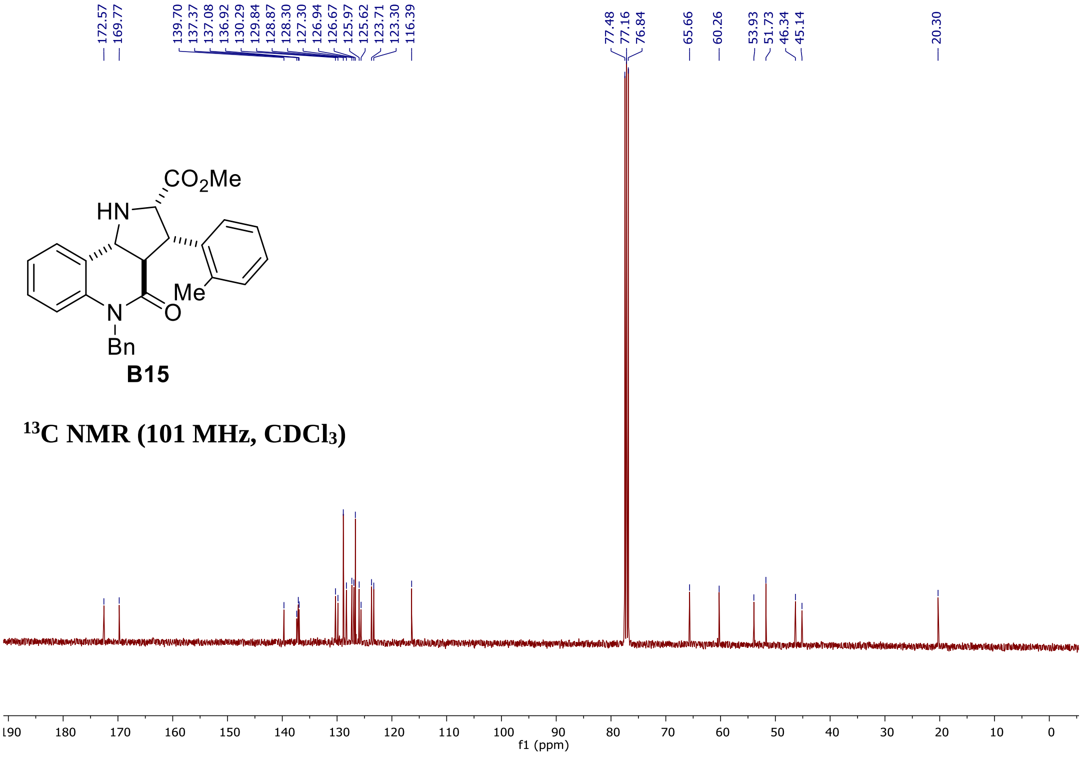
CO2Me
HN
Me
N
O
Bn
B15
13C NMR (101 MHz, CDCl3)
f1 (ppm)

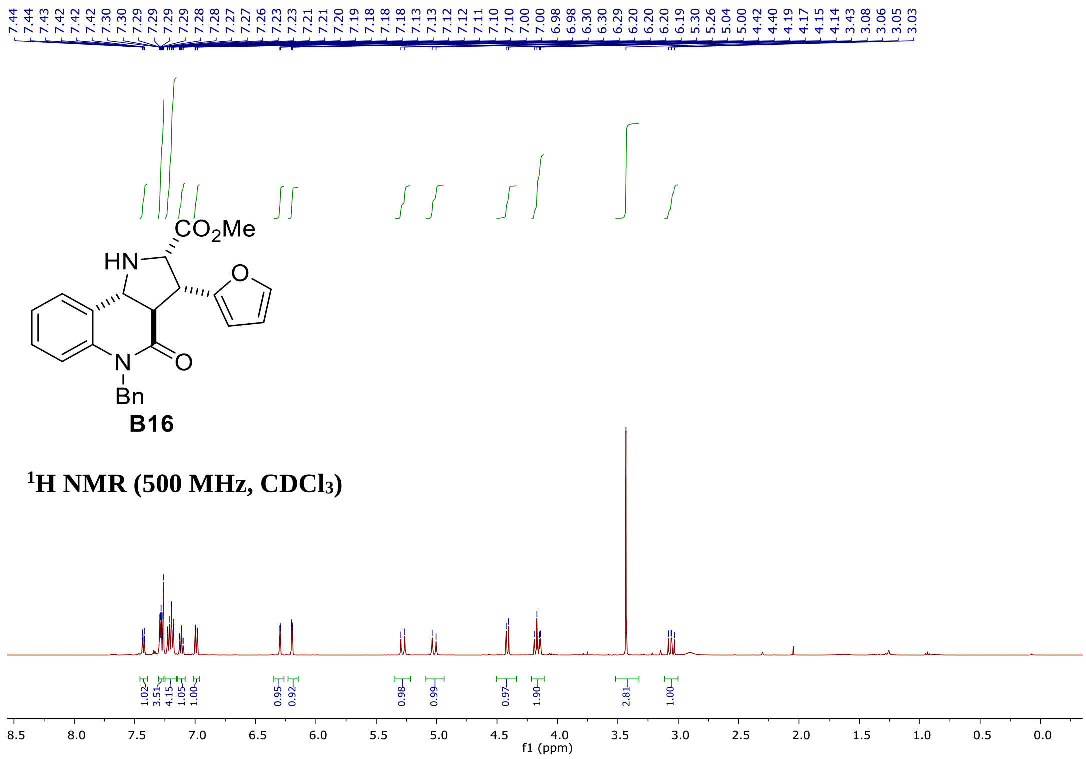

B16

^{1}H NMR (500 MHz, CDCl3)

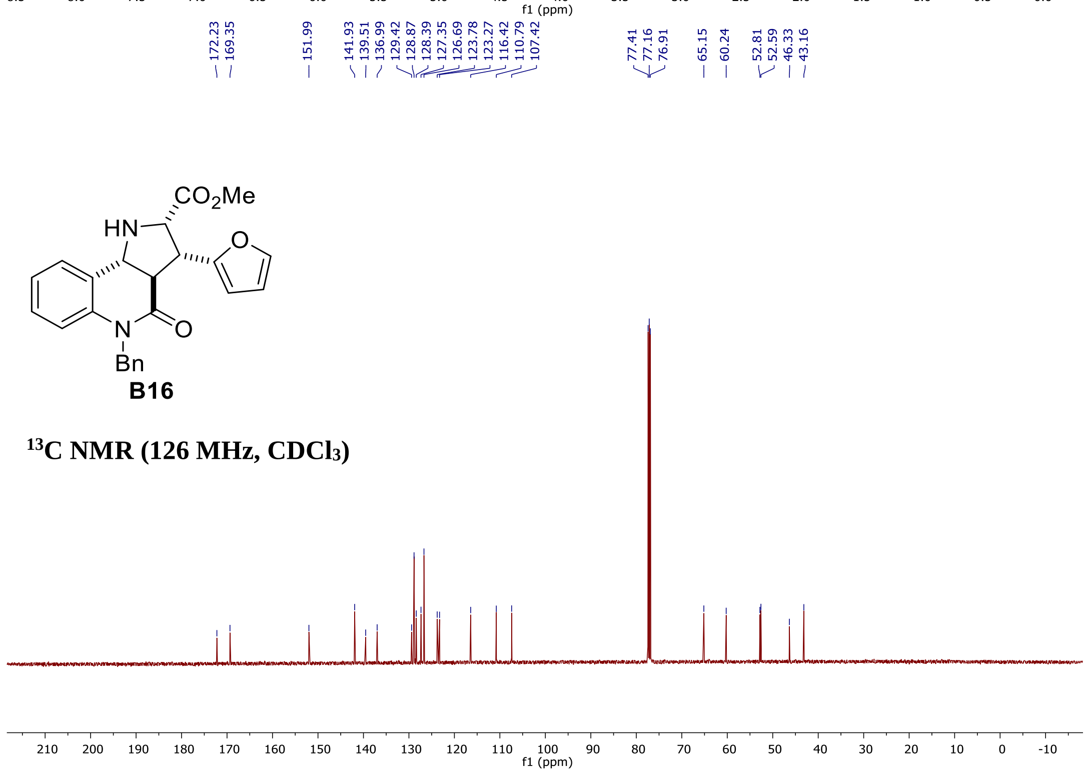

B16

^{13}C NMR (126 MHz, CDCl3)

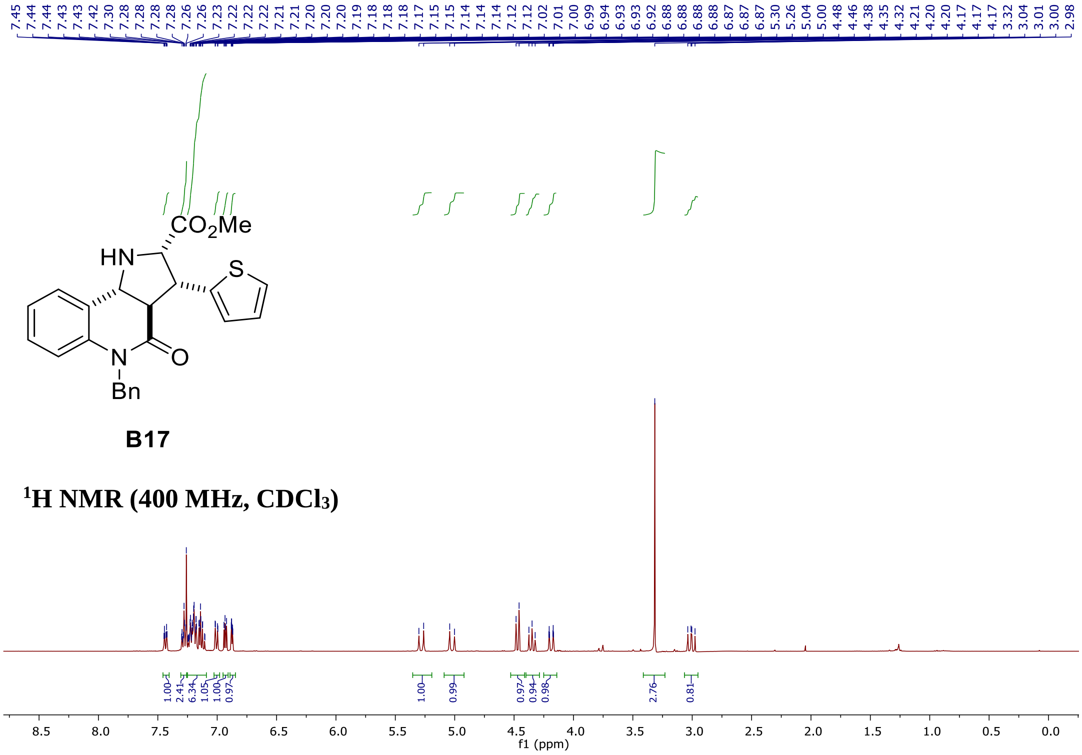
7.45 7.44 7.44 7.43 7.43 7.42 7.30 7.28 7.28 7.28 7.28 7.26 7.26 7.23 7.22 7.22 7.22 7.21 7.21 7.20 7.20 7.20 7.19 7.18 7.18 7.18 7.17 7.15 7.15 7.14 7.14 7.14 7.12 7.12 7.02 7.01 7.00 6.99 6.94 6.93 6.93 6.92 6.88 6.88 6.88 6.88 6.87 6.87 6.87 5.30 5.26 5.04 5.00 4.48 4.46 4.38 4.35 4.32 4.21 4.20 4.20 4.17 4.17 4.17 3.32 3.04 3.01 3.00 2.98
CO2Me
HN
S
N
O
Bn
B17
1H NMR (400 MHz, CDCl3)
1.00 2.41 6.34 1.05 1.00 0.97 1.00 0.99 0.97 0.94 0.98 2.76 0.81
8.5 8.0 7.5 7.0 6.5 6.0 5.5 5.0 4.5 4.0 3.5 3.0 2.5 2.0 1.5 1.0 0.5 0.0
f1 (ppm)

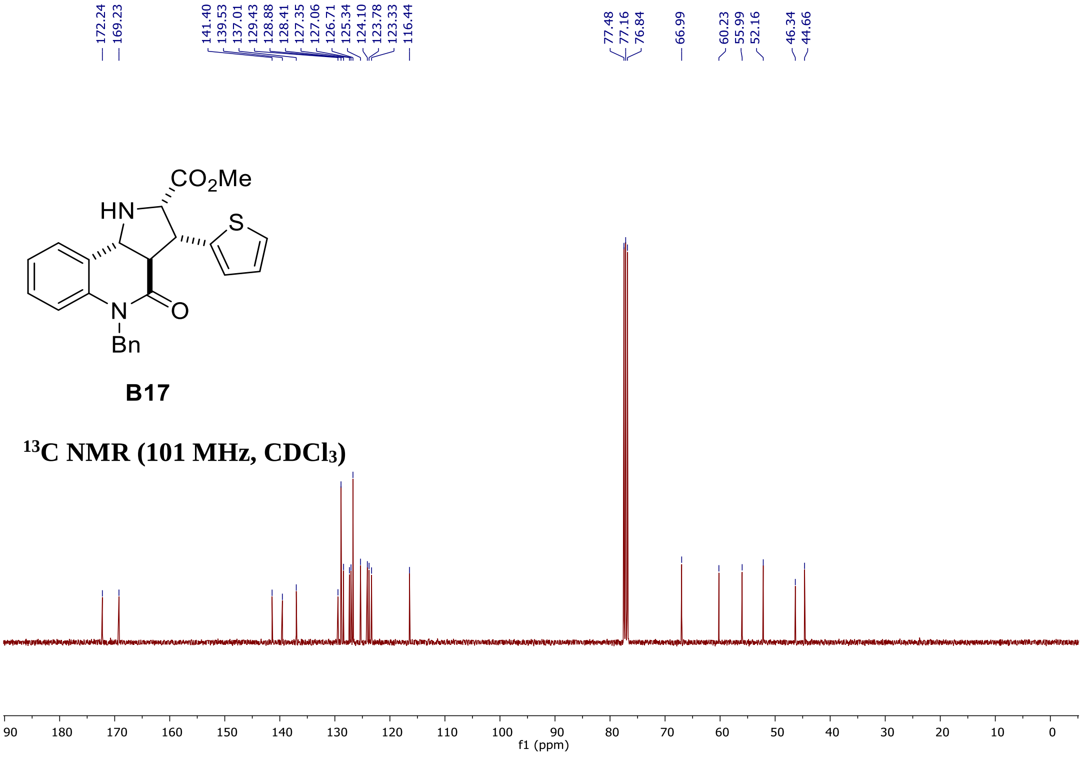
172.24 169.23 141.40 139.53 137.01 129.43 128.88 128.41 127.35 127.06 126.71 125.34 124.10 123.78 123.33 116.44 77.48 77.16 76.84 66.99 60.23 55.99 52.16 46.34 44.66
CO2Me
HN
S
N
O
Bn
B17
13C NMR (101 MHz, CDCl3)
90 180 170 160 150 140 130 120 110 100 90 80 70 60 50 40 30 20 10 0
f1 (ppm)

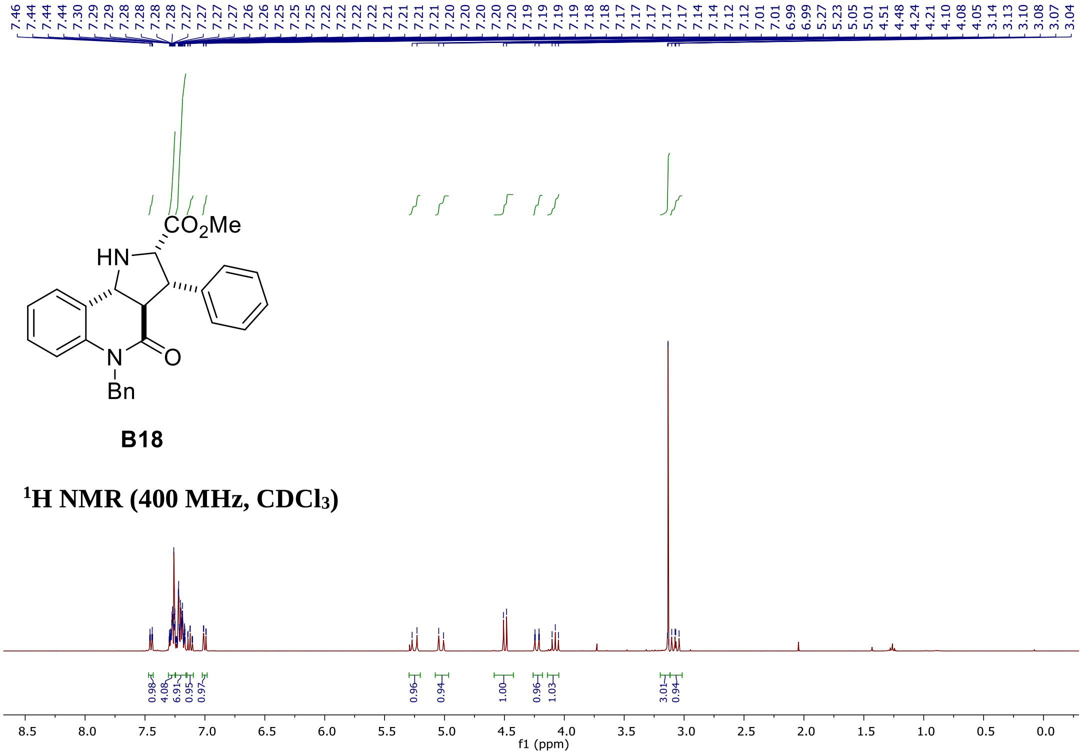

B18

^{1}H NMR (400 MHz, $CDCl_3$)

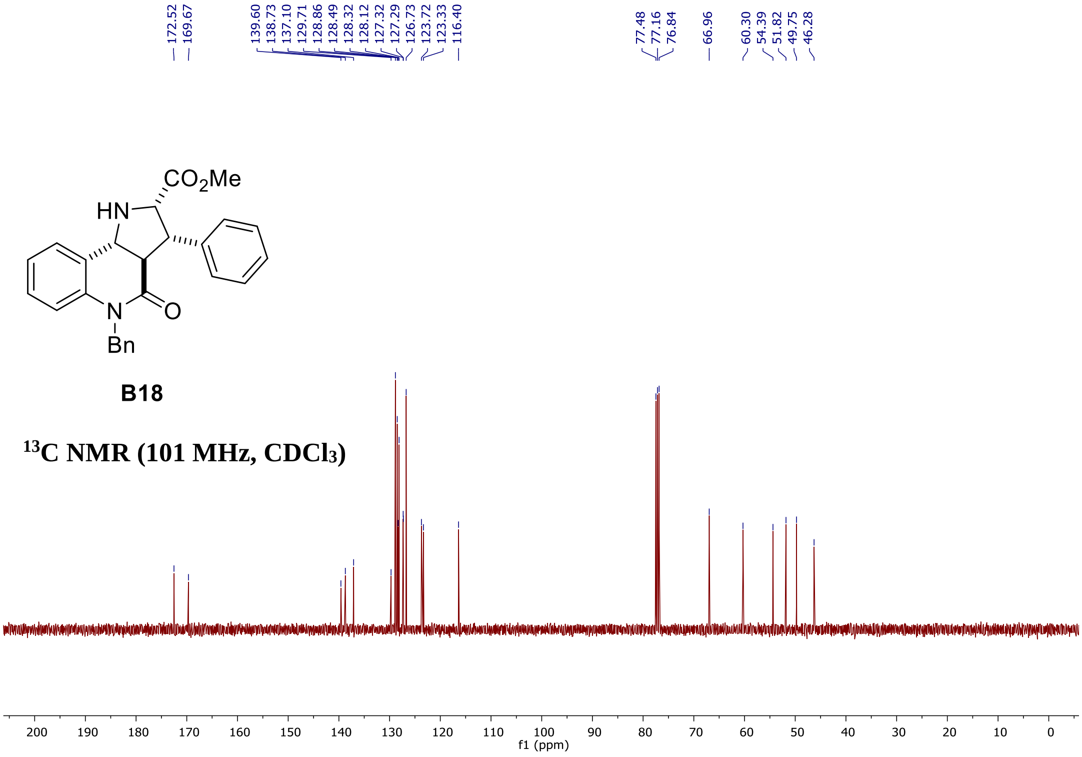

B18

^{13}C NMR (101 MHz, $CDCl_3$)

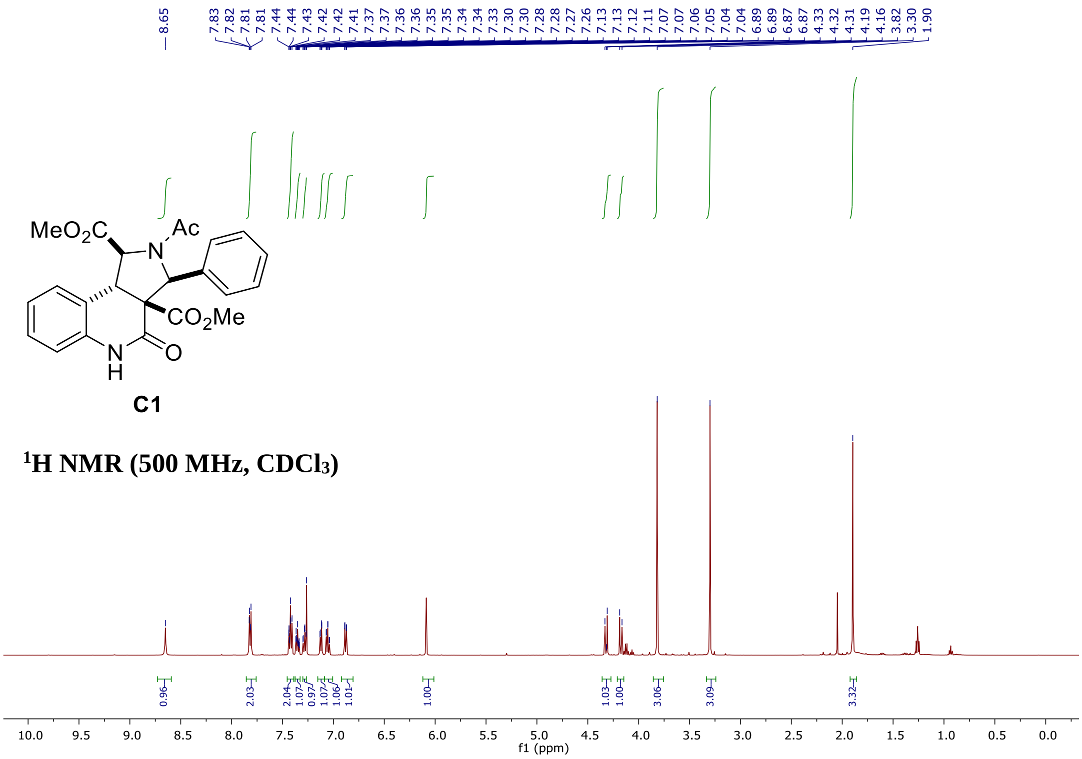

C1

^{1}H NMR (500 MHz, CDCl3)

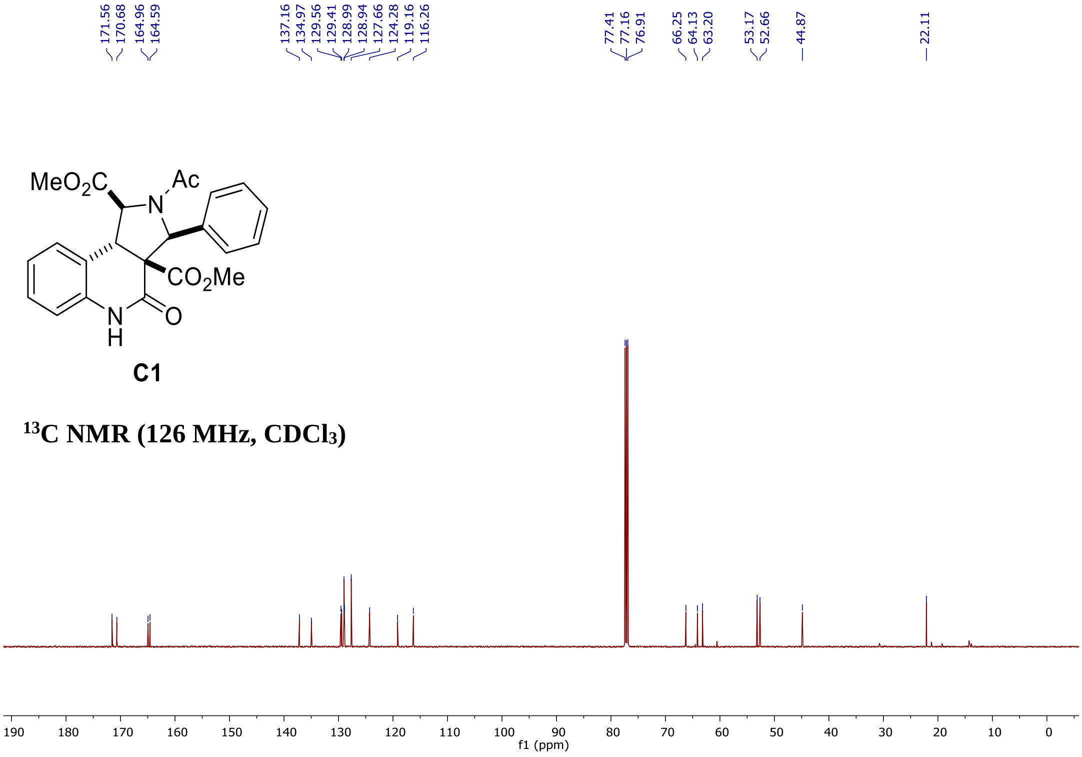

C1

^{13}C NMR (126 MHz, CDCl3)

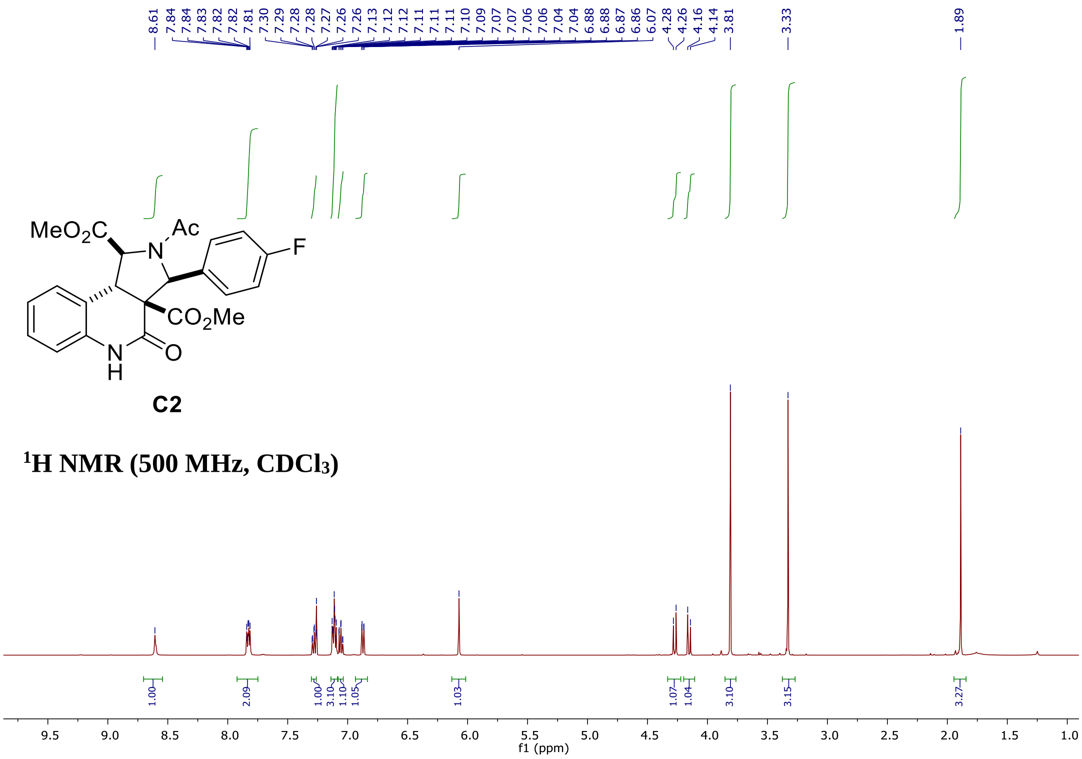

C2

^{1}H NMR (500 MHz, $CDCl_3$)

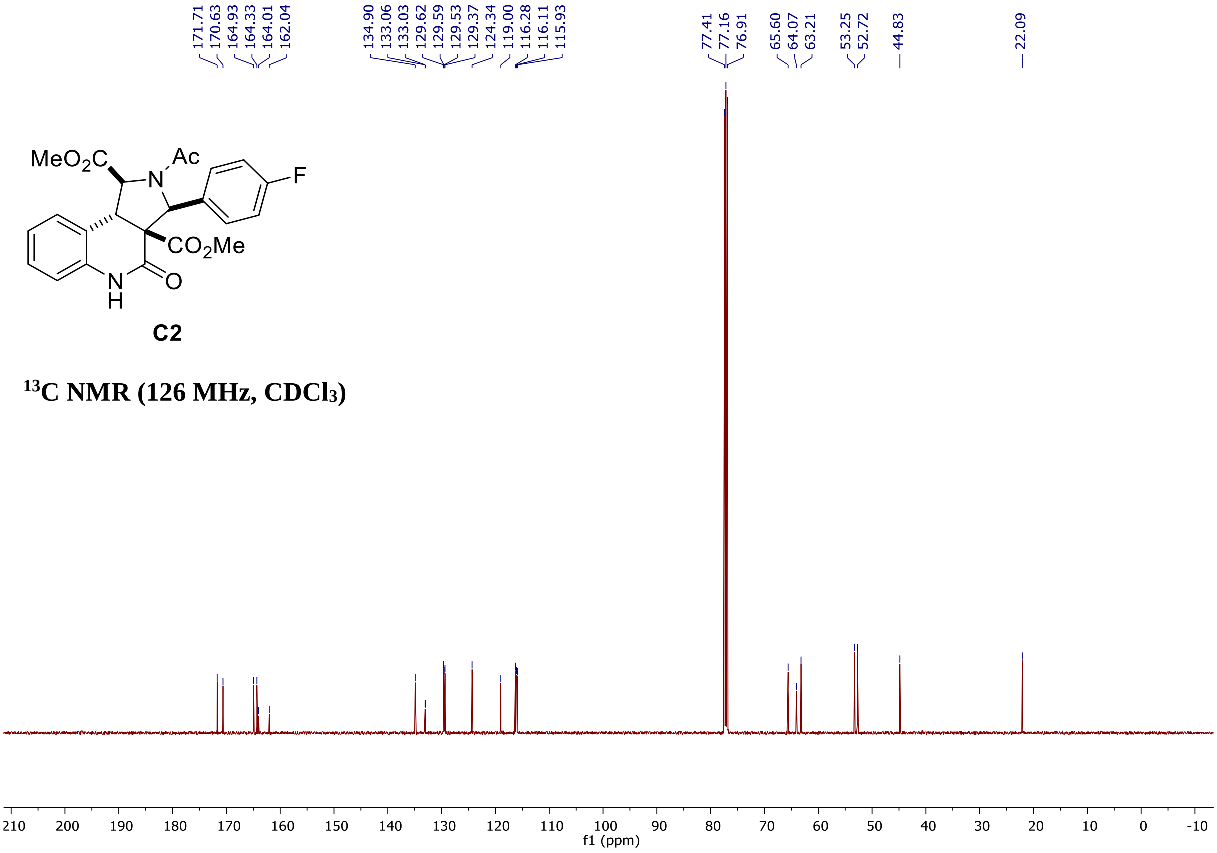

C2

^{13}C NMR (126 MHz, $CDCl_3$)

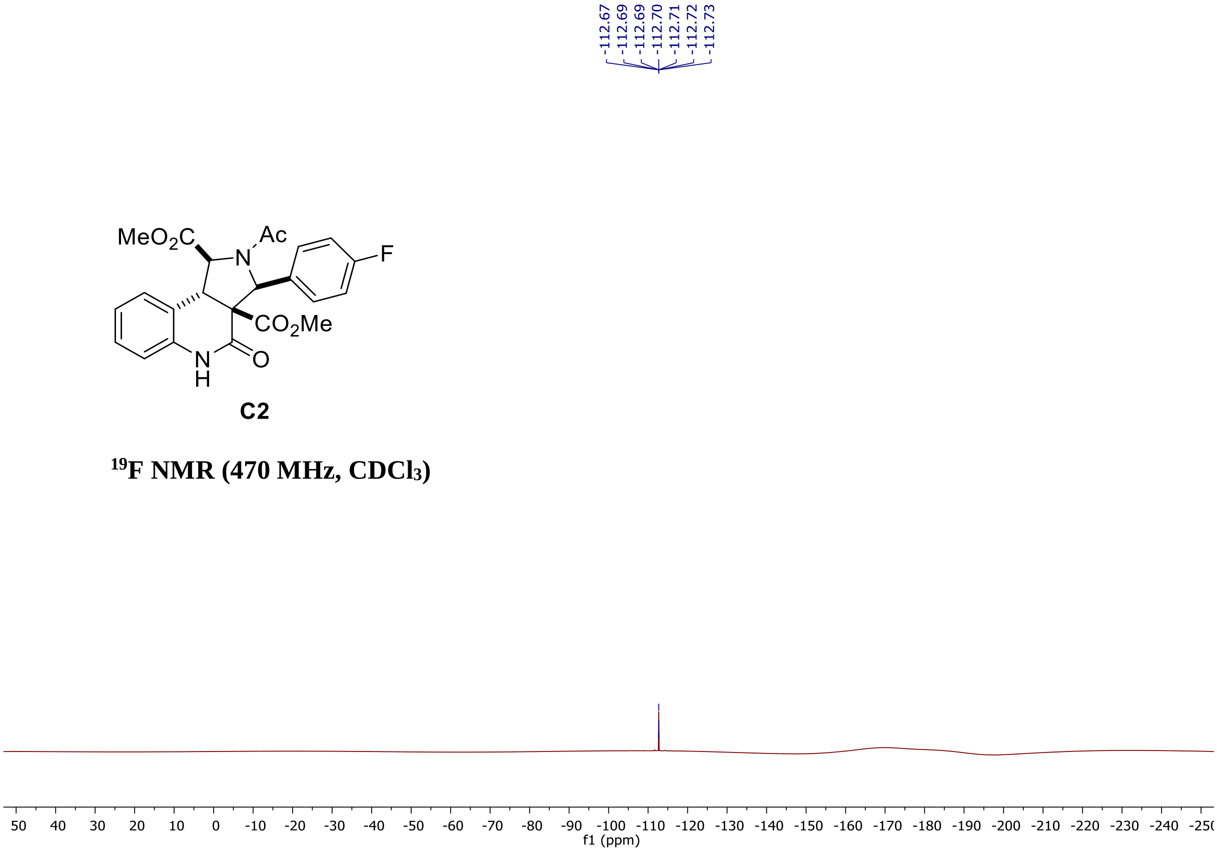

C2

^{19}F NMR (470 MHz, CDCl$_3$)

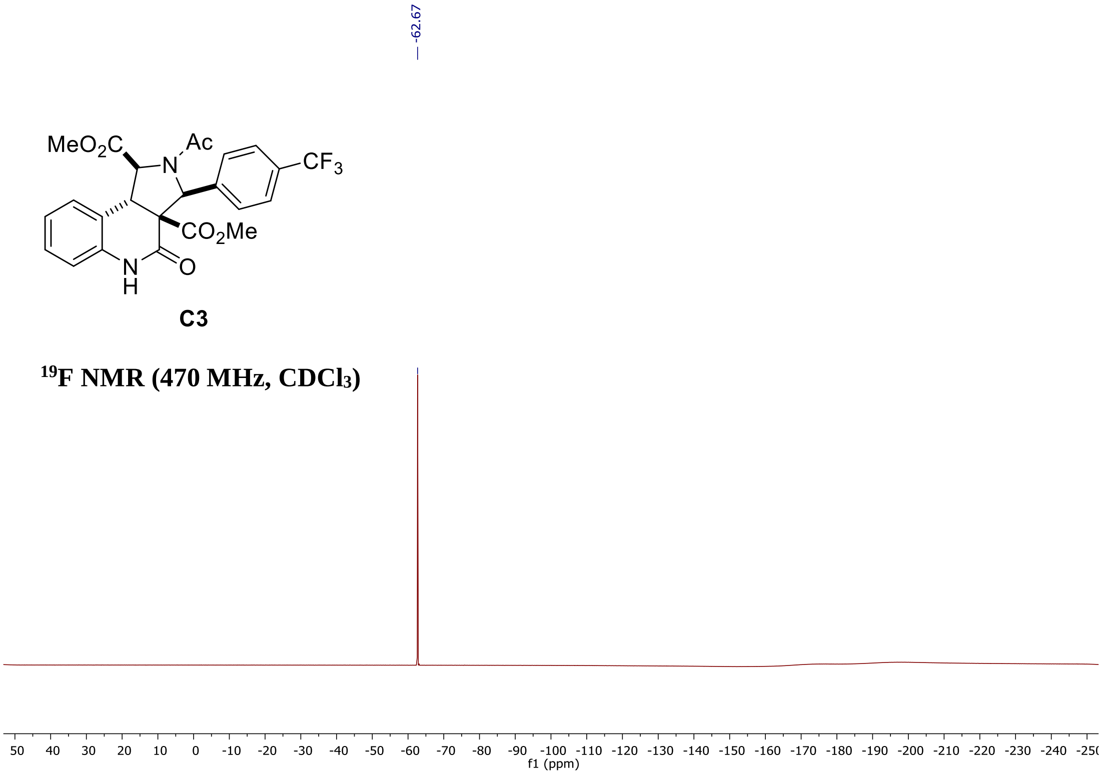

C3

^{19}F NMR (470 MHz, CDCl$_3$)

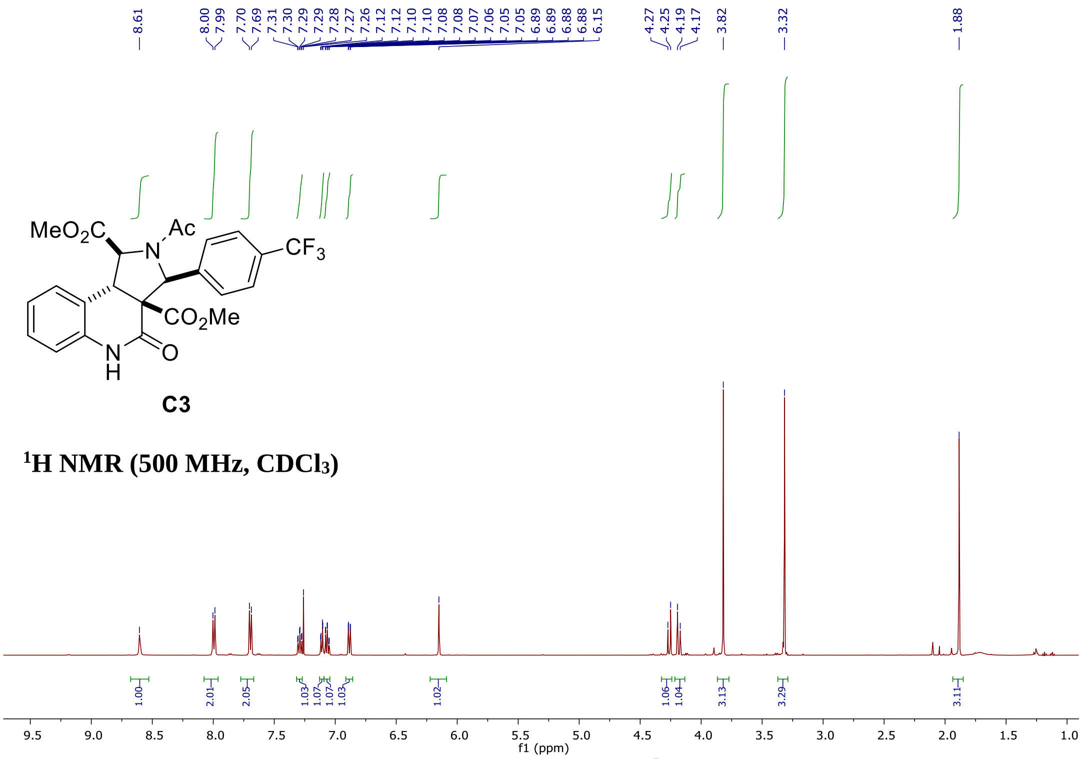
8.61
8.00
7.99
7.70
7.69
7.31
7.30
7.29
7.29
7.28
7.27
7.26
7.12
7.12
7.10
7.10
7.08
7.08
7.07
7.06
7.05
7.05
6.89
6.89
6.88
6.88
6.15
4.27
4.25
4.19
4.17
3.82
3.32
1.88
MeO2C
Ac
N
CF3
CO2Me
N
H
O
C3
1H NMR (500 MHz, CDCl3)
1.00
2.01
2.05
1.03
1.07
1.07
1.03
1.02
1.06
1.04
3.13
3.29
3.11
f1 (ppm)

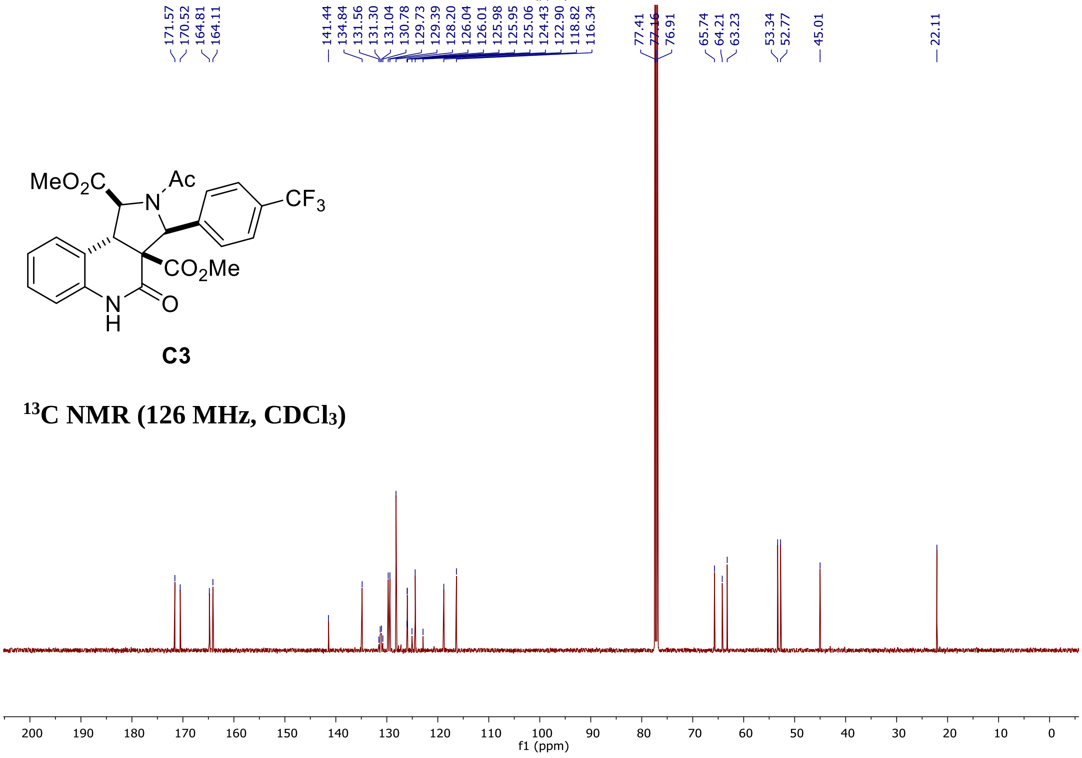
171.57
170.52
164.81
164.11
141.44
134.84
131.56
131.30
131.04
130.78
129.73
129.39
128.20
126.04
126.01
125.98
125.95
125.06
124.43
122.90
118.82
116.34
77.41
77.16
76.91
65.74
64.21
63.23
53.34
52.77
45.01
22.11
MeO2C
Ac
N
CF3
CO2Me
N
H
O
C3
13C NMR (126 MHz, CDCl3)
f1 (ppm)

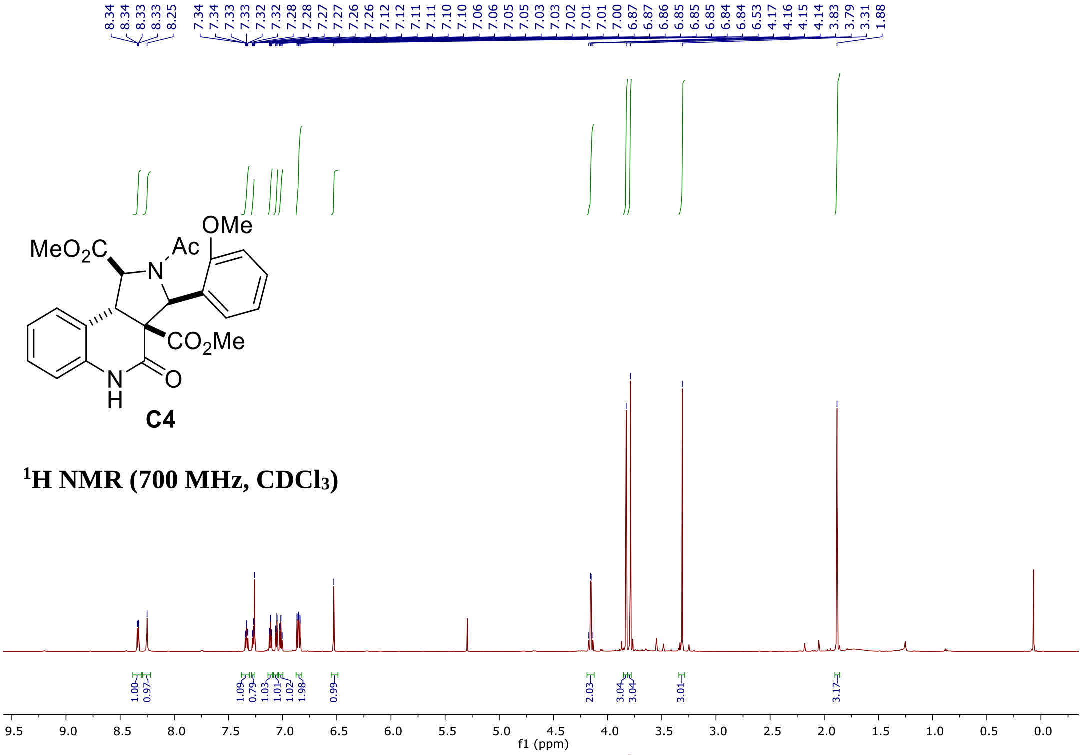
8.34 8.34 8.33 8.33 8.25 7.34 7.34 7.33 7.33 7.32 7.32 7.32 7.28 7.28 7.27 7.27 7.26 7.26 7.12 7.12 7.11 7.11 7.10 7.10 7.06 7.06 7.05 7.05 7.03 7.03 7.02 7.01 7.01 7.00 6.87 6.87 6.86 6.85 6.85 6.85 6.84 6.84 6.53 4.17 4.16 4.15 4.14 3.83 3.79 3.31 1.88
OMe
MeO2C
Ac
N
CO2Me
N
H
O
C4
1H NMR (700 MHz, CDCl3)
1.00 0.97 1.09 0.79 1.03 1.01 1.02 1.98 0.99 2.03 3.04 3.04 3.01 3.17
f1 (ppm)

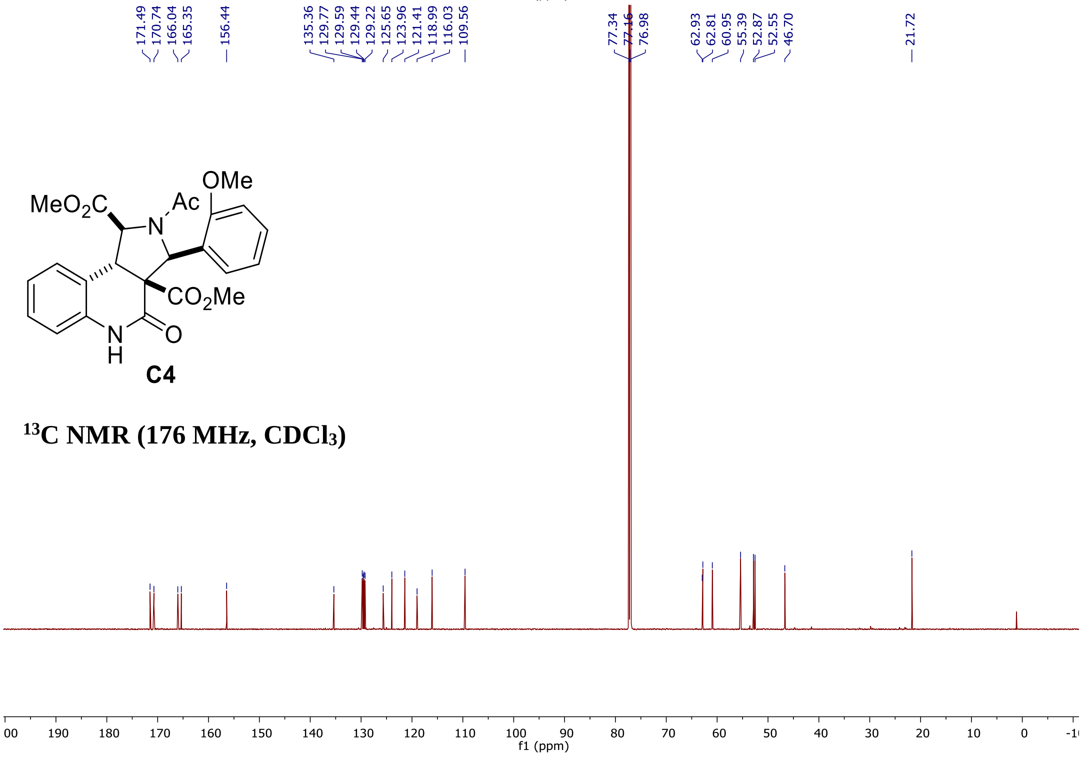
171.49 170.74 166.04 165.35 156.44 135.36 129.77 129.59 129.44 129.22 125.65 123.96 121.41 118.99 116.03 109.56 77.34 77.16 76.98 62.93 62.81 60.95 55.39 52.87 52.55 46.70 21.72
OMe
MeO2C
Ac
N
CO2Me
N
H
O
C4
13C NMR (176 MHz, CDCl3)
f1 (ppm)

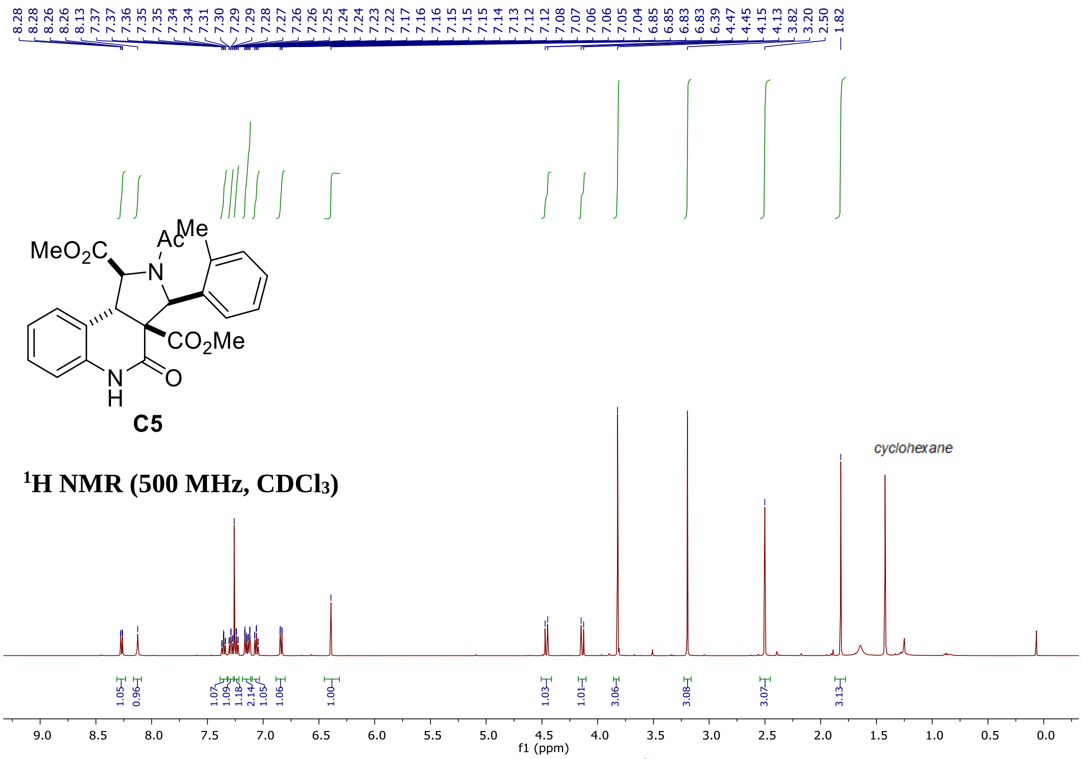
8.28
8.28
8.26
8.26
8.13
7.37
7.37
7.36
7.35
7.35
7.34
7.34
7.31
7.30
7.29
7.29
7.28
7.27
7.26
7.26
7.25
7.24
7.24
7.23
7.22
7.17
7.16
7.15
7.15
7.15
7.14
7.13
7.12
7.12
7.08
7.07
7.06
7.06
7.05
7.04
6.85
6.85
6.83
6.83
6.39
4.47
4.45
4.15
4.13
3.82
3.20
2.50
1.82
MeO2C
Ac
Me
N
CO2Me
N
H
O
C5
1H NMR (500 MHz, CDCl3)
cyclohexane
1.05
0.96
1.07
1.09
1.18
2.14
1.05
1.06
1.00
1.03
1.01
3.06
3.08
3.07
3.13
9.0
8.5
8.0
7.5
7.0
6.5
6.0
5.5
5.0
4.5
4.0
3.5
3.0
2.5
2.0
1.5
1.0
0.5
0.0
f1 (ppm)

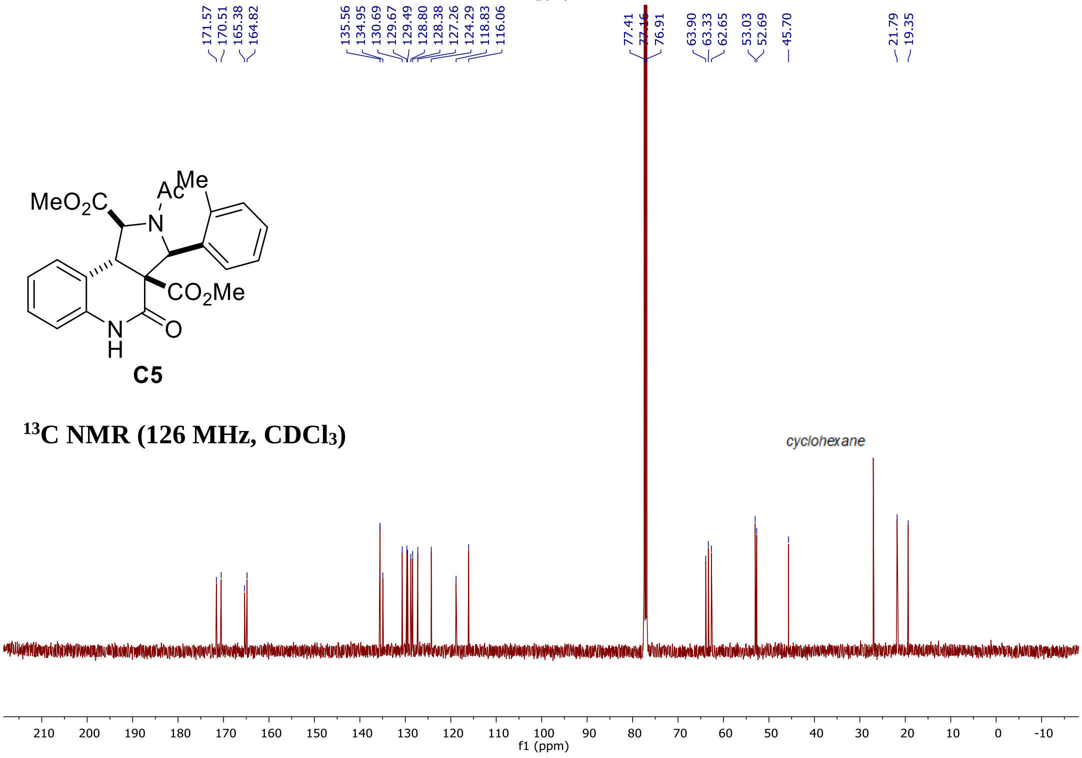
171.57
170.51
165.38
164.82
135.56
134.95
130.69
129.67
129.49
128.80
128.38
127.26
124.29
118.83
116.06
77.41
77.16
76.91
63.90
63.33
62.65
53.03
52.69
45.70
21.79
19.35
MeO2C
Ac
Me
N
CO2Me
N
H
O
C5
13C NMR (126 MHz, CDCl3)
cyclohexane
210
200
190
180
170
160
150
140
130
120
110
100
90
80
70
60
50
40
30
20
10
0
-10
f1 (ppm)

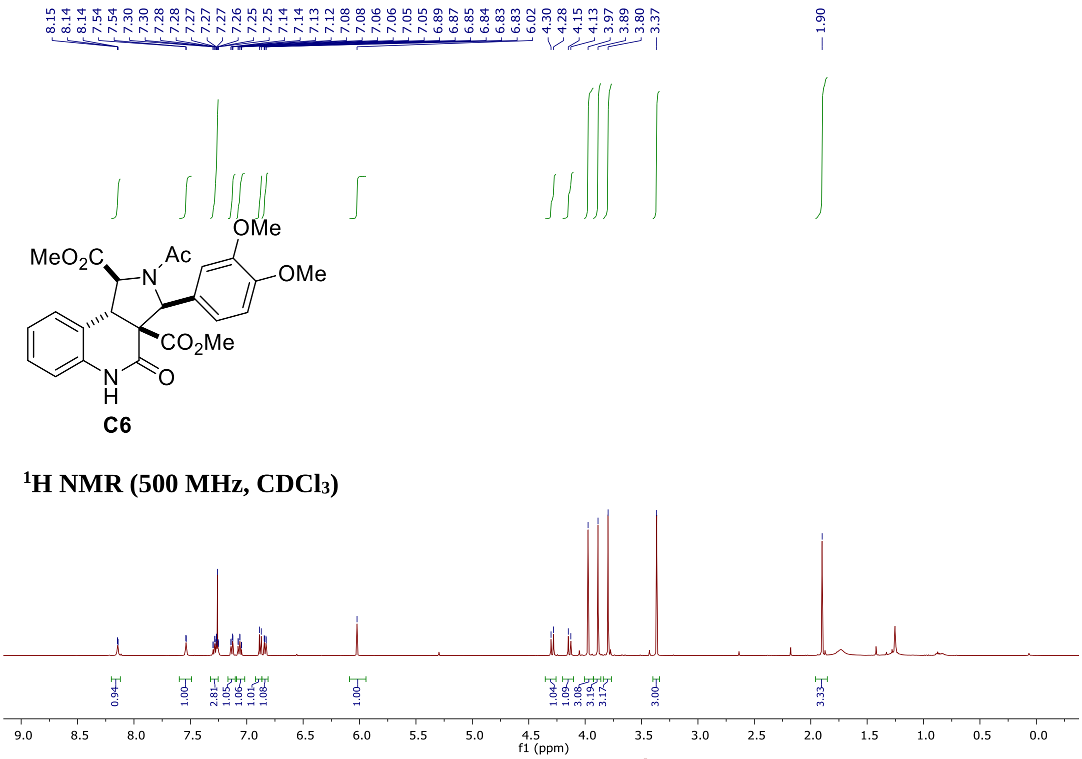

^{1}H NMR (500 MHz, CDCl$_3$)

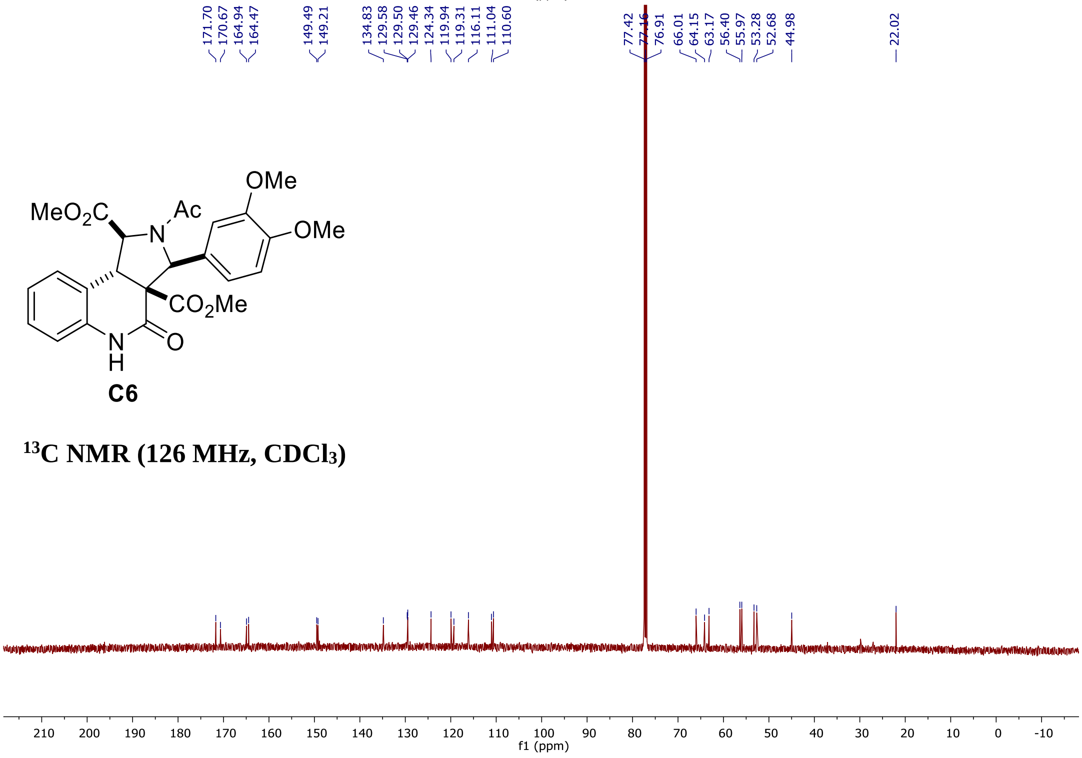

^{13}C NMR (126 MHz, CDCl$_3$)

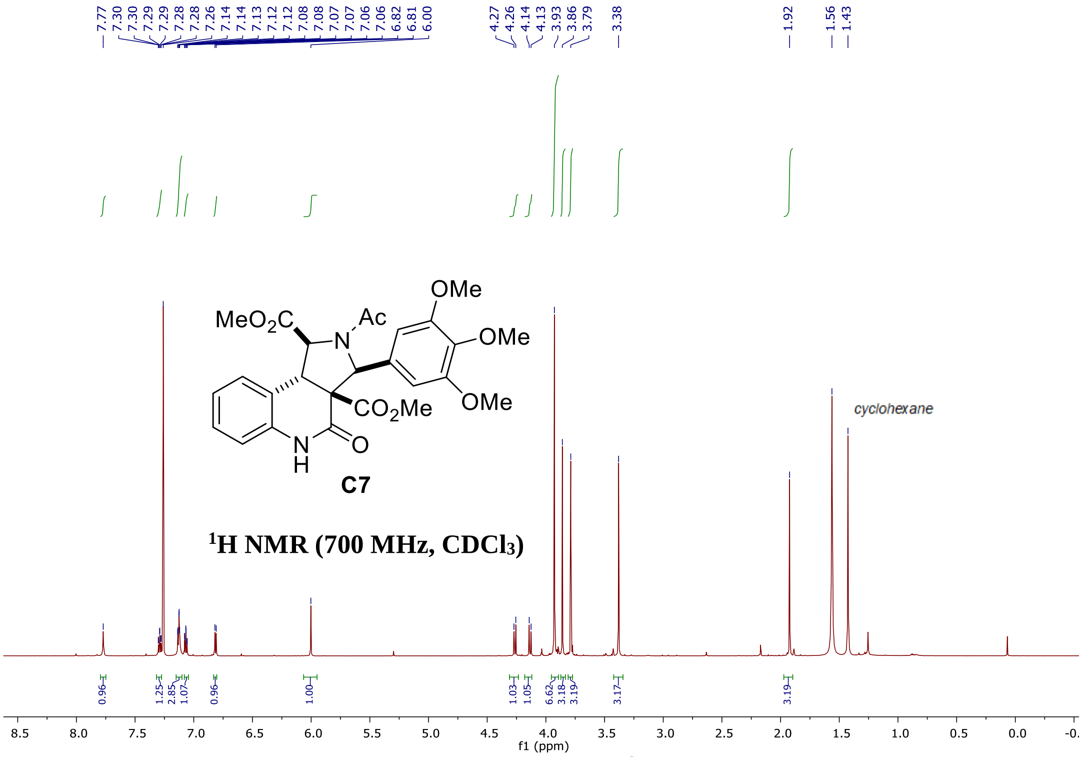

7.77
7.30
7.30
7.29
7.29
7.28
7.28
7.26
7.14
7.14
7.13
7.12
7.12
7.08
7.08
7.07
7.07
7.06
7.06
6.82
6.81
6.00
4.27
4.26
4.14
4.13
3.93
3.86
3.79
3.38
1.92
1.56
1.43
MeO2C
N
Ac
OMe
OMe
OMe
CO2Me
N
H
O
C7
1H NMR (700 MHz, CDCl3)
cyclohexane
0.96
1.25
2.85
1.07
0.96
1.00
1.03
1.05
6.62
3.18
3.19
3.17
3.19
8.5
8.0
7.5
7.0
6.5
6.0
5.5
5.0
4.5
4.0
3.5
3.0
2.5
2.0
1.5
1.0
0.5
0.0
f1 (ppm)

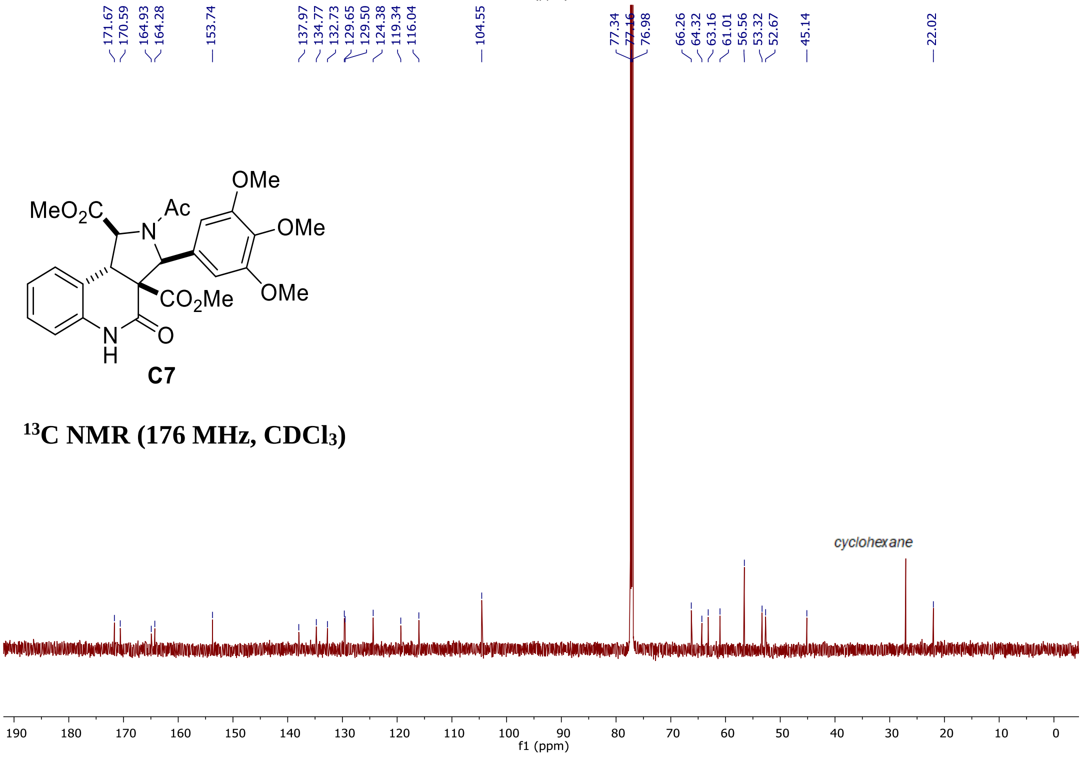

171.67
170.59
164.93
164.28
153.74
137.97
134.77
132.73
129.65
129.50
124.38
119.34
116.04
104.55
77.34
77.16
76.98
66.26
64.32
63.16
61.01
56.56
53.32
52.67
45.14
22.02
MeO2C
N
Ac
OMe
OMe
OMe
CO2Me
N
H
O
C7
13C NMR (176 MHz, CDCl3)
cyclohexane
190
180
170
160
150
140
130
120
110
100
90
80
70
60
50
40
30
20
10
0
f1 (ppm)

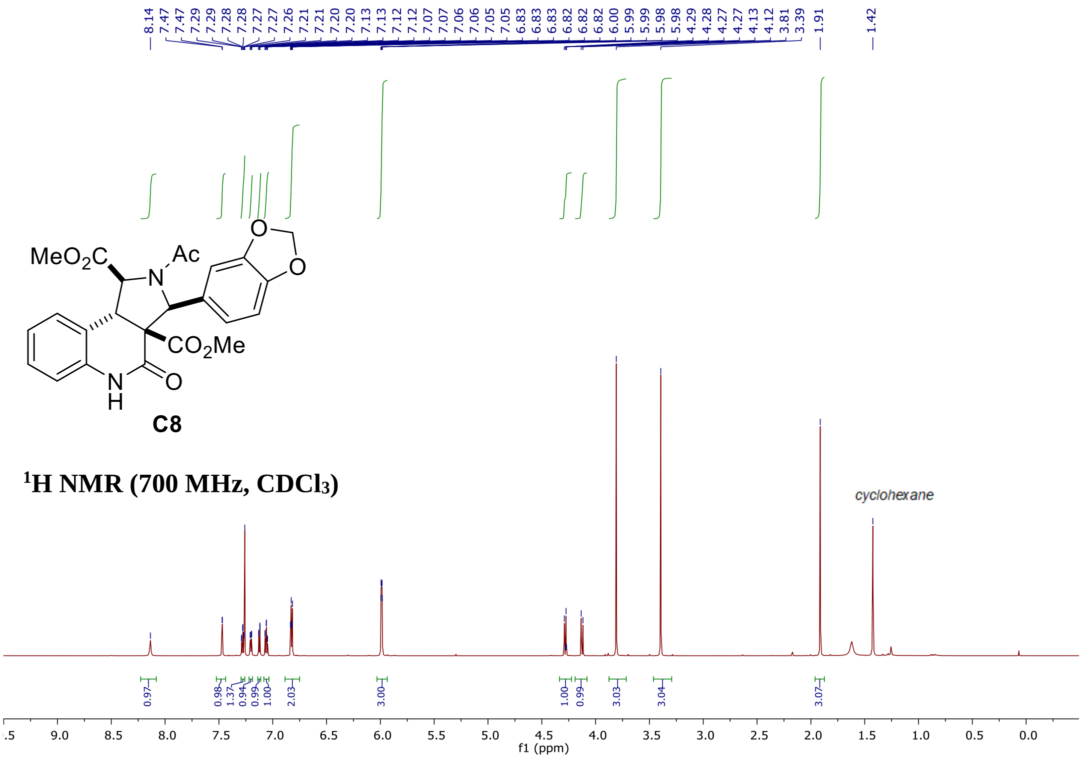

^{1}H NMR (700 MHz, CDCl$_3$)

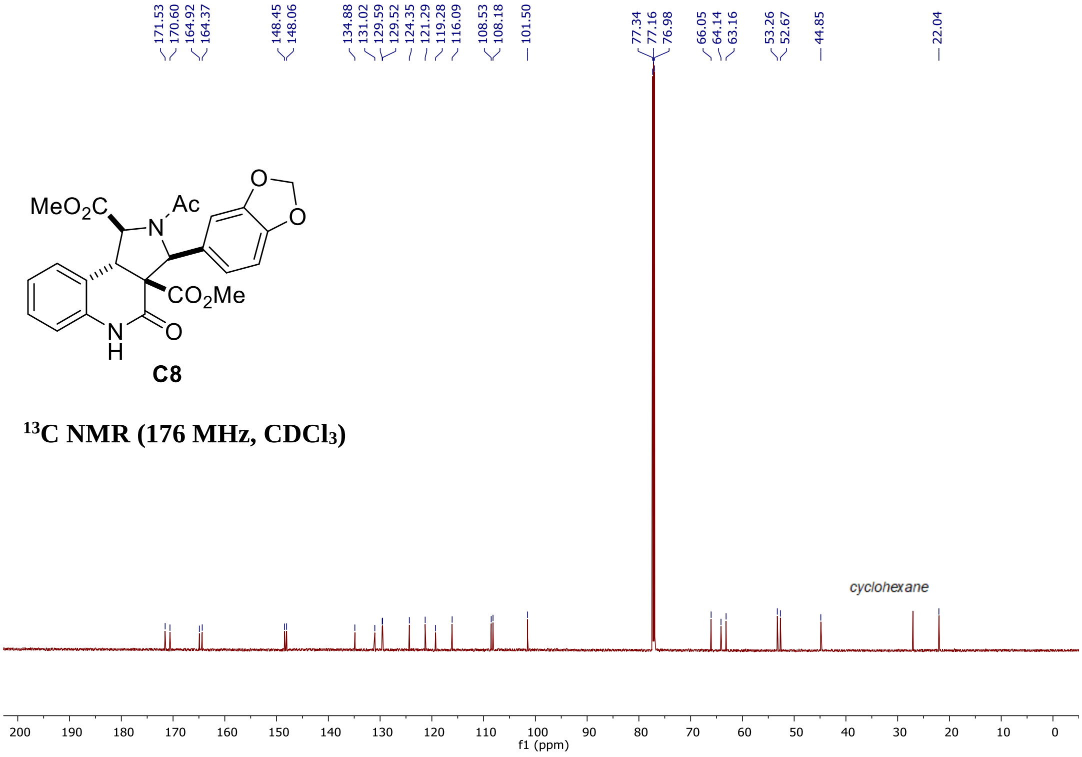

^{13}C NMR (176 MHz, CDCl$_3$)

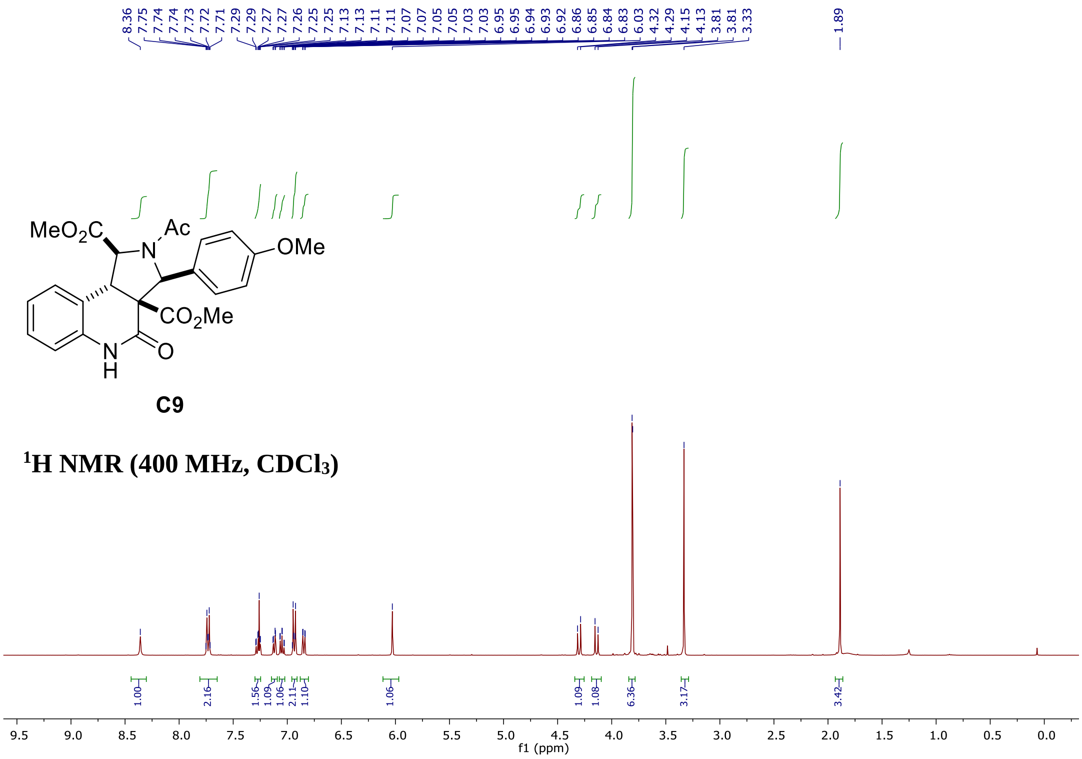
8.36 7.75 7.74 7.74 7.73 7.72 7.71 7.29 7.29 7.27 7.27 7.26 7.25 7.25 7.13 7.13 7.11 7.11 7.07 7.07 7.05 7.05 7.03 7.03 6.95 6.95 6.94 6.93 6.92 6.86 6.85 6.84 6.83 6.03 4.32 4.29 4.15 4.13 3.81 3.81 3.33
1.89
MeO2C
Ac
N
OMe
CO2Me
N
H
O
C9
1H NMR (400 MHz, CDCl3)
1.00 2.16 1.56 1.09 1.06 2.11 1.10 1.06 1.09 1.08 6.36 3.17 3.42
9.5 9.0 8.5 8.0 7.5 7.0 6.5 6.0 5.5 5.0 4.5 4.0 3.5 3.0 2.5 2.0 1.5 1.0 0.5 0.0
f1 (ppm)

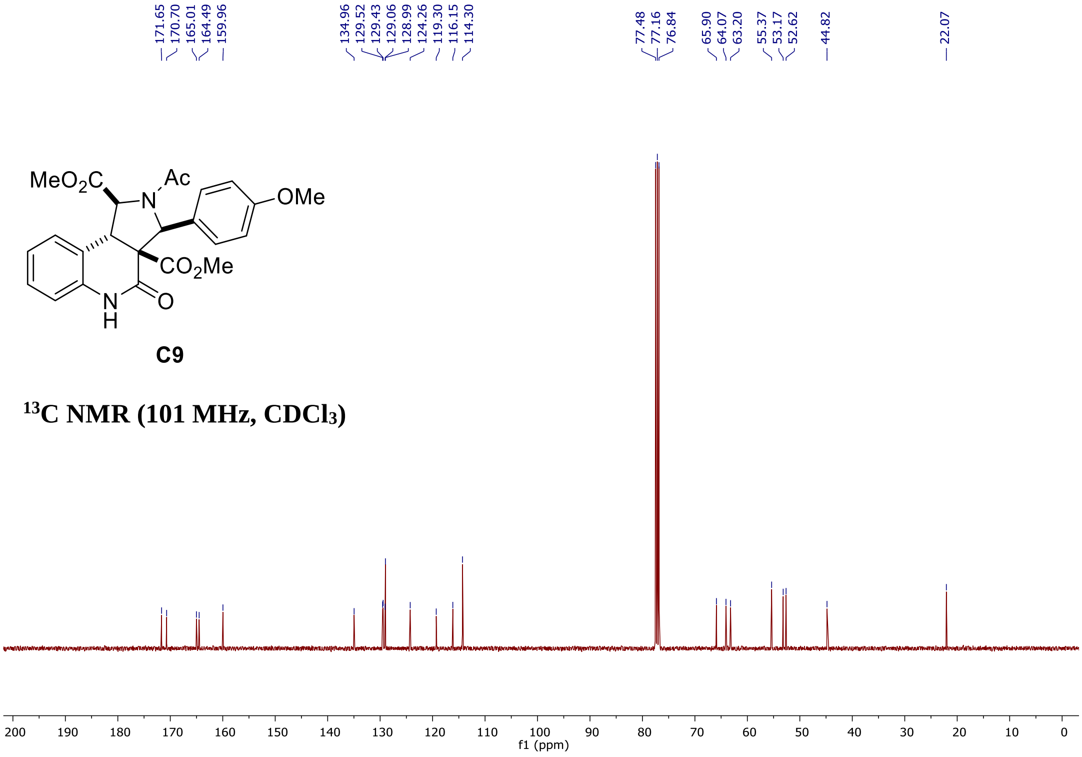
171.65 170.70 165.01 164.49 159.96
134.96 129.52 129.43 129.06 128.99 124.26 119.30 116.15 114.30
77.48 77.16 76.84
65.90 64.07 63.20 55.37 53.17 52.62 44.82
22.07
MeO2C
Ac
N
OMe
CO2Me
N
H
O
C9
13C NMR (101 MHz, CDCl3)
200 190 180 170 160 150 140 130 120 110 100 90 80 70 60 50 40 30 20 10 0
f1 (ppm)

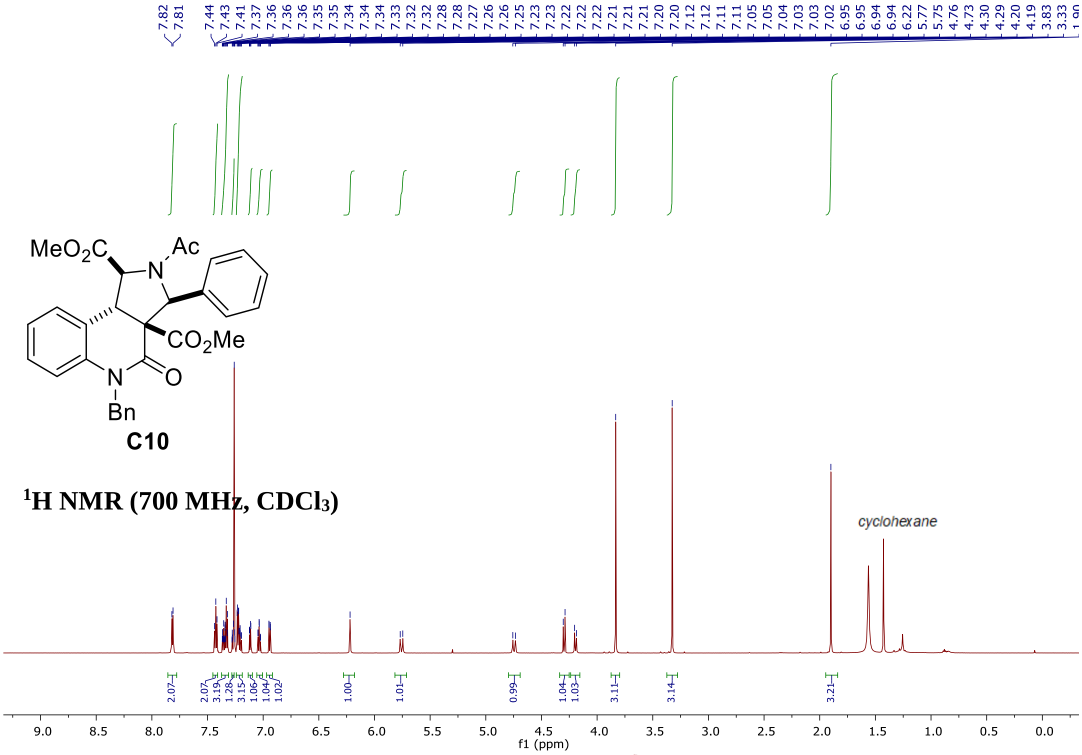
MeO2C
Ac
N
CO2Me
N
O
Bn
C10
1H NMR (700 MHz, CDCl3)
cyclohexane
f1 (ppm)

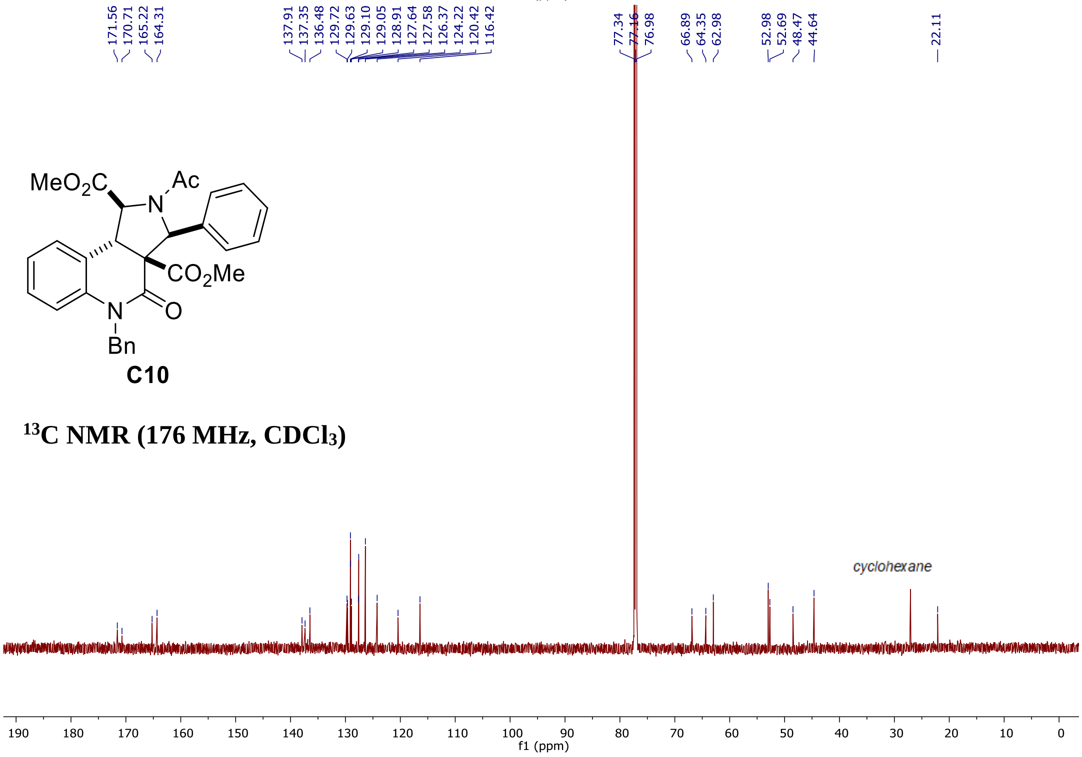
171.56
170.71
165.22
164.31
137.91
137.35
136.48
129.72
129.63
129.10
129.05
128.91
127.64
127.58
126.37
124.22
120.42
116.42
77.34
77.16
76.98
66.89
64.35
62.98
52.98
52.69
48.47
44.64
22.11
MeO2C
Ac
N
CO2Me
N
O
Bn
C10
13C NMR (176 MHz, CDCl3)
cyclohexane
f1 (ppm)

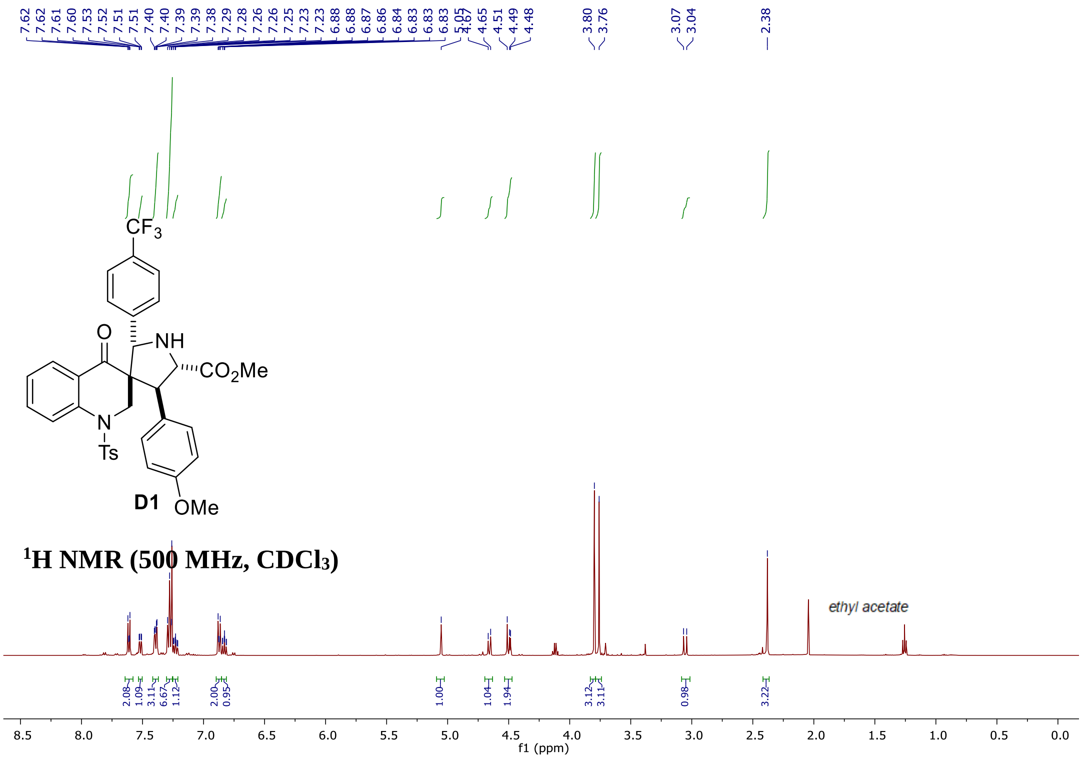
7.62 7.62 7.61 7.60 7.53 7.52 7.51 7.51 7.40 7.39 7.39 7.38 7.29 7.28 7.26 7.26 7.25 7.23 7.23 6.88 6.88 6.87 6.86 6.84 6.83 6.83 6.83 5.05 4.67 4.65 4.51 4.49 4.48 3.80 3.76 3.07 3.04 2.38
CF3
O
NH
CO2Me
N
Ts
D1
OMe
1H NMR (500 MHz, CDCl3)
ethyl acetate
2.08 1.09 3.11 6.67 1.12 2.00 0.95 1.00 1.04 1.94 3.12 3.11 0.98 3.22
8.5 8.0 7.5 7.0 6.5 6.0 5.5 5.0 4.5 4.0 3.5 3.0 2.5 2.0 1.5 1.0 0.5 0.0
f1 (ppm)

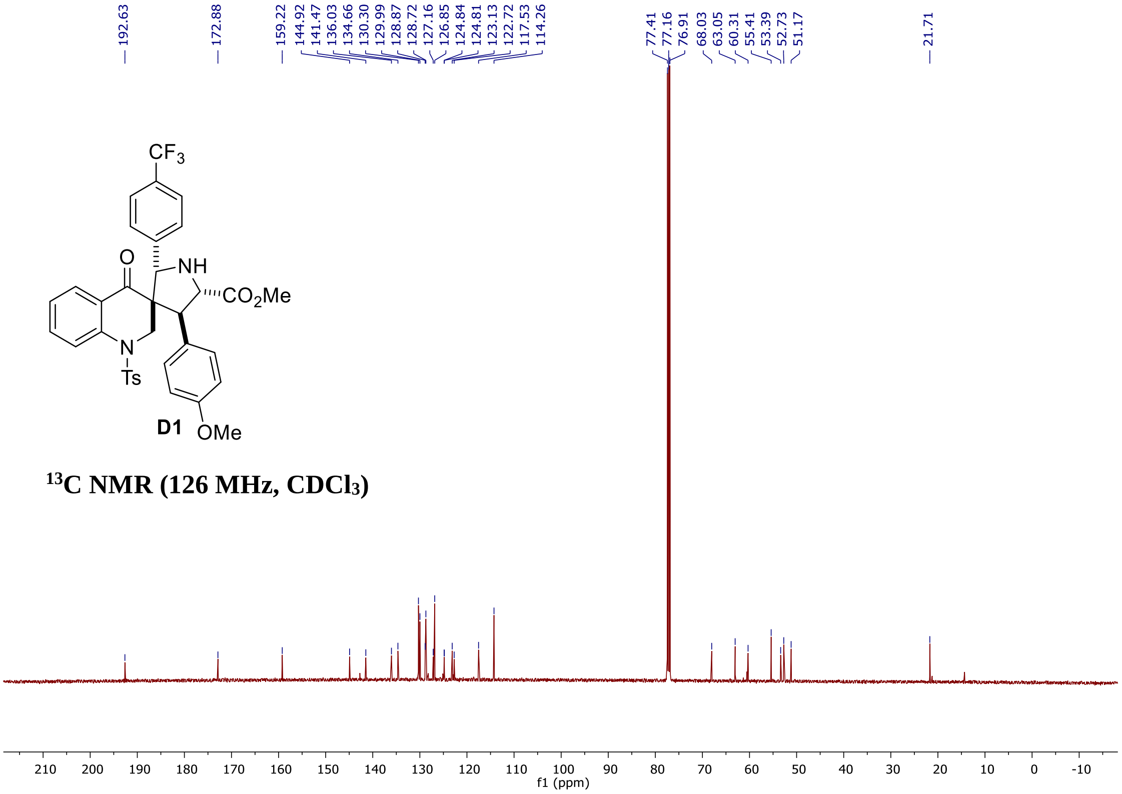
192.63 172.88 159.22 144.92 141.47 136.03 134.66 130.30 129.99 128.87 128.72 127.16 126.85 124.84 124.81 123.13 122.72 117.53 114.26 77.41 77.16 76.91 68.03 63.05 60.31 55.41 53.39 52.73 51.17 21.71
CF3
O
NH
CO2Me
N
Ts
D1
OMe
13C NMR (126 MHz, CDCl3)
210 200 190 180 170 160 150 140 130 120 110 100 90 80 70 60 50 40 30 20 10 0 -10
f1 (ppm)

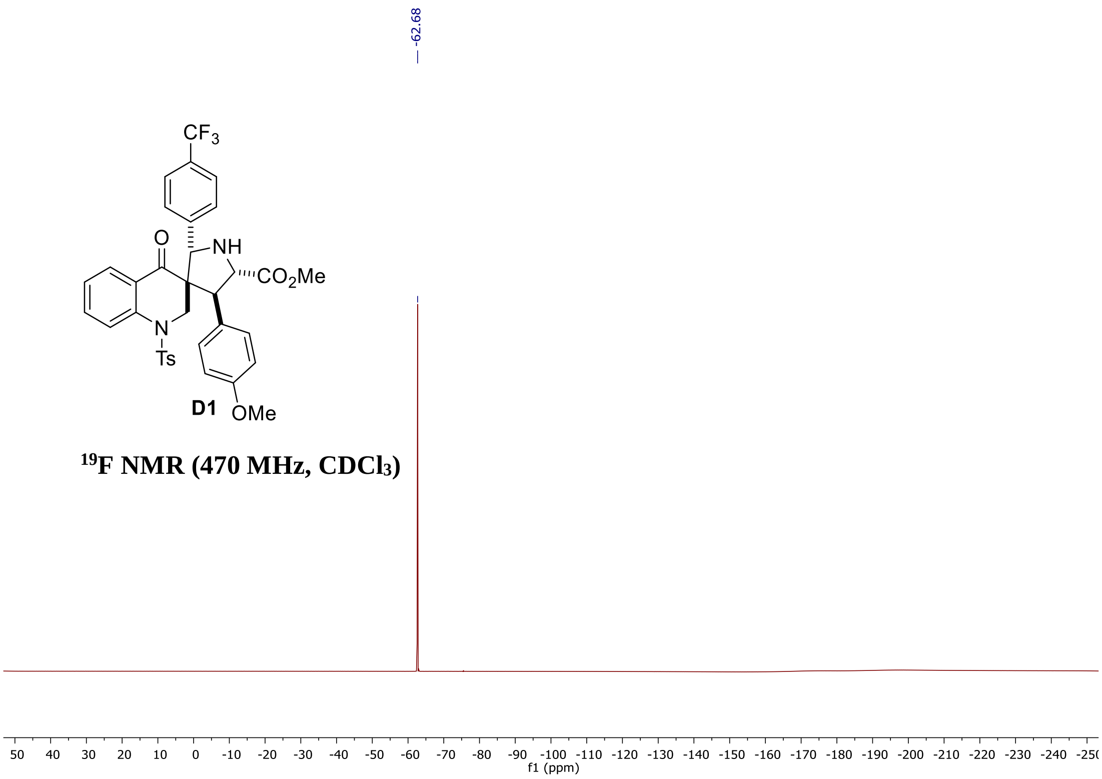

^{19}F NMR (470 MHz, $CDCl_3$)

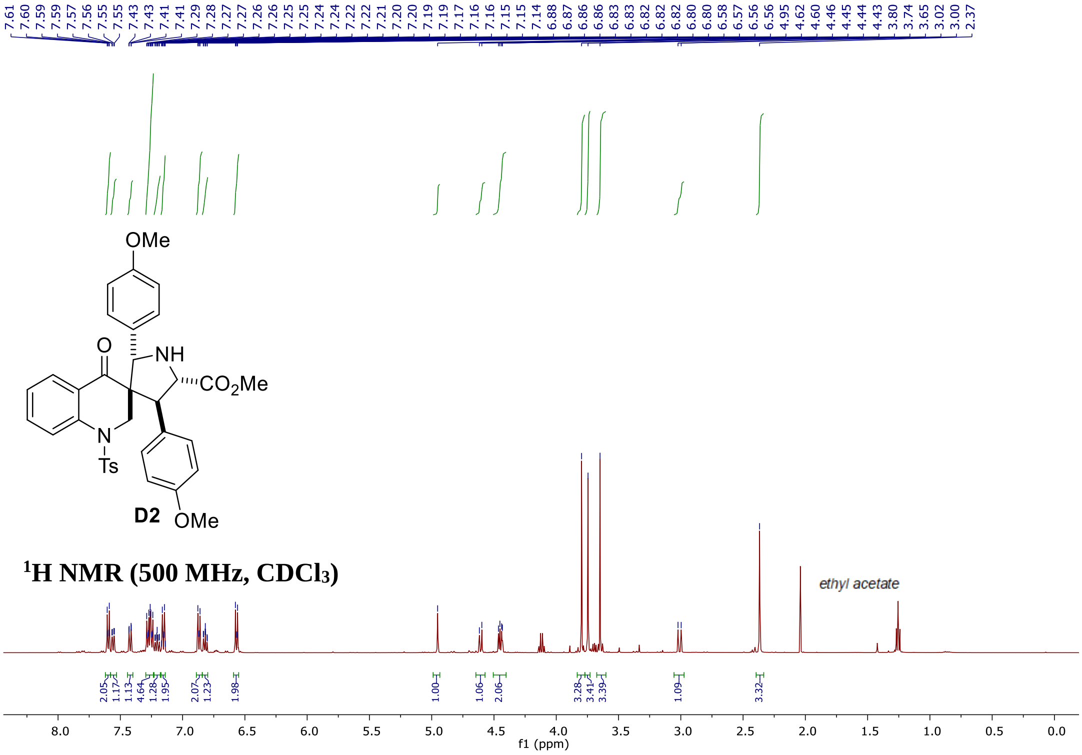
OMe
NH
CO2Me
N
Ts
D2
OMe
1H NMR (500 MHz, CDCl3)
ethyl acetate
f1 (ppm)

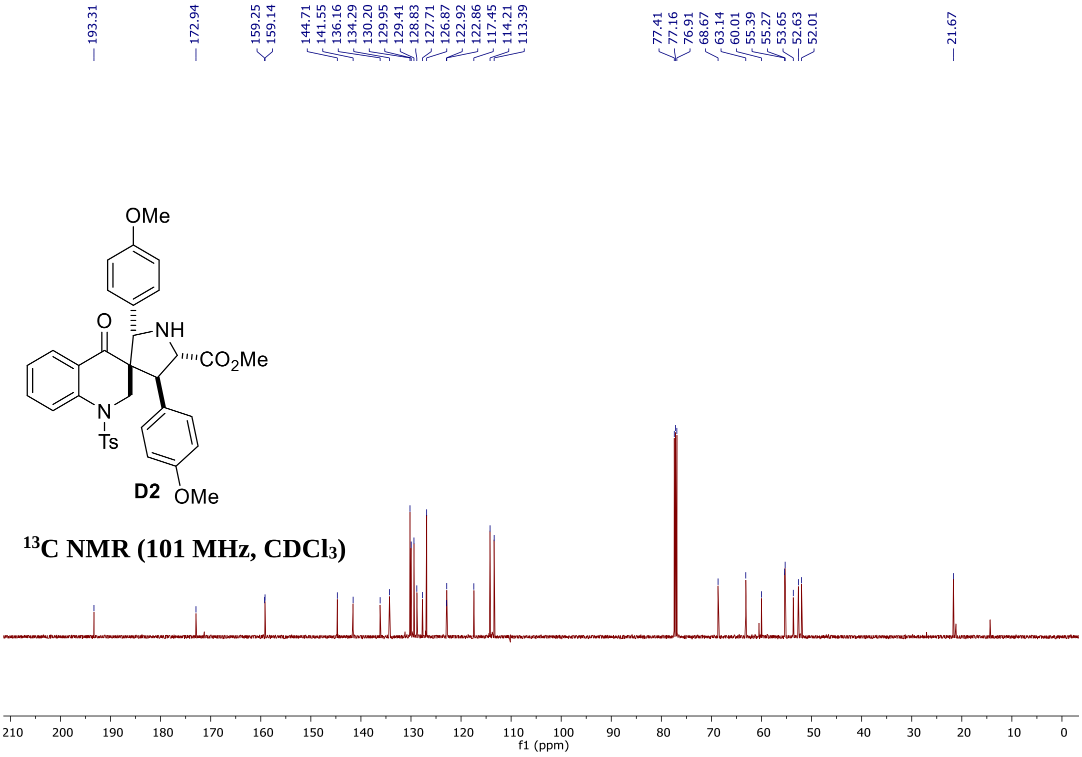
OMe
NH
CO2Me
N
Ts
D2
OMe
13C NMR (101 MHz, CDCl3)
f1 (ppm)

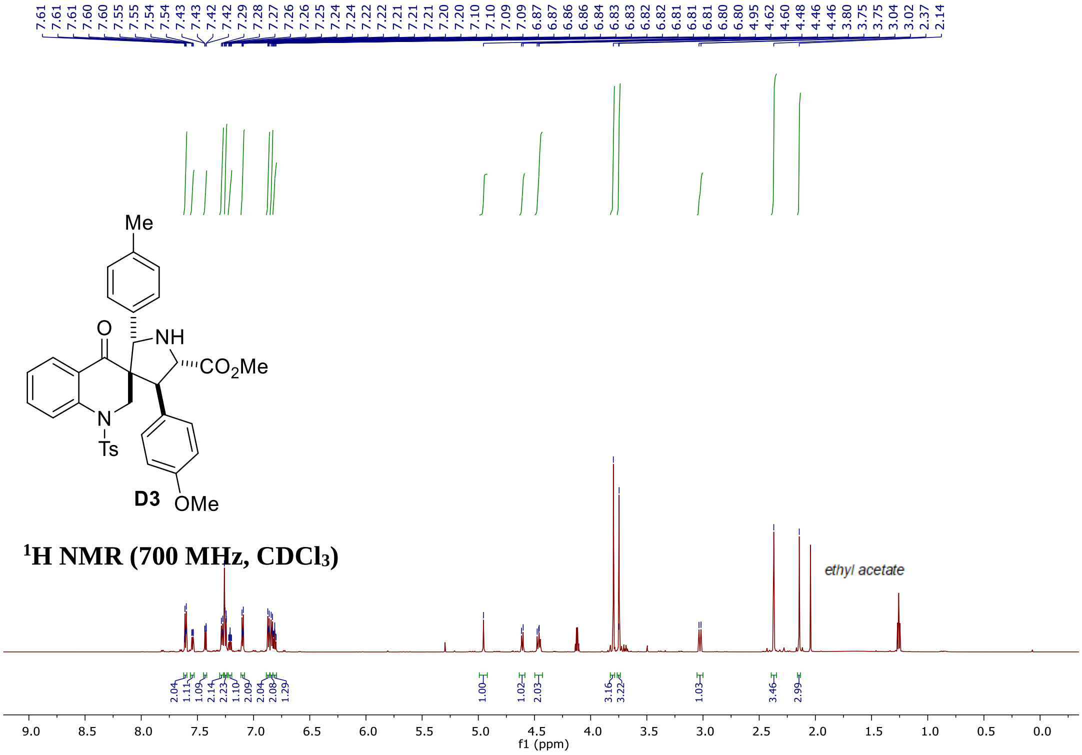
7.61 7.61 7.61 7.60 7.55 7.55 7.54 7.54 7.43 7.43 7.42 7.42 7.29 7.28 7.27 7.26 7.26 7.25 7.24 7.24 7.22 7.22 7.21 7.21 7.21 7.21 7.20 7.20 7.10 7.10 7.09 7.09 6.87 6.87 6.86 6.84 6.83 6.83 6.82 6.82 6.81 6.81 6.81 6.80 6.80 4.95 4.62 4.60 4.48 4.46 4.46 3.80 3.75 3.75 3.04 3.02 2.37 2.14
Me
O
NH
CO_2Me
N
Ts
D3
OMe
^1H NMR (700 MHz, $CDCl_3$)
ethyl acetate
2.04 1.11 1.09 2.14 2.23 1.10 2.09 2.04 2.08 1.29 1.00 1.02 2.03 3.16 3.22 1.03 3.46 2.99
9.0 8.5 8.0 7.5 7.0 6.5 6.0 5.5 5.0 4.5 4.0 3.5 3.0 2.5 2.0 1.5 1.0 0.5 0.0
f1 (ppm)

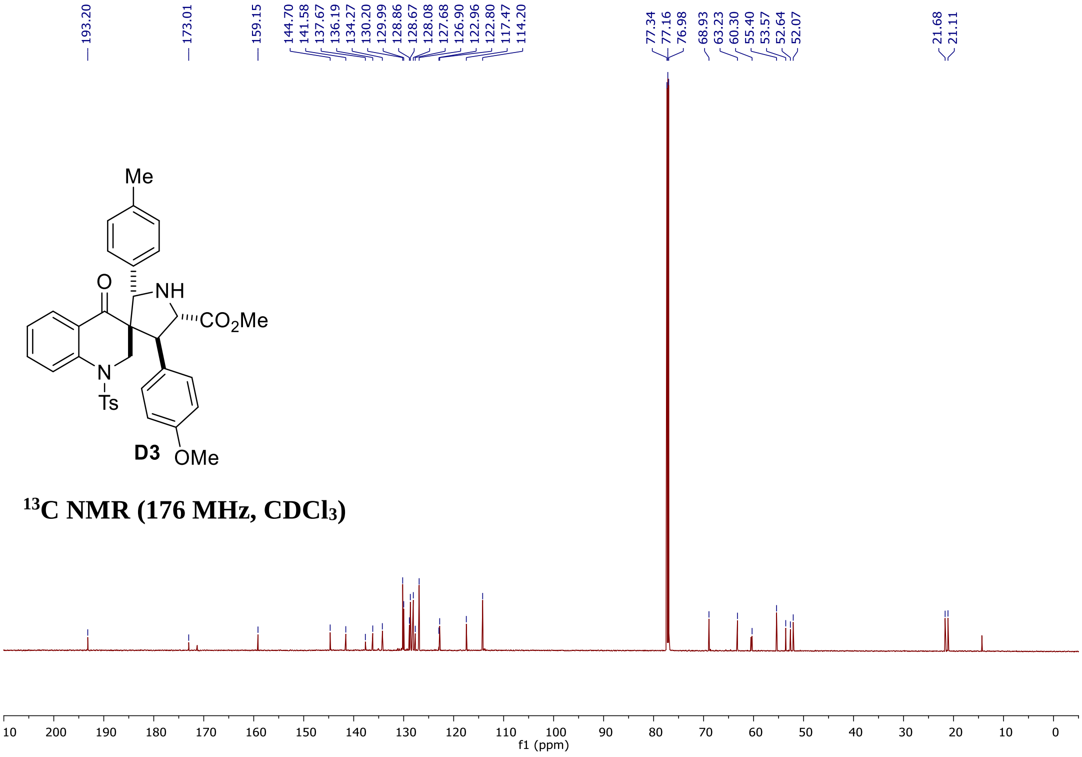
193.20 173.01 159.15 144.70 141.58 137.67 136.19 134.27 130.20 129.99 128.86 128.67 128.08 127.68 126.90 122.96 122.80 117.47 114.20 77.34 77.16 76.98 68.93 63.23 60.30 55.40 53.57 52.64 52.07 21.68 21.11
Me
O
NH
CO_2Me
N
Ts
D3
OMe
^{13}C NMR (176 MHz, $CDCl_3$)
10 200 190 180 170 160 150 140 130 120 110 100 90 80 70 60 50 40 30 20 10 0
f1 (ppm)

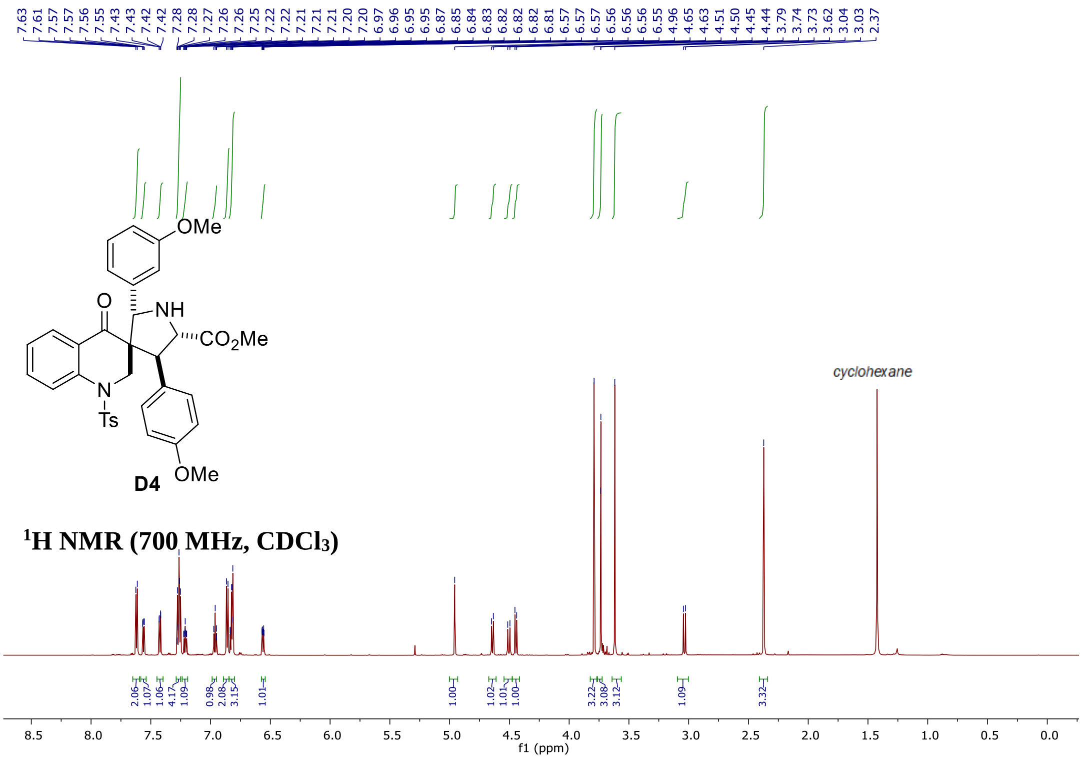

^{1}H NMR (700 MHz, $CDCl_3$)

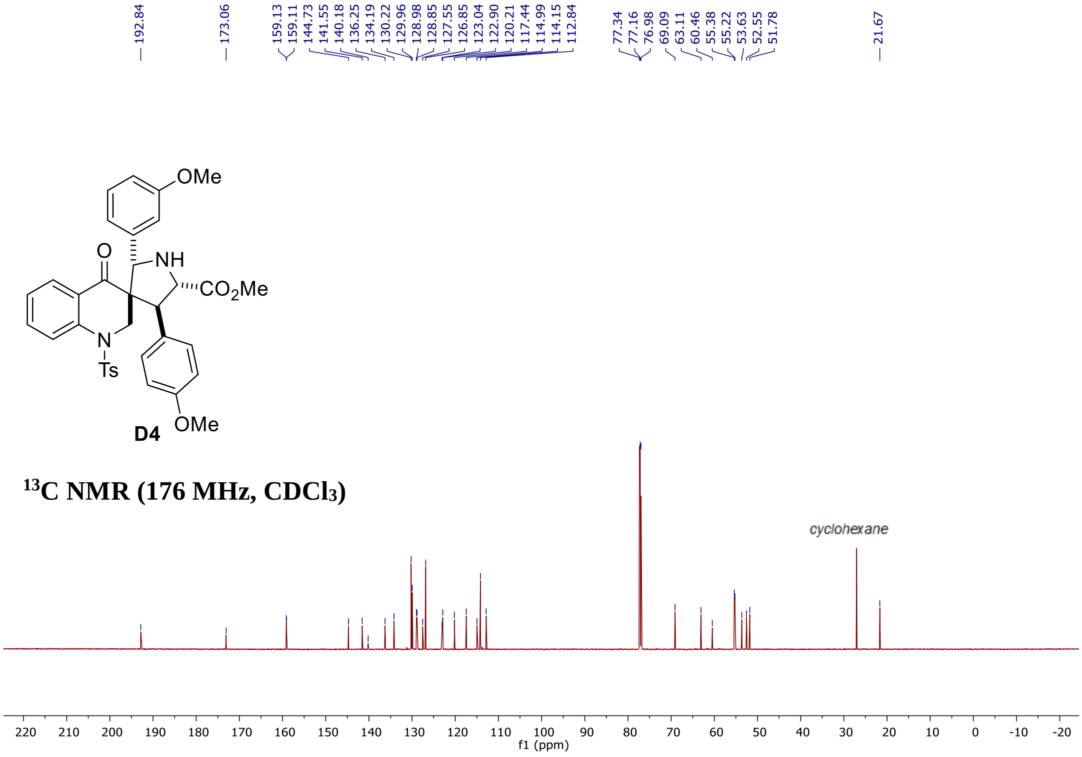

^{13}C NMR (176 MHz, $CDCl_3$)

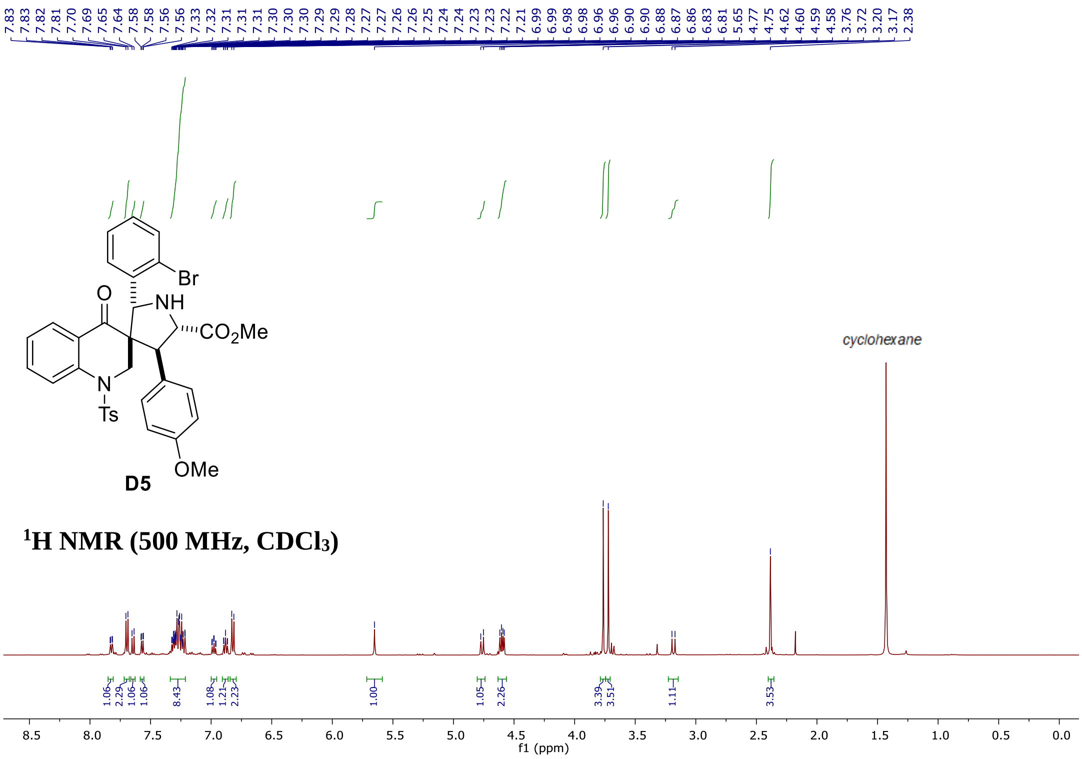

^{1}H NMR (500 MHz, $CDCl_3$)

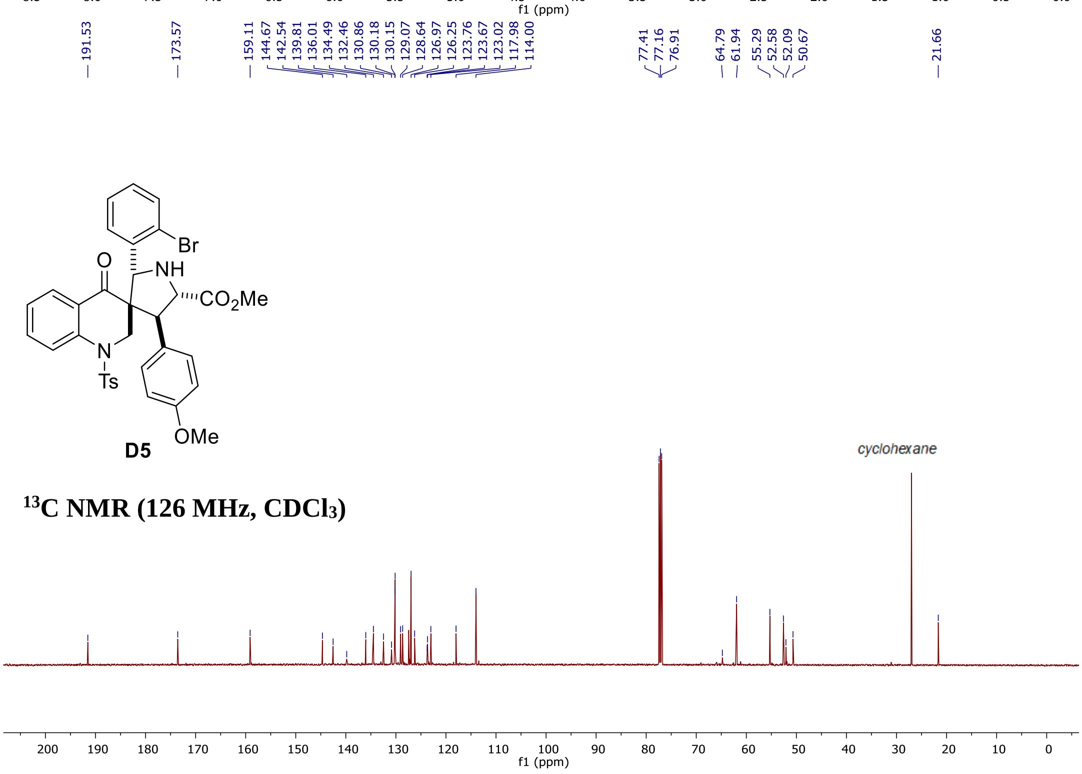

^{13}C NMR (126 MHz, $CDCl_3$)

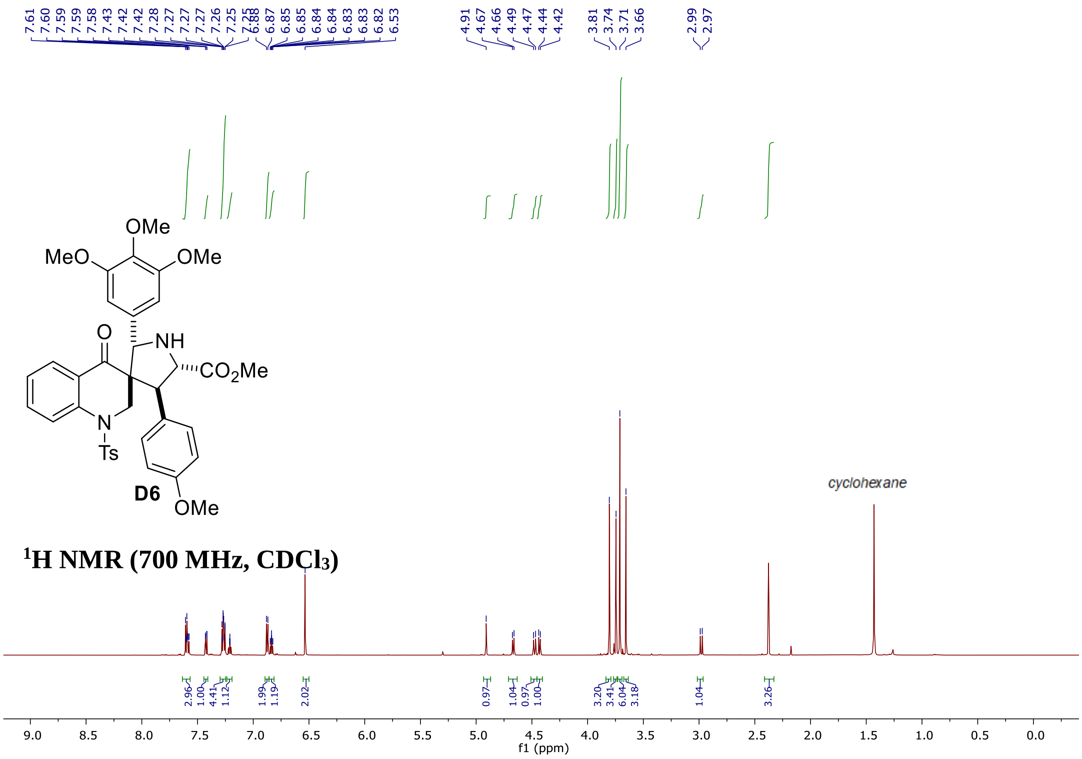

^{1}H NMR (700 MHz, $CDCl_3$)

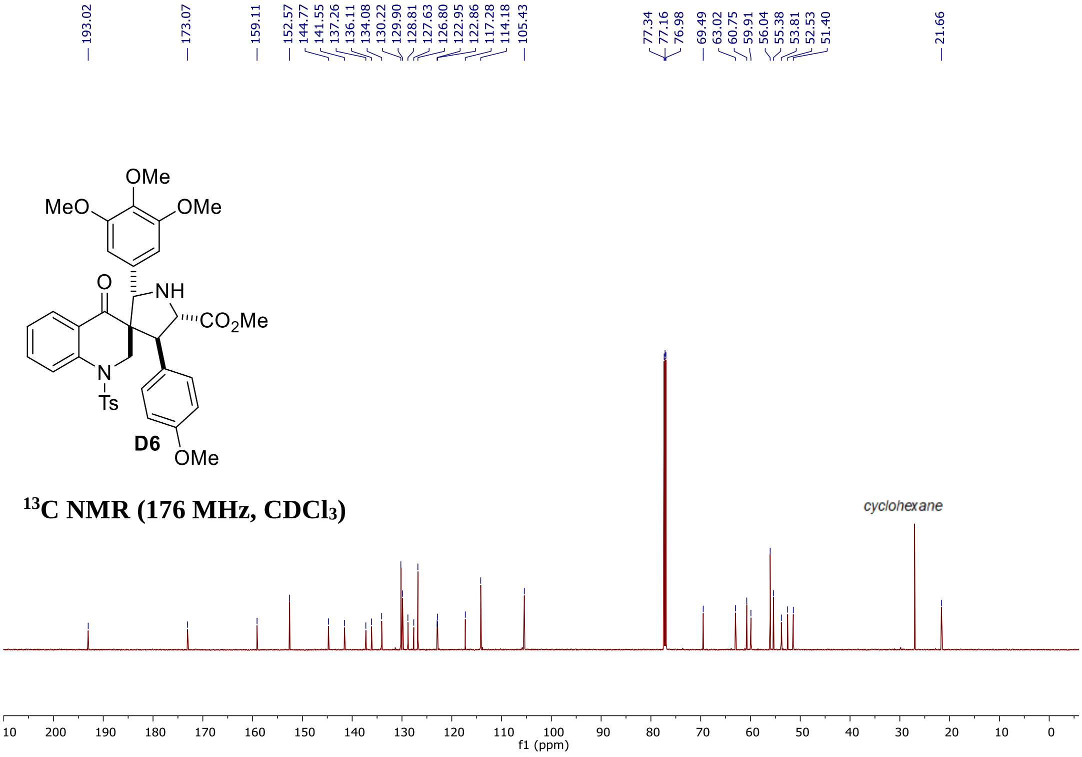

^{13}C NMR (176 MHz, $CDCl_3$)

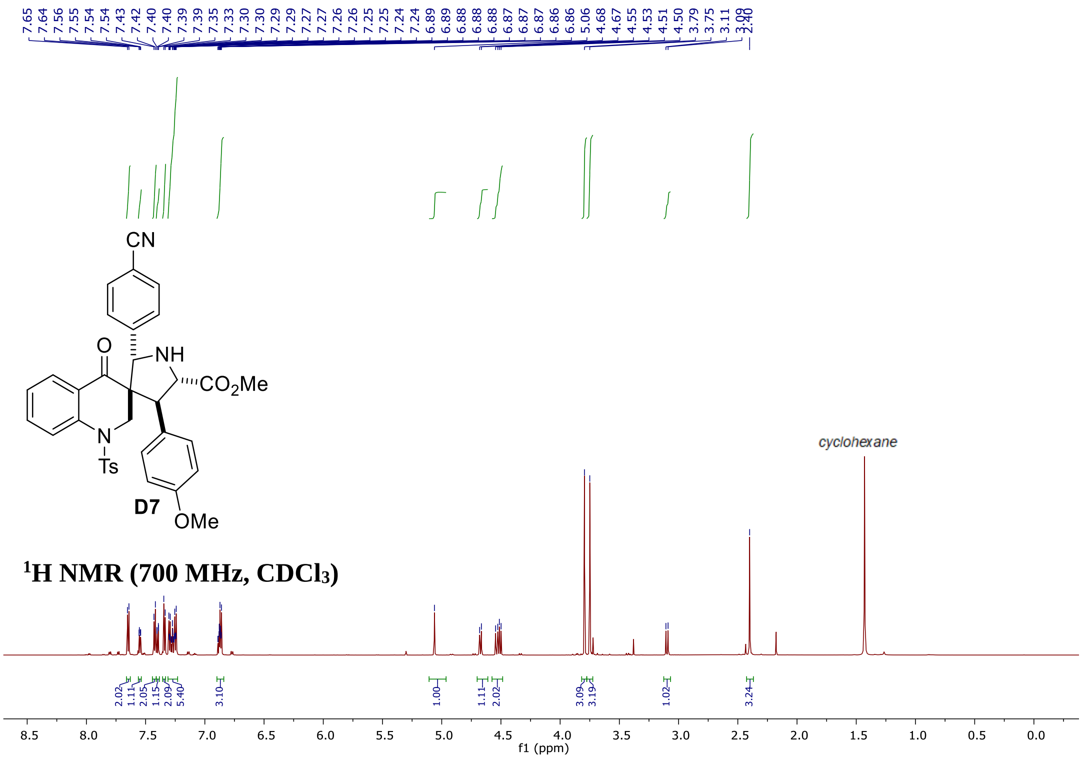

D7

^{1}H NMR (700 MHz, CDCl3)

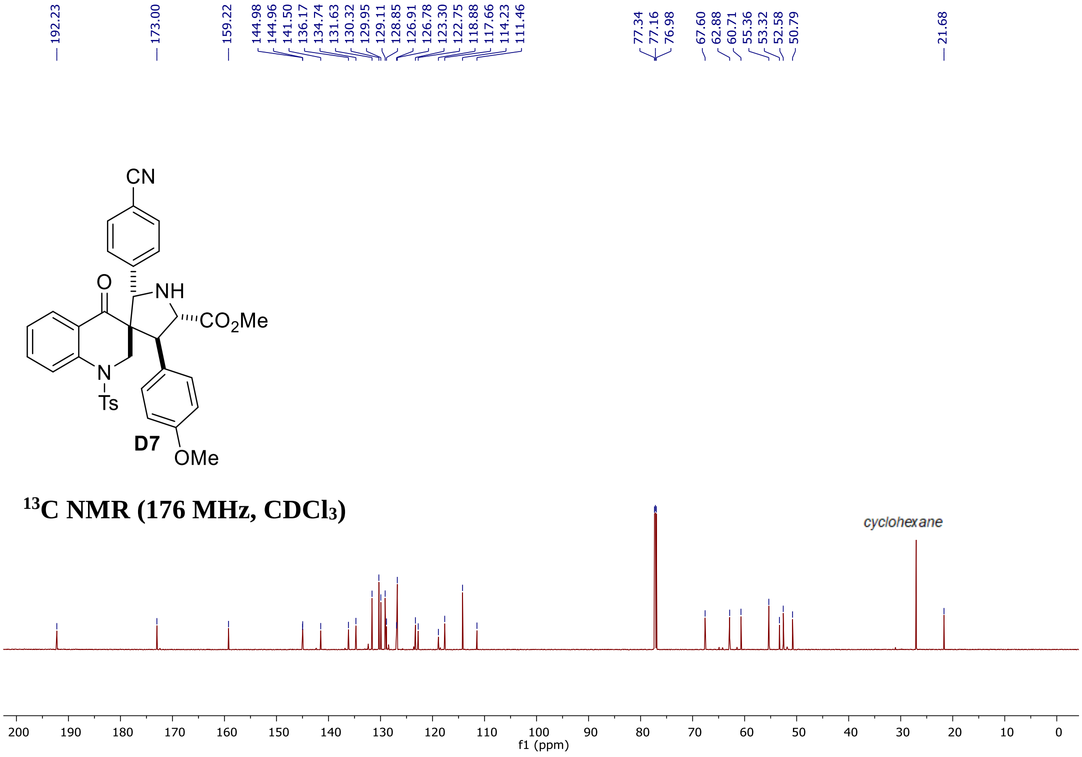

D7

^{13}C NMR (176 MHz, CDCl3)

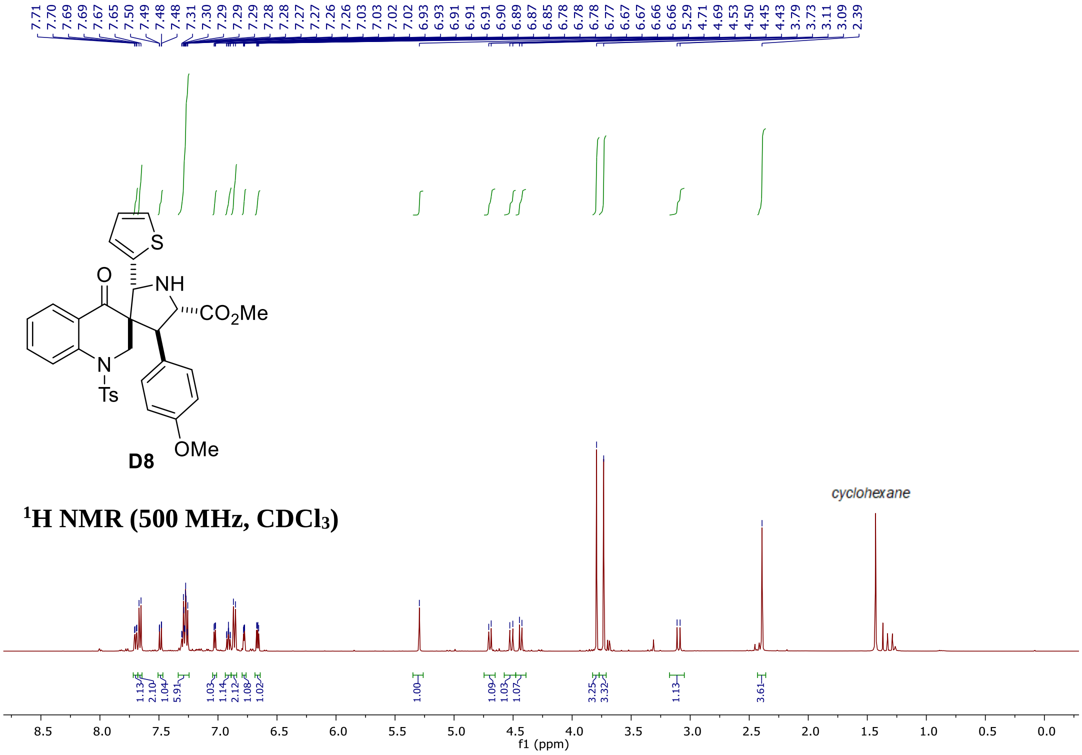

7.71 7.70 7.69 7.67 7.65 7.50 7.49 7.48 7.48 7.31 7.30 7.29 7.29 7.29 7.28 7.27 7.27 7.26 7.03 7.03 7.02 7.02 6.93 6.93 6.91 6.91 6.91 6.90 6.89 6.87 6.85 6.78 6.78 6.78 6.77 6.67 6.67 6.66 6.66 5.29 4.71 4.69 4.53 4.50 4.45 4.43 3.79 3.73 3.11 3.09 2.39
O
NH
CO2Me
N
Ts
S
OMe
D8
¹H NMR (500 MHz, CDCl3)
cyclohexane
1.13 2.10 1.04 5.91 1.03 1.14 2.12 1.08 1.02 1.00 1.09 1.03 1.07 3.25 3.32 1.13 3.61
8.5 8.0 7.5 7.0 6.5 6.0 5.5 5.0 4.5 4.0 3.5 3.0 2.5 2.0 1.5 1.0 0.5 0.0
f1 (ppm)

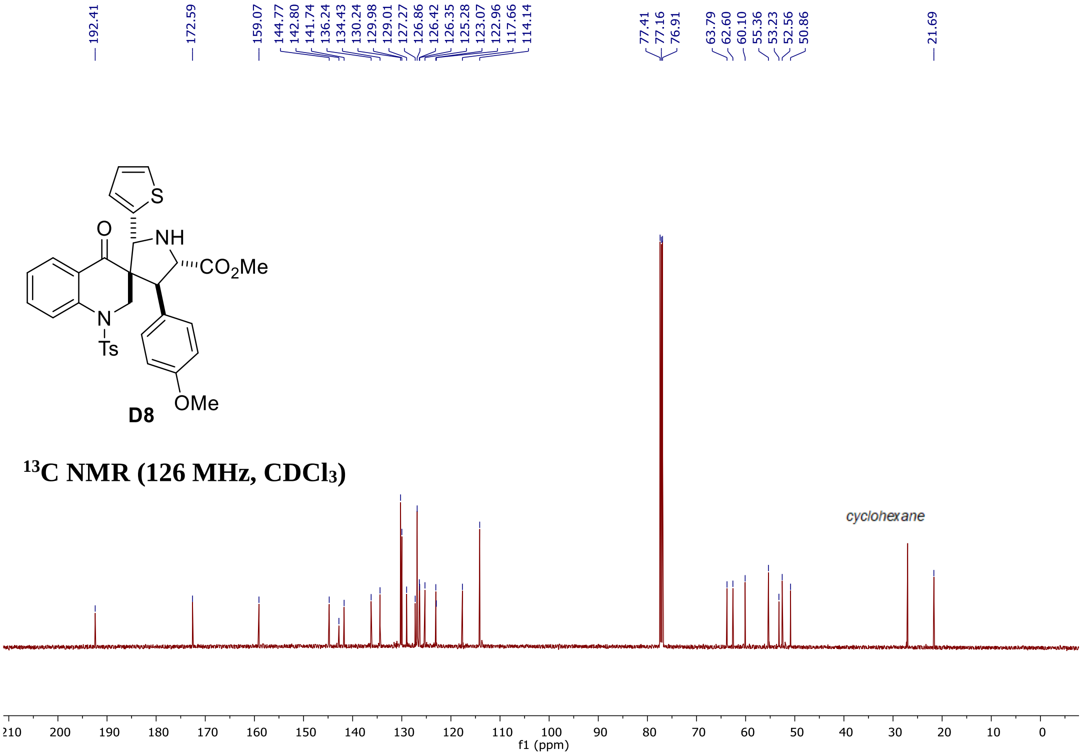

192.41 172.59 159.07 144.77 142.80 141.74 136.24 134.43 130.24 129.98 129.01 127.27 126.86 126.42 126.35 125.28 123.07 122.96 117.66 114.14 77.41 77.16 76.91 63.79 62.60 60.10 55.36 53.23 52.56 50.86 21.69
O
NH
CO2Me
N
Ts
S
OMe
D8
¹³C NMR (126 MHz, CDCl3)
cyclohexane
210 200 190 180 170 160 150 140 130 120 110 100 90 80 70 60 50 40 30 20 10 0
f1 (ppm)

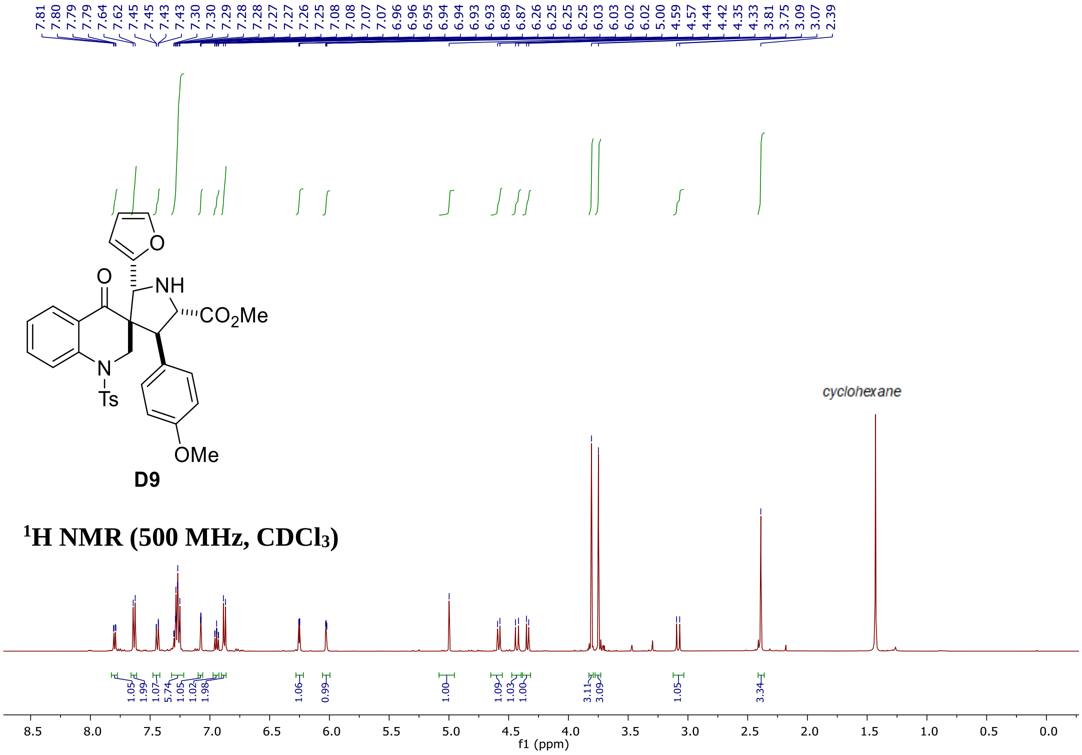
7.81 7.80 7.79 7.79 7.64 7.62 7.45 7.45 7.43 7.43 7.30 7.29 7.28 7.28 7.27 7.26 7.25 7.08 7.08 7.07 7.07 6.96 6.96 6.95 6.94 6.94 6.93 6.93 6.89 6.87 6.26 6.25 6.25 6.25 6.03 6.03 6.02 6.02 5.00 4.59 4.57 4.44 4.42 4.35 4.33 3.81 3.75 3.09 3.07 2.39
O
NH
CO2Me
N
Ts
OMe
D9
1H NMR (500 MHz, CDCl3)
cyclohexane
1.05 1.99 1.07 5.74 1.05 1.02 1.98 1.06 0.99 1.00 1.09 1.03 1.00 3.11 3.09 1.05 3.34
f1 (ppm)

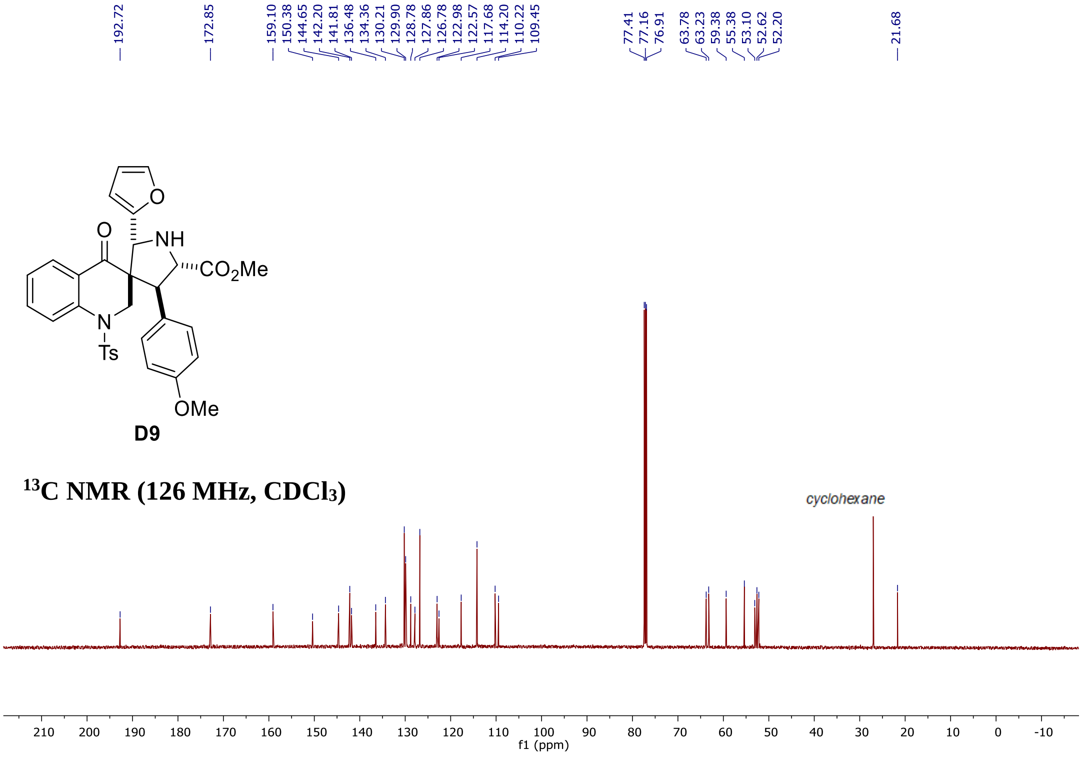
192.72
172.85
159.10 150.38 144.65 142.20 141.81 136.48 134.36 130.21 129.90 128.78 127.86 126.78 122.98 122.57 117.68 114.20 110.22 109.45
77.41 77.16 76.91
63.78 63.23 59.38 55.38 53.10 52.62 52.20
21.68
O
NH
CO2Me
N
Ts
OMe
D9
13C NMR (126 MHz, CDCl3)
cyclohexane
f1 (ppm)

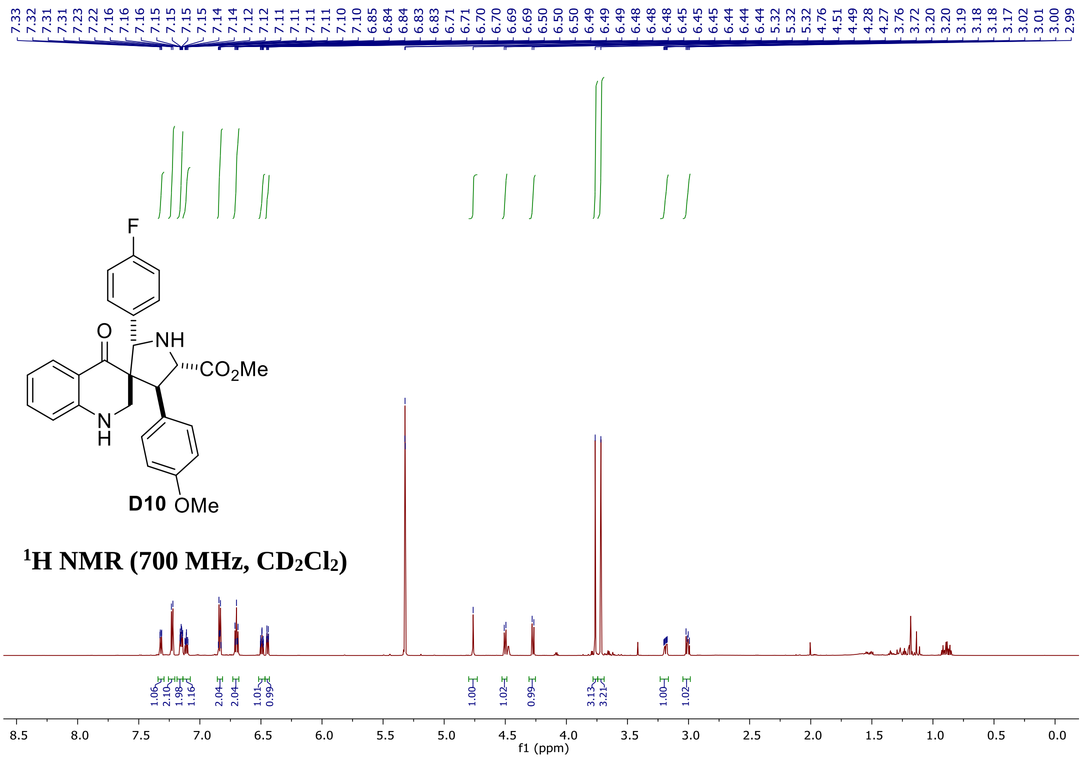

^{1}H NMR (700 MHz, CD_2Cl_2)

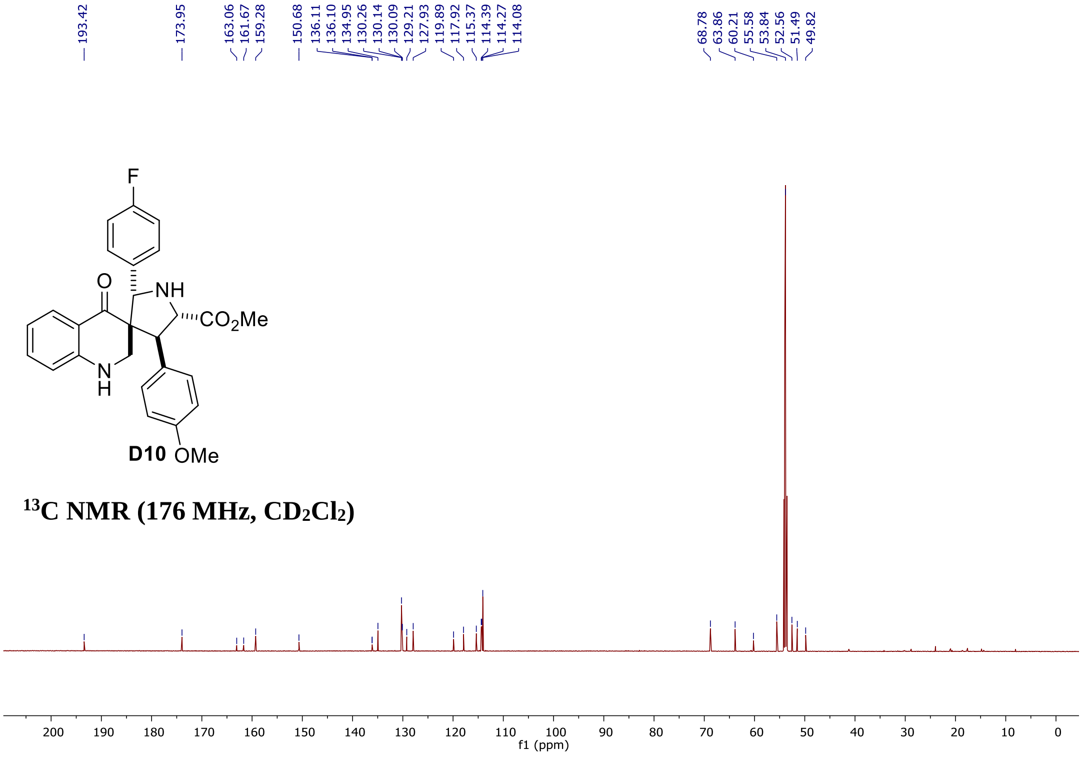

^{13}C NMR (176 MHz, CD_2Cl_2)

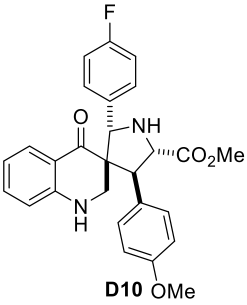

D10

^{19}F NMR (470 MHz, CDCl$_3$)

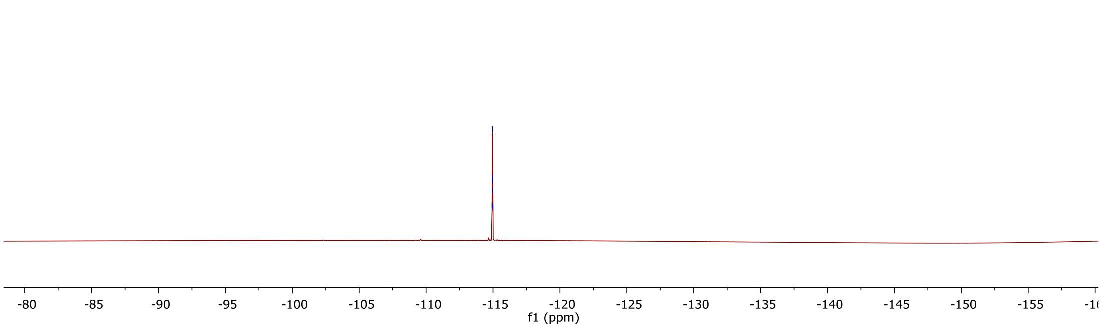

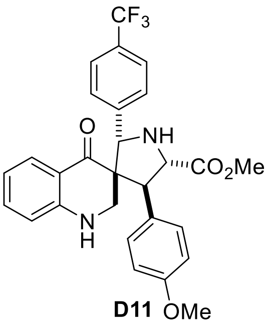

D11

^{19}F NMR (470 MHz, CDCl$_3$)

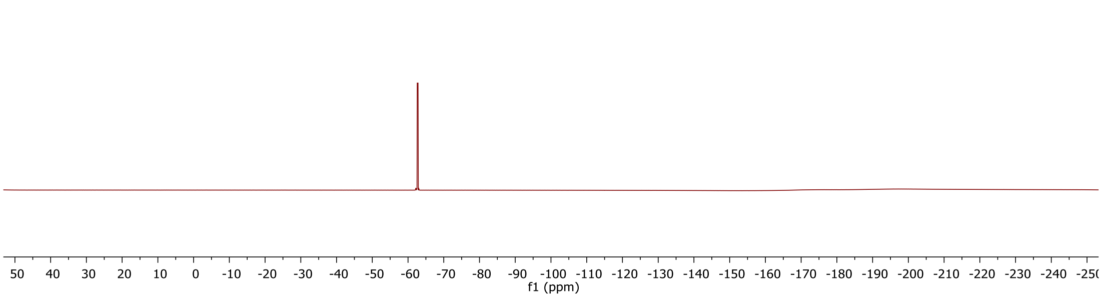

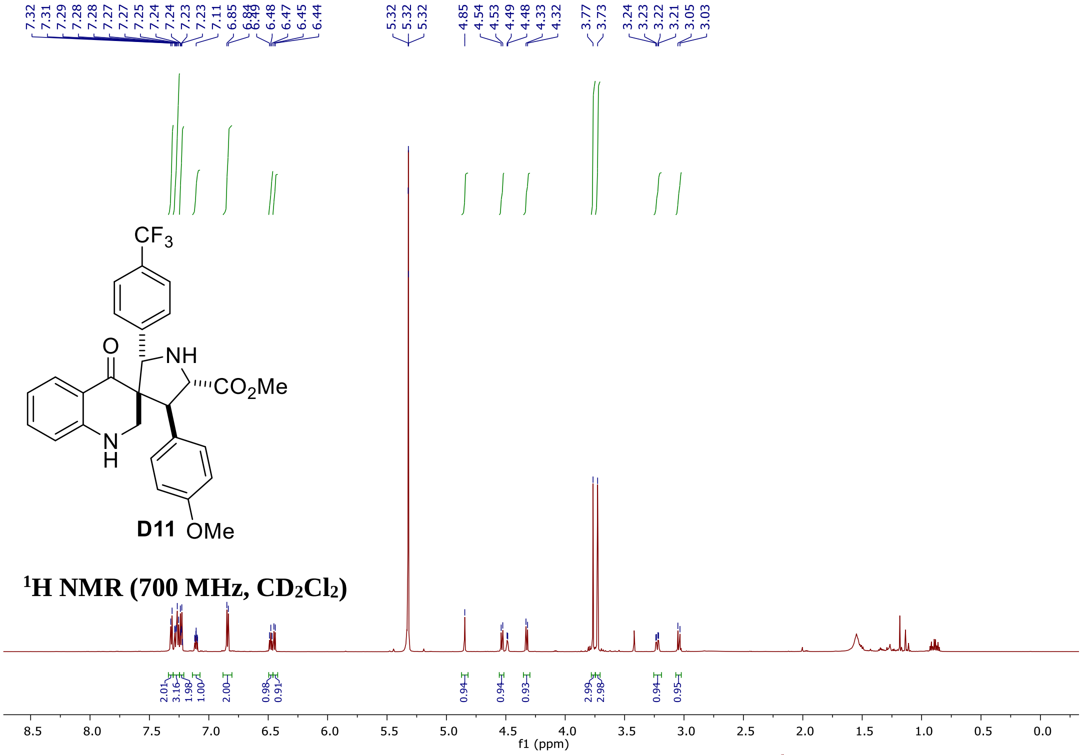

^{1}H NMR (700 MHz, CD_2Cl_2)

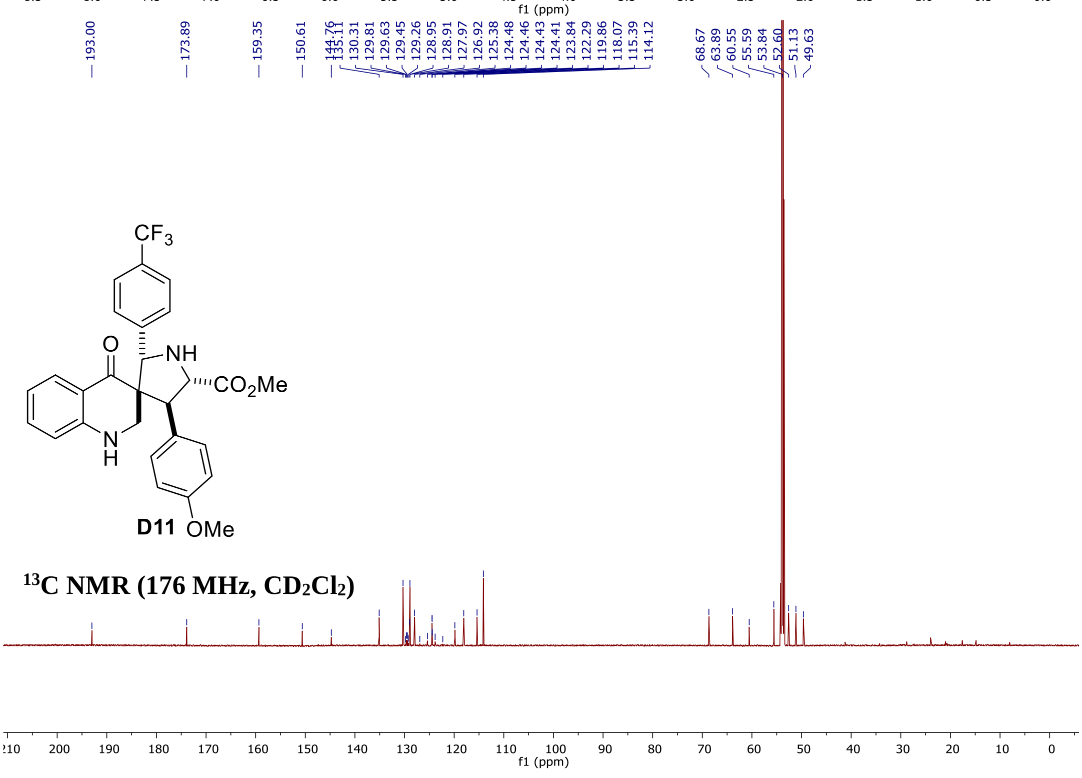

^{13}C NMR (176 MHz, CD_2Cl_2)

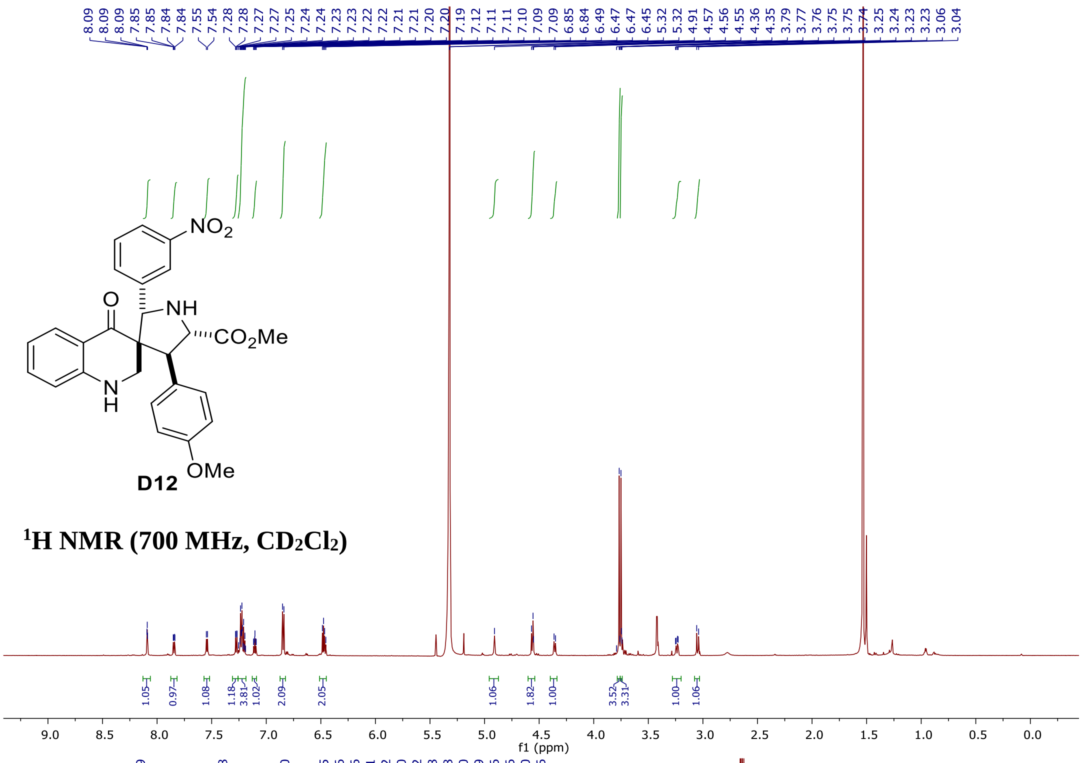
NO2
NH
CO2Me
N
H
OMe
D12
1H NMR (700 MHz, CD2Cl2)
f1 (ppm)

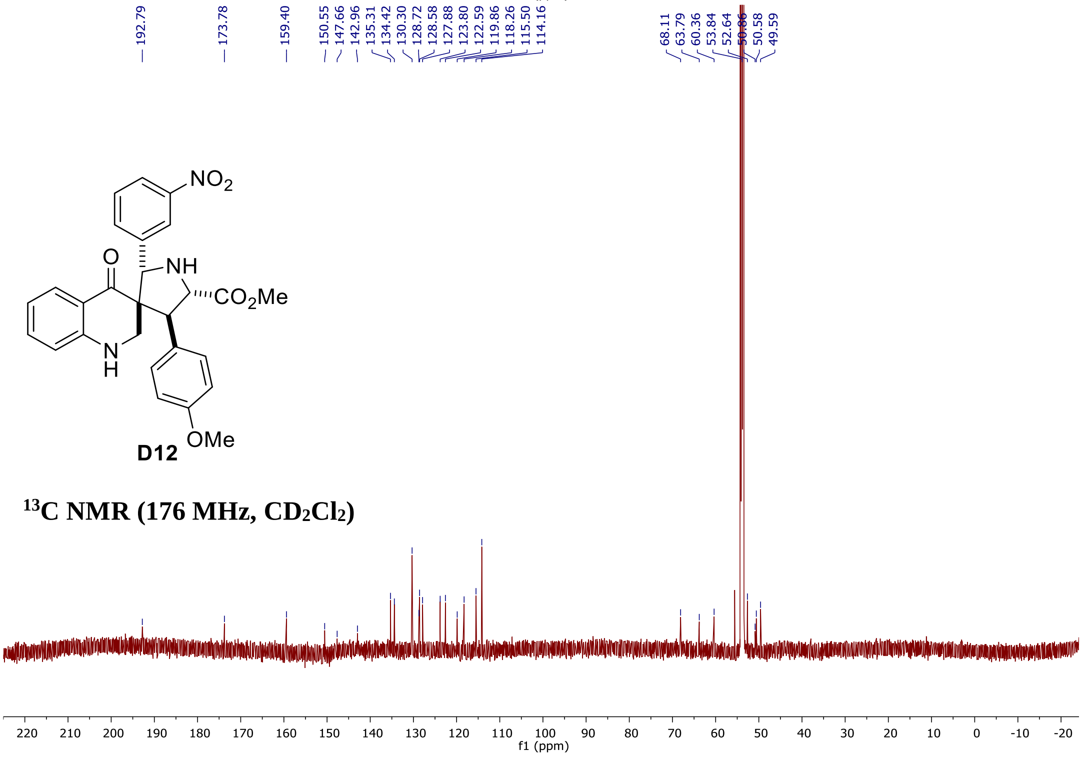
NO2
NH
CO2Me
N
H
OMe
D12
13C NMR (176 MHz, CD2Cl2)
f1 (ppm)

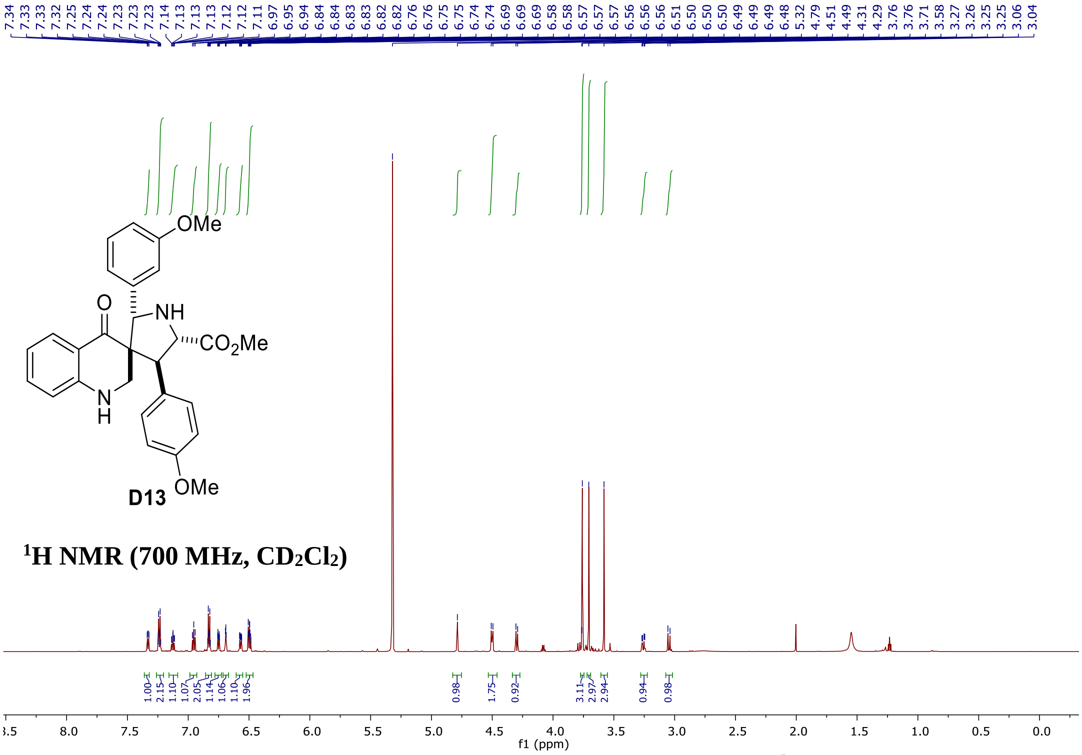
7.34
7.33
7.33
7.32
7.25
7.24
7.24
7.23
7.23
7.23
7.14
7.13
7.13
7.13
7.12
7.12
7.11
6.97
6.95
6.94
6.84
6.84
6.83
6.83
6.82
6.82
6.76
6.76
6.75
6.75
6.74
6.74
6.69
6.69
6.69
6.58
6.57
6.57
6.57
6.56
6.56
6.56
6.51
6.50
6.50
6.49
6.49
6.49
6.48
5.32
4.79
4.51
4.49
4.31
4.29
3.76
3.76
3.71
3.58
3.27
3.26
3.25
3.25
3.06
3.04
OMe
O
NH
CO2Me
N
H
D13
OMe
1H NMR (700 MHz, CD2Cl2)
1.00
2.15
1.10
1.07
2.05
1.14
1.06
1.10
1.96
0.98
1.75
0.92
3.11
2.97
2.94
0.94
0.98
f1 (ppm)

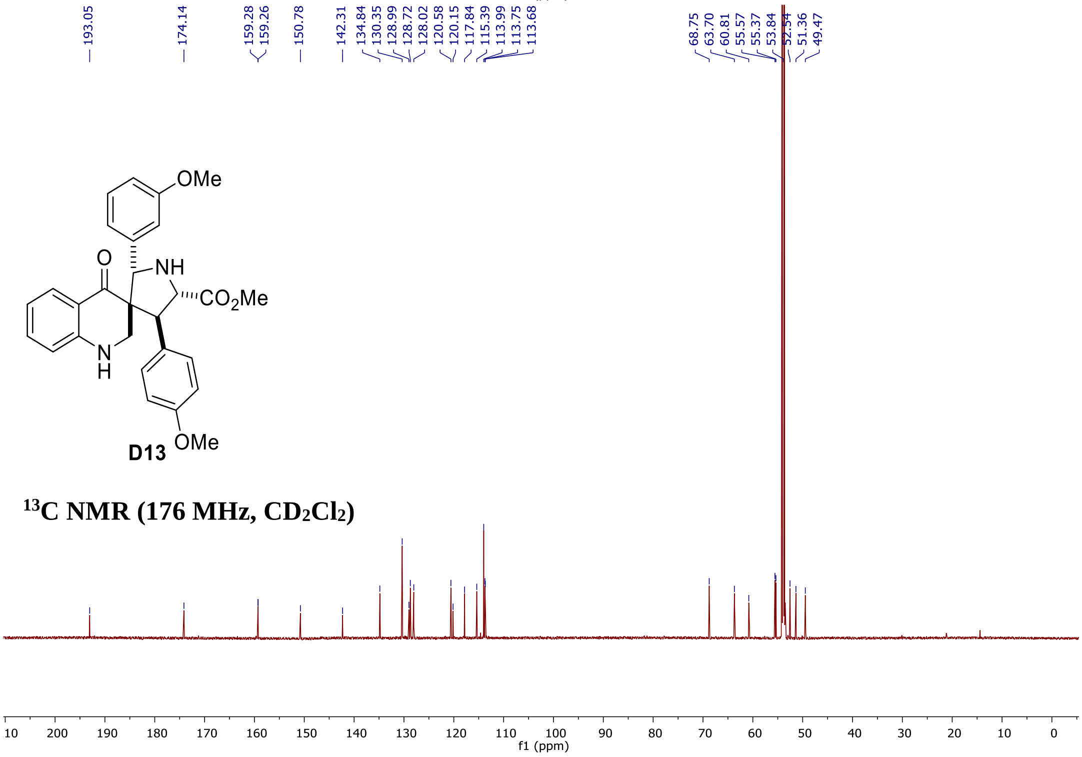
193.05
174.14
159.28
159.26
150.78
142.31
134.84
130.35
128.99
128.72
128.02
120.58
120.15
117.84
115.39
113.99
113.75
113.68
68.75
63.70
60.81
55.57
55.37
53.84
52.54
51.36
49.47
OMe
O
NH
CO2Me
N
H
D13
OMe
13C NMR (176 MHz, CD2Cl2)
f1 (ppm)

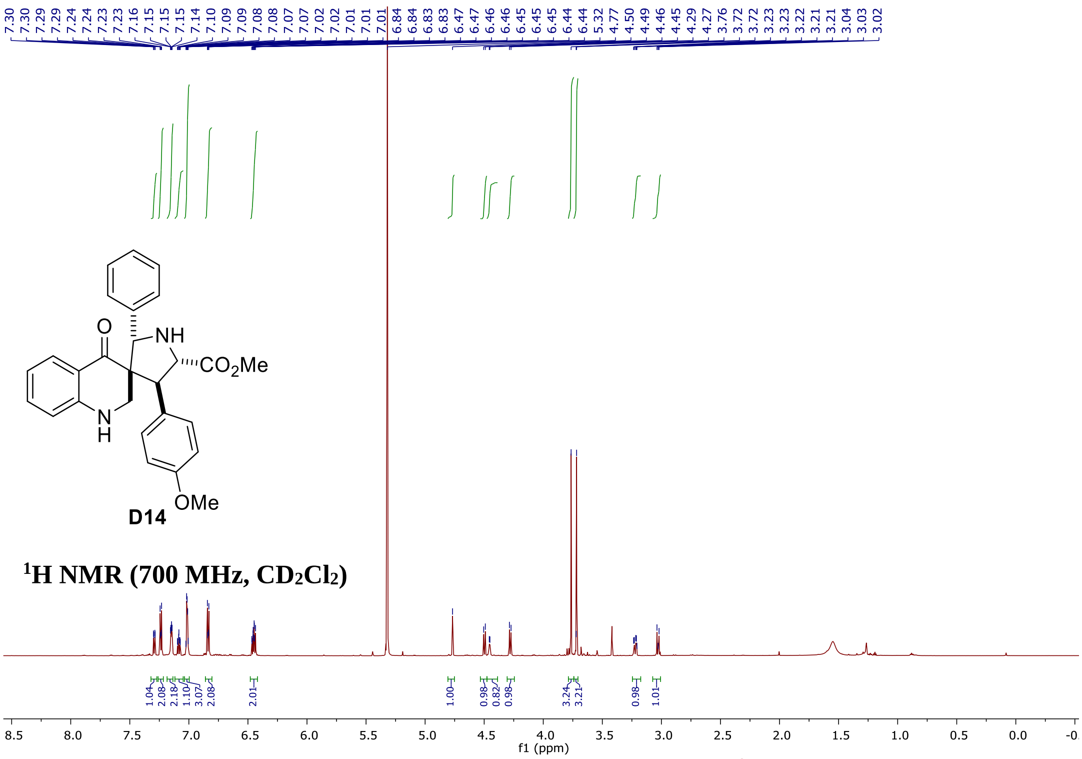
D14
1H NMR (700 MHz, CD2Cl2)
f1 (ppm)

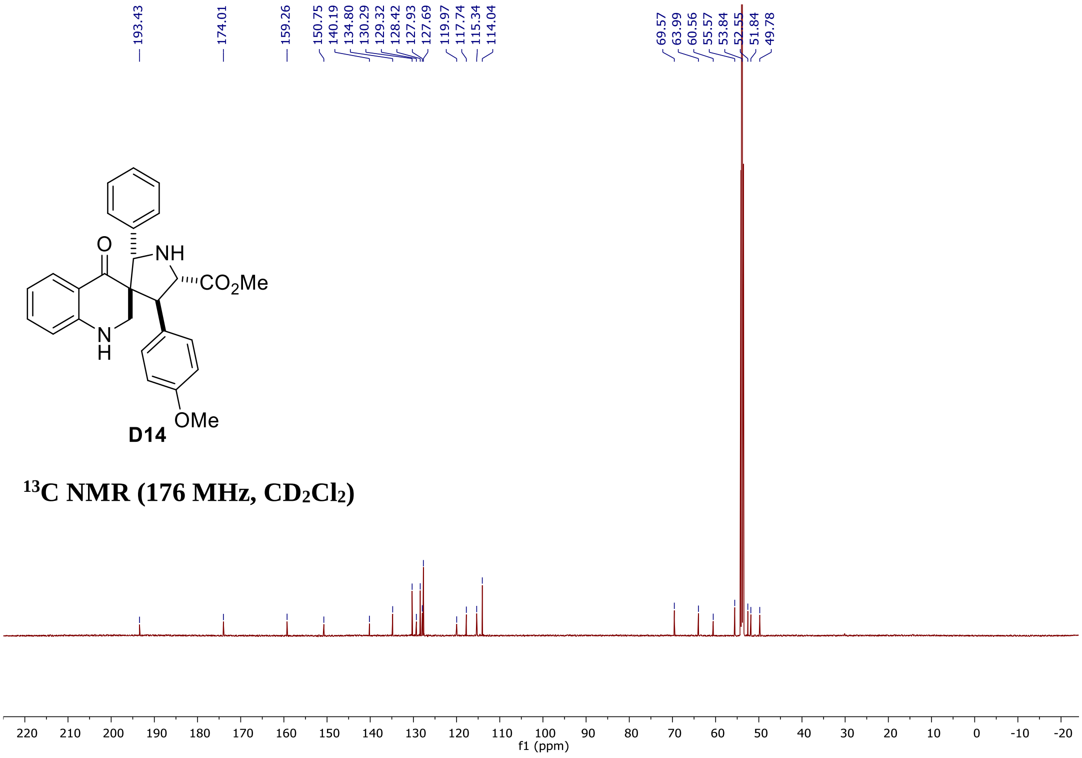
D14
13C NMR (176 MHz, CD2Cl2)
f1 (ppm)

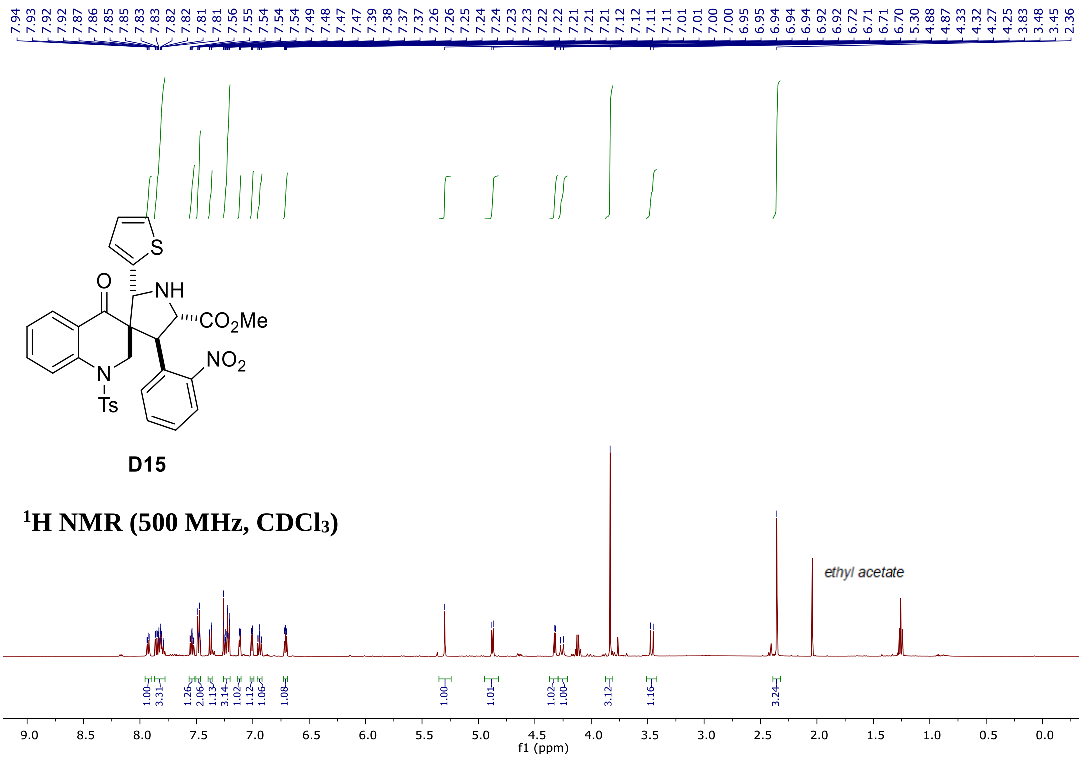

D15

^{1}H NMR (500 MHz, CDCl$_3$)

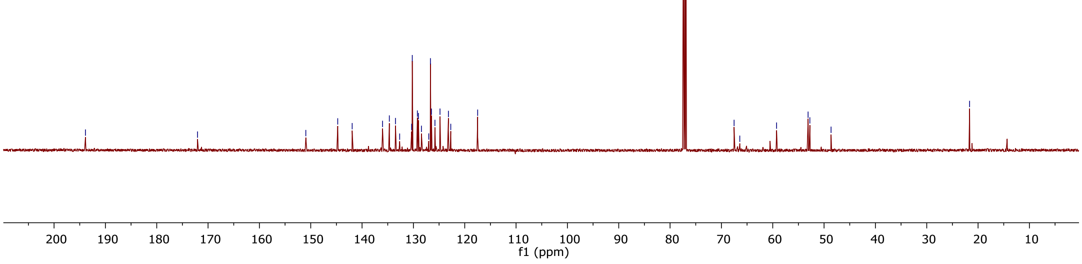

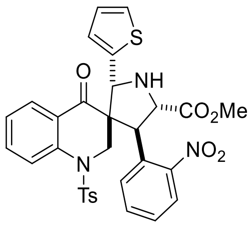

D15

^{13}C NMR (126 MHz, CDCl$_3$)

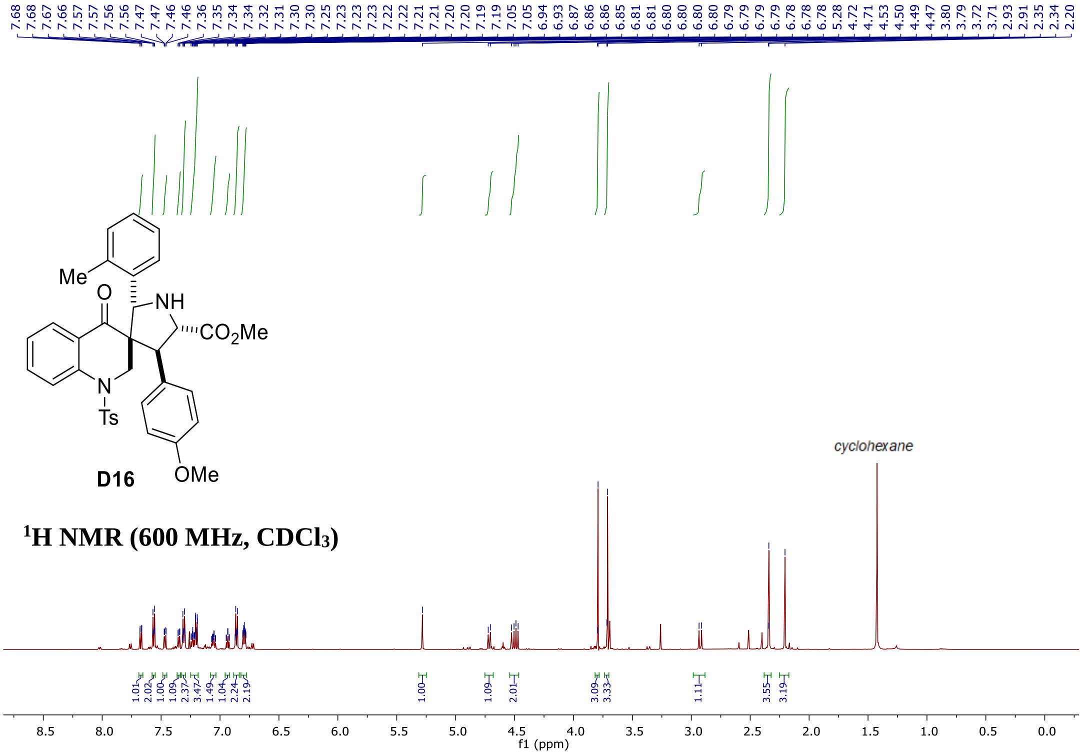

D16

^{1}H NMR (600 MHz, CDCl3)

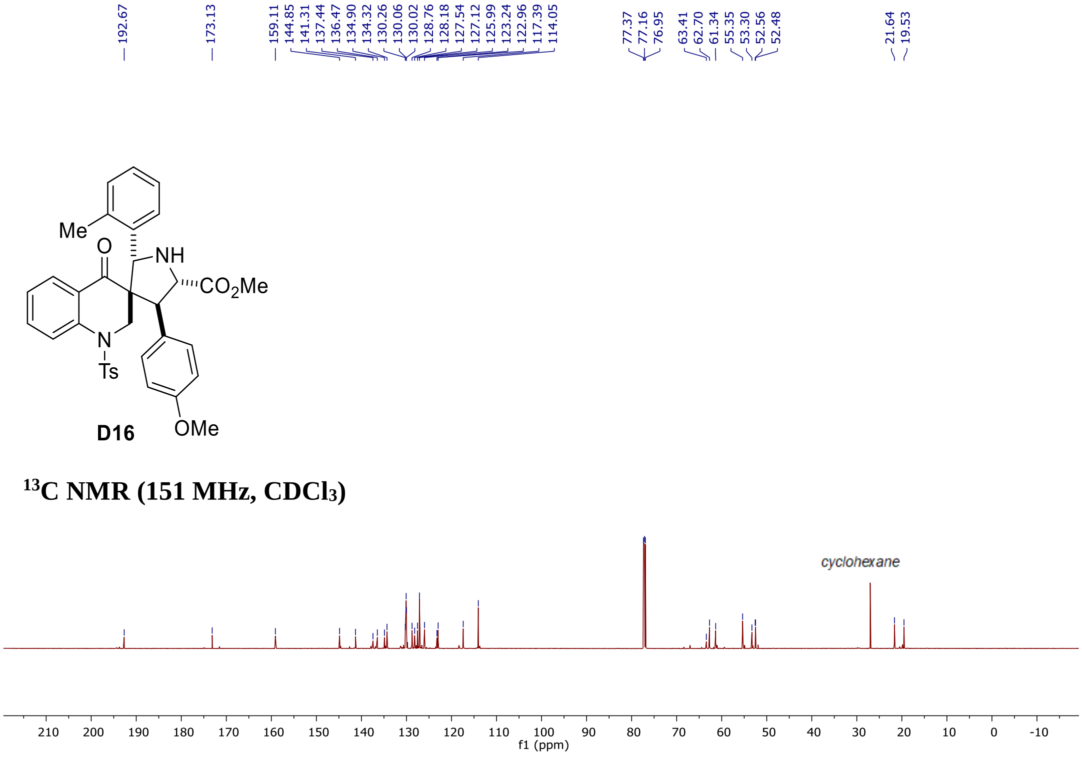

D16

^{13}C NMR (151 MHz, CDCl3)

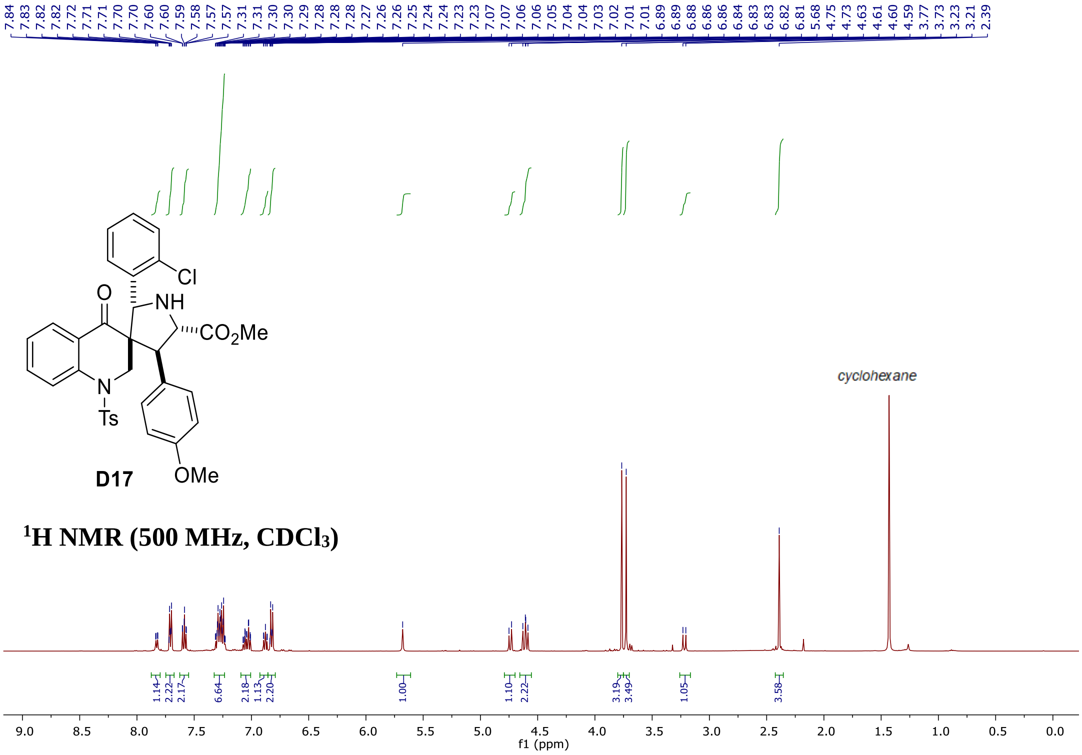
D17
OMe
^{1}H NMR (500 MHz, CDCl3)
cyclohexane
f1 (ppm)

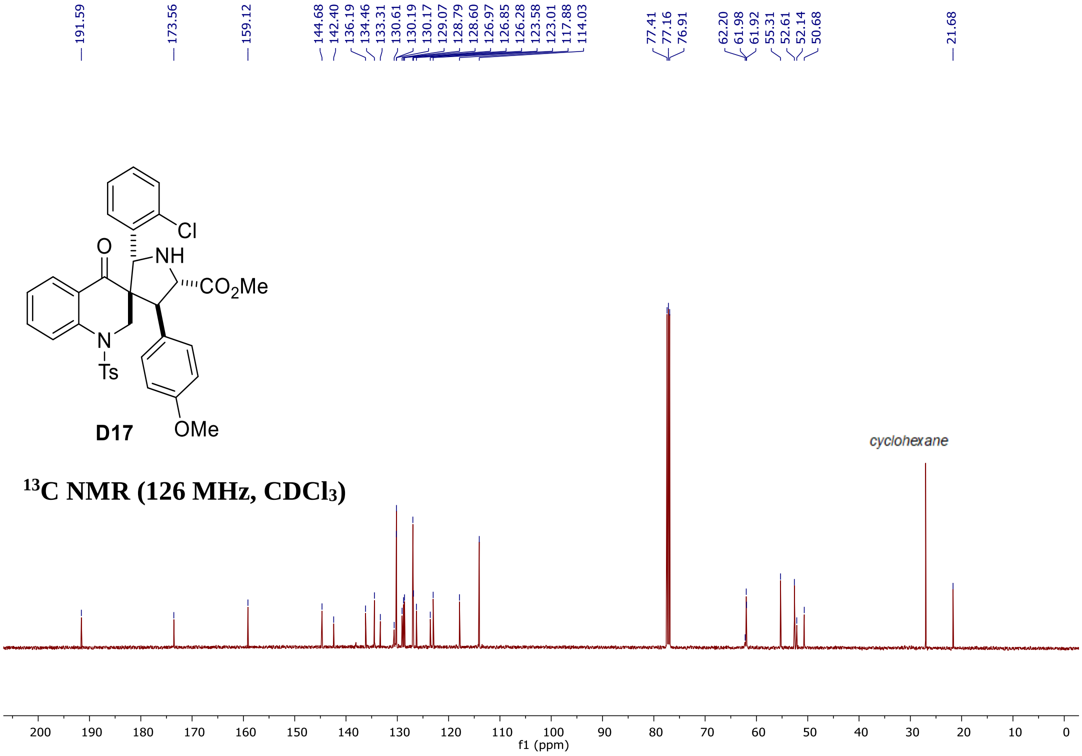
D17
OMe
^{13}C NMR (126 MHz, CDCl3)
cyclohexane
f1 (ppm)

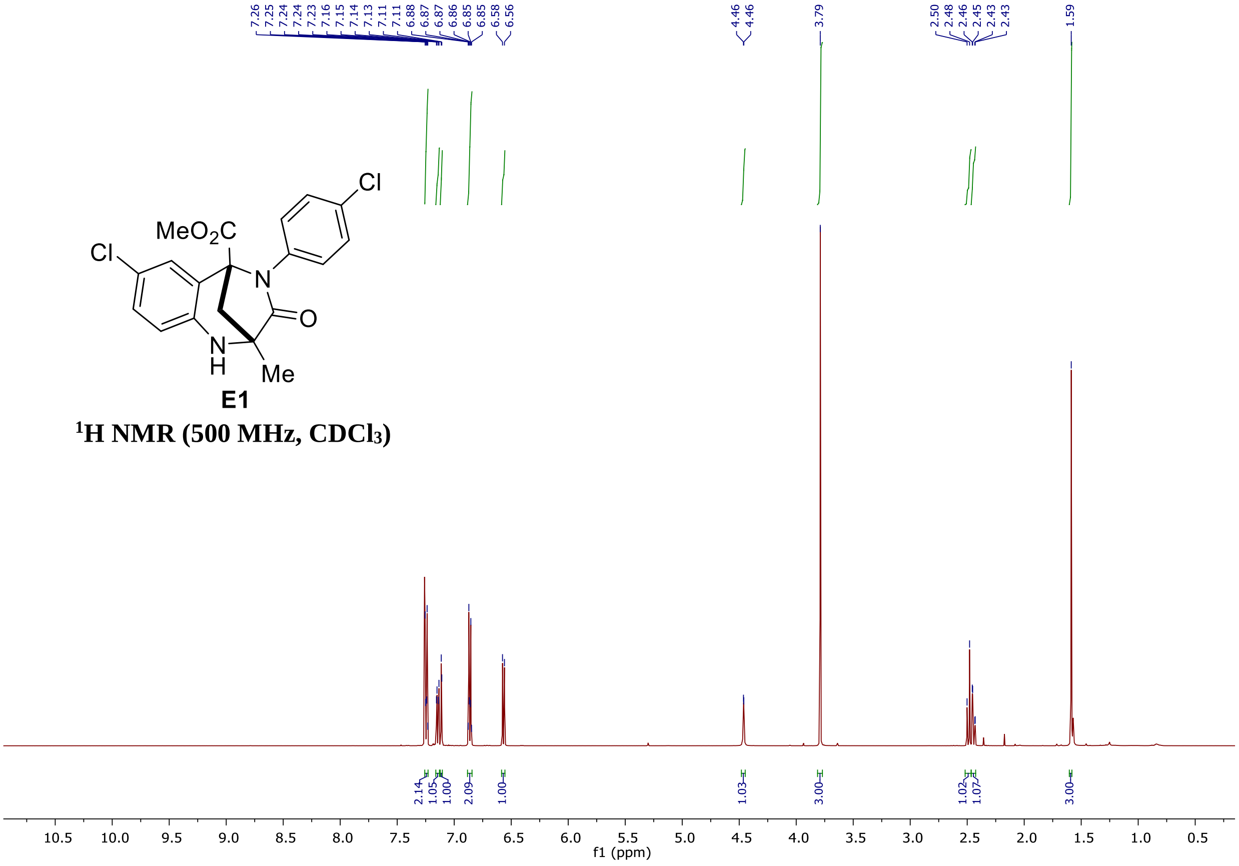

E1

^{1}H NMR (500 MHz, $CDCl_3$)

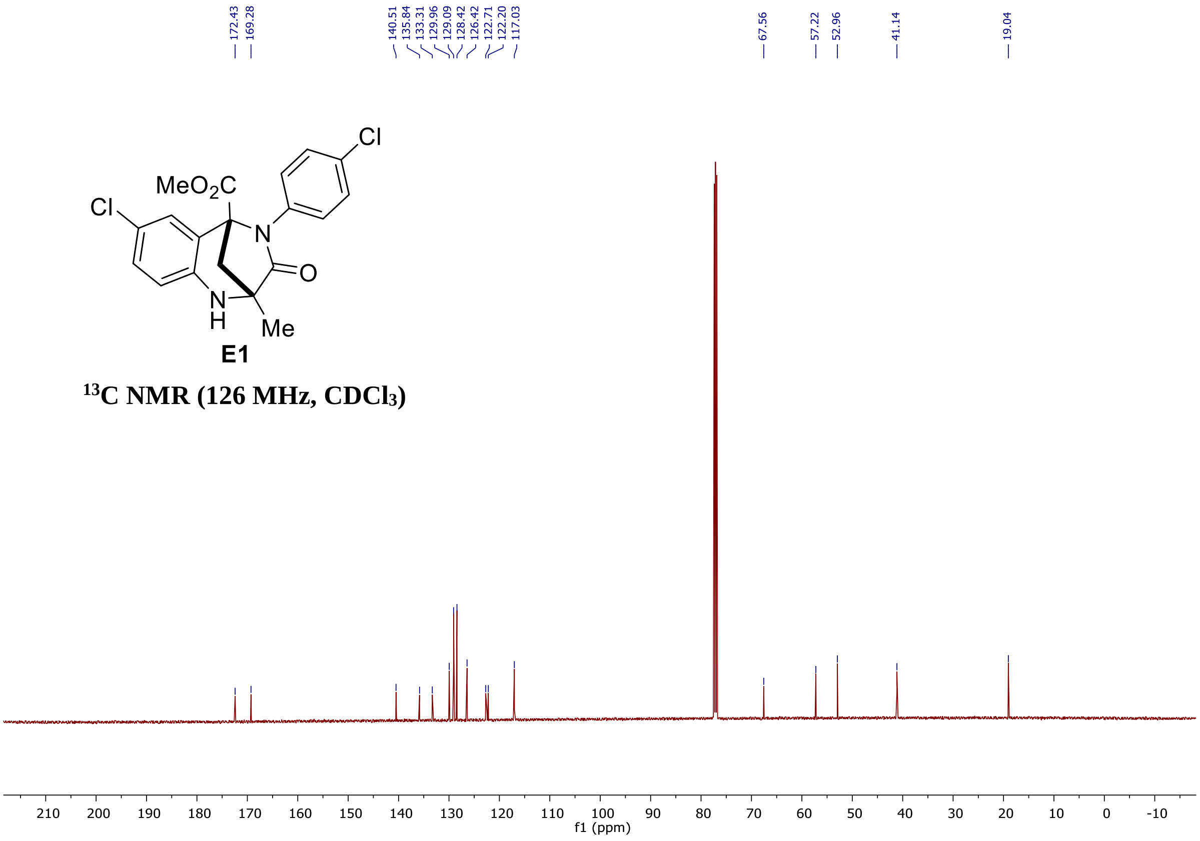

E1

^{13}C NMR (126 MHz, $CDCl_3$)

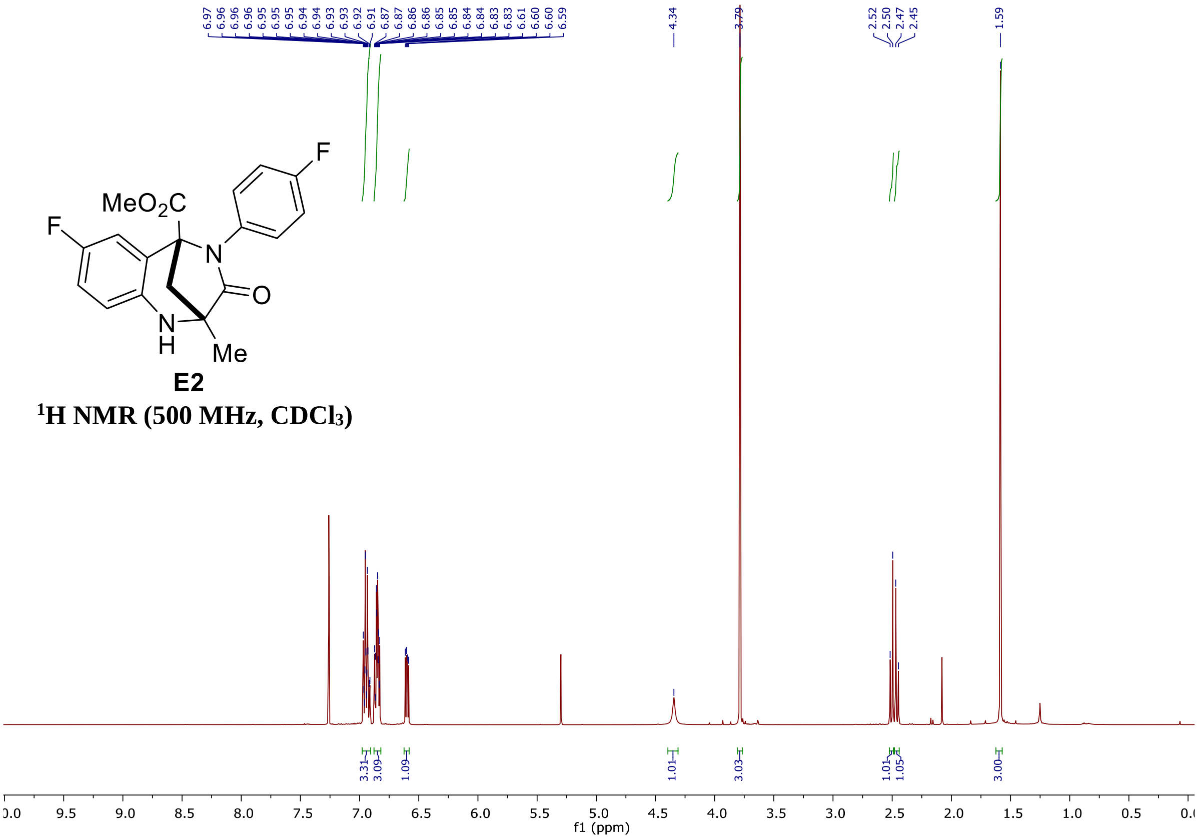
6.97 6.96 6.96 6.96 6.95 6.95 6.95 6.94 6.94 6.93 6.93 6.92 6.91 6.87 6.87 6.86 6.86 6.85 6.85 6.84 6.84 6.83 6.83 6.61 6.60 6.60 6.59
4.34
3.79
2.52 2.50 2.47 2.45
1.59
MeO2C
F
N
O
NH
Me
E2
1H NMR (500 MHz, CDCl3)
3.31 3.09 1.09 1.01 3.03 1.01 1.05 3.00
f1 (ppm)

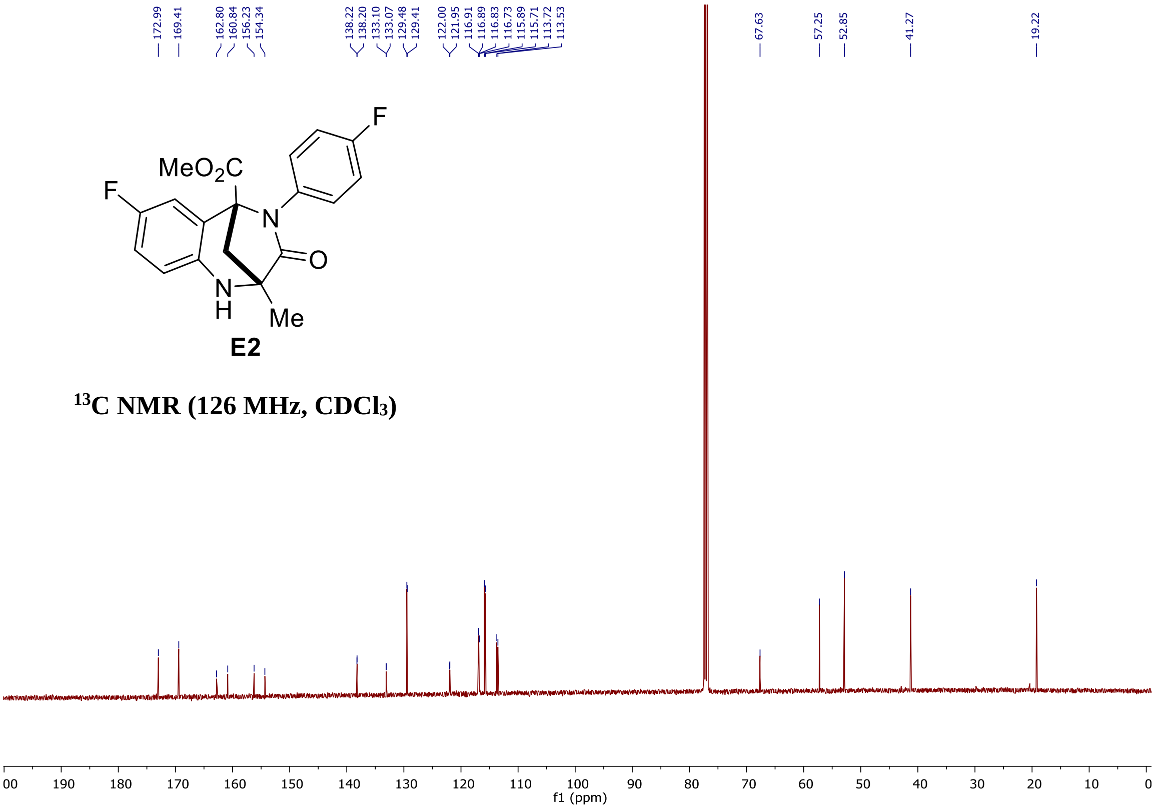
172.99 169.41 162.80 160.84 156.23 154.34
138.22 138.20 133.10 133.07 129.48 129.41 122.00 121.95 116.91 116.89 116.83 116.73 115.89 115.71 113.72 113.53
67.63 57.25 52.85 41.27 19.22
MeO2C
F
N
O
NH
Me
E2
13C NMR (126 MHz, CDCl3)
f1 (ppm)

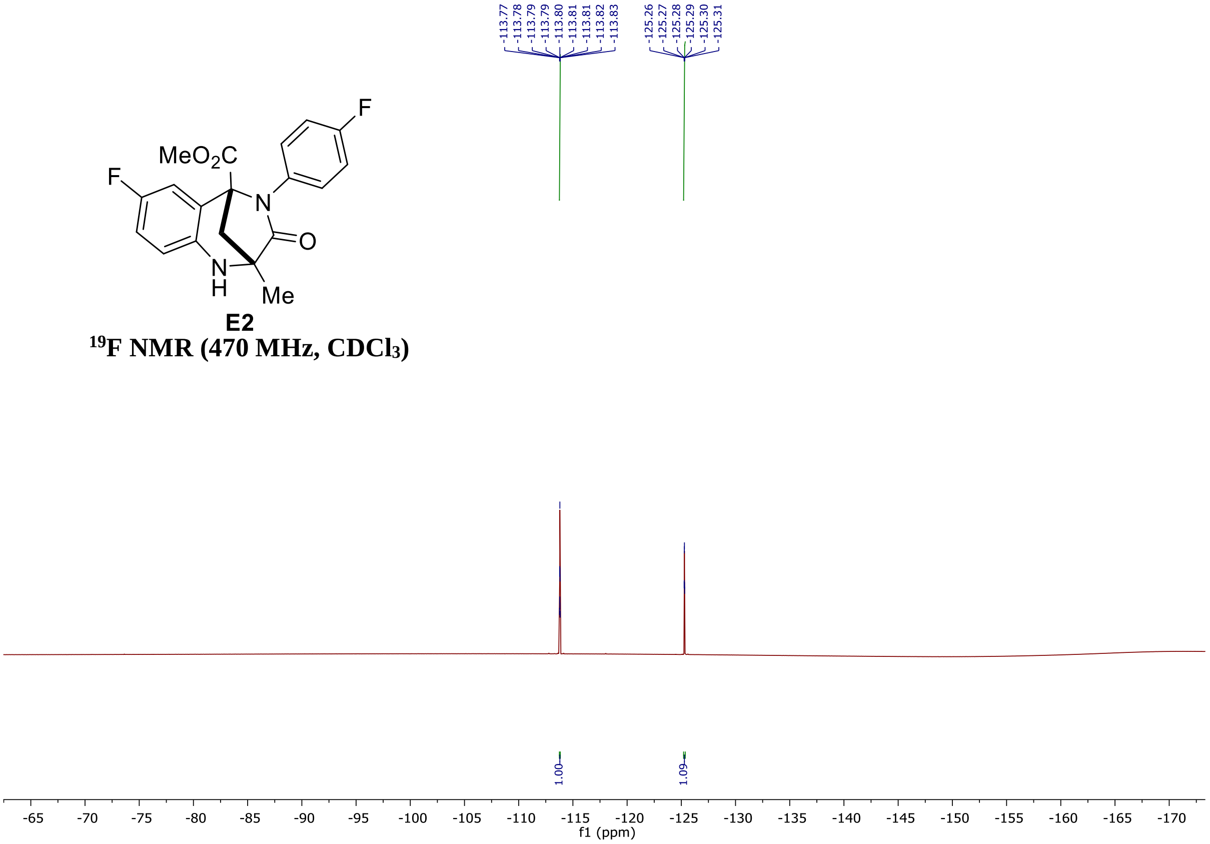
MeO2C
F
F
N
O
N
H
Me
E2
19F NMR (470 MHz, CDCl3)
f1 (ppm)

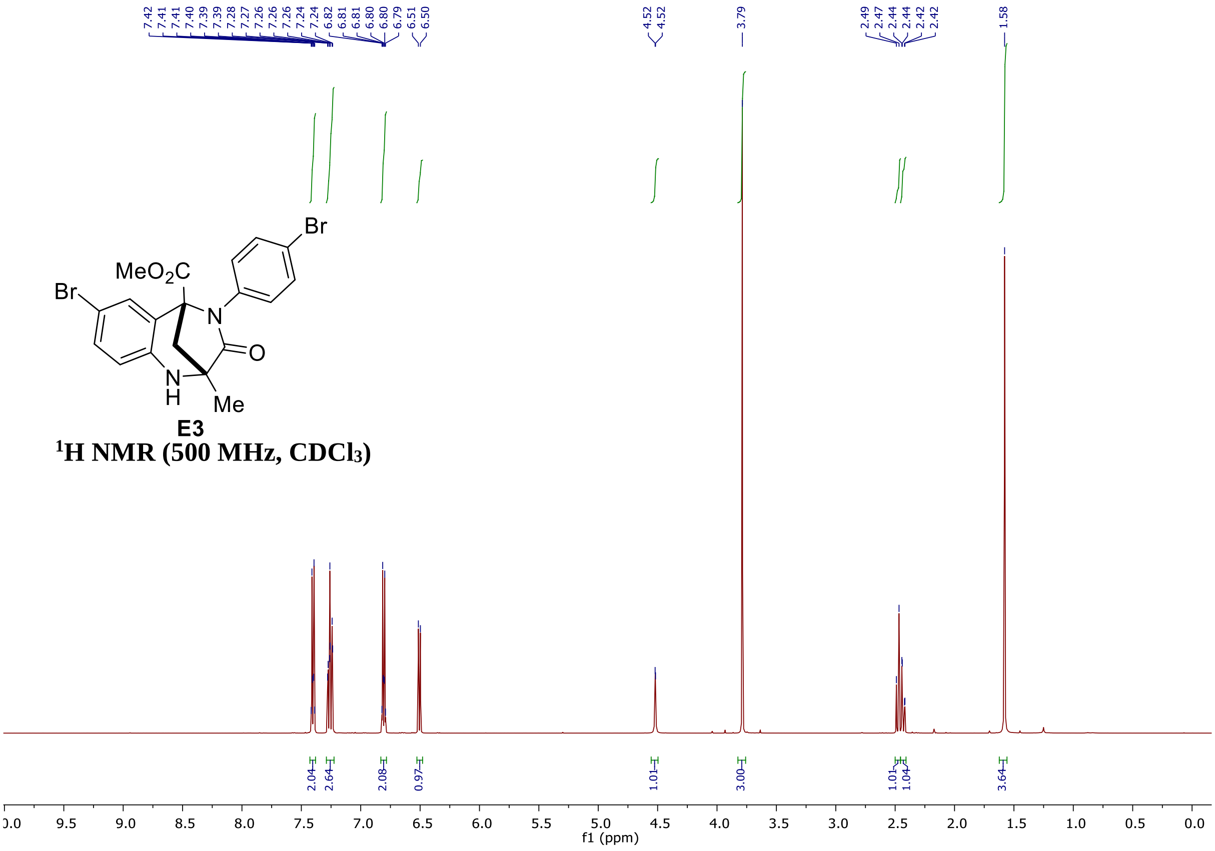
MeO2C
Br
Br
N
O
N
H
Me
E3
1H NMR (500 MHz, CDCl3)
f1 (ppm)

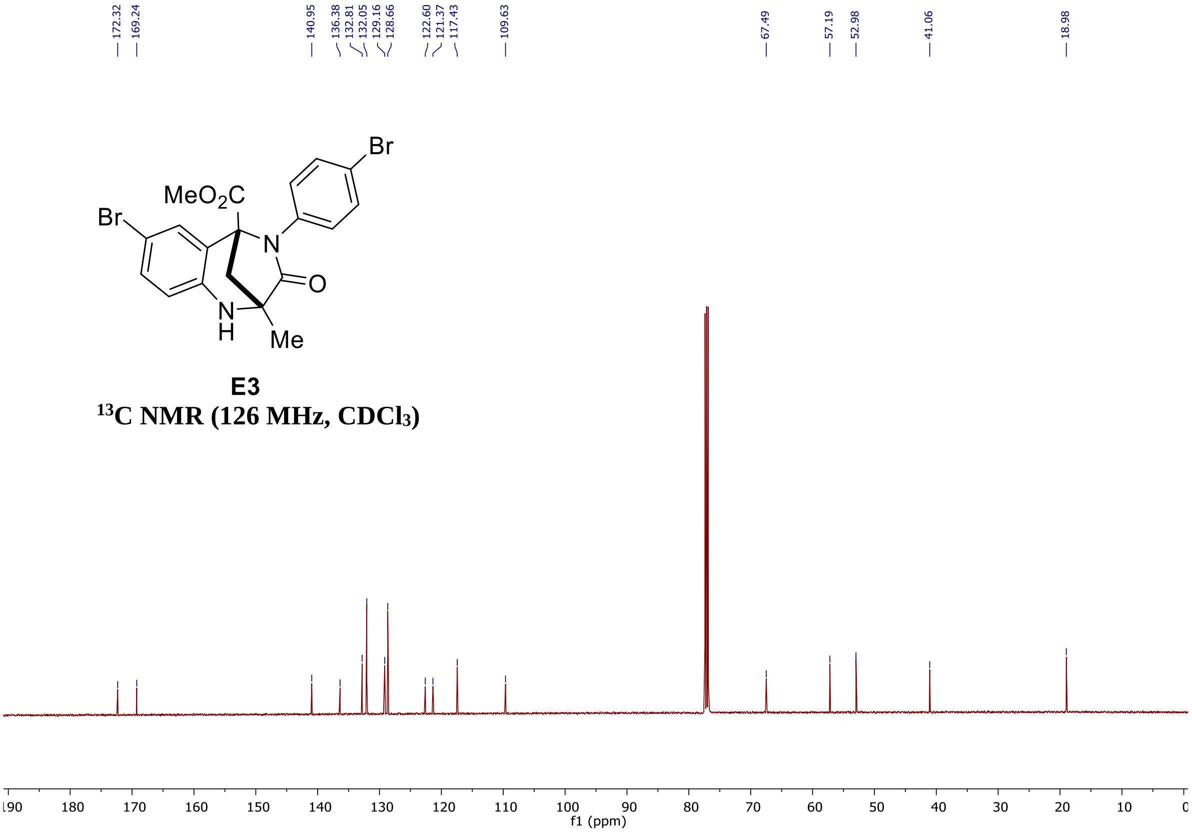

E3

^{13}C NMR (126 MHz, CDCl$_3$)

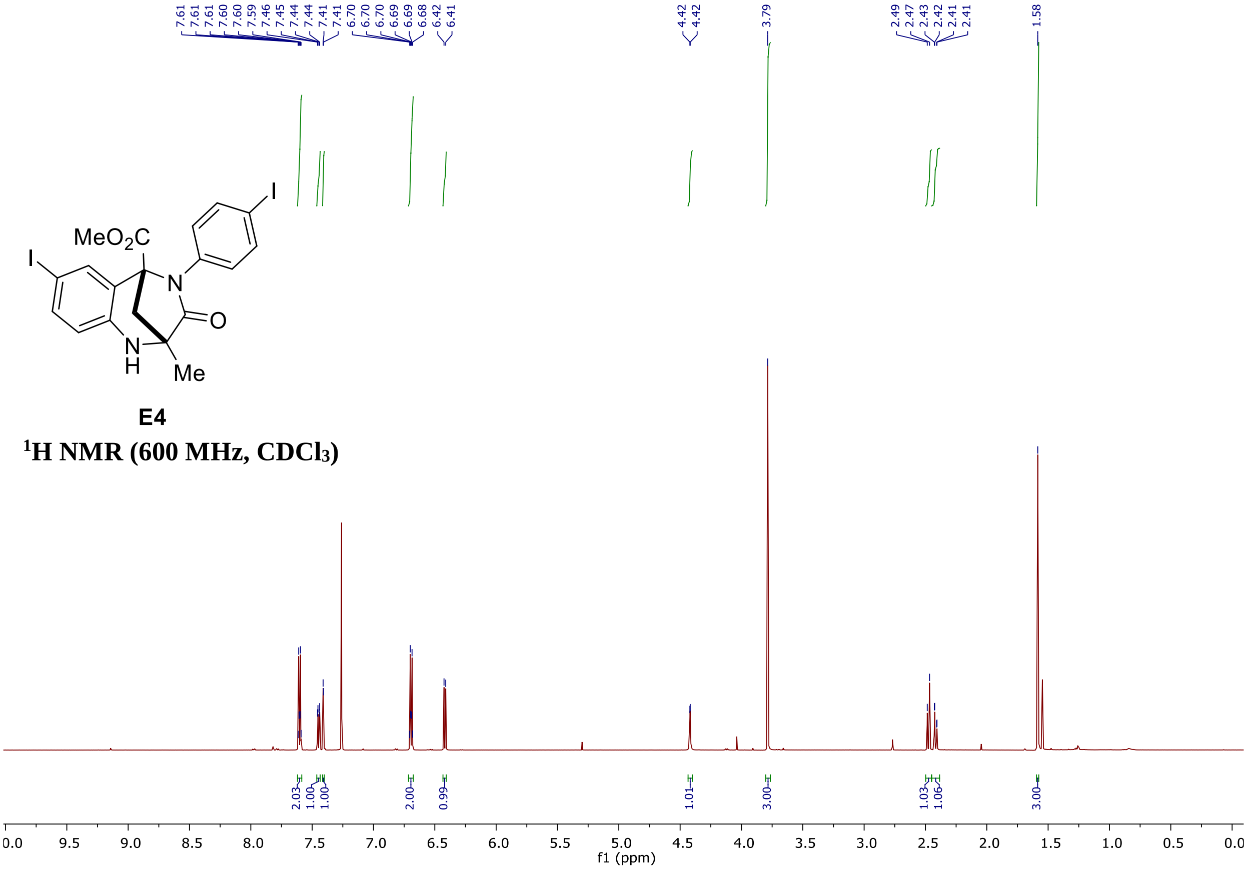

E4

^{1}H NMR (600 MHz, CDCl$_3$)

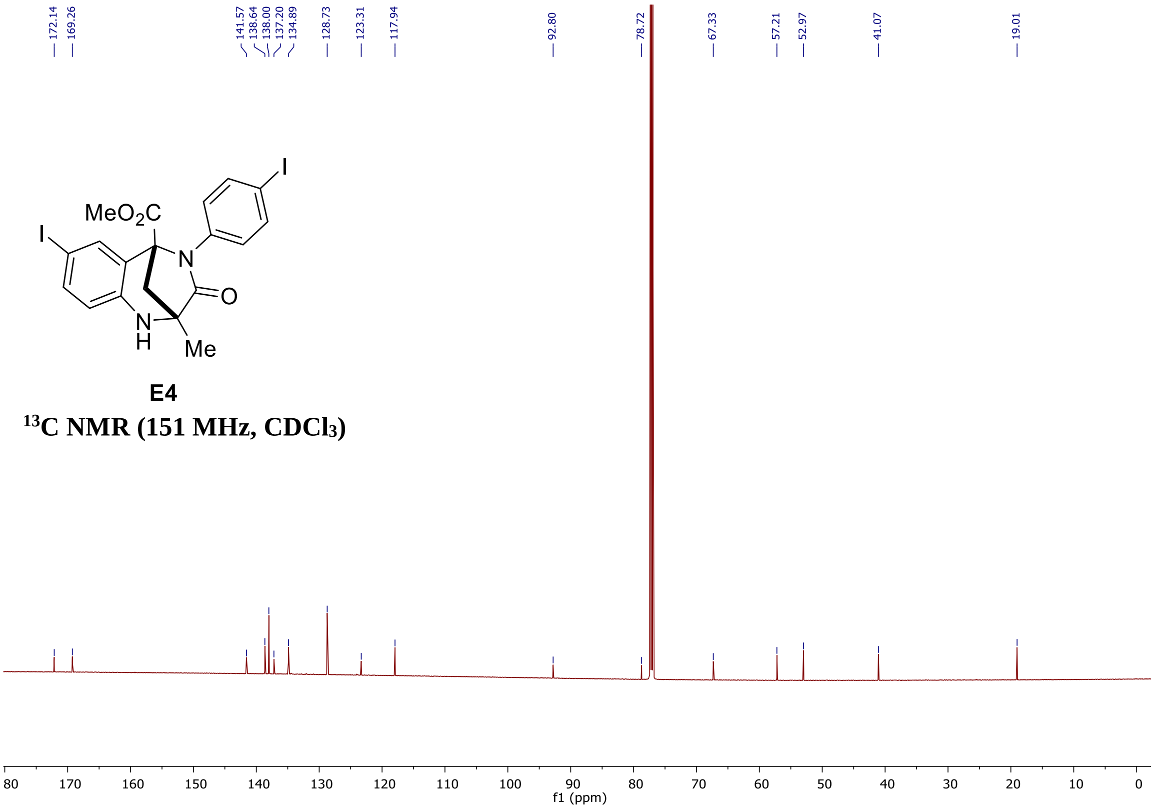

E4

^{13}C NMR (151 MHz, CDCl$_3$)

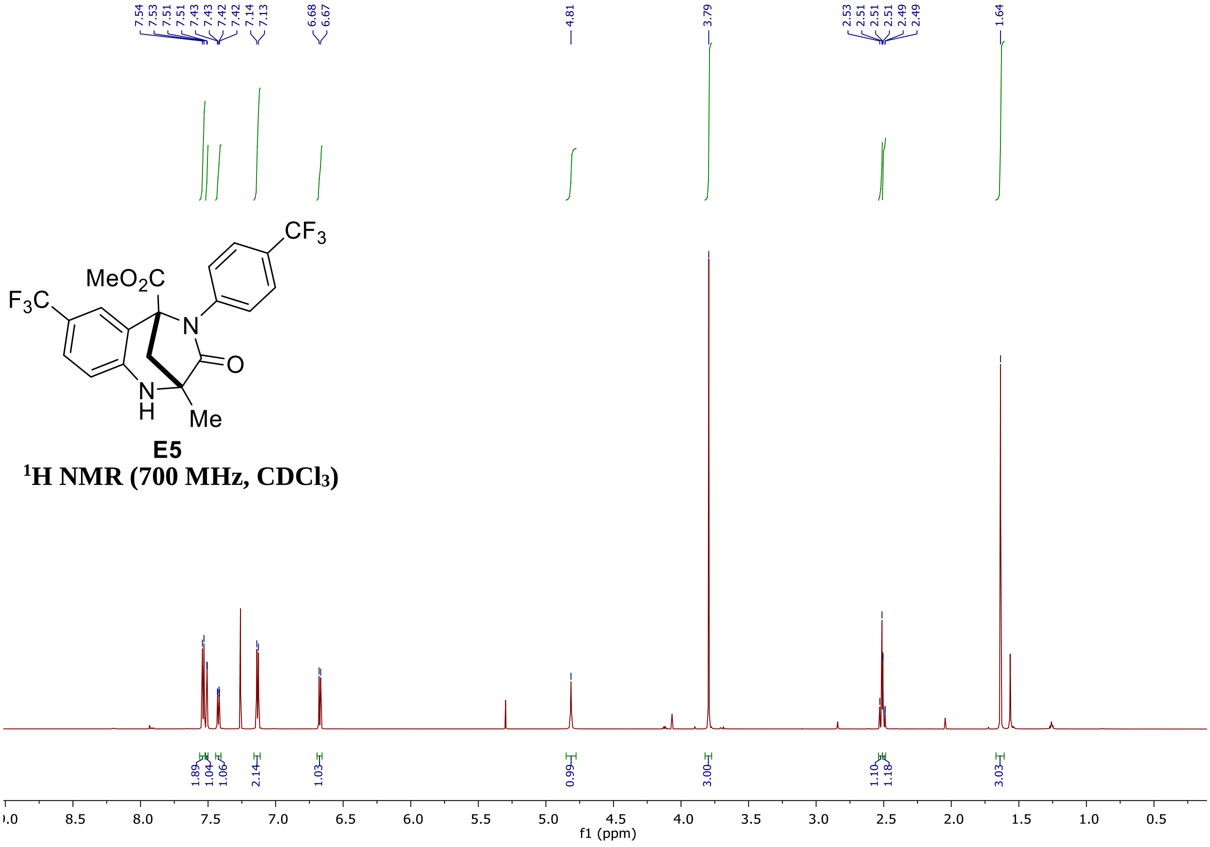

E5

^{1}H NMR (700 MHz, CDCl$_3$)

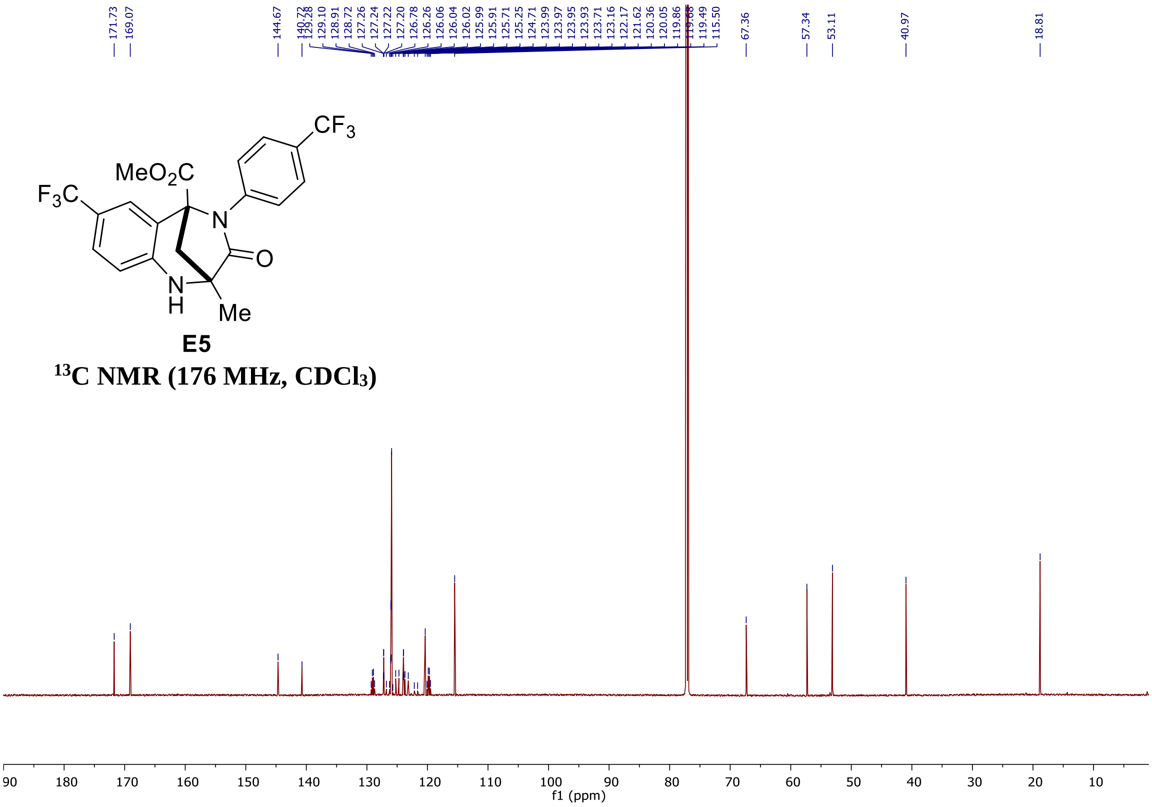
171.73
169.07
144.67
140.72
129.28
129.10
128.91
128.72
127.26
127.24
127.22
127.20
126.78
126.26
126.06
126.04
126.02
125.99
125.91
125.71
125.25
124.71
123.99
123.97
123.95
123.93
123.71
123.16
122.17
121.62
120.36
120.05
119.86
119.68
119.49
115.50
67.36
57.34
53.11
40.97
18.81
CF3
MeO2C
F3C
N
O
N
H
Me
E5
13C NMR (176 MHz, CDCl3)
f1 (ppm)

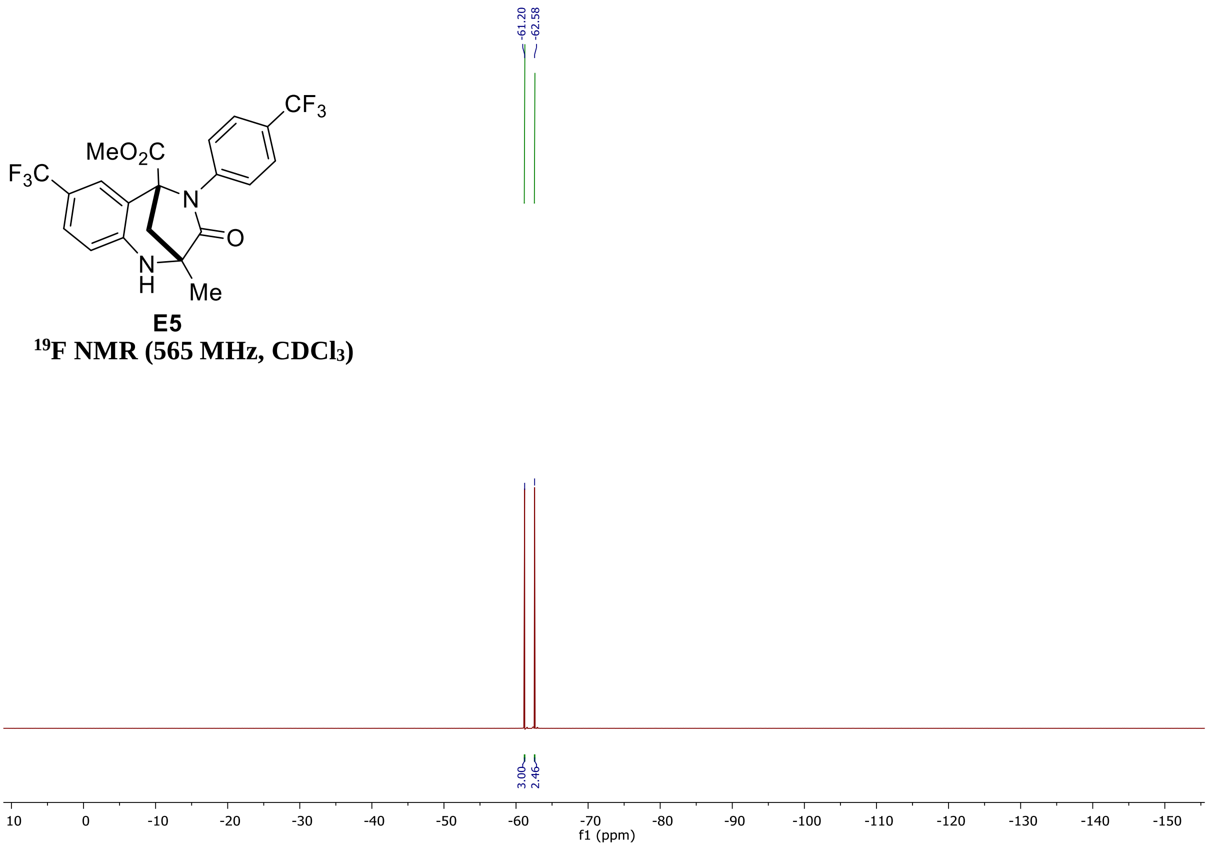
-61.20
-62.58
CF3
MeO2C
F3C
N
O
N
H
Me
E5
19F NMR (565 MHz, CDCl3)
3.00
2.46
f1 (ppm)

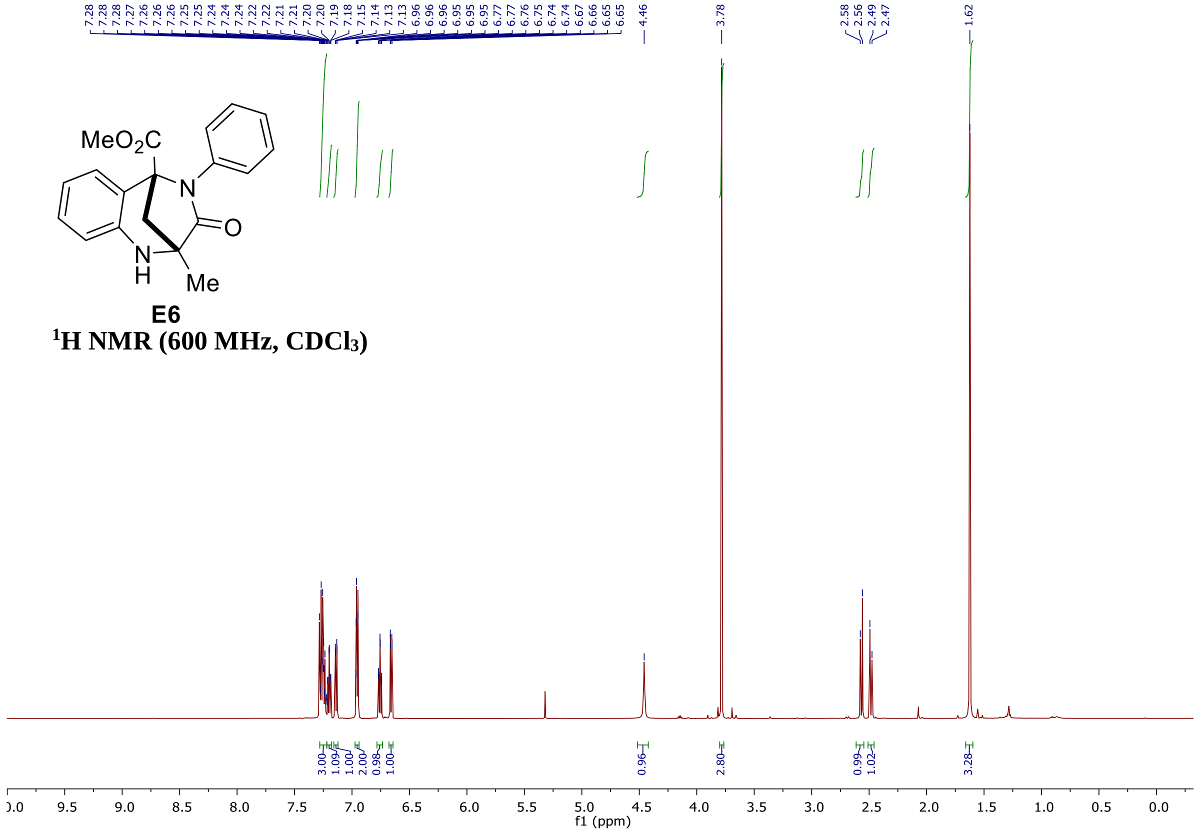
7.28 7.28 7.28 7.27 7.26 7.26 7.26 7.25 7.25 7.24 7.24 7.24 7.22 7.22 7.21 7.21 7.20 7.20 7.19 7.18 7.15 7.14 7.13 7.13 6.96 6.96 6.96 6.95 6.95 6.95 6.77 6.77 6.76 6.75 6.74 6.74 6.67 6.66 6.65 6.65
4.46
3.78
2.58 2.56 2.49 2.47
1.62
MeO2C
N
O
N
H
Me
E6
1H NMR (600 MHz, CDCl3)
3.00 1.09 1.00 2.00 0.98 1.00
0.96
2.80
0.99 1.02
3.28
f1 (ppm)

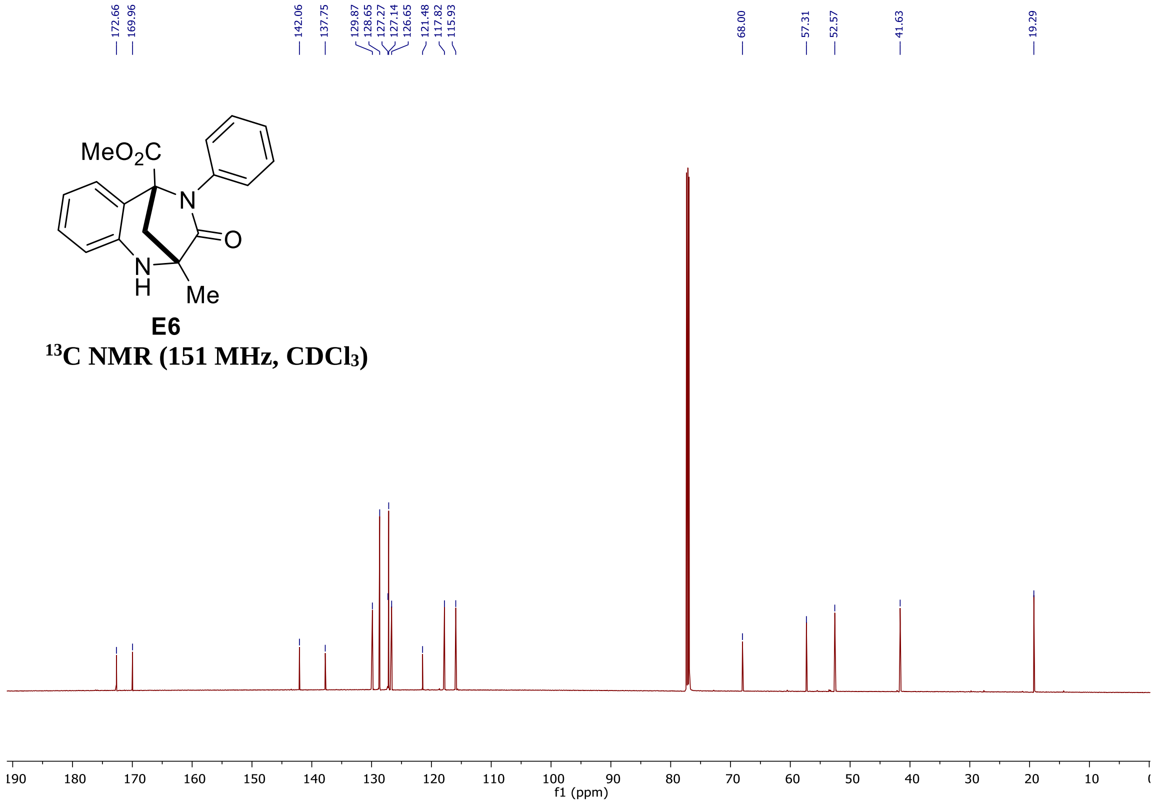
172.66 169.96
142.06 137.75
129.87 128.65 127.27 127.14 126.65 121.48 117.82 115.93
68.00
57.31 52.57
41.63
19.29
MeO2C
N
O
N
H
Me
E6
13C NMR (151 MHz, CDCl3)
f1 (ppm)

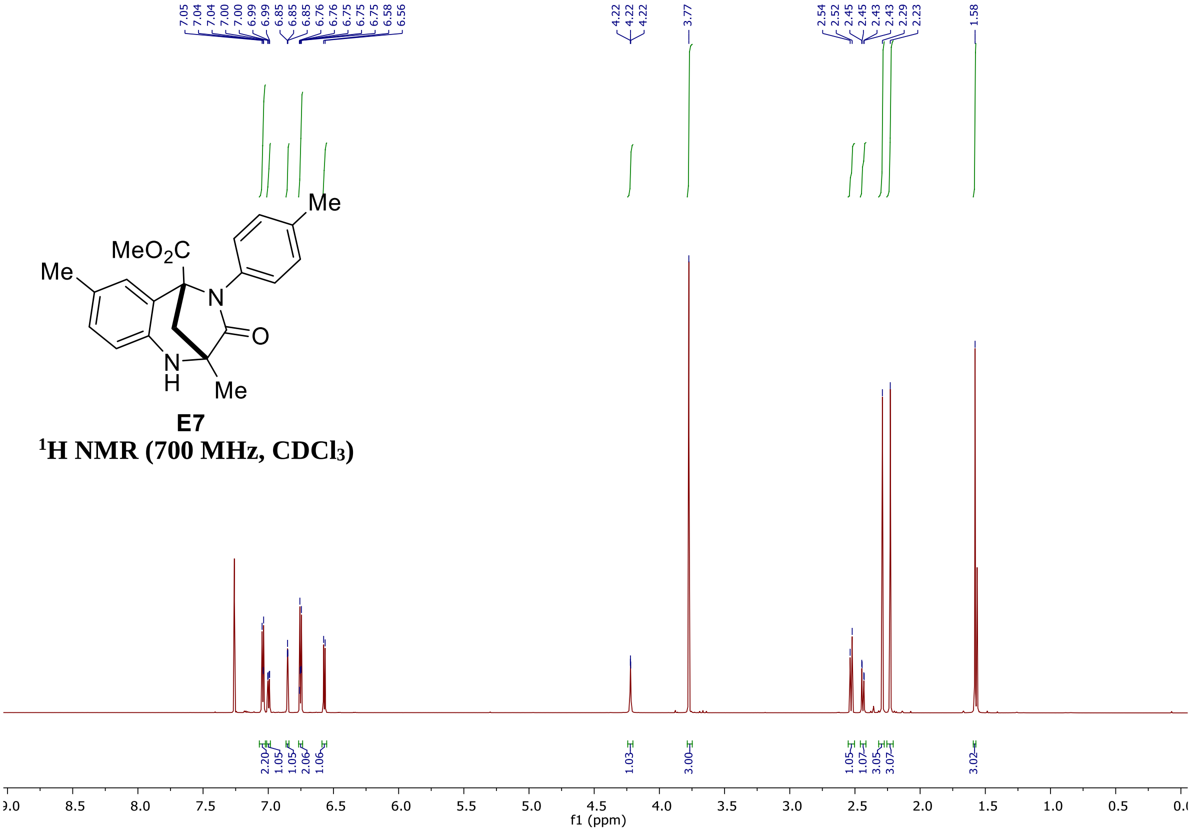

E7

^{1}H NMR (700 MHz, CDCl3)

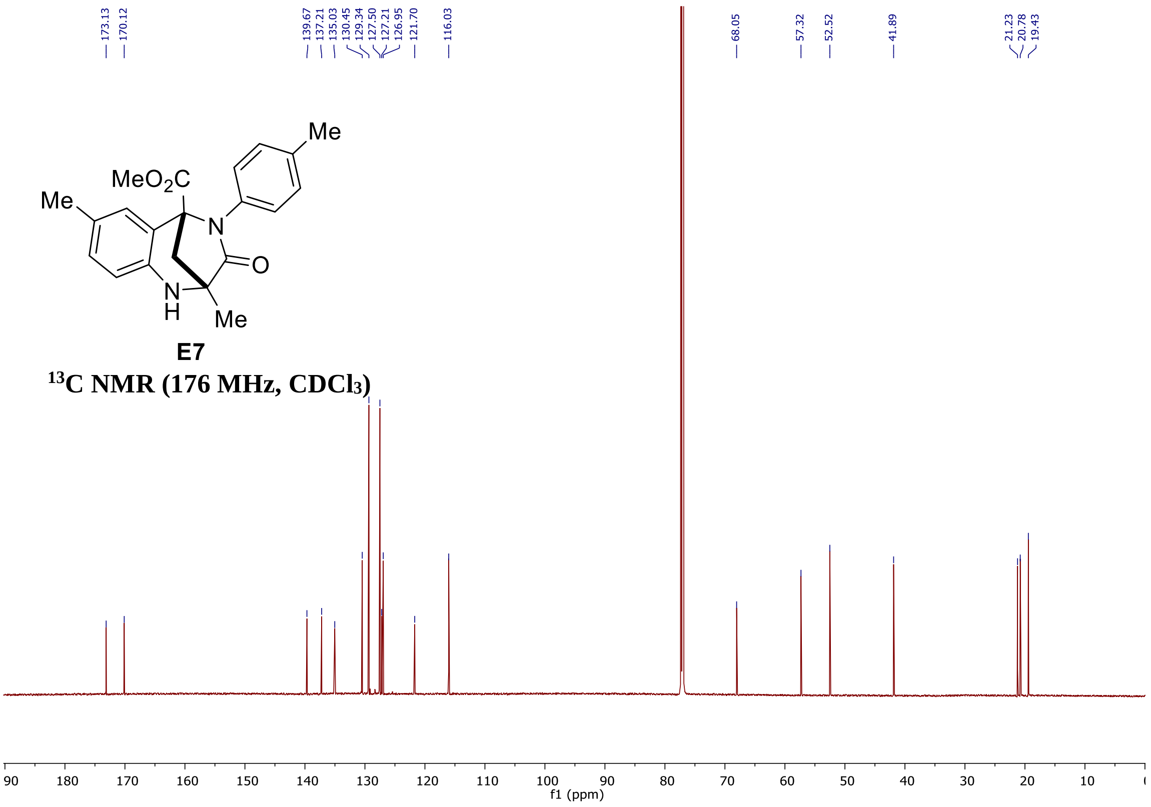

E7

^{13}C NMR (176 MHz, CDCl3)

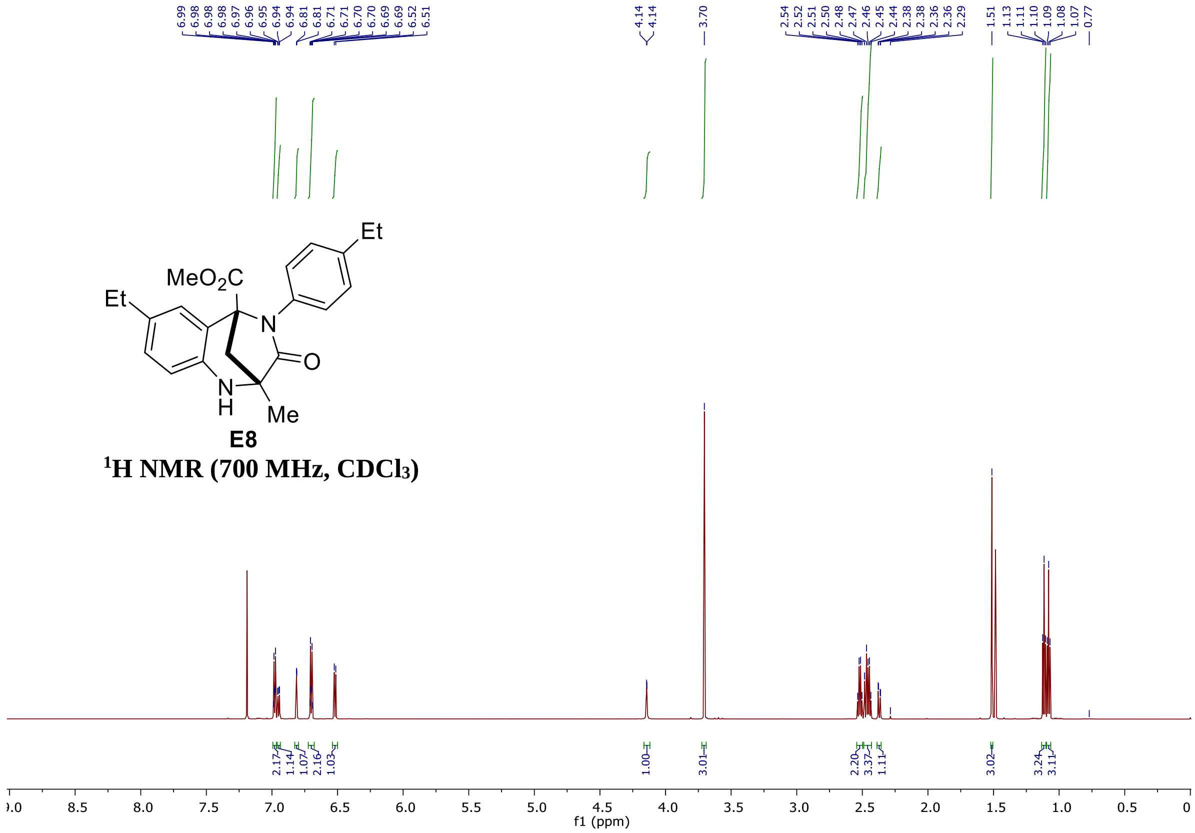

Et
MeO2C
Et
N
O
N
H
Me
E8
1H NMR (700 MHz, CDCl3)
f1 (ppm)

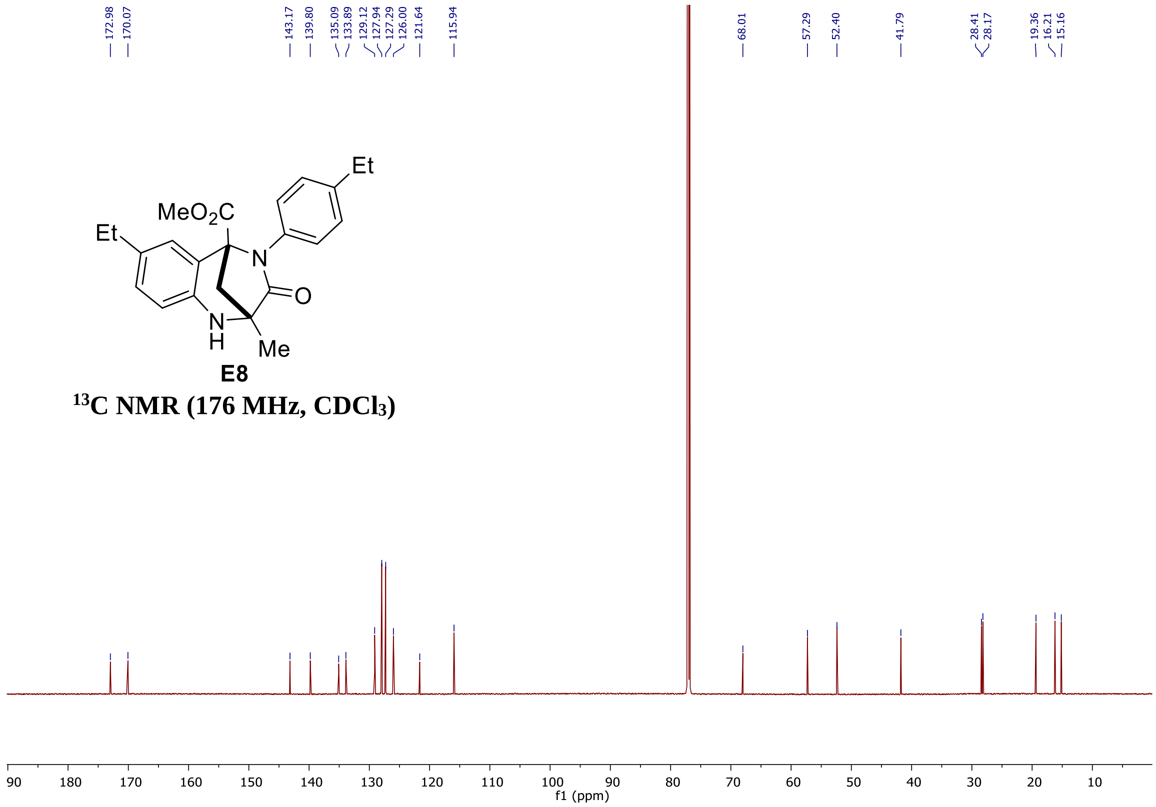

Et
MeO2C
Et
N
O
N
H
Me
E8
13C NMR (176 MHz, CDCl3)
f1 (ppm)

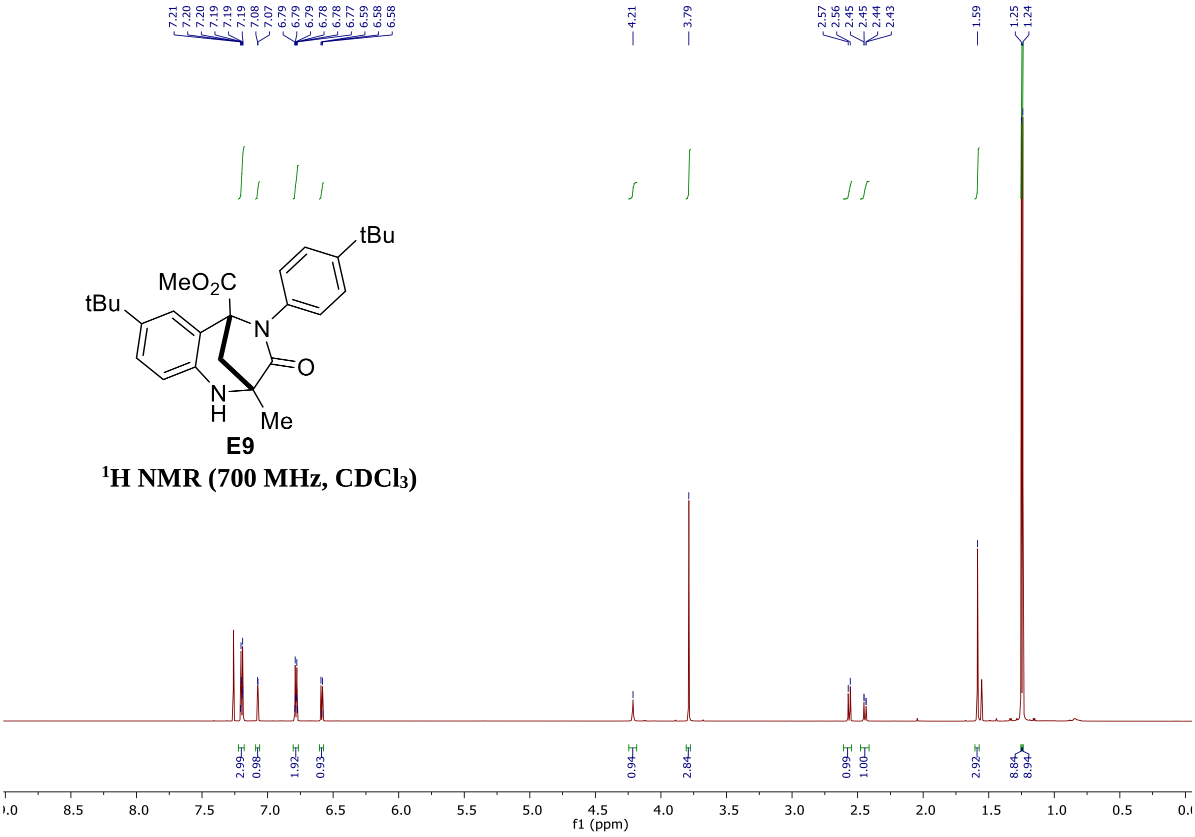
7.21 7.20 7.20 7.19 7.19 7.19 7.08 7.07 6.79 6.79 6.79 6.78 6.78 6.77 6.59 6.58 6.58
4.21
3.79
2.57 2.56 2.45 2.45 2.44 2.43
1.59
1.25 1.24
tBu
MeO2C
tBu
N
O
N
H
Me
E9
1H NMR (700 MHz, CDCl3)
2.99 0.98 1.92 0.93 0.94 2.84 0.99 1.00 2.92 8.84 8.94
f1 (ppm)

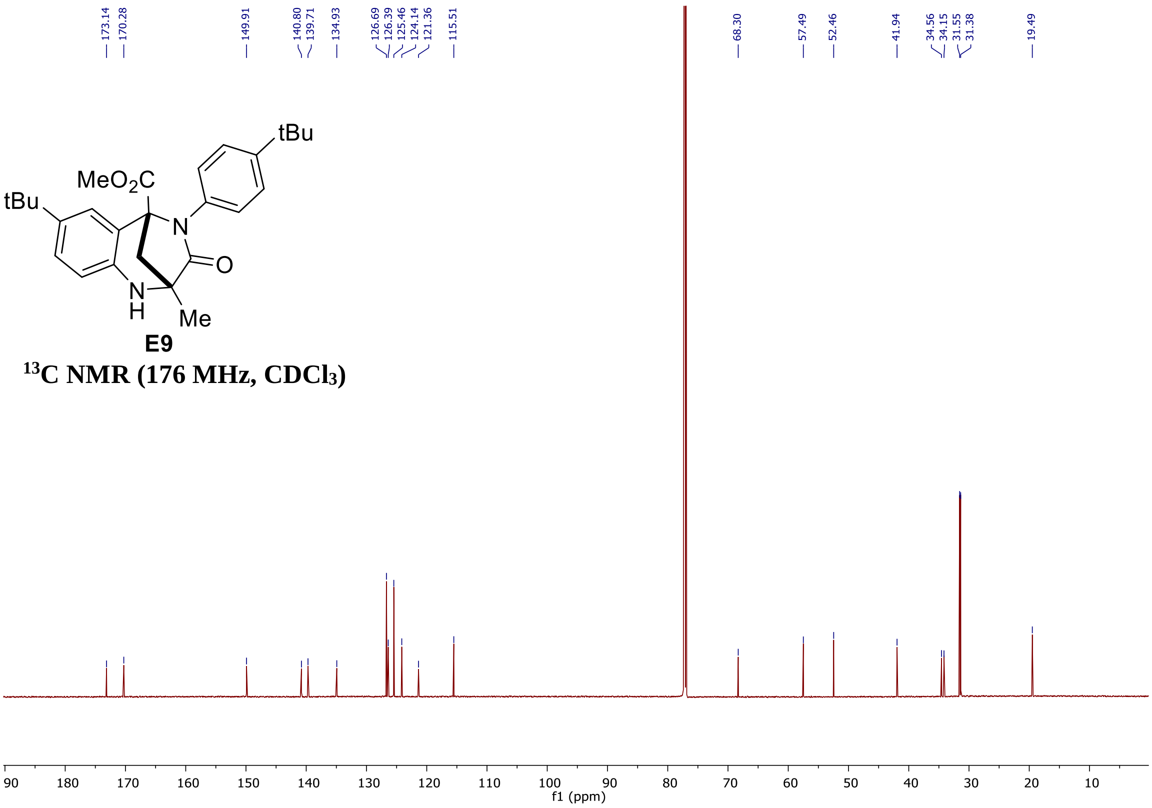
173.14 170.28
149.91
140.80 139.71
134.93
126.69 126.39 125.46 124.14 121.36
115.51
68.30
57.49
52.46
41.94
34.56 34.15 31.55 31.38
19.49
tBu
MeO2C
tBu
N
O
N
H
Me
E9
13C NMR (176 MHz, CDCl3)
f1 (ppm)

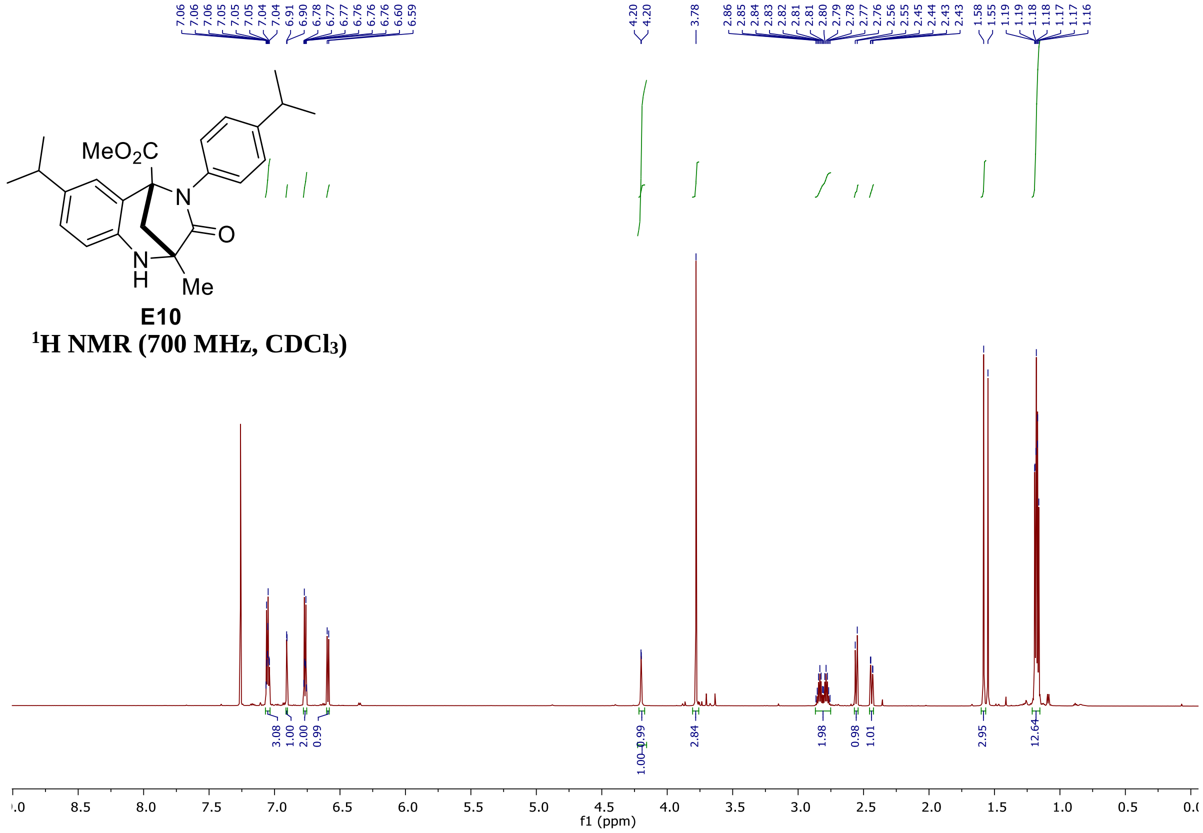

E10

^{1}H NMR (700 MHz, $CDCl_3$)

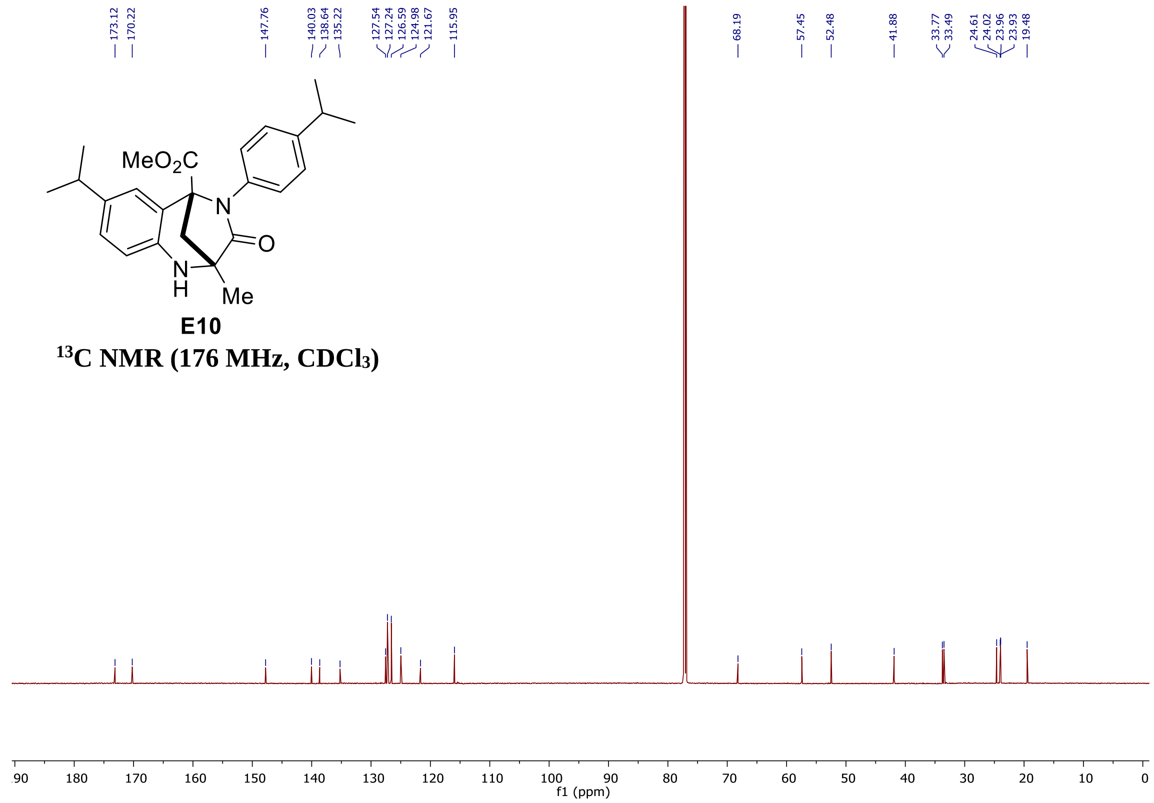

E10

^{13}C NMR (176 MHz, $CDCl_3$)

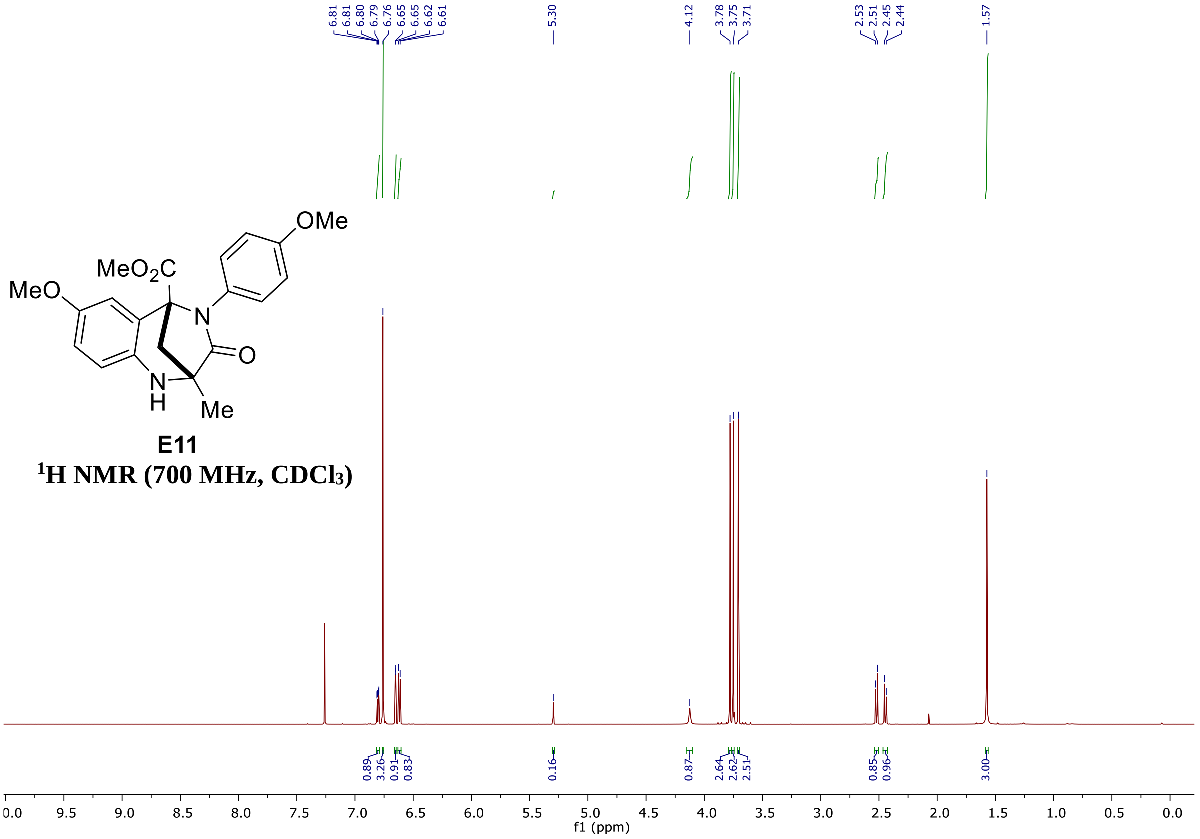

E11

^{1}H NMR (700 MHz, $CDCl_3$)

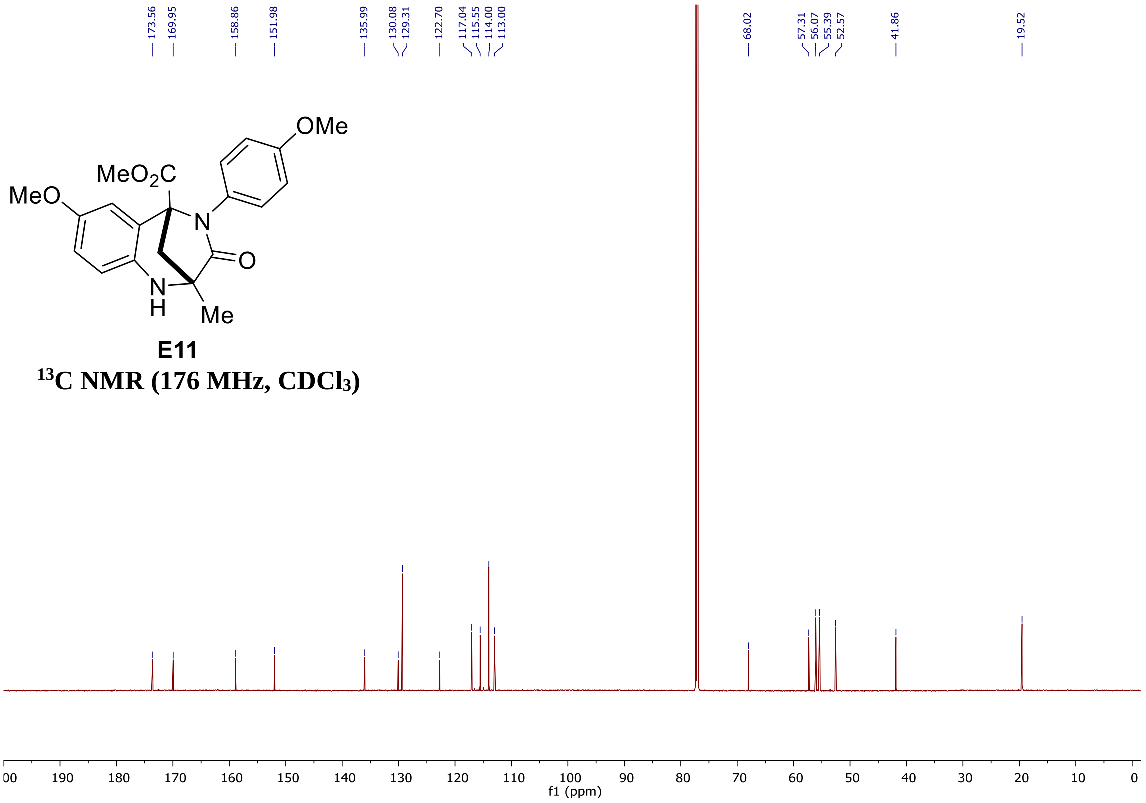

E11

^{13}C NMR (176 MHz, $CDCl_3$)

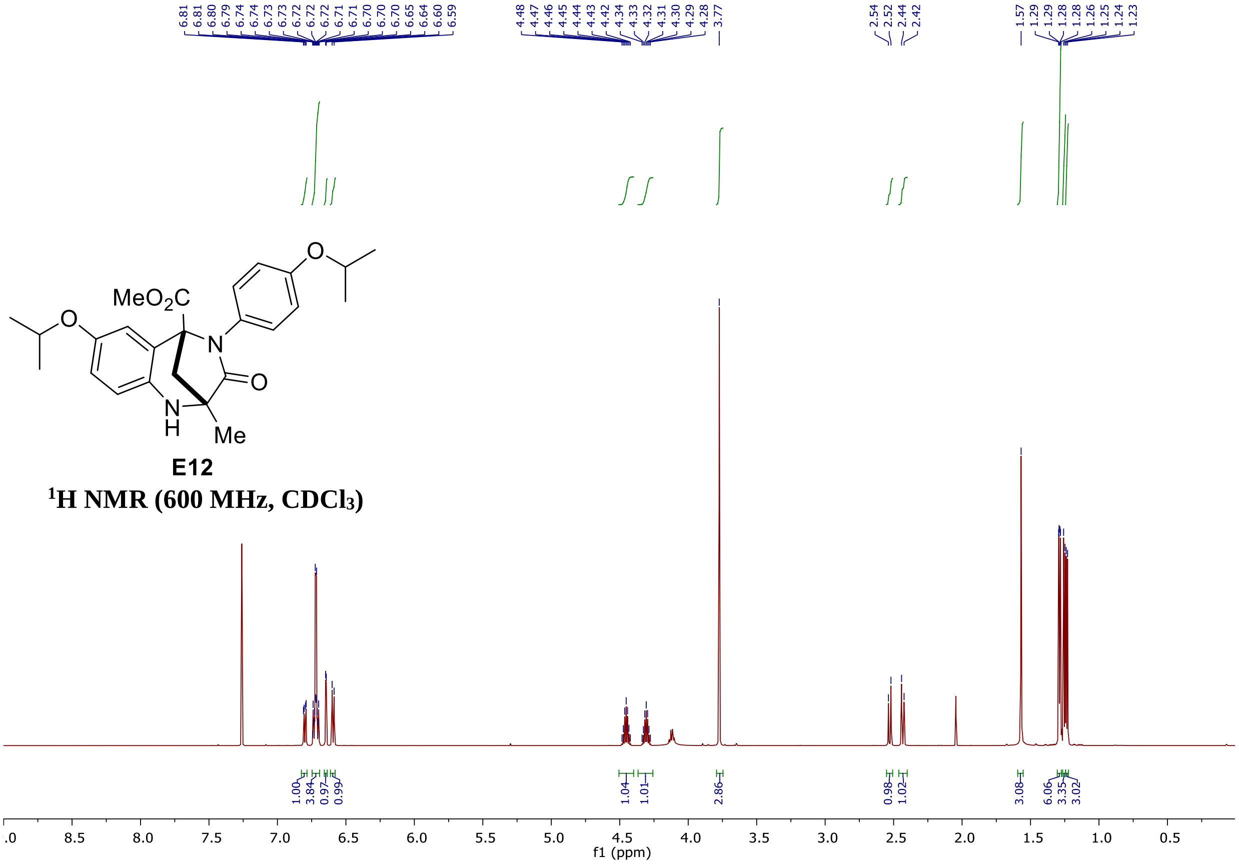
MeO2C
N
O
N
H
Me
O
O
E12
1H NMR (600 MHz, CDCl3)
f1 (ppm)

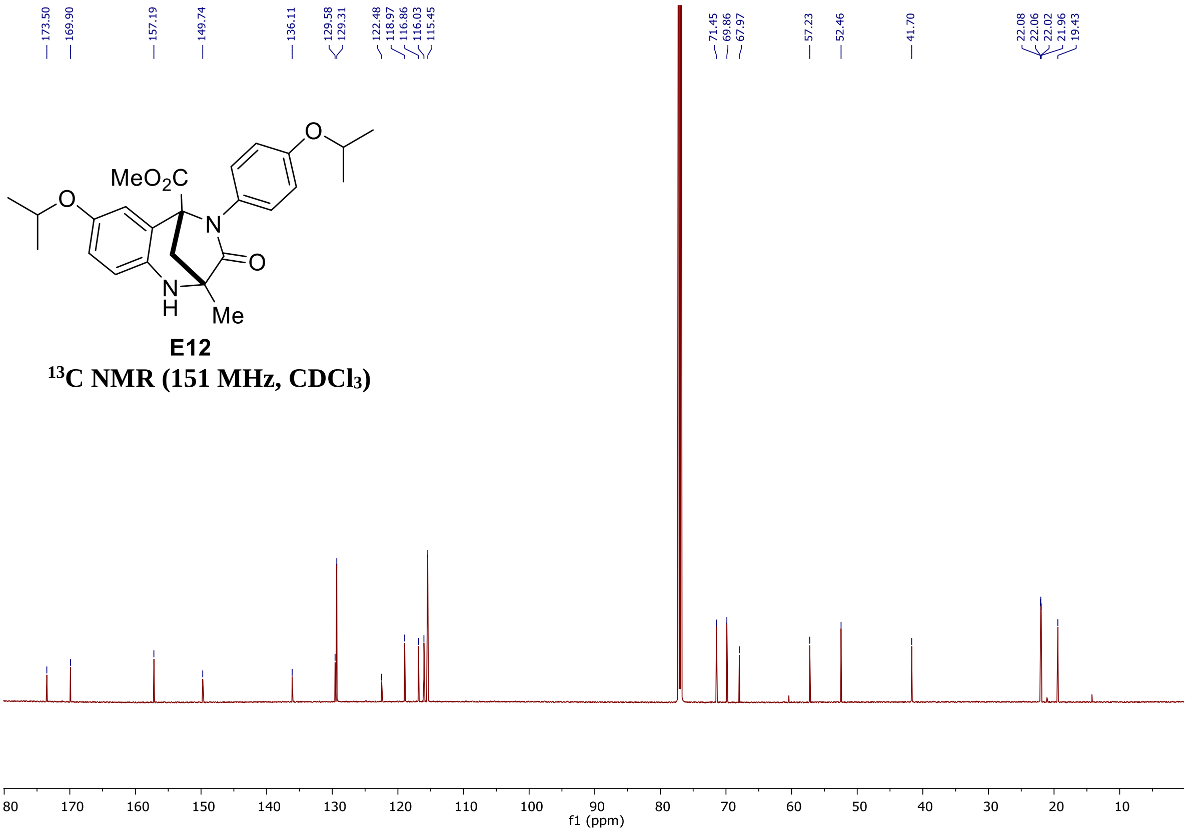
MeO2C
N
O
N
H
Me
O
O
E12
13C NMR (151 MHz, CDCl3)
f1 (ppm)

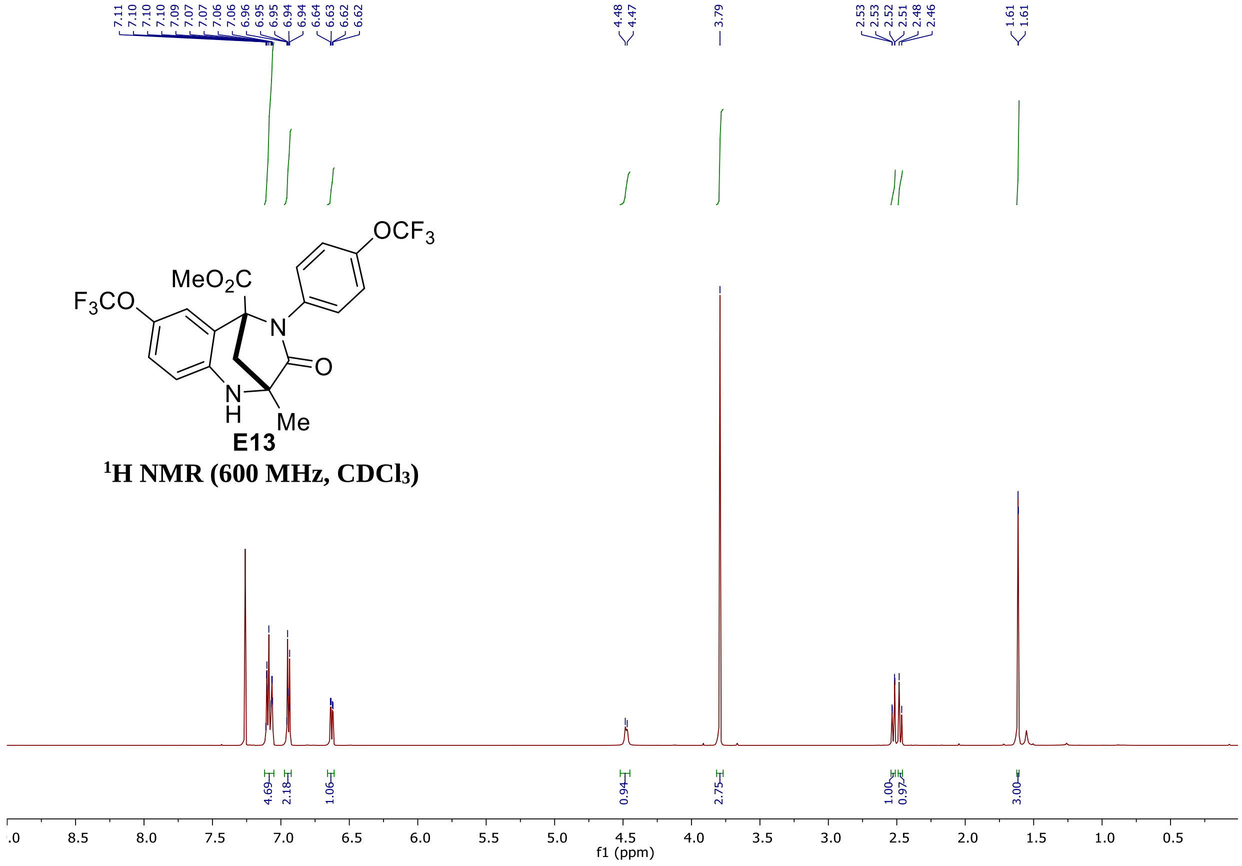
7.11 7.10 7.10 7.09 7.07 7.07 7.06 7.06 6.96 6.95 6.95 6.94 6.94 6.64 6.63 6.62 6.62
4.48 4.47
3.79
2.53 2.53 2.52 2.51 2.48 2.46
1.61 1.61
OCF3
MeO2C
F3CO
N
O
N
H
Me
E13
1H NMR (600 MHz, CDCl3)
4.69 2.18 1.06 0.94 2.75 1.00 0.97 3.00
f1 (ppm)

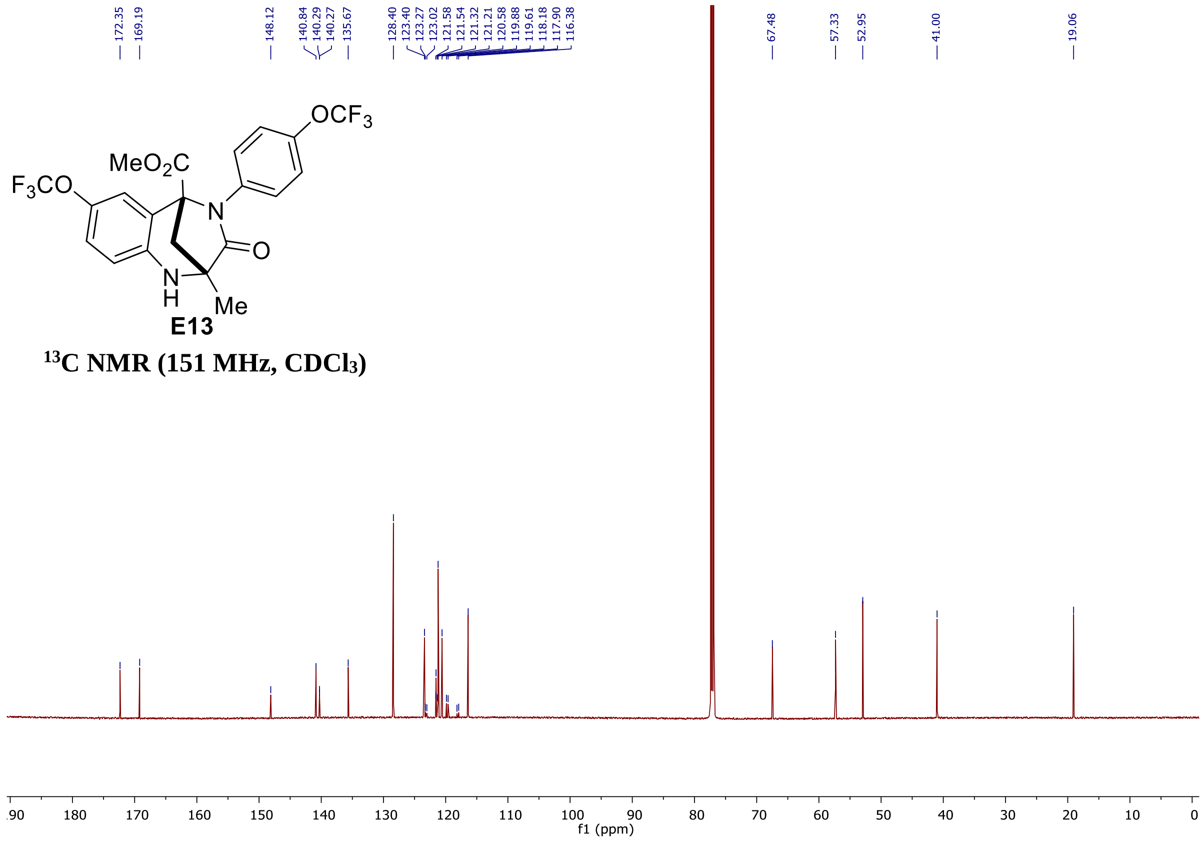
172.35 169.19 148.12 140.84 140.29 140.27 135.67 128.40 123.40 123.27 123.02 121.58 121.54 121.32 121.21 120.58 119.88 119.61 118.18 117.90 116.38 67.48 57.33 52.95 41.00 19.06
OCF3
MeO2C
F3CO
N
O
N
H
Me
E13
13C NMR (151 MHz, CDCl3)
f1 (ppm)

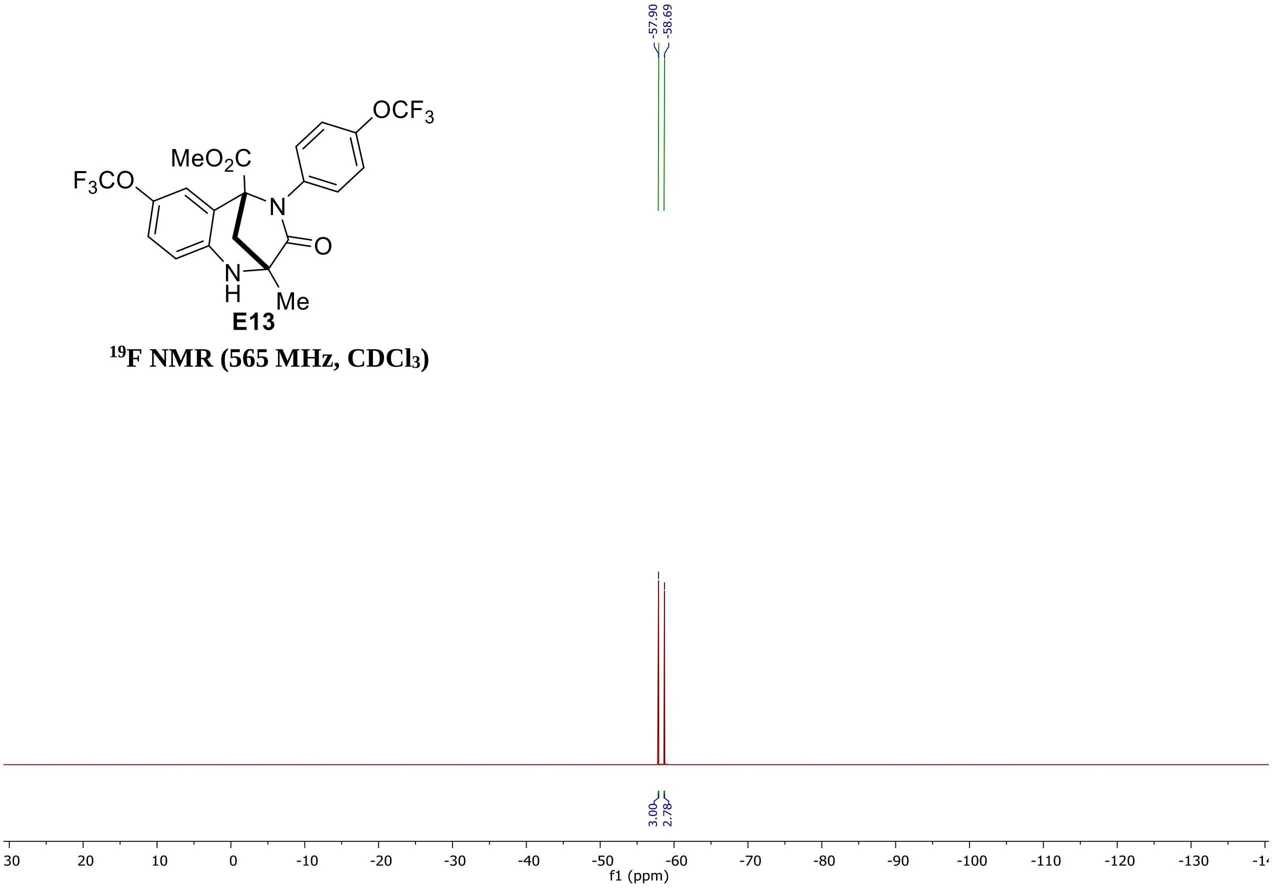

E13

^{19}F NMR (565 MHz, $CDCl_3$)

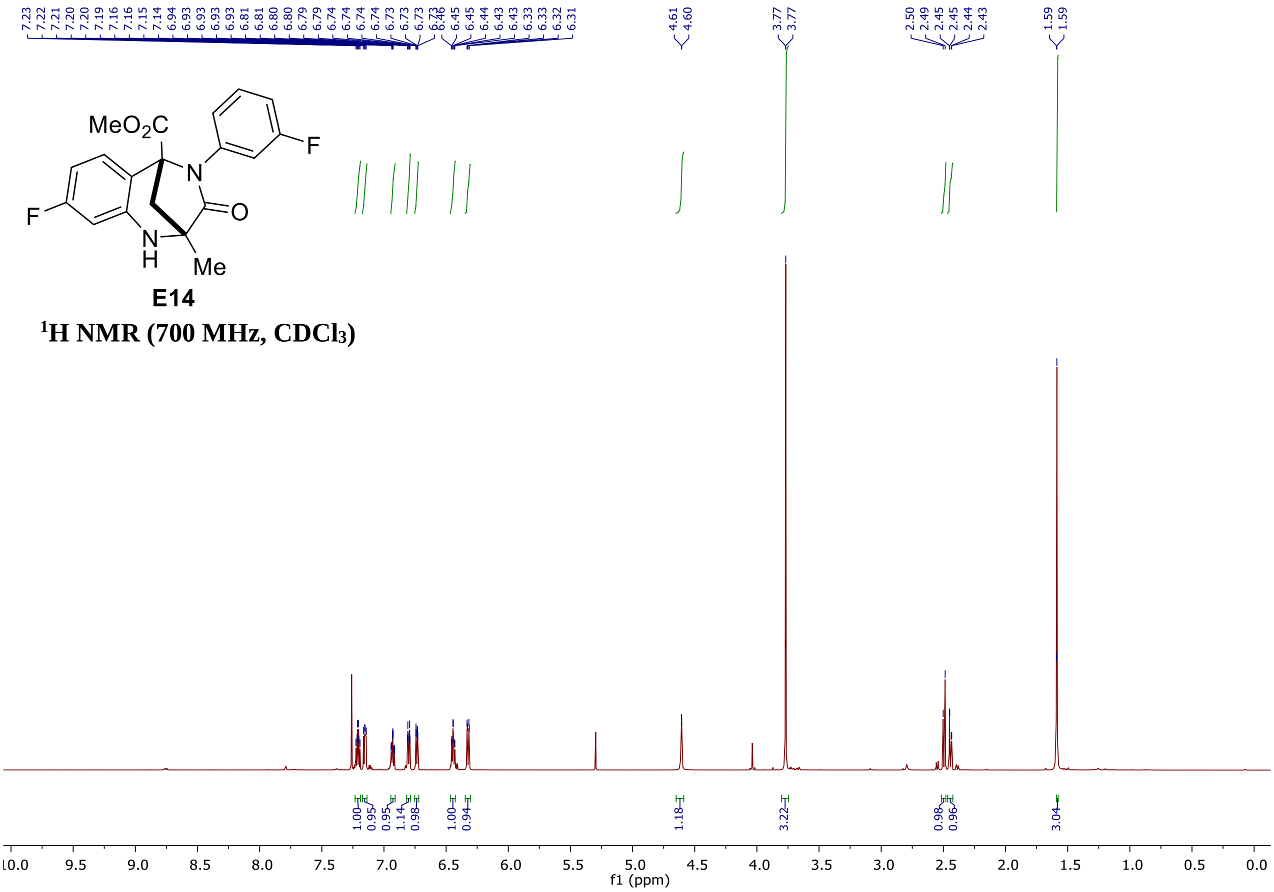

E14

^{1}H NMR (700 MHz, $CDCl_3$)

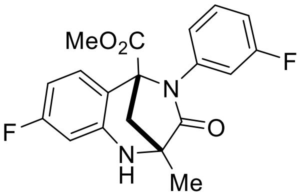

E14

^{13}C NMR (176 MHz, CDCl3)

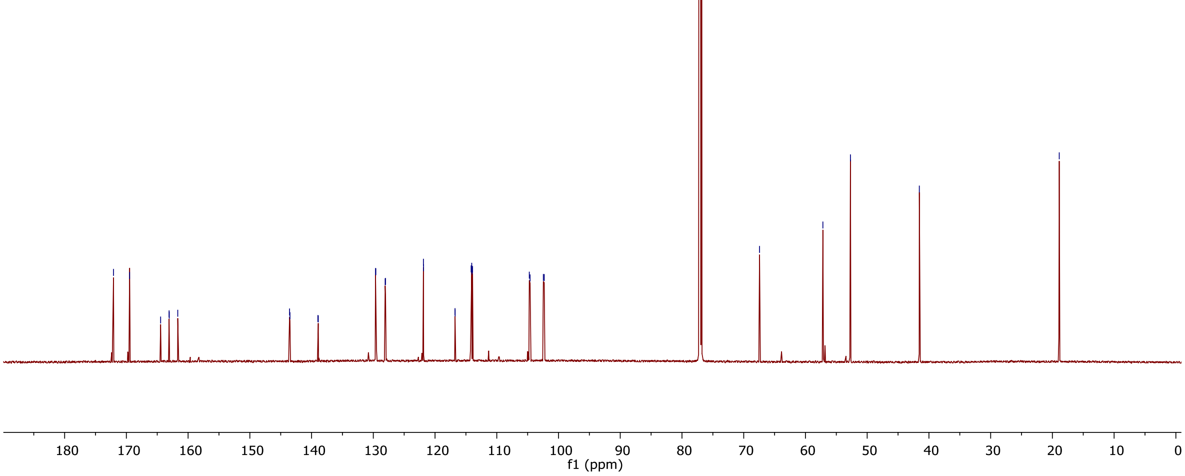

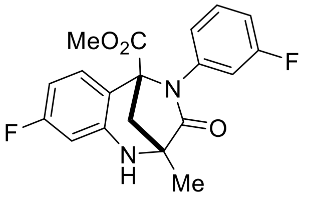

E14

^{19}F NMR (377 MHz, CDCl3)

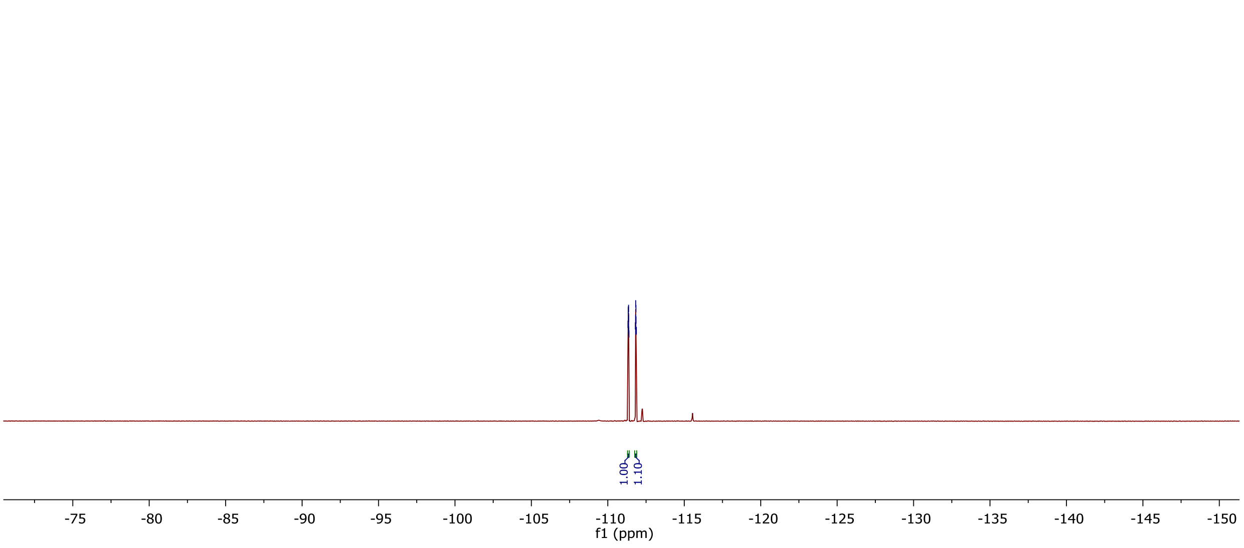

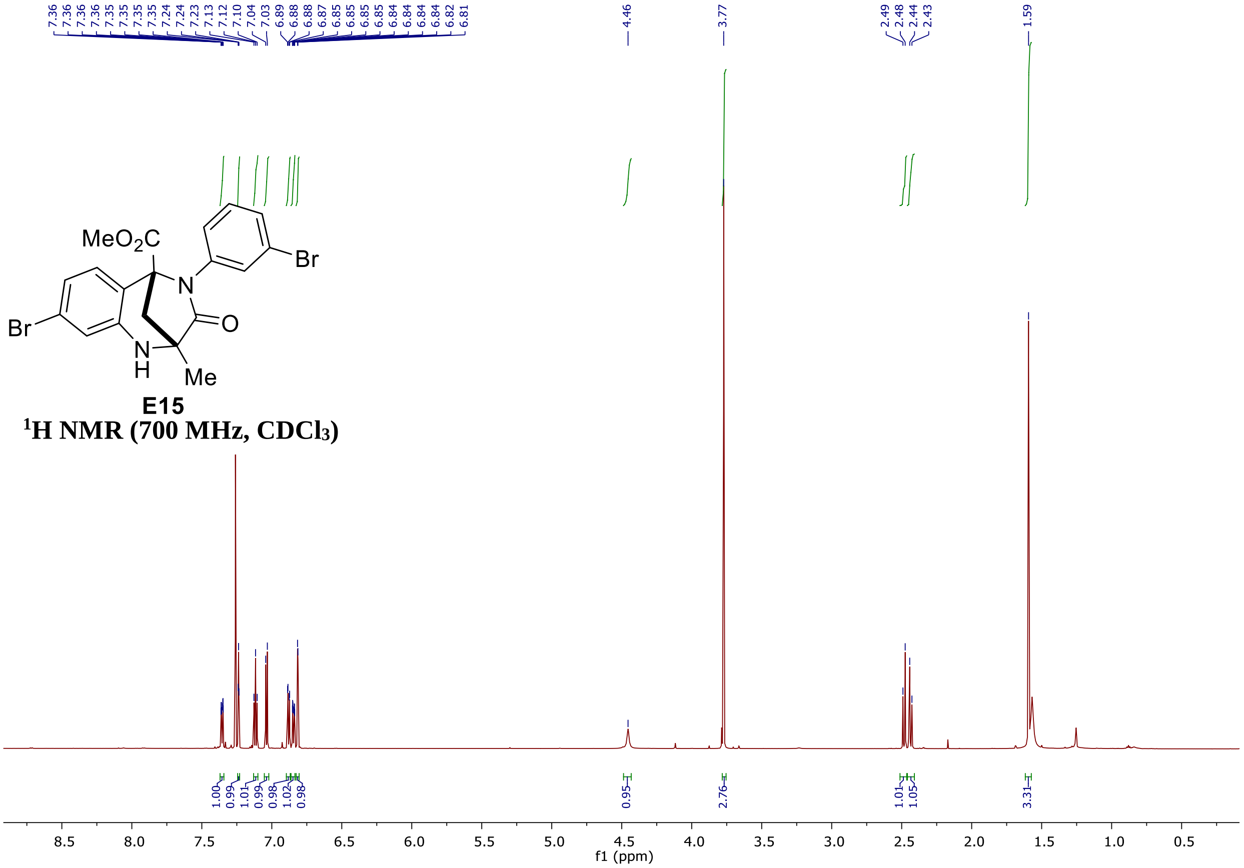

E15

^{1}H NMR (700 MHz, $CDCl_3$)

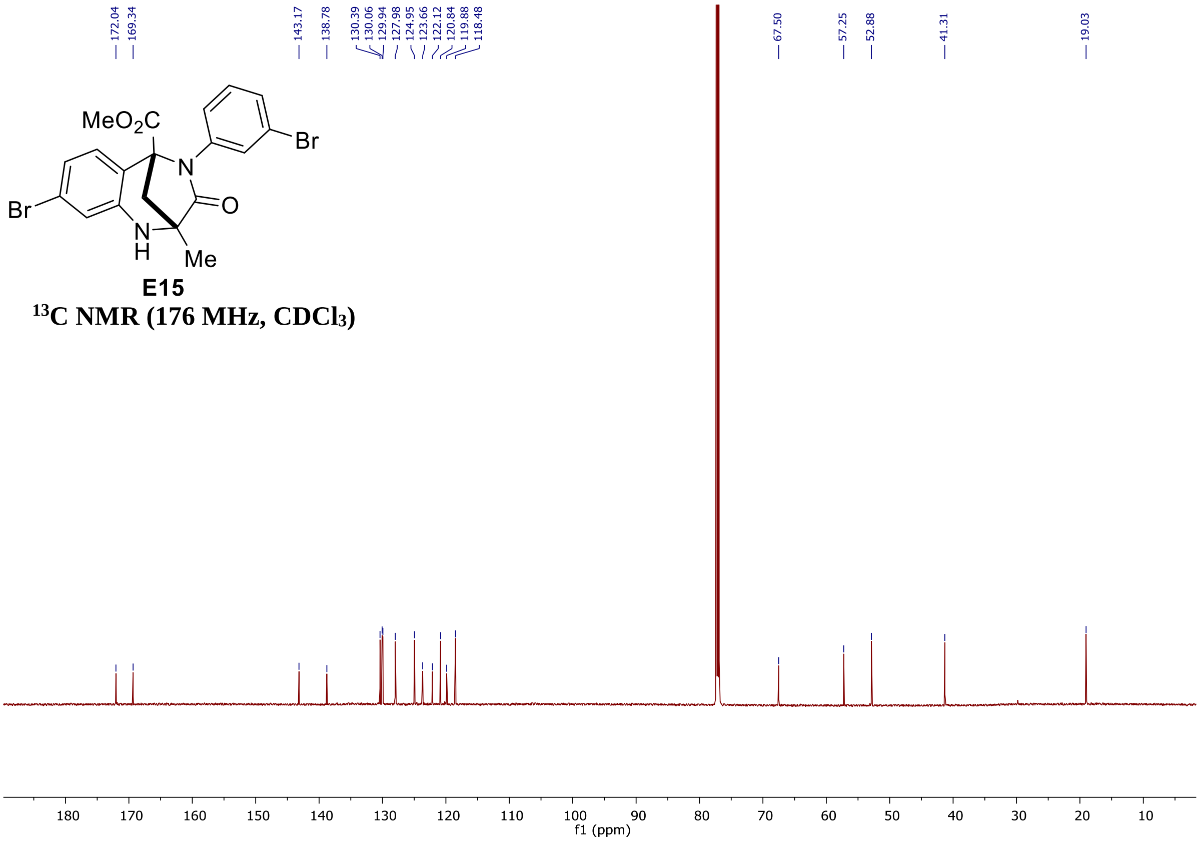

E15

^{13}C NMR (176 MHz, $CDCl_3$)

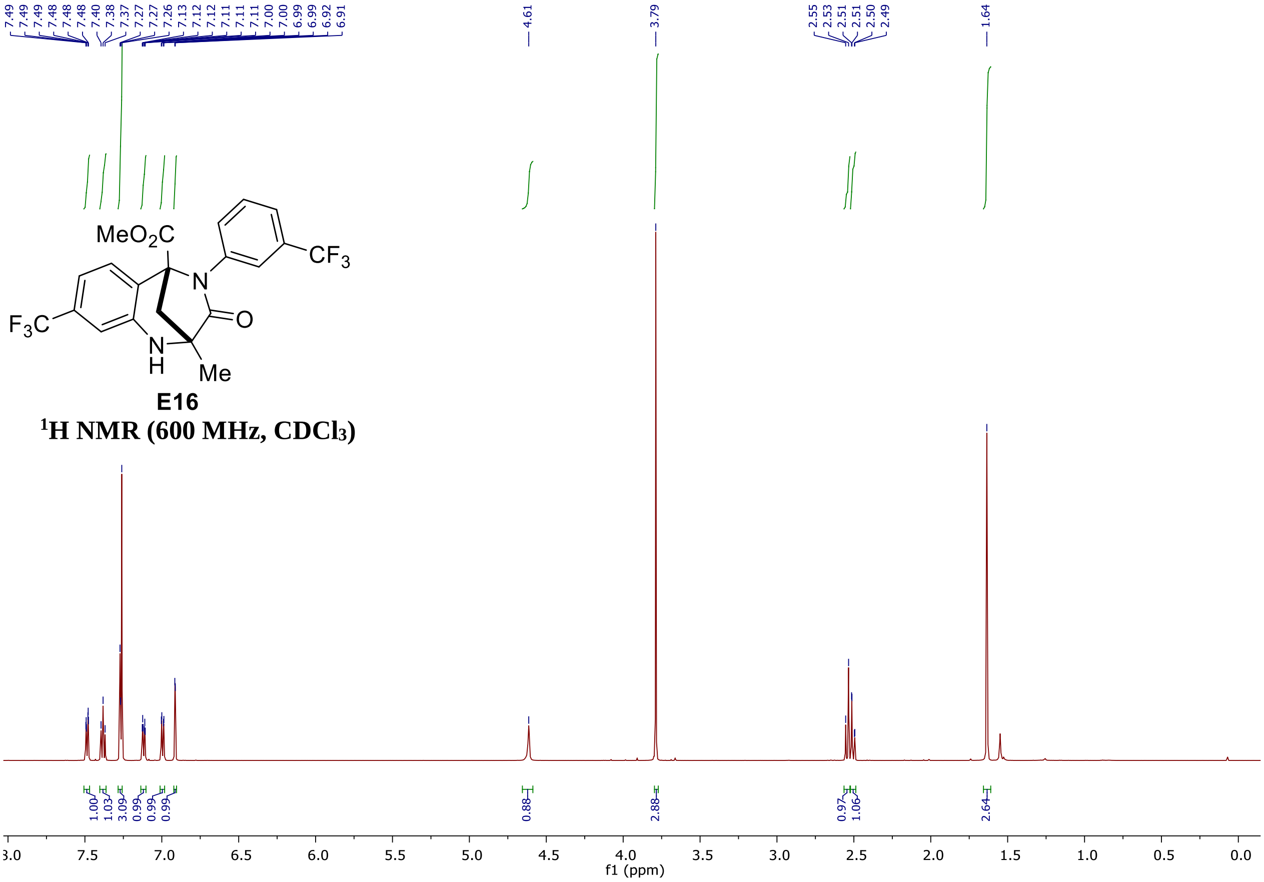
7.49 7.49 7.48 7.48 7.40 7.38 7.37 7.27 7.27 7.26 7.13 7.12 7.12 7.11 7.11 7.11 7.00 7.00 6.99 6.99 6.92 6.91
4.61
3.79
2.55 2.53 2.51 2.51 2.50 2.49
1.64
MeO2C
CF3
F3C
N
O
N
H
Me
E16
1H NMR (600 MHz, CDCl3)
1.00 1.03 3.09 0.99 0.99 0.99
0.88
2.88
0.97 1.06
2.64
f1 (ppm)

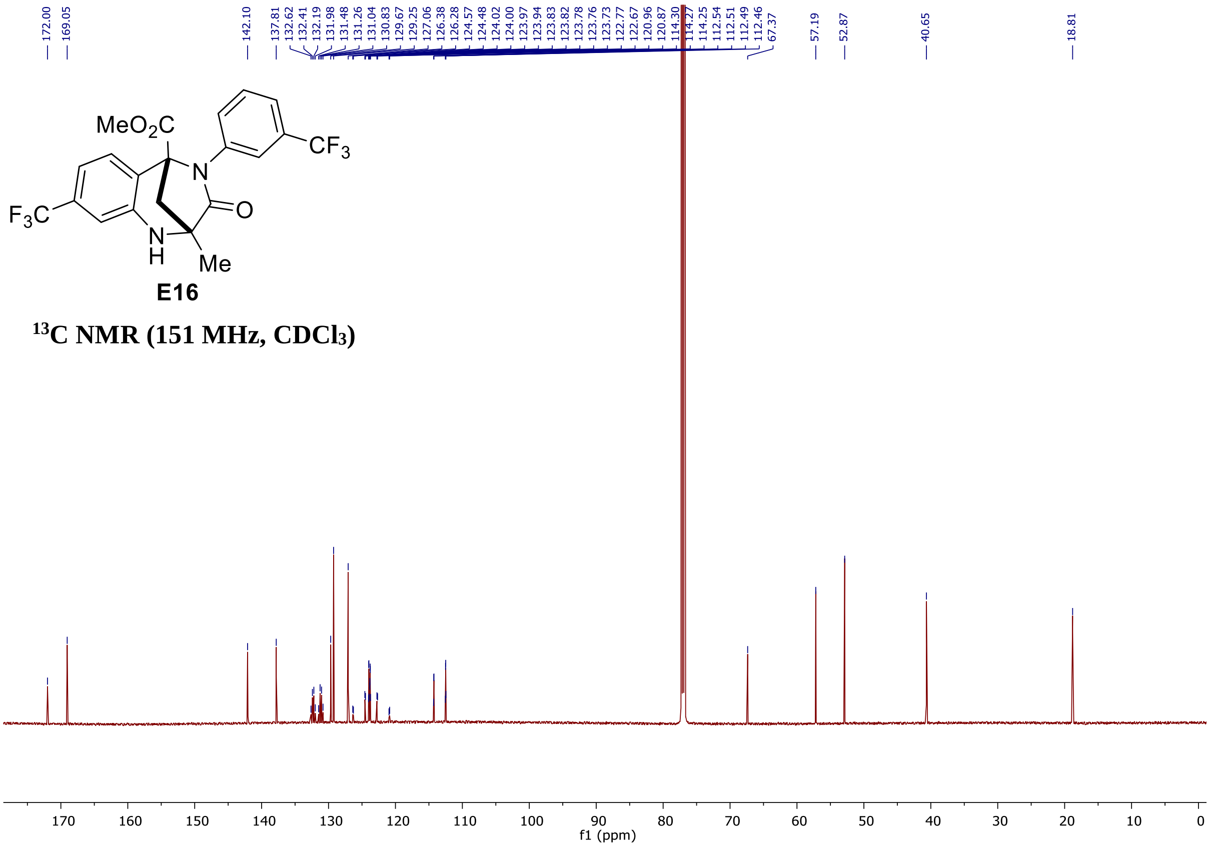
172.00 169.05
142.10
137.81 132.62 132.41 132.19 131.98 131.48 131.26 131.04 130.83 129.67 129.25 127.06 126.38 126.28 124.57 124.48 124.02 124.00 123.97 123.94 123.83 123.82 123.78 123.76 123.73 122.77 122.67 120.96 120.87 114.30 114.27 114.25 112.54 112.51 112.49 112.46 67.37
57.19
52.87
40.65
18.81
MeO2C
CF3
F3C
N
O
N
H
Me
E16
13C NMR (151 MHz, CDCl3)
f1 (ppm)

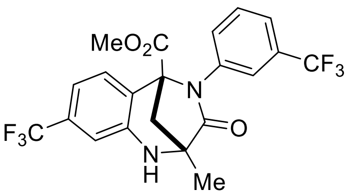

E16

^{19}F NMR (565 MHz, CDCl₃)

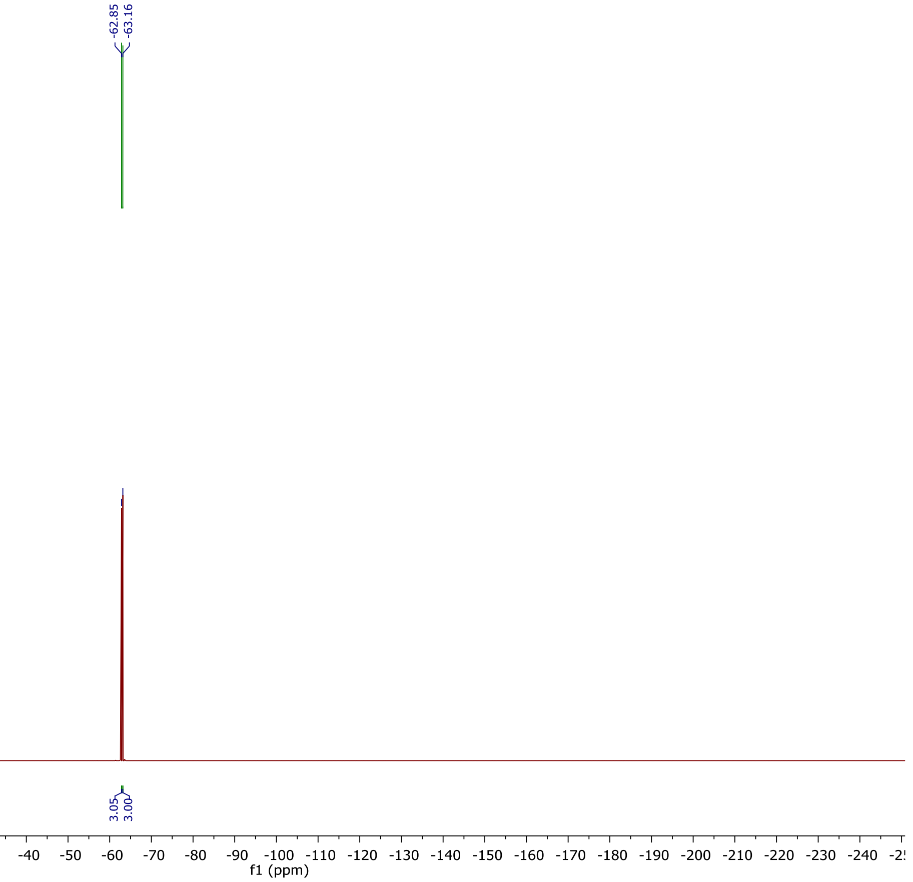

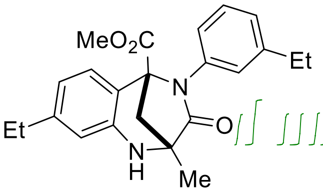

E17

^{1}H NMR (700 MHz, CDCl₃)

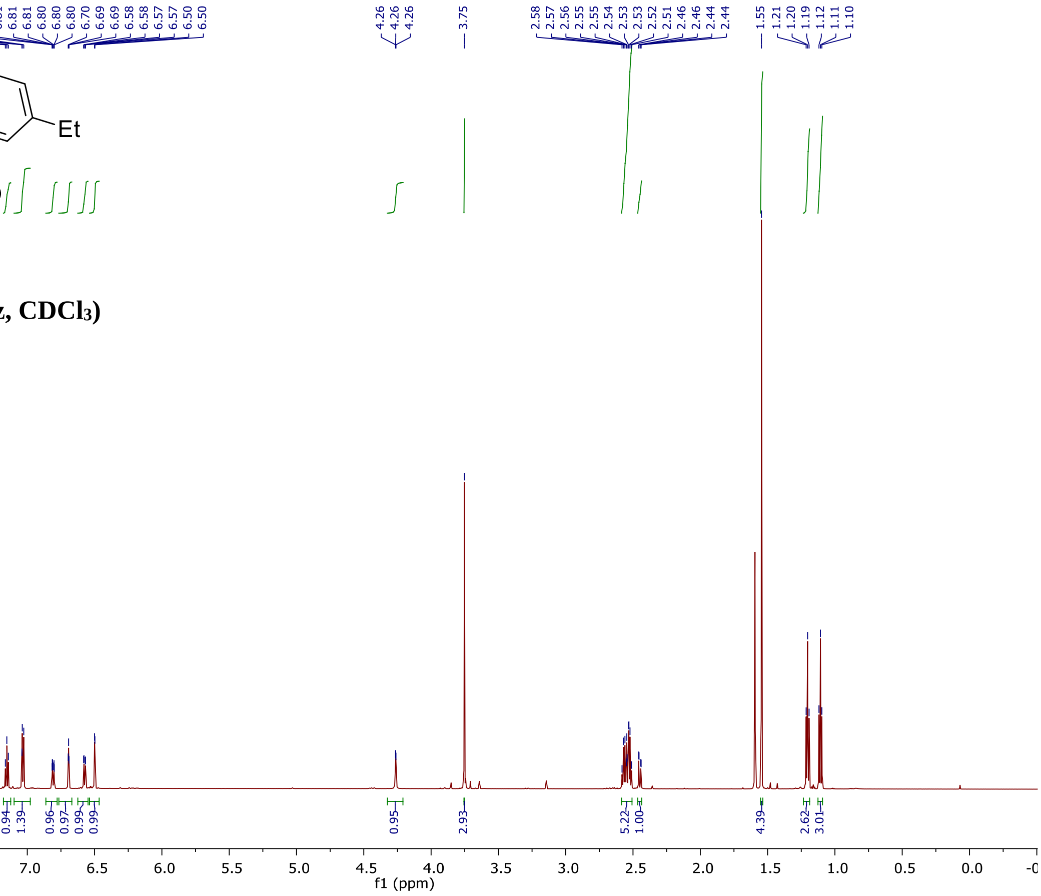

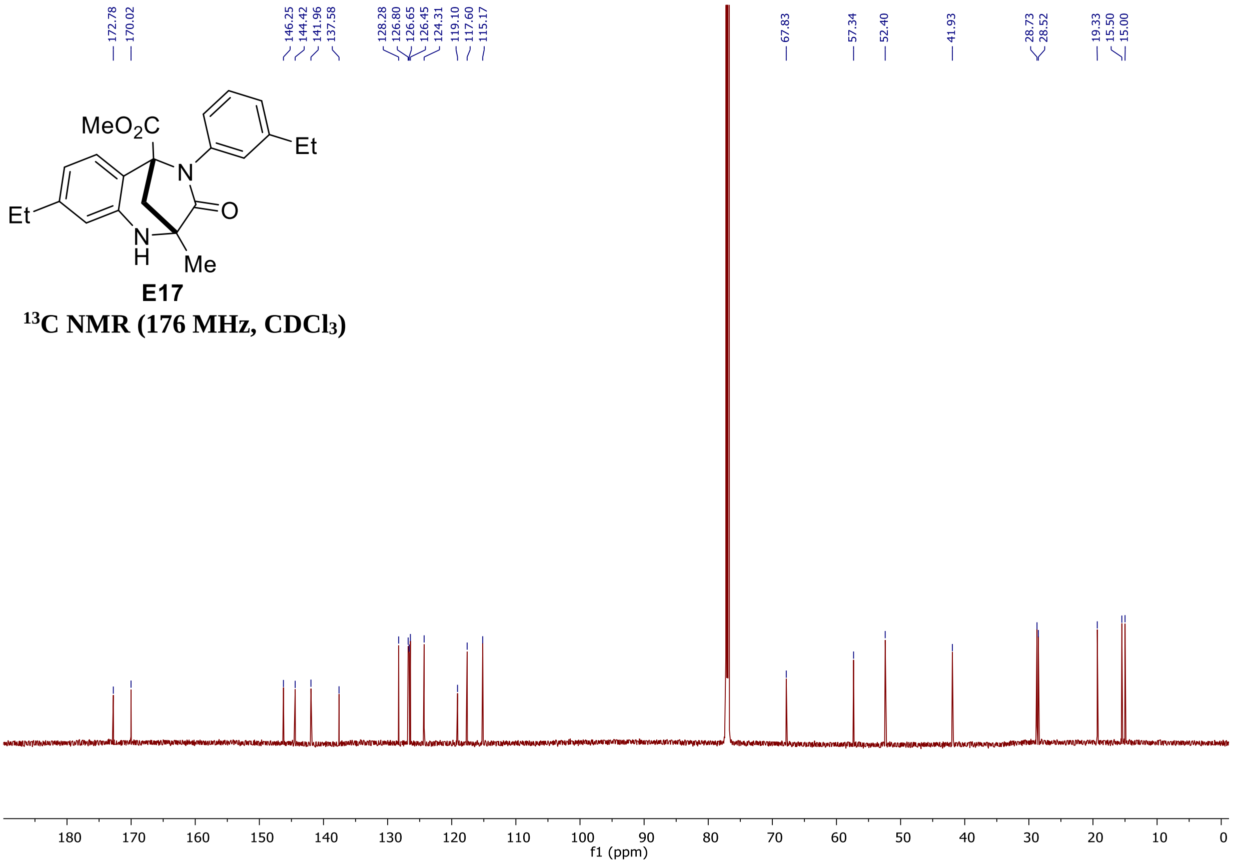

E17

^{13}C NMR (176 MHz, $CDCl_3$)

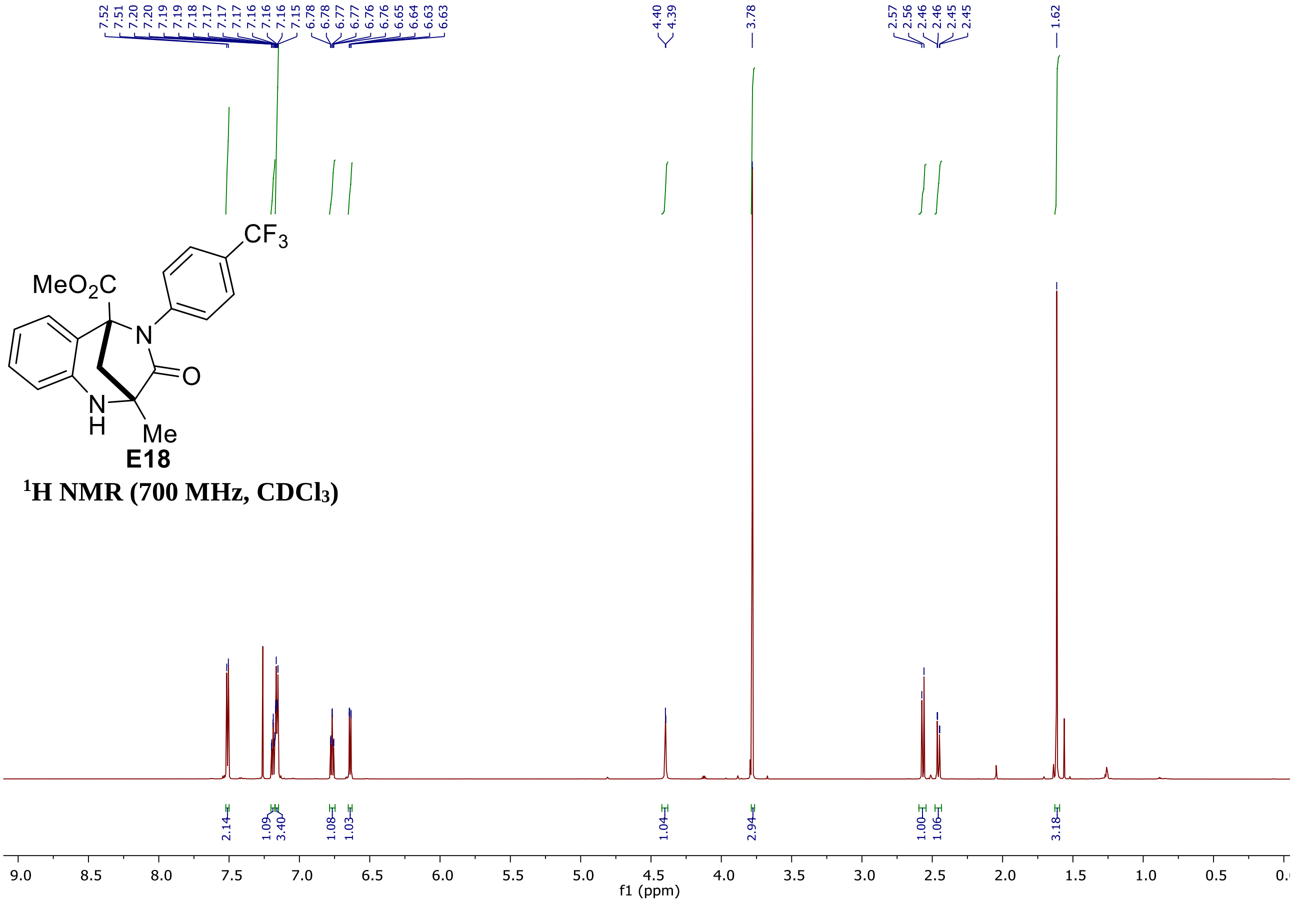

E18

^{1}H NMR (700 MHz, $CDCl_3$)

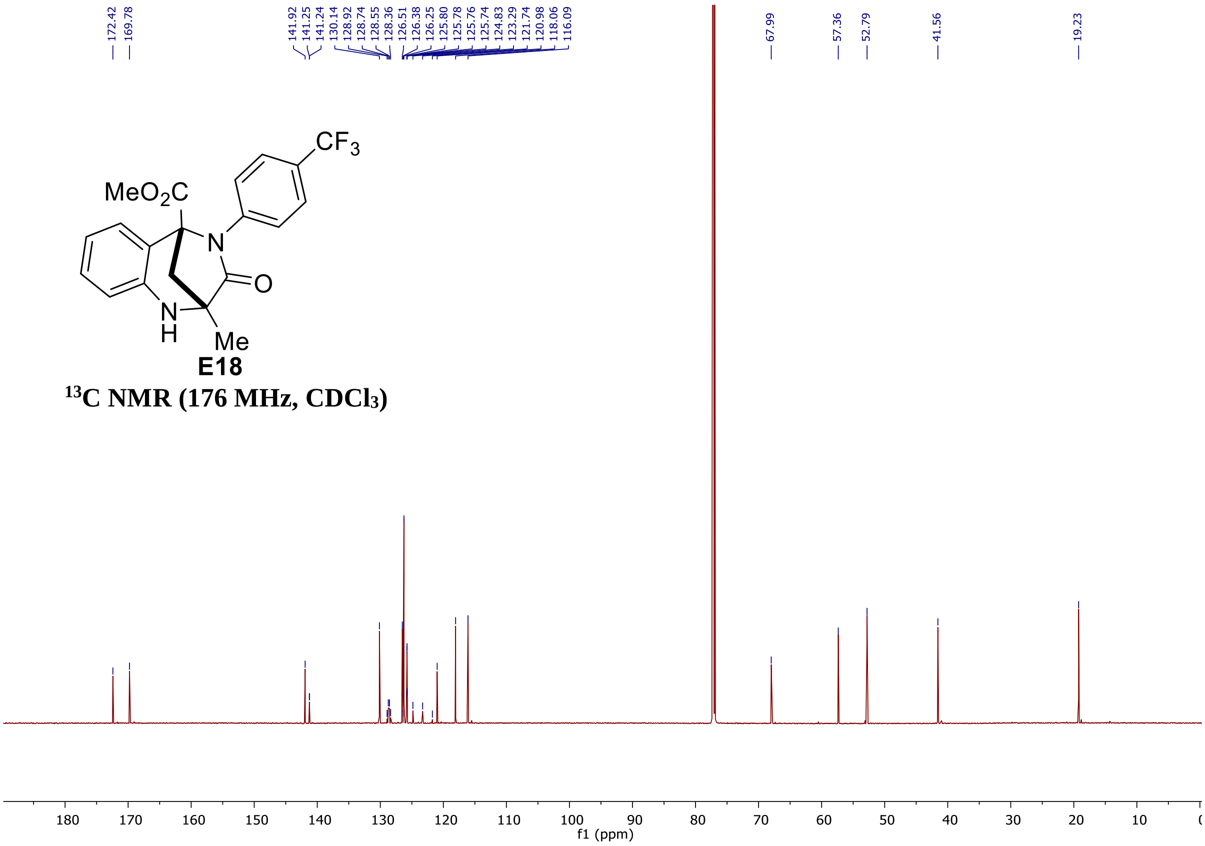
172.42
169.78
141.92
141.25
141.24
130.14
128.92
128.74
128.55
128.36
126.51
126.38
126.25
125.80
125.78
125.76
125.74
124.83
123.29
121.74
120.98
118.06
116.09
67.99
57.36
52.79
41.56
19.23
CF3
MeO2C
N
O
N
H
Me
E18
13C NMR (176 MHz, CDCl3)
180 170 160 150 140 130 120 110 100 90 80 70 60 50 40 30 20 10 0
f1 (ppm)

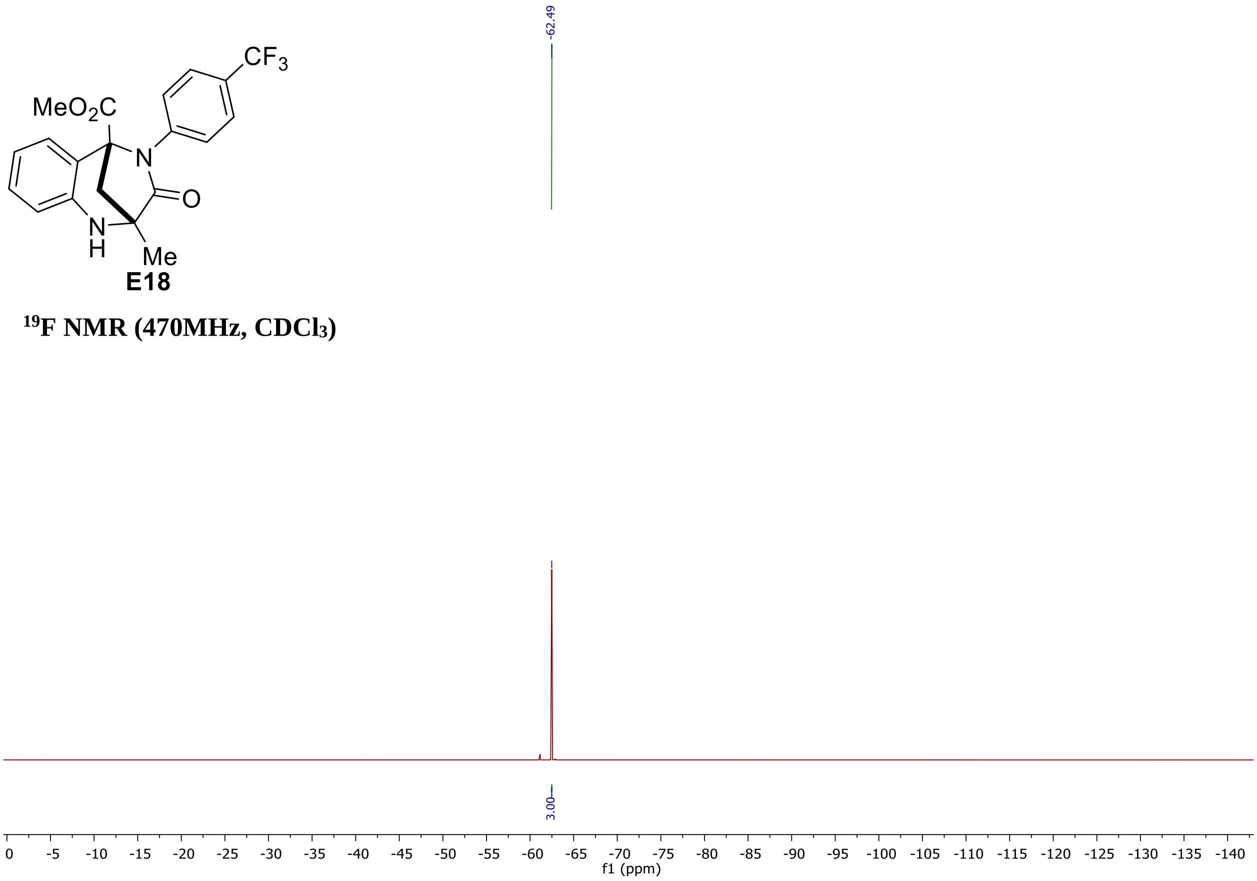
-62.49
CF3
MeO2C
N
O
N
H
Me
E18
19F NMR (470MHz, CDCl3)
3.00
0 -5 -10 -15 -20 -25 -30 -35 -40 -45 -50 -55 -60 -65 -70 -75 -80 -85 -90 -95 -100 -105 -110 -115 -120 -125 -130 -135 -140
f1 (ppm)

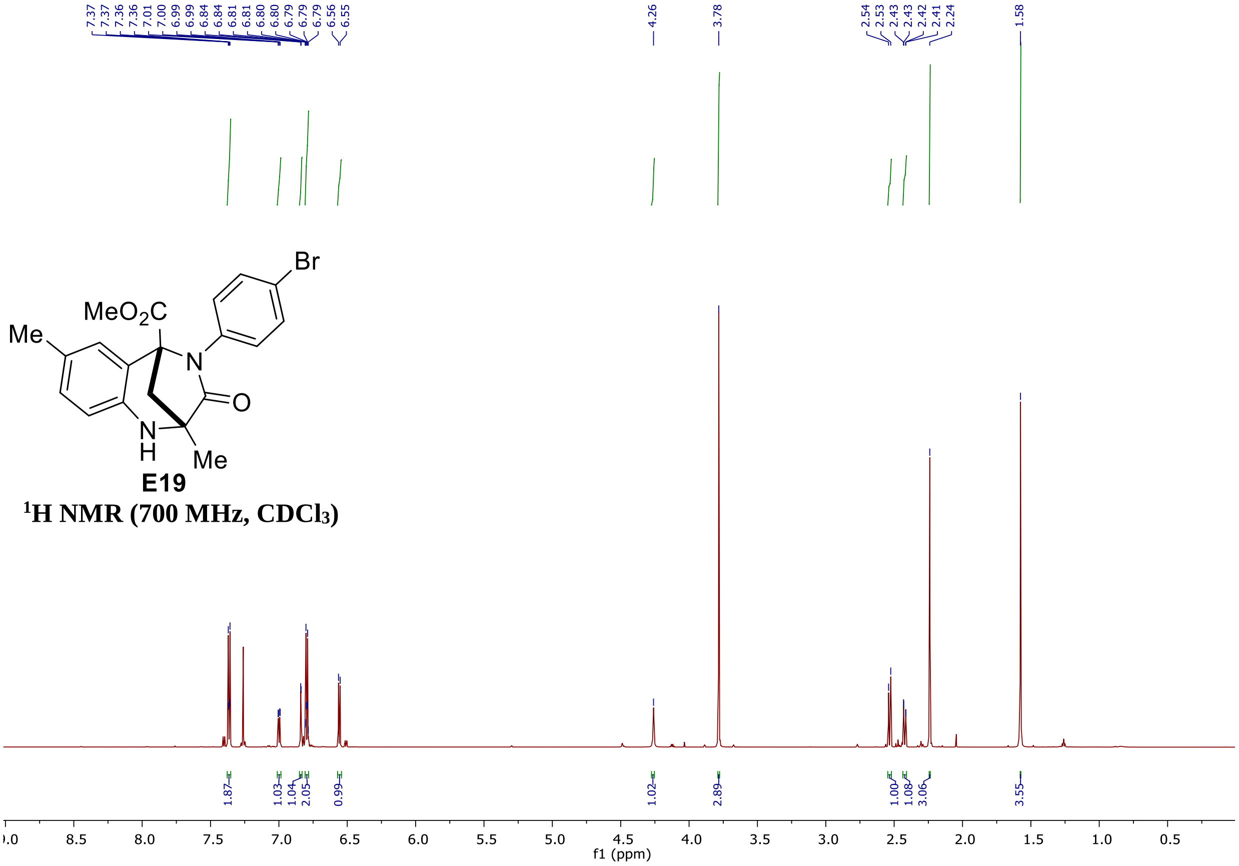

E19
¹H NMR (700 MHz, CDCl3)

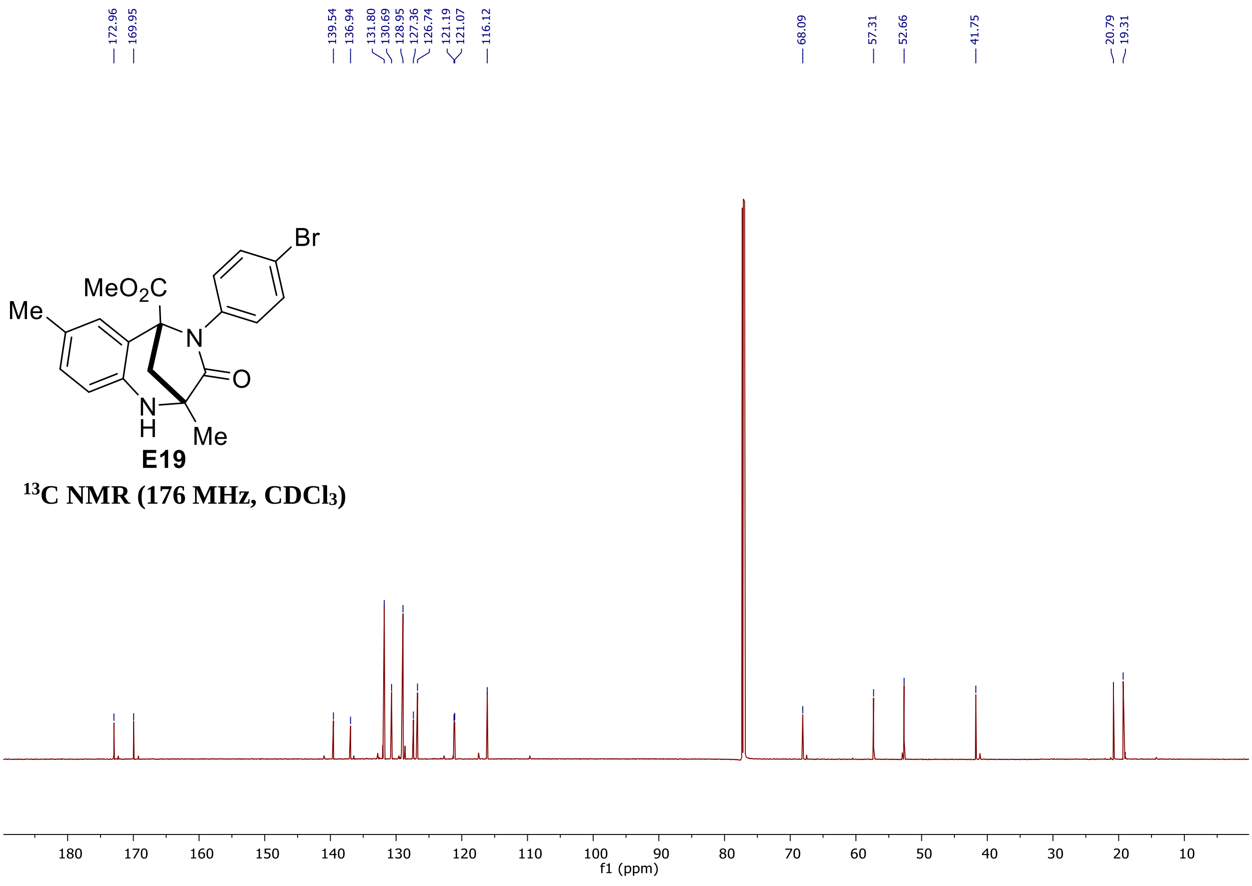

E19
¹³C NMR (176 MHz, CDCl3)

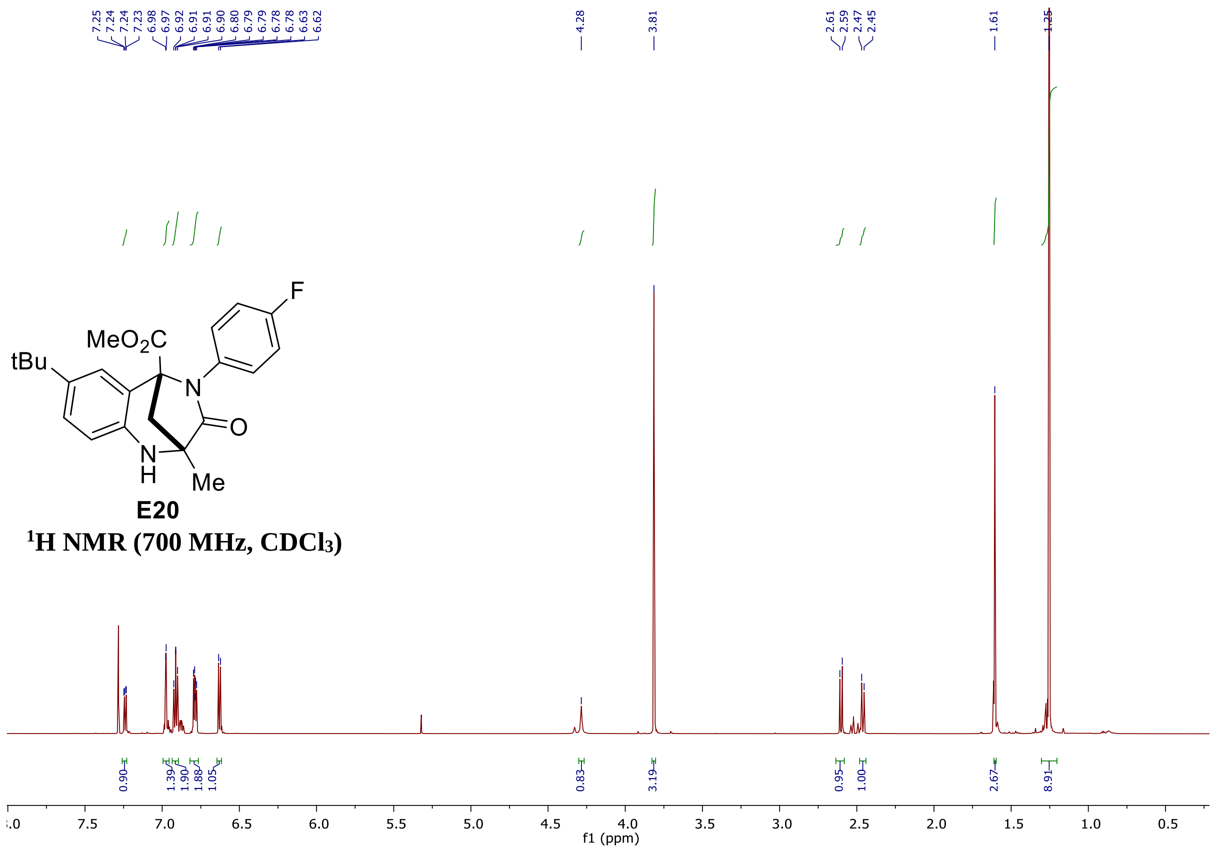
MeO2C
tBu
F
N
O
N
H
Me
E20
1H NMR (700 MHz, CDCl3)
f1 (ppm)

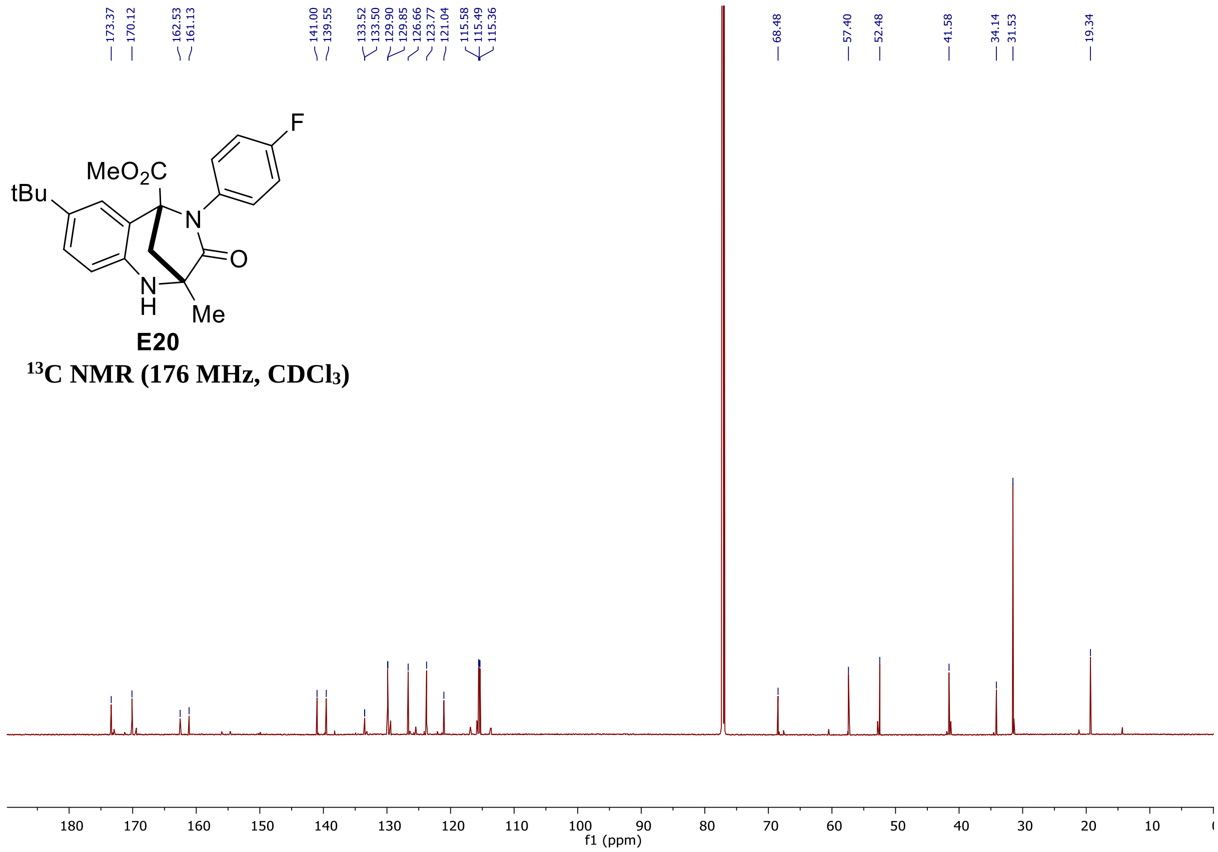
MeO2C
tBu
F
N
O
N
H
Me
E20
13C NMR (176 MHz, CDCl3)
f1 (ppm)

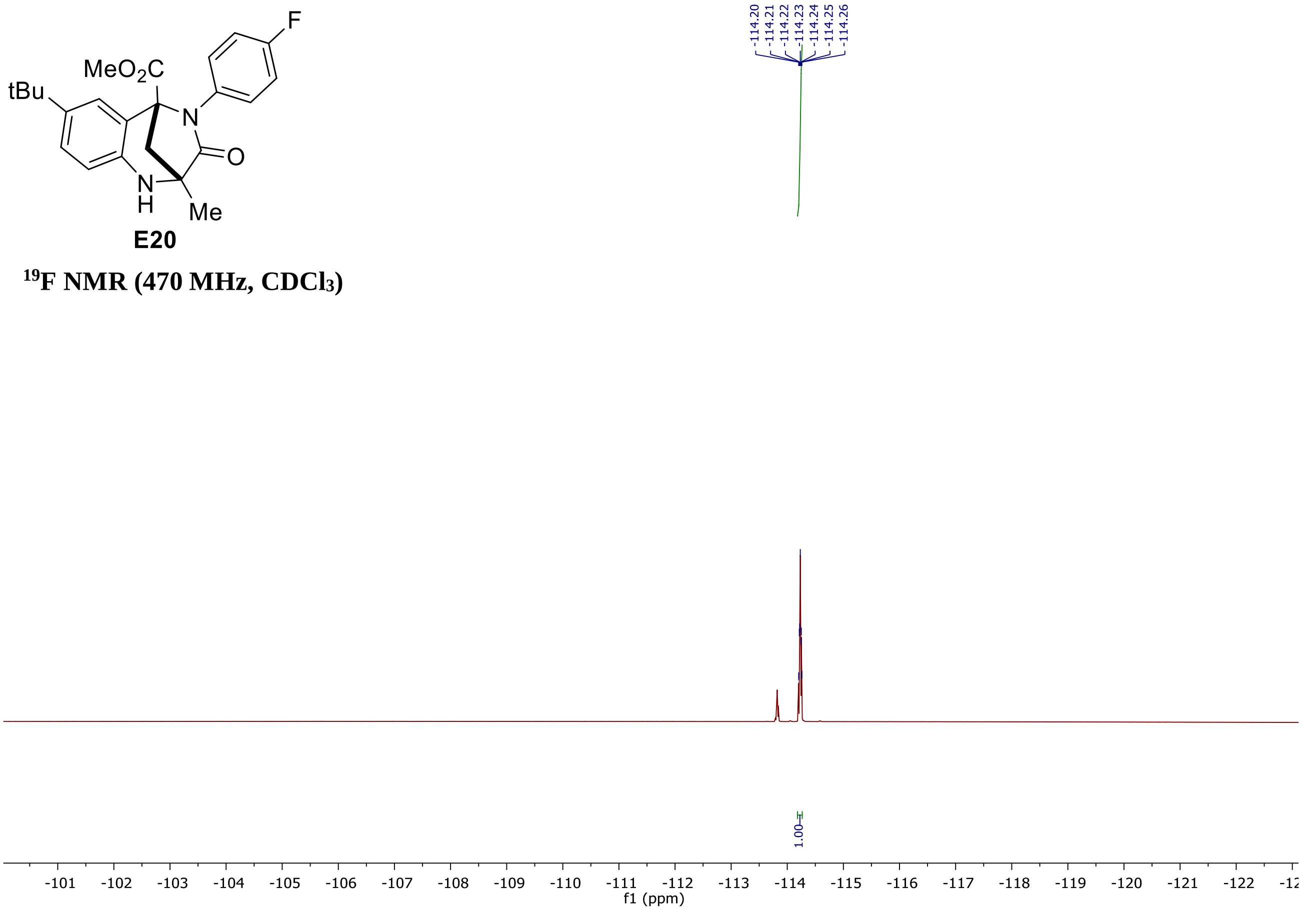

E20

^{19}F NMR (470 MHz, CDCl3)

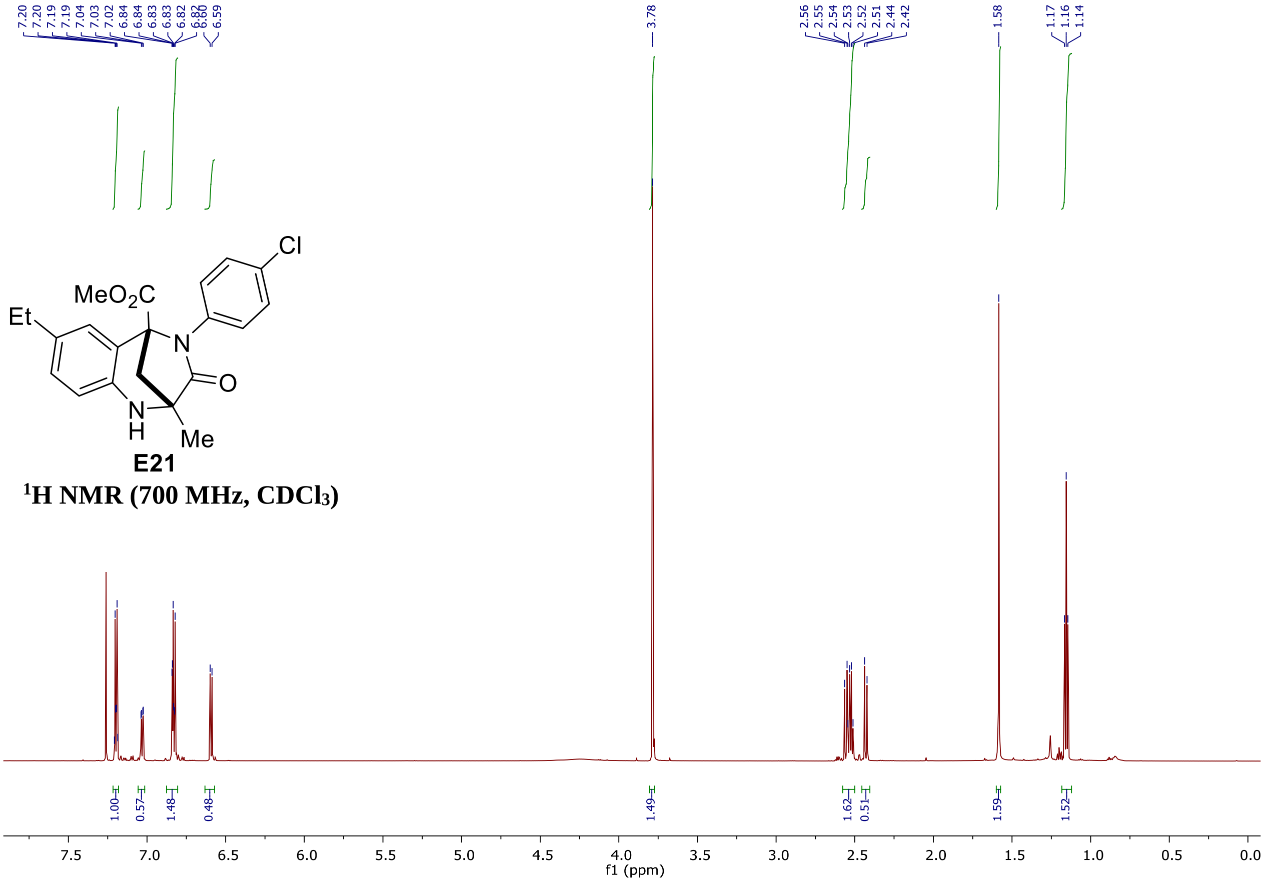

E21

^{1}H NMR (700 MHz, CDCl3)

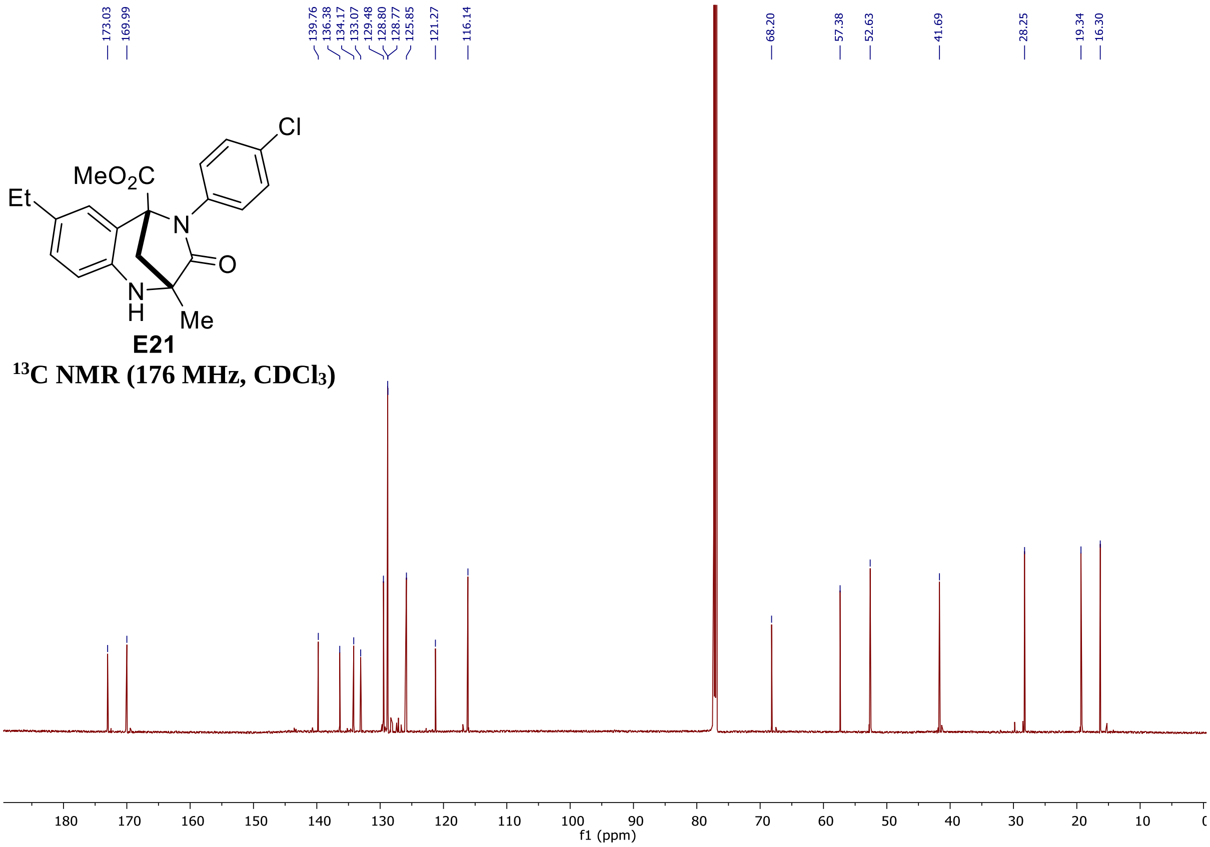
MeO2C
Et
Cl
N
O
N
H
Me
E21
13C NMR (176 MHz, CDCl3)
f1 (ppm)

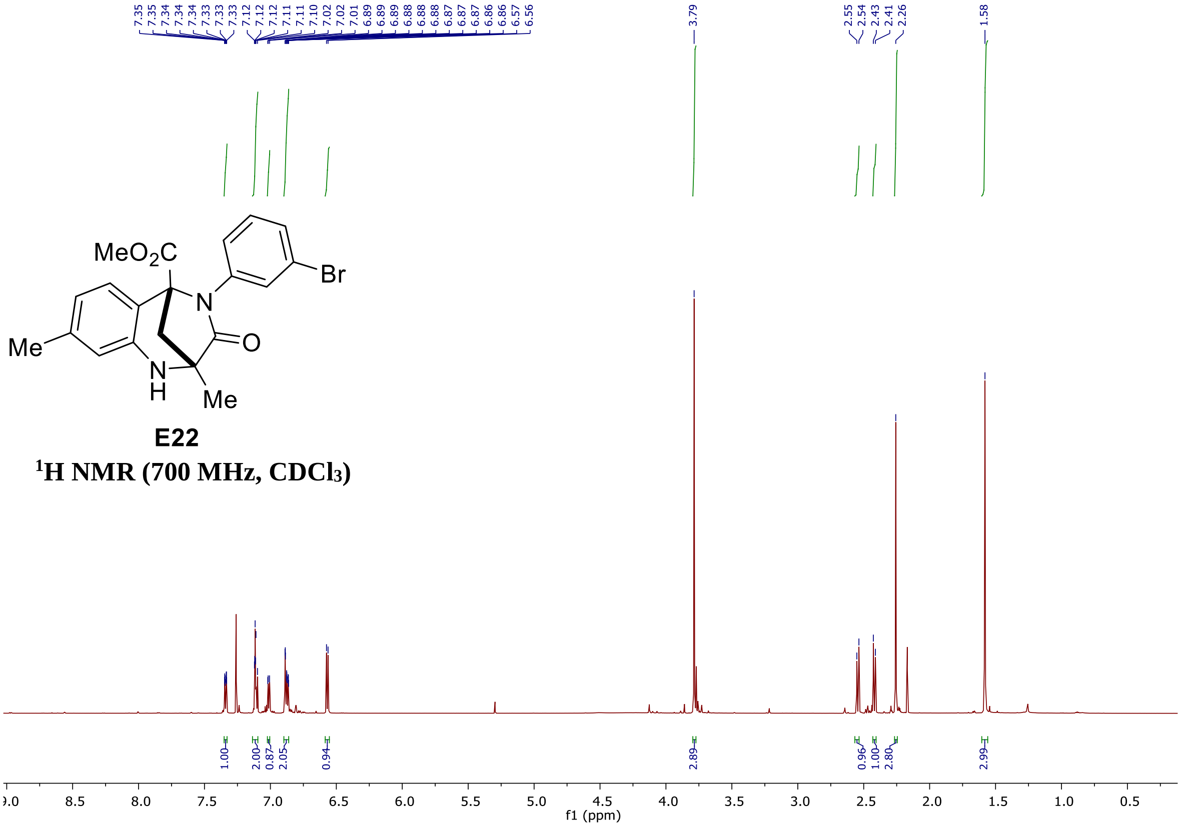
MeO2C
Br
N
O
Me
N
H
Me
E22
1H NMR (700 MHz, CDCl3)
f1 (ppm)

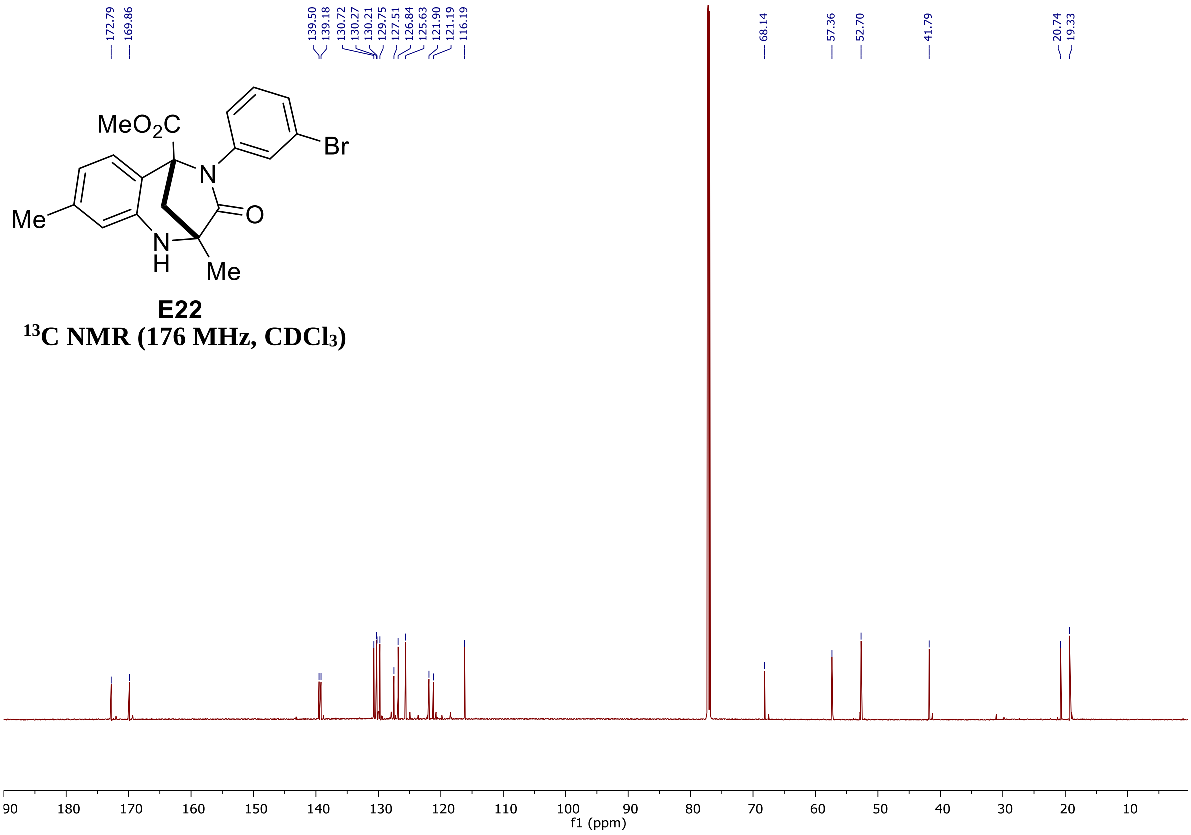

E22
^{13}C NMR (176 MHz, $CDCl_3$)

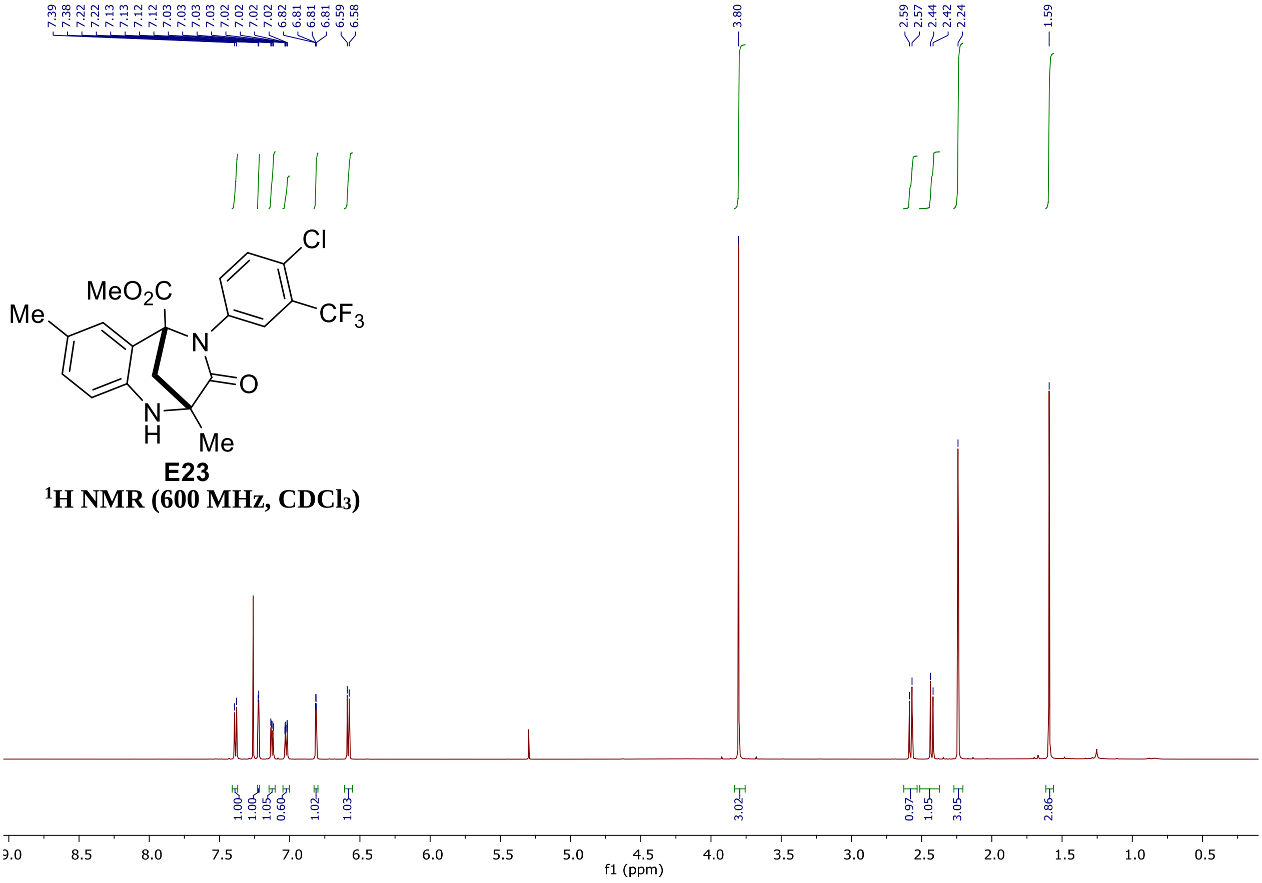

E23
^{1}H NMR (600 MHz, $CDCl_3$)

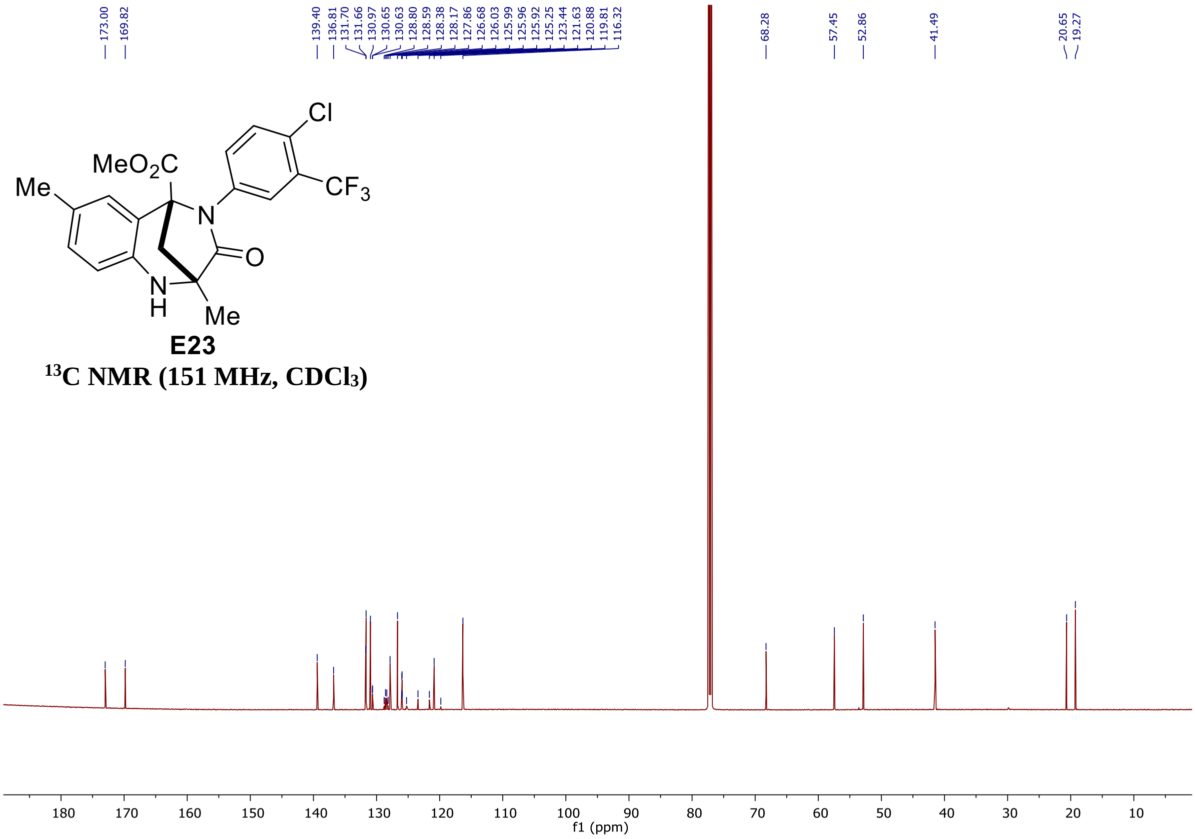

E23

^{13}C NMR (151 MHz, CDCl$_3$)

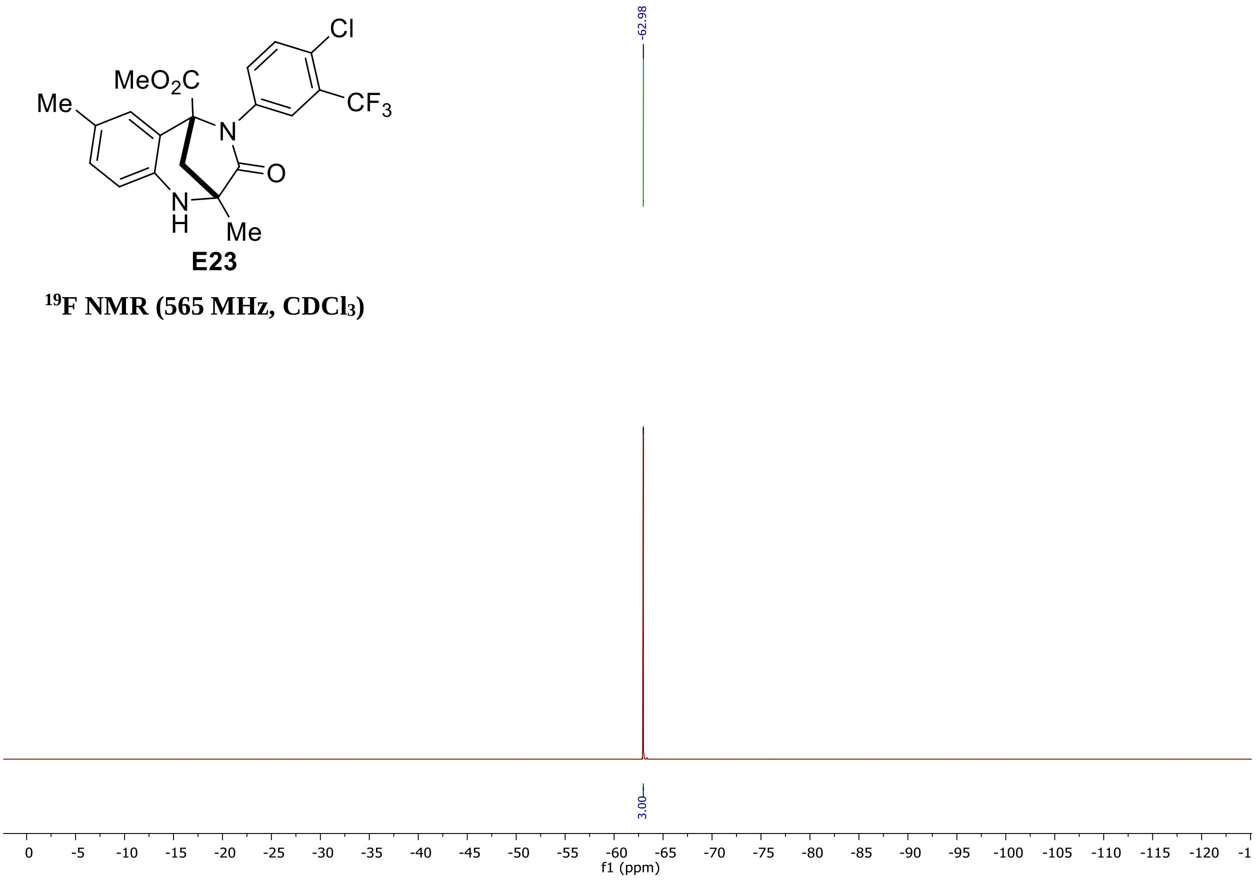

E23

^{19}F NMR (565 MHz, CDCl$_3$)

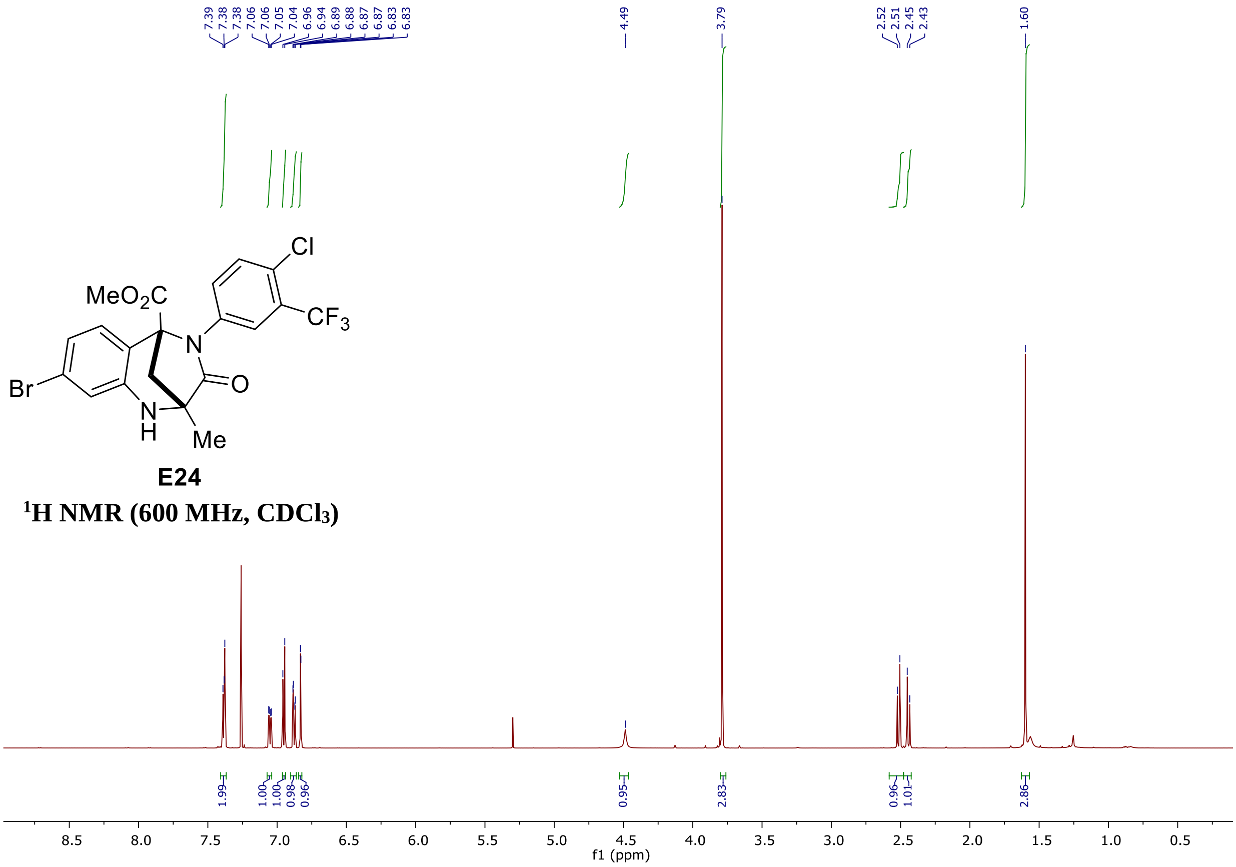

E24

^{1}H NMR (600 MHz, $CDCl_3$)

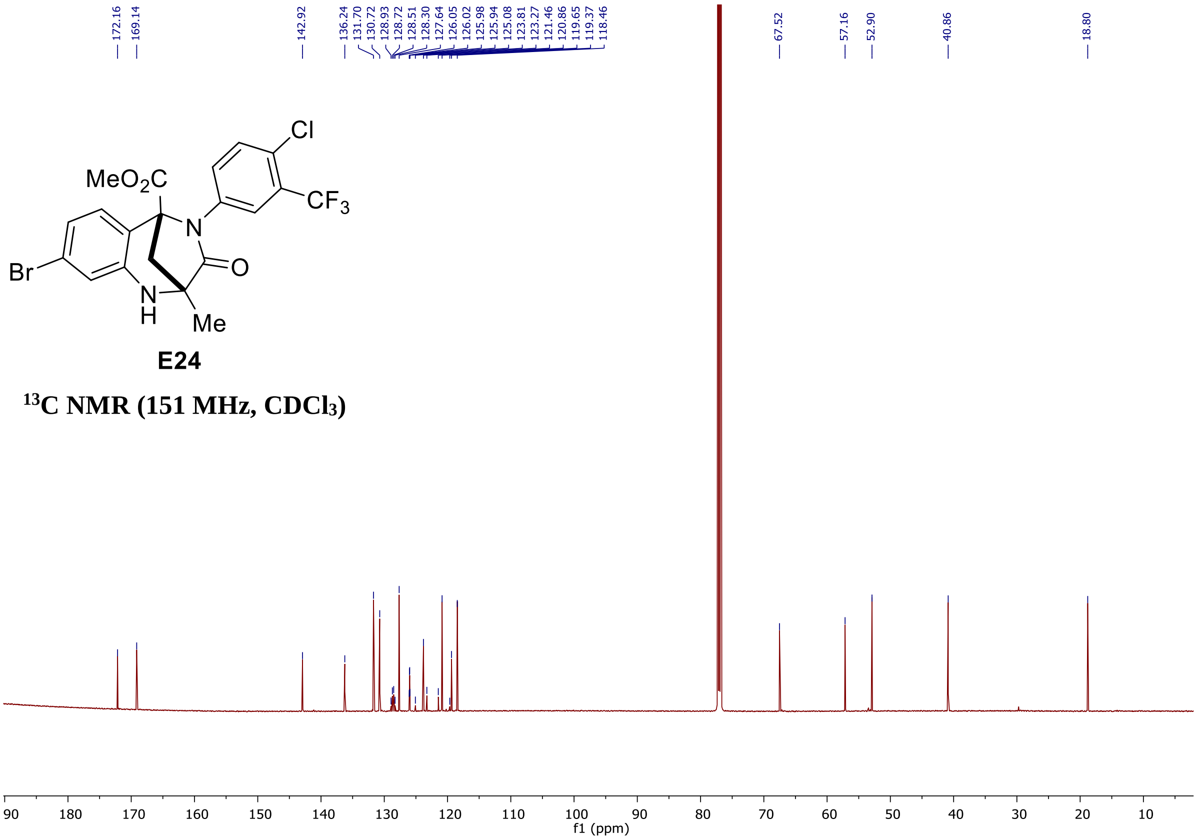

E24

^{13}C NMR (151 MHz, $CDCl_3$)

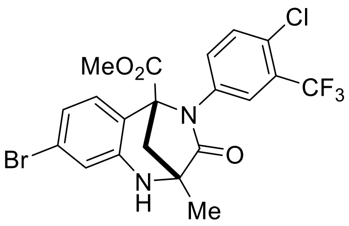

E24

^{19}F NMR (565 MHz, CDCl$_3$)

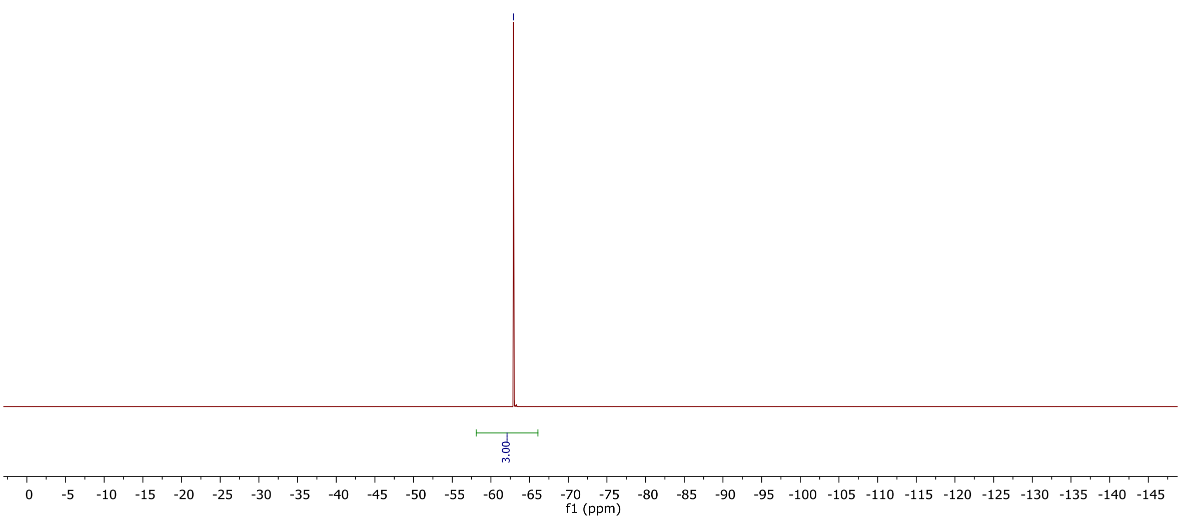

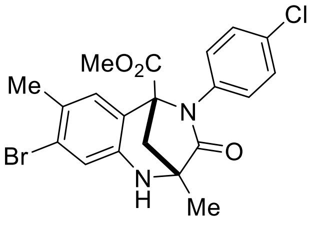

E25

^{1}H NMR (700 MHz, CDCl$_3$)

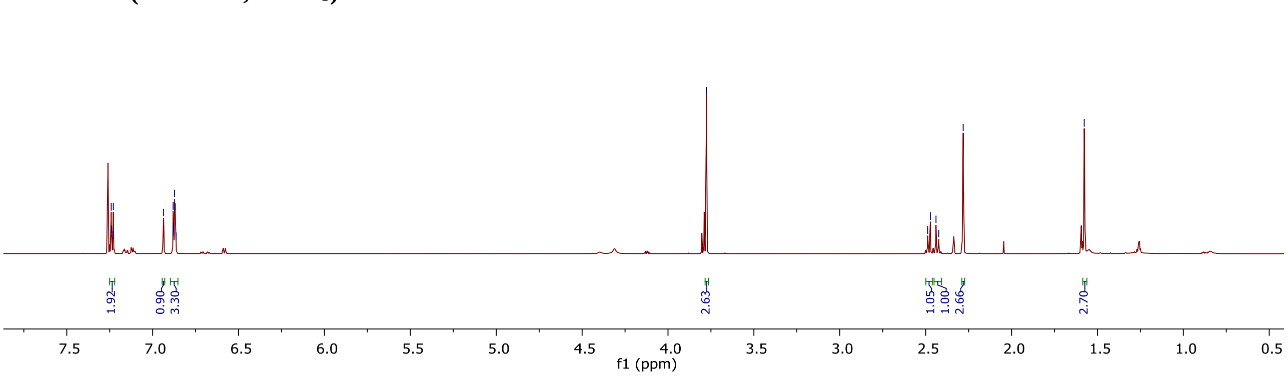

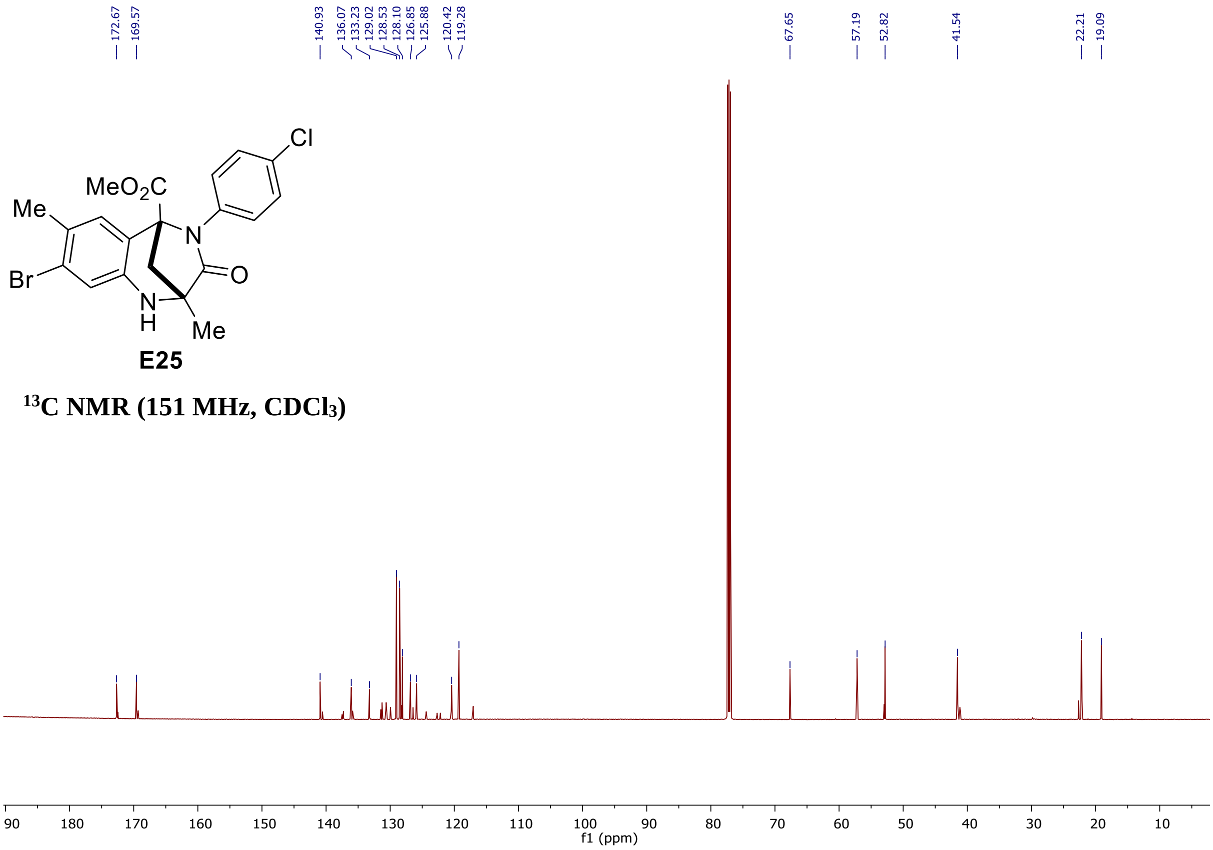

E25

^{13}C NMR (151 MHz, CDCl$_3$)

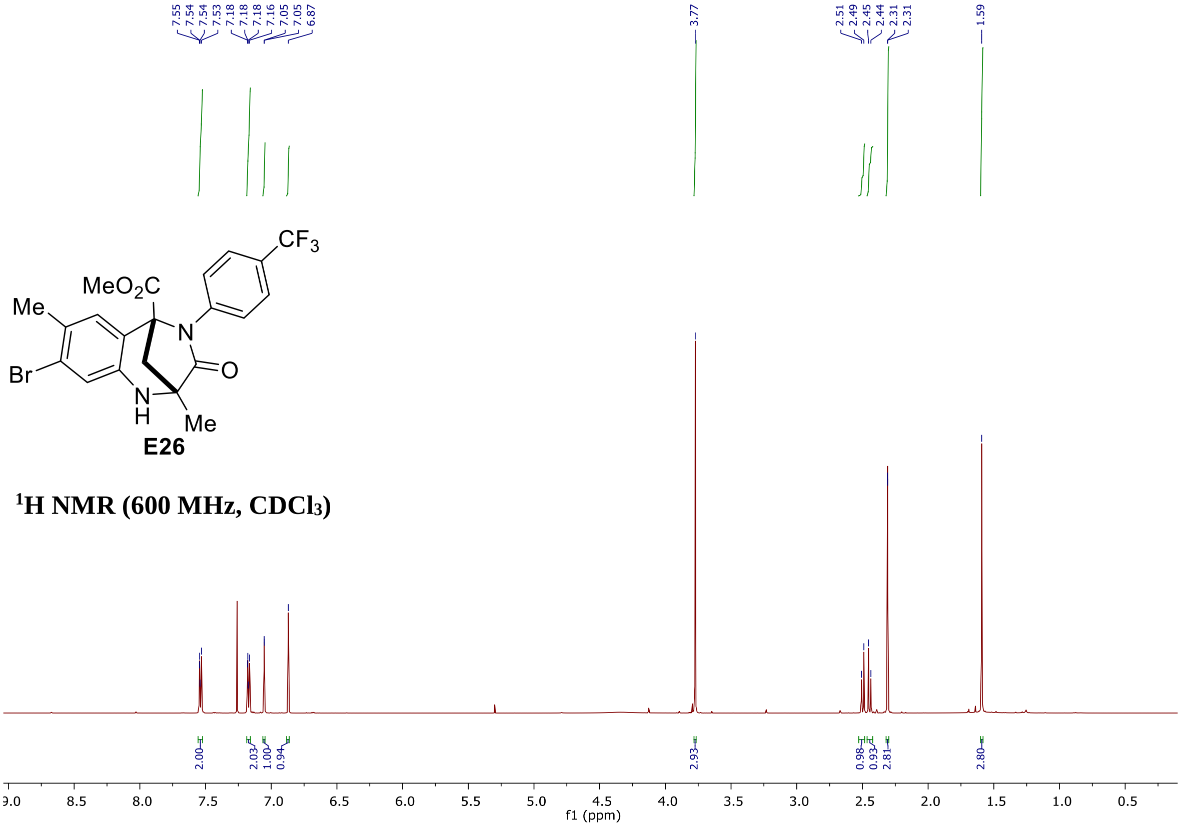

E26

^{1}H NMR (600 MHz, CDCl$_3$)

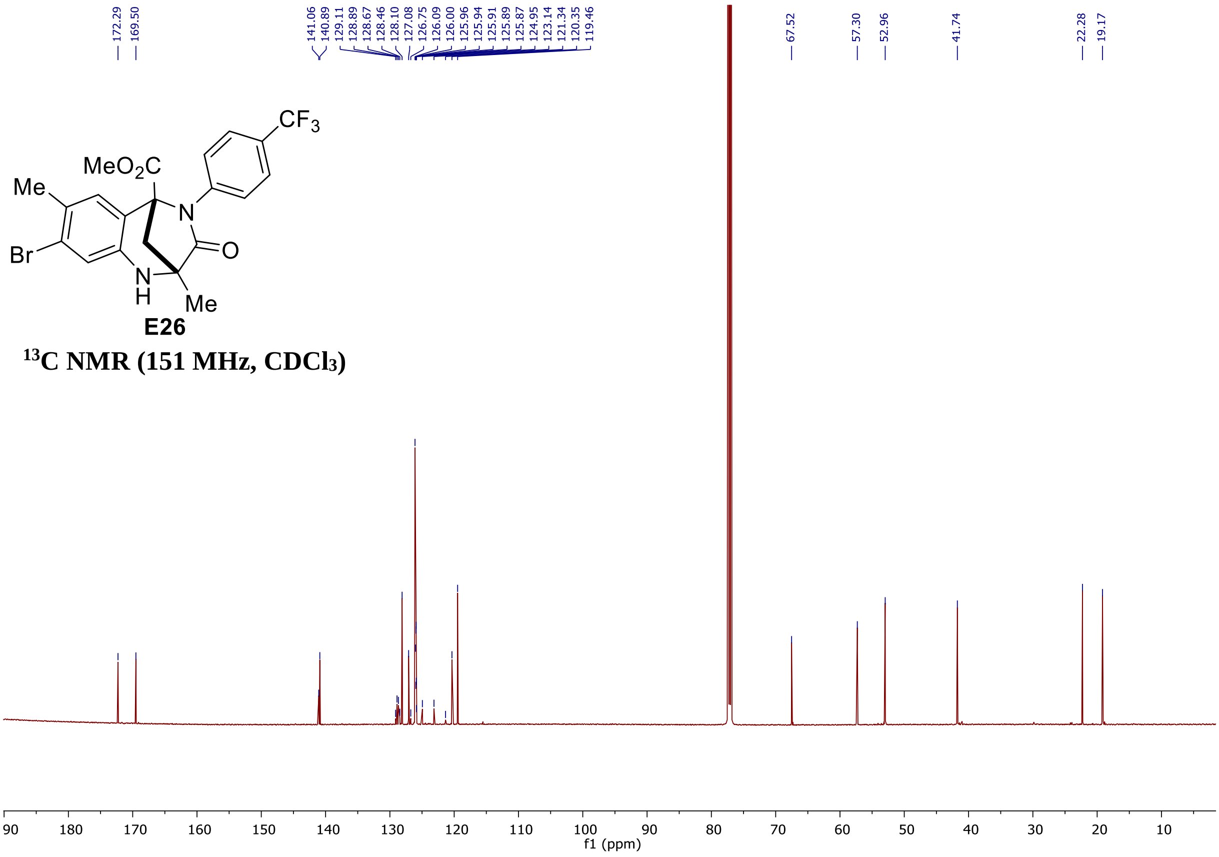

E26

^{13}C NMR (151 MHz, CDCl3)

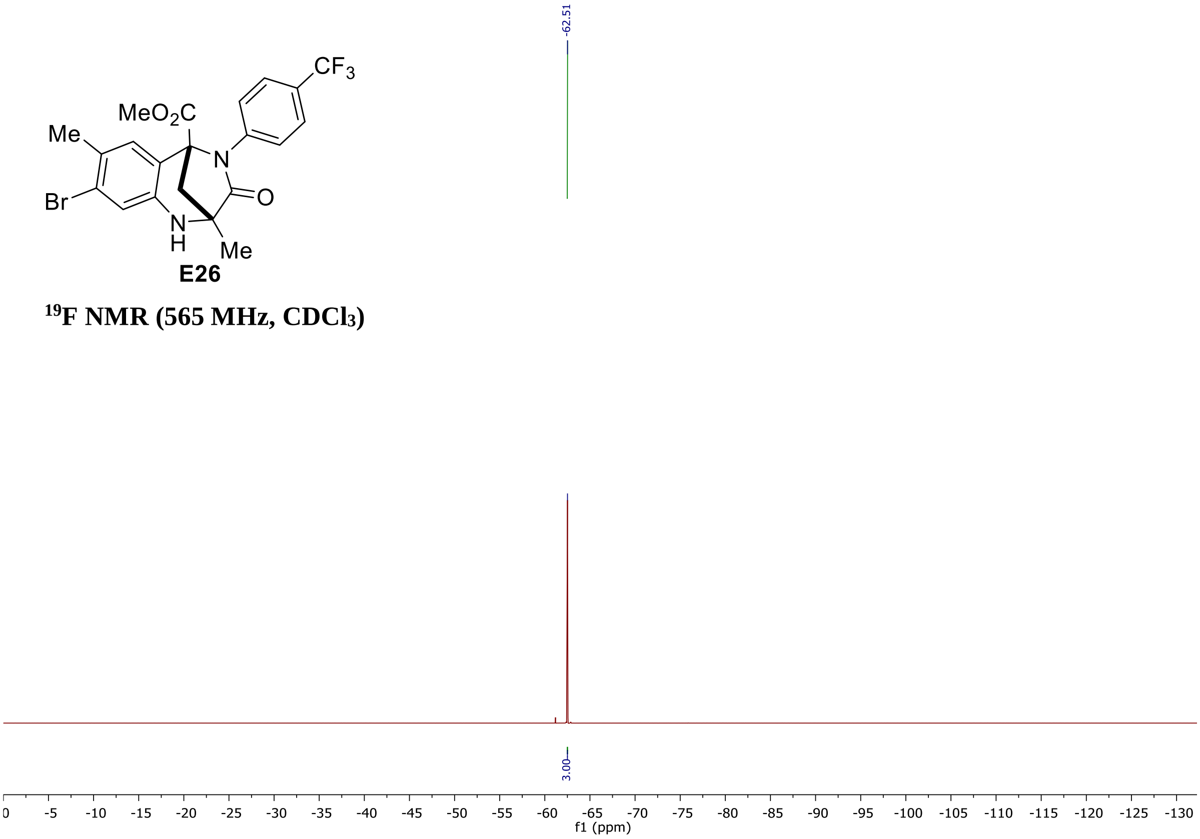

E26

^{19}F NMR (565 MHz, CDCl3)

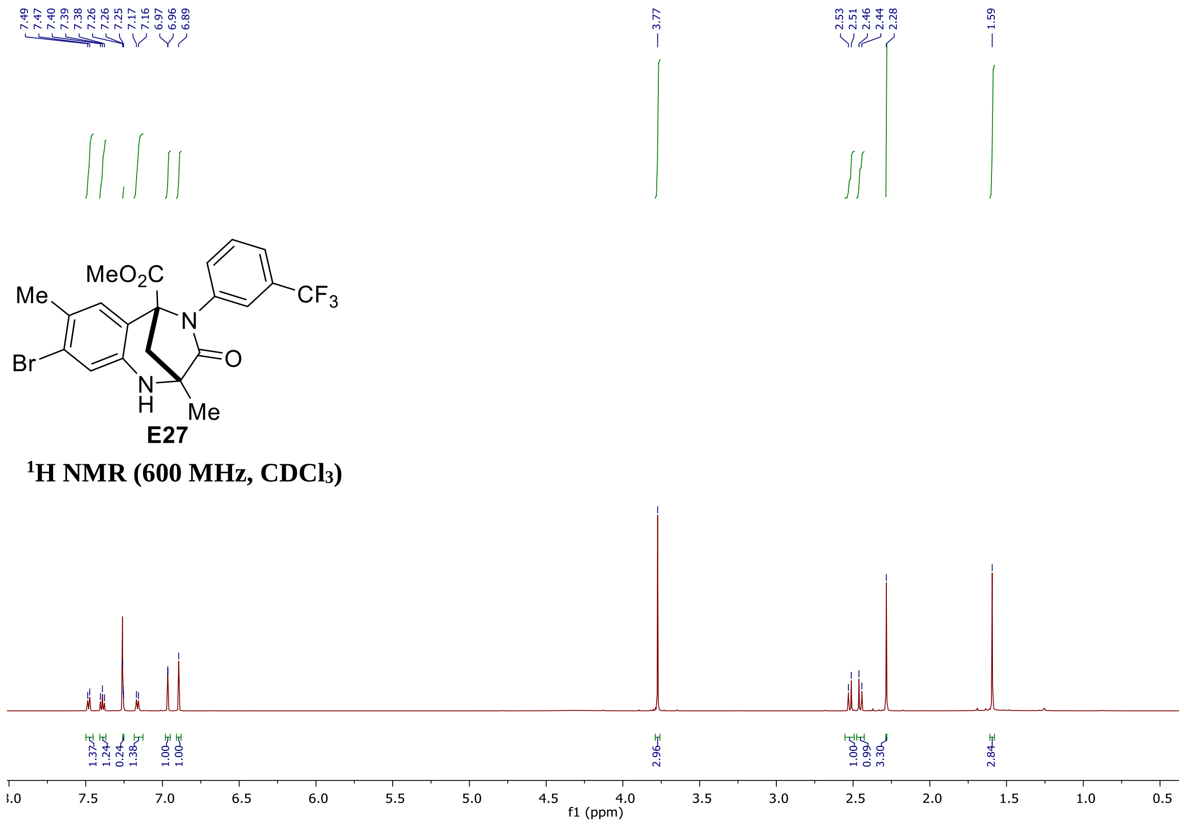
MeO2C
Me
Br
N
O
CF3
N
H
Me
E27
1H NMR (600 MHz, CDCl3)
f1 (ppm)

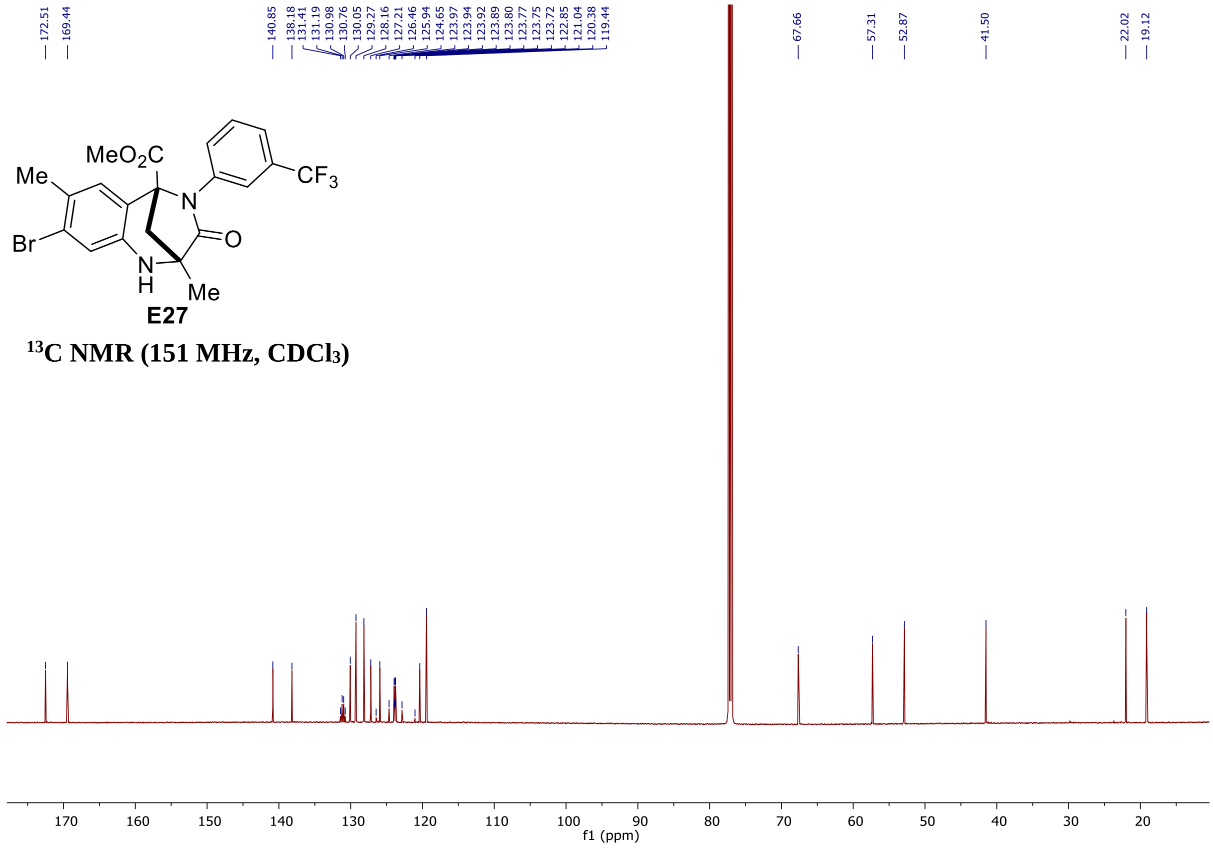
MeO2C
Me
Br
N
O
CF3
N
H
Me
E27
13C NMR (151 MHz, CDCl3)
f1 (ppm)

E27

^{19}F NMR (565 MHz, CDCl₃)

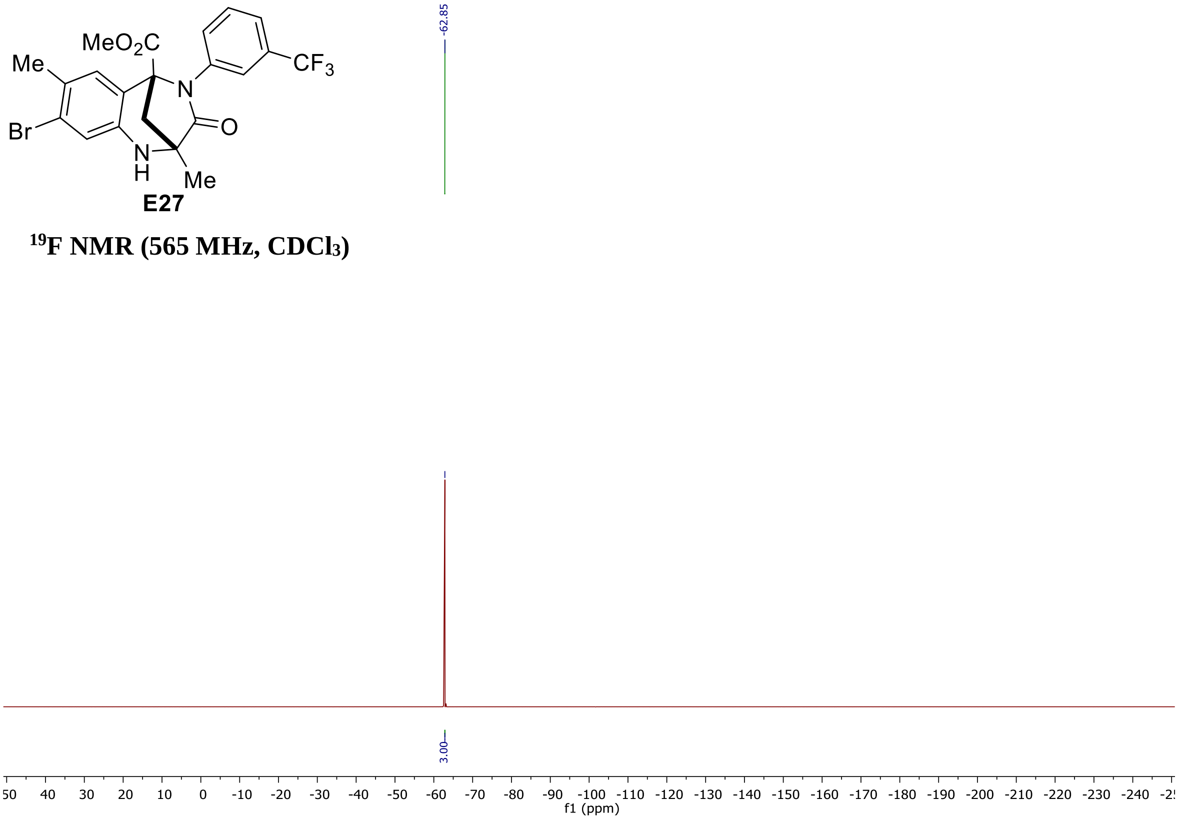

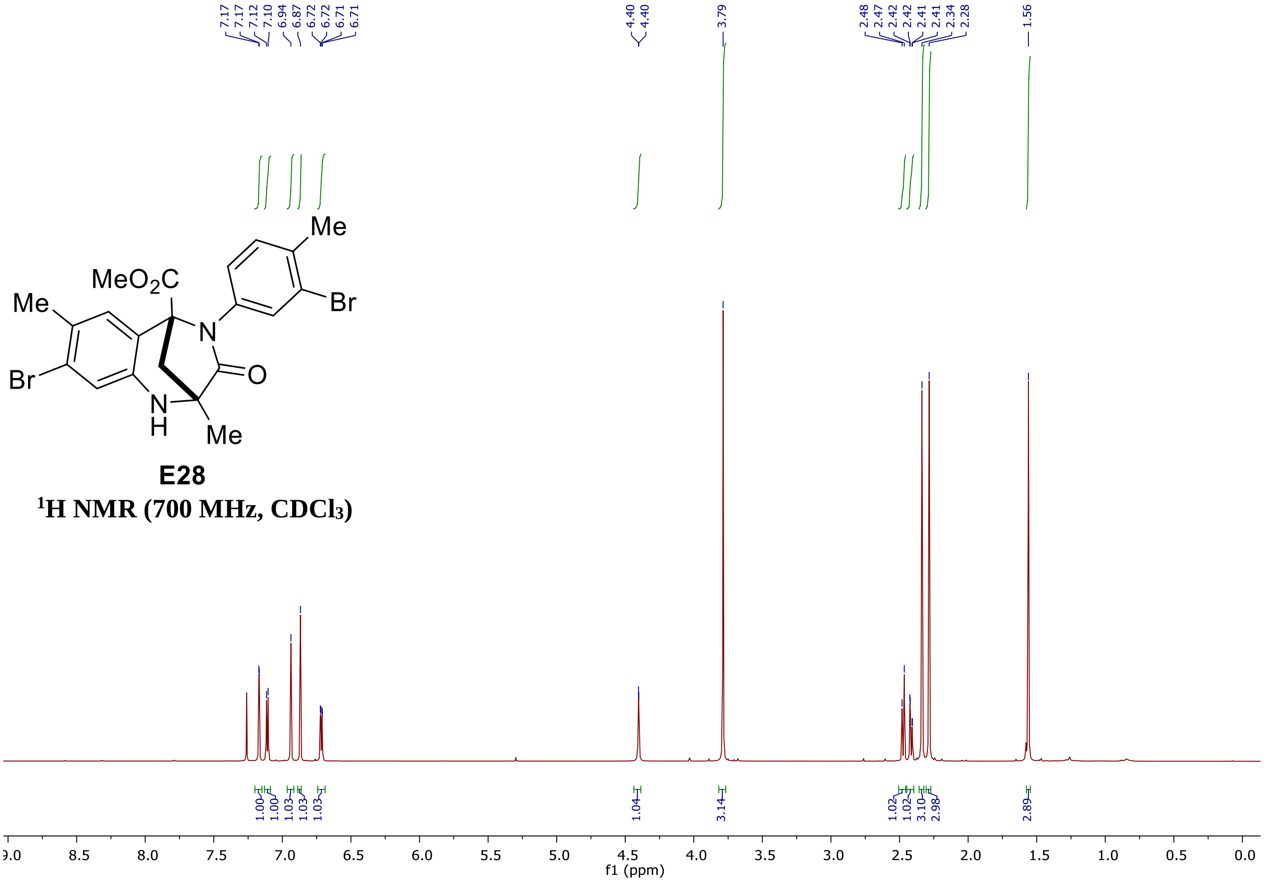

E28

^{1}H NMR (700 MHz, CDCl₃)

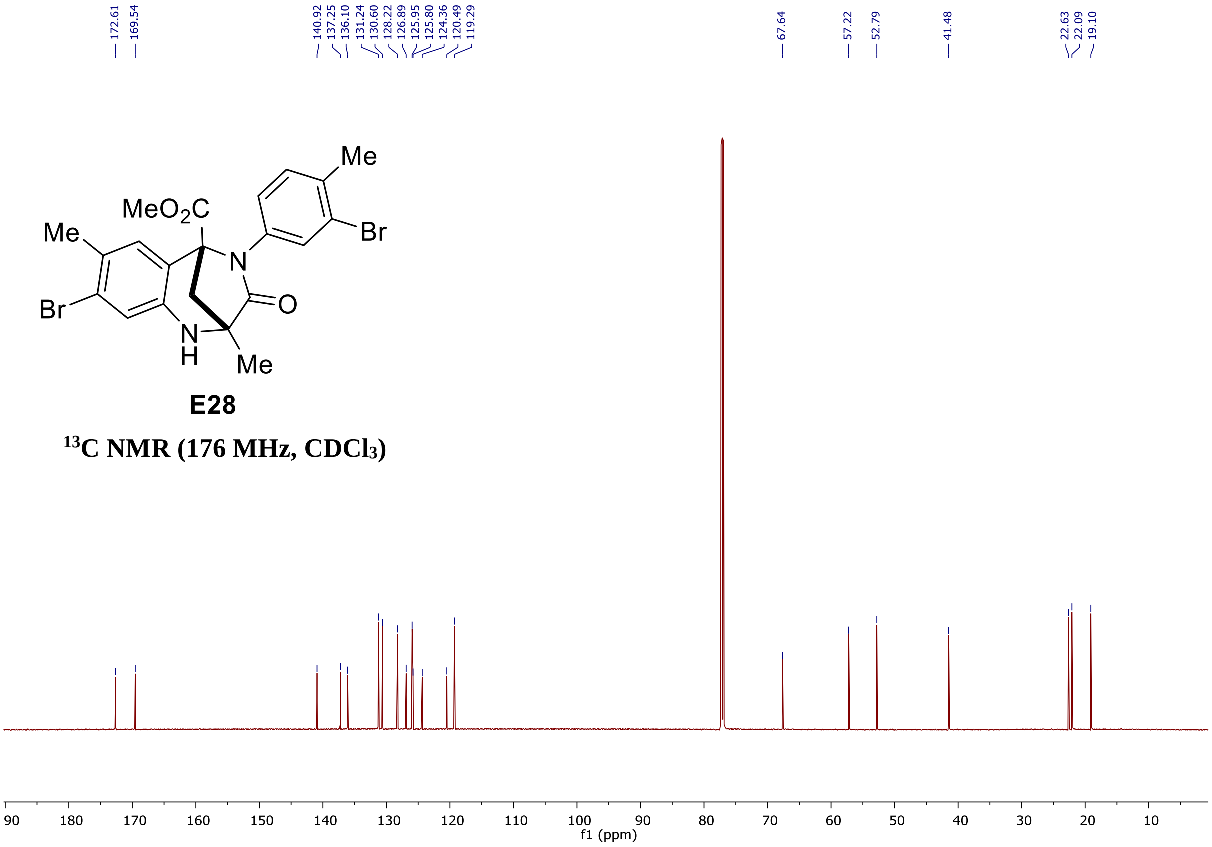
172.61
169.54
140.92
137.25
136.10
131.24
130.60
128.22
126.89
125.95
125.80
124.36
120.49
119.29
67.64
57.22
52.79
41.48
22.63
22.09
19.10
MeO2C
Me
Me
Br
Br
N
N
H
O
Me
E28
13C NMR (176 MHz, CDCl3)
f1 (ppm)

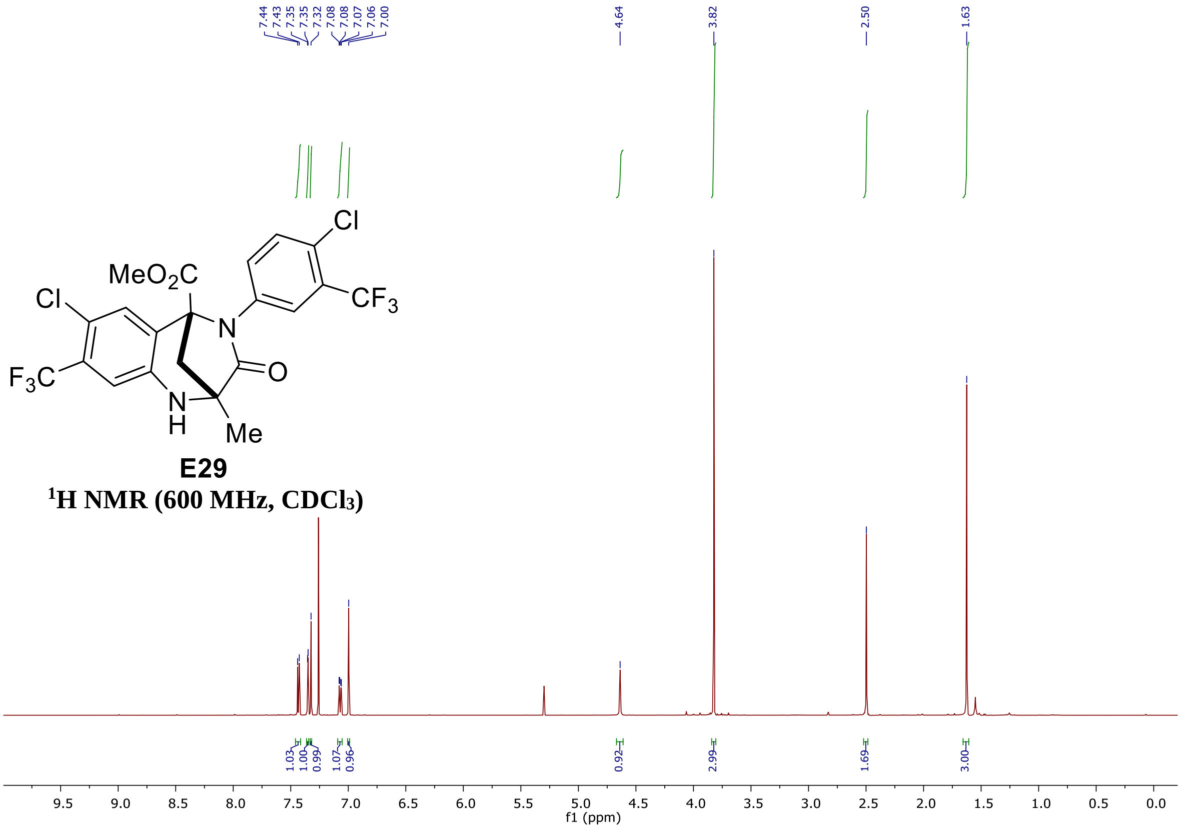
7.44
7.43
7.35
7.35
7.32
7.08
7.08
7.07
7.06
7.00
4.64
3.82
2.50
1.63
Cl
MeO2C
Cl
CF3
F3C
N
N
H
O
Me
E29
1H NMR (600 MHz, CDCl3)
1.03
1.00
0.99
1.07
0.96
0.92
2.99
1.69
3.00
f1 (ppm)

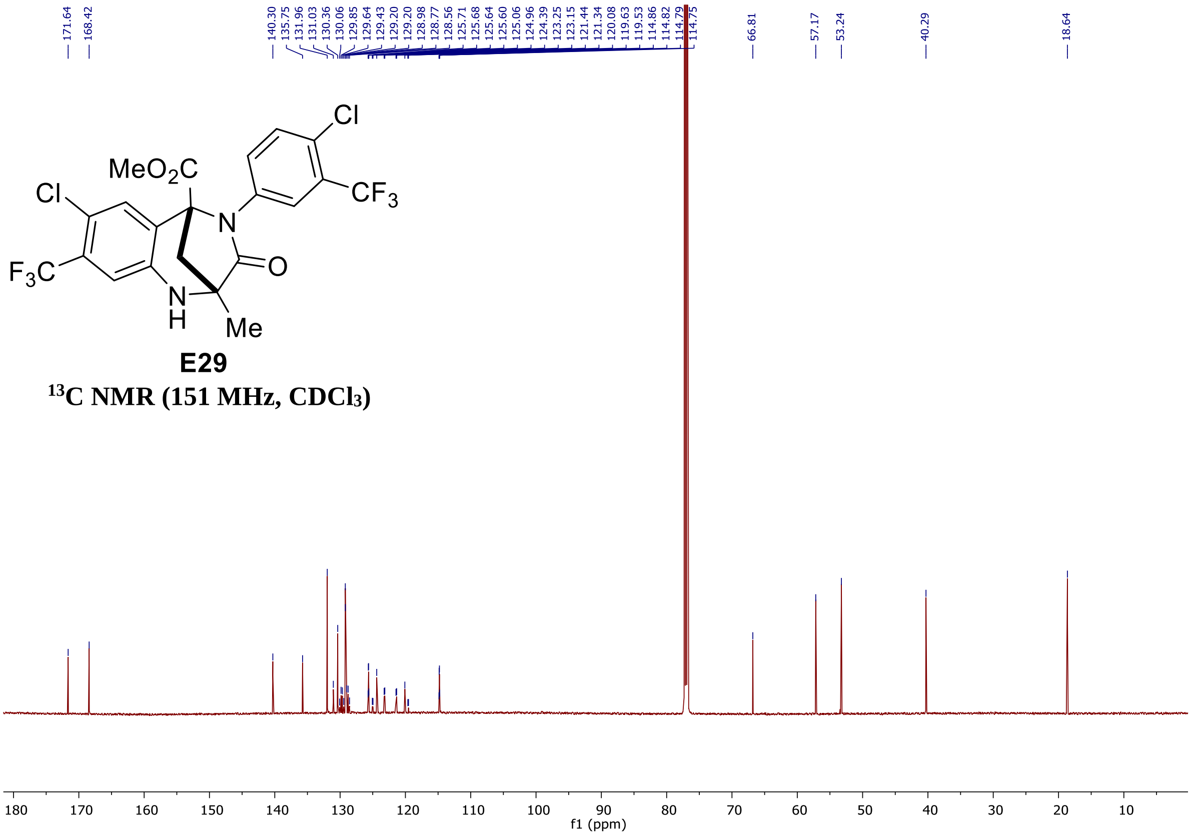

E29

^{13}C NMR (151 MHz, $CDCl_3$)

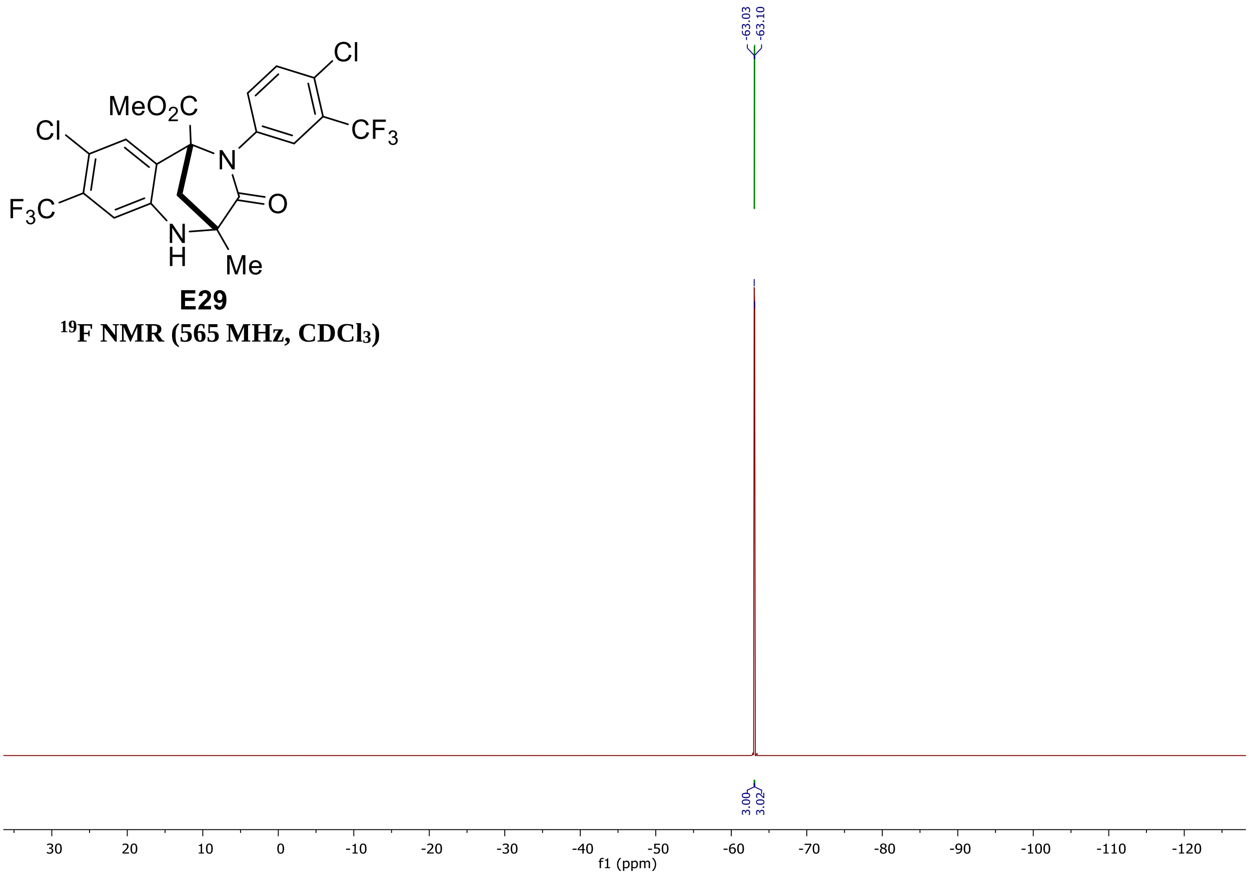

E29

^{19}F NMR (565 MHz, $CDCl_3$)

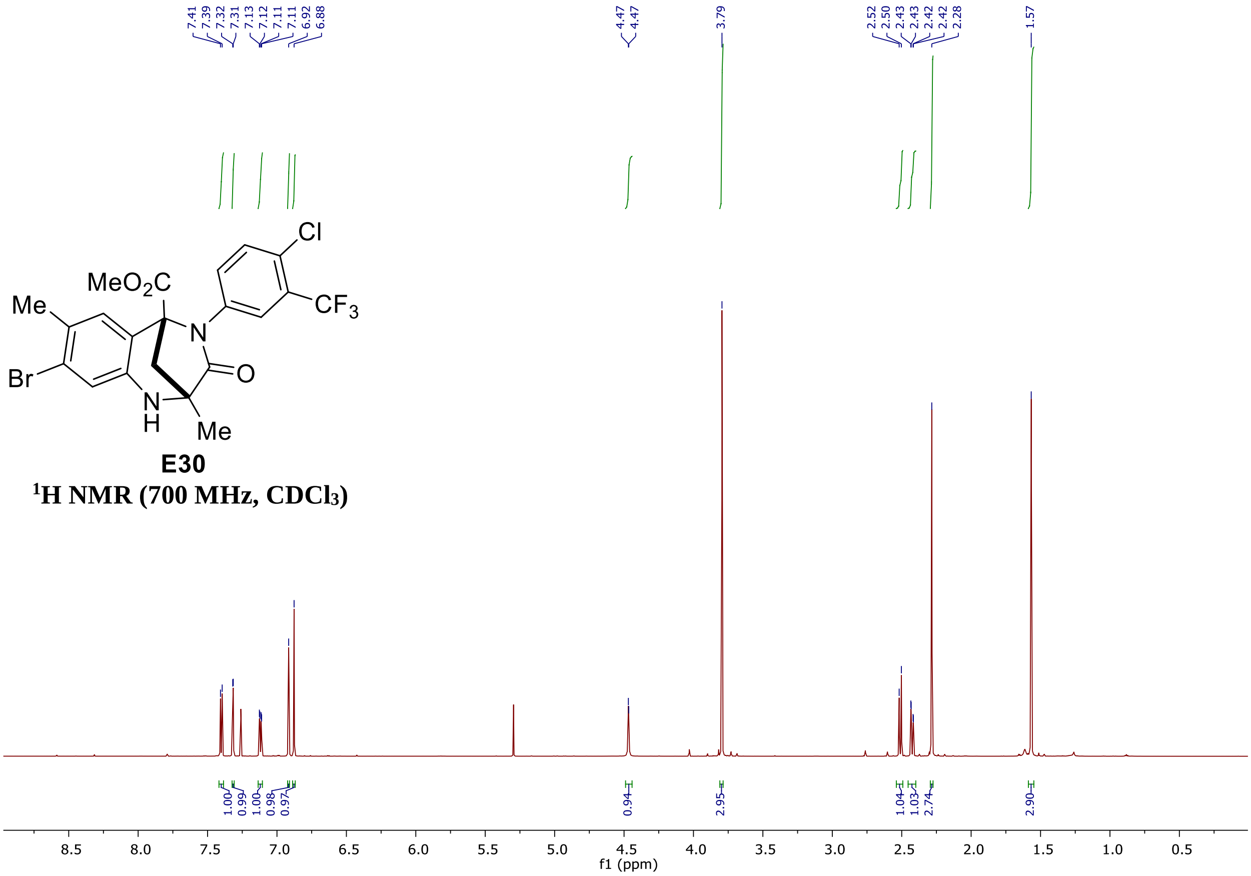
E30
^{1}H NMR (700 MHz, CDCl$_3$)
f1 (ppm)

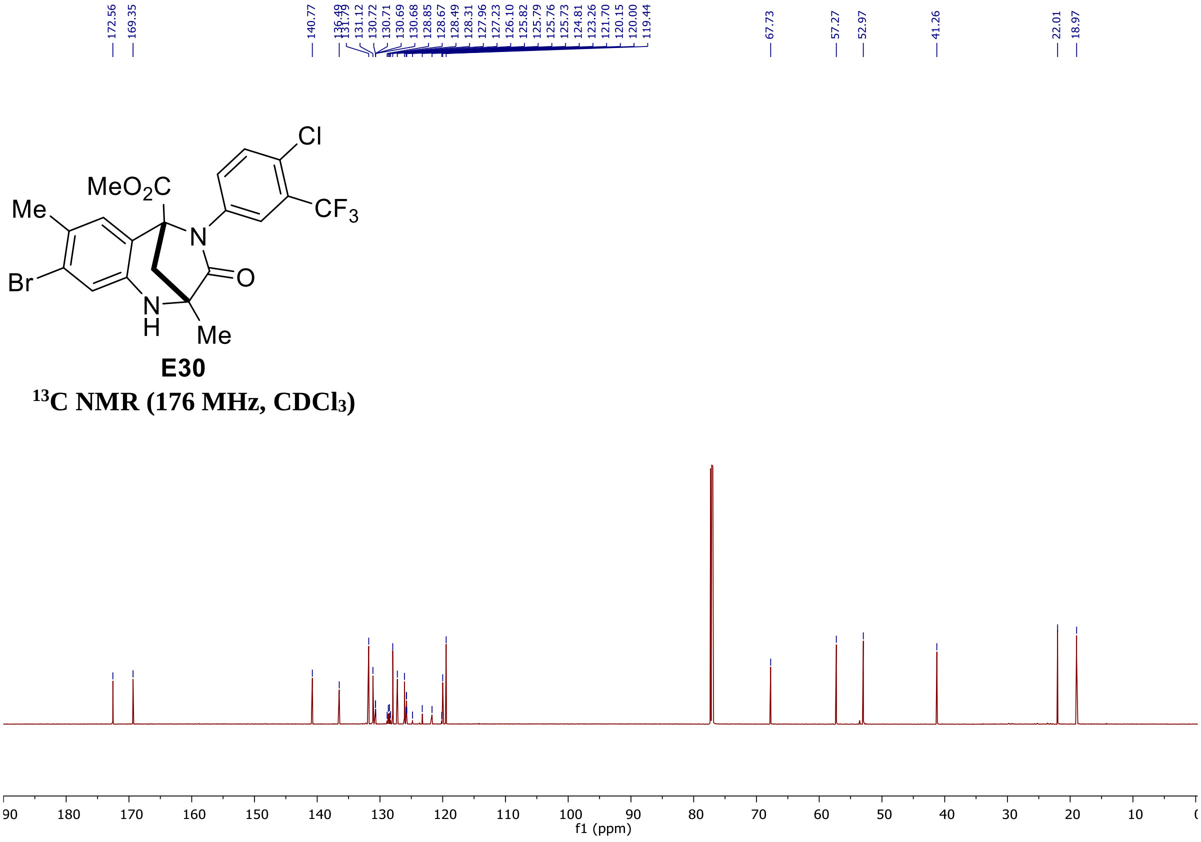
E30
^{13}C NMR (176 MHz, CDCl$_3$)
f1 (ppm)

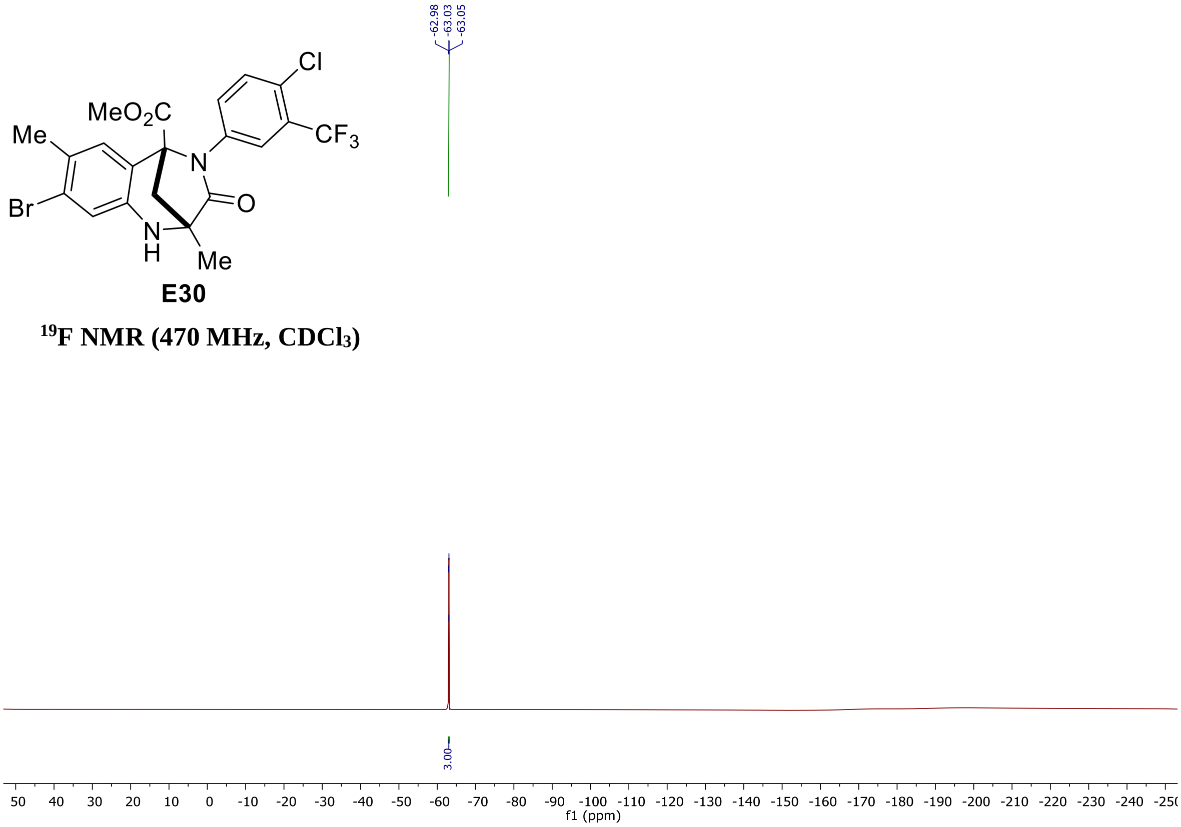

E30

^{19}F NMR (470 MHz, $CDCl_3$)

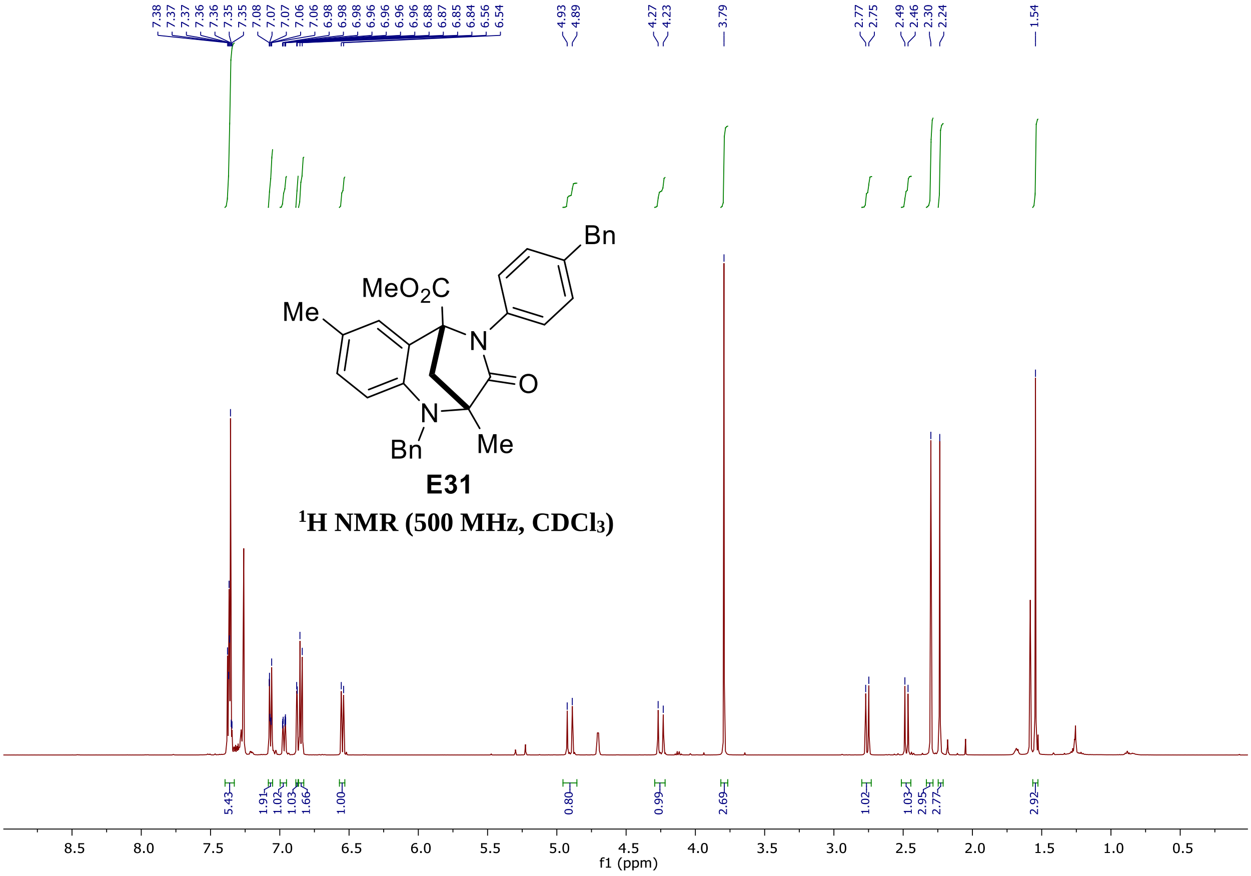

E31

^{1}H NMR (500 MHz, $CDCl_3$)

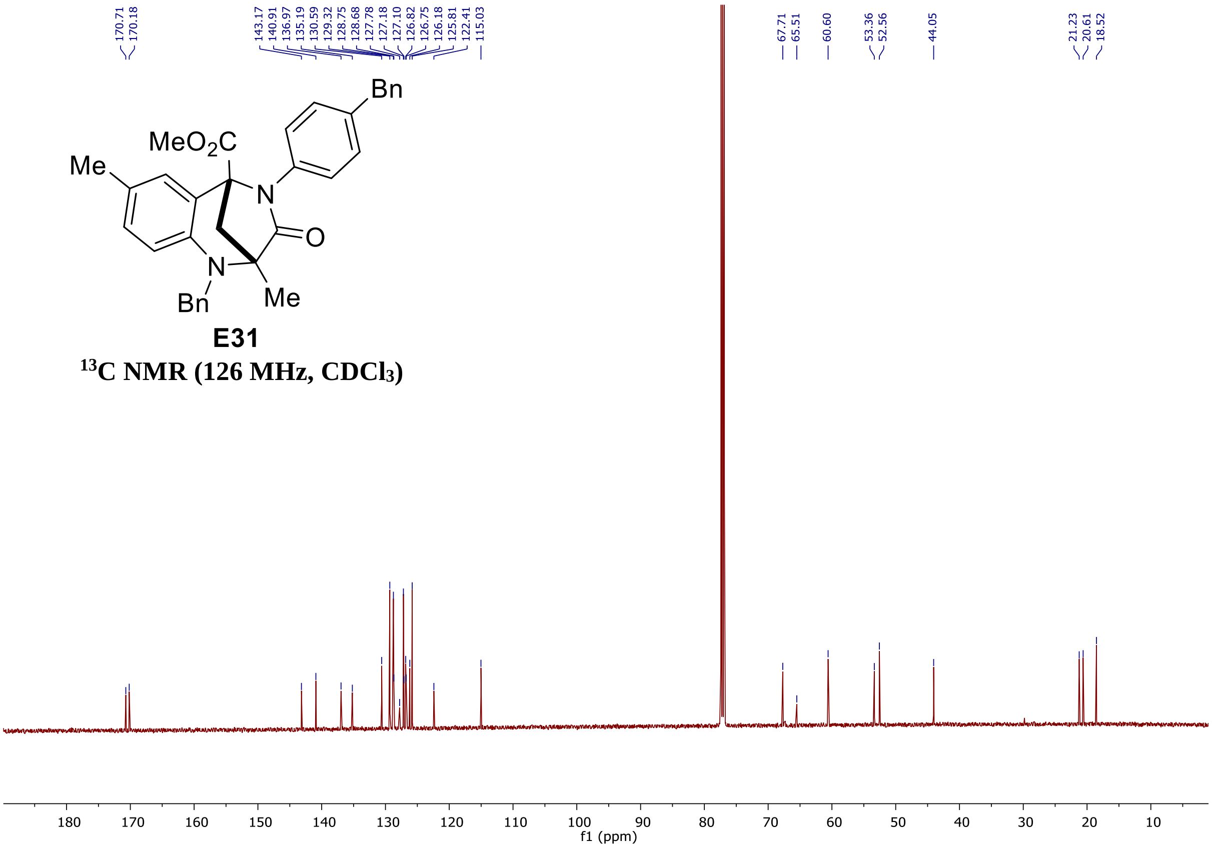

Bn
MeO_2C
Me
N
O
N
Bn
Me
E31
^{13}C NMR (126 MHz, $CDCl_3$)
170.71
170.18
143.17
140.91
136.97
135.19
130.59
129.32
128.75
128.68
127.78
127.18
127.10
126.82
126.75
126.18
125.81
122.41
115.03
67.71
65.51
60.60
53.36
52.56
44.05
21.23
20.61
18.52
f1 (ppm)

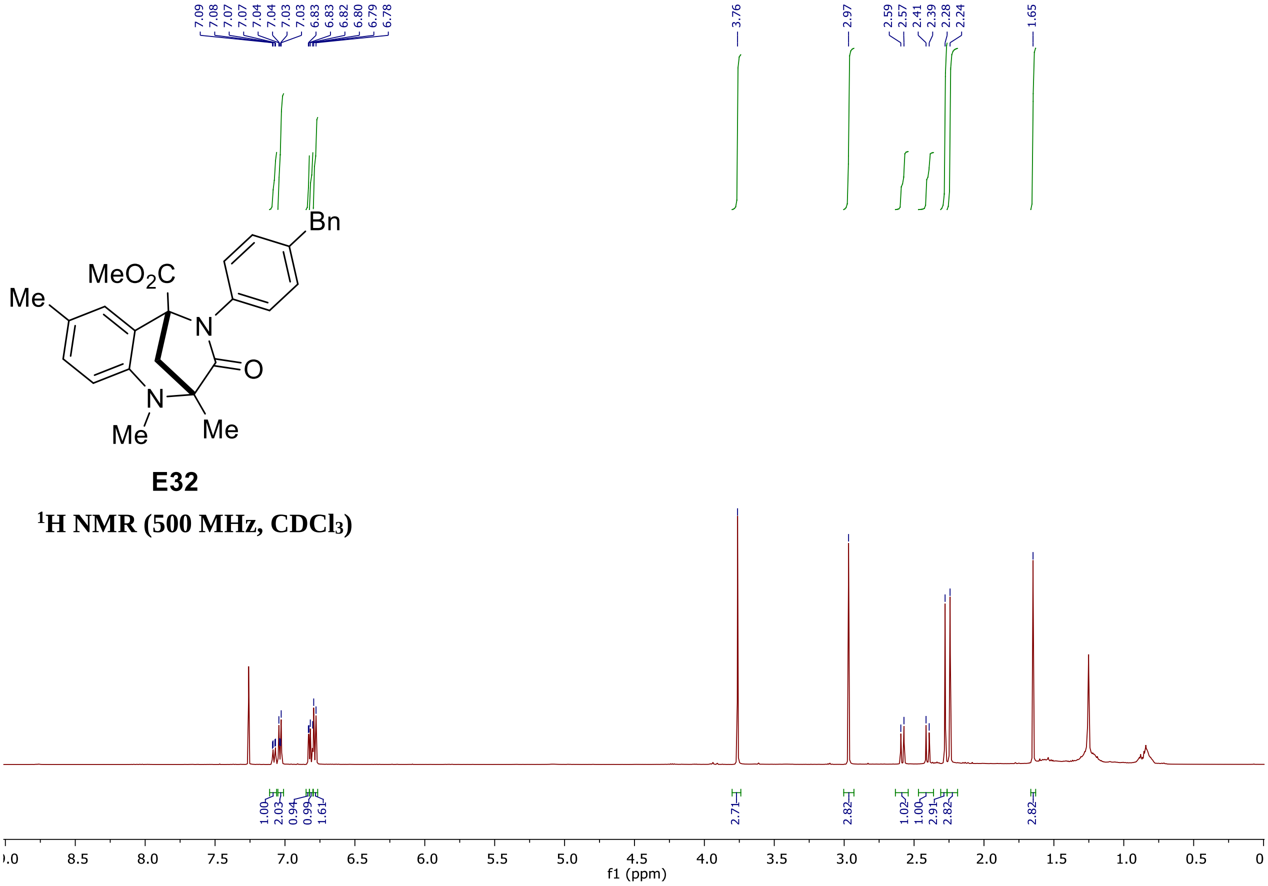

Bn
MeO_2C
Me
N
O
N
Me
Me
E32
^{1}H NMR (500 MHz, $CDCl_3$)
7.09
7.08
7.07
7.07
7.04
7.04
7.03
7.03
6.83
6.83
6.82
6.80
6.79
6.78
3.76
2.97
2.59
2.57
2.41
2.39
2.28
2.24
1.65
1.00
2.03
0.94
0.99
1.61
2.71
2.82
1.02
1.00
2.91
2.82
2.82
f1 (ppm)

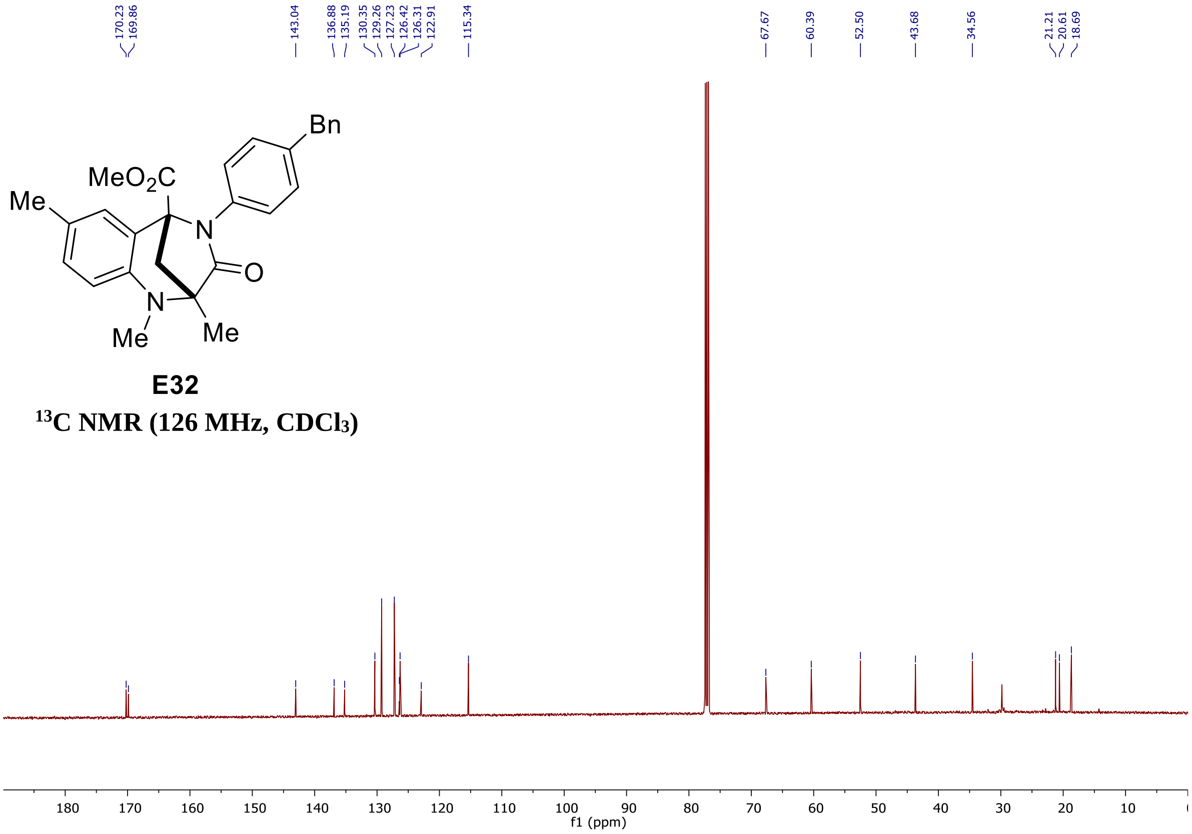

E32

^{13}C NMR (126 MHz, $CDCl_3$)

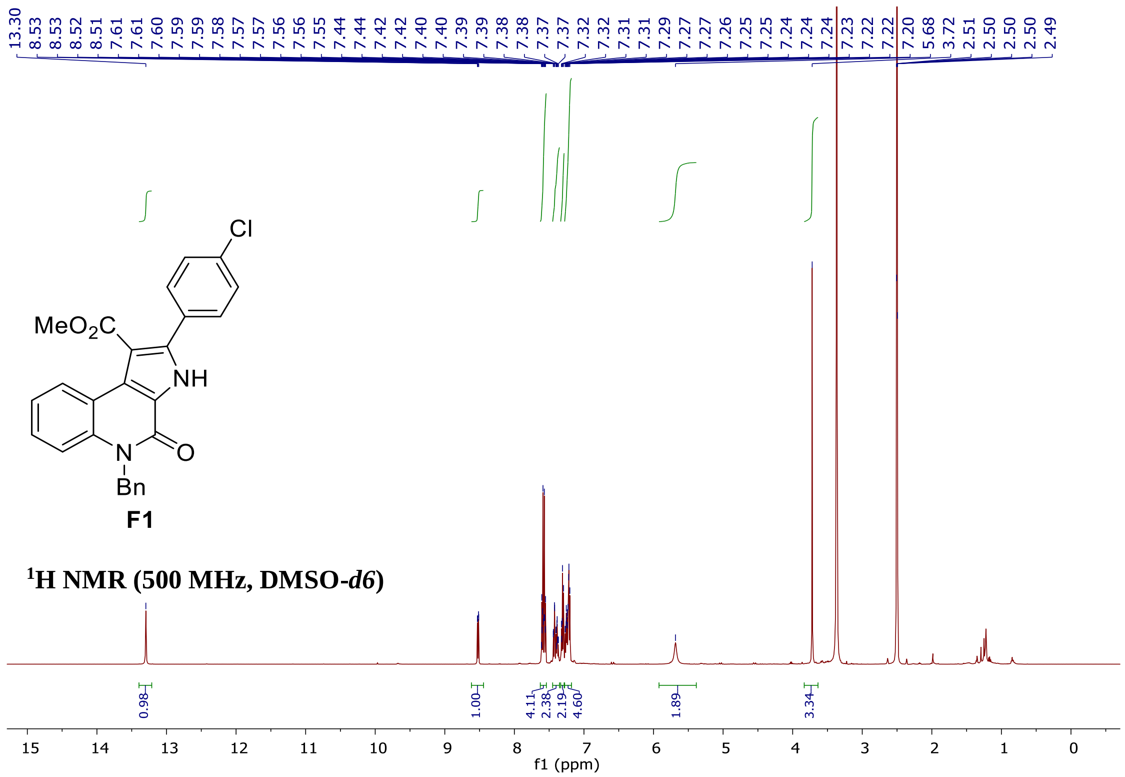
Cl
MeO2C
NH
N
O
Bn
F1
1H NMR (500 MHz, DMSO-d6)
f1 (ppm)

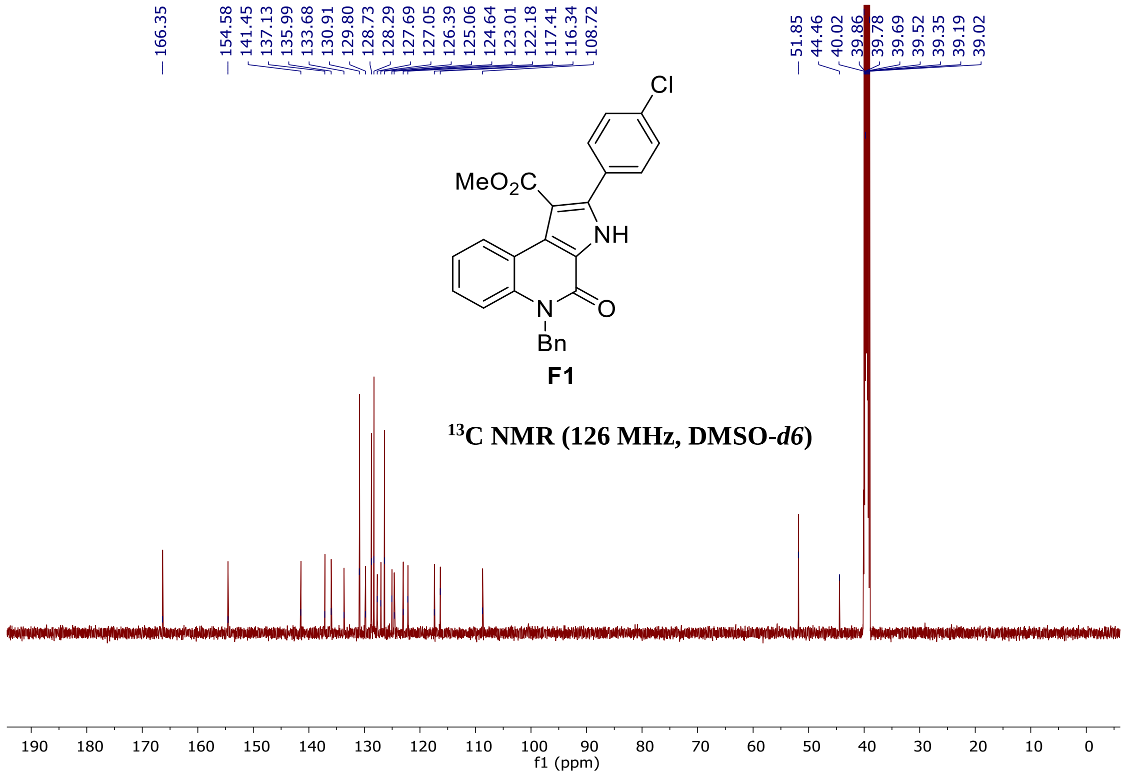
Cl
MeO2C
NH
N
O
Bn
F1
13C NMR (126 MHz, DMSO-d6)
f1 (ppm)

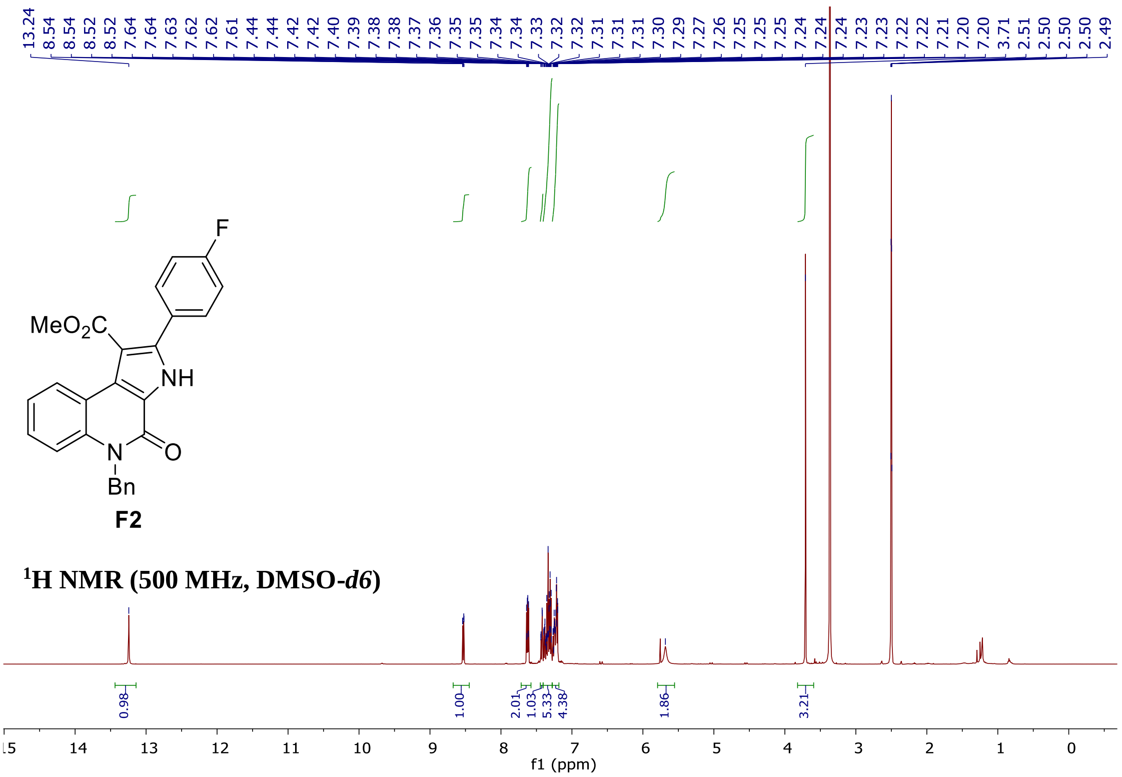

F2

^{1}H NMR (500 MHz, DMSO-*d6*)

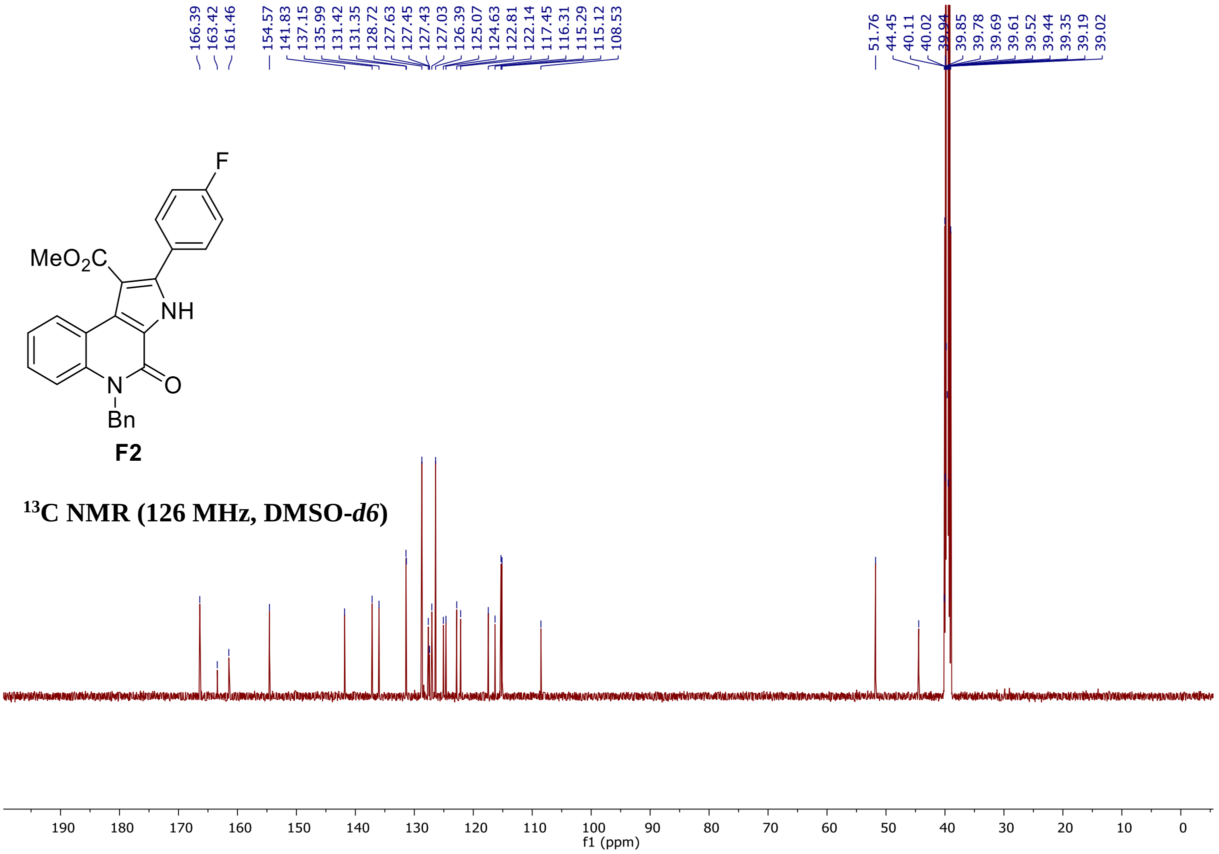

F2

^{13}C NMR (126 MHz, DMSO-*d6*)

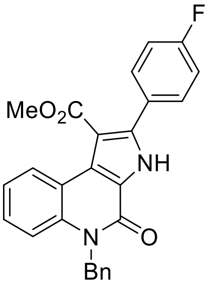

F2

^{19}F NMR (470 MHz, DMSO-*d6*)

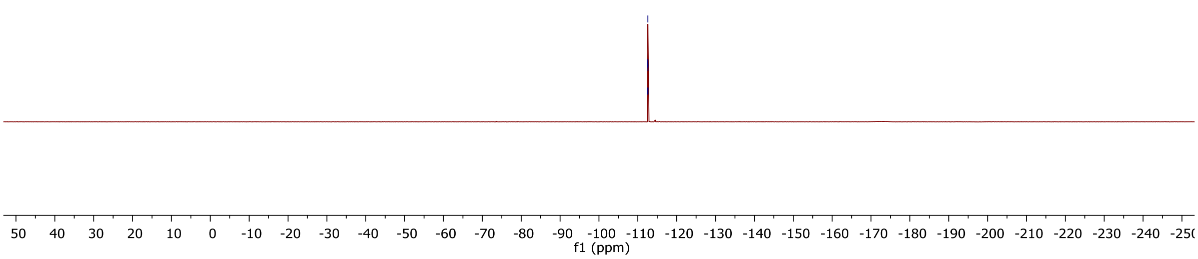

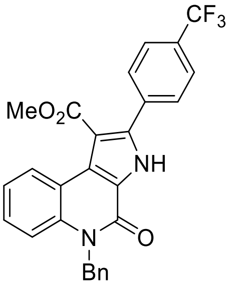

F3

^{19}F NMR (470 MHz, DMSO-*d6*)

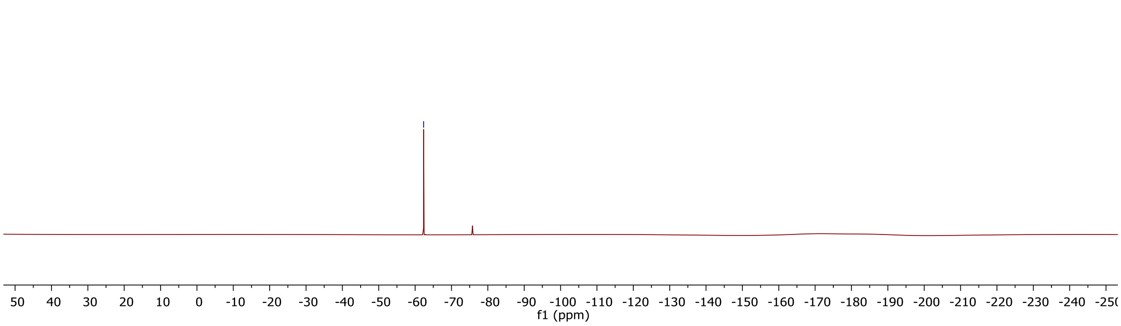

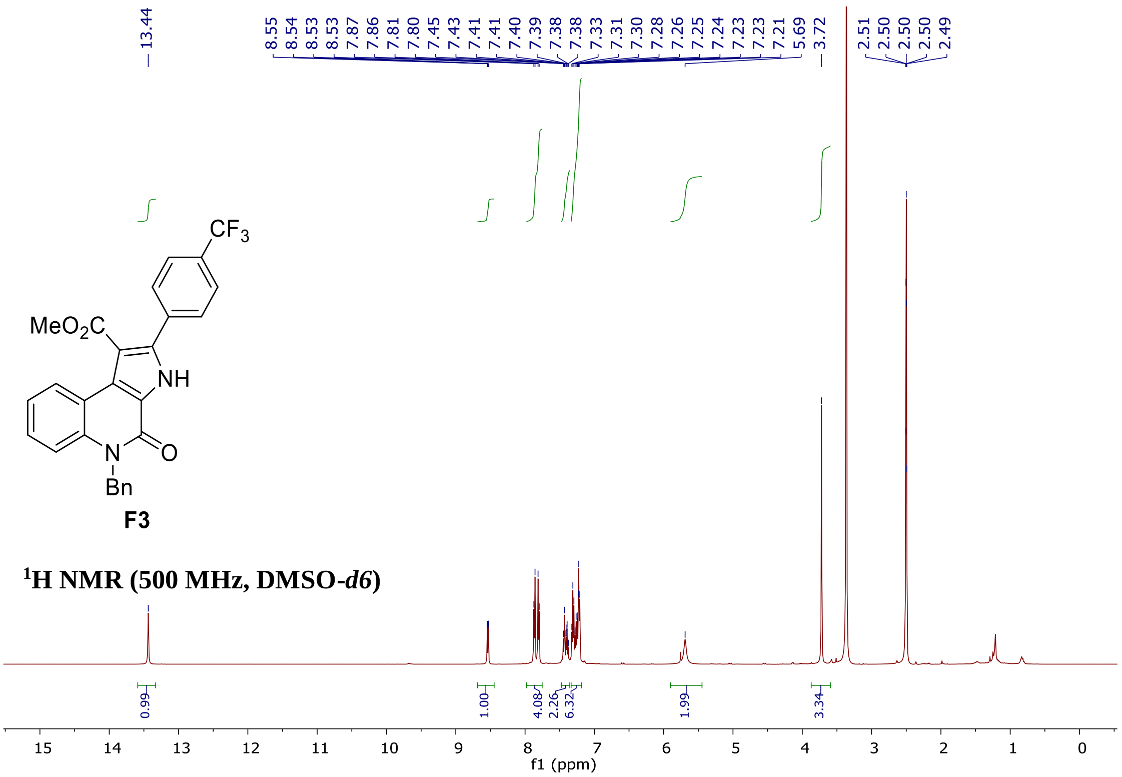
13.44
8.55 8.54 8.53 8.53 7.87 7.86 7.81 7.80 7.45 7.43 7.41 7.41 7.40 7.39 7.38 7.38 7.33 7.31 7.30 7.28 7.26 7.25 7.24 7.23 7.23 7.21 5.69 3.72
2.51 2.50 2.50 2.50 2.49
CF3
MeO2C
NH
N
O
Bn
F3
1H NMR (500 MHz, DMSO-d6)
0.99
1.00
4.08
2.26
6.32
1.99
3.34
15 14 13 12 11 10 9 8 7 6 5 4 3 2 1 0
f1 (ppm)

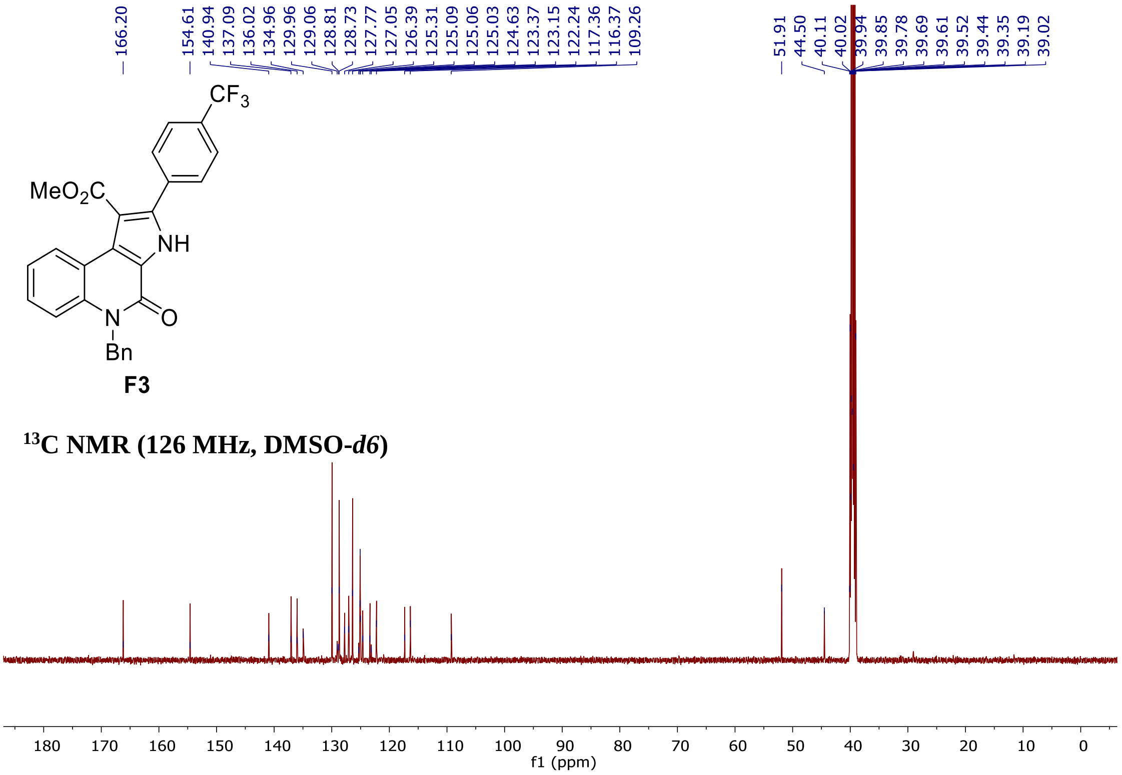
166.20
154.61 140.94 137.09 136.02 134.96 129.96 129.06 128.81 128.73 127.77 127.05 126.39 125.31 125.09 125.06 125.03 124.63 123.37 123.15 122.24 117.36 116.37 109.26
51.91 44.50 40.11 40.02 39.94 39.85 39.78 39.69 39.61 39.52 39.44 39.35 39.19 39.02
CF3
MeO2C
NH
N
O
Bn
F3
13C NMR (126 MHz, DMSO-d6)
180 170 160 150 140 130 120 110 100 90 80 70 60 50 40 30 20 10 0
f1 (ppm)

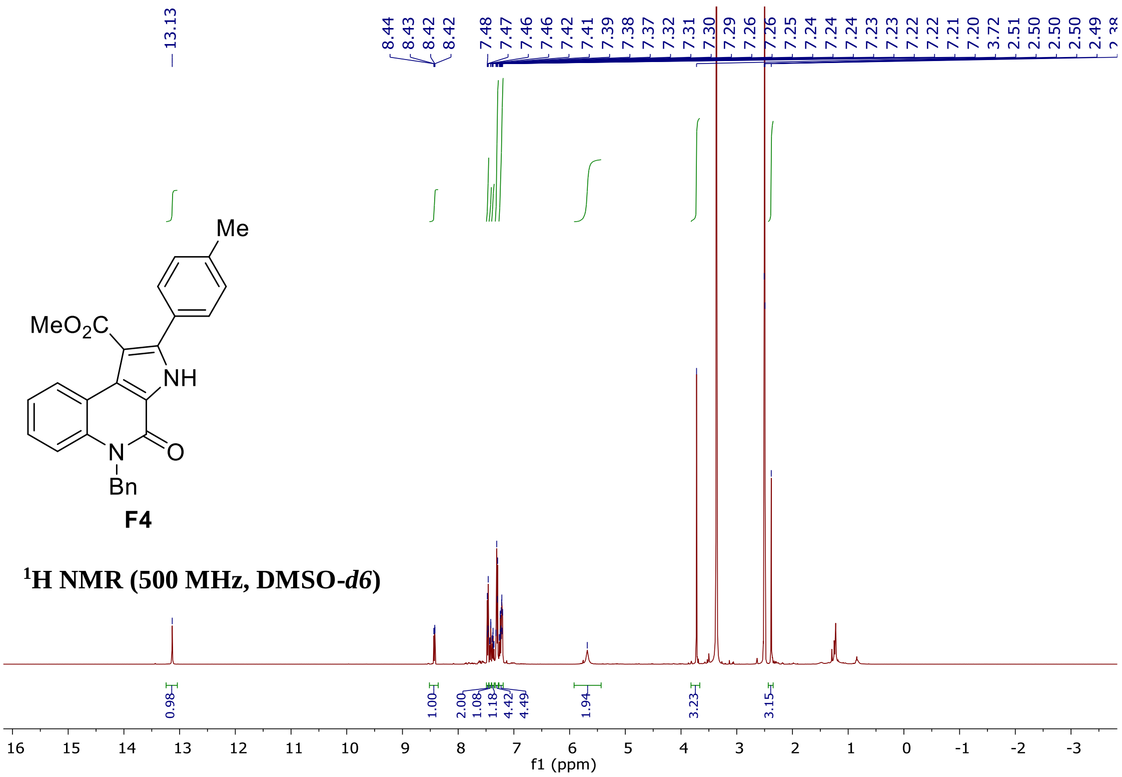
13.13
8.44
8.43
8.42
8.42
7.48
7.47
7.46
7.46
7.42
7.41
7.39
7.38
7.37
7.32
7.31
7.30
7.29
7.26
7.26
7.25
7.24
7.24
7.24
7.23
7.23
7.22
7.22
7.21
7.20
3.72
2.51
2.50
2.50
2.50
2.49
Me
MeO2C
NH
N
O
Bn
F4
1H NMR (500 MHz, DMSO-d6)
0.98
1.00
2.00
1.08
1.18
4.42
4.49
1.94
3.23
3.15
f1 (ppm)

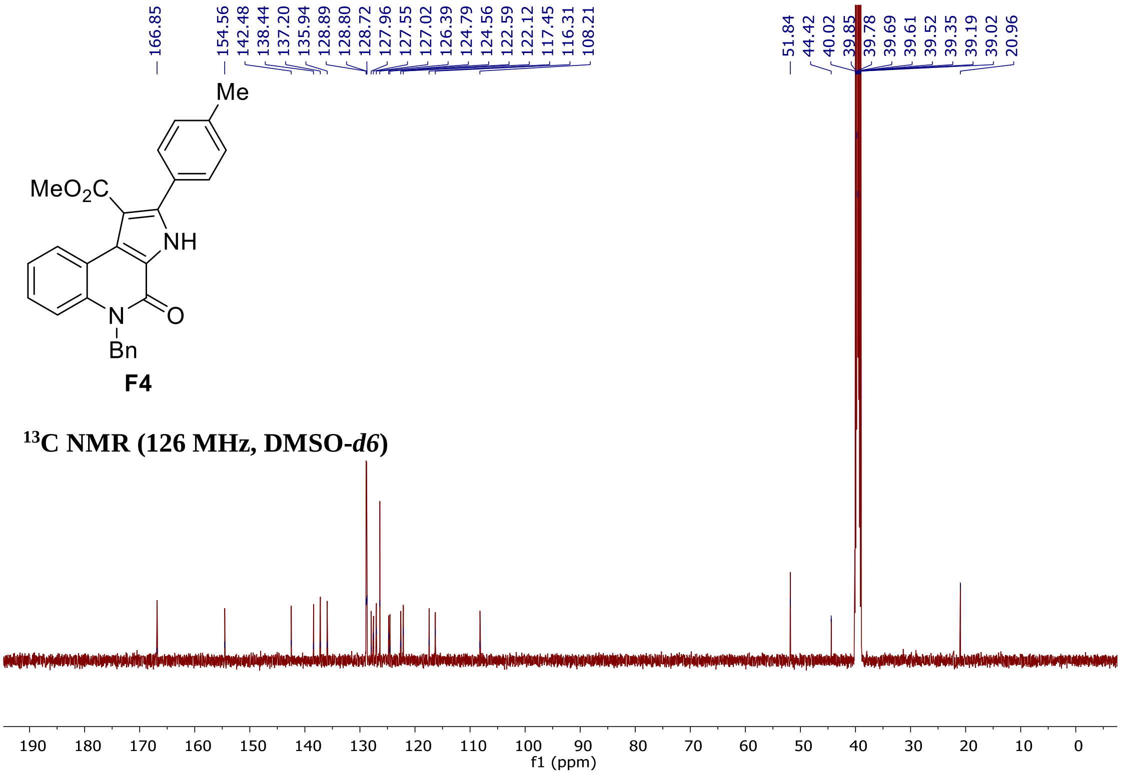
166.85
154.56
142.48
138.44
137.20
135.94
128.89
128.80
128.72
127.96
127.55
127.02
126.39
124.79
124.56
122.59
122.12
117.45
116.31
108.21
51.84
44.42
40.02
39.85
39.78
39.69
39.61
39.52
39.35
39.19
39.02
20.96
Me
MeO2C
NH
N
O
Bn
F4
13C NMR (126 MHz, DMSO-d6)
f1 (ppm)

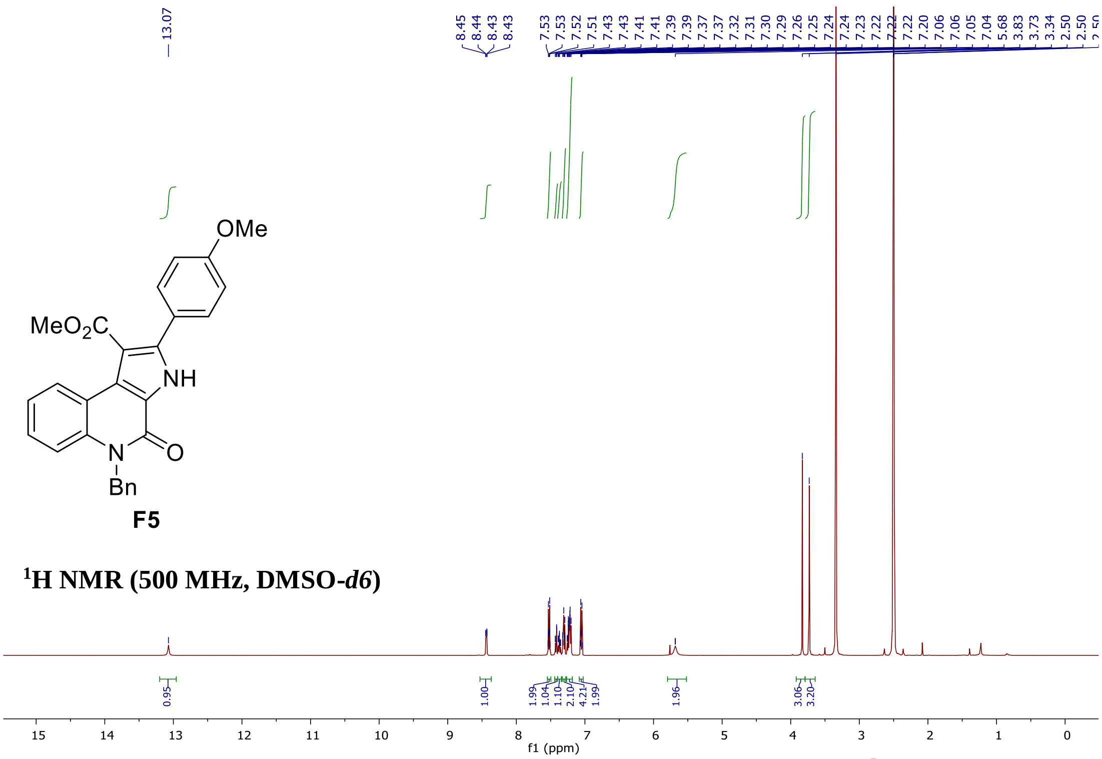
13.07
8.45 8.44 8.43 8.43
7.53 7.53 7.52 7.51 7.43 7.43 7.41 7.41 7.39 7.39 7.37 7.37 7.32 7.31 7.30 7.29 7.26 7.25 7.24 7.24 7.23 7.22 7.22 7.22 7.20 7.06 7.06 7.05 7.04 5.68 3.83 3.73 3.34 2.50 2.50
OMe
MeO_2C
NH
N
O
Bn
F5
^1H NMR (500 MHz, DMSO-*d6*)
0.95
1.00
1.99
1.04
1.10
2.10
4.21
1.99
1.96
3.06
3.20
15 14 13 12 11 10 9 8 7 6 5 4 3 2 1 0
f1 (ppm)

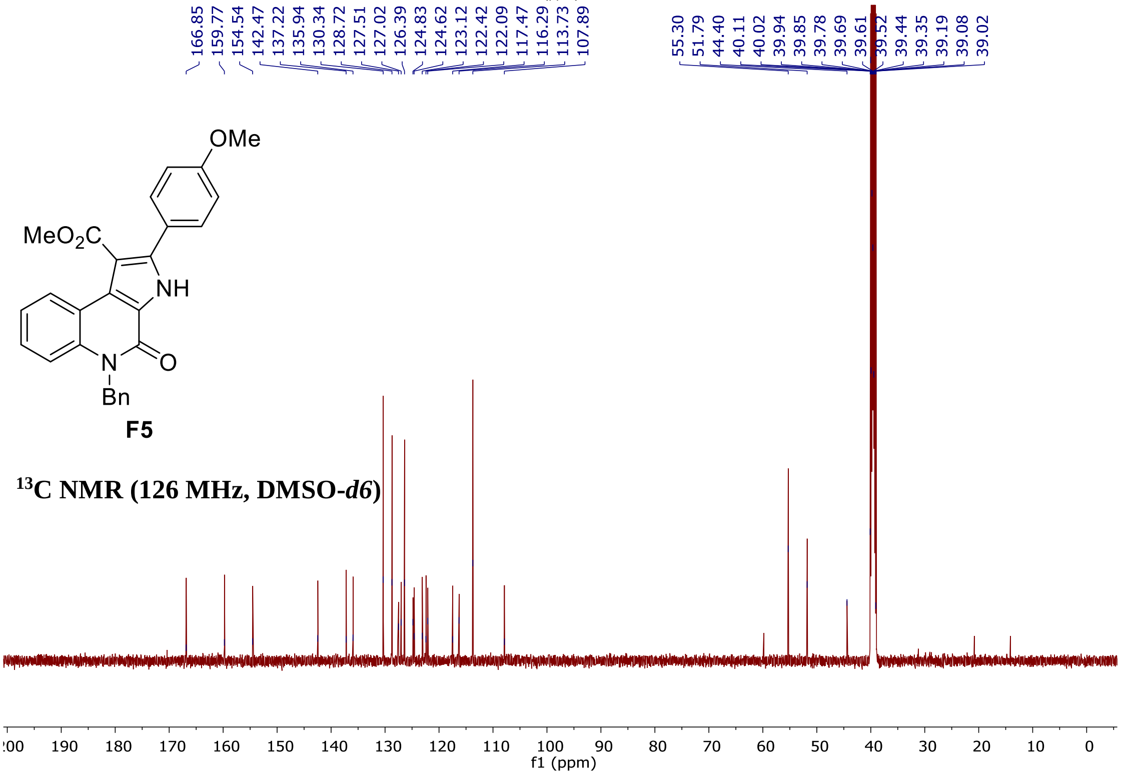
166.85 159.77 154.54 142.47 137.22 135.94 130.34 128.72 127.51 127.02 126.39 124.83 124.62 123.12 122.42 122.09 117.47 116.29 113.73 107.89
55.30 51.79 44.40 40.11 40.02 39.94 39.85 39.78 39.69 39.61 39.52 39.44 39.35 39.19 39.08 39.02
OMe
MeO_2C
NH
N
O
Bn
F5
^{13}C NMR (126 MHz, DMSO-*d6*)
200 190 180 170 160 150 140 130 120 110 100 90 80 70 60 50 40 30 20 10 0
f1 (ppm)

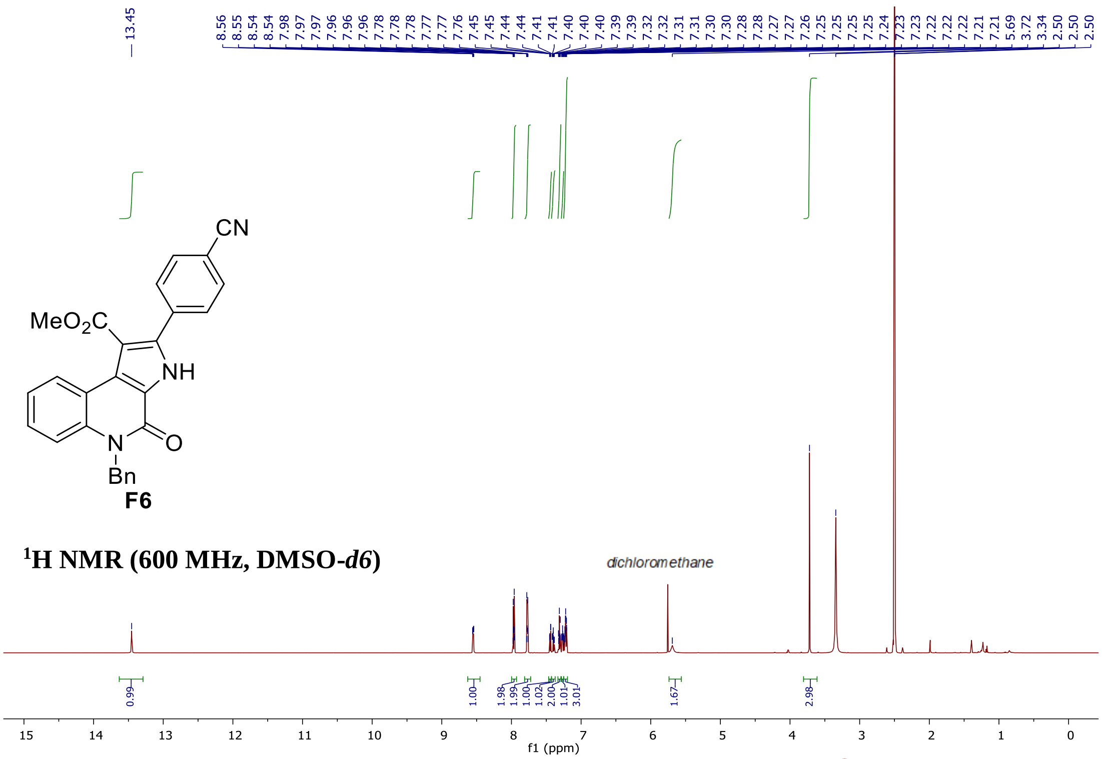
13.45
8.56 8.55 8.54 8.54 7.98 7.97 7.97 7.96 7.96 7.96 7.78 7.78 7.77 7.77 7.76 7.45 7.45 7.44 7.44 7.41 7.41 7.40 7.40 7.40 7.39 7.39 7.32 7.32 7.31 7.31 7.30 7.28 7.28 7.27 7.27 7.26 7.25 7.25 7.25 7.25 7.24 7.23 7.23 7.22 7.22 7.22 7.21 7.21 5.69 3.72 3.34 2.50 2.50 2.50
CN
MeO$_2$C
NH
N
O
Bn
F6
^{1}H NMR (600 MHz, DMSO-*d6*)
dichloromethane
0.99
1.00
1.98
1.99
1.00
1.02
2.00
1.01
3.01
1.67
2.98
f1 (ppm)

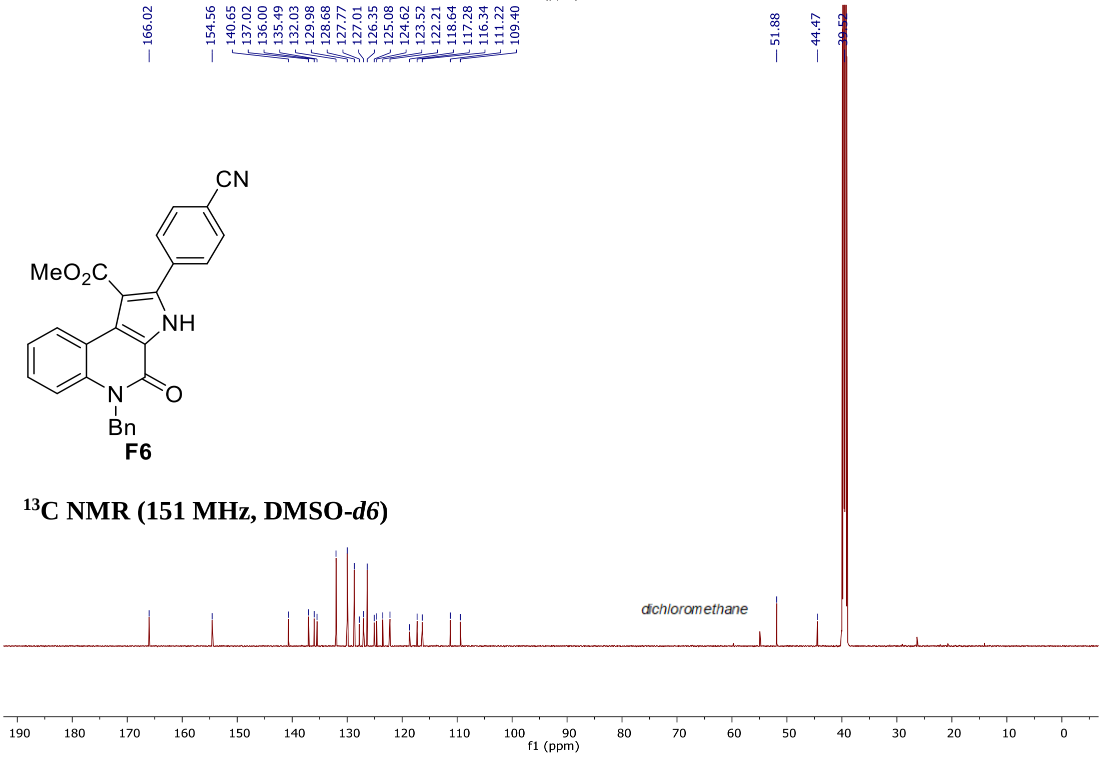
166.02
154.56 140.65 137.02 136.00 135.49 132.03 129.98 128.68 127.77 127.01 126.35 125.08 124.62 123.52 122.21 118.64 117.28 116.34 111.22 109.40
51.88
44.47
39.52
CN
MeO$_2$C
NH
N
O
Bn
F6
^{13}C NMR (151 MHz, DMSO-*d6*)
dichloromethane
f1 (ppm)

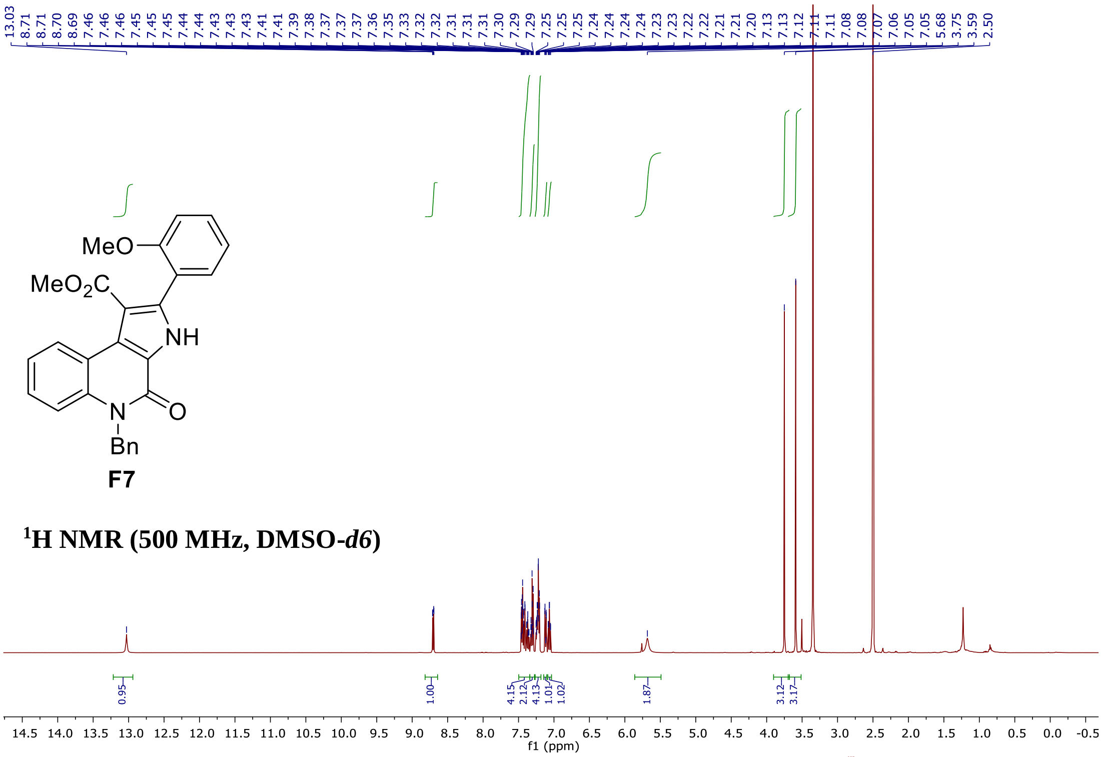
MeO
MeO2C
NH
N
O
Bn
F7
1H NMR (500 MHz, DMSO-d6)
f1 (ppm)

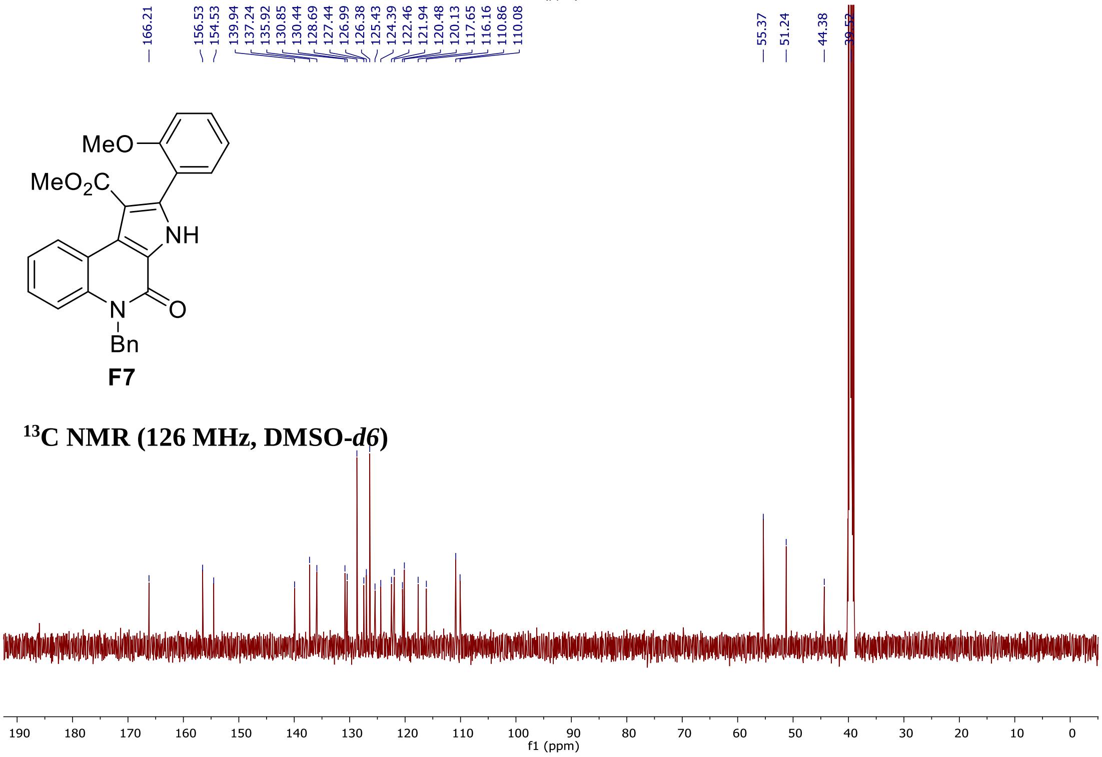
MeO
MeO2C
NH
N
O
Bn
F7
13C NMR (126 MHz, DMSO-d6)
f1 (ppm)

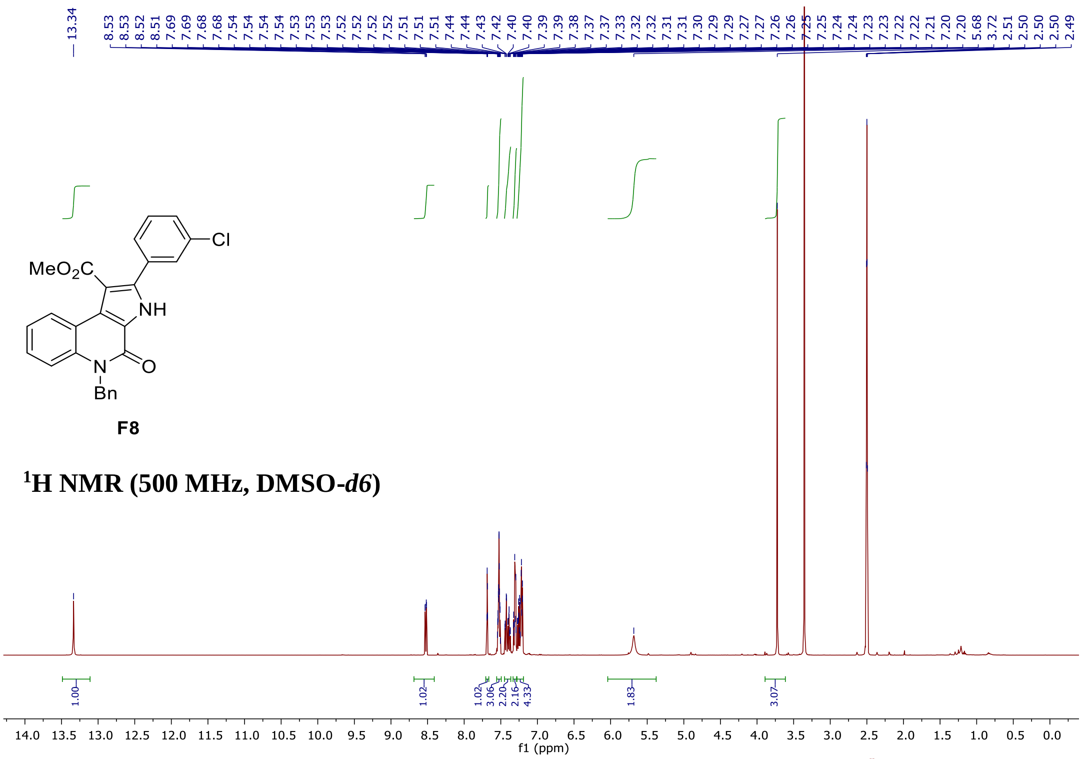
13.34
8.53 8.53 8.52 8.51 7.69 7.69 7.68 7.54 7.54 7.54 7.54 7.53 7.53 7.53 7.52 7.52 7.52 7.52 7.51 7.51 7.44 7.44 7.43 7.42 7.40 7.39 7.38 7.37 7.37 7.33 7.32 7.32 7.31 7.30 7.29 7.29 7.27 7.26 7.26 7.25 7.25 7.24 7.24 7.23 7.23 7.22 7.22 7.21 7.20 7.20 5.68 3.72 2.51 2.50 2.50 2.49
MeO2C
NH
N
O
Bn
Cl
F8
1H NMR (500 MHz, DMSO-d6)
1.00 1.02 1.02 3.06 2.20 2.16 4.33 1.83 3.07
14.0 13.5 13.0 12.5 12.0 11.5 11.0 10.5 10.0 9.5 9.0 8.5 8.0 7.5 7.0 6.5 6.0 5.5 5.0 4.5 4.0 3.5 3.0 2.5 2.0 1.5 1.0 0.5 0.0
f1 (ppm)

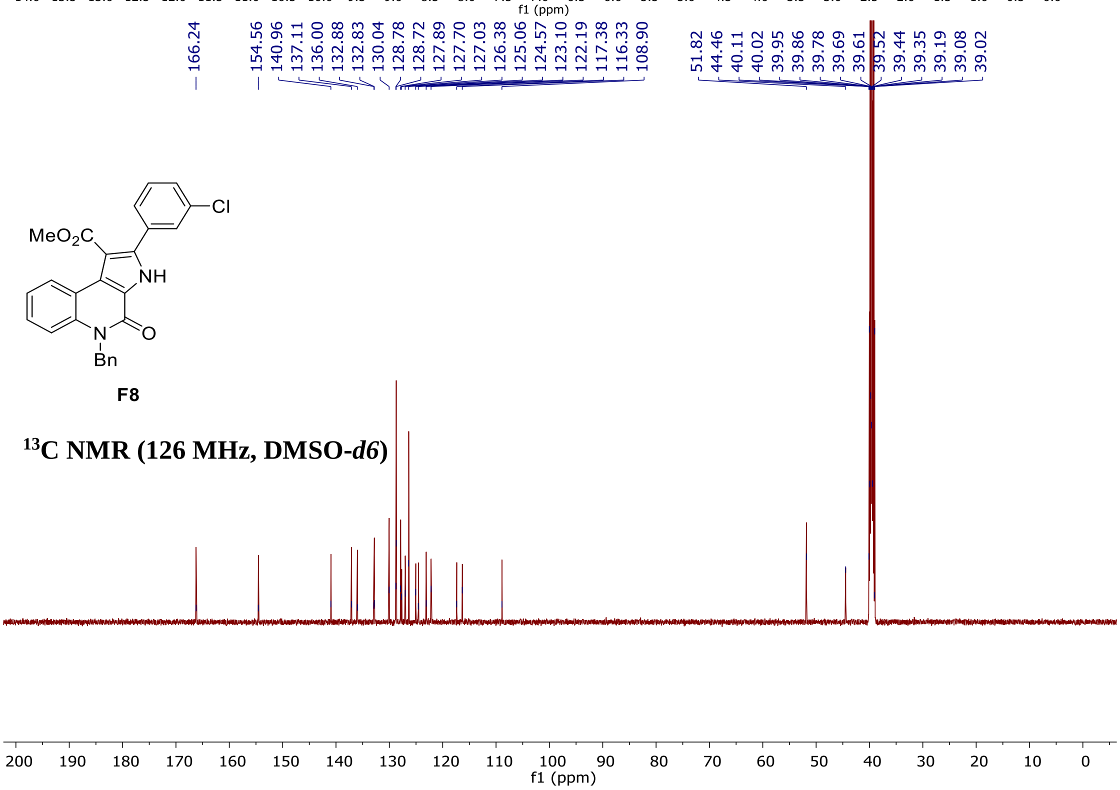
166.24
154.56 140.96 137.11 136.00 132.88 132.83 130.04 128.78 128.72 127.89 127.70 127.03 126.38 125.06 124.57 123.10 122.19 117.38 116.33 108.90
51.82 44.46 40.11 40.02 39.95 39.86 39.78 39.69 39.61 39.52 39.44 39.35 39.19 39.08 39.02
MeO2C
NH
N
O
Bn
Cl
F8
13C NMR (126 MHz, DMSO-d6)
200 190 180 170 160 150 140 130 120 110 100 90 80 70 60 50 40 30 20 10 0
f1 (ppm)

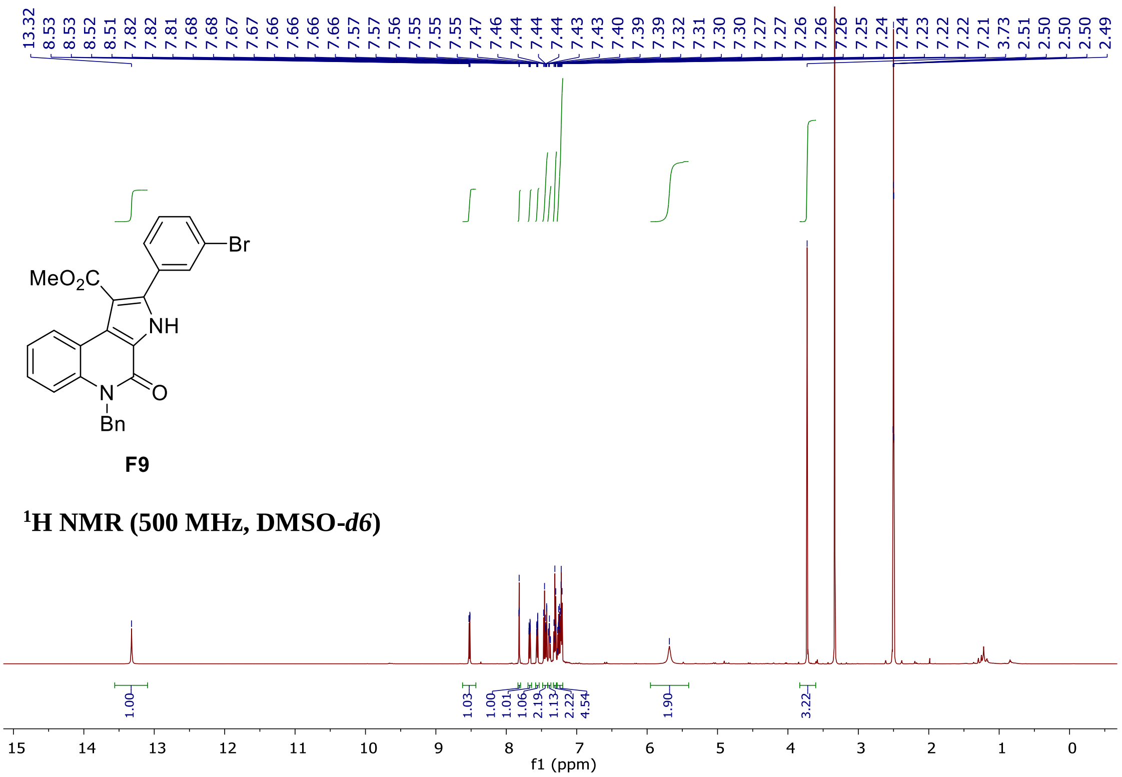
13.32 8.53 8.53 8.52 8.51 7.82 7.82 7.81 7.68 7.67 7.67 7.66 7.66 7.66 7.66 7.57 7.57 7.56 7.55 7.55 7.55 7.47 7.46 7.44 7.44 7.44 7.43 7.43 7.40 7.39 7.39 7.32 7.31 7.30 7.30 7.27 7.27 7.26 7.26 7.26 7.25 7.24 7.24 7.23 7.22 7.22 7.21 3.73 2.51 2.50 2.50 2.50 2.49
Br
MeO2C
NH
N
O
Bn
F9
1H NMR (500 MHz, DMSO-d6)
1.00 1.03 1.00 1.01 1.06 2.19 1.13 2.22 4.54 1.90 3.22
15 14 13 12 11 10 9 8 7 6 5 4 3 2 1 0
f1 (ppm)

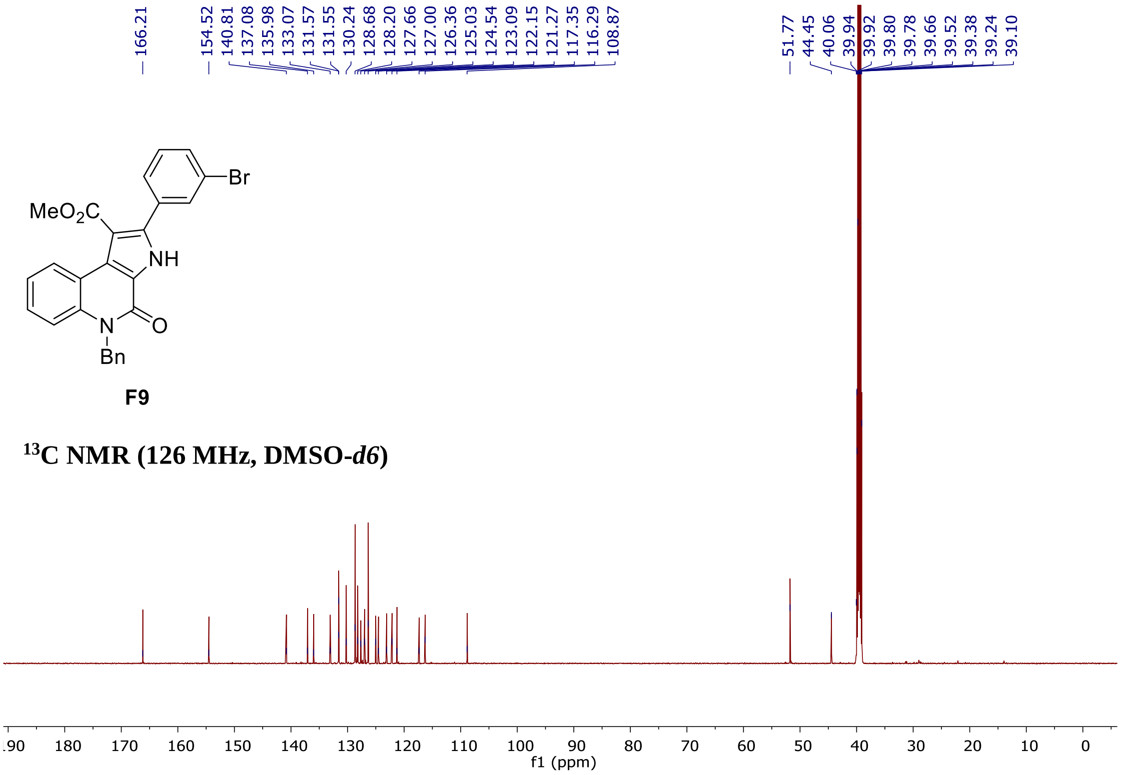
166.21
154.52 140.81 137.08 135.98 133.07 131.57 131.55 130.24 128.68 128.20 127.66 127.00 126.36 125.03 124.54 123.09 122.15 121.27 117.35 116.29 108.87
51.77 44.45 40.06 39.94 39.92 39.80 39.78 39.66 39.52 39.38 39.24 39.10
Br
MeO2C
NH
N
O
Bn
F9
13C NMR (126 MHz, DMSO-d6)
190 180 170 160 150 140 130 120 110 100 90 80 70 60 50 40 30 20 10 0
f1 (ppm)

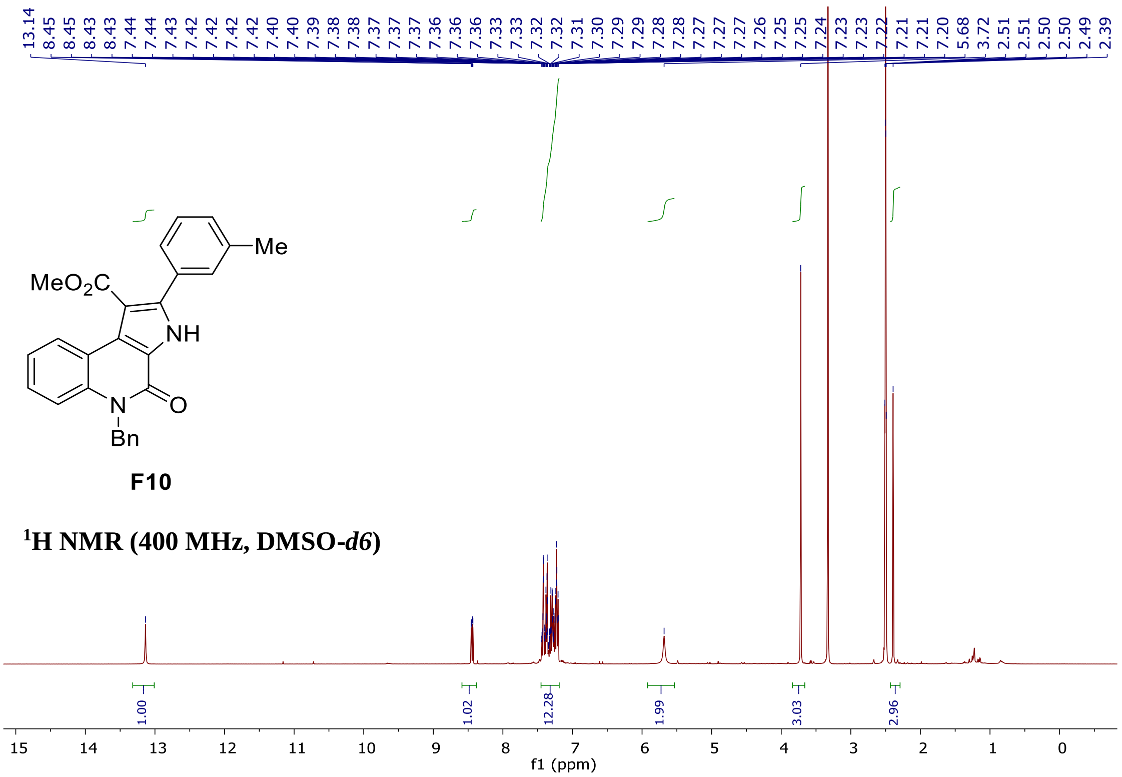

F10

^{1}H NMR (400 MHz, DMSO-*d6*)

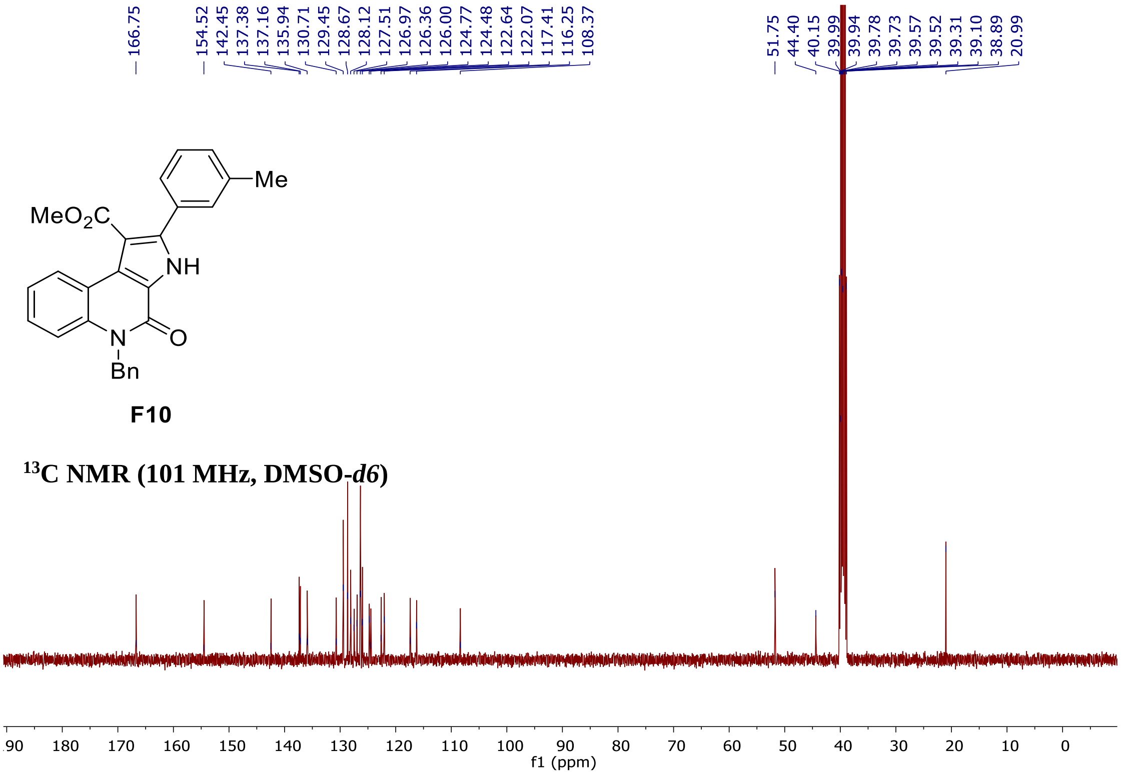

F10

^{13}C NMR (101 MHz, DMSO-*d6*)

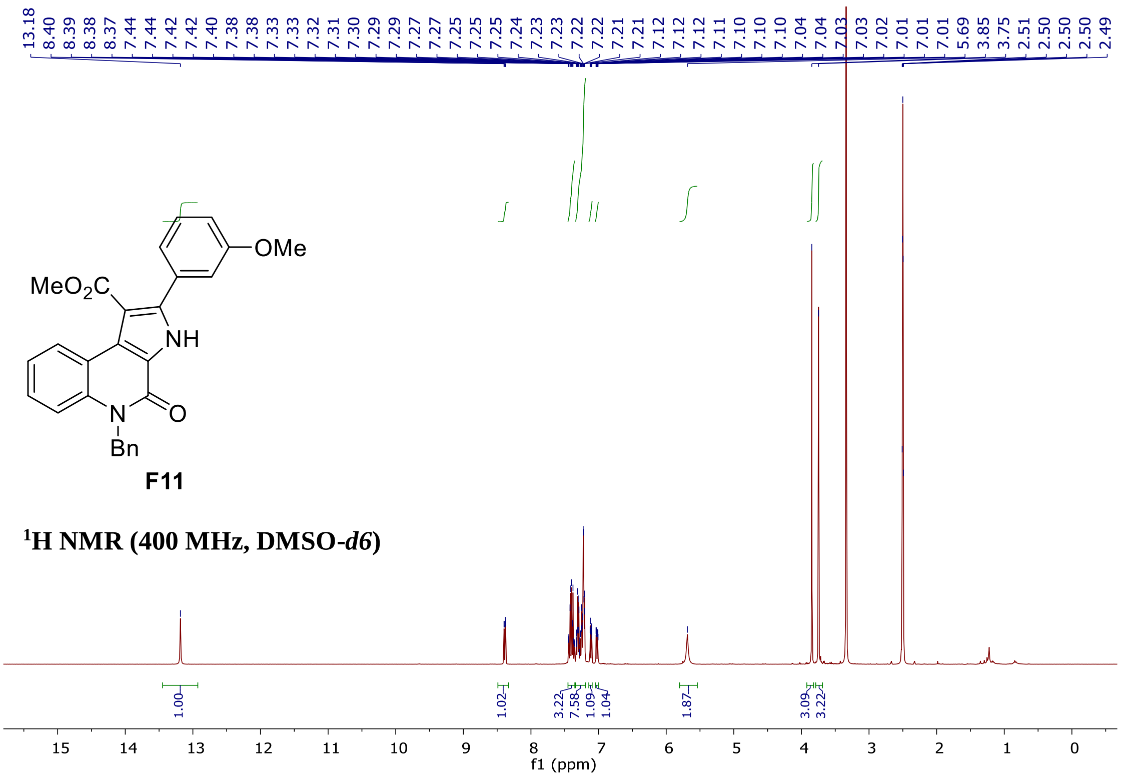
13.18
8.40
8.39
8.38
8.37
7.44
7.44
7.42
7.42
7.40
7.38
7.38
7.33
7.33
7.32
7.31
7.30
7.29
7.29
7.27
7.27
7.25
7.25
7.25
7.24
7.23
7.23
7.22
7.22
7.22
7.21
7.21
7.12
7.12
7.12
7.11
7.10
7.10
7.10
7.04
7.04
7.03
7.03
7.02
7.01
7.01
7.01
5.69
3.85
3.75
2.51
2.50
2.50
2.50
2.49
OMe
MeO2C
NH
N
O
Bn
F11
1H NMR (400 MHz, DMSO-d6)
1.00
1.02
3.22
7.58
1.09
1.04
1.87
3.09
3.22
15 14 13 12 11 10 9 8 7 6 5 4 3 2 1 0
f1 (ppm)

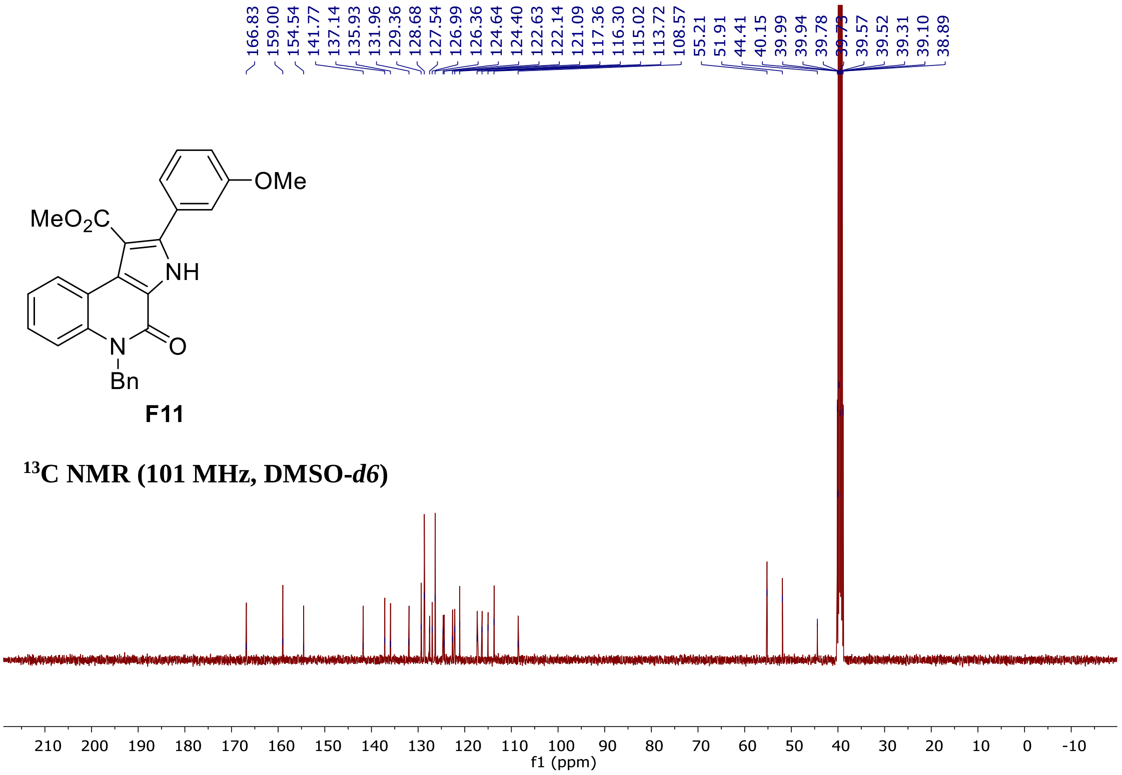
166.83
159.00
154.54
141.77
137.14
135.93
131.96
129.36
128.68
127.54
126.99
126.36
124.64
124.40
122.63
122.14
121.09
117.36
116.30
115.02
113.72
108.57
55.21
51.91
44.41
40.15
39.99
39.94
39.78
39.73
39.57
39.52
39.31
39.10
38.89
OMe
MeO2C
NH
N
O
Bn
F11
13C NMR (101 MHz, DMSO-d6)
210 200 190 180 170 160 150 140 130 120 110 100 90 80 70 60 50 40 30 20 10 0 -10
f1 (ppm)

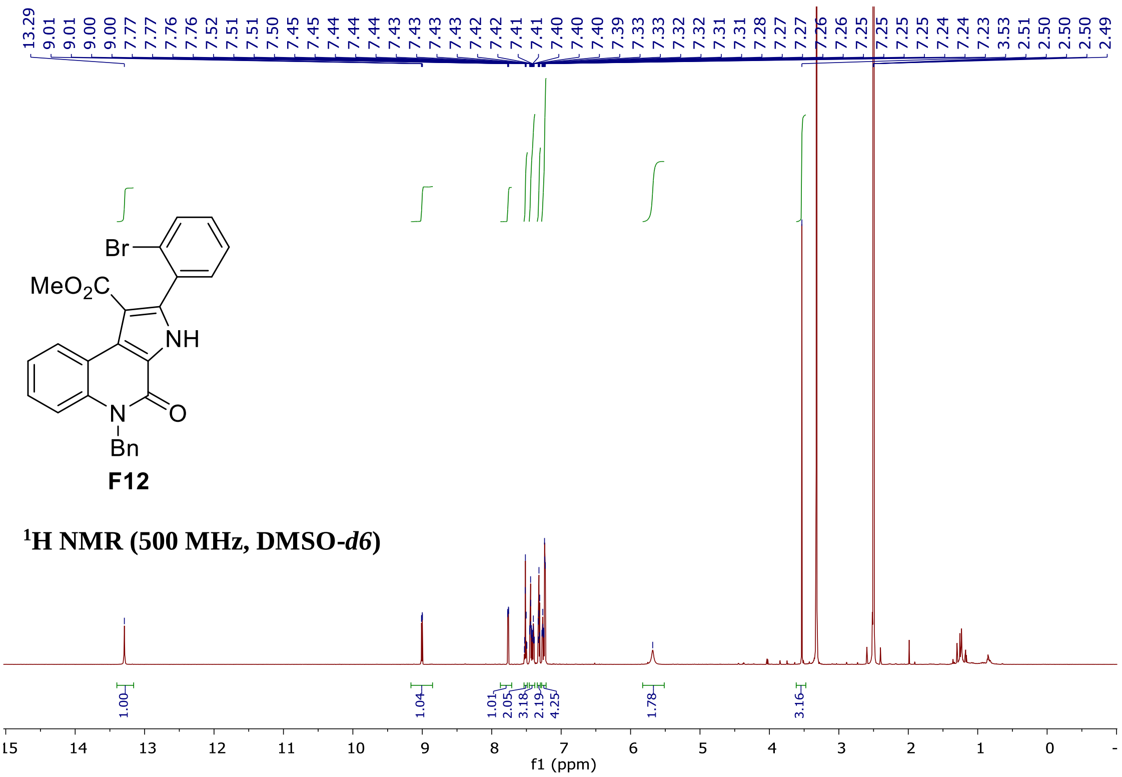
13.29 9.01 9.01 9.00 9.00 7.77 7.77 7.76 7.76 7.52 7.51 7.51 7.50 7.45 7.45 7.44 7.44 7.44 7.43 7.43 7.43 7.43 7.42 7.42 7.41 7.41 7.40 7.40 7.40 7.39 7.33 7.33 7.32 7.32 7.31 7.31 7.28 7.27 7.27 7.26 7.26 7.25 7.25 7.25 7.25 7.24 7.24 7.23 3.53 2.51 2.50 2.50 2.50 2.49
Br
MeO2C
NH
N
O
Bn
F12
1H NMR (500 MHz, DMSO-d6)
1.00
1.04
1.01
2.05
3.18
2.19
4.25
1.78
3.16
f1 (ppm)

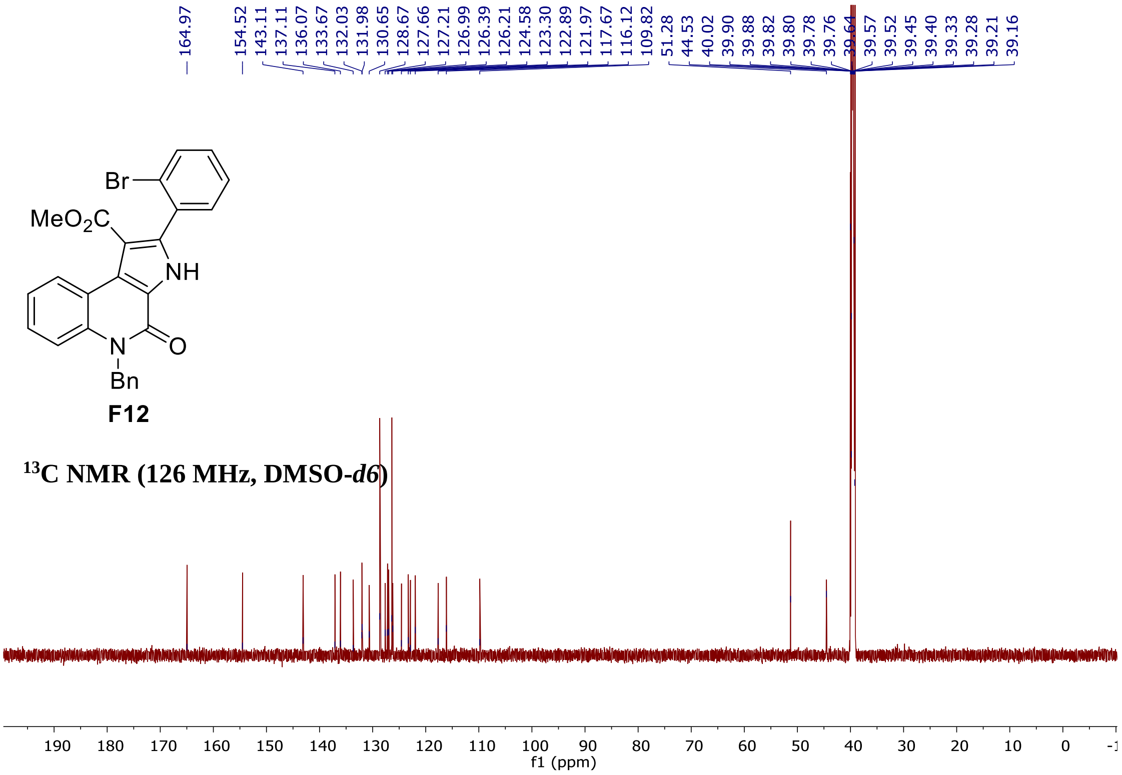
164.97
154.52 143.11 137.11 136.07 133.67 132.03 131.98 130.65 128.67 127.66 127.21 126.99 126.39 126.21 124.58 123.30 122.89 121.97 117.67 116.12 109.82
51.28 44.53 40.02 39.90 39.88 39.82 39.80 39.78 39.76 39.64 39.57 39.52 39.45 39.40 39.33 39.28 39.21 39.16
Br
MeO2C
NH
N
O
Bn
F12
13C NMR (126 MHz, DMSO-d6)
f1 (ppm)

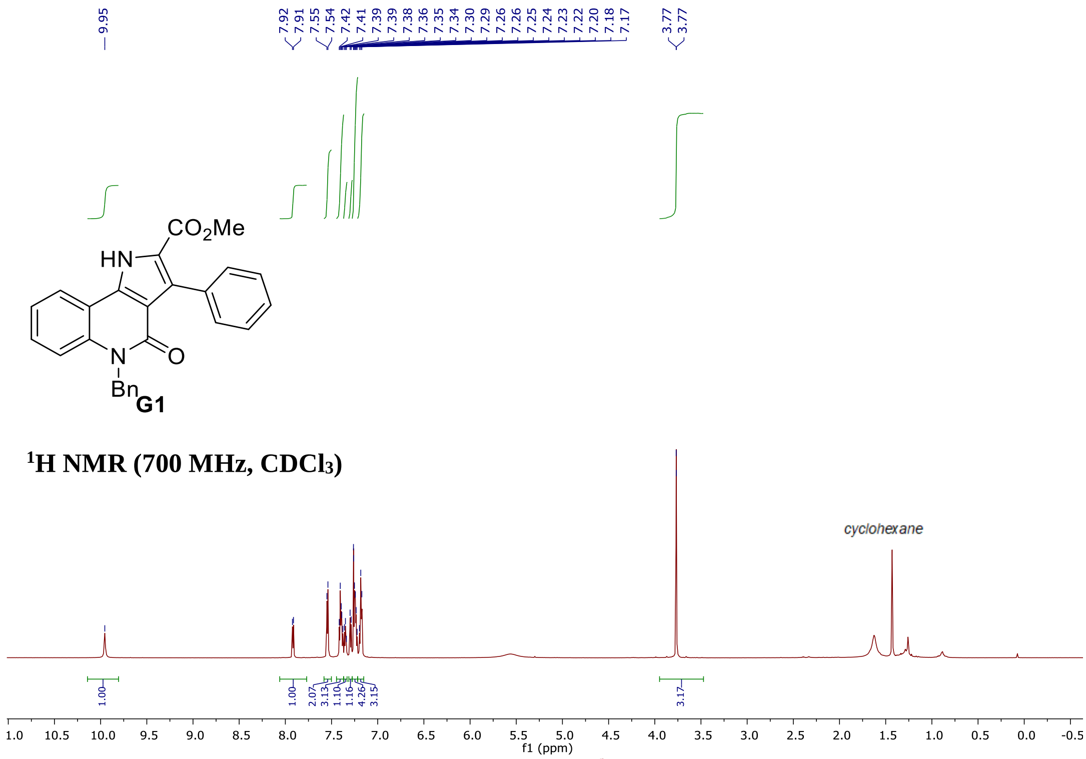

^{1}H NMR (700 MHz, $CDCl_3$)

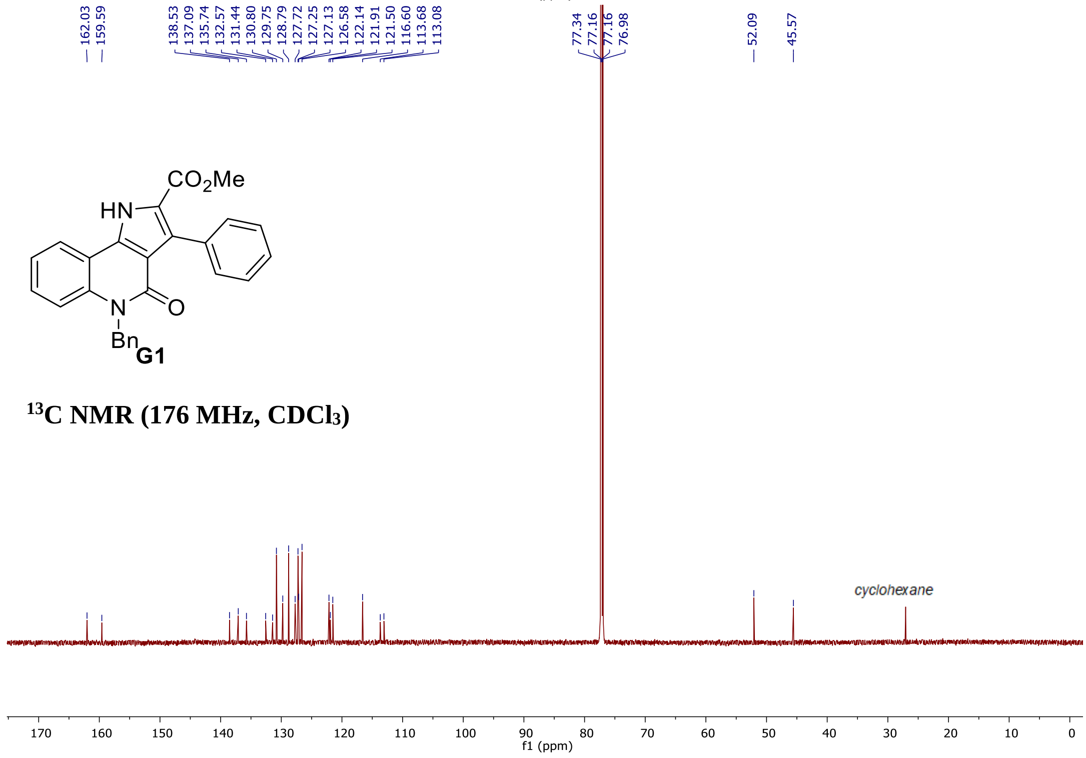

^{13}C NMR (176 MHz, $CDCl_3$)

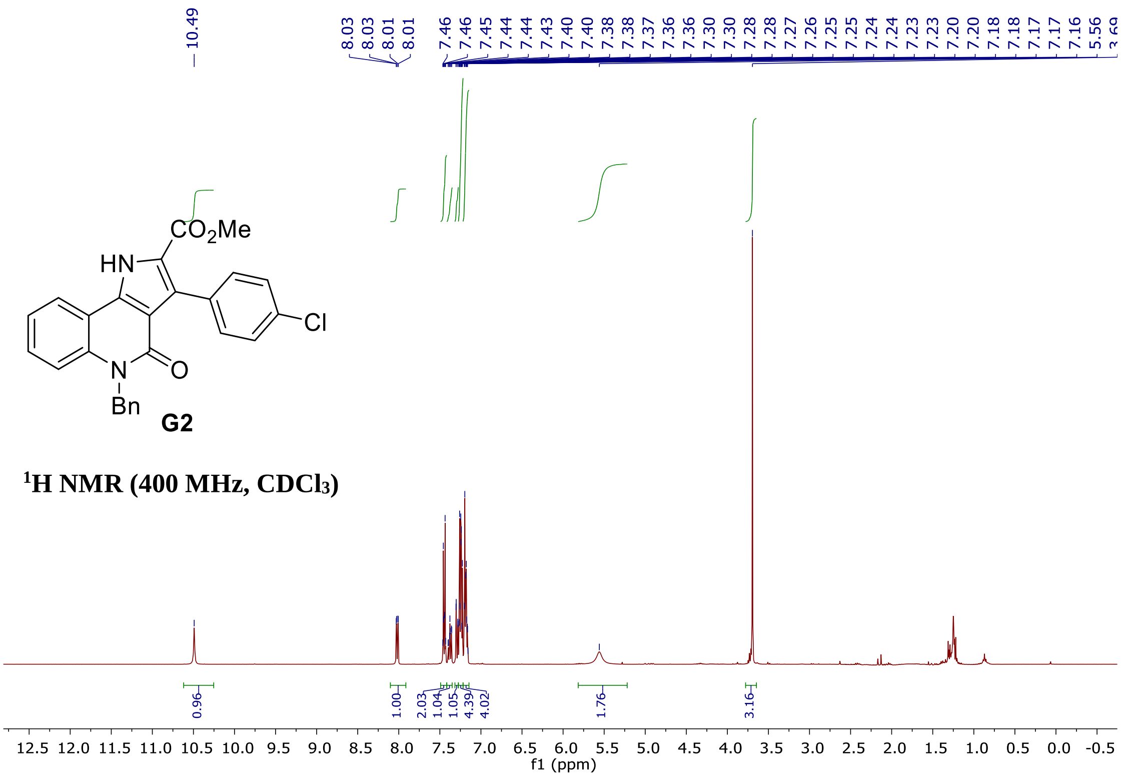

G2

^{1}H NMR (400 MHz, CDCl3)

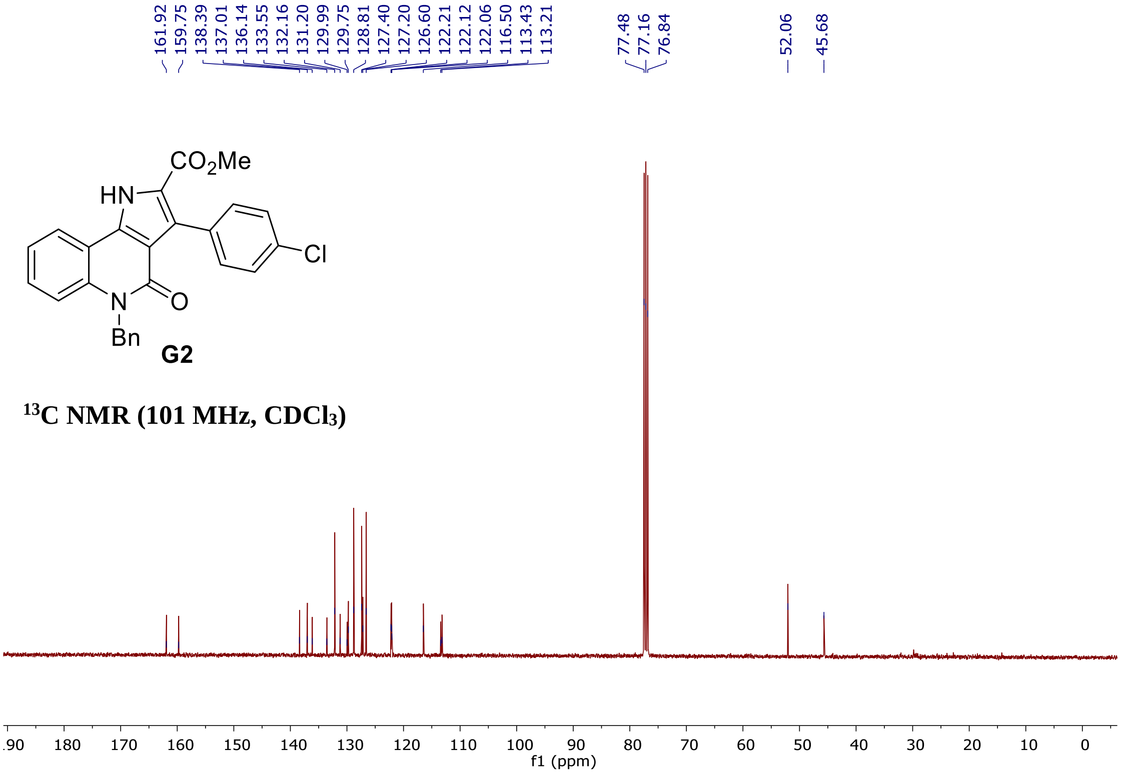

G2

^{13}C NMR (101 MHz, CDCl3)

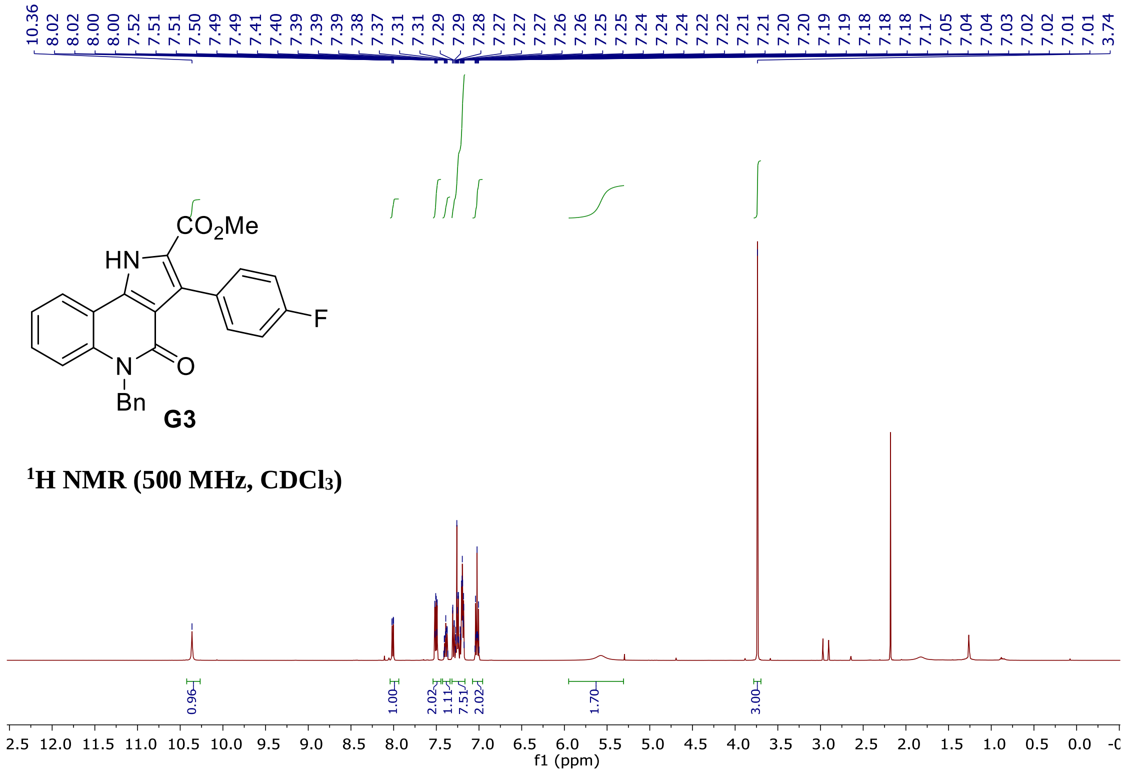

G3

^{1}H NMR (500 MHz, CDCl3)

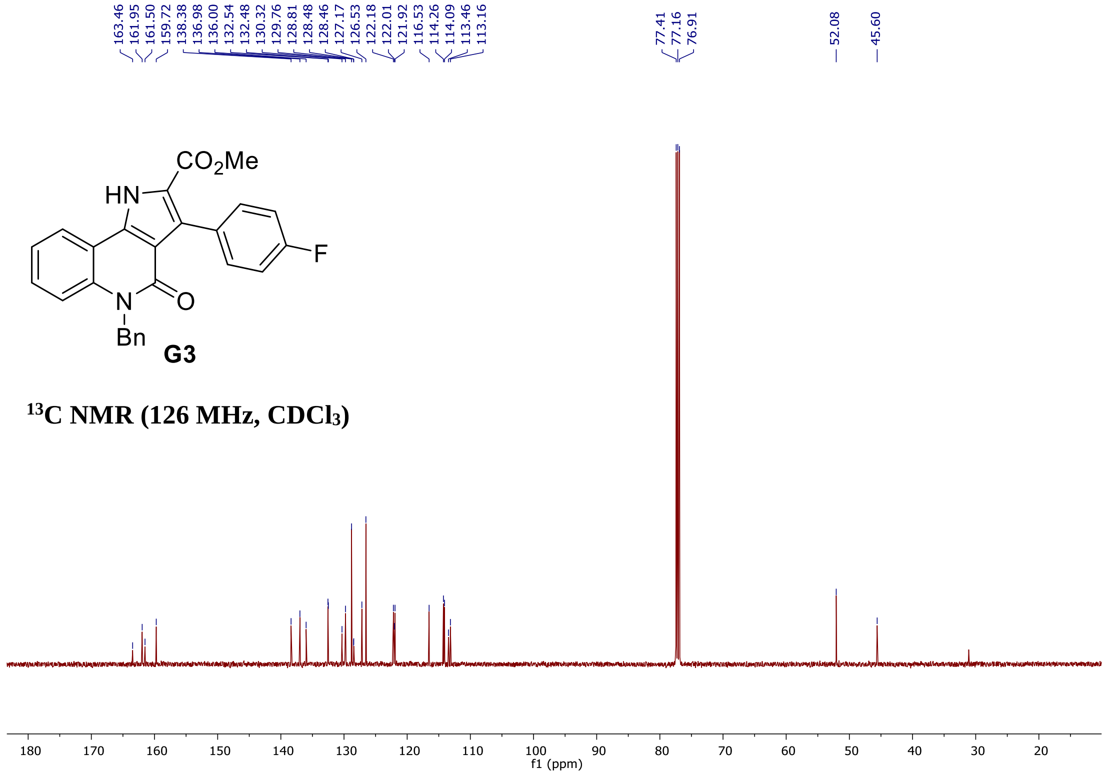

G3

^{13}C NMR (126 MHz, CDCl3)

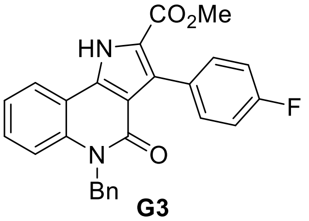

G3

^{19}F NMR (470 MHz, CDCl$_3$)

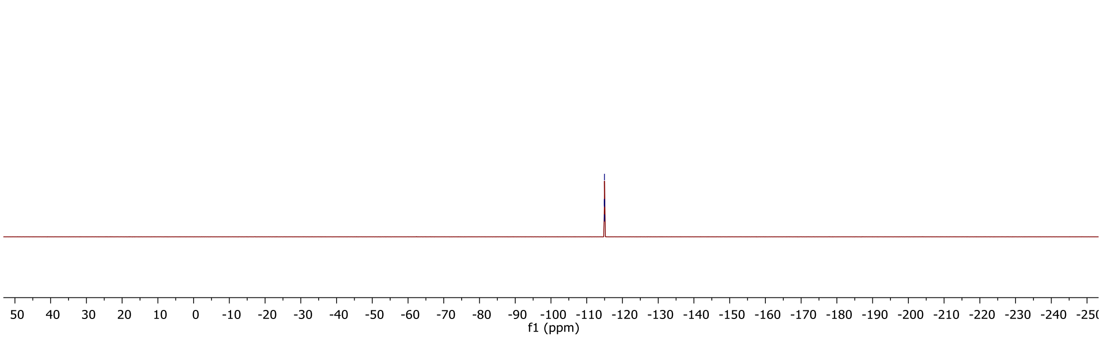

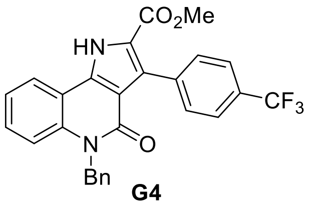

G4

^{19}F NMR (470 MHz, CDCl$_3$)

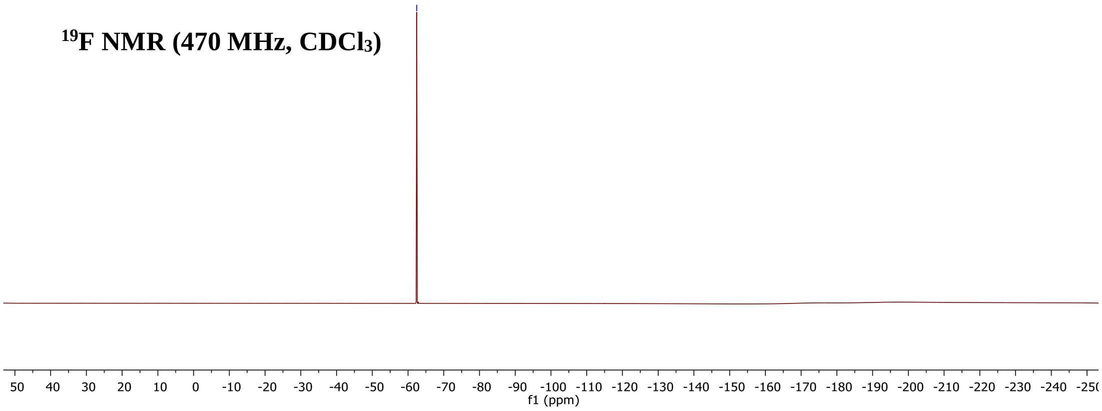

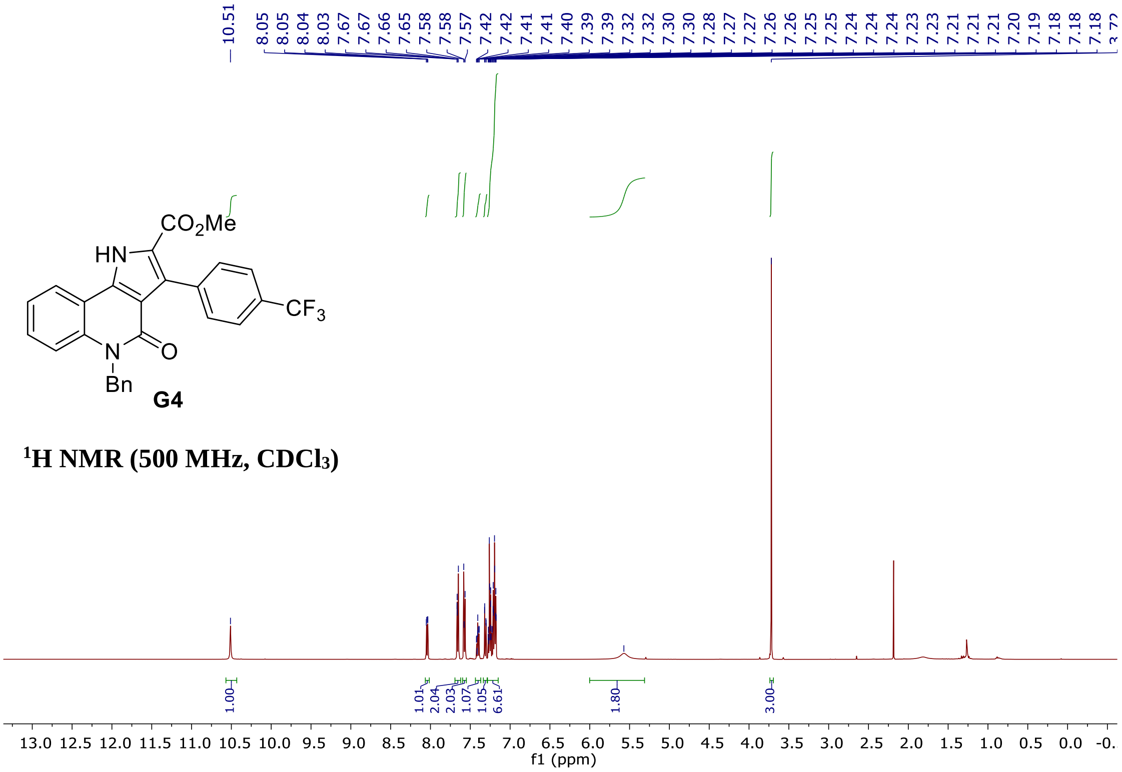

^{1}H NMR (500 MHz, $CDCl_3$)

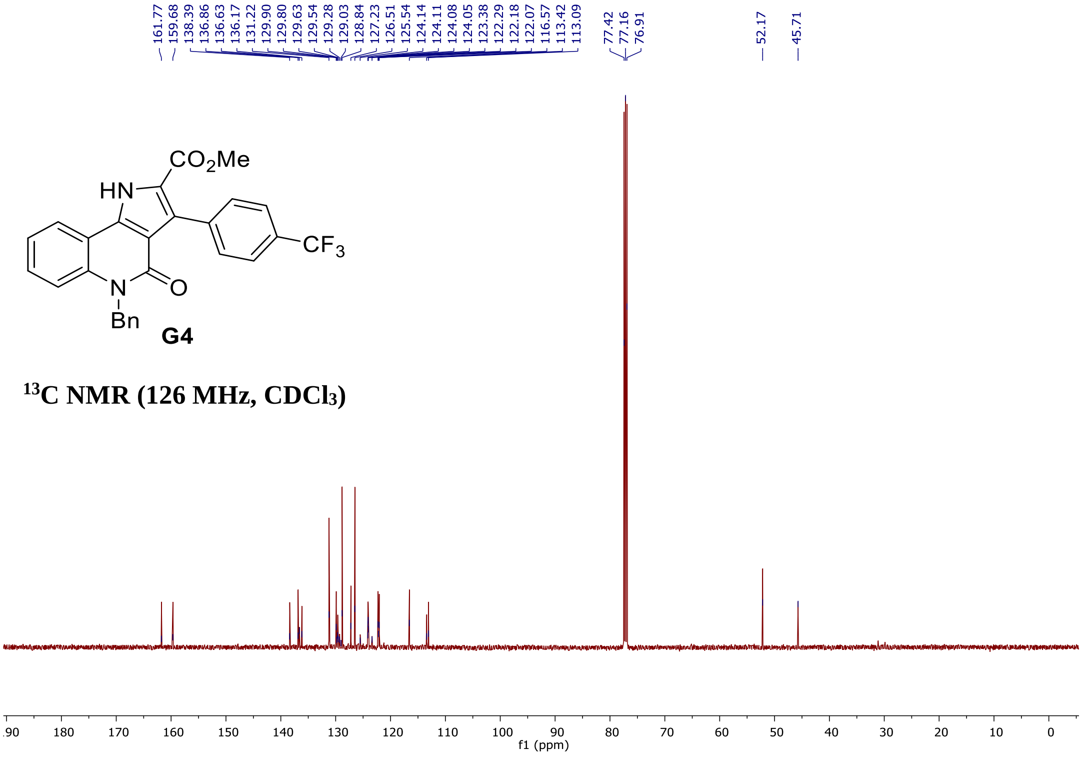

^{13}C NMR (126 MHz, $CDCl_3$)

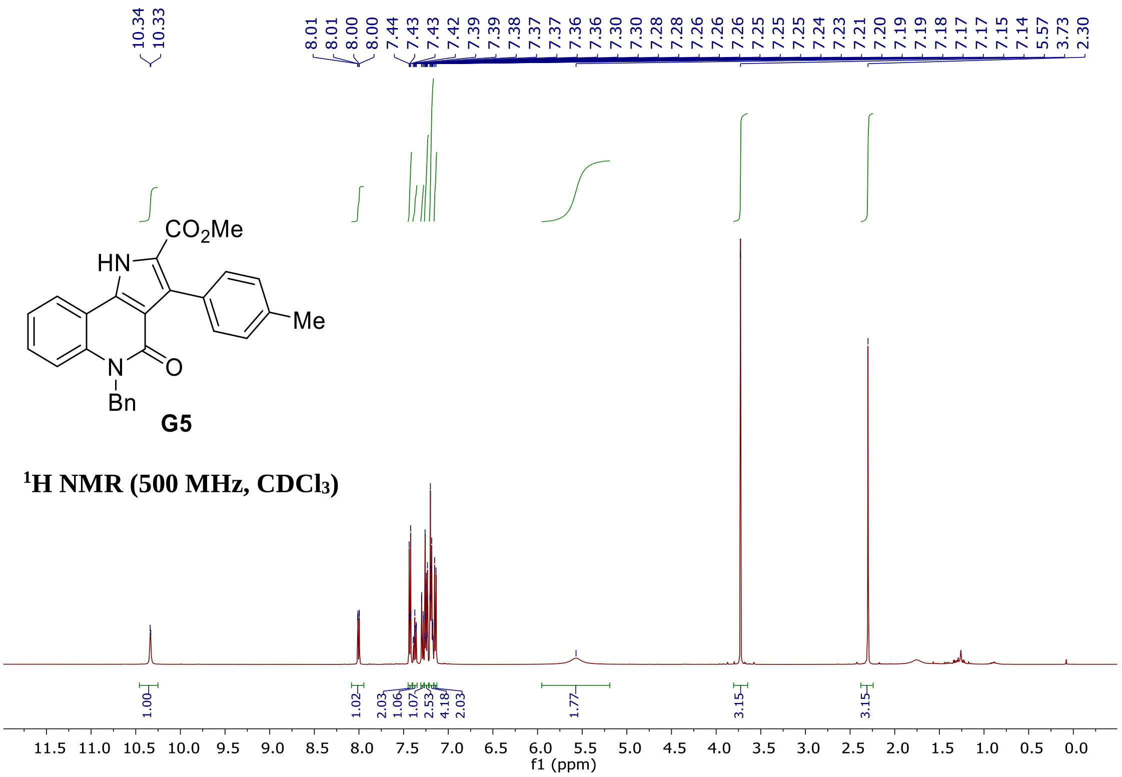

^{1}H NMR (500 MHz, CDCl₃)

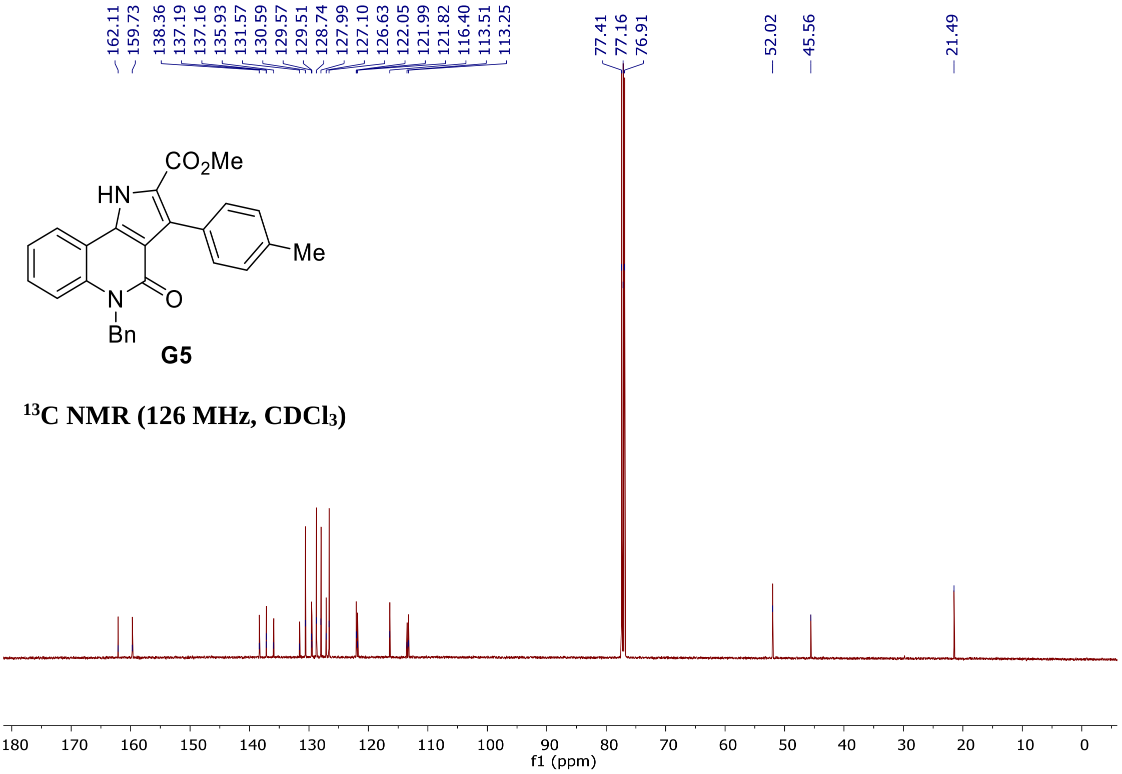

^{13}C NMR (126 MHz, CDCl₃)

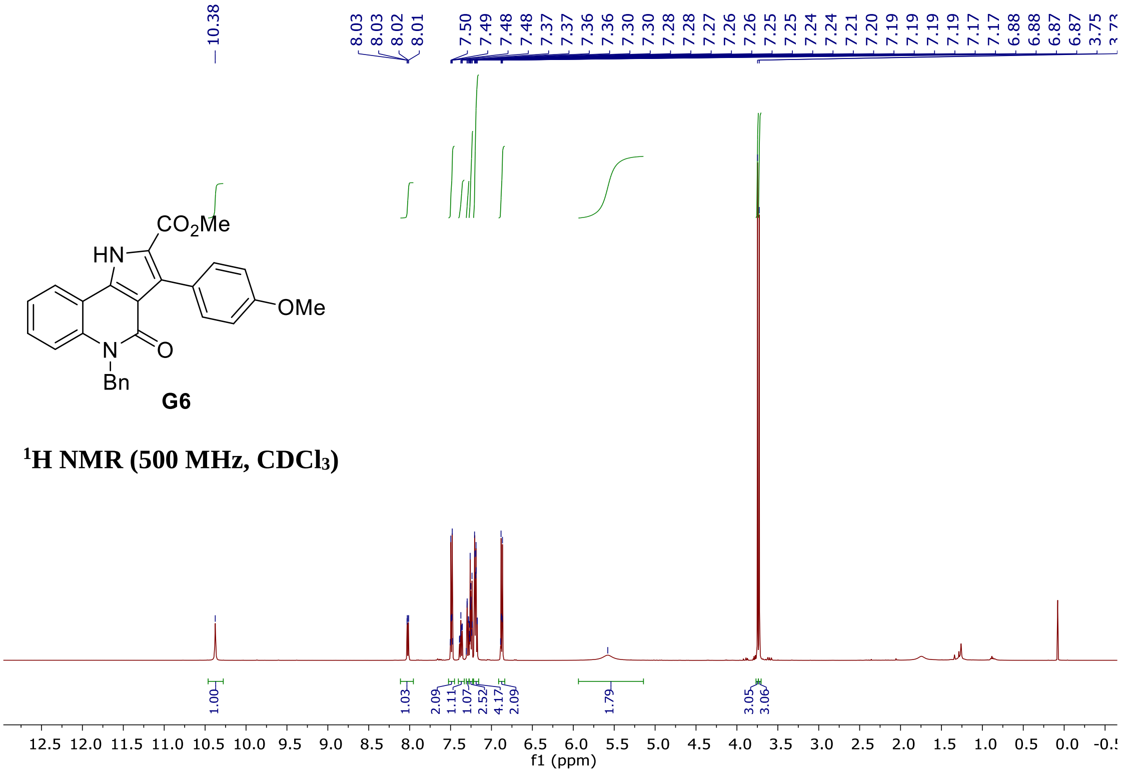
CO2Me
HN
OMe
N
O
Bn
G6
1H NMR (500 MHz, CDCl3)
f1 (ppm)

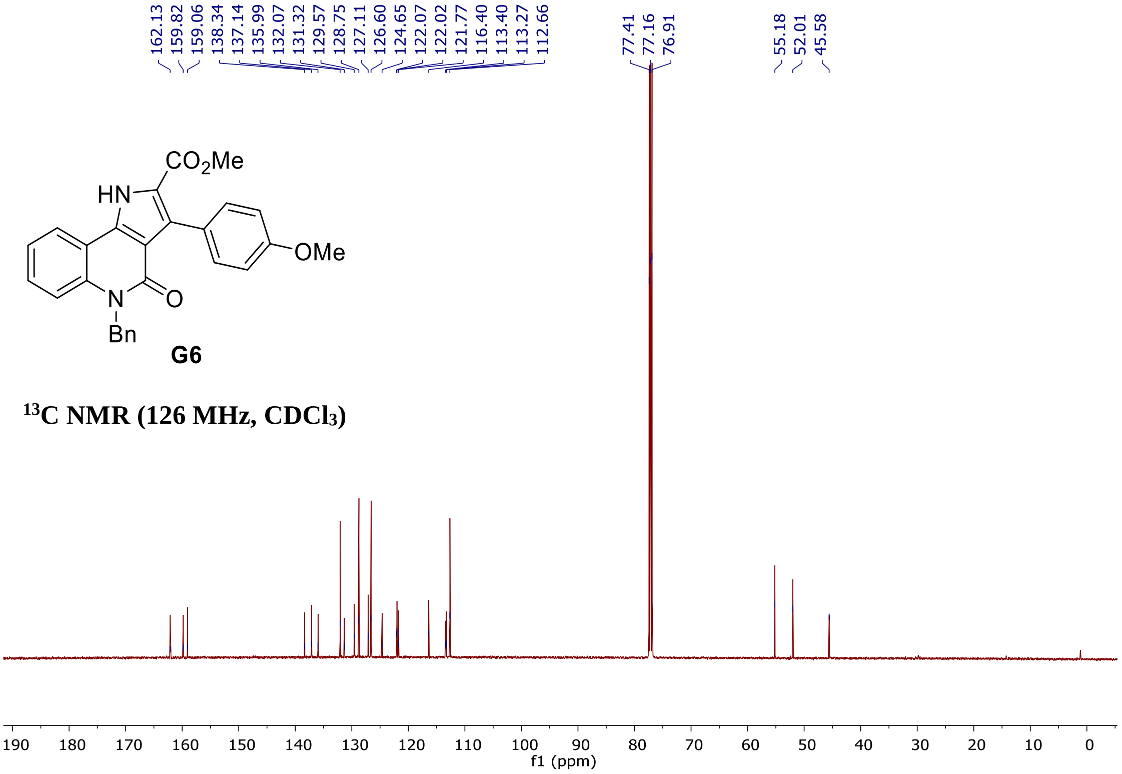
CO2Me
HN
OMe
N
O
Bn
G6
13C NMR (126 MHz, CDCl3)
f1 (ppm)

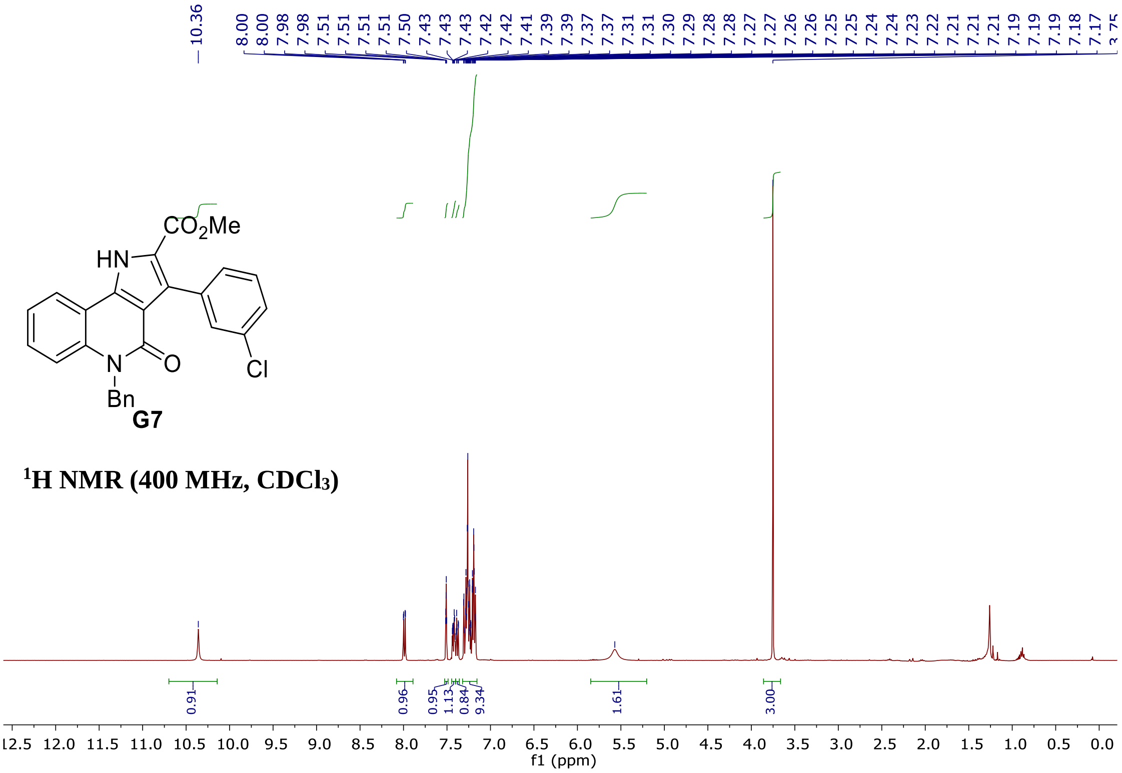

^{1}H NMR (400 MHz, $CDCl_3$)

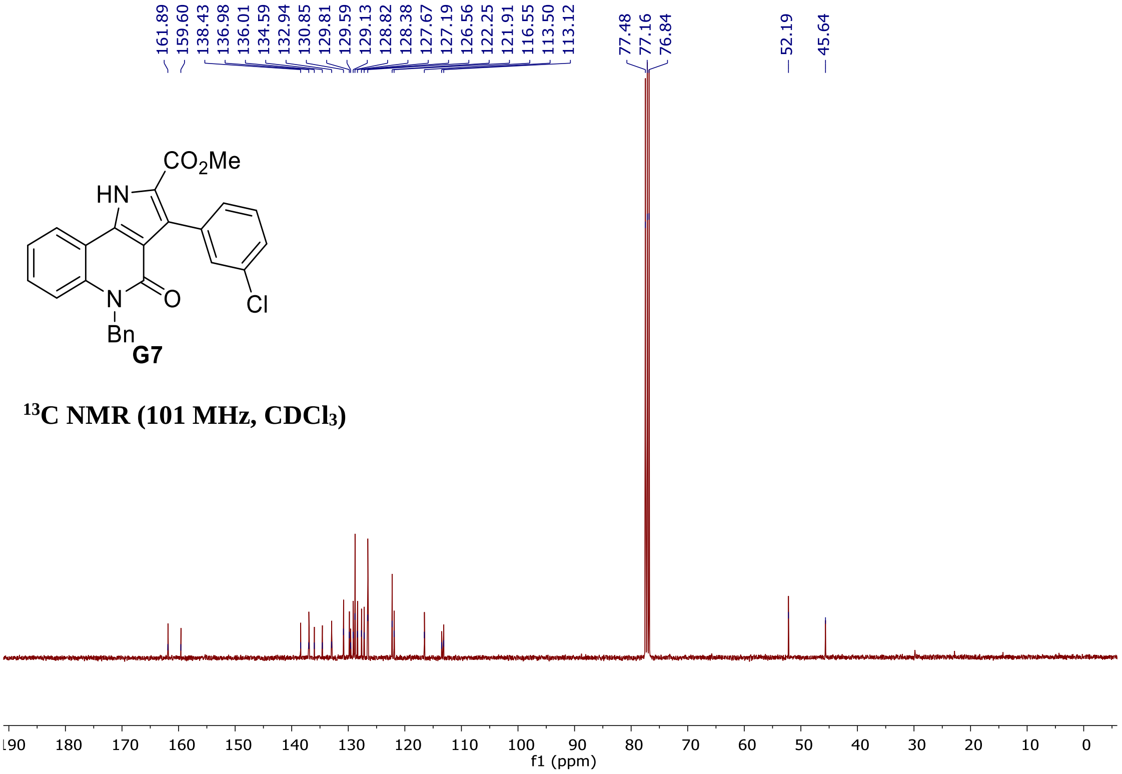

^{13}C NMR (101 MHz, $CDCl_3$)

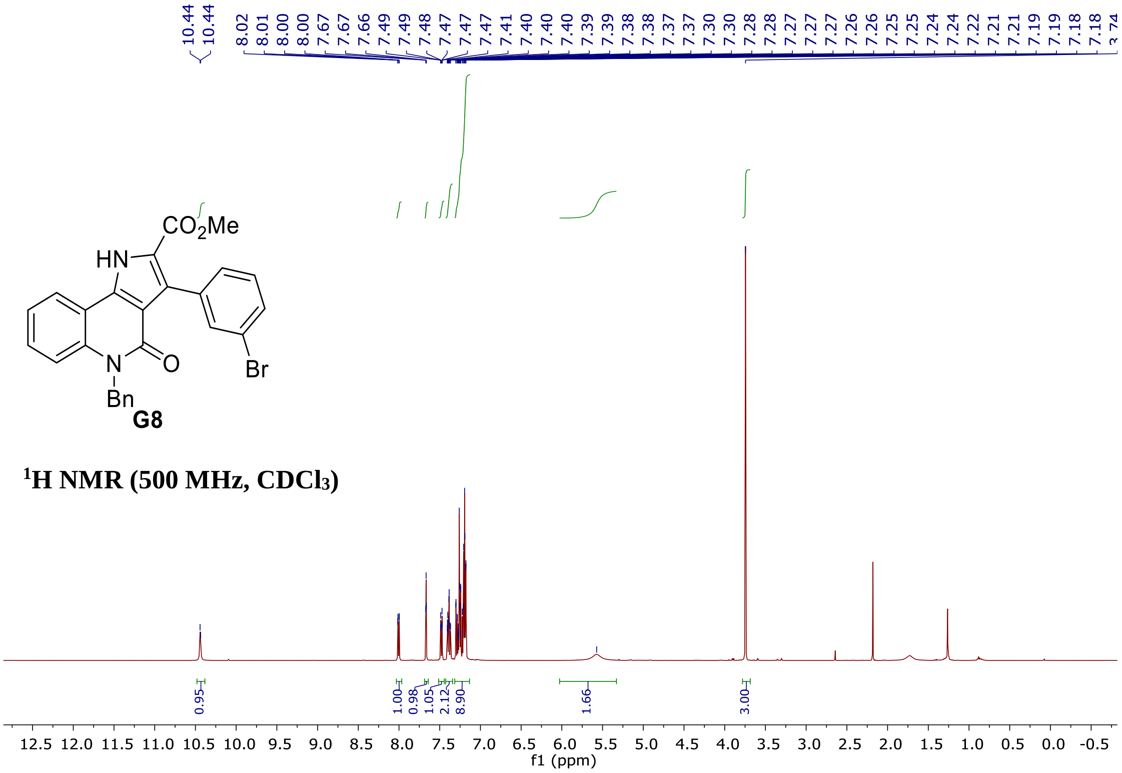

G8

^{1}H NMR (500 MHz, CDCl3)

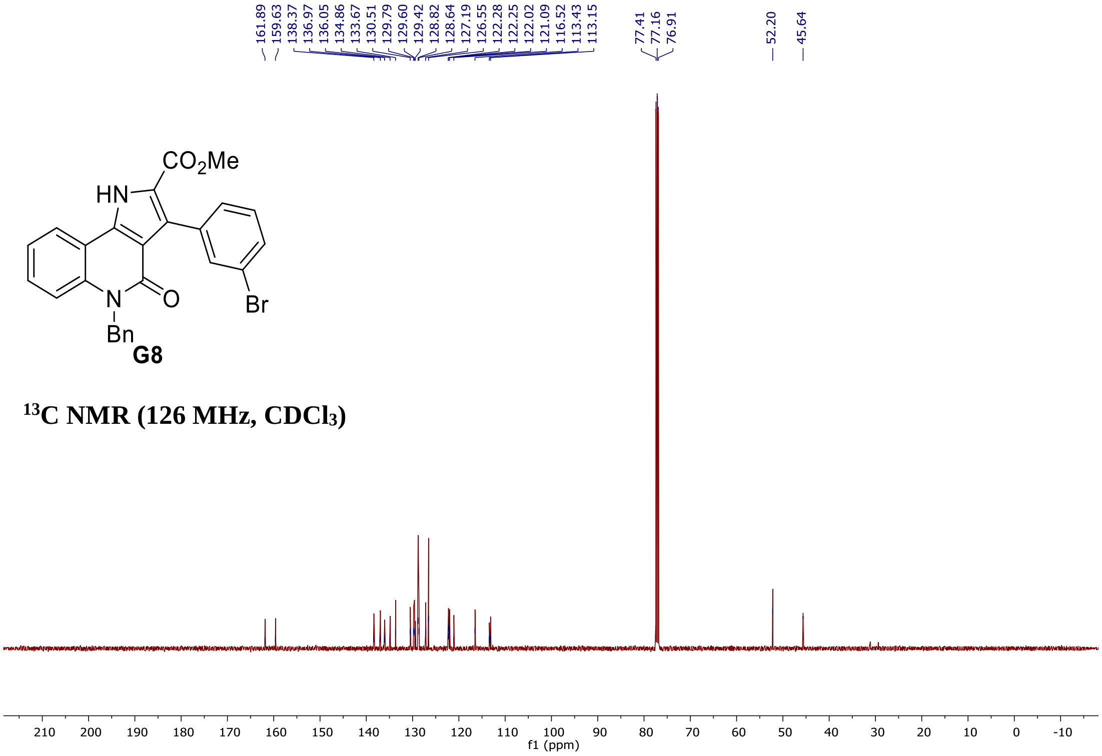

G8

^{13}C NMR (126 MHz, CDCl3)

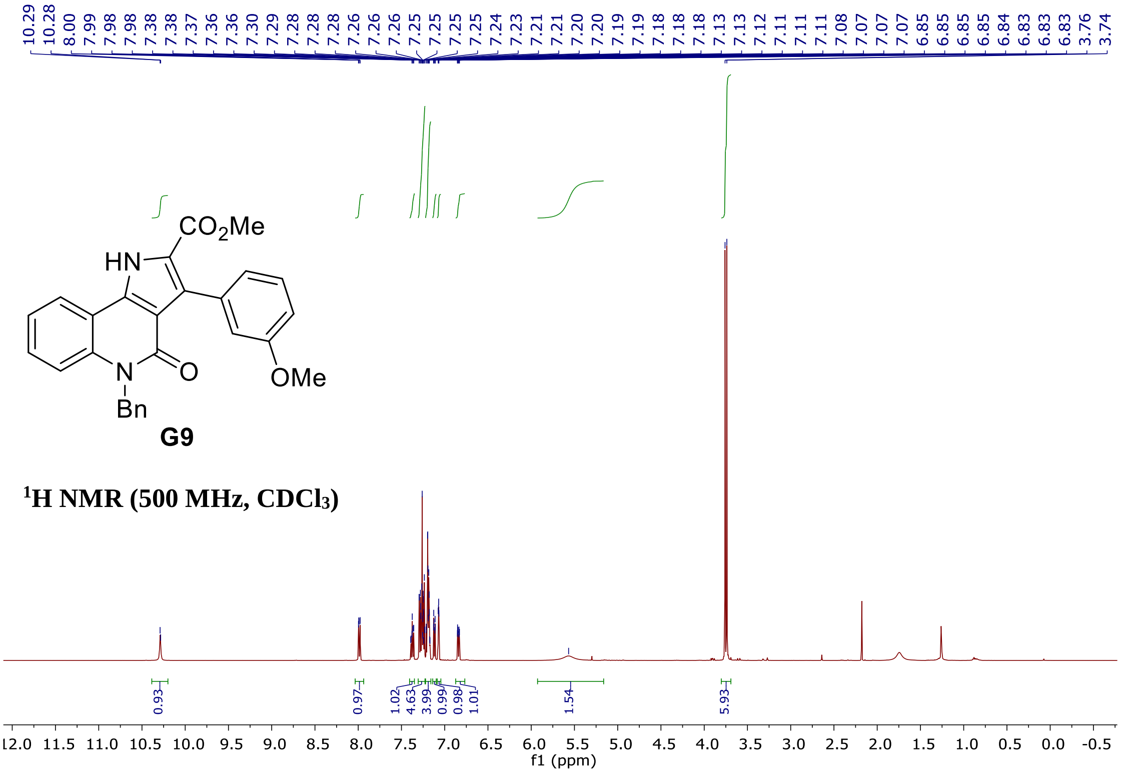
10.29 10.28 8.00 7.99 7.98 7.98 7.38 7.38 7.37 7.36 7.36 7.30 7.29 7.28 7.28 7.28 7.26 7.26 7.25 7.25 7.25 7.25 7.24 7.23 7.21 7.21 7.20 7.19 7.19 7.18 7.18 7.18 7.13 7.13 7.12 7.11 7.11 7.11 7.08 7.07 7.07 7.07 6.85 6.85 6.85 6.84 6.83 6.83 6.83 3.76 3.74
CO2Me
HN
N
O
OMe
Bn
G9
1H NMR (500 MHz, CDCl3)
0.93
0.97
1.02
4.63
3.99
0.99
0.98
1.01
1.54
5.93
12.0 11.5 11.0 10.5 10.0 9.5 9.0 8.5 8.0 7.5 7.0 6.5 6.0 5.5 5.0 4.5 4.0 3.5 3.0 2.5 2.0 1.5 1.0 0.5 0.0 -0.5
f1 (ppm)

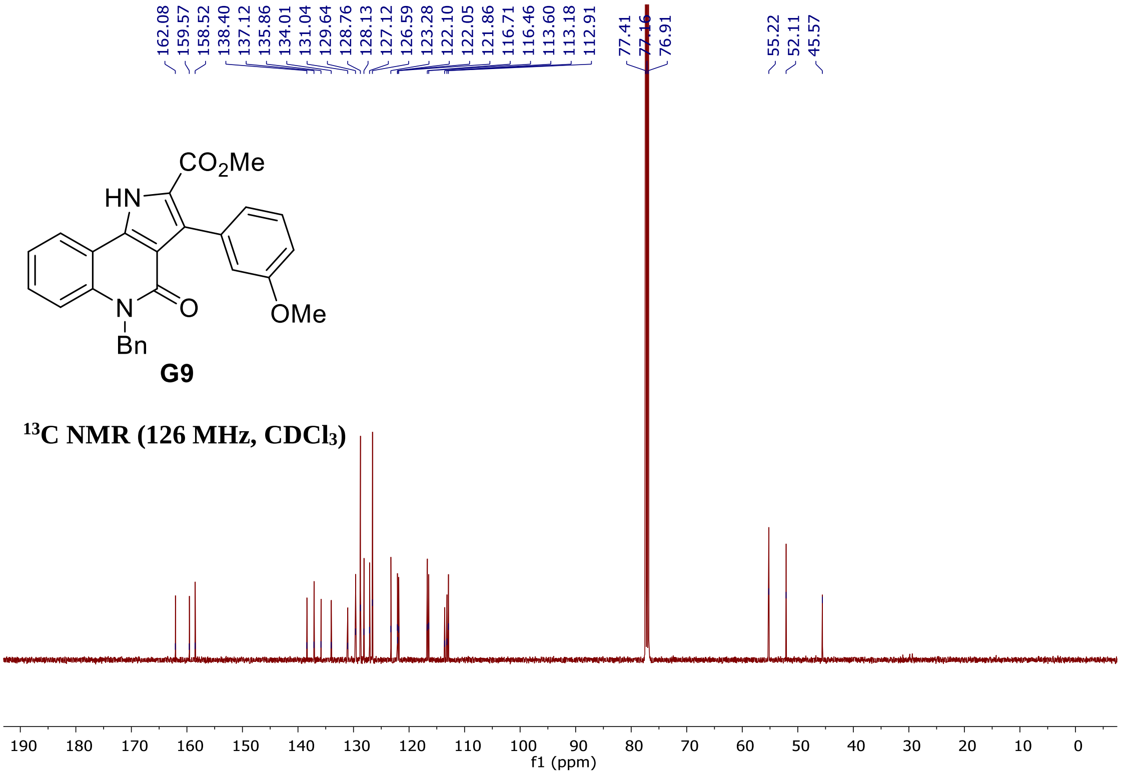
162.08 159.57 158.52 138.40 137.12 135.86 134.01 131.04 129.64 128.76 128.13 127.12 126.59 123.28 122.10 122.05 121.86 116.71 116.46 113.60 113.18 112.91
77.41 77.16 76.91
55.22 52.11 45.57
CO2Me
HN
N
O
OMe
Bn
G9
13C NMR (126 MHz, CDCl3)
190 180 170 160 150 140 130 120 110 100 90 80 70 60 50 40 30 20 10 0
f1 (ppm)

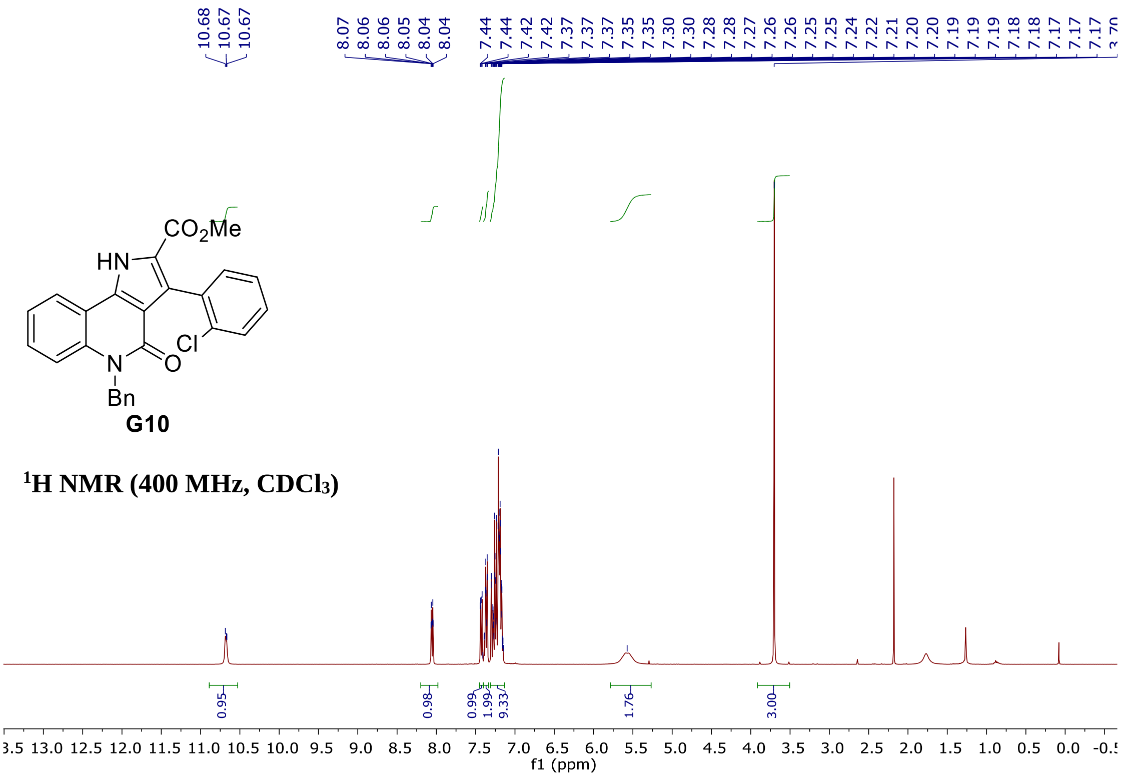
10.68
10.67
10.67
8.07
8.06
8.06
8.05
8.04
8.04
7.44
7.44
7.42
7.42
7.37
7.37
7.37
7.35
7.35
7.30
7.30
7.28
7.28
7.27
7.26
7.26
7.25
7.25
7.24
7.22
7.21
7.20
7.20
7.19
7.19
7.19
7.18
7.18
7.17
7.17
7.17
CO2Me
HN
Cl
N
O
Bn
G10
1H NMR (400 MHz, CDCl3)
0.95
0.98
0.99
1.99
9.33
1.76
3.00
f1 (ppm)

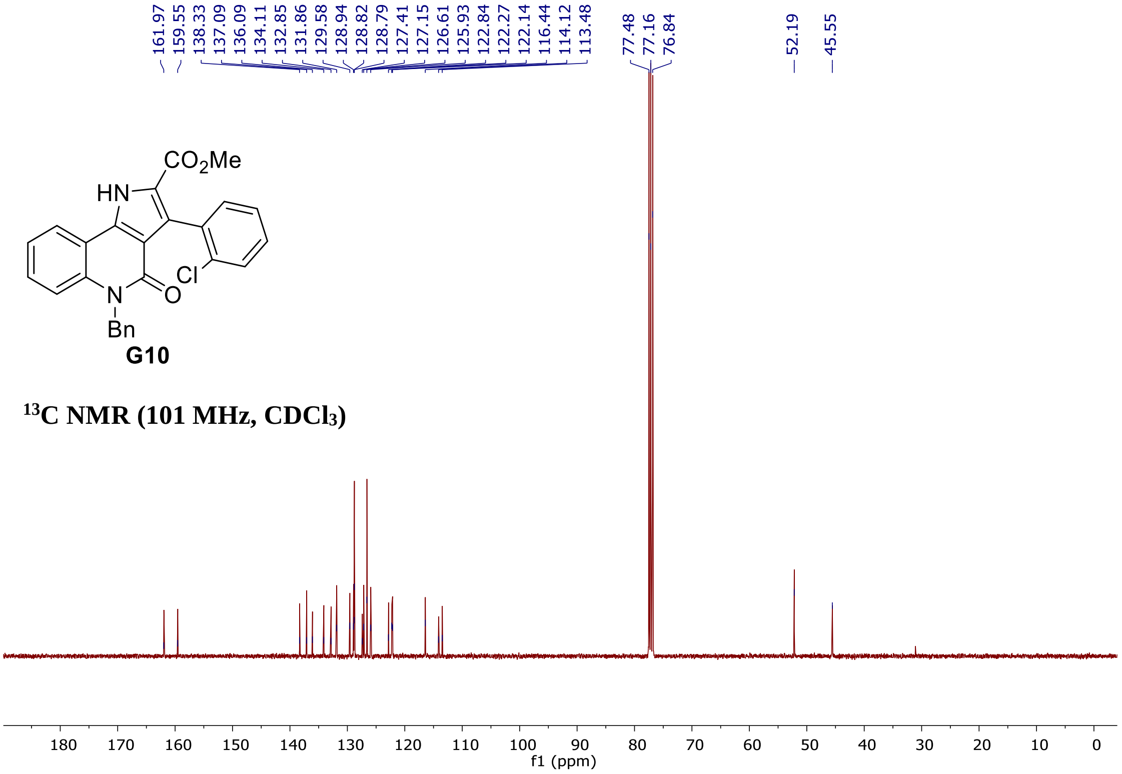
161.97
159.55
138.33
137.09
136.09
134.11
132.85
131.86
129.58
128.94
128.82
128.79
127.41
127.15
126.61
125.93
122.84
122.27
122.14
116.44
114.12
113.48
77.48
77.16
76.84
52.19
45.55
CO2Me
HN
Cl
N
O
Bn
G10
13C NMR (101 MHz, CDCl3)
f1 (ppm)

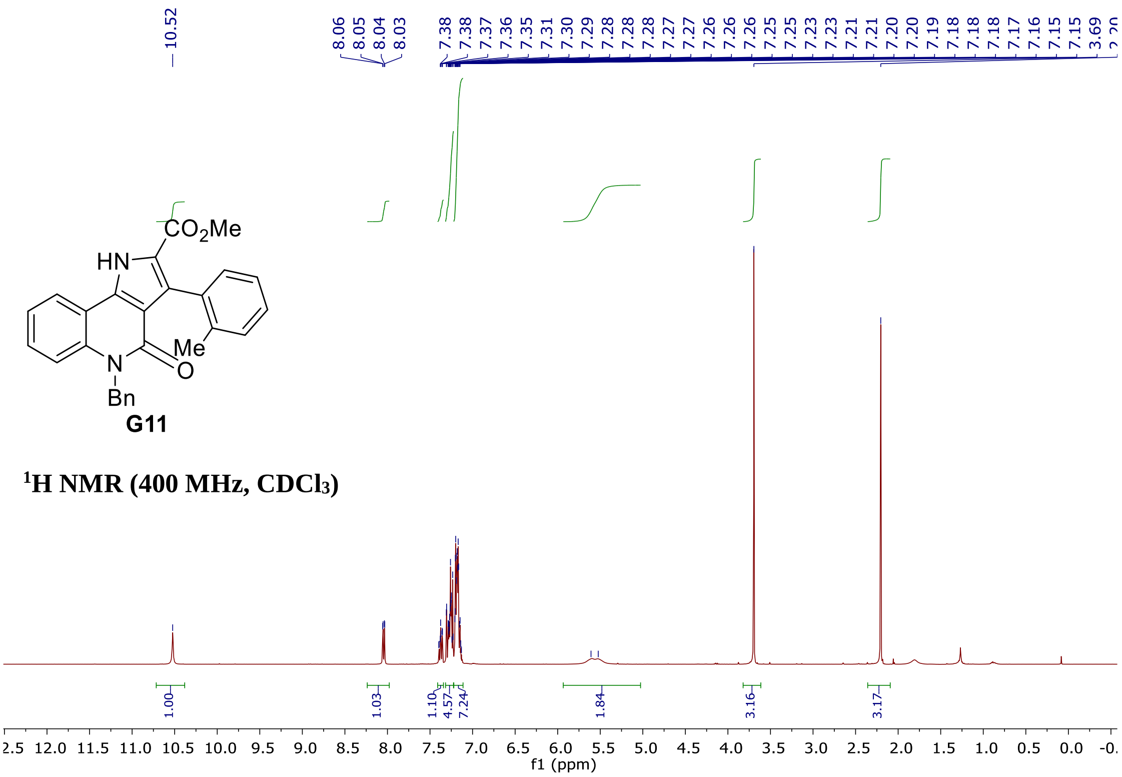

^{1}H NMR (400 MHz, CDCl3)

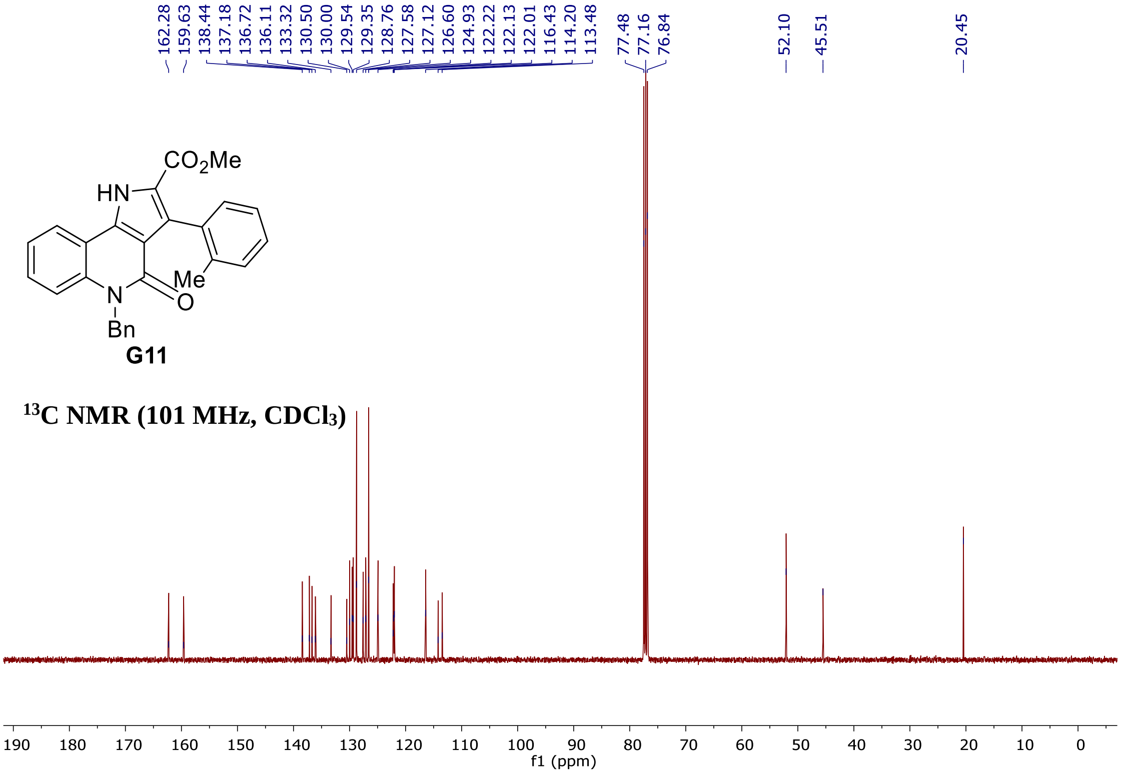

^{13}C NMR (101 MHz, CDCl3)

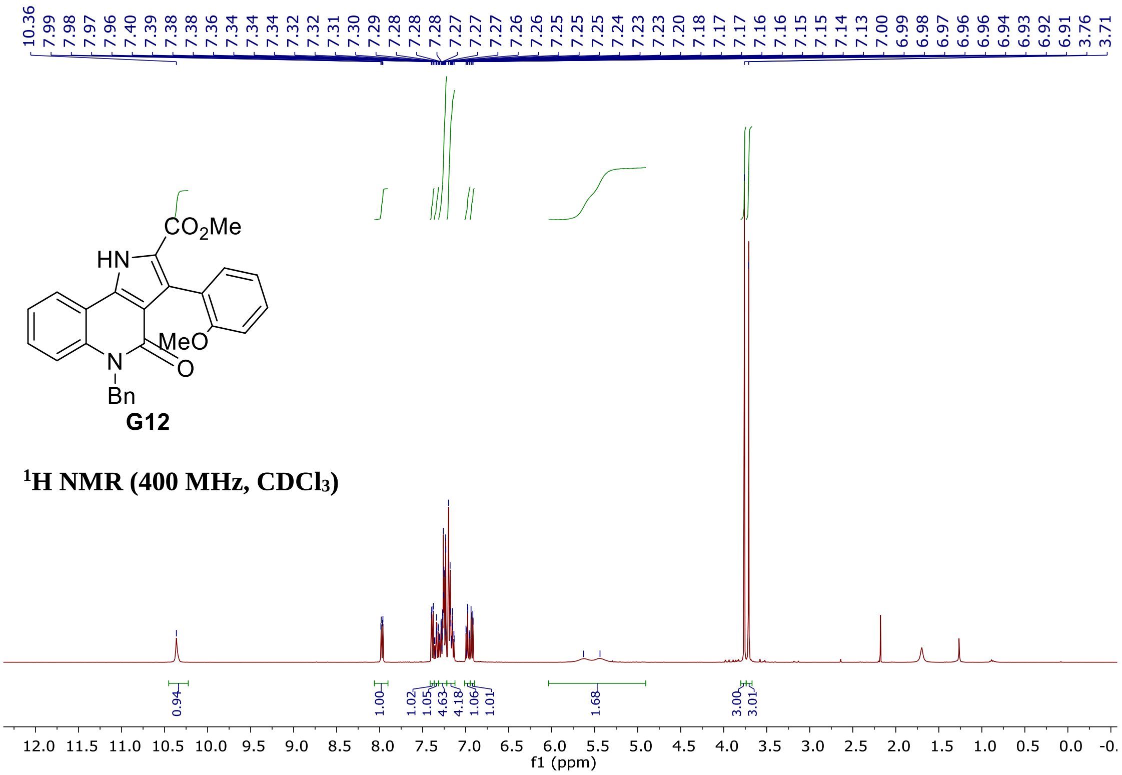
10.36 7.99 7.98 7.97 7.96 7.40 7.39 7.38 7.38 7.36 7.34 7.34 7.32 7.32 7.31 7.30 7.29 7.28 7.28 7.28 7.27 7.27 7.27 7.26 7.26 7.25 7.25 7.25 7.24 7.23 7.23 7.20 7.18 7.17 7.17 7.16 7.16 7.15 7.15 7.14 7.13 7.00 6.99 6.98 6.97 6.96 6.96 6.94 6.93 6.92 6.91 3.76 3.71
CO2Me
HN
MeO
N
O
Bn
G12
1H NMR (400 MHz, CDCl3)
0.94
1.00
1.02
1.05
4.63
4.18
1.06
1.01
1.68
3.00
3.01
12.0 11.5 11.0 10.5 10.0 9.5 9.0 8.5 8.0 7.5 7.0 6.5 6.0 5.5 5.0 4.5 4.0 3.5 3.0 2.5 2.0 1.5 1.0 0.5 0.0
f1 (ppm)

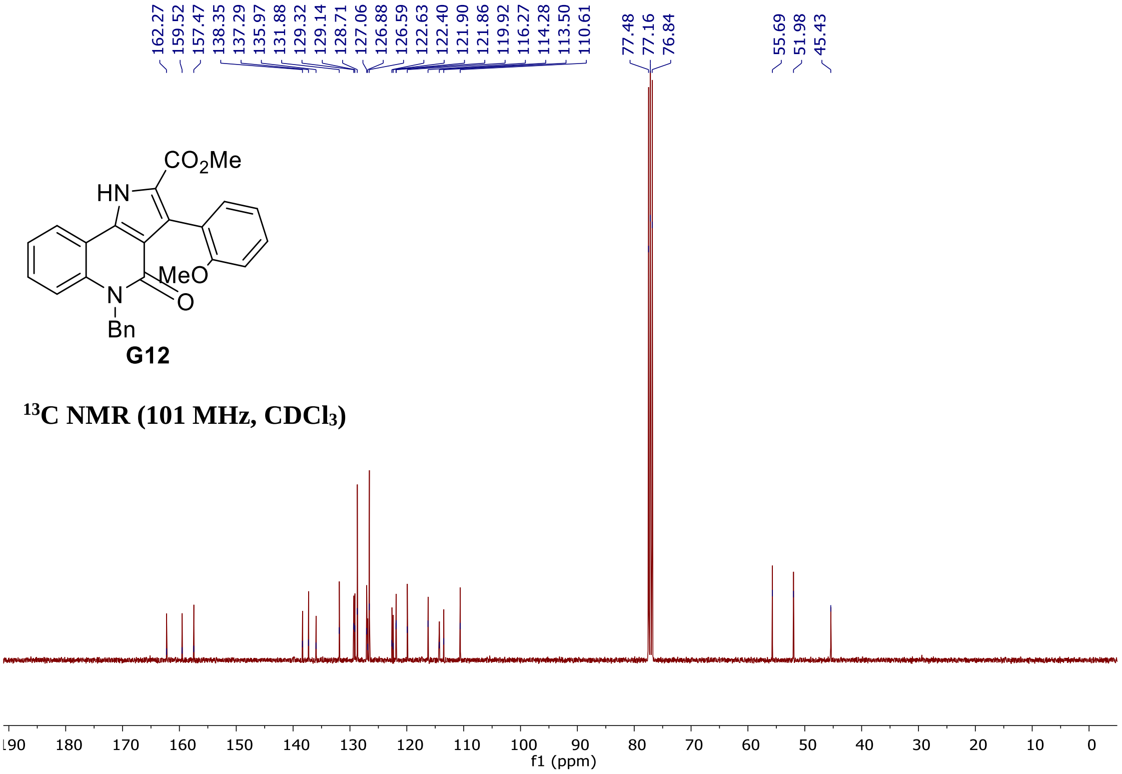
162.27 159.52 157.47 138.35 137.29 135.97 131.88 129.32 129.14 128.71 127.06 126.88 126.59 122.63 122.40 121.90 121.86 119.92 116.27 114.28 113.50 110.61
77.48 77.16 76.84
55.69 51.98 45.43
CO2Me
HN
MeO
N
O
Bn
G12
13C NMR (101 MHz, CDCl3)
180 170 160 150 140 130 120 110 100 90 80 70 60 50 40 30 20 10 0
f1 (ppm)

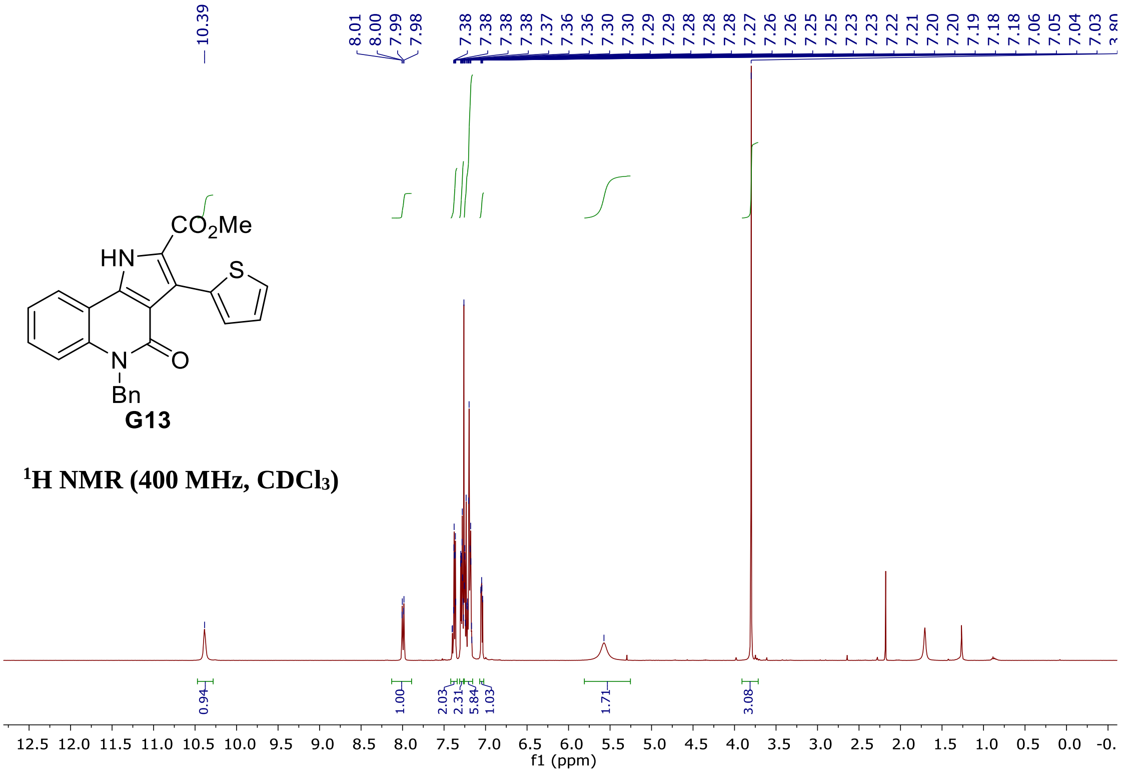
10.39
8.01 8.00 7.99 7.98
7.38 7.38 7.38 7.38 7.37 7.36 7.36 7.30 7.30 7.29 7.29 7.28 7.28 7.28 7.27 7.26 7.26 7.25 7.25 7.23 7.23 7.22 7.21 7.20 7.20 7.19 7.18 7.18 7.06 7.05 7.04 7.03
CO2Me
HN
S
N
O
Bn
G13
^{1}H NMR (400 MHz, CDCl$_3$)
0.94
1.00
2.03
2.31
5.84
1.03
1.71
3.08
12.5 12.0 11.5 11.0 10.5 10.0 9.5 9.0 8.5 8.0 7.5 7.0 6.5 6.0 5.5 5.0 4.5 4.0 3.5 3.0 2.5 2.0 1.5 1.0 0.5 0.0
f1 (ppm)

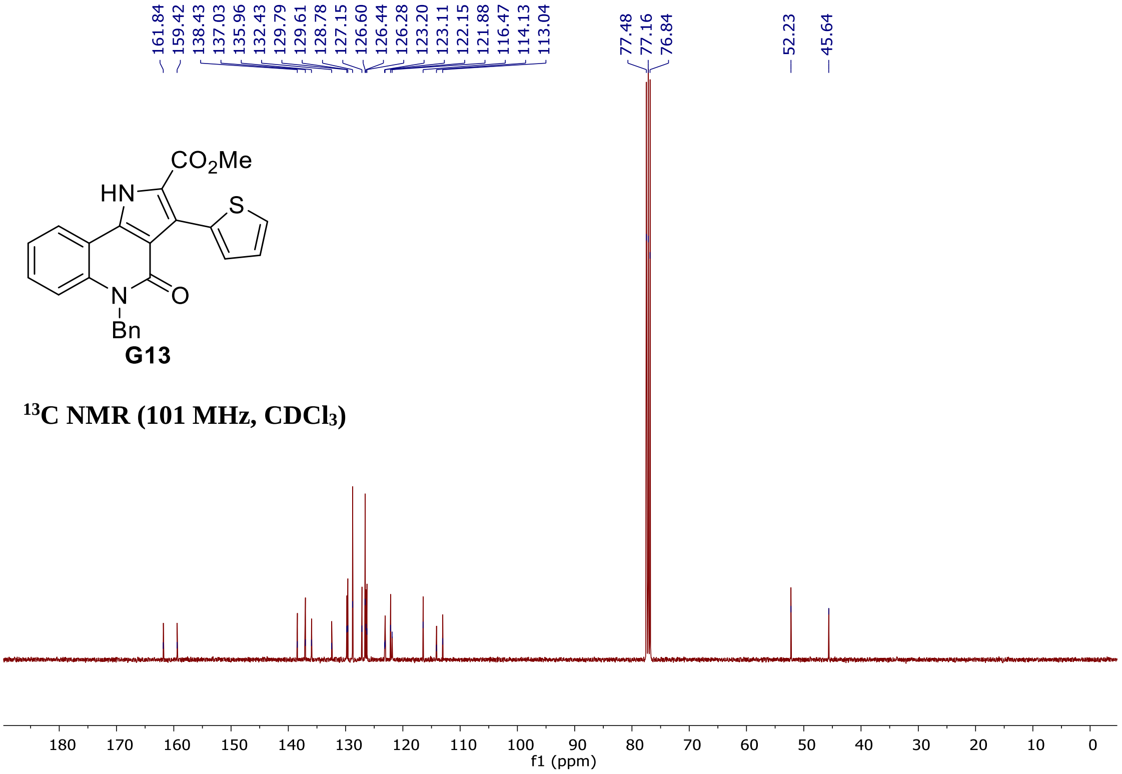
161.84 159.42 138.43 137.03 135.96 132.43 129.79 129.61 128.78 127.15 126.60 126.44 126.28 123.20 123.11 122.15 121.88 116.47 114.13 113.04
77.48 77.16 76.84
52.23
45.64
CO2Me
HN
S
N
O
Bn
G13
^{13}C NMR (101 MHz, CDCl$_3$)
180 170 160 150 140 130 120 110 100 90 80 70 60 50 40 30 20 10 0
f1 (ppm)

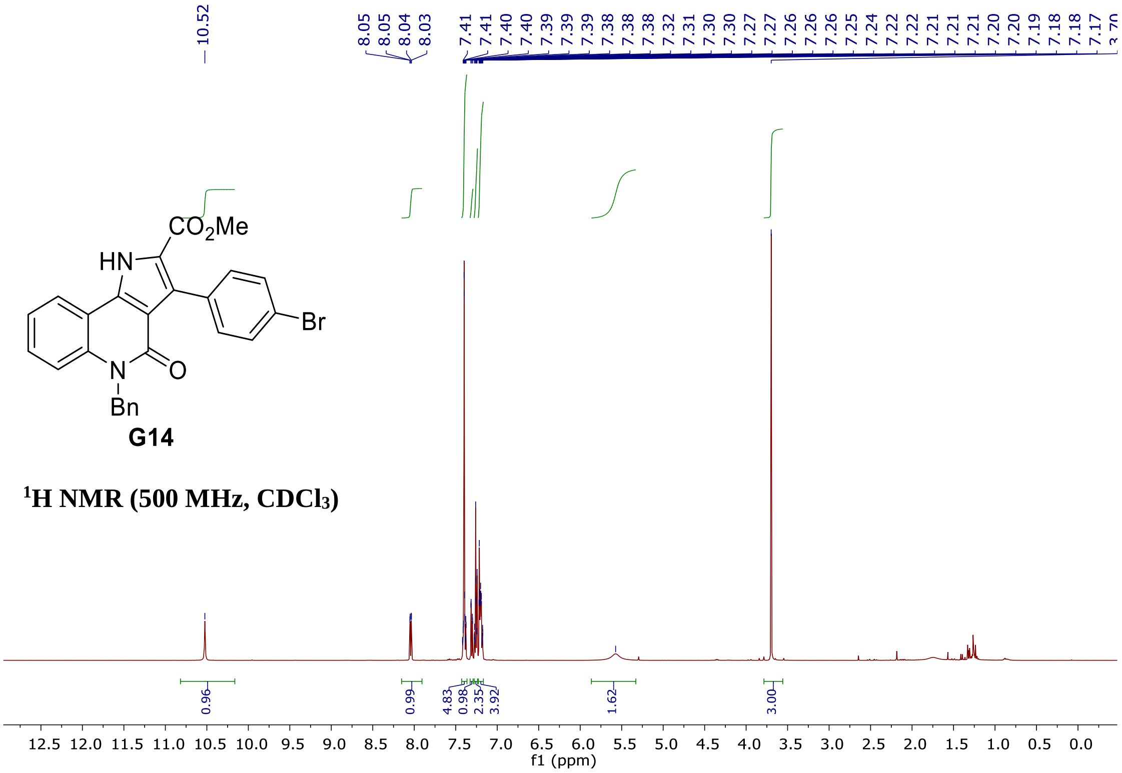
10.52
8.05 8.05 8.04 8.03
7.41 7.41 7.40 7.40 7.39 7.39 7.39 7.38 7.38 7.38 7.32 7.31 7.30 7.30 7.27 7.27 7.26 7.26 7.25 7.24 7.22 7.22 7.21 7.21 7.21 7.20 7.20 7.19 7.18 7.18 7.17
CO2Me
HN
Br
N
O
Bn
G14
^{1}H NMR (500 MHz, CDCl3)
0.96
0.99
4.83 0.98 2.35 3.92
1.62
3.00
12.5 12.0 11.5 11.0 10.5 10.0 9.5 9.0 8.5 8.0 7.5 7.0 6.5 6.0 5.5 5.0 4.5 4.0 3.5 3.0 2.5 2.0 1.5 1.0 0.5 0.0
f1 (ppm)

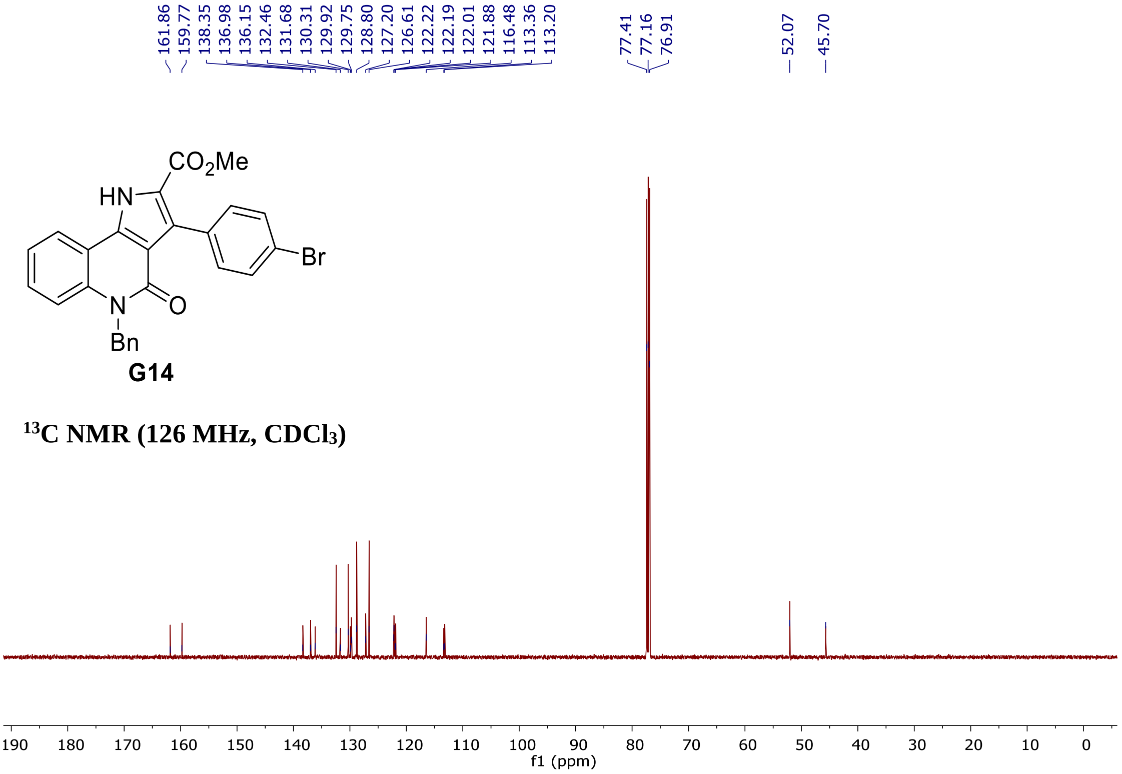
161.86 159.77 138.35 136.98 136.15 132.46 131.68 130.31 129.92 129.75 128.80 127.20 126.61 122.22 122.19 122.01 121.88 116.48 113.36 113.20
77.41 77.16 76.91
52.07
45.70
CO2Me
HN
Br
N
O
Bn
G14
^{13}C NMR (126 MHz, CDCl3)
190 180 170 160 150 140 130 120 110 100 90 80 70 60 50 40 30 20 10 0
f1 (ppm)

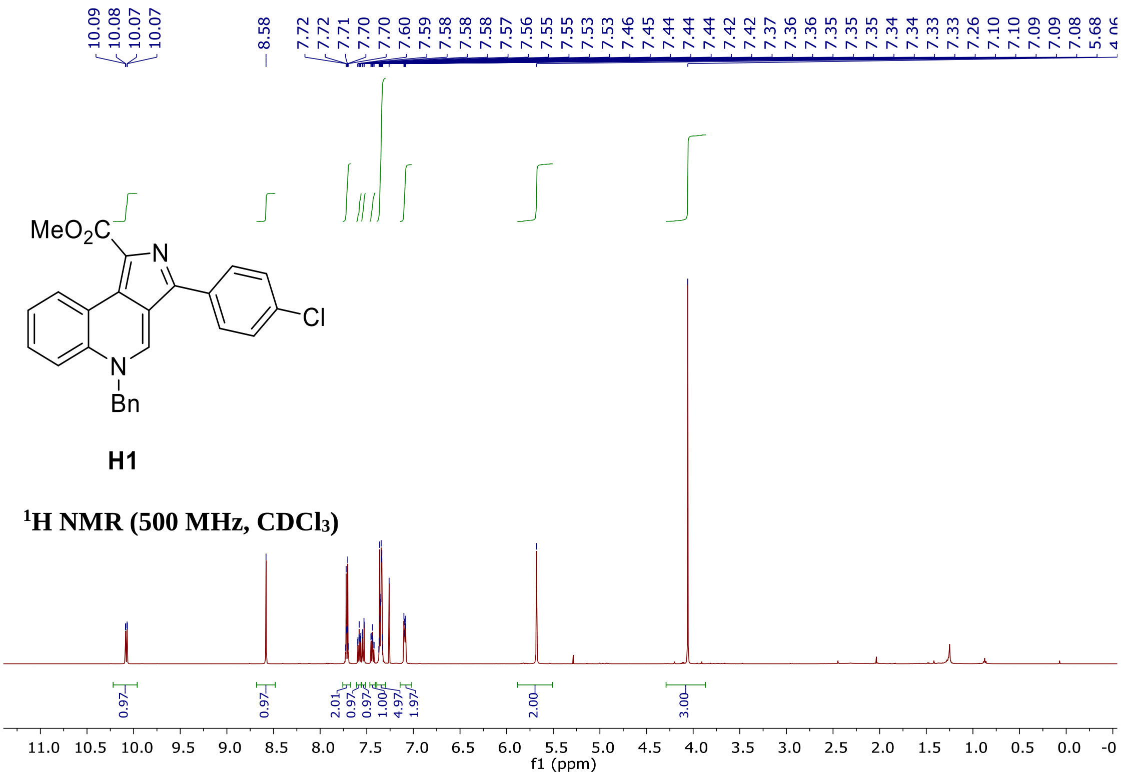

MeO2C
N
Cl
N
Bn
H1
1H NMR (500 MHz, CDCl3)
f1 (ppm)

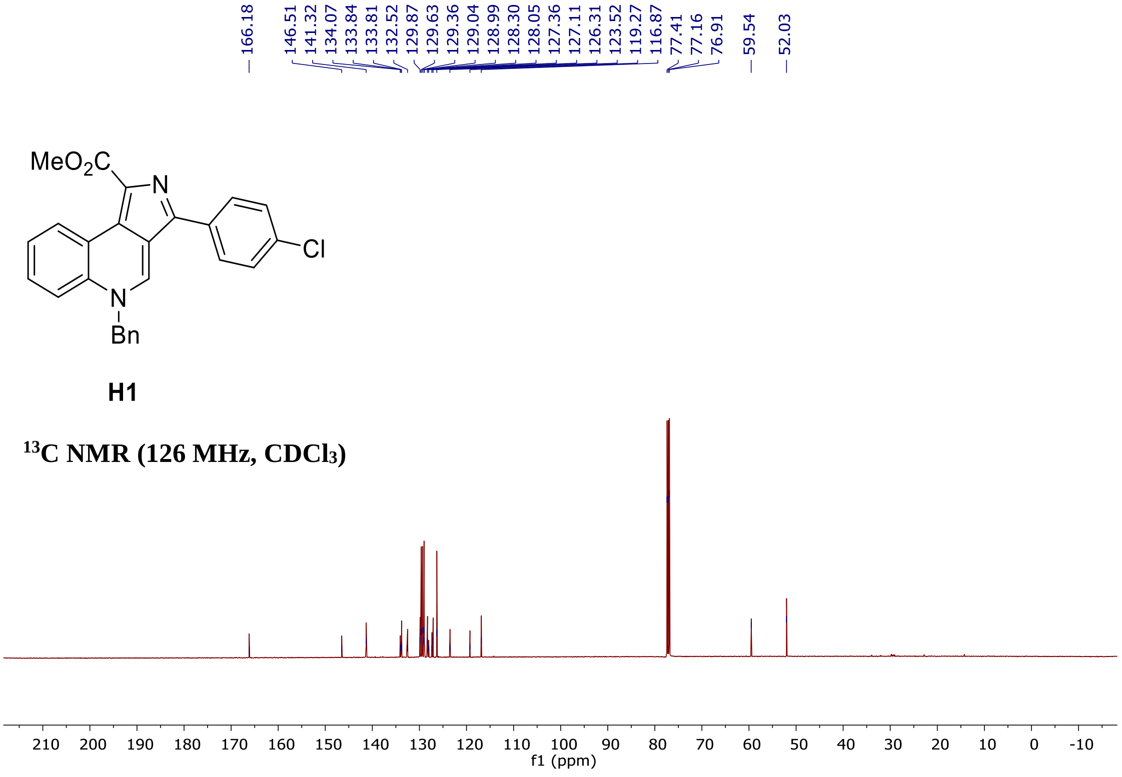

MeO2C
N
Cl
N
Bn
H1
13C NMR (126 MHz, CDCl3)
f1 (ppm)

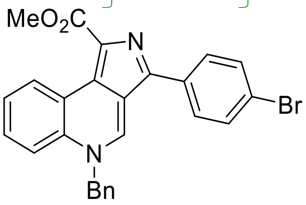

H2

^{1}H NMR (500 MHz, CDCl3)

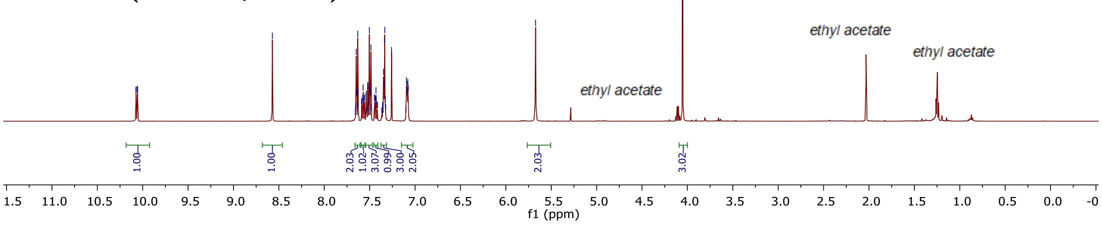

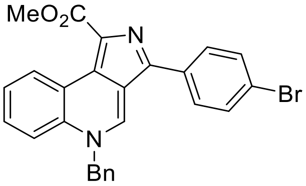

H2

^{13}C NMR (126 MHz, CDCl3)

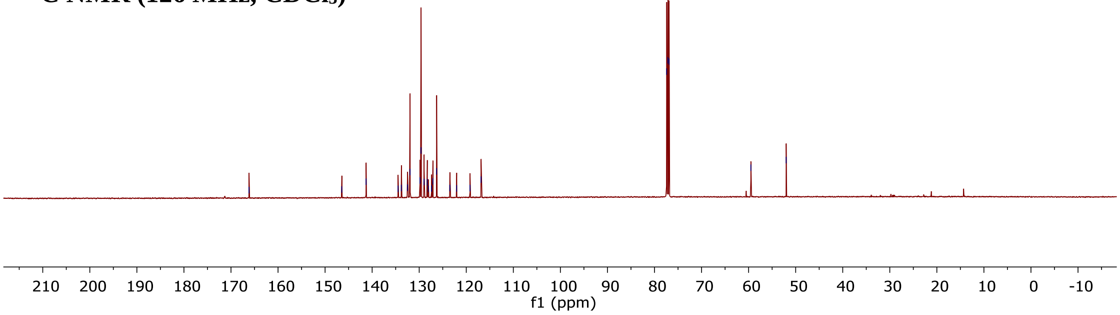

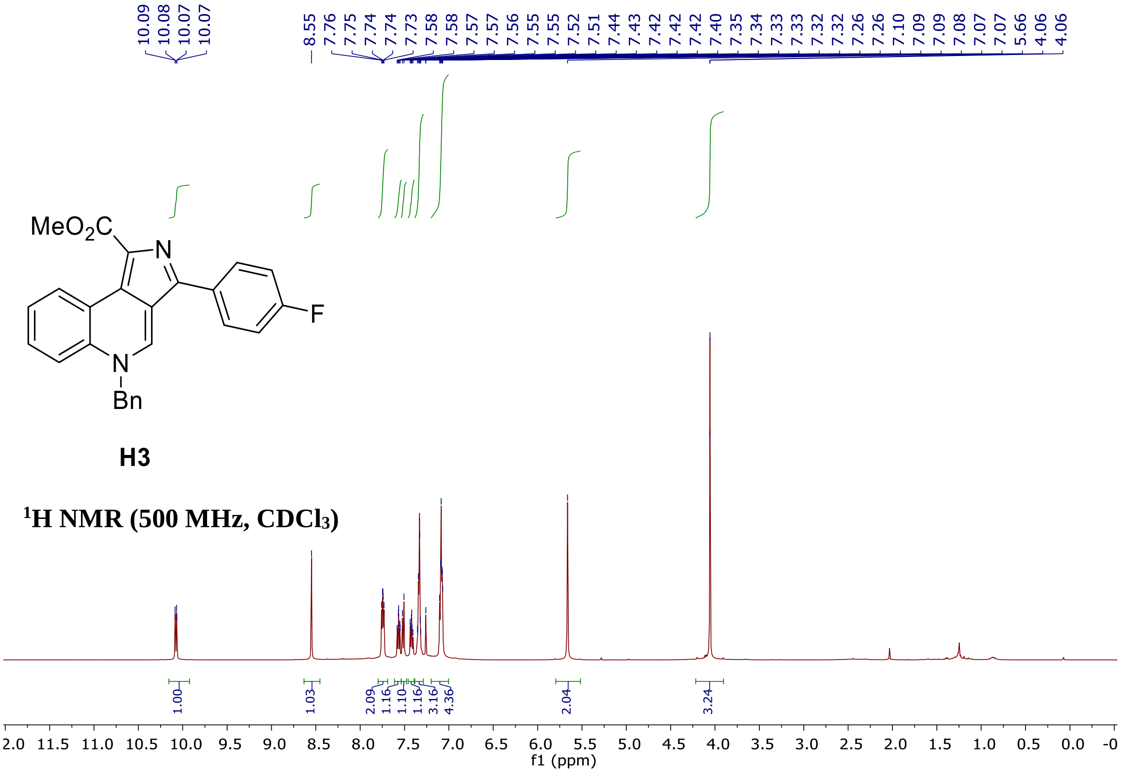
10.09
10.08
10.07
10.07
8.55
7.76
7.75
7.74
7.74
7.73
7.58
7.58
7.57
7.57
7.56
7.55
7.55
7.52
7.51
7.44
7.43
7.42
7.42
7.42
7.40
7.35
7.34
7.33
7.33
7.32
7.32
7.26
7.26
7.10
7.09
7.09
7.08
7.07
7.07
5.66
4.06
4.06
MeO2C
N
F
N
Bn
H3
1H NMR (500 MHz, CDCl3)
1.00
1.03
2.09
1.16
1.10
1.16
3.16
4.36
2.04
3.24
f1 (ppm)

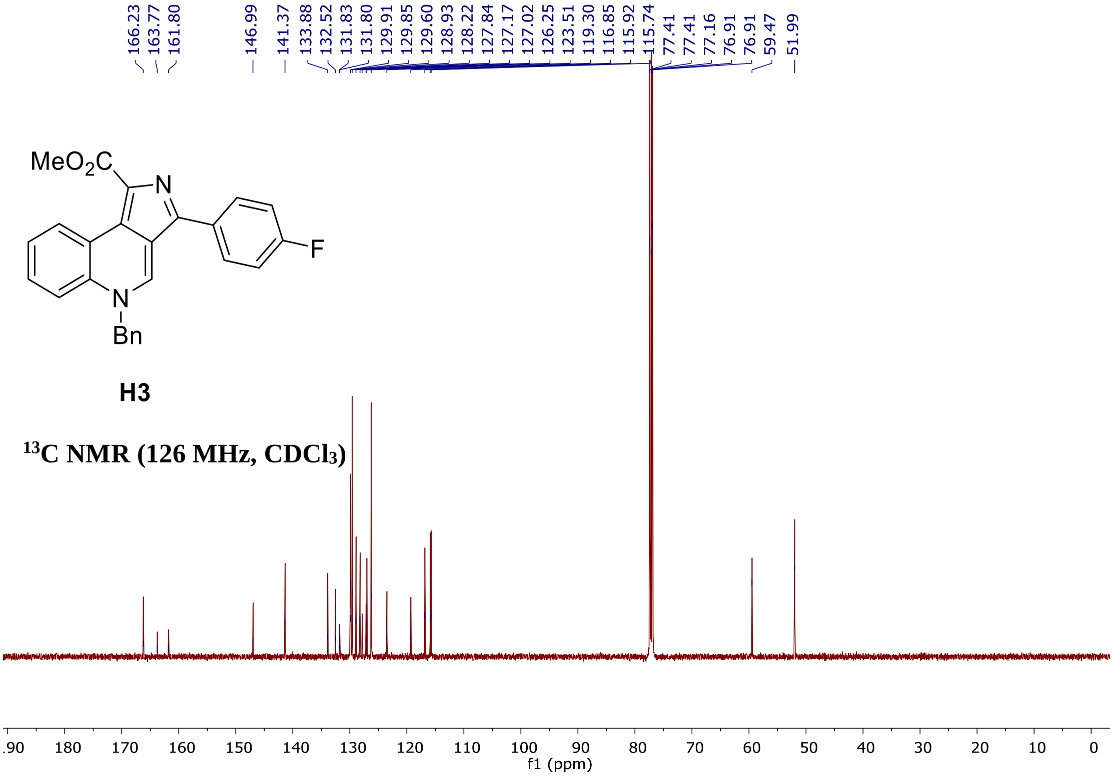
166.23
163.77
161.80
146.99
141.37
133.88
132.52
131.83
131.80
129.91
129.85
129.60
128.93
128.22
127.84
127.17
127.02
126.25
123.51
119.30
116.85
115.92
115.74
77.41
77.41
77.16
76.91
76.91
59.47
51.99
MeO2C
N
F
N
Bn
H3
13C NMR (126 MHz, CDCl3)
f1 (ppm)

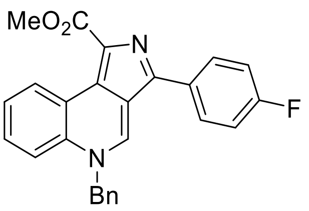

H3

^{19}F NMR (470 MHz, $CDCl_3$)

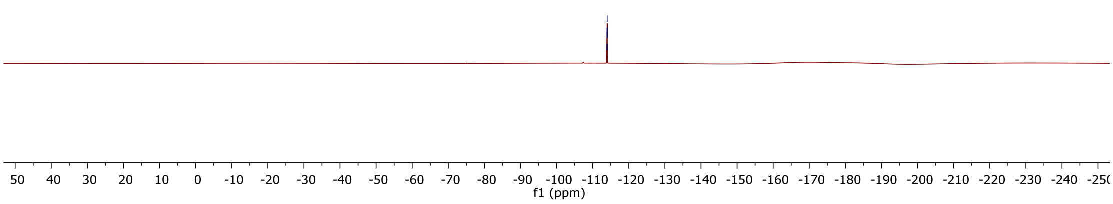

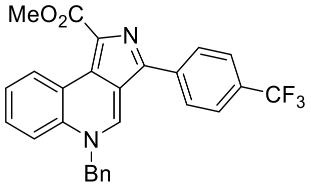

H4

^{19}F NMR (470 MHz, $CDCl_3$)

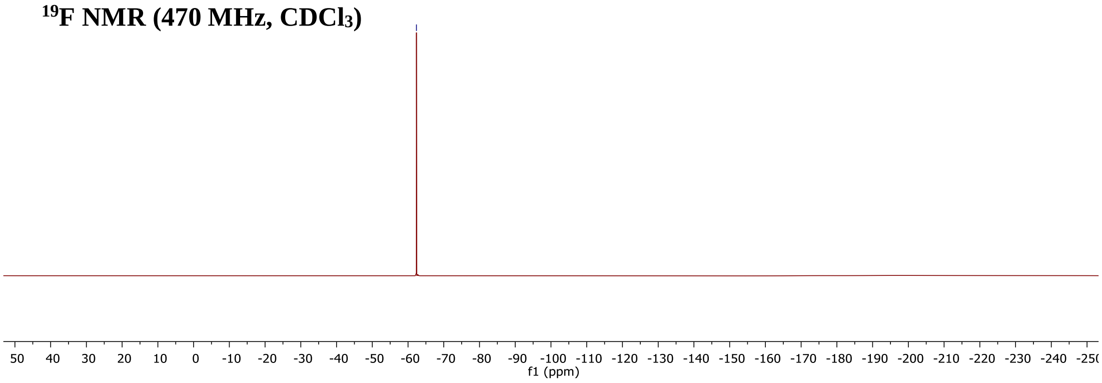

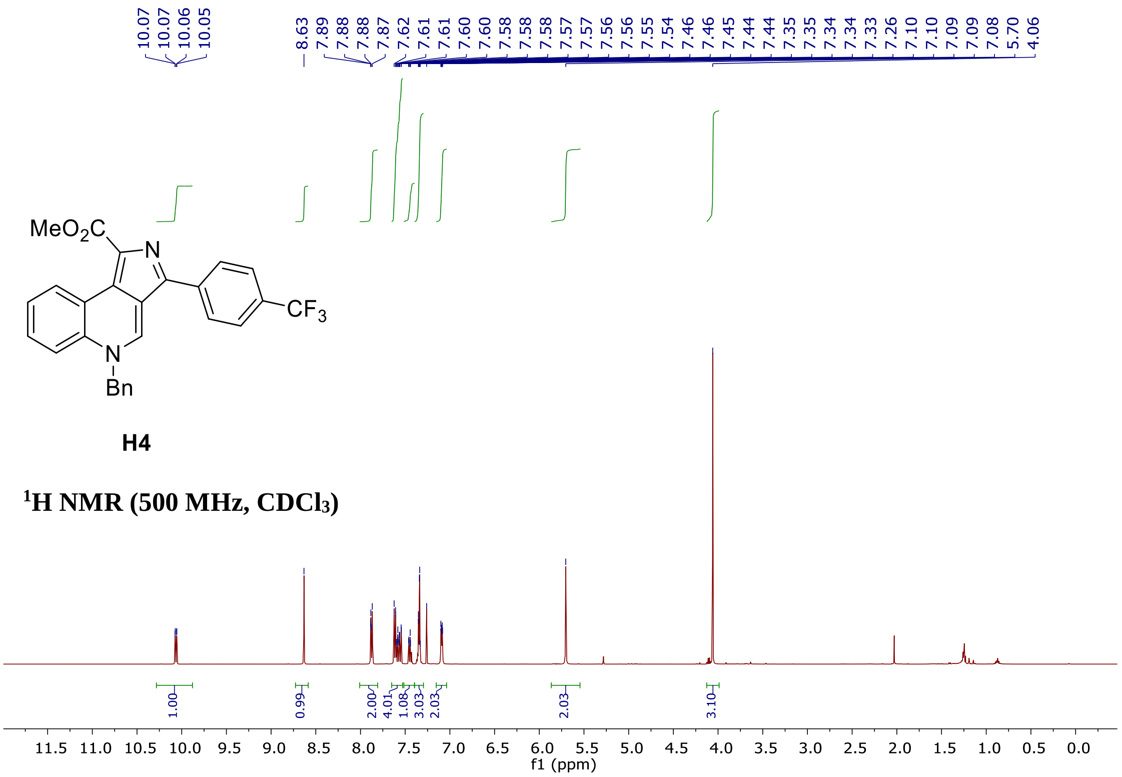

H4

^{1}H NMR (500 MHz, CDCl$_3$)

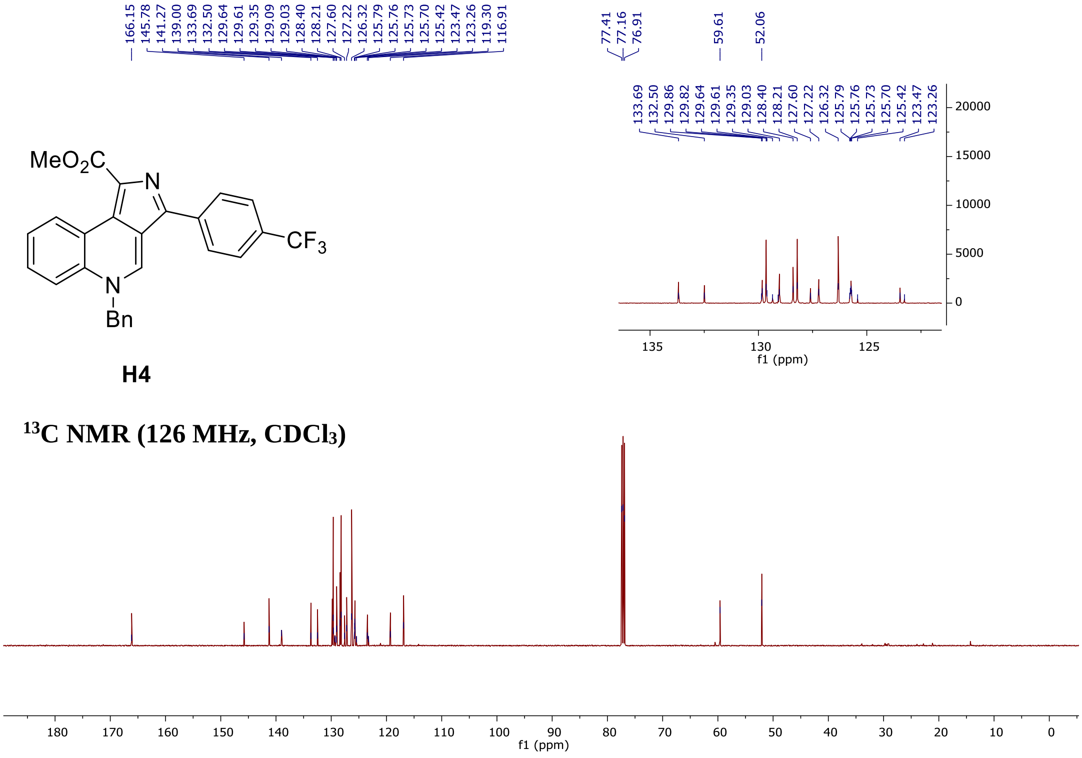

H4

^{13}C NMR (126 MHz, CDCl$_3$)

H5

^{1}H NMR (700 MHz, $CDCl_3$)

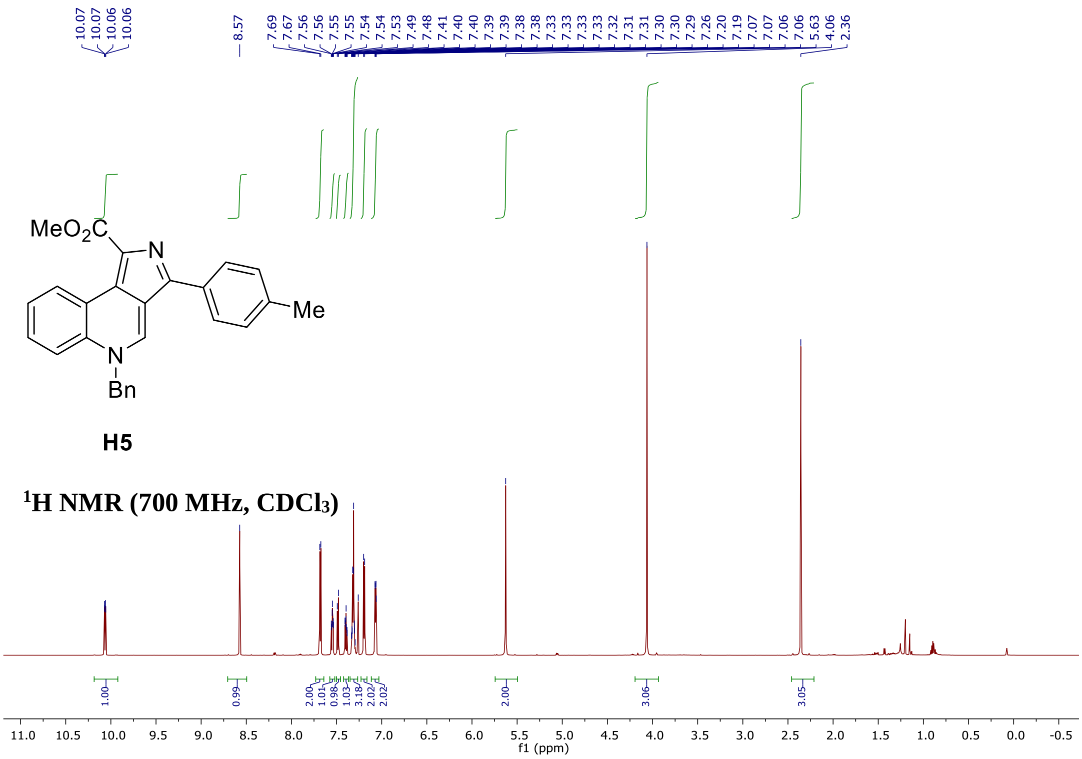

H5

^{13}C NMR (176 MHz, $CDCl_3$)

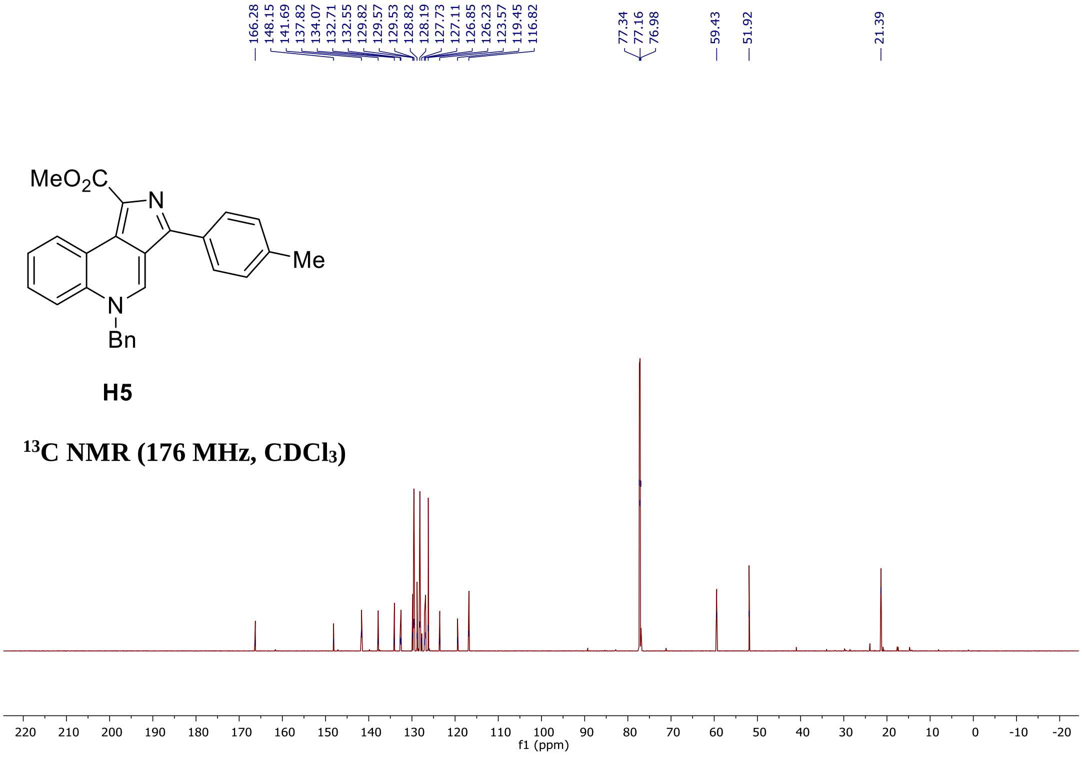

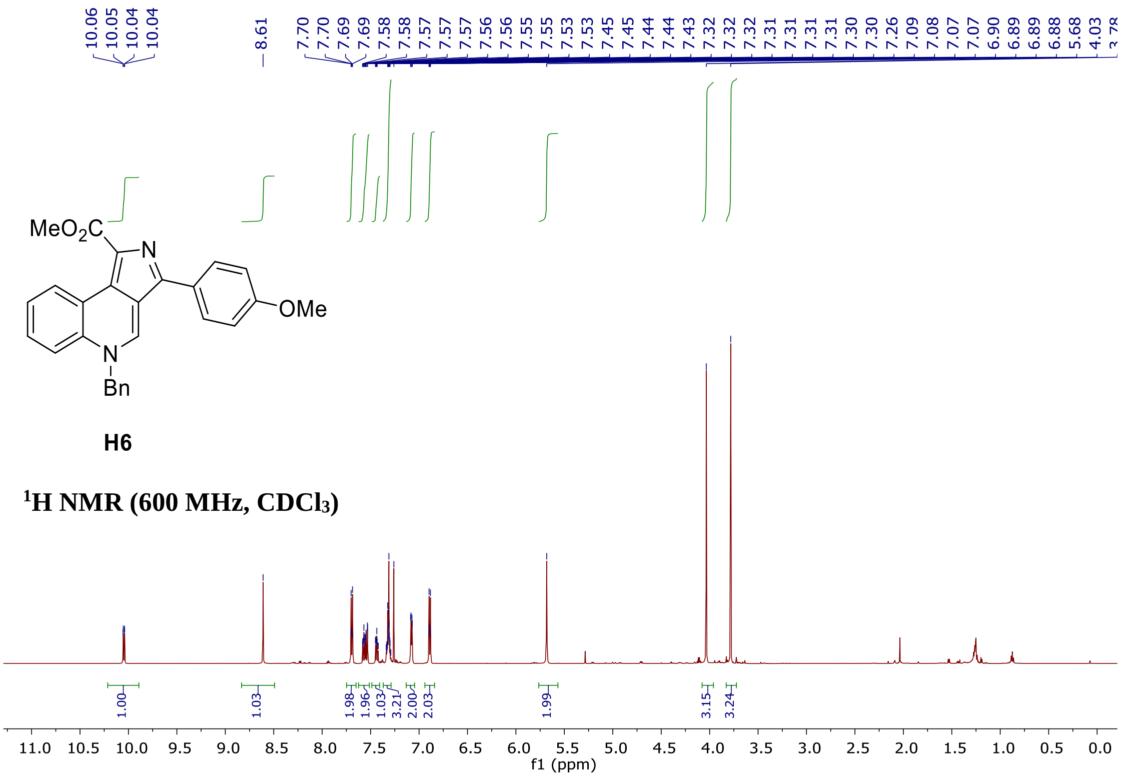

H6

^{1}H NMR (600 MHz, $CDCl_3$)

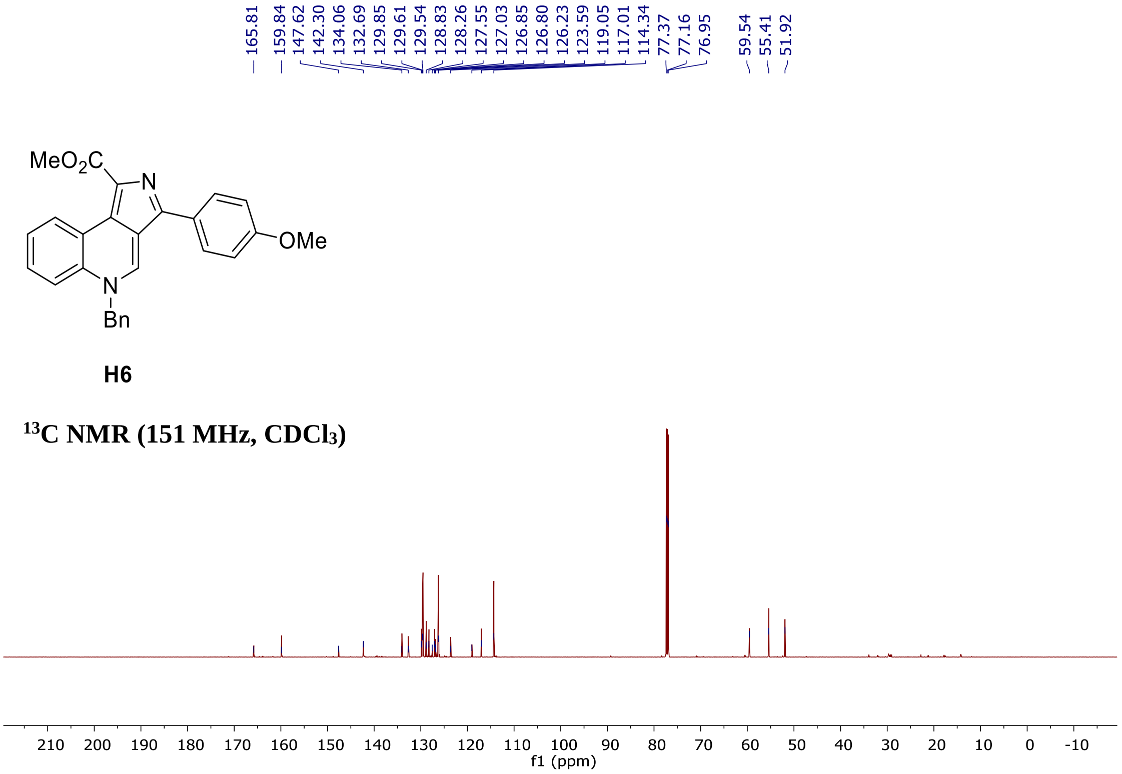

H6

^{13}C NMR (151 MHz, $CDCl_3$)

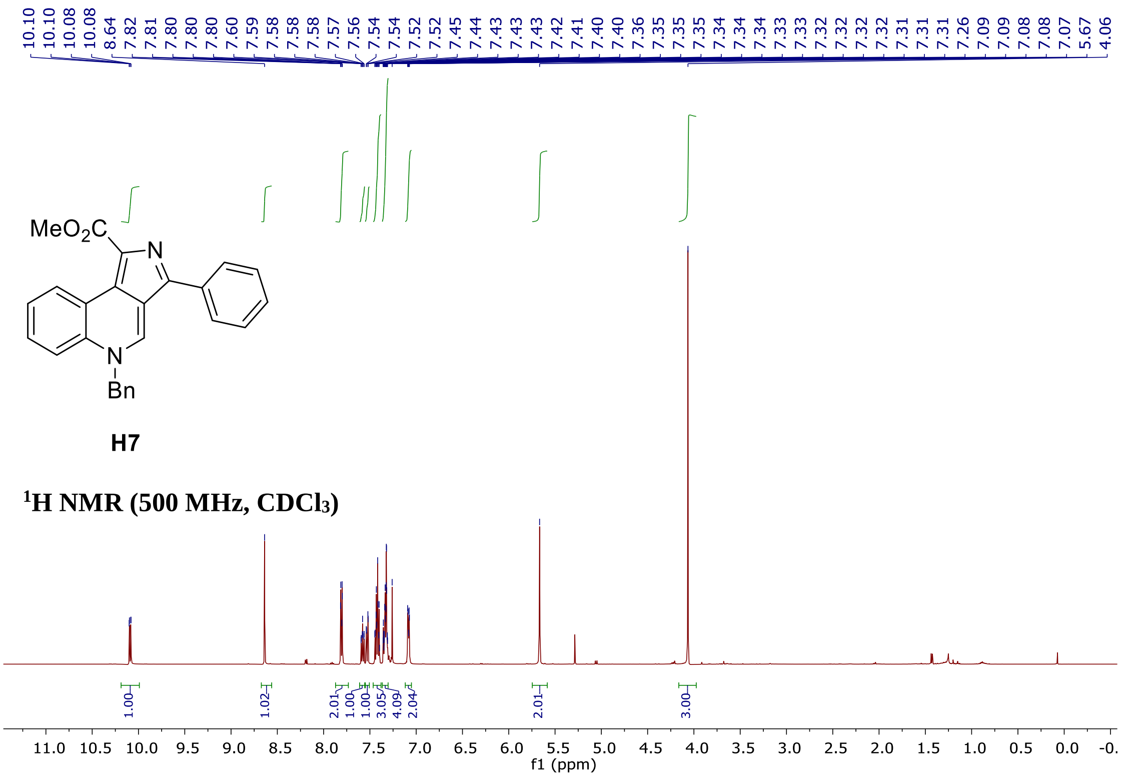

H7

^{1}H NMR (500 MHz, $CDCl_3$)

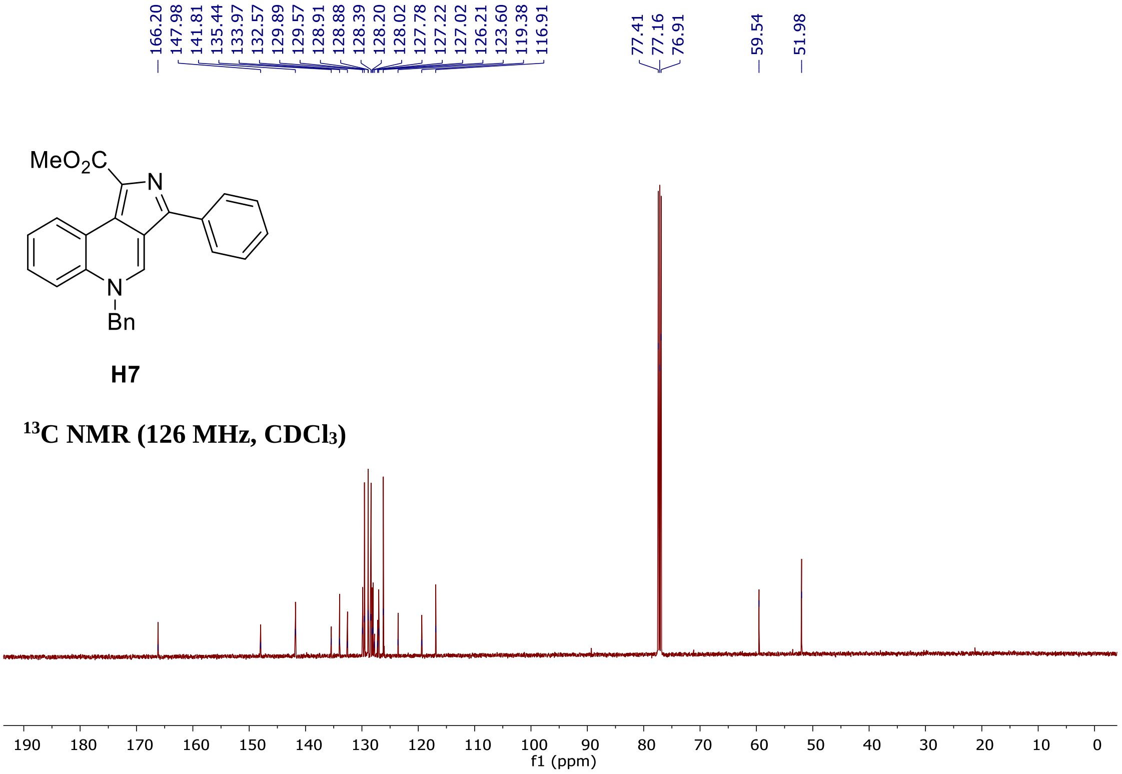

H7

^{13}C NMR (126 MHz, $CDCl_3$)

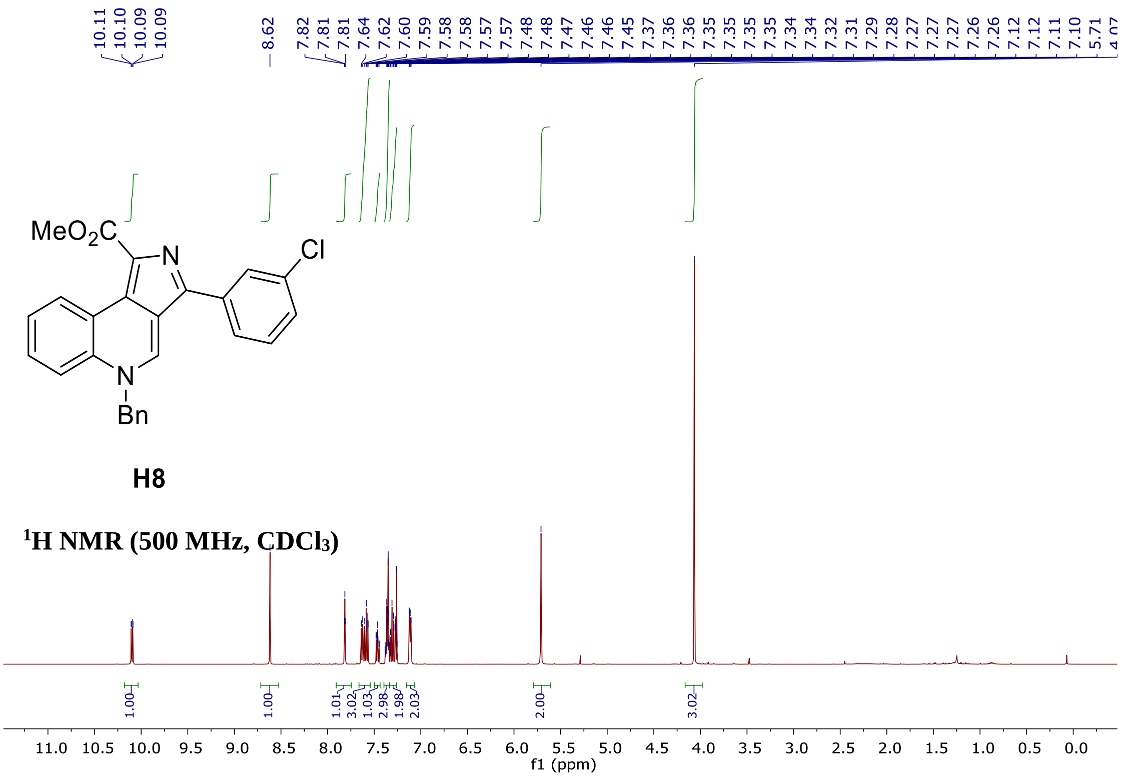
10.11 10.10 10.09 10.09
8.62
7.82 7.81 7.81 7.64 7.62 7.60 7.59 7.58 7.58 7.57 7.57 7.48 7.48 7.47 7.46 7.46 7.45 7.37 7.36 7.36 7.35 7.35 7.35 7.34 7.34 7.32 7.31 7.29 7.28 7.27 7.27 7.27 7.26 7.26 7.12 7.12 7.11 7.10
5.71
MeO2C
N
Cl
N
Bn
H8
1H NMR (500 MHz, CDCl3)
1.00 1.00 1.01 3.02 1.03 2.98 1.98 2.03 2.00 3.02
11.0 10.5 10.0 9.5 9.0 8.5 8.0 7.5 7.0 6.5 6.0 5.5 5.0 4.5 4.0 3.5 3.0 2.5 2.0 1.5 1.0 0.5 0.0
f1 (ppm)

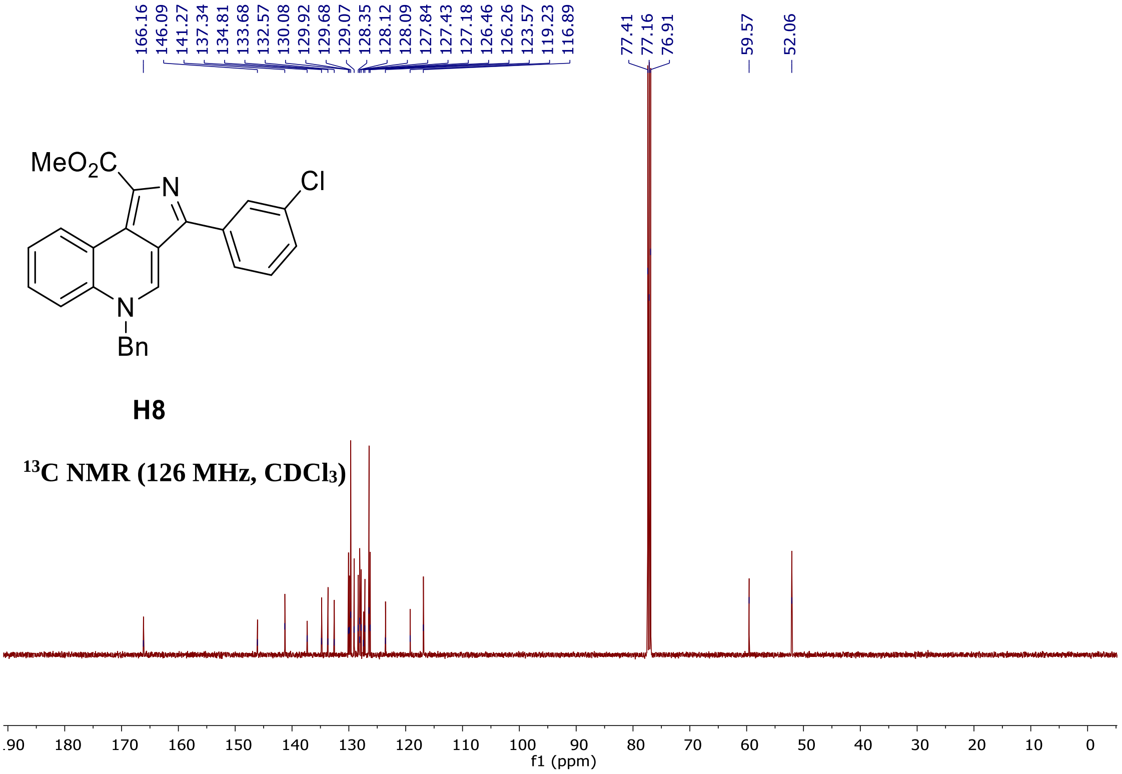
166.16 146.09 141.27 137.34 134.81 133.68 132.57 130.08 129.92 129.68 129.07 128.35 128.12 128.09 127.84 127.43 127.18 126.46 126.26 123.57 119.23 116.89
77.41 77.16 76.91
59.57
52.06
MeO2C
N
Cl
N
Bn
H8
13C NMR (126 MHz, CDCl3)
180 170 160 150 140 130 120 110 100 90 80 70 60 50 40 30 20 10 0
f1 (ppm)

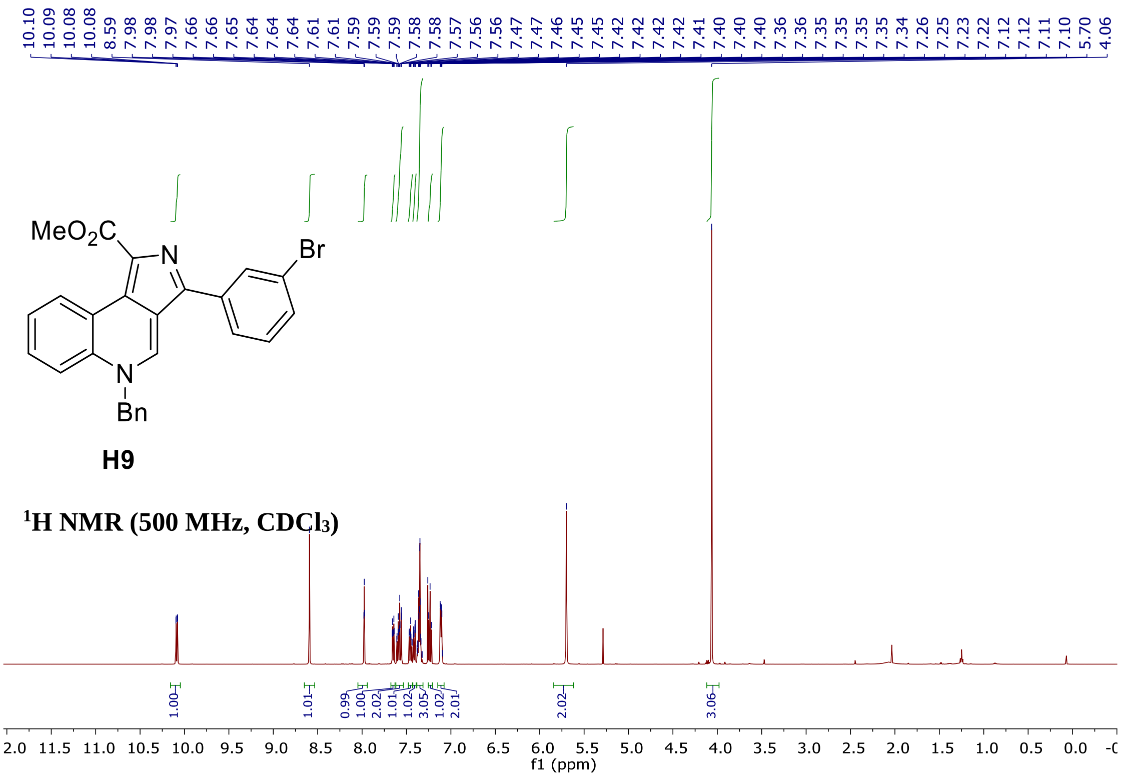
10.10 10.09 10.08 10.08 8.59 7.98 7.98 7.97 7.66 7.66 7.65 7.64 7.64 7.64 7.61 7.61 7.59 7.59 7.59 7.58 7.58 7.57 7.56 7.56 7.47 7.47 7.46 7.45 7.45 7.42 7.42 7.42 7.42 7.41 7.40 7.40 7.40 7.36 7.36 7.35 7.35 7.35 7.35 7.34 7.26 7.25 7.23 7.22 7.12 7.12 7.11 7.10 5.70 4.06
MeO2C
N
Br
N
Bn
H9
1H NMR (500 MHz, CDCl3)
1.00 1.01 0.99 1.00 2.02 1.01 1.02 3.05 1.02 2.01 2.02 3.06
f1 (ppm)

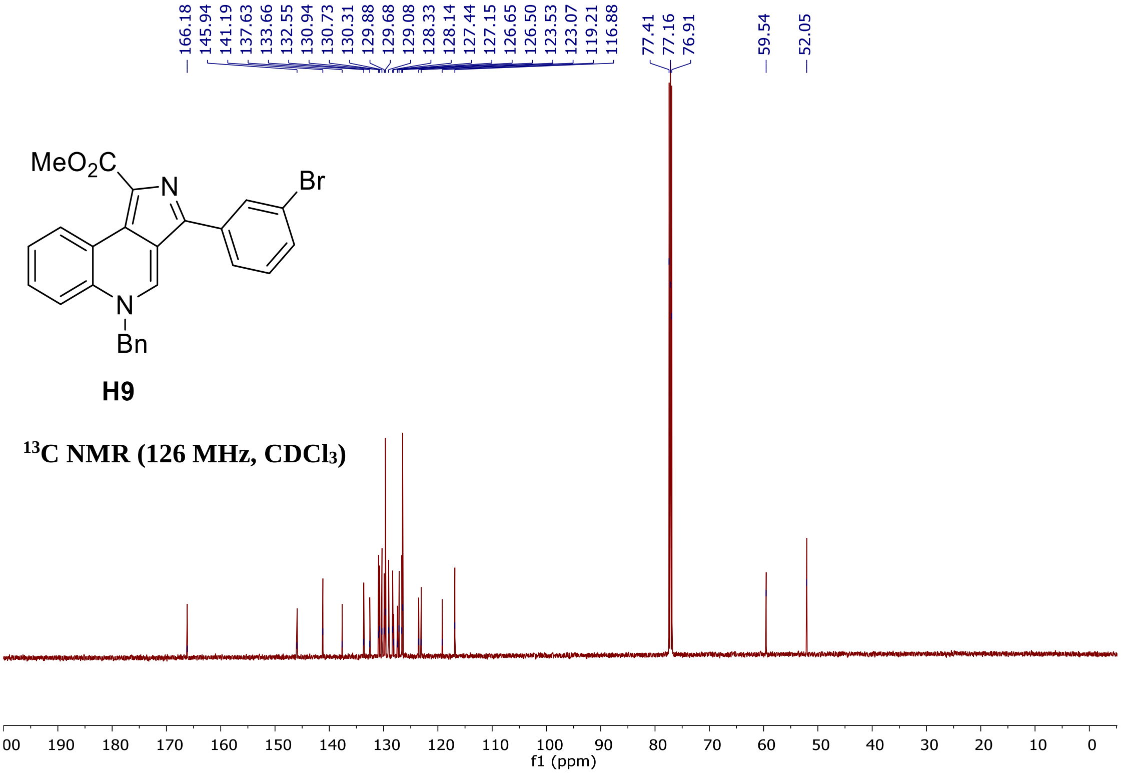
166.18 145.94 141.19 137.63 133.66 132.55 130.94 130.73 130.31 129.88 129.68 129.08 128.33 128.14 127.44 127.15 126.65 126.50 123.53 123.07 119.21 116.88 77.41 77.16 76.91 59.54 52.05
MeO2C
N
Br
N
Bn
H9
13C NMR (126 MHz, CDCl3)
f1 (ppm)

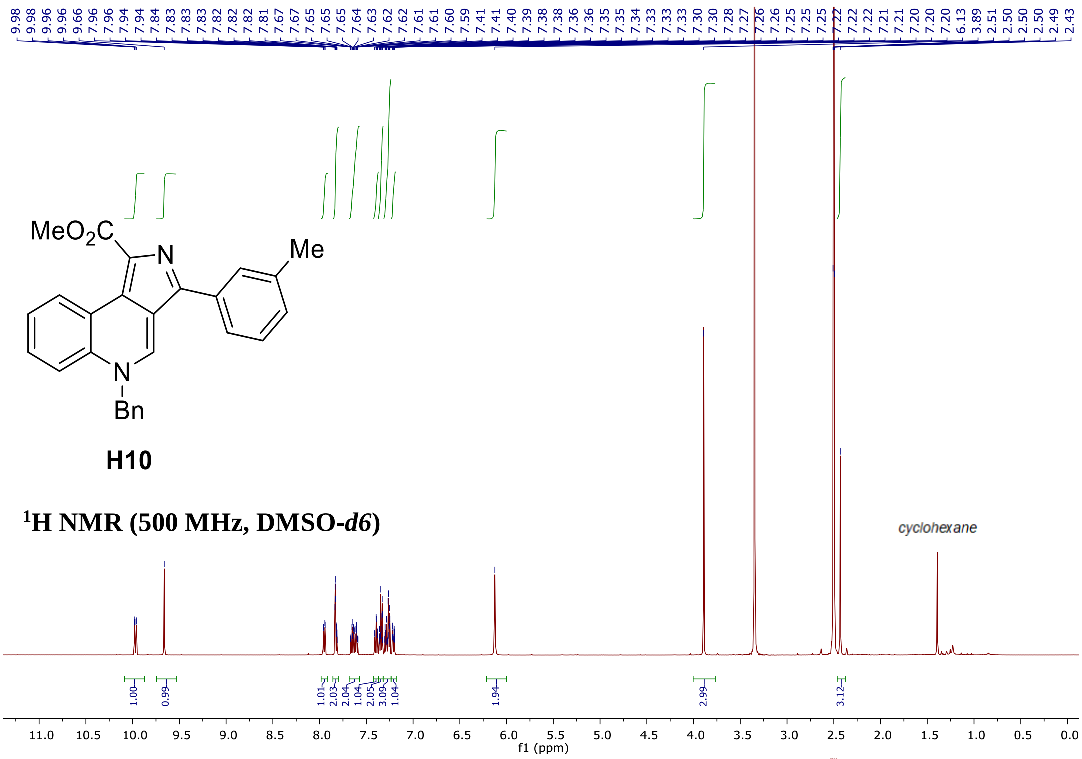

H10

^{1}H NMR (500 MHz, DMSO-*d6*)

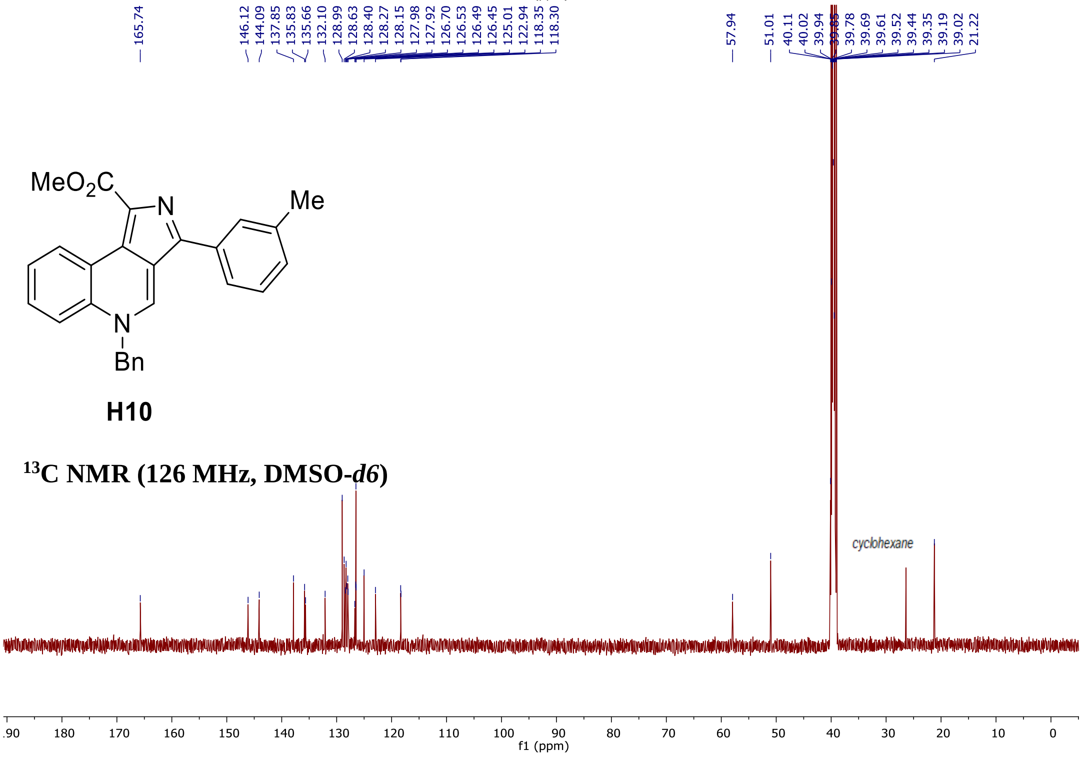

H10

^{13}C NMR (126 MHz, DMSO-*d6*)

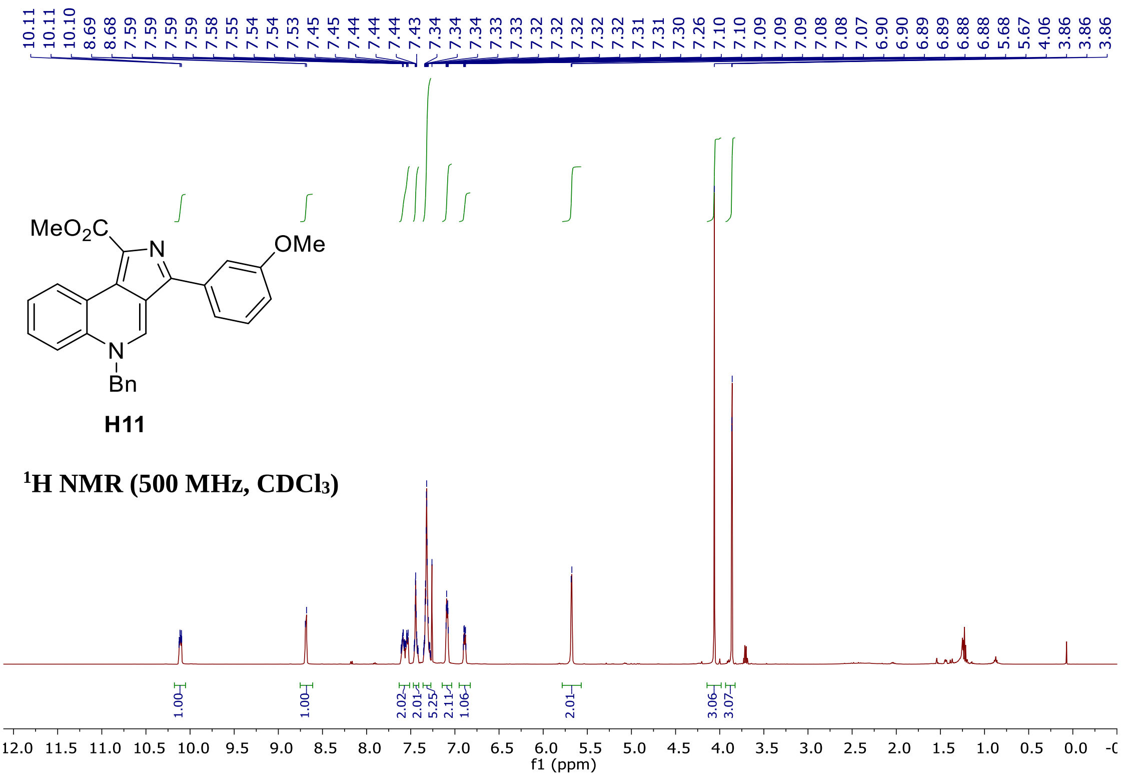
10.11 10.11 10.10 8.69 8.68 7.59 7.59 7.59 7.58 7.55 7.54 7.54 7.53 7.45 7.45 7.44 7.44 7.44 7.43 7.34 7.34 7.33 7.33 7.32 7.32 7.32 7.32 7.31 7.31 7.30 7.26 7.10 7.10 7.09 7.09 7.08 7.08 7.07 6.90 6.90 6.89 6.89 6.88 6.88 5.68 5.67 4.06 3.86 3.86
MeO2C
N
OMe
N
Bn
H11
1H NMR (500 MHz, CDCl3)
1.00 1.00 2.02 2.01 5.25 2.11 1.06 2.01 3.06 3.07
f1 (ppm)

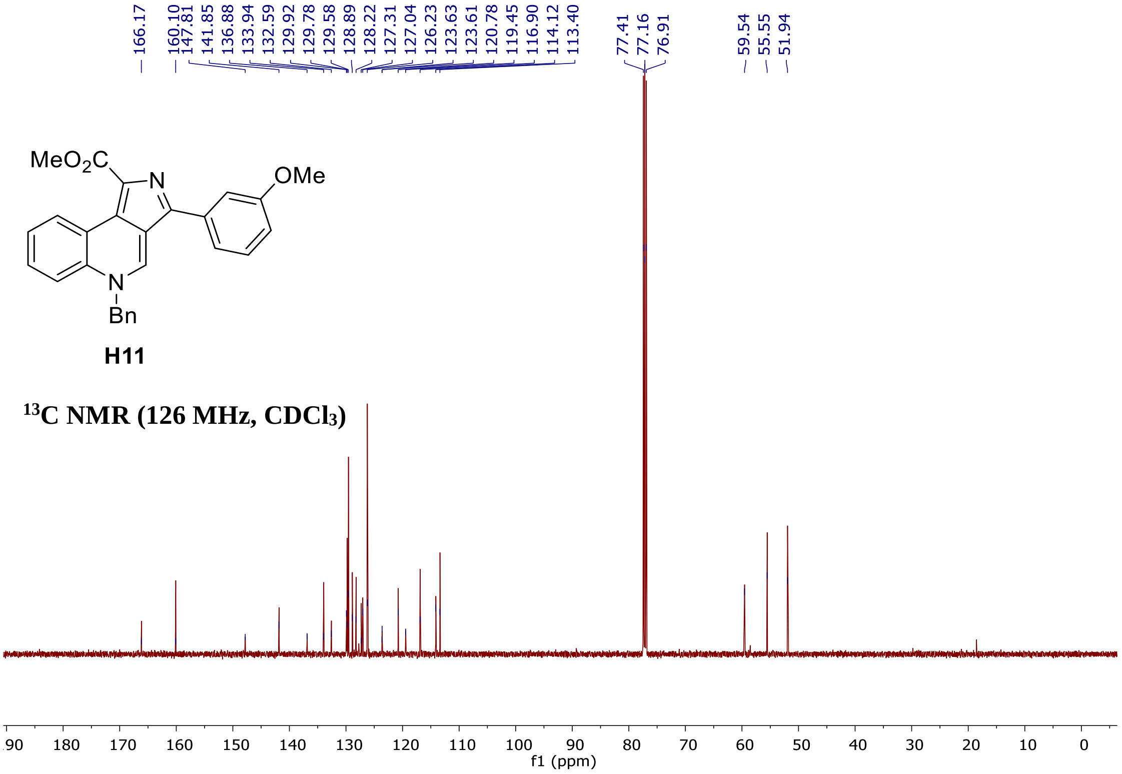
166.17 160.10 147.81 141.85 136.88 133.94 132.59 129.92 129.78 129.58 128.89 128.22 127.31 127.04 126.23 123.63 123.61 120.78 119.45 116.90 114.12 113.40 77.41 77.16 76.91 59.54 55.55 51.94
MeO2C
N
OMe
N
Bn
H11
13C NMR (126 MHz, CDCl3)
f1 (ppm)

H12

^{1}H NMR (500 MHz, CDCl₃)

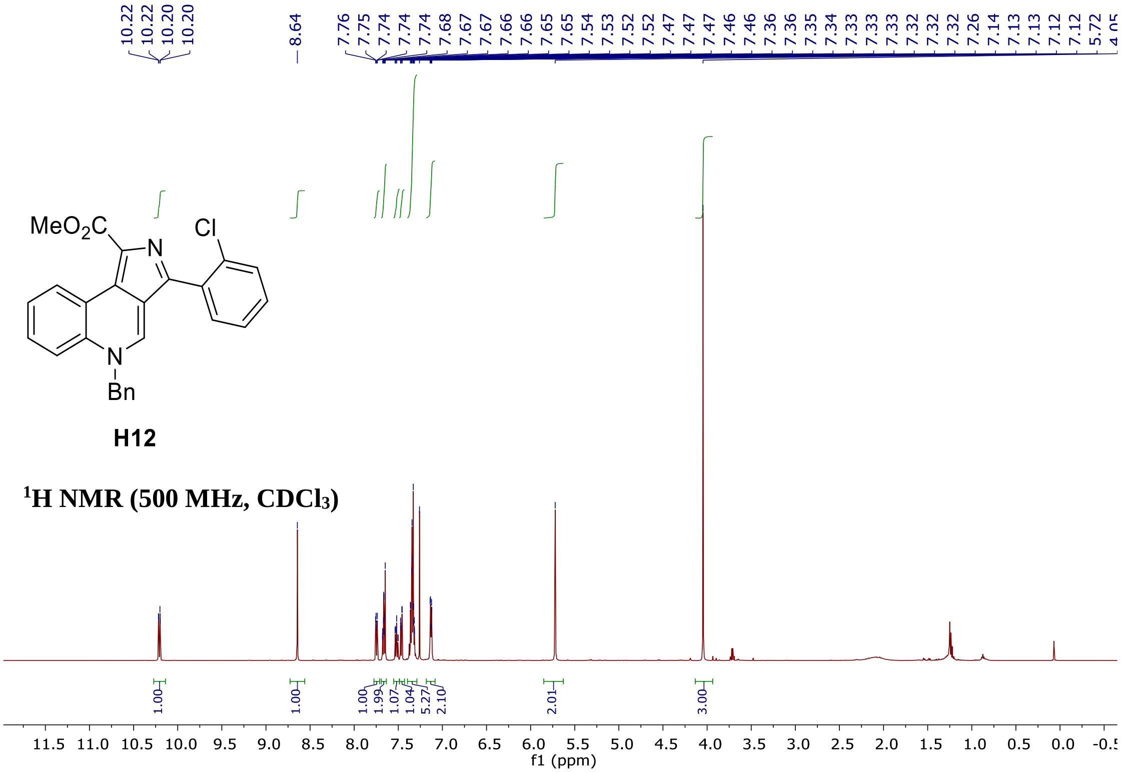

H12

^{13}C NMR (126 MHz, CDCl₃)

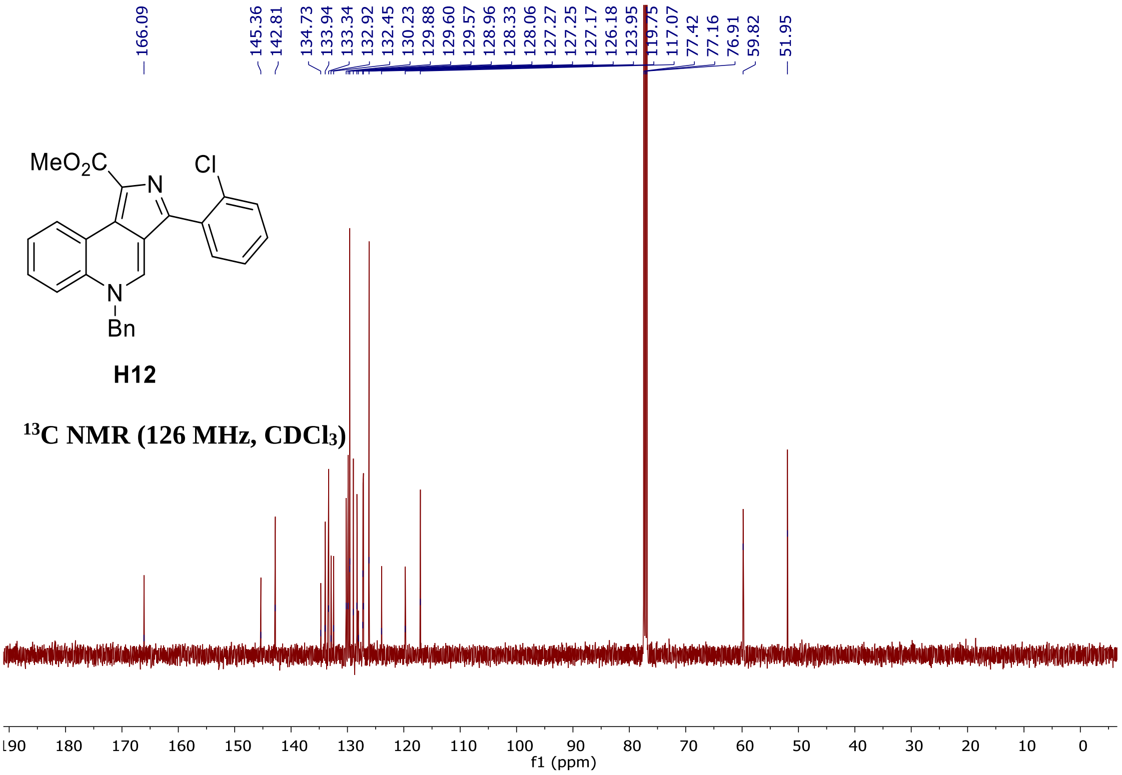

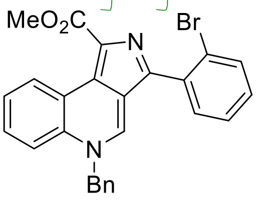

H13

^{1}H NMR (500 MHz, DMSO-*d6*)

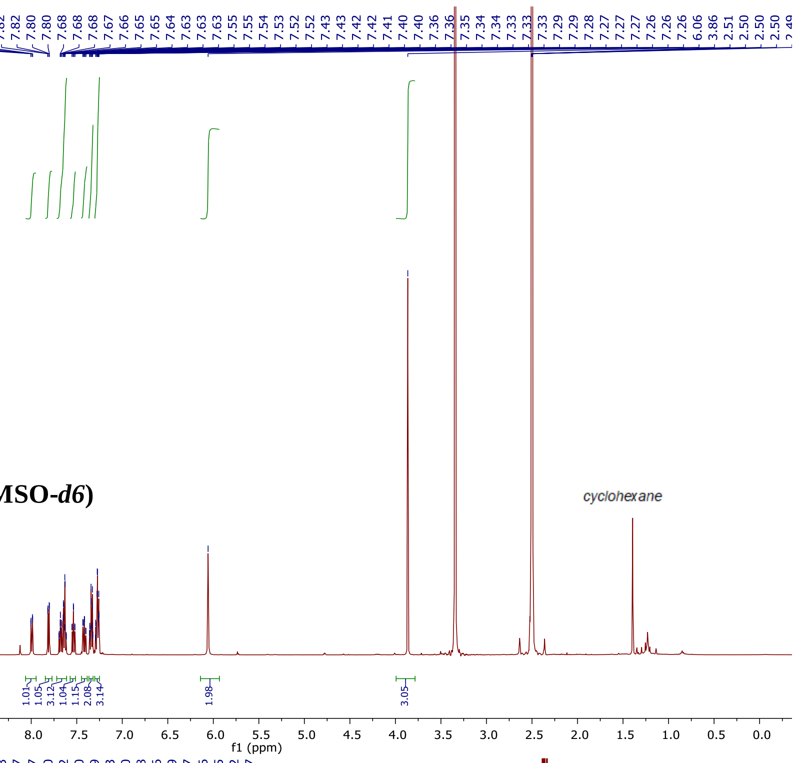

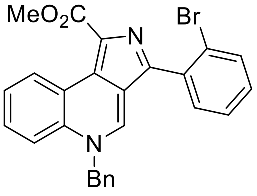

H13

^{13}C NMR (126 MHz, DMSO-*d6*)

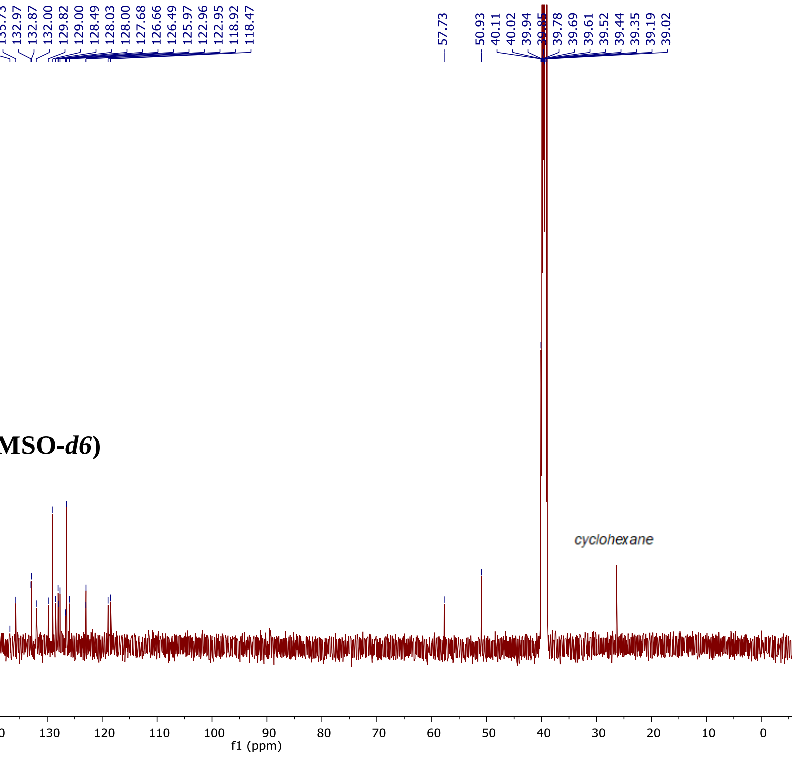

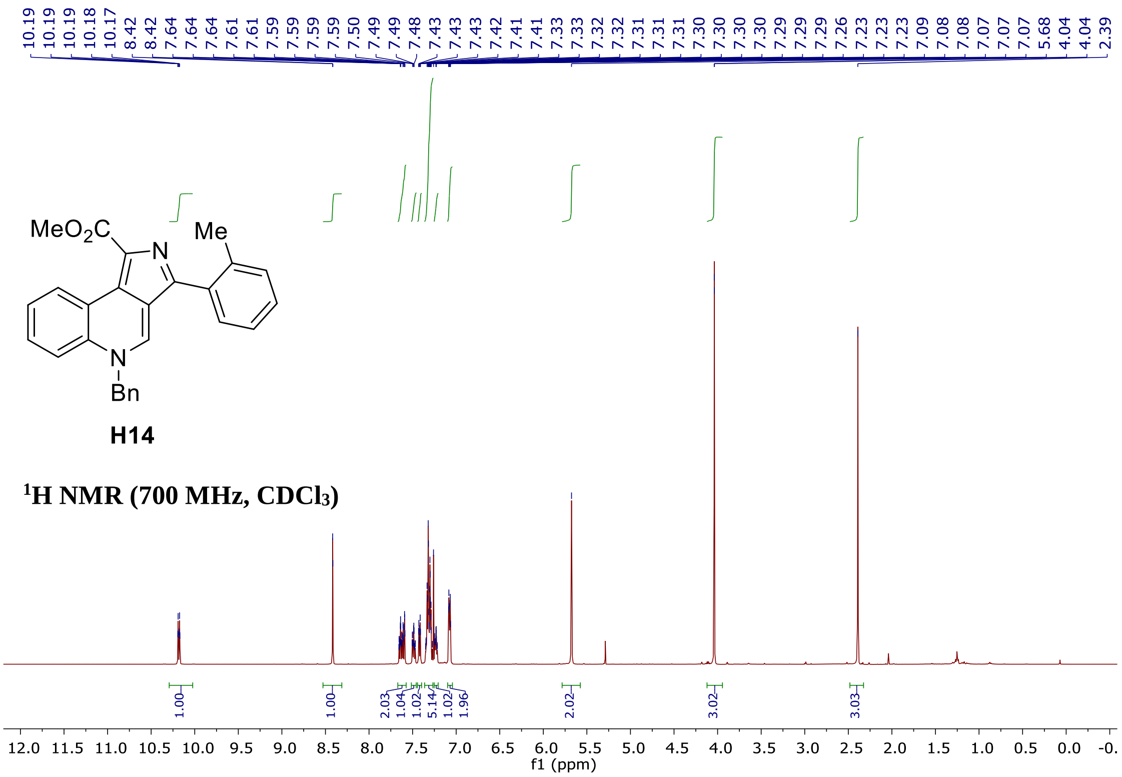
10.19 10.19 10.19 10.18 10.17 8.42 8.42 7.64 7.64 7.64 7.61 7.61 7.59 7.59 7.59 7.59 7.50 7.49 7.49 7.48 7.43 7.43 7.43 7.42 7.41 7.41 7.33 7.33 7.32 7.32 7.31 7.31 7.30 7.30 7.30 7.30 7.29 7.29 7.29 7.26 7.23 7.23 7.23 7.09 7.08 7.08 7.07 7.07 7.07 5.68 4.04 4.04 2.39
MeO2C
N
Me
N
Bn
H14
1H NMR (700 MHz, CDCl3)
1.00 1.00 2.03 1.04 1.02 5.14 1.02 1.96 2.02 3.02 3.03
12.0 11.5 11.0 10.5 10.0 9.5 9.0 8.5 8.0 7.5 7.0 6.5 6.0 5.5 5.0 4.5 4.0 3.5 3.0 2.5 2.0 1.5 1.0 0.5 0.0 -0.
f1 (ppm)
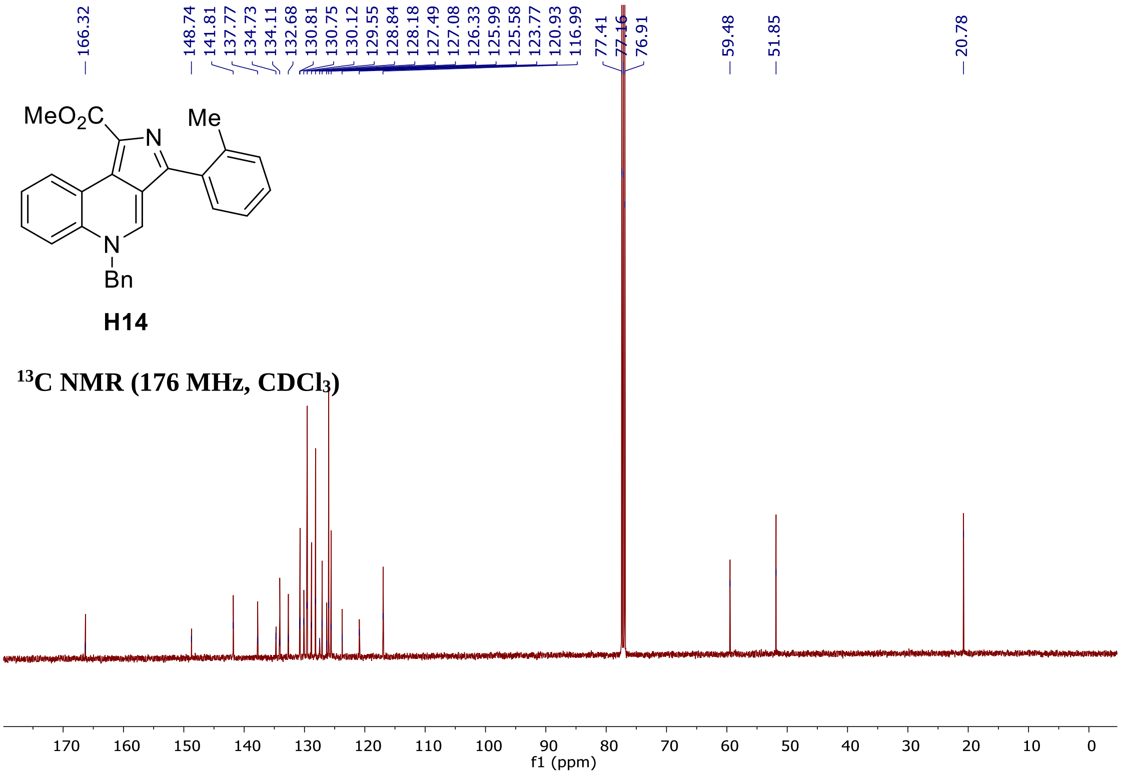
166.32 148.74 141.81 137.77 134.73 134.11 132.68 130.81 130.75 130.12 129.55 128.84 128.18 127.49 127.08 126.33 125.99 125.58 123.77 120.93 116.99 77.41 77.16 76.91 59.48 51.85 20.78
MeO2C
N
Me
N
Bn
H14
13C NMR (176 MHz, CDCl3)
170 160 150 140 130 120 110 100 90 80 70 60 50 40 30 20 10 0
f1 (ppm)

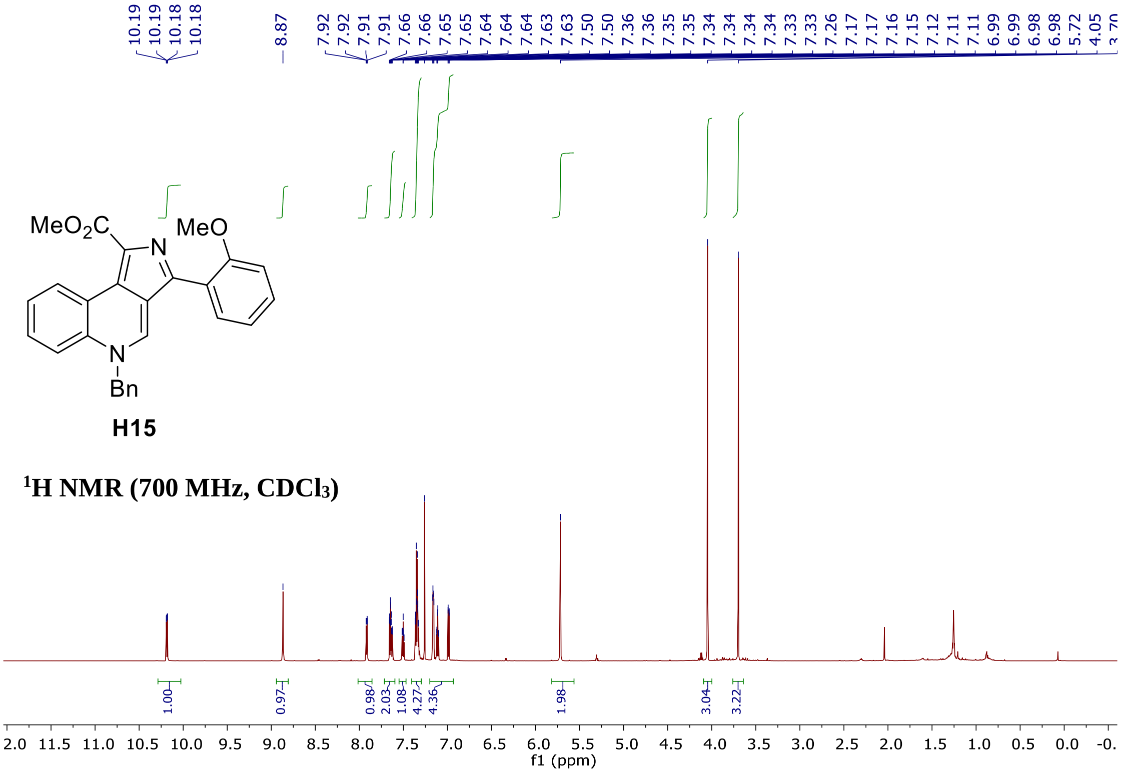
10.19 10.19 10.18 10.18
8.87
7.92 7.92 7.91 7.91 7.66 7.66 7.65 7.65 7.64 7.64 7.64 7.63 7.63 7.50 7.50 7.36 7.36 7.35 7.35 7.34 7.34 7.34 7.34 7.33 7.33 7.26 7.17 7.17 7.16 7.15 7.12 7.11 7.11 6.99 6.99 6.98 6.98 5.72 4.05
MeO2C
MeO
N
N
Bn
H15
1H NMR (700 MHz, CDCl3)
1.00
0.97
0.98
2.03
1.08
4.27
4.36
1.98
3.04
3.22
f1 (ppm)

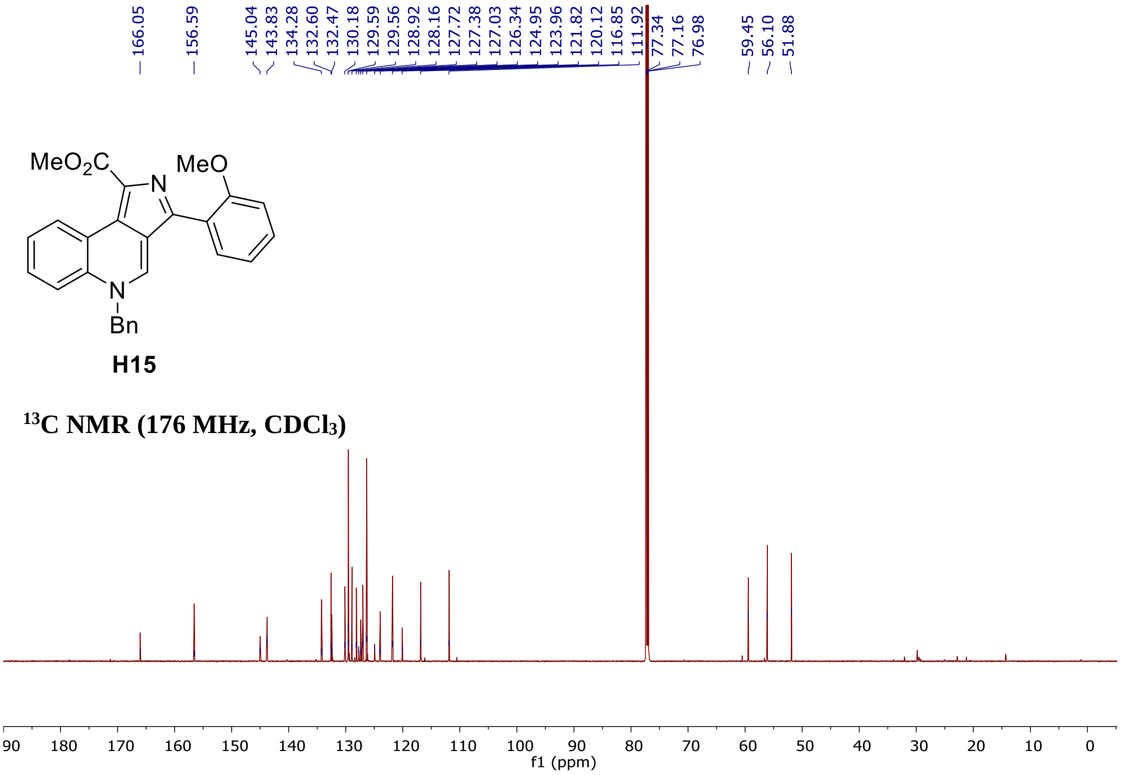
166.05
156.59
145.04 143.83 134.28 132.60 132.47 130.18 129.59 129.56 128.92 128.16 127.72 127.38 127.03 126.34 124.95 123.96 121.82 120.12 116.85 111.92 77.34 77.16 76.98
59.45 56.10 51.88
MeO2C
MeO
N
N
Bn
H15
13C NMR (176 MHz, CDCl3)
f1 (ppm)

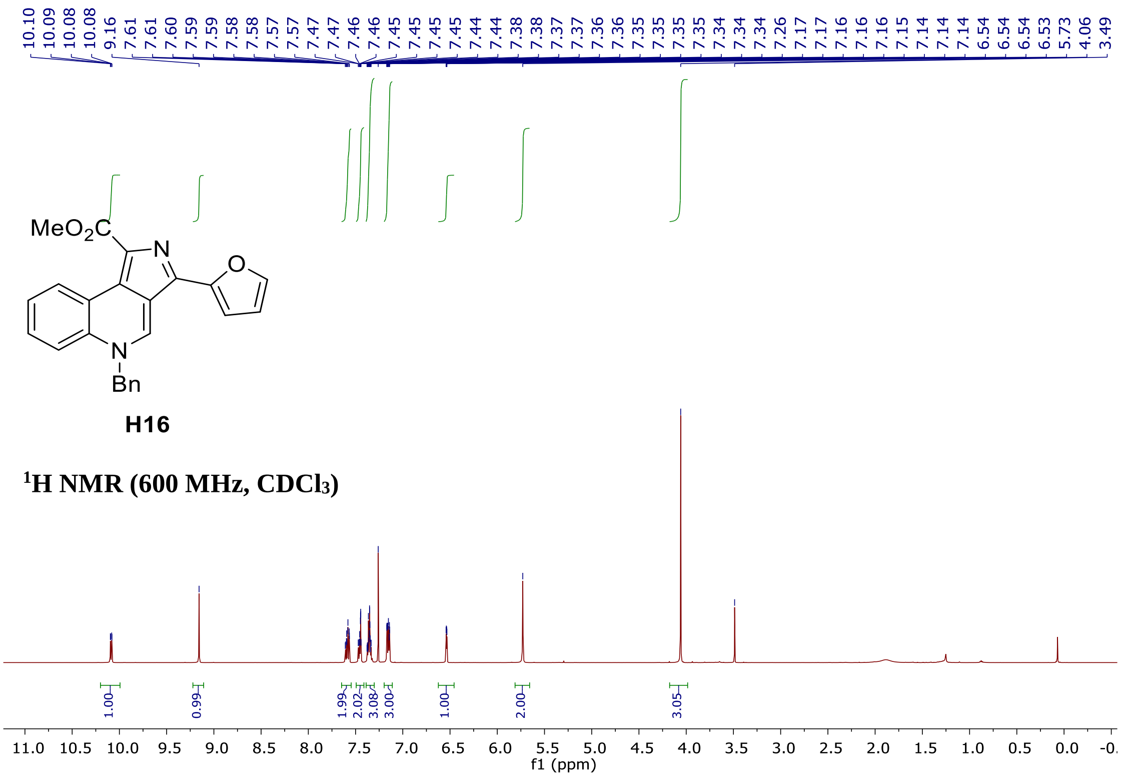

H16

^{1}H NMR (600 MHz, CDCl$_3$)

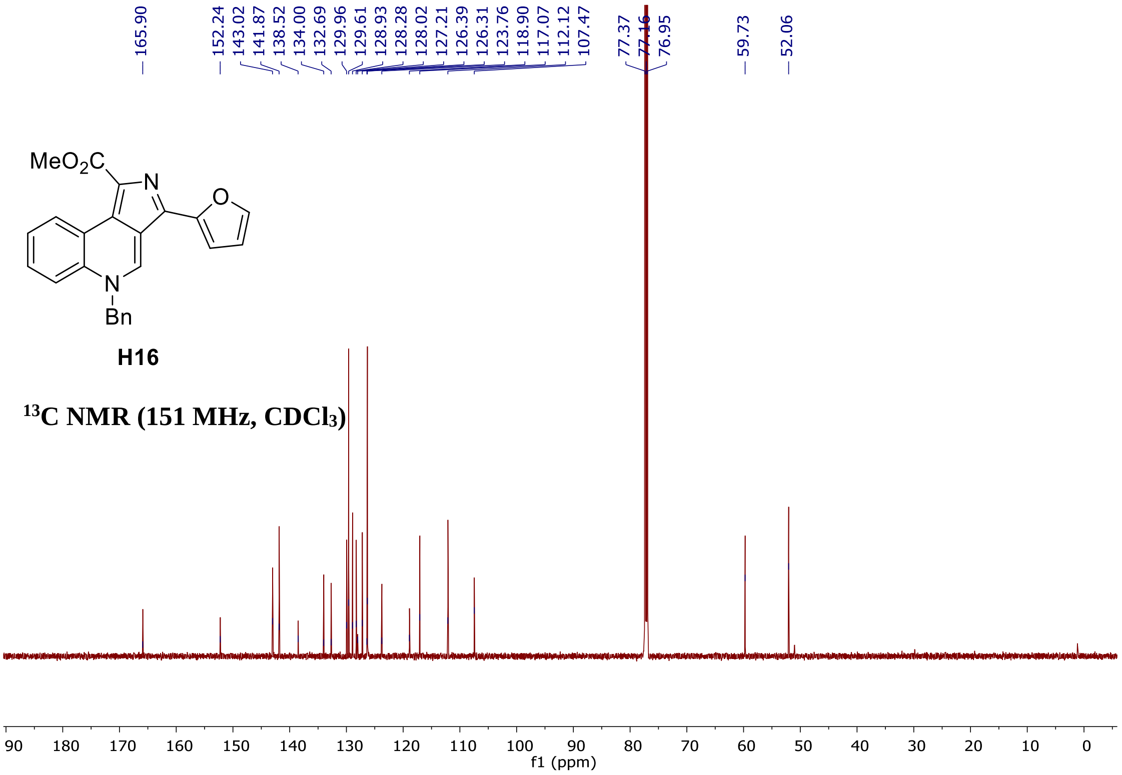

H16

^{13}C NMR (151 MHz, CDCl$_3$)

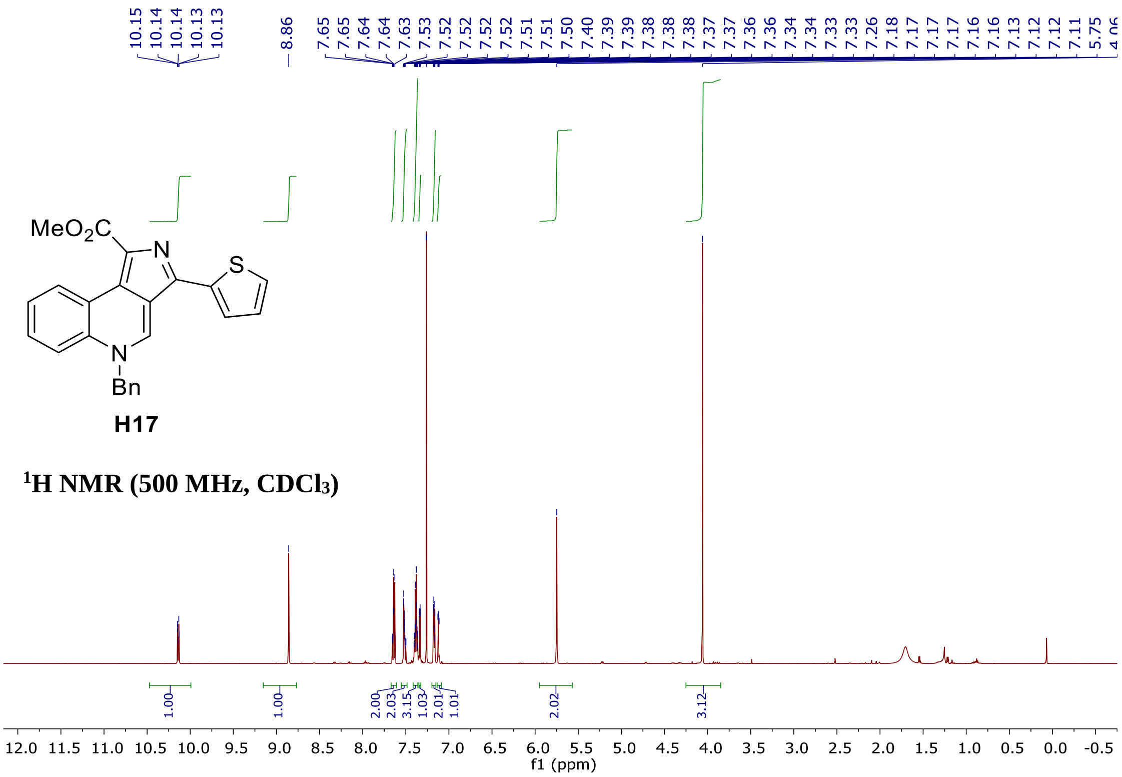
10.15
10.14
10.14
10.13
10.13
8.86
7.65
7.65
7.64
7.64
7.63
7.53
7.52
7.52
7.52
7.52
7.51
7.51
7.50
7.40
7.39
7.39
7.38
7.38
7.38
7.37
7.37
7.36
7.36
7.34
7.34
7.33
7.33
7.26
7.18
7.17
7.17
7.17
7.16
7.16
7.13
7.12
7.12
7.11
5.75
MeO2C
N
S
N
Bn
H17
1H NMR (500 MHz, CDCl3)
1.00
1.00
2.00
2.03
3.15
1.03
2.01
1.01
2.02
3.12
12.0 11.5 11.0 10.5 10.0 9.5 9.0 8.5 8.0 7.5 7.0 6.5 6.0 5.5 5.0 4.5 4.0 3.5 3.0 2.5 2.0 1.5 1.0 0.5 0.0 -0.5
f1 (ppm)

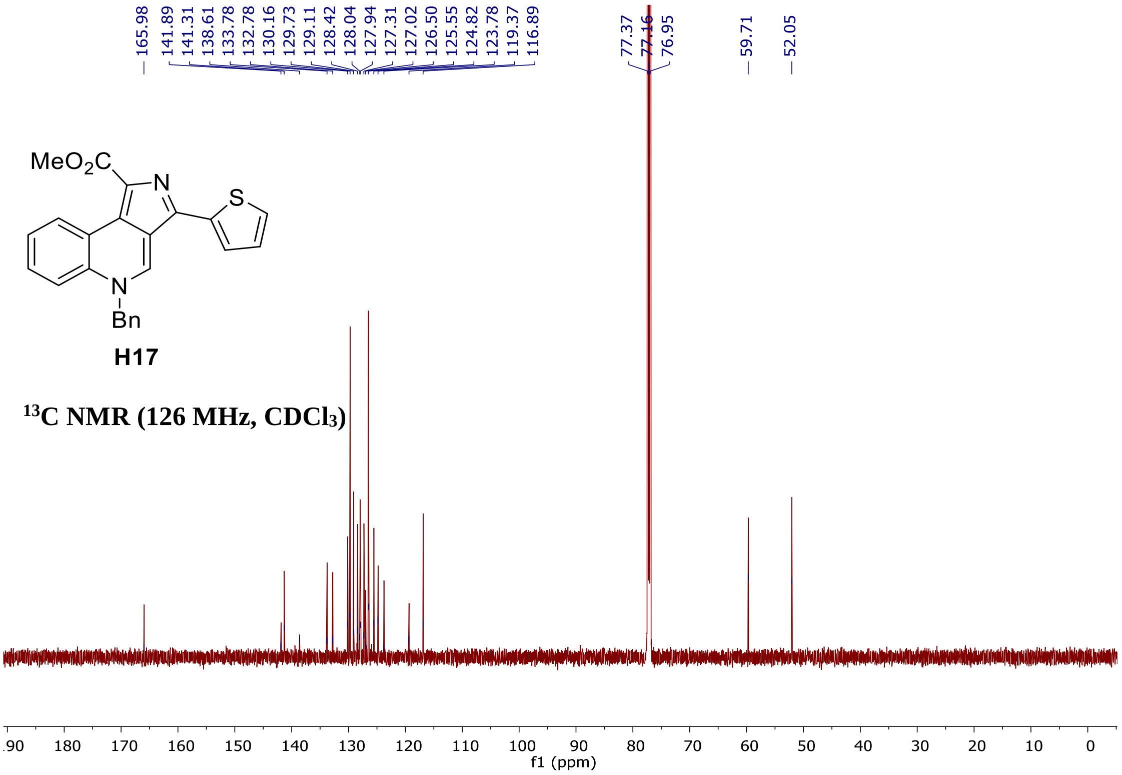
165.98
141.89
141.31
138.61
133.78
132.78
130.16
129.73
129.11
128.42
128.04
127.94
127.31
127.02
126.50
125.55
124.82
123.78
119.37
116.89
77.37
77.16
76.95
59.71
52.05
MeO2C
N
S
N
Bn
H17
13C NMR (126 MHz, CDCl3)
180 170 160 150 140 130 120 110 100 90 80 70 60 50 40 30 20 10 0
f1 (ppm)

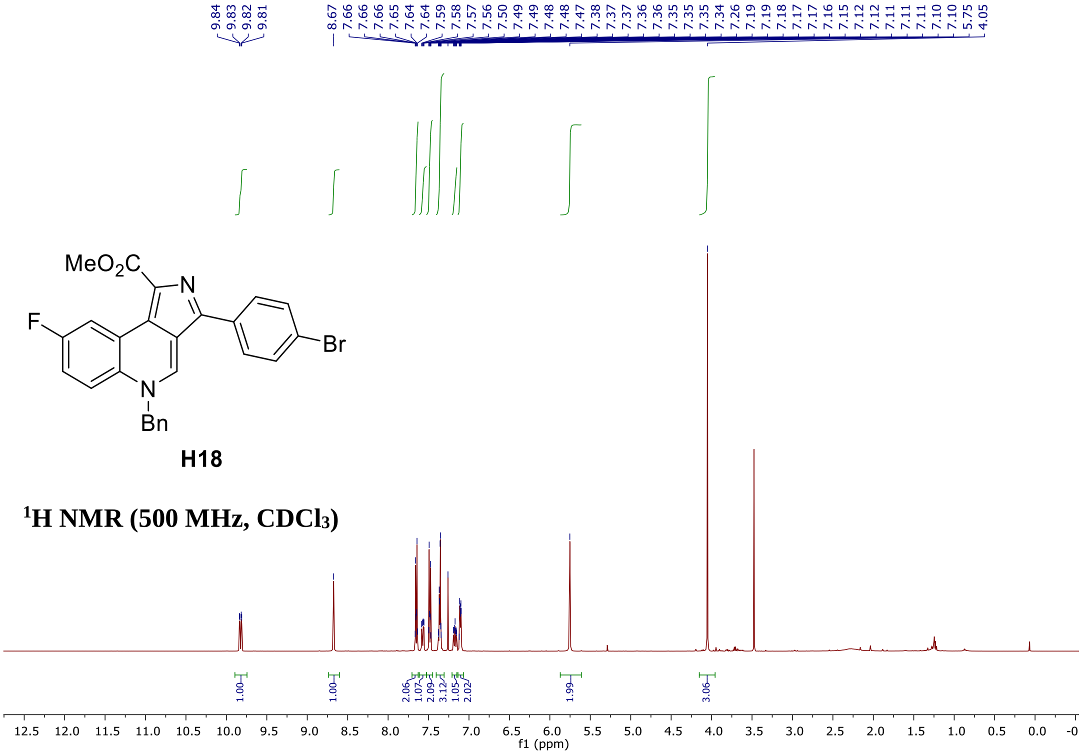
9.84 9.83 9.82 9.81 8.67 7.66 7.66 7.65 7.64 7.64 7.59 7.58 7.57 7.56 7.50 7.49 7.49 7.48 7.48 7.47 7.38 7.37 7.37 7.36 7.36 7.35 7.35 7.35 7.34 7.26 7.19 7.19 7.18 7.17 7.17 7.16 7.15 7.12 7.12 7.11 7.11 7.11 7.10 5.75 4.05
MeO2C
N
F
Br
N
Bn
H18
1H NMR (500 MHz, CDCl3)
1.00 1.00 2.06 1.07 2.09 3.12 1.05 2.02 1.99 3.06
12.5 12.0 11.5 11.0 10.5 10.0 9.5 9.0 8.5 8.0 7.5 7.0 6.5 6.0 5.5 5.0 4.5 4.0 3.5 3.0 2.5 2.0 1.5 1.0 0.5 0.0 -0
f1 (ppm)

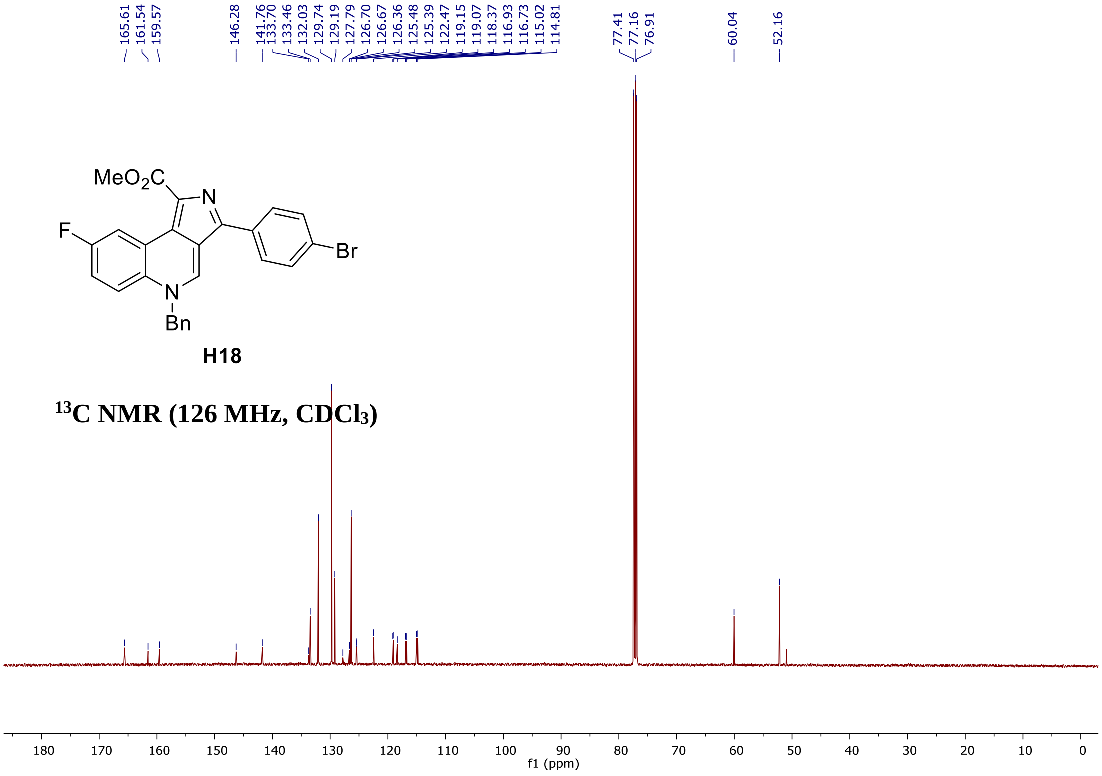
165.61 161.54 159.57 146.28 141.76 133.70 133.46 132.03 129.74 129.19 127.79 126.70 126.67 126.36 125.48 125.39 122.47 119.15 119.07 118.37 116.93 116.73 115.02 114.81 77.41 77.16 76.91 60.04 52.16
MeO2C
N
F
Br
N
Bn
H18
13C NMR (126 MHz, CDCl3)
180 170 160 150 140 130 120 110 100 90 80 70 60 50 40 30 20 10 0
f1 (ppm)

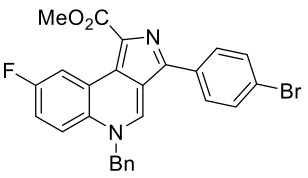

H18

^{19}F NMR (470 MHz, CDCl$_3$)

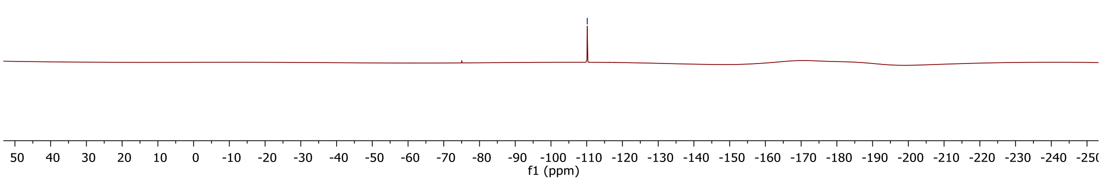

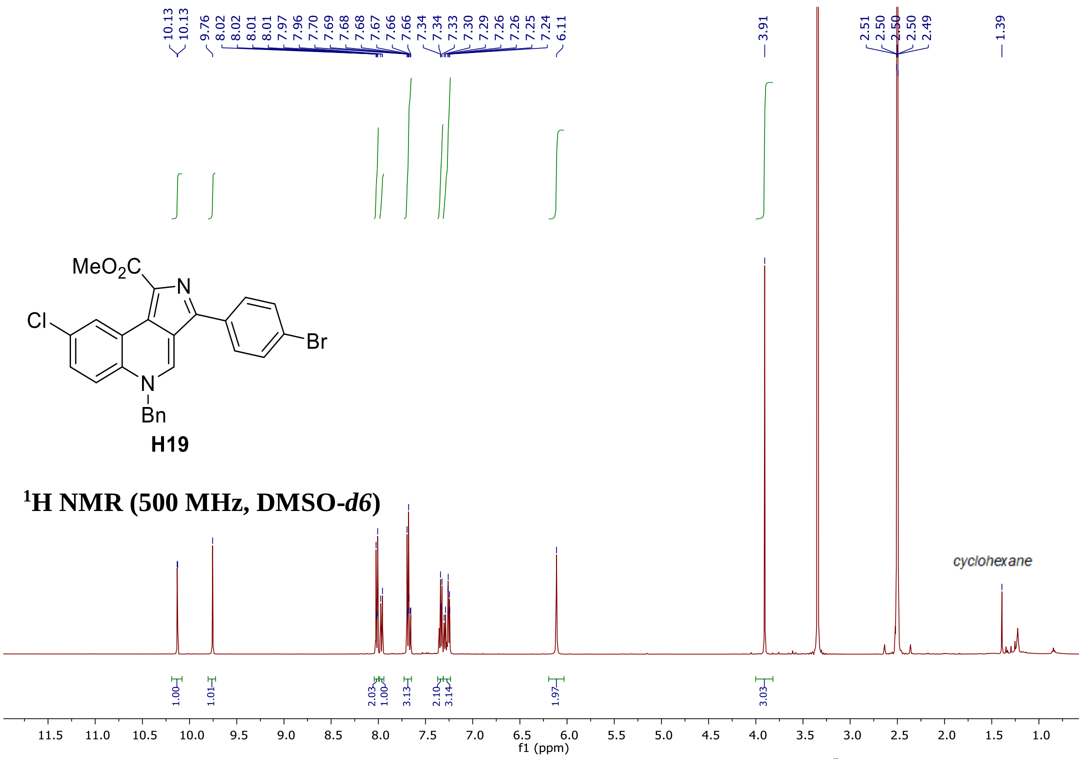
10.13
10.13
9.76
8.02
8.02
8.01
8.01
7.97
7.96
7.70
7.69
7.68
7.68
7.67
7.66
7.66
7.34
7.34
7.33
7.30
7.29
7.26
7.26
7.25
7.24
6.11
3.91
2.51
2.50
2.50
2.50
2.49
1.39
MeO$_2$C
N
Cl
Br
N
Bn
H19
^{1}H NMR (500 MHz, DMSO-*d6*)
cyclohexane
1.00
1.01
2.03
1.00
3.13
2.10
3.14
1.97
3.03
f1 (ppm)

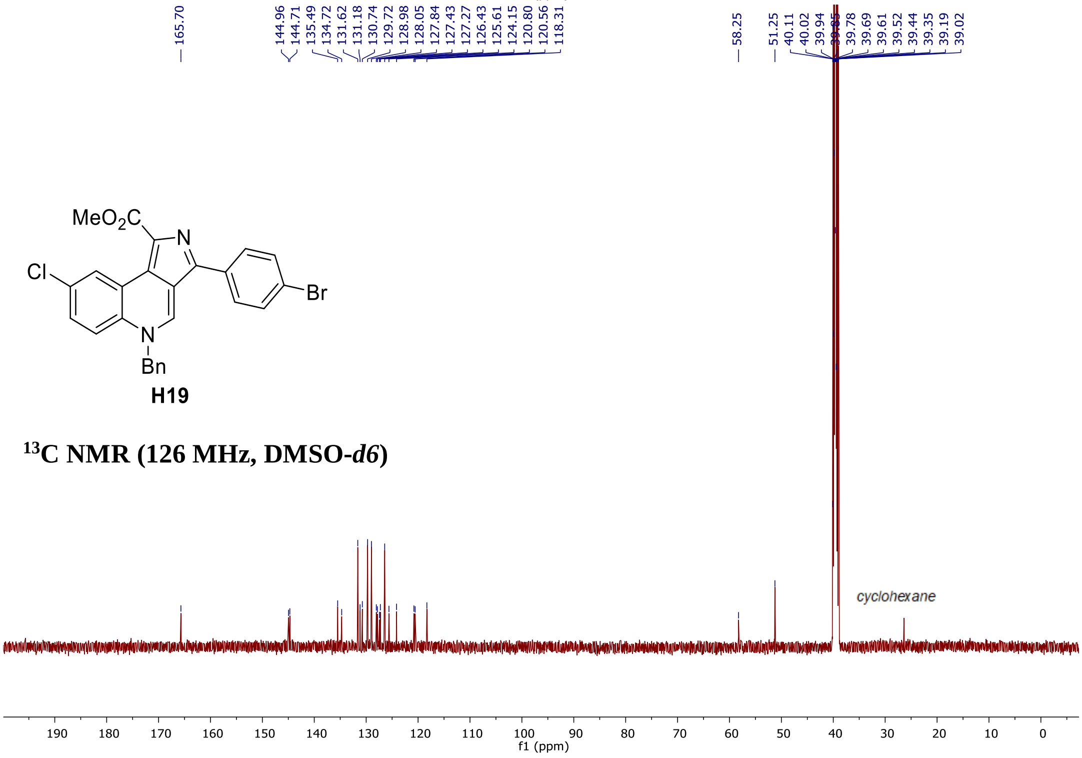
165.70
144.96
144.71
135.49
134.72
131.62
131.18
130.74
129.72
128.98
128.05
127.84
127.43
127.27
126.43
125.61
124.15
120.80
120.56
118.31
58.25
51.25
40.11
40.02
39.94
39.78
39.69
39.61
39.52
39.44
39.35
39.19
39.02
MeO$_2$C
N
Cl
Br
N
Bn
H19
^{13}C NMR (126 MHz, DMSO-*d6*)
cyclohexane
f1 (ppm)

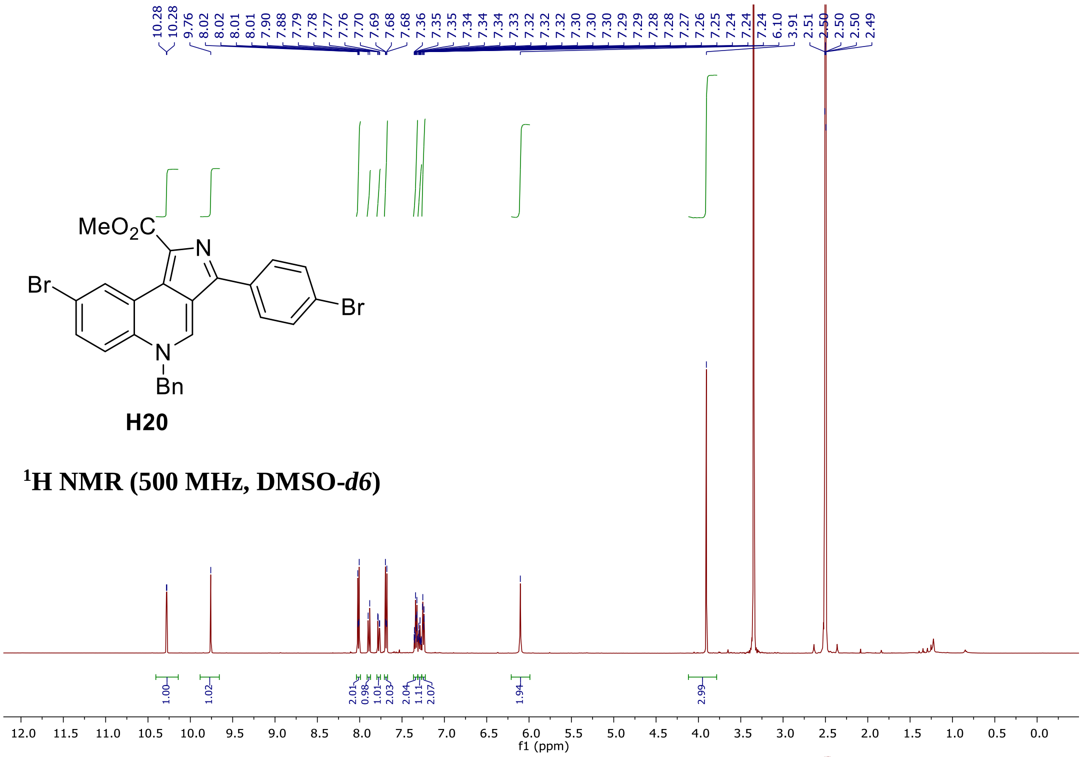

H20

^{1}H NMR (500 MHz, DMSO-*d6*)

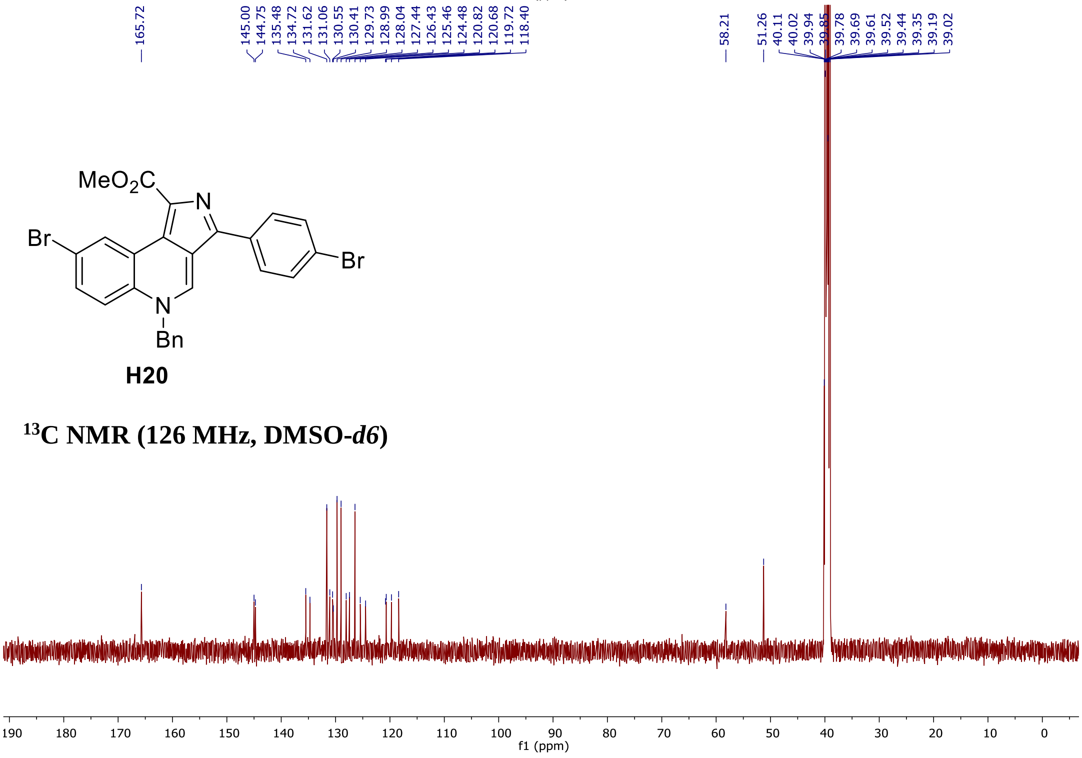

H20

^{13}C NMR (126 MHz, DMSO-*d6*)

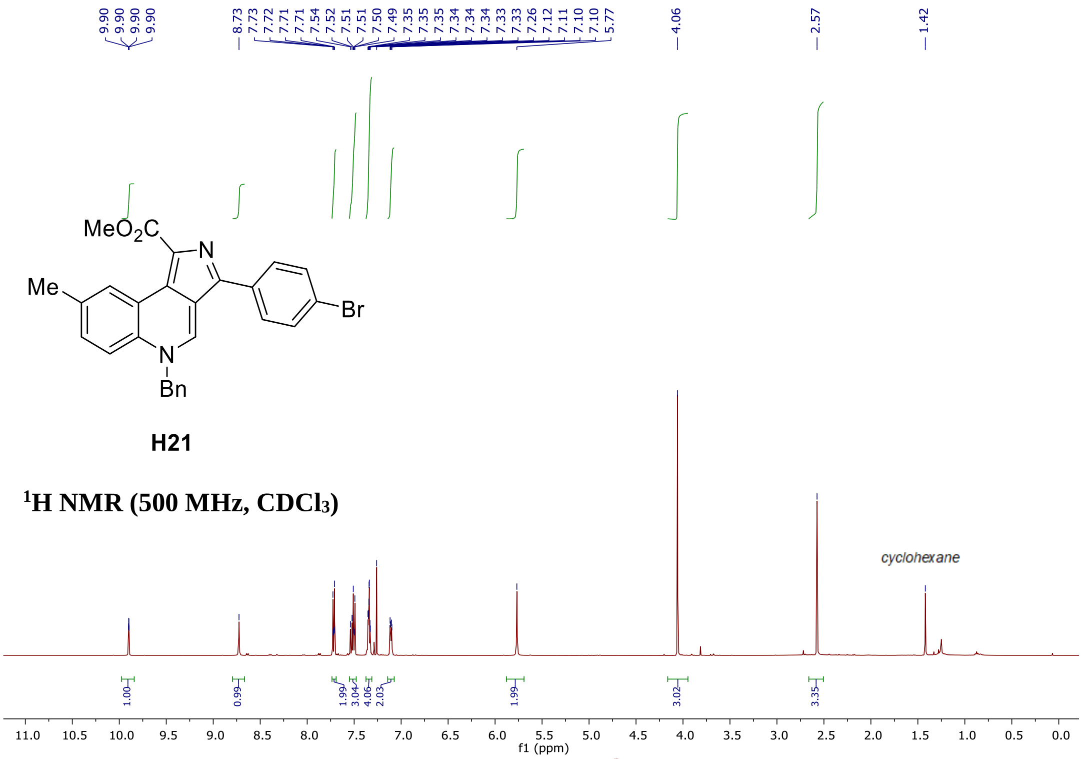

H21

^{1}H NMR (500 MHz, $CDCl_3$)

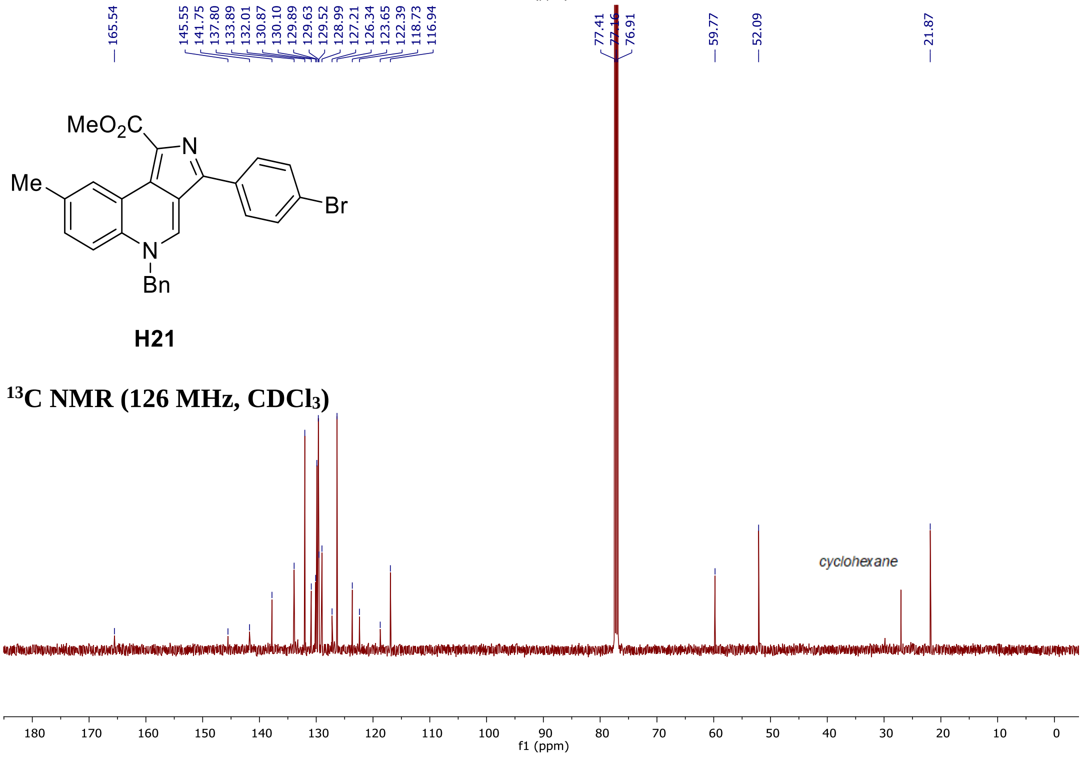

H21

^{13}C NMR (126 MHz, $CDCl_3$)

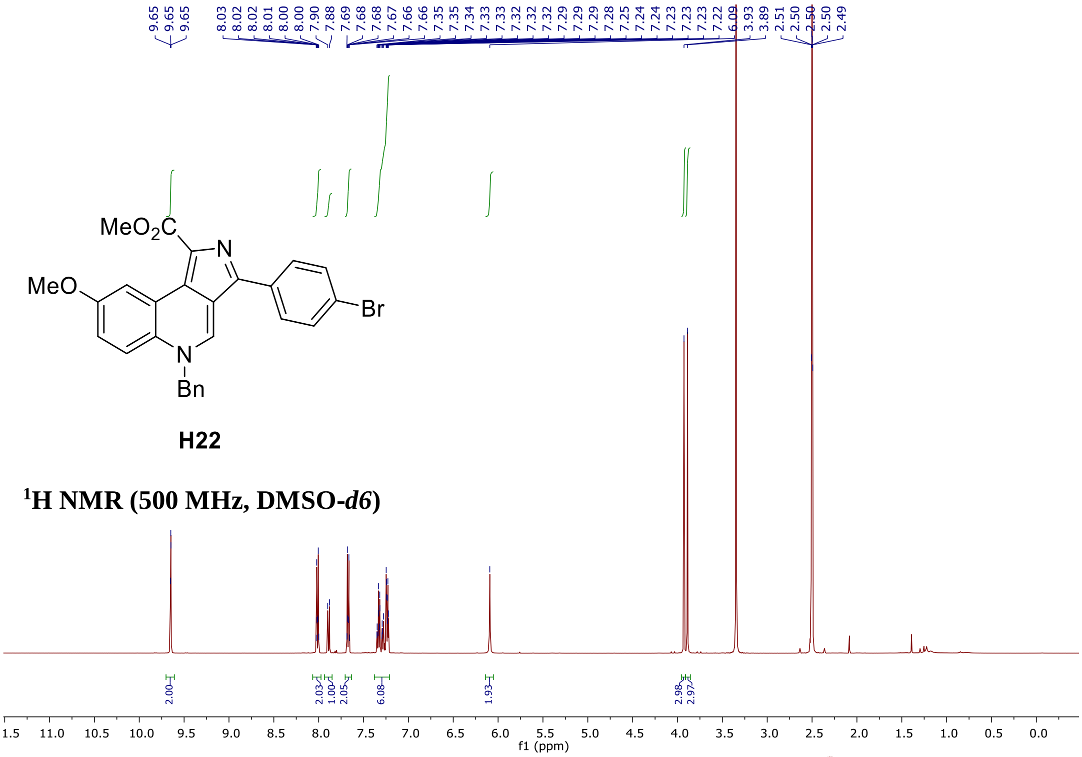

9.65 9.65 9.65 8.03 8.02 8.02 8.01 8.00 8.00 7.90 7.88 7.69 7.68 7.68 7.67 7.66 7.35 7.35 7.34 7.33 7.33 7.32 7.32 7.32 7.29 7.29 7.29 7.28 7.25 7.24 7.23 7.23 7.23 7.22 6.09 3.93 3.89 2.51 2.50 2.50 2.50 2.49
MeO$_2$C
N
MeO
Br
N
Bn
H22
^{1}H NMR (500 MHz, DMSO-*d6*)
2.00 2.03 1.00 2.05 6.08 1.93 2.98 2.97
f1 (ppm)

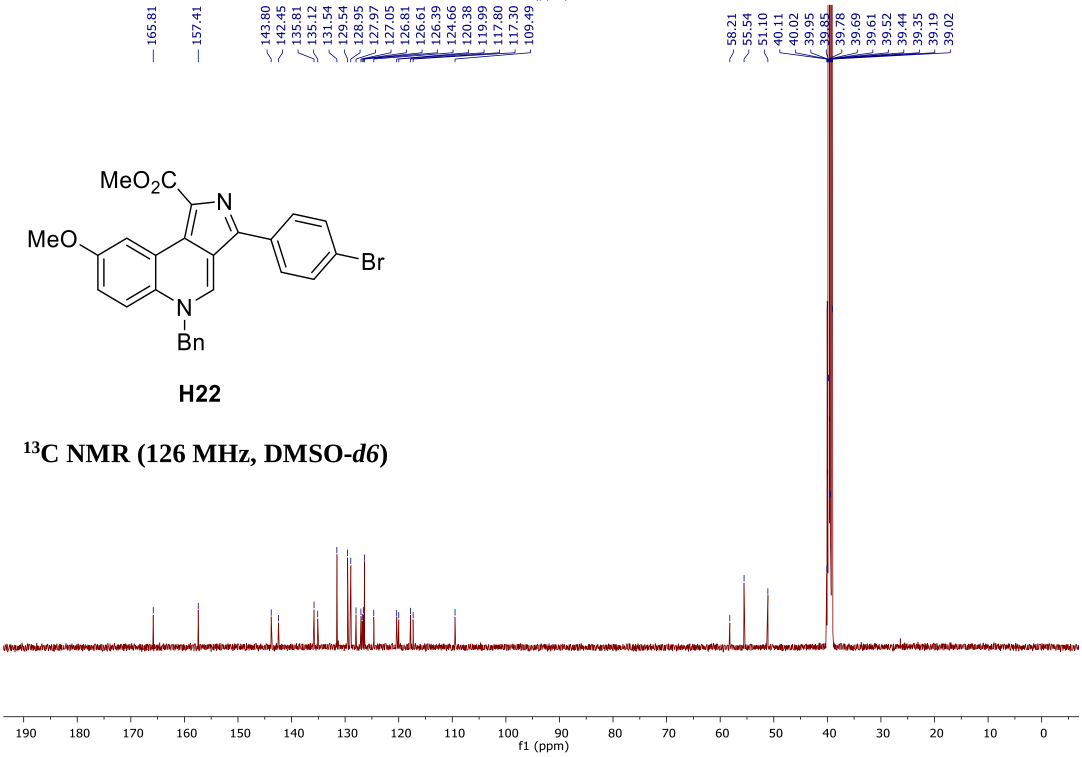

165.81 157.41 143.80 142.45 135.81 135.12 131.54 129.54 128.95 127.97 127.05 126.81 126.61 126.39 124.66 120.38 119.99 117.80 117.30 109.49 58.21 55.54 51.10 40.11 40.02 39.95 39.85 39.78 39.69 39.61 39.52 39.44 39.35 39.19 39.02
MeO$_2$C
N
MeO
Br
N
Bn
H22
^{13}C NMR (126 MHz, DMSO-*d6*)
f1 (ppm)

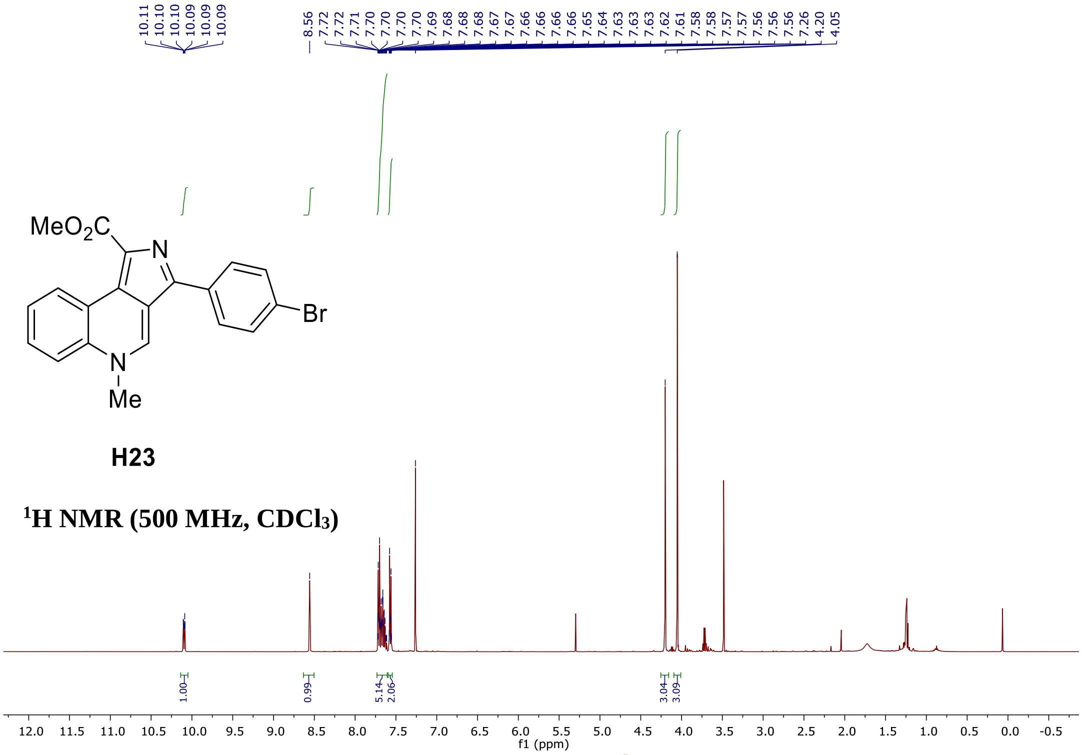

H23

^{1}H NMR (500 MHz, CDCl3)

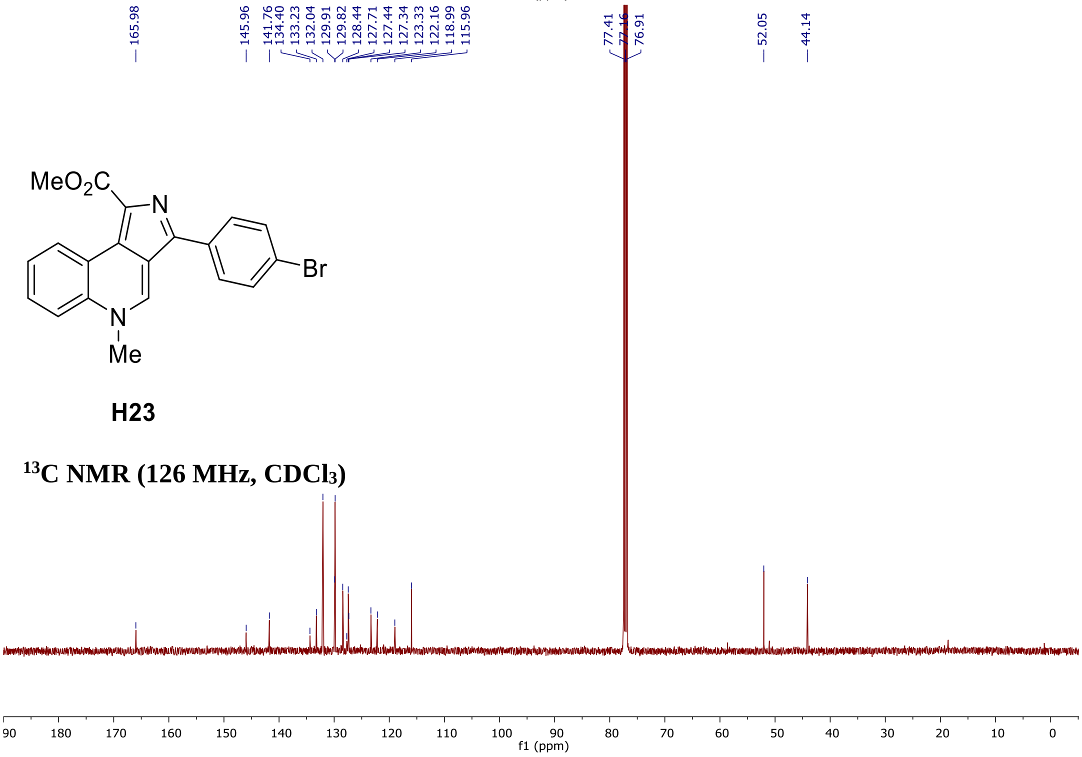

H23

^{13}C NMR (126 MHz, CDCl3)

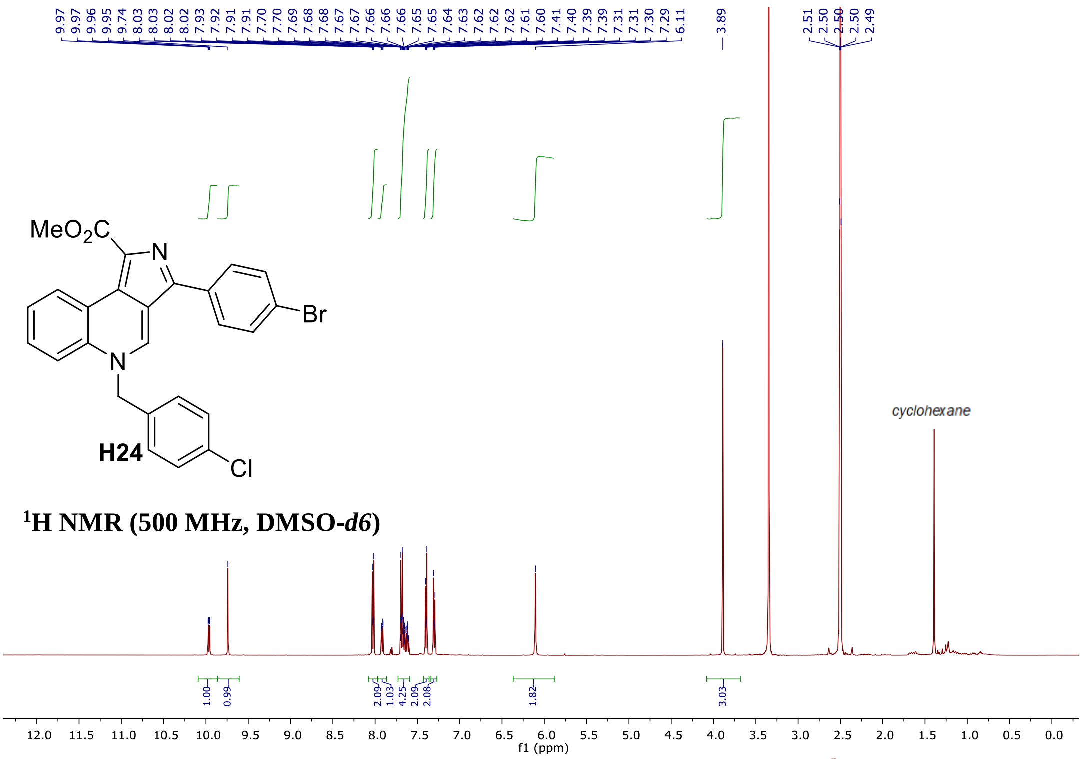
9.97 9.97 9.96 9.95 9.74 8.03 8.03 8.02 8.02 7.93 7.92 7.91 7.70 7.70 7.69 7.68 7.68 7.67 7.67 7.66 7.66 7.66 7.65 7.65 7.64 7.63 7.62 7.62 7.62 7.61 7.60 7.41 7.40 7.39 7.39 7.31 7.31 7.30 7.29 6.11
3.89
2.51 2.50 2.50 2.50 2.49
MeO2C
N
Br
N
H24
Cl
cyclohexane
1H NMR (500 MHz, DMSO-d6)
1.00 0.99 2.09 1.03 4.25 2.09 2.08 1.82 3.03
f1 (ppm)

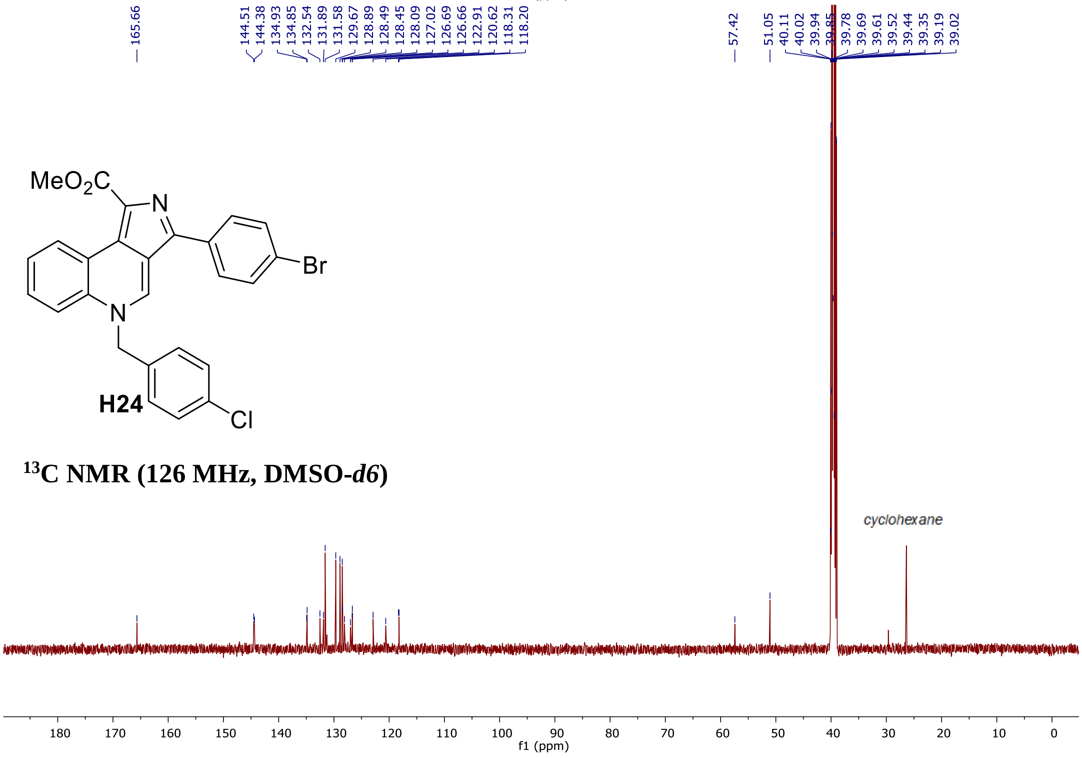
165.66
144.51 144.38 134.93 134.85 132.54 131.89 131.58 129.67 128.89 128.49 128.45 128.09 127.02 126.69 126.66 122.91 120.62 118.31 118.20
57.42
51.05 40.11 40.02 39.94 39.85 39.78 39.69 39.61 39.52 39.44 39.35 39.19 39.02
MeO2C
N
Br
N
H24
Cl
13C NMR (126 MHz, DMSO-d6)
cyclohexane
f1 (ppm)

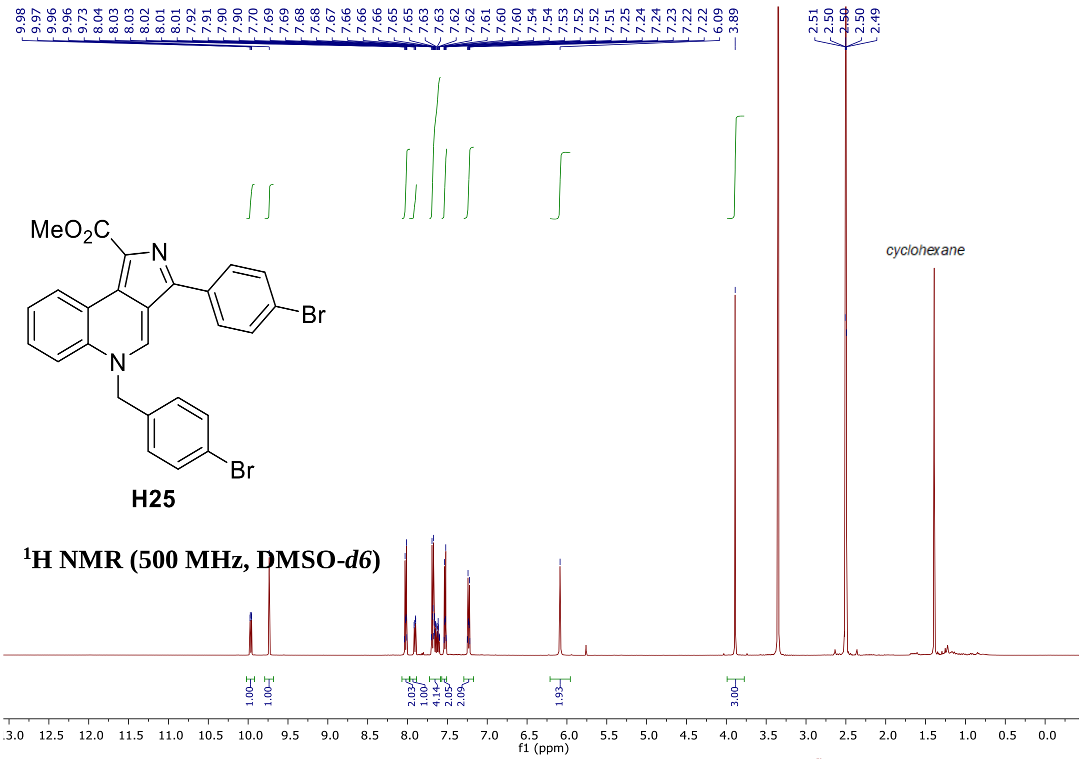
9.98 9.97 9.96 9.96 9.73 8.04 8.03 8.03 8.02 8.01 8.01 7.92 7.91 7.90 7.90 7.70 7.69 7.69 7.68 7.68 7.67 7.66 7.66 7.65 7.65 7.63 7.63 7.62 7.62 7.61 7.60 7.54 7.53 7.52 7.52 7.51 7.25 7.24 7.24 7.23 7.22 7.22 6.09 3.89
2.51 2.50 2.50 2.50 2.49
MeO2C
N
Br
N
Br
H25
1H NMR (500 MHz, DMSO-d6)
cyclohexane
1.00 1.00 2.03 1.00 4.14 2.05 2.09 1.93 3.00
f1 (ppm)

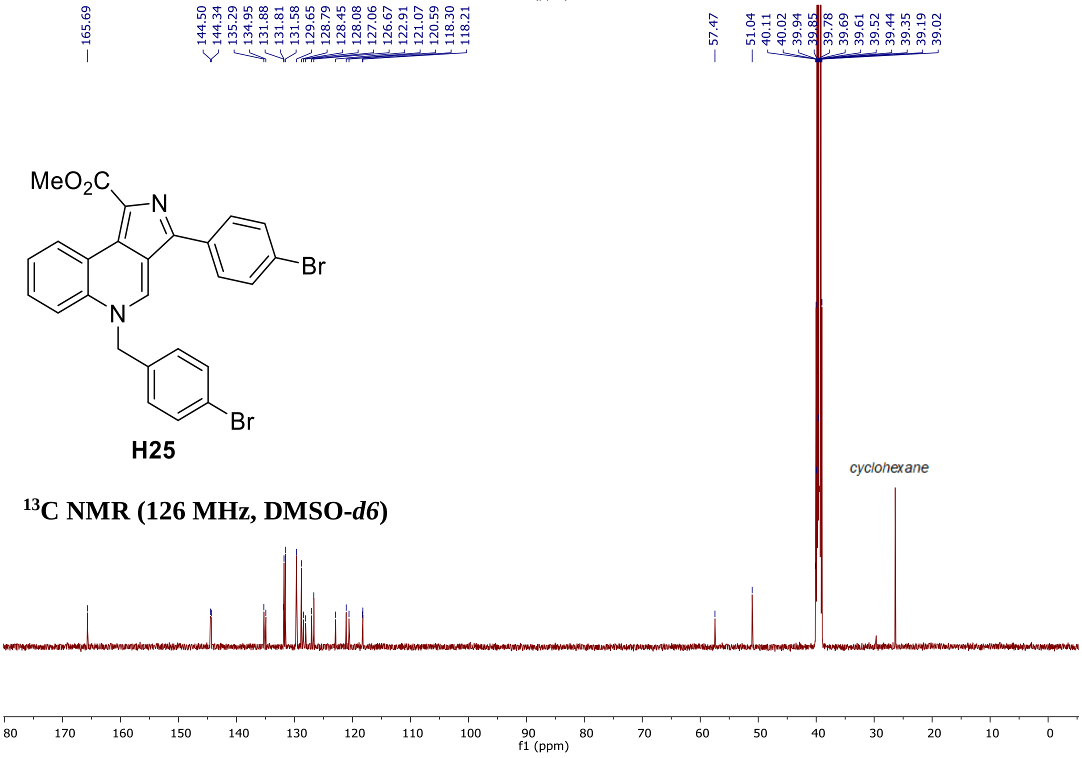
165.69
144.50 144.34 135.29 134.95 131.88 131.81 131.58 129.65 128.79 128.45 128.08 127.06 126.67 122.91 121.07 120.59 118.30 118.21
57.47
51.04 40.11 40.02 39.94 39.85 39.78 39.69 39.61 39.52 39.44 39.35 39.19 39.02
MeO2C
N
Br
N
Br
H25
13C NMR (126 MHz, DMSO-d6)
cyclohexane
f1 (ppm)

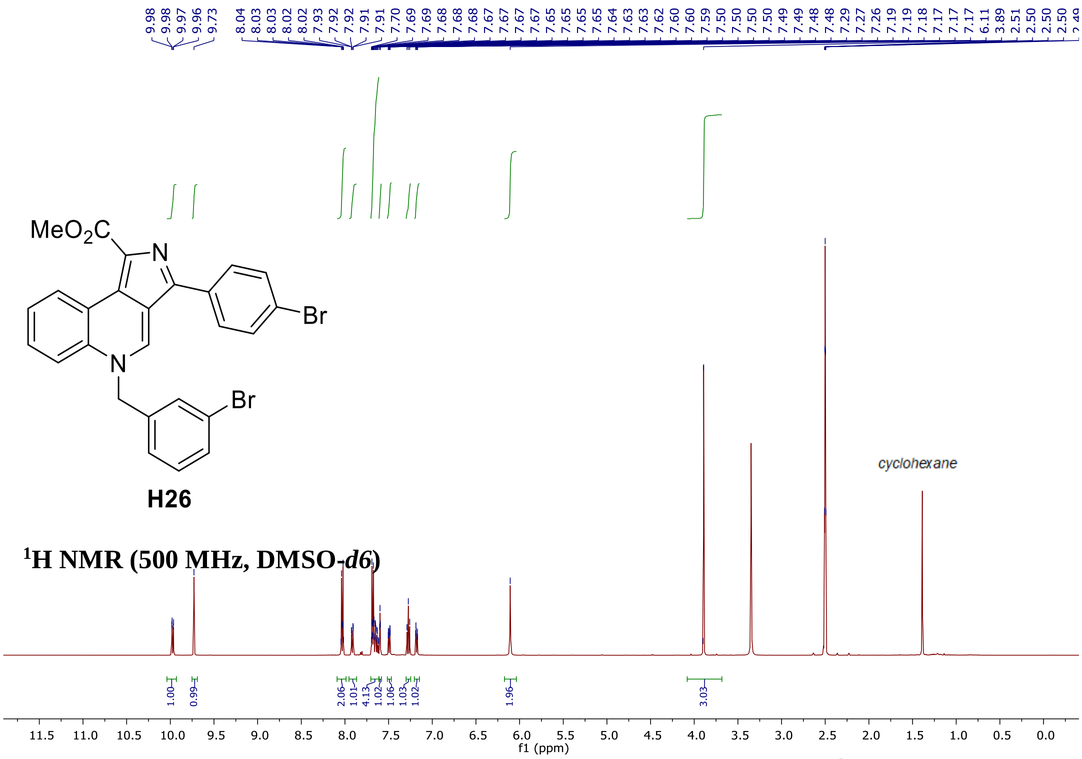

^{1}H NMR (500 MHz, DMSO-*d6*)

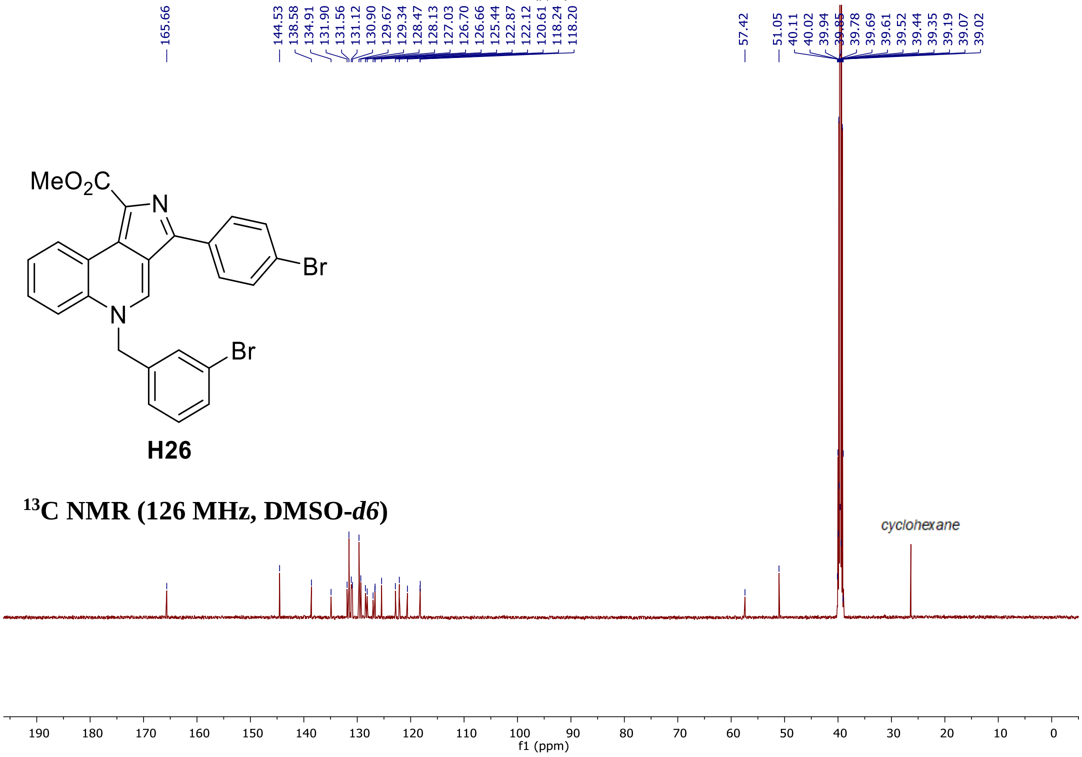

^{13}C NMR (126 MHz, DMSO-*d6*)

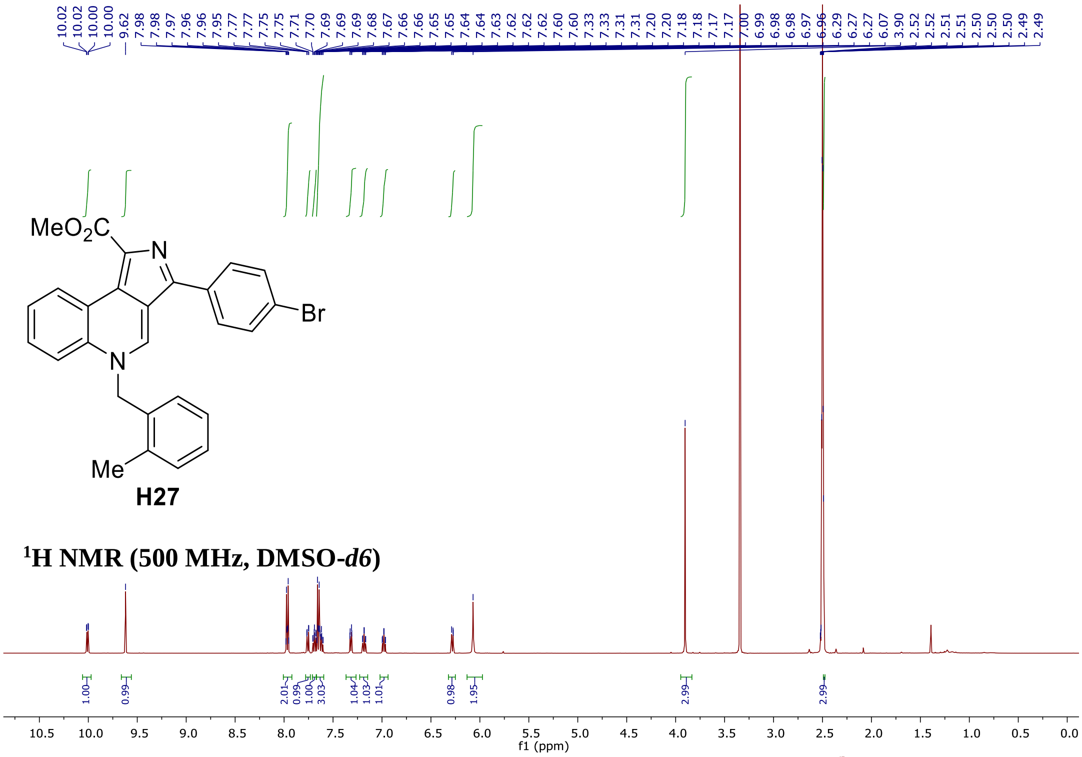
10.02 10.02 10.00 10.00 9.62 7.98 7.98 7.97 7.96 7.96 7.95 7.77 7.77 7.75 7.75 7.71 7.70 7.69 7.69 7.69 7.68 7.67 7.66 7.66 7.65 7.65 7.64 7.64 7.63 7.62 7.62 7.62 7.60 7.60 7.33 7.33 7.31 7.31 7.20 7.20 7.18 7.18 7.17 7.17 7.00 6.99 6.98 6.98 6.97 6.96 6.29 6.27 6.27 6.07 3.90 2.52 2.52 2.51 2.51 2.50 2.50 2.49 2.49
MeO_2C
N
Br
N
Me
H27
^{1}H NMR (500 MHz, DMSO-*d6*)
1.00 0.99 2.01 0.99 1.00 3.03 1.04 1.03 1.01 0.98 1.95 2.99 2.99
10.5 10.0 9.5 9.0 8.5 8.0 7.5 7.0 6.5 6.0 5.5 5.0 4.5 4.0 3.5 3.0 2.5 2.0 1.5 1.0 0.5 0.0
f1 (ppm)

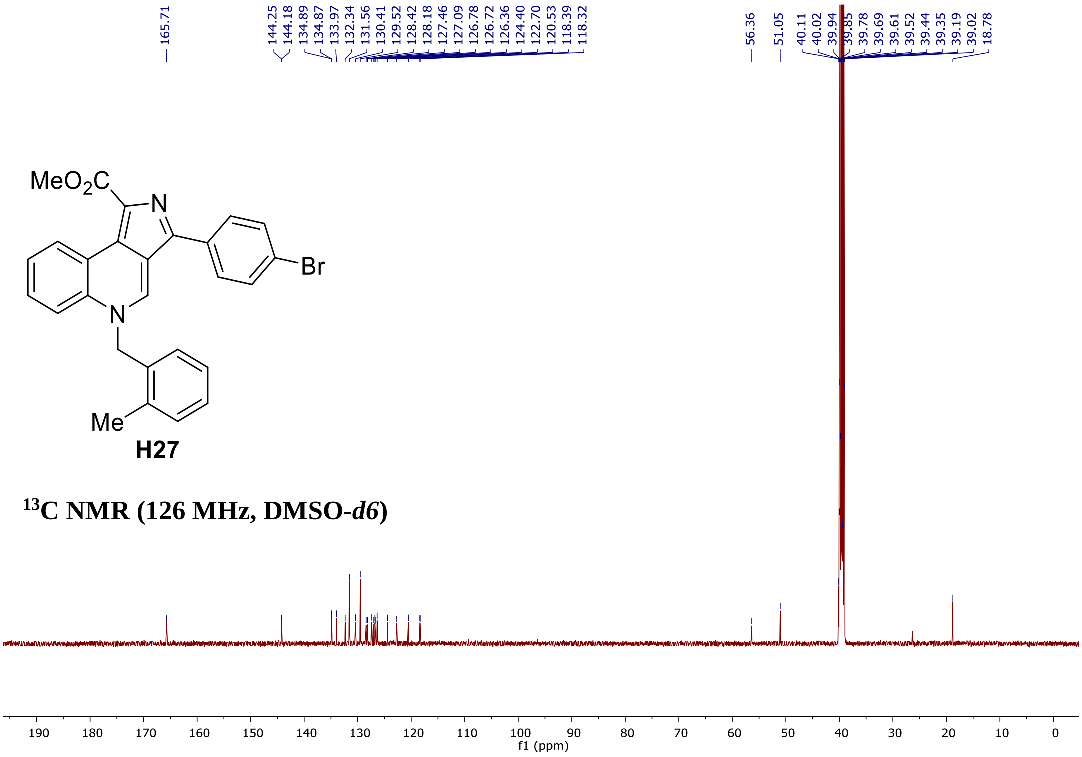
165.71 144.25 144.18 134.89 134.87 133.97 132.34 131.56 130.41 129.52 128.42 128.18 127.46 127.09 126.78 126.72 126.36 124.40 122.70 120.53 118.39 118.32 56.36 51.05 40.11 40.02 39.94 39.85 39.78 39.69 39.61 39.52 39.44 39.35 39.19 39.02 18.78
MeO_2C
N
Br
N
Me
H27
^{13}C NMR (126 MHz, DMSO-*d6*)
190 180 170 160 150 140 130 120 110 100 90 80 70 60 50 40 30 20 10 0
f1 (ppm)

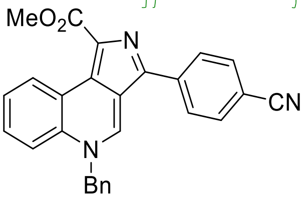

H28

^{1}H NMR (500 MHz, DMSO-*d6*)

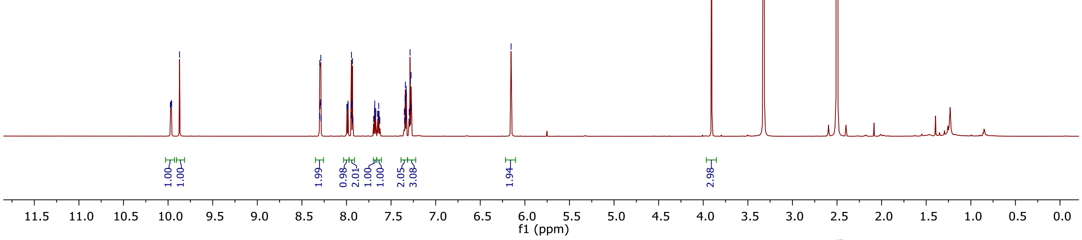

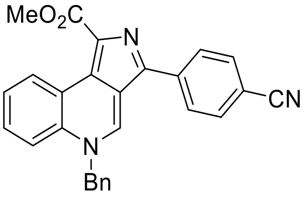

H28

^{13}C NMR (126 MHz, DMSO-*d6*)

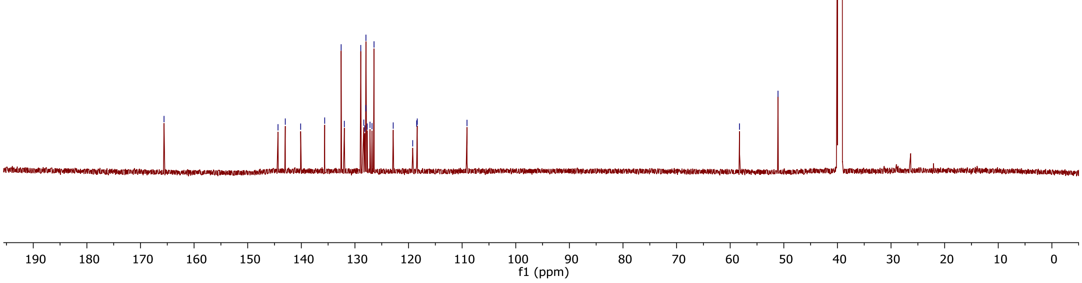

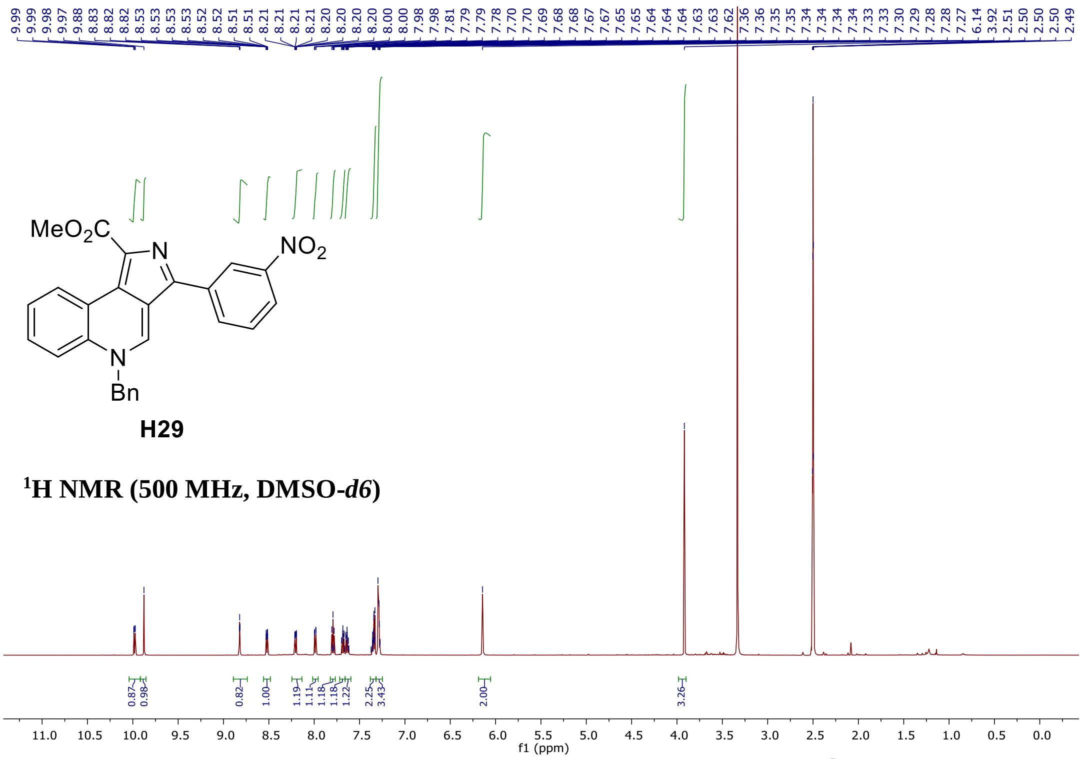
9.99 9.99 9.98 9.97 9.88 8.83 8.82 8.82 8.53 8.53 8.53 8.53 8.52 8.52 8.51 8.51 8.21 8.21 8.21 8.21 8.20 8.20 8.20 8.20 8.00 8.00 7.98 7.98 7.81 7.79 7.79 7.78 7.70 7.70 7.69 7.68 7.68 7.67 7.67 7.65 7.65 7.64 7.64 7.64 7.63 7.63 7.62 7.36 7.36 7.35 7.35 7.34 7.34 7.34 7.34 7.33 7.33 7.30 7.29 7.28 7.28 7.27 6.14 3.92 2.51 2.50 2.50 2.50 2.49
MeO2C
N
NO2
N
Bn
H29
1H NMR (500 MHz, DMSO-d6)
0.87 0.98 0.82 1.00 1.19 1.11 1.18 1.18 1.22 2.25 3.43 2.00 3.26
11.0 10.5 10.0 9.5 9.0 8.5 8.0 7.5 7.0 6.5 6.0 5.5 5.0 4.5 4.0 3.5 3.0 2.5 2.0 1.5 1.0 0.5 0.0
f1 (ppm)

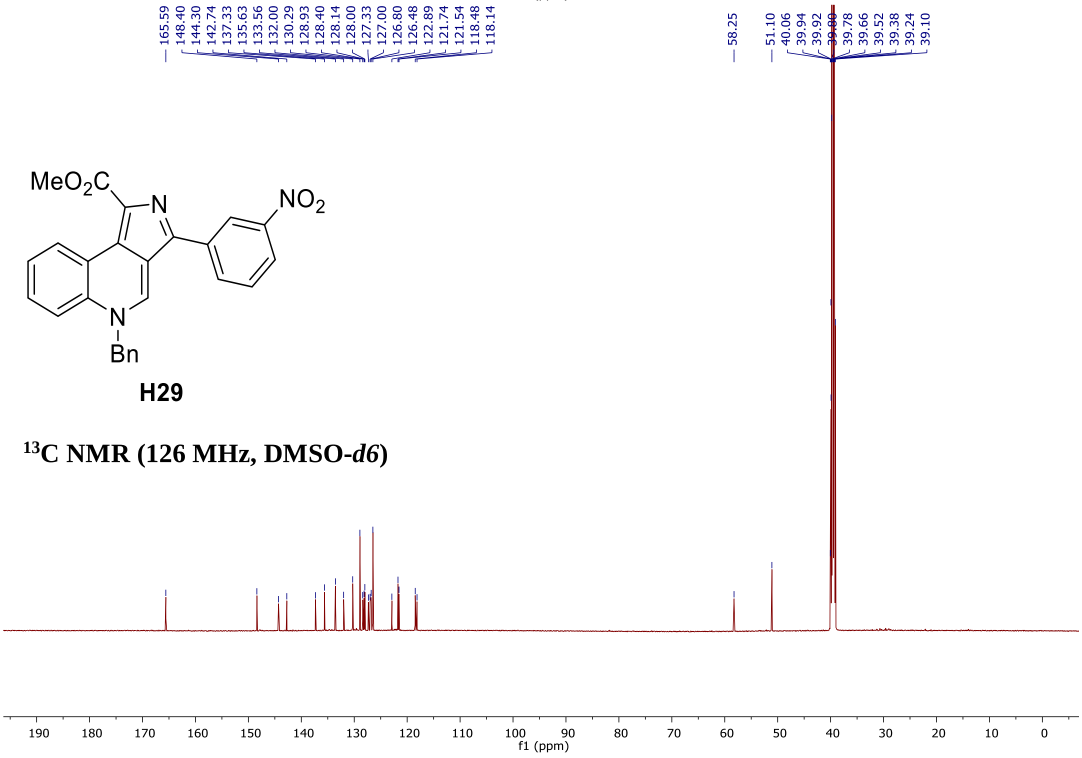
165.59 148.40 144.30 142.74 137.33 135.63 133.56 132.00 130.29 128.93 128.40 128.14 128.00 127.33 127.00 126.80 126.48 122.89 121.74 121.54 118.48 118.14
58.25
51.10 40.06 39.94 39.92 39.80 39.78 39.66 39.52 39.38 39.24 39.10
MeO2C
N
NO2
N
Bn
H29
13C NMR (126 MHz, DMSO-d6)
190 180 170 160 150 140 130 120 110 100 90 80 70 60 50 40 30 20 10 0
f1 (ppm)

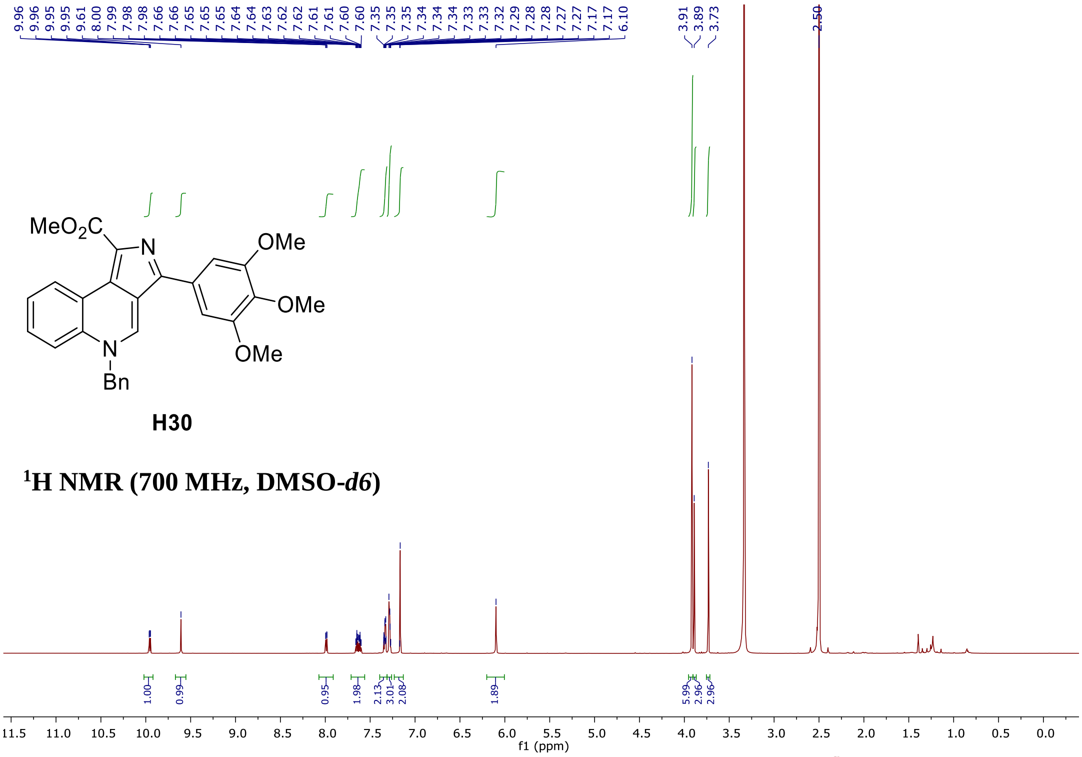
MeO2C
N
OMe
OMe
OMe
N
Bn
H30
1H NMR (700 MHz, DMSO-d6)
9.96 9.96 9.95 9.95 9.61 8.00 7.99 7.98 7.98 7.66 7.66 7.65 7.65 7.65 7.64 7.63 7.62 7.62 7.61 7.60 7.60 7.35 7.35 7.35 7.34 7.34 7.33 7.33 7.32 7.29 7.28 7.28 7.27 7.27 7.17 7.17 6.10
3.91 3.89 3.73
2.50
1.00 0.99 0.95 1.98 2.13 3.01 2.08 1.89 5.99 2.96 2.96
f1 (ppm)

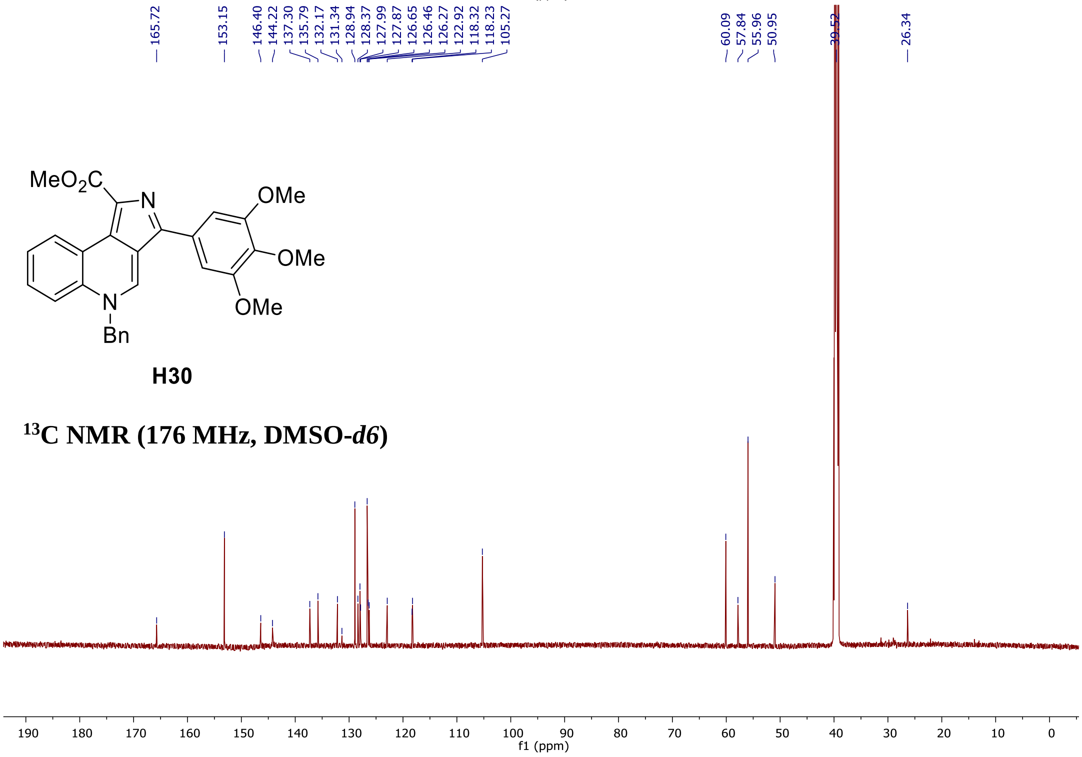
165.72
153.15
146.40 144.22 137.30 135.79 132.17 131.34 128.94 128.37 127.99 127.87 126.65 126.46 126.27 122.92 118.32 118.23 105.27
60.09 57.84 55.96 50.95
39.52
26.34
MeO2C
N
OMe
OMe
OMe
N
Bn
H30
13C NMR (176 MHz, DMSO-d6)
f1 (ppm)

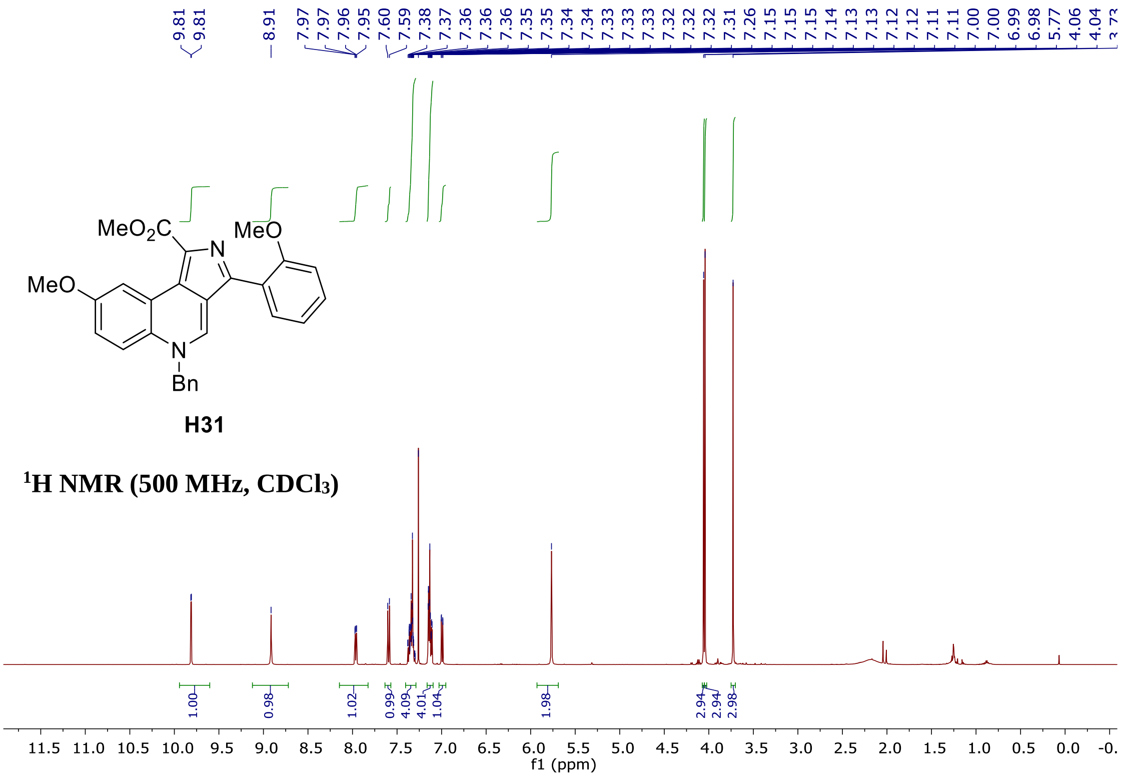

H31

^{1}H NMR (500 MHz, $CDCl_3$)

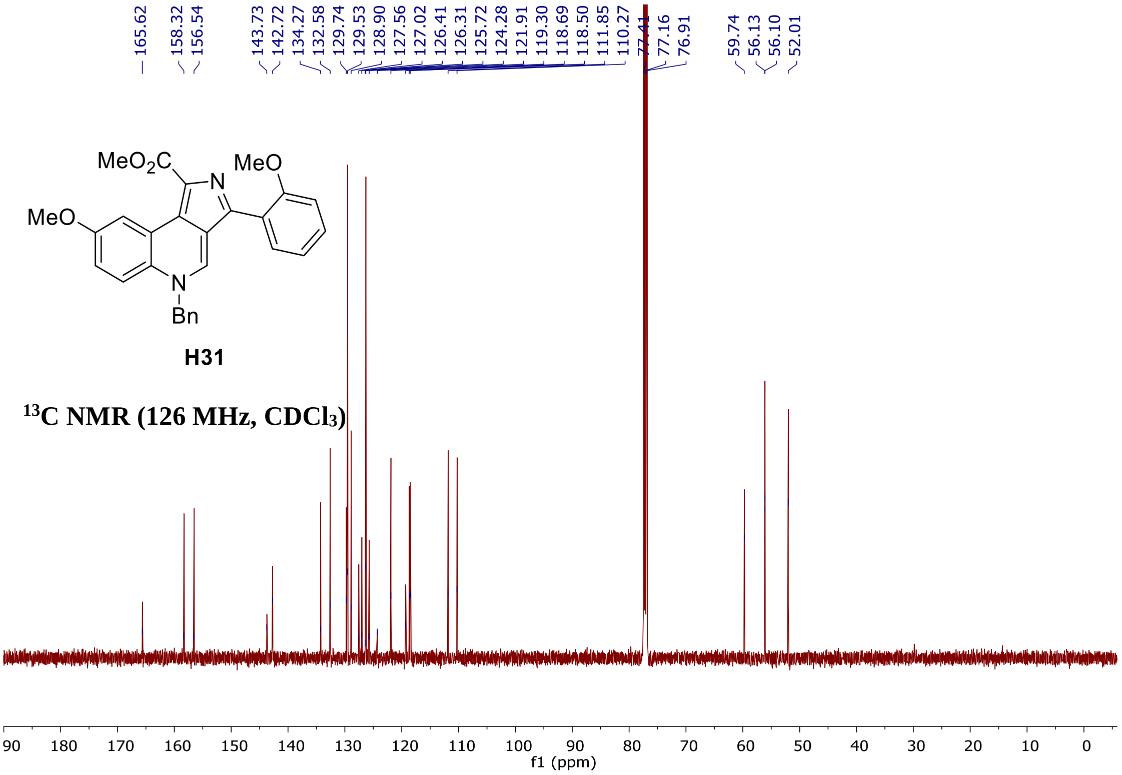

H31

^{13}C NMR (126 MHz, $CDCl_3$)

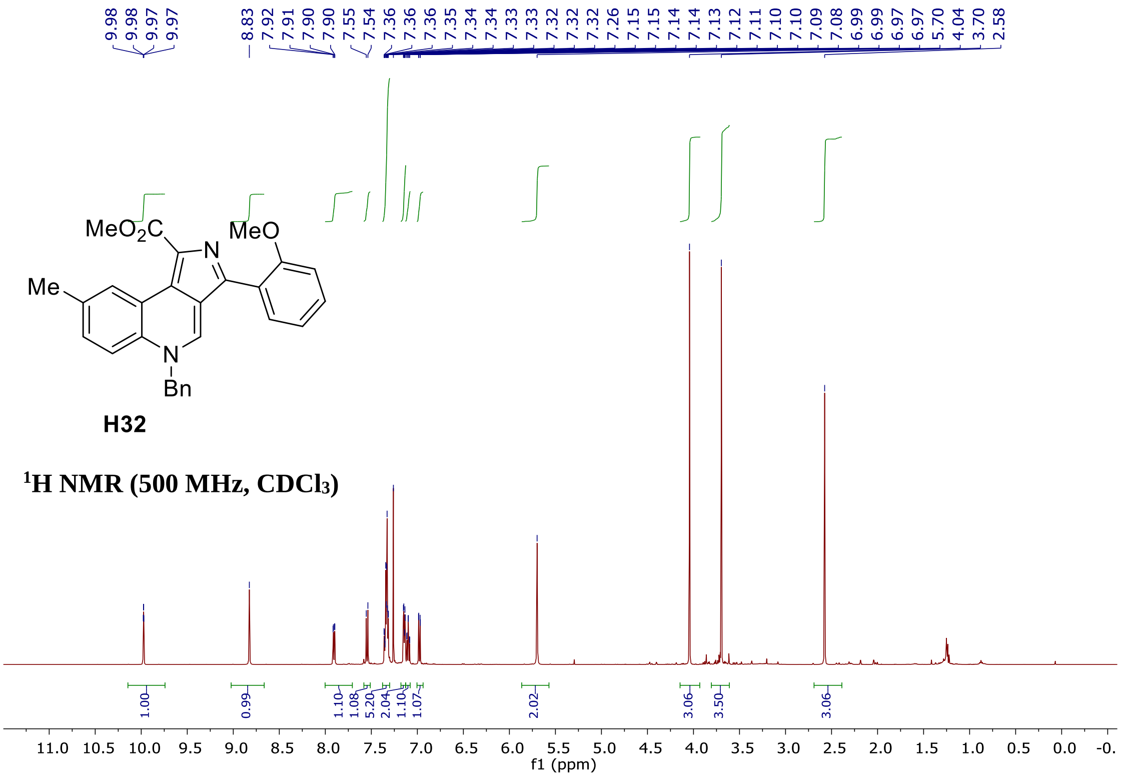
9.98 9.98 9.97 9.97 8.83 7.92 7.91 7.90 7.90 7.55 7.54 7.36 7.36 7.36 7.35 7.34 7.34 7.33 7.33 7.32 7.32 7.32 7.26 7.15 7.15 7.14 7.14 7.13 7.12 7.11 7.10 7.10 7.09 7.08 6.99 6.99 6.97 6.97 5.70 4.04 3.70 2.58
MeO2C
N
MeO
Me
N
Bn
H32
1H NMR (500 MHz, CDCl3)
1.00 0.99 1.10 1.08 5.20 2.04 1.10 1.07 2.02 3.06 3.50 3.06
11.0 10.5 10.0 9.5 9.0 8.5 8.0 7.5 7.0 6.5 6.0 5.5 5.0 4.5 4.0 3.5 3.0 2.5 2.0 1.5 1.0 0.5 0.0 -0.
f1 (ppm)

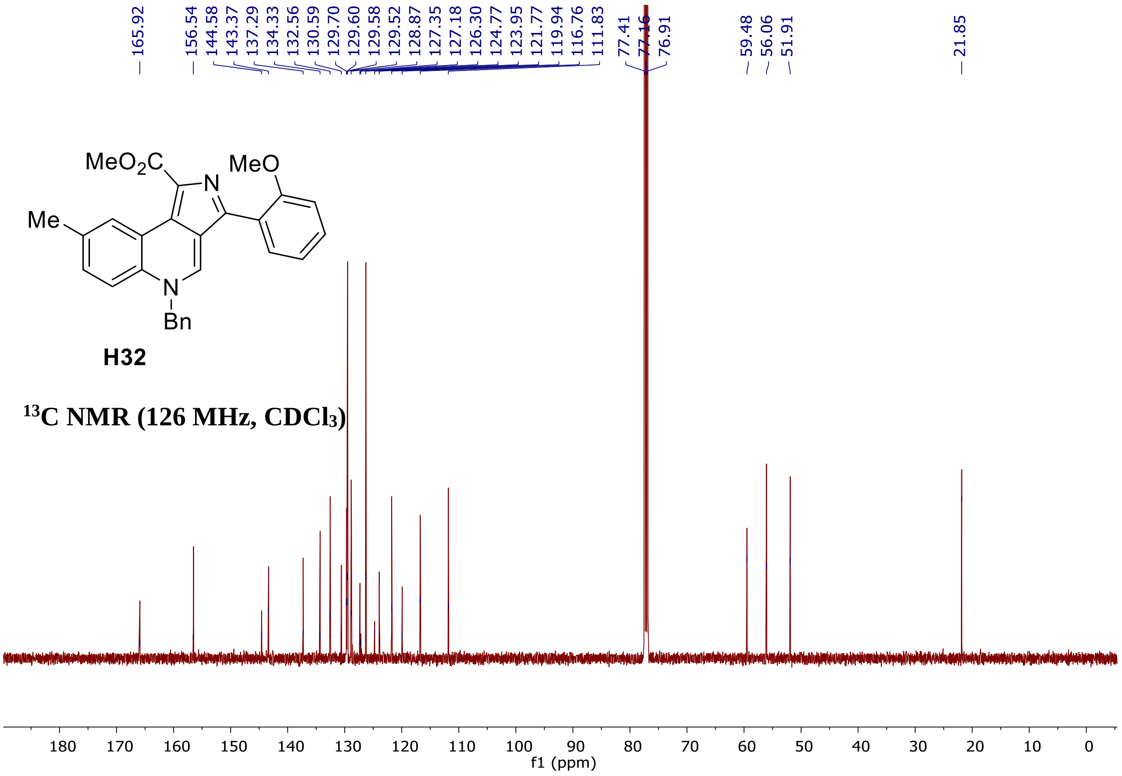
165.92 156.54 144.58 143.37 137.29 134.33 132.56 130.59 129.70 129.60 129.58 129.52 128.87 127.35 127.18 126.30 124.77 123.95 121.77 119.94 116.76 111.83 77.41 77.16 76.91 59.48 56.06 51.91 21.85
MeO2C
N
MeO
Me
N
Bn
H32
13C NMR (126 MHz, CDCl3)
180 170 160 150 140 130 120 110 100 90 80 70 60 50 40 30 20 10 0
f1 (ppm)

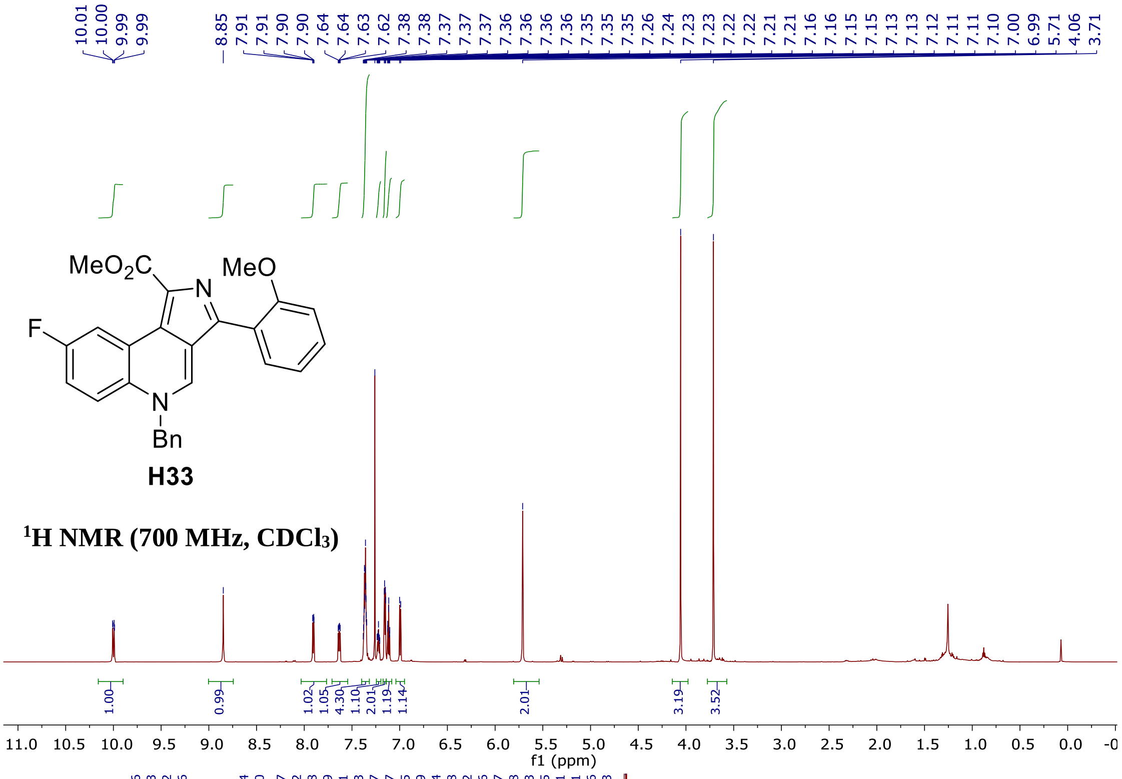

H33

^{1}H NMR (700 MHz, $CDCl_3$)

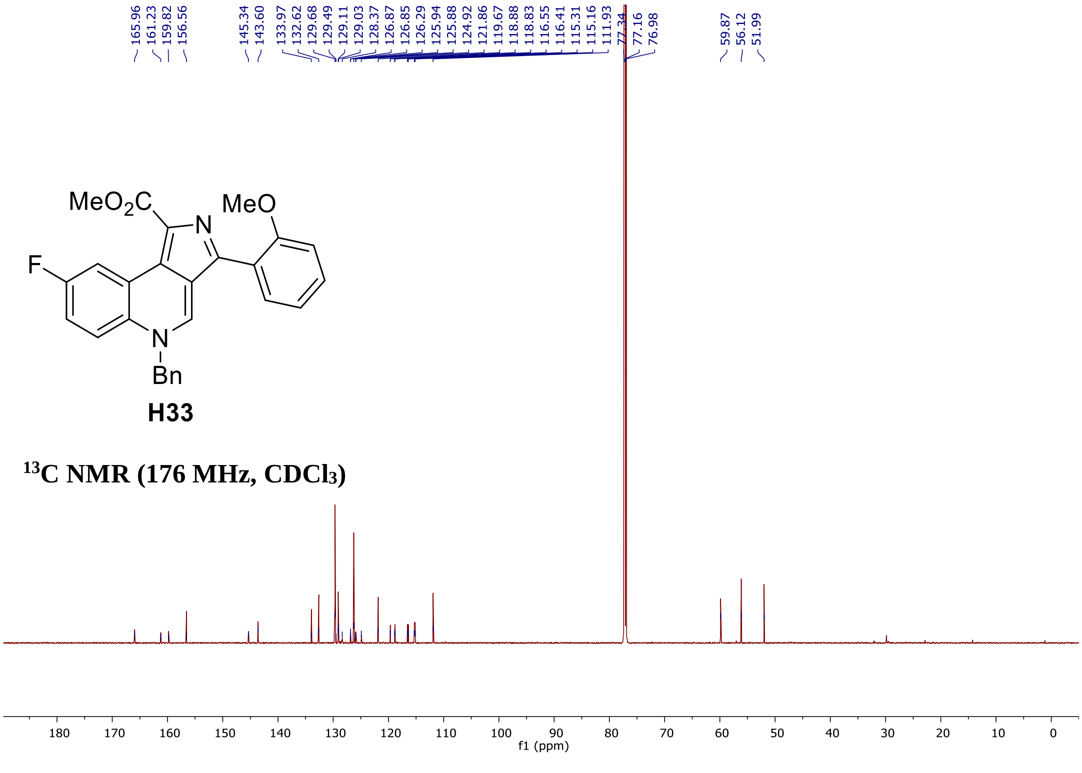

H33

^{13}C NMR (176 MHz, $CDCl_3$)

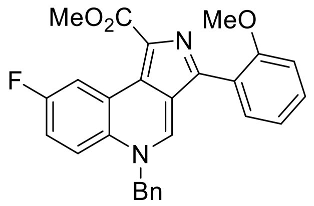

H33

^{19}F NMR (470 MHz, CDCl$_3$)

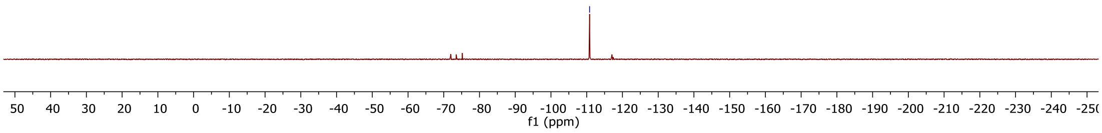

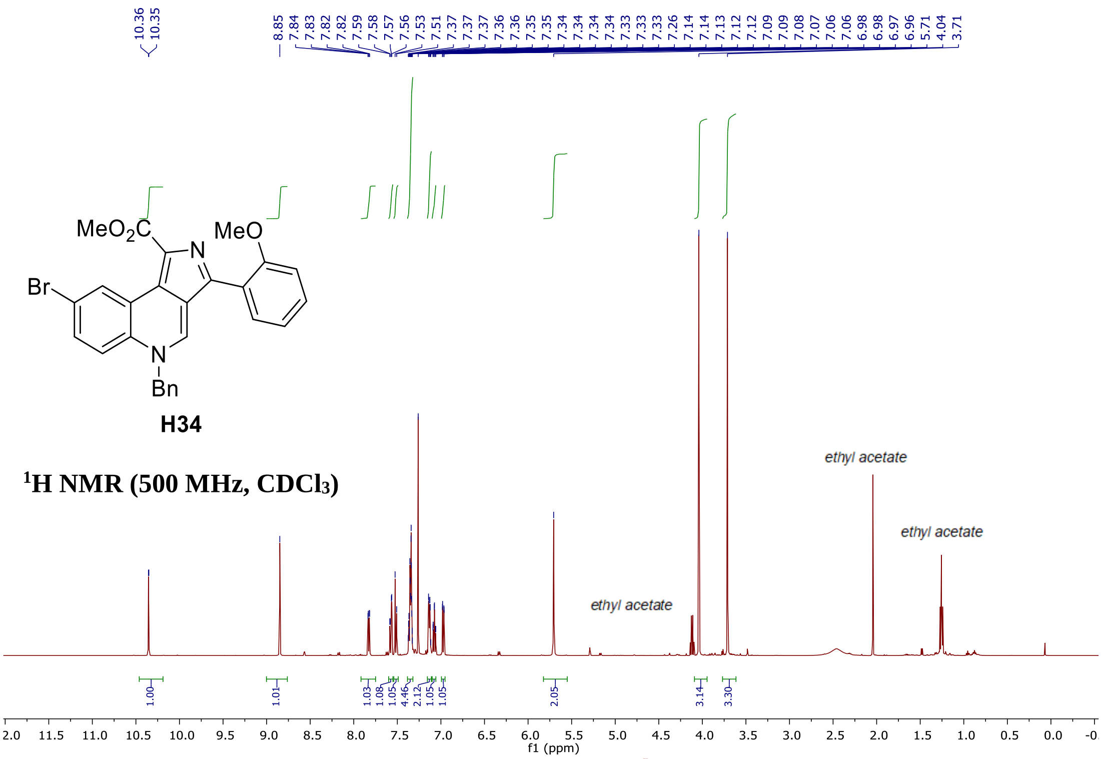

H34

^{1}H NMR (500 MHz, $CDCl_3$)

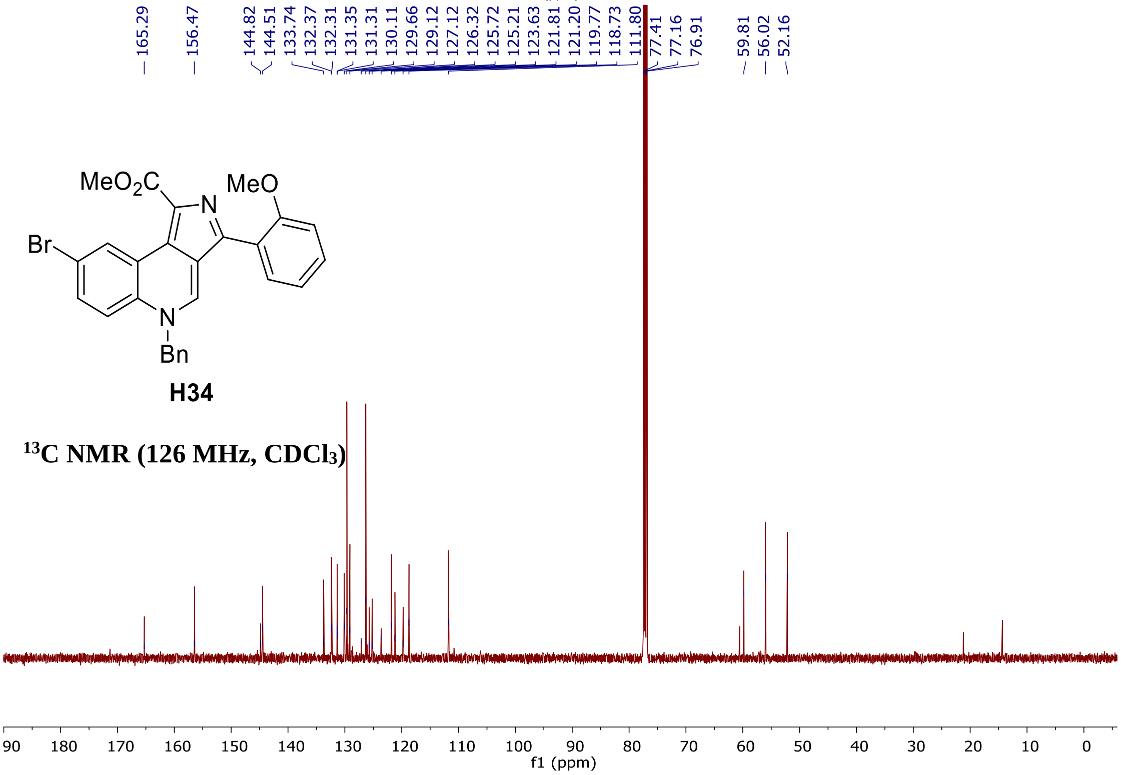

H34

^{13}C NMR (126 MHz, $CDCl_3$)

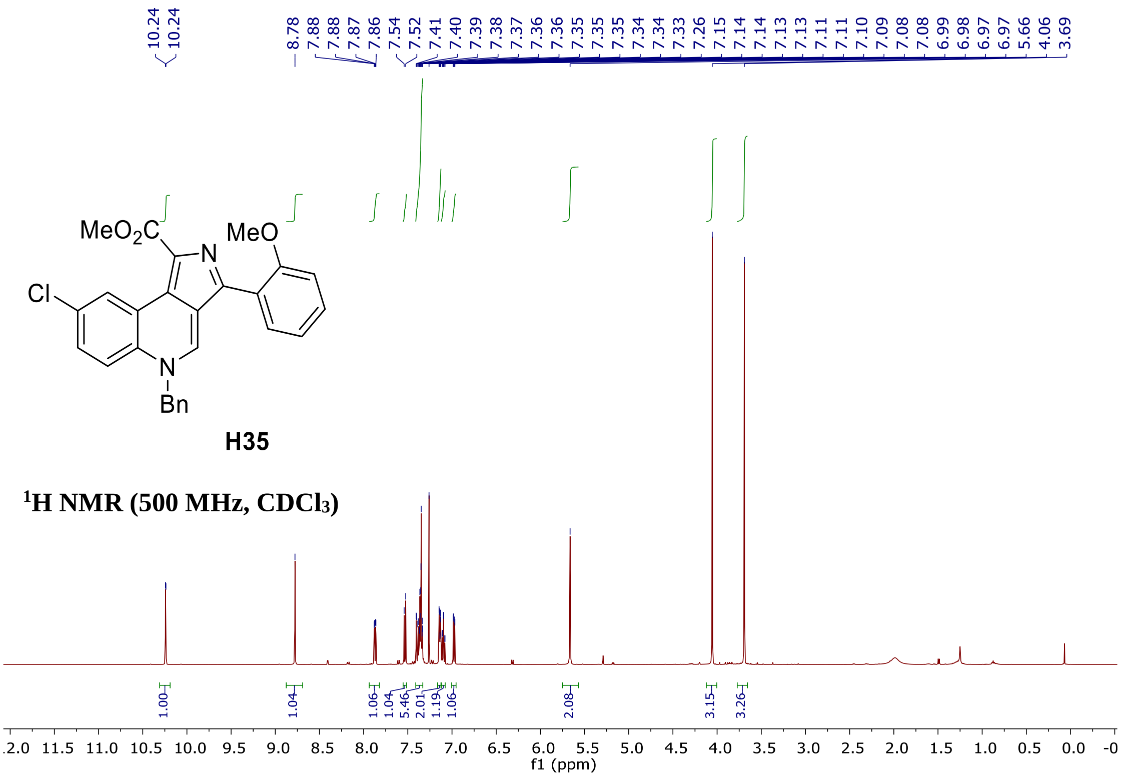

H35

^{1}H NMR (500 MHz, CDCl3)

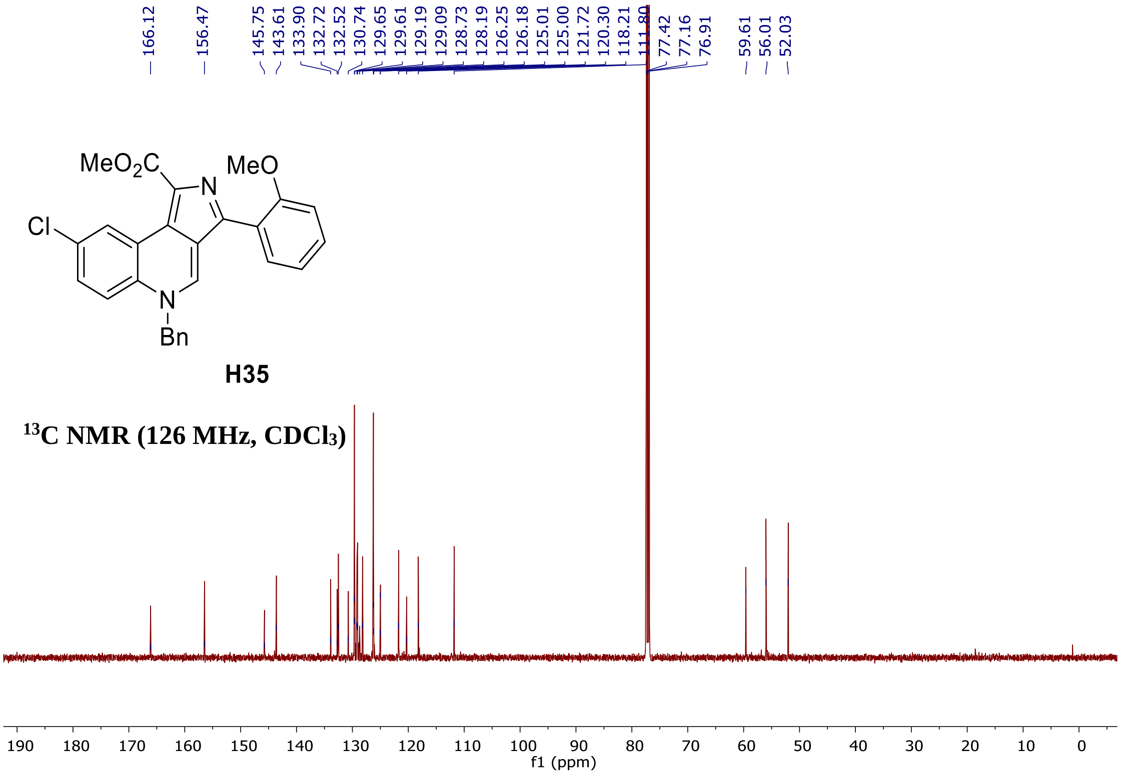

H35

^{13}C NMR (126 MHz, CDCl3)

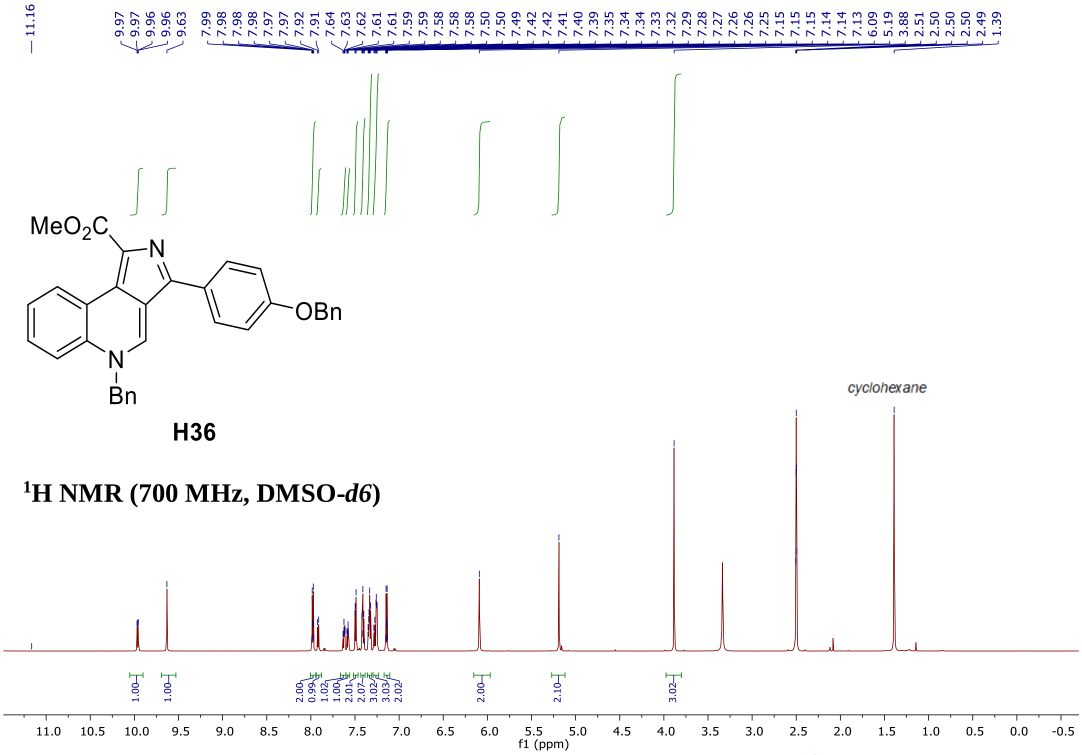
11.16
9.97
9.97
9.96
9.96
9.63
7.99
7.98
7.98
7.98
7.97
7.97
7.92
7.91
7.64
7.63
7.62
7.61
7.61
7.59
7.59
7.58
7.58
7.58
7.50
7.50
7.49
7.42
7.42
7.41
7.40
7.39
7.35
7.34
7.34
7.33
7.32
7.29
7.28
7.27
7.26
7.26
7.25
7.15
7.15
7.15
7.14
7.14
7.13
6.09
5.19
3.88
2.51
2.50
2.50
2.50
2.49
1.39
MeO2C
N
OBn
N
Bn
H36
1H NMR (700 MHz, DMSO-d6)
cyclohexane
1.00
1.00
2.00
0.99
1.02
1.00
2.01
2.07
3.02
3.03
2.02
2.00
2.10
3.02
f1 (ppm)

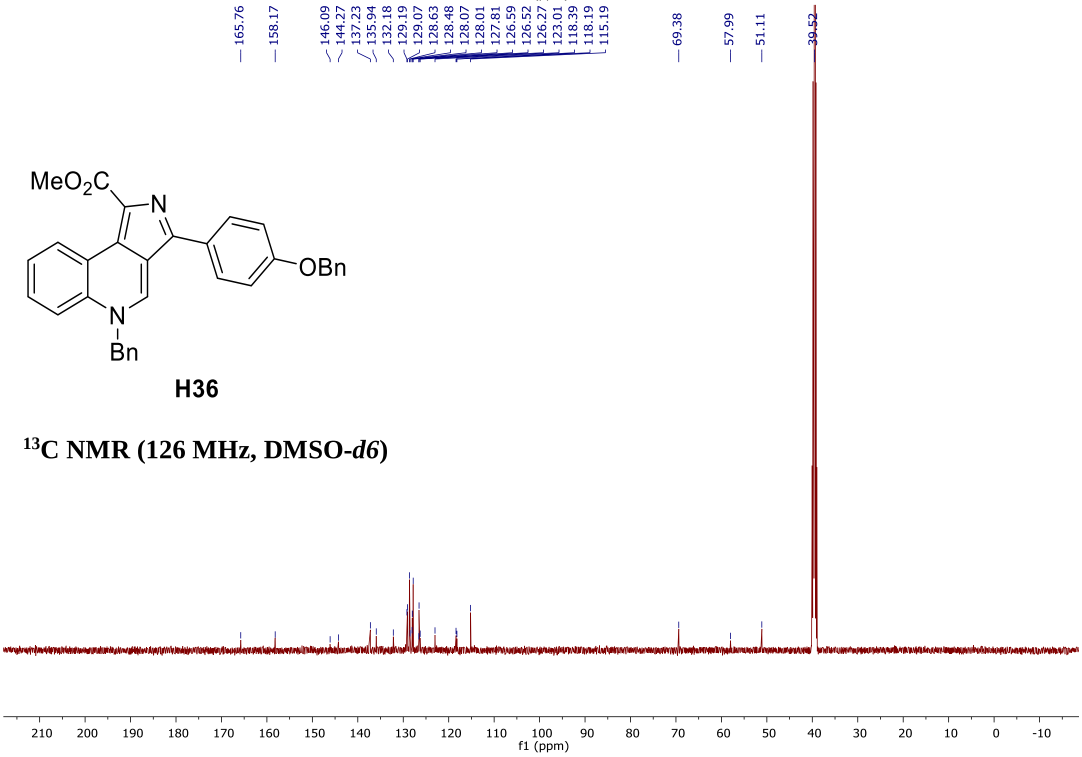
165.76
158.17
146.09
144.27
137.23
135.94
132.18
129.19
129.07
128.63
128.48
128.07
128.01
127.81
126.59
126.52
126.27
123.01
118.39
118.19
115.19
69.38
57.99
51.11
39.52
MeO2C
N
OBn
N
Bn
H36
13C NMR (126 MHz, DMSO-d6)
f1 (ppm)

Full spectra of representative scaffolds (class **B**, **C**, **D**)

B7

Key ^{1}H and ^{13}C NMR signals

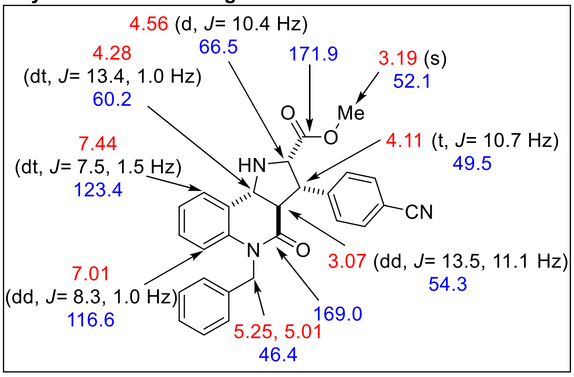

Key ^{1}H-^{1}H COSY correlations

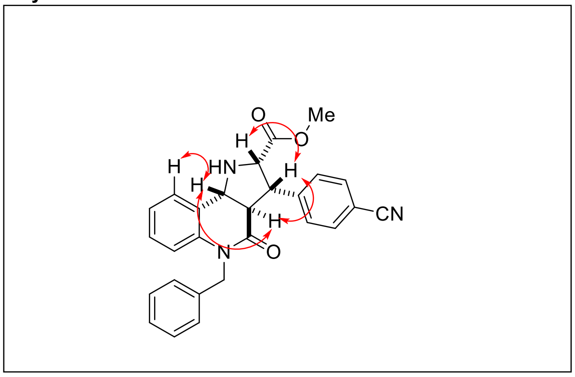

Key HMBC correlations

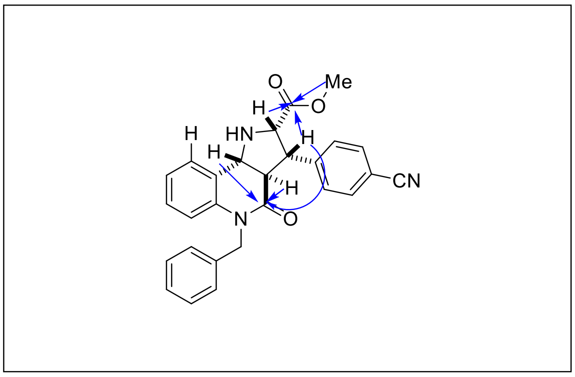

Key NOESY correlations

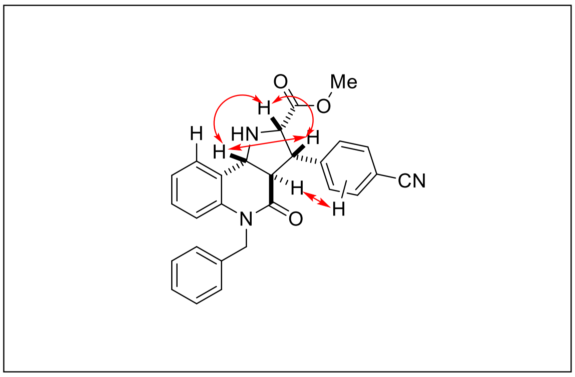

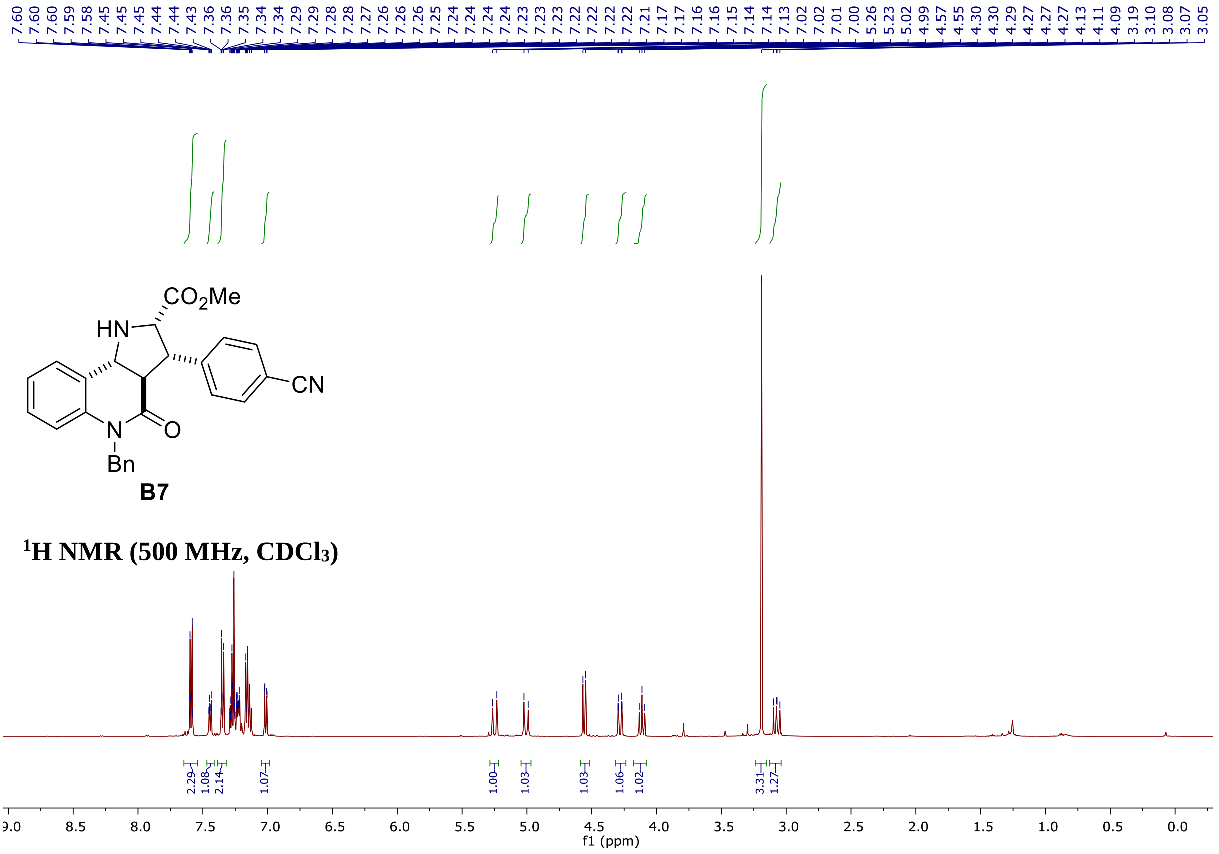

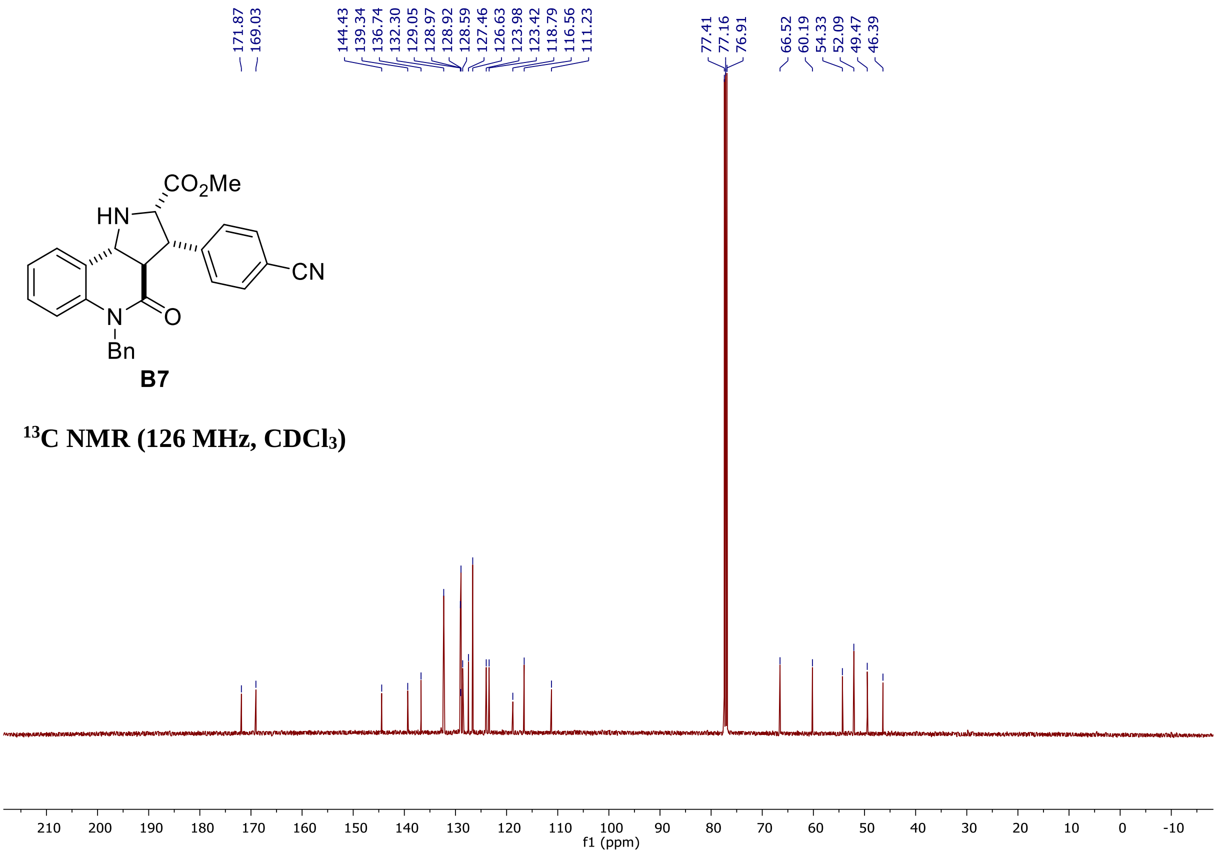
171.87
169.03
144.43
139.34
136.74
132.30
129.05
128.97
128.92
128.59
127.46
126.63
123.98
123.42
118.79
116.56
111.23
77.41
77.16
76.91
66.52
60.19
54.33
52.09
49.47
46.39
CO2Me
HN
CN
N
O
Bn
B7
13C NMR (126 MHz, CDCl3)
f1 (ppm)

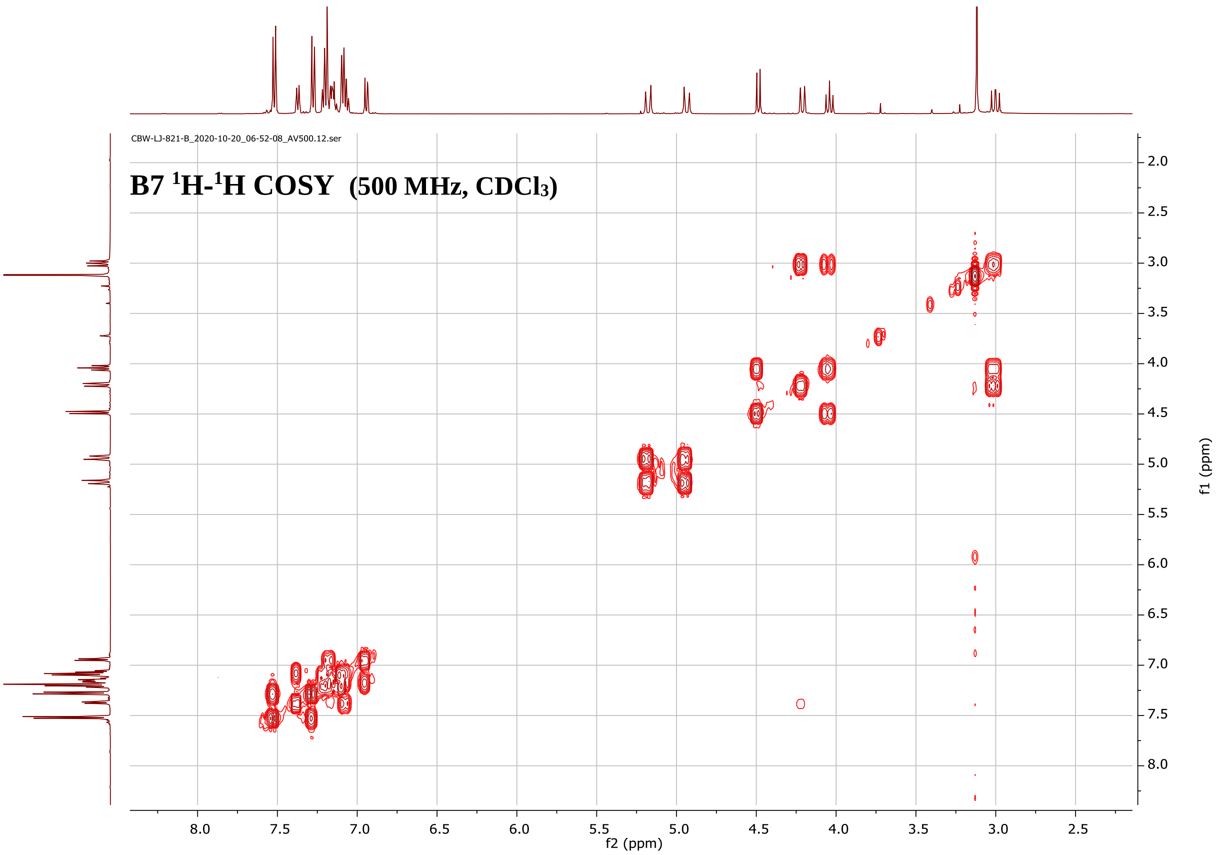
CBW-LJ-821-B_2020-10-20_06-52-08_AV500.12.ser
B7 1H-1H COSY (500 MHz, CDCl3)
f2 (ppm)
f1 (ppm)

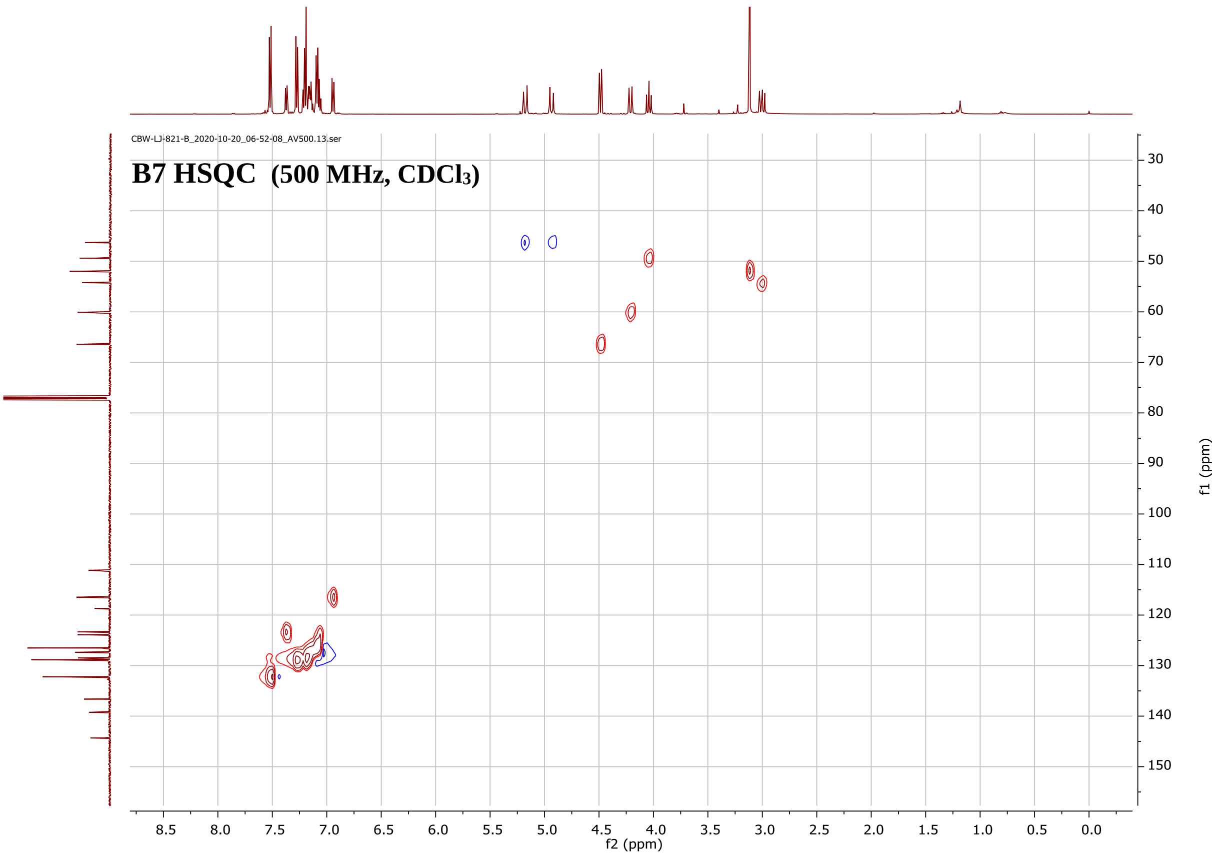
CBW-LJ-821-B_2020-10-20_06-52-08_AV500.13.ser
B7 HSQC (500 MHz, CDCl3)
f2 (ppm)
f1 (ppm)

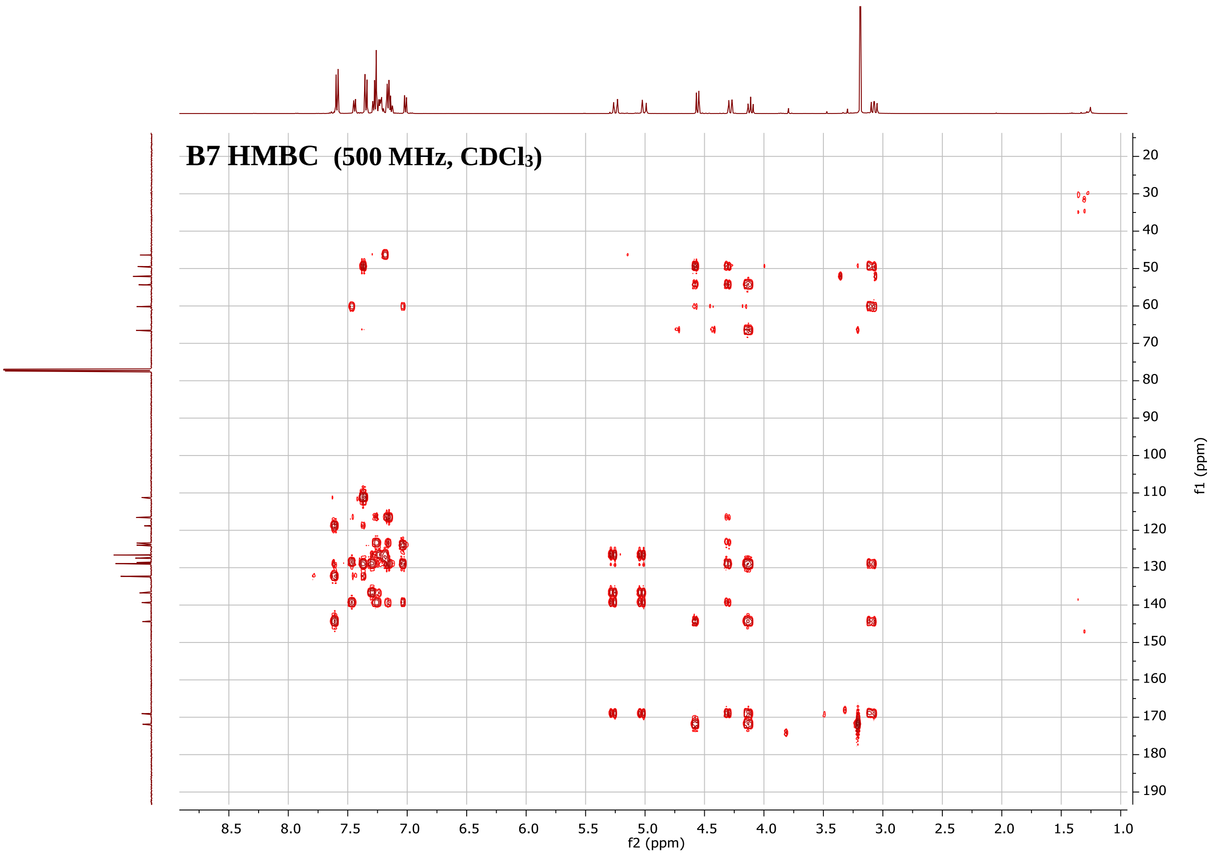
B7 HMBC (500 MHz, CDCl3)
f2 (ppm)
f1 (ppm)

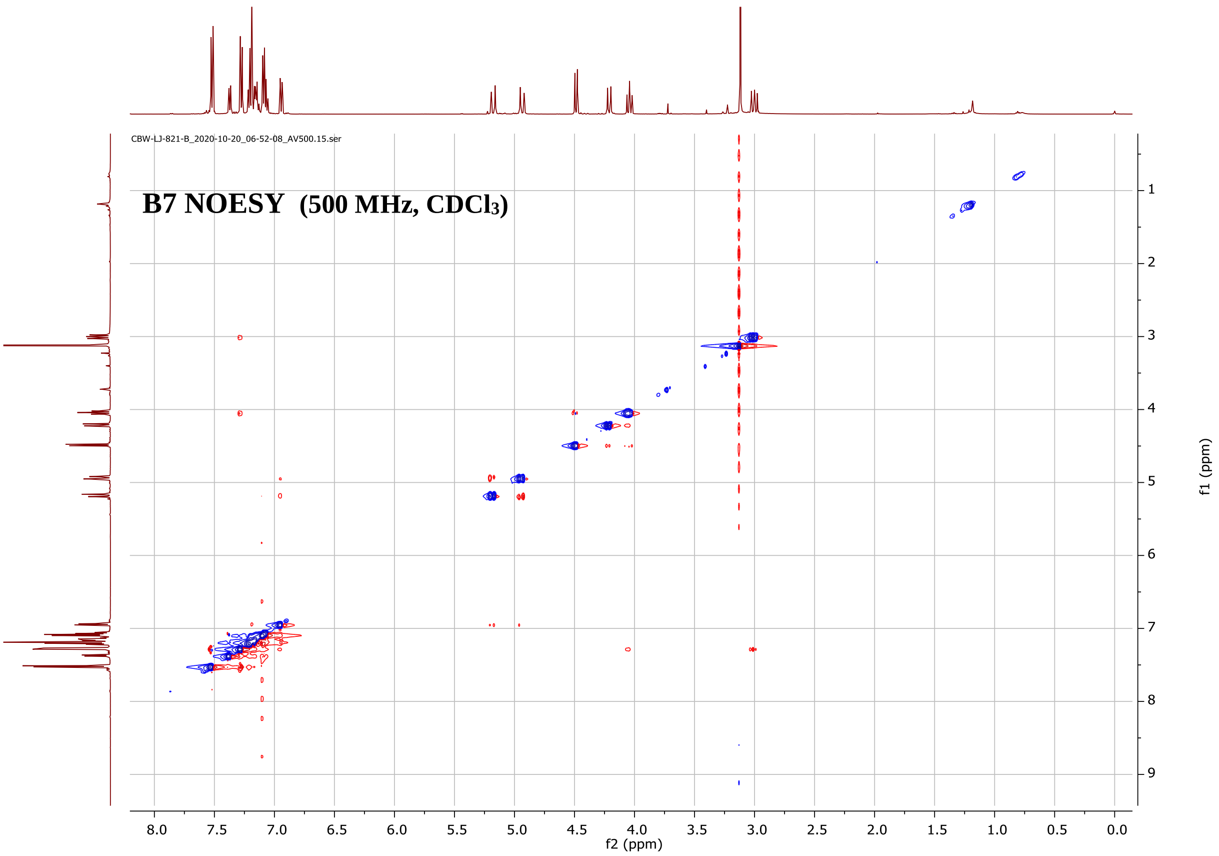

C1

Key ^{1}H and ^{13}C NMR signals

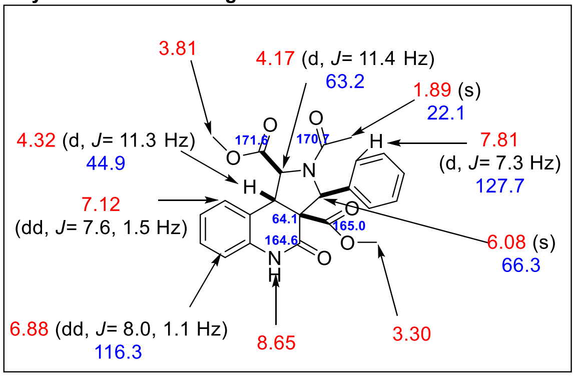

Key ^{1}H-^{1}H COSY correlations

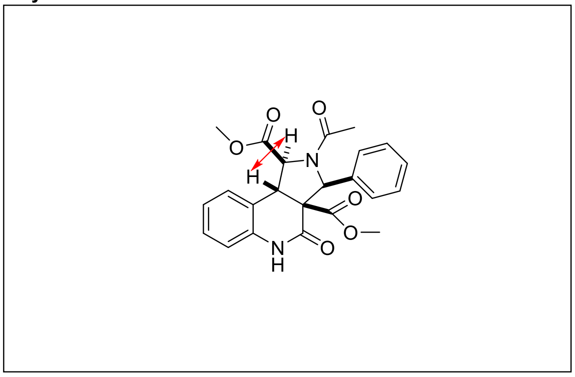

Key HMBC correlations

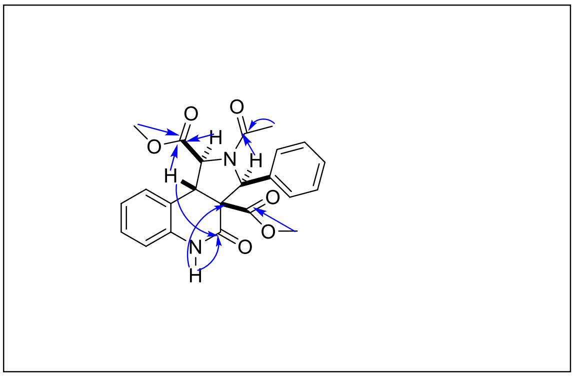

Key NOESY correlations

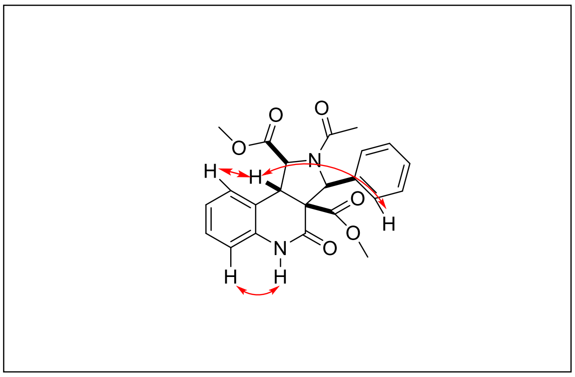

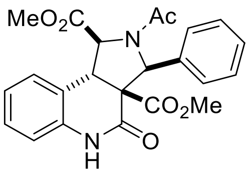

C1

^{1}H NMR (500 MHz, CDCl$_3$)

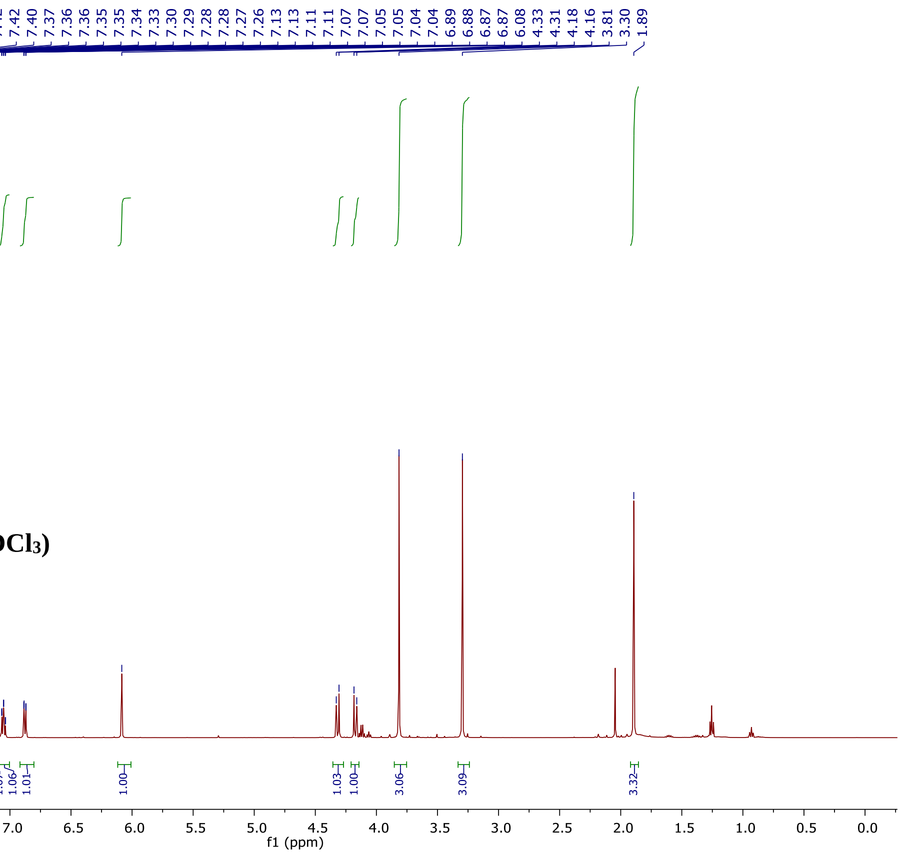

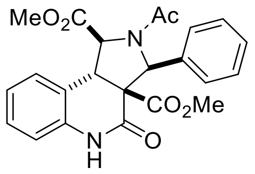

C1

^{13}C NMR (126 MHz, CDCl$_3$)

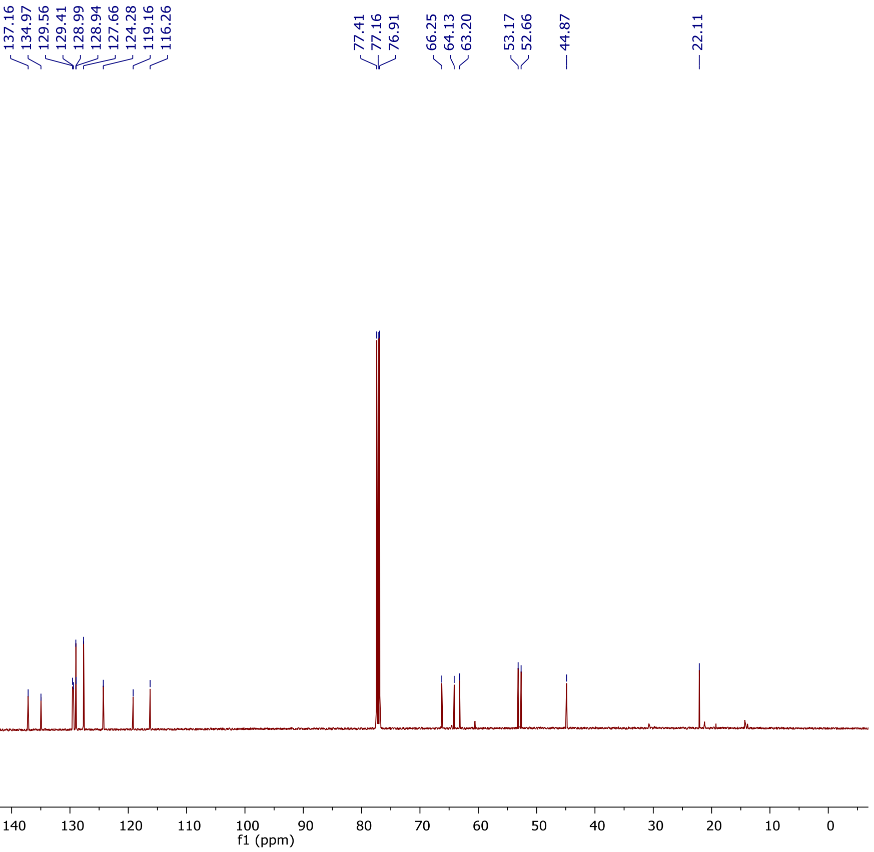

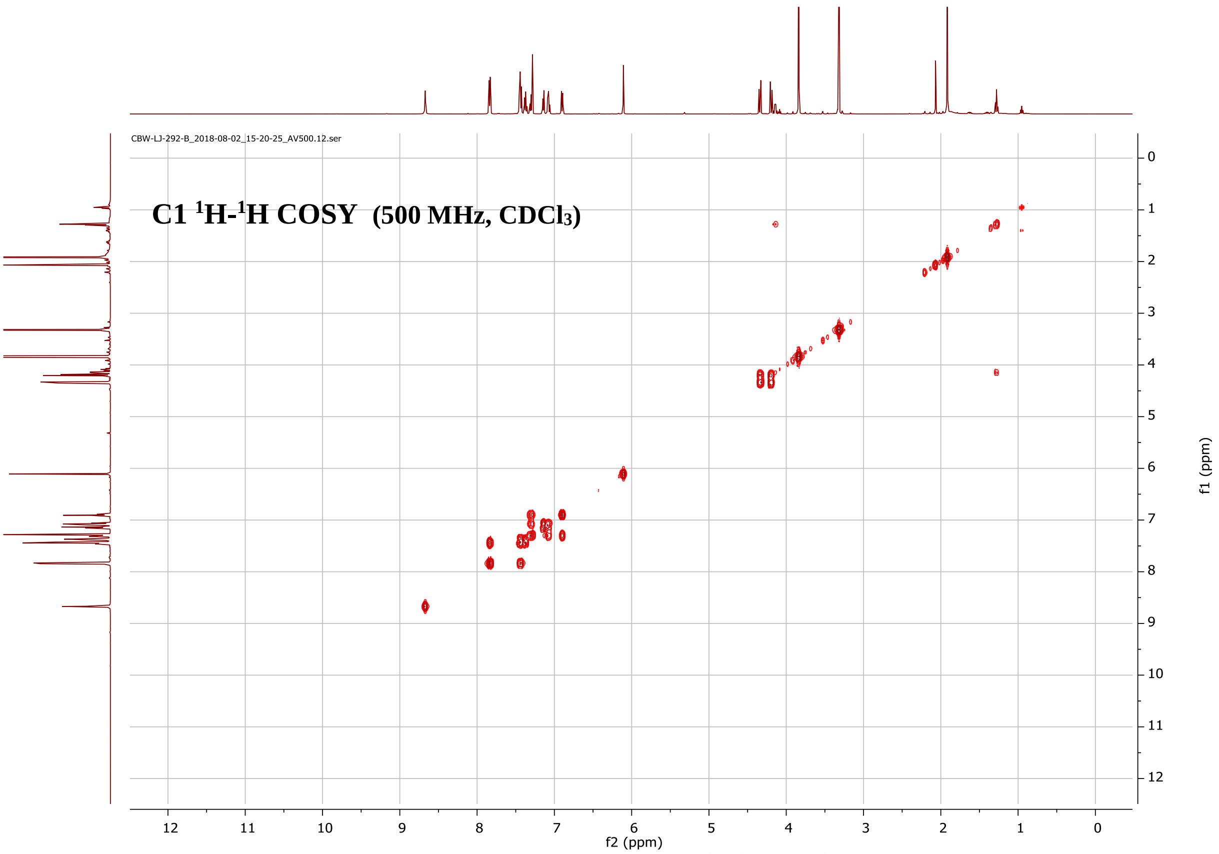
CBW-LJ-292-B_2018-08-02_15-20-25_AV500.12.ser
C1 ¹H-¹H COSY (500 MHz, CDCl₃)
f2 (ppm)
f1 (ppm)

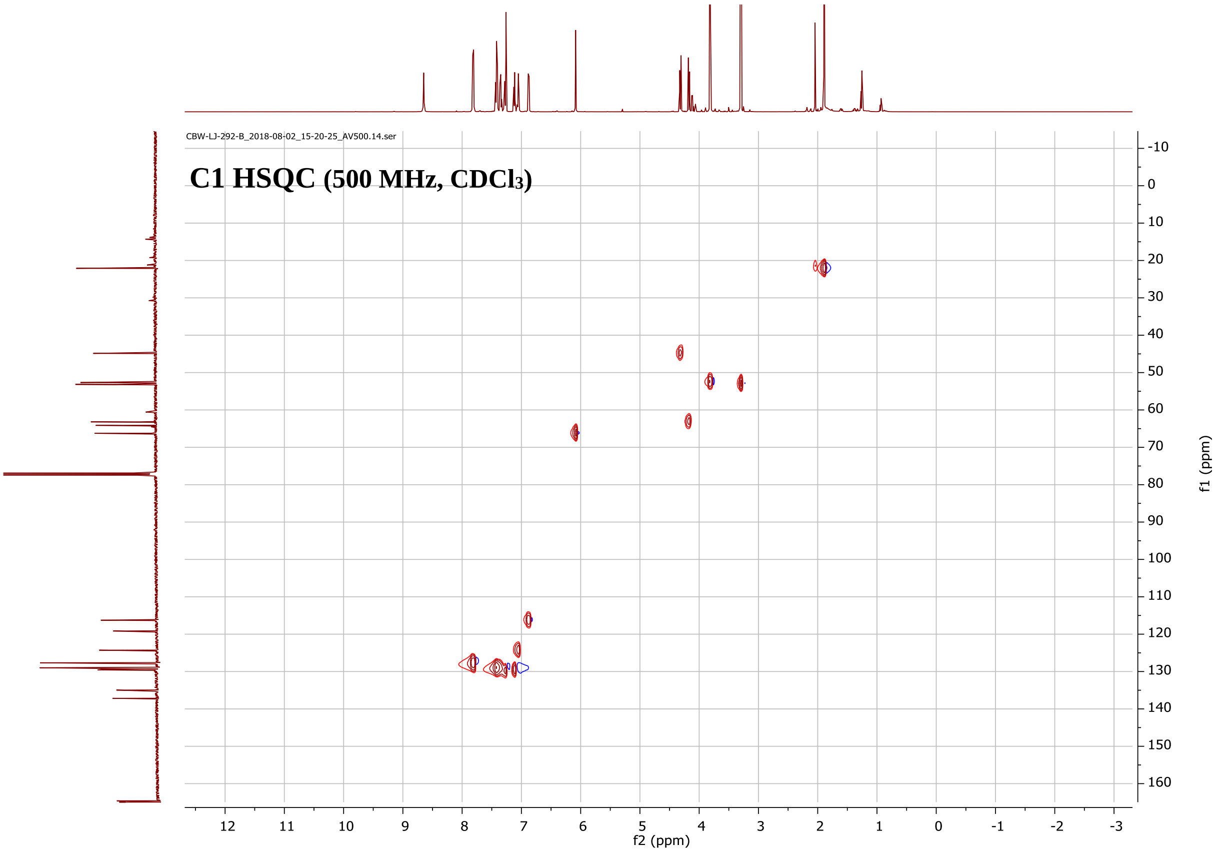
CBW-LJ-292-B_2018-08-02_15-20-25_AV500.14.ser
C1 HSQC (500 MHz, CDCl₃)
f2 (ppm)
f1 (ppm)

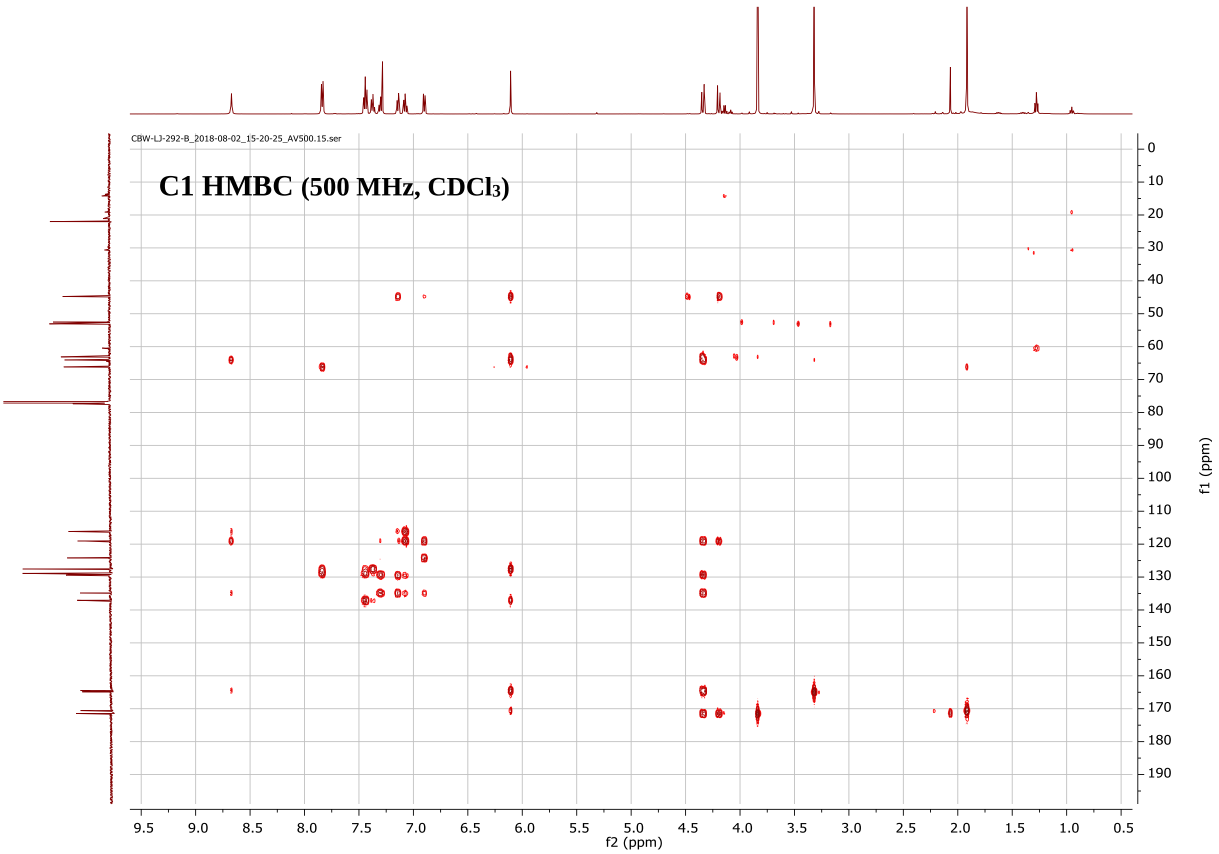
CBW-LJ-292-B_2018-08-02_15-20-25_AV500.15.ser
C1 HMBC (500 MHz, CDCl3)
f2 (ppm)
f1 (ppm)

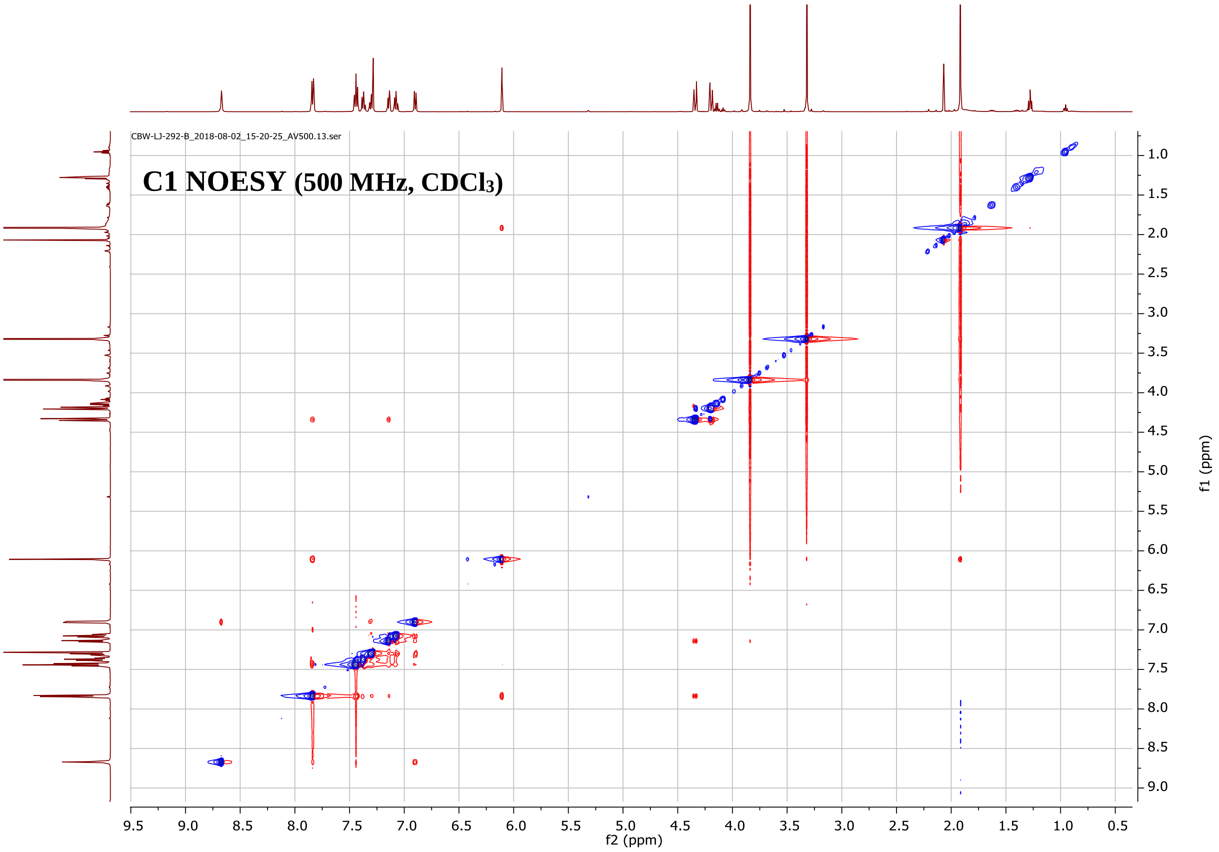
CBW-LJ-292-B_2018-08-02_15-20-25_AV500.13.ser
C1 NOESY (500 MHz, CDCl3)
f2 (ppm)
f1 (ppm)

D10

Key ^{1}H and ^{13}C NMR signals

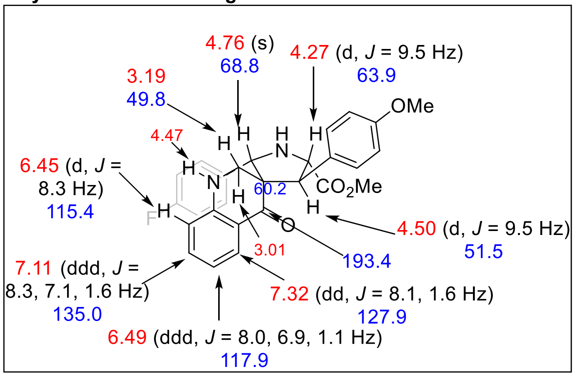

Key ^{1}H-^{1}H COSY correlations

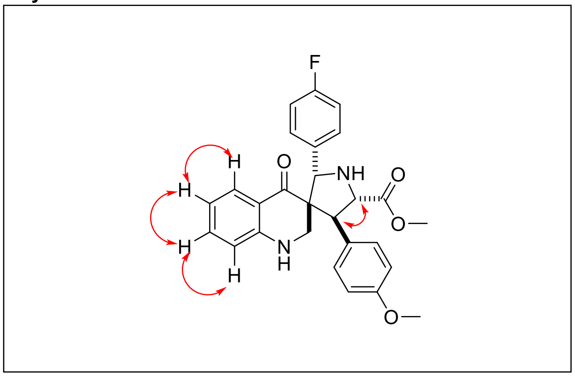

Key HMBC correlations

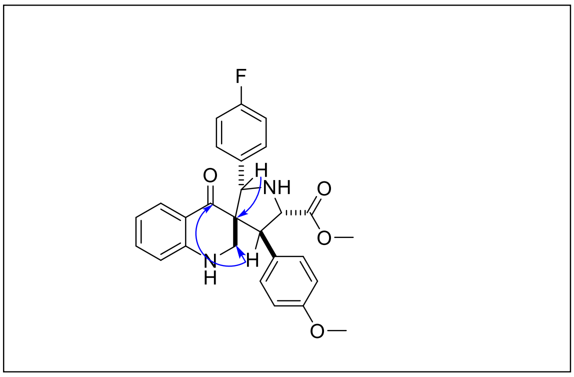

Key NOESY correlations

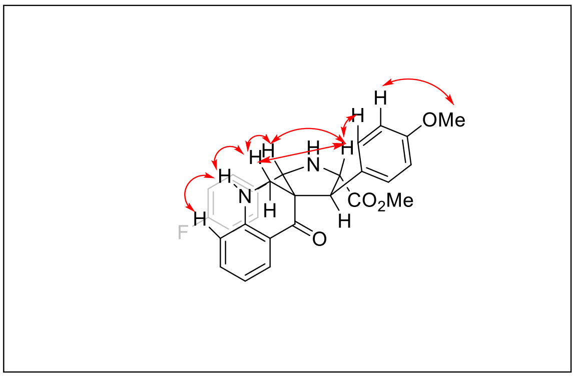

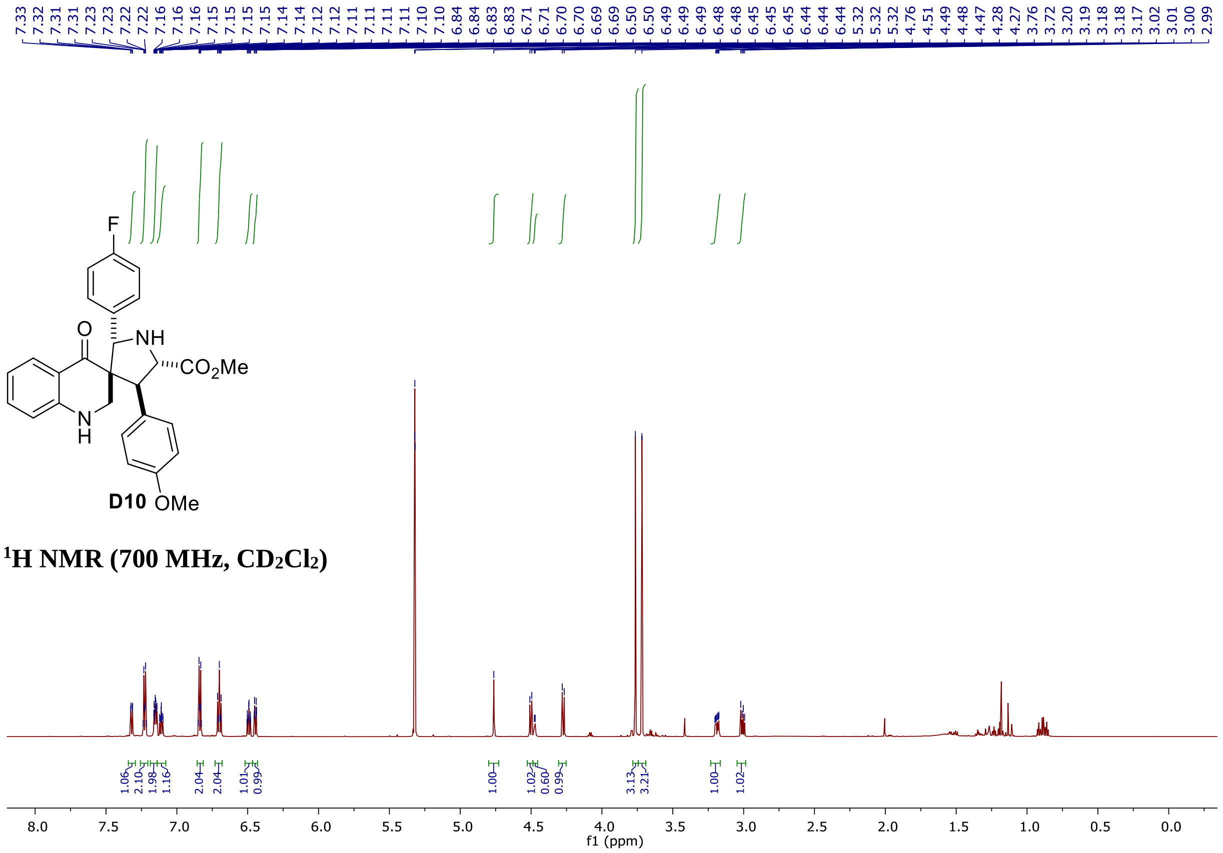

^{1}H NMR (700 MHz, CD_2Cl_2)

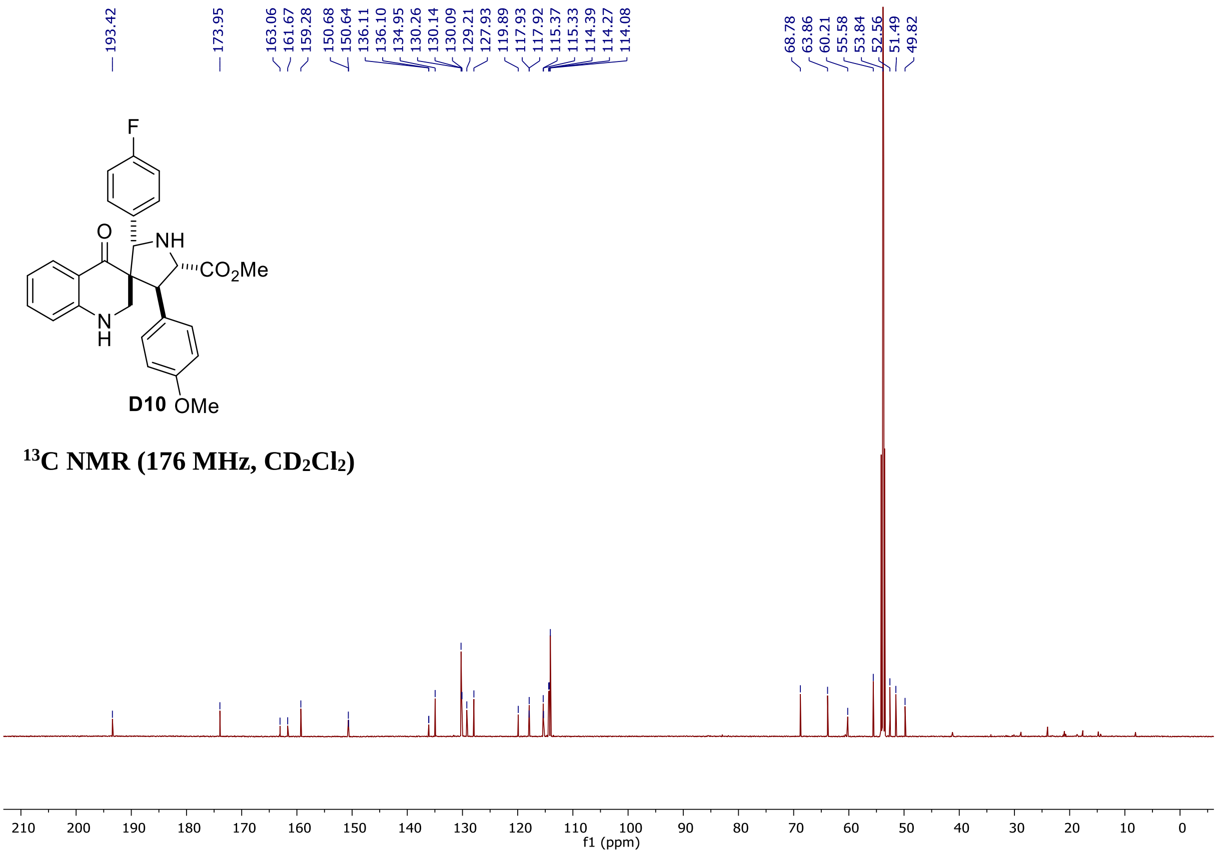

193.42
173.95
163.06
161.67
159.28
150.68
150.64
136.11
136.10
134.95
130.26
130.14
130.09
129.21
127.93
119.89
117.93
117.92
115.37
115.33
114.39
114.27
114.08
68.78
63.86
60.21
55.58
53.84
52.56
51.49
49.82
F
O
NH
CO2Me
N
H
D10
OMe
13C NMR (176 MHz, CD2Cl2)
210 200 190 180 170 160 150 140 130 120 110 100 90 80 70 60 50 40 30 20 10 0
f1 (ppm)

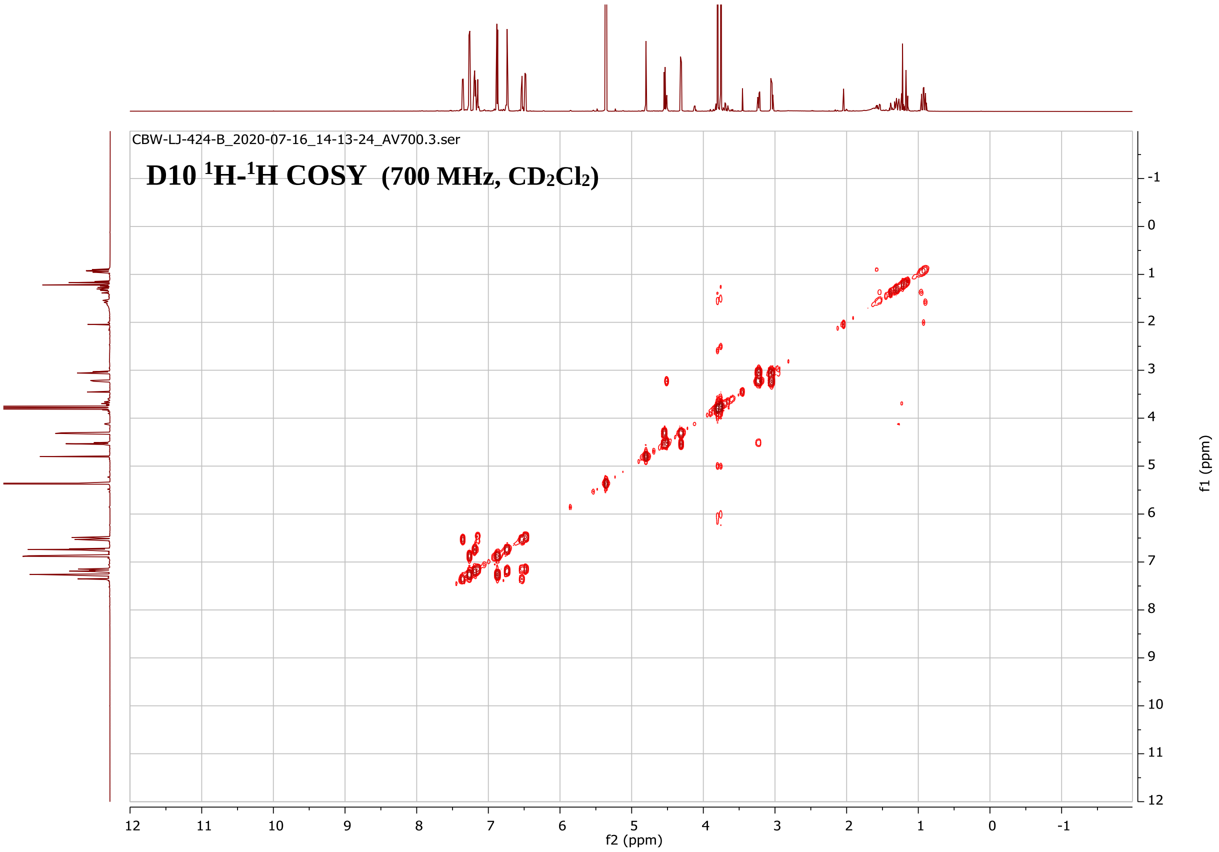

CBW-LJ-424-B_2020-07-16_14-13-24_AV700.3.ser
D10 1H-1H COSY (700 MHz, CD2Cl2)
-1 0 1 2 3 4 5 6 7 8 9 10 11 12
f1 (ppm)
12 11 10 9 8 7 6 5 4 3 2 1 0 -1
f2 (ppm)

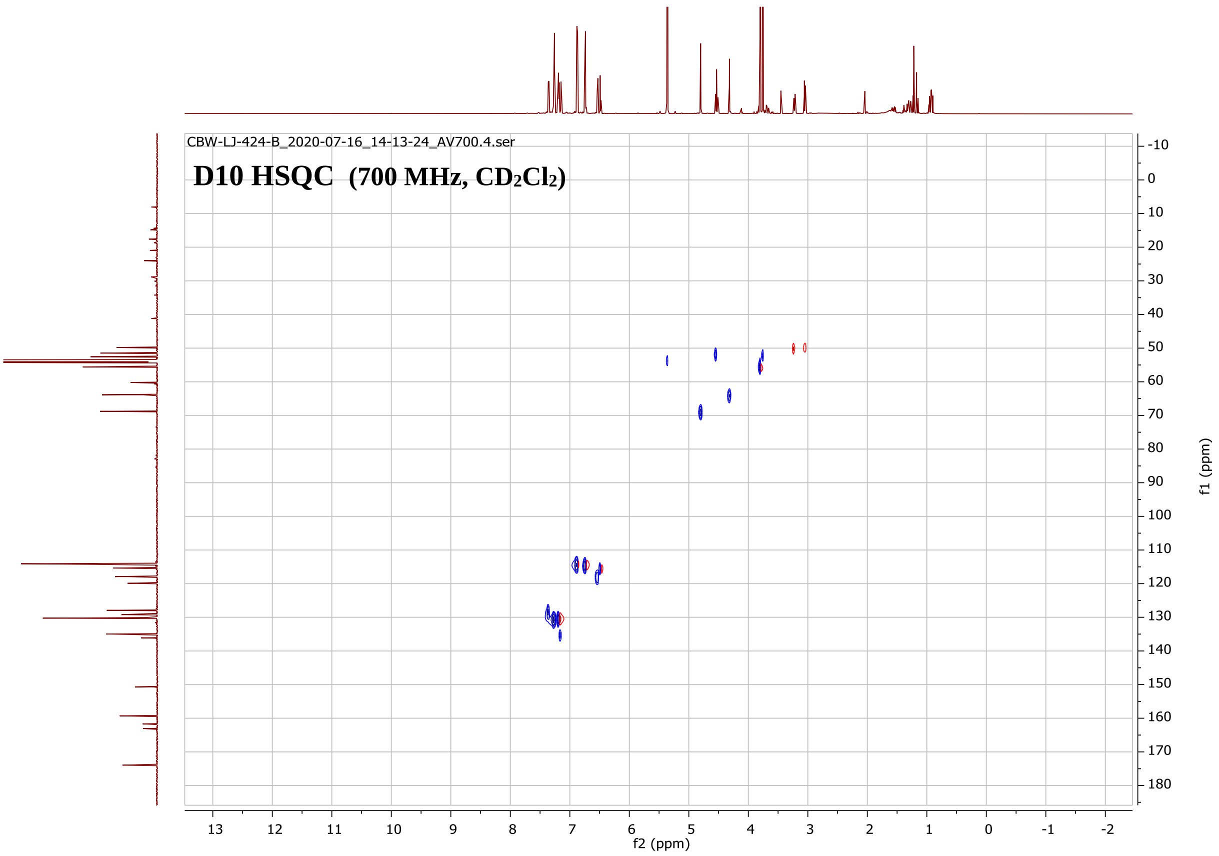
CBW-LJ-424-B_2020-07-16_14-13-24_AV700.4.ser
D10 HSQC (700 MHz, CD2Cl2)
f2 (ppm)
f1 (ppm)

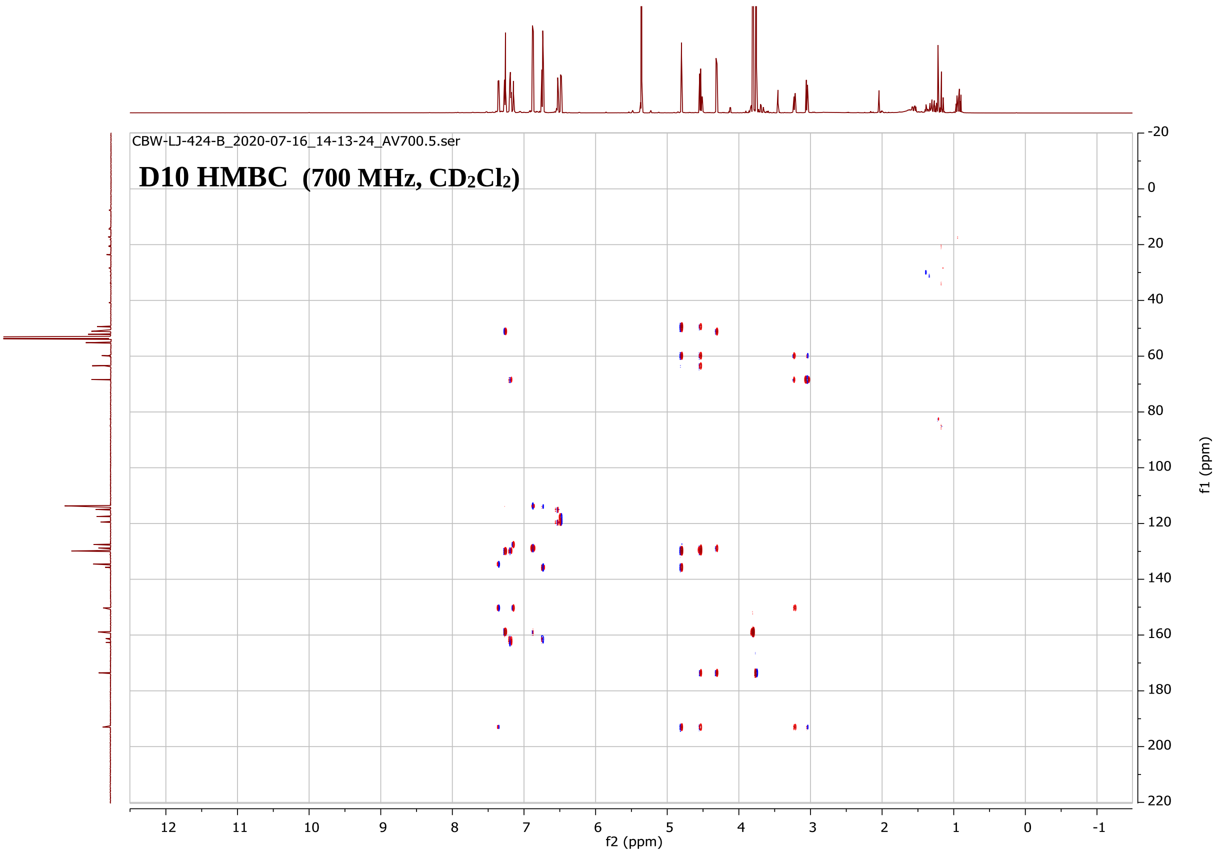
CBW-LJ-424-B_2020-07-16_14-13-24_AV700.5.ser
D10 HMBC (700 MHz, CD2Cl2)
f2 (ppm)
f1 (ppm)

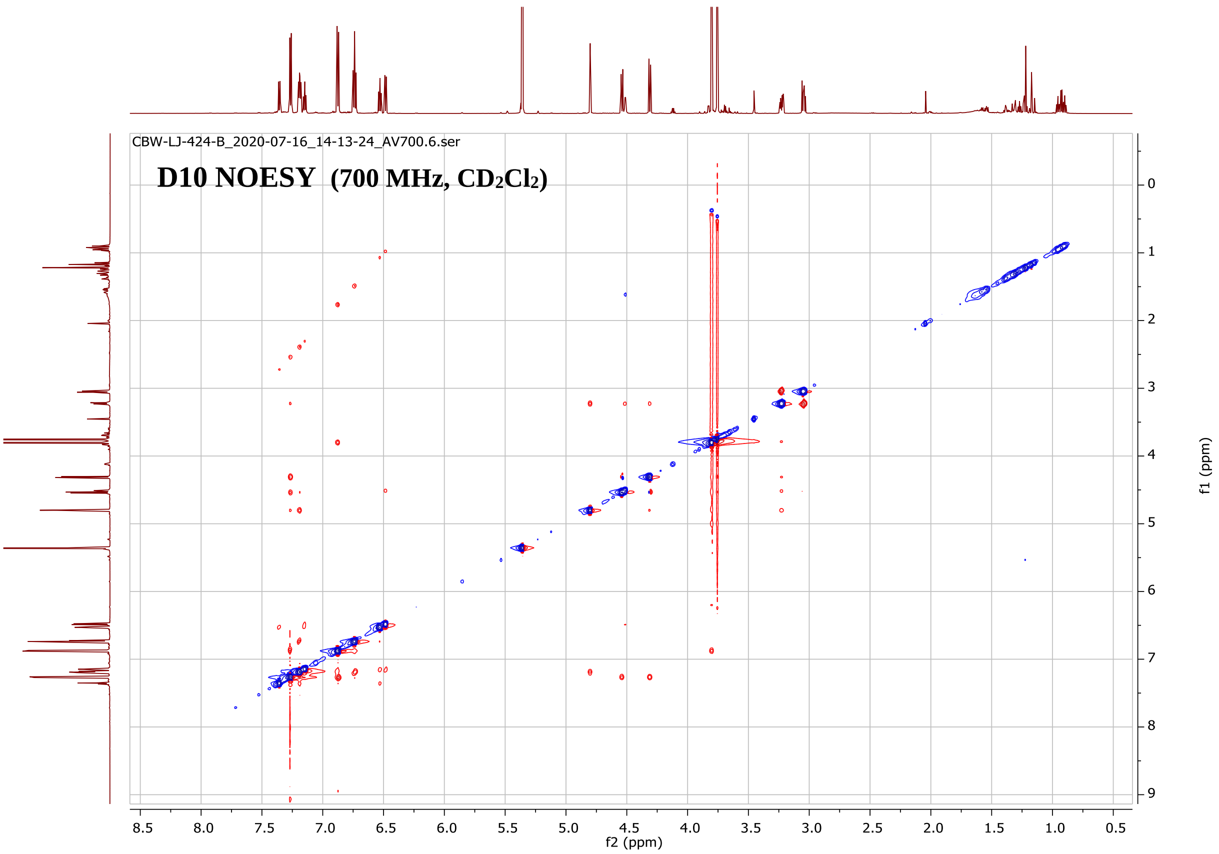

D11

Key ^{1}H and ^{13}C NMR signals

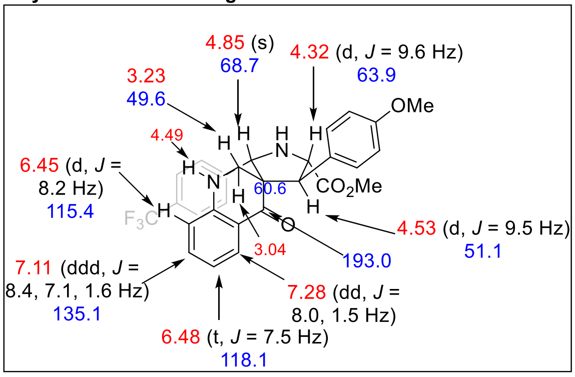

Key ^{1}H-^{1}H COSY correlations

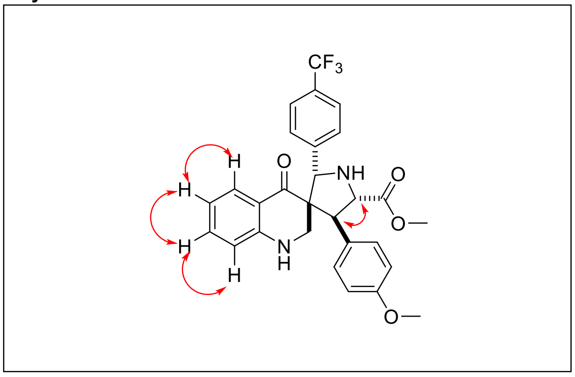

Key HMBC correlations

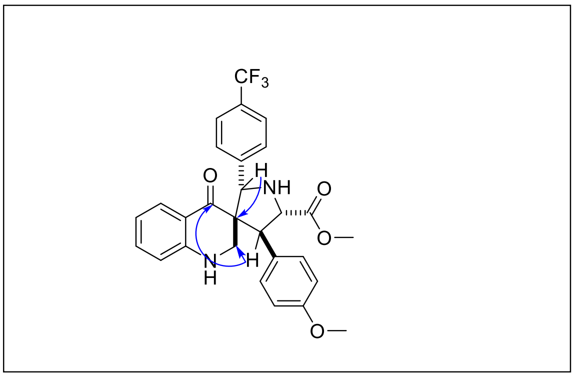

Key NOESY correlations

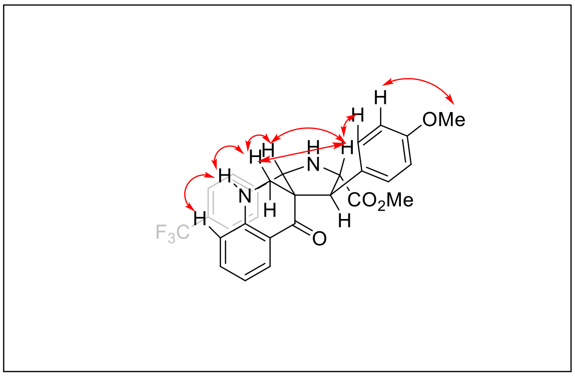

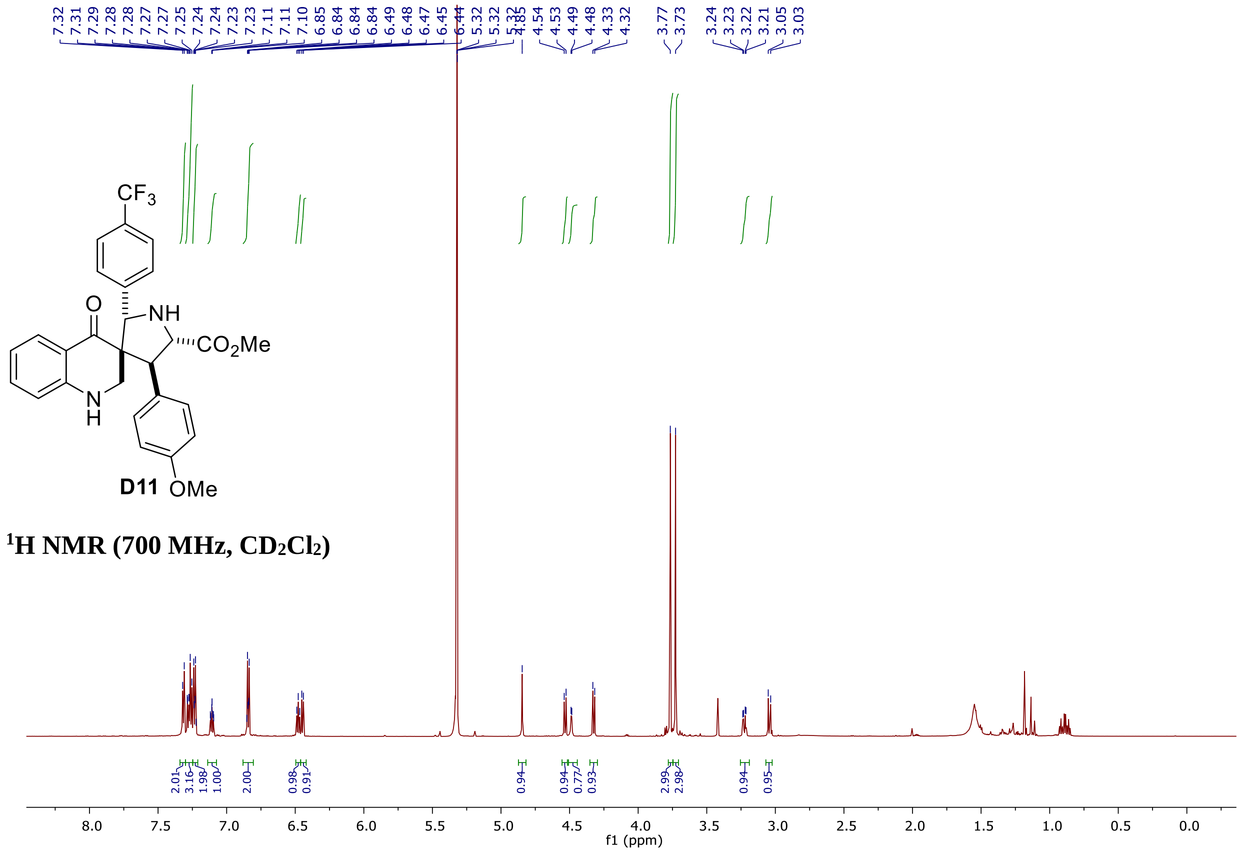

^{1}H NMR (700 MHz, CD_2Cl_2)

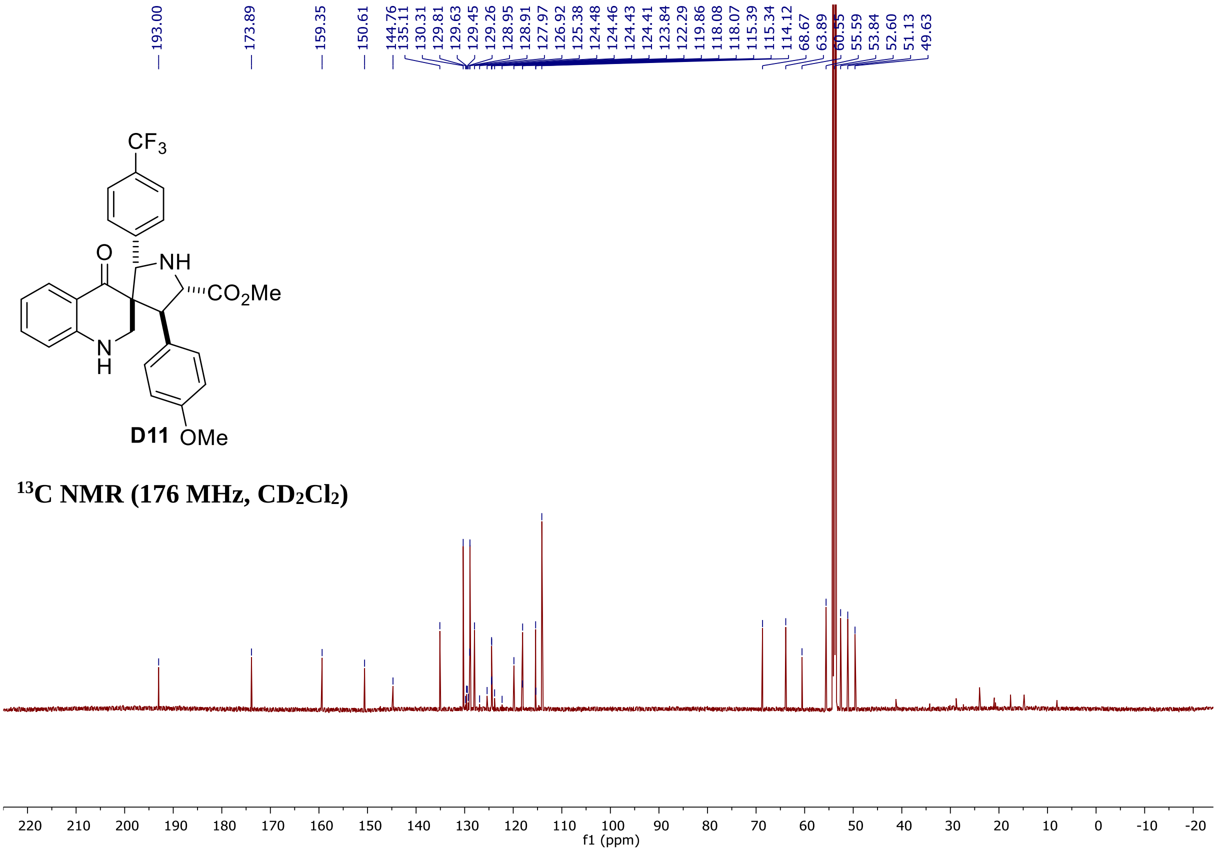

^{13}C NMR (176 MHz, CD_2Cl_2)

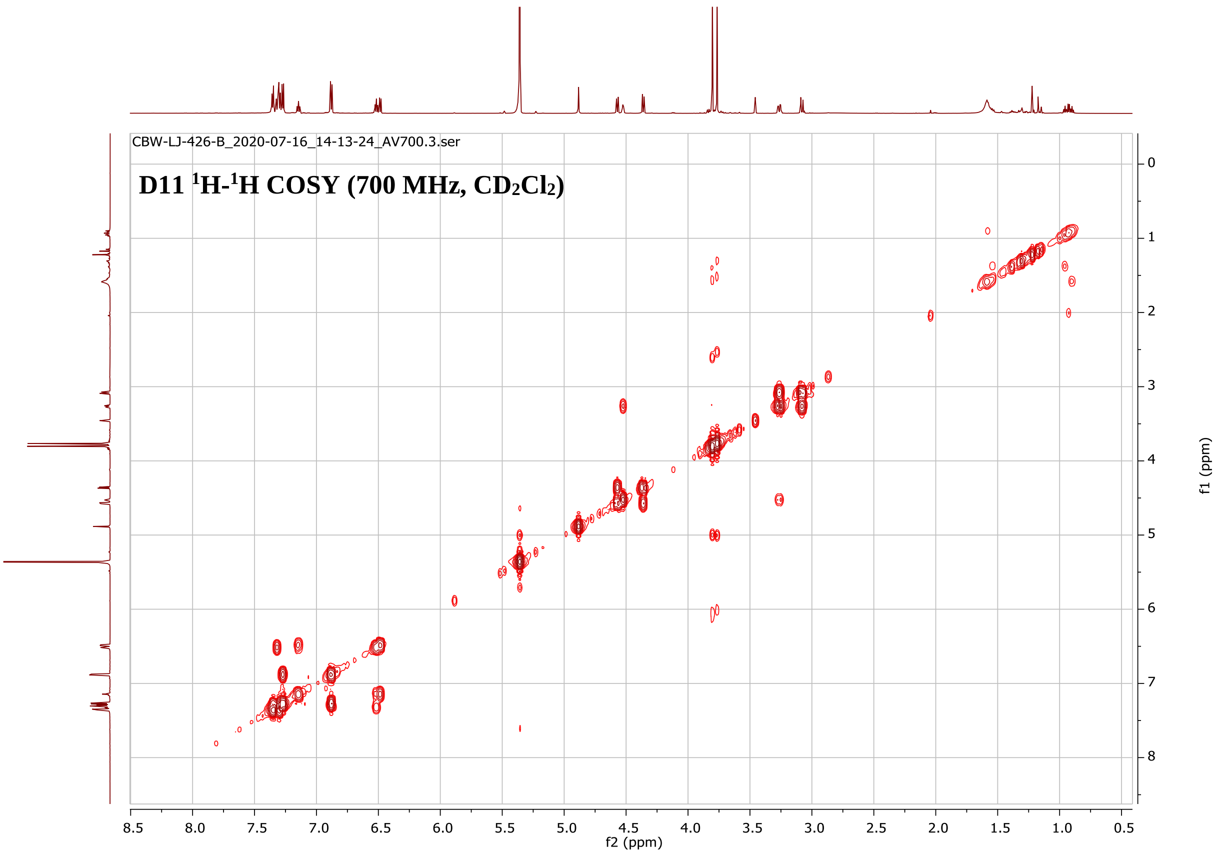
CBW-LJ-426-B_2020-07-16_14-13-24_AV700.3.ser
D11 ¹H-¹H COSY (700 MHz, CD2Cl2)
f2 (ppm)
f1 (ppm)

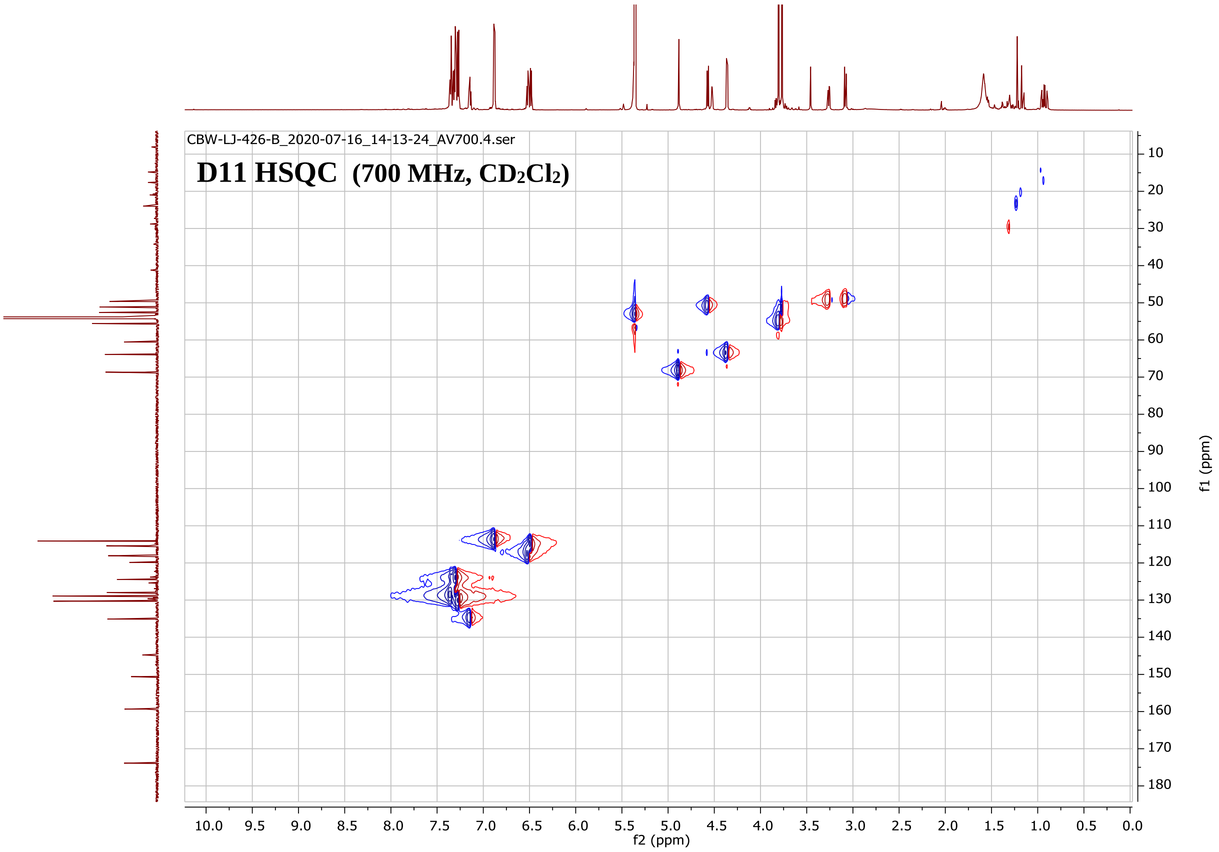
CBW-LJ-426-B_2020-07-16_14-13-24_AV700.4.ser
D11 HSQC (700 MHz, CD2Cl2)
f2 (ppm)
f1 (ppm)

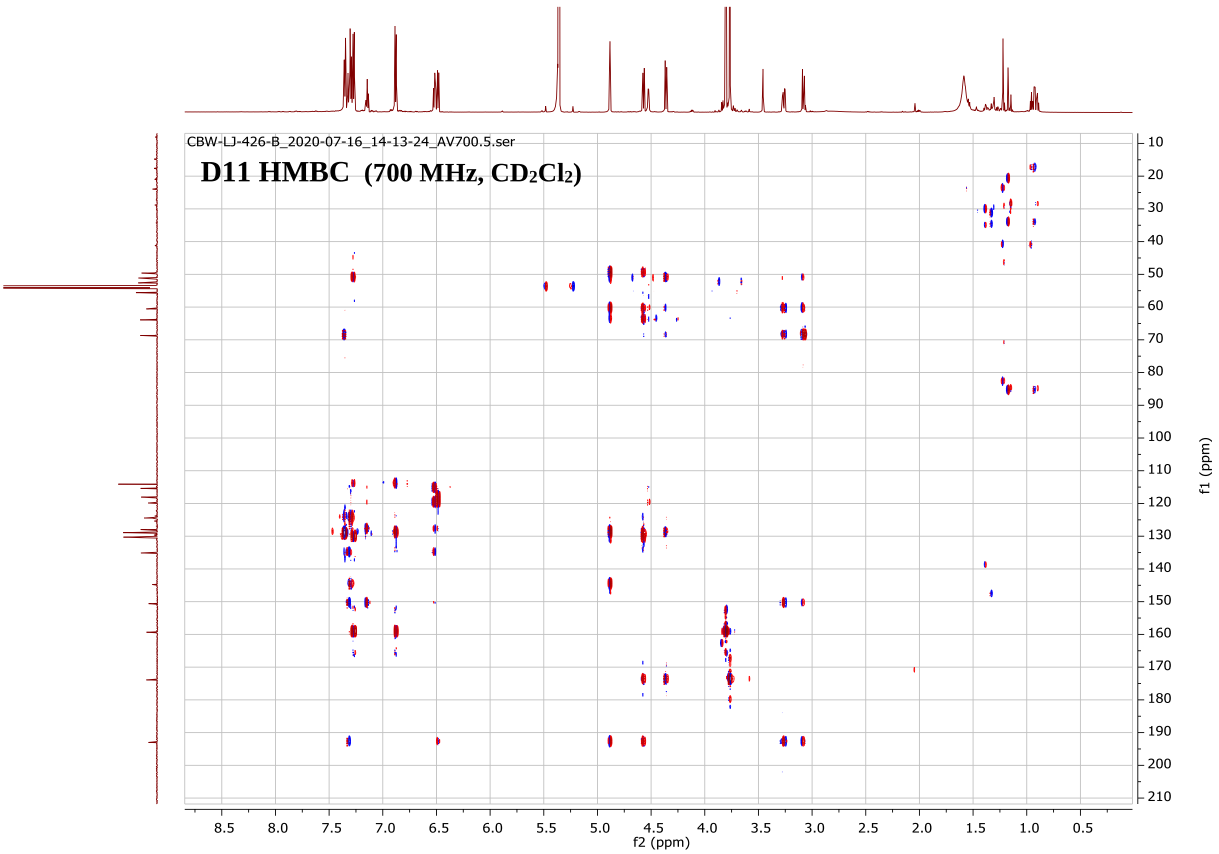
CBW-LJ-426-B_2020-07-16_14-13-24_AV700.5.ser
D11 HMBC (700 MHz, CD2Cl2)
f1 (ppm)
f2 (ppm)

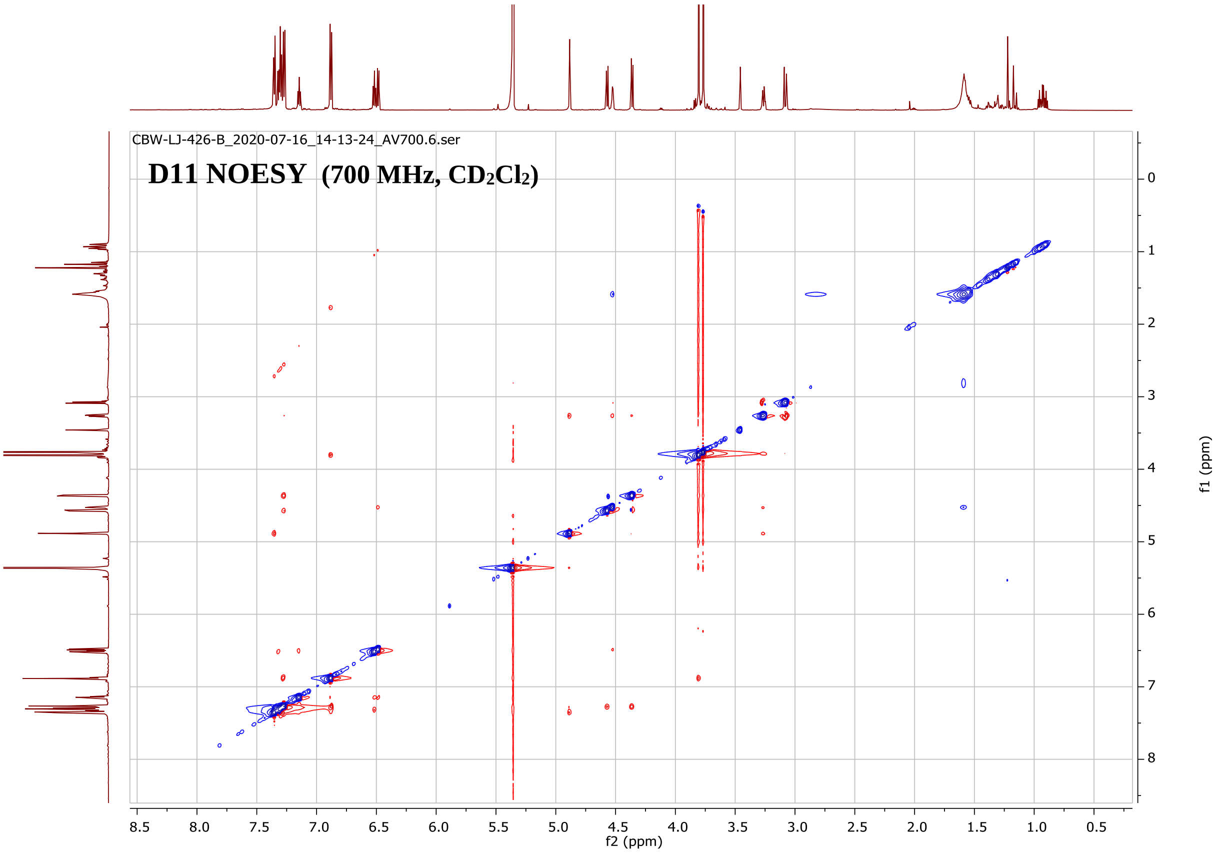
CBW-LJ-426-B_2020-07-16_14-13-24_AV700.6.ser
D11 NOESY (700 MHz, CD2Cl2)
f1 (ppm)
f2 (ppm)
